# Effective Detoxification of Olive Mill Wastewater Using Multi-Step Surfactant-Based Treatment: Assessment of Environmental and Health Impact

**DOI:** 10.3390/molecules29184284

**Published:** 2024-09-10

**Authors:** Yazan Akkam, Mohammad Zaitoun, Islam Aljarrah, Aiman Jaradat, Ali Hmedat, Hassan Alhmoud, Taha Rababah, Ali Almajwal, Numan Al-Rayyan

**Affiliations:** 1 Department of Medicinal Chemistry and Pharmacognosy, Faculty of Pharmacy, Yarmouk University, Irbid 21163, Jordan; m.zaitoun@yu.edu.jo (M.Z.); islamsubhi12@gmail.com (I.A.); 2 Department of Civil Engineering, Hijjawi Faculty for Engineering Technology, Yarmouk University, Irbid 21163, Jordan; ayman.j@yu.edu.jo; 3 Department of Pharmaceutical Technology and Pharmaceutics, Faculty of Pharmacy, Yarmouk University, Irbid 21163, Jordan; ali.hmedat@yu.edu.jo (A.H.); hassan.alhmoud@yu.edu.jo (H.A.); 4 Department of Nutrition and Food Technology, Jordan University of Science and Technology, Irbid 22110, Jordan; trababah@just.edu.jo; 5 Department of Community Health Sciences, College of Applied Medical Sciences, King Saud University, P.O. Box 10219, Riyadh 11433, Saudi Arabia; aalmajwal@ksu.edu.sa; 6 Department of Pediatrics, School of Medicine and Public Health, University of Wisconsin, Madison, WI 53706, USA; alrayyan@wisc.edu

**Keywords:** olive mill wastewater, detoxification, sodium dodecyl sulfate, micelles, tissue culture, antibacterial, characterization

## Abstract

Olive mill wastewater (OMW) poses a significant environmental challenge and health concern in olive-producing countries, including Jordan. Surfactant micelles are frequently employed as solubilizing agents to enhance the water solubility of chemical compounds. This study aims to leverage the sodium dodecyl sulfate (SDS) micelles in a multi-step process to detoxify OMW for agricultural and industrial uses and reduce its impact. The OMW was treated in multiple steps: screening, coagulation with different chemicals, and distillation with different surfactants. The treatment steps were monitored using LC–MS, GC–MS, ICP–MS, chemical oxygen demand contents, and total phenolic compounds. The detoxification of OMW was evaluated using standard germination assays, MTT assays using tissue culture, and toxicity assays using fluorescence bacteria. Following the treatment, the seed growth rate improved significantly from 0% to 100%. The GC–MS revealed a substantial decrease in pollutants. The concentration of polyphenols was reduced to 2.5%, while the COD level decreased to 35%. The toxicity in bacteria was significantly reduced in a time-dependent manner, and the toxicity in human cells decreased by 95%. Additionally, between 50% and 95% of metals in OMW were removed. The multi-step SDS-based approach successfully detoxified the OMW and enhanced water quality, which would pave the road for its direct application in industry and agriculture.

## 1. Introduction

The Mediterranean countries are known as the primary global producers of olive oil, as they account for 98% of global olive oil production [1]. For instance, Jordan produced more than 20 thousand tons of olive oils in 2022 [2]. Unfortunately, the olive oil extraction process produces large amounts of polluted water as a liquid by-product called olive mill wastewater (OMW) [3], the estimated volume of which is about 30 million m^3^ per year [4]. Subsequently, thousands of cubic meters of OMW and thousands of tons of solid waste are produced in Jordan [5].

Olive oil mill wastewater (OMW) is a significant environmental challenge due to its high chemical oxygen demand (COD), organic load, and phenolic content, all of which can cause phytotoxicity and impede microbial growth [6,7,8]. Moreover, as OMW is discharged into surface water or spread on the land, it can pollute surface and groundwater bodies, harm agricultural fields and the environment, and harm public health [2,4,9,10,11,12]. Furthermore, researchers have found that OMW is toxic to plants even after 100-fold dilution and affects seed germination [13].

Various methods have been explored to address this problem, such as electro-coagulation [14], electro-Fenton reaction combined with anaerobic digestion [15], and the integration of Fenton’s reaction with anaerobic treatment [16]. Additionally, technologies like reverse osmosis [17], electrochemical oxidation [18], catalytic oxidation [19], and distillation [20] have been investigated for their efficacy in treating OMW. More about the current methods of treatment were discussed in detail previously [2]. However, despite advancements, each treatment approach encounters its own set of challenges and limitations, hindering their widespread adoption and effectiveness [2], such as high operational costs [21], incomplete treatment [22], seasonal variability [23], environmental concerns and safety [24], and limited applicability [25].

The efficiency of conventional treatment methods is limited due to the presence of a large amount of organic matter [22]. Moreover, the presence of substances like tyrosol, hydroxytyrosol, and polyphenols in OMW further complicates the treatment process [26,27]. Therefore, the treatment of OMW is complex and expensive [28,29]. Moreover, the current treatment methods may not fully comply with discharge limits and regulatory standards [30,31]. Thus, a unique, safe, and cost-effective treatment of OMW that produces harmless by-products without harming plants, humans, or microorganisms is required [28].

Micelles have been extensively studied for their ability to enhance the solubility of poorly water-soluble compounds, including drugs. The formation of micelles using surfactants has been shown to significantly improve the water solubility of drugs, leading to enhanced bioavailability, reduced toxicity, improved drug stability, and altered drug distribution [32,33].

Micelles are self-assembling colloidal systems composed of a hydrophobic core surrounded by a hydrophilic corona, which can enhance the solubility of poorly water-soluble compounds [4]. Hence, micelles can effectively capture hydrophobic molecules from water, assisting in the elimination of pollutants [34]. Furthermore, micelles have been investigated for their capacity to eliminate both anionic and neutral pollutants from water [35].

Sodium dodecyl sulfate (SDS) is a strong anionic surfactant with the formula NaC_12_H_25_SO_4_. SDS is often selected for the effective removal of heavy metals in water [36]. Also, SDS was used to extract the phenolic compounds from OMW for industrial uses by a mixture of nonionic/anionic surfactants [36].

Previous applications of surfactants were observed in micellar-enhanced ultrafiltration (MEUF). The proposed approach involves the introduction of a surfactant into contaminated water at a concentration exceeding the critical micelle concentration (CMC). The micelles should be larger in size to be retained in a membrane with a pore size larger than required for pollutant retention [37]. However, this method has many limitations, such as frequent pore obstruction. This may lead to permeate flux reduction and permeation of surfactant monomers and, subsequently, potential toxicity due to the leakage of the surfactant monomer [38].

In this work, a multi-step method (*coagulation–flocculation–sedimentation*) along with SDS micelle-distillation was used to reduce the toxicity of OMW, prevent potential secondary toxicity from the surfactant, and collect the polyphenols from OMW. The detoxification steps were monitored using liquid chromatography–mass spectrometry (LC–MS), gas chromatography–mass spectrometry (GC–MS), inductively coupled plasma–mass spectrometry (ICP–MS), high chemical oxygen demand contents, and total phenolic compounds. The detoxification of OMW was evaluated using standard germination assay, MTT assay using tissue culture, and toxicity assay using fluorescence bacteria.

## 2. Results and Discussion

### 2.1. Coagulation–Flocculation–Sedimentation

This step is to manipulate the electrical charges to promote the aggregation of suspended solid materials into larger flocs, which can then settle more readily. This floccule can capture various hazardous compounds and remove them from the liquid [39]. Several chemicals were tested: alum, ferrous sulfate, eggshell, and lime (CaOH_2_). The best result was achieved using lime (CaOH_2_) at a concentration of 30 g/L; the volume of sediment was 60% of the total volume, whereas it was less in other agents. This result suits well with the previously published works [39].

Typically, the pH of untreated OMW falls within the acidic range of 4–6 [40]. In this study, the pH of the OMW was measured at 4.5. Upon coagulation, the pH increased significantly, reaching a pH = 12. The significant increase in pH is due to the use of a high concentration of lime [41]. This high alkalinity may facilitate further degradation of compounds that are hard to remove. It is important to mention that the treated water after this step did not neutralize. The neutralization would add an extra step, and it has been reported that electrolytes that may be generated from the neutralization step affect the micelle formation [42].

### 2.2. Selection of Surfactant and Determination of Total Phenolic Compounds

According to the results of total phenolic compounds (Table 1), the coagulation step alone with lime (CaOH_2_) successfully removed 56% of the total polyphenols. Subjecting the coagulated OMW to distillation (without the presence of any surfactant) reduced the polyphenols by 88%. To further remove the polyphenol contaminants, distillation with three different types of surfactants (anionic, cationic, and neutral) was evaluated. The neutral surfactants were used to keep the reaction as environmentally safe as possible. Neutral surfactants, in general, are biodegradable and based on natural fatty acids and sugar alcohol sorbitol [43].

Distillation with sodium lauryl sulfate was the best in removing polyphenolic compounds, as the residual percentage decreased to approximately 2.5%. The results of other surfactants (span 80, span 20, and CTAB) were 14%, 11%, and 11.2%, respectively. Therefore, SDS was solely used in further experiments.

Furthermore, to determine the optimum concentration of SDS, five different concentrations, CMC, CMC*2, CMC*4, CMC/2, and CMC/4, were evaluated. The highest clearance percentage was observed at all concentrations that were either equal to or more than the CMC, or 2.31 g/L or 0.008 mol/L. Micelles form at these concentrations.

Micelles have a hydrophobic core where many types of lipids and lipophilic compounds are entrapped, and the hydrophilic surface can form many interactions with polar compounds [3]. Micelles have been extensively studied for their ability to enhance the solubility of poorly water-soluble compounds, including drugs. The formation of micelles using surfactants has been shown to significantly improve water solubility [32,33]. For instance, the use of micelles has been reported to increase the solubility of drugs like imiquimod, fluocinolone acetonide, docetaxel, paclitaxel, curcumin, and thymoquinone [44,45,46,47,48]. Moreover, the solubility enhancement achieved through micelles has been linked to their ability to form nano-sized structures [49]. The formation of micelles is influenced by factors such as surfactant properties, critical micelle concentration (CMC), and the structure of the micelles themselves [50,51]. These micelles, typically ranging around 80 nm in size, provide a favorable environment for solubilizing poorly soluble compounds [52,53]. The micelle formation process involves interactions between hydrophobic and hydrophilic segments of surfactants, leading to the creation of stable structures that can encapsulate the chemicals or other hydrophobic molecules [54,55].

Sodium dodecyl sulfate (SDS) micelles have been extensively studied for their ability to enhance the solubility of various chemicals [56]. These micelles can interact with different compounds to form micelle–chemical aggregates, improving solubility. For instance, SDS has been used to enhance the solubility and dissolution rate of poorly soluble active pharmaceutical ingredients [53,57], such as cosmetics [58].

Furthermore, the use of SDS micelles has been explored in the removal of lead and mercury from aqueous solutions, showcasing the diverse applications of SDS micelles beyond solubility enhancement [59,60].

OMW contains a high load of organic compounds, including the following organic groups: benzoic acids and derivatives, aromatic conpounds, fatty acids, cinnamic acids, phenyl ethyl alcohols, other phenolic acids, flavonols, flavones, lignans, isochromans, and secoiridoids [61]. It is expected that distillation may remove 90% of the polyphenols and organic compounds. However, OMW contains a high concentration of volatile organic compounds, including organic acids. It has been reported that more than 60 volatile chemical compounds are present in OMW [61], including short-chain fatty acids (six or fewer carbons) [62]. Hence, by distillation, all volatile organic compounds will be concentrated in the distilled product. SDS-micelle will form extra interactions with such compounds; therefore, a large portion of the volatile compounds will be removed.

**Figure 1:**
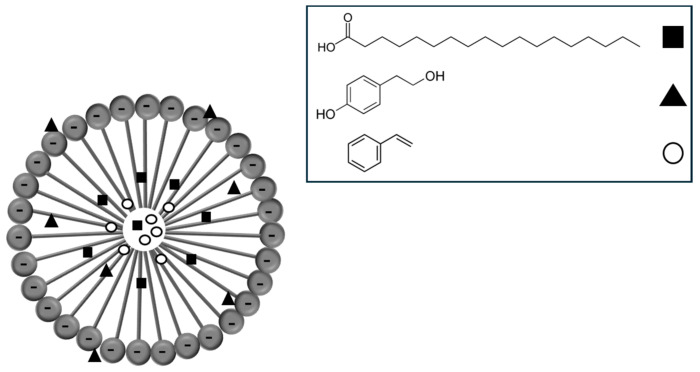
Possible site of interaction between micelle and some OMW, where the site of interactions differs according to hydrophobicity and the tendency of hydrogen bond. The rectangle is stearic acid, the triangle is tyrosol, and the circle is styrene.

Different types of bonds are established during the interaction between micelles and the contaminants. For example, fatty acids possessing an extended alkyl chain, such as stearic acid, may establish a hydrophobic interaction with the micelle’s core. Additionally, aromatic compounds such as styrene are also bound to the core. The toxic compound, alcohol tyrosol, is more likely to interact with the head and tail of the micelle in a hydrophilic and hydrophobic manner, respectively (Figure 1).

Several studies have shown the efficacy of micelles in eliminating volatile organic contaminants from water [34,35,63]. Knowing that, along with the results, the addition of SDS micelles to OWM effectively slowed down the release of toxic compounds during the distillation. Hence, micelles can potentially augment the effectiveness of remediation procedures by augmenting the solubility of pollutants, thereby facilitating their removal or degradation.

### 2.3. Germination Assay

**Figure 2:**
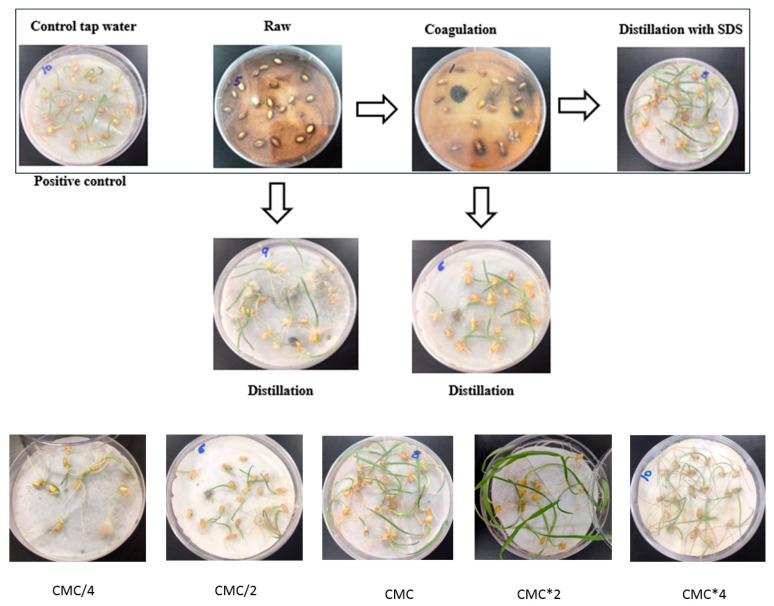
Germination assay for treatment steps.

The germination assay was conducted for each treatment, and several parameters were measured (stem length and fungal contamination). Tap water was used as a positive control. Both filtered raw OMW and coagulated did not exhibit any signs of growth, and the mold was observed after a few days (Figure 2; Table 2). On the other hand, there was a significant increase in stem growth and germination percentage when distillation was applied immediately to raw and coagulated OMW; however, this was still 50% less than in the control group. Additionally, after one week, the presence of hazy molds became apparent. Moreover, as illustrated in Figure 2, the lime coagulation has demonstrated its effectiveness in promoting growth in both shoot and root length and preventing mold formation compared to the distilled raw OMW sample.

When SDS was applied at CMC, the outcomes closely resembled those of the control sample (tap water) in terms of the length of roots and shoots and the absence of any fungal development.

The effect on germination has not shown a significant difference in the growth between CMC and concentrations above it (Table 3). Therefore, SDS at CMC concentration was used for further steps.

It has been reported that the seed can germinate in a wide range of pH (5–8) [64]; in this experiment, the pH of treated water after all steps was 9.0. Hence, the effect of basic pH on growth was tested, and the results showed no differences. Subsequently, there was no need to neutralize the pH for any further experiments.

A hypothesis was formulated suggesting that the pollutants could disrupt the earliest stage of germination and cause the death of the embryo. Therefore, the seeds were soaked in tap water for 8 h to initiate germination, followed by moistening with OMW over a week. Based on the results, although the seeds effectively developed stems and roots, their growth was slower, and the growth rate was smaller than that of the positive control. In addition, clouds of fungal contamination were observed.

OMW contains a high concentration of phytotoxic and phenolic compounds that have antioxidant and antimicrobial properties, such as hydroxytyrosol, tyrosol, hydroquinone, 4-aminophenol, phenol, gallic acid, caffeic acid, 3,5-di-tert-butylcatechol, quercetin, oleuropein, and catechol [65,66]. It has been reported that OMW can inhibit the growth of several fungi, such as Trichoderma spp., Fusarium spp., and Aspergillus spp. [13].

According to the fungal growth, it appears that only using SDS at CMC and above can stop the fungal growth. So fungal contamination may already be in the OWW, and the contaminants (polyphenols) kill all microorganisms except the fungi, or the Fungai is already on the seeds, and the OMW pollutants facilitate the growth. In both cases, the pollutants that were removed by SDS are responsible for both actions.

### 2.4. Liquid Chromatography–Mass Spectrometry Analysis

**Figure 3:**
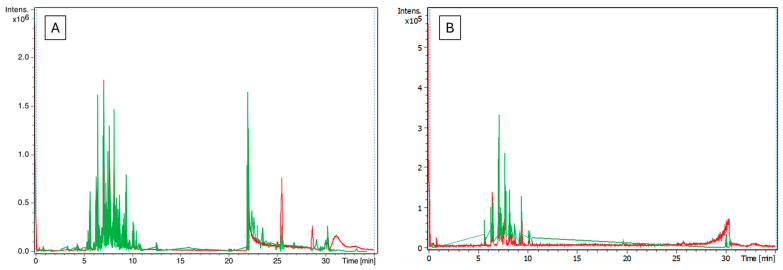
The LC–MS chromatogram SDS-treated OMW. (**A**) The positive ion mode. (**B**) The negative ion mode. The red color represents the total ion chromatogram and the green color represents the elective ion chromatogram.

LC–MS analysis was conducted to determine the compounds present in OMW and to evaluate the efficiency of the detoxification treatment. The list of highest-scoring compounds identified in the OMW at the different treatment steps is shown in Table 4 and the total ion chromatogram (positive and negative ionization modes) is shown in Figure 3.

It has been reported that simple biophenols (tyrosol, hydroxytyrosol, and homovanillic alcohol) and complex biophenols (decarbomethoxy ligstroside aglycone and decarbomethoxy oleuropein aglycone) are the most abundant analytes in OMW [61].

The most suggested compound to have the highest toxicity in OMW is hydroxyl tyrosol, which is thought to be produced from the hydrolysis reaction of oleuropein during the milling step [4]. As shown in Table 4, hydroxy tyrosol was successfully removed via the CMC step while existing in all other steps.

According to the results, there were unexpected compounds such as sunscreens, pesticides, food additives, plasticizers, and pharmaceutical drugs. These chemicals may come from water contaminants during the washing step, preparation steps, or the actual compounds that were not included in the library. To avoid the possibility that the compound is not included, the main olive compounds were used as a standard for identification (Supplement Data S2).

In conclusion, the LC–MS results were inconclusive, so we conducted further instrumental analysis procedures.

### 2.5. Gas Chromatography–Mass Spectrometry Analysis

**Figure 4:**
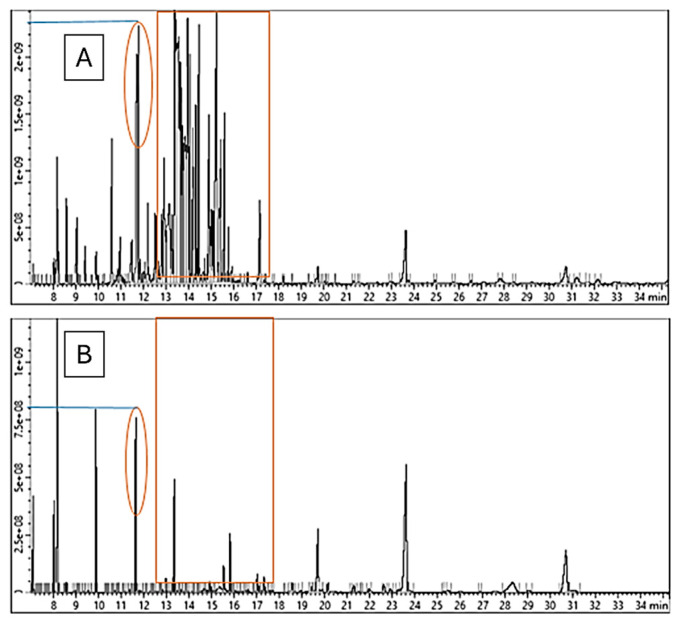
The GC–MS total ion chromatogram. (**A**) Untreated OMW. (**B**) OMW after SDS treatment.

The OMW samples were assessed after the last stage of treatment (SDS) and in their untreated state (raw). Significant variations can be seen in the chromatograms before and after treatment. The CMC-treated OMW showed a considerable reduction in both peak count and intensity, as shown in Figure 4.

**Figure 5:**
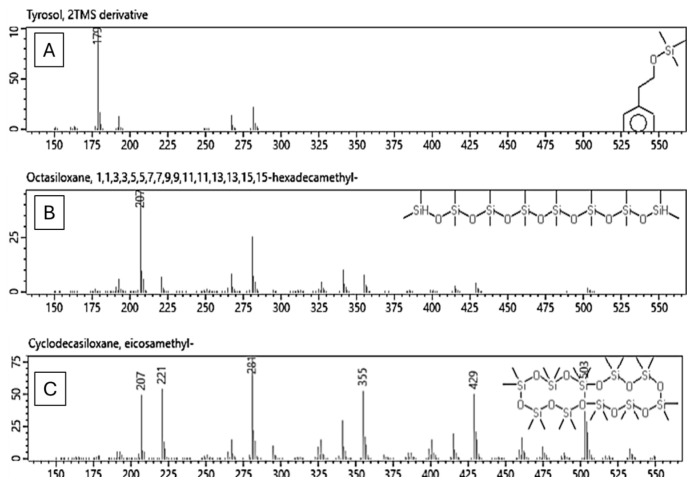
Mass spectrum of (**A**) the TMS derivative of tyrosol. (**B**,**C**) are examples of by-products of the derivatization process.

For example, the tyrosol peak (Figure 4 and Figure 5A), which was initially observed at 11.6–11.8 min, fell to one-third of its original intensity, and other neighboring peaks vanished. Furthermore, the peaks that were present between 12.5 and 17.5 min have completely disappeared.

The compounds in that area include organic acids like Octanoic acid, Aconitic acid, 4-Coumaric acid, Citric acid, Tranexamic acid, Quininic acid, Labdanolic acid, Fumaric acid, Octadecenoic acid, and Stearic acid. Additionally, there are other Glucosides like Syringaresinol diglucoside, hexopyranoside, Matairesinoside, and a wide variety of compounds like Brefeldin, Triphenylene, Homovanillyl alcohol, Lapachol, Styrene, Rhodamine B cation, Isobenzofuran, Gibban, Trimethoxyflavone, Prostaglandin A3, dimethoxyflavanone, Oxytetracycline. The complete list of identified compounds is in the Supplement Data S3.

It is important to clarify that not all peaks in the chromatogram are chemicals from OWM, as some of them are due to the derivatization process in Figure 5B,C. Both compounds were identified in the area between 15.88 and 15.91 min.

### 2.6. Inductively Coupled Plasma Mass Spectrometry Analysis

It has been reported that OMW contains a significant concentration of heavy metals [67,68]. Heavy metal contamination can take place during the handling and processing of olive fruits as well as from the soil [68]. Hence, all possible types of metal were measured, including heavy metals. The mechanism of adsorption of SDS towards heavy metals involves several complex interactions primarily driven by electrostatic forces and hydrophobic interactions. The primary mechanism is through electrostatic attraction. The negatively charged sulfate groups of SDS can bind to positively charged metal ions, such as Pb^2^⁺, Cd^2^⁺, and Cu^2^⁺, leading to the formation of metal–surfactant complexes on the surface of adsorbents [69,70,71]. Moreover, hydrophobic interaction also plays a significant role in the adsorption process. The hydrophobic tail of SDS can interact with organic contaminants, which might be chelated or interact with heavy metals, promoting the aggregation of metal ions within the micellar structure of SDS [72,73].

**Figure 6:**
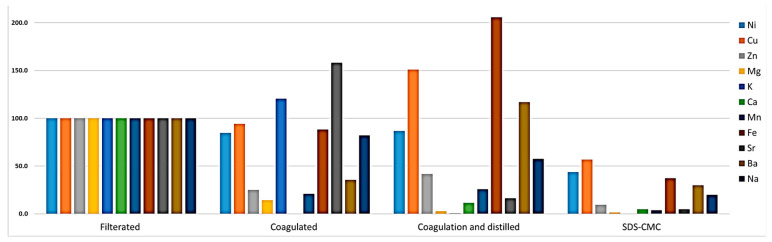
Percentage of metal in OMW samples at different stages.

The initial concentrations of metals in raw OMW are shown in Table 5. There were many variations in metal percentages at the different stages of treatment; however, all of the following heavy metals (Hg, Pb, Cd, As, and Mo) were under the detection limit in all samples (Figure 6). The highest concentration of metals was in the filtered raw sample. After the coagulation step, Ca ions have disappeared completely; it might be due to the precipitation by lime (CaOH_2_), and there was a sudden increase in Sr concentration. After distillation, K ions approach zero, and metals like Cu and Fe have increased greatly compared to raw samples. This increase in the concentration of these ions can be explained by their transfer to the product container of distillation, accompanied by the decrease in the water volume [74]. At the final SDS-CMC step, the total amount of ions has decreased significantly; Ni, Fe, and Cu approach half of their original amounts in the raw sample due to their interaction with SDS micelles [75]. SDS is already known for its capability for the removal of heavy metal ions [76].

### 2.7. High Chemical Oxygen Demand (COD)

The Chemical Oxygen Demand (COD) test is utilized to predict the amount of oxygen needed by the effluent. It is employed for the purpose of monitoring and regulating discharges, as well as evaluating the effectiveness of treatment plants [77]. OMW contains high concentrations of chemical oxygen demand (COD), reaching 220 g/L, mainly consisting of phenol, lipids, acids, and sugars [18]. Hence, it has a negative impact on aquatic and terrestrial ecosystems upon disposal [78]. Since the chemical composition of olive mill wastewater is highly variable both qualitatively and quantitatively according to the country and method of extraction [2,79], the COD was measured in all treatment stages. According to the data presented in Table 6, the concentration of COD decreases gradually during the treatment process, ultimately achieving a 65% reduction following the SDS-CMC phase.

### 2.8. Bacteria Toxicity Assay

**Figure 7:**
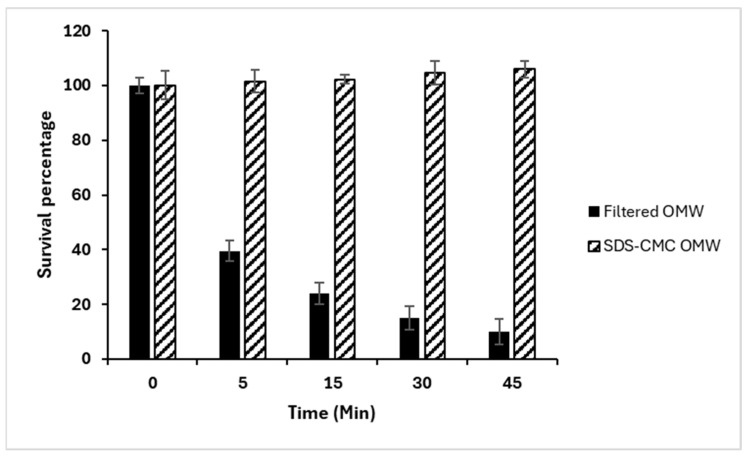
Toxicity assay using fluorescence bacteria over a 45 min period. Experiments were conducted in triplicates, and the error bars represent the means ± standard deviation.

The presence of OMW in the environment can disrupt microbial consortia and bacterial populations [78]. Moreover, the antimicrobial properties of OMW, particularly in its liquid form, can inhibit the growth of plants and microorganisms, affecting the overall biodiversity of ecosystems [78,80]. Two samples of OMW were analyzed: the first sample was filtered, and the final sample was treated with SDS-CMC. The survival percentage was determined by measuring the luminescence intensity of Vibrio fischeri at various time intervals over a period of 45 min. As shown in Figure 7, extended duration has significantly amplified the toxicity and mortality rates in filtered samples as compared to CMC samples. For instance, the survival rate in the filter sample decreased substantially, reaching 50% after the incubation period, while it remained constant in the SDS-CMC-treated sample. The toxicity develops in a time-dependent manner. As per the kit instructions, the assay can last for a maximum of 45 min before the fluorescence signal fades. The toxicity results of filtered OMW on photobacterium Vibrio fischeri suit well with previously published [81,82].

According to reports, the main cause of toxicity in V. fischeri is the polar portion of OMW. Therefore, it has been recommended that diluting OMW should not be considered a viable remedy for disposing of OMW, as the toxicity remains even at low concentrations [82]. The incubation period with tested bacteria was extended to 45 min to confirm that there was no toxicity, as previous studies have only examined toxicity for a duration of 10 to 15 min.

### 2.9. MTT Assay

In addition to its phytotoxic and bactericidal effects, OMW also exhibits high toxicity towards human cells, with the polyphenols being the main contributors to this activity [83]. However, multiple studies reported that the polyphenol extracted from OMW (esp. hydroxy tyrosol) has anti-cancer and antioxidant activity and improves the chemotherapy and cytoprotective [84,85,86]. Furthermore, OMW extract also showed a promising ingredient for dermal applications to improve skin health and skin protection [87].

**Figure 8:**
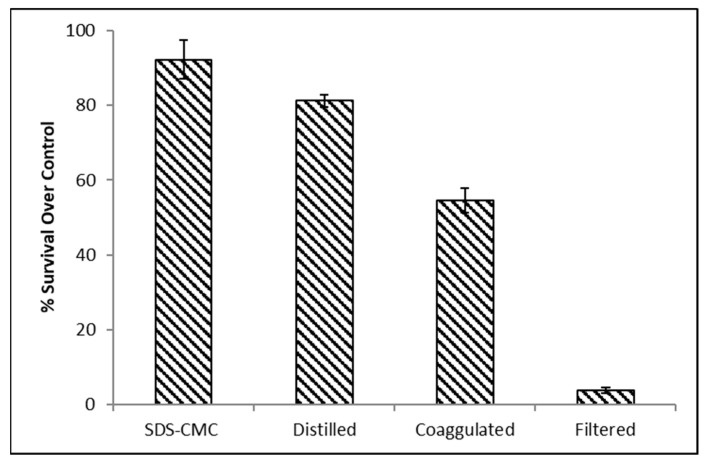
Toxicity assay of OMW treatment steps using human fibroblast and MTT assay. Experiments were conducted in triplicate, and the error bars represent the means ± standard deviation.

The purification steps were evaluated using a human fibroblast (non-cancer cells), one of the most abundant cell types in the stroma. It has a variety of functions and composes the basic framework for tissues and organs [88]. The survival rate relative to the control sample was quantified following each treatment stage. Figure 8 illustrates that the survival percentage, relative to the control sample of distilled water with equivalent tonicity, was just 3% for filtered OMW. However, it gradually climbed and reached approximately 95% with SDS-CMC.

### 2.10. Possible Use for Mass Productions

**Figure 9:**
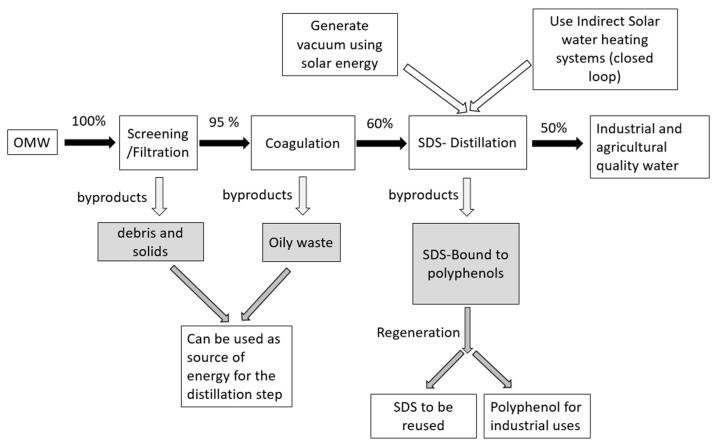
Diagram showing the detoxification steps of OMW, by-products, and possible solutions. The percent represents the percentage of treated water after each step compared to the initial volume.

Several factors can affect the feasibility of mass production, including energy consumption, capacity, production rate, and SDS (cost and toxicity). Figure 9 illustrates the process of detoxification, including the by-products and possible solutions. The debris, sludge, and oily waste can serve as fuel for distillation [89]. To decrease the consumption of fossil fuels and reduce the emission of carbon dioxide, it is recommended to utilize vacuum distillation as a means to lower the boiling point and, thus, minimize the requirement for heating during the distillation process. Organic compound distillation has been conducted using vacuum distillation [89]. Moreover, solar energy can be harnessed to produce the electrical energy required for vacuum distillation. In addition, OMW can be preheated before distillation by utilizing an indirect solar water heating system. This technology can raise the temperature of the water to 90 °C. Additionally, the impact of OMW on the efficiency of the system is negligible [90]. Moreover, it is essential to economically recover and recycle the SDS since the surfactant comprises a significant proportion of expenses and to avoid additional contamination of the environment. There are multiple techniques for recovering the surfactant. These include the addition of an excess of multivalent cations (such as Ca+2) beyond the stoichiometric value, which can lead to a significant amount (45–55%) of surfactant precipitation [91]. Another method is to lower the temperature of the SDS solution to 4 °C, which is below the reported Kriff point of SDS (16 °C) [92]. Other techniques include foam fractionation [93], electrochemical treatment [94], and acidification at Ph = 1 [36]. The capacity and rate of detoxification have to be further evaluated.

## 3. Materials and Methods

### 3.1. Chemicals and Materials

Analytical-grade reagents, chemicals, and HPLC-grade solvents were obtained from Sigma-Aldrich and the local market. OMW was freshly collected in 20 L polyethylene containers from the decenter outlet of three-phase olive oil mill processing systems located in Irbid/Jordan during the olive harvesting season and then stored at 4 °C until use in laboratory experiments.

The natural eggshell waste materials (ES) were obtained from local homes and restaurants to serve as coagulant material. The ES was washed with water to remove impurities, dried for 3 h at 60 °C, and crushed mechanically in a mixer. The dried eggshell product was calcined (CES) at 800 °C for 3 h using a muffle furnace (Carbolite, Sheffield-England), sieved in a mesh number (40–60) to obtain grain size diameters ranging from 425–600 μm, and stored in a dry box until use.

### 3.2. Methods

#### 3.2.1. Purification Steps

##### Pretreatment

Samples were completely stationary for 24 h and divided into two net layers at the macro level, and then the water layer was filtered using locally made fabric mesh to remove large debris and particles.

##### Coagulation–Flocculation–Sedimentation

The process was conducted as described elsewhere [36] using the optimum concentration of different agents: alum (600 mg/L) [37], ferrous sulfate (300 mg/L) [37], eggshell (6 g/L) [38], and lime (CaOH2) (15 g/L) [36].

The OMW samples were added first into a graduated Imhoff Cone for one hour to allow for any readily settleable solids to settle down by gravity. The supernatant was then taken and treated by coagulation and flocculation using a conventional 6-station Jar test apparatus (Stuart-SW6) supplied by six beakers (1.0 L each). Alum is used as a coagulant material, and the optimum dose for TSS removal was predetermined first. A total of 500 mL of OMW was transferred to each jar-test beaker, and then six different doses of alum (0.5–3.0 at a 0.5 g/L interval) were added into all beakers. All beakers were subjected to rapid mixing initially at 120 rpm for 3 min and slow mixing thereafter at 20 rpm for 30 min. Then, the contents of the beakers were allowed to settle by gravity for 1.0 h. The alum dose corresponded to the least turbidity, and the TSS value was taken as an optimum dose. The predetermined optimum dose of alum was then added to all OMW samples and treated in the same manner using a jar-test apparatus. The supernatant solution was then withdrawn, collected, and used in the next experiments. Part of this supernatant solution was filtered through filter paper (Whattmann 40) and tested for the same parameters tested for raw OMW to assess the effect of coagulation on OMW quality. All the experiments and sample analysis were performed in triplicate at room temperature of 25 ± 1 °C without pH adjustment.

##### Micellar Distillation Treatment

**Figure 10:**
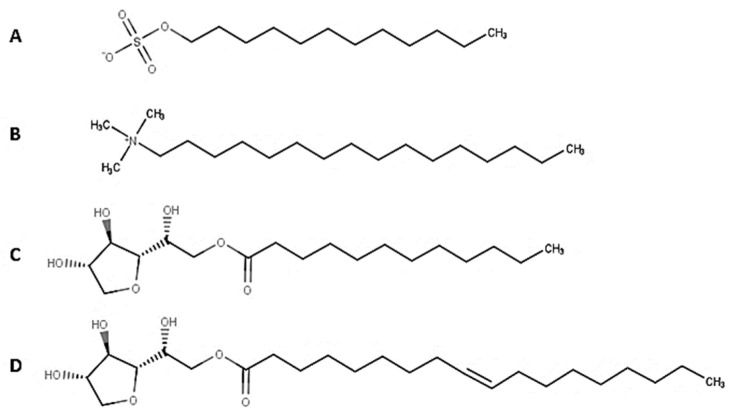
Surfactants (**A**) SDS, (**B**) CTAB, (**C**) Span 20, (**D**) Span 80.

Different types of surfactants at the critical micelle concentration (CMC) were evaluated separately: Span 80 at 0.014 g/L, Span 20 at 0.07 mg/L, hexadecyltrimethylammonium (CTAB) at 0.334 g/L, and sodium dodecyl sulfate at 2.3 g/L (Figure 10). Moreover, SDS was tested at different concentrations (above or below CMC: half CMC concentration, quarter CMC concentration, double CMC concentration, and fourfold CMC concentrations). The surfactant was dissolved in 100 mL of OMW and then distilled at 90 °C using standard distillation apparatus and method [39].

#### 3.2.2. Total Phenolic Compounds

The test was conducted as described previously elsewhere [40,41] using Spectrophotometer-UV 1800, Biotech Engineering Management Co., Ltd., Milton Keynes, UK. Briefly, 125 µL of 100-diluted extract was mixed with 500 µL of distilled water and 125 µL of Folin–Ciocalteu reagent. Following 3 min of stirring, 1250 µL of 7% sodium carbonate solution was added to the mixture. The mixture was adjusted to 3 mL with ultrapure water and left at room temperature for 90 min in the dark. The results were represented as mg gallic acid per mL of extract.

#### 3.2.3. Germination Assay

The experiment was carried out following the previously described protocol, with some modifications [36]. Ten locally obtained wheat seeds were presoaked in the tested OMWW for 8 h before germinating under the light of a fume hood at room temperature. To prevent drying during germination, 5 mL of treated water was added periodically to the seeds. The entire process of germination lasted around two weeks.

#### 3.2.4. High Chemical Oxygen Demand (COD)

The COD experiment was performed using the Lovibond COD high-range kit (200–15,000 mg/L) following the manufacturer’s instructions [42]. The samples underwent a 10-fold dilution, and the experiment was conducted using the Lovibond thermal reactor for 120 min at a temperature of 150 °C. The Lovibond Spectro Direct was used at a wavelength of 602 nm (lambda, λ).

#### 3.2.5. Liquid Chromatography–Mass Spectrometry Analysis (LC–MS)

The unknown sample (0.1 mL) was diluted with 0.9 mL of distilled water. Then, 1.0 mL was transferred to the autosampler, and inject 3.0 µL. The samples were analyzed on a Bruker Daltonik (Bremen, Germany) Impact II ESI-Q-TOF System equipped with Bruker Dalotonik (Bremen, Germany) using direct injection. The instrument was operated using the Ion Source Apollo II ion Funnel electrospray source. The capillary voltage was 2500 V, the nebulizer gas was 2.0 bar, the dry gas (nitrogen) flow was 8 L/min, and the dry temperature was 200 °C. The mass accuracy was ˂1 ppm; the mass resolution was 50,000 FSR (Full Sensitivity Resolution), and the TOF repetition rate was up to 20 kHz. Standards were used for the identification of *m*/*z* with high resolution. To evaluate the methods, standards were used (Supplement Data S1).

#### 3.2.6. Gas Chromatography–Mass Spectrometry Analysis (GC–MS)

The OMWW samples were extracted using ethyl acetate and silylated using BSTFA [43]. The GC–MS analysis was conducted following the procedure described previously [44,45]. Briefly, one µL of the sample was subjected to the GC–MS (Chromatec Crystal GC-MSD, Yoshkar-Ola, Russia) equipped with a CR-5 MS column (5% diphenyl, 95% dimethyl polysiloxane, 30 m × 0.25 mm, 0.25-µm film thicknesses). In the MS detector, 70 eV electron ionization was used. MS source temperature was 300 °C, and the transfer line temperature was 230 °C. The temperature column was controlled from 40 °C for 1 min (isothermal) to 280 °C at 3 °C/min, maintaining constant lower and upper temperatures for 3 min. The carrier gas was helium (1.0 mL/min). Estimated compound percentage concentrations were based on relative peak areas. The same chromatographic conditions were used to evaluate a C8–C30 n-alkane standard solution. The chemical constituents were identified by comparing their computed Kovats retention index (KI), matching their mass spectra with the built-in library spectra.

#### 3.2.7. Inductively Coupled Plasma Mass Spectrometry Analysis (ICP–MS)

The experiment was conducted at the Jordanian Atomic Energy Commission as previously published [46,47] on the Thermo Scientific™ iCAP™ TQ spectrometer (Bremen, Germany).

#### 3.2.8. Anti-Bacterial Activity

BioTox™ WaterTox™ EVO Kit was used to determine the toxic effect of OMW on the living bacteria Vibrio fischeri. Samples were prepared according to the manufacturer’s protocol and using the kit reagents. The luminescence was measured using a multi-well plate reader (Synergy HTX Multimode Reader, BioTek, Santa Clara, CA, USA).

#### 3.2.9. Cell Culture Assay

The Human Fibroblast (PDL) cell line was obtained as a kind gift from Prof. Khaled Al-Qaoud (Yarmouk University, Irbid, Jordan). PDL cells were cultured at 37 °C under a 5% CO2 humidified atmosphere in DMEM (with high glucose and sodium pyruvate) supplemented with 10% fetal bovine serum, 1% penicillin/streptomycin, and 1% NEAA.

Cell viability was evaluated using 3-(4,5-dimethylthiazol-2-yl)-2,5-diphenyltetrazolium bromide (MTT) assays to assess cell metabolic activities [95]. Exponentially growing PDL cells were washed with PBS, shortly trypsinated, and counted. Then, cells were seeded at a density of 5000 cells/100 µL/well in 96-well plates and cultured overnight. Afterward, the cells were treated for 72 h with 100 µL of the water samples diluted in DMEM (1:2.5 dilutions).

The absorbance of the samples was recorded at 570 nm in a multiwall plate reader (Synergy HTX Multimode Reader, BioTek, USA). Results were plotted as the mean values of duplicates from a representative experiment that was repeated three independent times. Survival is expressed as a percentage of control.

#### 3.2.10. Statistical Analysis

Data were presented as mean ± SD and analyzed using Microsoft Excel with one-way analysis of variance (ANOVA) (*p* < 0.05).

## 4. Conclusions

The multi-step SDS-based approach successfully detoxified Olive Mill Wastewater (OMW) and significantly improved water quality, making it suitable for direct use in industry and agriculture.

- The treatment process, involving screening, coagulation with various chemicals, and distillation with different surfactants, effectively reduced the environmental and health impacts of OMW.
- Monitoring via LC–MS, GC–MS, ICP–MS, chemical oxygen demand (COD), and total phenolic compounds confirmed the efficacy of the process.
- GC–MS and LC–MS show that the total number of contaminants was reduced significantly.
- The chemical oxygen demand in treated OMW was reduced by 66%.
- The polyphenol contents were reduced by 98%.
- The treatment process interfered with the metals, resulting in the following reductions: Cu (45%), Ni (57%), Fe and Ba (70%), Na (80%), Zn, Mg, K, Ca, Mn, and Sr (more than 95%). All other heavy metals (Hg, Pb, Cd, As, and Mo) were under the detection limit in the raw samples.
- The toxicity of treated OMW was successfully improved: 100% in germination assay, 100% elimination of mold growth, 95% on human cells, and 100% on fluorescence bacteria.
- The potential use on a large scale was also discussed, and solutions were offered.
- The overall reduction in pollutants and toxicity highlights the potential of using SDS micelles for OMW detoxification, offering a viable solution for environmental management in olive-producing regions.
- Future work may focus on mass production.

## Figures and Tables

**Table 1:** The effect of different surfactants on the residual polyphenol contents. Experiments were conducted in triplicate, and the error represents the means ± standard deviation.

Treatment Step	Residual Phenols %
Coagulation	43 ± 7.1
Distillation of coagulated OMW	11.9 ± 2.2
Distillation of coagulated OMW + SDS	2.5 ± 0.6
Distillation of coagulated OMW + span 80	14 ± 2.1
Distillation of coagulated OMW + span 20	11 ± 1.6
Distillation of coagulated OMW + CTAB	11.2 ± 2.4

**Table 2:** Germination assay parameters for all treatment steps.

Sample Name	Germination %	Stem Length %	Fungi Growth %
Control tap water	100	100	−
Raw OMW	-	-	+
Distilled Raw OMW	40	50	+++
Coagulated OMW	-	-	+++
Distilled Coagulated OMW	50	80	+
CMC Distillation	100	100	−

CMC: Critical micelle concentration. Experiments were conducted in triplicate. The (+) sign represents the presence and severity of fungal growth, the (−) sign represents the absence of the growth.

**Table 3:** Germination assay parameters for different concentrations of SDS.

Sample Name	Germination %	Stem Length %	Fungi Growth %
CMC/4	60	40	+++
CMC/2	60	40	+
CMC	100	100	−
CMC*2	100	100	−
CMC*4	100	100	−

CMC: Critical micelle concentration. Experiments were conducted in triplicate.

**Table 4:** The highest-score compounds were identified in the OMW using LC–MS at the different treatment steps.

Compound Name	Raw-Distilled	Raw-Coagulated-Distilled	Raw-Coagulated-Distilled- CMC	Source	Aromatic	Toxicity
Scopoletin	Yes	Yes	No	Natural	Yes	No
Capsaicin	Yes	Yes	Yes	Natural	Yes	No
Humulone	Yes	No	No	Natural	No	No
Caffeic Acid	Yes	No	No	Natural	Yes	No
Umbelliferone	Yes	Yes	Yes	Natural	Yes	No
4-Tert-butyl 2-methylphenol	Yes	Yes	No	Synthetic	Yes	No
Tetramethrin	Yes	Yes	Yes	Synthetic—potent insecticide	No	Yes
Eusolex 6007	Yes	Yes	No	Synthetic—Essential component in sunscreens	Yes	Yes
Isopentyl-4-methoxycinnamate	Yes	Yes	No	Synthetic—sunscreening agent.	Yes	Yes
Dodecylphenol	Yes	No	No	Synthetic—used in the manufacturing of epoxy resins	Yes	Yes
4-Hydroxybenzoic acid n-butyl ester	Yes	Yes	No	Synthetic—bactericidal/fungicidal additives in cosmetics.	Yes	Yes
4-n-Propylphenol	Yes	No	No	Synthetic—Food additive	Yes	No
Dibutylphthalate	Yes	Yes	Yes	Synthetic—plasticizer	Yes	Yes
Octocrylene	Yes	Yes	No	Synthetic—sunscreens and cosmetics	Yes	Yes
Hydrocortisone	Yes	Yes	Yes	Synthetic—Anti-inflammatory agent	No	No
(4 or 7) Hydroxy-Coumarin Plus Hydrate	Yes	Yes	No	Synthetic—Insecticides	Yes	Yes
Trans-nonachlor	Yes	Yes	No	Synthetic—Insecticides	No	No
3-(2,2-Dichlorovinyl)-2,2-dimethylcyclopropene carboxylic acid	Yes	Yes	No	Synthetic—Pesticide	No	Yes
4-hydroxybenzoic acid propyl ester	Yes	Yes	Yes	Natural	Yes	No
4-Nonylphenol	Yes	Yes	Yes	Synthetic	Yes	Yes
Cyprodinil	Yes	Yes	No	Synthetic Fungicide	Yes	Yes
2-ethylhexyl 3-(methoxyphenyl)-2-propenoate	Yes	Yes	Yes	Synthetwunscreen	Yes	Yes
Ethyl-4-aminobenzoate	Yes	Yes	Yes	Synthetic Local anesthetic	Yes	Yes
3-Hydroxy-4-methoxycinnamic acid (isoferulic acid)	Yes	Yes	Yes	Natural	Yes	No
Hydroxyl tyrosol	Yes	Yes	No	Natural	yes	yes

**Table 5:** The concentrations of metals in raw OMW. Experiments were conducted in triplicate, and the error represents the means ± standard deviation.

Treatment Step	mg/L	Typical OMW Values [70]
Ni	0.29 ± 0.05	-
Cu	0.25 ± 0.03	0.0021 (%)
Zn	0.575 ± 0.09	0.0057 (%)
Mg	376.036 ± 11.5	100–400 mg/L
K	1343.48 ± 23.54	2700–7200 mg/L
Ca	690.40 ± 12.4	120–750 mg/L
Mn	0.88 ± 0.20	0.0015 (%)
Fe	0.636 ± 0.08	-
Sr	6.789 ± 1.4	-
Ba	0.739 ± 0.17	-
Li	0.013 ± 0.006	-
Na	40.09 ± 2.4	40–900 mg/L
Hg	BDL	
Pb	BDL	-
Cd	BDL	-
As	BDL	-
Co	BDL	-
Mo	BDL	-

BDL: below detection limits.

**Table 6:** The concentration of COD and the percentage of removal for each treatment step. Experiments were conducted in triplicate, and the bars represent the means ± standard deviation.

Treatment Step	COD (g/L)	% Removal
Filtered OMW	124.6 ± 15.3	0
Coagulated OMW	108.0 ± 9.7	13.3
Distilled OMW	86.58 ± 7.5	30.5
CMC step	44.14 ± 4.3	65.6

## Data Availability

The data presented in this study are available upon request from the corresponding author.

## Supplementary Materials

The following supporting information can be downloaded at: https://www.mdpi.com/article/10.3390/molecules29184284/s1, S1: The list of standards used to validate the LC-MS method, S2: The olive oil compounds used for the identification in the LC-MS method, S3: The complete list of identified compounds using the GC-MS method.
