# Supplementary material for: Effective Detoxification of Olive Mill Wastewater Using Multi-Step Surfactant-Based Treatment: Assessment of Environmental and Health Impact
Source: Molecules. 2024 Sep 10;29(18):4284. doi: 10.3390/molecules29184284 (PMC11434139; doi:10.3390/molecules29184284)
Supplement: Supplementary file 1 [file molecules-29-04284-s001.zip › cmc-1micro.pdf]

# Analysis report

## Sample Info

| | |
|---|---|
| Analyst: | nora |
| Chromatogram: | №307 2023-01-23 14:16:23 |
| Data file: | G:\Analytic 3\Projects\2291421+МСД 2160148\.chromatograms\2023-01-23 14-16-23 0307.chrx |
| Instrument: | Chromatec-Crystal 5000 S/N: 2291421 Firmware version: v 03.21.17.732 |
| Method: | C:\ProgramData\Chromatec\Chromatec.Panel.UI\TempMethods\صيدلة_Scheduler_83624966a69340a6bab53bb828f0e871.mthx |
| Sample name: | cmc-1micro |
| Sample volume: | 1 |
| Dilution: | 1 |
| Column: | |
| Comments: | |

## Instrument Info

```
Instrument method: صيدلة.mthx
Channel - 1
Run time, min: 35.31
PostRun, min: 0
Equilibration time, min: 0
Oven - 1
Temperature, °C: (80; 3)(13; 240; 20.0023)
Inlet - 1
Mode: Splitless [+ gas saver]
PrepRun: Off
Split flow, ml/min: (0; 1)(0; 30; 3)(200; 10; 0.0)
Septum purge flow, ml/min: (0; 1)(0; 3; 0.0)
Temperature, °C: 250.0
Column - 1
Control mode: Constant capillary flow
Capillary flow, ml/min: 1.000
Solenoid valve - 1
PrepRun: On
Switching time: (On; 1)
MSD
Runtime, min = 35.31
Ion source temperature, ℃ = 280
Transfer line temperature, ℃ = 200
Calibration gas = Off
Ionization type = Electron ionization
Data collection type = Centroid
Segments count = 1
Segment number = 1
Start time, min = 7
Filament = On
Emmission current = 100
Gain = 300000
Scans count (in segment) = 1
Scan number = 1
Acquisition type = Mass range
Mass range = 150 - 550
Dwell time, sec = 0.2
Settings = Current tune

Vial number : 1
Sampling type : Simple
Vial needle depth, mm : 28
Solvent washing
Vials for washing : A
Solvent volume, ul : 5
Number of washing cycles : 3
Drain speed, µl/min : 300
Sample washing
Number of washing cycles : 3
Injection speed, µl/min : 2
Sample volume, ul : 3
Sampling
Sampling speed, µl/min : 2
Number of strokes : 0
Viscosity delay, sec : 0
Sample volume, ul : 1
Injection
Pre injection delay, sec : 0
Post injection delay, sec : 0
Injection speed, µl/min : 4500
Solvent washing
Vials for washing : A
Solvent volume, ul : 3
Number of washing cycles : 3
Drain speed, µl/min : 2
```

## Chromatogram

**MSD-1 : TIC**

Range from 7.000 min. to 35.274 min.

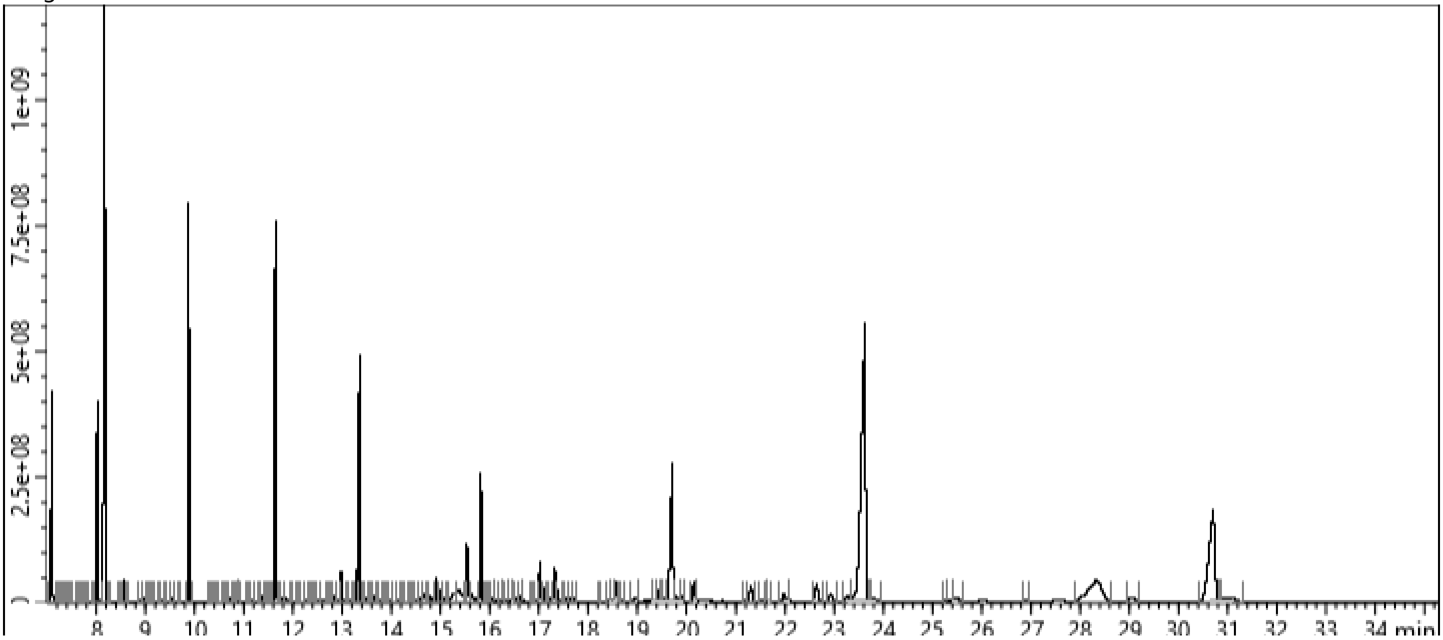


# Spectra

**Silane, (4-methoxyphenyl)trimethyl-**

Peak: 7.06 - 7.14 min (scan #16 - #39) Subtracted noise: 7.00 - 7.00 min (scan #0 - #1), 7.18 - 7.26 min (scan #51 - #73)

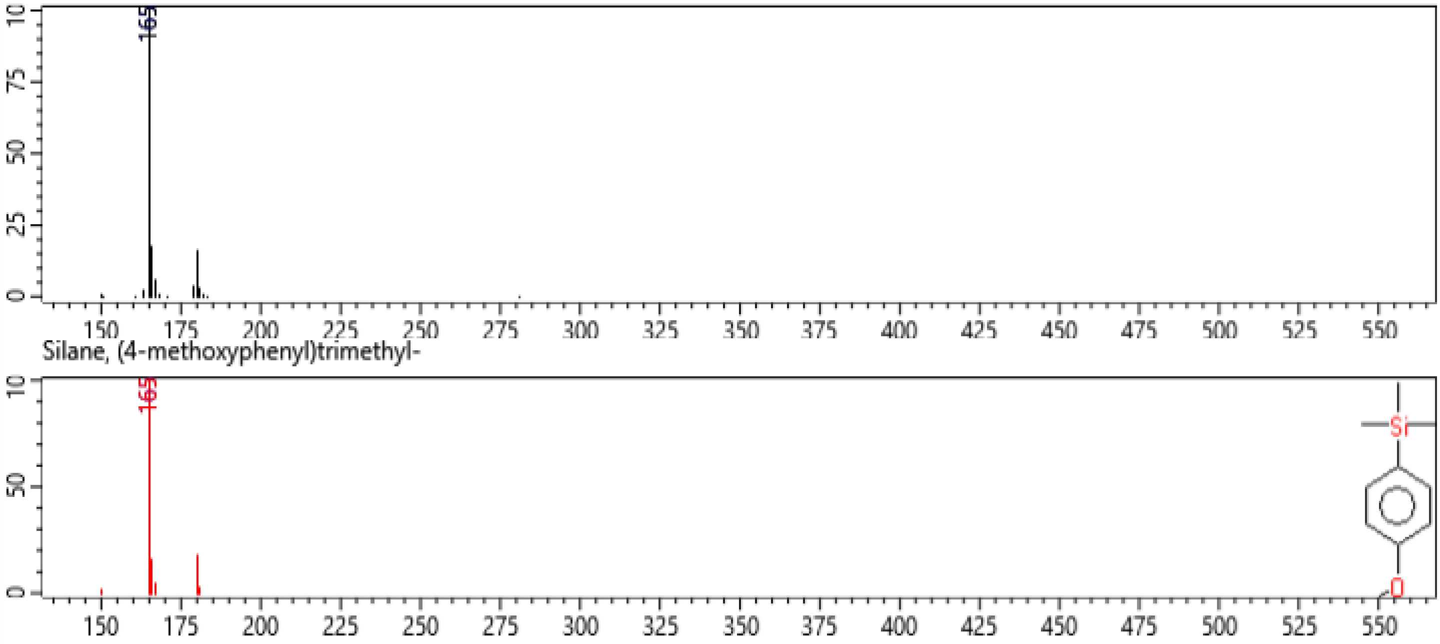

m-Cresol, TMS derivative

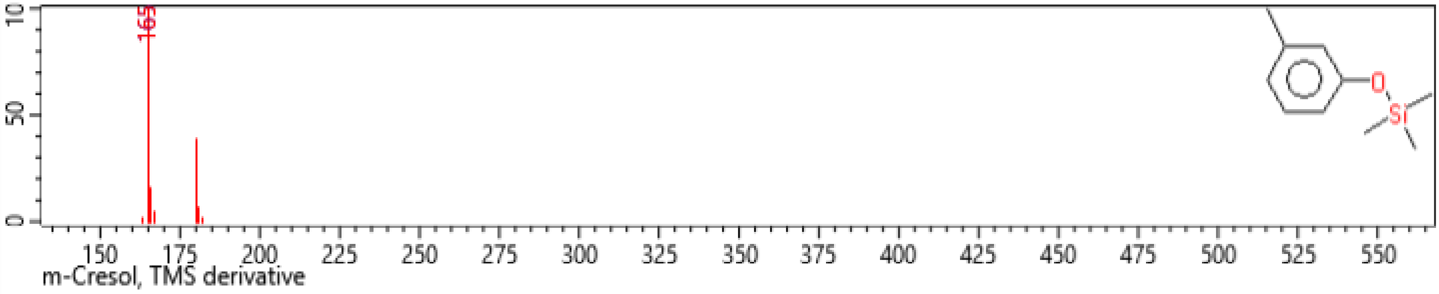


m-Cresol, TMS derivative

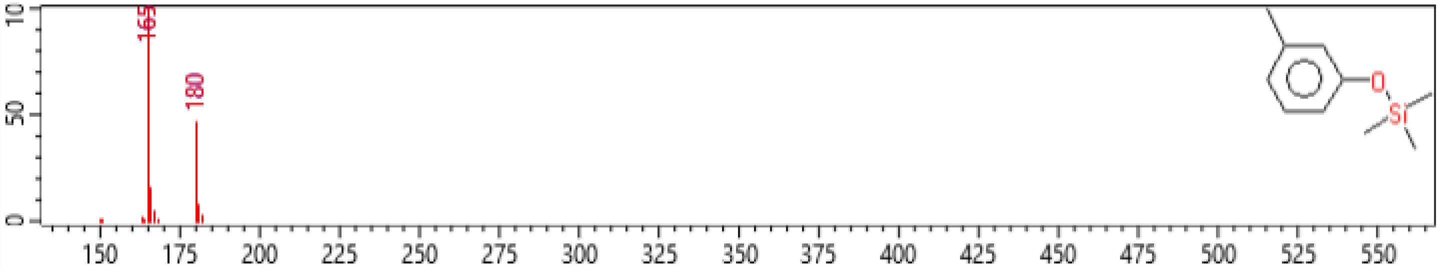


m-Cresol, TMS derivative

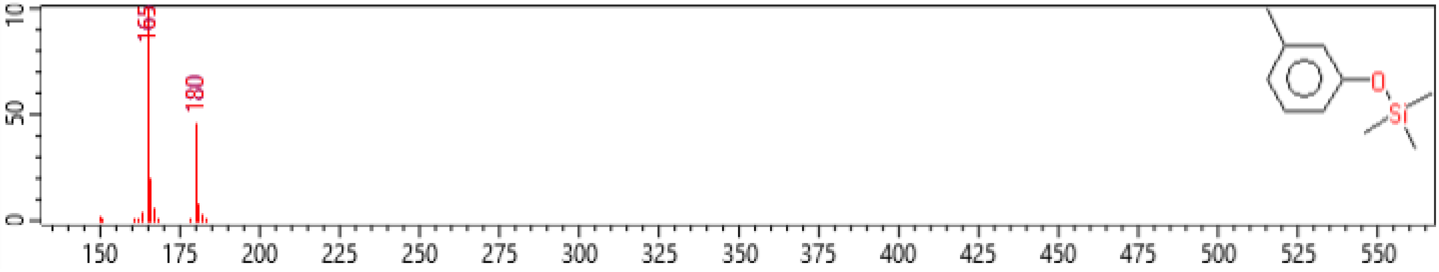


p-Cresol, TMS derivative

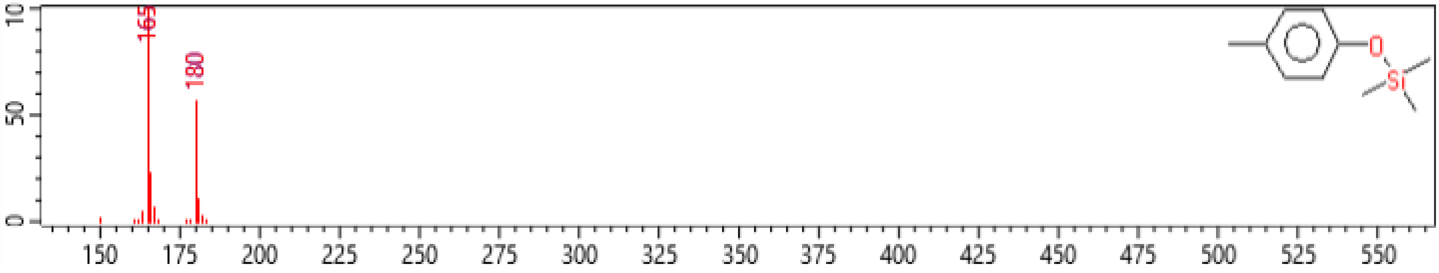


**O-(Desmorpholinopropyl)gefitinib**

Peak: 7.18 - 7.20 min (scan #50 - #56) Subtracted noise: 7.16 - 7.18 min (scan #44 - #50), 7.23 - 7.25 min (scan #65 - #71)

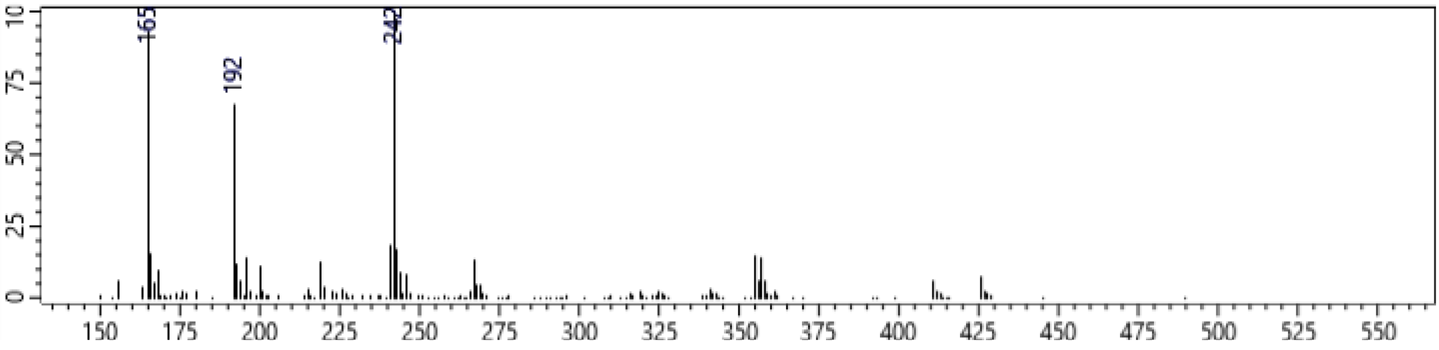

O-(Desmorpholinopropyl)gefitinib

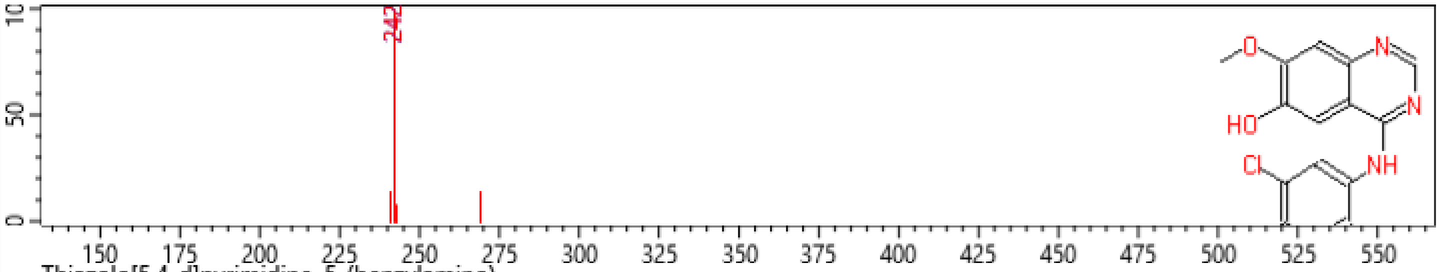


Thiazolo[5,4-d]pyrimidine, 5-(benzylamino)-

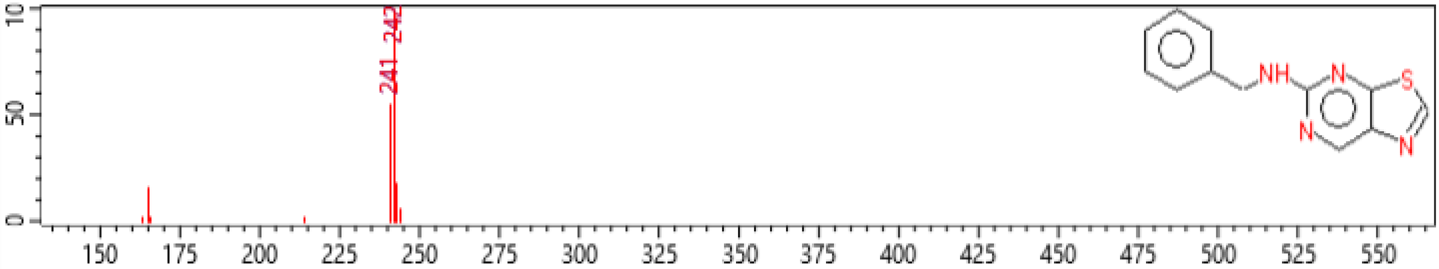


Methanone, [1-(4-hydroxypentyl)-1H-indol-3-yl](4-hydroxyphenyl)-

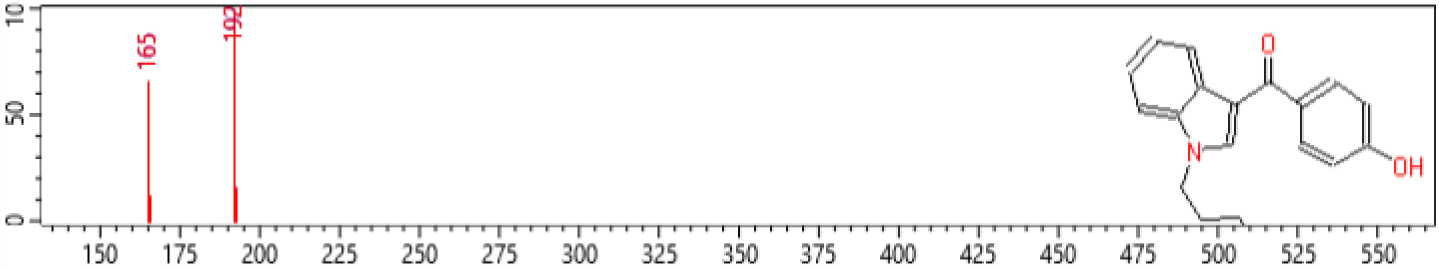


Benzaldehyde, (4-methoxy-6-methyl-2-pyrimidinyl)hydrazone

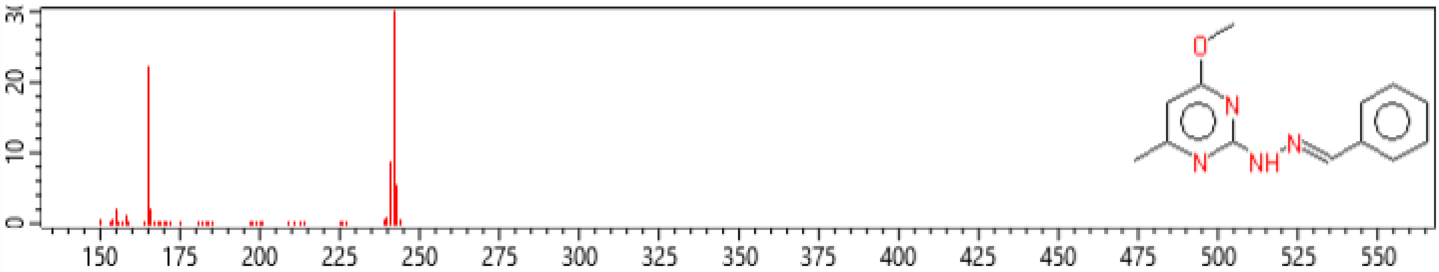


2-(4-Phenylphenyl)-N-(pyridin-2-yl)acetamide

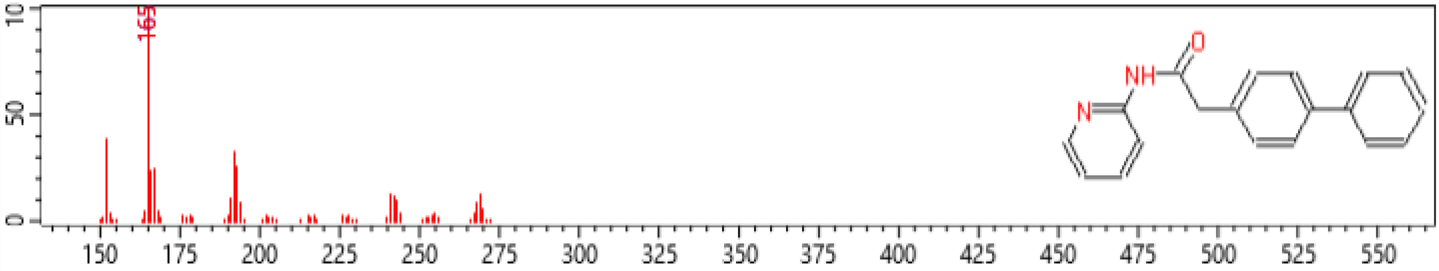

**4-[2-(4-Formyl-2-methoxyphenoxy)ethoxy]-3-methoxybenzaldehyde**

Peak: 7.25 - 7.26 min (scan #69 - #73) Subtracted noise: 7.21 - 7.23 min (scan #58 - #64), 7.27 - 7.29 min (scan #76 - #82)

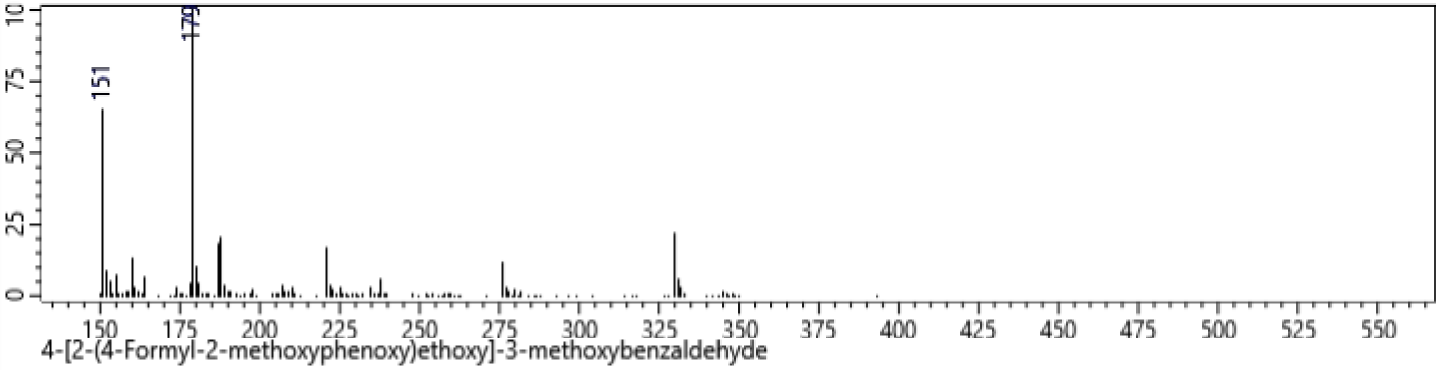


4-[2-(4-Formyl-2-methoxyphenoxy)ethoxy]-3-methoxybenzaldehyde

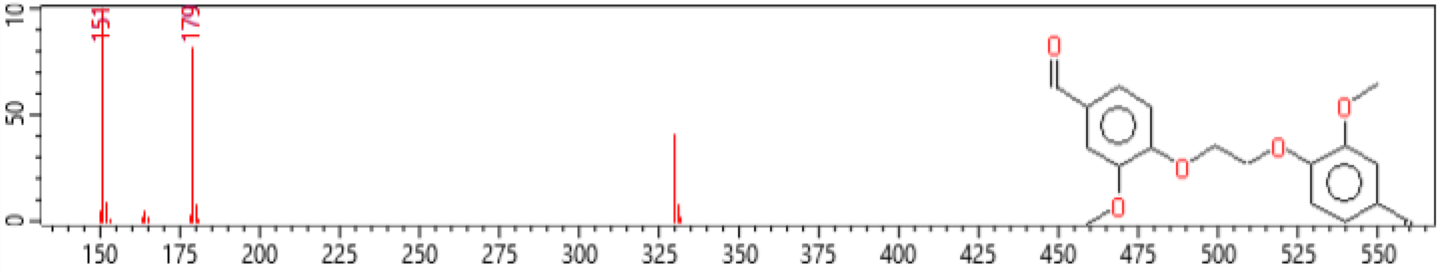


2-(2-Hydroxyphenoxy)-N-(2-methoxyphenyl)acetamide, TBDMS derivative

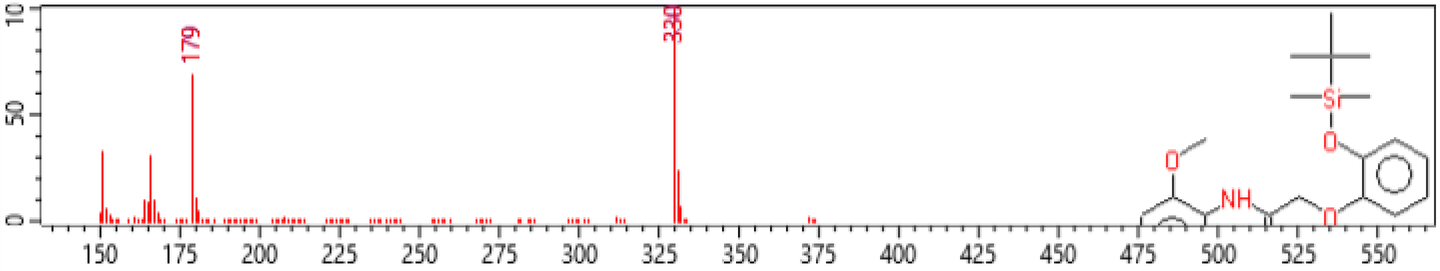


3-{[(1Z)-4-(4-Hydroxyphenyl)phthalazin-1(2H)-ylidene]amino}phenol

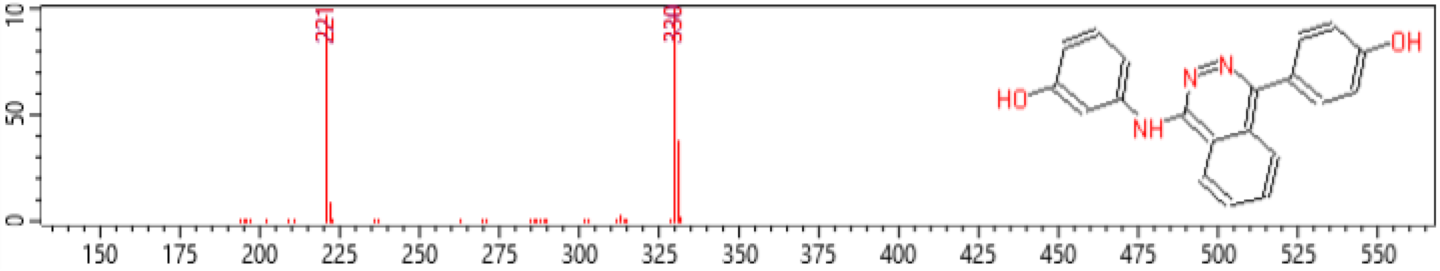


7-Chloro-4-hydroxyquinoline, acetate

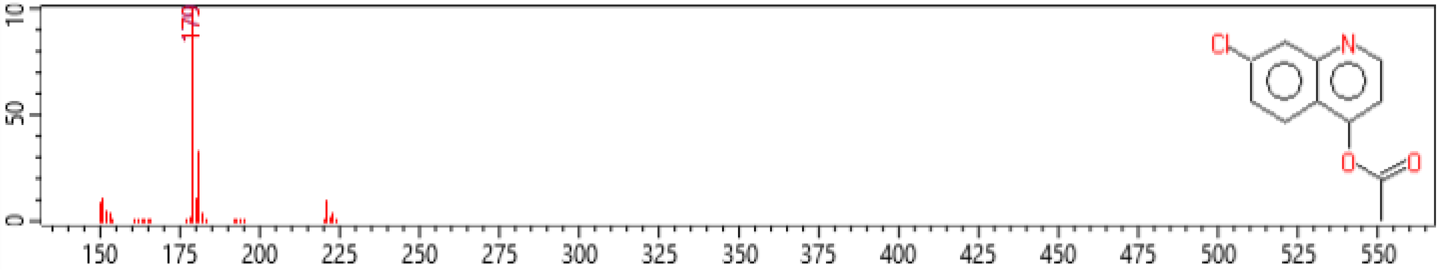

Tiocarlide

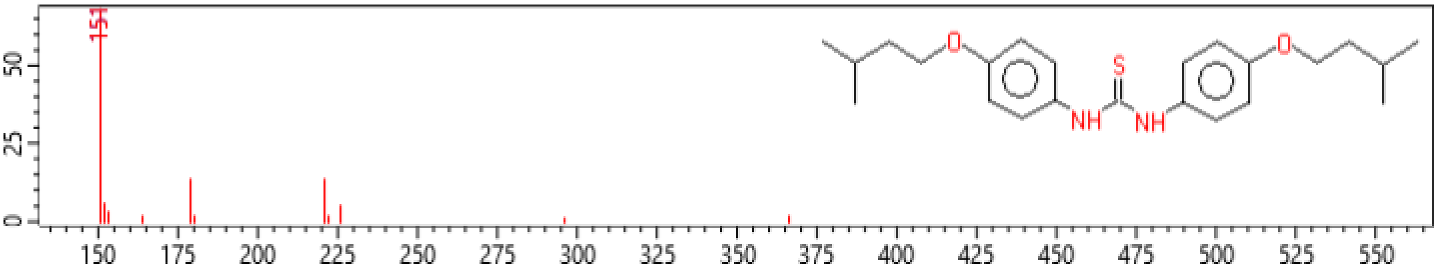


**Pentasiloxane, dodecamethyl-**

Peak: 7.32 - 7.33 min (scan #88 - #92) Subtracted noise: 7.29 - 7.30 min (scan #81 - #84), 7.34 - 7.35 min (scan #94 - #98)

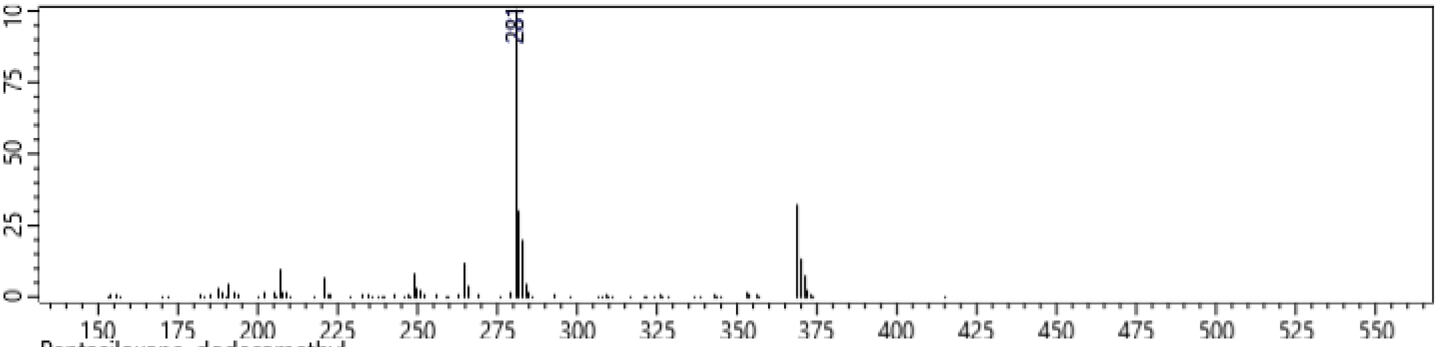


Pentasiloxane, dodecamethyl-

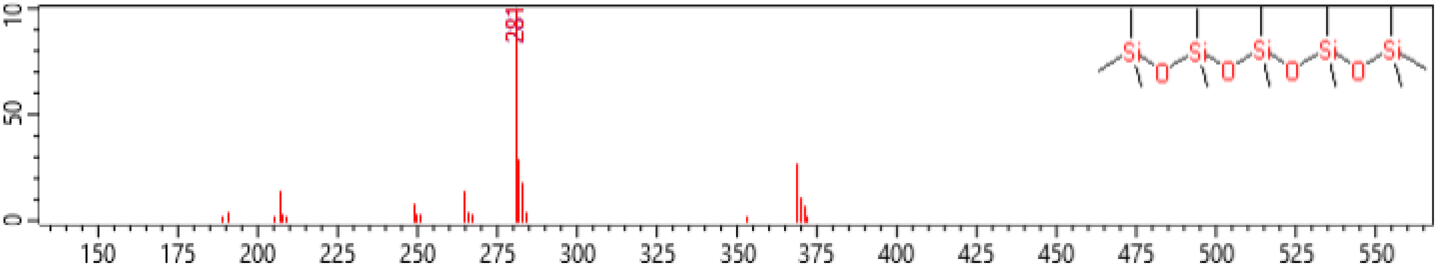


Pentasiloxane, dodecamethyl-

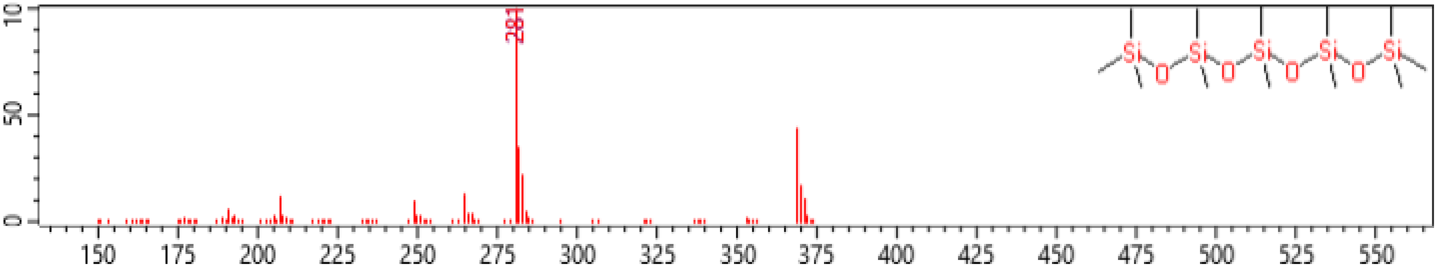


Pentasiloxane, dodecamethyl-

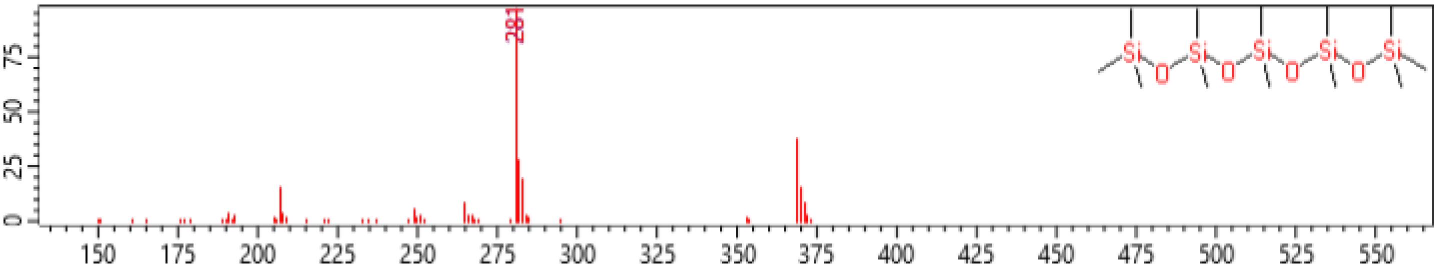

Pentasiloxane, dodecamethyl-

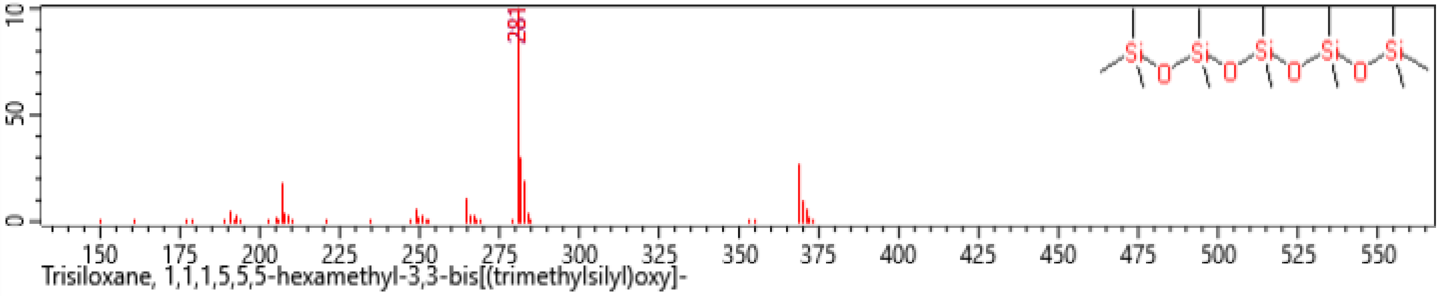


Trisiloxane, 1,1,1,5,5,5-hexamethyl-3,3-bis[(trimethylsilyl)oxy]-

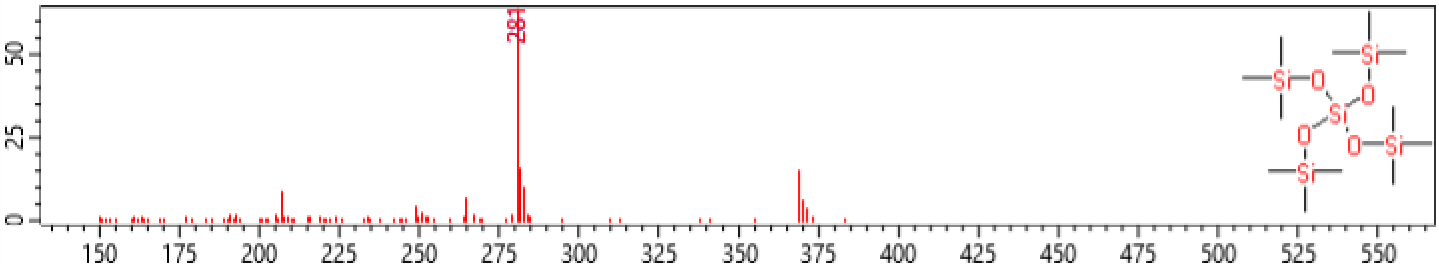


**Silane, (dibromomethyl)trimethyl-**

Peak: 7.37 - 7.38 min (scan #103 - #106) Subtracted noise: 7.34 - 7.36 min (scan #96 - #100), 7.39 - 7.40 min (scan #109 - #111)

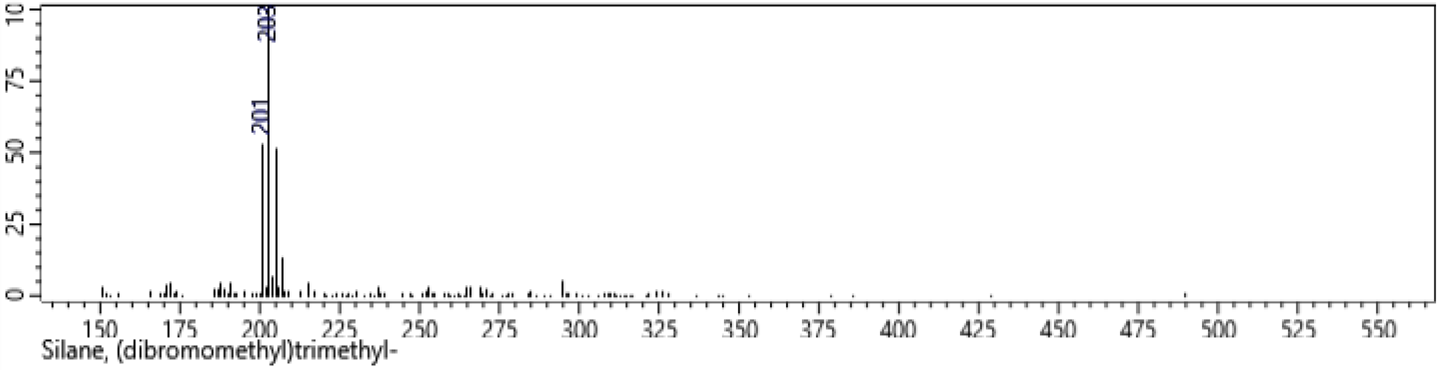


Silane, (dibromomethyl)trimethyl-

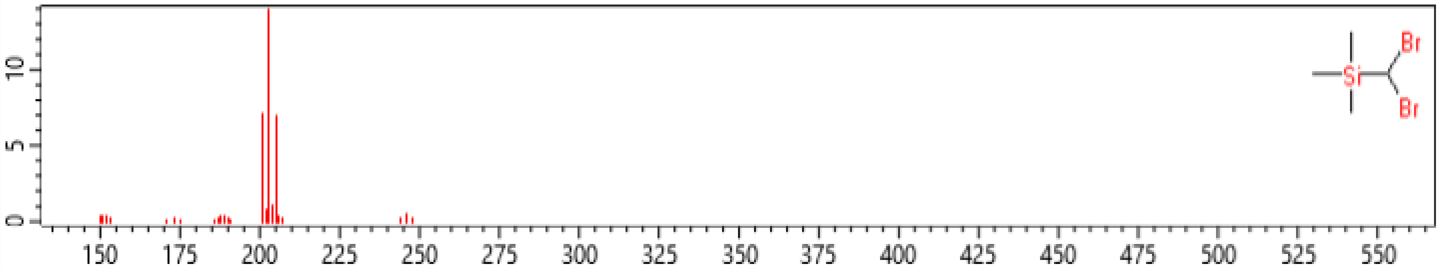


Silane, (dibromomethyl)trimethyl-

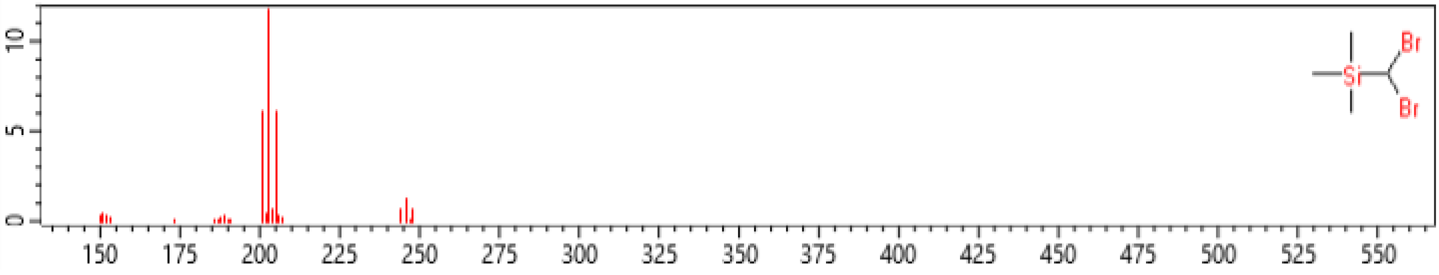

Ethane, 2-bromo-2-chloro-1,1,1-trimethoxy-

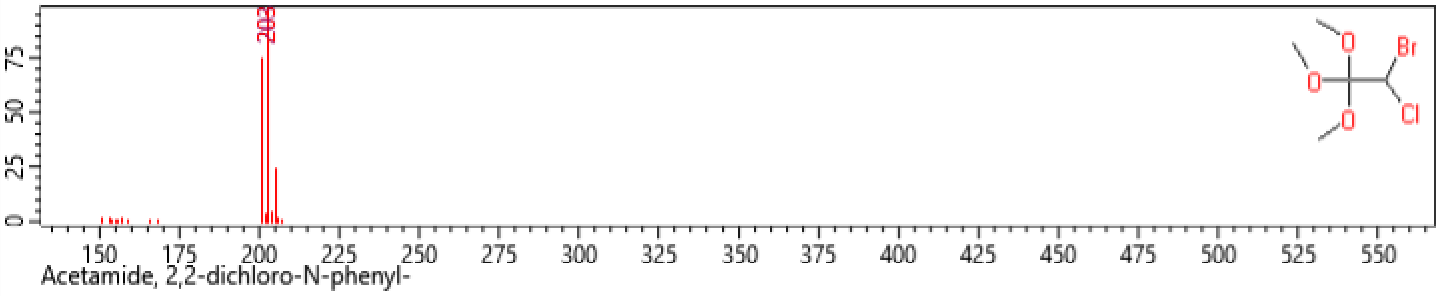


Acetamide, 2,2-dichloro-N-phenyl-

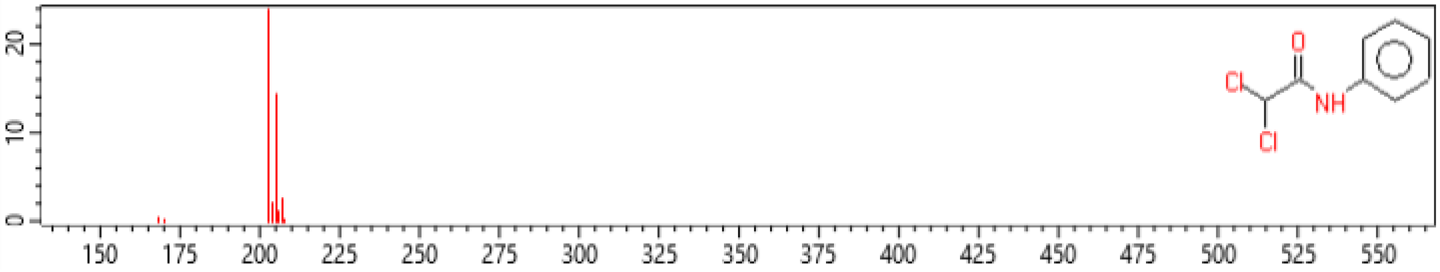


Ethanol, 2,2,2-tribromo-

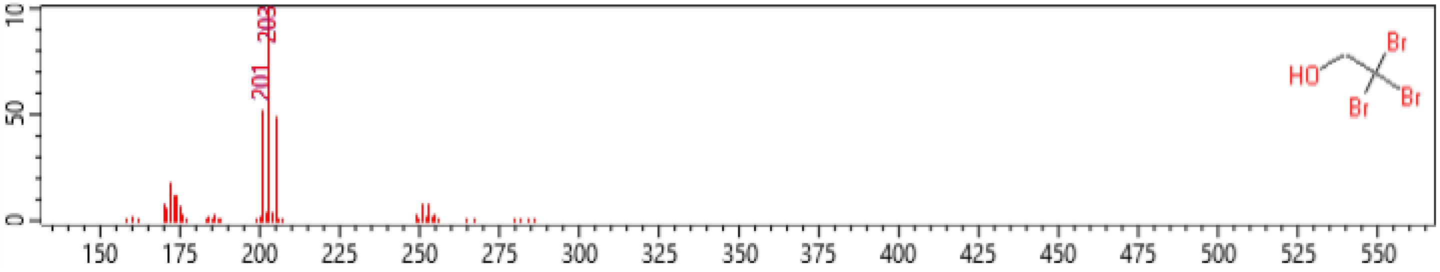


**13,14-Dihydro-15(R)-prostaglandin E1**

Peak: 7.45 - 7.46 min (scan #125 - #129) Subtracted noise: 7.41 - 7.43 min (scan #114 - #119), 7.47 - 7.49 min (scan #132 - #136)

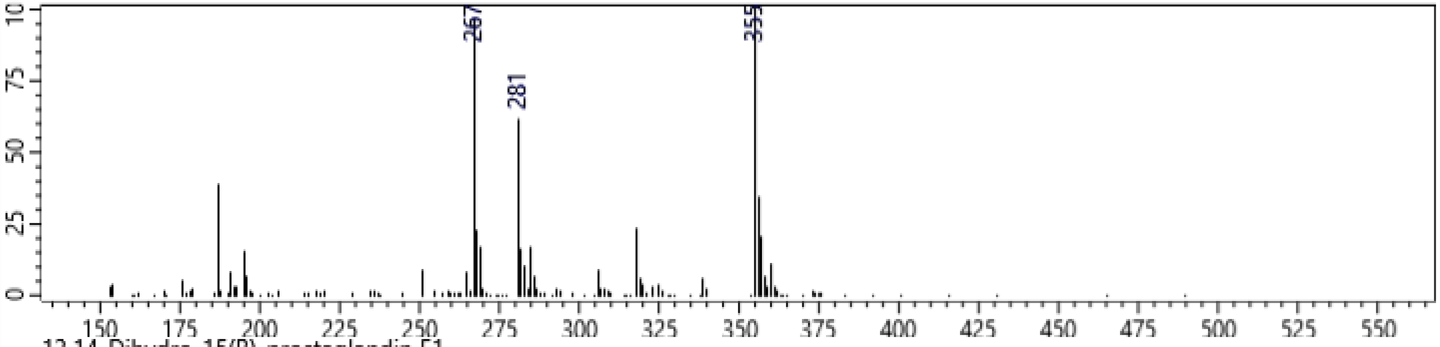


13,14-Dihydro-15(R)-prostaglandin E1

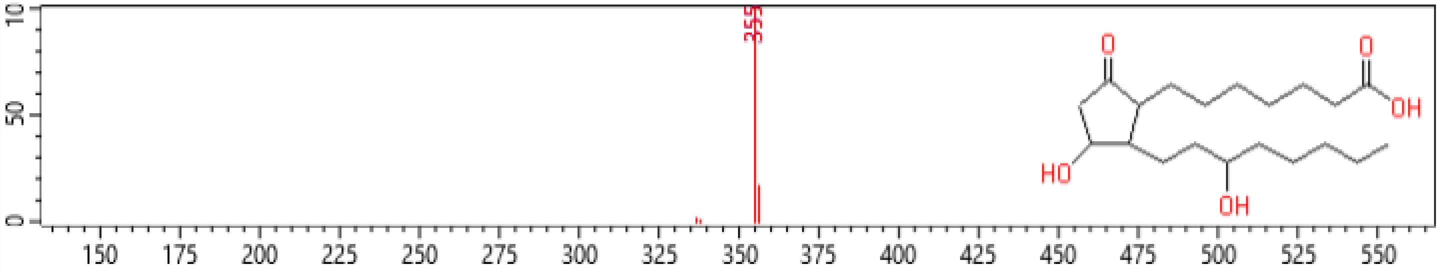

13,14-Dihydro-15(R)-prostaglandin E1

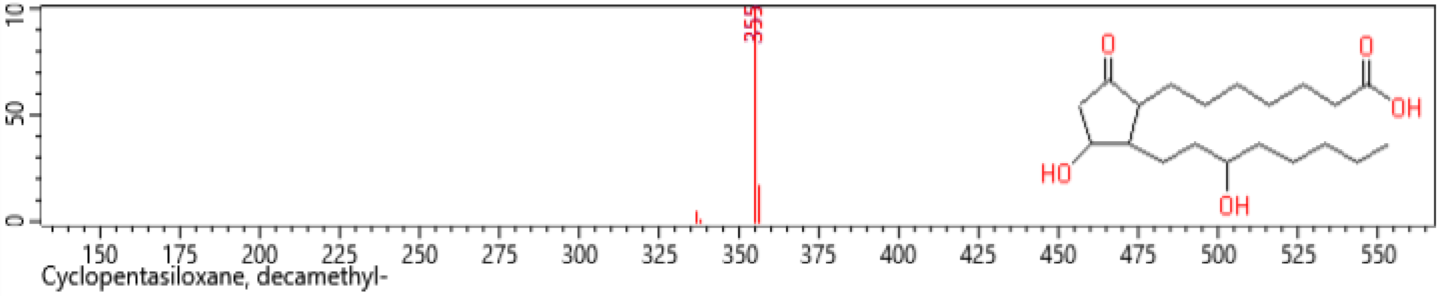


Cyclopentasiloxane, decamethyl-

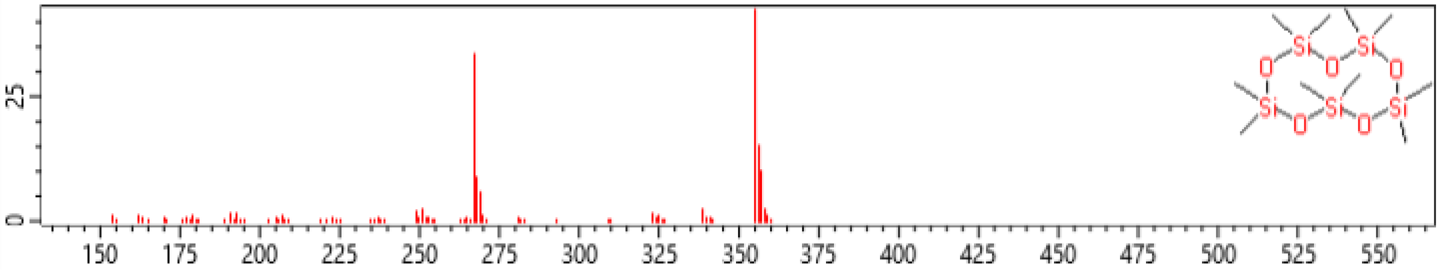

Cyclopentasiloxane, decamethyl-

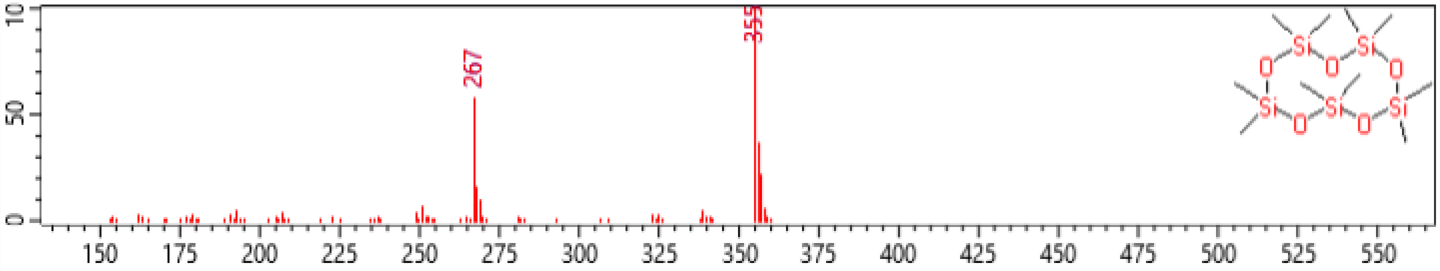


Cyclopentasiloxane, decamethyl-

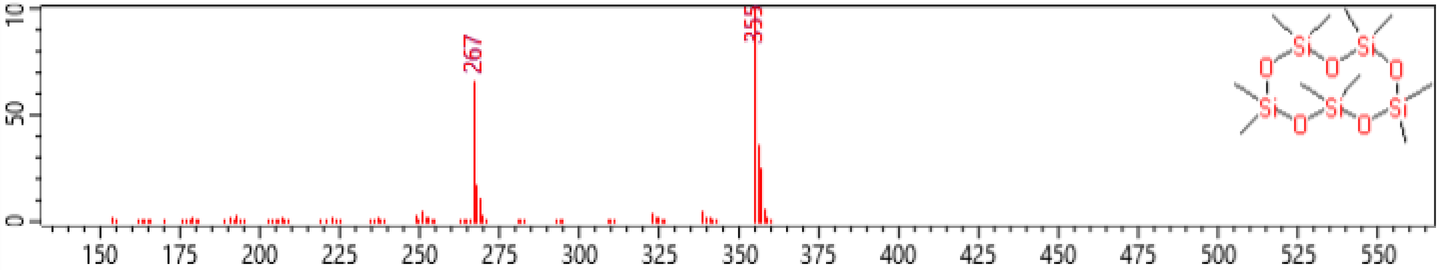


**4,5-thiazolidinedione, 3-ethyl-2-thioxo-, 5-[2-[3-methyl-2(3H)-benzothiazolylidene]hydrazone]**
Peak: 7.48 - 7.50 min (scan #134 - #140) Subtracted noise: 7.45 - 7.47 min (scan #125 - #131), 7.52 - 7.54 min (scan #145 - #151)

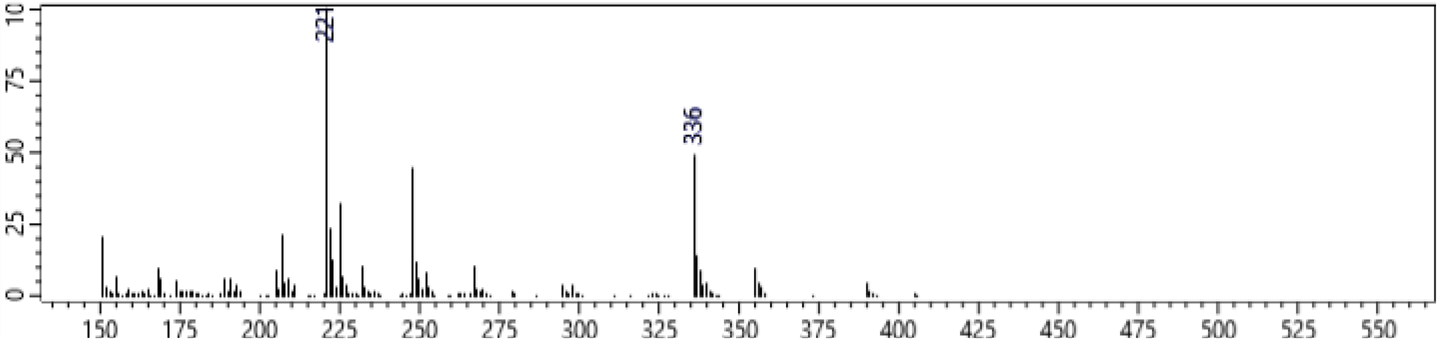

4,5-thiazolidinedione, 3-ethyl-2-thioxo-, 5-[2-[3-methyl-2(3H)-benzothiazolylidene]hydrazone]

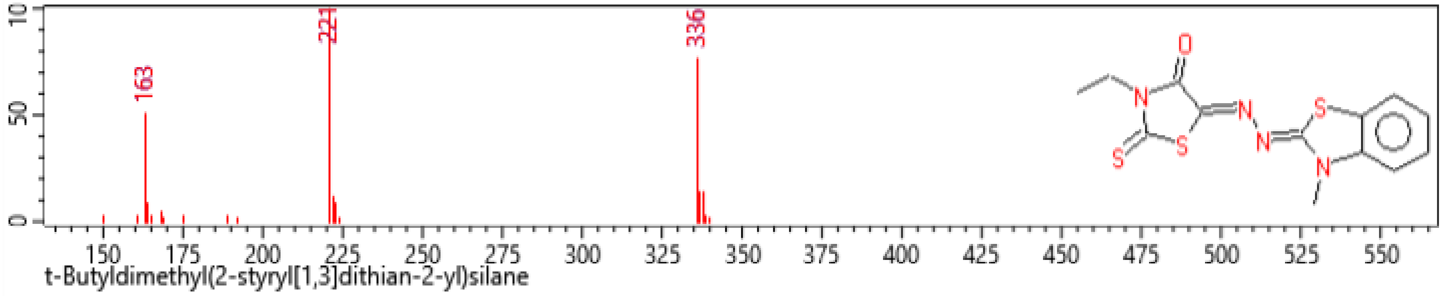


t-Butyldimethyl(2-styryl[1,3]dithian-2-yl)silane

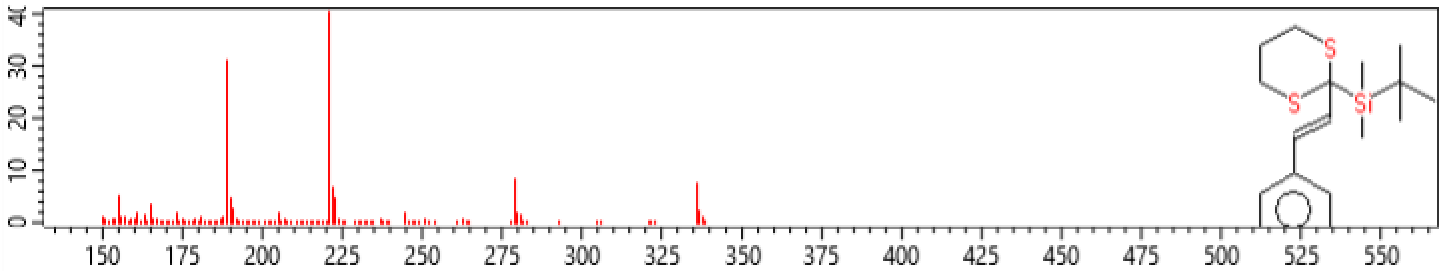

2,3-Dimercaptobutane-1,4-diol, 4TMS

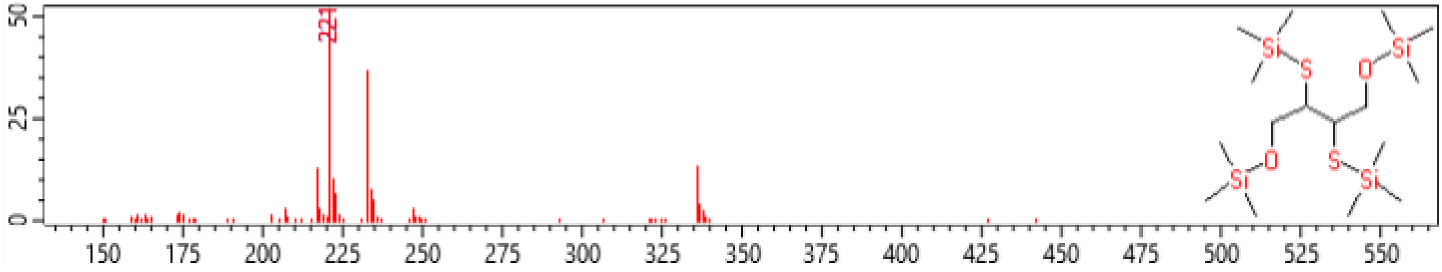


1-(4-Chlorophenyl)-2-phenyl-4-hydroxy-5-piperidino-4,5-dihydroimidazole

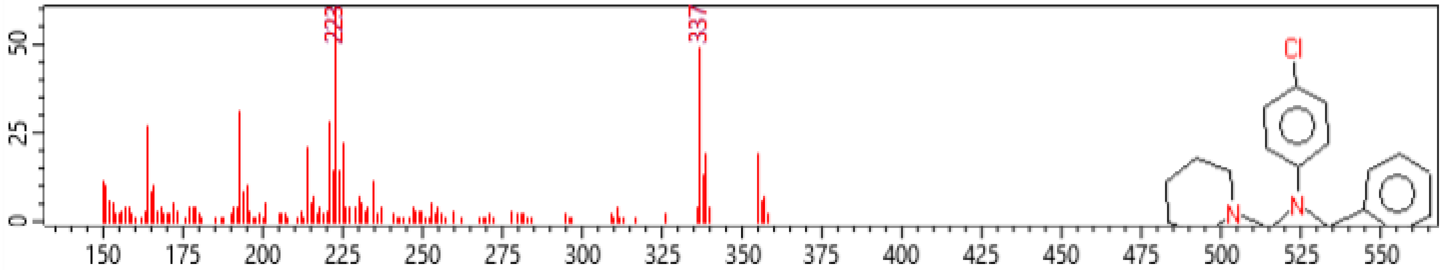


Silane, (3,3-diphenyl-1,2-propadienylidene)bis[trimethyl-

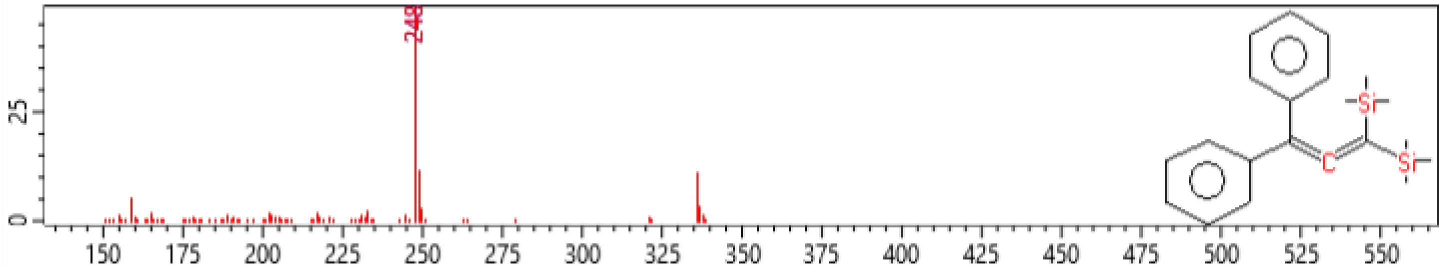

**Thiophene-2-acetic acid, 2-methylbutyl ester**

Peak: 7.53 - 7.56 min (scan #147 - #155) Subtracted noise: 7.49 - 7.52 min (scan #136 - #144), 7.59 - 7.62 min (scan #165 - #173)

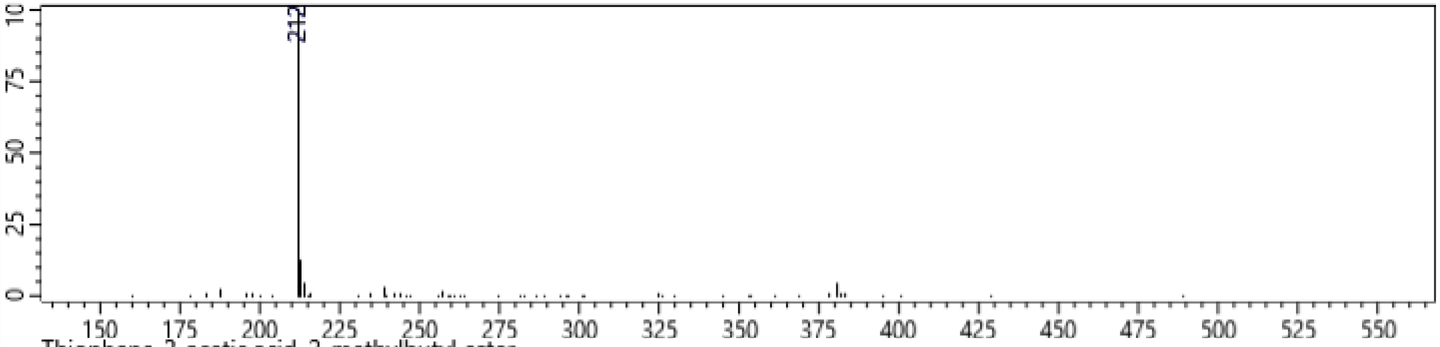


Thiophene-2-acetic acid, 2-methylbutyl ester

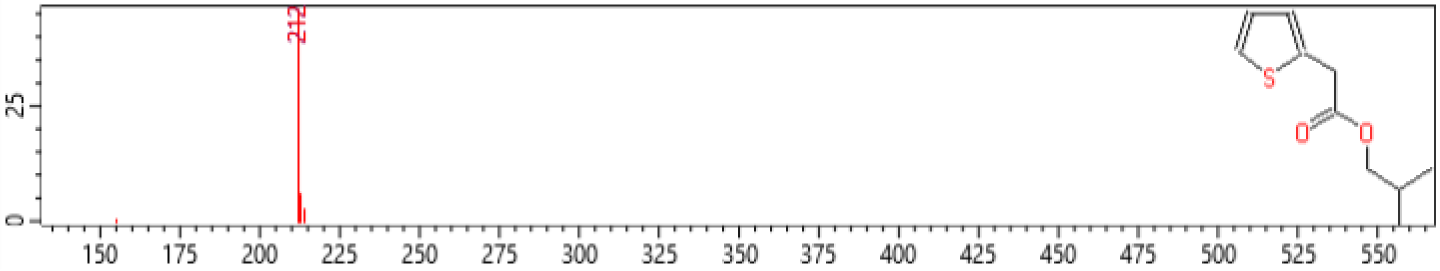


S-[2-Aminophenyl]-dl-cysteine

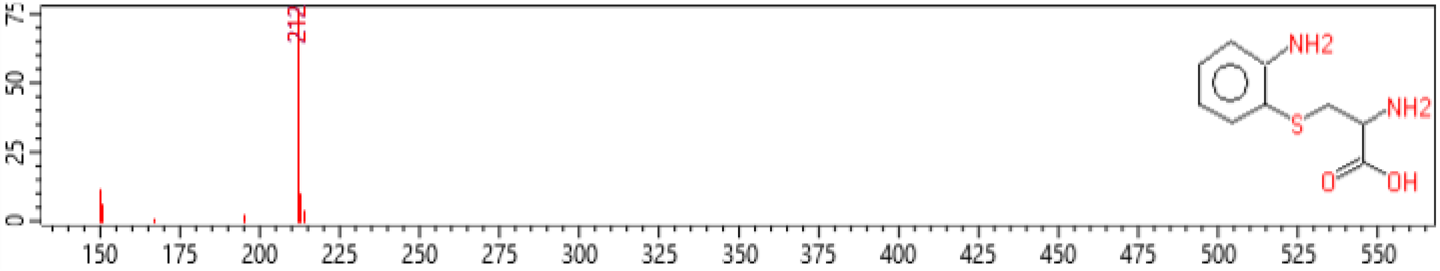


Benzofuran, 2,3-dihydro-2-[(methylsulfonyl)methyl]-

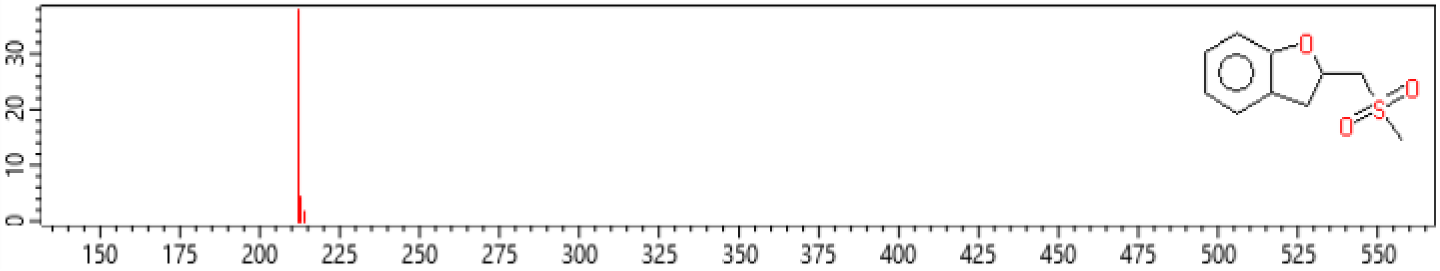


Benzenesulfonamide, 4-methyl-N-ethyl-N-2-ethylhexyl-

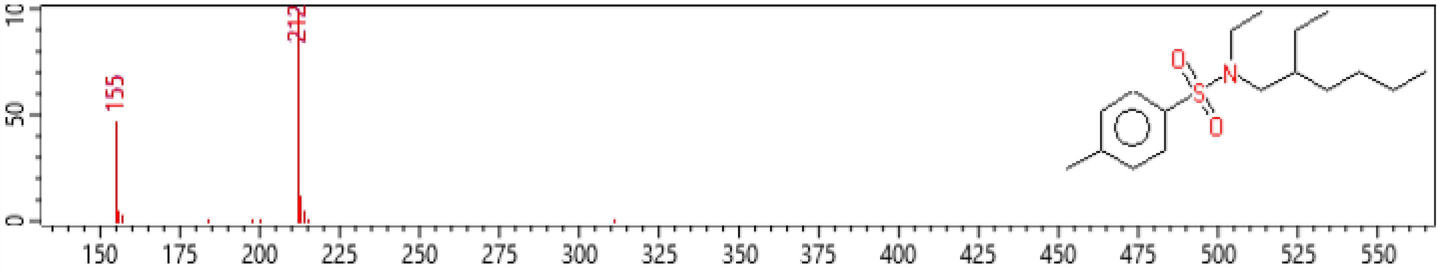

2-Thiopheneacetic acid, 2,2-dimethylpropyl ester

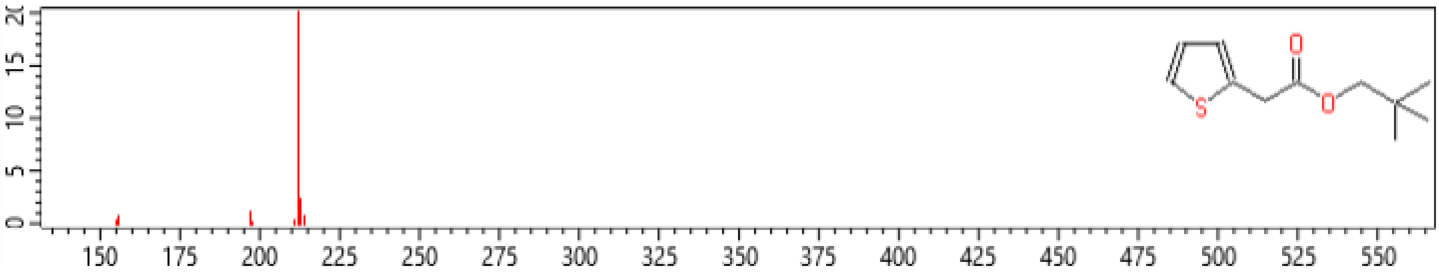

**7,7,9,9,11,11-Hexamethyl-3,6,8,10,12,15-hexaoxa-7,9,11-trisilaheptadecane**
Peak: 7.61 - 7.62 min (scan #169 - #173) Subtracted noise: 7.57 - 7.59 min (scan #160 - #165), 7.63 - 7.65 min (scan #177 - #181)

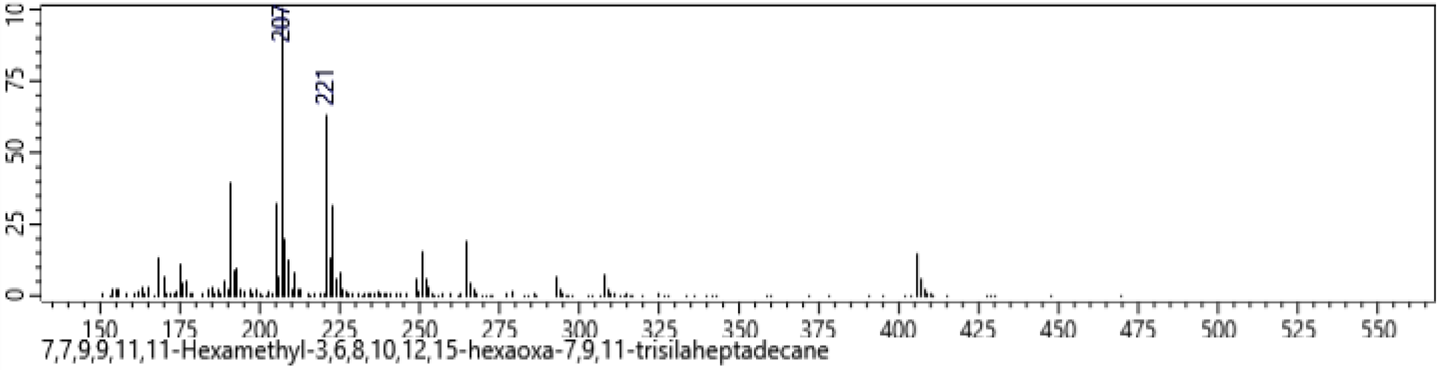


7,7,9,9,11,11-Hexamethyl-3,6,8,10,12,15-hexaoxa-7,9,11-trisilaheptadecane

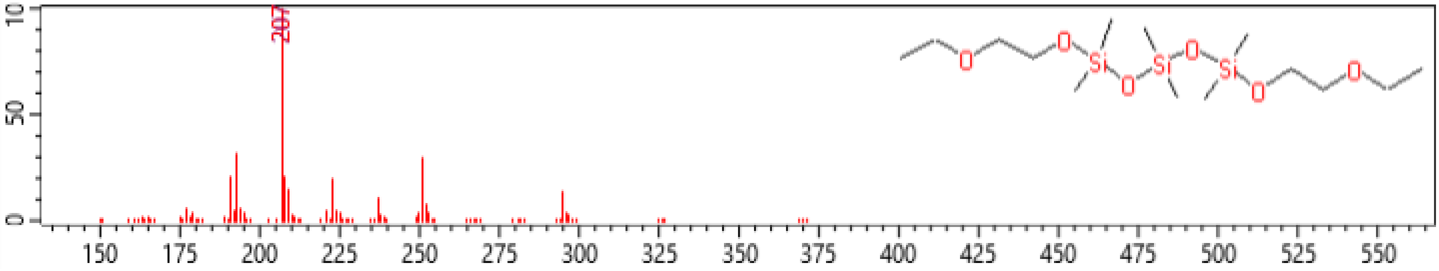


2-(1H-Benzimidazol-2-ylsulfanyl)-N,N-diethylethanamine, TMS derivative

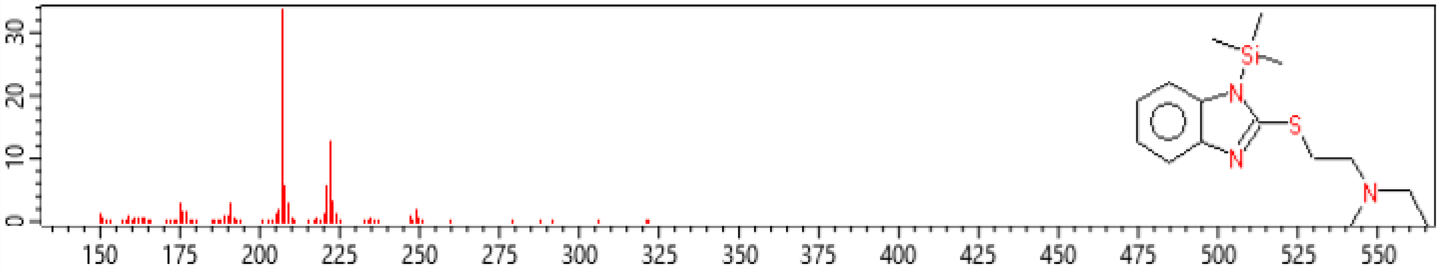

2-[(2-Phenoxyethyl)thio]-1H-benzimidazole, TMS

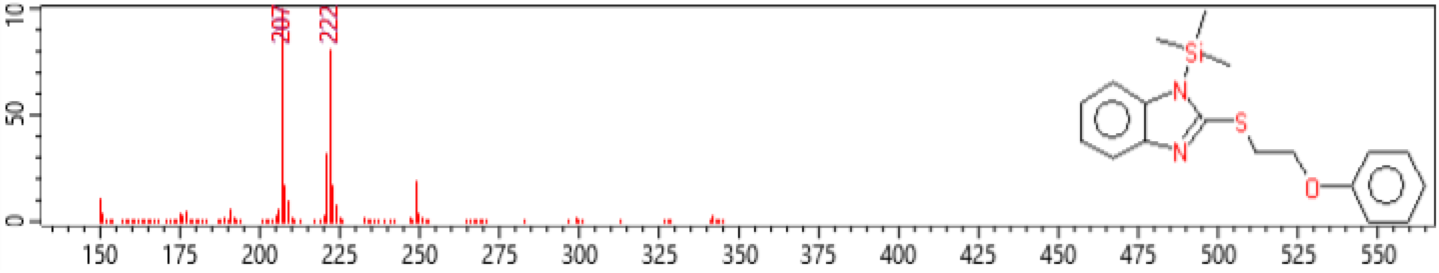

4-Amino-2-fluoro-5-methylbenzonitrile, TMS

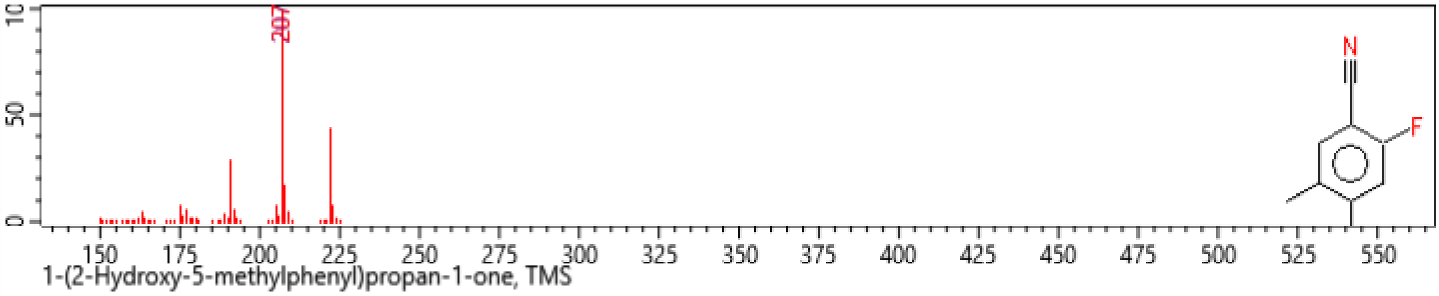


1-(2-Hydroxy-5-methylphenyl)propan-1-one, TMS

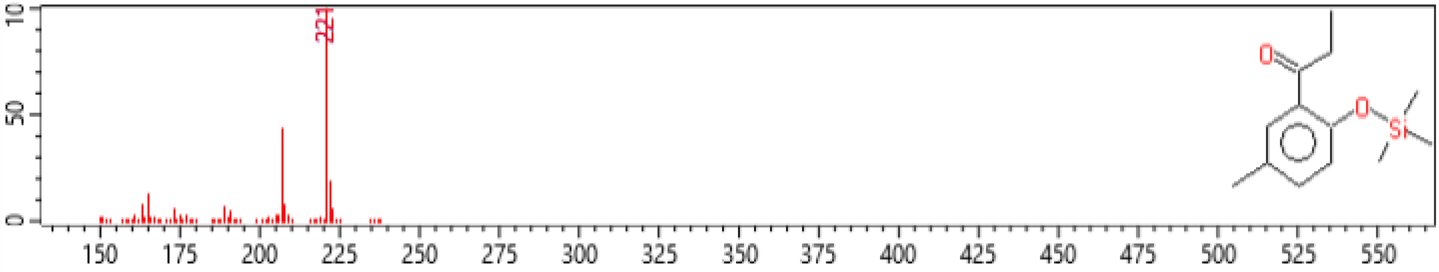


**t-Butyldimethyl(2-styryl[1,3]dithian-2-yl)silane**

Peak: 7.69 - 7.72 min (scan #192 - #202) Subtracted noise: 7.62 - 7.65 min (scan #173 - #182), 7.73 - 7.77 min (scan #205 - #214)

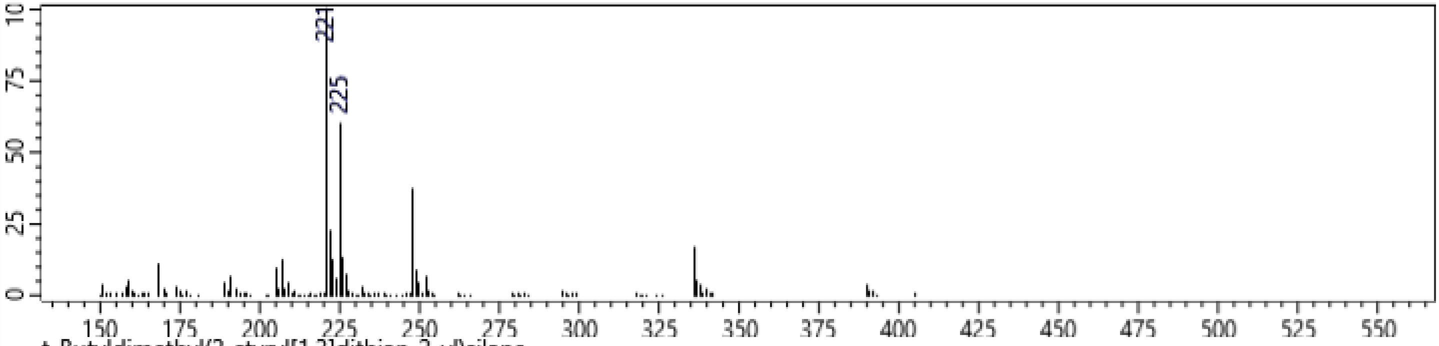


t-Butyldimethyl(2-styryl[1,3]dithian-2-yl)silane

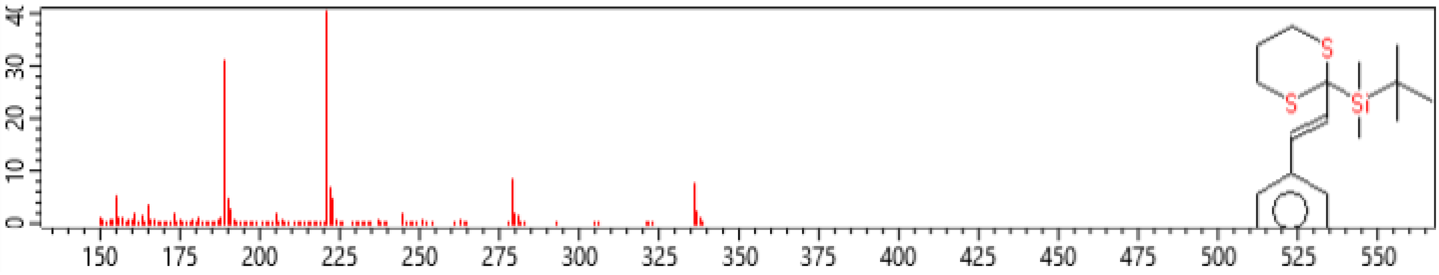


4-Pyrimidinethiol, 5-(4-chlorophenyl)-

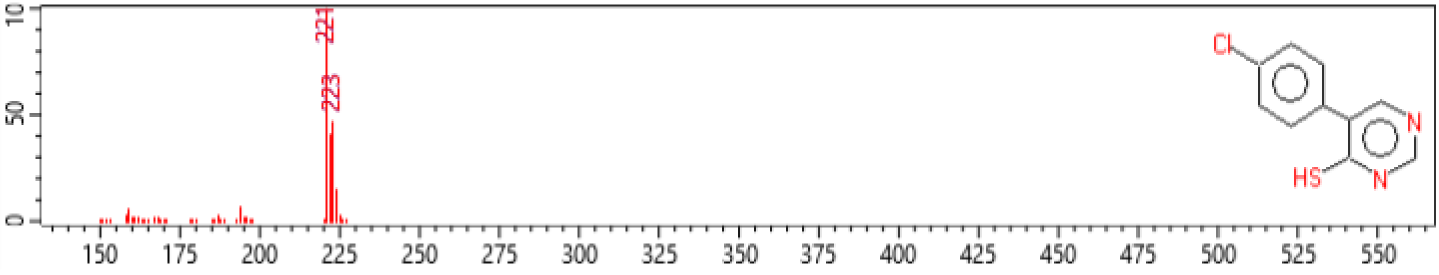

1,4-Benzenediol, 2,5-bis(1,1-dimethylpropyl)-

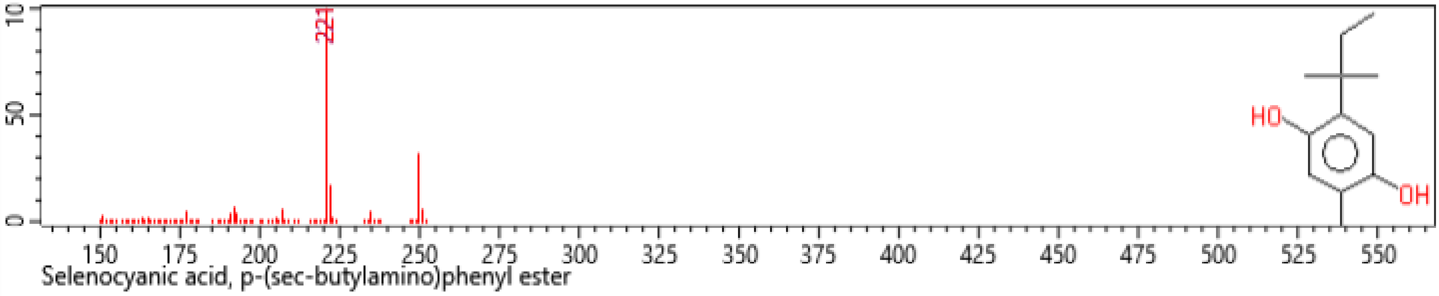


Selenocyanic acid, p-(sec-butylamino)phenyl ester

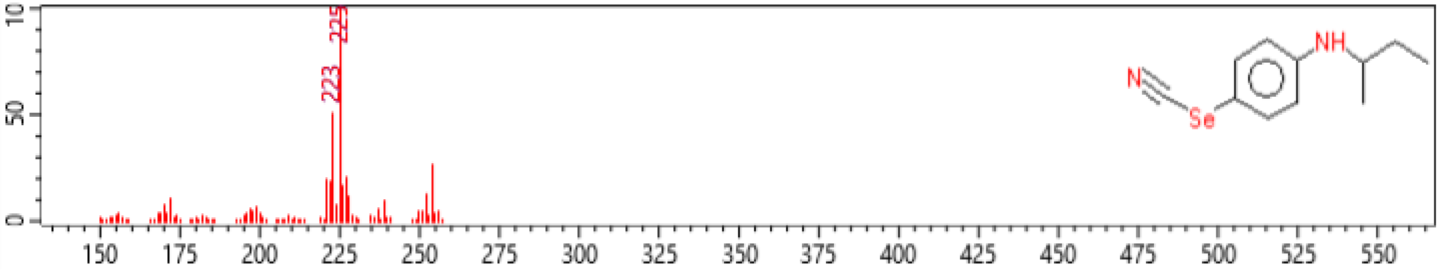


Triethyl(methylthio)stannane

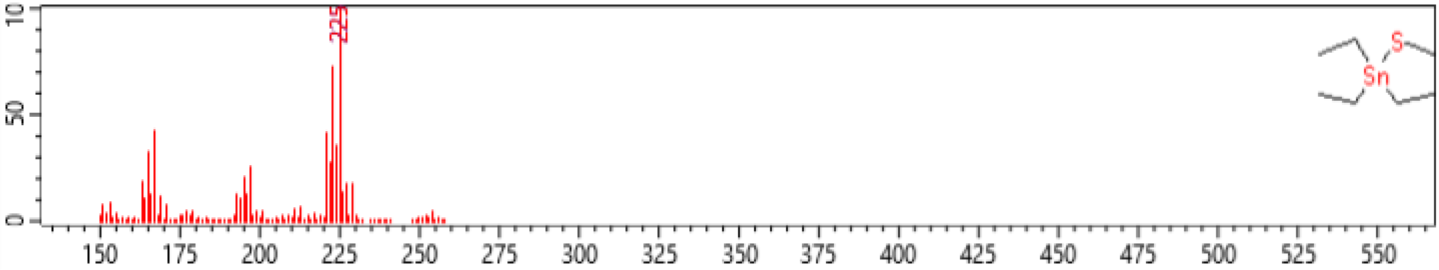


**2-(Methylsulfonyl)acetophenone**

Peak: 7.75 - 7.76 min (scan #208 - #212) Subtracted noise: 7.71 - 7.73 min (scan #199 - #204), 7.77 - 7.78 min (scan #215 - #218)

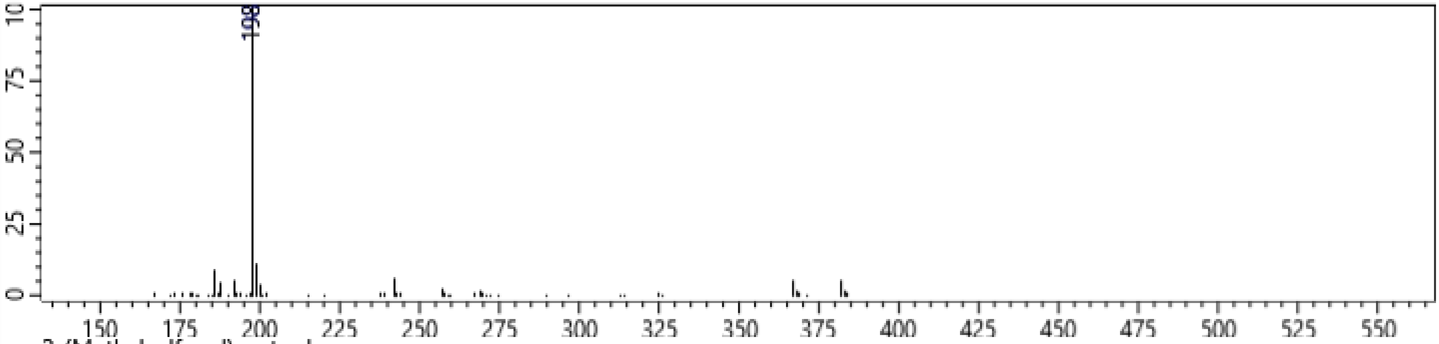


2-(Methylsulfonyl)acetophenone

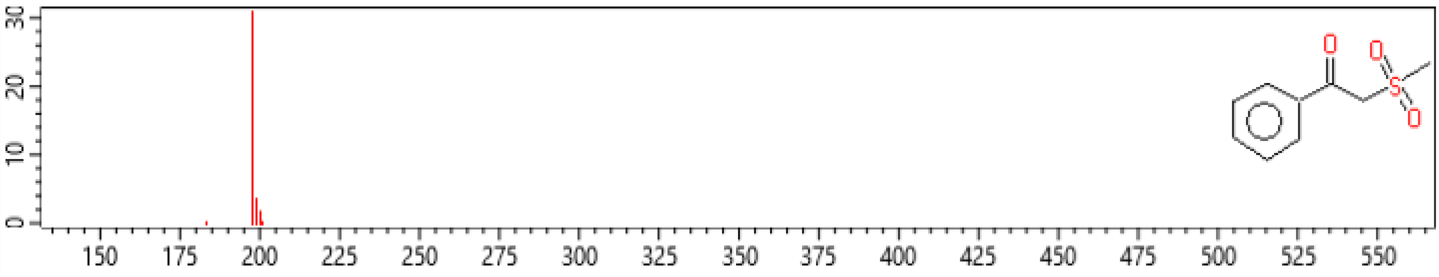

1-Aminocyclopentanecarboxylic acid, N-neopentyloxycarbonyl-, undecyl ester

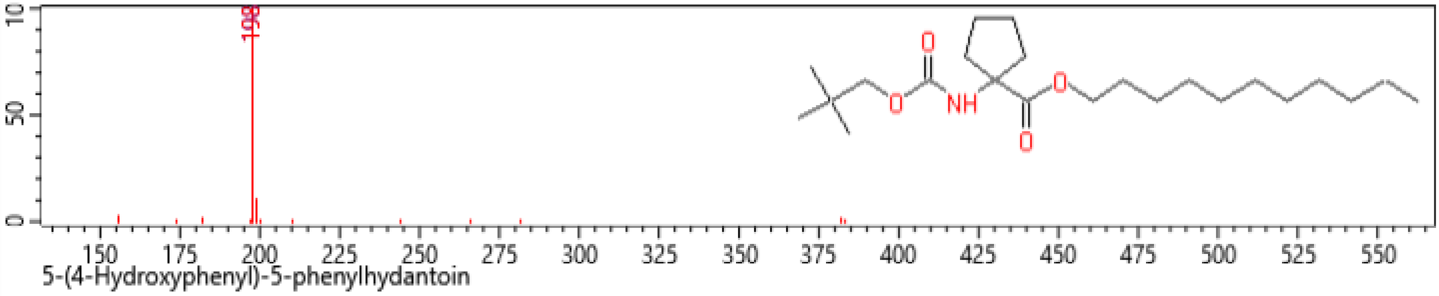


5-(4-Hydroxyphenyl)-5-phenylhydantoin

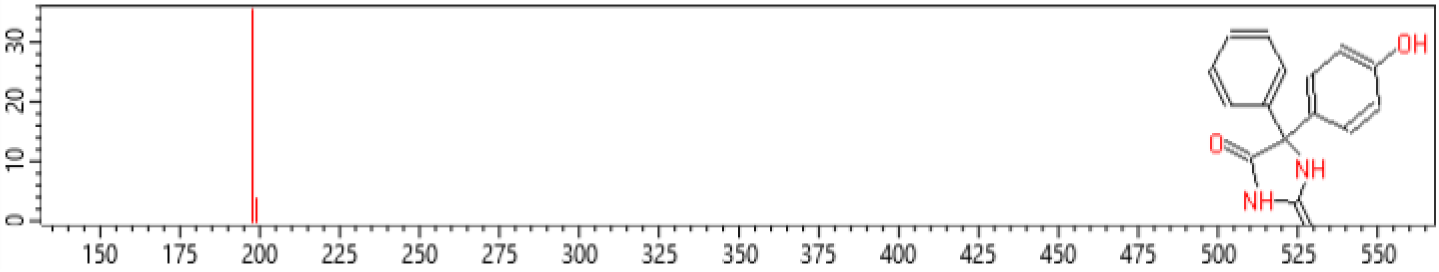

5-(4-Hydroxyphenyl)-5-phenylhydantoin

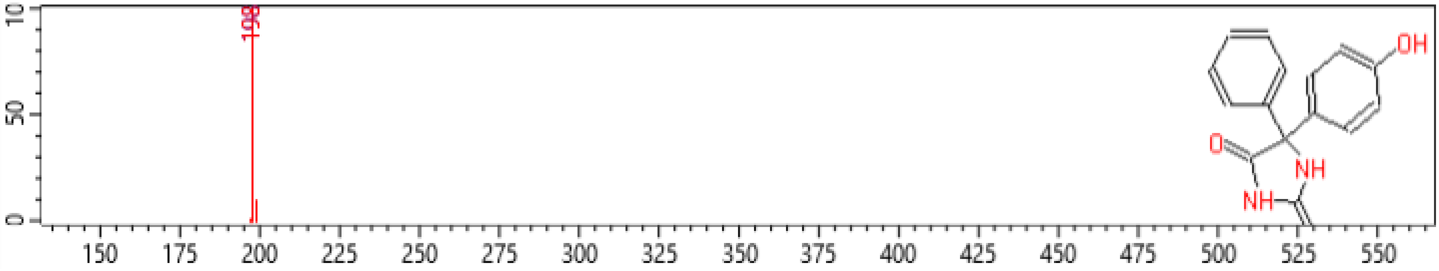


N-(tert-Butyl)benzenesulfonamide

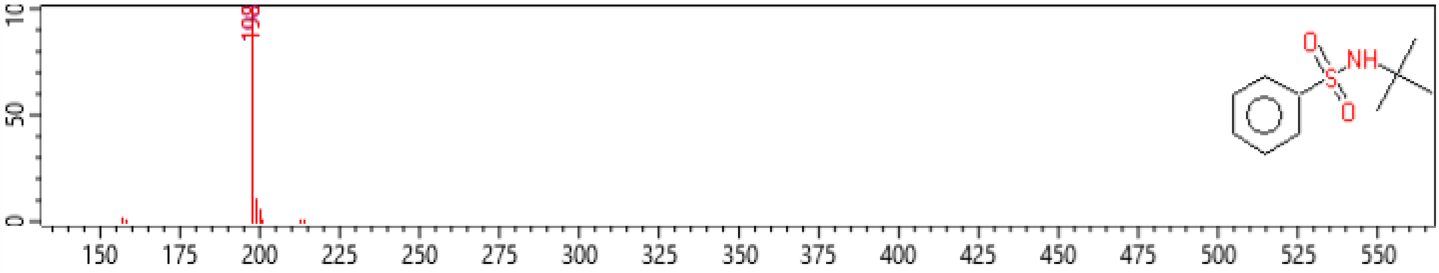


**Pentasiloxane, dodecamethyl-**

Peak: 7.81 - 7.83 min (scan #226 - #232) Subtracted noise: 7.77 - 7.79 min (scan #215 - #221), 7.85 - 7.87 min (scan #238 - #244)

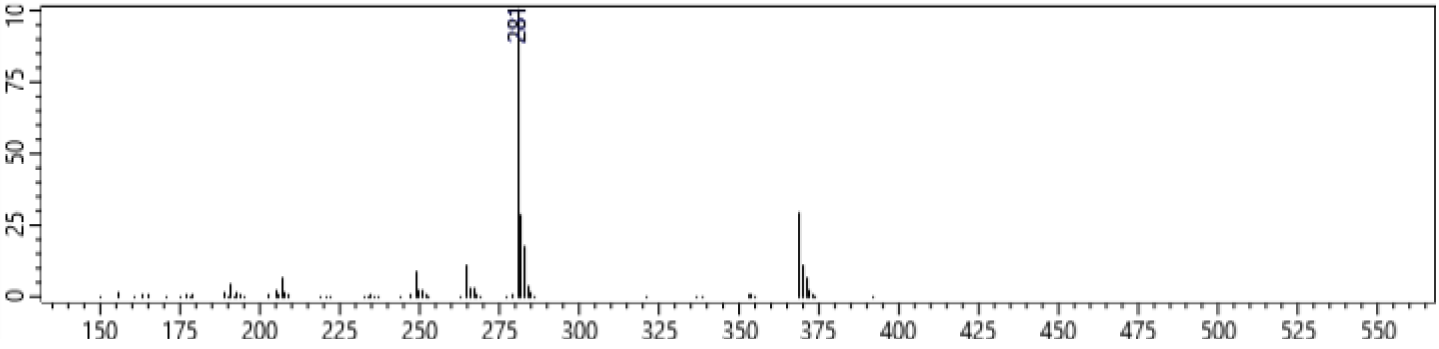

Pentasiloxane, dodecamethyl-

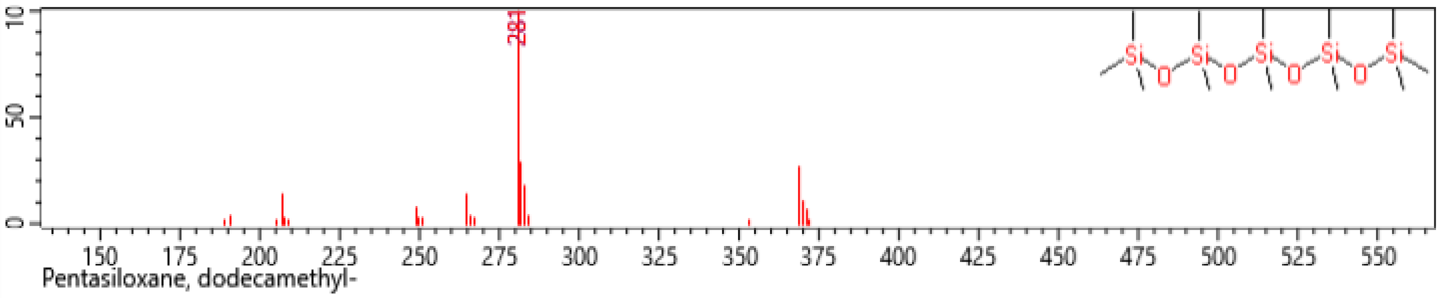


Pentasiloxane, dodecamethyl-

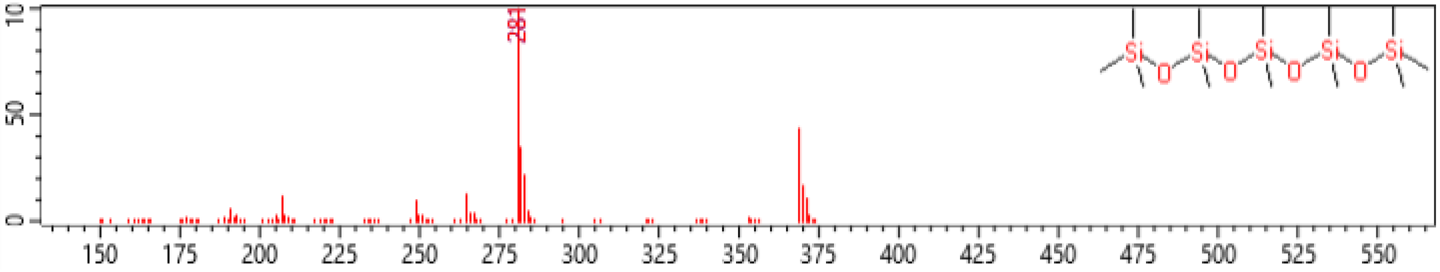


Pentasiloxane, dodecamethyl-

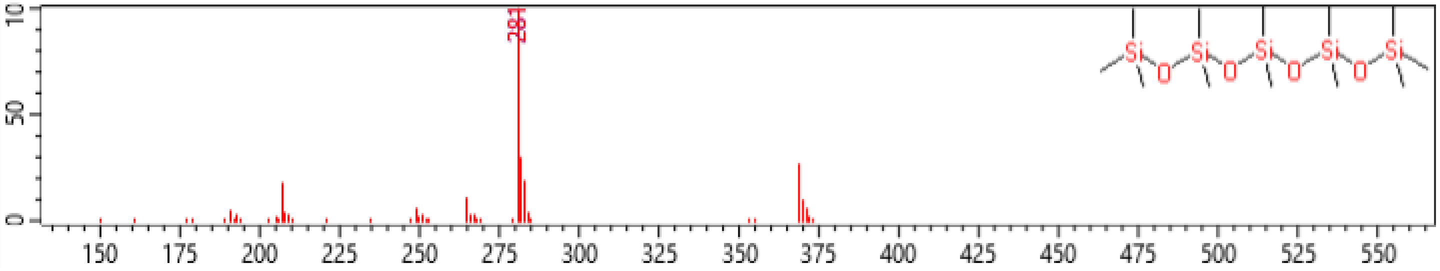


Pentasiloxane, dodecamethyl-

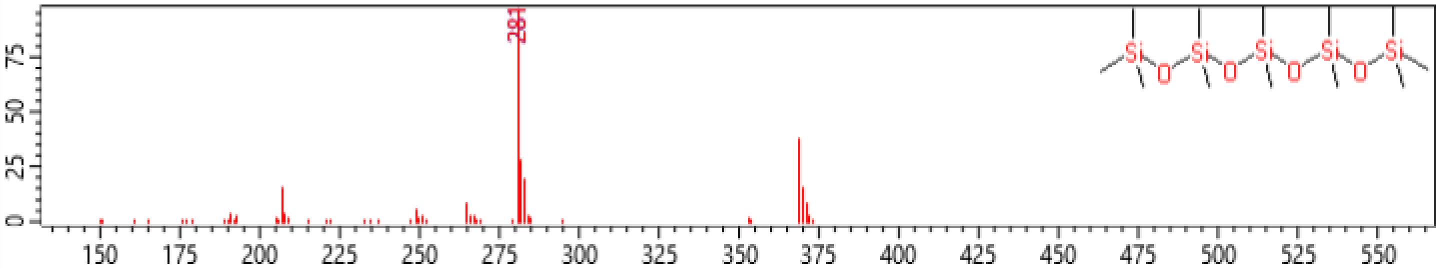


Trisiloxane, 1,1,1,5,5,5-hexamethyl-3,3-bis[(trimethylsilyl)oxy]-

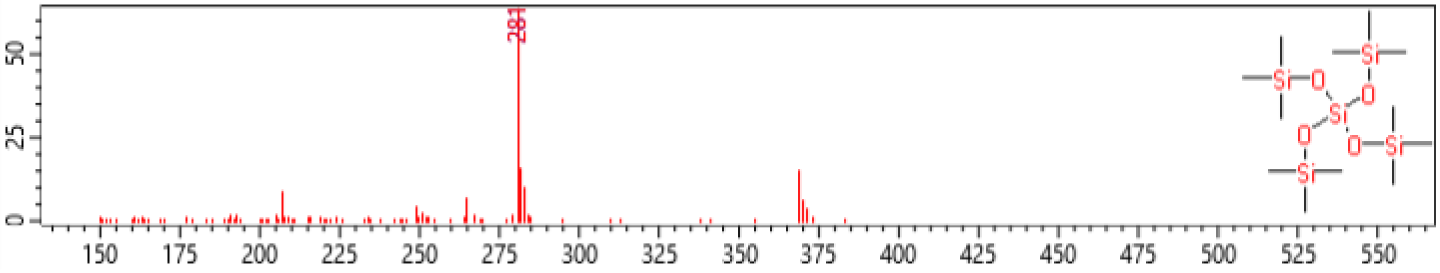

**3-Isopropoxy-1,1,1,5,5,5-hexamethyl-3-(trimethylsiloxy)trisiloxane**
Peak: 7.92 - 7.94 min (scan #257 - #261) Subtracted noise: 7.90 - 7.91 min (scan #251 - #254), 7.95 - 7.96 min (scan #265 - #268)

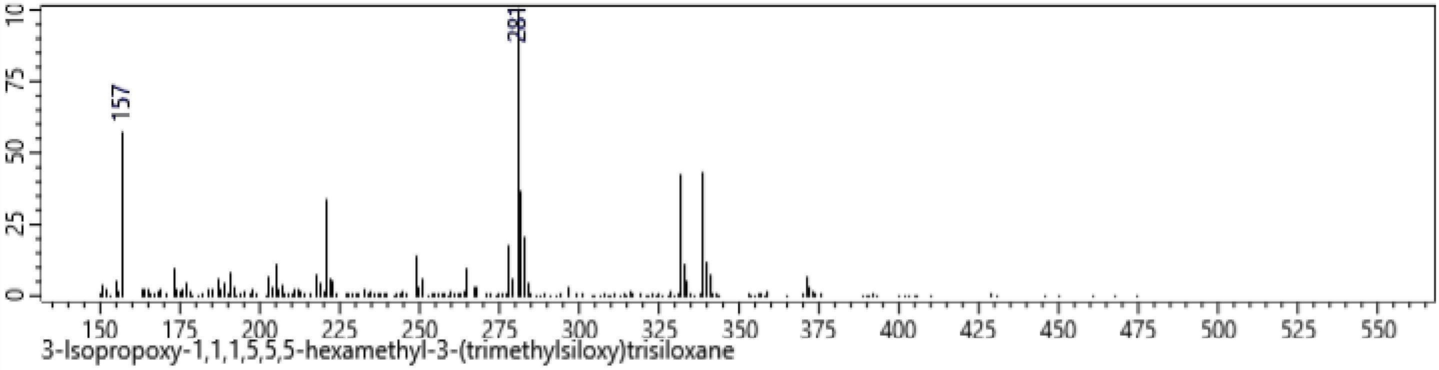


3-Isopropoxy-1,1,1,5,5,5-hexamethyl-3-(trimethylsiloxy)trisiloxane

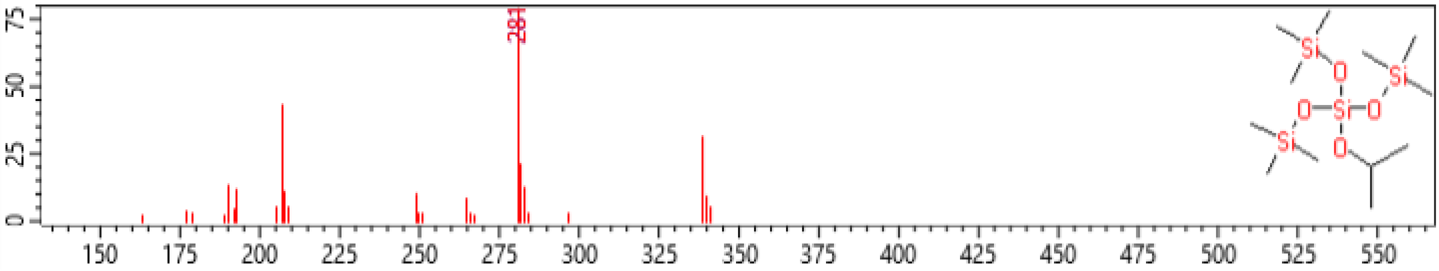


3-(5-Methyl-1,3-benzoxazol-2-yl)phenylamine, N-(tert-butyldimethylsilyl)-

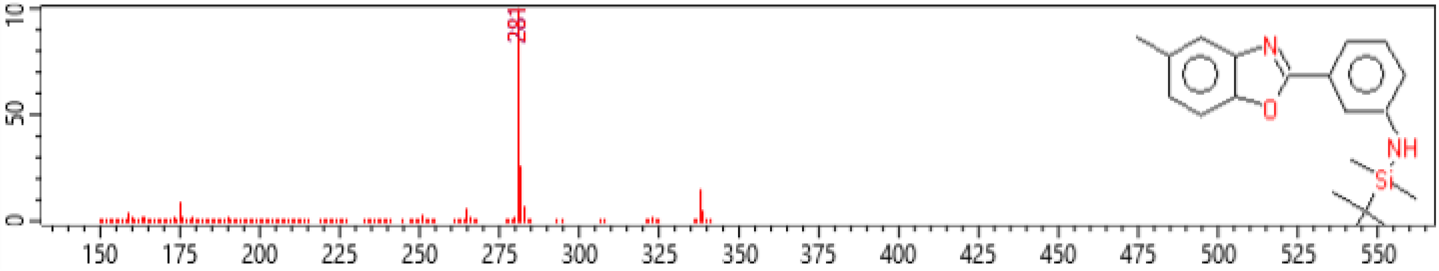


4-(Methoxymethyl)-2,3,5,6-tetrafluorobenzyl alcohol, TMS

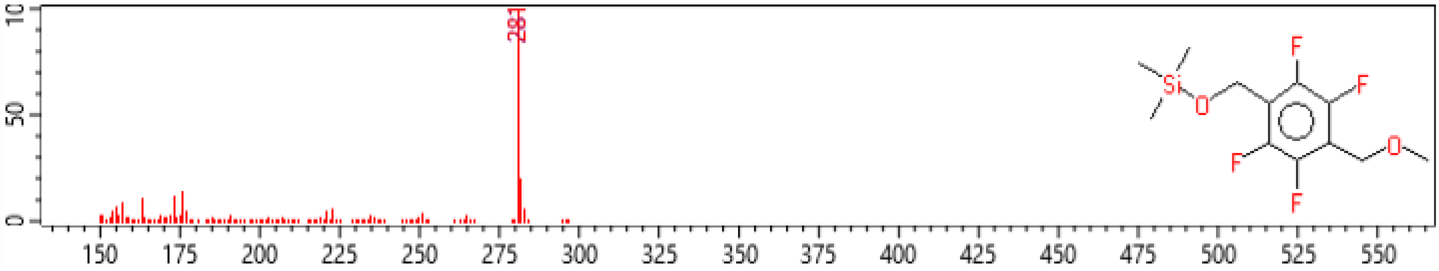


2-Hydroxy-5-dibenzosuberone, tert-butyldimethylsilyl ether

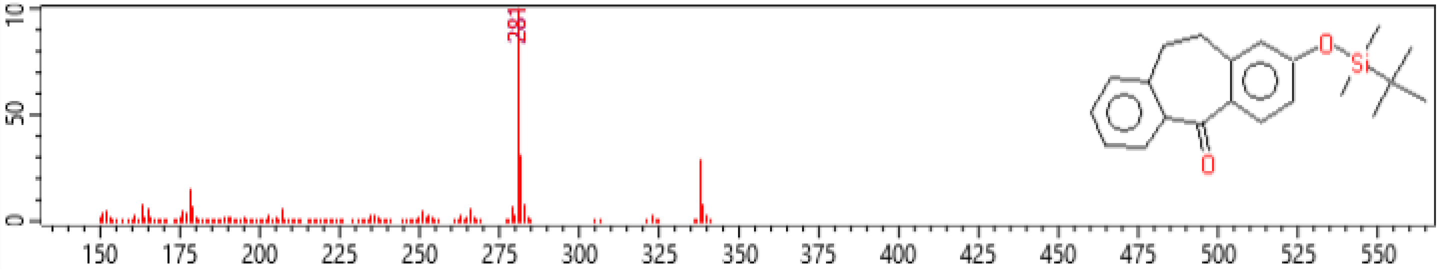

4'-(Tert.-butyldimethylsilyl)oxychalcone

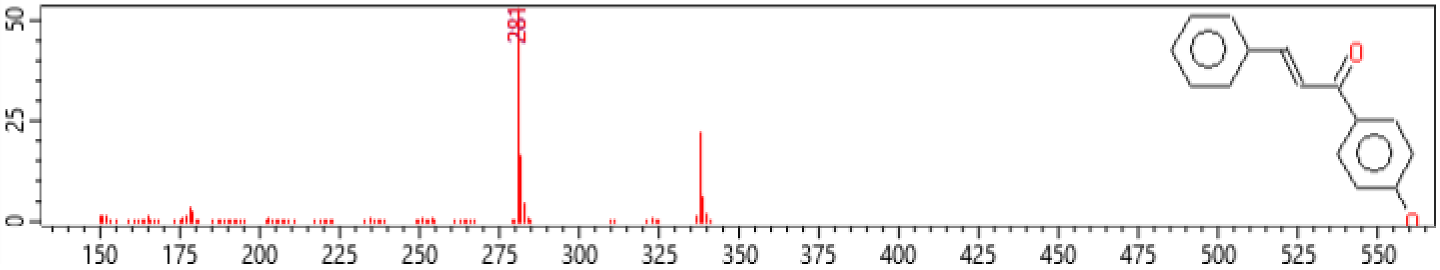


**2-Aminoquinolin-8-ol**

Peak: 7.99 - 8.06 min (scan #275 - #296) Subtracted noise: 7.89 - 7.96 min (scan #247 - #267), 8.12 - 8.20 min (scan #313 - #334)

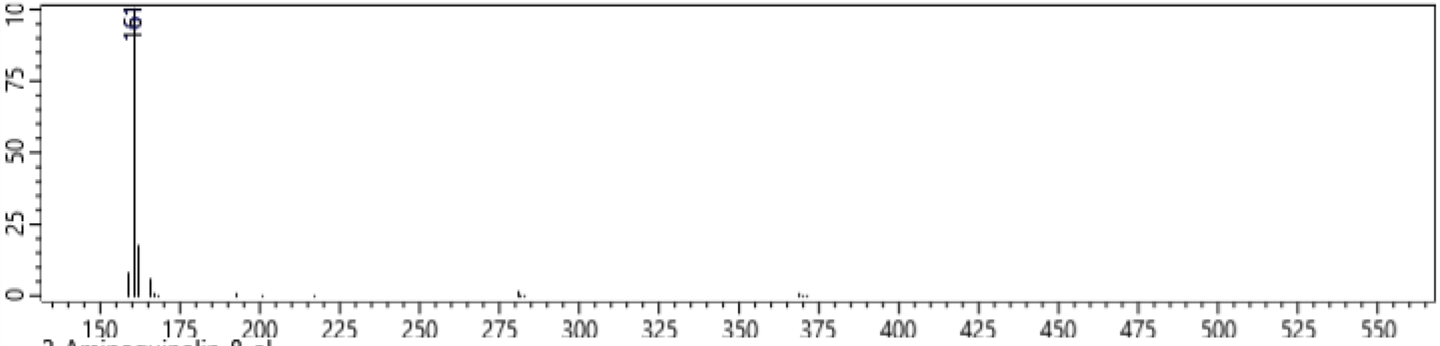


2-Aminoquinolin-8-ol

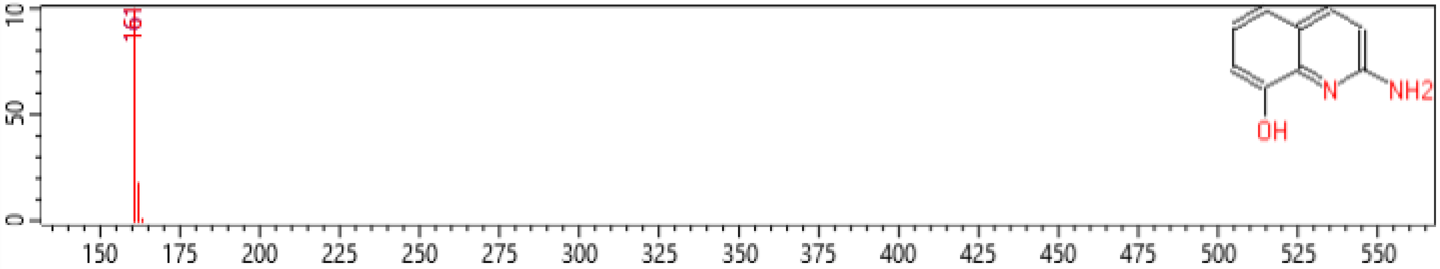


2-Aminoquinolin-4-ol

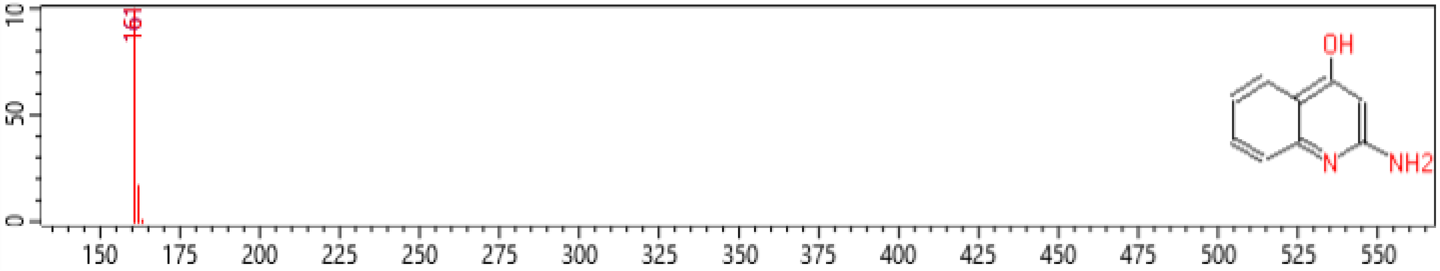


3-trans-Hydroxynorcotinine

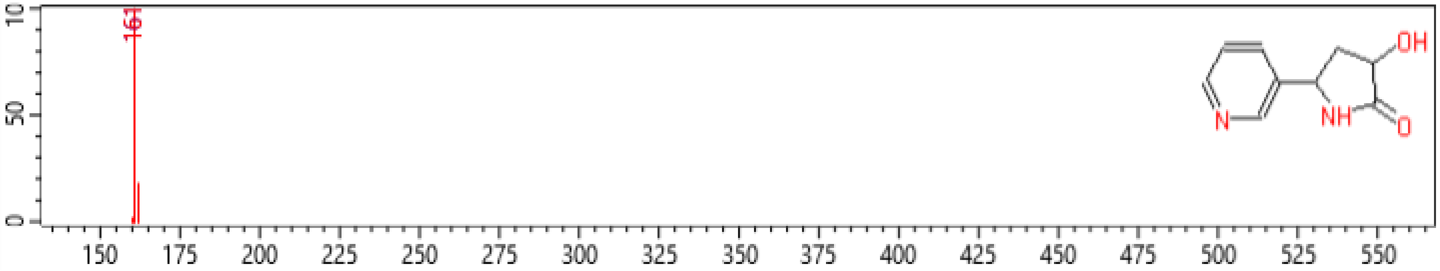

1-Methyl-3,4-dihydro-1H-quinolin-2-one

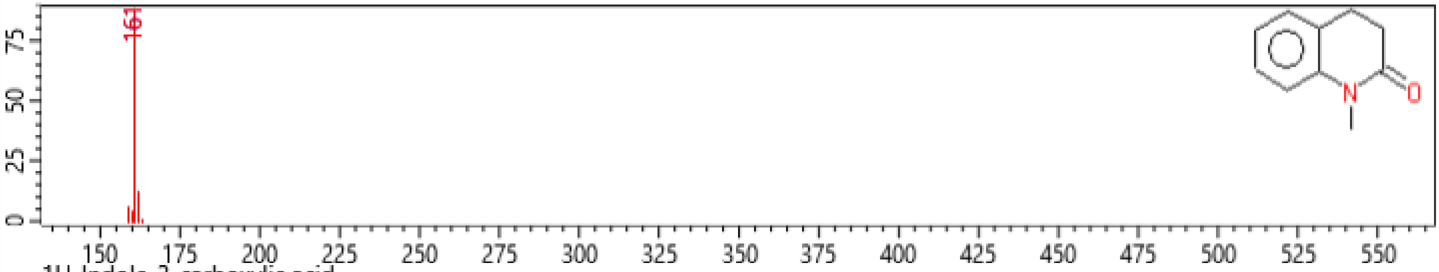


1H-Indole-3-carboxylic acid

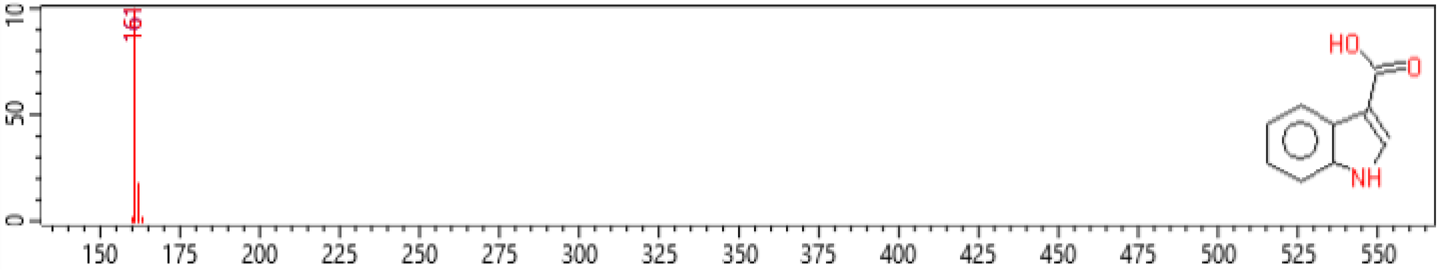


**3-Ethylphenol, TMS derivative**

Peak: 8.15 - 8.20 min (scan #321 - #335) Subtracted noise: 8.06 - 8.12 min (scan #297 - #312), 8.23 - 8.28 min (scan #344 - #358)

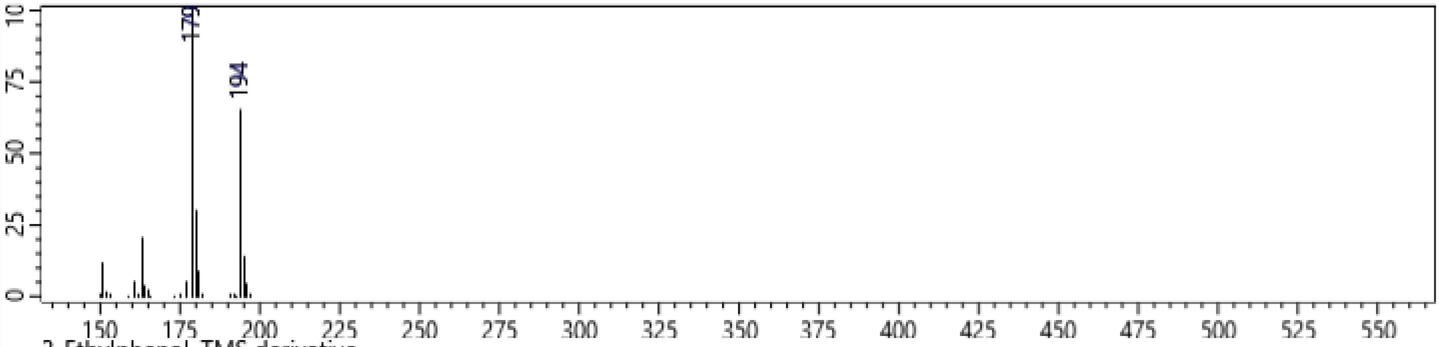


3-Ethylphenol, TMS derivative

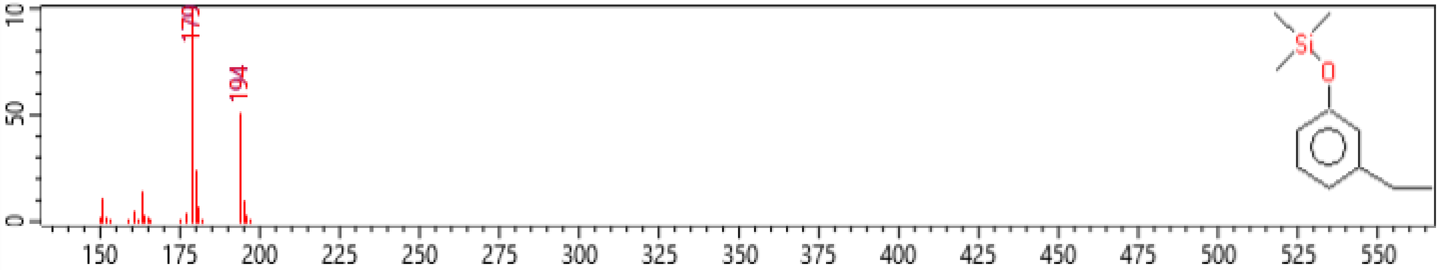


2-Ethylphenol, TMS derivative

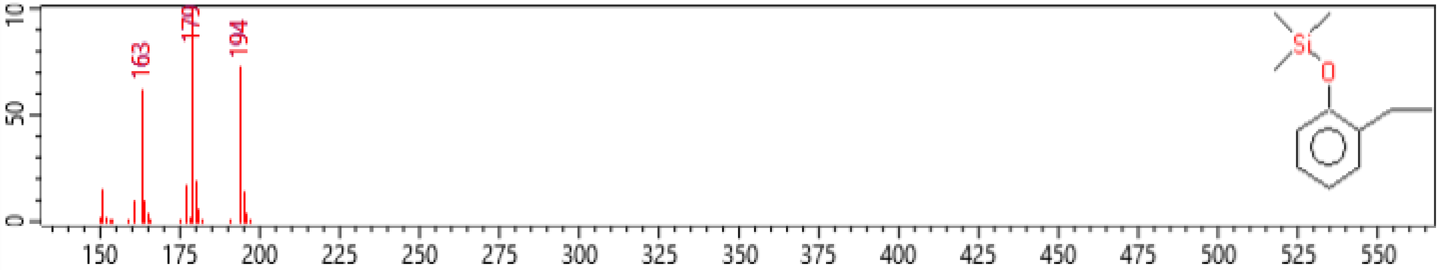

2-Ethylphenol, TMS derivative

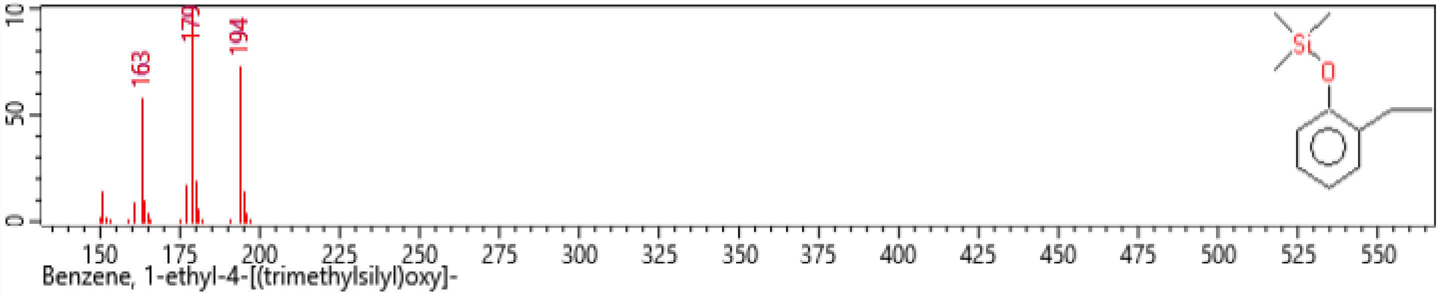


Benzene, 1-ethyl-4-[(trimethylsilyl)oxy]-

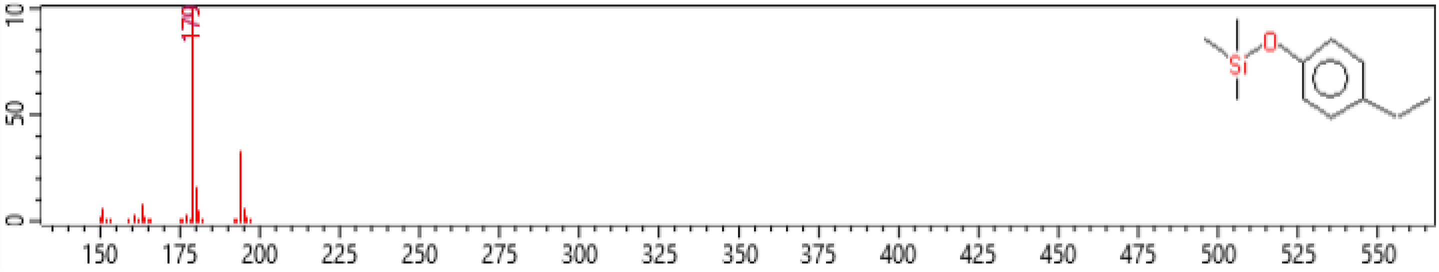


2,4-Dimethylphenol, TMS derivative

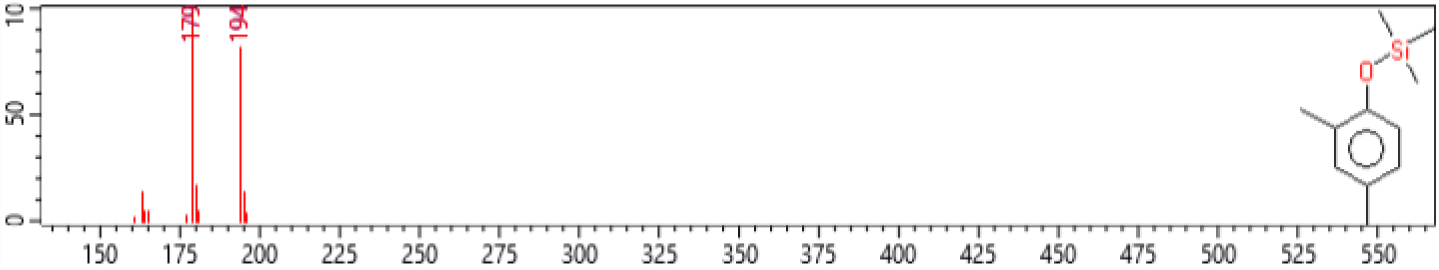


**Silacycloundec-6-yne, 1,1-dimethyl-**

Peak: 8.24 - 8.25 min (scan #346 - #350) Subtracted noise: 8.21 - 8.23 min (scan #339 - #343), 8.27 - 8.28 min (scan #353 - #357)

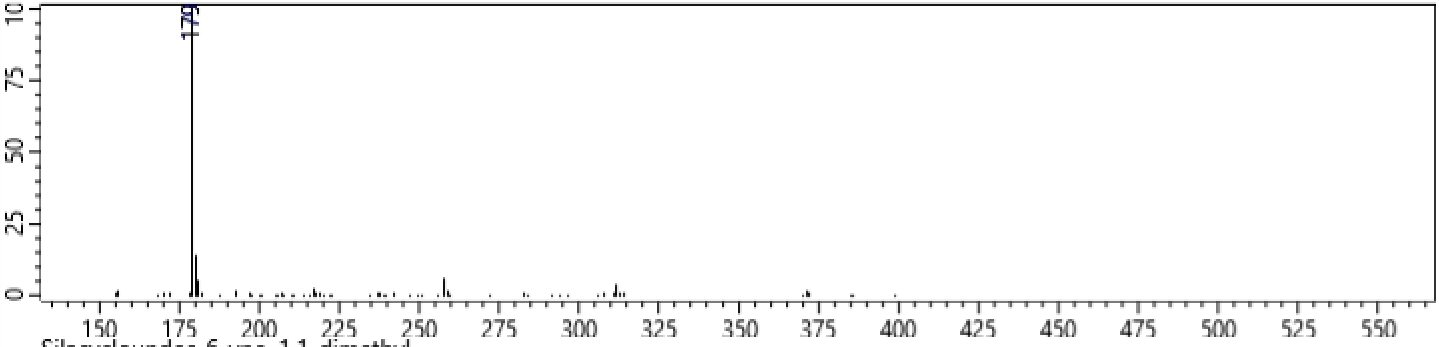


Silacycloundec-6-yne, 1,1-dimethyl-

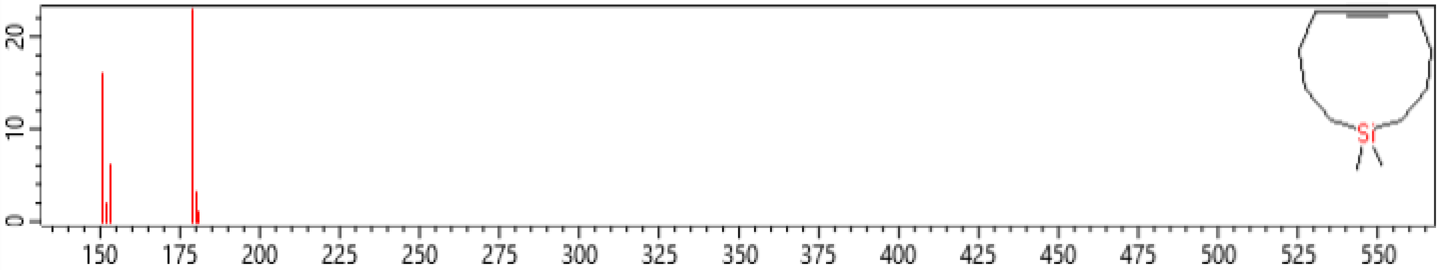

2-Amino-4,6-pteridinediol

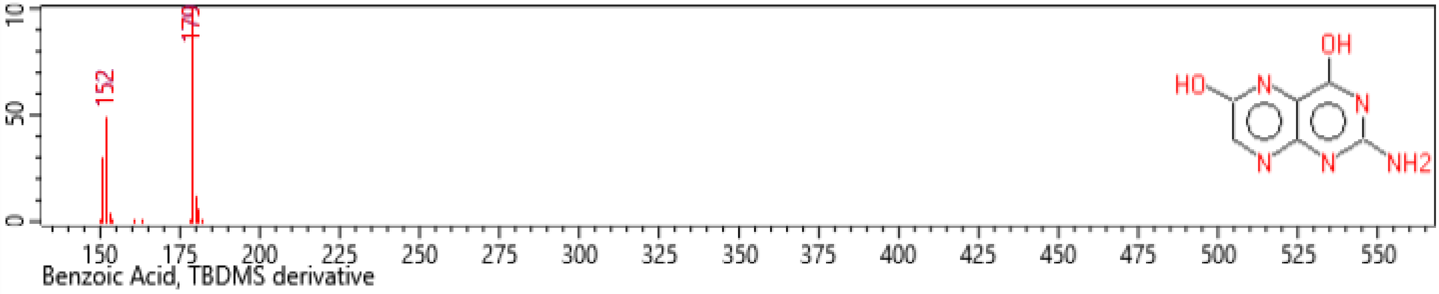


Benzoic Acid, TBDMS derivative

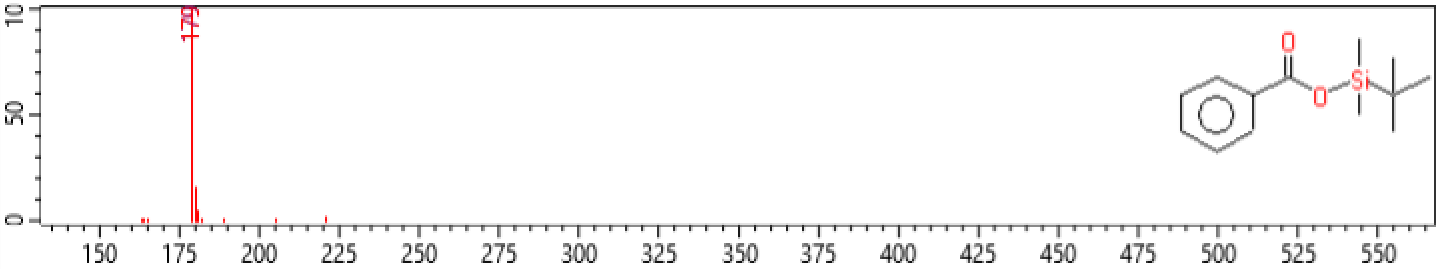


N,N-Dimethyl phenyl(thioacetamide)

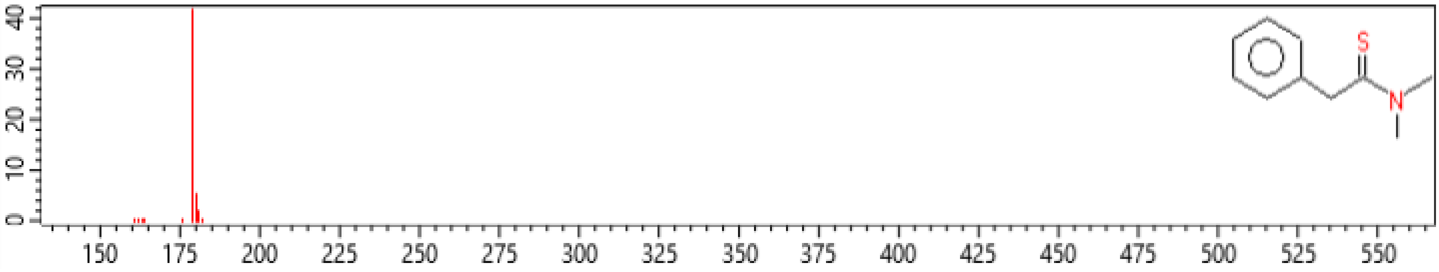


Carbamic acid, phenyl-, 1-methylethyl ester

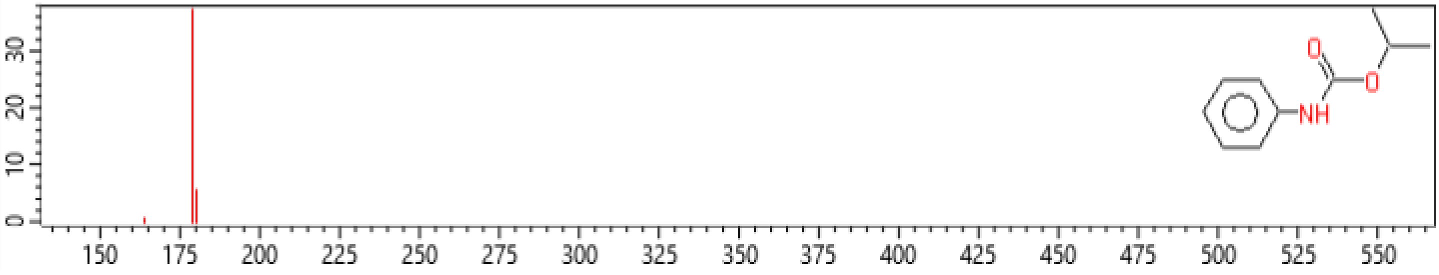


**Sumithrin**

Peak: 8.44 - 8.45 min (scan #402 - #404) Subtracted noise: 8.42 - 8.43 min (scan #396 - #399), 8.46 - 8.47 min (scan #407 - #409)

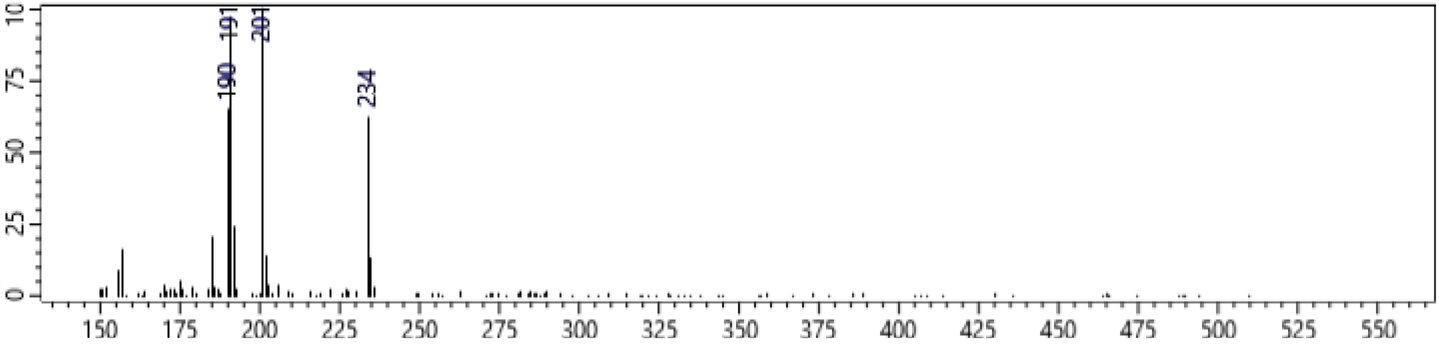

Sumithrin

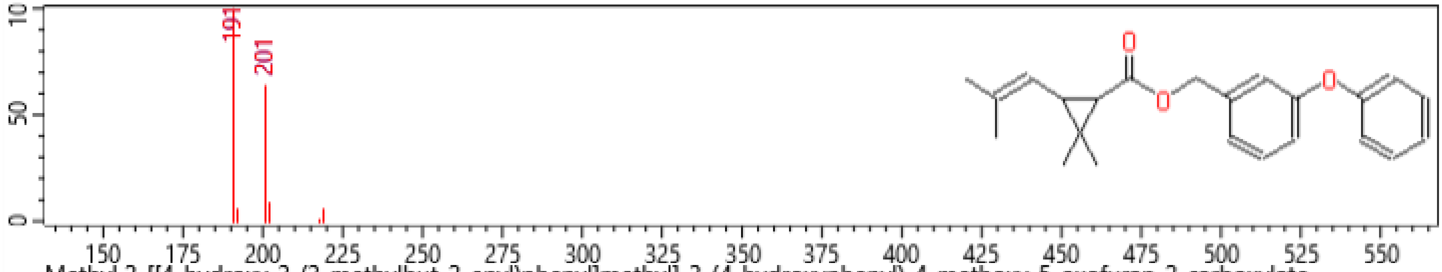


Methyl 2-[[4-hydroxy-3-(3-methylbut-2-enyl)phenyl]methyl]-3-(4-hydroxyphenyl)-4-methoxy-5-oxofuran-2-carboxylate

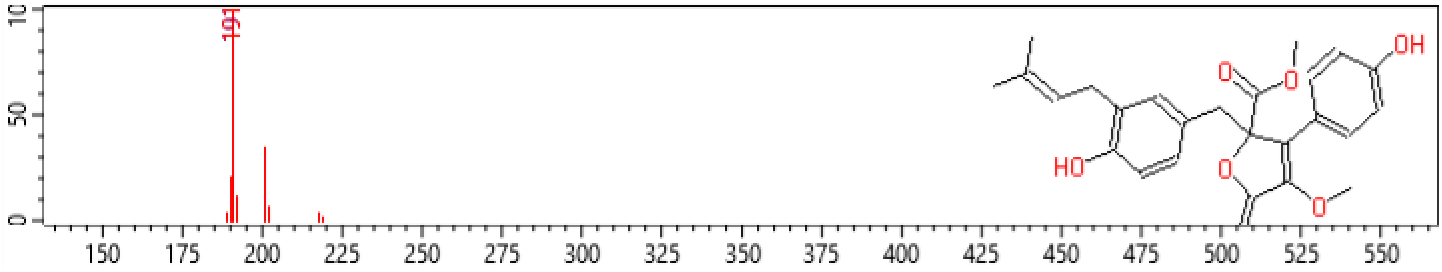


Bindone

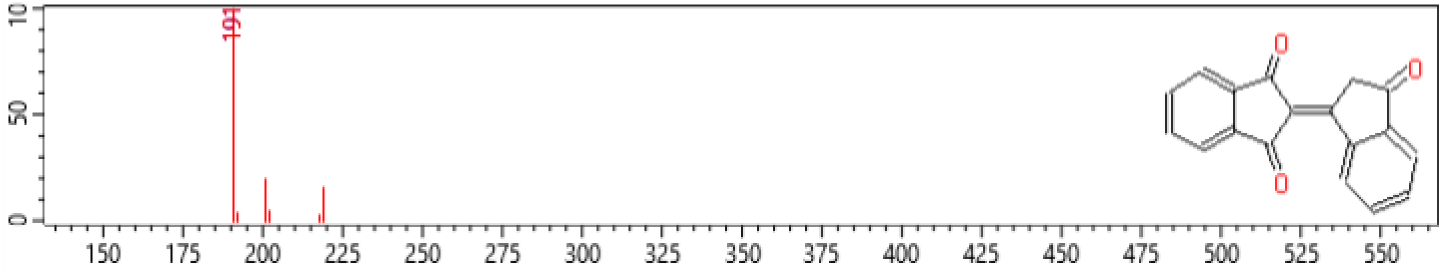


Anthracene, 9-butyl-

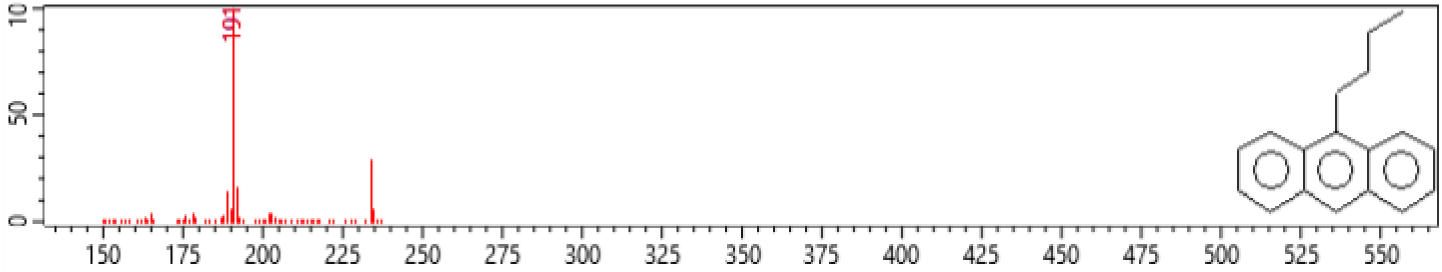


Spiro(1,3-benzodioxole-2,1'-cyclohexane)-5-carboxylic acid

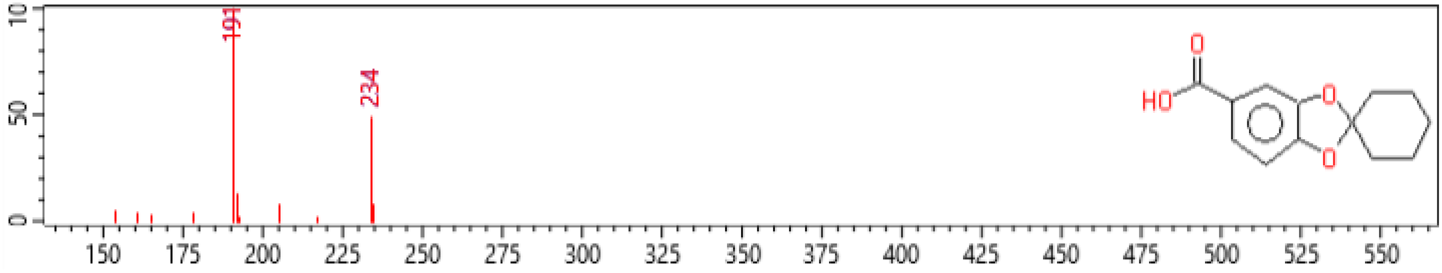

**l-Leucine, N-benzyloxycarbonyl-N-methyl-, dodecyl ester**

Peak: 8.46 - 8.48 min (scan #408 - #412) Subtracted noise: 8.44 - 8.46 min (scan #402 - #406), 8.49 - 8.50 min (scan #416 - #419)

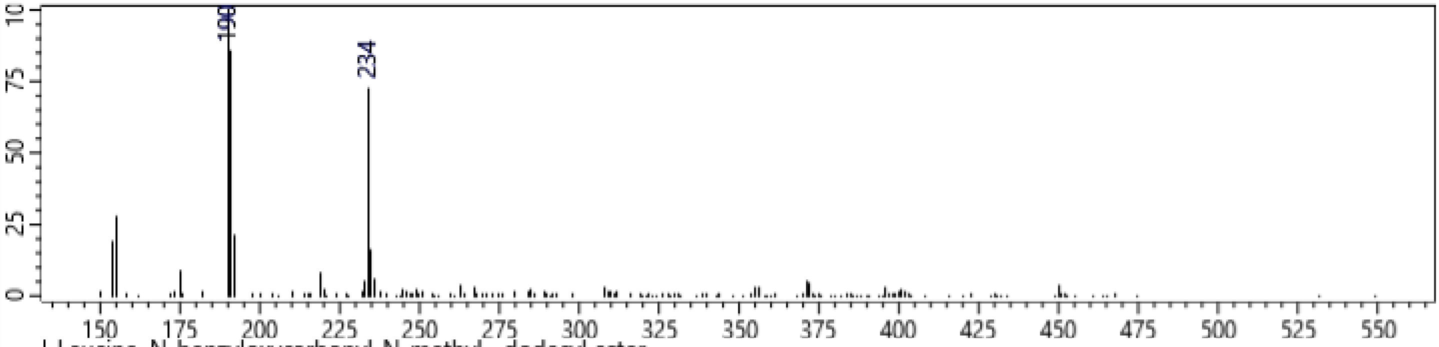


l-Leucine, N-benzyloxycarbonyl-N-methyl-, dodecyl ester

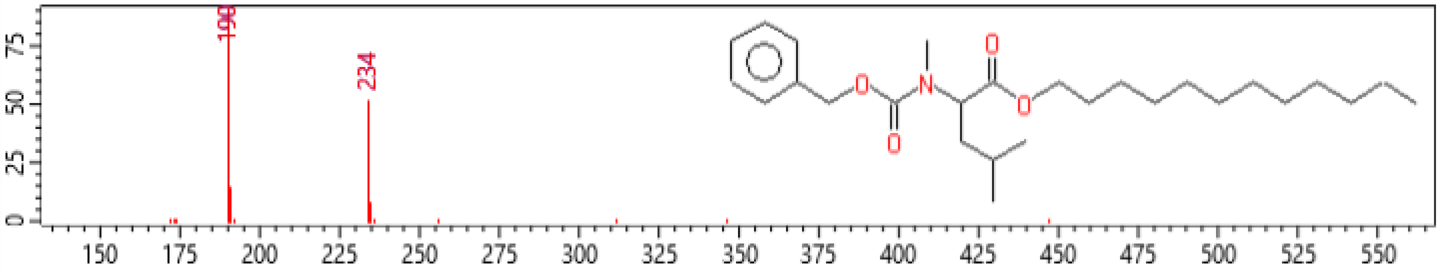


l-Leucine, N-benzyloxycarbonyl-N-methyl-, nonyl ester

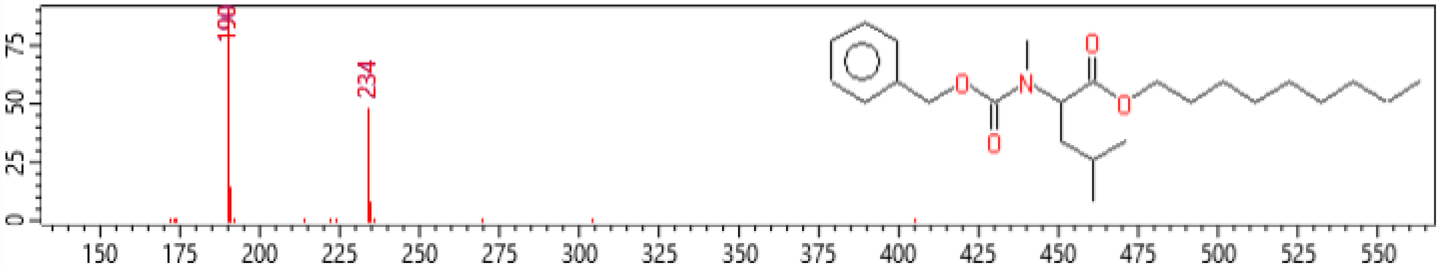


l-Leucine, N-benzyloxycarbonyl-N-methyl-, undecyl ester

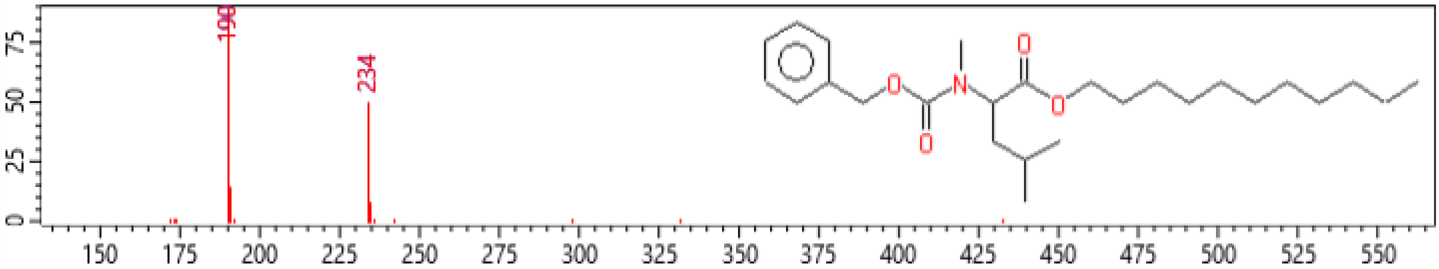


l-Leucine, N-benzyloxycarbonyl-N-methyl-, isohexyl ester

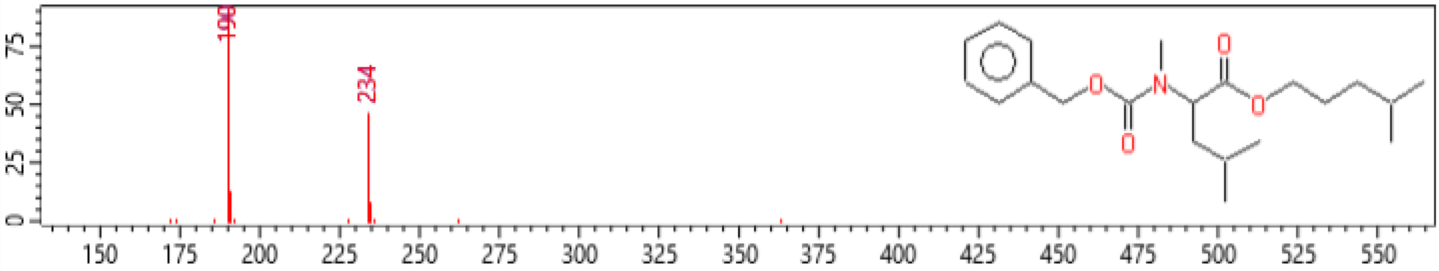

l-Leucine, N-benzyloxycarbonyl-N-methyl-, butyl ester

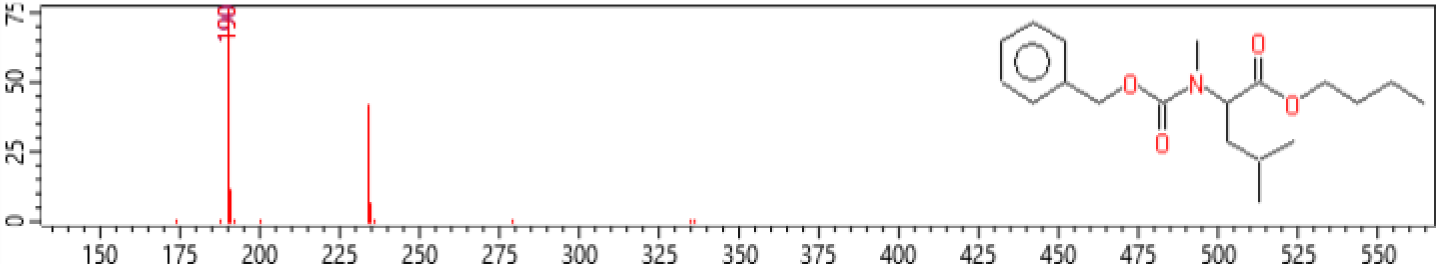


**Silanol, trimethyl-, phosphate (3:1)**

Peak: 8.50 - 8.52 min (scan #418 - #424) Subtracted noise: 8.46 - 8.49 min (scan #408 - #415), 8.54 - 8.57 min (scan #430 - #437)

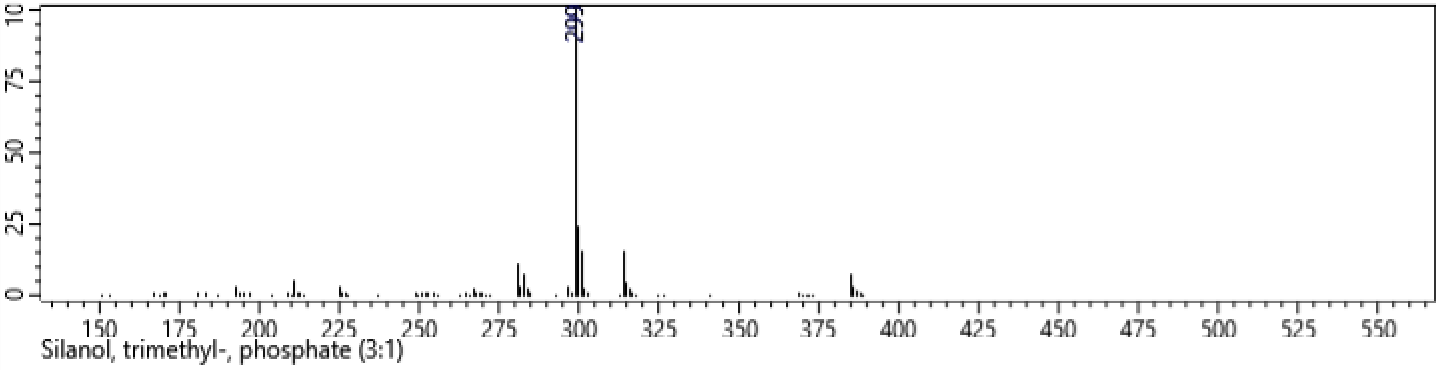


Silanol, trimethyl-, phosphate (3:1)

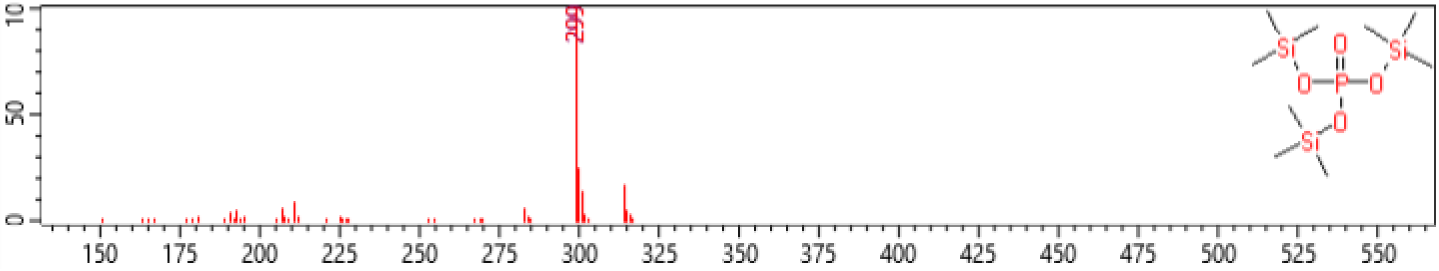


Silanol, trimethyl-, phosphate (3:1)

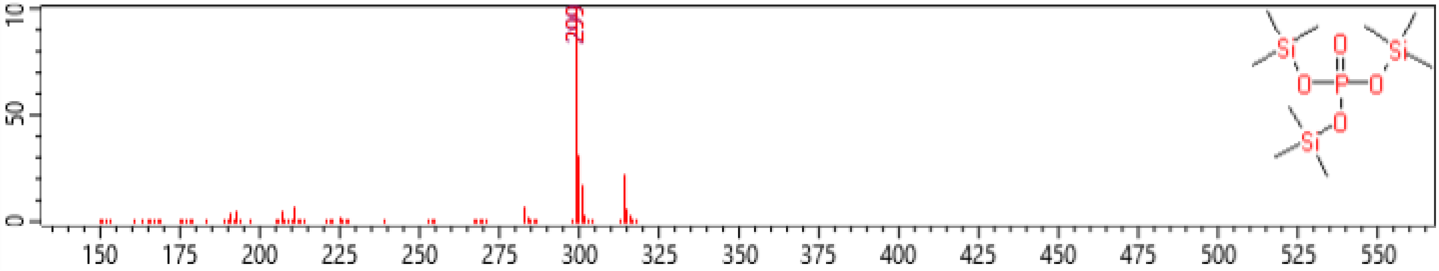


Silanol, trimethyl-, phosphate (3:1)

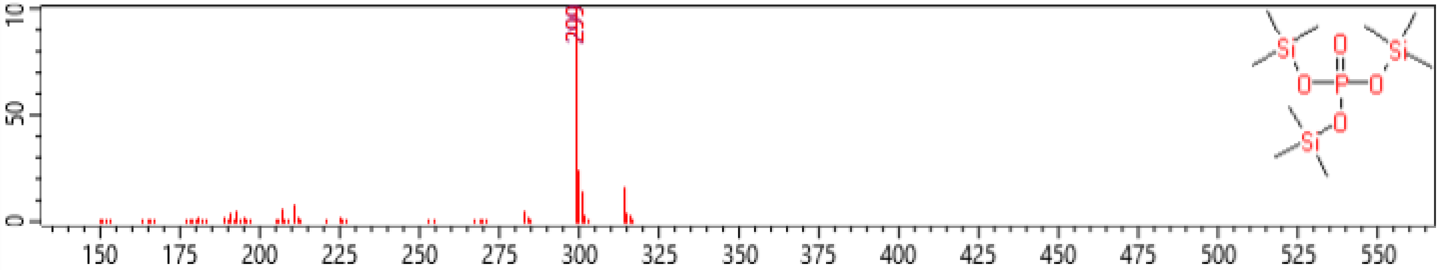

Silanol, trimethyl-, phosphate (3:1)

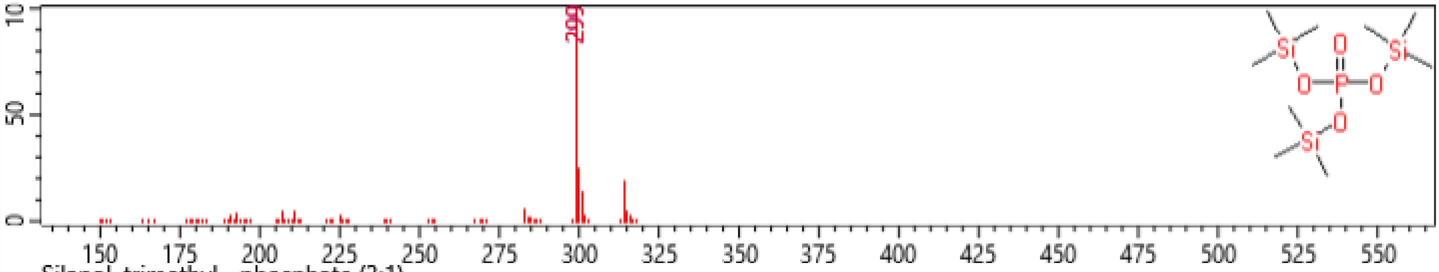


Silanol, trimethyl-, phosphate (3:1)

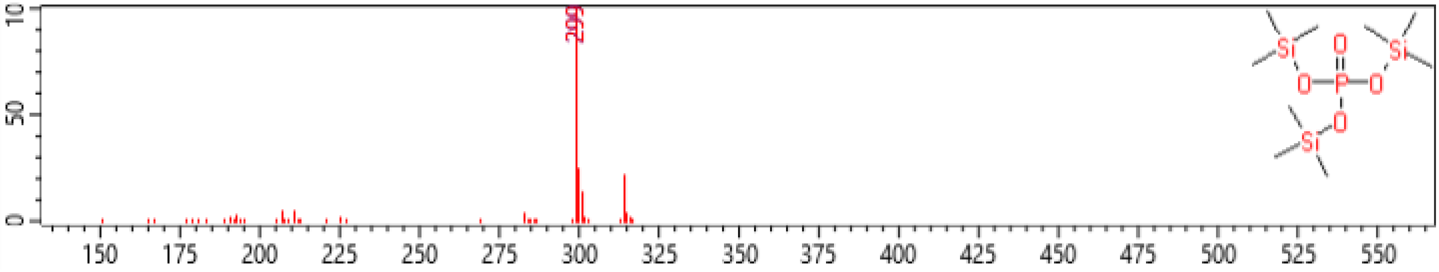


**Glycerol, 3TMS derivative**

Peak: 8.55 - 8.58 min (scan #432 - #440) Subtracted noise: 8.51 - 8.54 min (scan #422 - #429), 8.60 - 8.63 min (scan #447 - #455)

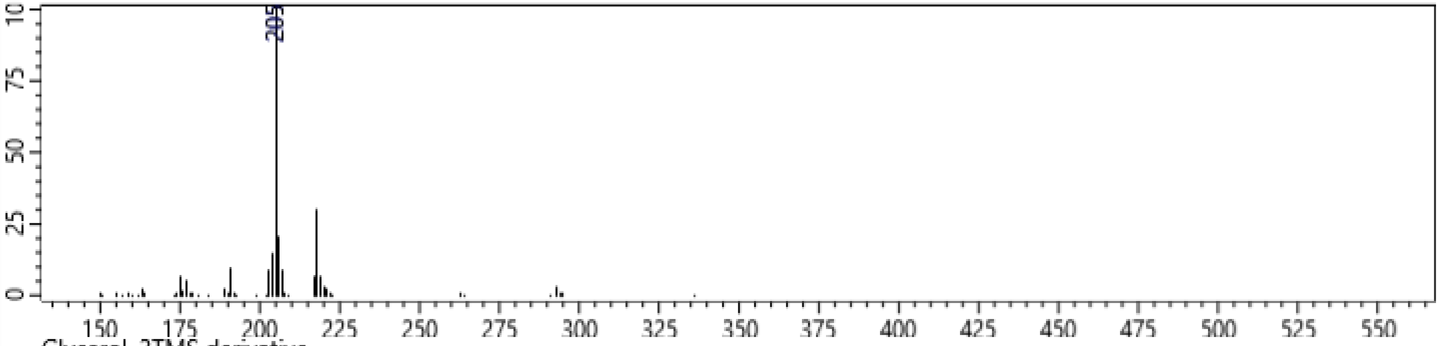


Glycerol, 3TMS derivative

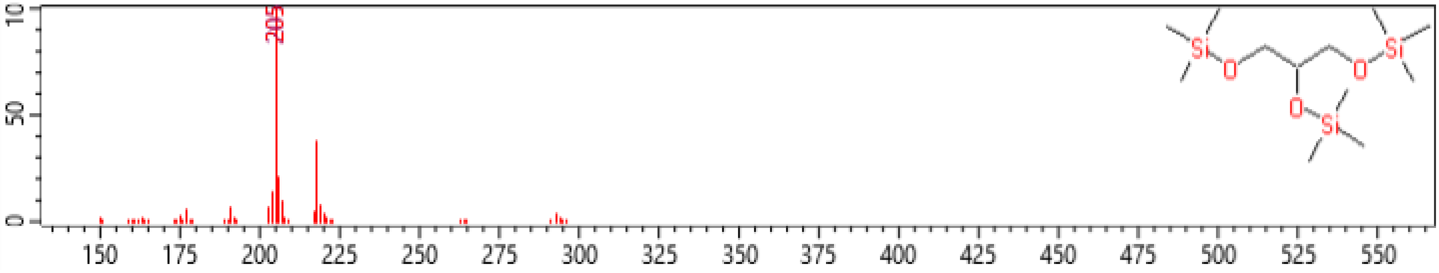


Glycerol, 3TMS derivative

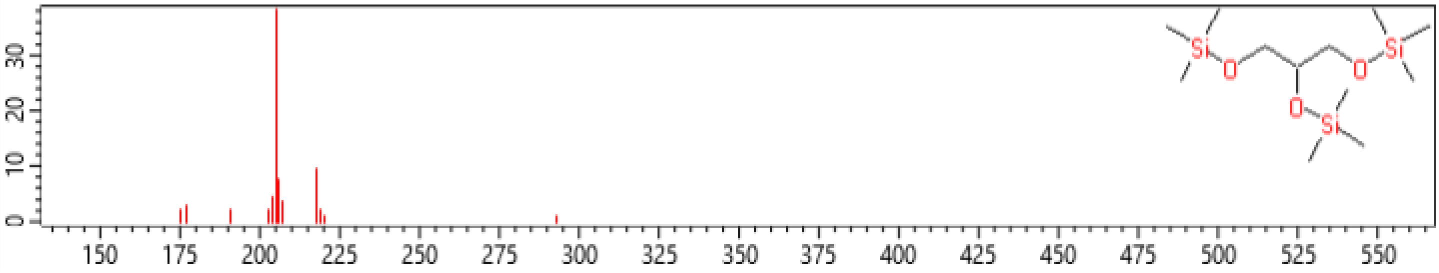

Glycerol, 3TMS derivative

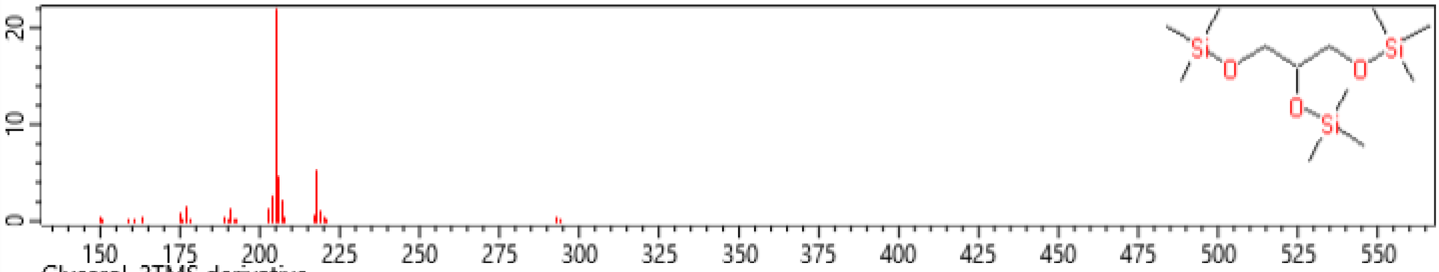

Glycerol, 3TMS derivative

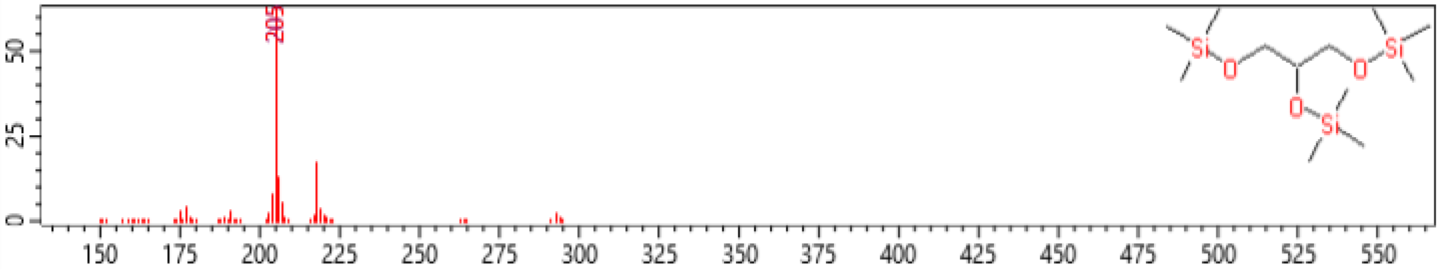


Glycerol, 3TMS derivative

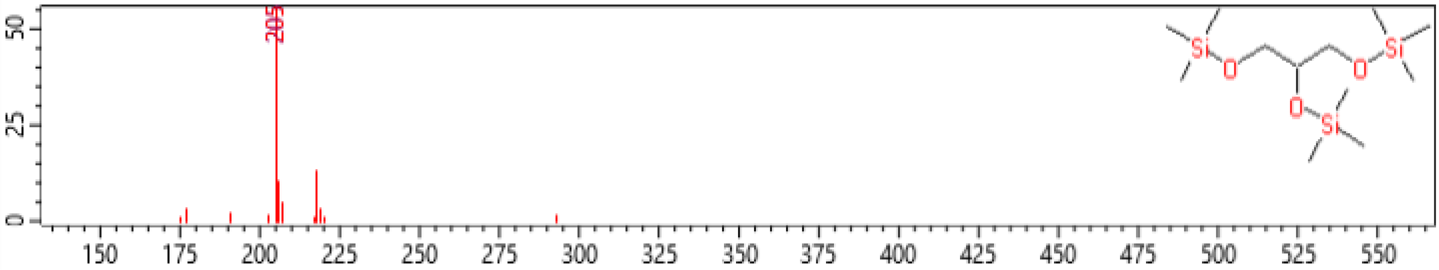


**4-Vinylphenol, TMS derivative**

Peak: 8.62 - 8.63 min (scan #451 - #455) Subtracted noise: 8.59 - 8.60 min (scan #443 - #446), 8.64 - 8.65 min (scan #457 - #460)

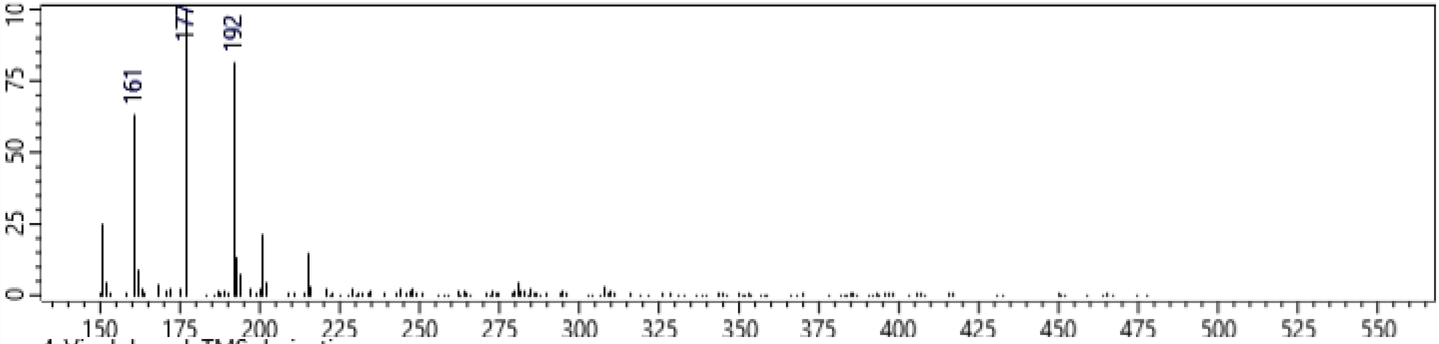


4-Vinylphenol, TMS derivative

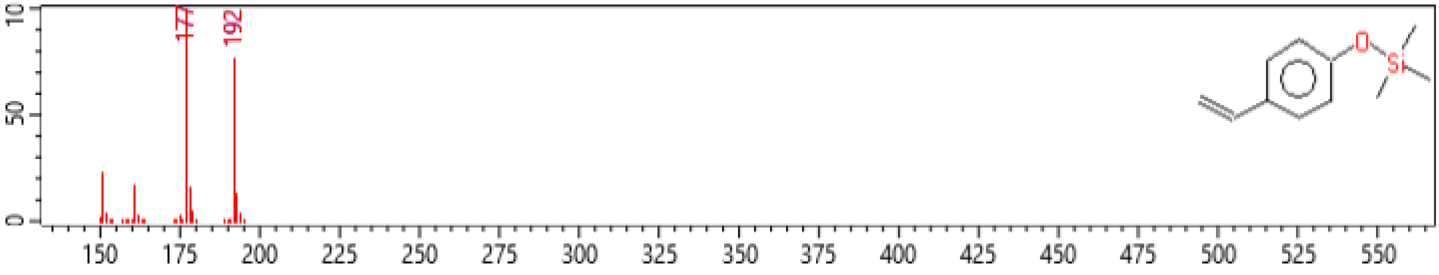

Benzene, [2-[(trimethylsilyl)oxy]ethenyl]-

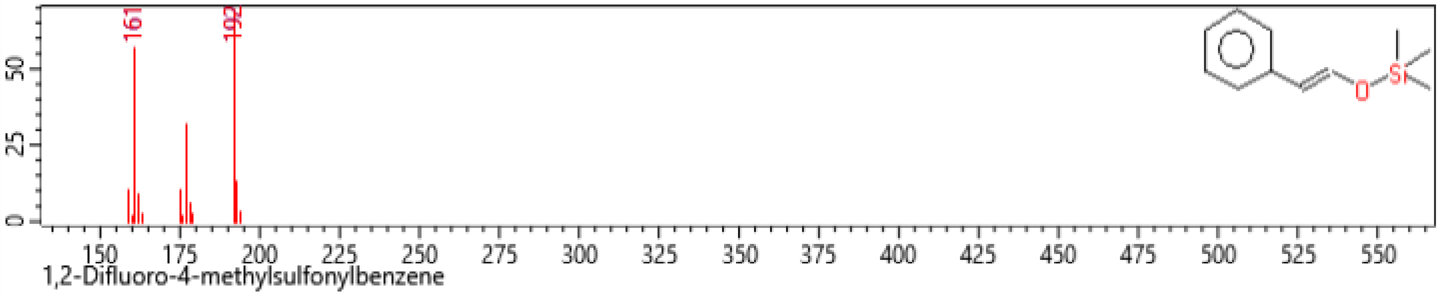


1,2-Difluoro-4-methylsulfonylbenzene

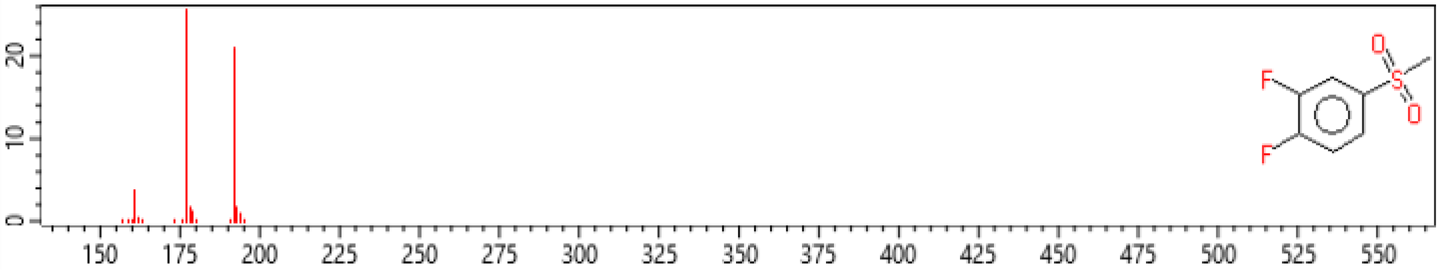


2-(Dimethyl-.lambda.(4)-sulfanylidene)malonic acid, dimethyl ester

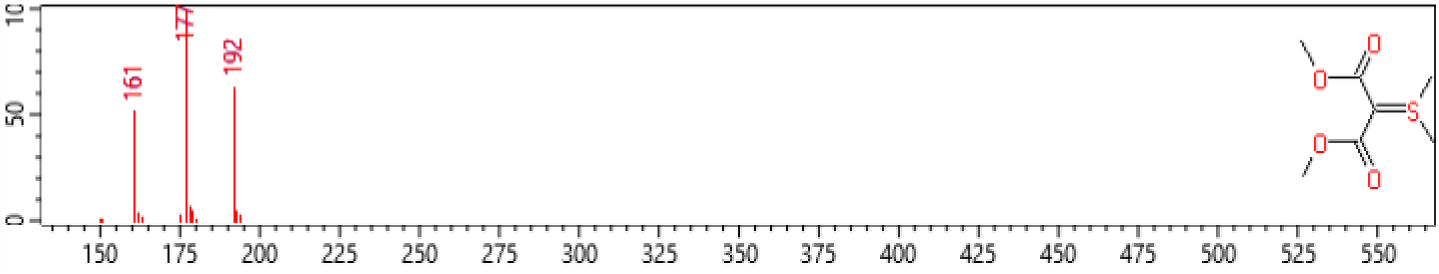


(Z)-4-(but-1-en-1-yl)-1,2-dimethoxybenzene

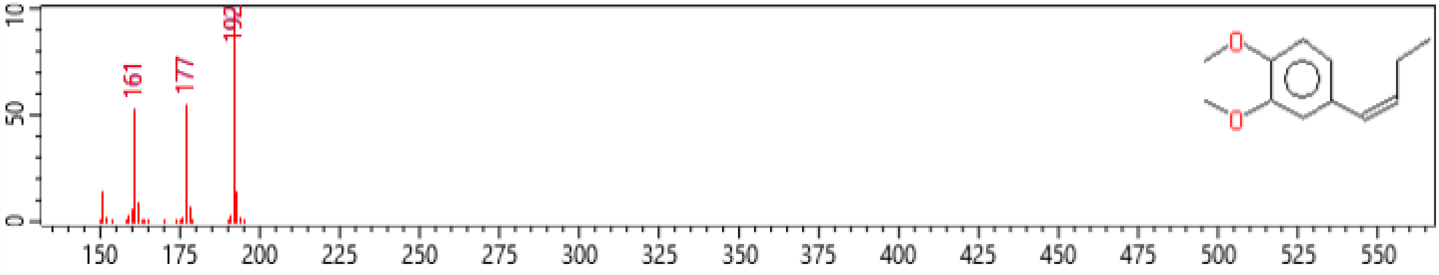


**Neurokinin A / Substance K**

Peak: 8.88 - 8.91 min (scan #524 - #534) Subtracted noise: 8.83 - 8.87 min (scan #511 - #521), 8.95 - 8.99 min (scan #544 - #554)

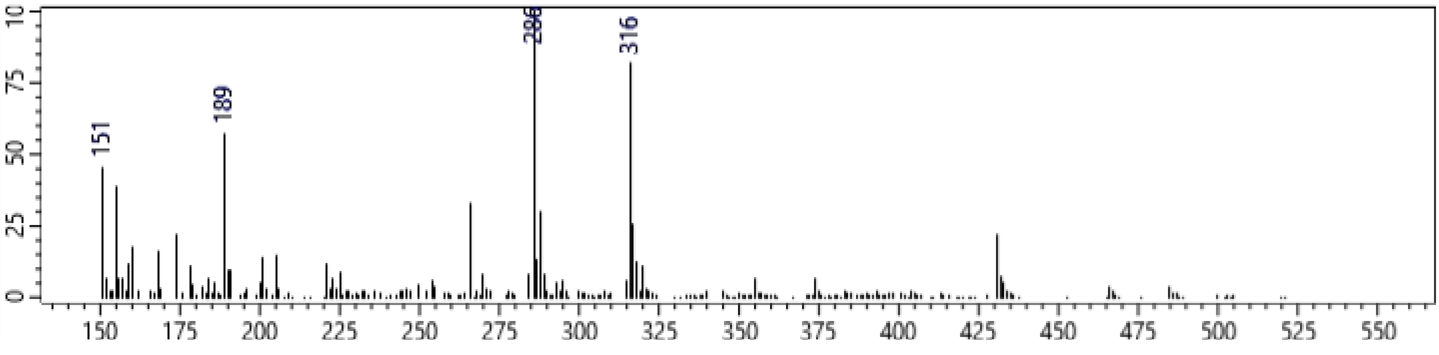

Neurokinin A / Substance K

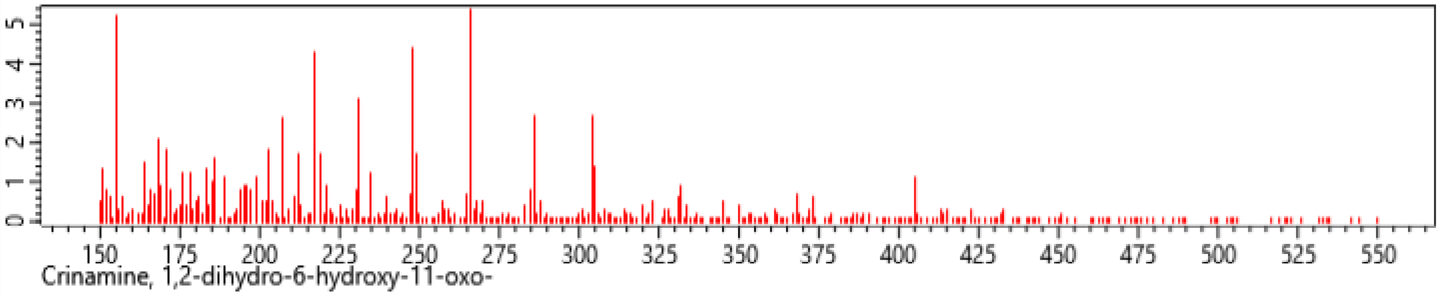

Crinamine, 1,2-dihydro-6-hydroxy-11-oxo-

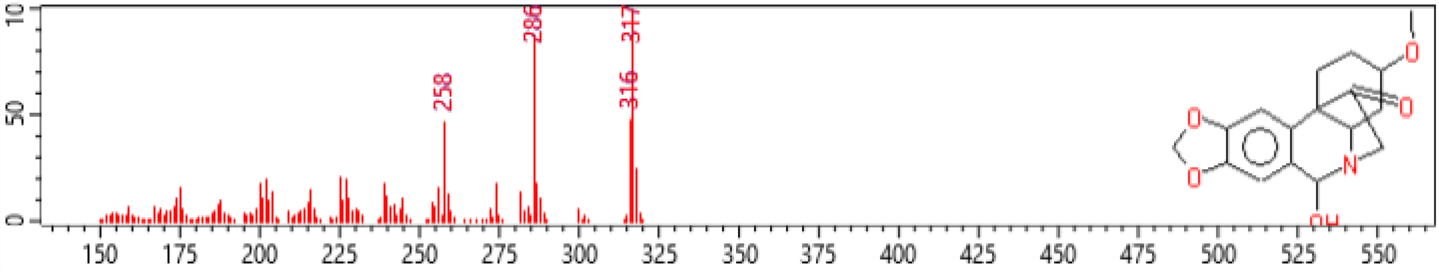


6-Chloro-2-[4-(dimethylamino)phenylazo]benzothiazole

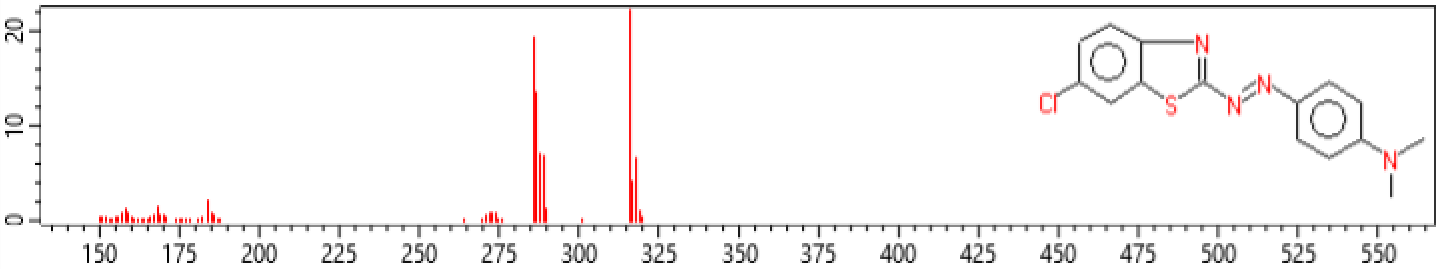

4-(4-Chlorophenyl)-3-(2-methoxyphenyl)-1H-1,2,4-triazole-5-thione

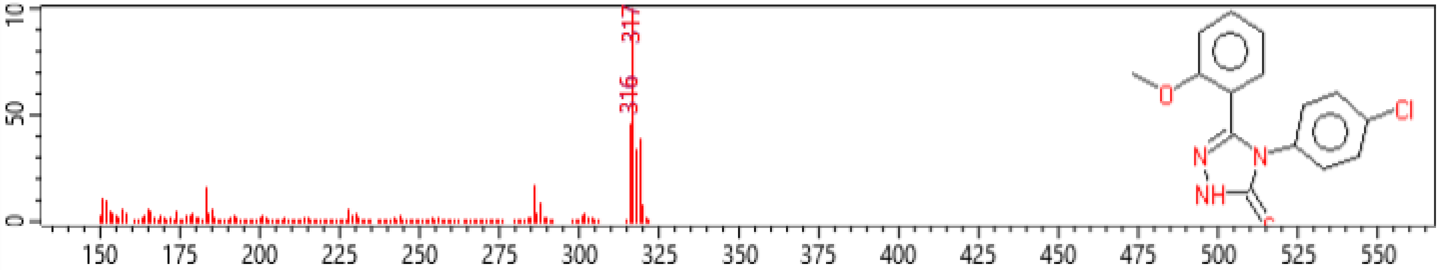


6-Chloro-4,7-dimethoxy-1,3-benzothiazol-2-amine, TMS derivative

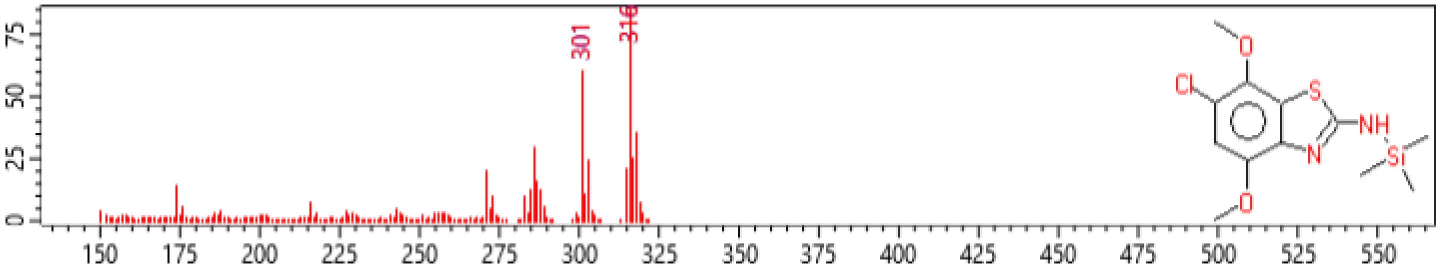

**(1-Benzyl-2-methyl-8-oxo-1,8-dihydroindeno[2,1-b]pyrrol-3-yl)-N,N-dimethylmethanamine**

Peak: 8.97 - 8.99 min (scan #549 - #555) Subtracted noise: 8.92 - 8.95 min (scan #537 - #544), 9.01 - 9.04 min (scan #560 - #568)

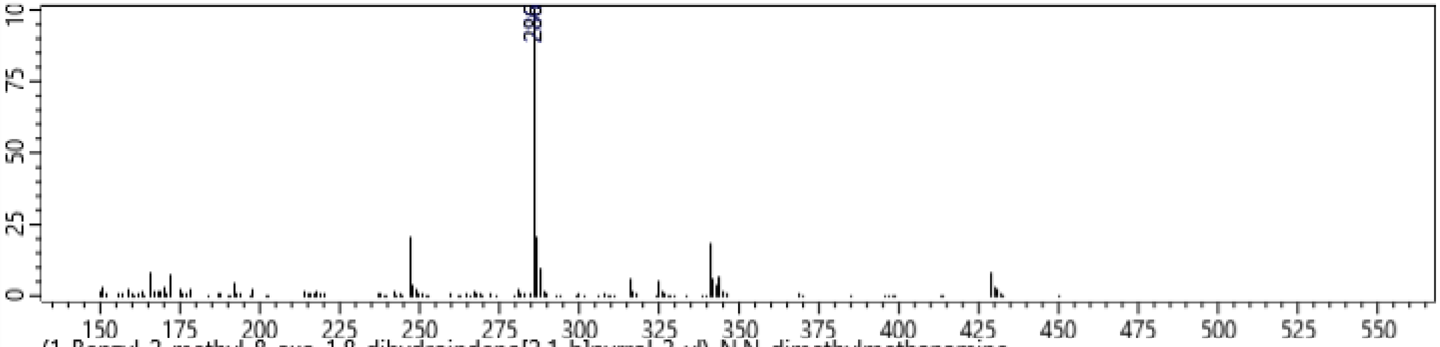

(1-Benzyl-2-methyl-8-oxo-1,8-dihydroindeno[2,1-b]pyrrol-3-yl)-N,N-dimethylmethanamine

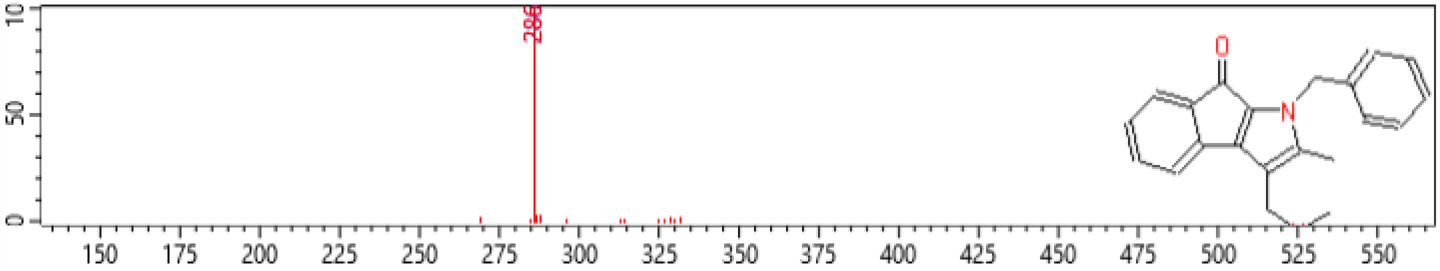

5-nitro-1H-pyrazole-3-carboxylic acid, N, O, di-TMS

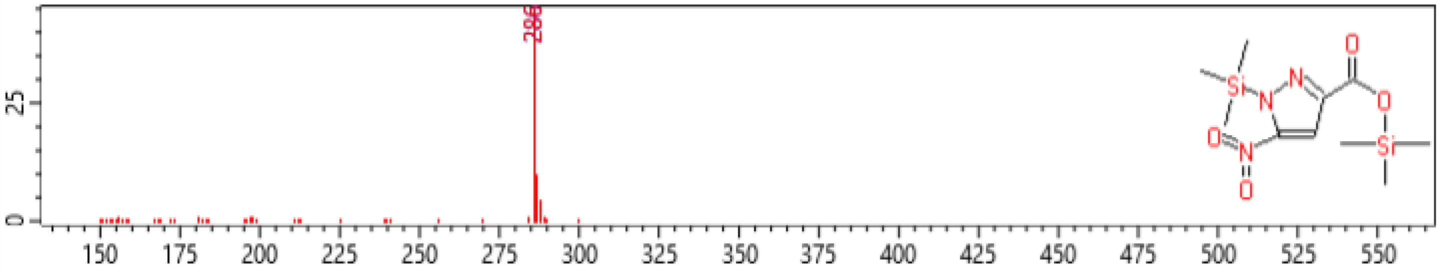

4-Nitro-1H-pyrazole-3-carboxylic acid, N, O, di-TMS

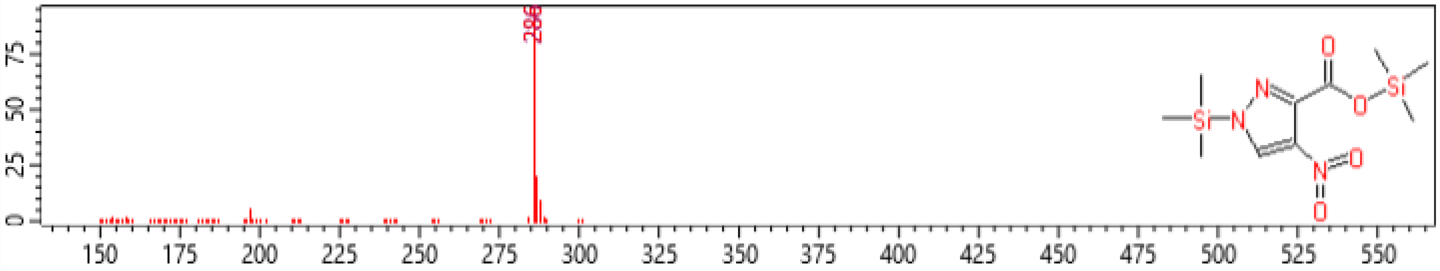

4-Nitro-1H-pyrazole-5-carboxylic acid, N,O di-TMS

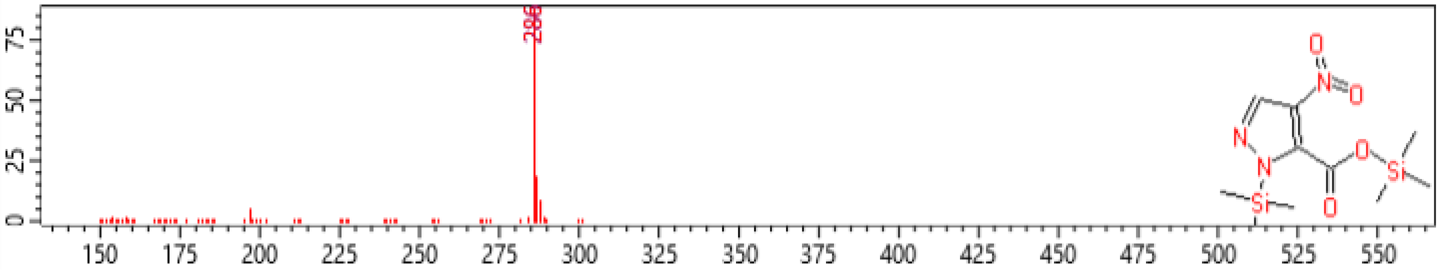

Benzenesulfonamide, 2TMS derivative

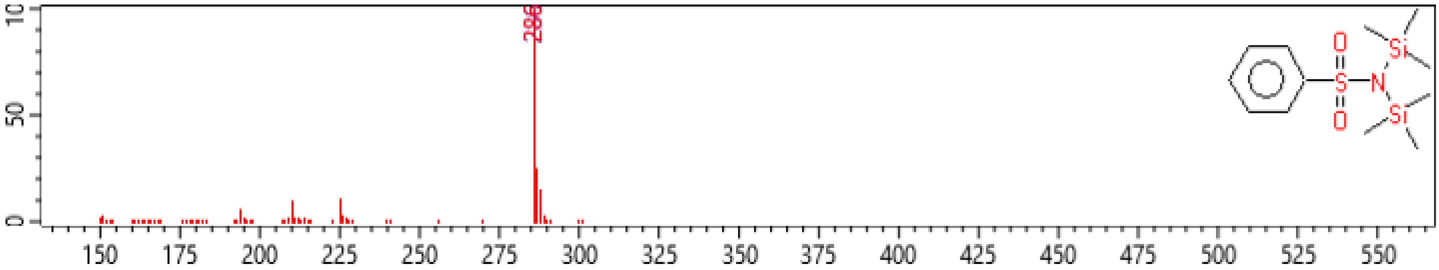

**Tolfenamic acid**

Peak: 9.02 - 9.05 min (scan #565 - #573) Subtracted noise: 8.98 - 9.00 min (scan #552 - #559), 9.07 - 9.10 min (scan #578 - #585)

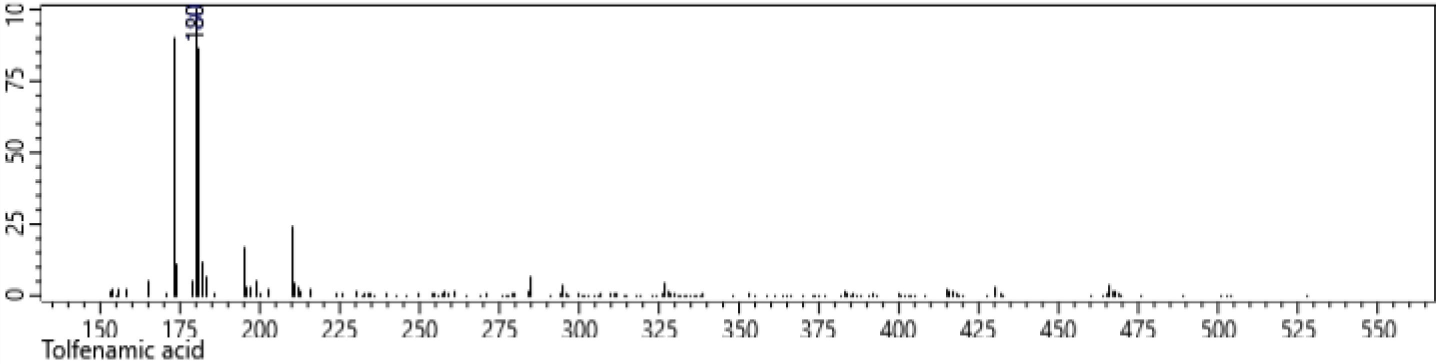

Tolfenamic acid

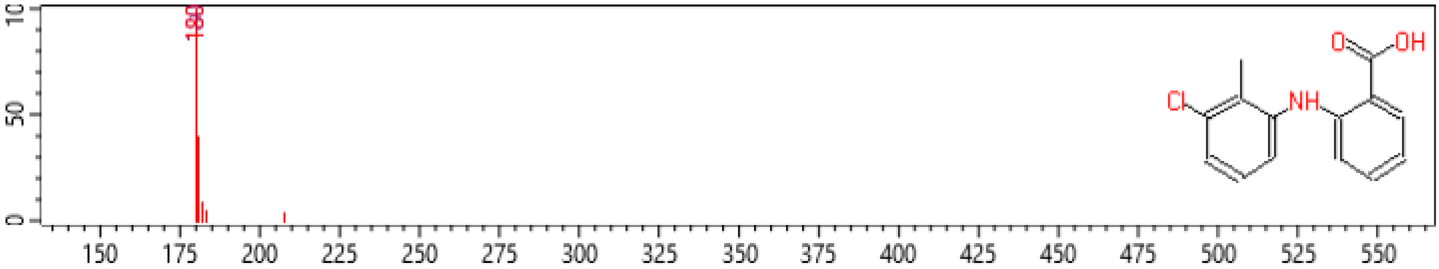

Benzenamine, N-(phenylmethylene)-

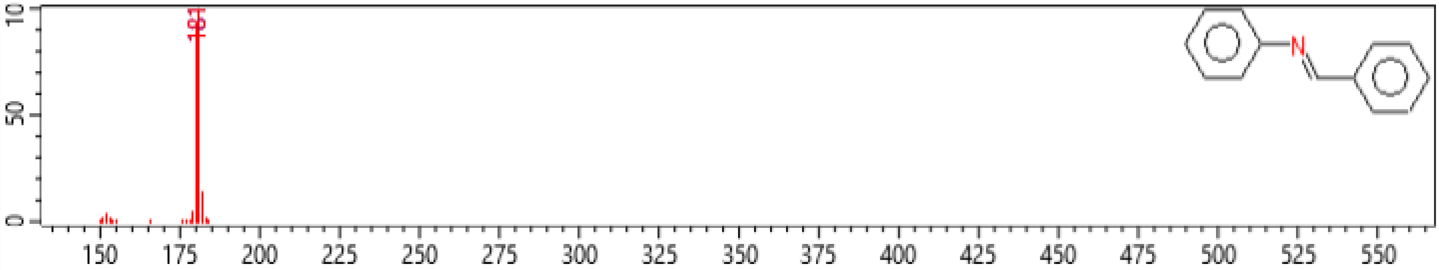

Benzenamine, N-(phenylmethylene)-

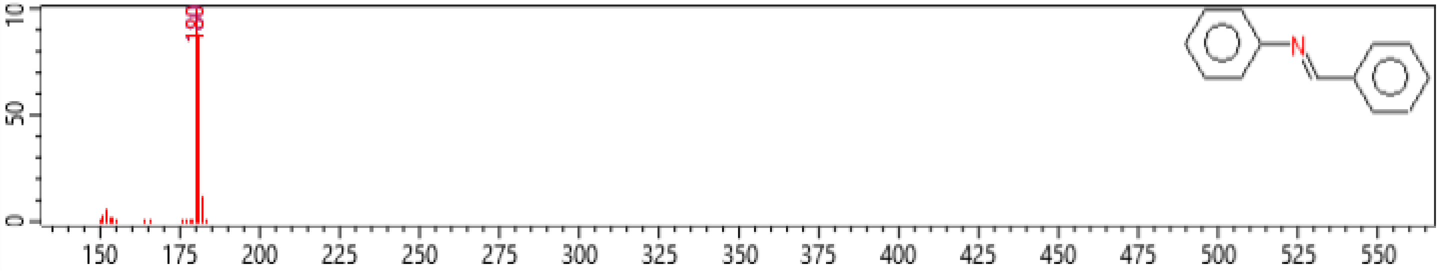

Benzenamine, N-(phenylmethylene)-

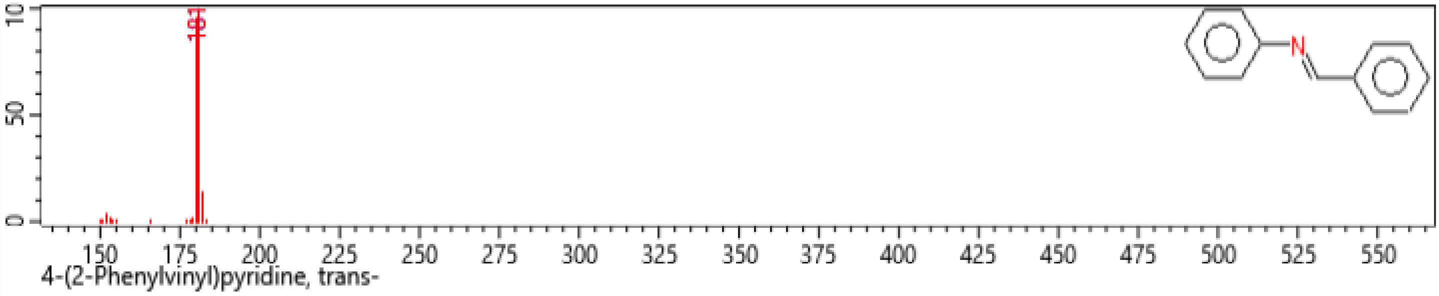


4-(2-Phenylvinyl)pyridine, trans-

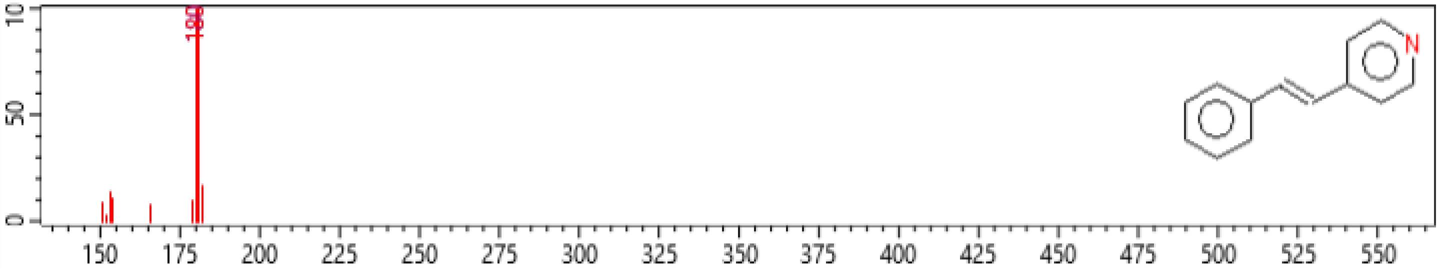


**Chavicol TMS**

Peak: 9.09 - 9.10 min (scan #582 - #586) Subtracted noise: 9.05 - 9.07 min (scan #573 - #577), 9.11 - 9.12 min (scan #589 - #592)

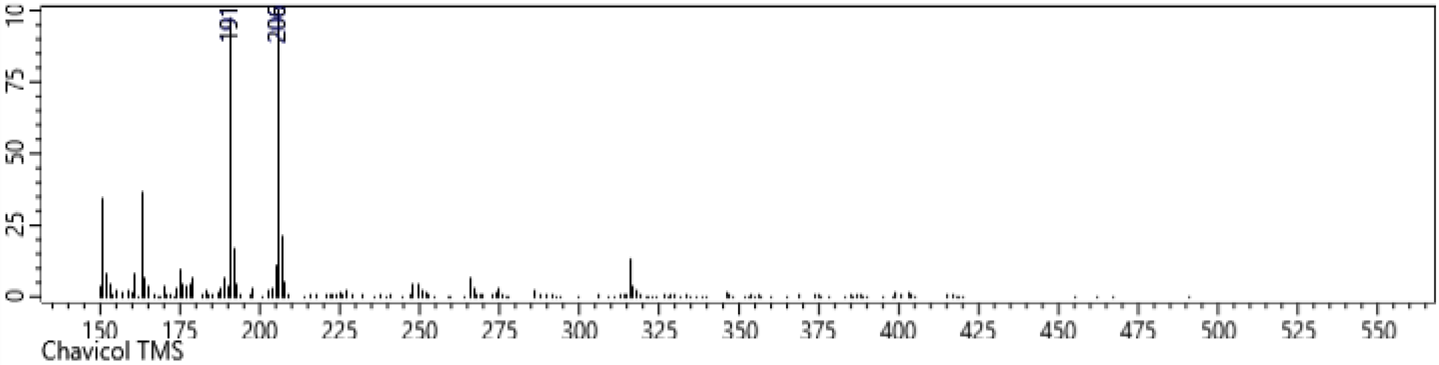


Chavicol TMS

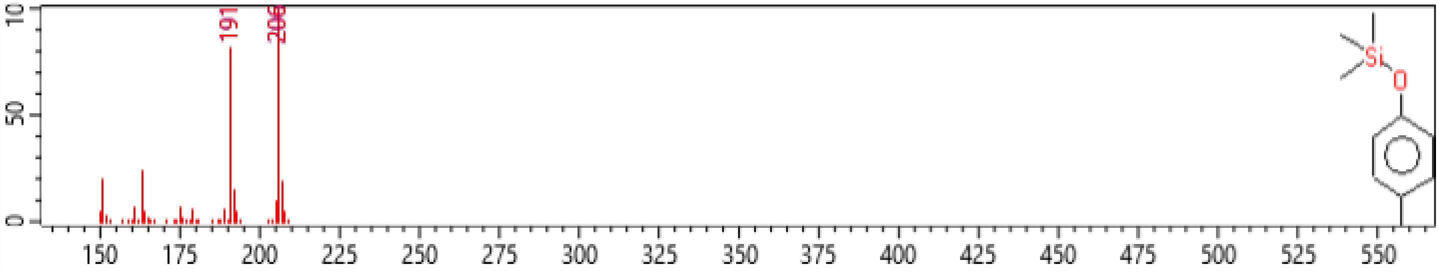


1H-Inden-1-one, 2,3-dihydro-5,6-dimethoxy-3-methyl-

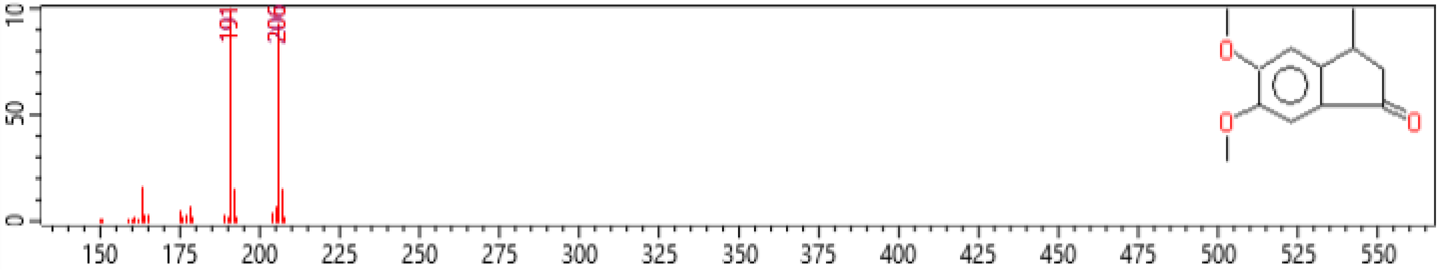

1H-Inden-1-one, 2,3-dihydro-5,6-dimethoxy-3-methyl-

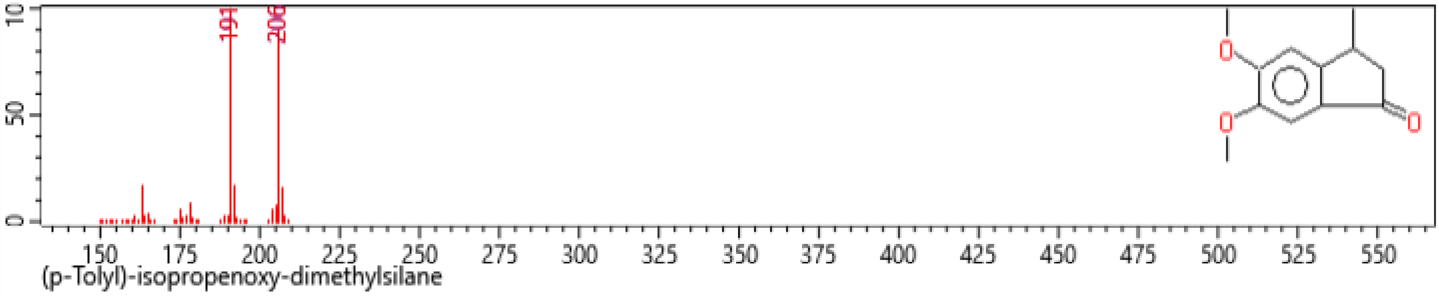


(p-Tolyl)-isopropenoxy-dimethylsilane

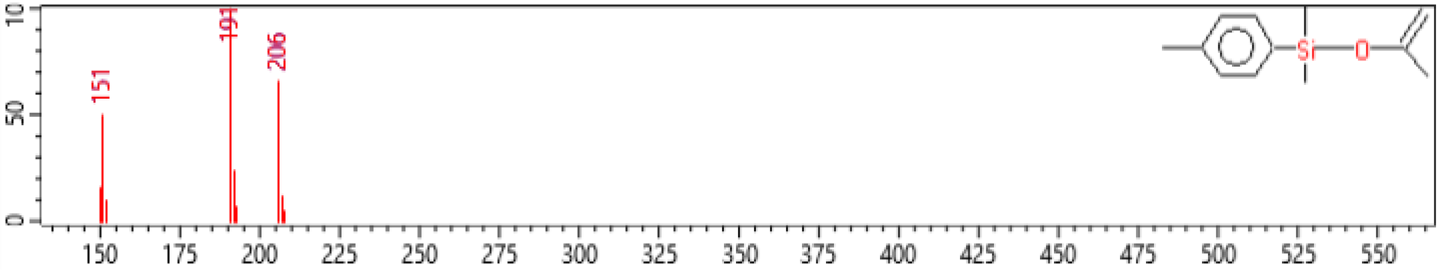


Cyclohexanone, 2,6-bis(2-methylpropylidene)-

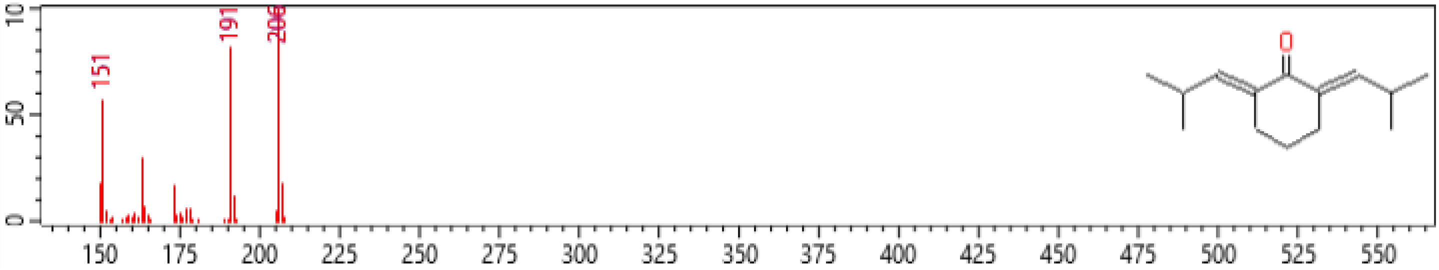


**Decamethyltetrasiloxane**

Peak: 9.16 - 9.17 min (scan #602 - #606) Subtracted noise: 9.12 - 9.14 min (scan #592 - #597), 9.19 - 9.20 min (scan #610 - #615)

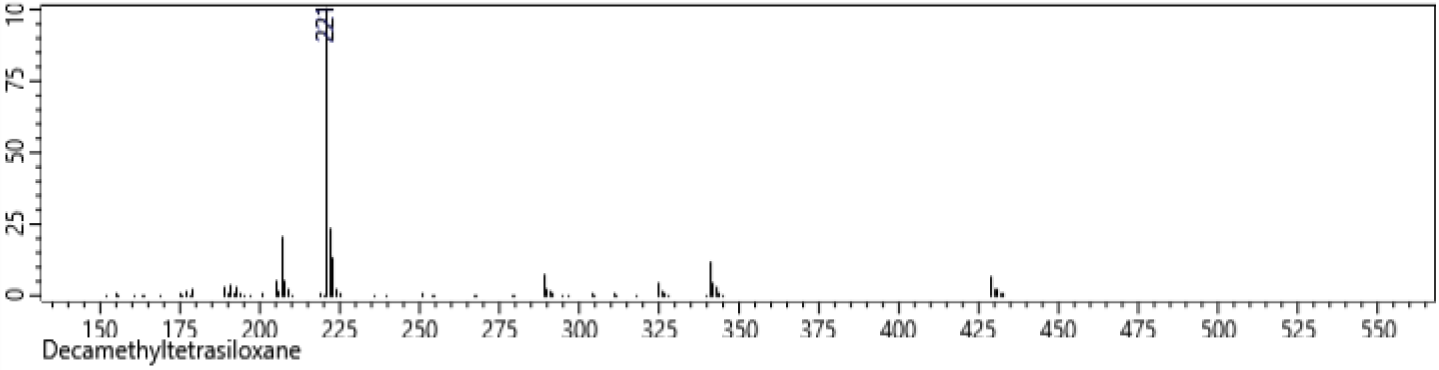


Decamethyltetrasiloxane

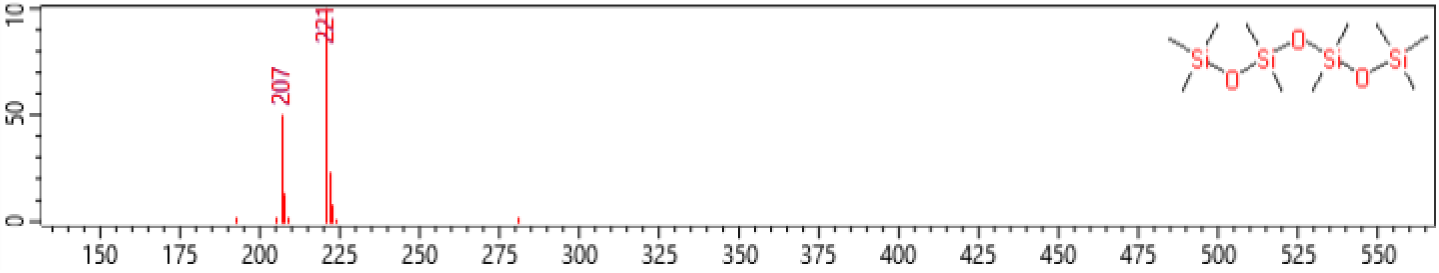

Trisiloxane, octamethyl-

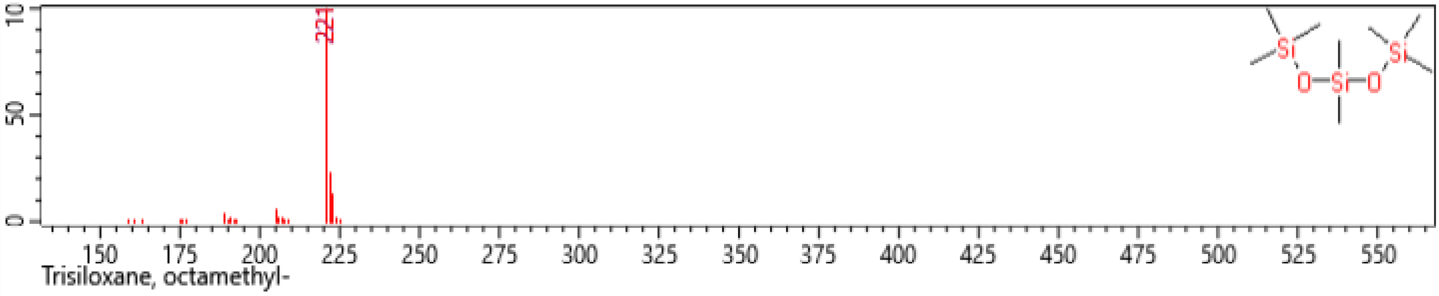


Trisiloxane, octamethyl-

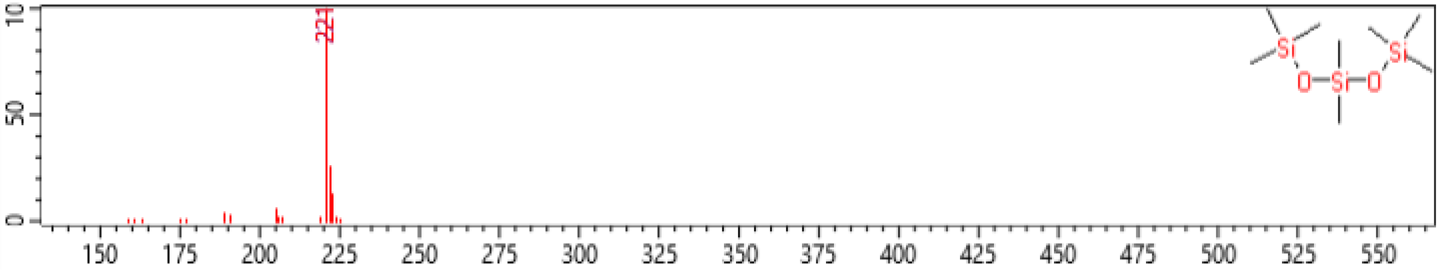


Trisiloxane, octamethyl-

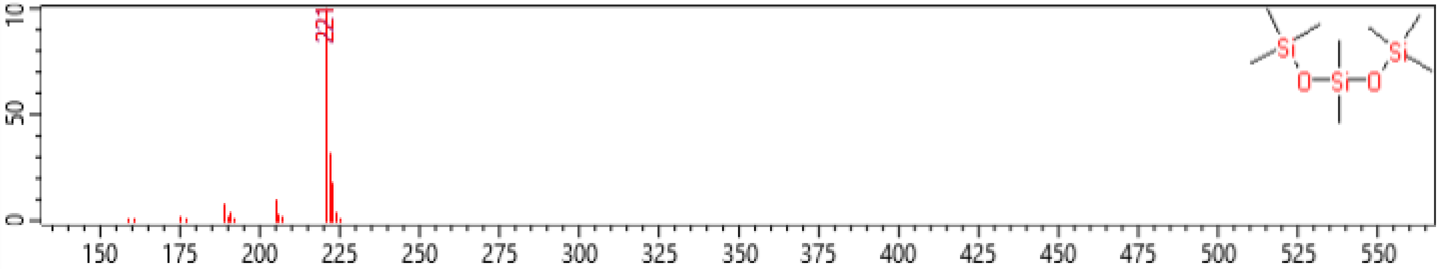


2-Mercapto-4,6-dimethylnicotinonitrile, TBDMS derivative

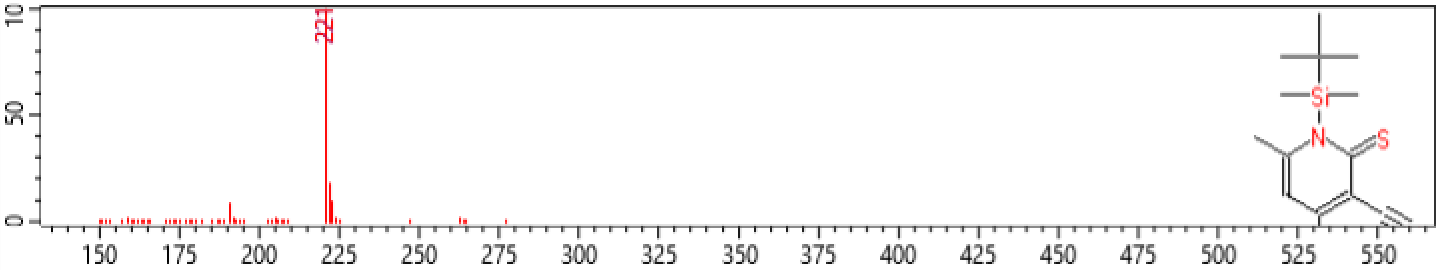


**Tecloftalam**

Peak: 9.29 - 9.30 min (scan #638 - #641) Subtracted noise: 9.26 - 9.27 min (scan #631 - #634), 9.30 - 9.31 min (scan #643 - #645)

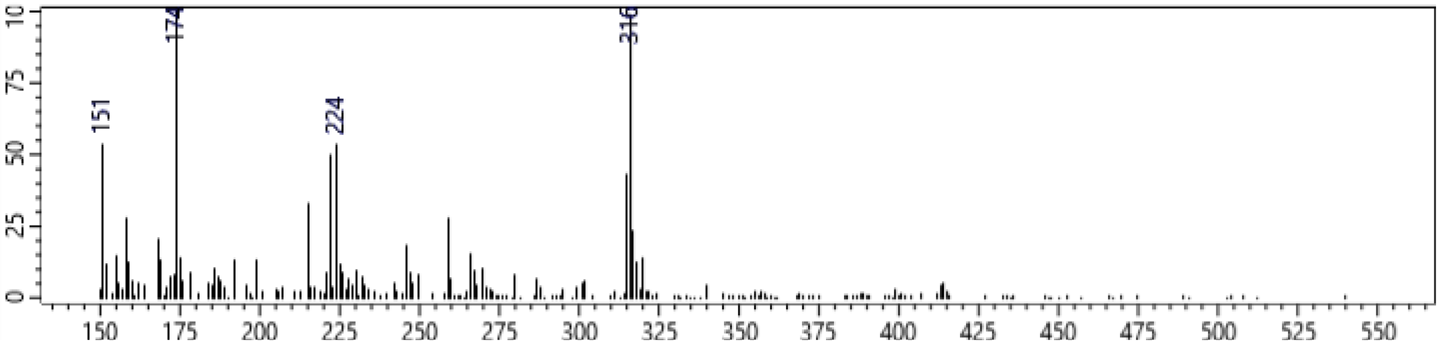

Tecloftalam

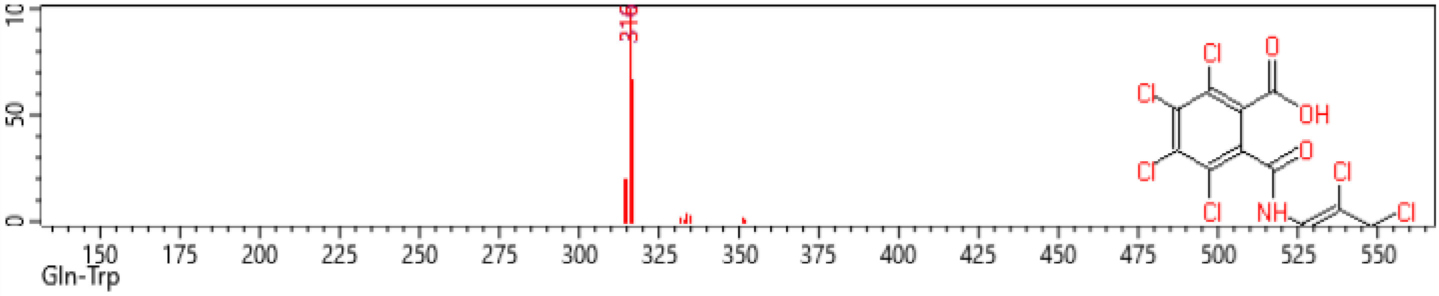


Gln-Trp

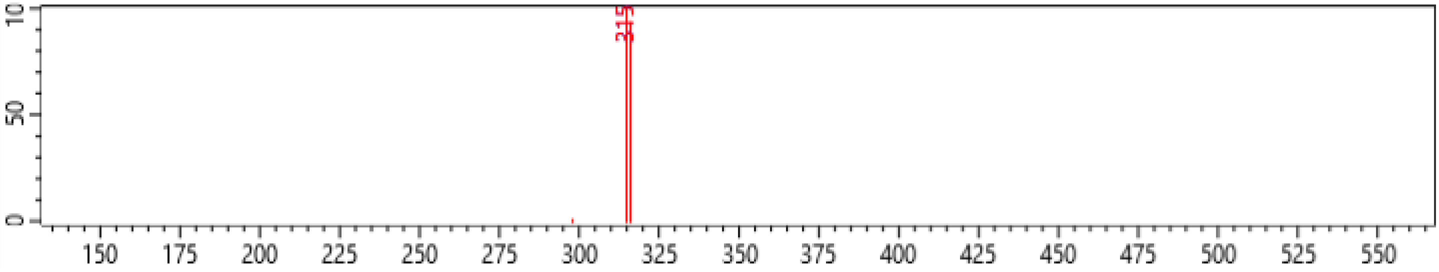


Gln-Trp

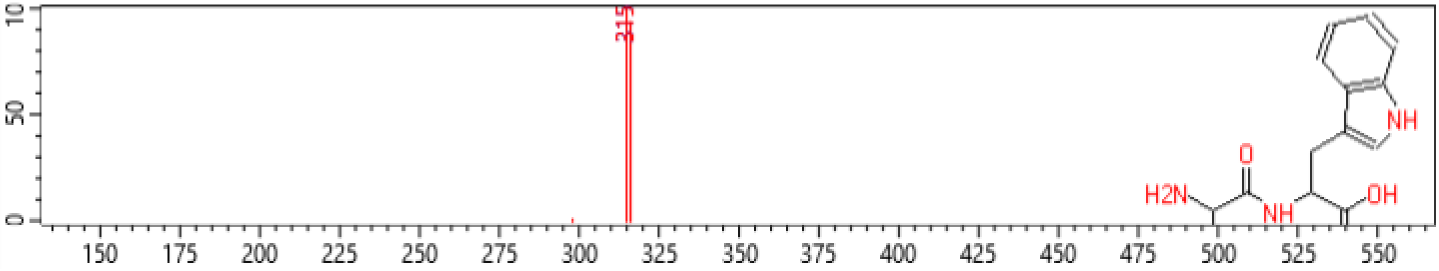


2-(2,4-Dichlorophenyl)-3-[4-(dimethylamino)phenyl]acrylonitrile

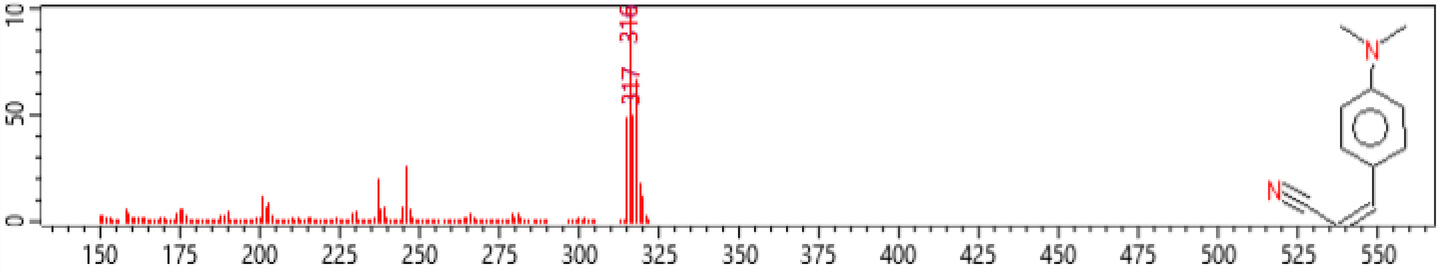


(3S,9aS)-3-Benzyl-2,3,4,6,7,8,9,9a-octahydropyrido[1,2-a]pyrazin-1-one, TMS

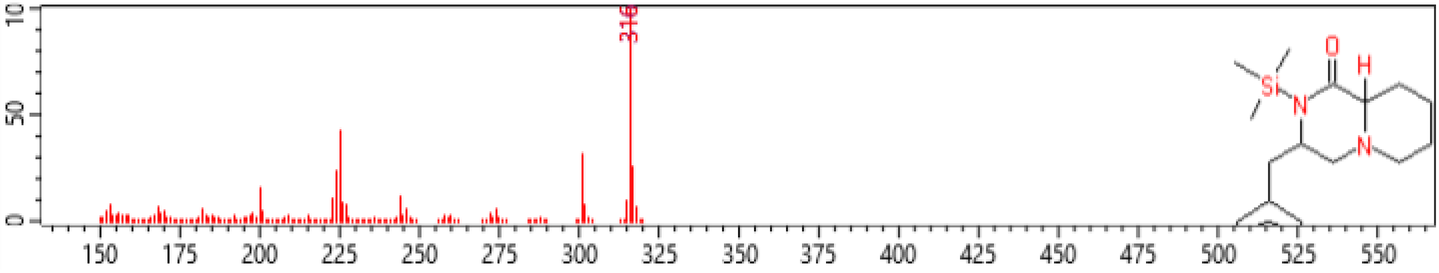

**Pyrido[2,3-d]pyrimidin-7(8H)-one, 6-(2,6-dichlorophenyl)-2-[[3-(hydroxymethyl)phenyl]amino]-8-methyl-**

Peak: 9.35 - 9.39 min (scan #657 - #667) Subtracted noise: 9.26 - 9.30 min (scan #631 - #642), 9.39 - 9.43 min (scan #667 - #677)

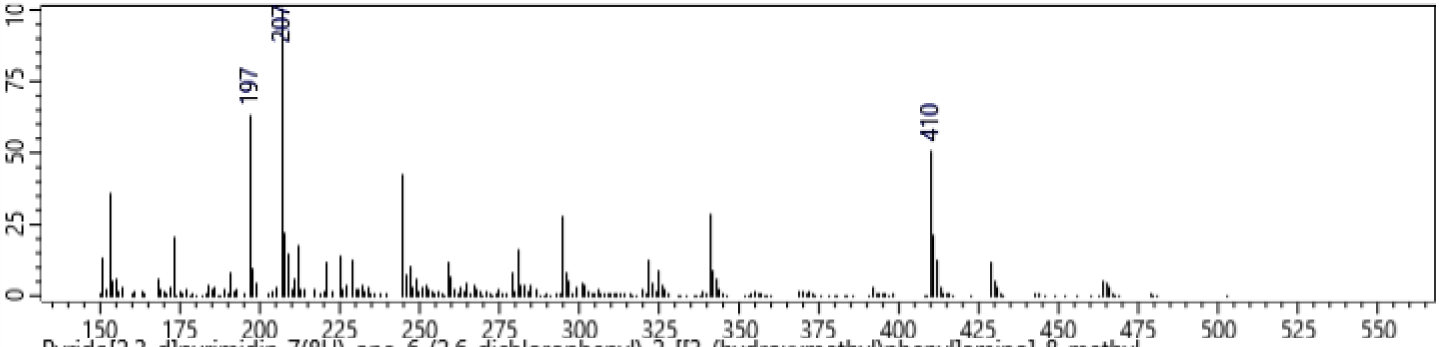


Pyrido[2,3-d]pyrimidin-7(8H)-one, 6-(2,6-dichlorophenyl)-2-[[3-(hydroxymethyl)phenyl]amino]-8-methyl-

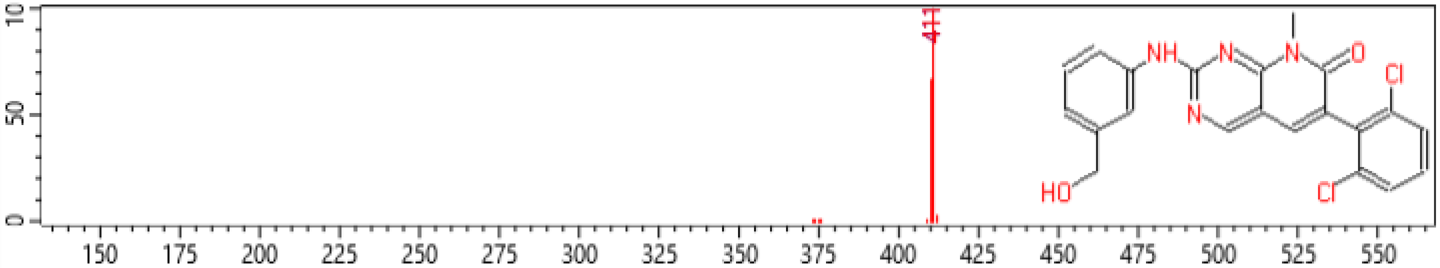


Pyrido[2,3-d]pyrimidin-7(8H)-one, 6-(2,6-dichlorophenyl)-2-[[3-(hydroxymethyl)phenyl]amino]-8-methyl-

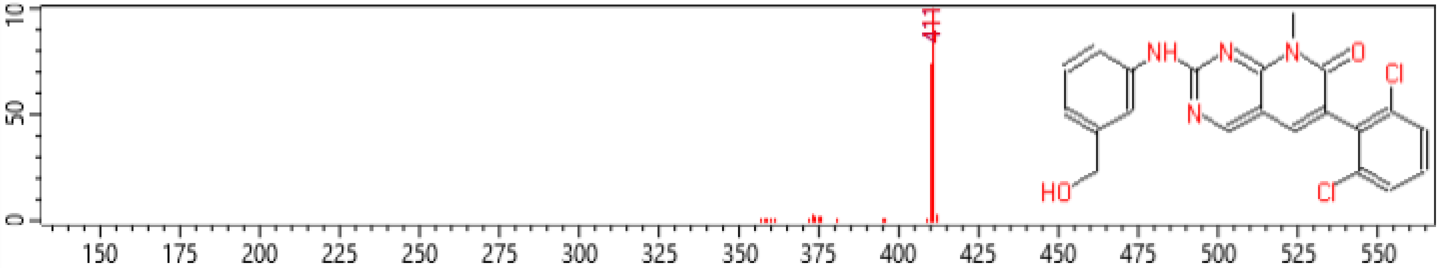


3,6-Dihydroxy-13-methoxy-5,5,7a,9,14b-pentamethyl-2,3,7,7a,10,14,14a,14b-octahydro-6H-3,5a-epoxyfuro[3,4-i]oxepino[4,3-a]xanth

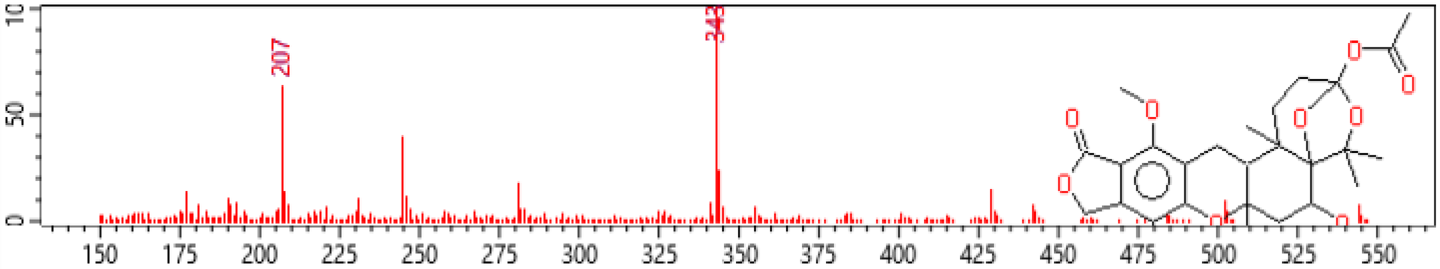


(3β,5α)-4,4-Dimethylcholesta-8,14,24-trien-3-ol

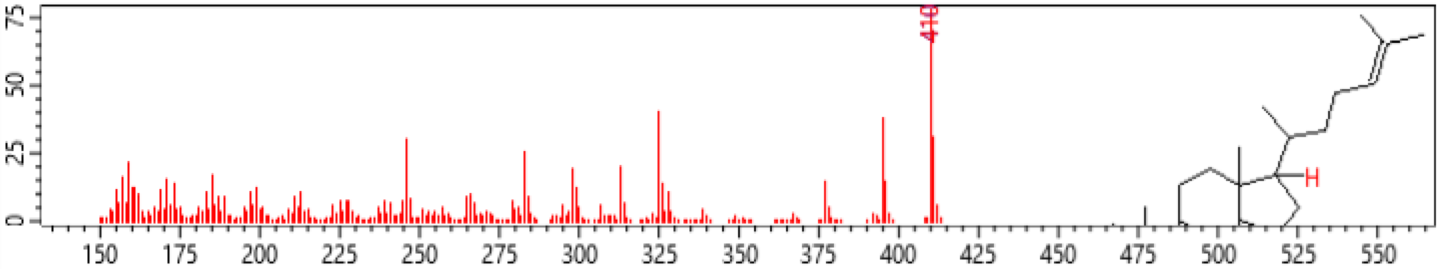

5,5'-Diallyl-2,2'-biphenyldiol, bis(trimethylsilyl) ether

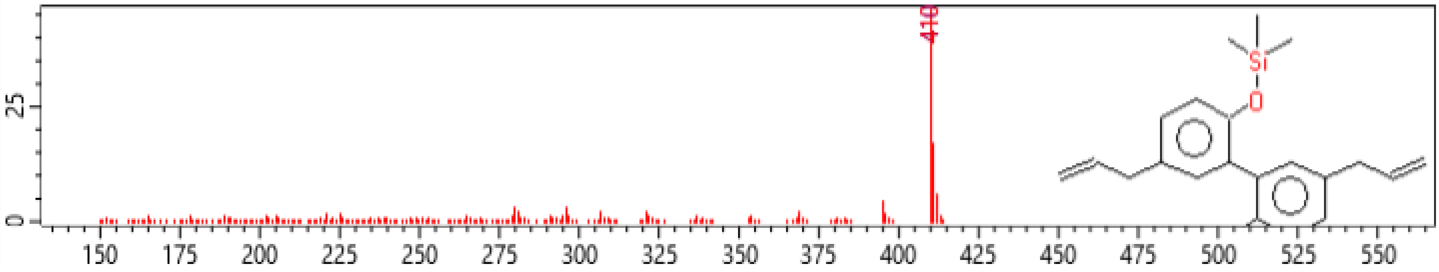


**Tetrasiloxane, decamethyl-**

Peak: 9.39 - 9.41 min (scan #668 - #672) Subtracted noise: 9.37 - 9.39 min (scan #661 - #666), 9.43 - 9.44 min (scan #678 - #682)

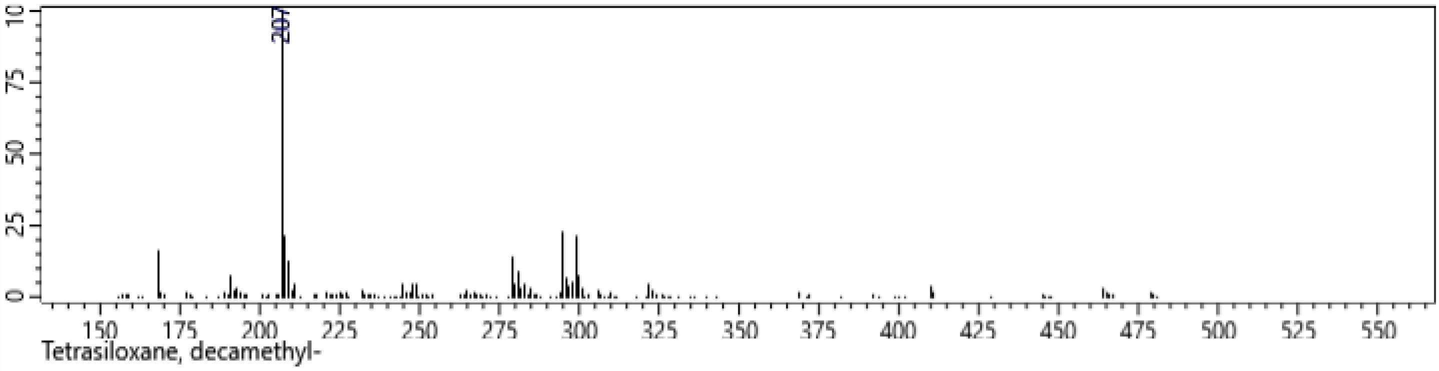


Tetrasiloxane, decamethyl-

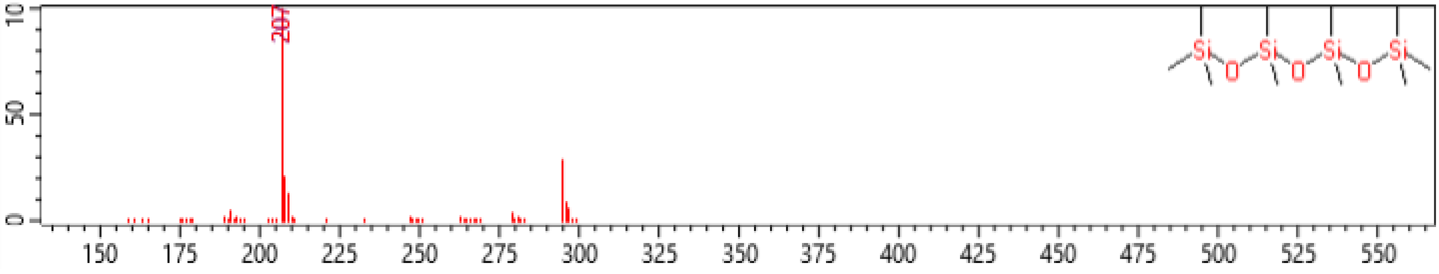


Tetrasiloxane, decamethyl-

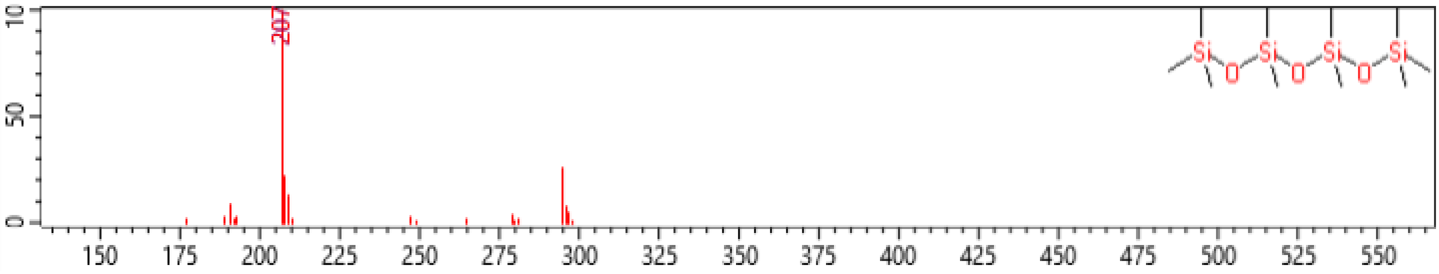


Tetrasiloxane, decamethyl-

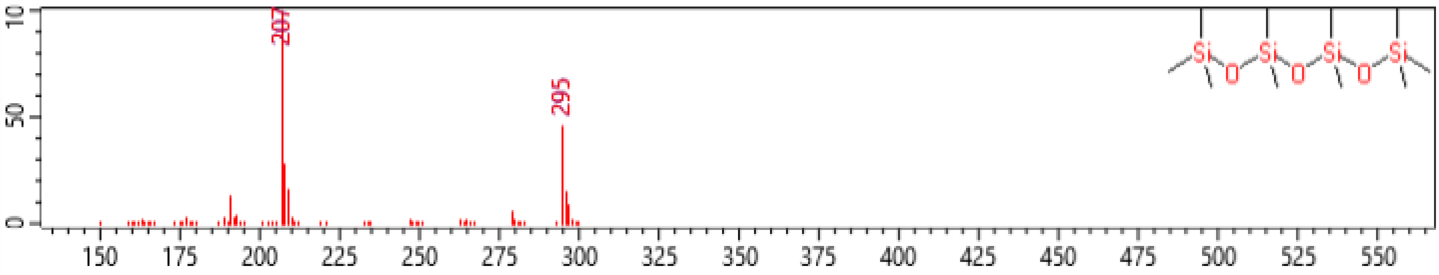

Methyltris(trimethylsiloxy)silane

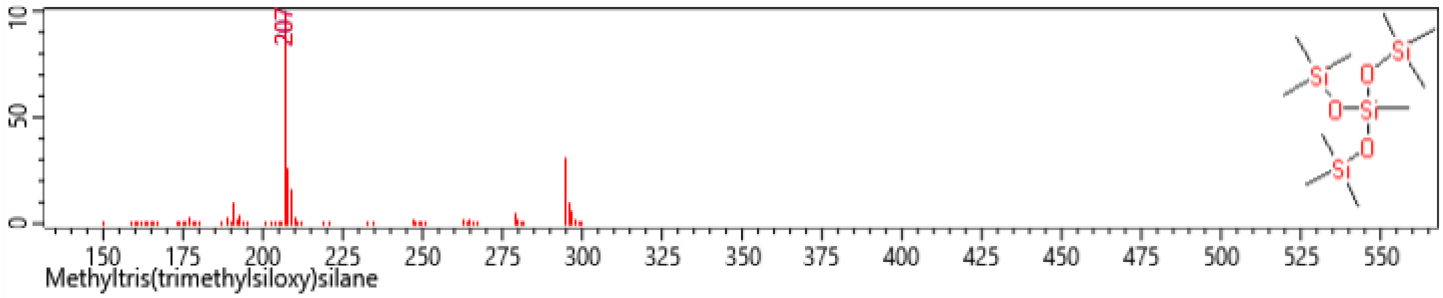


Methyltris(trimethylsiloxy)silane

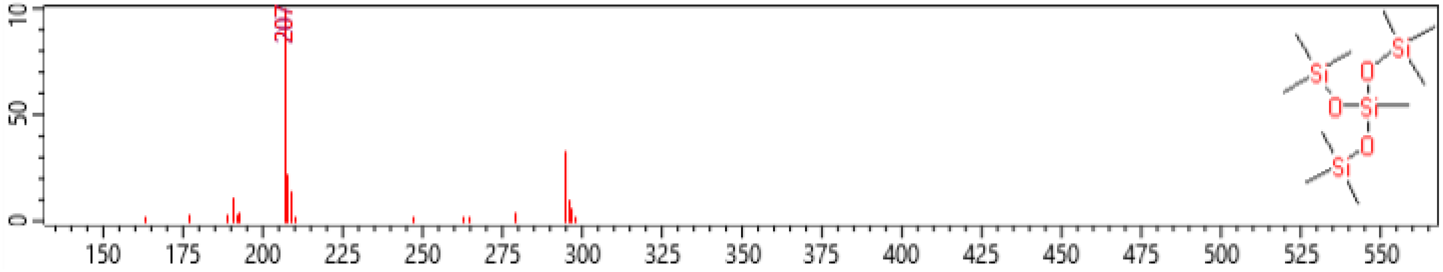


**Hexasiloxane, tetradecamethyl-**

Peak: 9.50 - 9.55 min (scan #698 - #711) Subtracted noise: 9.45 - 9.49 min (scan #683 - #696), 9.60 - 9.65 min (scan #725 - #739)

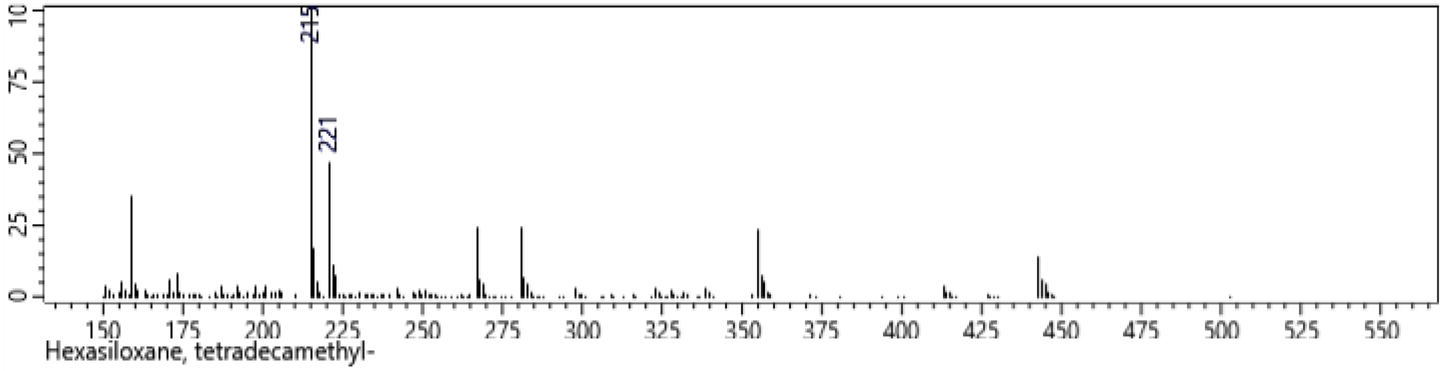


Hexasiloxane, tetradecamethyl-

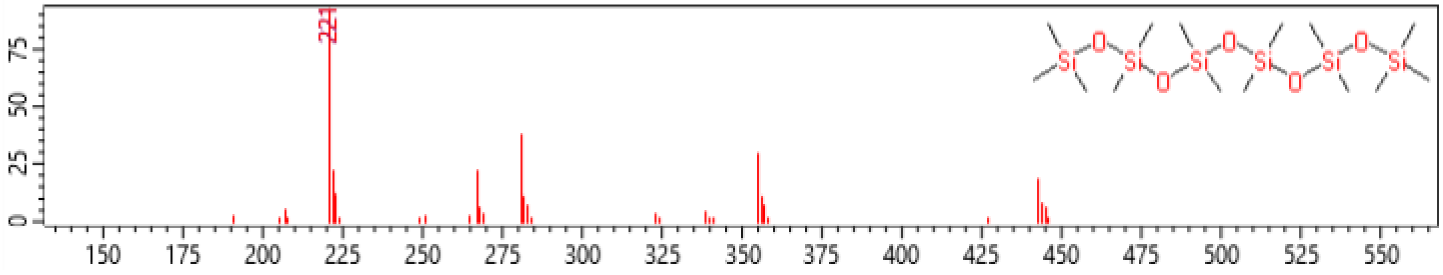


Hexasiloxane, tetradecamethyl-

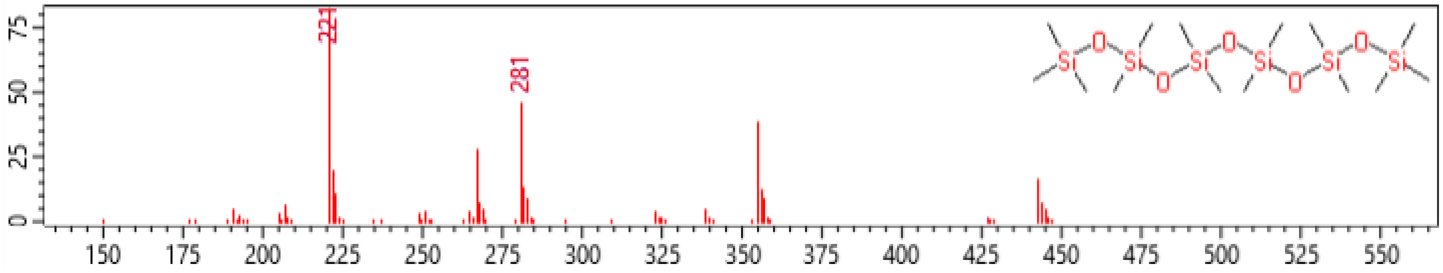

1,1,1,3,5,7,7,7-Octamethyl-3,5-bis(trimethylsiloxy)tetrasiloxane

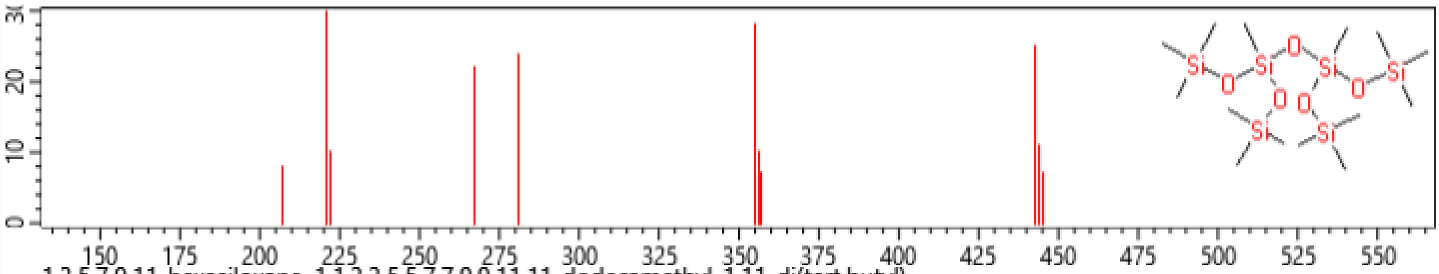

1,3,5,7,9,11-hexasiloxane, 1,1,3,3,5,5,7,7,9,9,11,11-dodecamethyl-1,11-di(tert.butyl)-

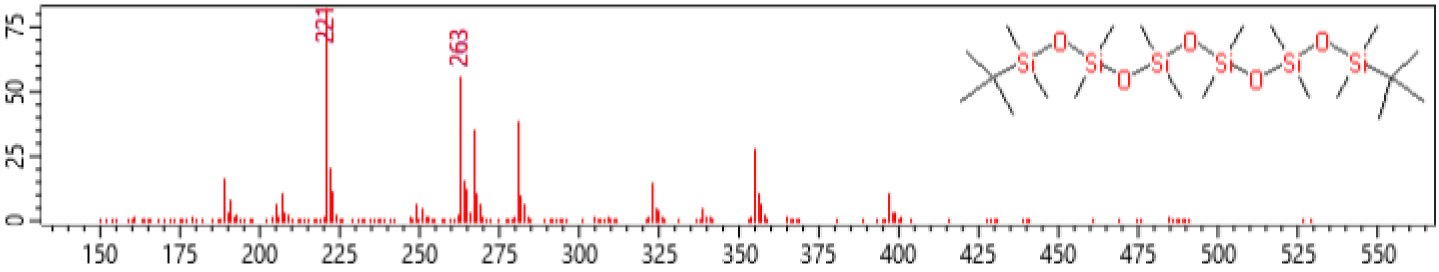


Glafenin

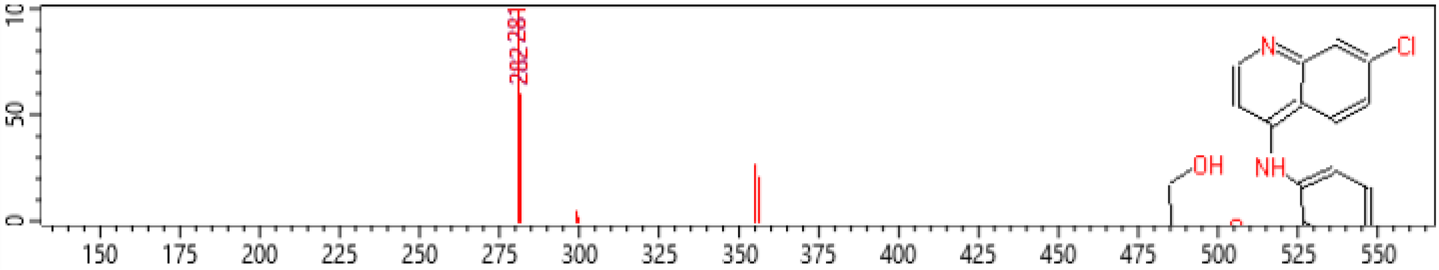


**Hexasiloxane, tetradecamethyl-**

Peak: 9.68 - 9.70 min (scan #748 - #754) Subtracted noise: 9.64 - 9.66 min (scan #738 - #743), 9.71 - 9.73 min (scan #757 - #763)

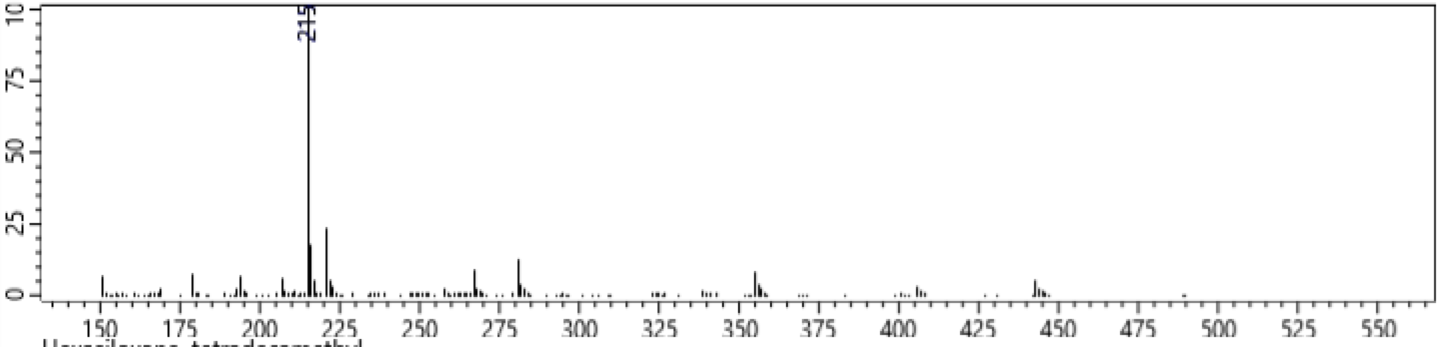


Hexasiloxane, tetradecamethyl-

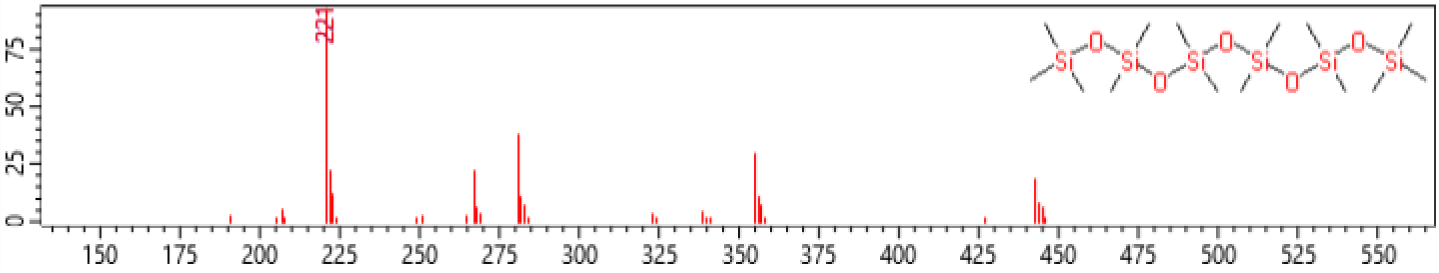

Hexasiloxane, tetradecamethyl-

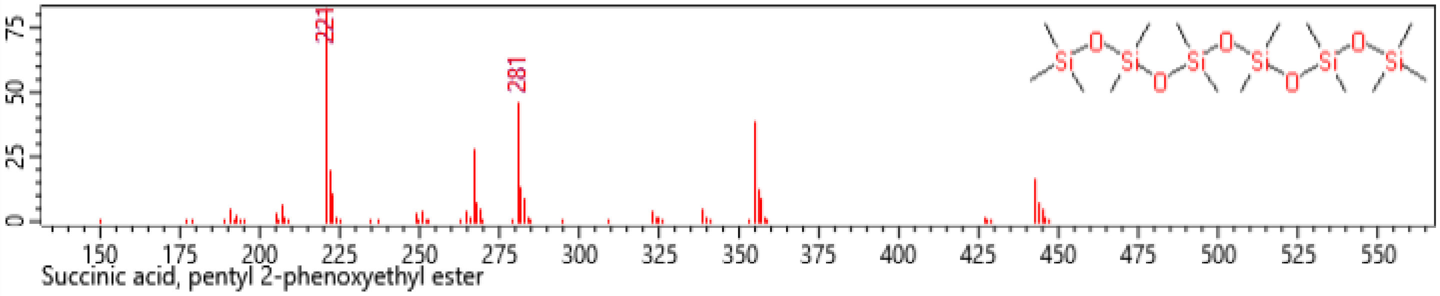


Succinic acid, pentyl 2-phenoxyethyl ester

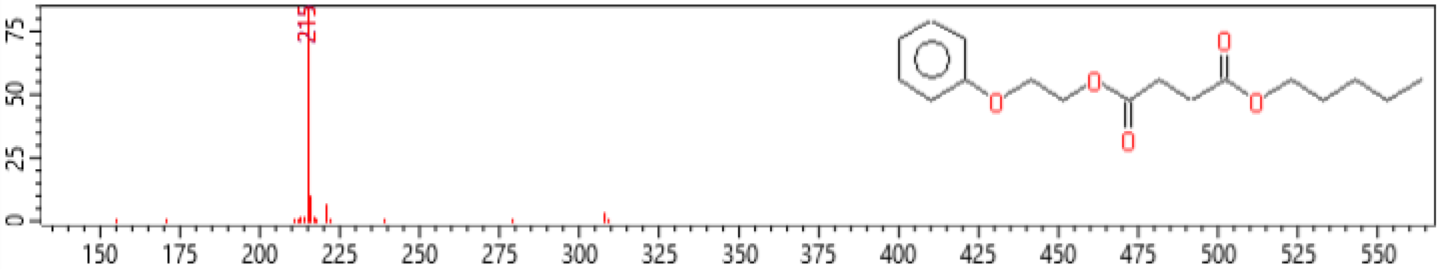


Heptacosan-9-ol, O-TMS

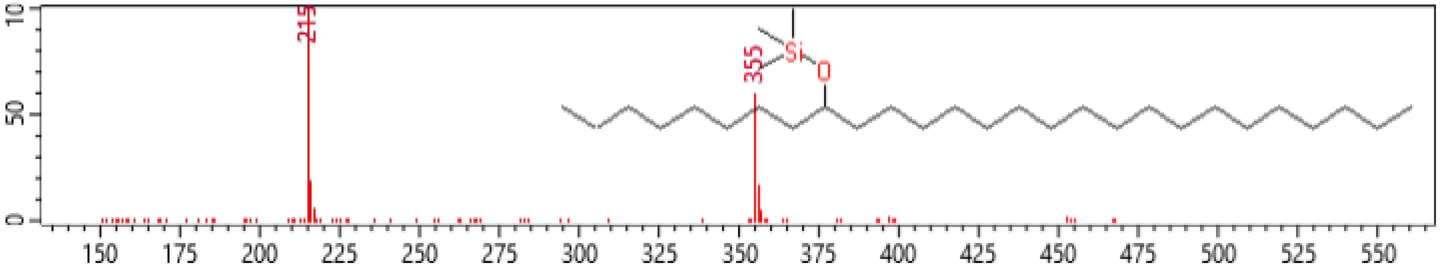


Zuclopentixol, TMS derivative

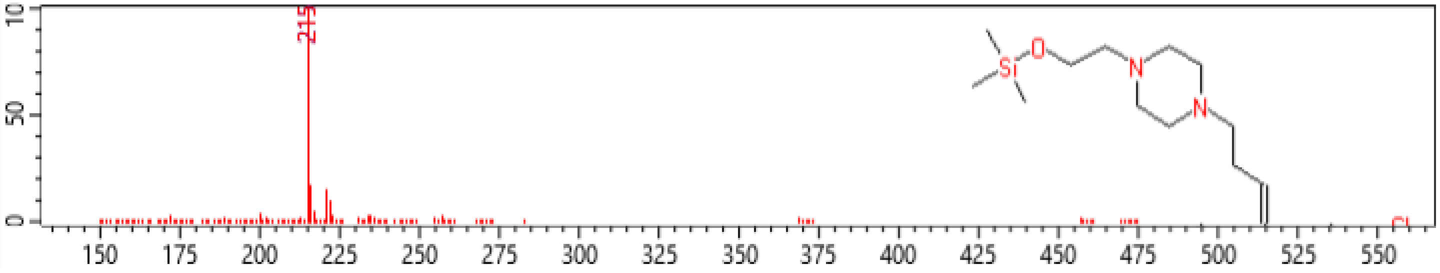


**Vanillin, TMS derivative**

Peak: 9.86 - 9.91 min (scan #799 - #812) Subtracted noise: 9.78 - 9.83 min (scan #776 - #789), 9.93 - 9.98 min (scan #819 - #831)

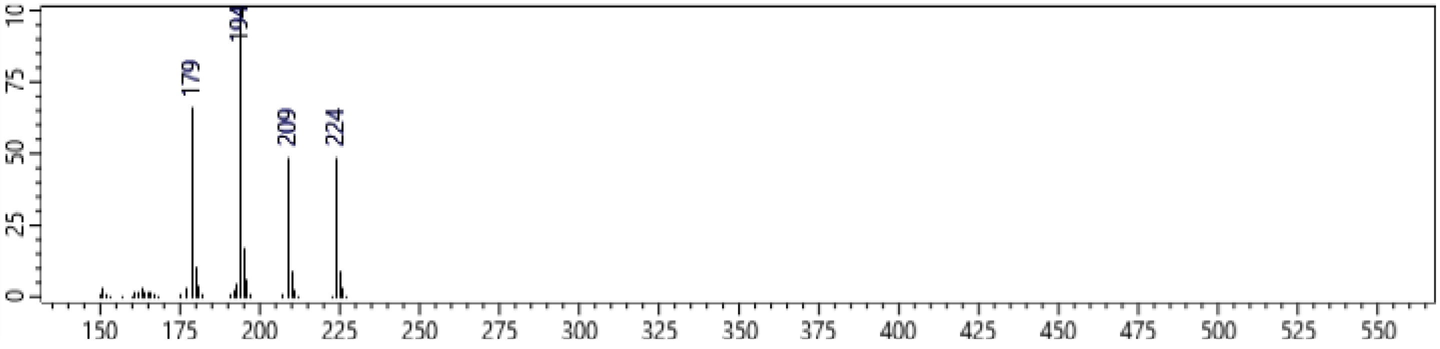

Vanillin, TMS derivative

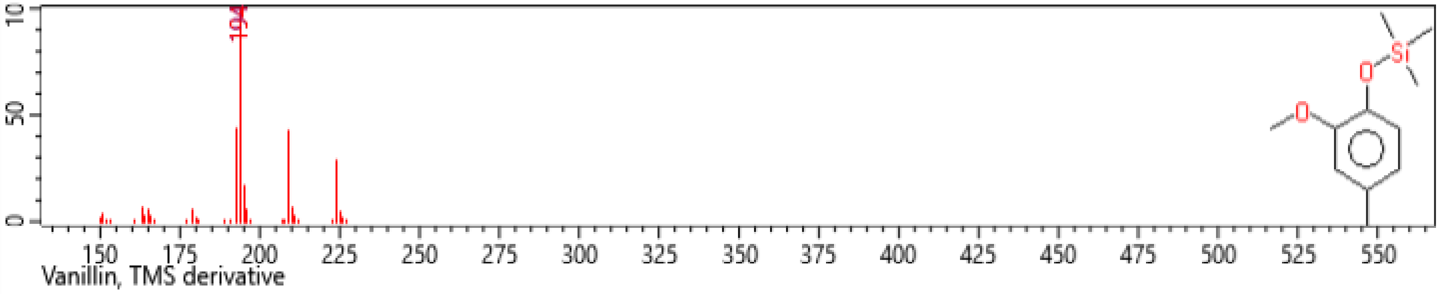


Vanillin, TMS derivative

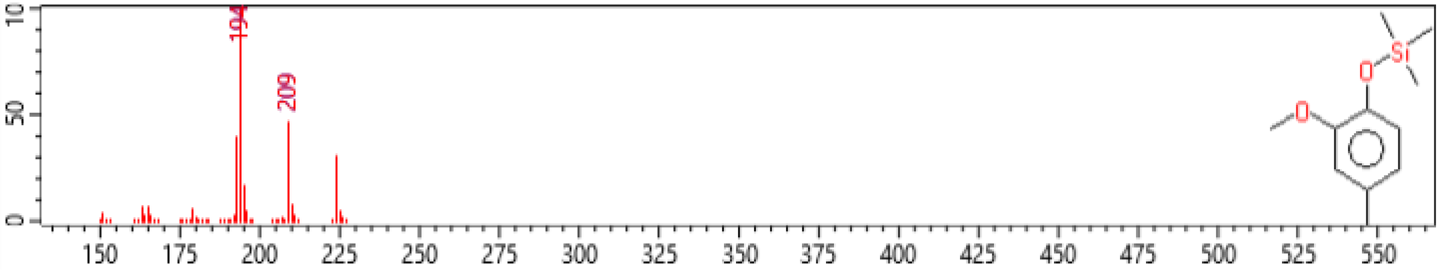


Vanillin, TMS derivative

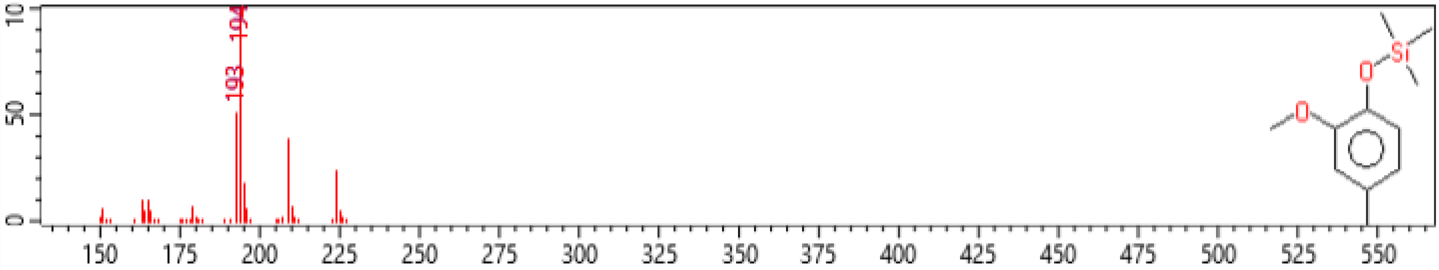


2-Methoxyphenethyl alcohol, TMS derivative

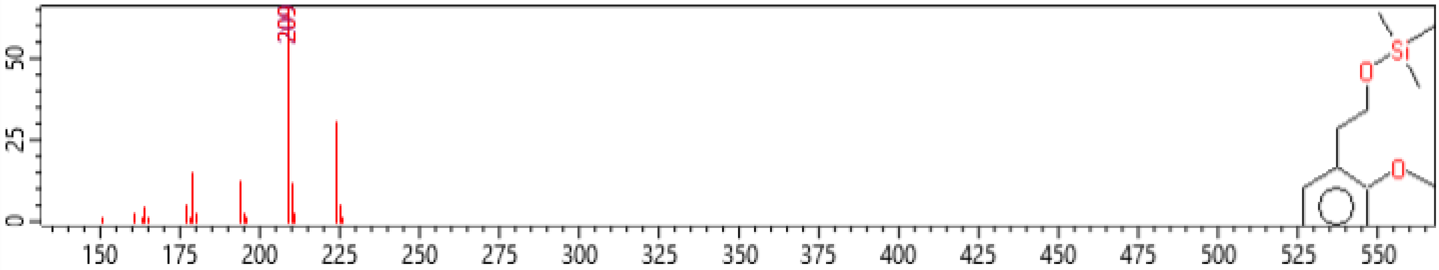


3-Hydroxy-4-methoxybenzaldehyde, TMS

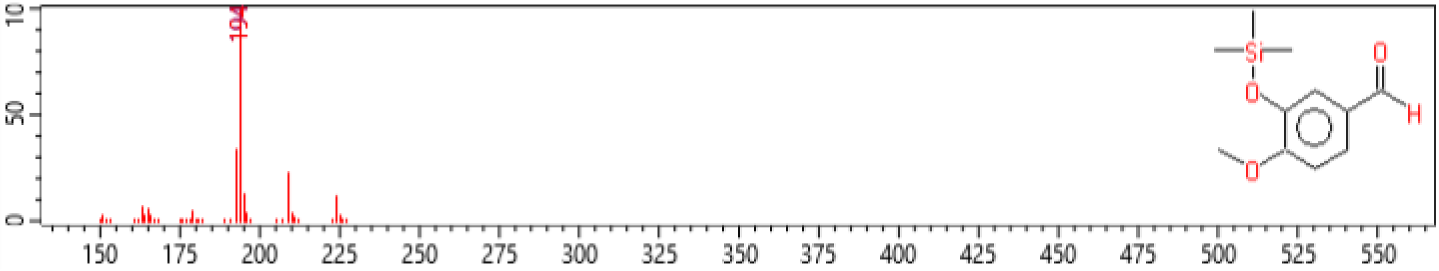

**2'-(Trimethylsilyl)oxy-6'-methoxychalcone**

Peak: 9.94 - 9.95 min (scan #821 - #823) Subtracted noise: 9.92 - 9.93 min (scan #816 - #818), 9.95 - 9.96 min (scan #824 - #827)

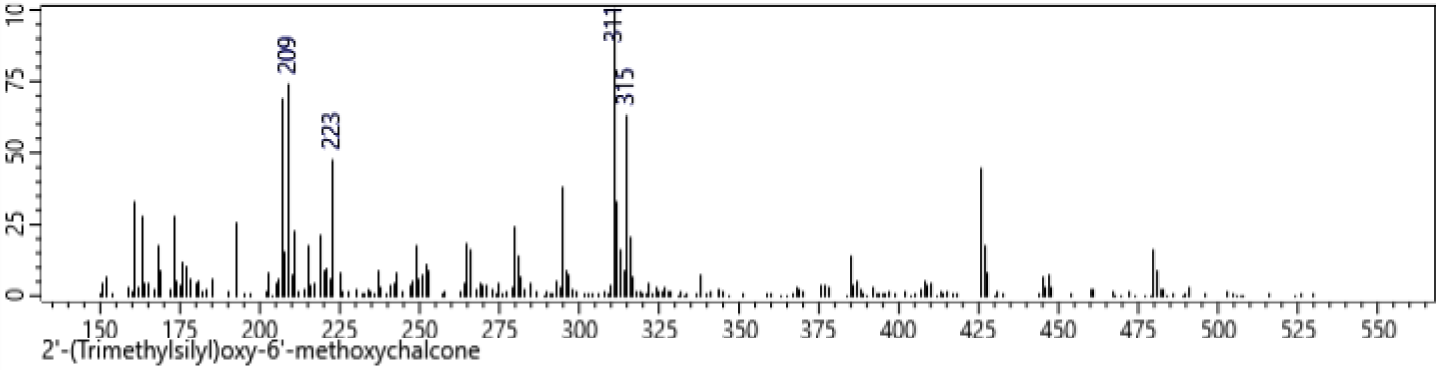


2'-(Trimethylsilyl)oxy-6'-methoxychalcone

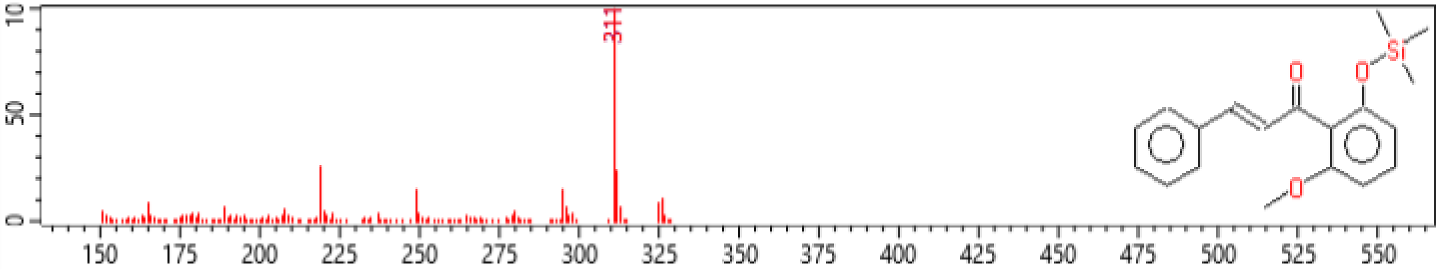


2'-(Tert.-butyldimethylsilyl)oxy-6'-methoxychalcone

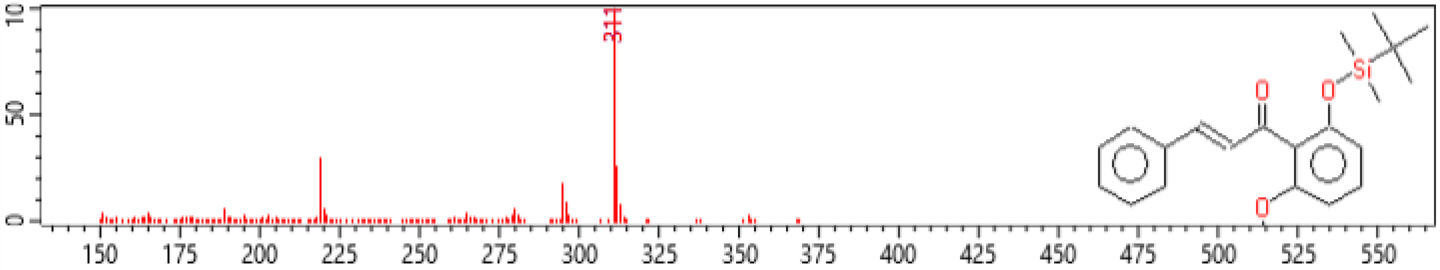


Betamethasone valerate

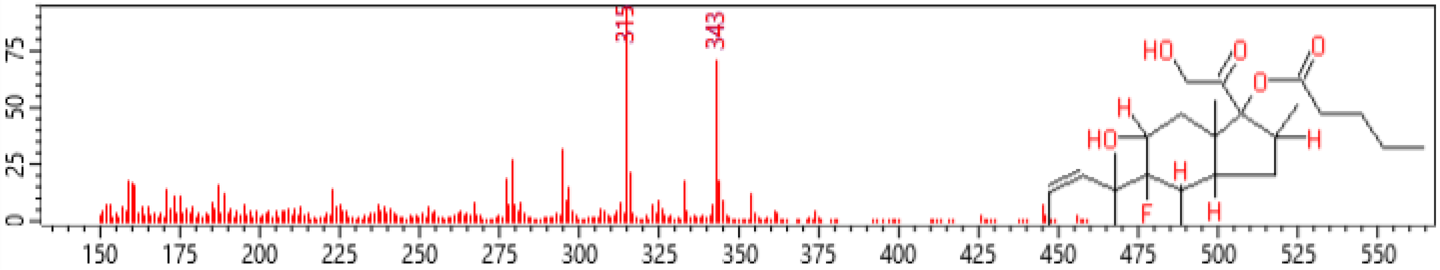


Phorbol 12,13-dihexanoate

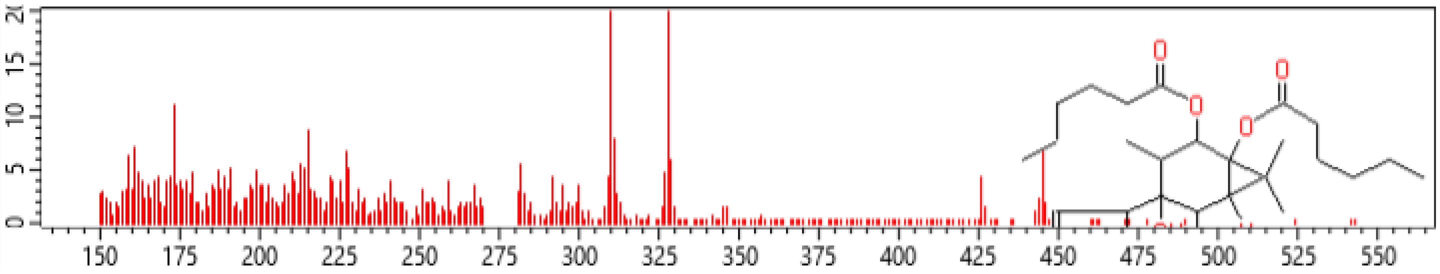

Dexamethasone-21-acetate

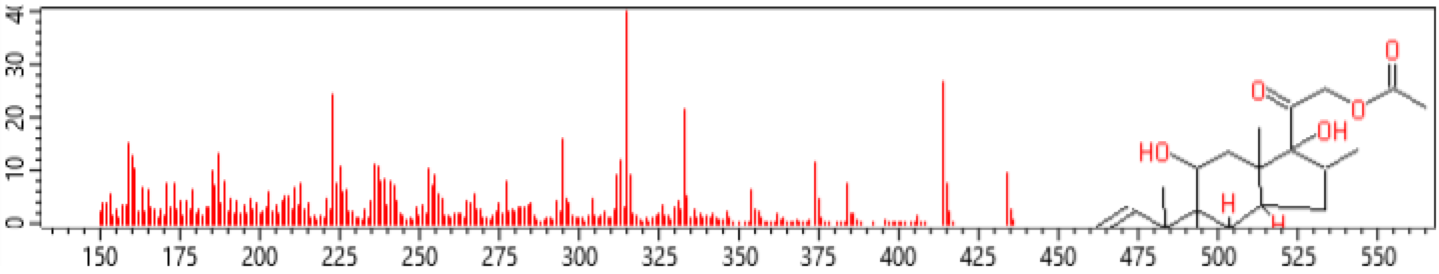


**1-(3-Chloro-2-pyridinyl)-4-piperidinylamine**

Peak: 10.28 - 10.30 min (scan #916 - #920) Subtracted noise: 10.26 - 10.27 min (scan #909 - #913), 10.30 - 10.32 min (scan #922 - #[illegible]

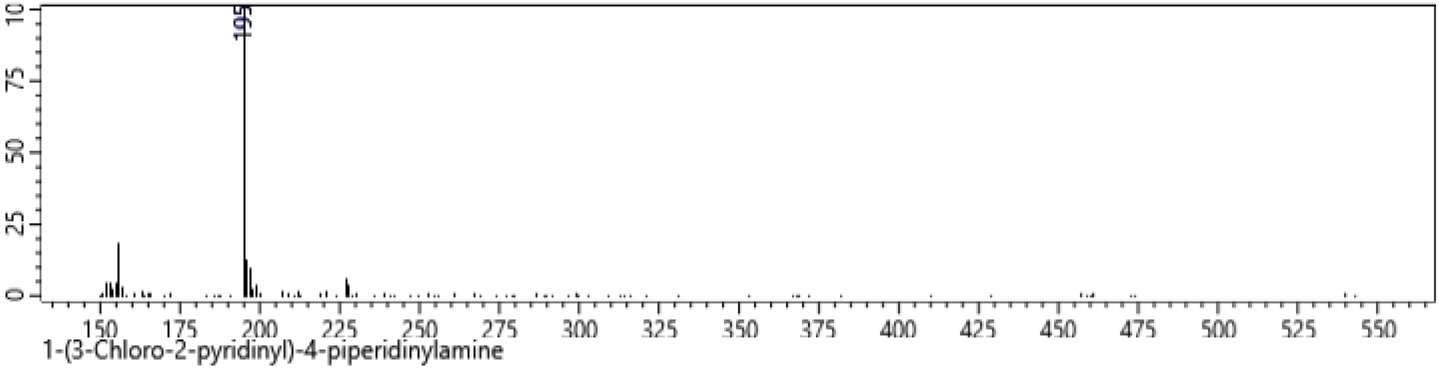


1-(3-Chloro-2-pyridinyl)-4-piperidinylamine

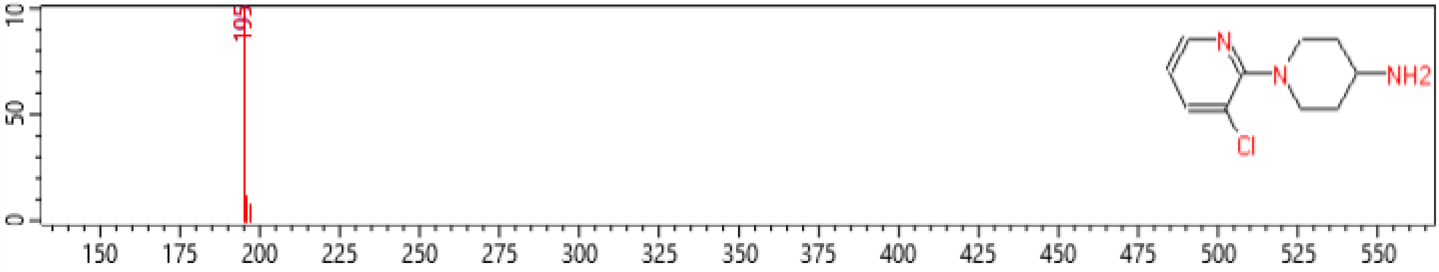


2-Isopropyl-2-phenyl-1,3-dithiane

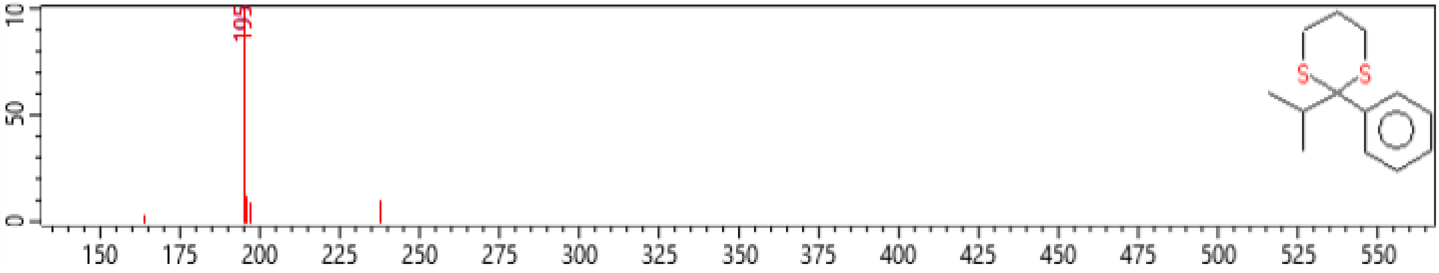


2-Amino-5-bromo-2'-fluorobenzophenone

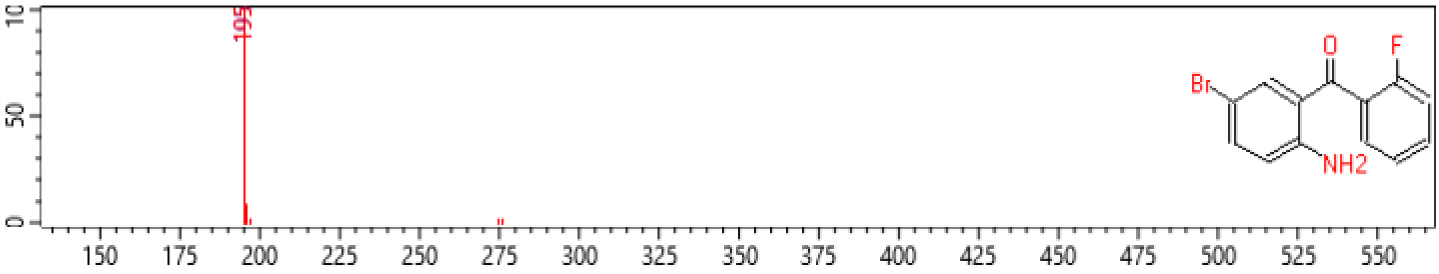

(2-Amino-5-bromophenyl)(2-chlorophenyl)methanone

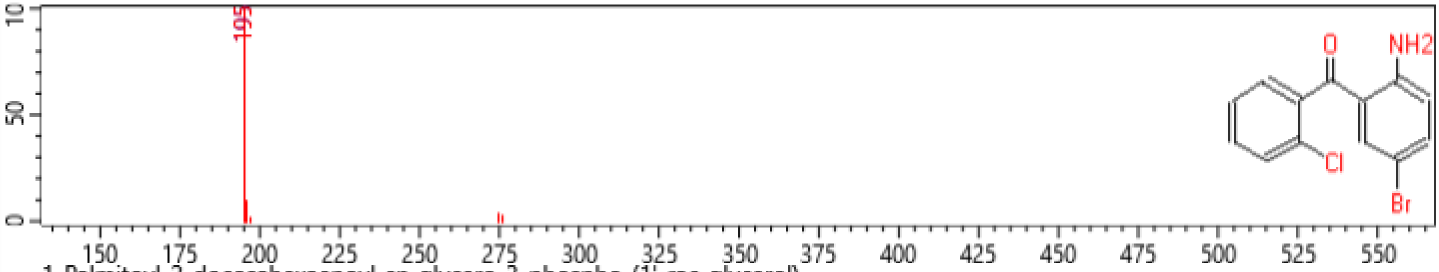


1-Palmitoyl-2-docosahexaenoyl-sn-glycero-3-phospho-(1'-rac-glycerol)

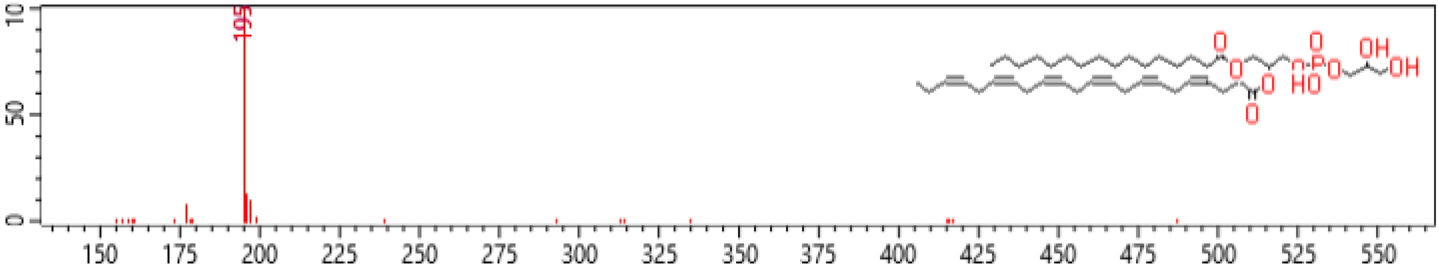


**2,8,9-Trioxa-5-aza-1-silabicyclo[3.3.3]undecane, 1-methyl-**

Peak: 10.37 - 10.38 min (scan #940 - #944) Subtracted noise: 10.34 - 10.35 min (scan #933 - #936), 10.39 - 10.41 min (scan #947 - #

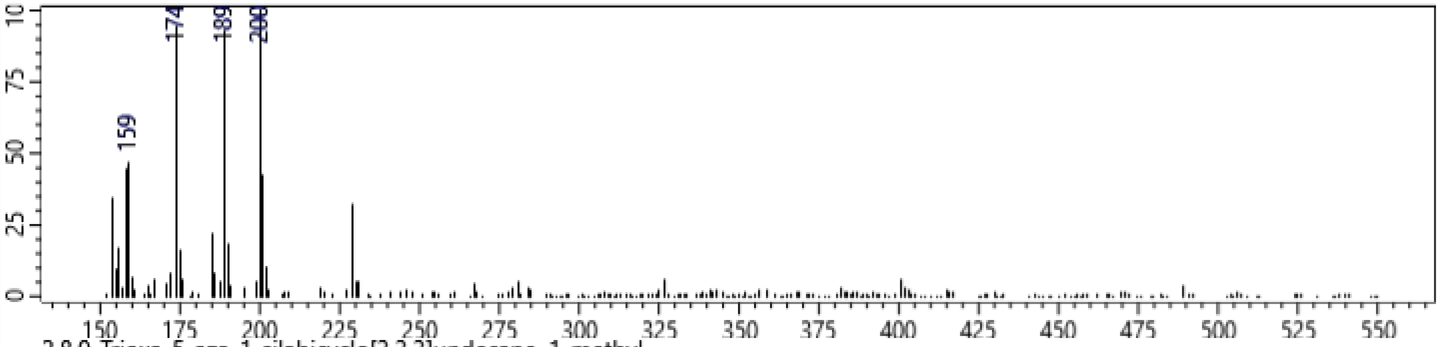


2,8,9-Trioxa-5-aza-1-silabicyclo[3.3.3]undecane, 1-methyl-

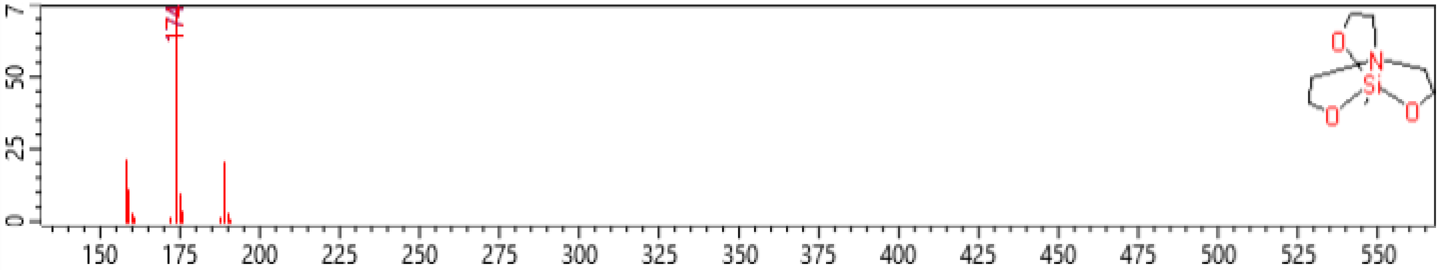


2,8,9-Trioxa-5-aza-1-silabicyclo[3.3.3]undecane, 1-methyl-

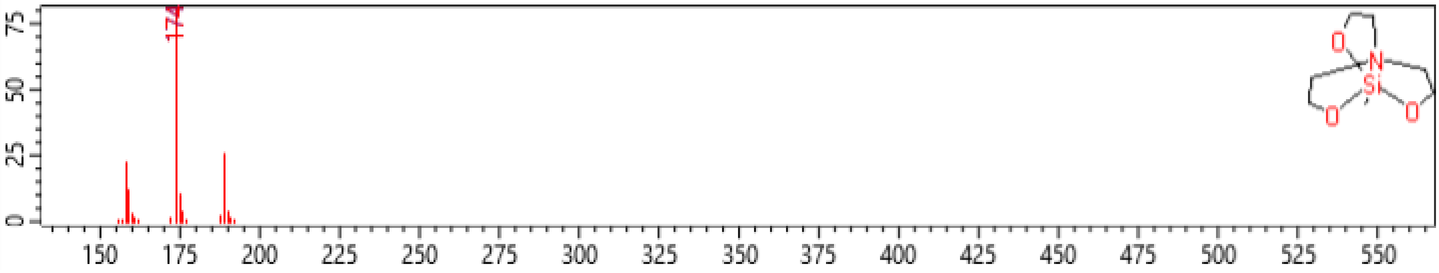

Quinoline, 1,2,3,4-tetrahydro-2,2,4,8-tetramethyl-

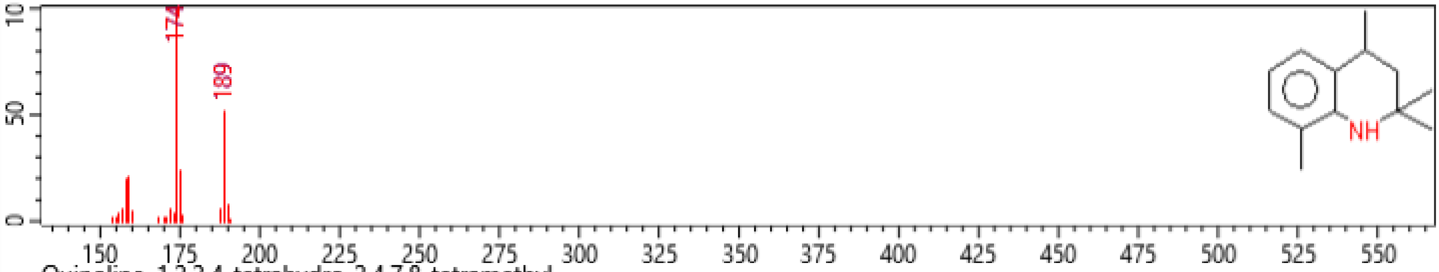


Quinoline, 1,2,3,4-tetrahydro-2,4,7,8-tetramethyl-

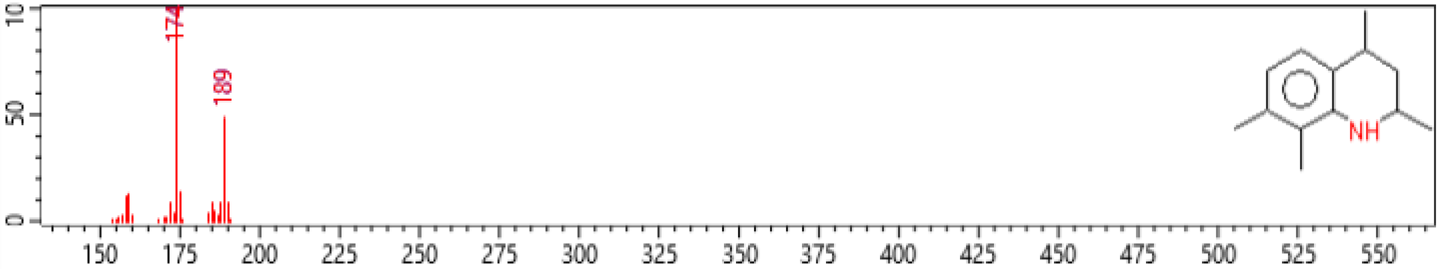


Quinoline, 1,2,3,4-tetrahydro-2,2,4,8-tetramethyl-

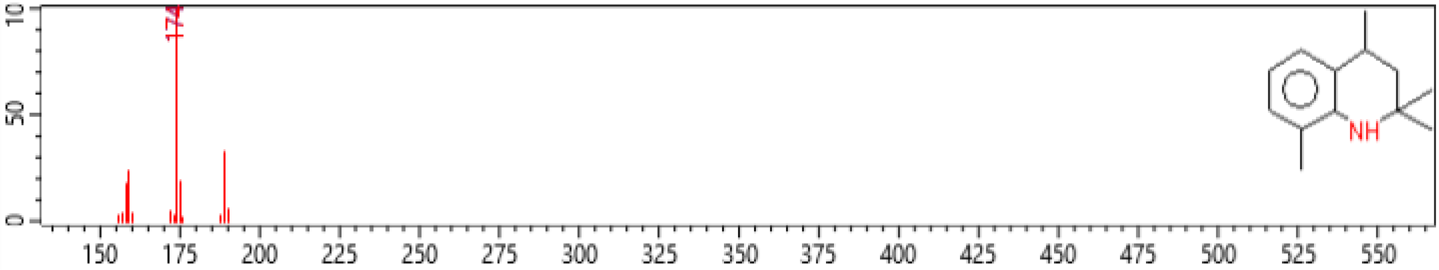


**2,6-Dioxo-4-piperidinecarboxylic acid, 2TMS**

Peak: 10.40 - 10.42 min (scan #949 - #953) Subtracted noise: 10.38 - 10.39 min (scan #944 - #946), 10.42 - 10.44 min (scan #955 - #[illegible]

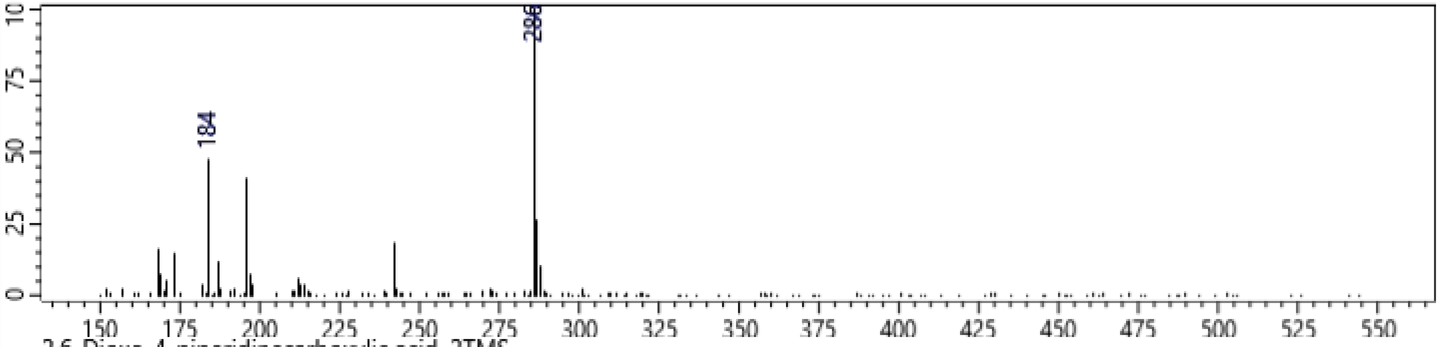


2,6-Dioxo-4-piperidinecarboxylic acid, 2TMS

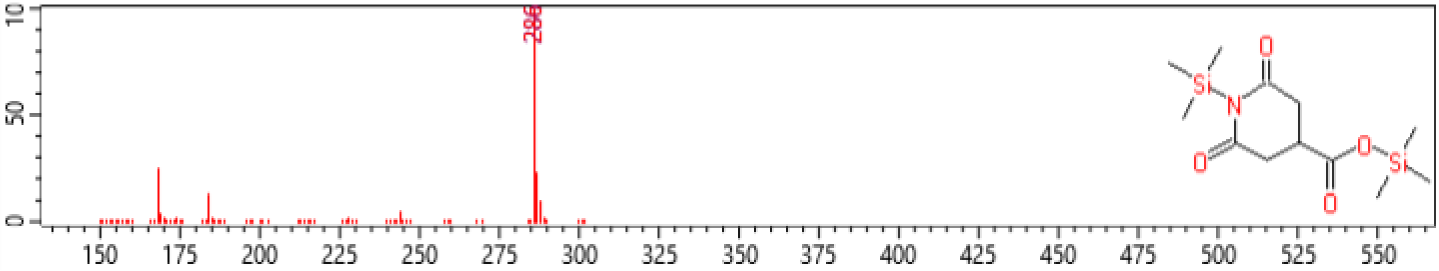

Tiglyl glycine, 2TMS derivative

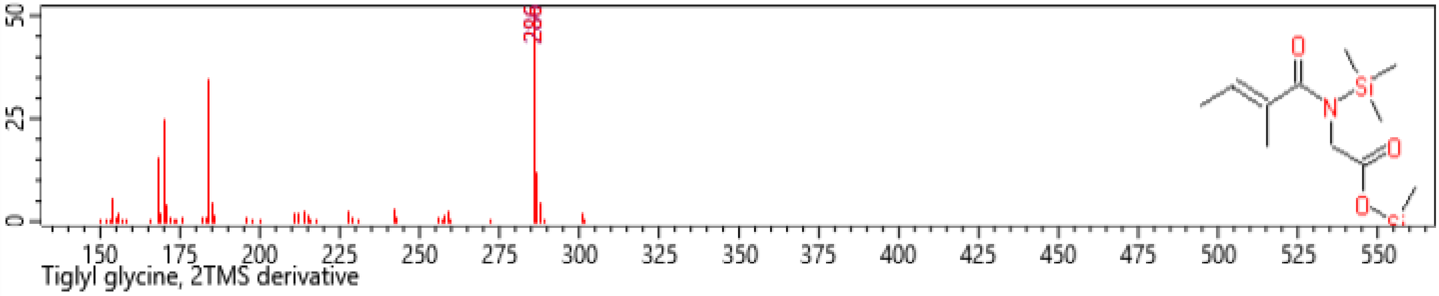


Tiglyl glycine, 2TMS derivative

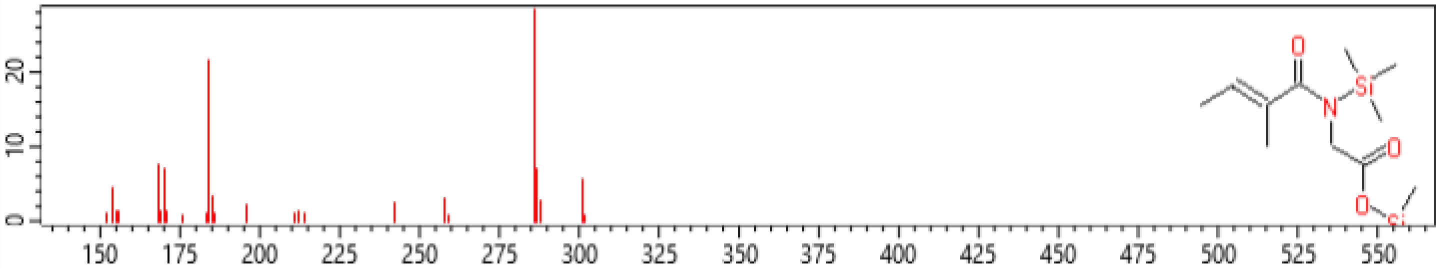

2-(4-Fluoro-phenyl)-4-nitro-isoindole-1,3-dione

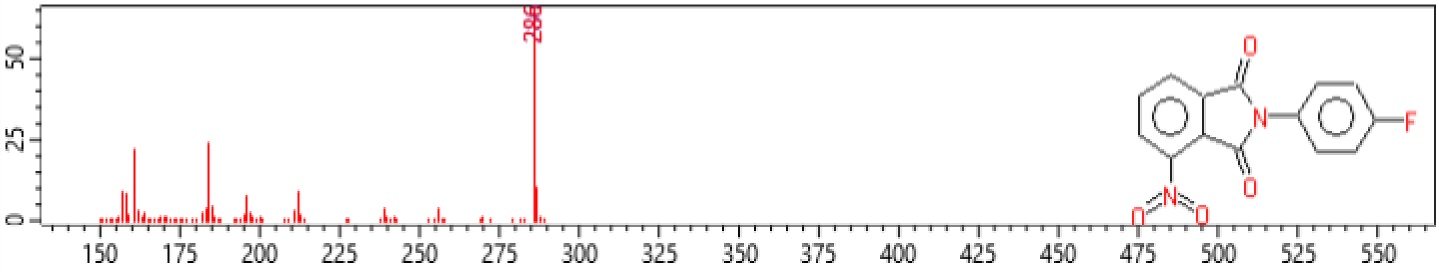


1-(Methanesulfonyl)piperidine-3-carbohydrazonic acid, 2 TMS derivative

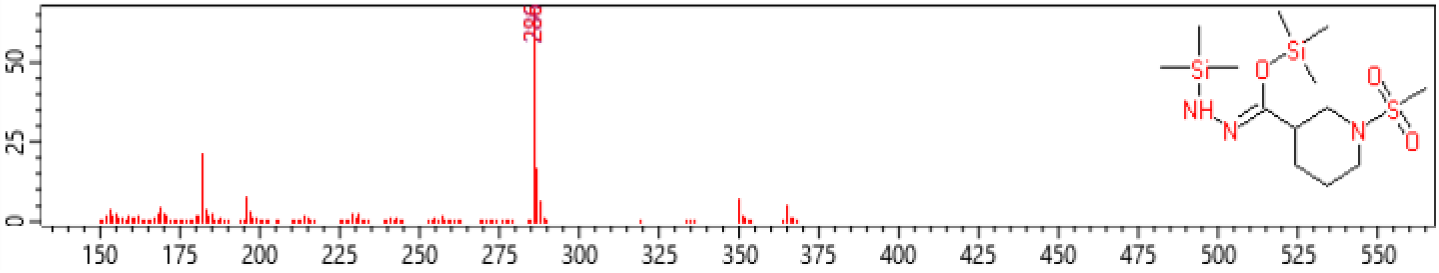


**2-Hydroxy-4-methoxybenzophenone**

Peak: 10.51 - 10.55 min (scan #980 - #990) Subtracted noise: 10.43 - 10.47 min (scan #958 - #968), 10.55 - 10.59 min (scan #991 - #

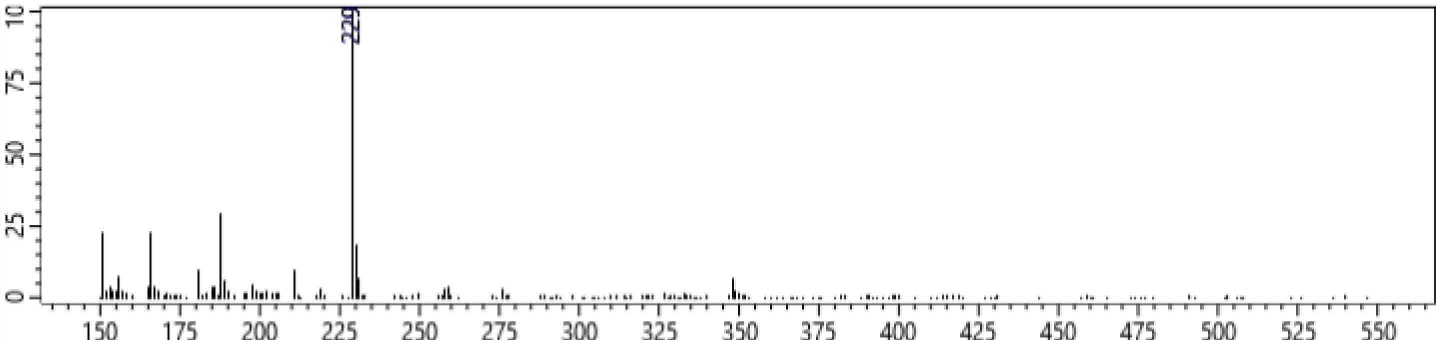

2-Hydroxy-4-methoxybenzophenone

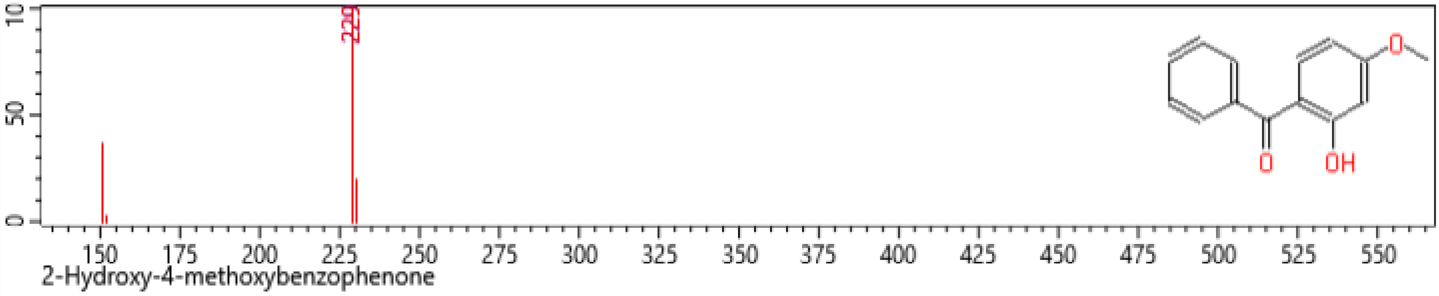


2-Hydroxy-4-methoxybenzophenone

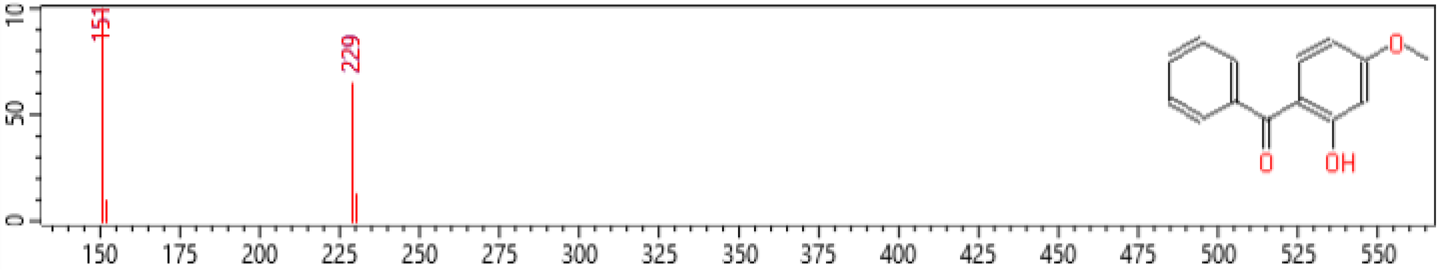


2-Hydroxy-4-methoxybenzophenone

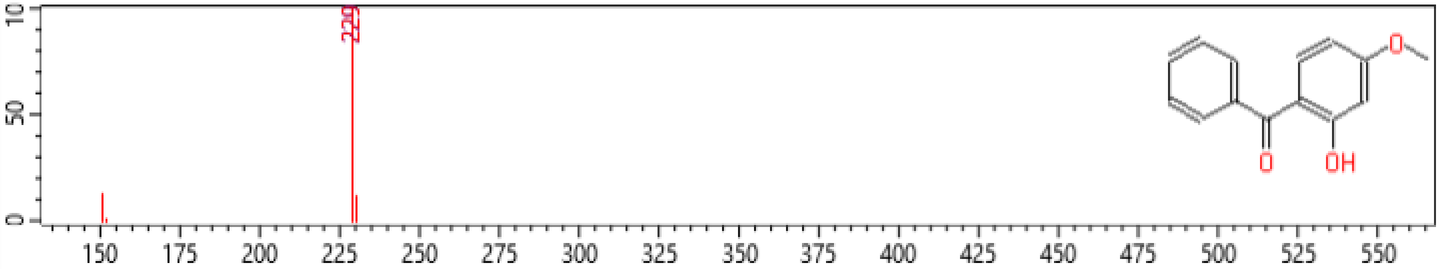


1-butanesulfonamide, N-(2-hydroxyphenyl)-

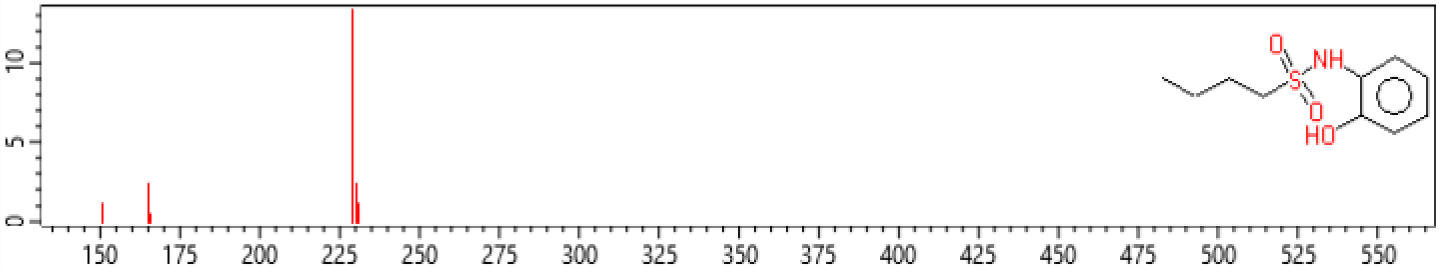


α-Hydroxy-α-phenyl-4-[2-fluoroethyl]phenylacetic acid,

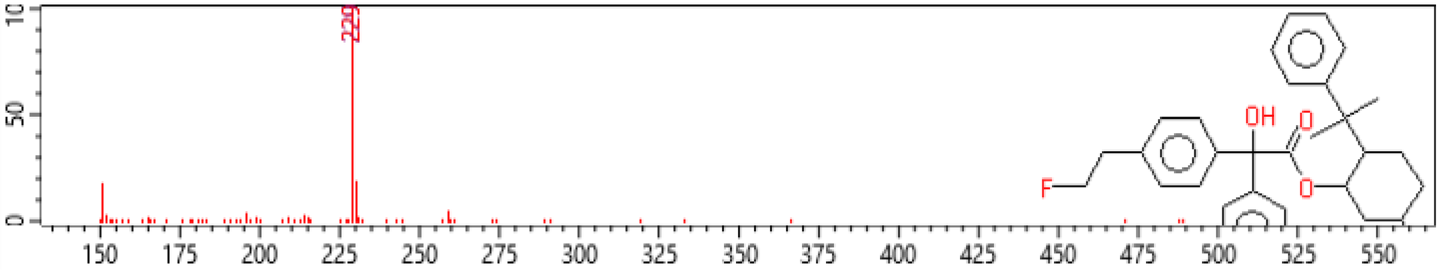

**Tetrasiloxane, decamethyl-**

Peak: 10.56 - 10.58 min (scan #994 - #998) Subtracted noise: 10.53 - 10.55 min (scan #986 - #990), 10.59 - 10.61 min (scan #1001 -

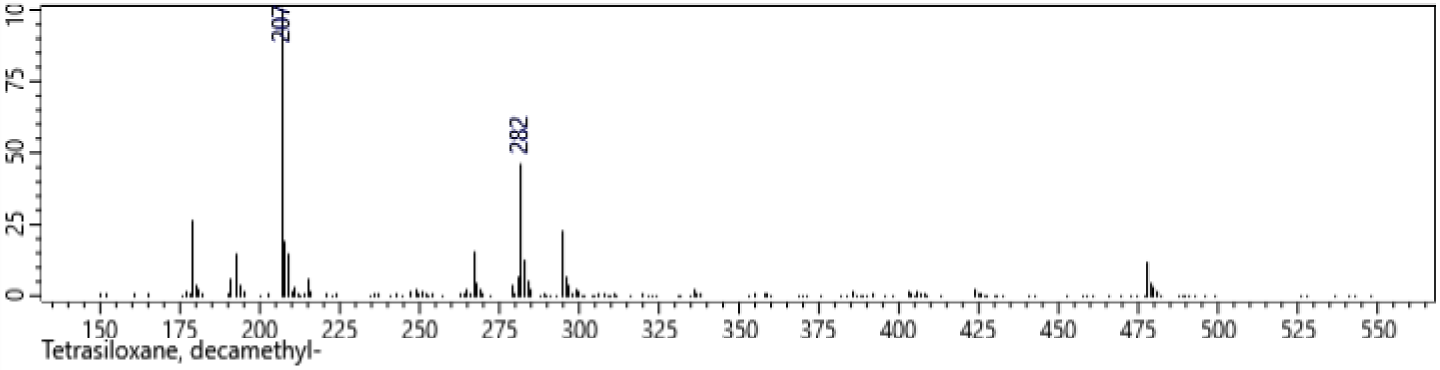


Tetrasiloxane, decamethyl-

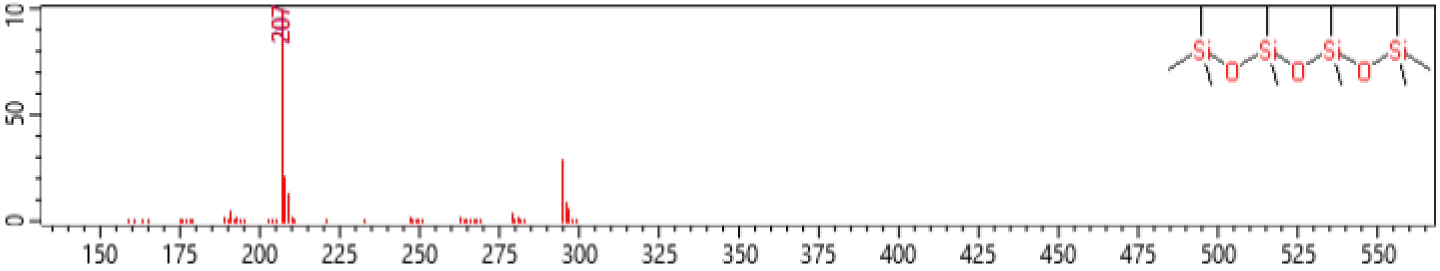


Tetrasiloxane, decamethyl-

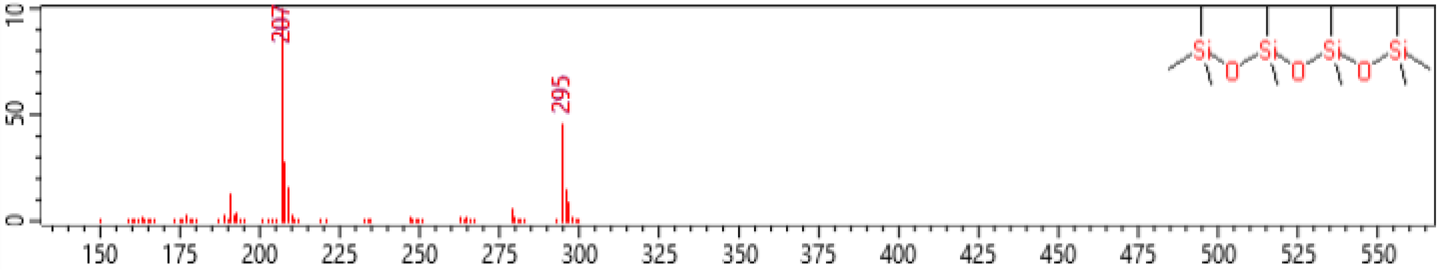


Ethyl homovanillate, TMS derivative

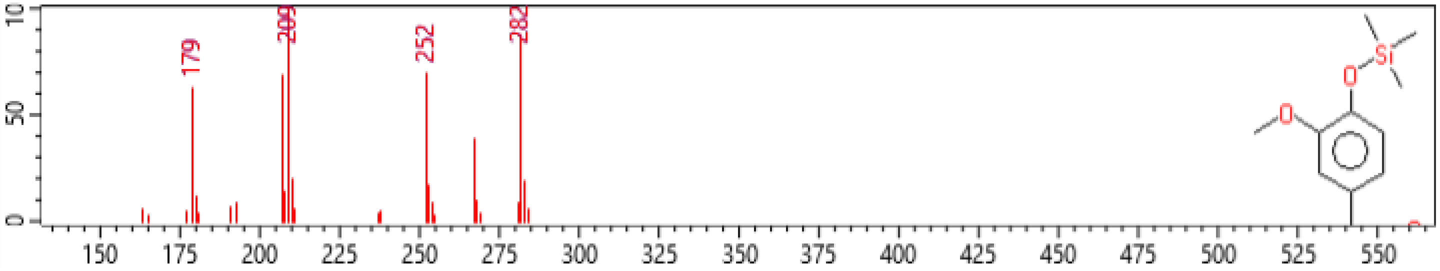


Methyltris(trimethylsiloxy)silane

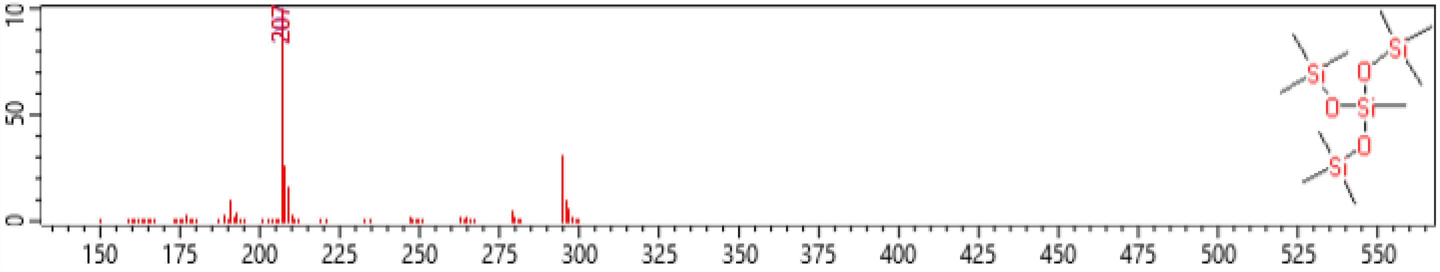

Methyltris(trimethylsiloxy)silane

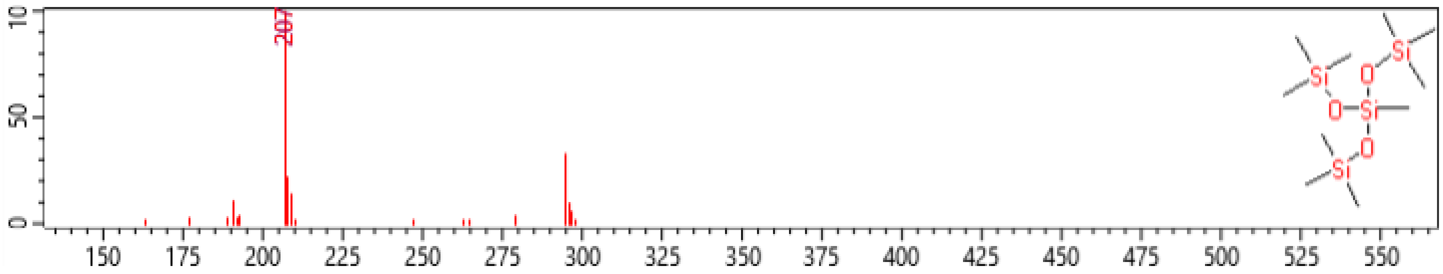


**5-(4-Morpholinyl)-1,3,4-thiadiazole-2-thiol, TMS**

Peak: 10.59 - 10.61 min (scan #1002 - #1006) Subtracted noise: 10.57 - 10.58 min (scan #997 - #1000), 10.63 - 10.64 min (scan #101

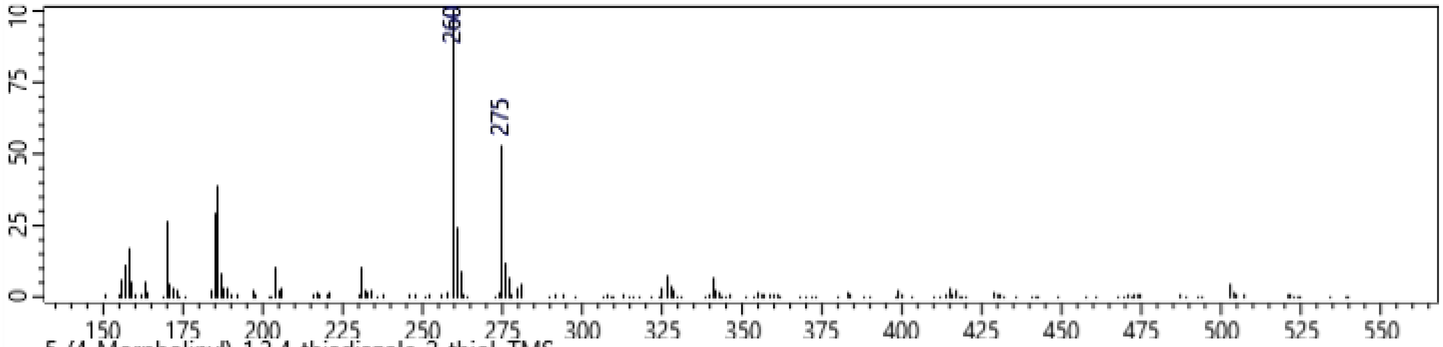


5-(4-Morpholinyl)-1,3,4-thiadiazole-2-thiol, TMS

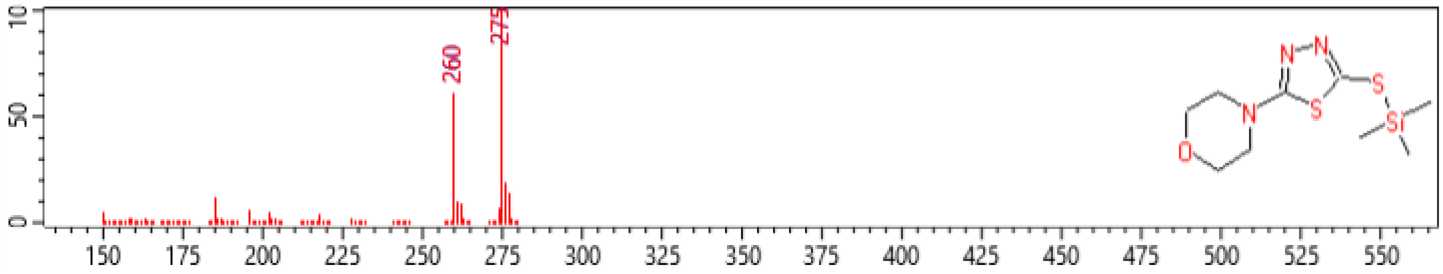


2-Ketovaleric acid ho-tms

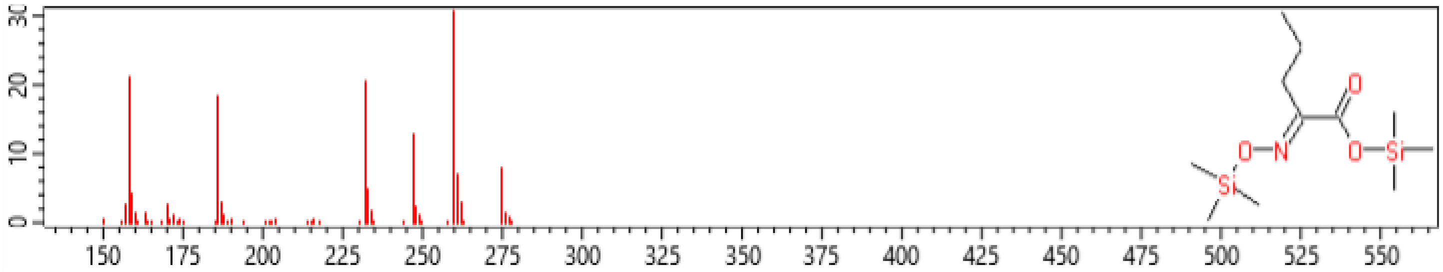

pentanoic acid, 2-methyl-2-[[(trimethylsilyl)oxy]methyl]-, trimethylsilyl ester

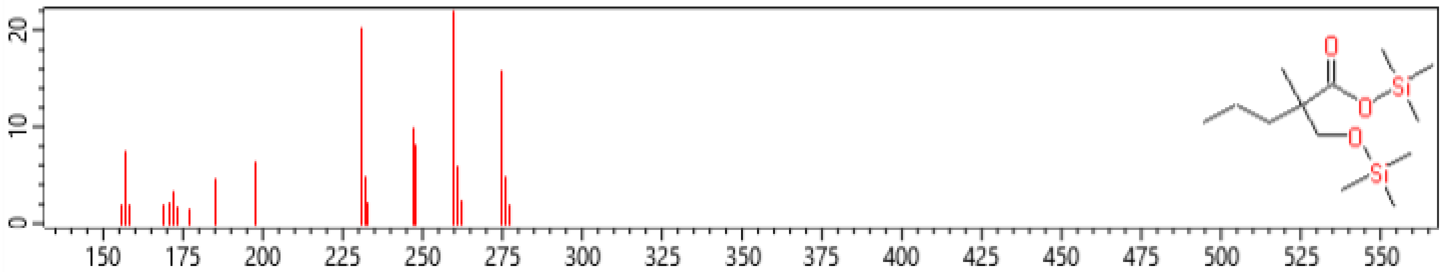

2-Ketoisovaleric acid oxime, bis(trimethylsilyl)- deriv.

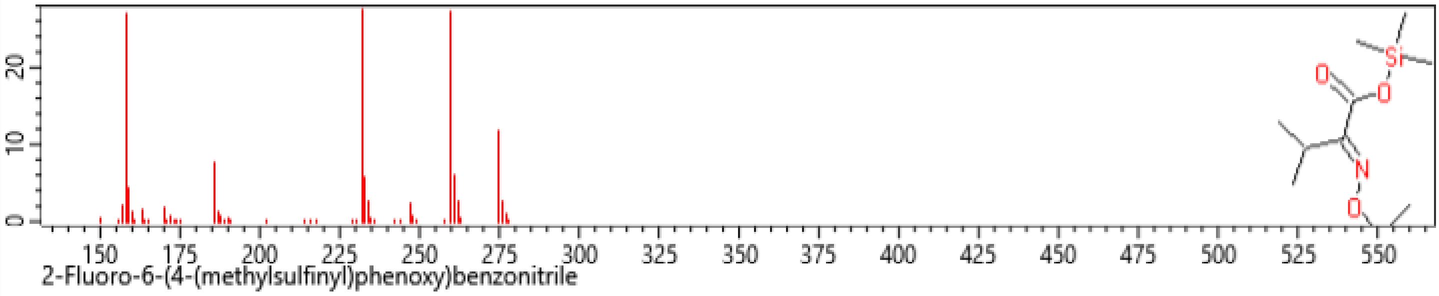

2-Fluoro-6-(4-(methylsulfinyl)phenoxy)benzonitrile

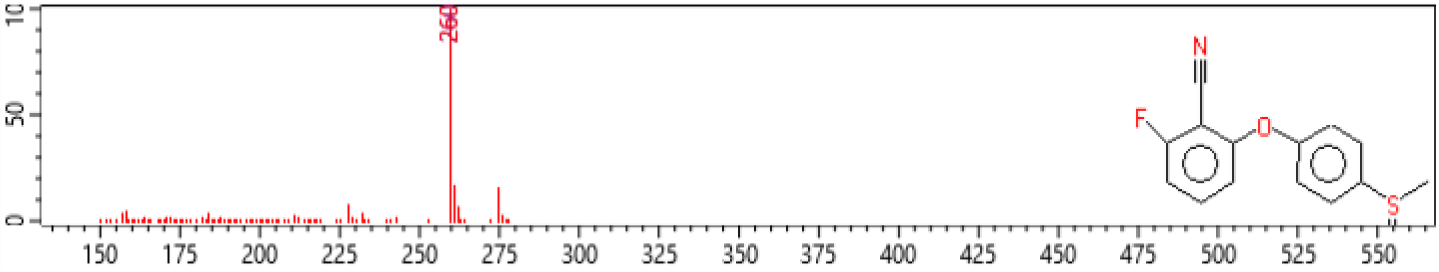


**Eugenol TMS**

Peak: 10.64 - 10.68 min (scan #1017 - #1027) Subtracted noise: 10.58 - 10.62 min (scan #1000 - #1011), 10.70 - 10.74 min (scan #10

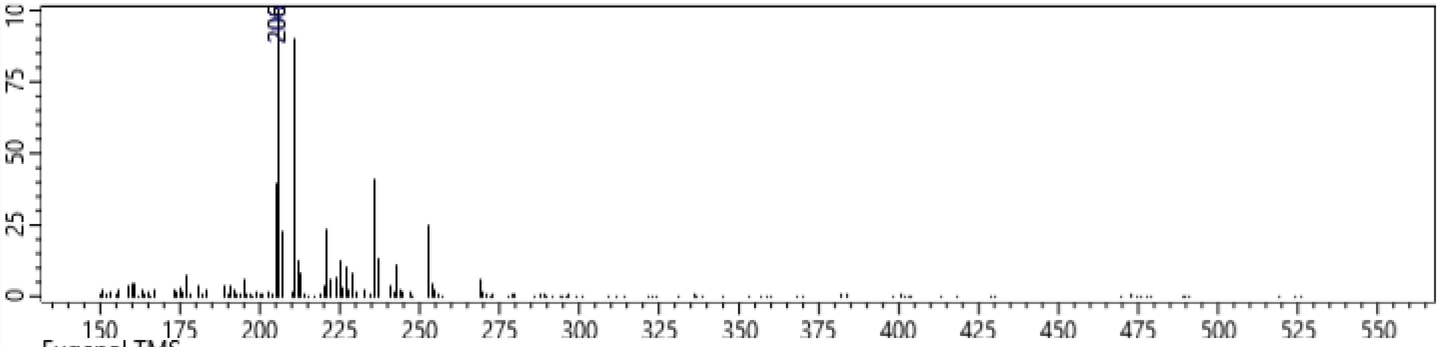


Eugenol TMS

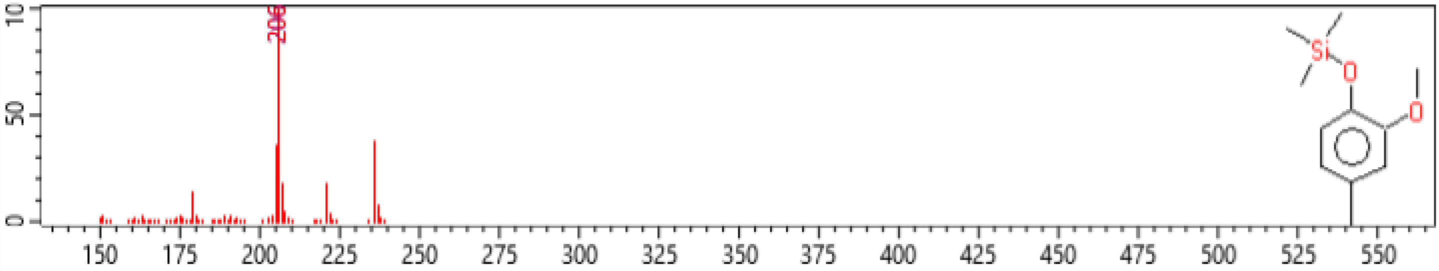


Isoeugenol, TMS derivative

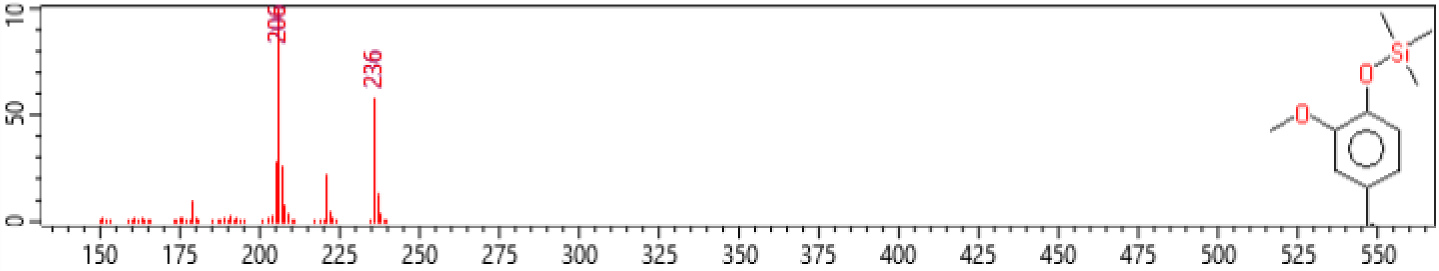

Isoeugenol, TMS derivative

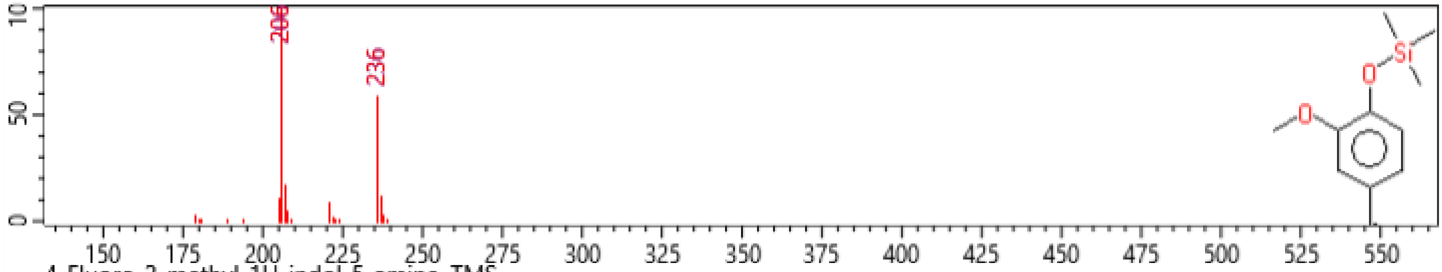


4-Fluoro-2-methyl-1H-indol-5-amine, TMS

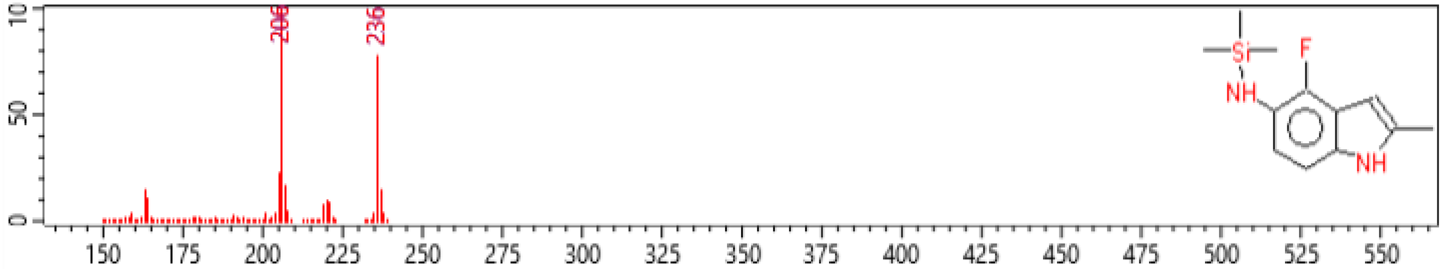


Thiethylperazine

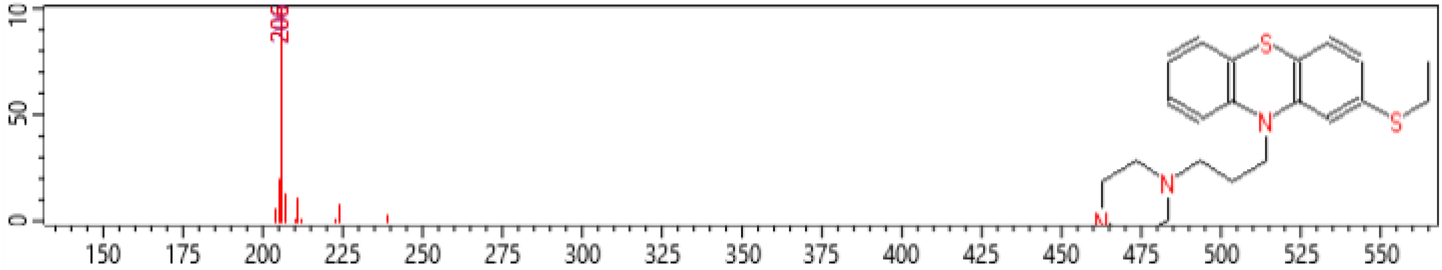


**Cycloheptasiloxane, tetradecamethyl-**

Peak: 10.72 - 10.74 min (scan #1037 - #1043) Subtracted noise: 10.68 - 10.70 min (scan #1026 - #1032), 10.76 - 10.78 min (scan #10[illegible]

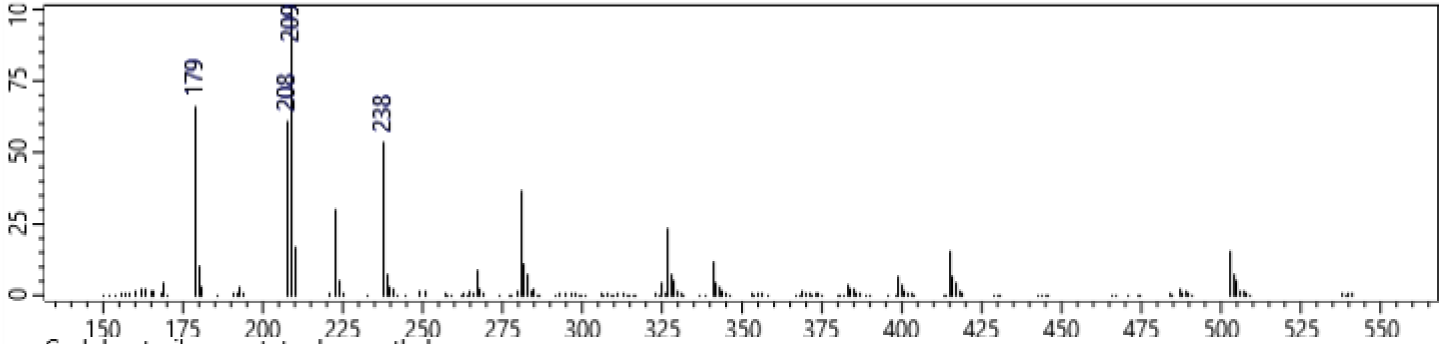


Cycloheptasiloxane, tetradecamethyl-

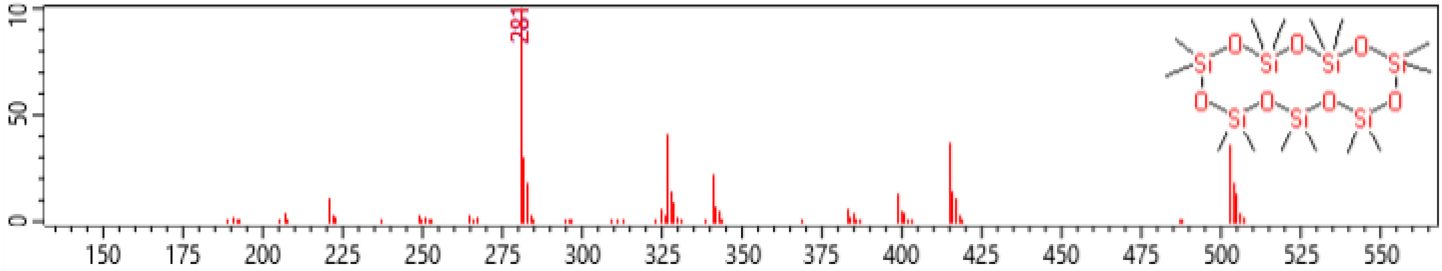

Cycloheptasiloxane, tetradecamethyl-

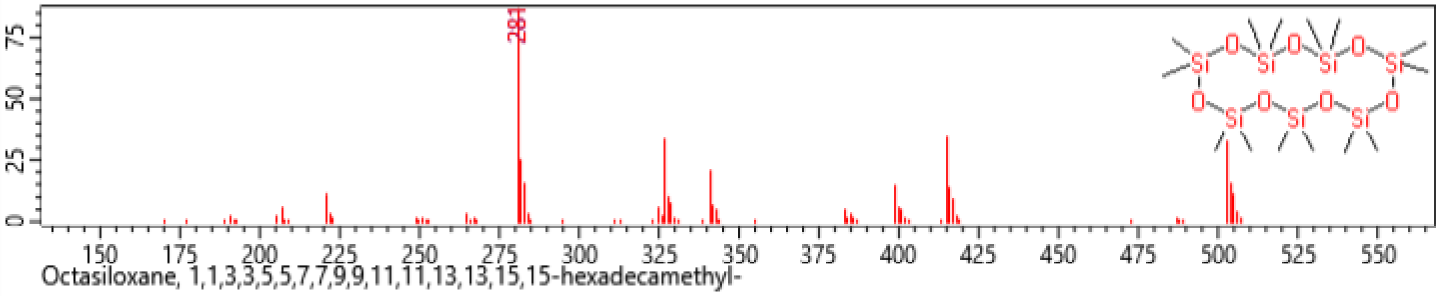


Octasiloxane, 1,1,3,3,5,5,7,7,9,9,11,11,13,13,15,15-hexadecamethyl-

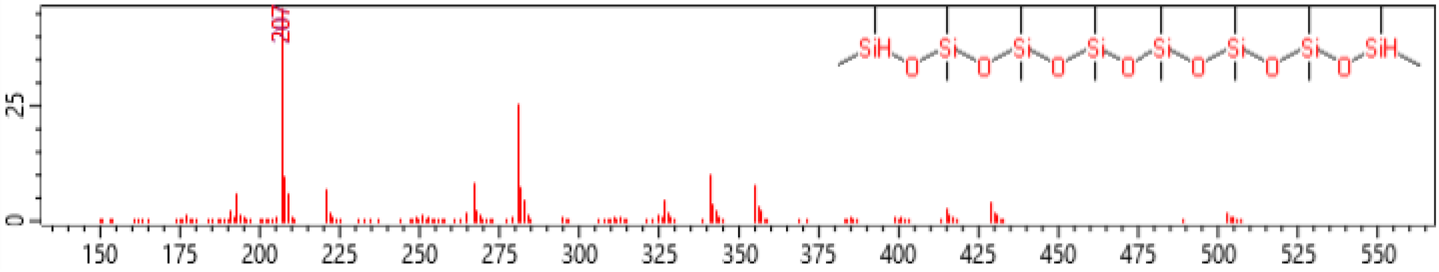


Cyclodecasiloxane, eicosamethyl-

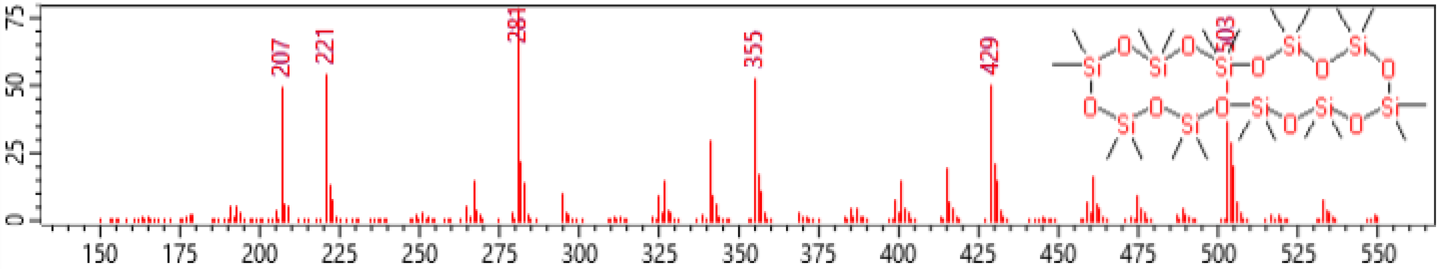


1,15-Di(1-phenylpropyl)-2,2,4,4,6,6,8,8,10,10,12,12,14,14-tetradecamethyl-1,3,5,7,9,11,13,15-octaoxa-2,4,6,8,10,12,14-heptasilapentac

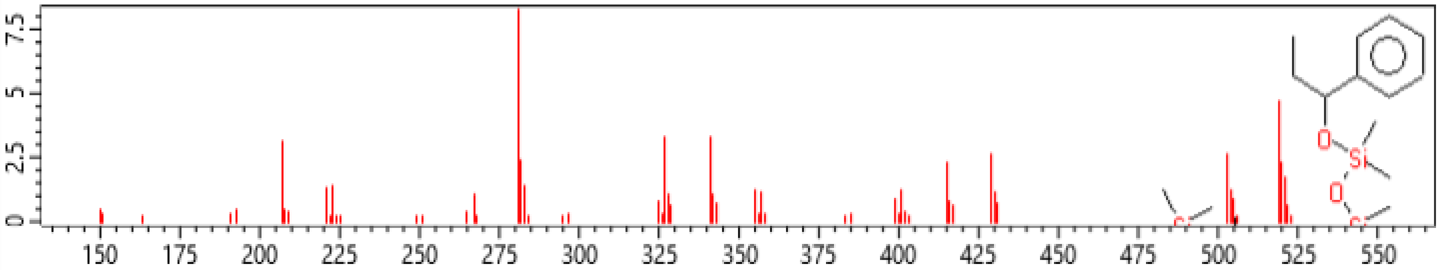

**1,2,3,4,5,6,7,8-Octahydrophenanthrene-3-carboxylic acid amide**

Peak: 10.79 - 10.81 min (scan #1058 - #1062) Subtracted noise: 10.77 - 10.78 min (scan #1051 - #1055), 10.82 - 10.83 min (scan #10

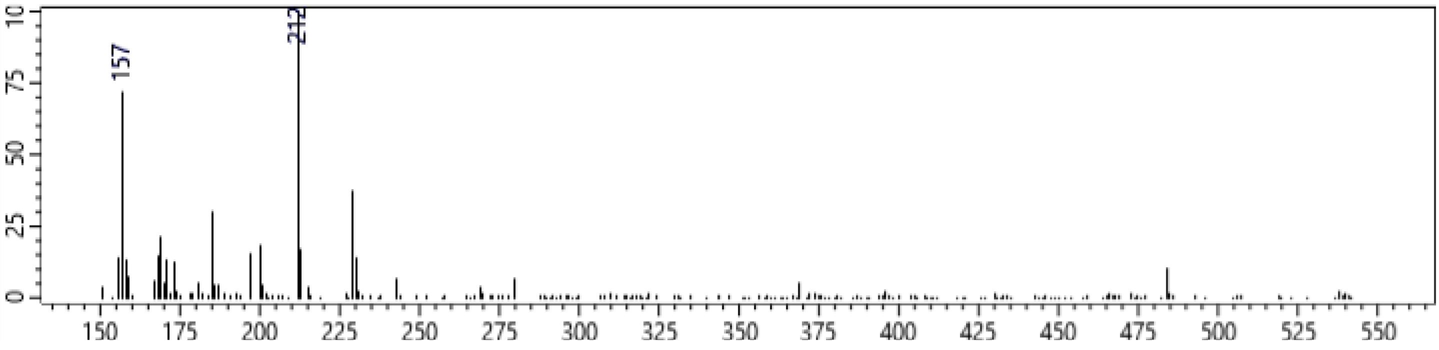

1,2,3,4,5,6,7,8-Octahydrophenanthrene-3-carboxylic acid amide

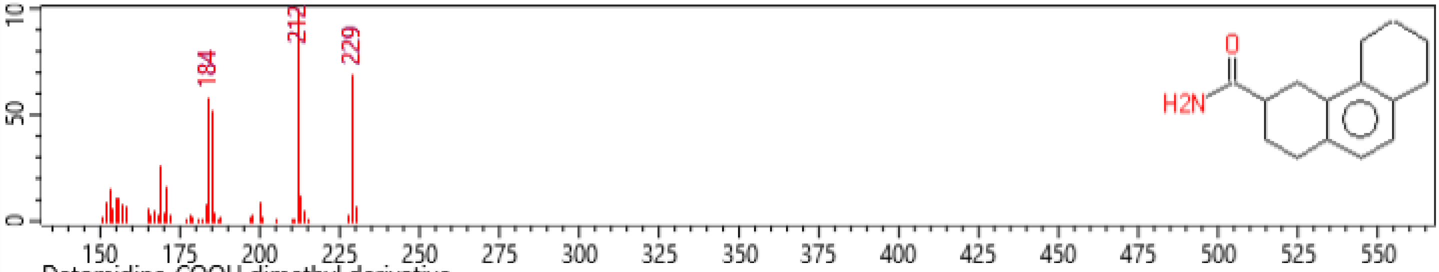


Detomidine-COOH dimethyl derivative

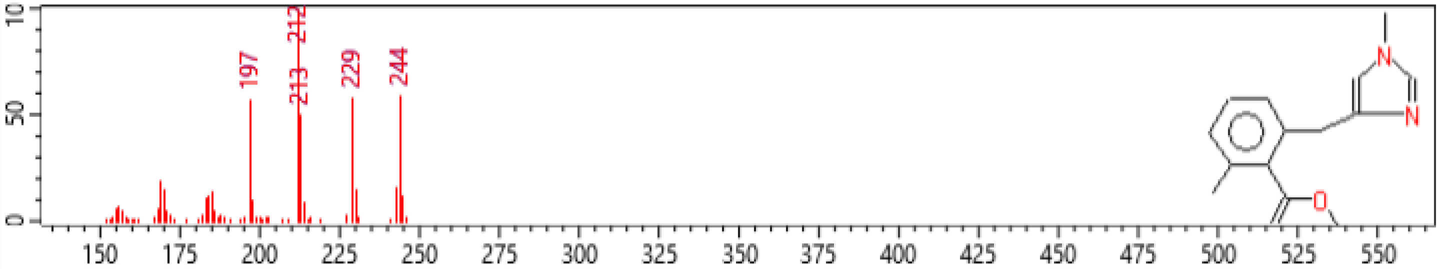


Minaprine

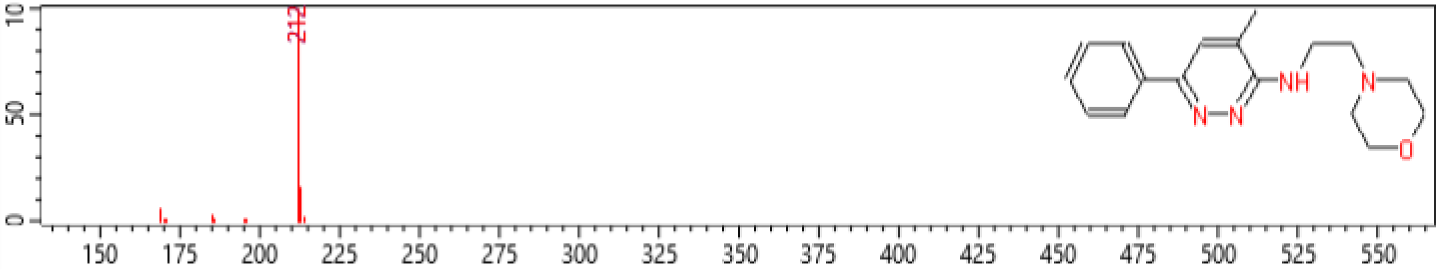


2-(3,3,6,7-Tetramethyl-3,4-dihydro-2H-isoquinolin-1-ylidene)-acetamide

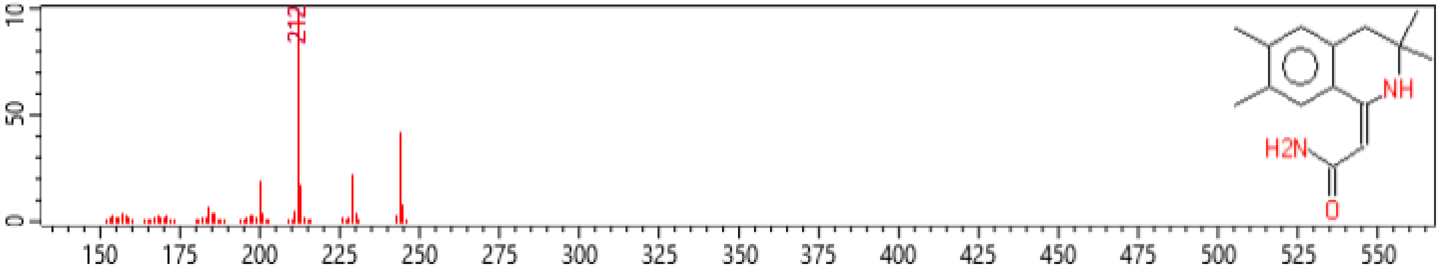


Benzeneethanamine, 2-fluoro-β-hydroxy-4,5-methoxy-α-methyl-

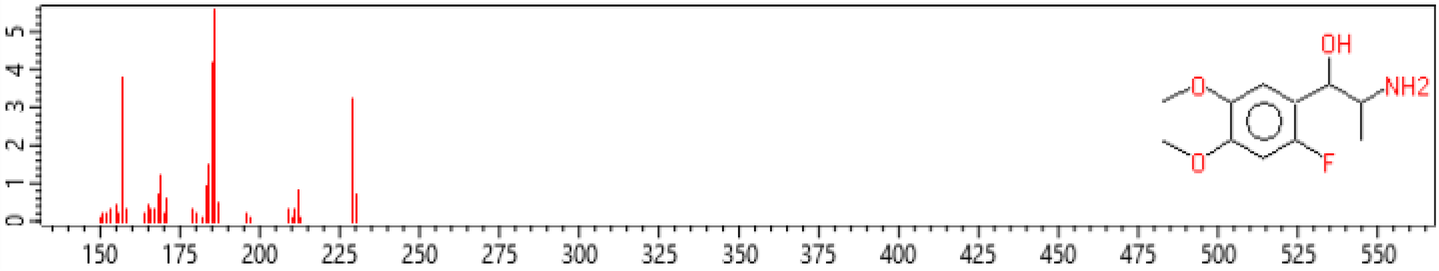

**Pentasiloxane, dodecamethyl-**

Peak: 10.83 - 10.85 min (scan #1069 - #1073) Subtracted noise: 10.80 - 10.82 min (scan #1060 - #1065), 10.86 - 10.87 min (scan #10[illegible]

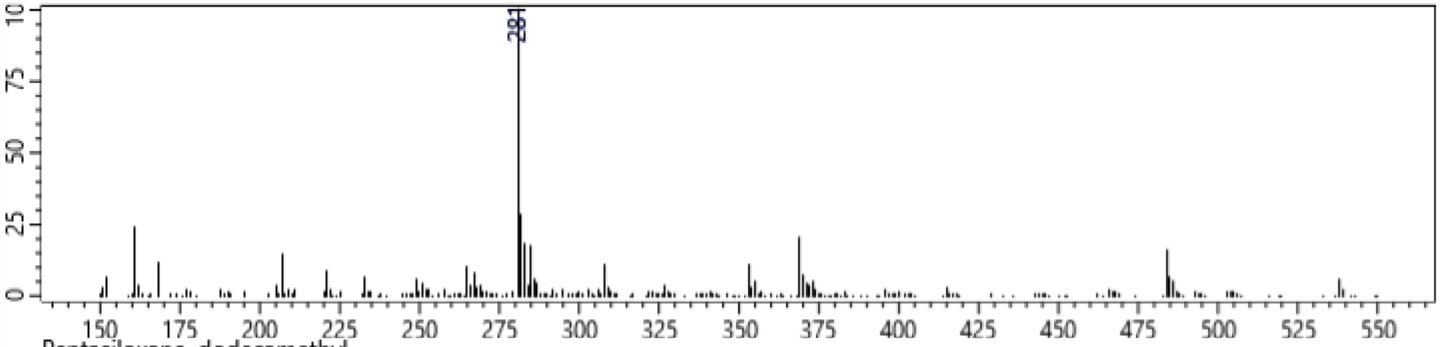


Pentasiloxane, dodecamethyl-

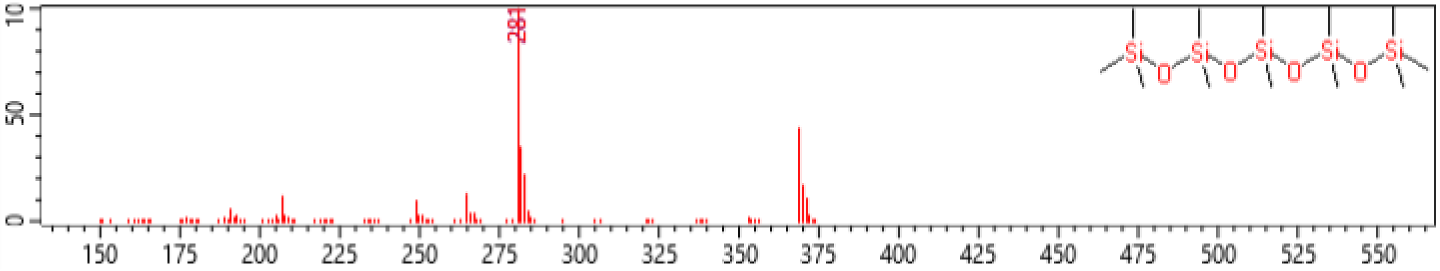


Pentasiloxane, dodecamethyl-

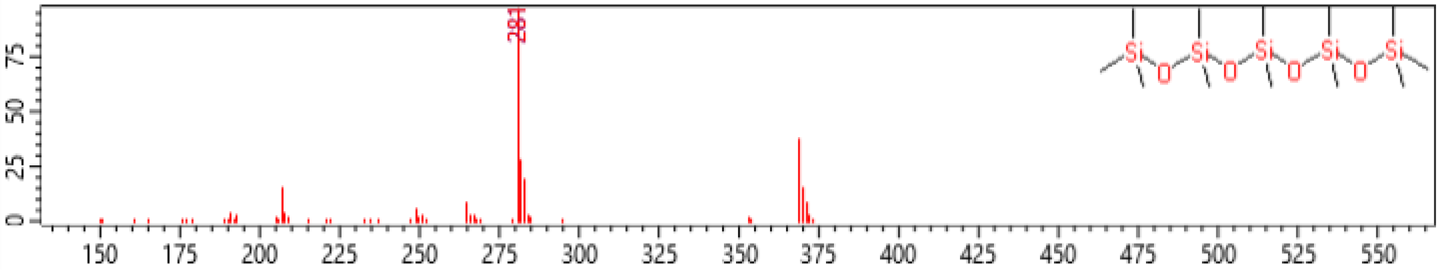


Pentasiloxane, dodecamethyl-

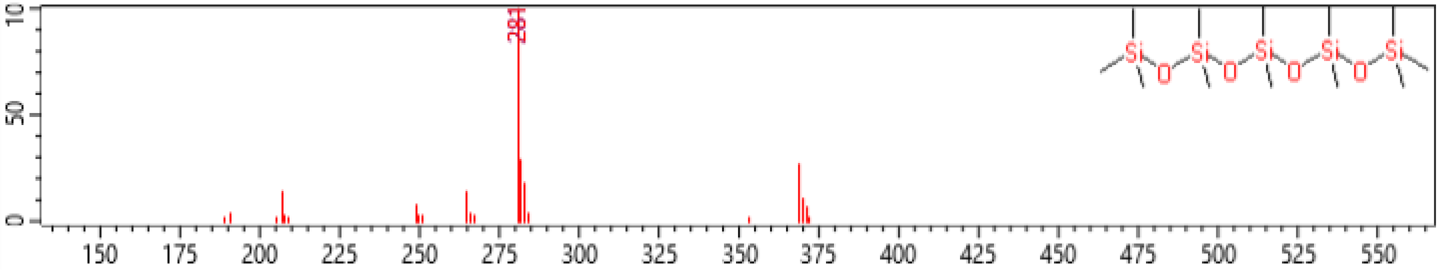


Pentasiloxane, dodecamethyl-

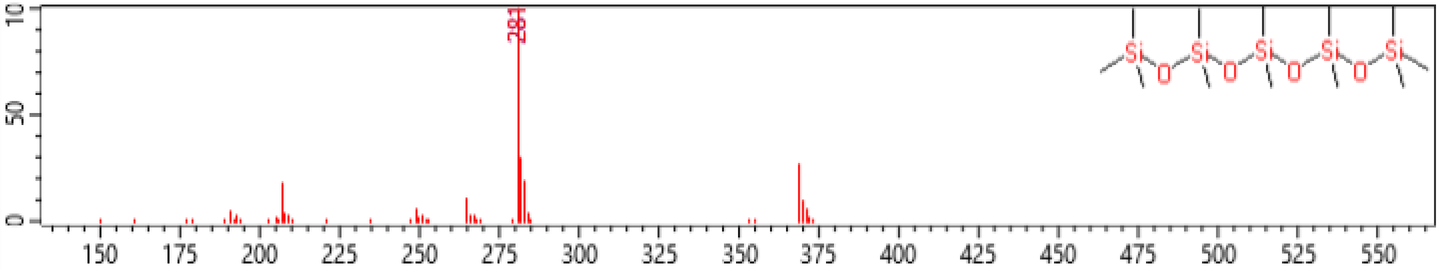

Trisiloxane, 1,1,1,5,5,5-hexamethyl-3,3-bis[(trimethylsilyl)oxy]-

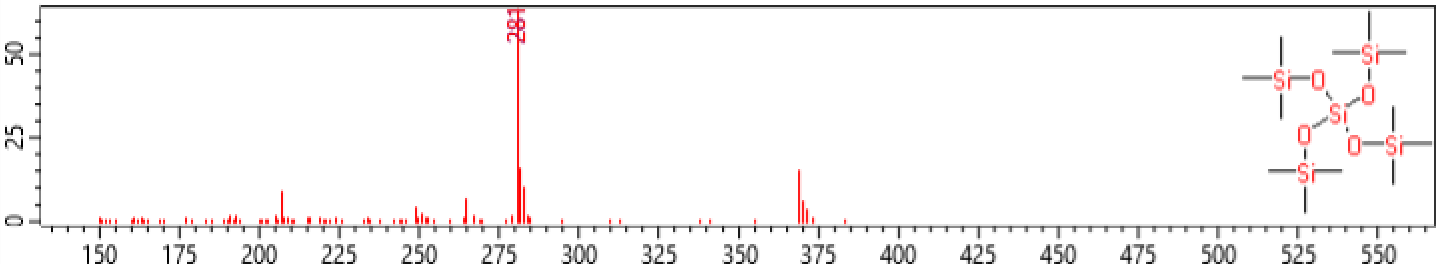


**Heptasiloxane, hexadecamethyl-**

Peak: 10.90 - 10.92 min (scan #1089 - #1095) Subtracted noise: 10.86 - 10.88 min (scan #1078 - #1084), 10.95 - 10.97 min (scan #11

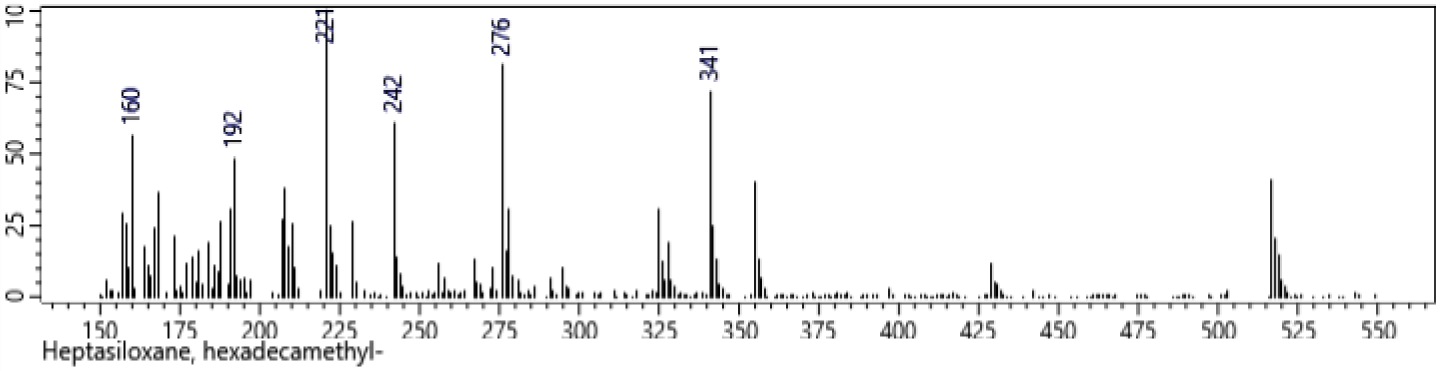


Heptasiloxane, hexadecamethyl-

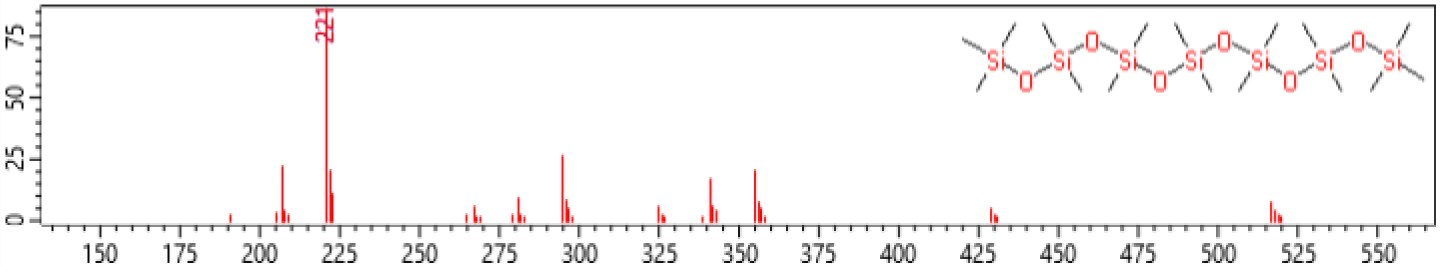


Heptasiloxane, hexadecamethyl-

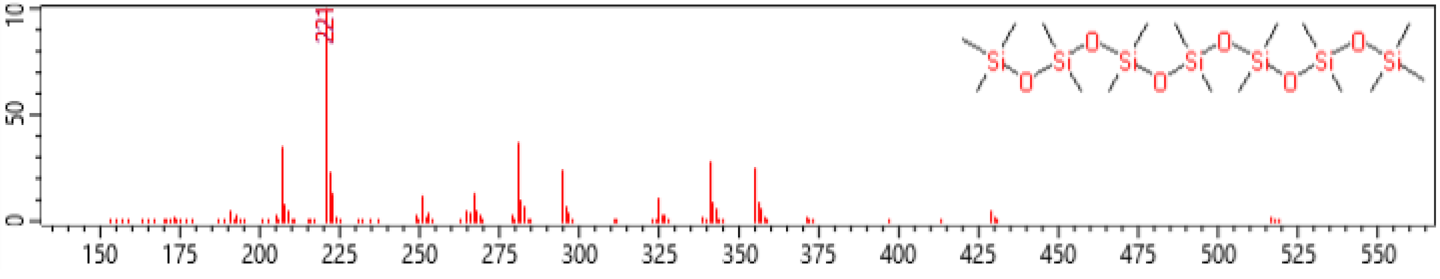


Thymopoietin II (32-35)

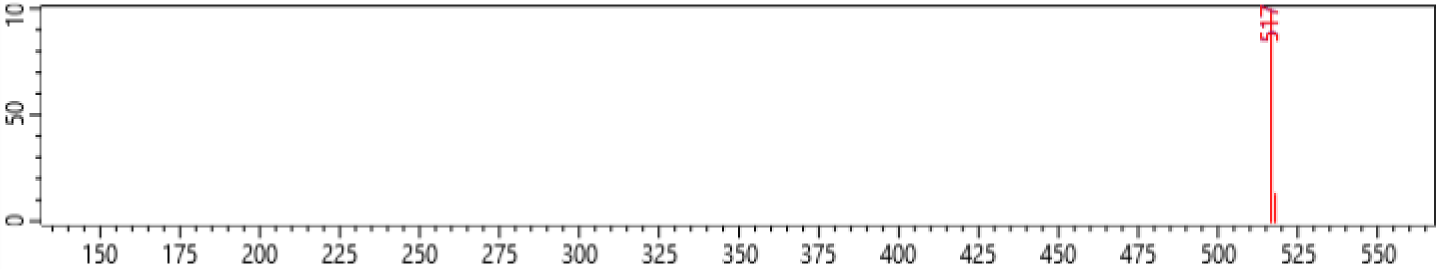

Thymopoietin II (32-35)

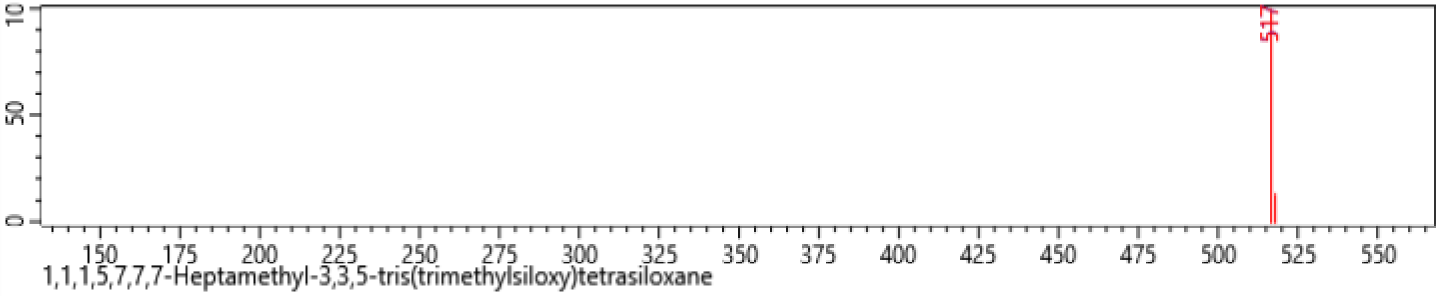


1,1,1,5,7,7,7-Heptamethyl-3,3,5-tris(trimethylsiloxy)tetrasiloxane

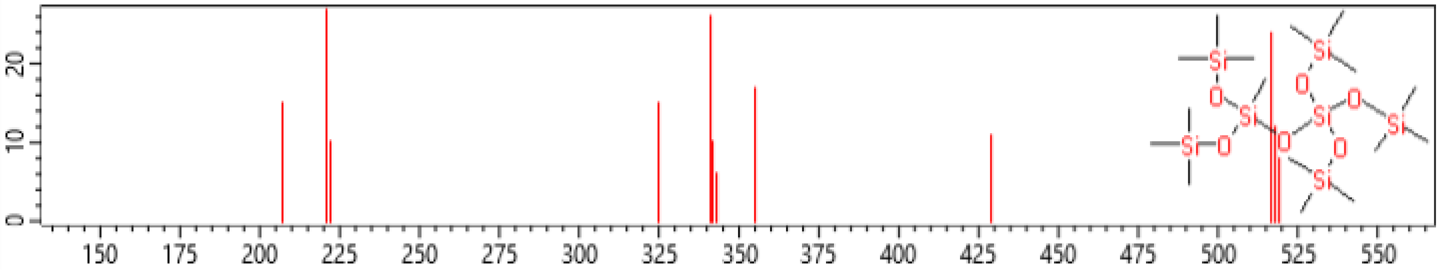

**Proadrenomedullin N-terminal 20 Peptide (9-20), human**

Peak: 11.05 - 11.06 min (scan #1129 - #1133) Subtracted noise: 11.02 - 11.04 min (scan #1121 - #1126), 11.08 - 11.09 min (scan #11[illegible]

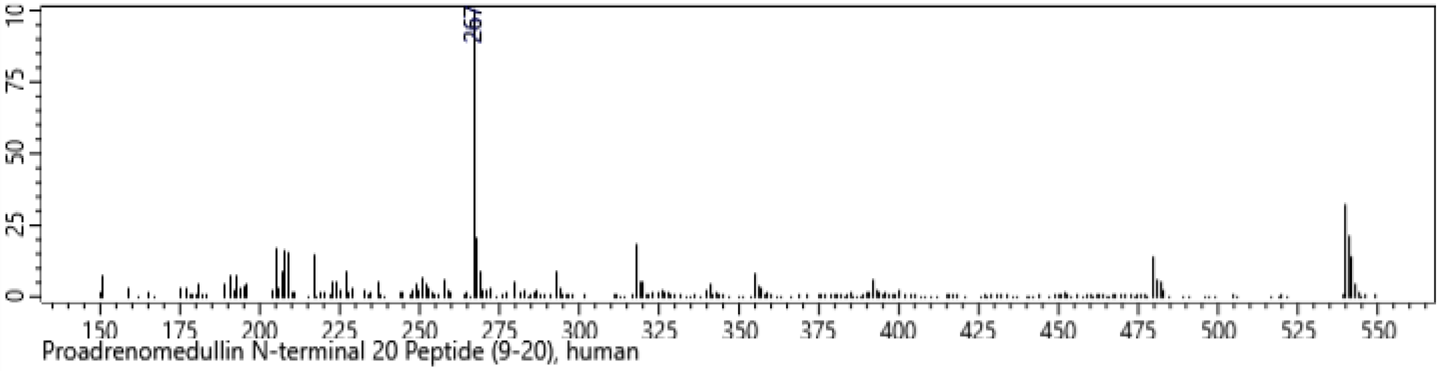


Proadrenomedullin N-terminal 20 Peptide (9-20), human

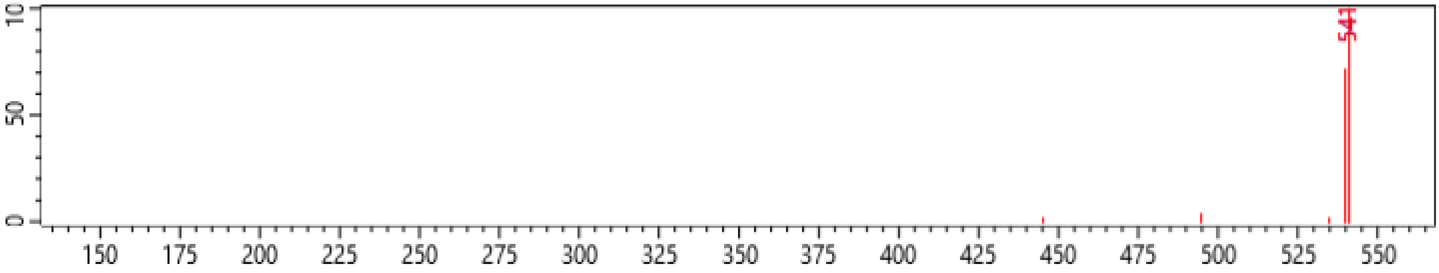


Tibolone, bis(tert.-butyldimethylsilyl) derivative

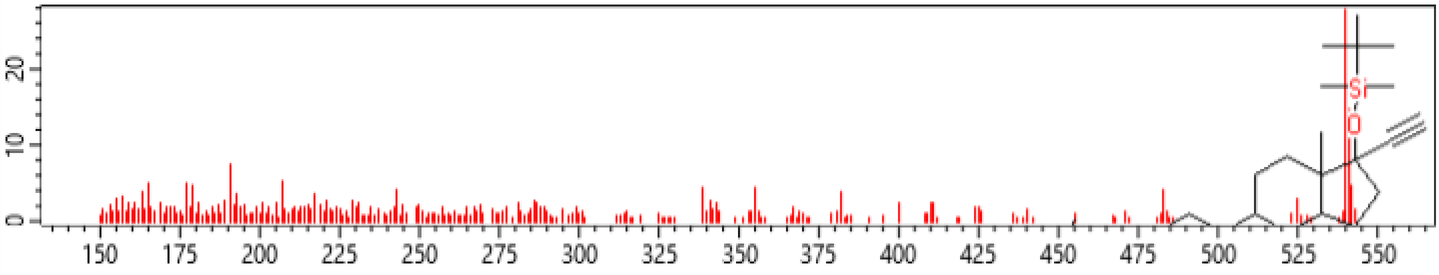

LeaLeX

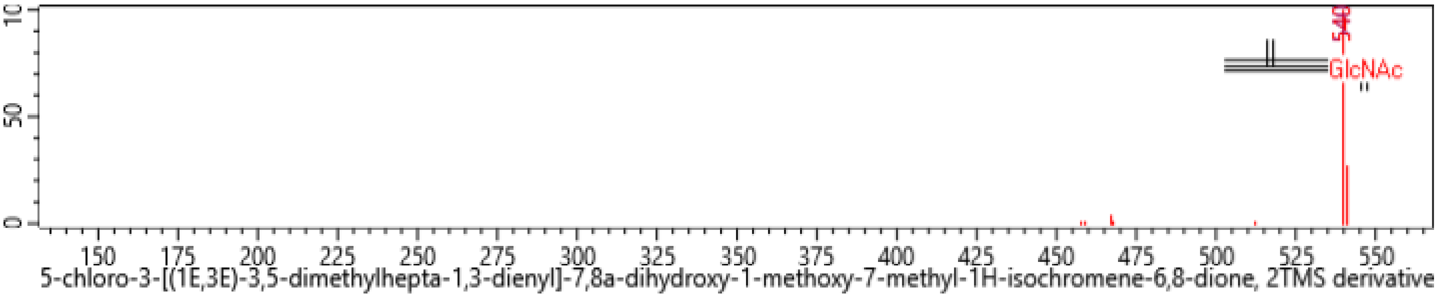


5-chloro-3-[(1E,3E)-3,5-dimethylhepta-1,3-dienyl]-7,8a-dihydroxy-1-methoxy-7-methyl-1H-isochromene-6,8-dione, 2TMS derivative

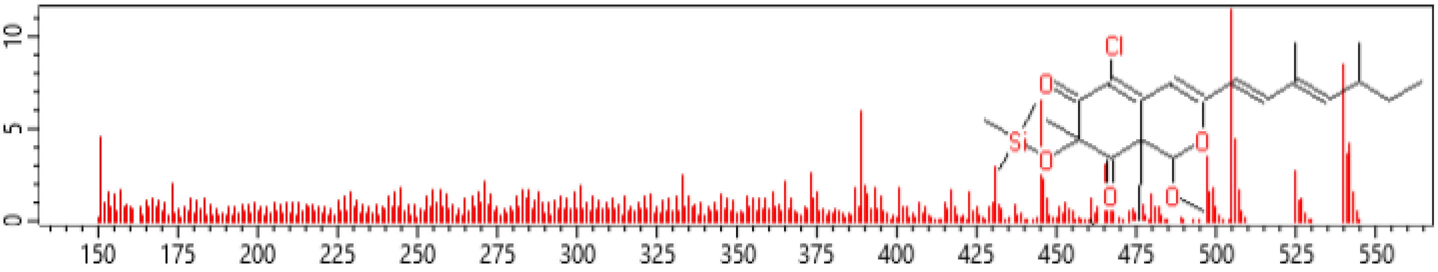


Cholest-3-eno[3,4-b]quinoxaline, 6',7'-dichloro-

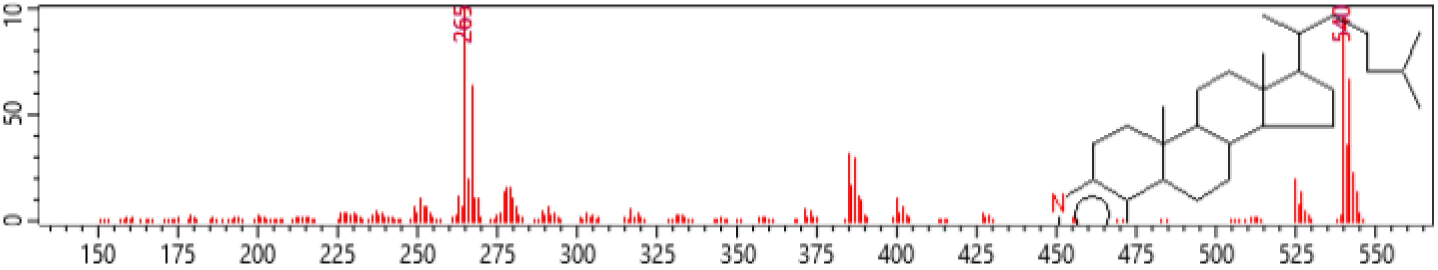


**Tromethamine, 4TMS derivative**

Peak: 11.08 - 11.10 min (scan #1139 - #1145) Subtracted noise: 11.05 - 11.07 min (scan #1130 - #1137), 11.13 - 11.15 min (scan #11

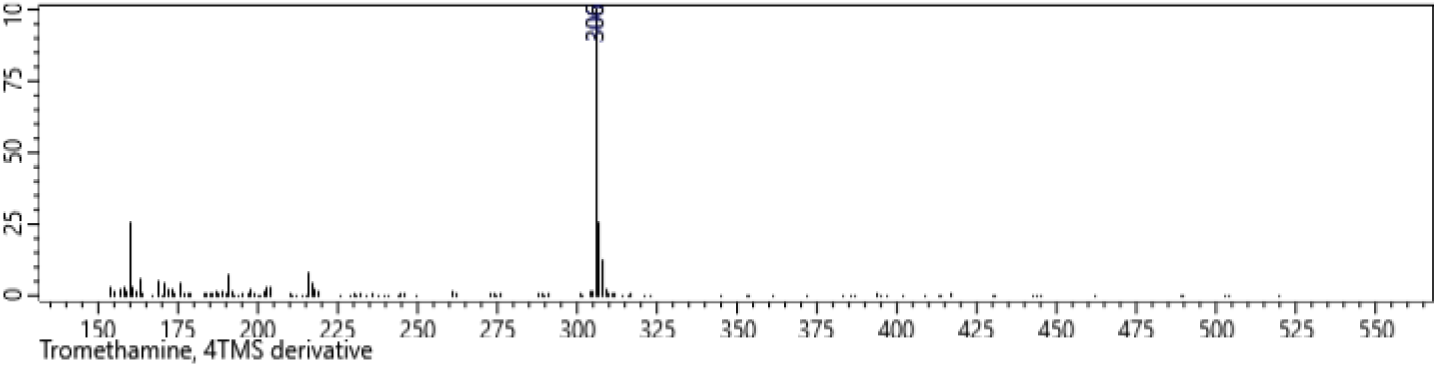


Tromethamine, 4TMS derivative

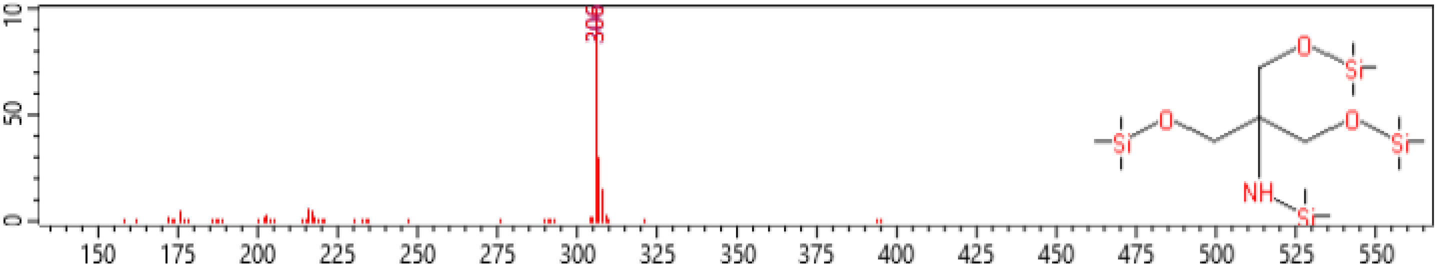

Tromethamine, 4TMS derivative

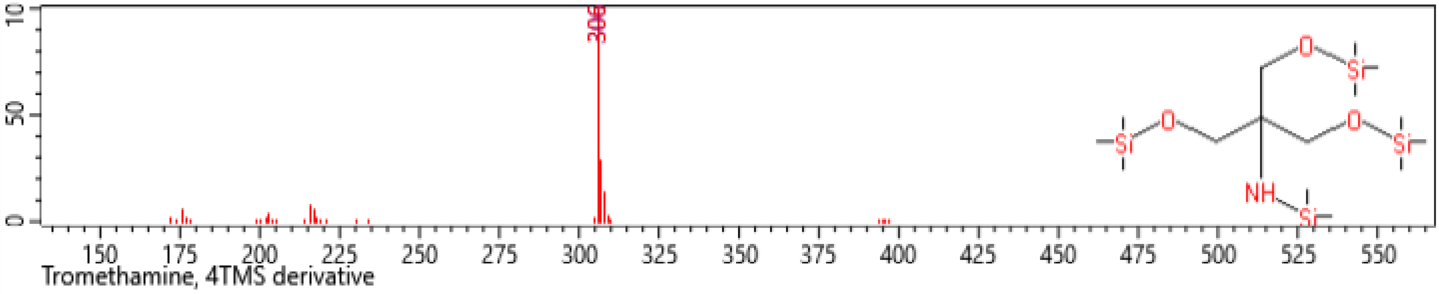


Tromethamine, 4TMS derivative

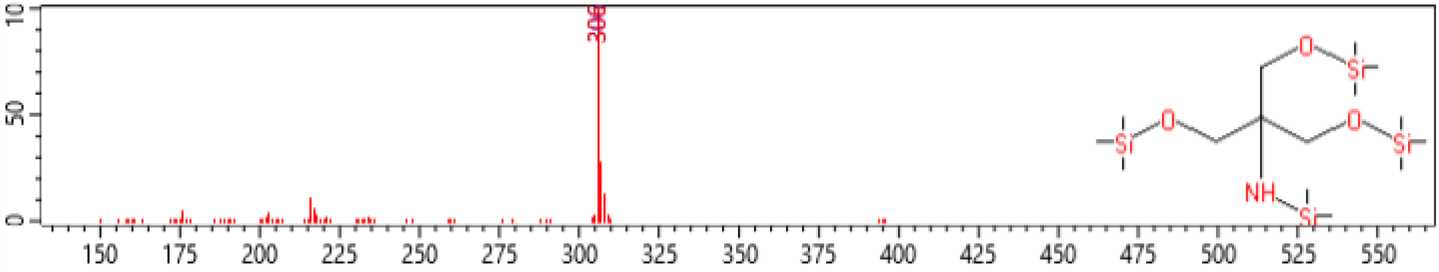


(3S)-3-Benzyloxycarbonylamino-1-chloro-4-(p-fluorophenyl)-2-butanone

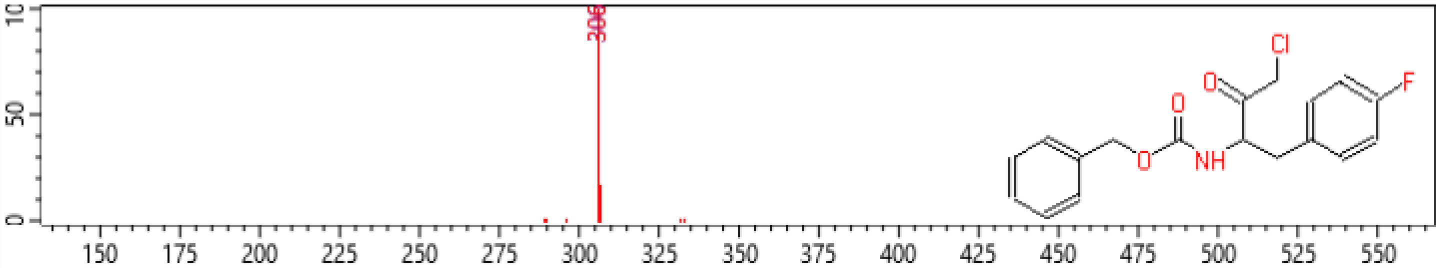


Cyclopropanecarboxamide, N-[3-[[6-[[3-(trifluoromethyl)phenyl]amino]-4-pyrimidinyl]amino]phenyl]-

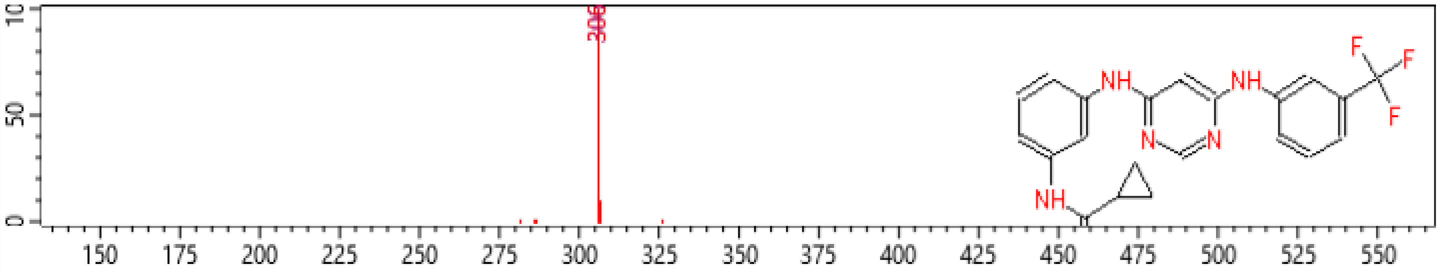


**Heptasiloxane, hexadecamethyl-**

Peak: 11.15 - 11.18 min (scan #1159 - #1167) Subtracted noise: 11.10 - 11.13 min (scan #1145 - #1152), 11.20 - 11.22 min (scan #11

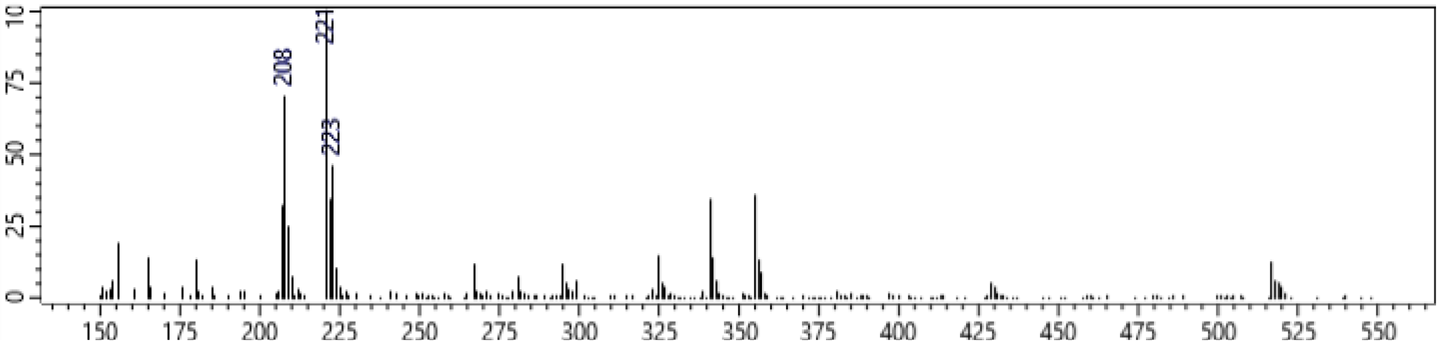

Heptasiloxane, hexadecamethyl-

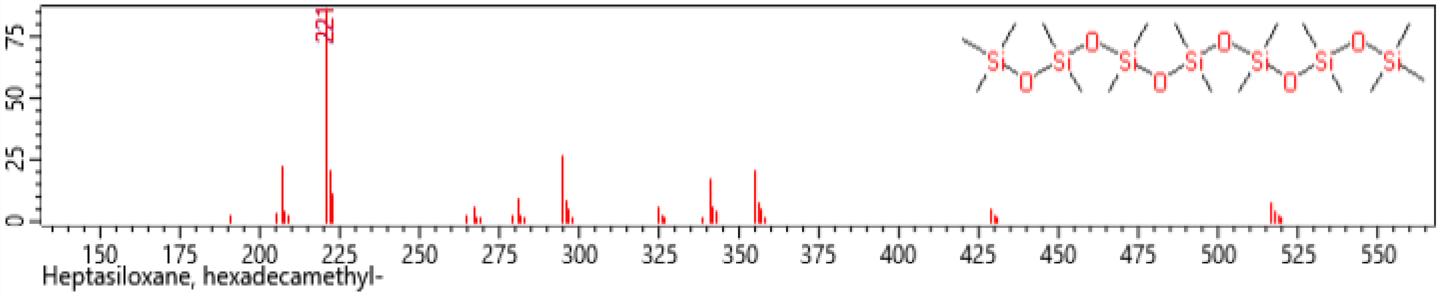


Heptasiloxane, hexadecamethyl-

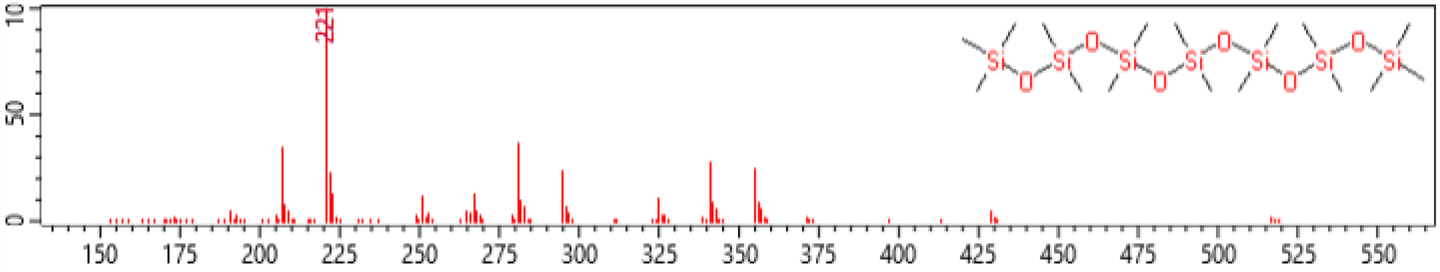


Heptasiloxane, 1,1,3,3,5,5,7,7,9,9,11,11,13,13-tetradecamethyl-

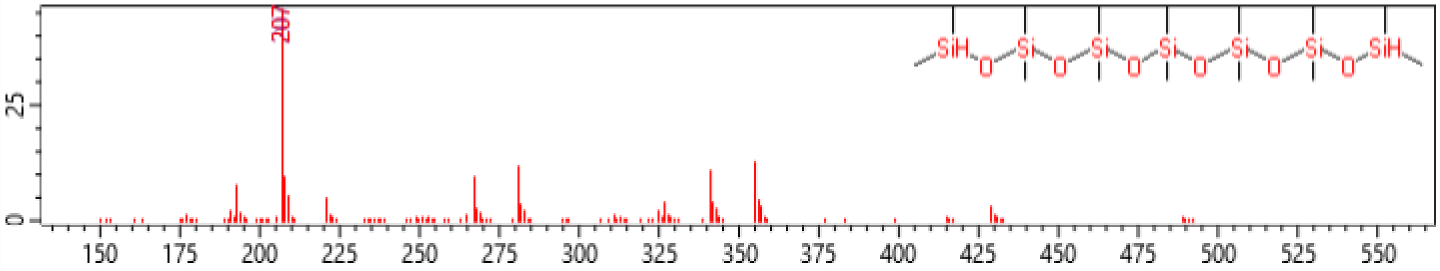


1,1,1,3,3,5,5,7,7,9,9,11,11,13,13,15,15,15-octadecamethyloctasiloxane

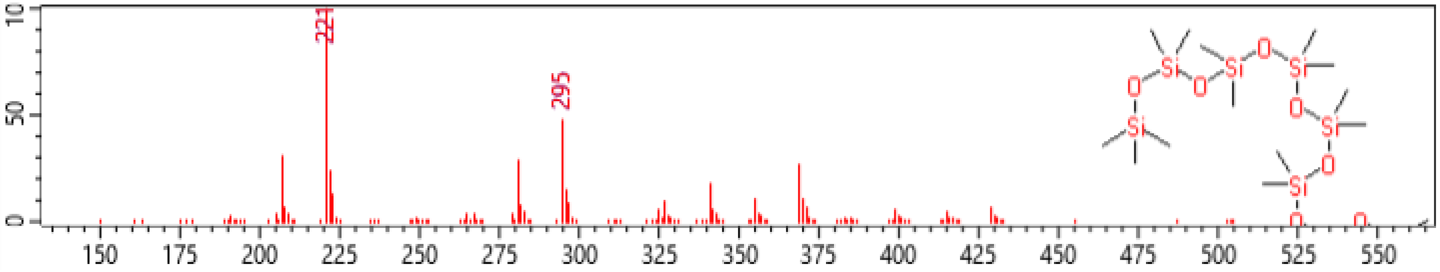


Octasiloxane, 1,1,3,3,5,5,7,7,9,9,11,11,13,13,15,15-hexadecamethyl-

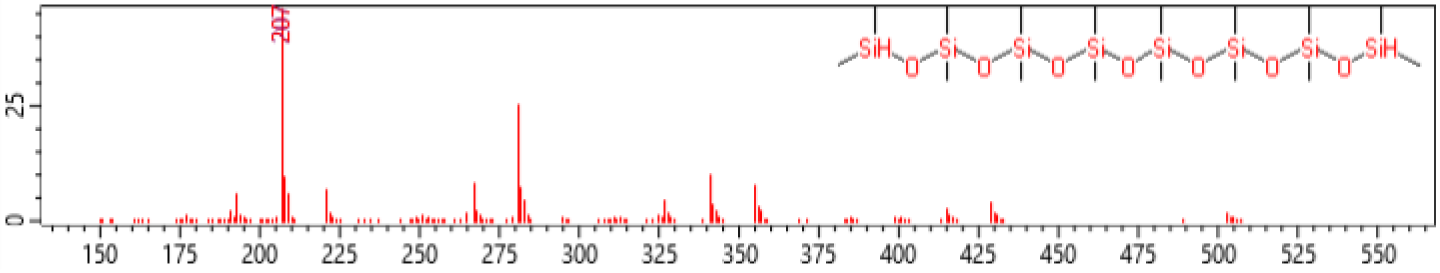

**Vanillin, TMS derivative**

Peak: 11.29 - 11.31 min (scan #1197 - #1203) Subtracted noise: 11.25 - 11.27 min (scan #1187 - #1192), 11.32 - 11.34 min (scan #12

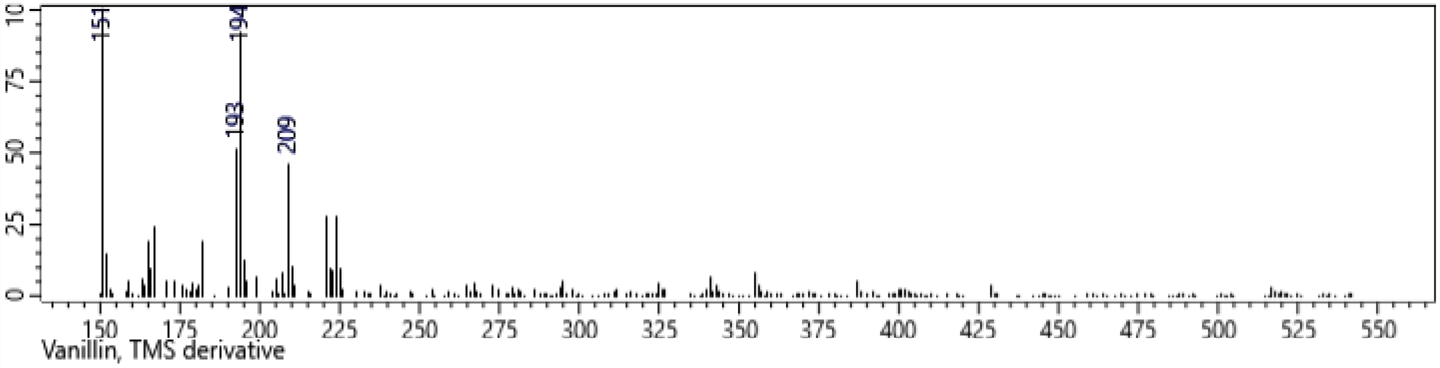


Vanillin, TMS derivative

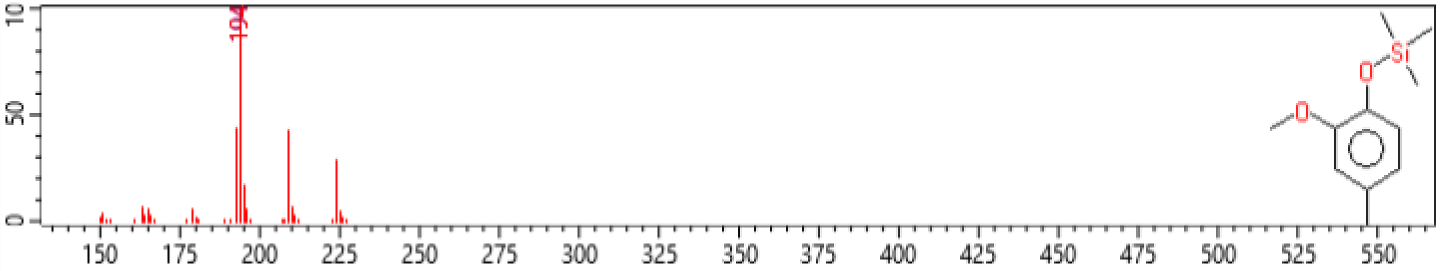


Vanillin, TMS derivative

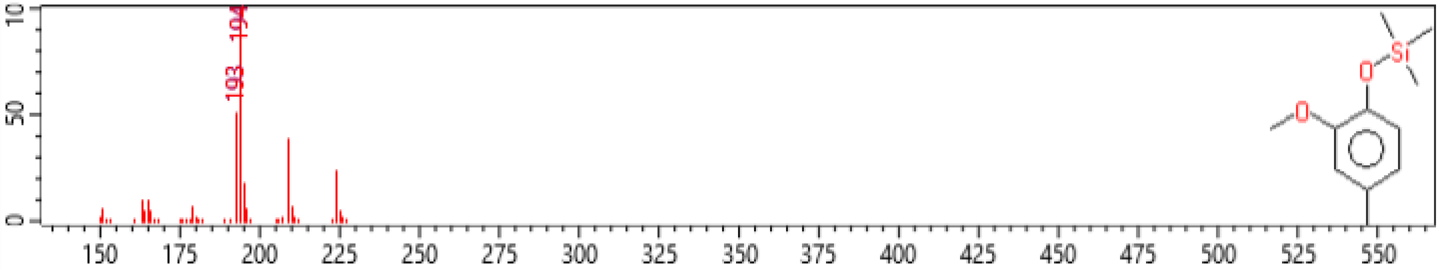


Vanillin, TMS derivative

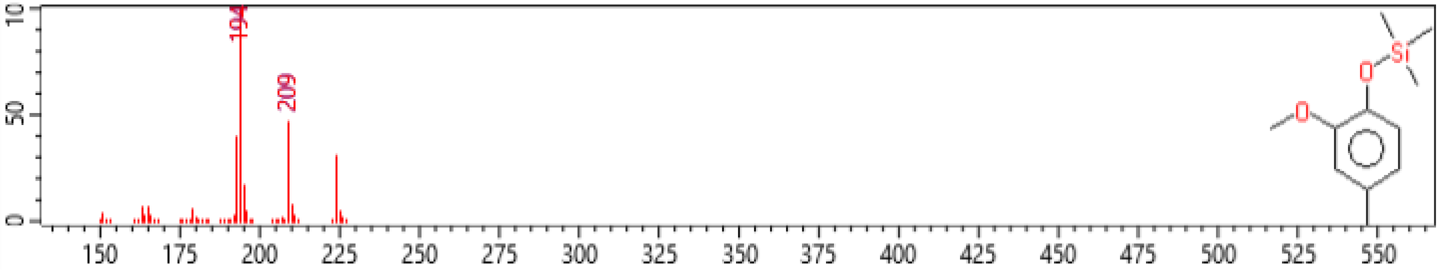


3-Hydroxy-4-methoxybenzaldehyde, TMS

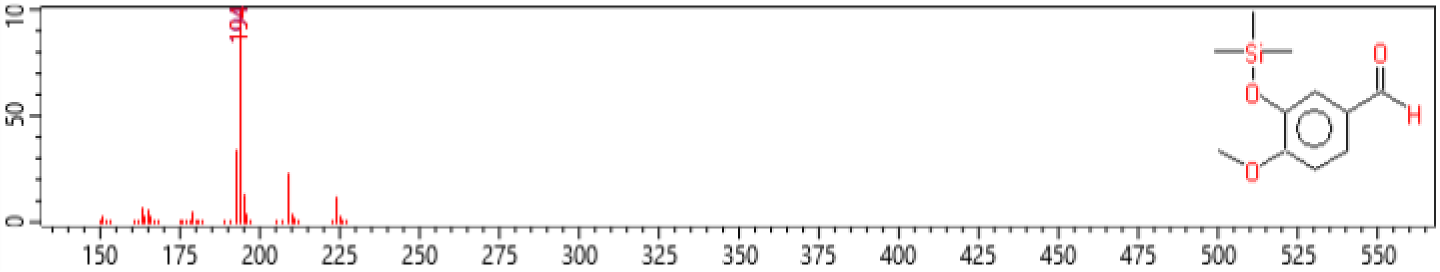

o-Vanilin, TMS

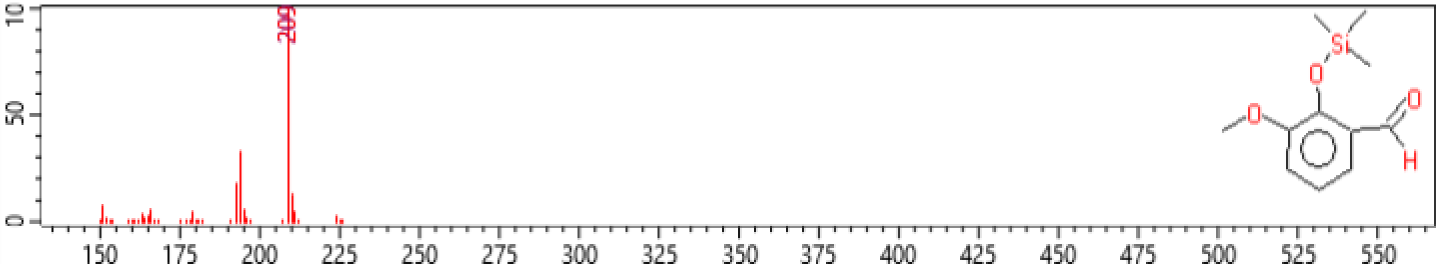


**3-Cyclopentene-1-carboxylic acid, 2-(α-chlorobenzyl)-N,N-dimethyl-, cis-**

Peak: 11.36 - 11.39 min (scan #1216 - #1226) Subtracted noise: 11.28 - 11.31 min (scan #1194 - #1204), 11.40 - 11.44 min (scan #12

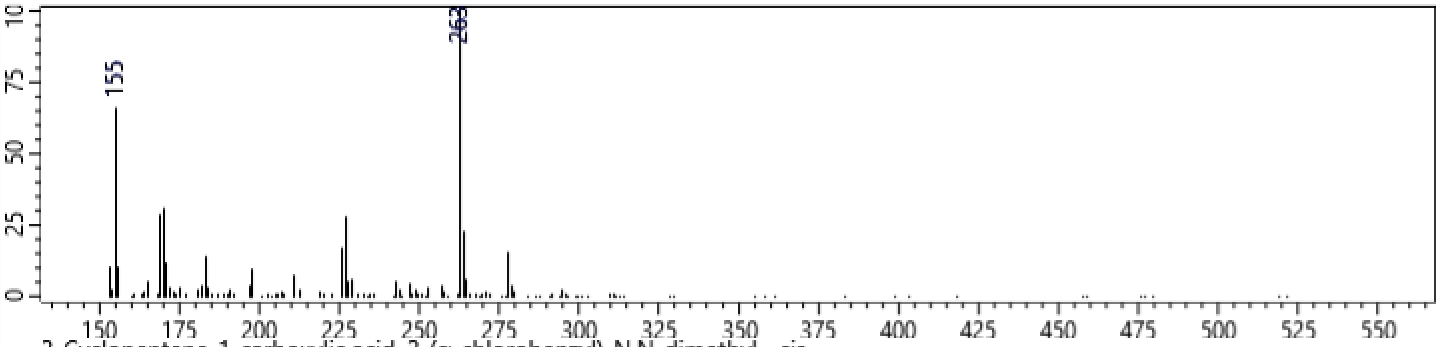


3-Cyclopentene-1-carboxylic acid, 2-(α-chlorobenzyl)-N,N-dimethyl-, cis-

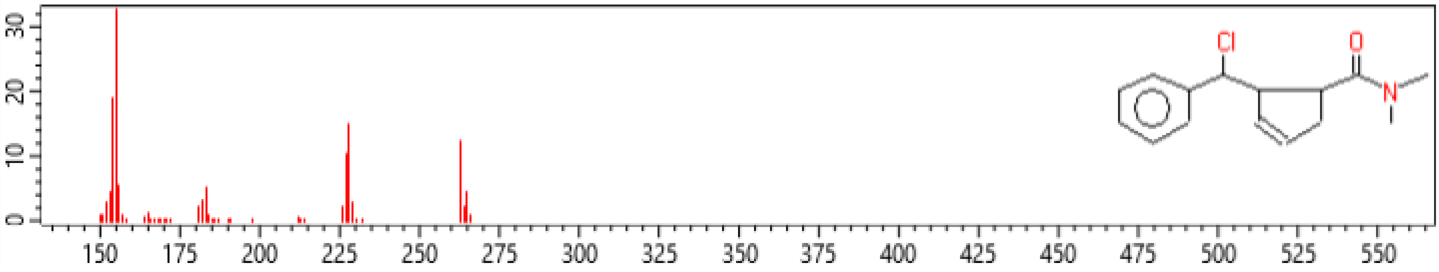

4,6-Dinitro-1,1,3,3,5-pentamethylindane

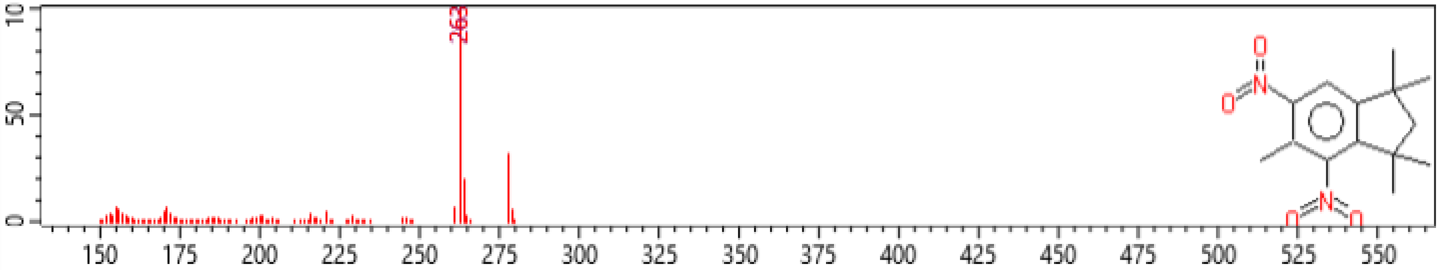


2,4-Di-tert-butylphenoxytrimethylsilane

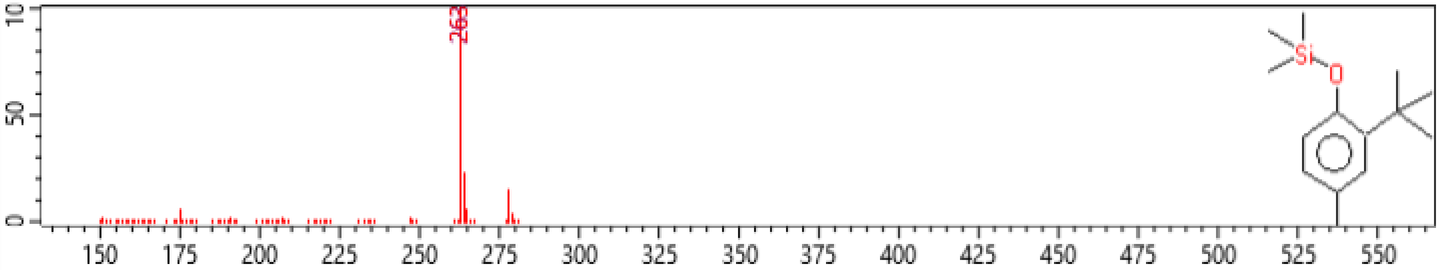

2,6-Bis(tert-butyl)phenol, TMS derivative

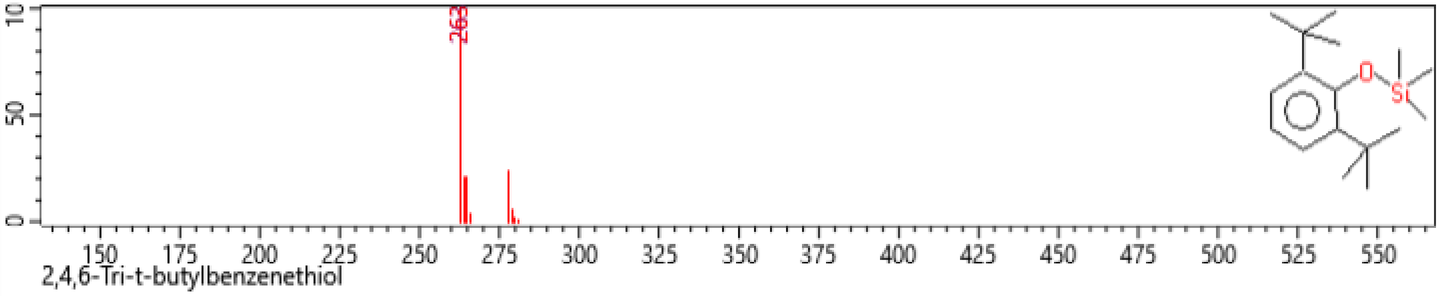


2,4,6-Tri-t-butylbenzenethiol

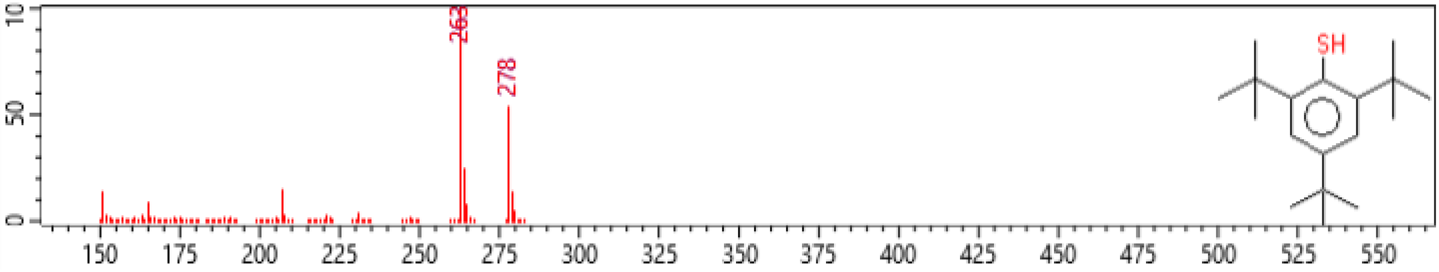


**4-Ethyl-2,6-dimethoxyphenol, TMS**

Peak: 11.41 - 11.43 min (scan #1230 - #1236) Subtracted noise: 11.38 - 11.40 min (scan #1222 - #1228), 11.45 - 11.48 min (scan #12

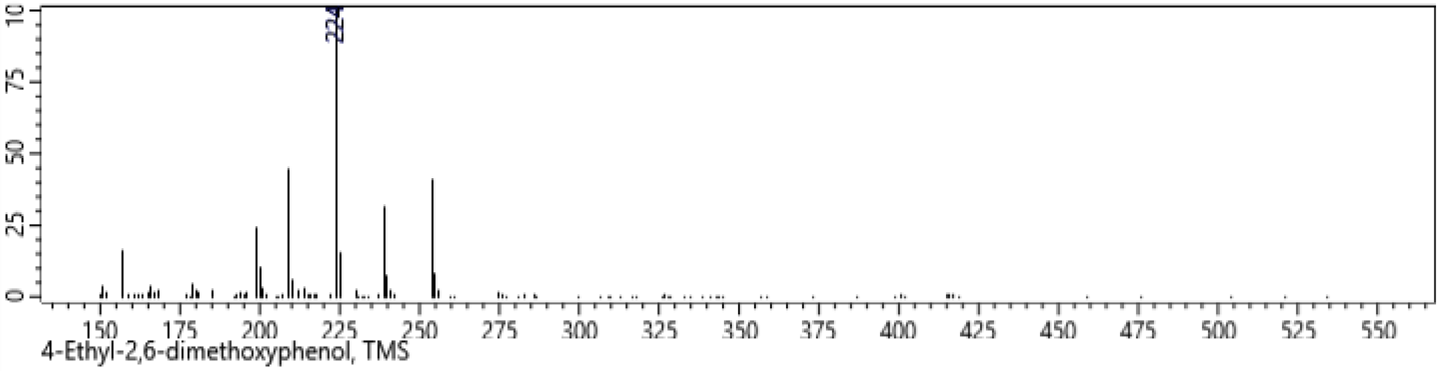


4-Ethyl-2,6-dimethoxyphenol, TMS

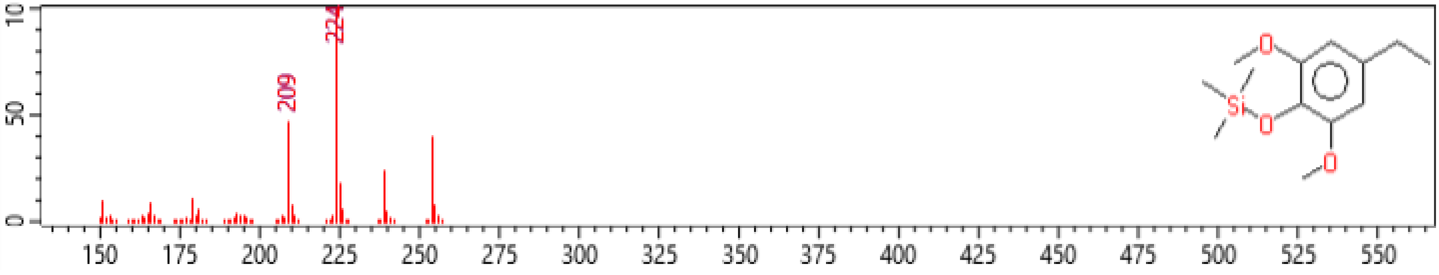


Syringaldehyde, TMS derivative

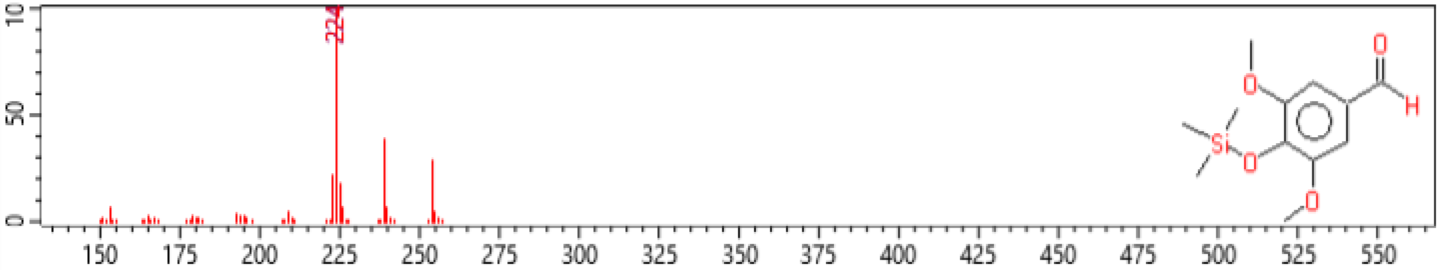

Syringaldehyde, TMS derivative

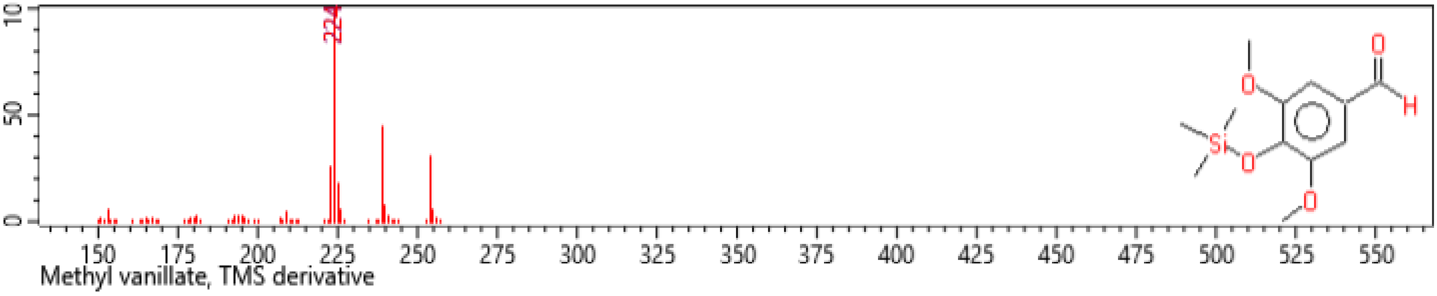


Methyl vanillate, TMS derivative

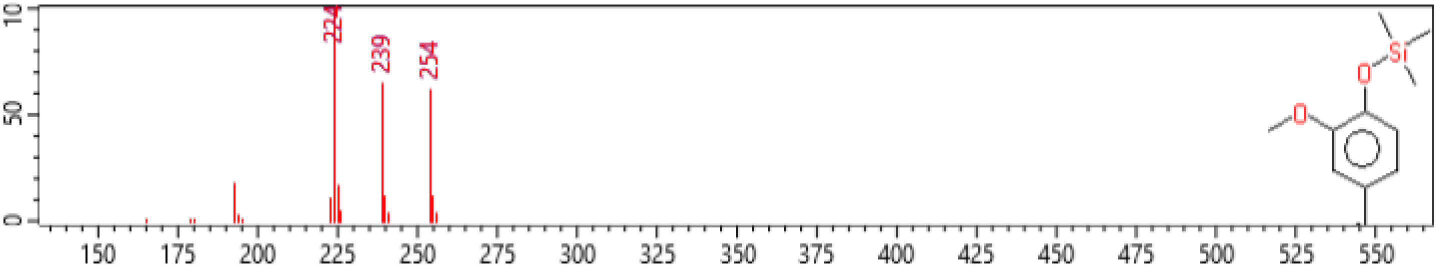


Bismuthine, trimethyl-

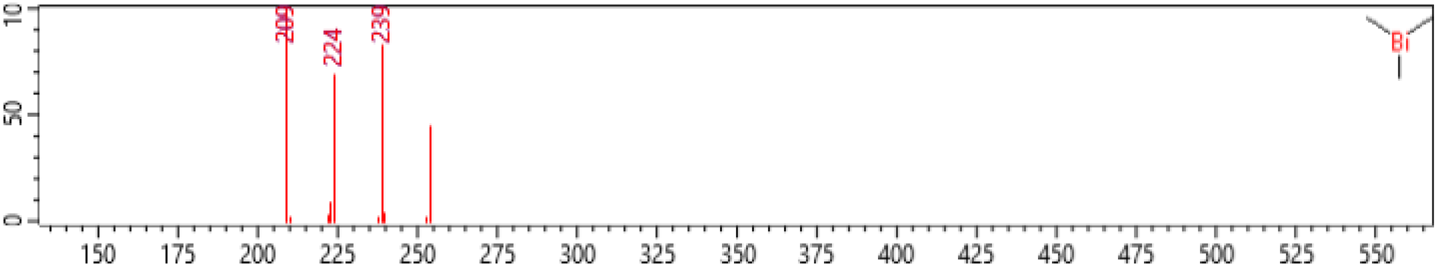


**2-[(4-Methyl-1-piperazinyl)carbonyl]-1H-indole**

Peak: 11.47 - 11.48 min (scan #1246 - #1250) Subtracted noise: 11.44 - 11.45 min (scan #1238 - #1242), 11.50 - 11.51 min (scan #12

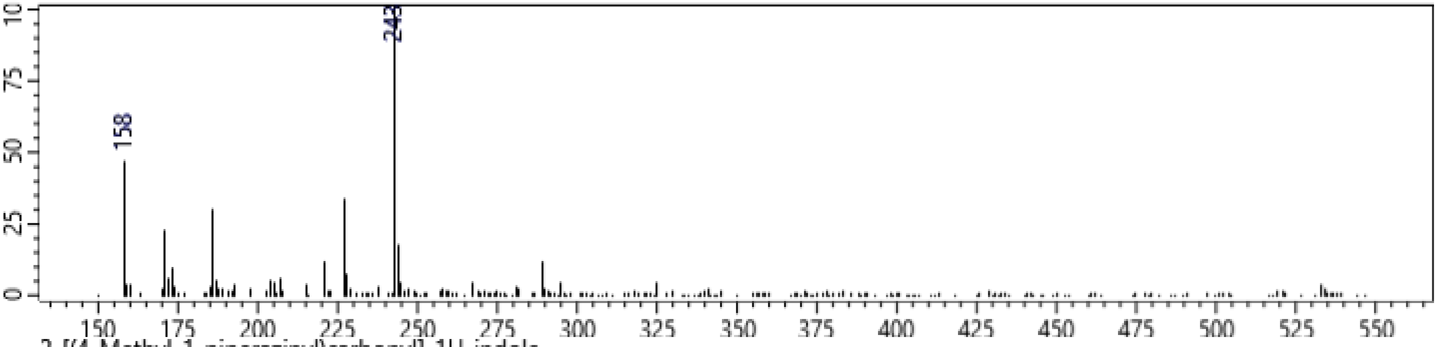


2-[(4-Methyl-1-piperazinyl)carbonyl]-1H-indole

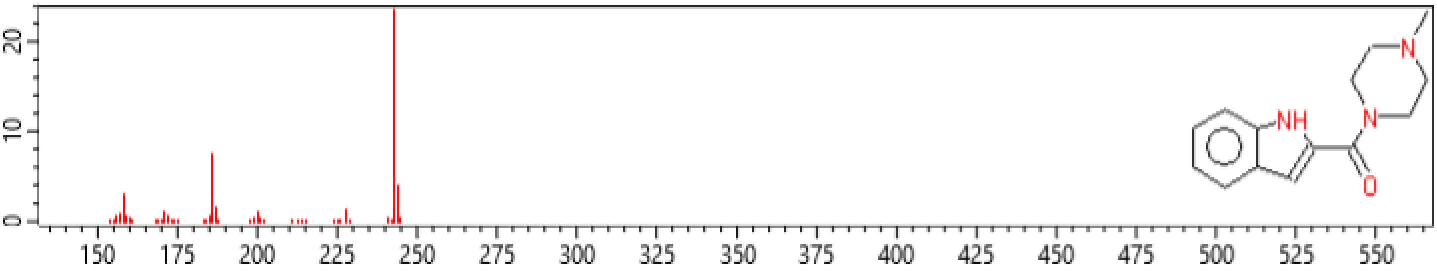

3,10-Dimethyl-pyrido[3,2-g]pteridin-2,4(3H,10H)-dione

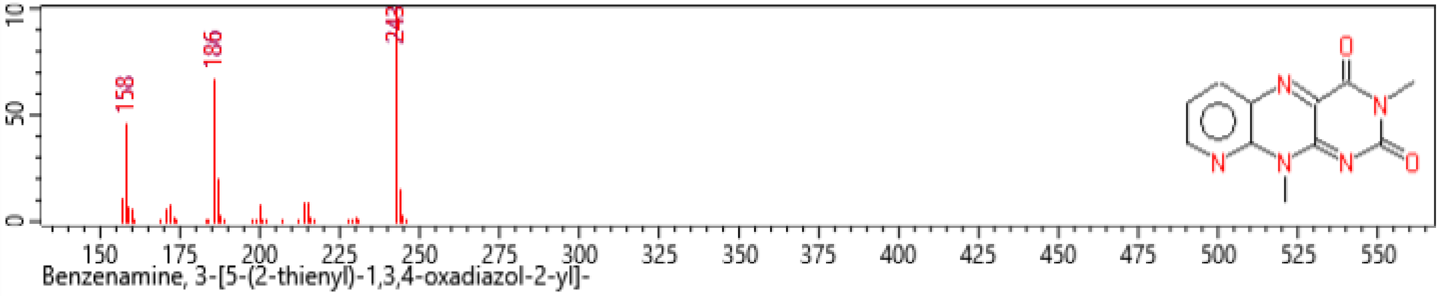

Benzenamine, 3-[5-(2-thienyl)-1,3,4-oxadiazol-2-yl]-

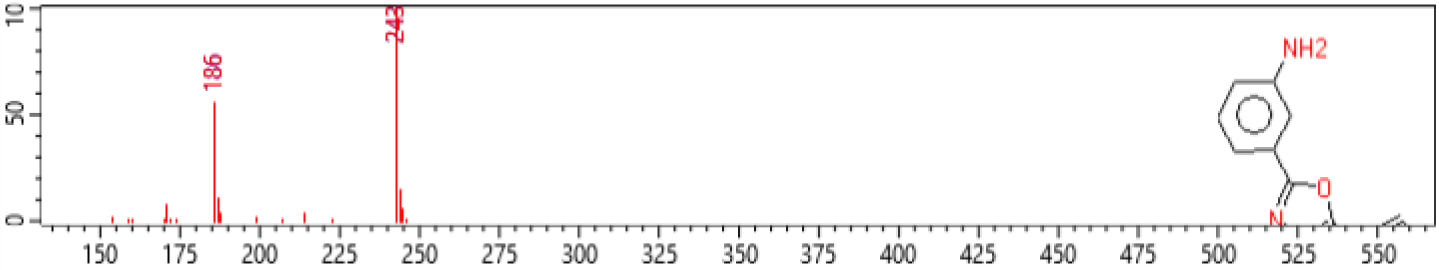

2,2-Di-morpholin-4-yl-3-oxo-butyric acid methyl ester

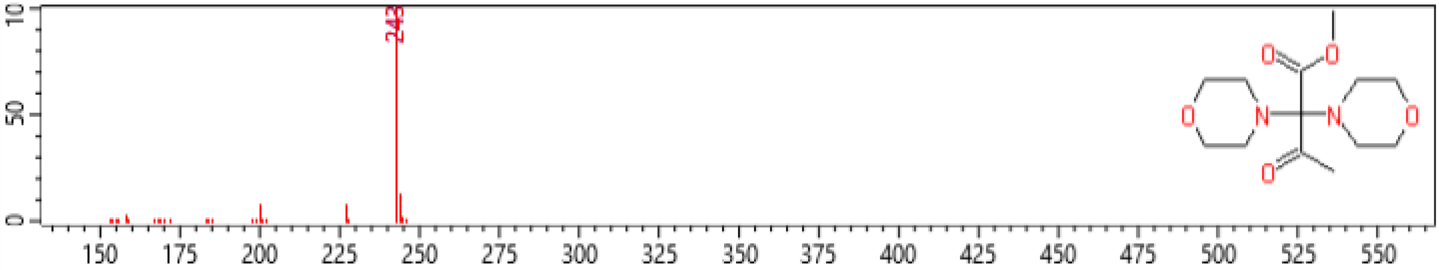

8H-Pyrano[2,3-e]benzothiophen-8-one, 4-formamido-6-methyl-

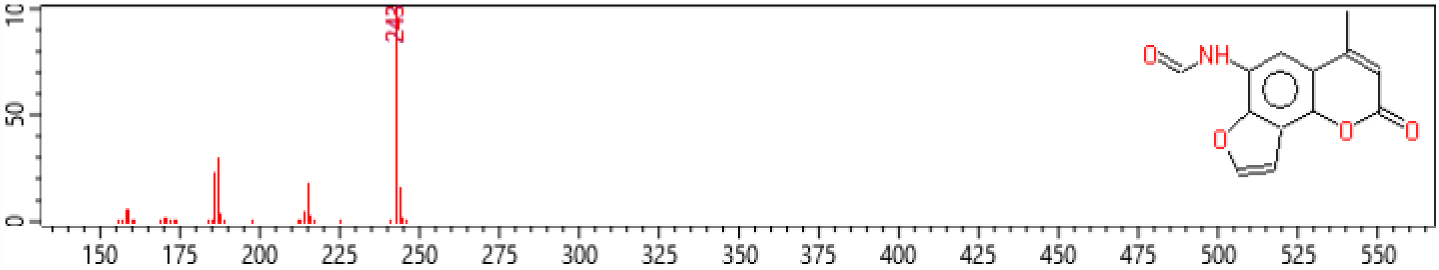

**3,4-Dimethyl-1-methyl(tetramethylene)silyloxycyclohexane**

Peak: 11.55 - 11.58 min (scan #1271 - #1277) Subtracted noise: 11.52 - 11.54 min (scan #1261 - #1267), 11.59 - 11.61 min (scan #12

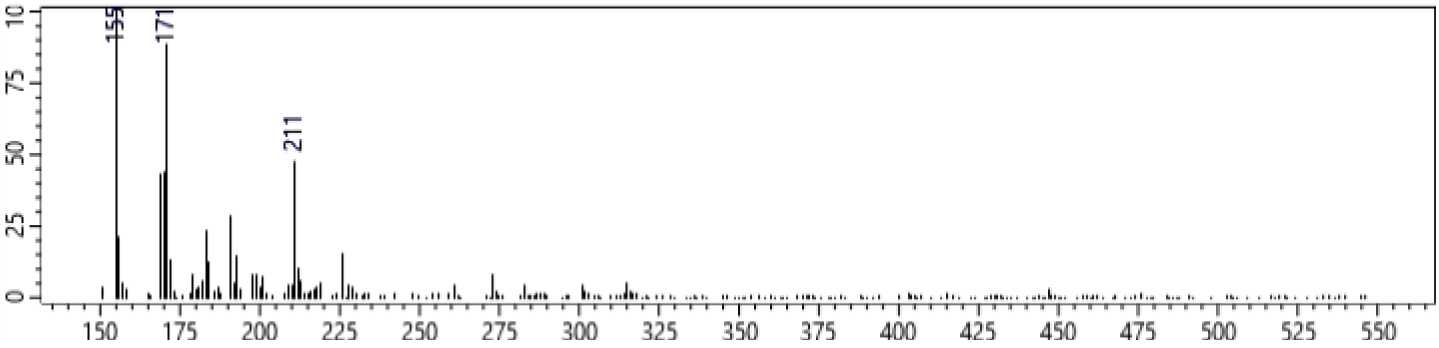

3,4-Dimethyl-1-methyl(tetramethylene)silyloxycyclohexane

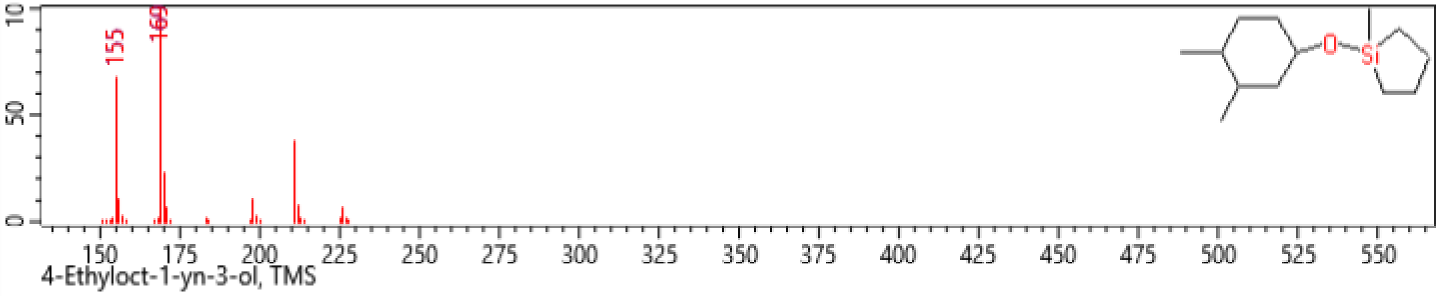


4-Ethyloct-1-yn-3-ol, TMS

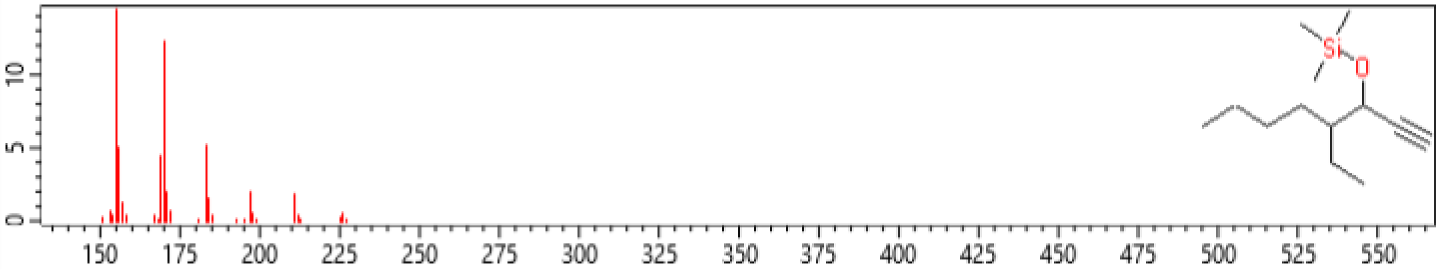


2',3'-Dideoxy-3'-fluorouridine, TMS

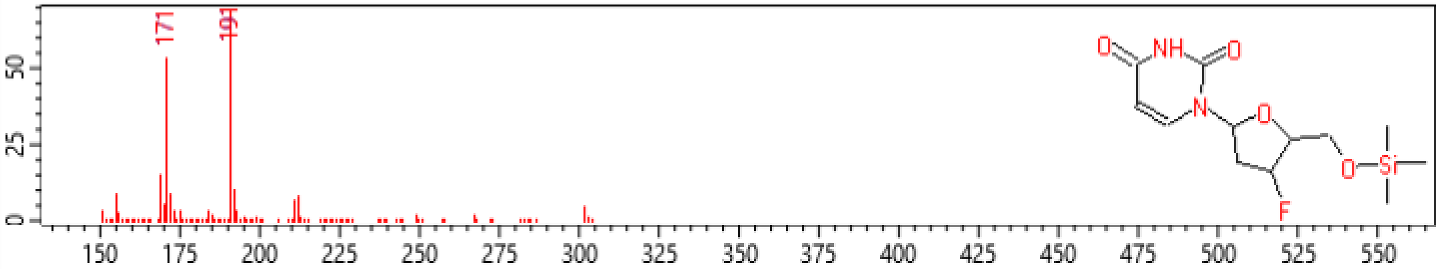


6β-Hydroxy-oral turinabol

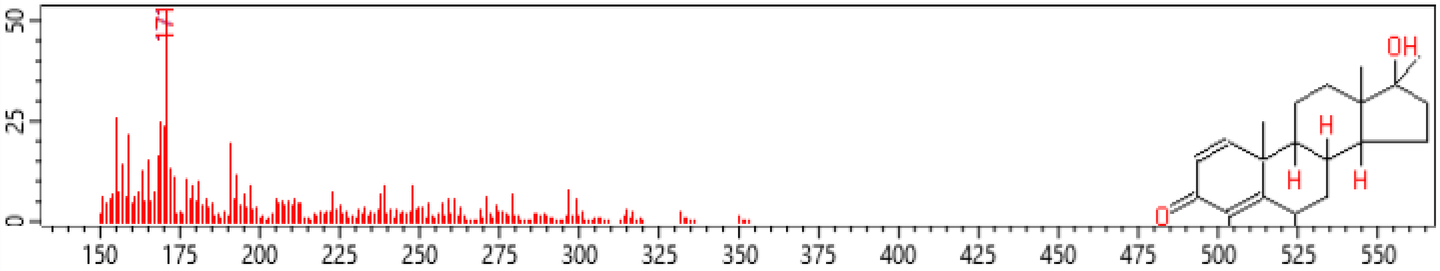


9(1H)-Phenanthrone, 2,3,4,4a-tetrahydro-1α,4aβ-dimethyl-

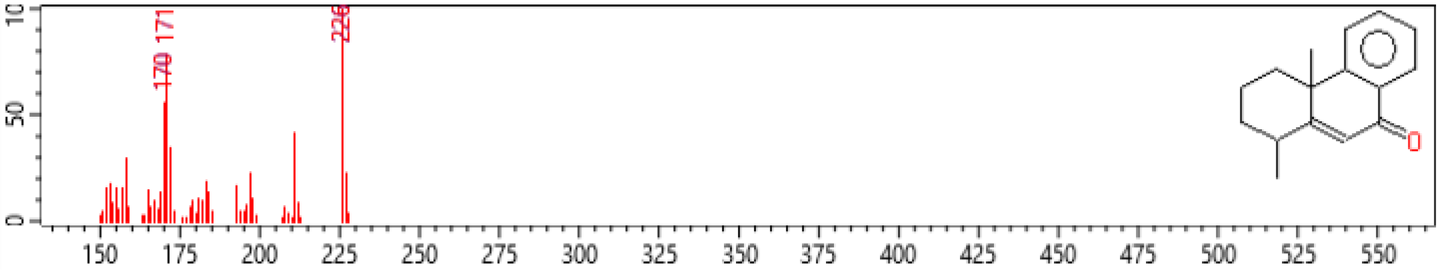

**1-Dodecanol, TMS derivative**

Peak: 11.62 - 11.68 min (scan #1289 - #1305) Subtracted noise: 11.53 - 11.59 min (scan #1264 - #1280), 11.72 - 11.77 min (scan #13

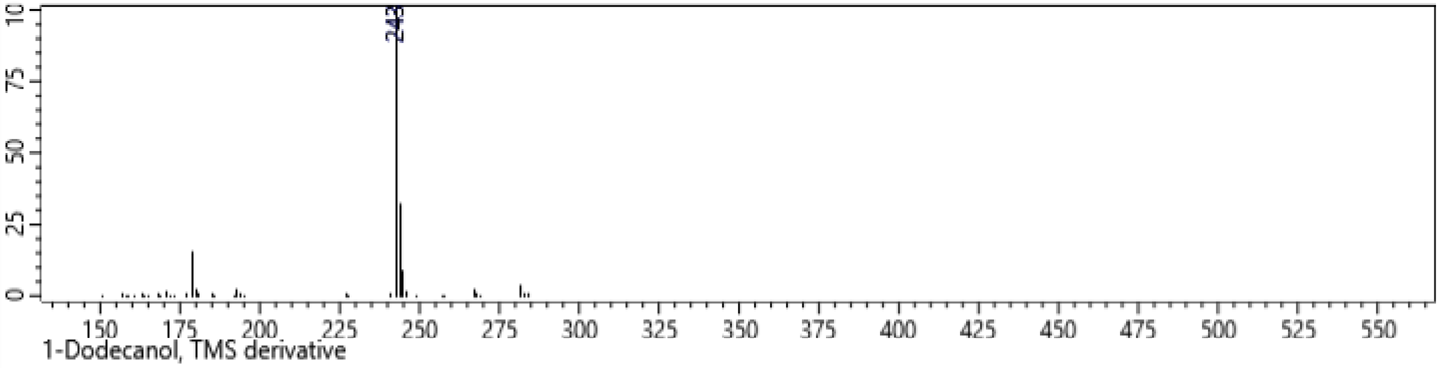


1-Dodecanol, TMS derivative

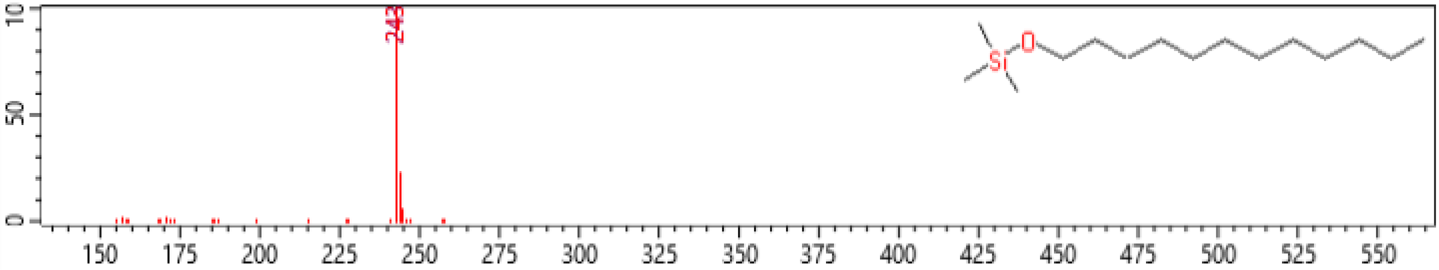


1-Dodecanol, TBDMS derivative

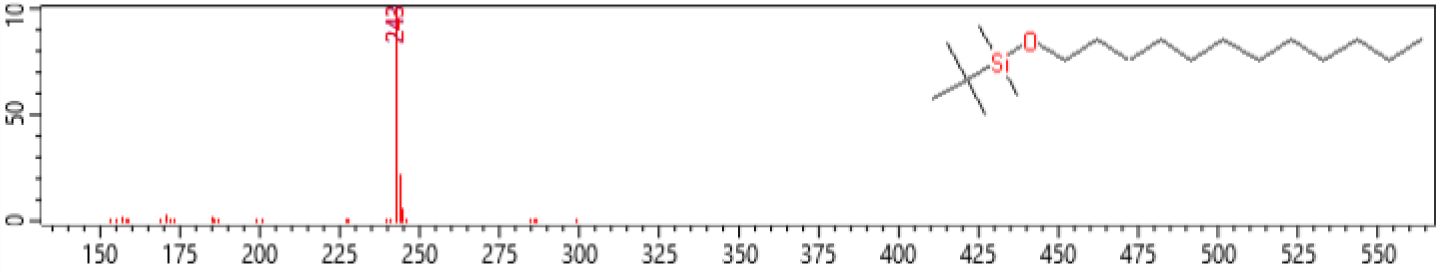


Undecanoic acid, TBDMS derivative

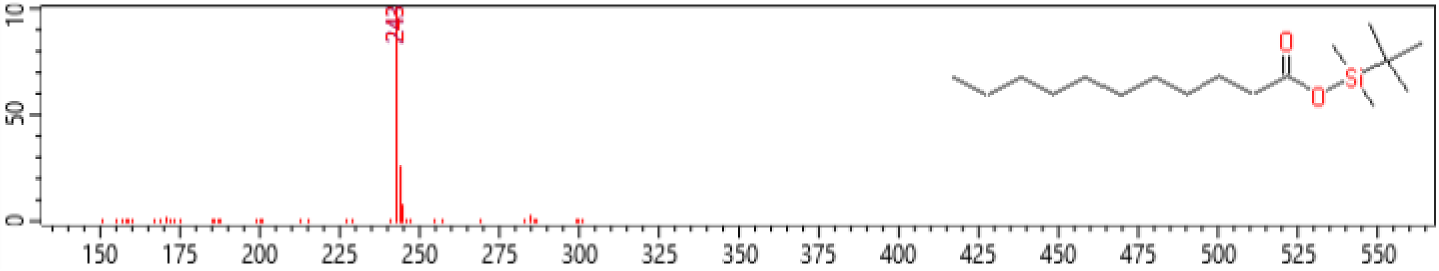


2-Butyloctanol, TMS derivative

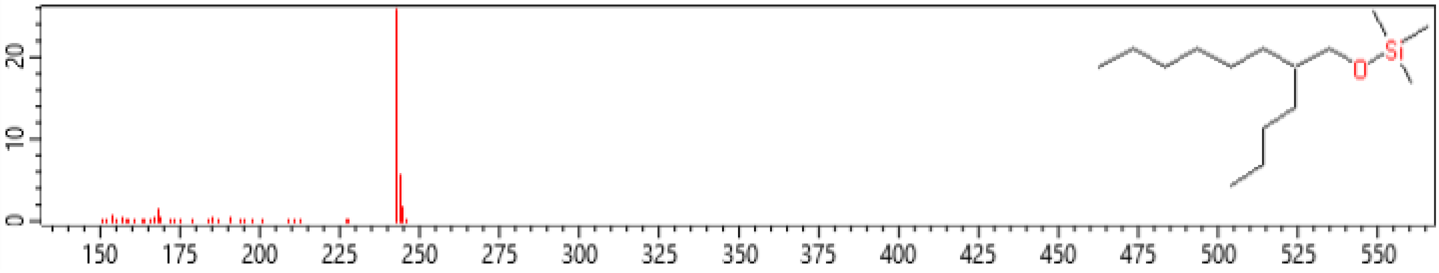

Undecanoic acid, TBDMS derivative

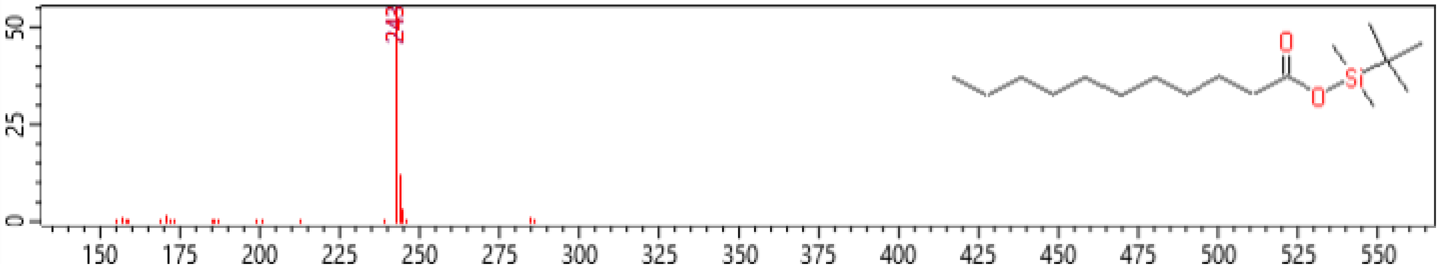


**Silane, (1-cyclohexen-1-yloxy)trimethyl-**

Peak: 11.78 - 11.81 min (scan #1333 - #1341) Subtracted noise: 11.72 - 11.75 min (scan #1317 - #1325), 11.81 - 11.84 min (scan #13

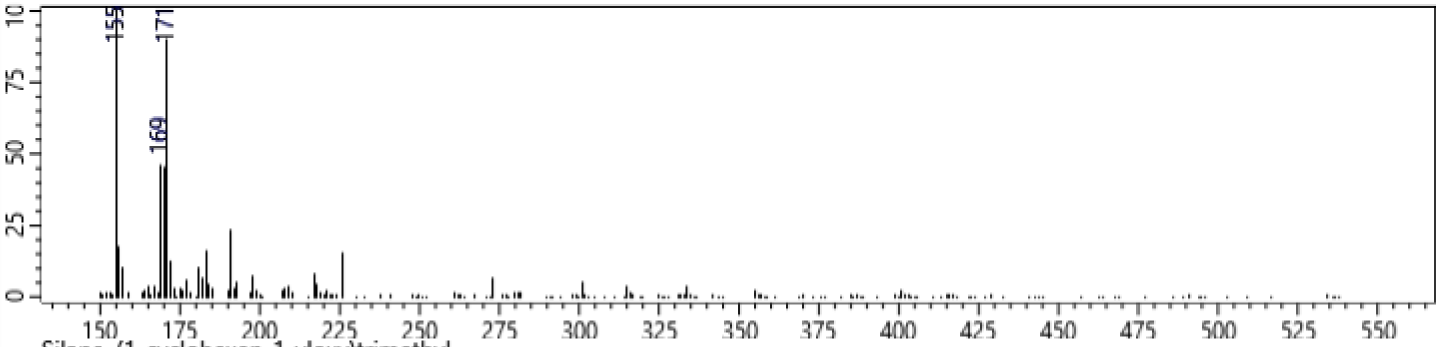


Silane, (1-cyclohexen-1-yloxy)trimethyl-

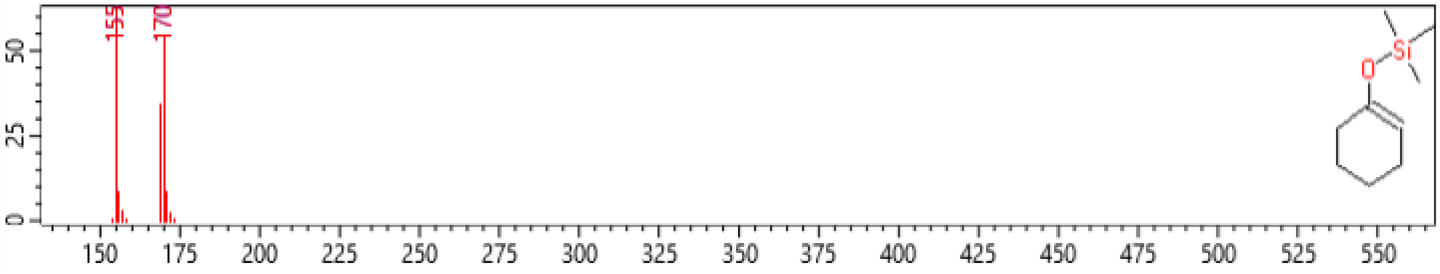


1-Chloro-3-methoxy-1,3-dimethyldisiloxane

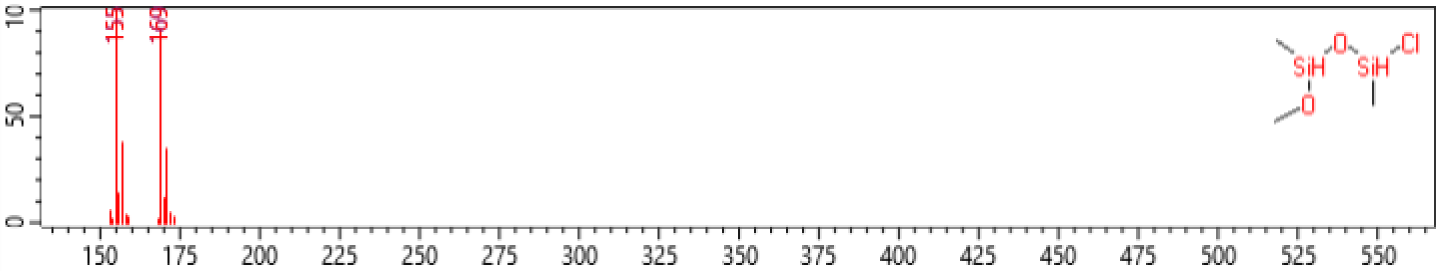


1,10-Decanedione, 1,10-di-1-naphthyl-

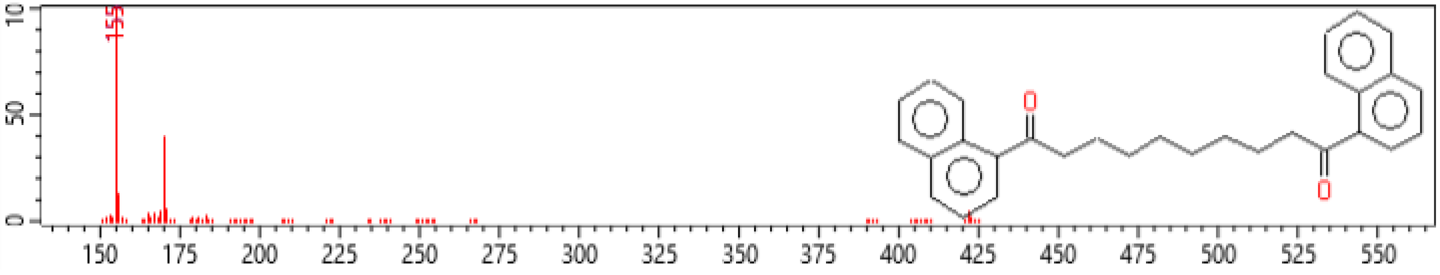

Butyl(trimethylsilyl)ketene

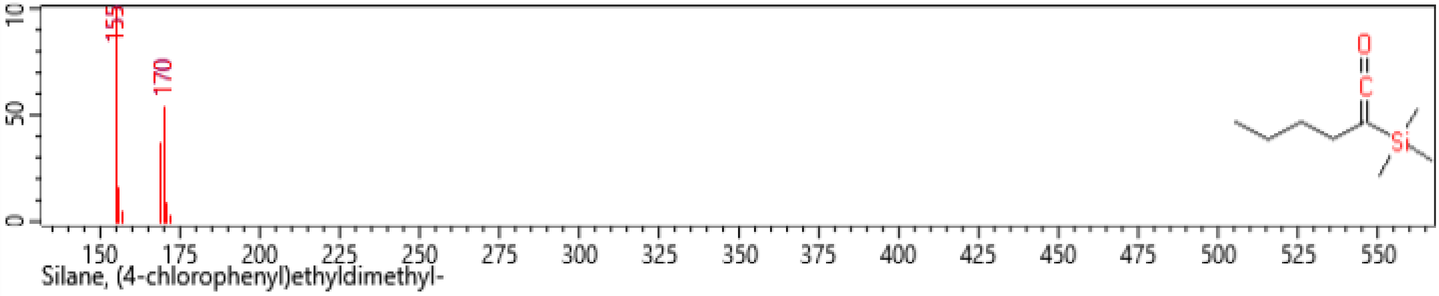


Silane, (4-chlorophenyl)ethyldimethyl-

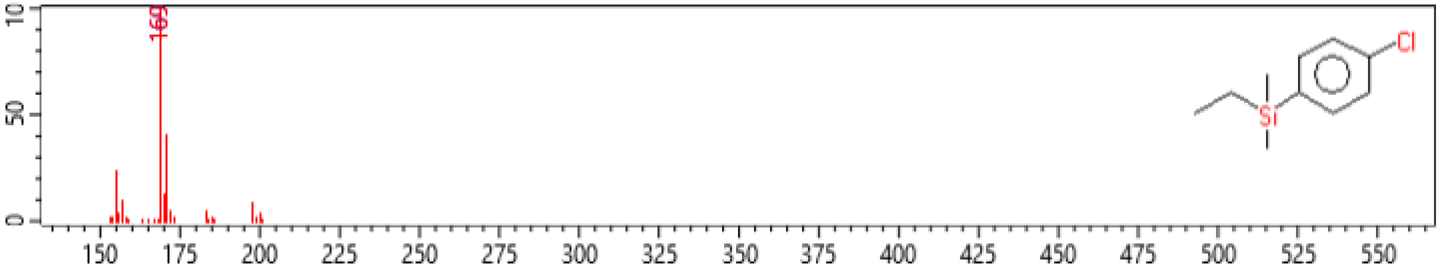


**Phosphoric acid, 2,2-diethoxyethyl bis(trimethylsilyl) ester**

Peak: 11.82 - 11.88 min (scan #1346 - #1362) Subtracted noise: 11.75 - 11.81 min (scan #1326 - #1341), 11.93 - 11.98 min (scan #13

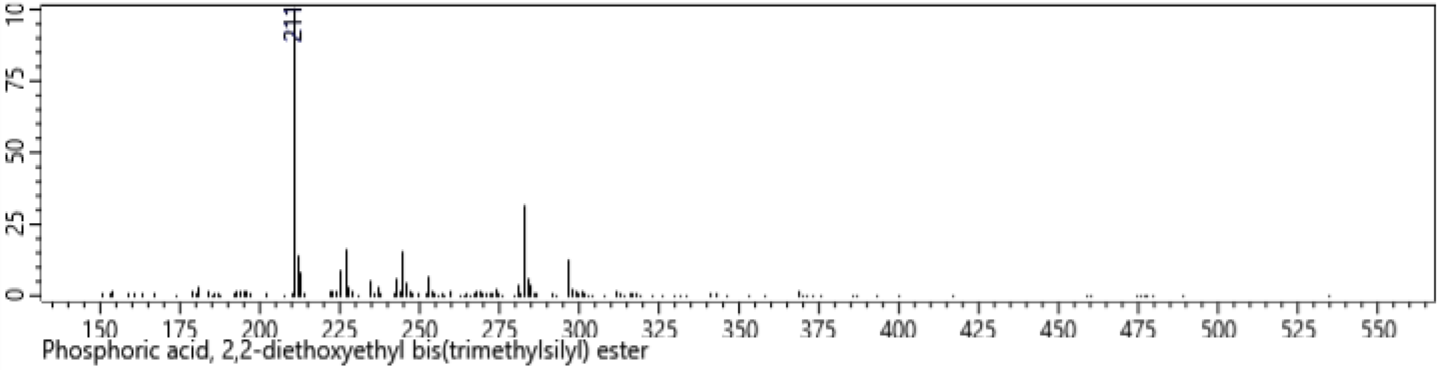


Phosphoric acid, 2,2-diethoxyethyl bis(trimethylsilyl) ester

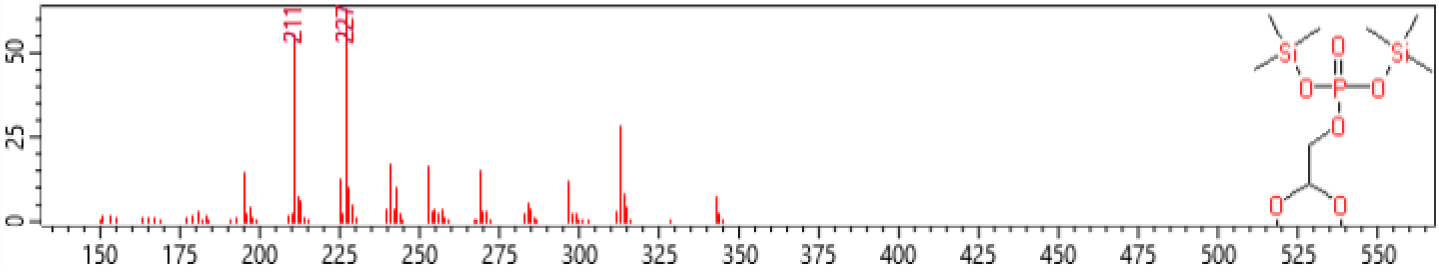


Benzenethanamine, N-acetyl-3-benzyloxy-β-oxo-

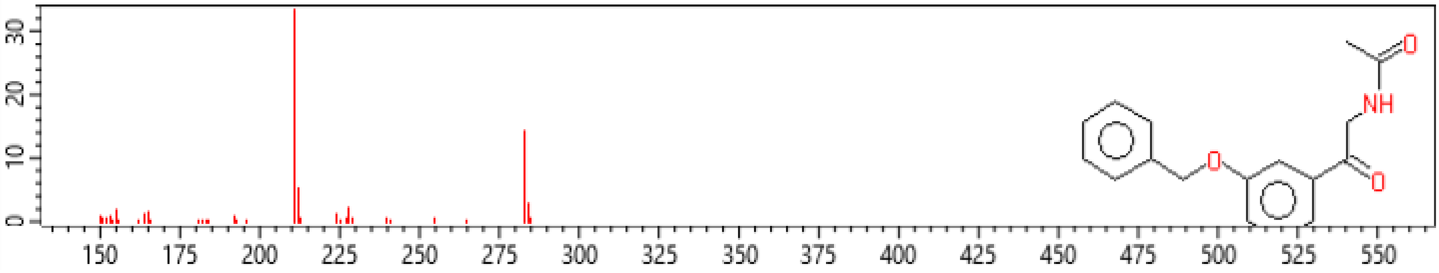

Dihydronormorphinone, 3-desoxy-N-formyl-

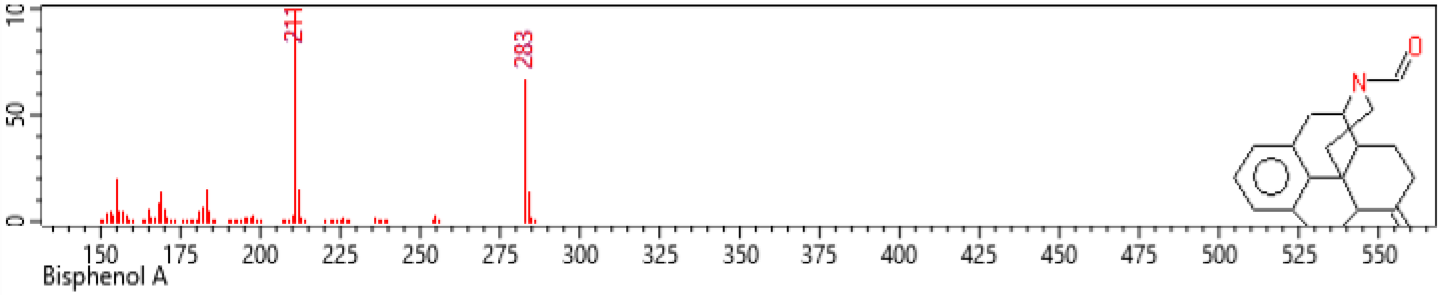


Bisphenol A

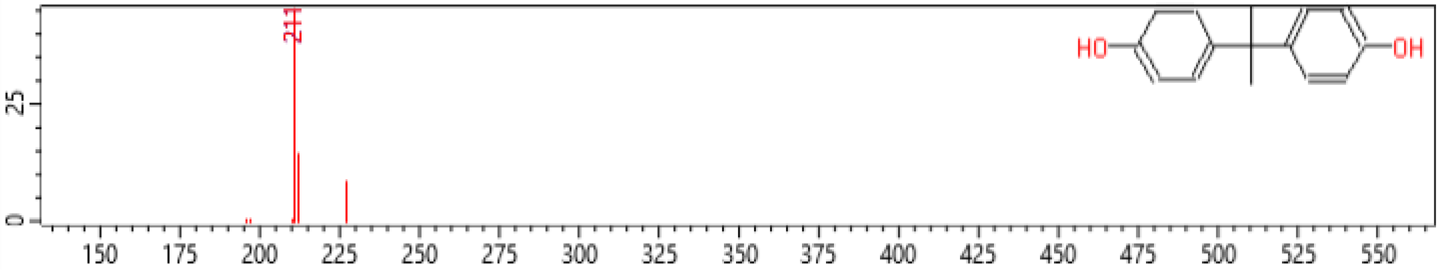


2-Ethylhexyl dihydrogen phosphate, 2TMS derivative

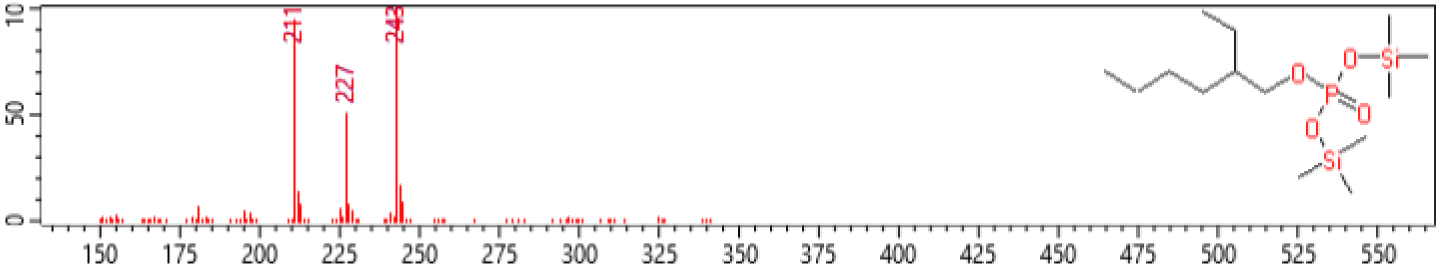


**3-(2-Benzothiazolyl)-6-methoxycoumarin**

Peak: 11.93 - 11.96 min (scan #1375 - #1385) Subtracted noise: 11.90 - 11.92 min (scan #1366 - #1374), 12.00 - 12.03 min (scan #13[illegible]

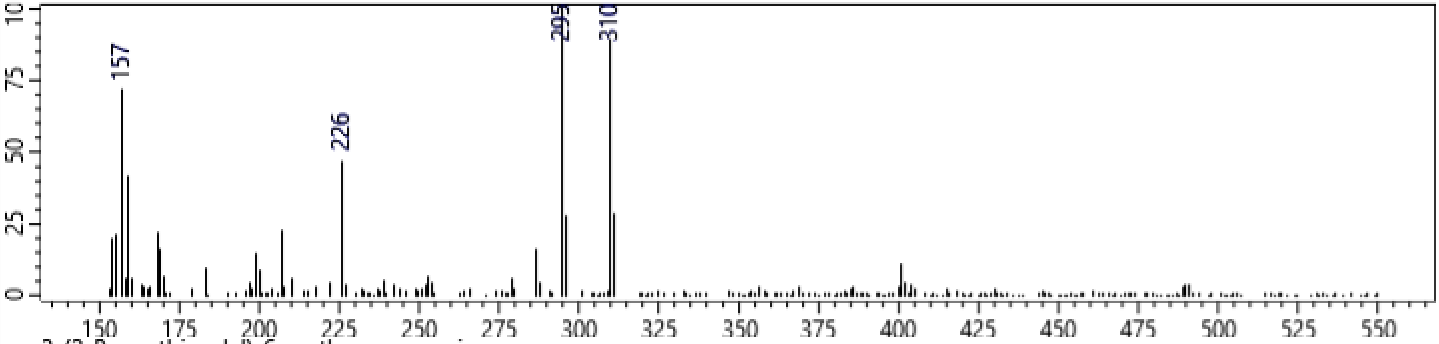


3-(2-Benzothiazolyl)-6-methoxycoumarin

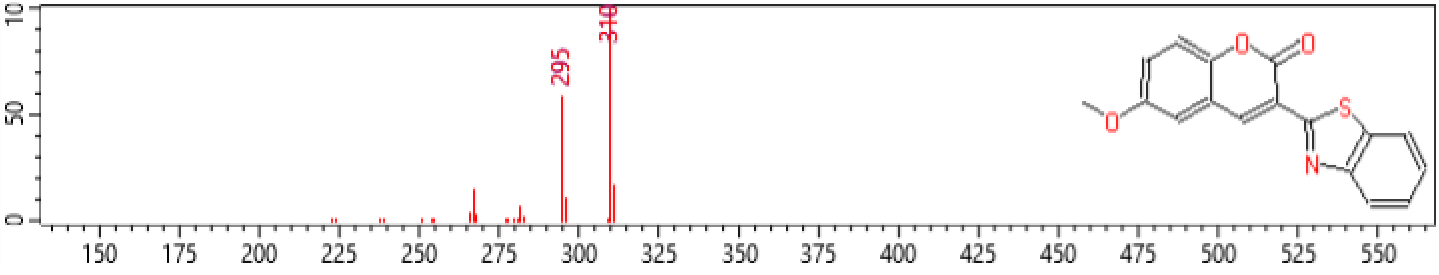

4-(4-Trifluoromethylphenyl)phenol, TMS

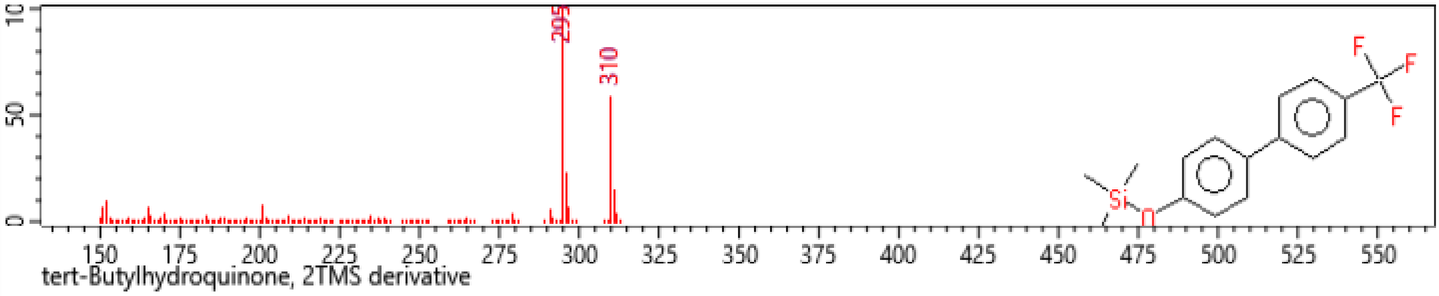

tert-Butylhydroquinone, 2TMS derivative

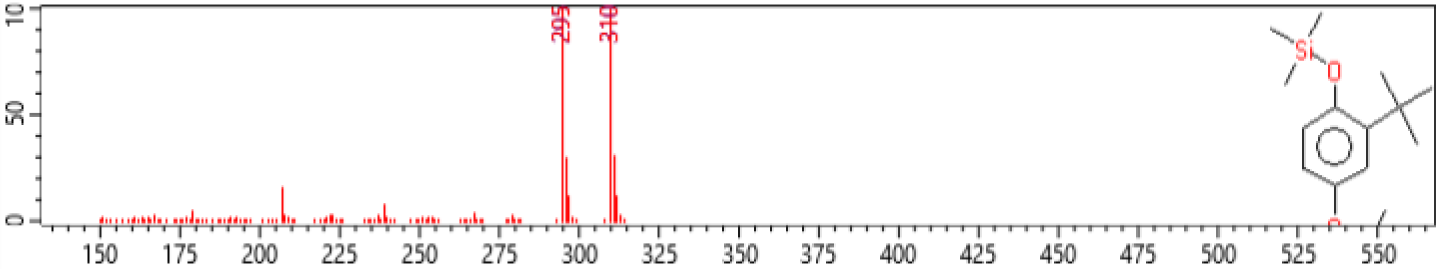

2,6-Di-tert-butyl-4-mercaptophenol, S-trimethylsilyl-

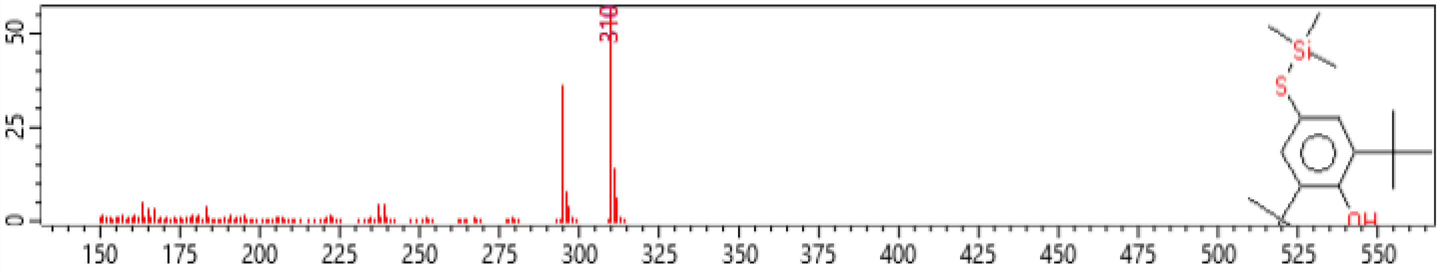

tert-Butylhydroquinone, 2TMS derivative

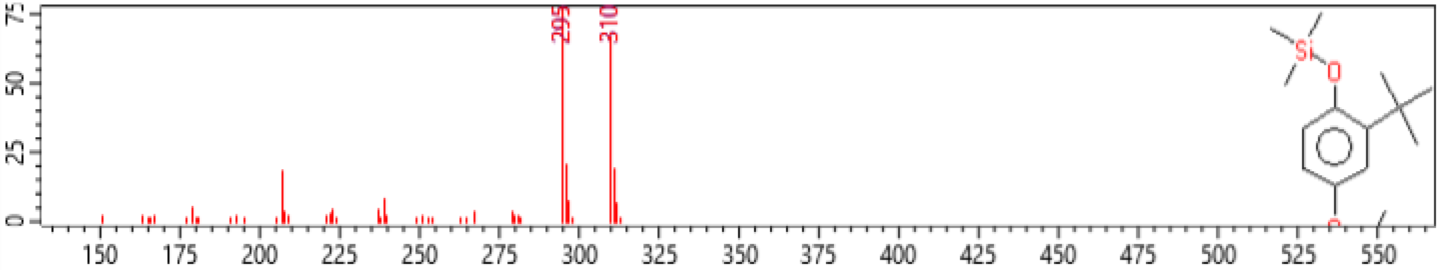

**Benzo[b]quinoline-1,9(2H,8H)-dione, 3,4,6,7-tetrahydro-3,3,7,7,10-pentamethyl-, dioxime**

Peak: 12.07 - 12.09 min (scan #1416 - #1421) Subtracted noise: 12.04 - 12.06 min (scan #1407 - #1412), 12.11 - 12.12 min (scan #14

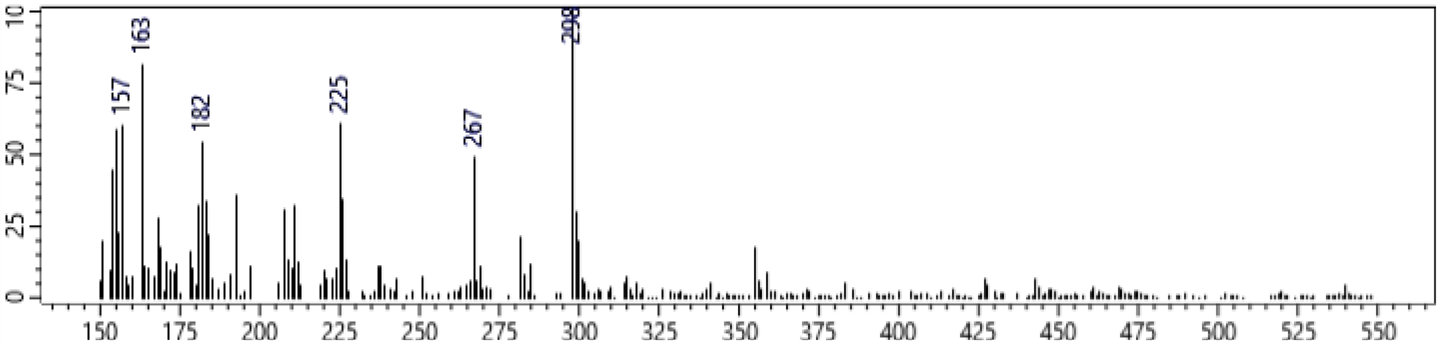

Benzo[b]quinoline-1,9(2H,8H)-dione, 3,4,6,7-tetrahydro-3,3,7,7,10-pentamethyl-, dioxime

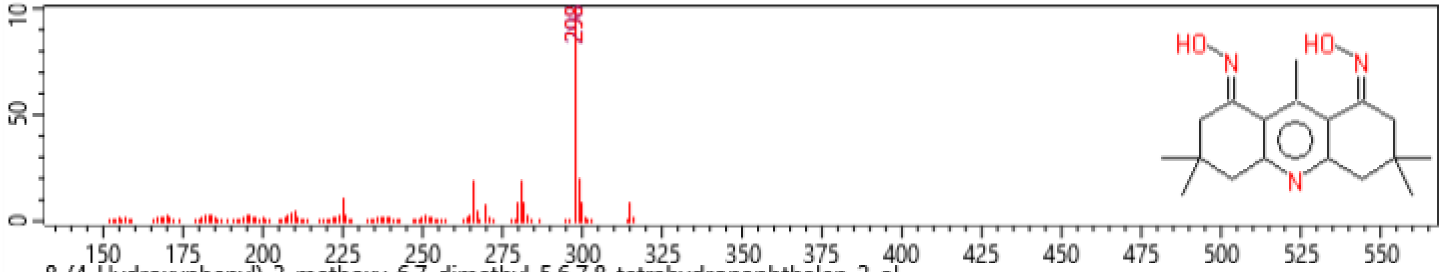


8-(4-Hydroxyphenyl)-3-methoxy-6,7-dimethyl-5,6,7,8-tetrahydronaphthalen-2-ol

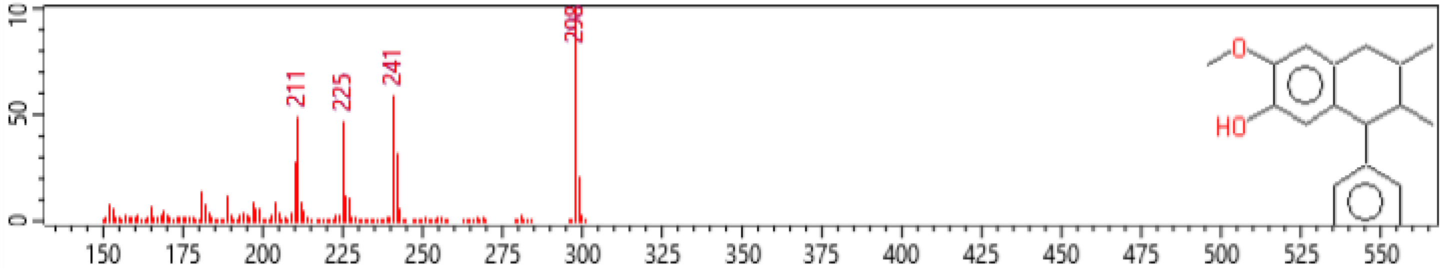


1-[1,8-bis(Dimethylamino)naphthalen-2-yl]pentan-1-one

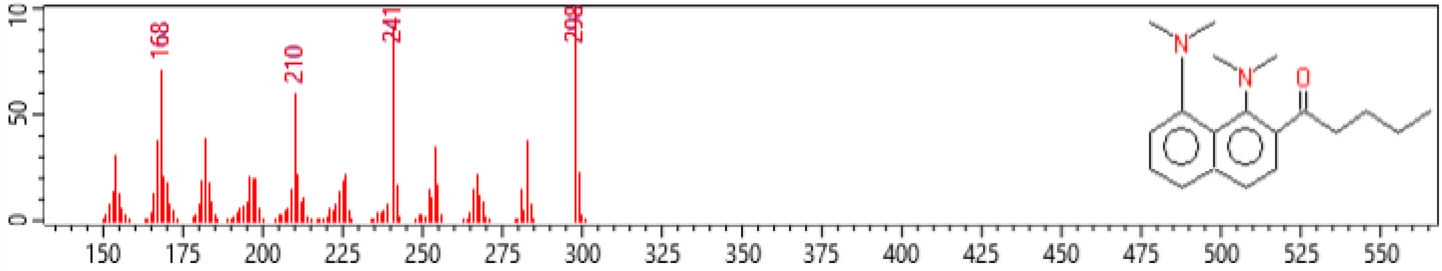


1,4,7-Androstatrien-3,17-dione

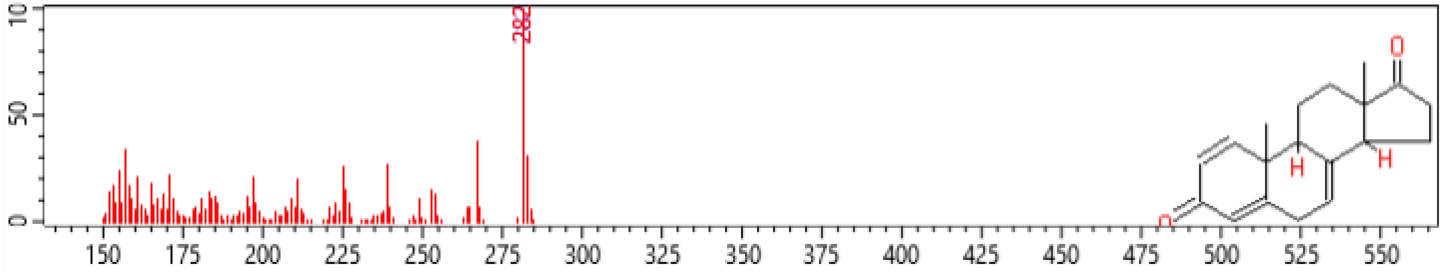


2,7-Diphenyl-1,6-dioxopyridazino[4,5:2',3']pyrrolo[4',5'-d]pyridazine

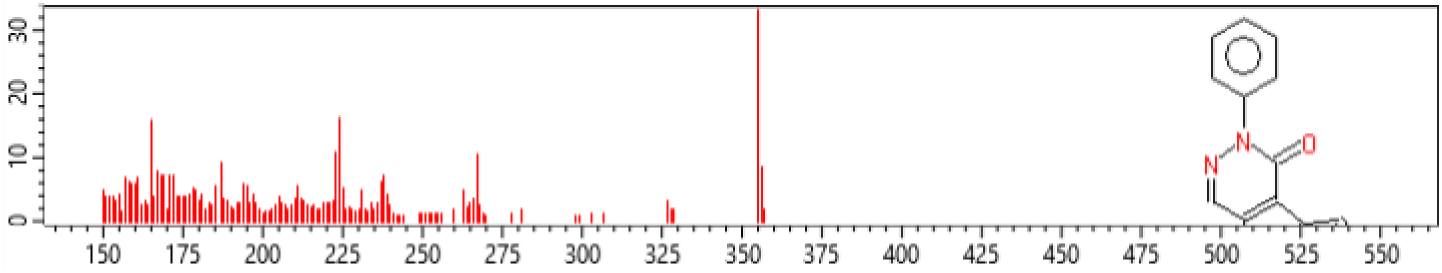

**4-Hydroxybenzoic acid, 2TMS derivative**

Peak: 12.14 - 12.18 min (scan #1435 - #1445) Subtracted noise: 12.09 - 12.13 min (scan #1421 - #1431), 12.21 - 12.25 min (scan #14

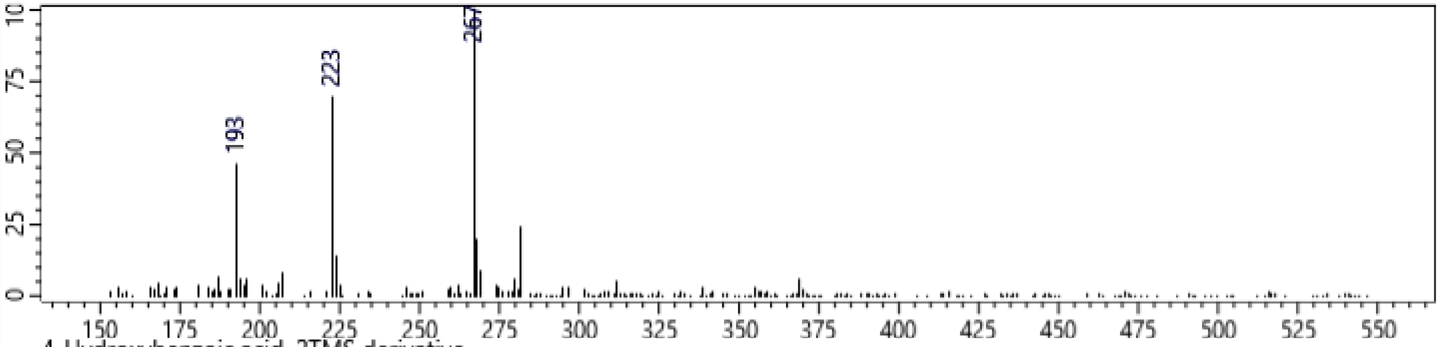


4-Hydroxybenzoic acid, 2TMS derivative

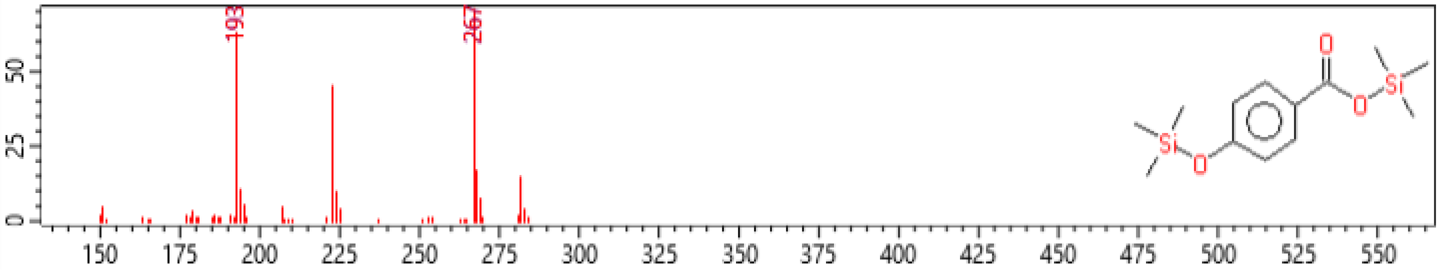


4-Hydroxybenzoic acid, 2TMS derivative

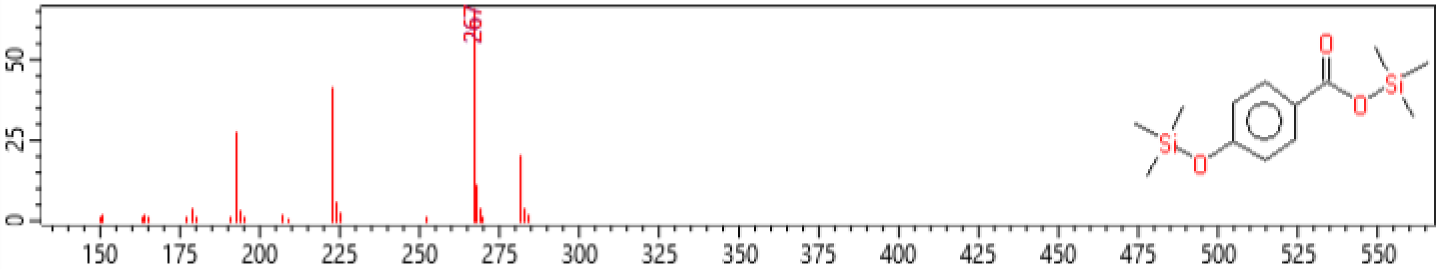


4-Hydroxybenzoic acid, 2TMS derivative

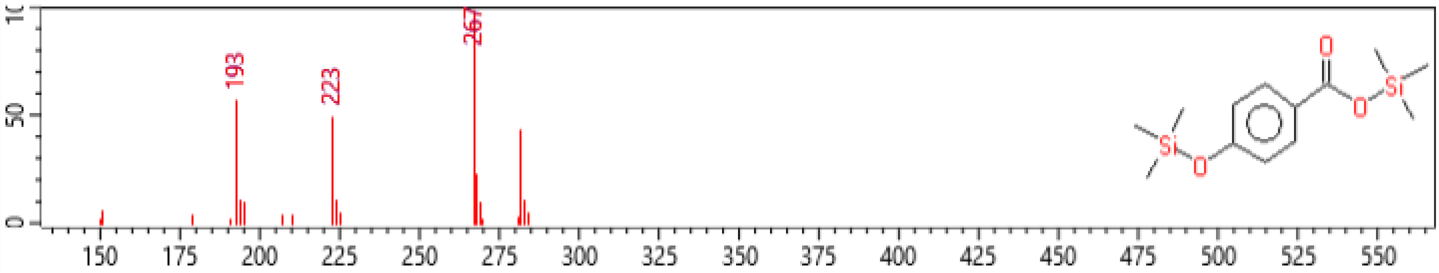


3-Hydroxybenzoic acid, 2TMS derivative

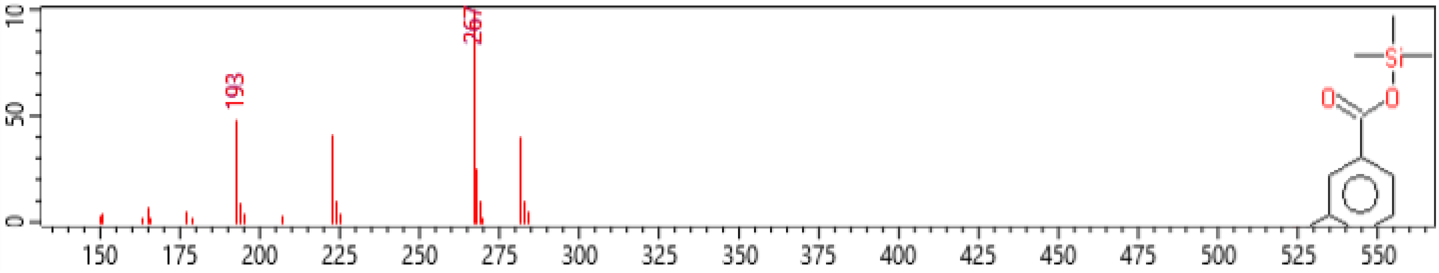

3-Hydroxybenzoic acid, 2TMS derivative

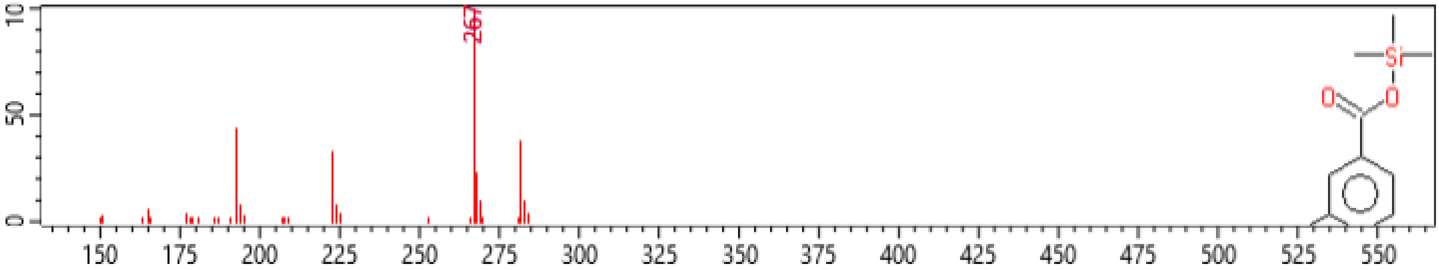


**2-Methoxy-5-nitrophenol, TMS**

Peak: 12.24 - 12.29 min (scan #1462 - #1475) Subtracted noise: 12.16 - 12.21 min (scan #1440 - #1453), 12.31 - 12.36 min (scan #14

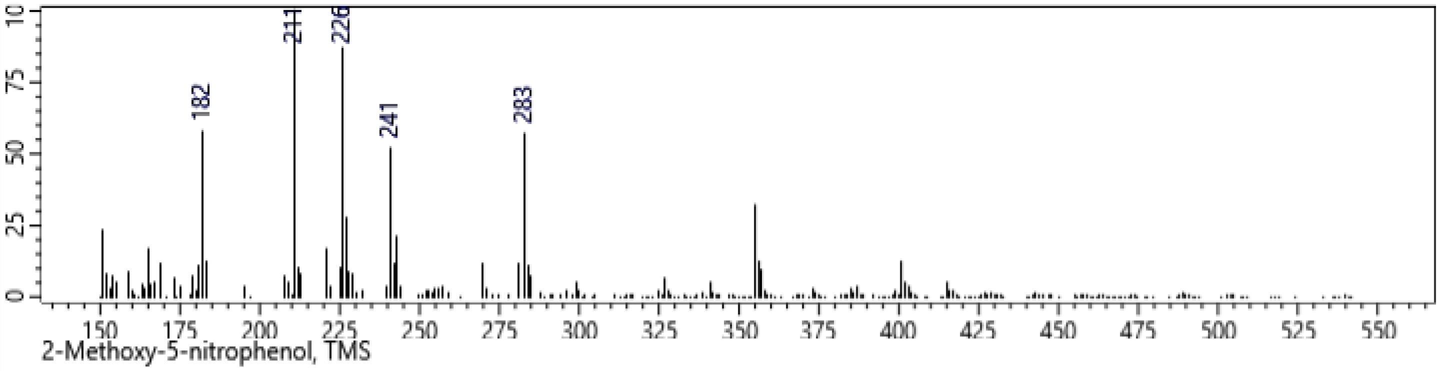


2-Methoxy-5-nitrophenol, TMS

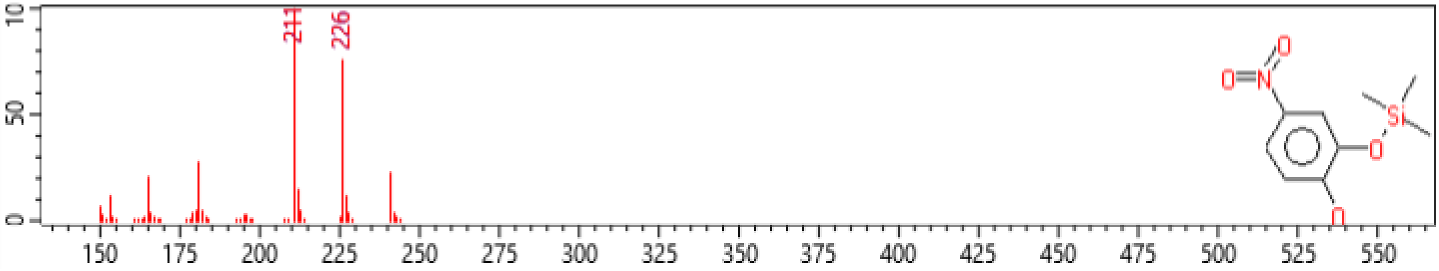


2,2-Bis(4-hydroxy-3-methylphenyl)-4-methylpentane, acetate

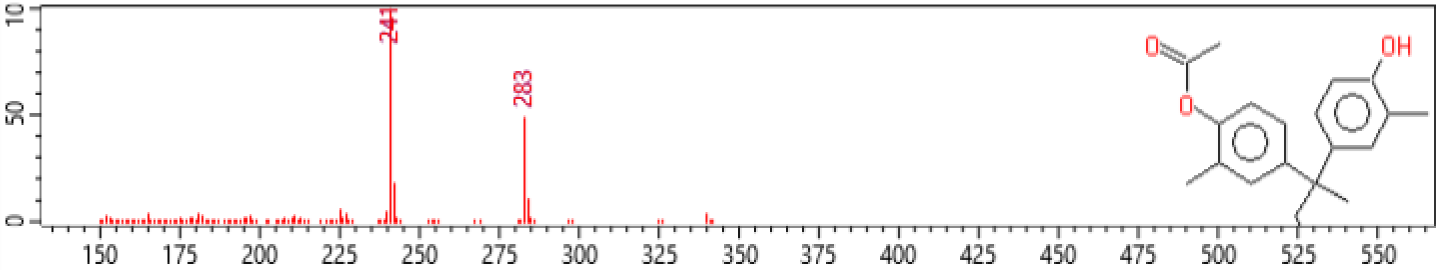


3,5-Ethano-3H-pyrrolo[2,3-d]carbazole-6-carboxylic acid, 1,2,3a,4,5,7-hexahydro-12-hydroxy-12-(1-hydroxyethyl)-, methyl ester

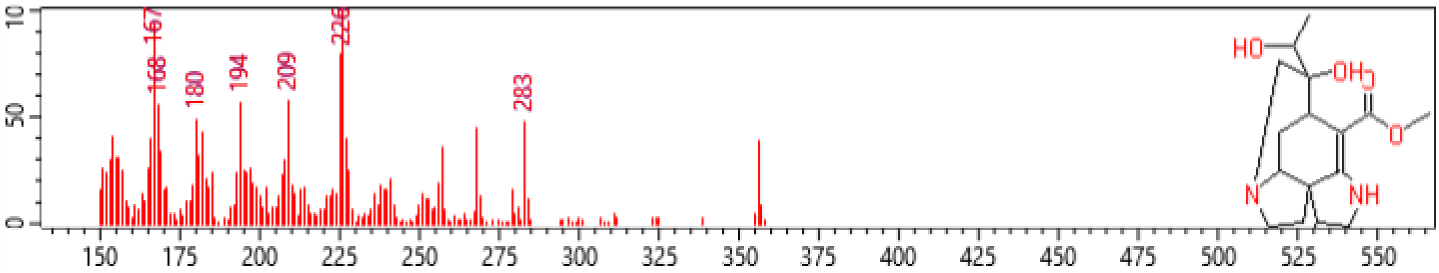

(5β,13β) Androst-8-en-3-one, 17-19-diacetoxy-4,4-dimethyl-

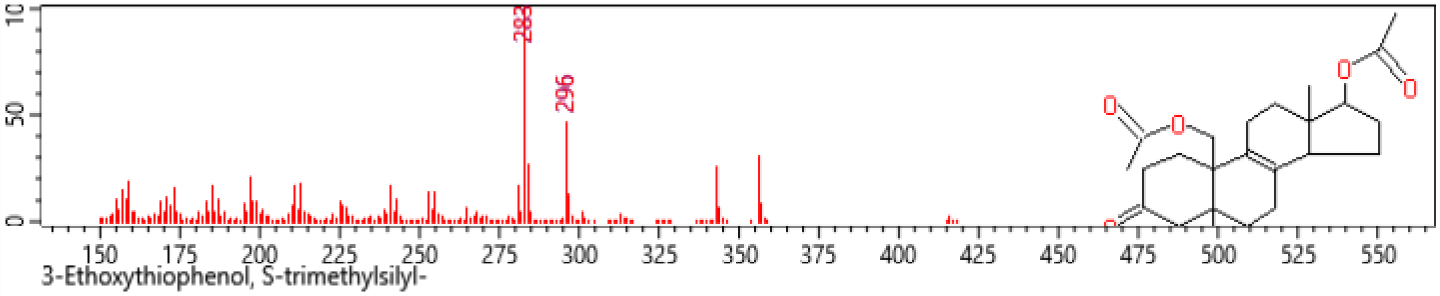


3-Ethoxythiophenol, S-trimethylsilyl-

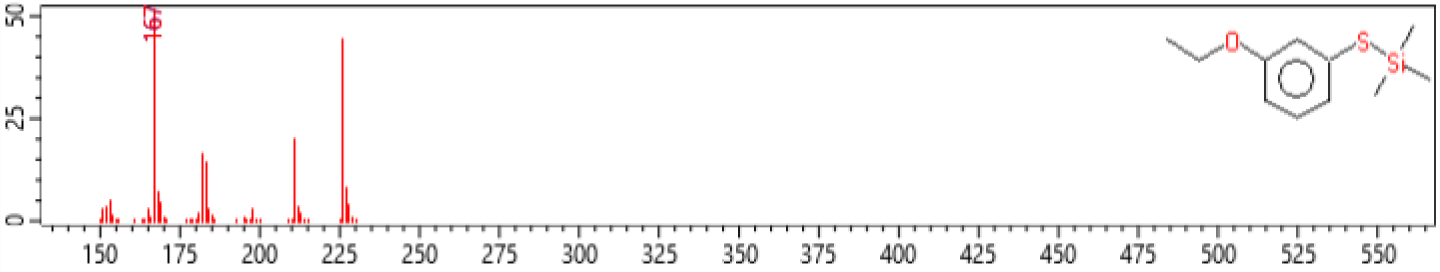


**N-[3-(1H-Imidazol-1-yl)propyl]-3-(trifluoromethyl)benzamide, TMS**

Peak: 12.33 - 12.34 min (scan #1486 - #1490) Subtracted noise: 12.29 - 12.31 min (scan #1476 - #1481), 12.35 - 12.37 min (scan #14

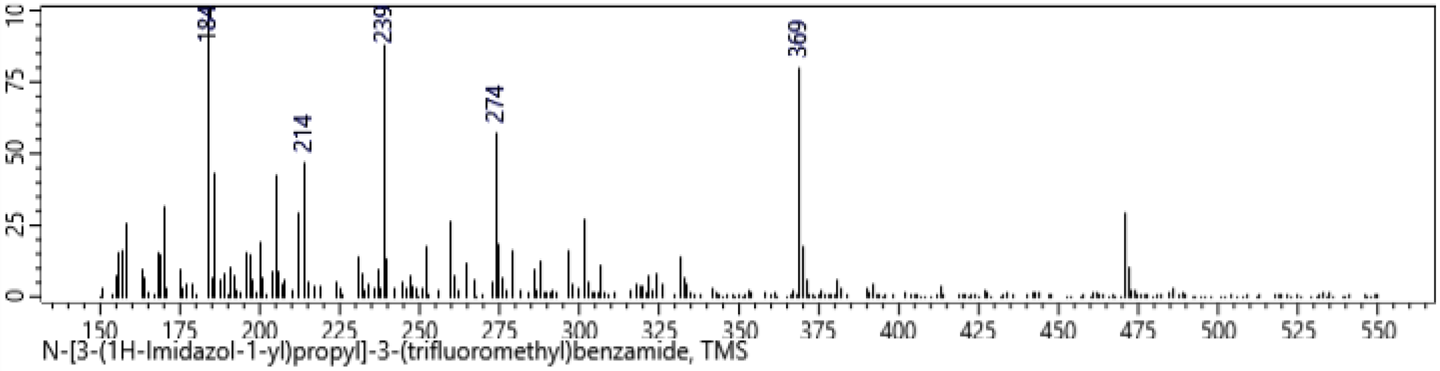


N-[3-(1H-Imidazol-1-yl)propyl]-3-(trifluoromethyl)benzamide, TMS

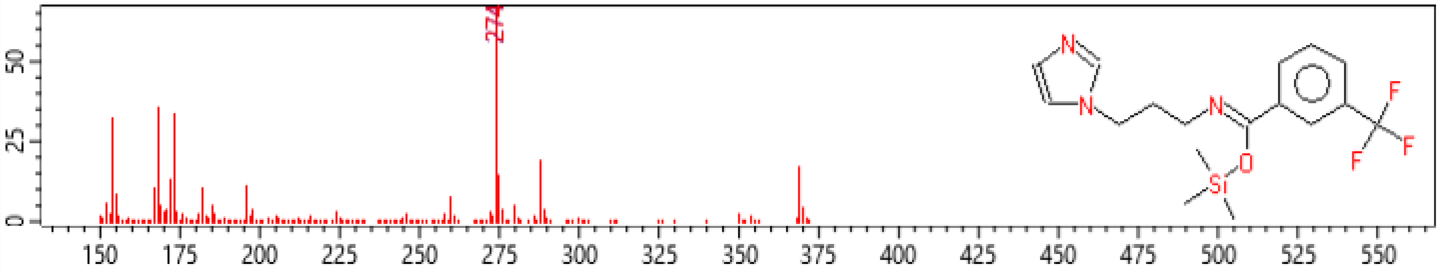


2-Heptadecanone, 1-[(2,2-dimethyl-1,3-dioxolan-4-yl)methoxy]-

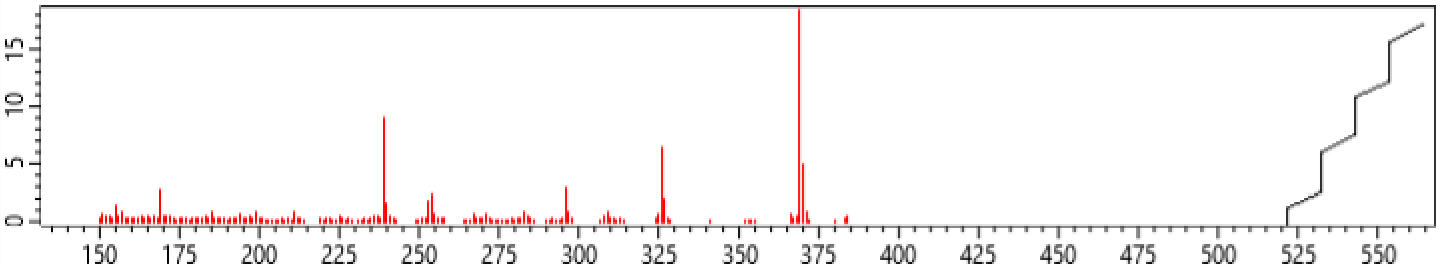

Corynan-16-carboxylic acid, 16,17-didehydro-17-methoxy-, methyl ester, (16E,20β)-

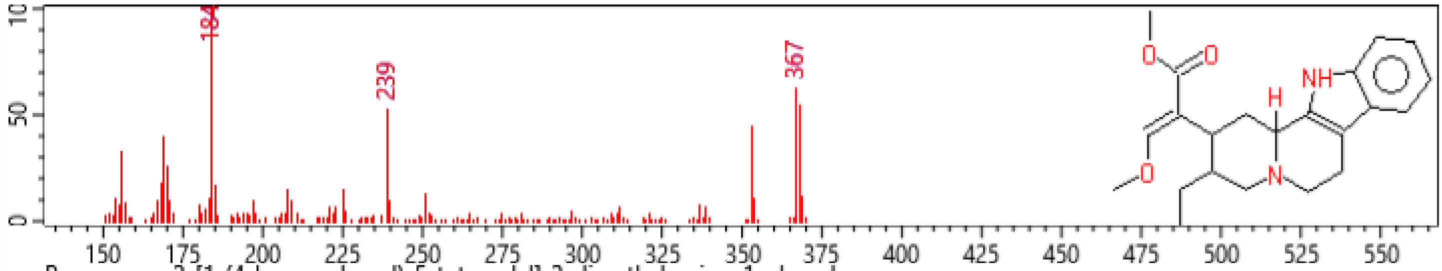


Propenone, 2-[1-(4-bromophenyl)-5-tetrazolyl]-3-dimethylamino-1-phenyl-

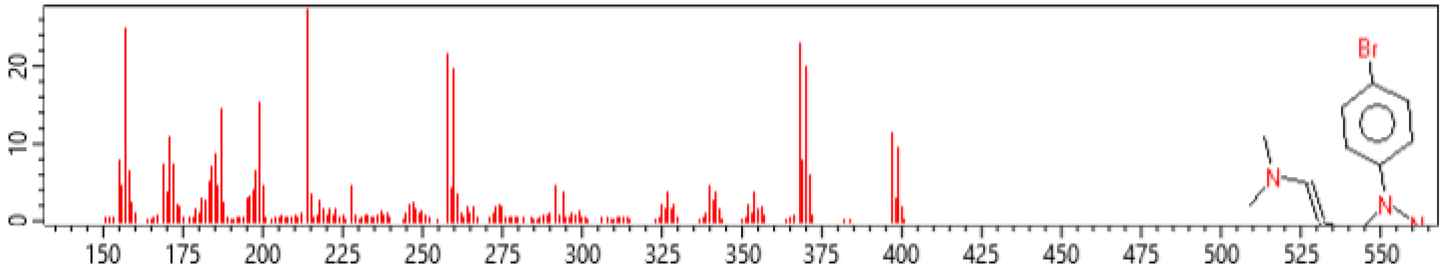

Thiazolo[3',2':1,2]pyrimido[5,4-b]indol-5(6H)-one, 9-fluoro-6-[(2-fluorophenyl)methyl]-2,3-dihydro-

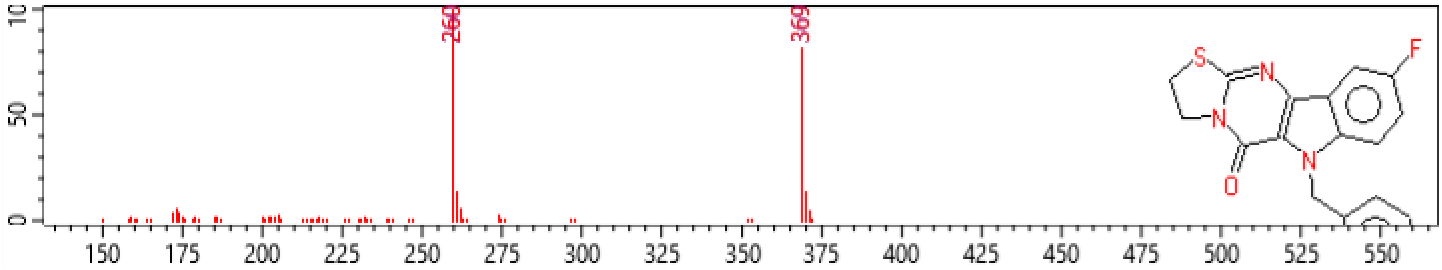


**Dodecanoic acid, TMS derivative**

Peak: 12.36 - 12.38 min (scan #1497 - #1501) Subtracted noise: 12.34 - 12.35 min (scan #1489 - #1493), 12.39 - 12.40 min (scan #15

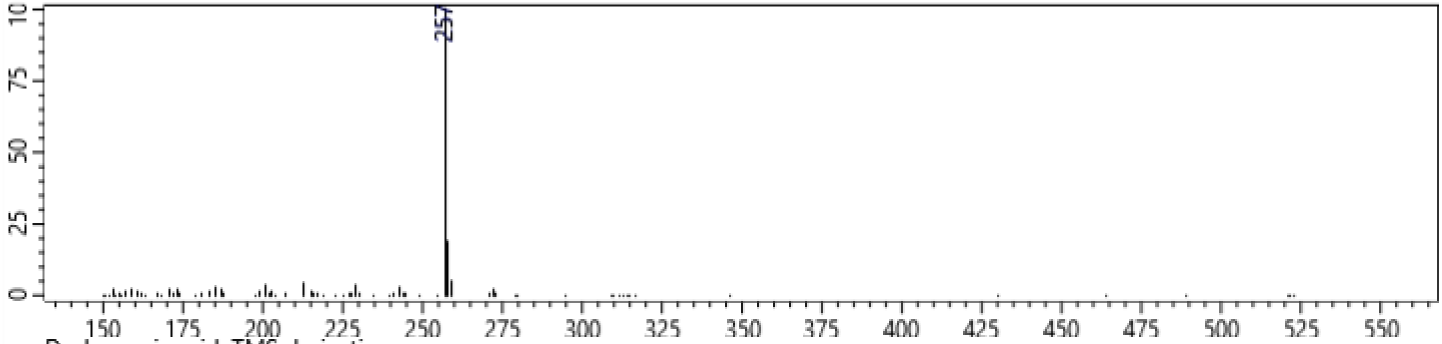


Dodecanoic acid, TMS derivative

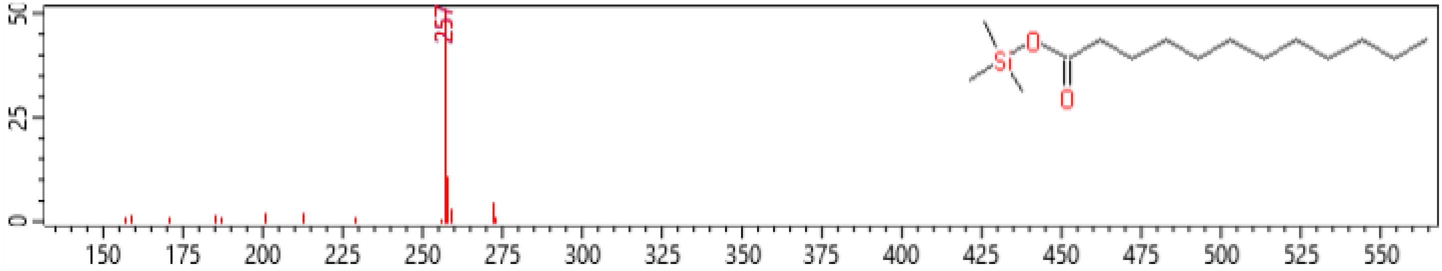

Dodecanoic acid, TMS derivative

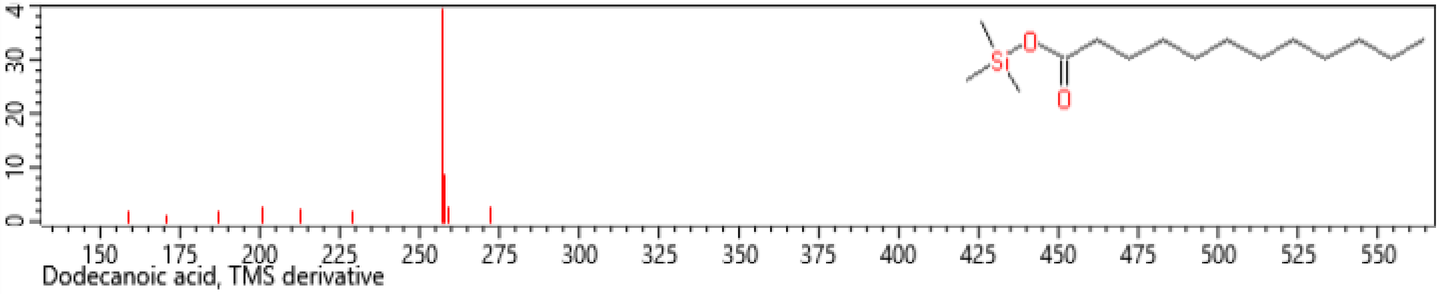


Dodecanoic acid, TMS derivative

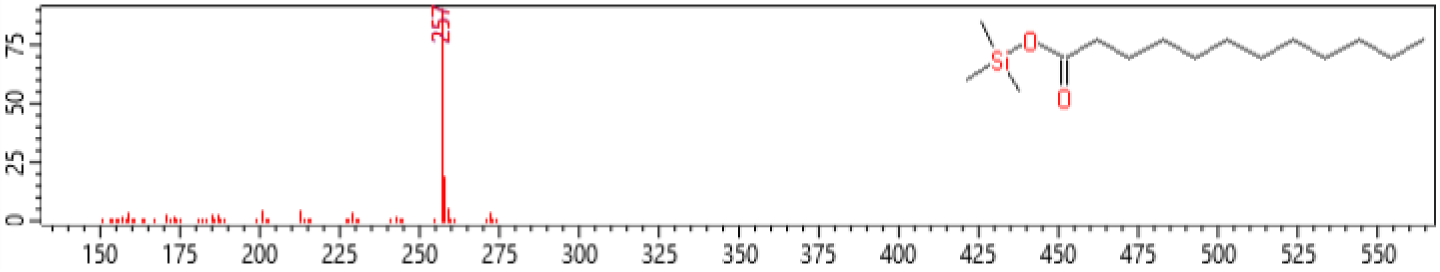


Dodecanoic acid, TMS derivative

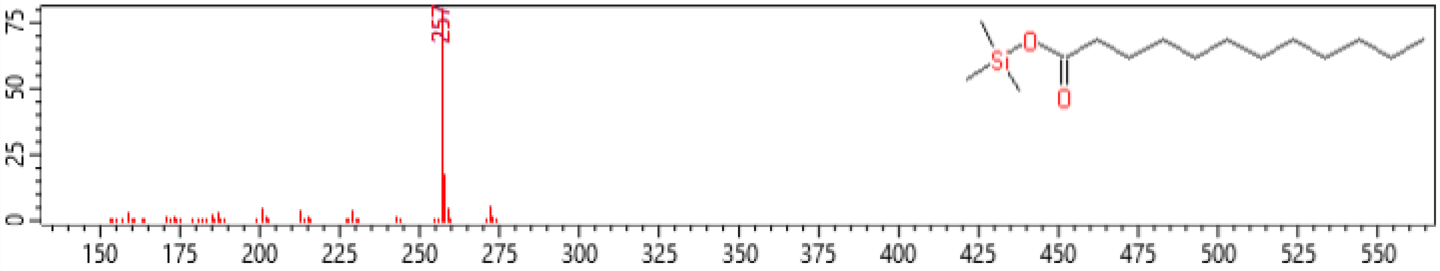


Silane, methylvinyl(nonyloxy)propoxy-

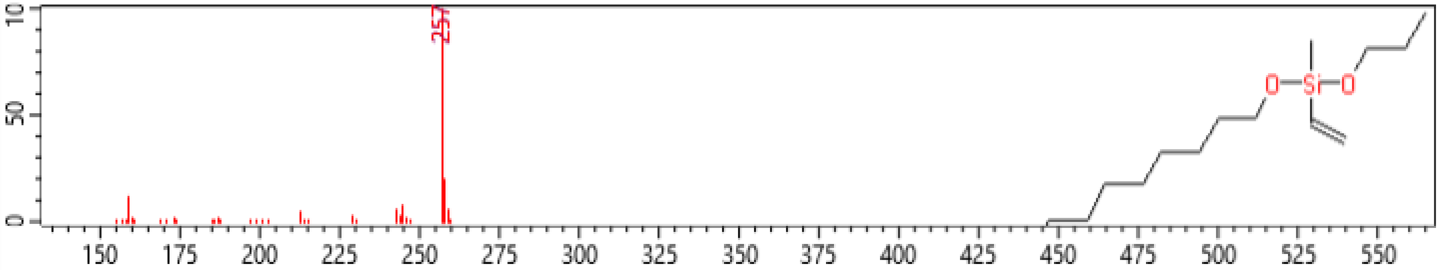


**D-erythro-2-Pentulose, 1,3,4-tris-O-(trimethylsilyl)-, 5-[bis(trimethylsilyl) phosphate]**

Peak: 12.44 - 12.45 min (scan #1518 - #1520) Subtracted noise: 12.42 - 12.43 min (scan #1512 - #1514), 12.46 - 12.47 min (scan #15

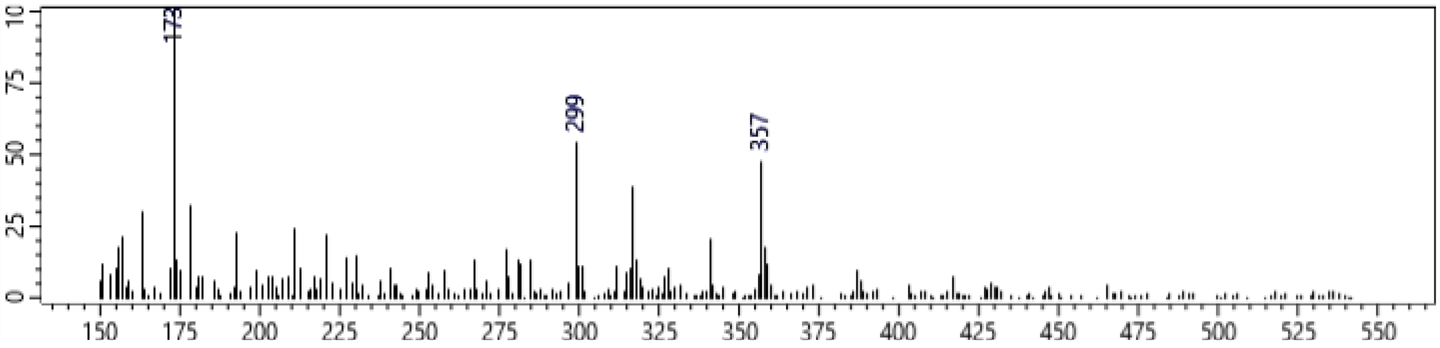

D-erythro-2-Pentulose, 1,3,4-tris-O-(trimethylsilyl)-, 5-[bis(trimethylsilyl) phosphate]

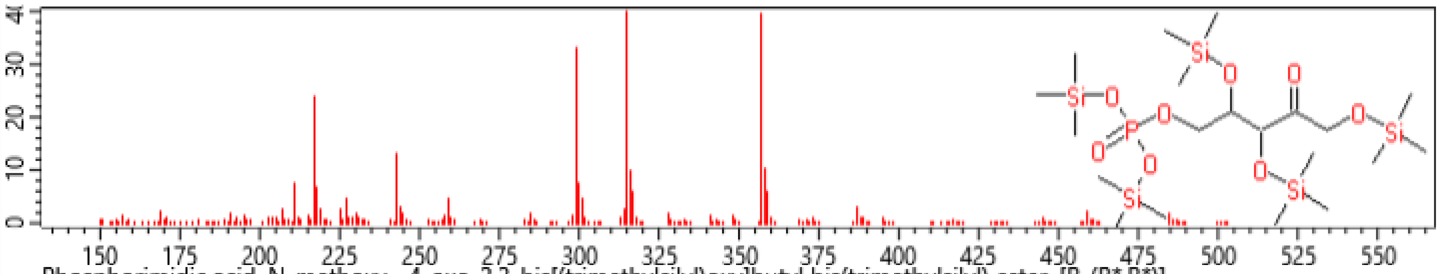

Phosphorimidic acid, N-methoxy-, 4-oxo-2,3-bis[(trimethylsilyl)oxy]butyl bis(trimethylsilyl) ester, [R-(R*,R*)]-

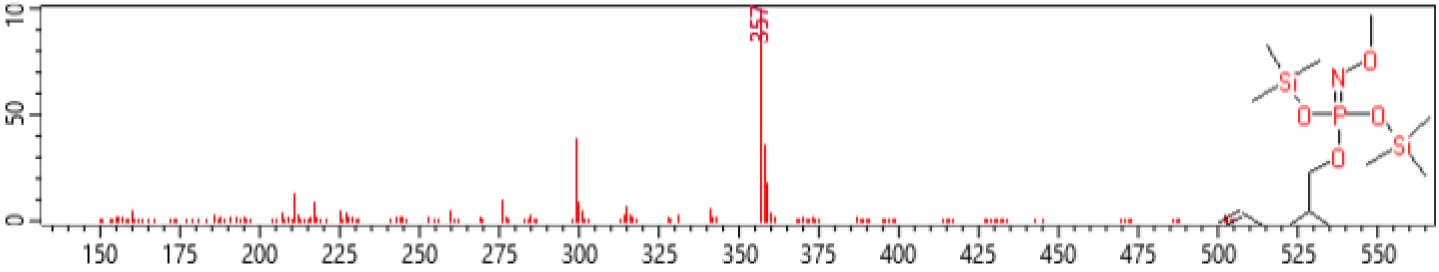


Phosphoric acid, 4-oxo-2,3-bis[(trimethylsilyl)oxy]butyl bis(trimethylsilyl) ester, [R-(R*,R*)]-

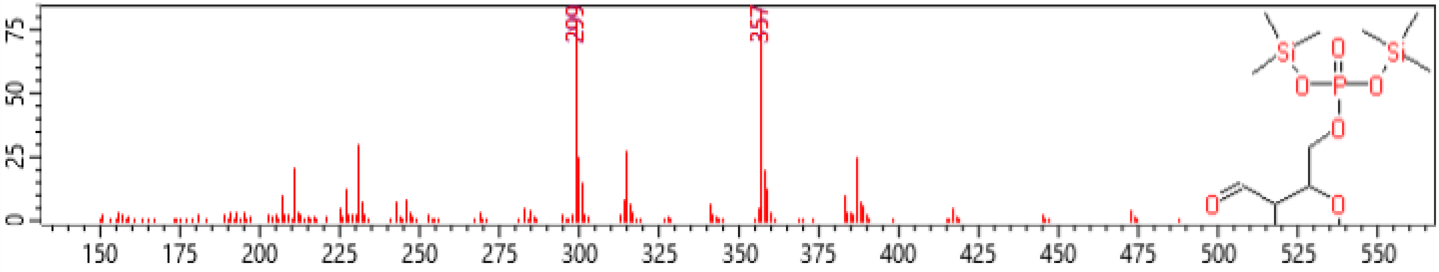


D-Erythro-Pentose, 2-deoxy-3,4-bis-O-(trimethylsilyl)-, O-methyloxime, 5-[bis(trimethylsilyl) phosphate]

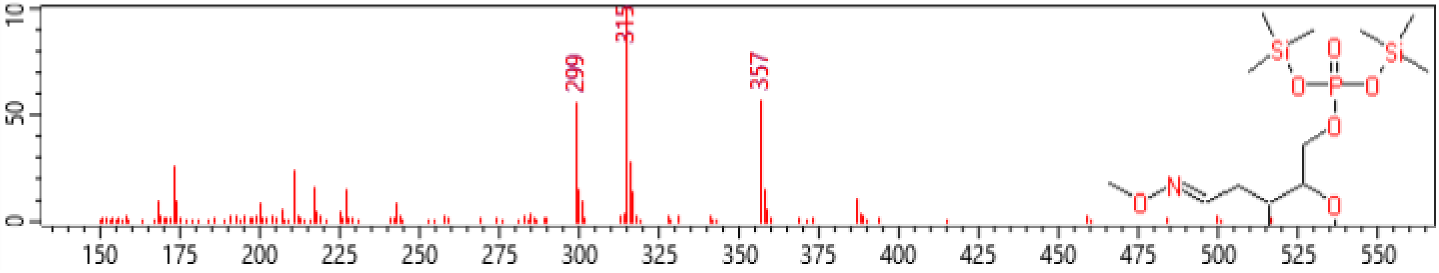


D-Erythro-Pentose, 2-deoxy-3,4-bis-O-(trimethylsilyl)-, 5-[bis(trimethylsilyl) methoxyphosphorimidate]

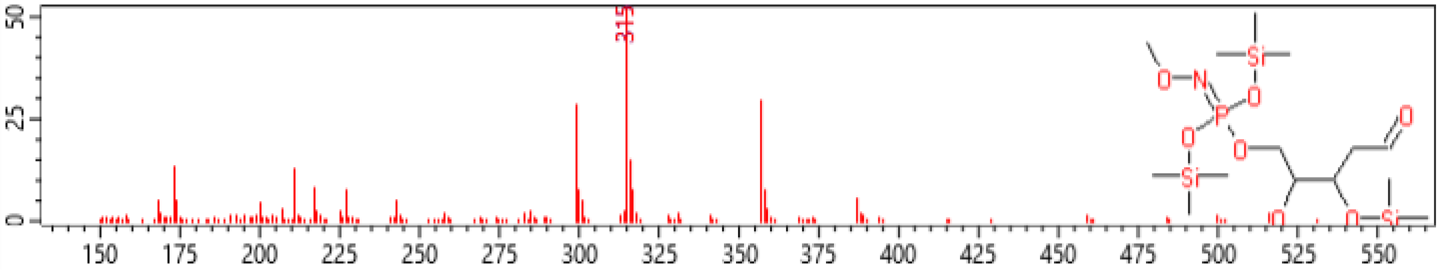

**5-Thiazolidinone, 4-(2-furanylmethylene)-3-phenyl-**

Peak: 12.49 - 12.53 min (scan #1532 - #1542) Subtracted noise: 12.43 - 12.46 min (scan #1514 - #1525), 12.54 - 12.58 min (scan #15

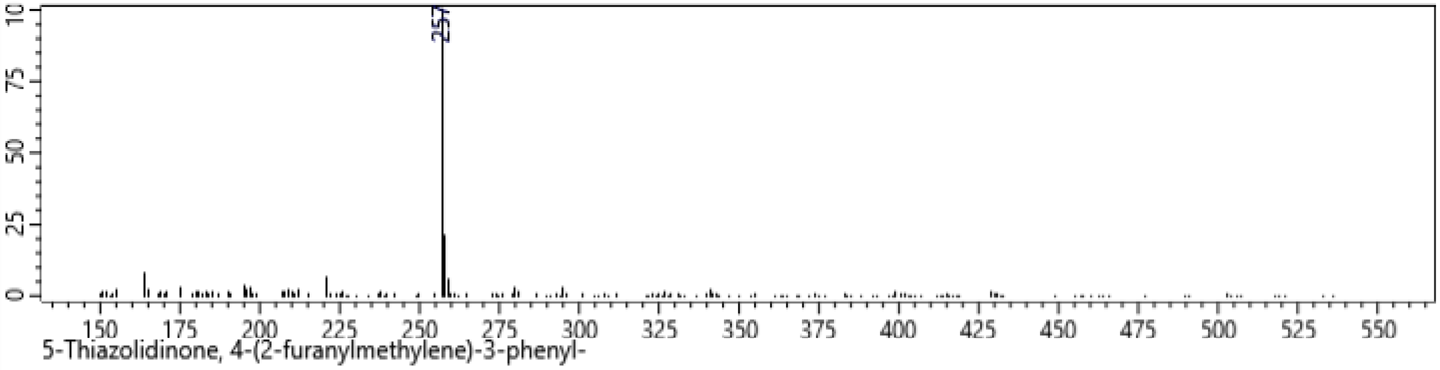


5-Thiazolidinone, 4-(2-furanylmethylene)-3-phenyl-

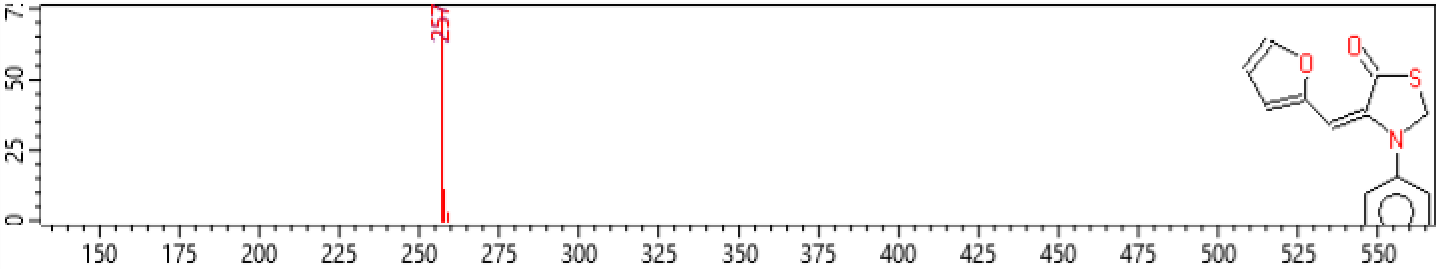


1-Dichloromethyl(dimethyl)silyloxytridecane

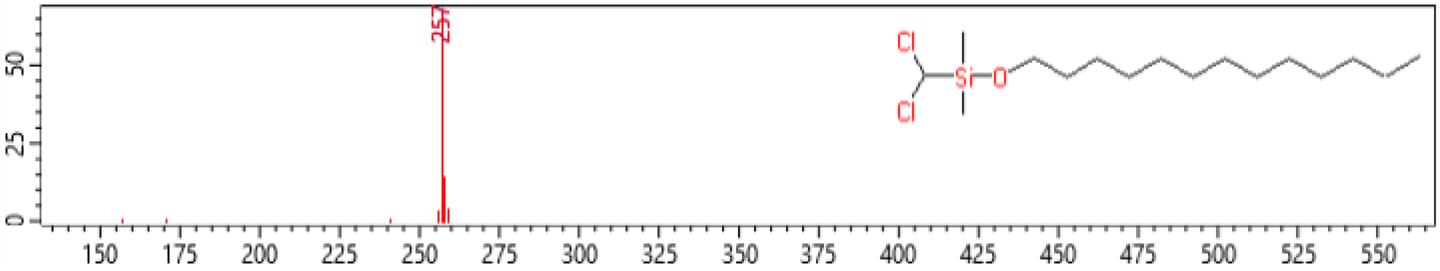


Acetamide, N-methyl-N-phenyl-2-phenylsulfanyl-

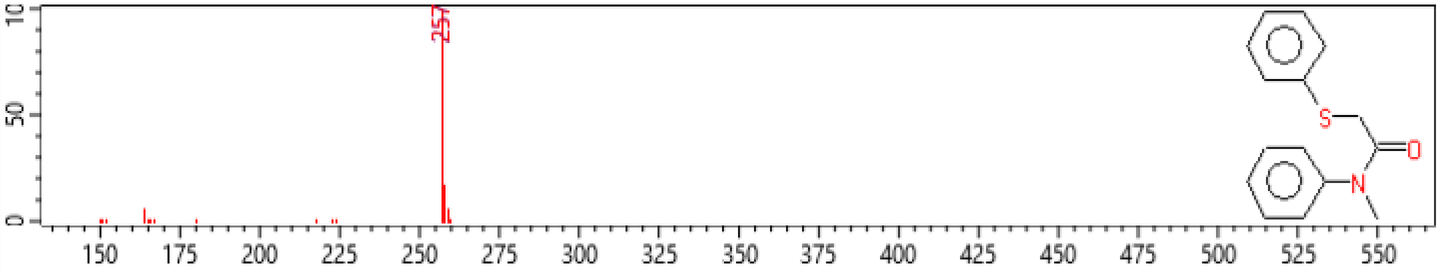


1-Dimethyl(prop-2-enyl)silyloxytridecane

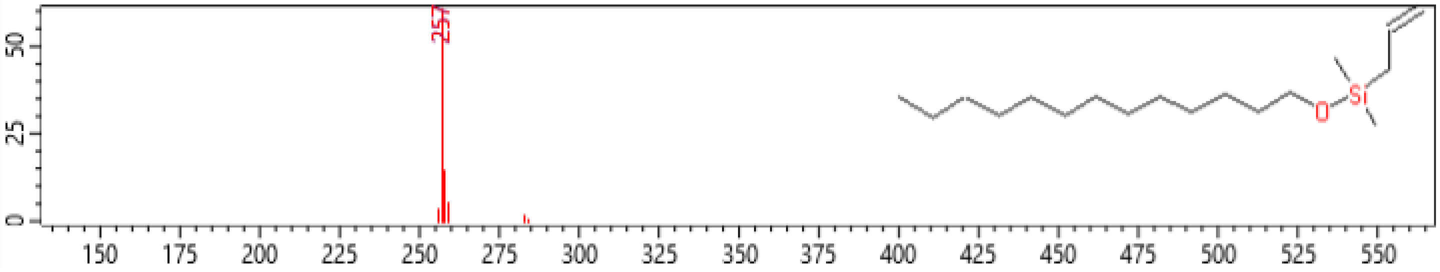

Dimethyldodecylsilyloxyethane

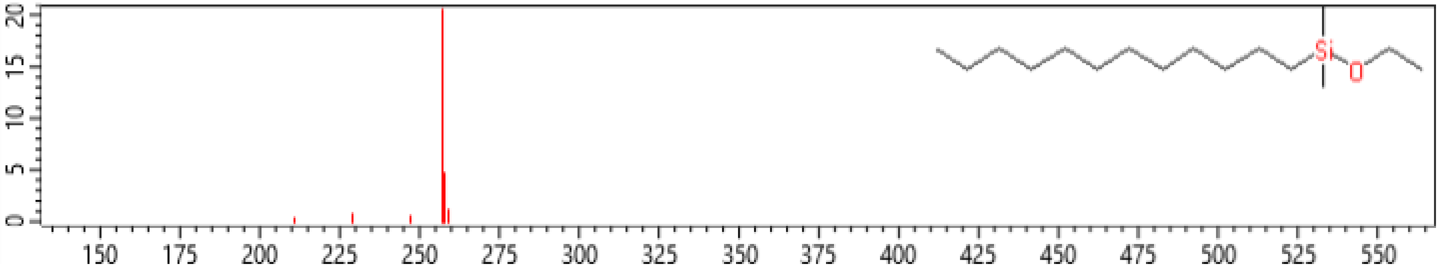

**1H-Benzimidazole, 2-(2-methylpropyl)-**

Peak: 12.59 - 12.65 min (scan #1561 - #1577) Subtracted noise: 12.49 - 12.54 min (scan #1531 - #1546), 12.67 - 12.72 min (scan #15[illegible]

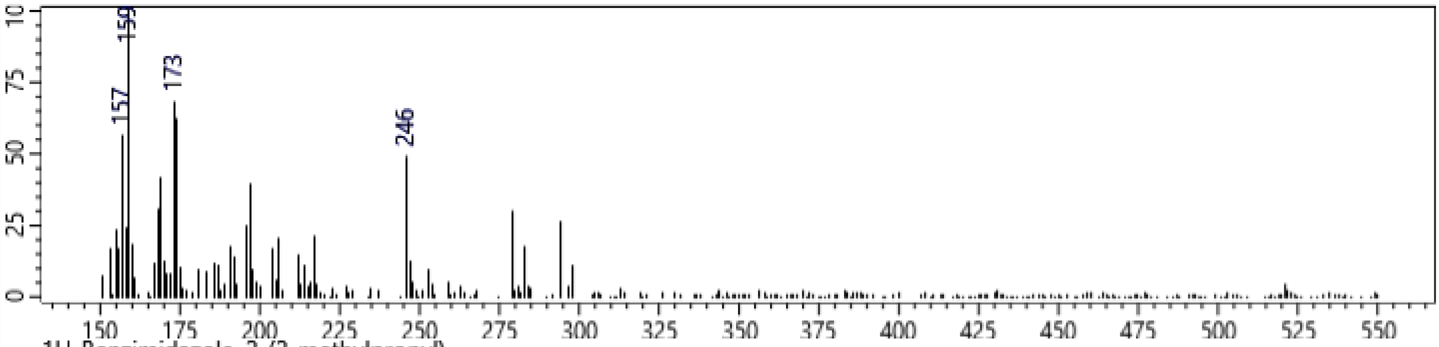


1H-Benzimidazole, 2-(2-methylpropyl)-

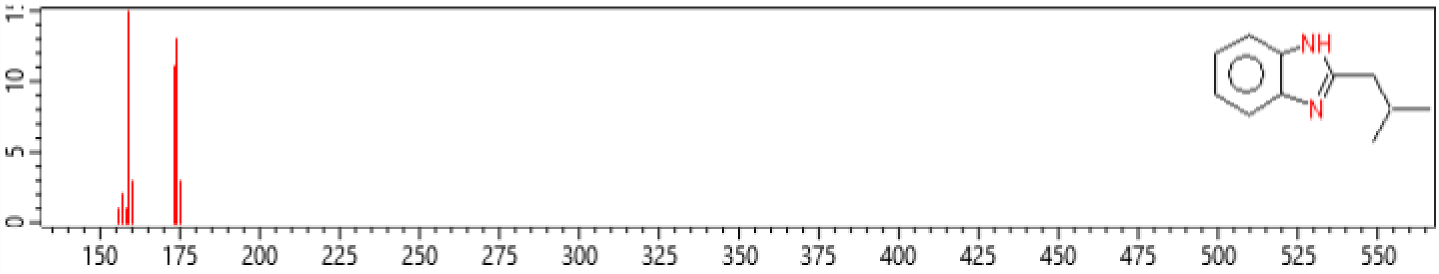

Acetic acid, 17-(1-acetoxy-ethyl)-10,13-dimethyl-3-oxo-2,3,8,9,10,11,12,13,14,15,16,17-dodecahydro-1H-cyclopenta[a]phenanthren-

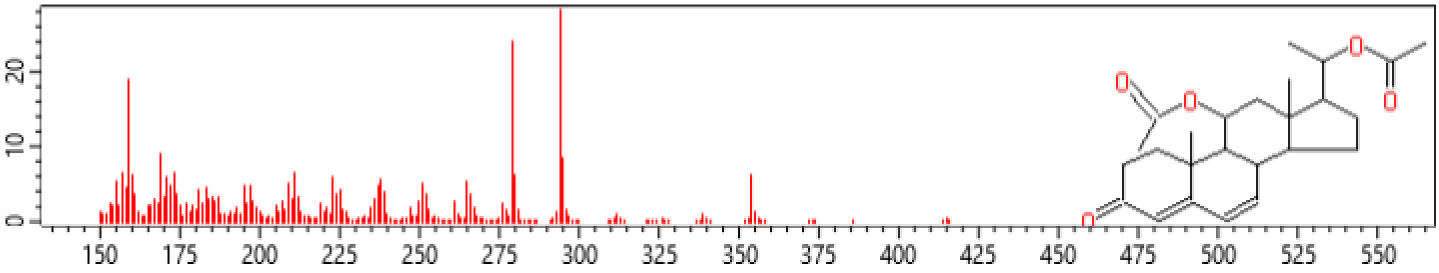

(3,8-Dihydroxy-6,9-dimethylidene-2-oxo-3a,4,5,6a,7,8,9a,9b-octahydroazuleno[4,5-b]furan-3-yl)methyl acetate

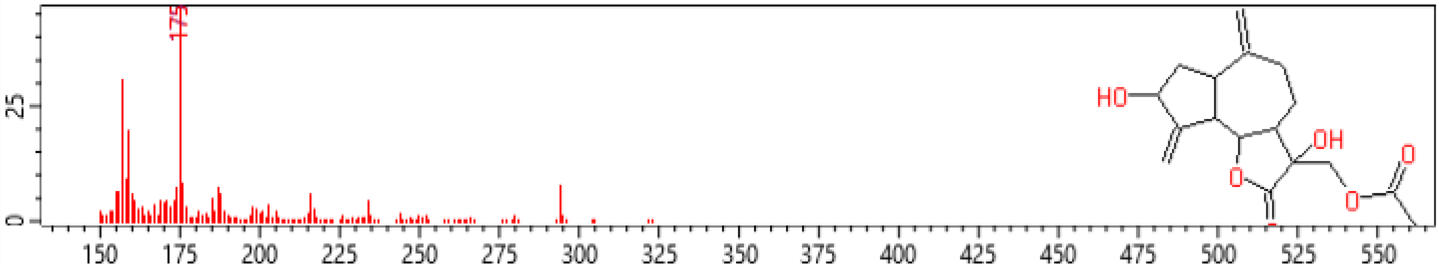

9a-(Acetyloxy)-7b-hydroxy-3-(hydroxymethyl)-1,1,6,8-tetramethyl-5-oxo-1a,1b,4,4a,5,7a,7b,8,9,9a-decahydro-1H-cyclopropa[3,4]ber

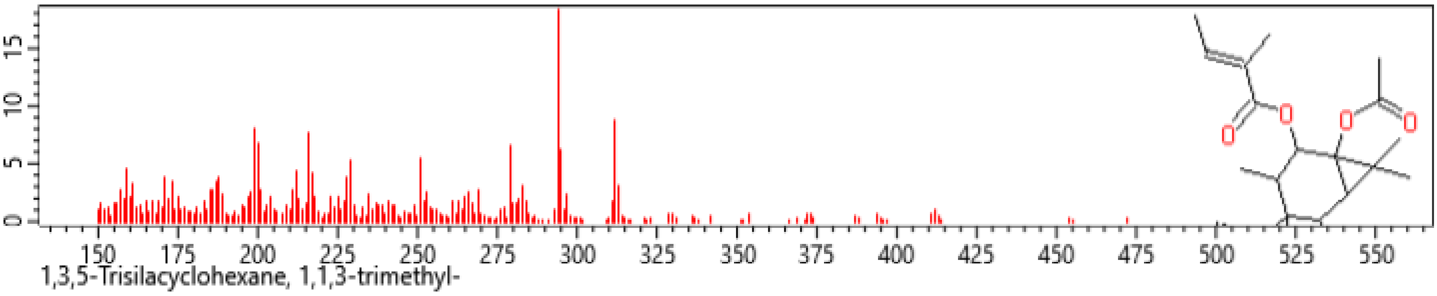


1,3,5-Trisilacyclohexane, 1,1,3-trimethyl-

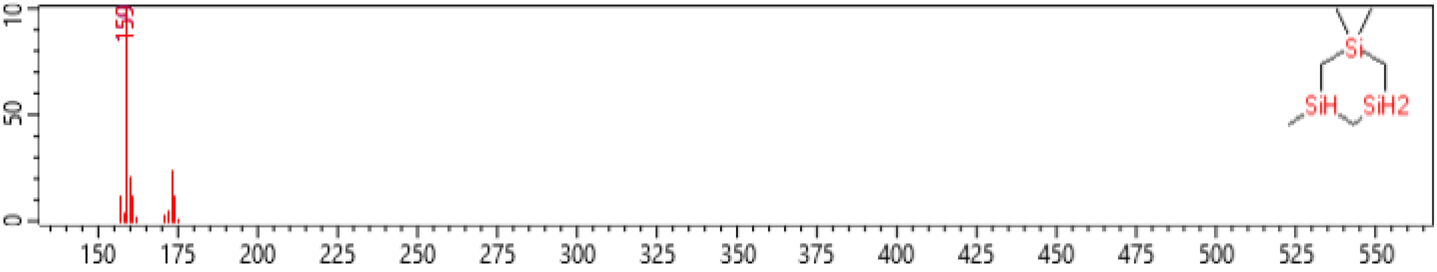


**1,7-di-iso-propylnaphthalene**

Peak: 12.69 - 12.72 min (scan #1587 - #1595) Subtracted noise: 12.64 - 12.66 min (scan #1573 - #1580), 12.73 - 12.76 min (scan #15

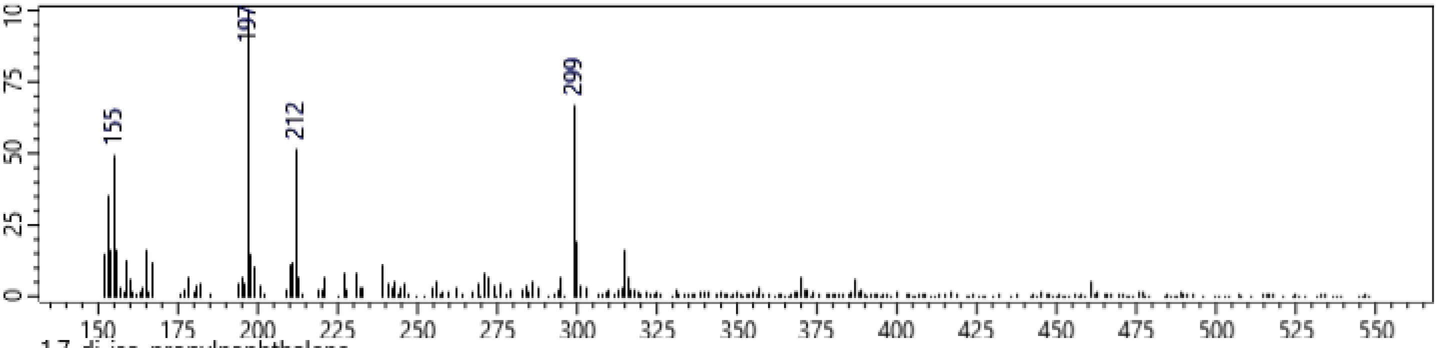


1,7-di-iso-propylnaphthalene

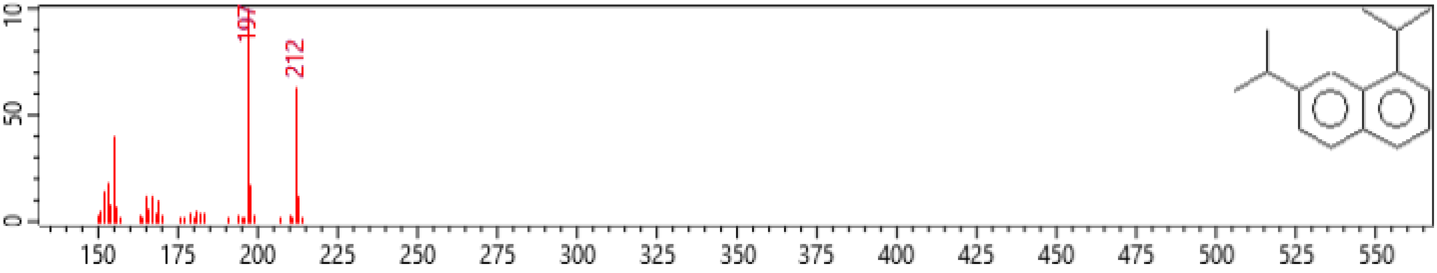


1,3-di-iso-propylnaphthalene

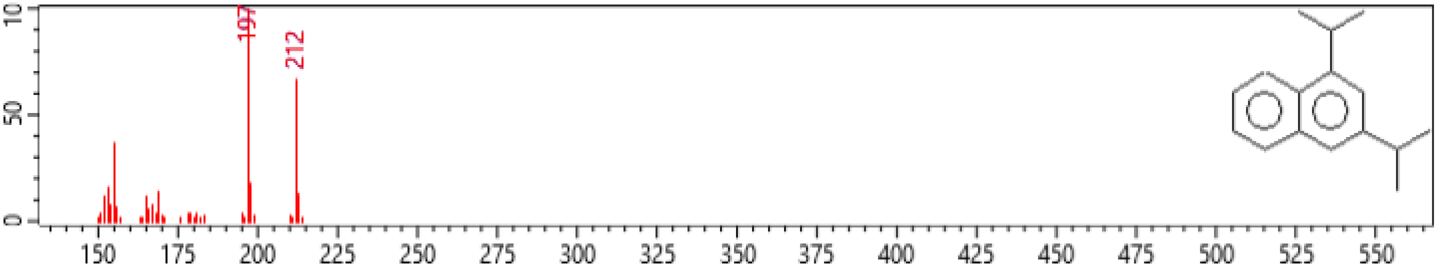

D-Arabino-Hexonic acid, 2-deoxy-3,5-bis-O-(trimethylsilyl)-, γ-lactone, bis(trimethylsilyl) phosphate

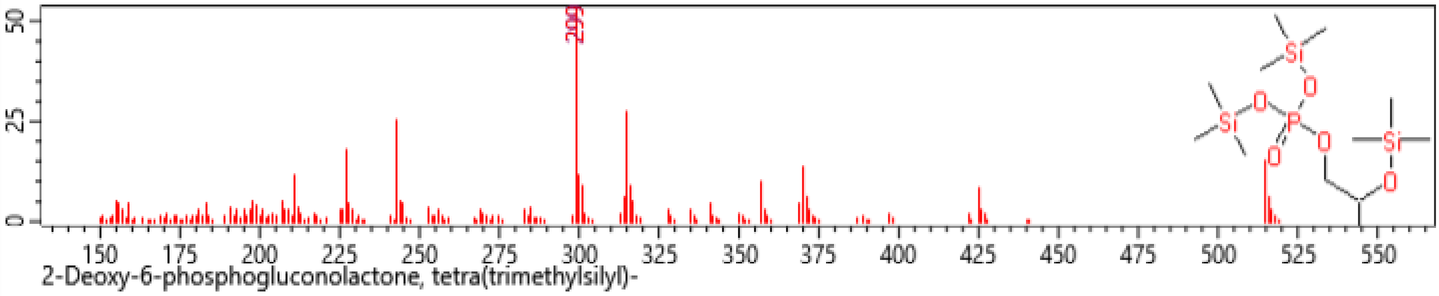


2-Deoxy-6-phosphogluconolactone, tetra(trimethylsilyl)-

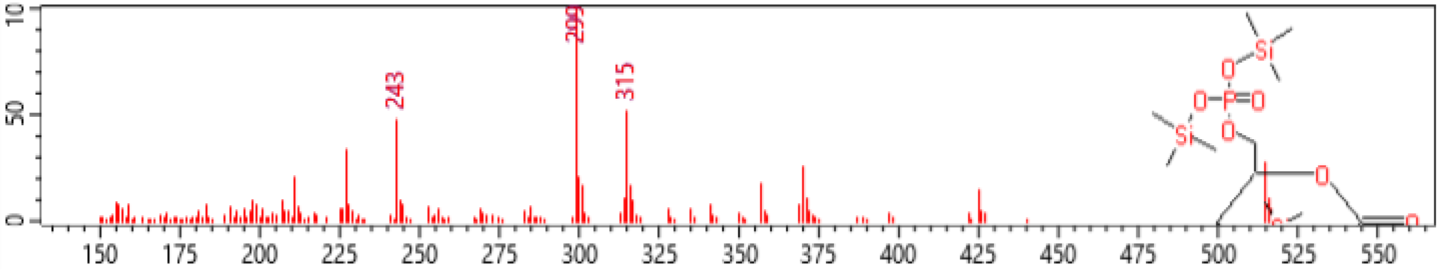


1,4-di-iso-propylnaphthalene

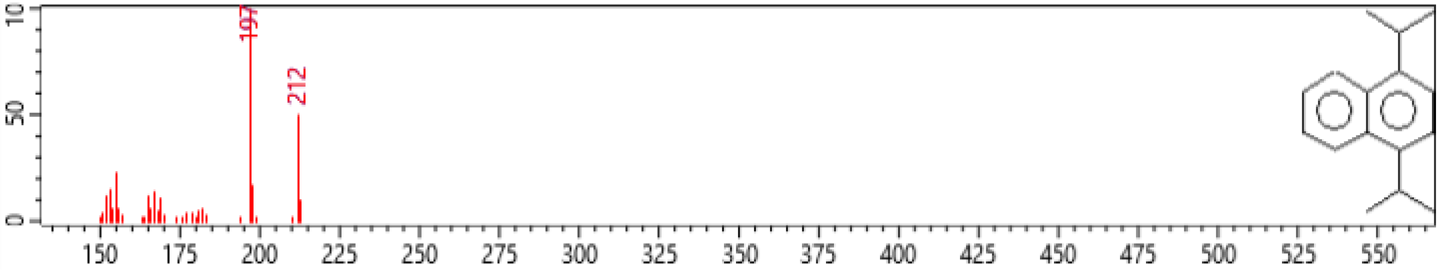


**Suberic acid, 2TMS derivative**

Peak: 12.74 - 12.76 min (scan #1602 - #1606) Subtracted noise: 12.71 - 12.72 min (scan #1593 - #1597), 12.77 - 12.78 min (scan #16

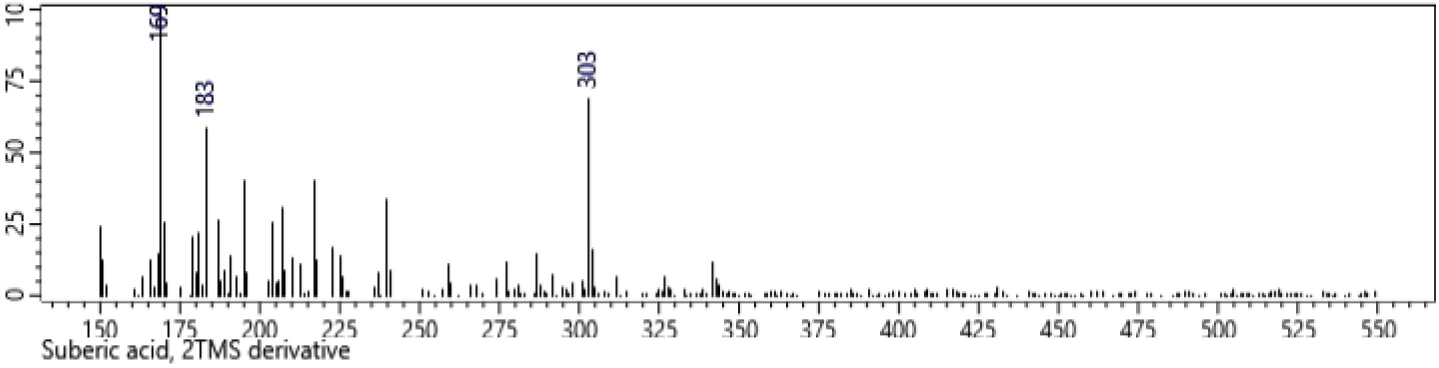


Suberic acid, 2TMS derivative

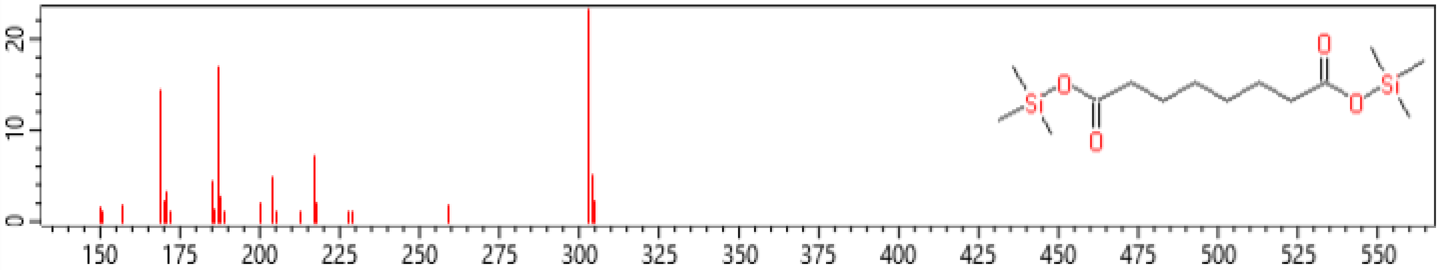

Suberic acid, 2TMS derivative

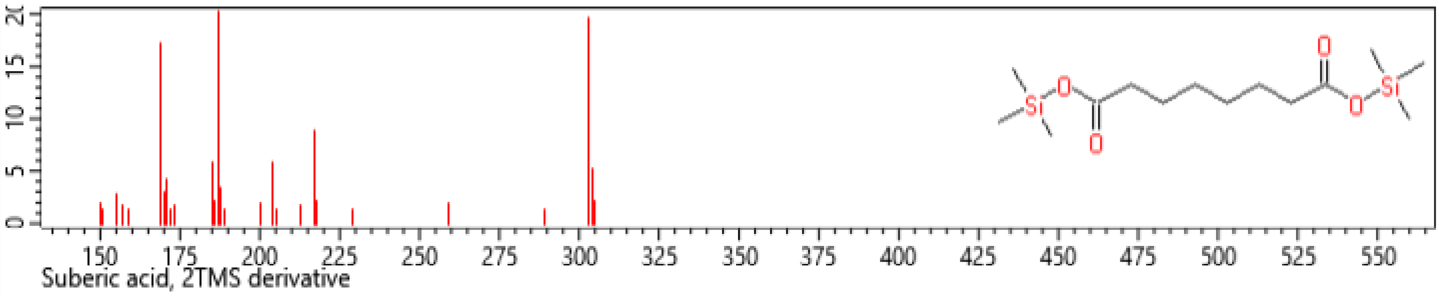

Suberic acid, 2TMS derivative

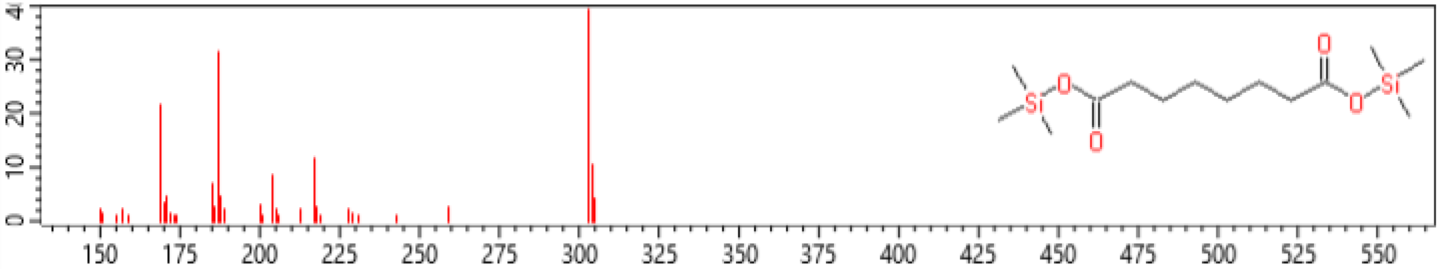

8-Hydroxy-8-(3-octyloxiran-2-yl)octanoic acid, 2TMS

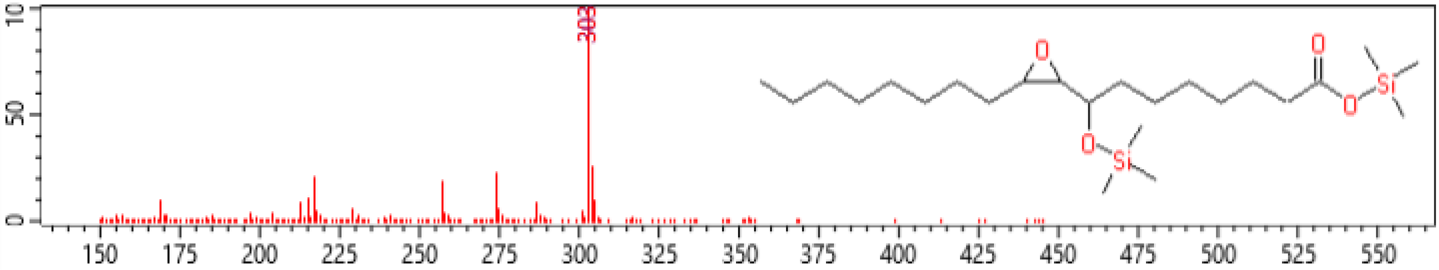


Heptanedioic acid, 4-methyl-, bis(trimethylsilyl) ester

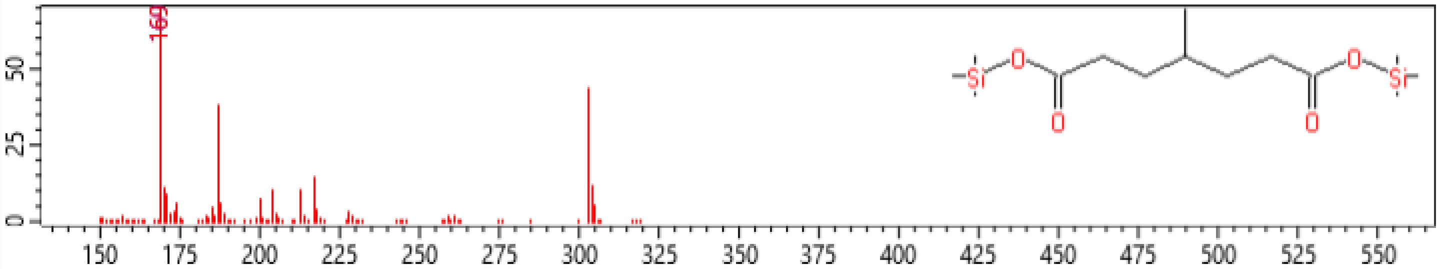


**Pyridine-3-carbonitrile, 4-methyl-6-methoxy-2-(2-thienyl)-**

Peak: 12.77 - 12.79 min (scan #1611 - #1617) Subtracted noise: 12.74 - 12.76 min (scan #1603 - #1608), 12.82 - 12.84 min (scan #16[illegible]

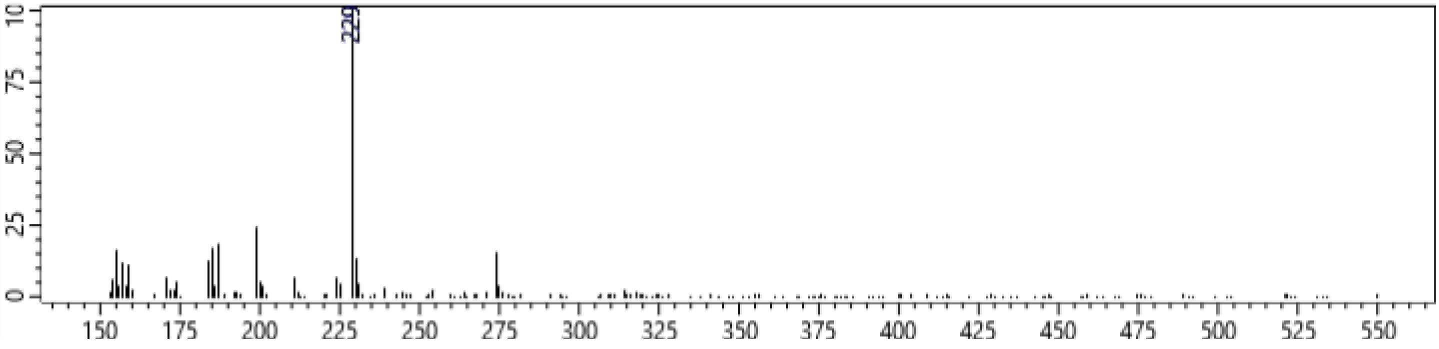

Pyridine-3-carbonitrile, 4-methyl-6-methoxy-2-(2-thienyl)-

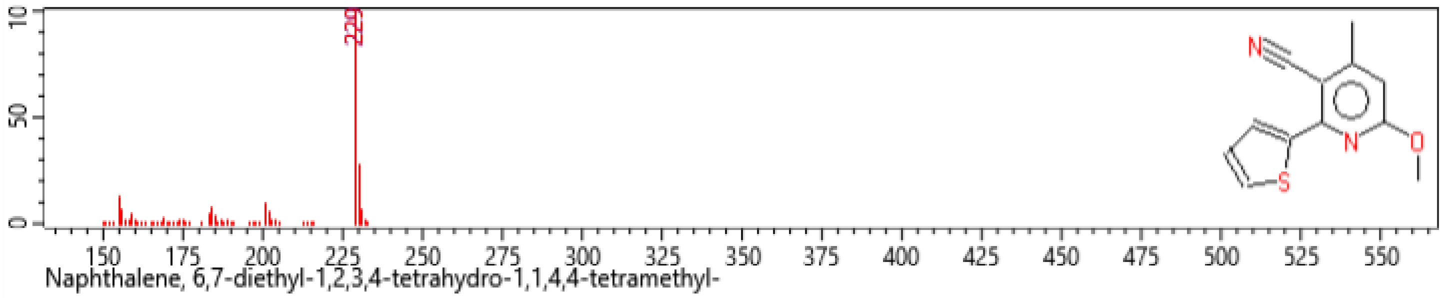


Naphthalene, 6,7-diethyl-1,2,3,4-tetrahydro-1,1,4,4-tetramethyl-

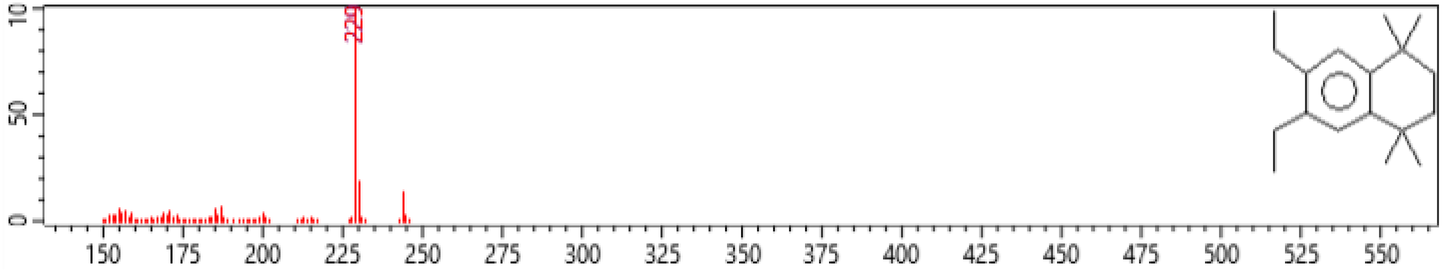


Silane, methylvinyl(3,3-dimethylbut-2-yloxy)heptyloxy-

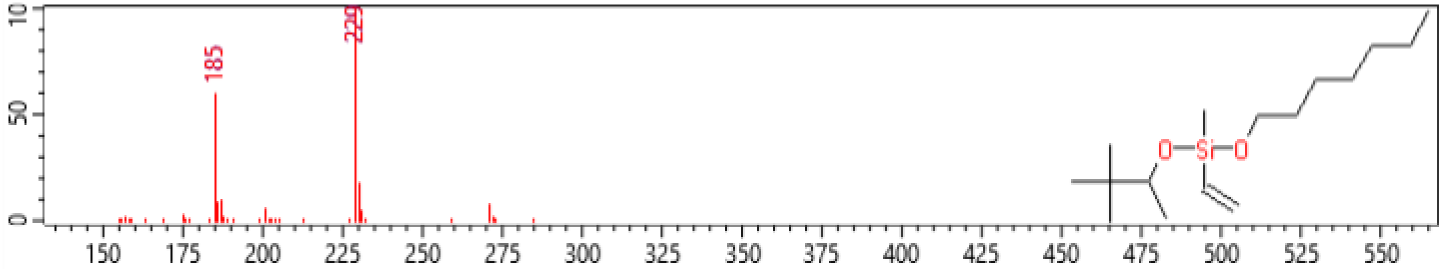


Dibenzothiophene, 2-nitro-

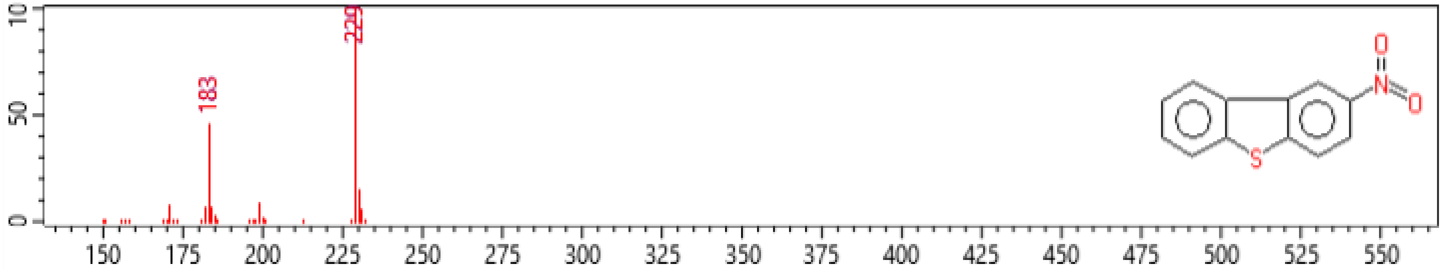


Silane, methylvinyl(4-methylpent-2-yloxy)heptyloxy-

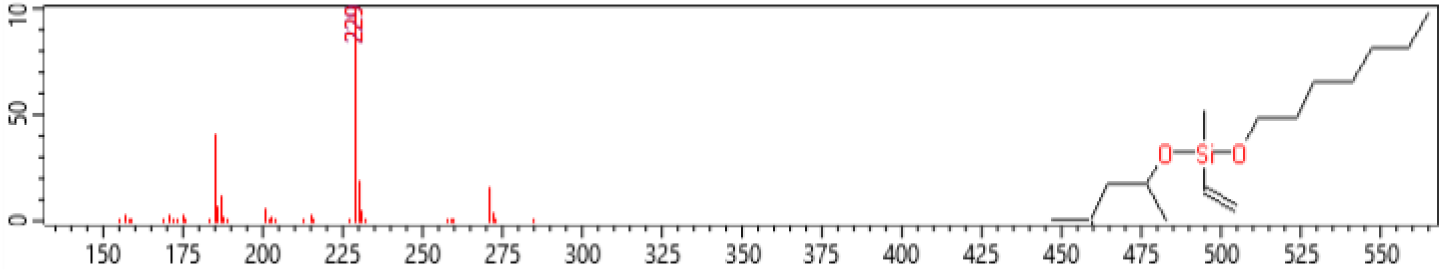

**Homovanillyl alcohol, 2TMS derivative**

Peak: 12.84 - 12.88 min (scan #1630 - #1640) Subtracted noise: 12.78 - 12.82 min (scan #1612 - #1623), 12.90 - 12.94 min (scan #16

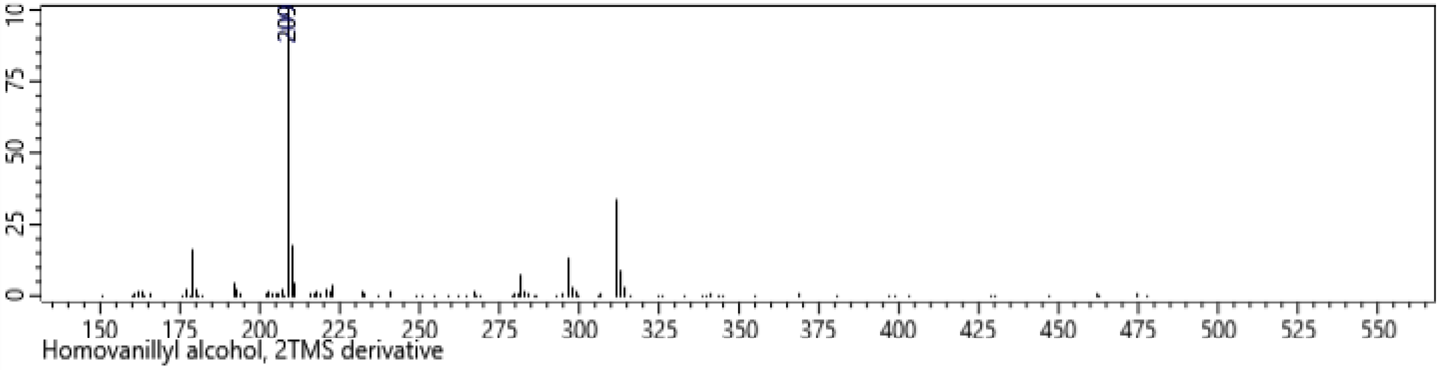


Homovanillyl alcohol, 2TMS derivative

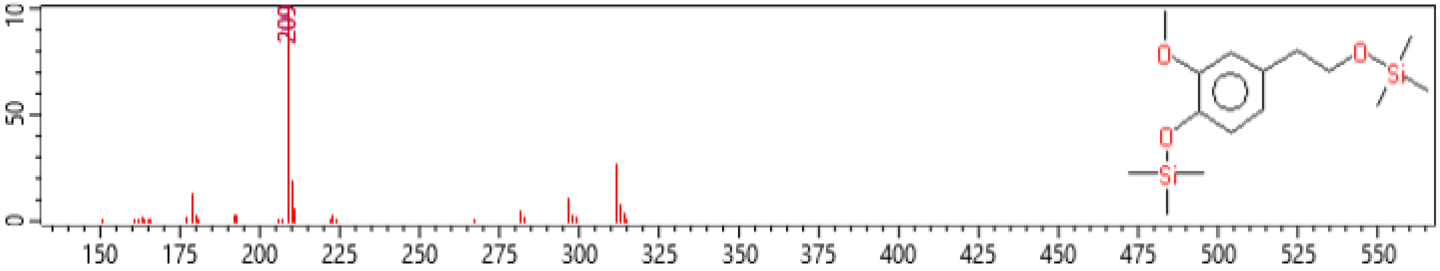


Homovanillyl alcohol, 2TMS derivative

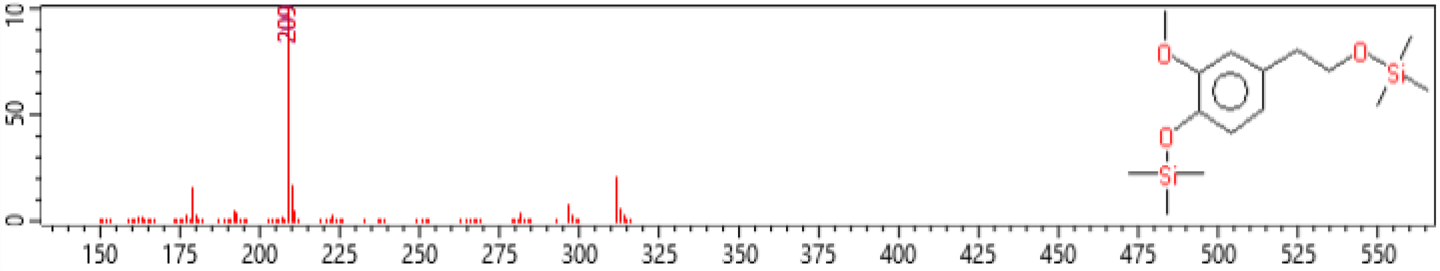


Homovanillyl alcohol, 2TMS derivative

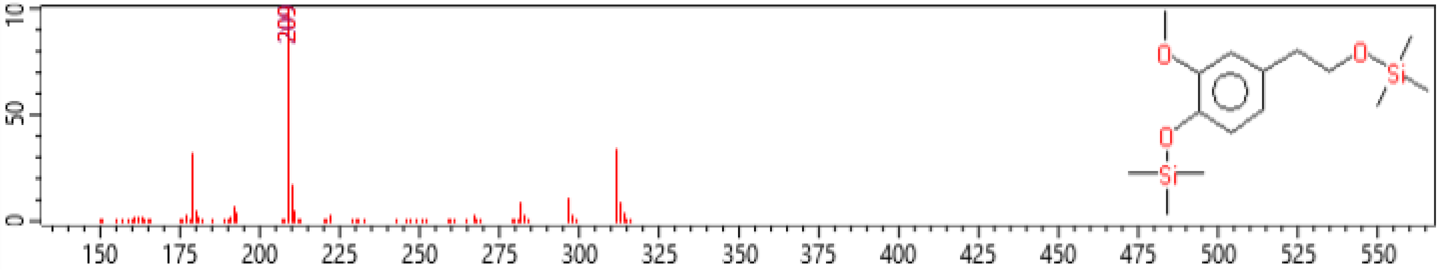


2-(3-Hydroxy-4-methoxyphenyl)ethanol bis(trimethylsilyl) ether

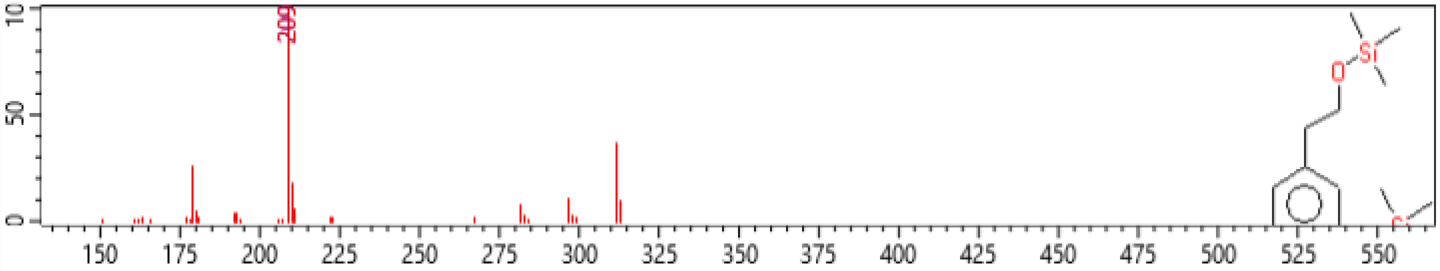

7,8-Dimethoxy-3-(4-methoxyphenyl)-4-oxo-4H-chromen-5-yl 2-O-pentopyranosylhexopyranoside

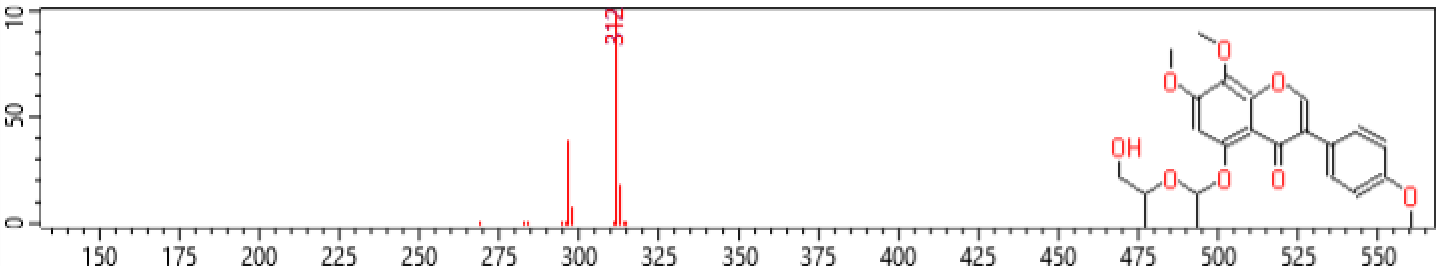


**Bisphenol F, acetate**

Peak: 12.90 - 12.93 min (scan #1647 - #1655) Subtracted noise: 12.87 - 12.89 min (scan #1638 - #1645), 12.96 - 12.99 min (scan #16

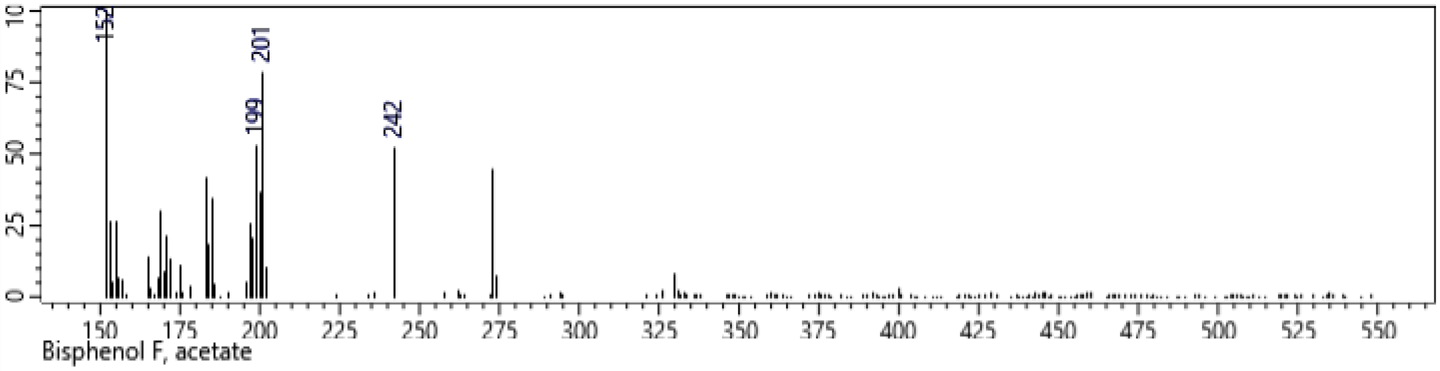


Bisphenol F, acetate

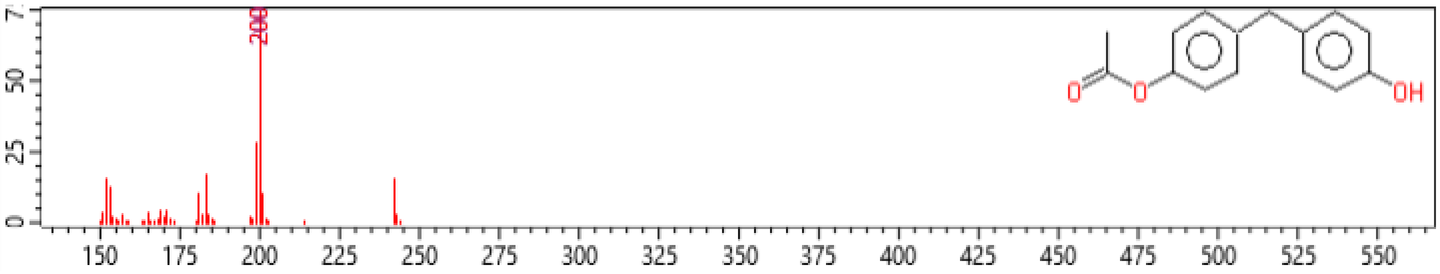


Phenol, 4,4'-methylenebis-

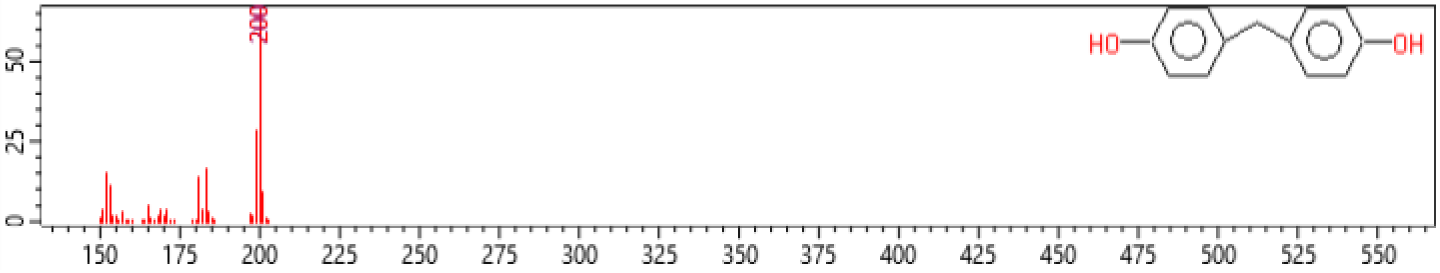


Bisphenol F, diacetate

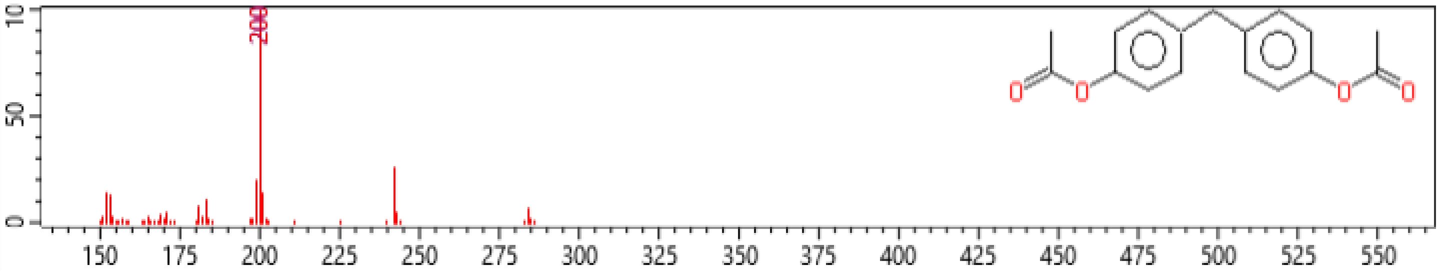

2,4'-Dihydroxydiphenylmethane, diacetate

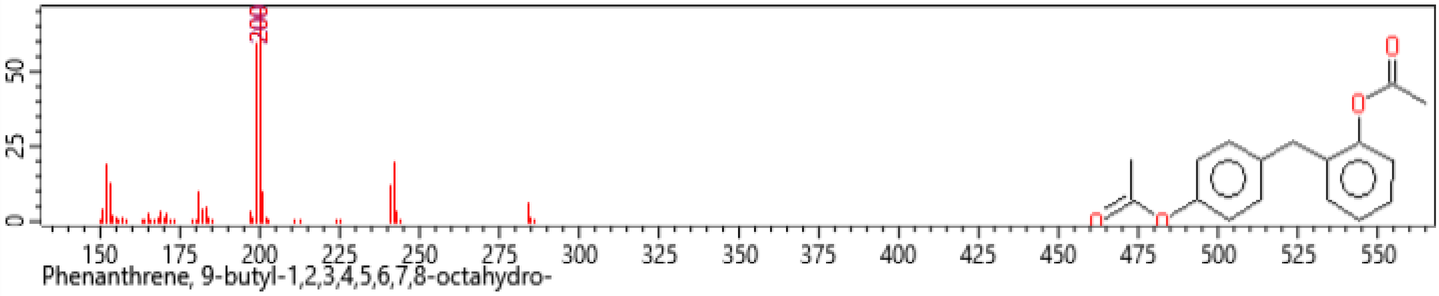


Phenanthrene, 9-butyl-1,2,3,4,5,6,7,8-octahydro-

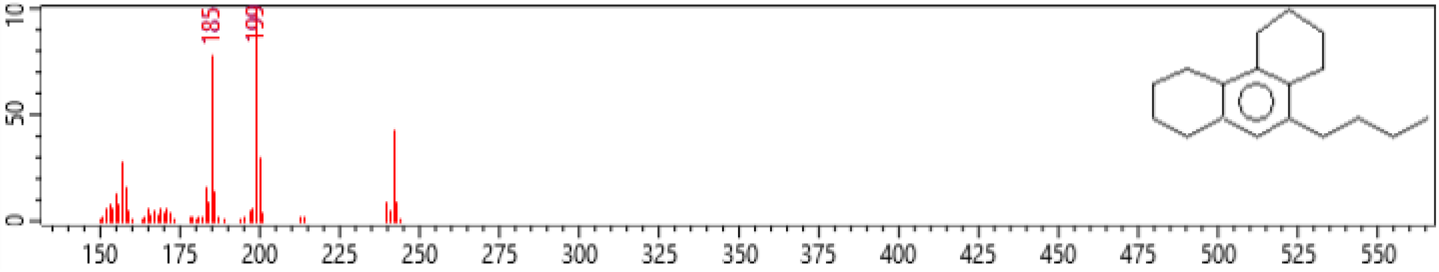


**Phosphoric acid, 2-(trimethylsiloxy)-1-[(trimethylsiloxy)methyl]ethyl bis(trimethylsilyl) ester**

Peak: 12.96 - 13.01 min (scan #1663 - #1677) Subtracted noise: 12.91 - 12.96 min (scan #1649 - #1663), 13.07 - 13.12 min (scan #16

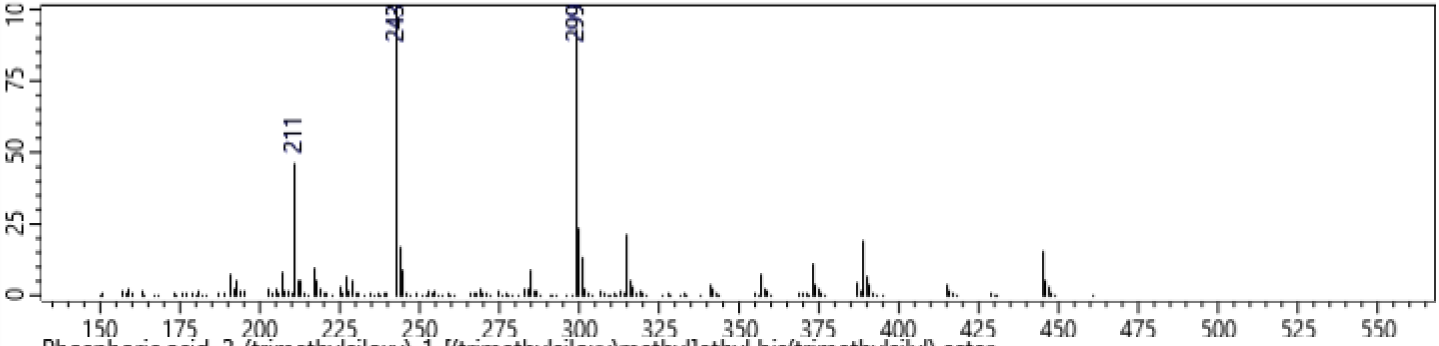


Phosphoric acid, 2-(trimethylsiloxy)-1-[(trimethylsiloxy)methyl]ethyl bis(trimethylsilyl) ester

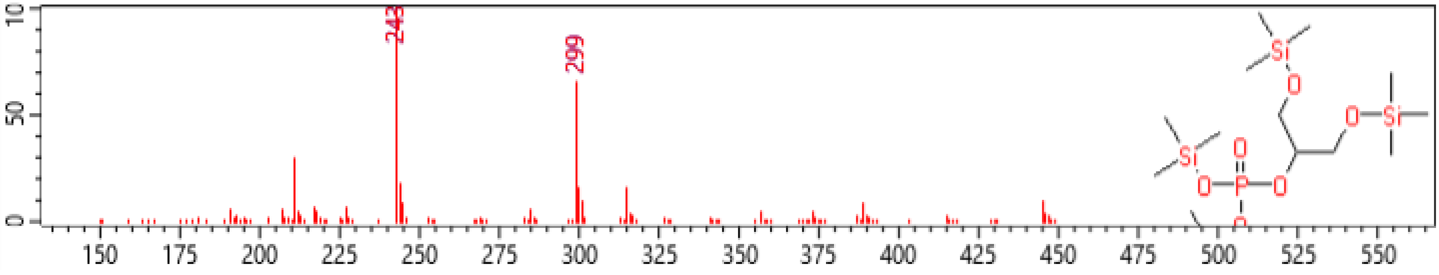


Phosphoric acid, 2-(trimethylsiloxy)-1-[(trimethylsiloxy)methyl]ethyl bis(trimethylsilyl) ester

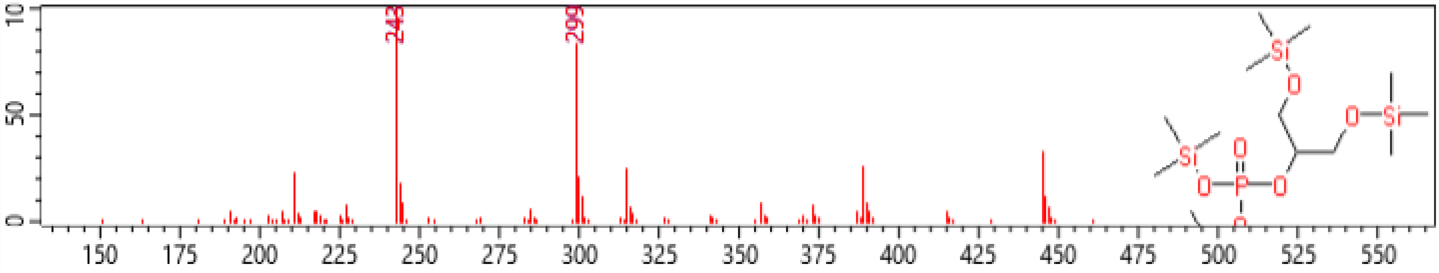

Phosphoric acid, 2-(trimethylsiloxy)-1-[(trimethylsiloxy)methyl]ethyl bis(trimethylsilyl) ester

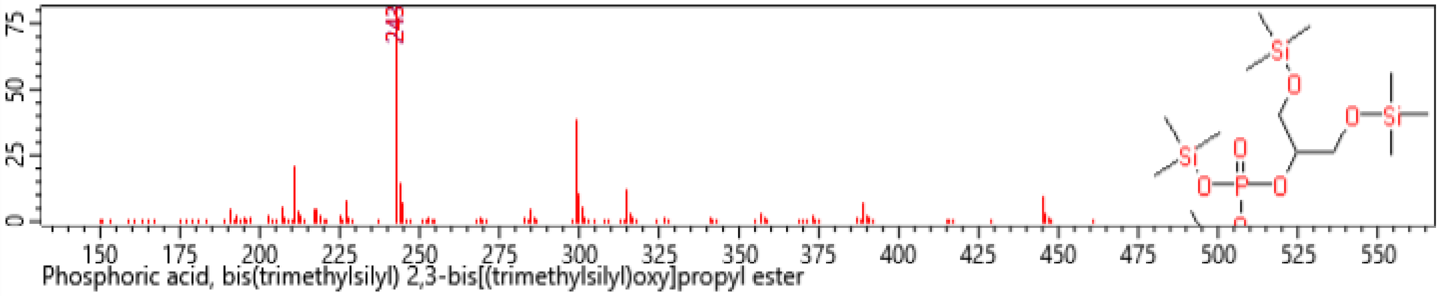


Phosphoric acid, bis(trimethylsilyl) 2,3-bis[(trimethylsilyl)oxy]propyl ester

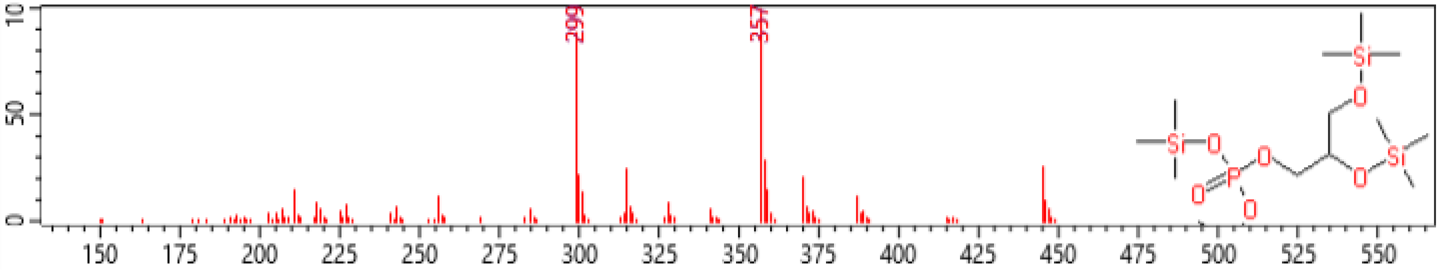


Phosphoric acid, bis(trimethylsilyl) 2,3-bis[(trimethylsilyl)oxy]propyl ester

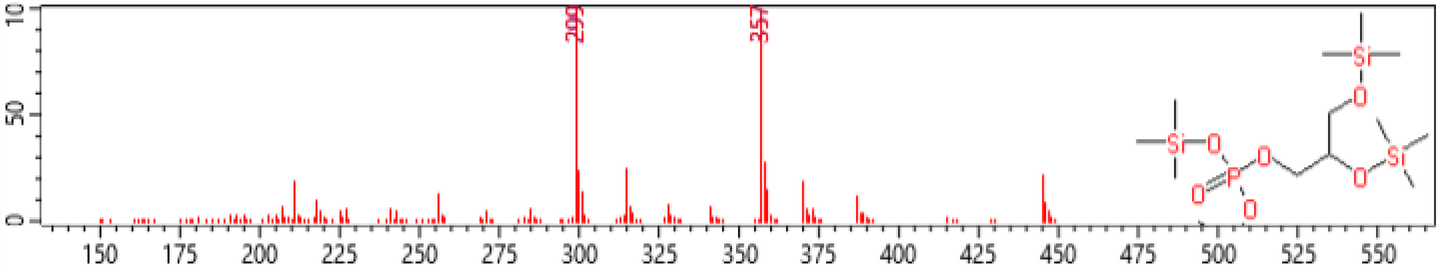


**2,6-Diisopropylnaphthalene**

Peak: 13.08 - 13.10 min (scan #1697 - #1701) Subtracted noise: 13.05 - 13.06 min (scan #1688 - #1692), 13.11 - 13.12 min (scan #17

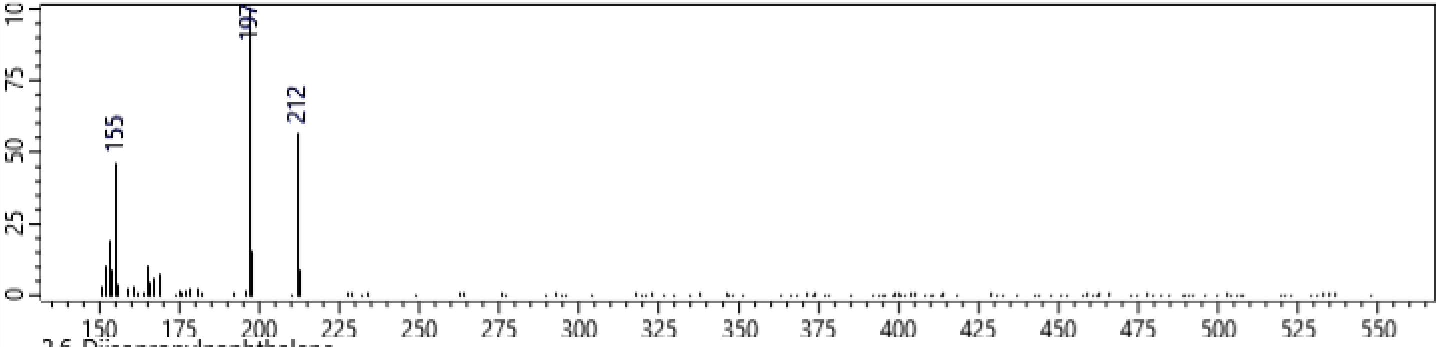


2,6-Diisopropylnaphthalene

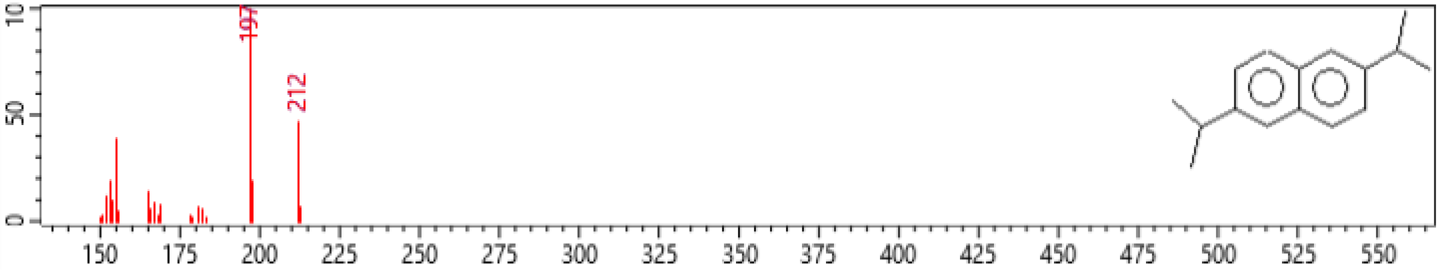

2,6-Diisopropylnaphthalene

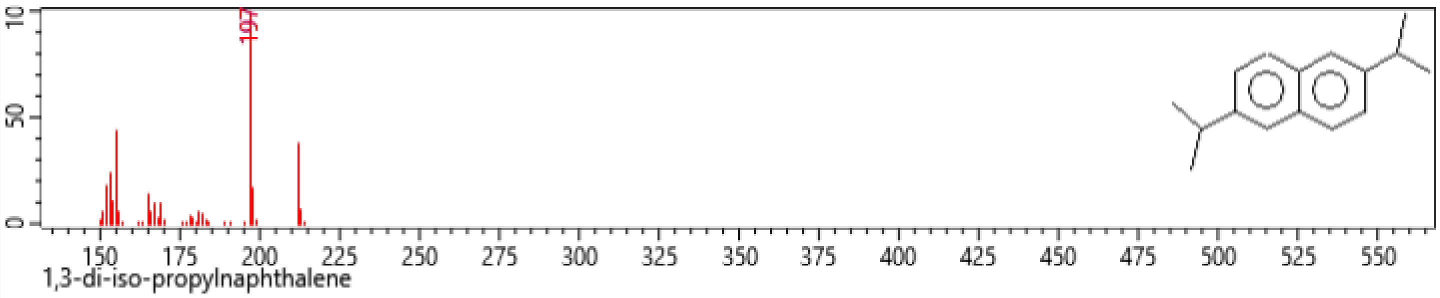


1,3-di-iso-propylnaphthalene

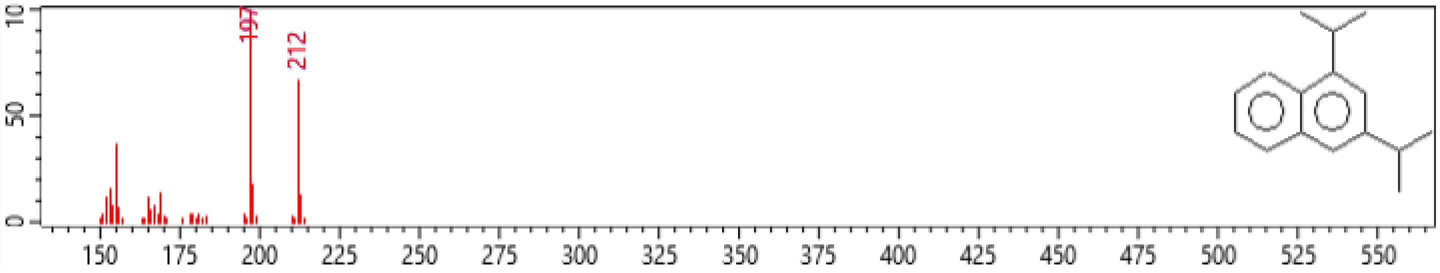


1,7-di-iso-propylnaphthalene

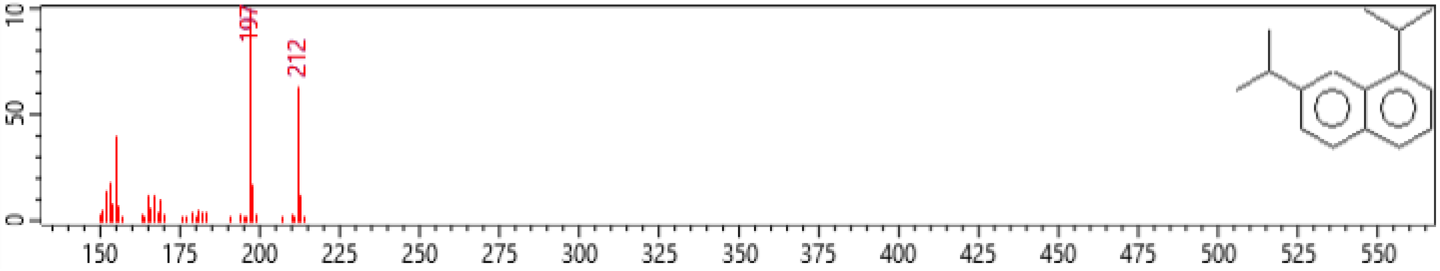


1,4-di-iso-propylnaphthalene

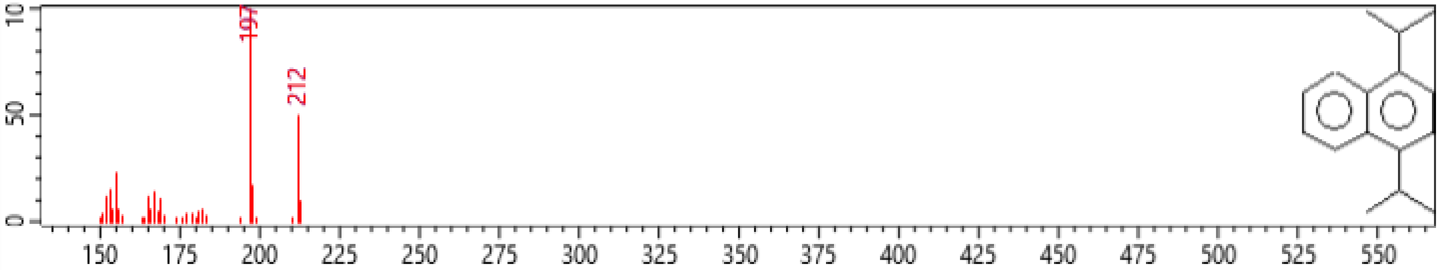


**Picras-4-en-16-one, 1,2,11,12,14,15-hexahydroxy-, (1β,2α,11β,12α,15β)-, 4TMS**
Peak: 13.12 - 13.15 min (scan #1707 - #1717) Subtracted noise: 13.07 - 13.10 min (scan #1693 - #1703), 13.19 - 13.22 min (scan #17

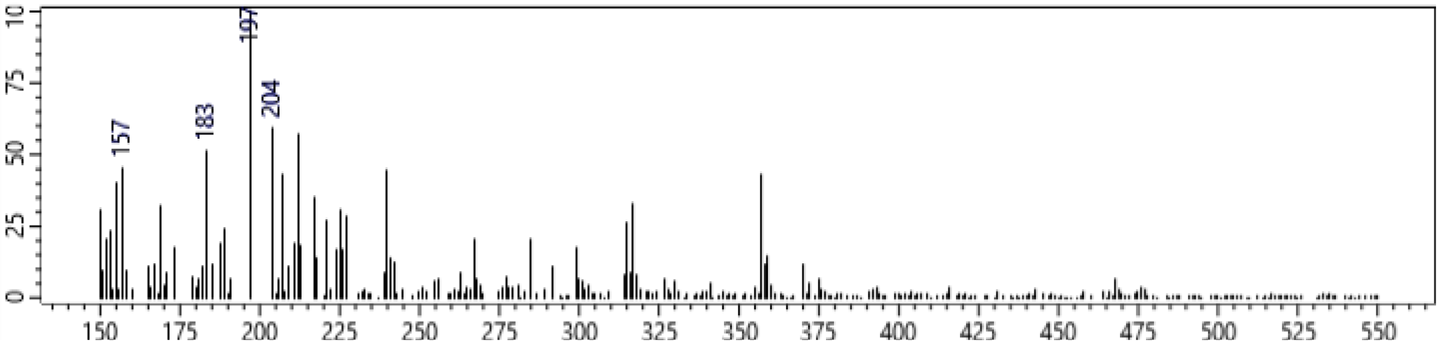

Picras-4-en-16-one, 1,2,11,12,14,15-hexahydroxy-, (1β,2α,11β,12α,15β)-, 4TMS

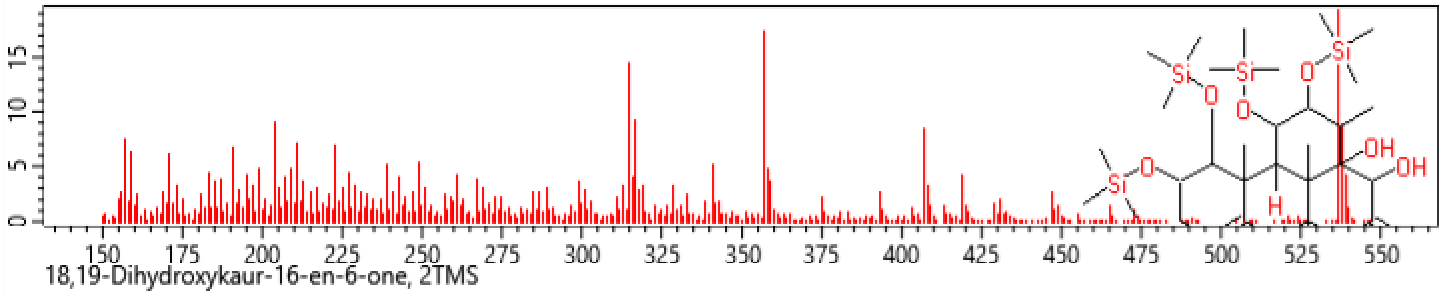

18,19-Dihydroxykaur-16-en-6-one, 2TMS

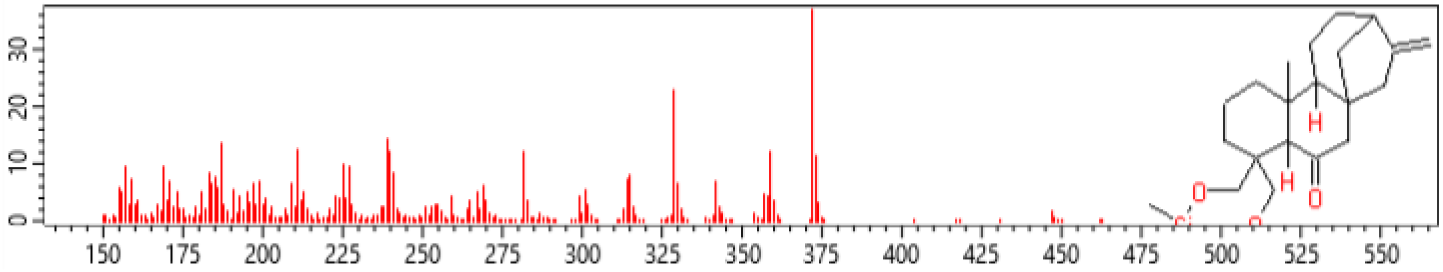

5-Methylethuliacoumarin A, TMS

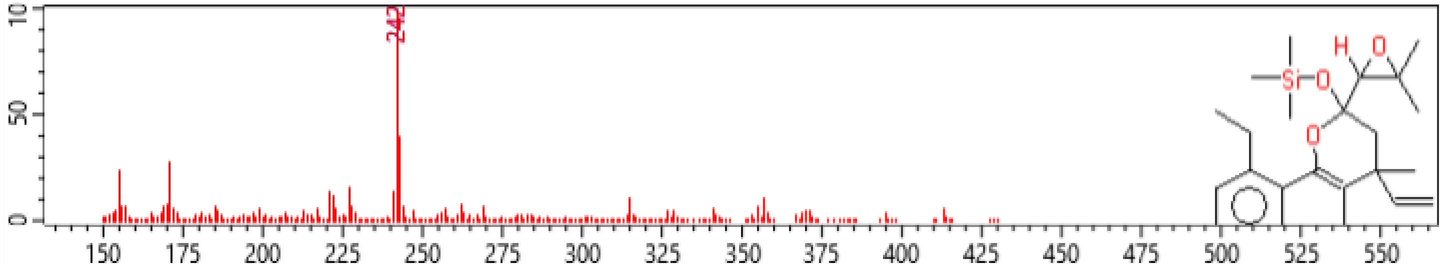

Lecocarpinolide J, TMS

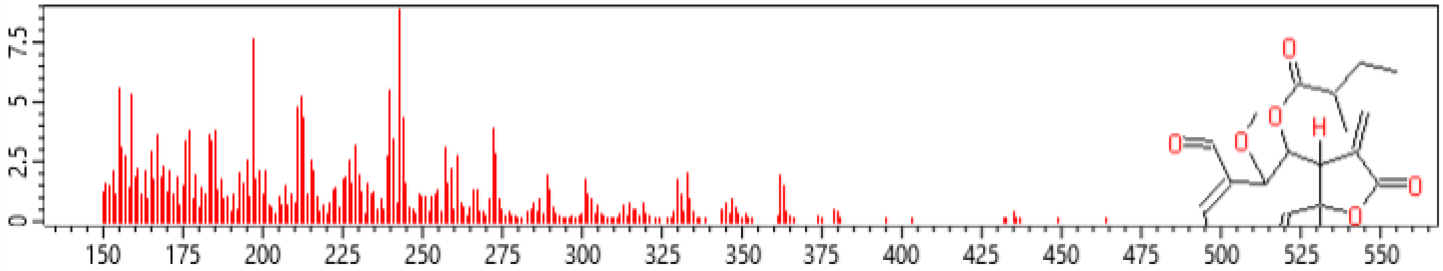

D-Erythro-Pentofuranose, 2-deoxy-1,3-bis-O-(trimethylsilyl)-, bis(trimethylsilyl) phosphate

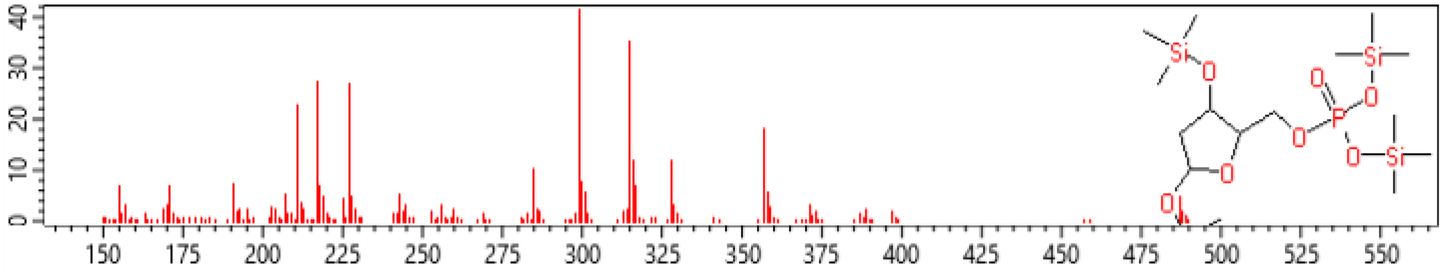

**Dihydronormorphinone**

Peak: 13.21 - 13.24 min (scan #1733 - #1741) Subtracted noise: 13.16 - 13.18 min (scan #1718 - #1725), 13.25 - 13.27 min (scan #17[illegible]

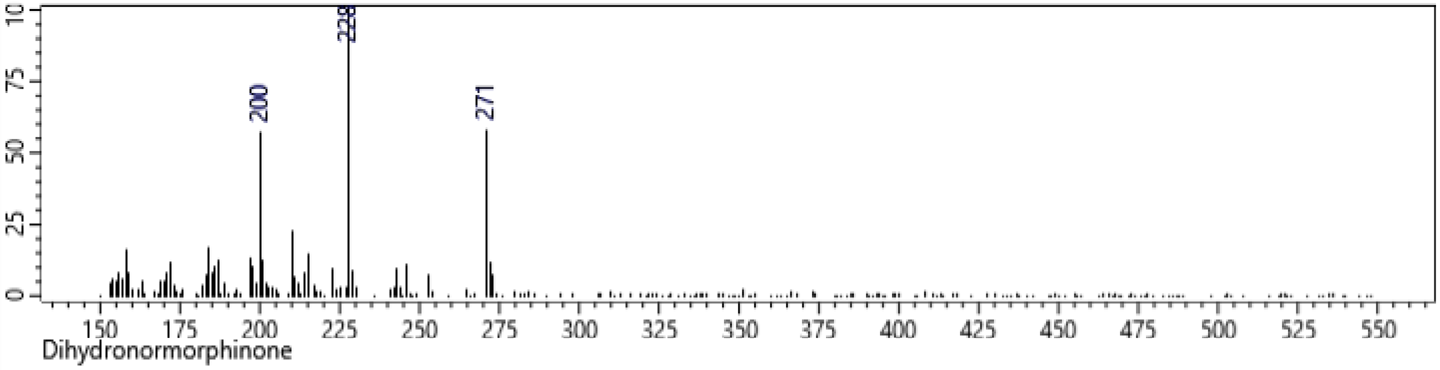


Dihydronormorphinone

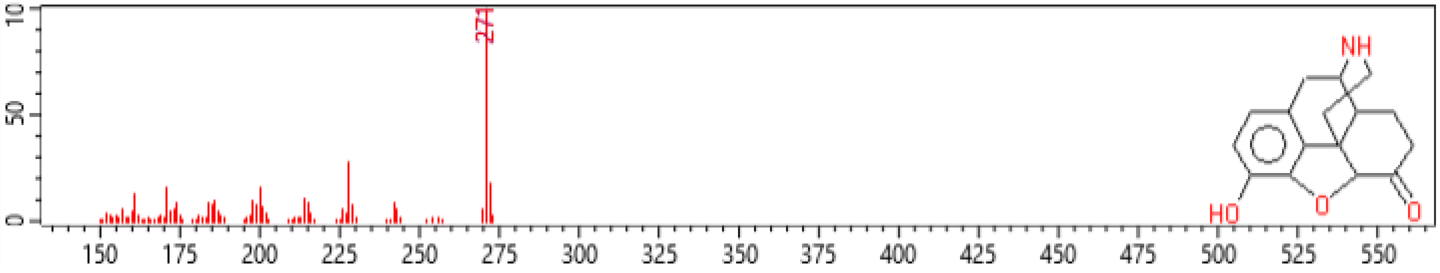


Dibucaine

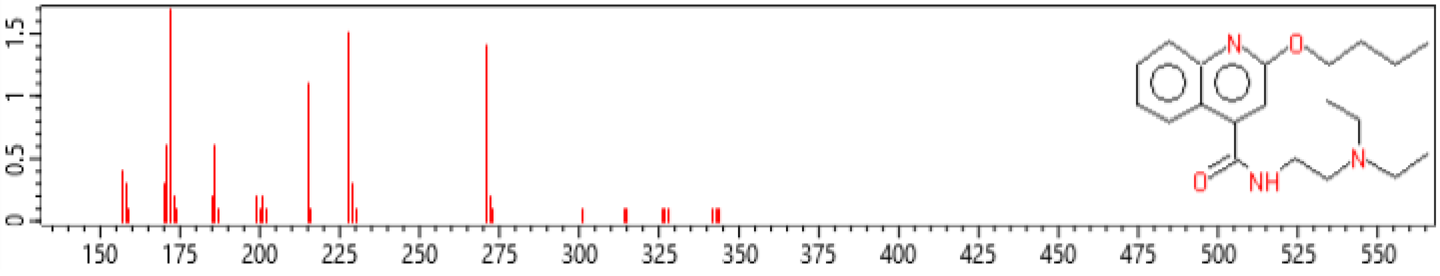


1-(4-Piperidinyl)-1H-benzimidazole, N-(2-methylpropionyl)-

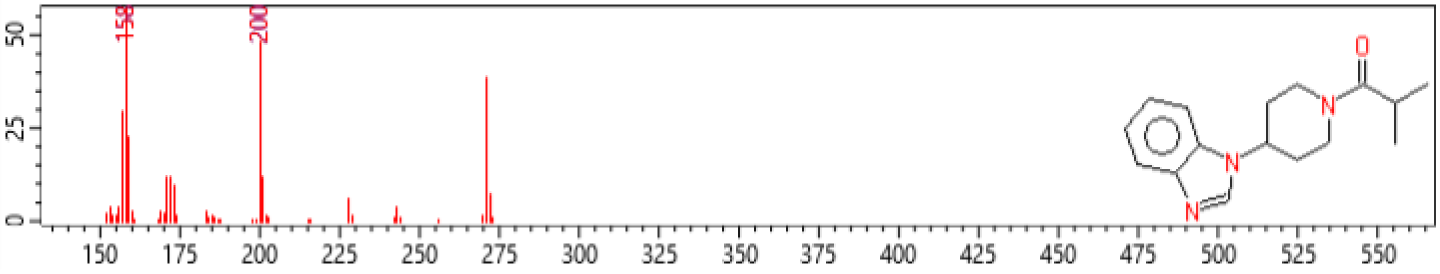


Crinan-1-one

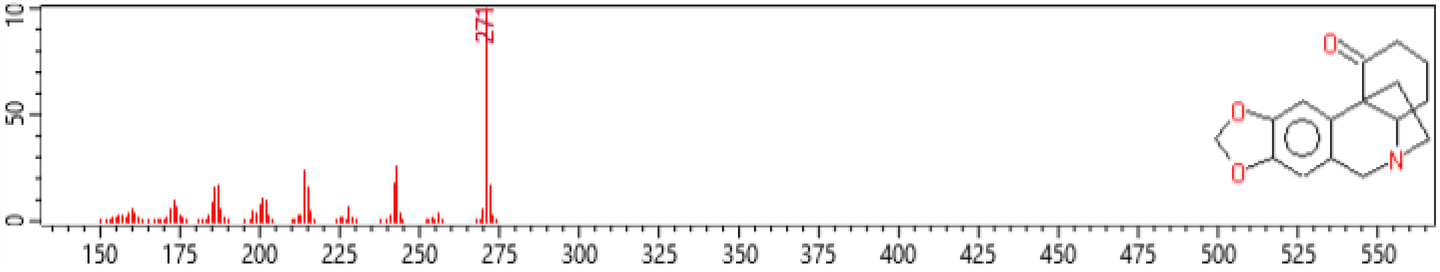

Crinan-1-ol, 2,3-didehydro-,(1α-

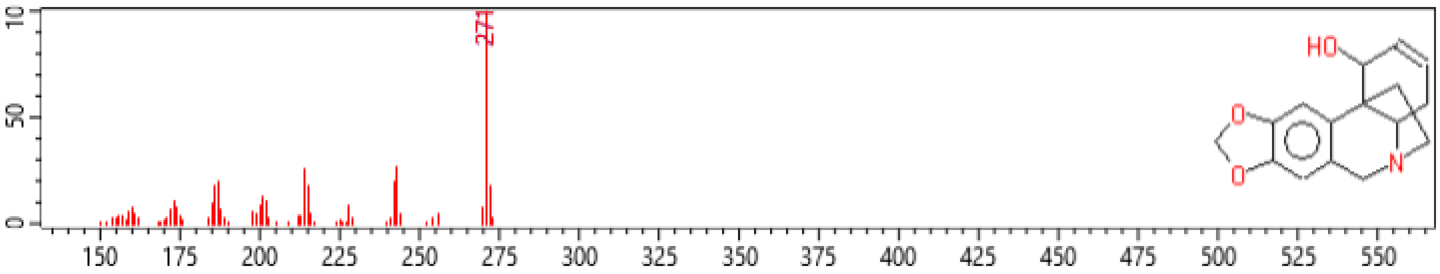


**Phosphoric acid, bis(trimethylsilyl) 2,3-bis[(trimethylsilyl)oxy]propyl ester**

Peak: 13.28 - 13.31 min (scan #1752 - #1760) Subtracted noise: 13.21 - 13.24 min (scan #1734 - #1742), 13.31 - 13.35 min (scan #17

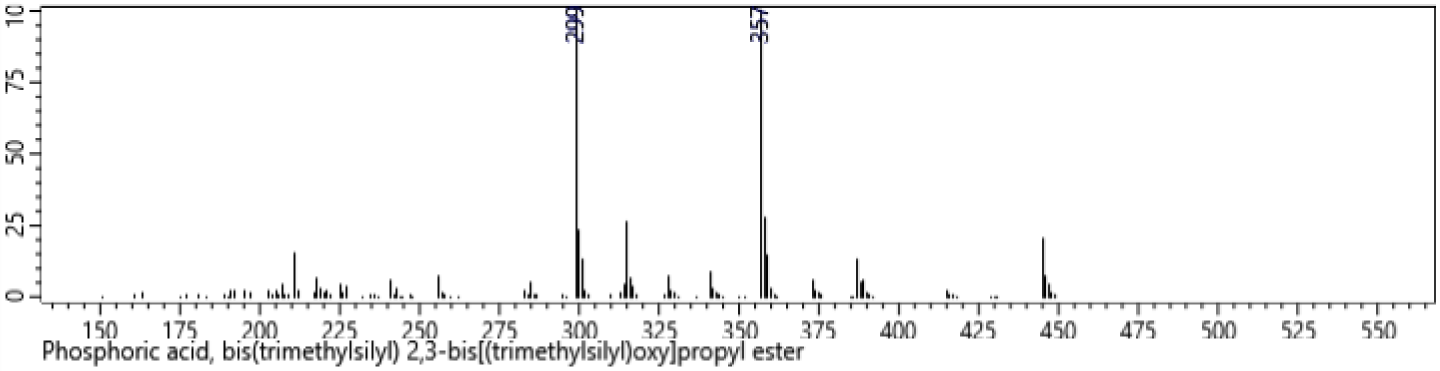


Phosphoric acid, bis(trimethylsilyl) 2,3-bis[(trimethylsilyl)oxy]propyl ester

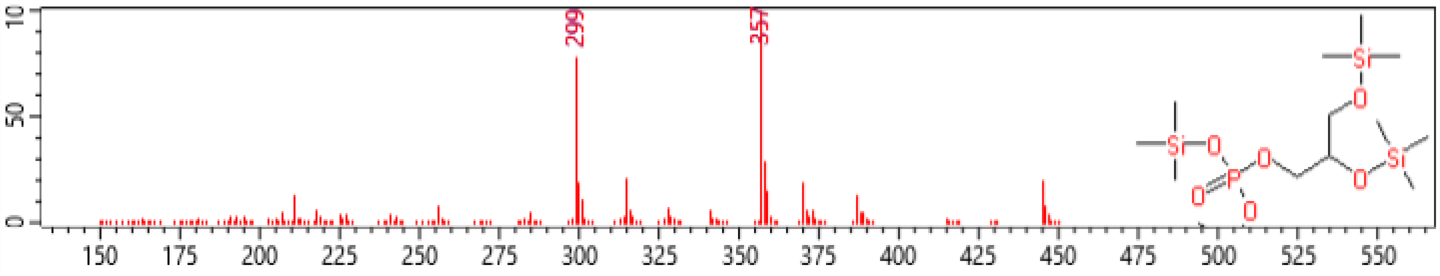


Phosphoric acid, bis(trimethylsilyl) 2,3-bis[(trimethylsilyl)oxy]propyl ester

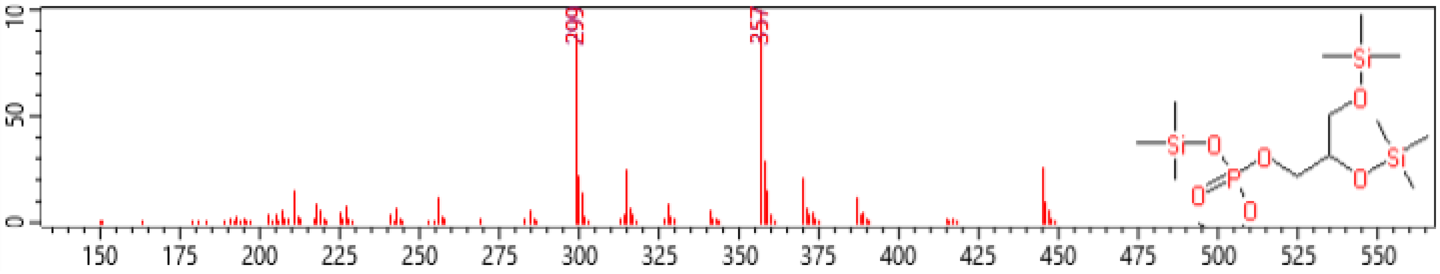


Phosphoric acid, bis(trimethylsilyl) 2,3-bis[(trimethylsilyl)oxy]propyl ester

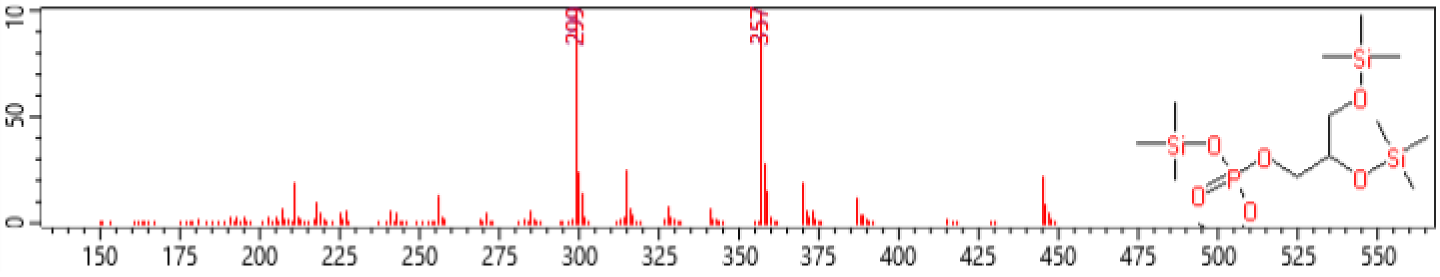

Phosphoric acid, bis(trimethylsilyl) 2,3-bis[(trimethylsilyl)oxy]propyl ester

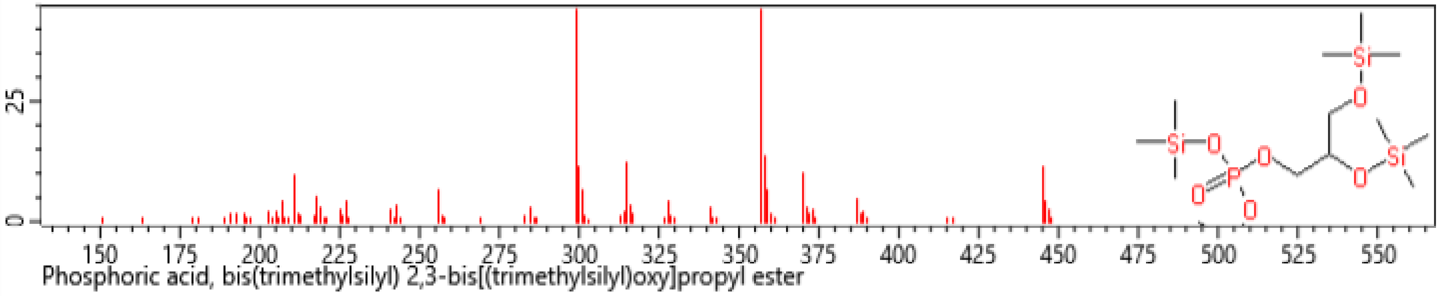

Phosphoric acid, bis(trimethylsilyl) 2,3-bis[(trimethylsilyl)oxy]propyl ester

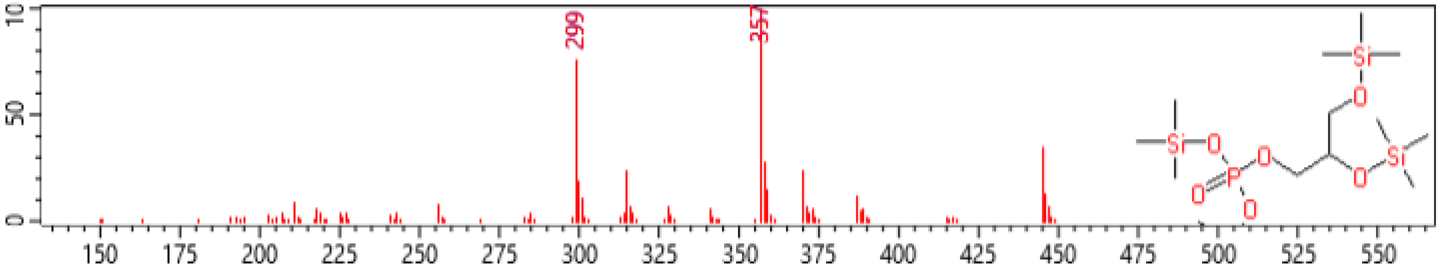


**1-Tetradecanol, TBDMS derivative**

Peak: 13.34 - 13.38 min (scan #1769 - #1779) Subtracted noise: 13.28 - 13.31 min (scan #1752 - #1761), 13.39 - 13.43 min (scan #17

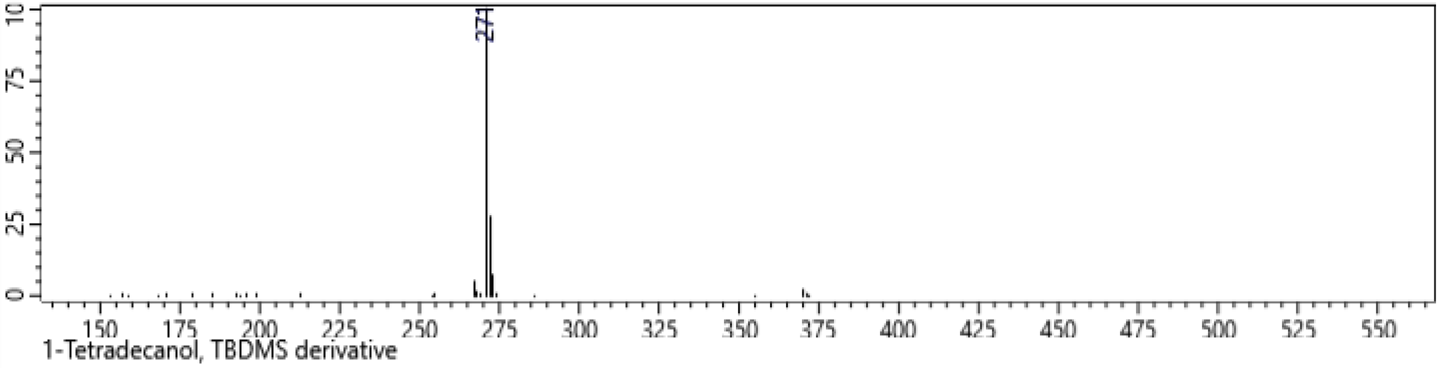


1-Tetradecanol, TBDMS derivative

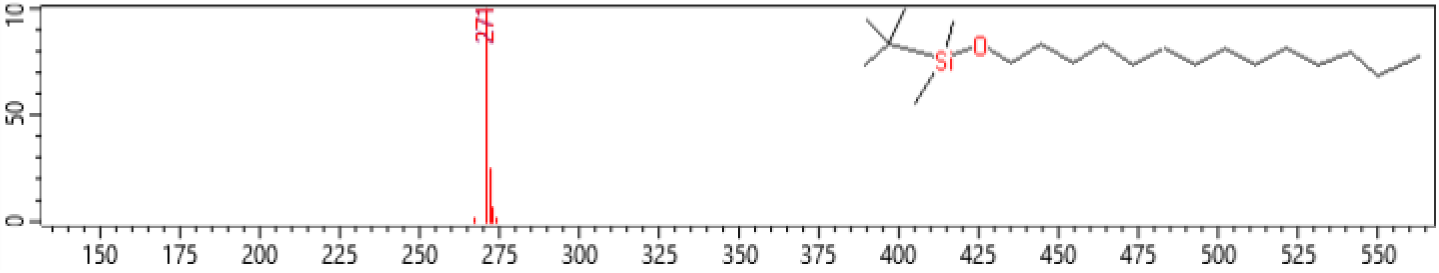


1-Tetradecanol, TMS derivative

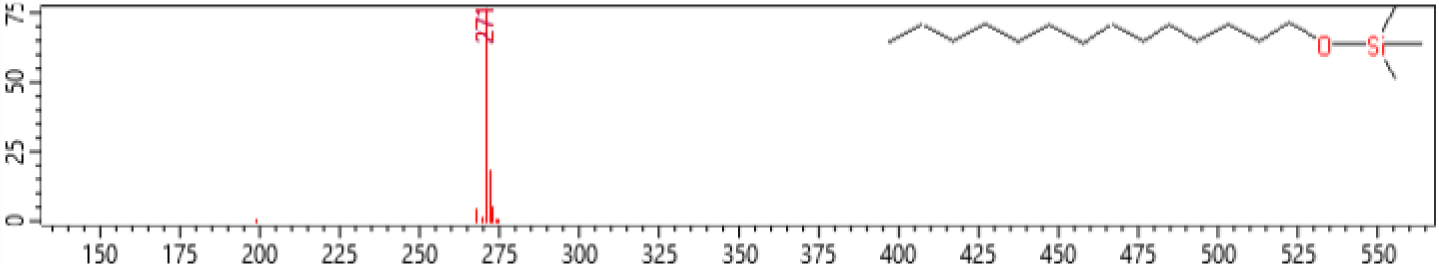

1-Tetradecanol, TMS derivative

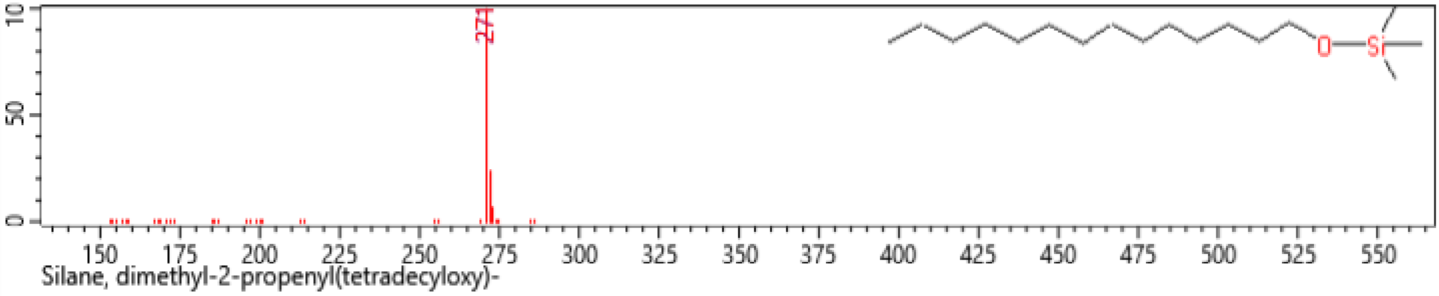


Silane, dimethyl-2-propenyl(tetradecyloxy)-

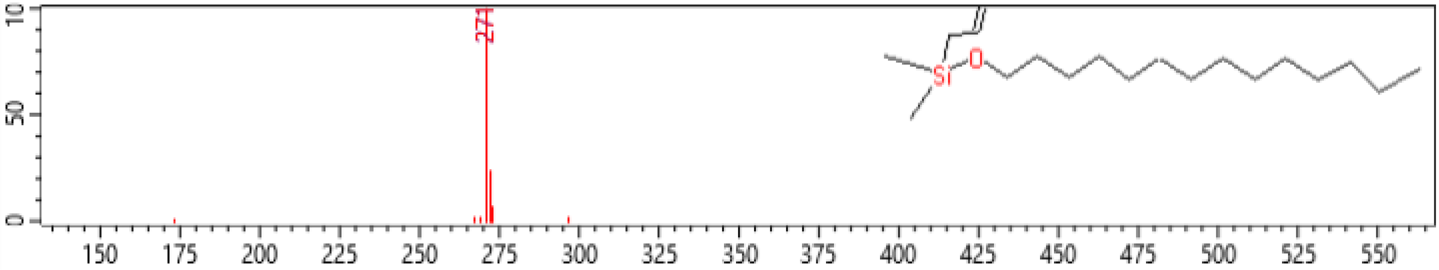


1-Dimethyl(chloromethyl)silyloxytetradecane

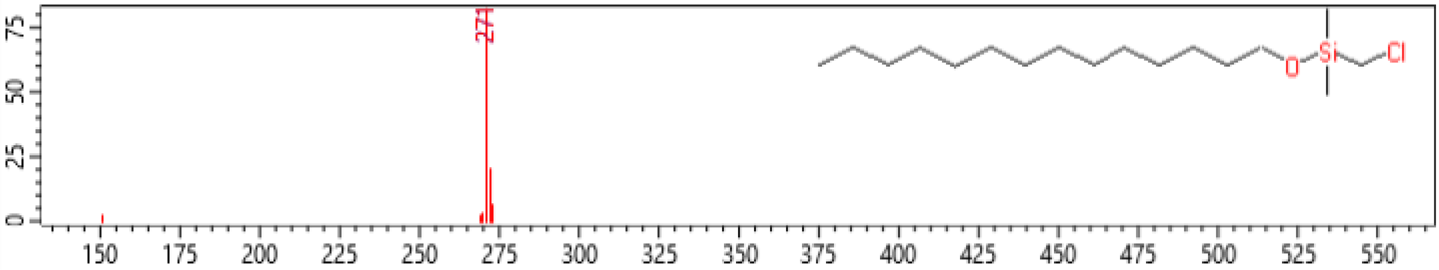


**4-Cyclohexyldimethylsilyloxy-2-methyloct-5-yne**

Peak: 13.39 - 13.42 min (scan #1783 - #1791) Subtracted noise: 13.36 - 13.39 min (scan #1775 - #1782), 13.46 - 13.49 min (scan #18

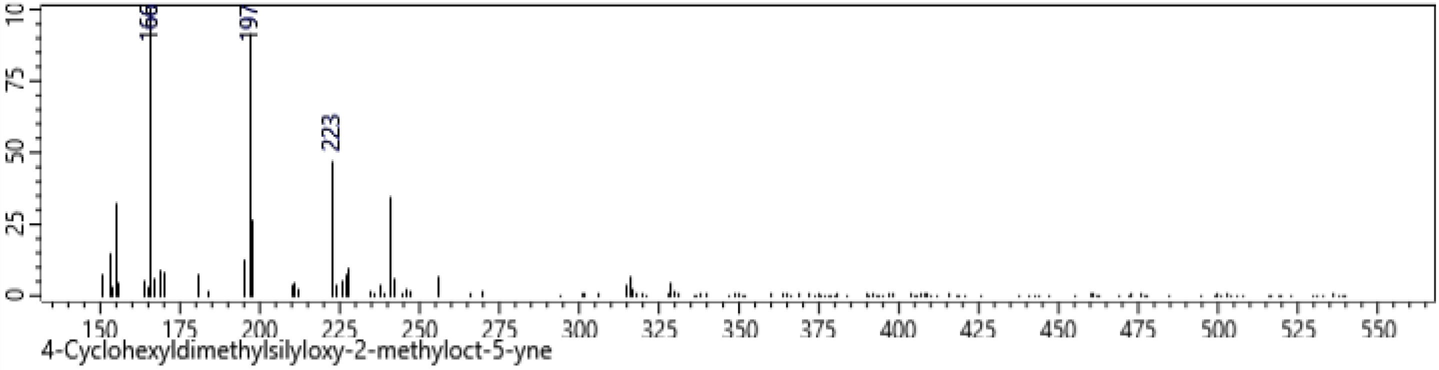


4-Cyclohexyldimethylsilyloxy-2-methyloct-5-yne

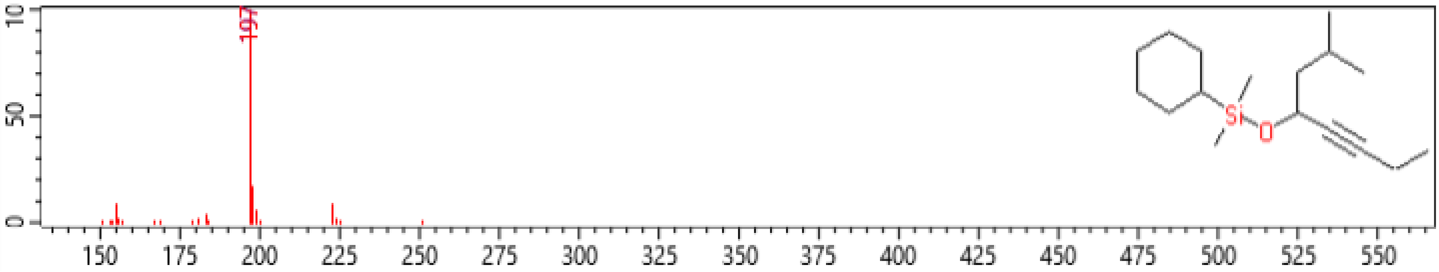

4-Chloro-6,7-bis(2-methoxyethoxy)quinazoline

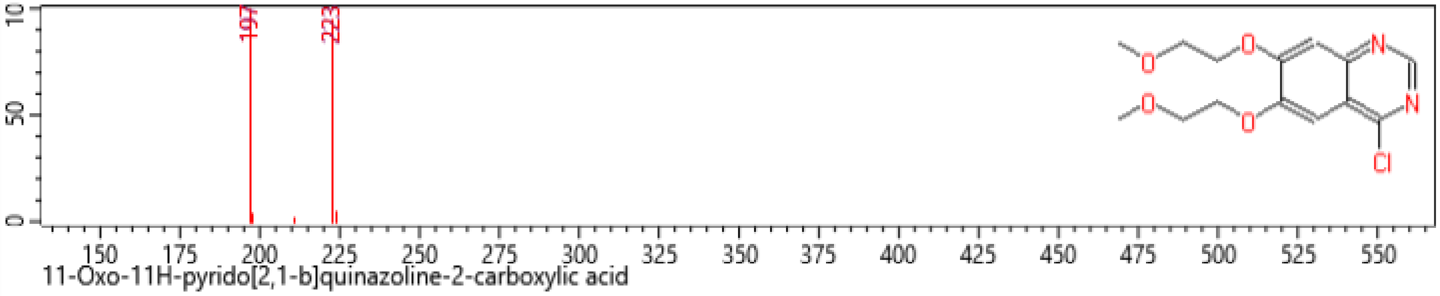


11-Oxo-11H-pyrido[2,1-b]quinazoline-2-carboxylic acid

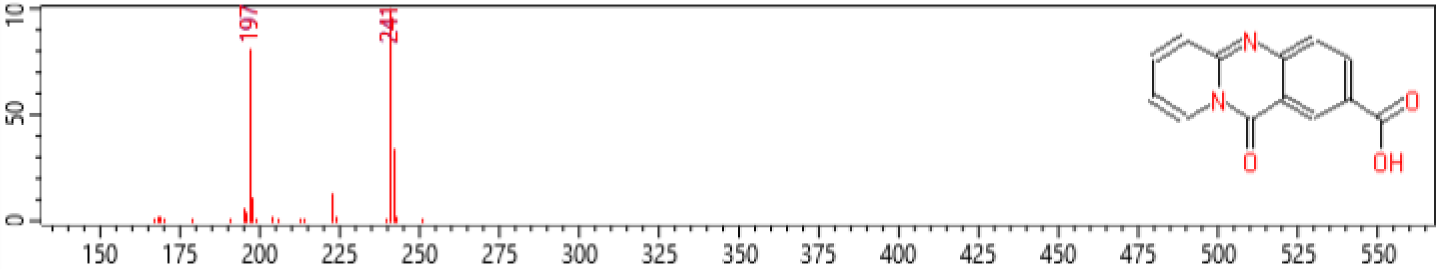


2-Hydrazinyl-4-phenyl-1,3-benzothiazole

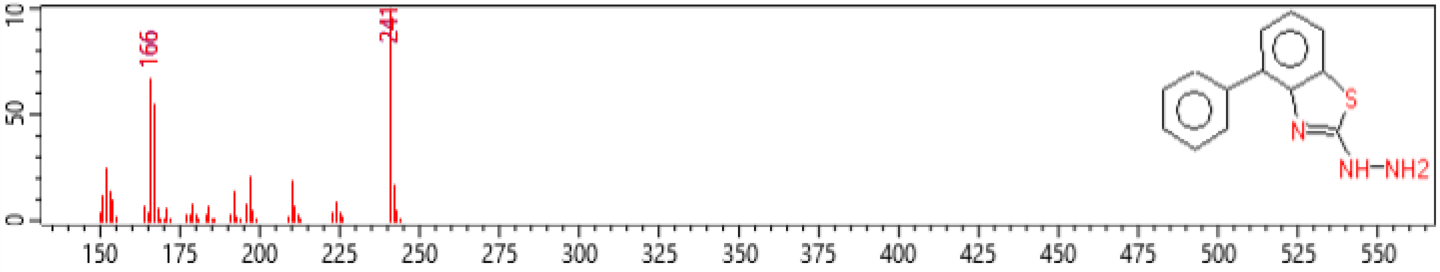


Allophanic acid, 4-(1-naphthoyl)-, phenyl ester

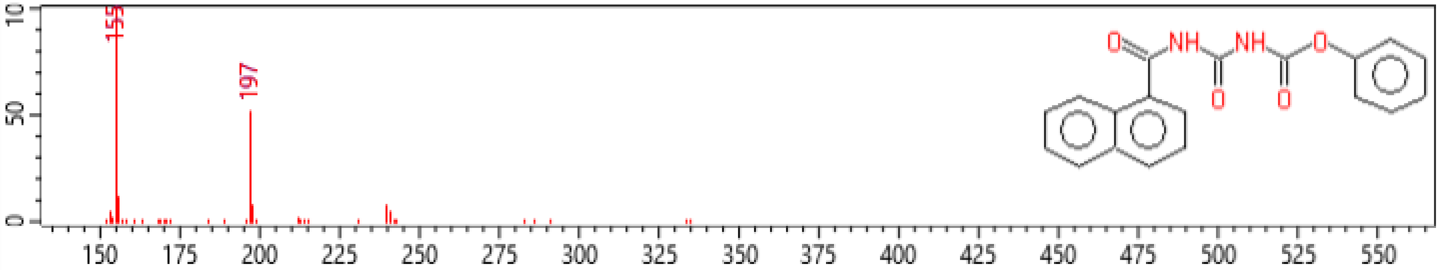


**3-Methoxycarbonyl-2-methylidenenonanoic acid, TMS derivative**

Peak: 13.48 - 13.50 min (scan #1807 - #1815) Subtracted noise: 13.43 - 13.45 min (scan #1793 - #1800), 13.52 - 13.55 min (scan #18

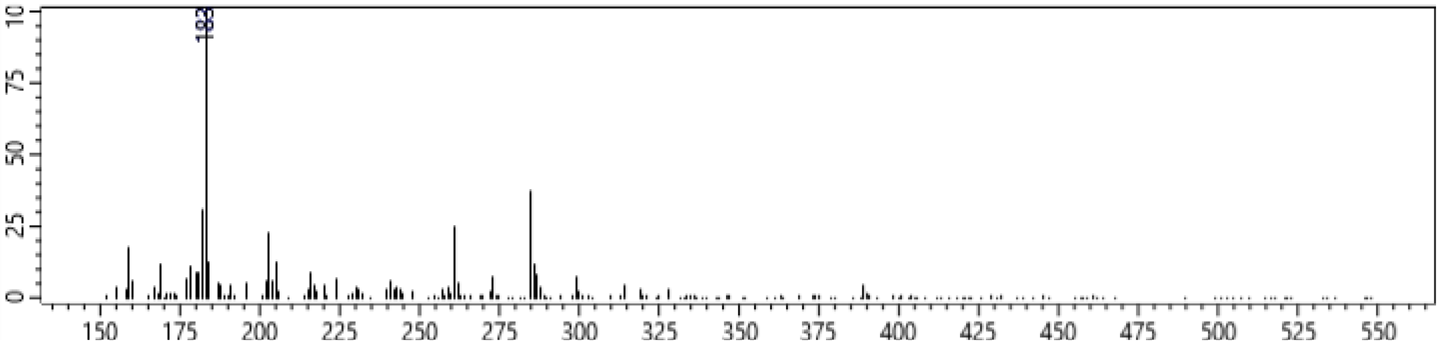

3-Methoxycarbonyl-2-methylidenenonanoic acid, TMS derivative

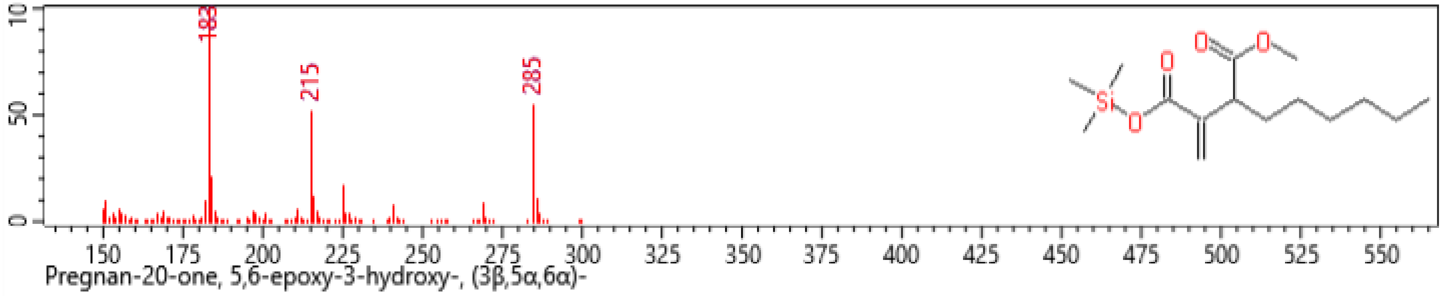


Pregnan-20-one, 5,6-epoxy-3-hydroxy-, (3β,5α,6α)-

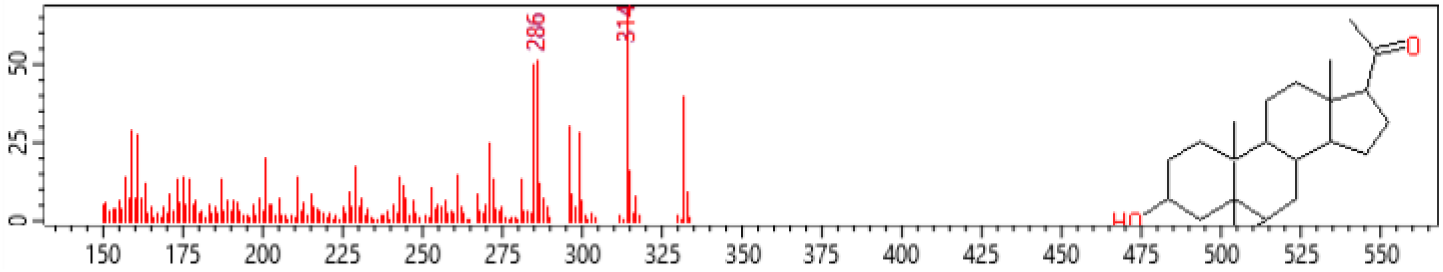


Fumarylacetone, bis(trimethylsilyl)-

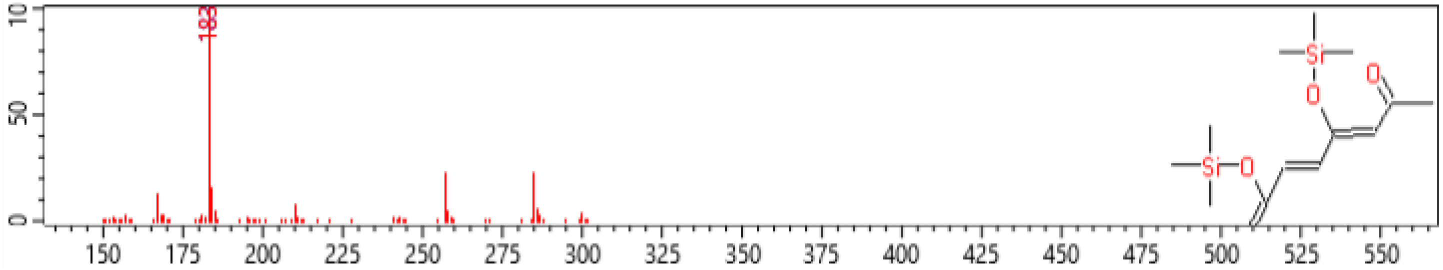


Silychristin

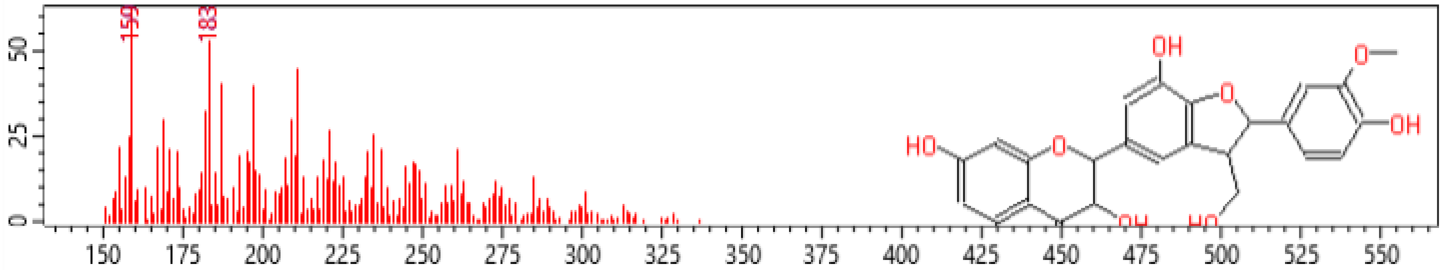


Silychristin

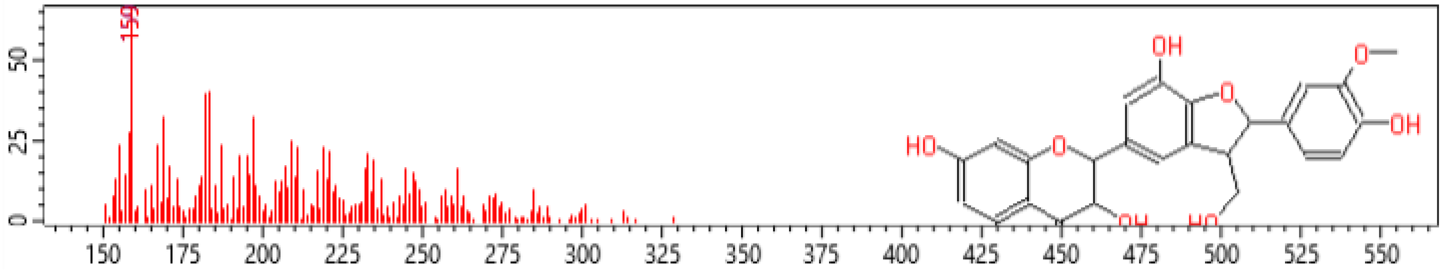

**Androst-5,7,9(11)-triene, 3-acetoxy-17-oxo-**

Peak: 13.53 - 13.55 min (scan #1823 - #1827) Subtracted noise: 13.50 - 13.51 min (scan #1814 - #1818), 13.56 - 13.58 min (scan #18

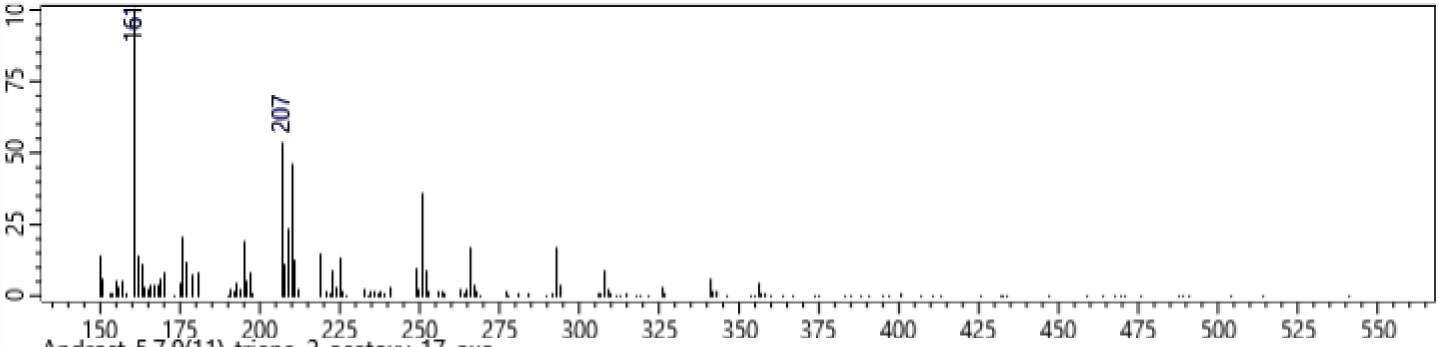


Androst-5,7,9(11)-triene, 3-acetoxy-17-oxo-

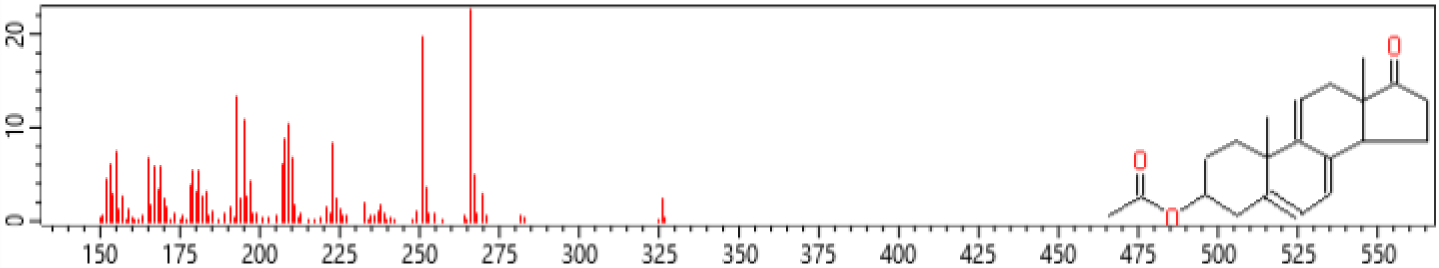

17-Methylandrosta-5,7,9(11)-trien-17-ol acetate

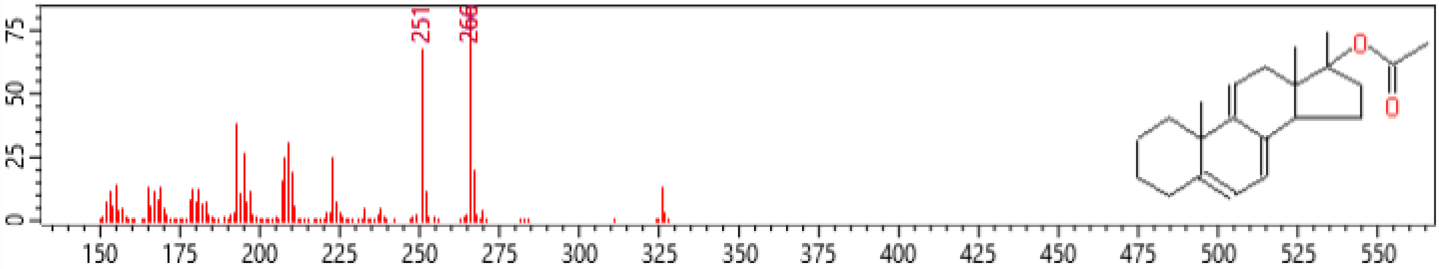


Benzothiazole-2,3-dicarboxylic acid, 2,3-dihydro-6-nitro-, dimethyl ester, 1,1-dioxide

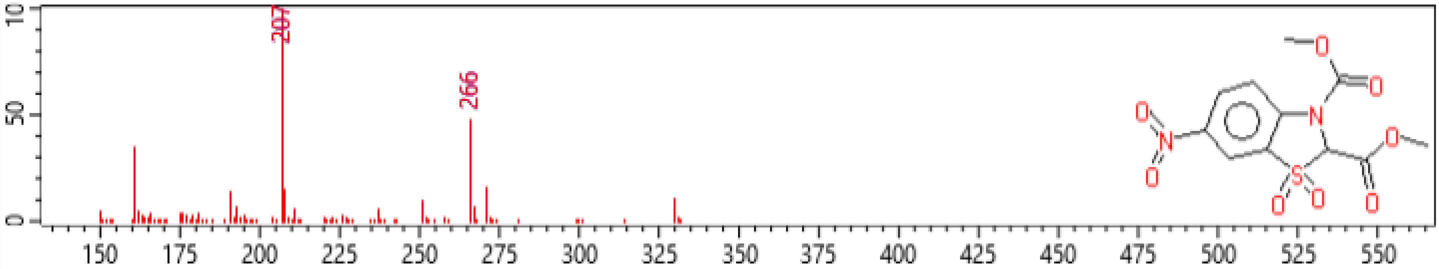


Terretonin

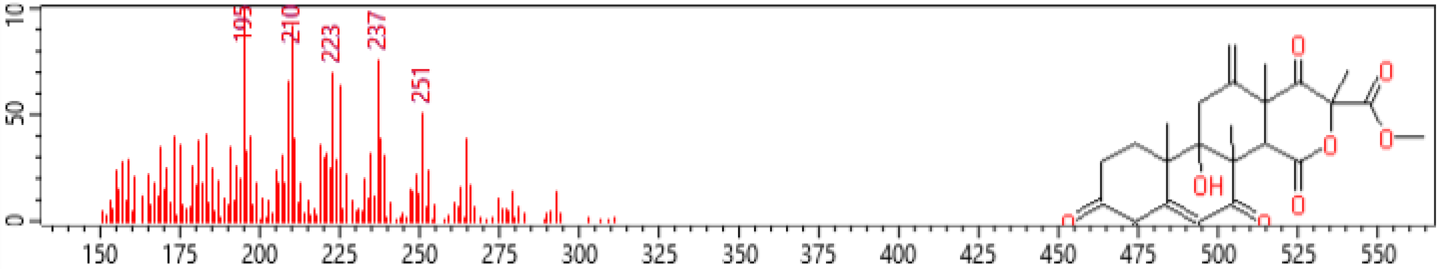

5,7,9(11)-Androstatriene, 3-hydroxy-17-oxo-

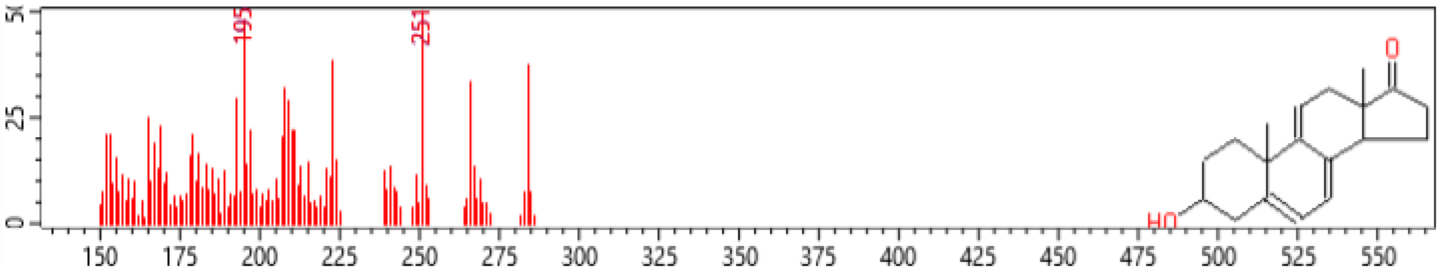


**Azelaic acid, 2TMS derivative**

Peak: 13.57 - 13.59 min (scan #1832 - #1840) Subtracted noise: 13.53 - 13.55 min (scan #1822 - #1829), 13.63 - 13.65 min (scan #18

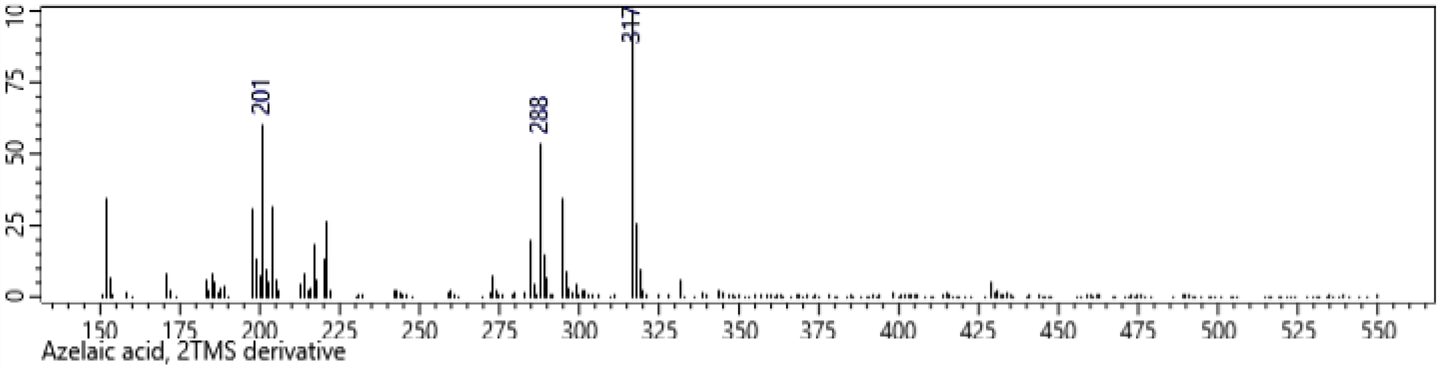


Azelaic acid, 2TMS derivative

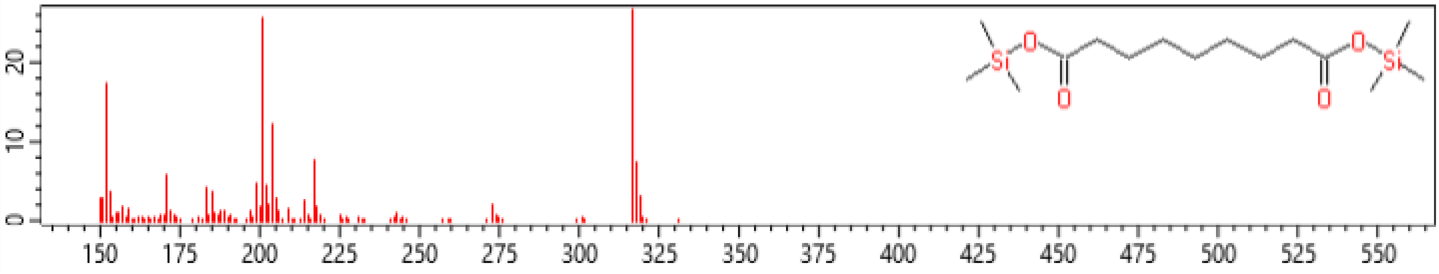


Azelaic acid, 2TMS derivative

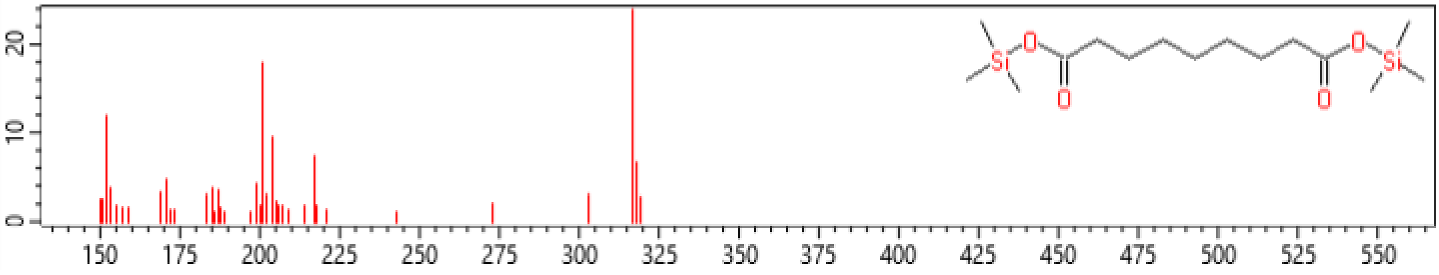


3-Isobutylpentanedioic acid, 2TMS derivative

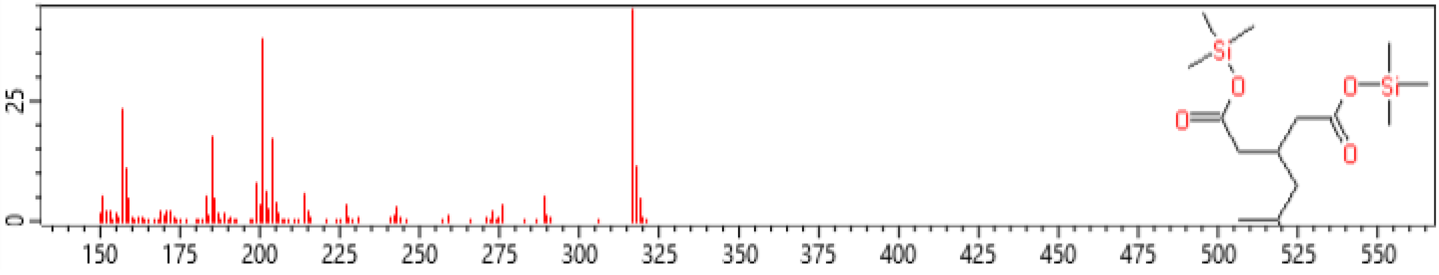

Trimethylsilyl 9-((trimethylsilyl)oxy)tetradecanoate

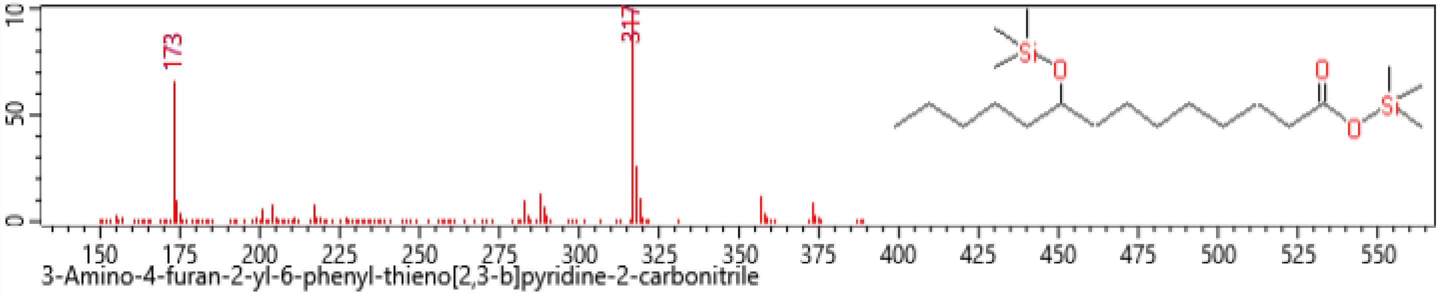


3-Amino-4-furan-2-yl-6-phenyl-thieno[2,3-b]pyridine-2-carbonitrile

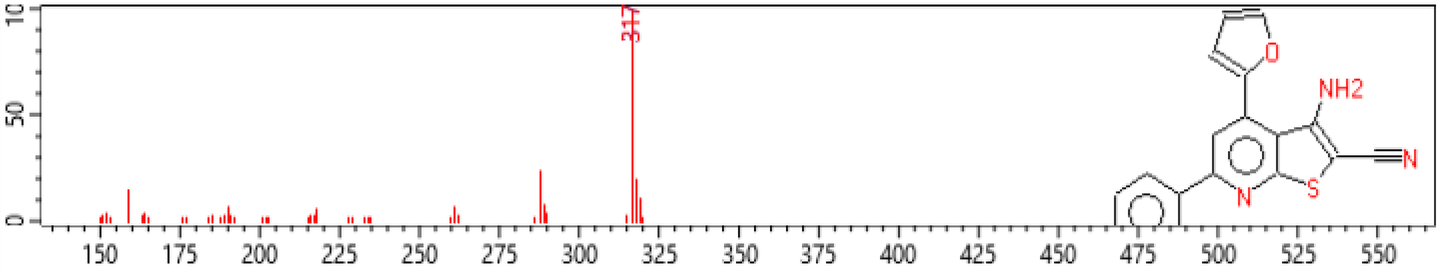


**3-Bromo-5-phenyl-7-(trifluoromethyl)pyrazolo[1,5-a]pyrimidine-2-carboxylic acid**

Peak: 13.65 - 13.68 min (scan #1856 - #1864) Subtracted noise: 13.59 - 13.62 min (scan #1839 - #1847), 13.69 - 13.72 min (scan #18

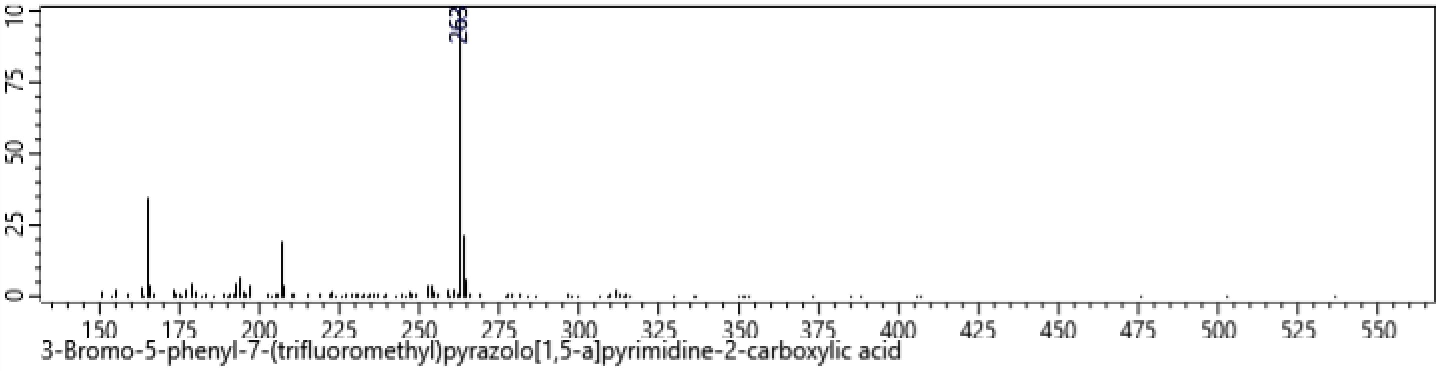


3-Bromo-5-phenyl-7-(trifluoromethyl)pyrazolo[1,5-a]pyrimidine-2-carboxylic acid

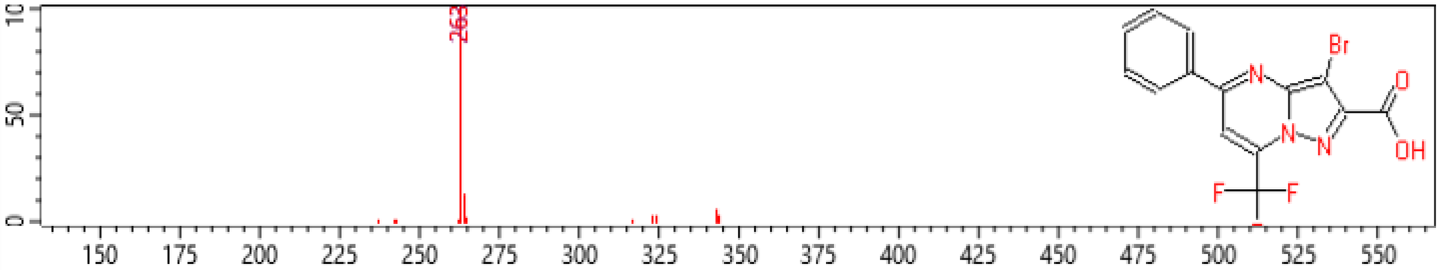


1-[2-(1-Adamantyl)ethyloxy]-1-ethyl-1-silacylopentane

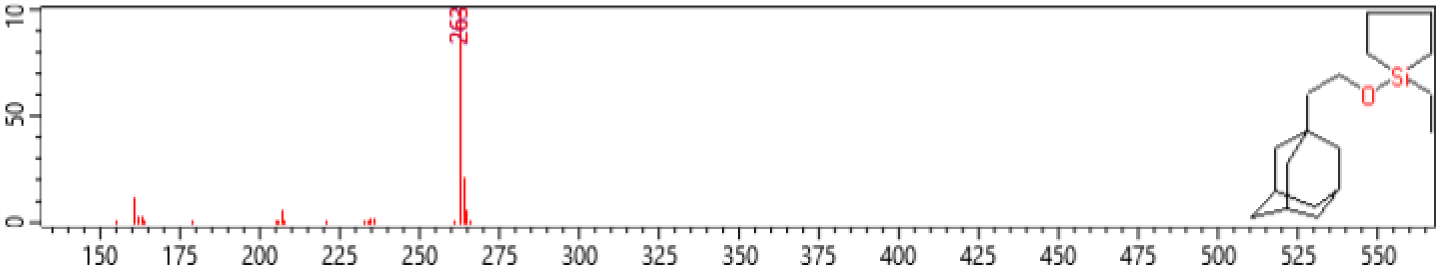

Monoallyl phthalate, TBDMS derivative

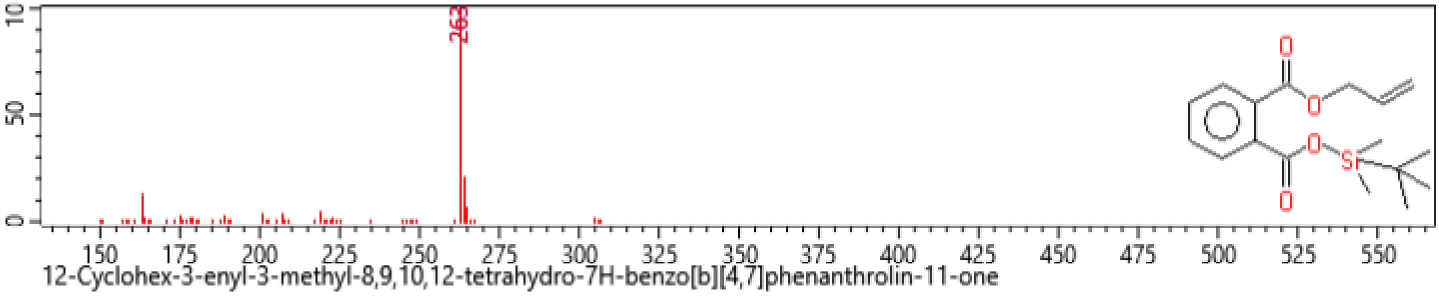


12-Cyclohex-3-enyl-3-methyl-8,9,10,12-tetrahydro-7H-benzo[b][4,7]phenanthrolin-11-one

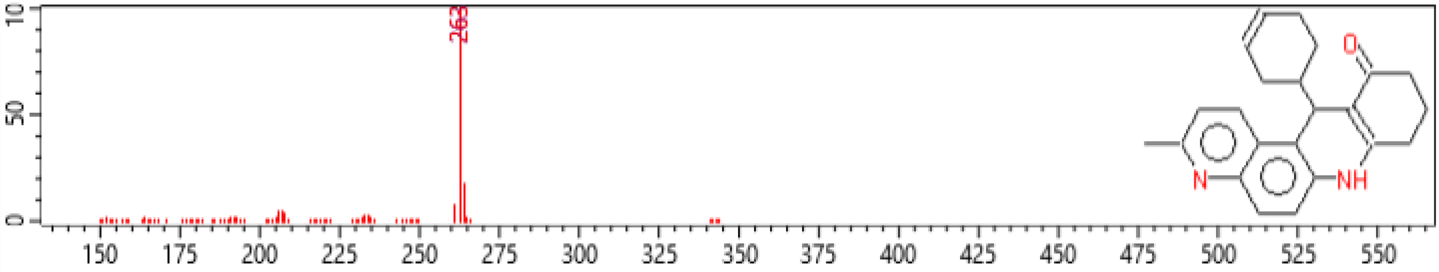


1-Tributylsilyloxy-2-phenylethane

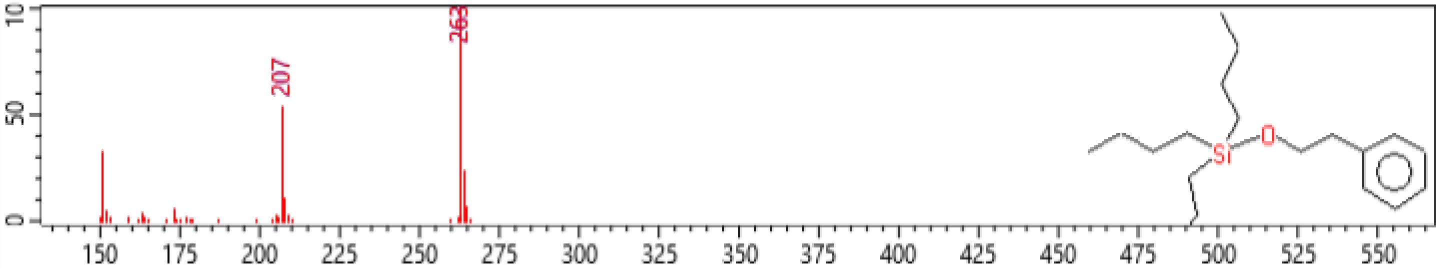


**Benzyl octyl maleate**

Peak: 13.70 - 13.72 min (scan #1870 - #1874) Subtracted noise: 13.67 - 13.69 min (scan #1862 - #1866), 13.73 - 13.75 min (scan #18

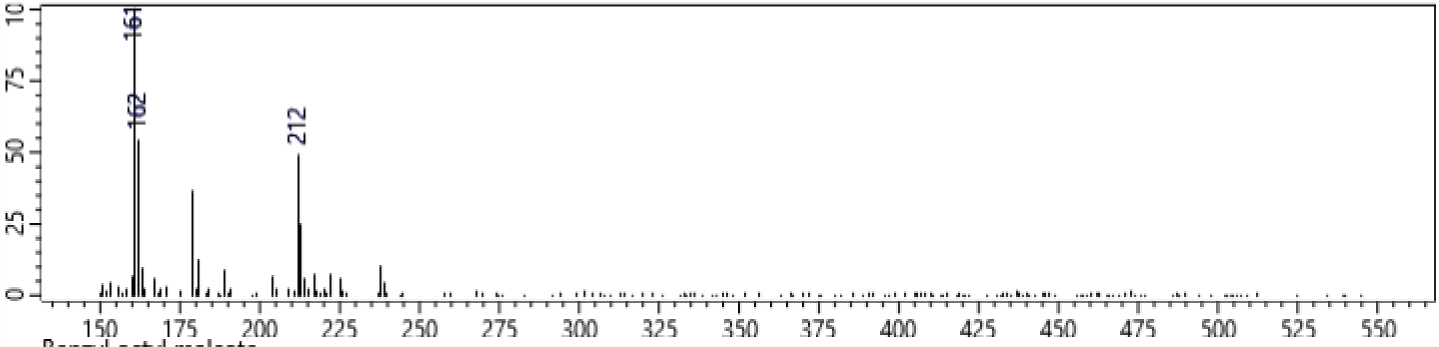


Benzyl octyl maleate

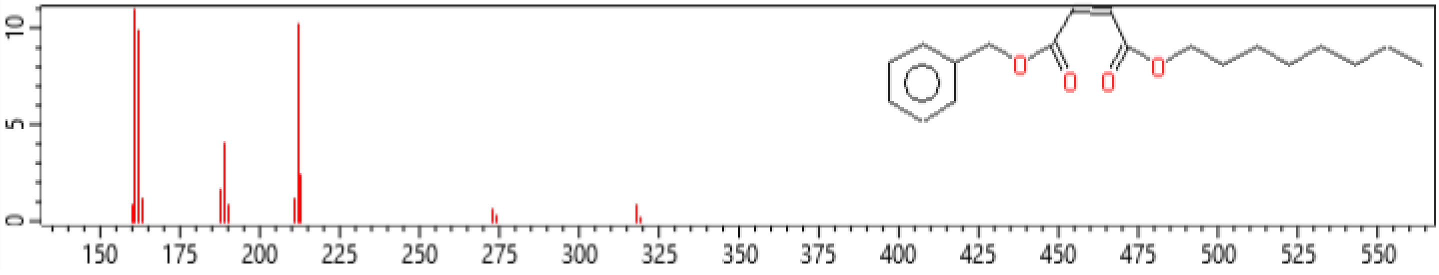

Ethofumesate

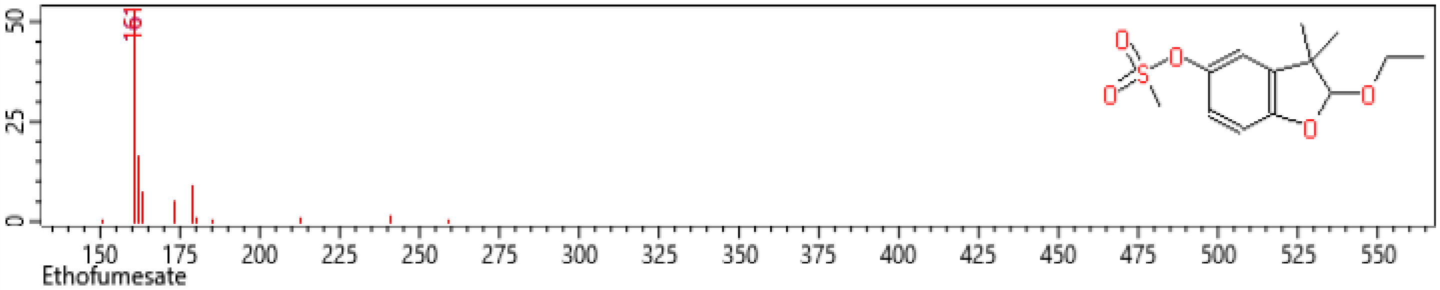


Ethofumesate

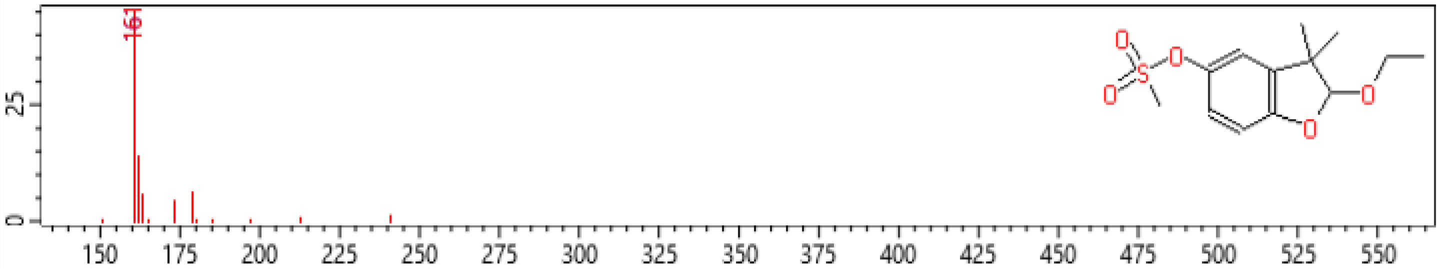


Ethofumesate

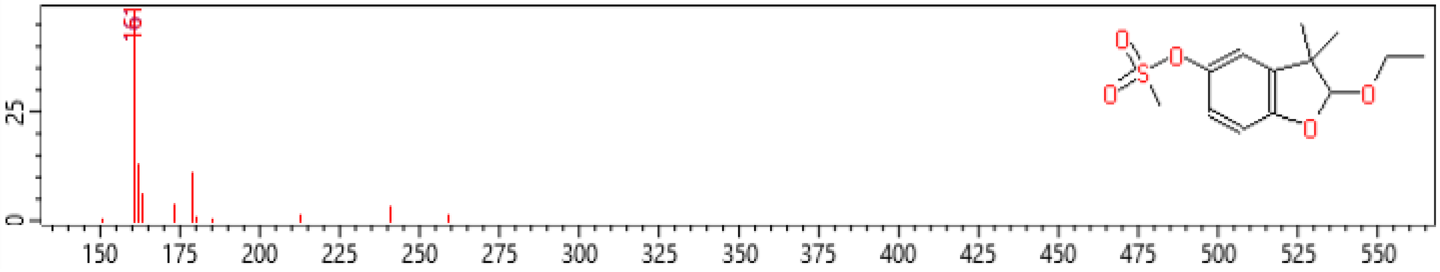


Ethofumesate

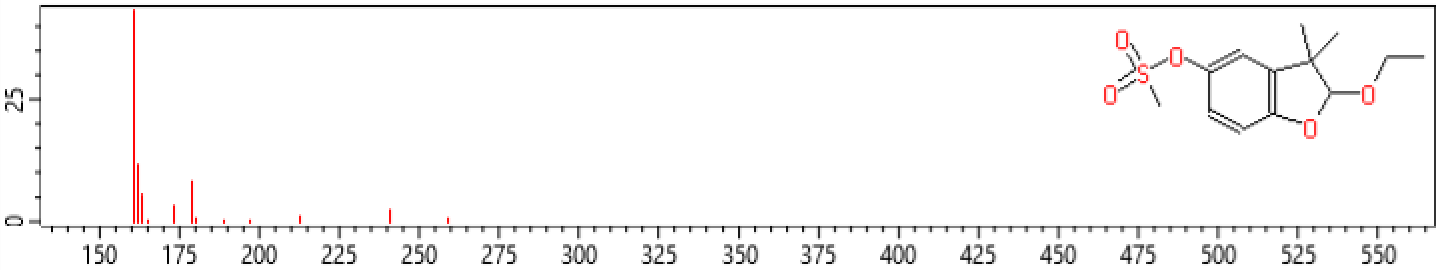

**Citric acid, 4TMS derivative**

Peak: 13.76 - 13.81 min (scan #1887 - #1899) Subtracted noise: 13.69 - 13.73 min (scan #1866 - #1877), 13.82 - 13.86 min (scan #19

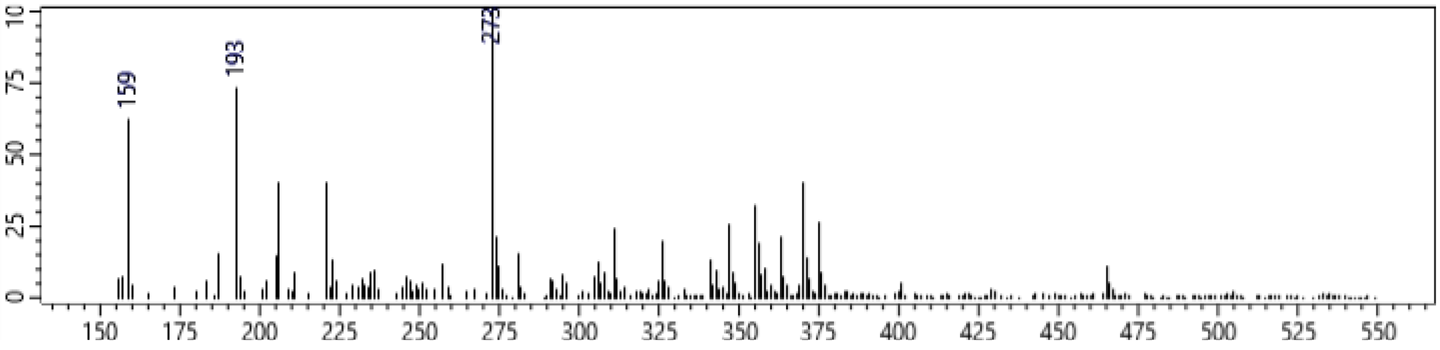

Citric acid, 4TMS derivative

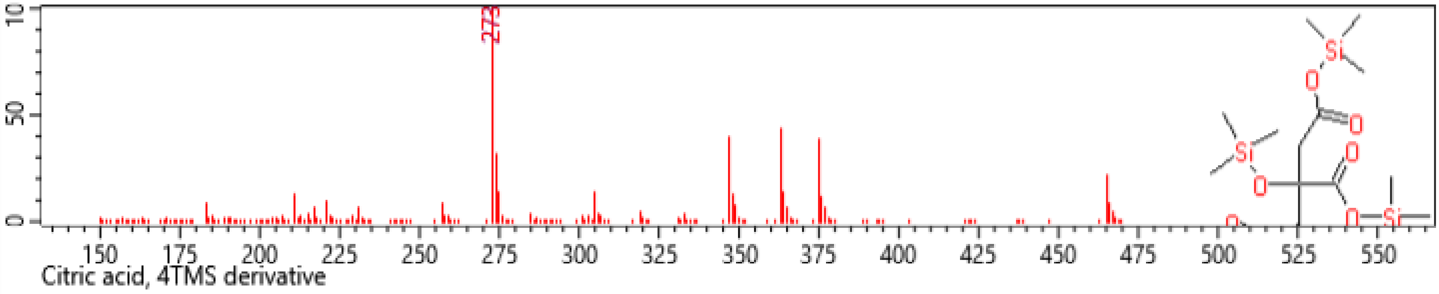


Citric acid, 4TMS derivative

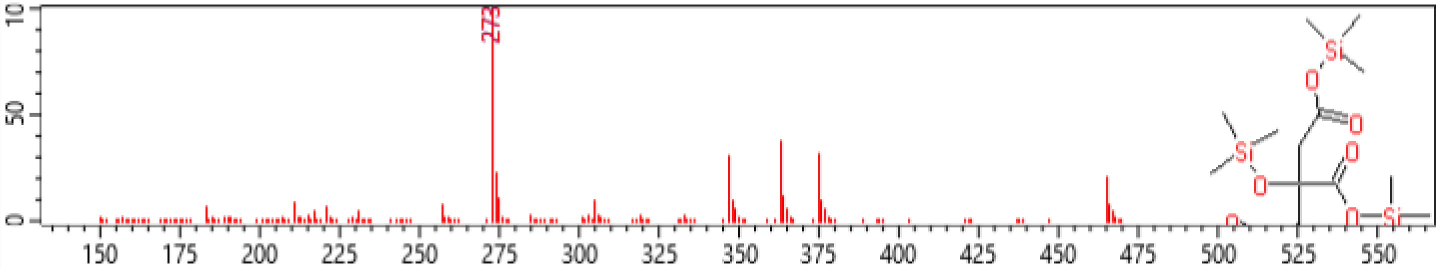


Citric acid, 4TMS derivative

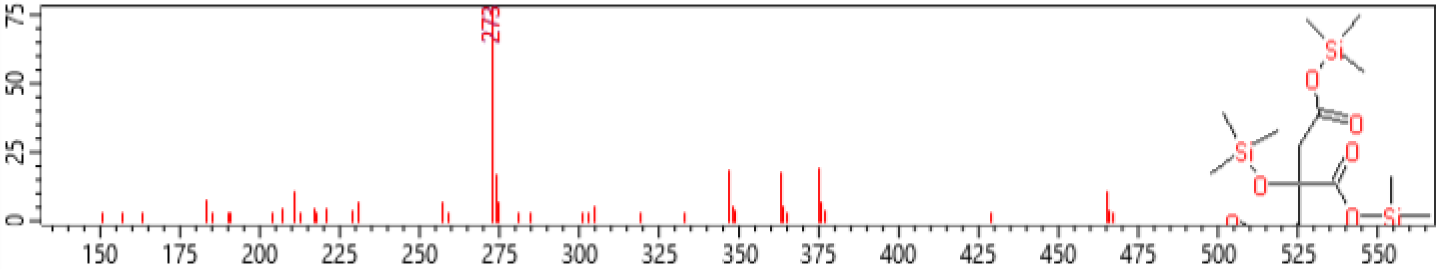


Citric acid, 4TMS derivative

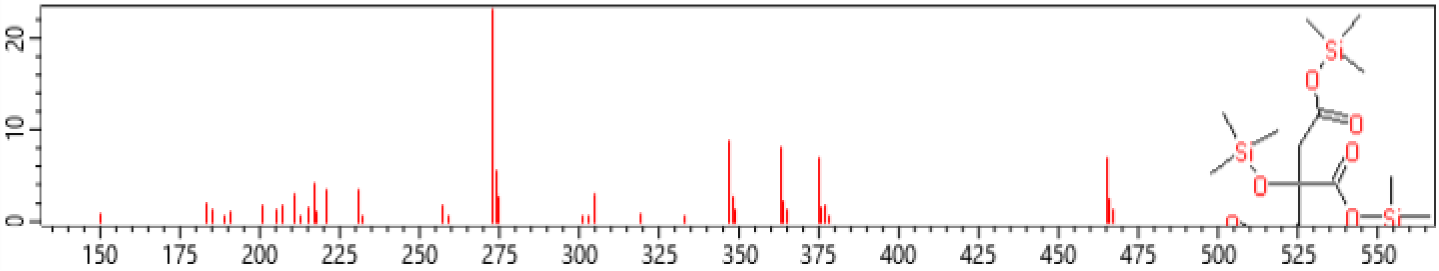

Protocatechoic acid, 3TMS derivative

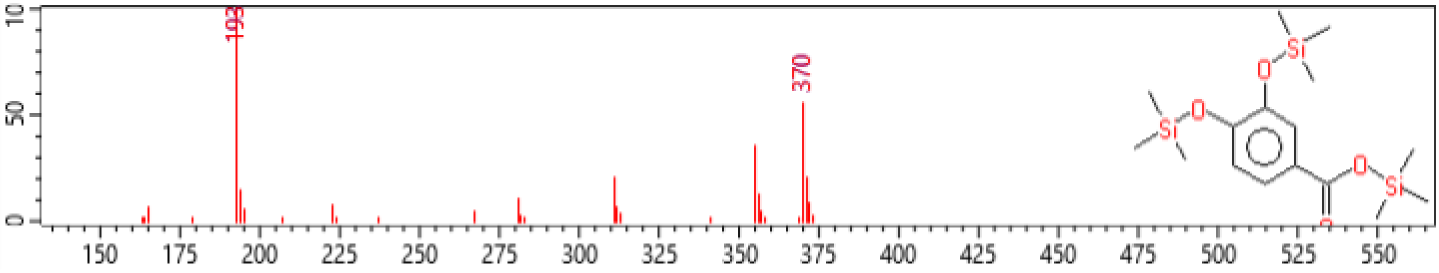

**2-Pentynoic acid, 4-[(t-butoxycarbonyl)amino]-(5S)-phenyl-, methyl ester**

Peak: 13.83 - 13.84 min (scan #1906 - #1908) Subtracted noise: 13.81 - 13.82 min (scan #1900 - #1902), 13.84 - 13.86 min (scan #19

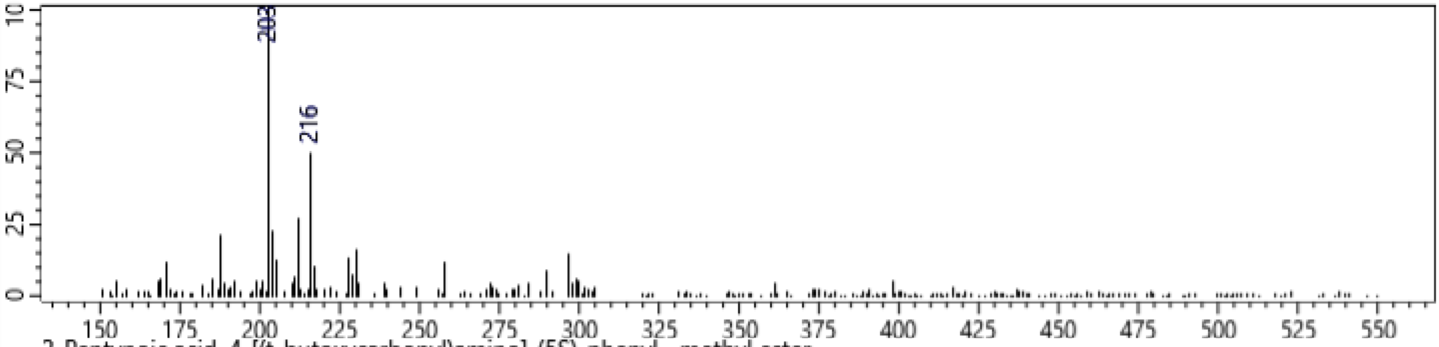


2-Pentynoic acid, 4-[(t-butoxycarbonyl)amino]-(5S)-phenyl-, methyl ester

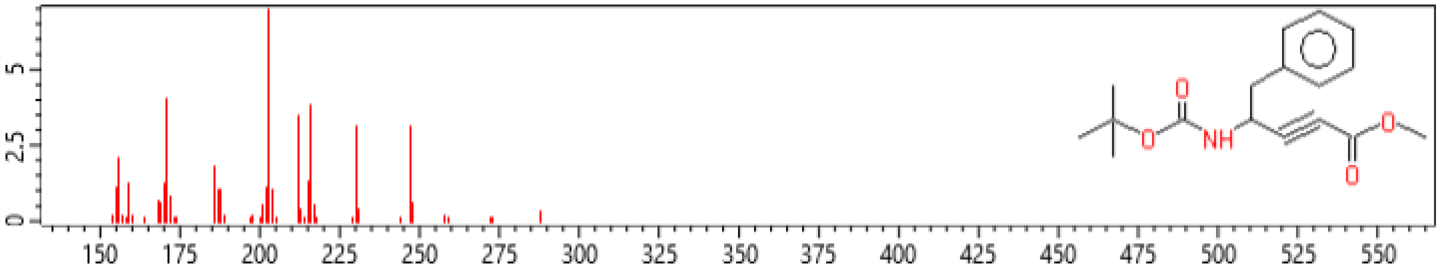

D-(+)-Glucosamine, 6TMS derivative

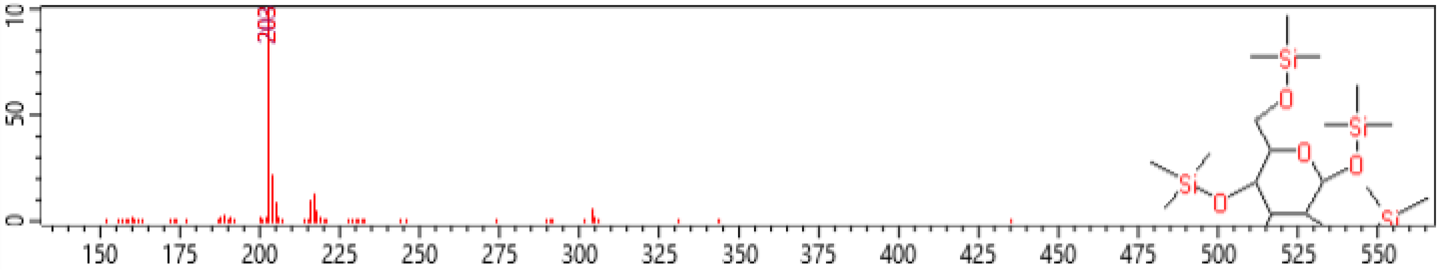


N-Acetylglutamine,N,N',O,O'- tetrakis(trimethylsilyl)-

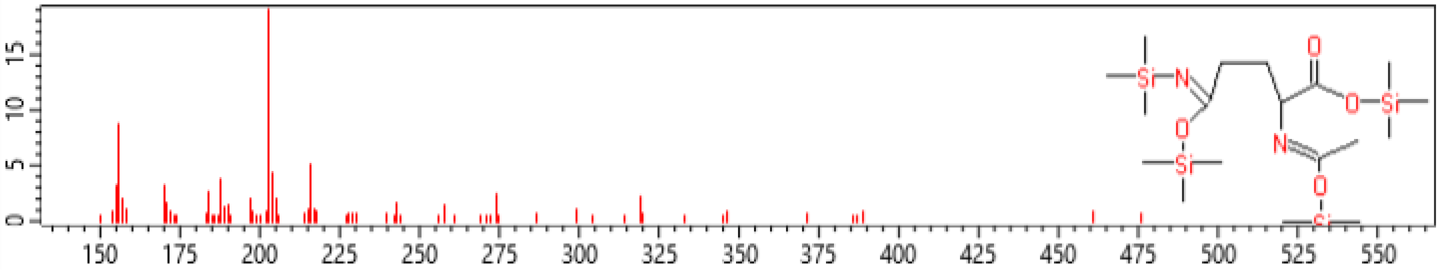

Octadecanamide, N-(2-methylpropyl)-N-nitroso-

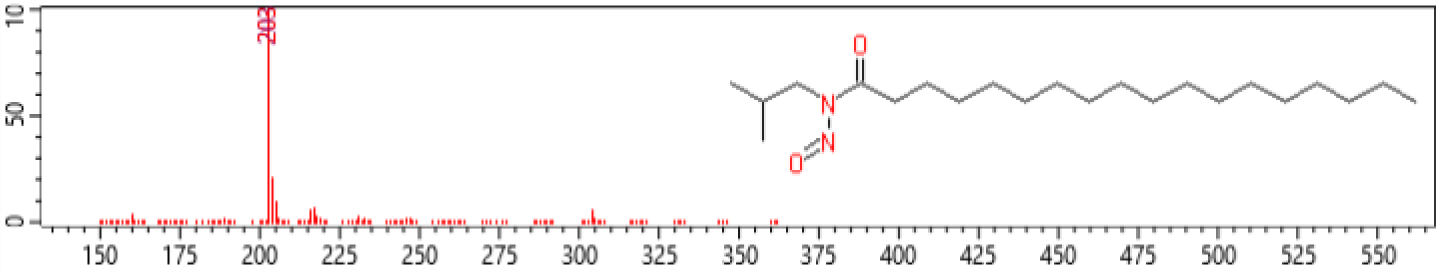

Olean-12-ene-3,28-diol, (3β)-

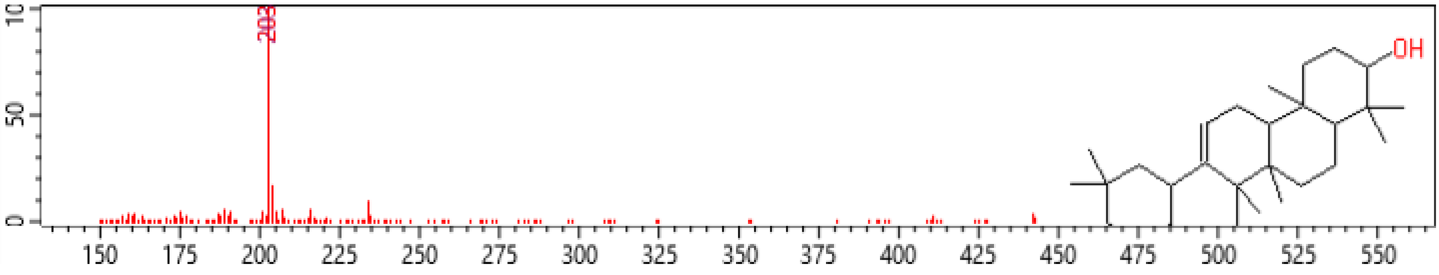


**Dimethyl 4-hydroxy-4-methyl-2-(3-nitrophenyl)-6-oxo-1,3-cyclohexanedicarboxylate, TMS derivative**

Peak: 13.91 - 13.93 min (scan #1928 - #1934) Subtracted noise: 13.87 - 13.89 min (scan #1917 - #1922), 13.93 - 13.96 min (scan #19

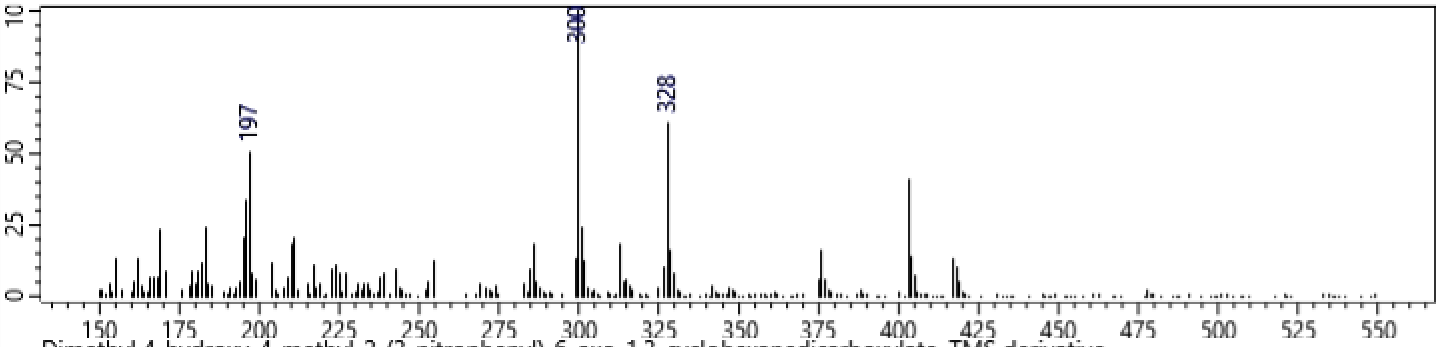


Dimethyl 4-hydroxy-4-methyl-2-(3-nitrophenyl)-6-oxo-1,3-cyclohexanedicarboxylate, TMS derivative

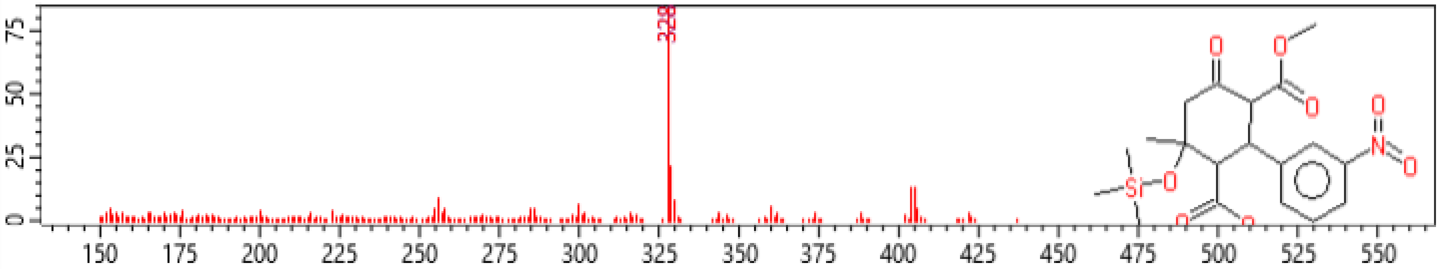


Gibbane-1,10-dicarboxylic acid, 2,3-epoxy-4a,7-dihydroxy-1-methyl-8-methylene-, 1,4a-lactone, 10-methyl ester, (1α,2β,3β,4aα,4bα,

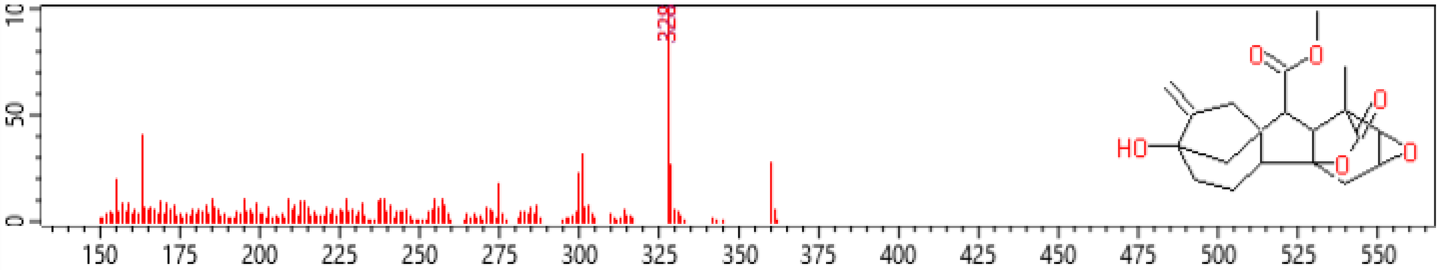


2',4,4',6'-Tetramethoxychalcone

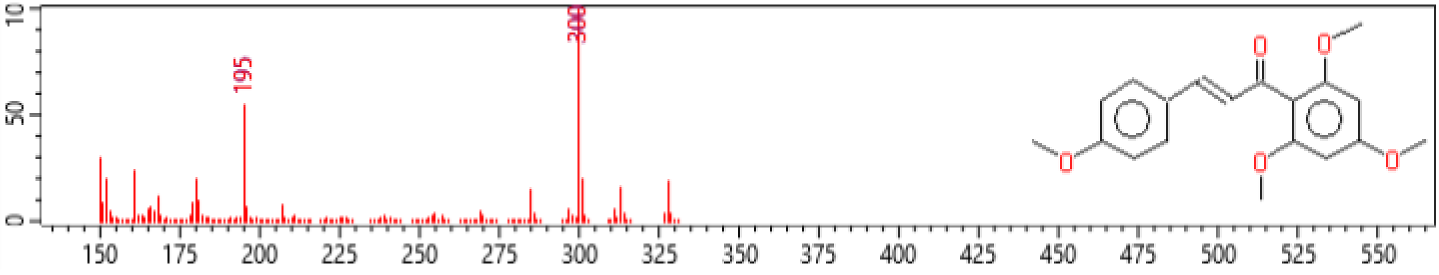

Gibberellic acid

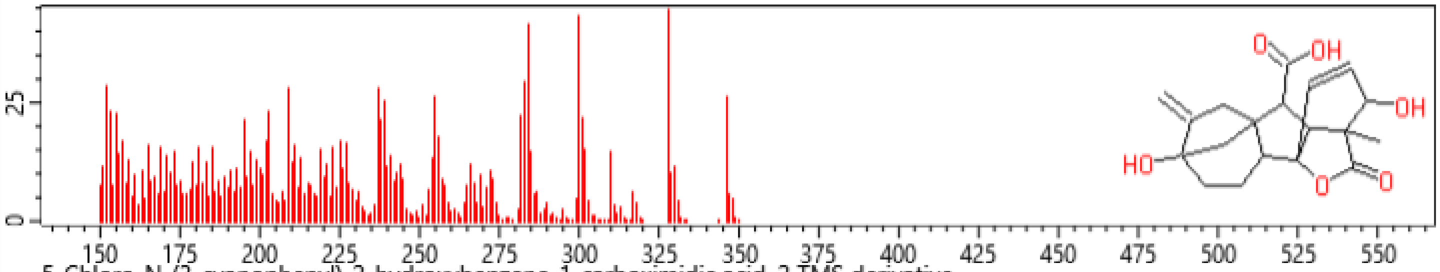


5-Chloro-N-(3-cyanophenyl)-2-hydroxybenzene-1-carboximidic acid, 2 TMS derivative

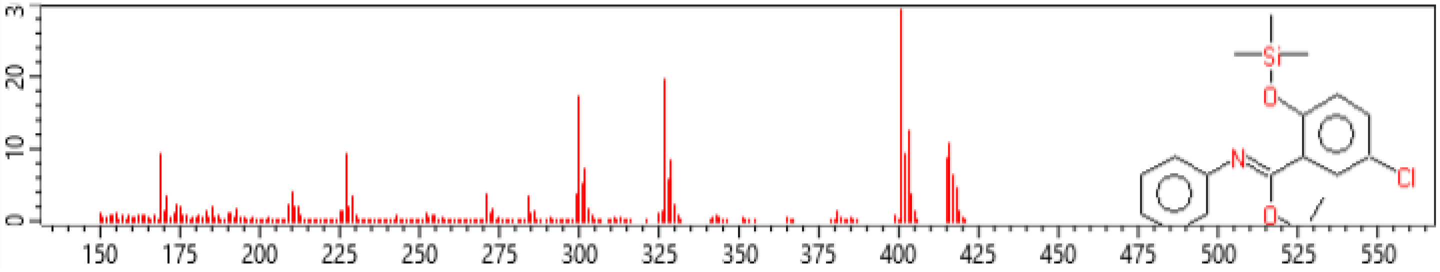


**Thiourea, N-phenyl-N'-(1-phenylethyl)-**

Peak: 13.93 - 13.96 min (scan #1935 - #1943) Subtracted noise: 13.91 - 13.93 min (scan #1927 - #1934), 14.00 - 14.03 min (scan #19

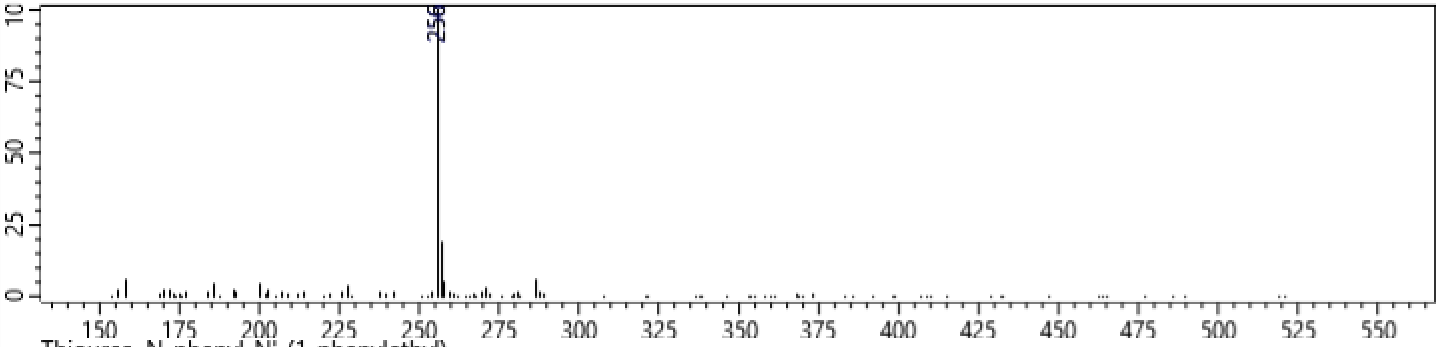


Thiourea, N-phenyl-N'-(1-phenylethyl)-

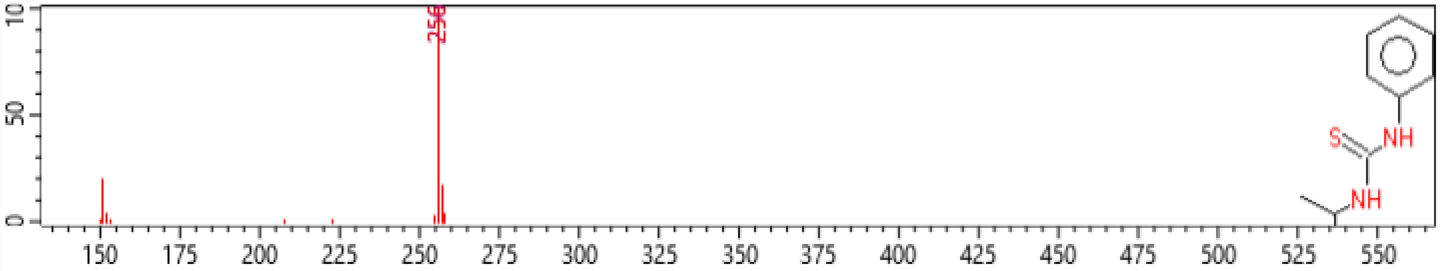


N-Methyl-N-(4-methoxyphenyl)-N'-phenyl-urea

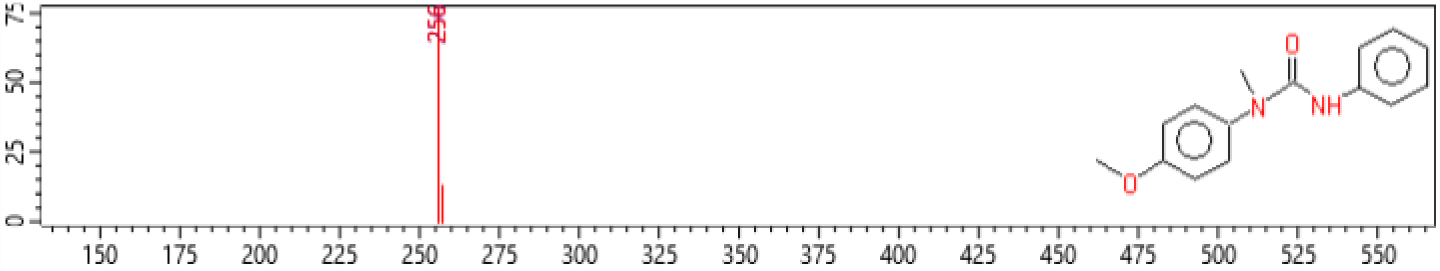

Carviolin

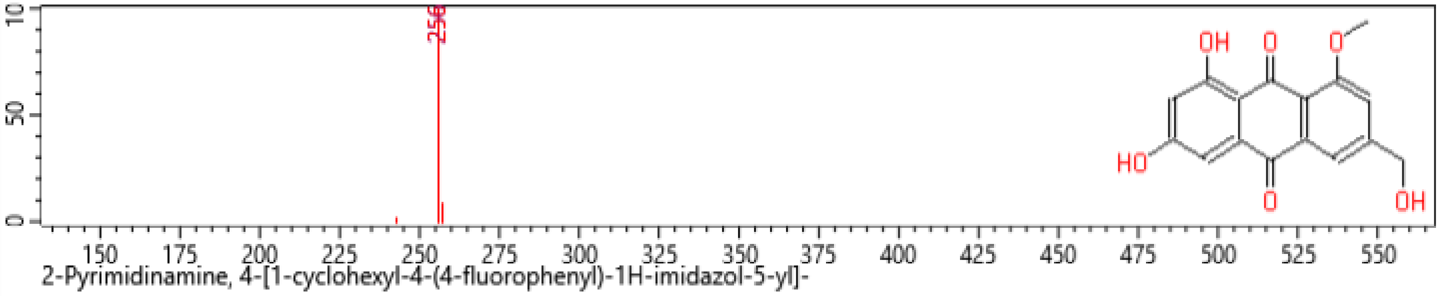


2-Pyrimidinamine, 4-[1-cyclohexyl-4-(4-fluorophenyl)-1H-imidazol-5-yl]-

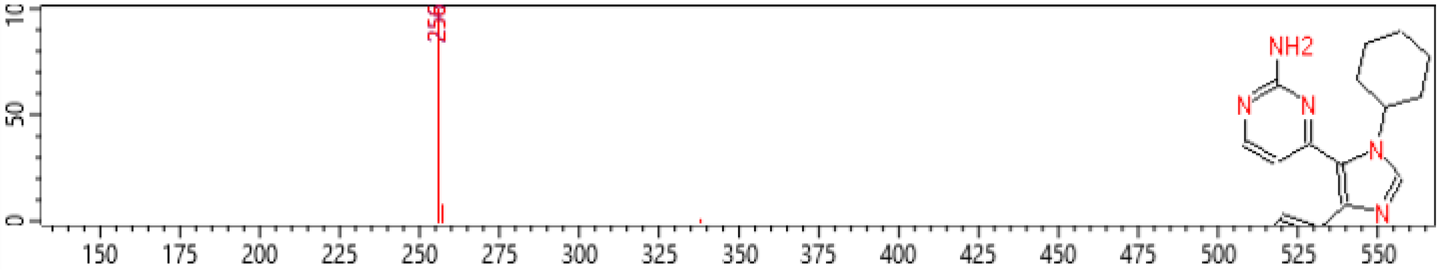


Olanzapine

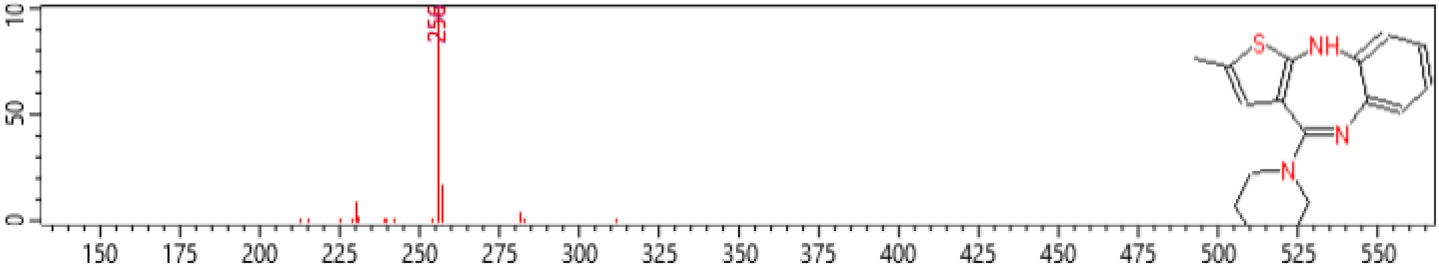


**Glycitin**

Peak: 14.00 - 14.05 min (scan #1954 - #1966) Subtracted noise: 13.96 - 14.00 min (scan #1942 - #1952), 14.08 - 14.12 min (scan #19

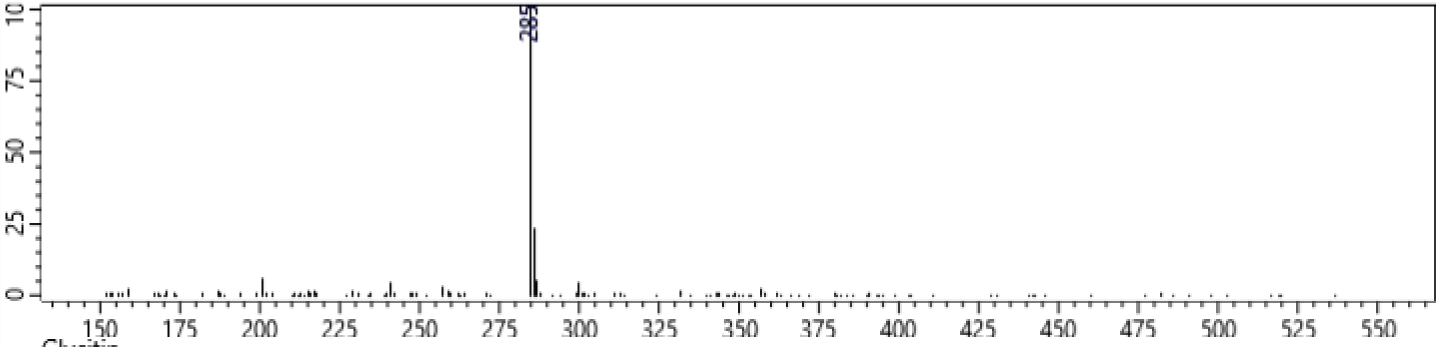


Glycitin

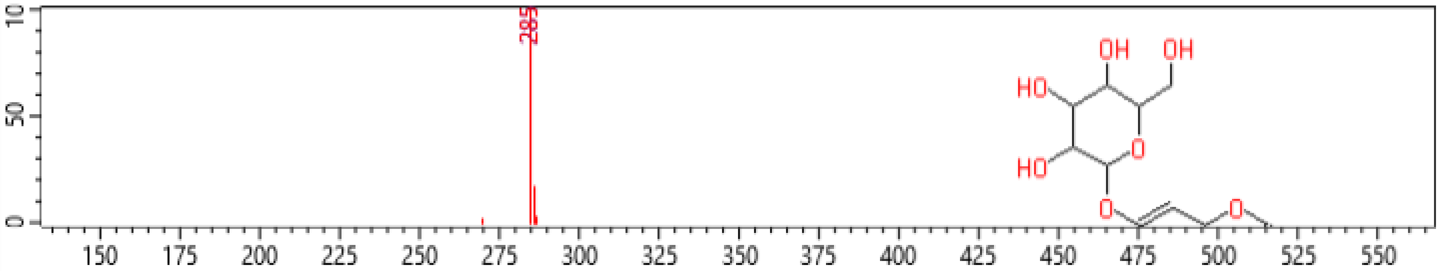

Glycitin

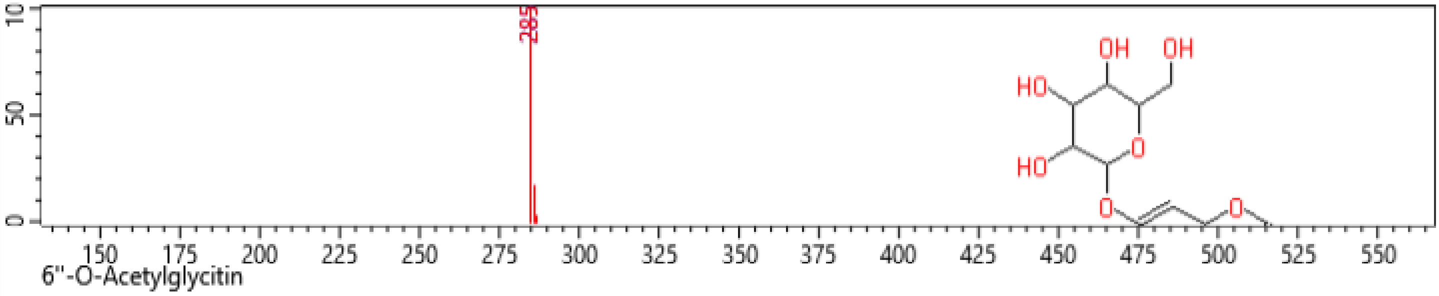


6''-O-Acetylglycitin

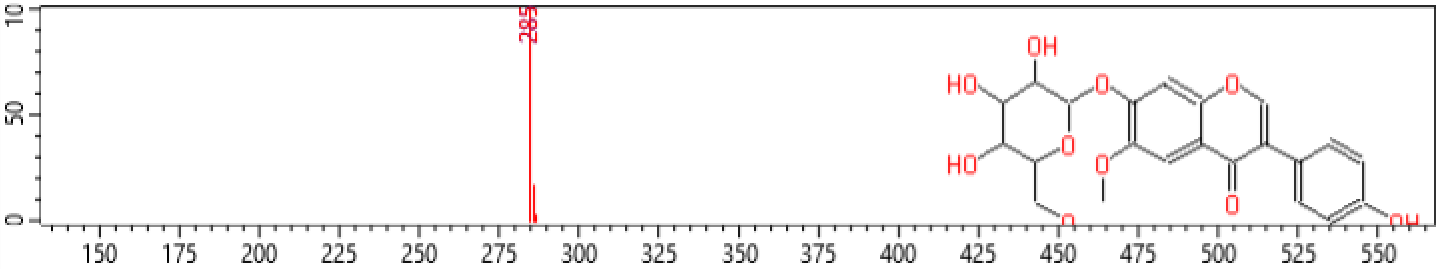


6''-O-Acetylglycitin

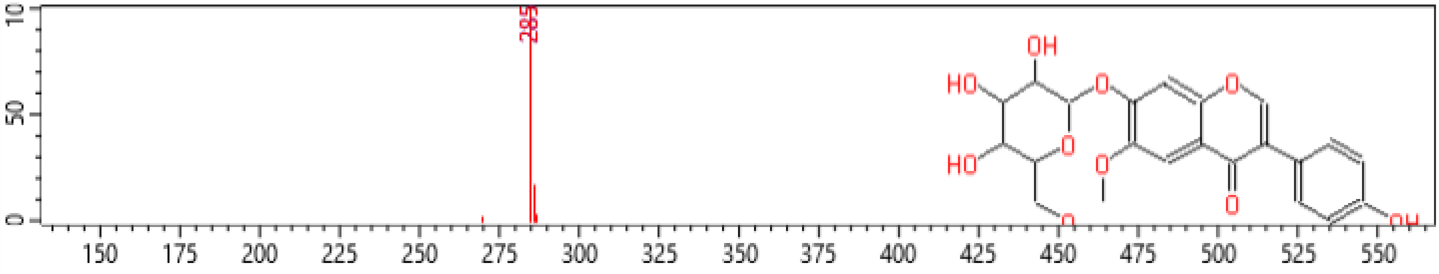


Acacetin

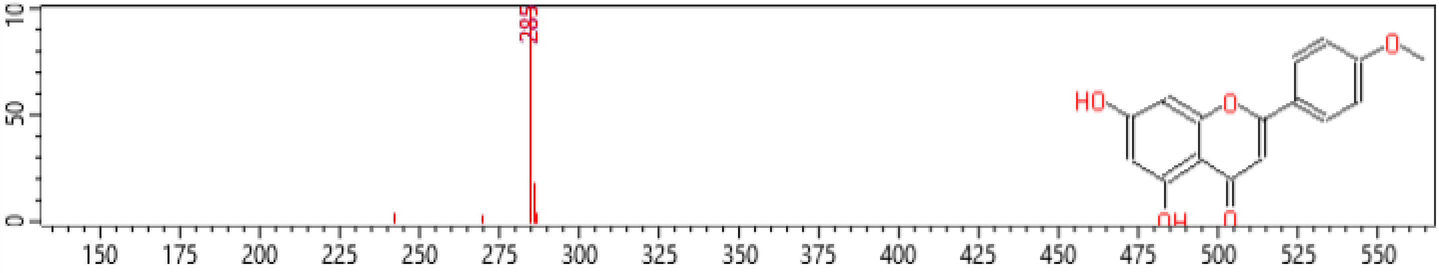


**Lormetazepam**

Peak: 14.10 - 14.13 min (scan #1980 - #1990) Subtracted noise: 14.05 - 14.08 min (scan #1967 - #1976), 14.17 - 14.20 min (scan #20

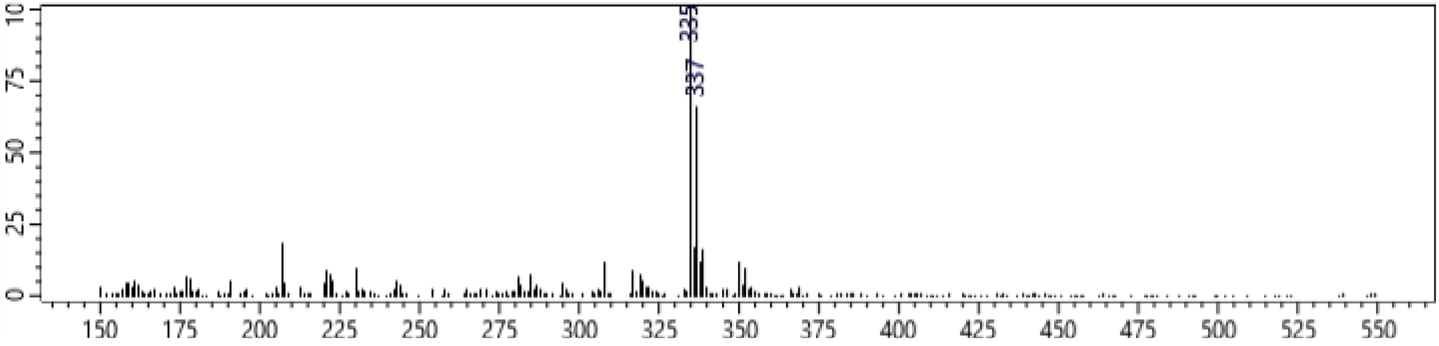

Lormetazepam

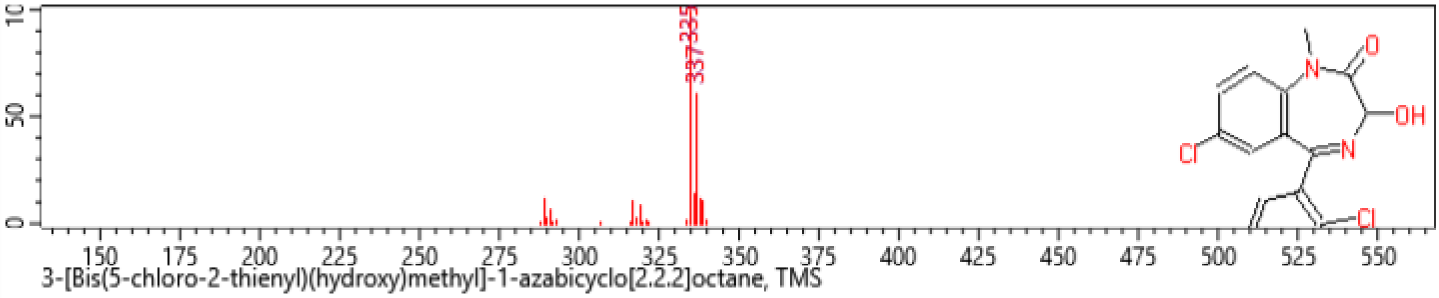


3-[Bis(5-chloro-2-thienyl)(hydroxy)methyl]-1-azabicyclo[2.2.2]octane, TMS

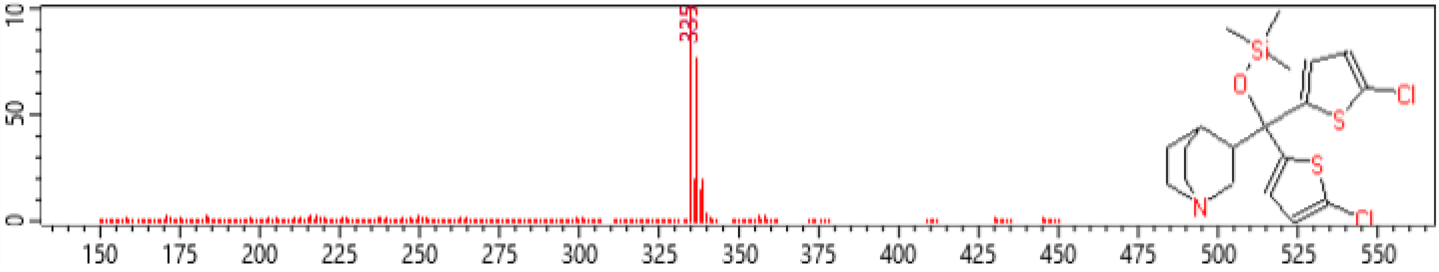


Aminopyralid, N,O-bis(trimethylsilyl)-

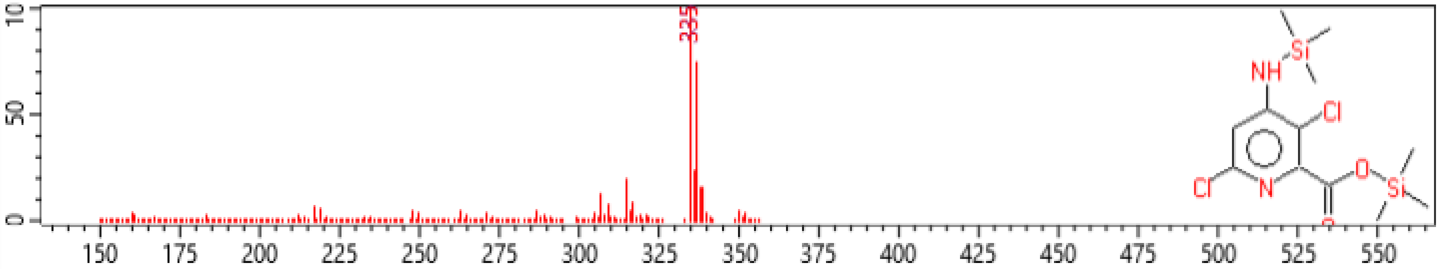


2-(3,4-Dichlorophenyl)-1,3-benzoxazol-5-amine, N-trimethylsilyl-

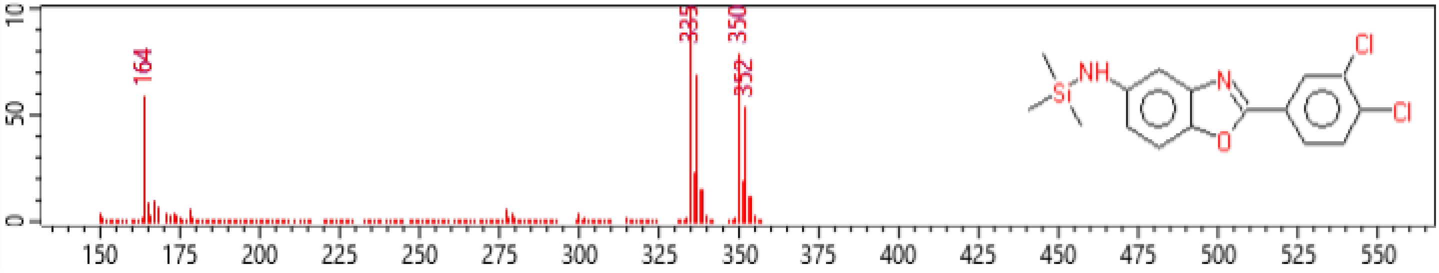


2-(2,5-Dichlorophenyl)-1,3-benzoxazol-5-amine, TMS derivative

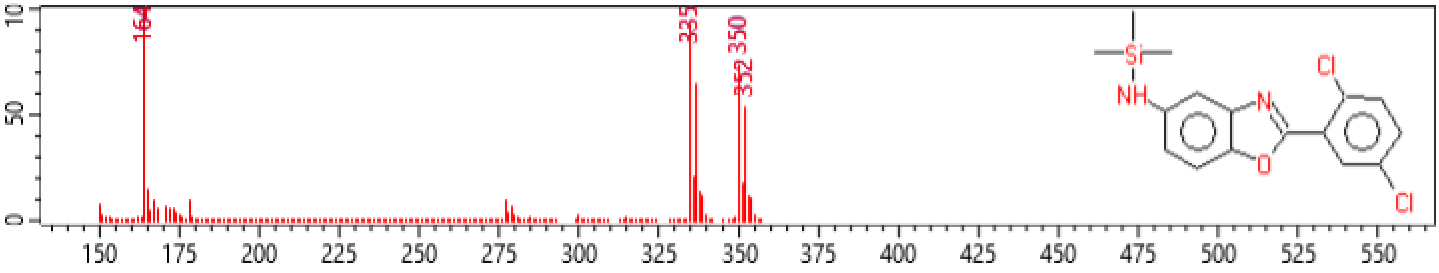

**13,14-Dihydro-15(R)-prostaglandin E1**

Peak: 14.22 - 14.25 min (scan #2015 - #2023) Subtracted noise: 14.17 - 14.20 min (scan #2002 - #2009), 14.26 - 14.29 min (scan #20

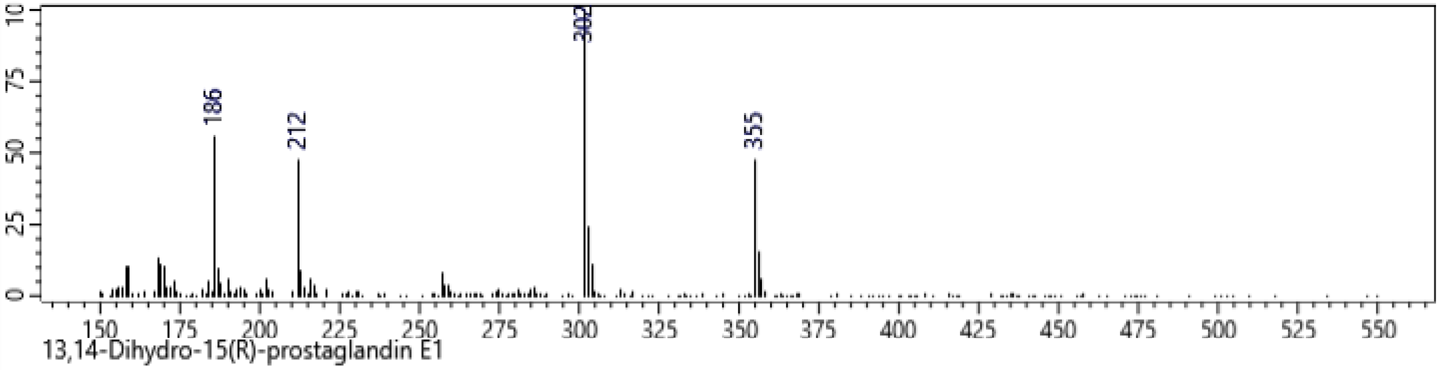


13,14-Dihydro-15(R)-prostaglandin E1

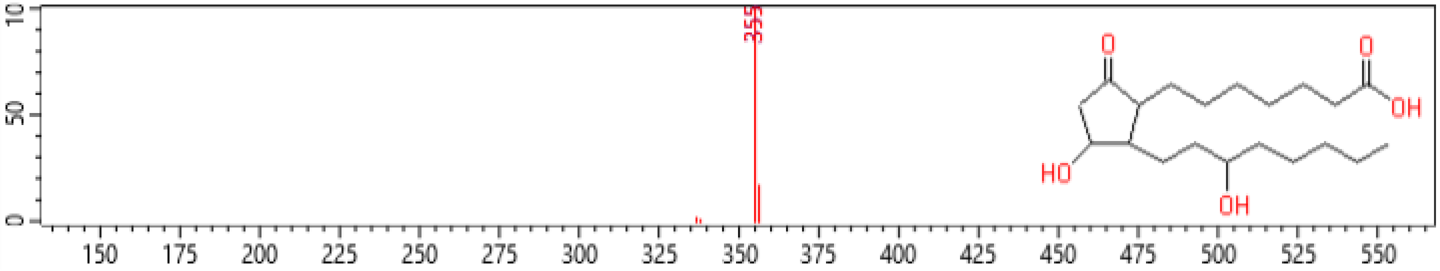


13,14-Dihydro-15(R)-prostaglandin E1

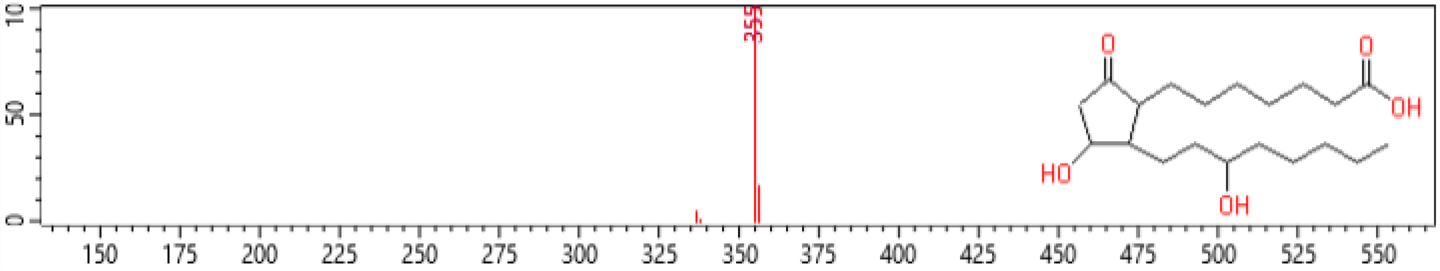


13,14-Dihydro-15(R)-prostaglandin E1

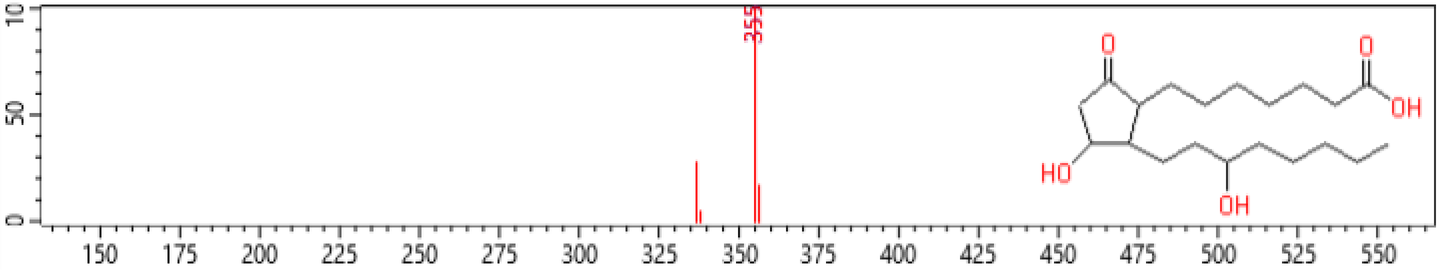


Leukotriene E4 methyl ester

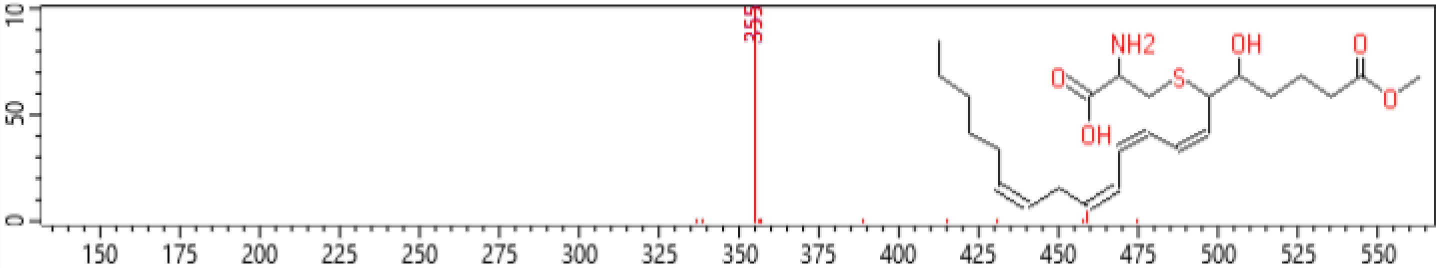

5-Heptenoic acid, 7-[(1R,4S,5S,6R)-6-[(1E,3S)-3-hydroxy-1-octenyl]-2-oxabicyclo[2.2.1]hept-5-yl]-, (5E)-

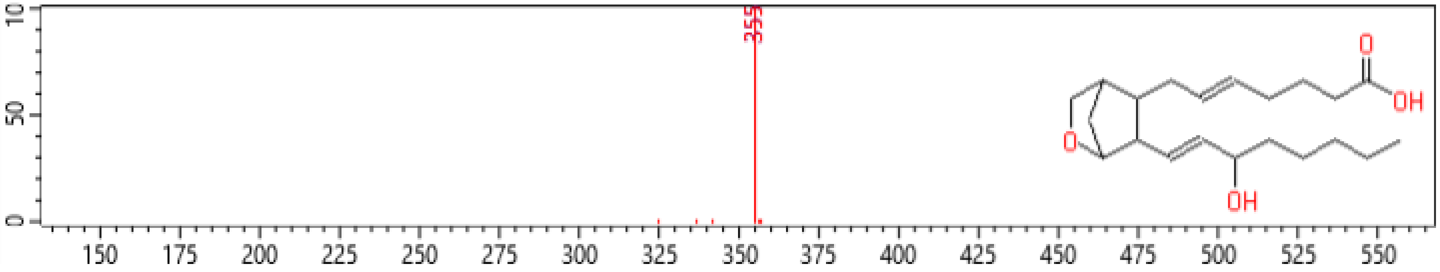


**1H-Indole-3-carboxaldehyde, 5-fluoro-1-(phenylmethyl)-**

Peak: 14.29 - 14.32 min (scan #2035 - #2043) Subtracted noise: 14.23 - 14.26 min (scan #2018 - #2026), 14.34 - 14.36 min (scan #20

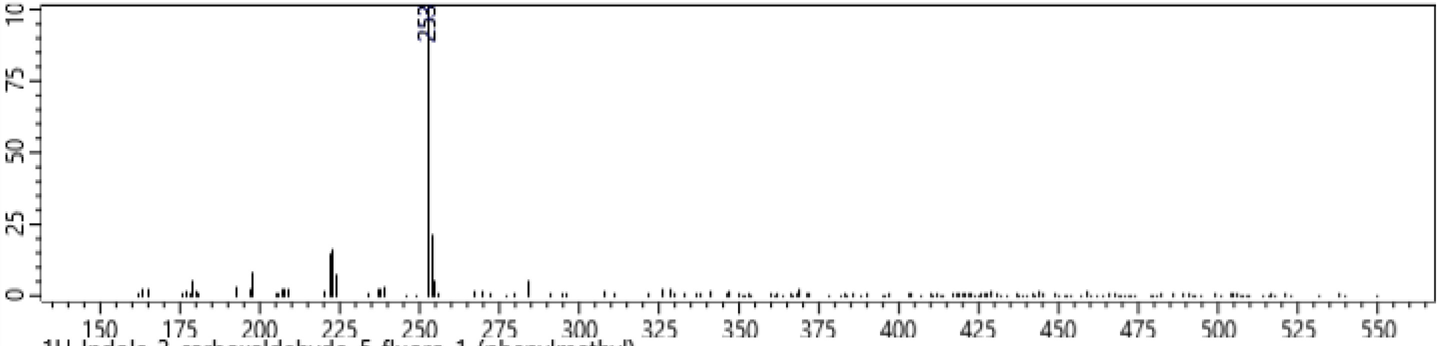


1H-Indole-3-carboxaldehyde, 5-fluoro-1-(phenylmethyl)-

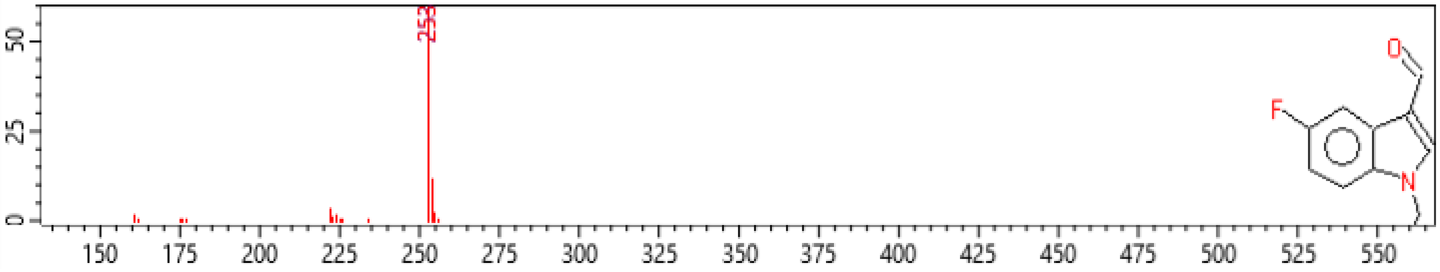


3-(2-Chlorophenyl)-2-thioxo-2,3-dihydro-4(1H)-quinazolinone

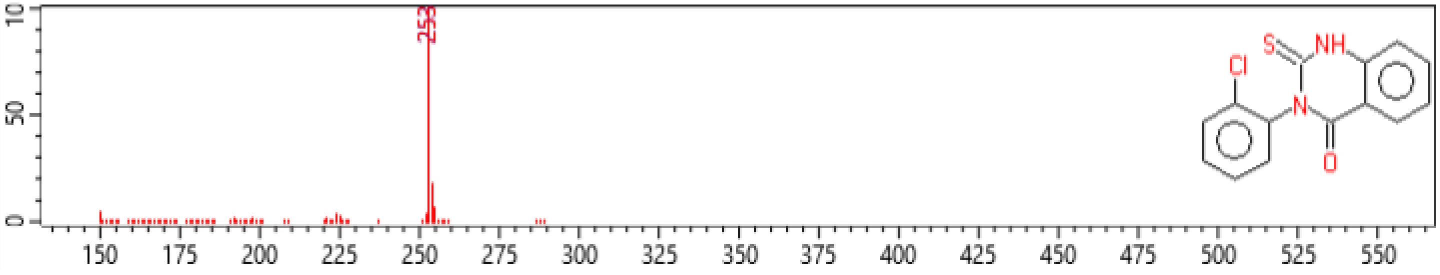


Benzenamine, 2,4,6-trimethyl-N-(2,4,6-trimethylphenyl)-

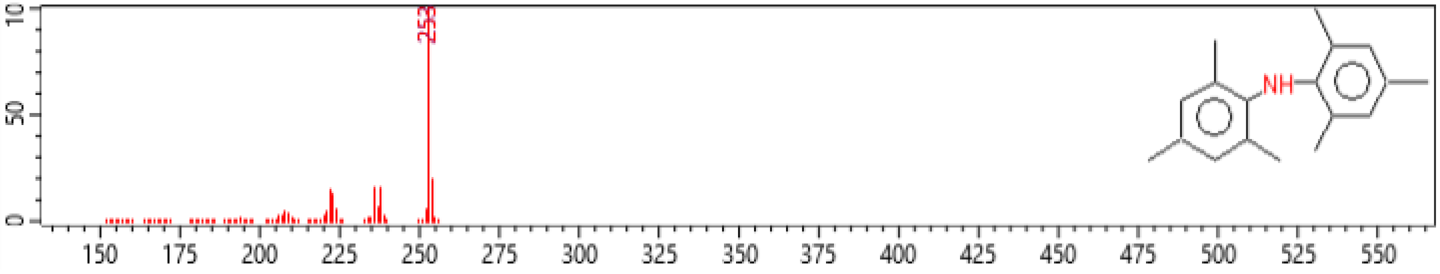

1H-Inden-5-ol, 2,3-dihydro-3-(4-hydroxyphenyl)-1,1,3-trimethyl-

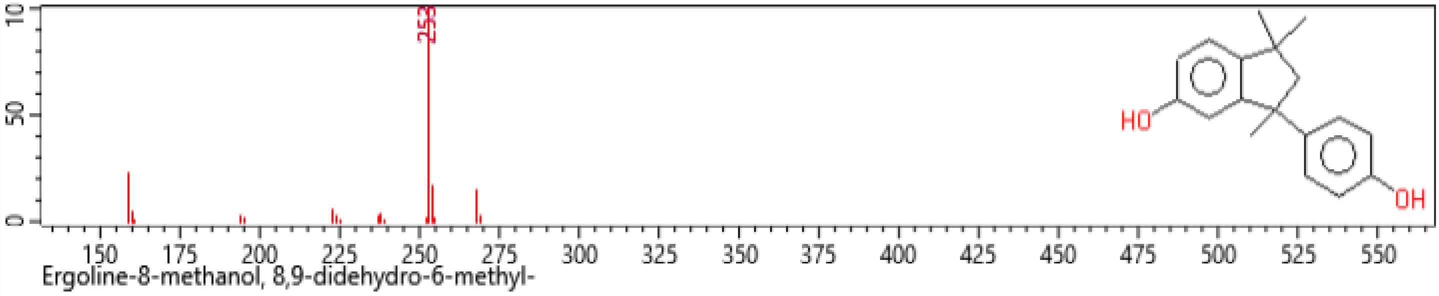


Ergoline-8-methanol, 8,9-didehydro-6-methyl-

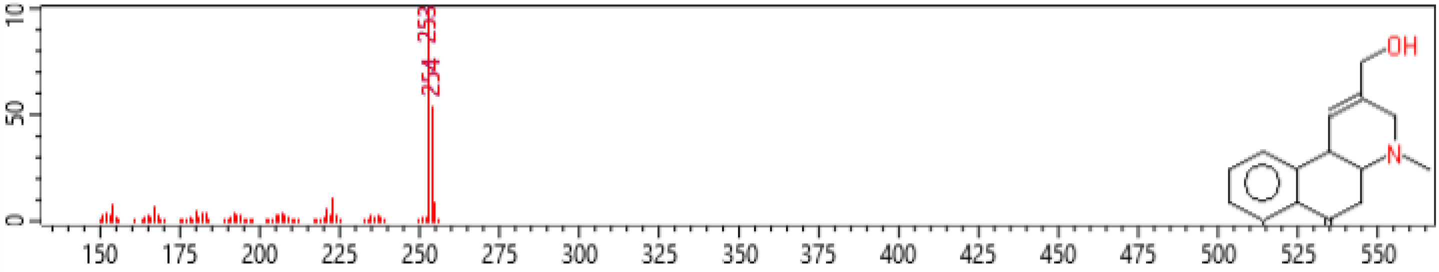


**Gomisin A**

Peak: 14.35 - 14.37 min (scan #2052 - #2058) Subtracted noise: 14.31 - 14.33 min (scan #2040 - #2046), 14.39 - 14.41 min (scan #20

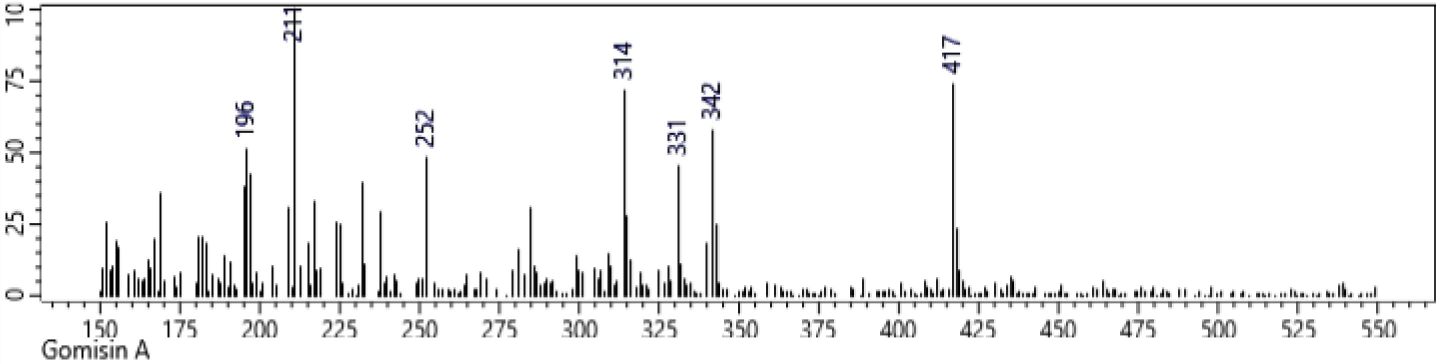


Gomisin A

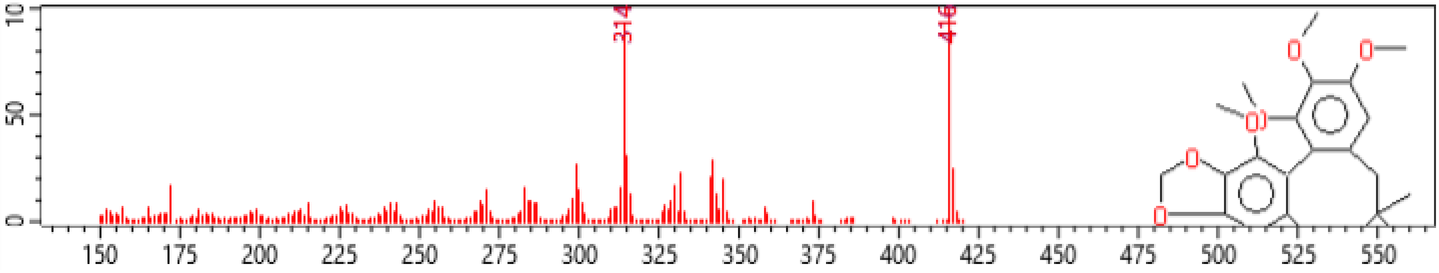


Acetic acid, 17-acetoxy-3-hydroxyimino-4,4,13-trimethyl-hexadecahydrocyclopenta[a]phenanthren-10-ylmethyl ester

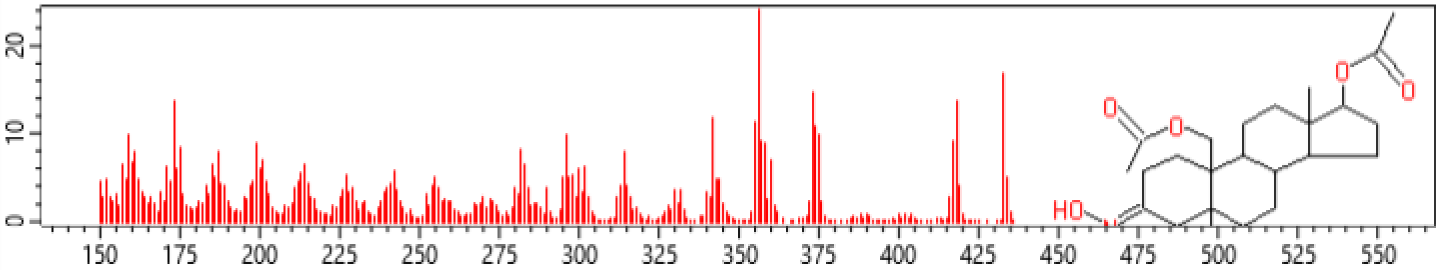

Androst-5-ene-3,11,17-triol, (3β,11β,17β)-, 3TMS derivative

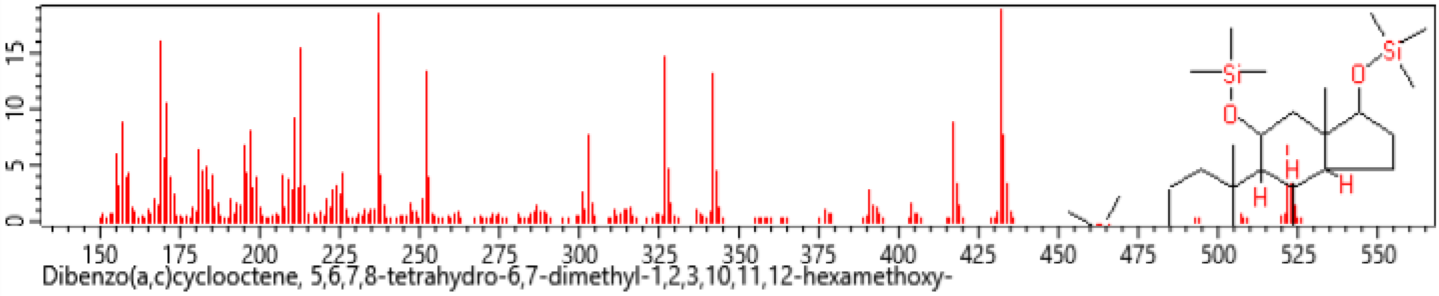

Dibenzo(a,c)cyclooctene, 5,6,7,8-tetrahydro-6,7-dimethyl-1,2,3,10,11,12-hexamethoxy-

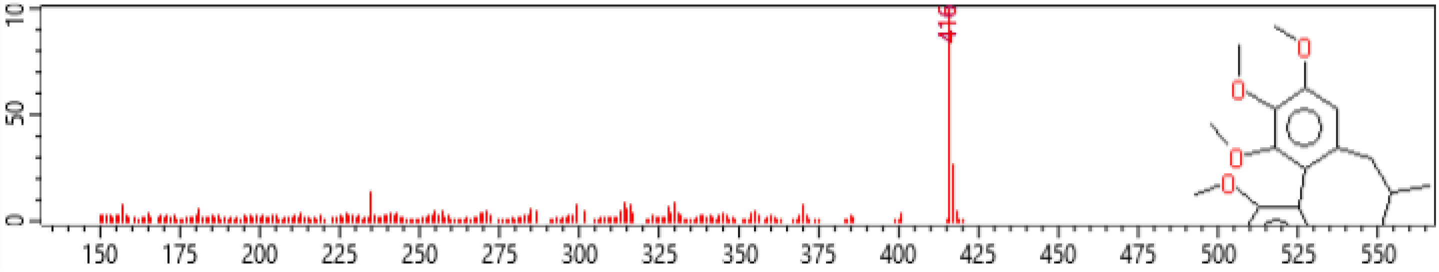


2,4a-Oxymethano-1,2,3,4,4a,4b,5,6,7,8,8a,9-dodecahydrophenanthren-9-one, 8-cyanomethyl-2-methoxy-7-methoxycarbonyl-1,1,7-t

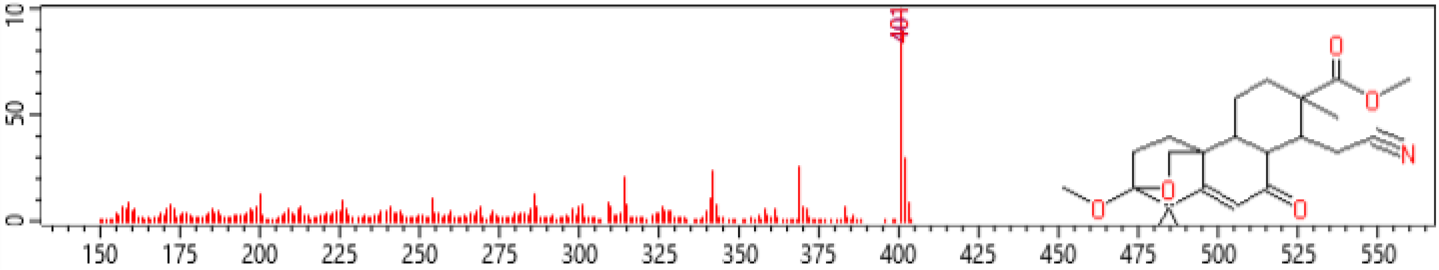


**Hexadecanenitrile**

Peak: 14.42 - 14.46 min (scan #2071 - #2081) Subtracted noise: 14.37 - 14.41 min (scan #2058 - #2067), 14.49 - 14.52 min (scan #20

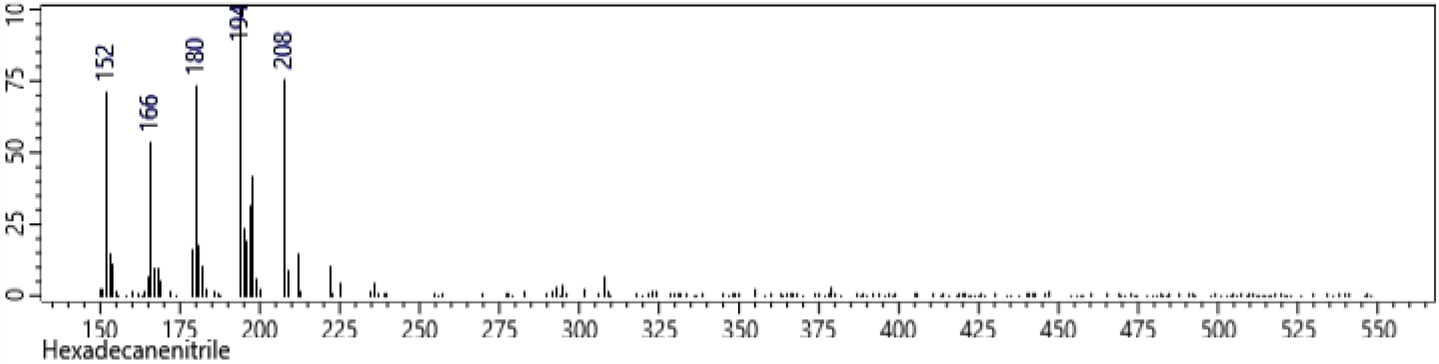


Hexadecanenitrile

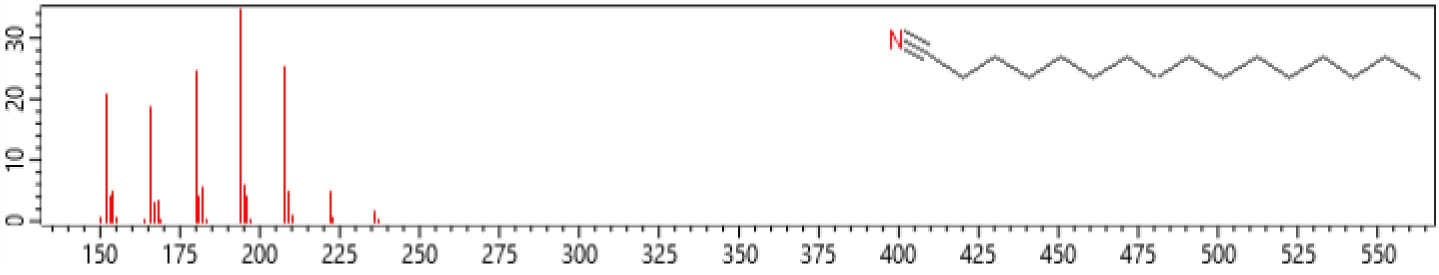

Hexadecanenitrile

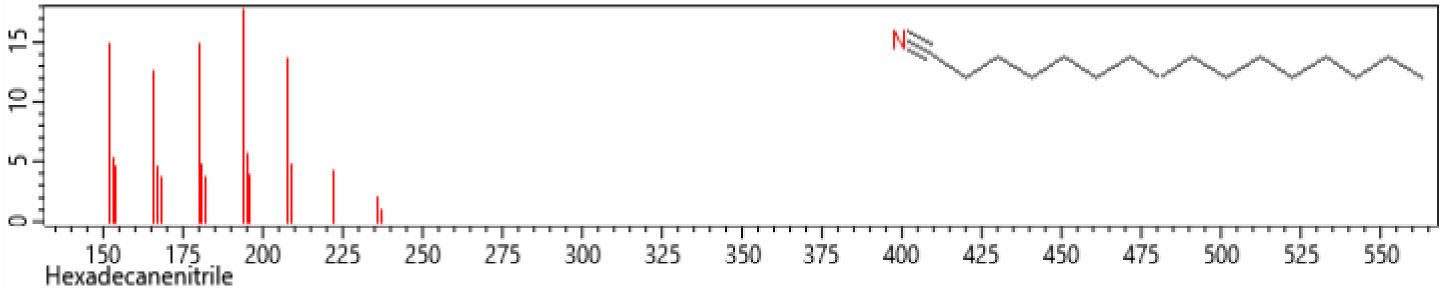

Hexadecanenitrile

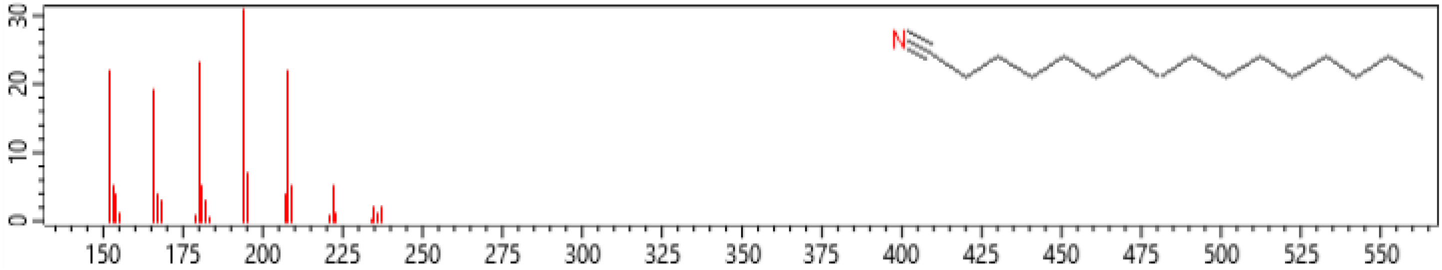

Oct-3-enoyl amide, N-allyl-N-propyl-

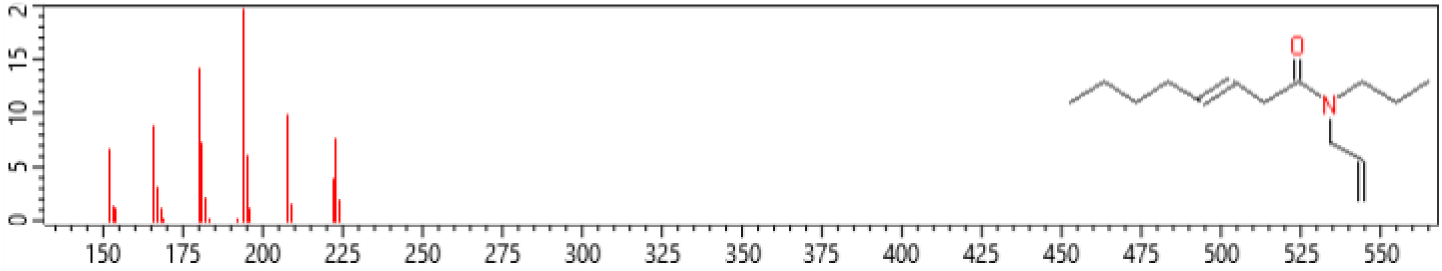

Pent-4-enoylamide, 2-methyl-N-allyl-N-hexyl-

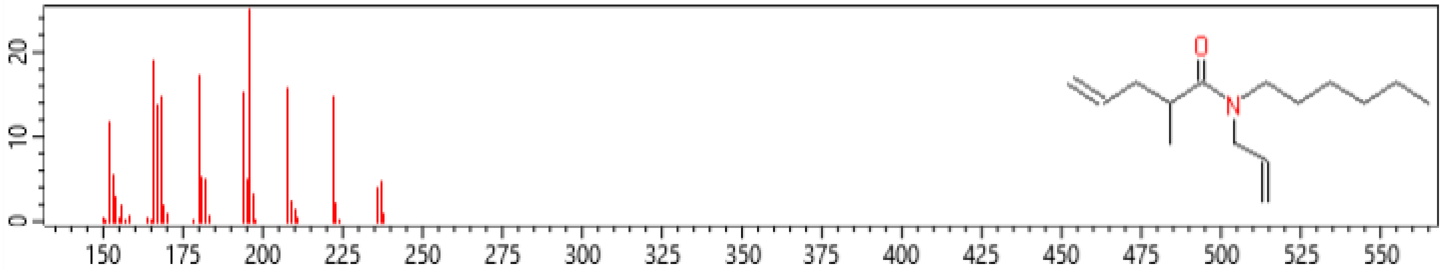

**7,9-Di-tert-butyl-1-oxaspiro(4,5)deca-6,9-diene-2,8-dione**

Peak: 14.52 - 14.55 min (scan #2099 - #2107) Subtracted noise: 14.45 - 14.48 min (scan #2080 - #2088), 14.55 - 14.58 min (scan #21

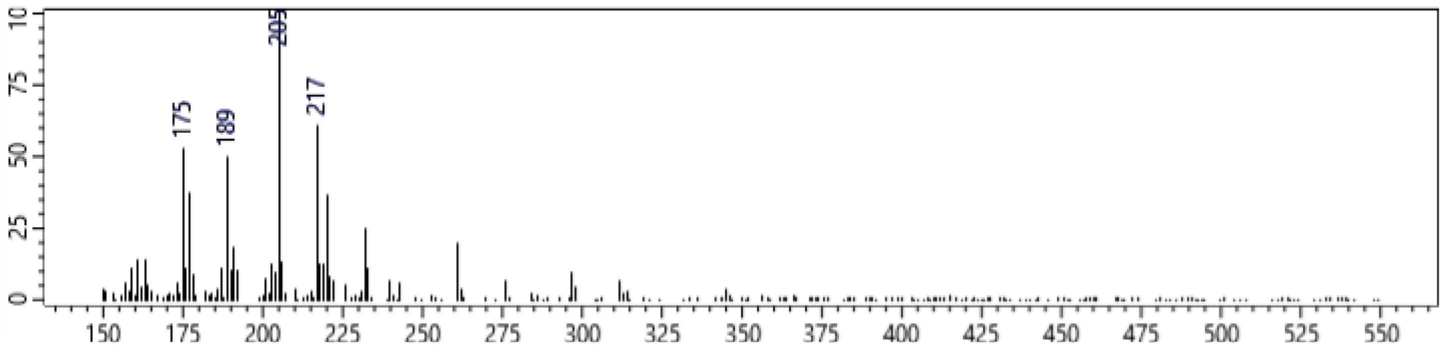

7,9-Di-tert-butyl-1-oxaspiro(4,5)deca-6,9-diene-2,8-dione

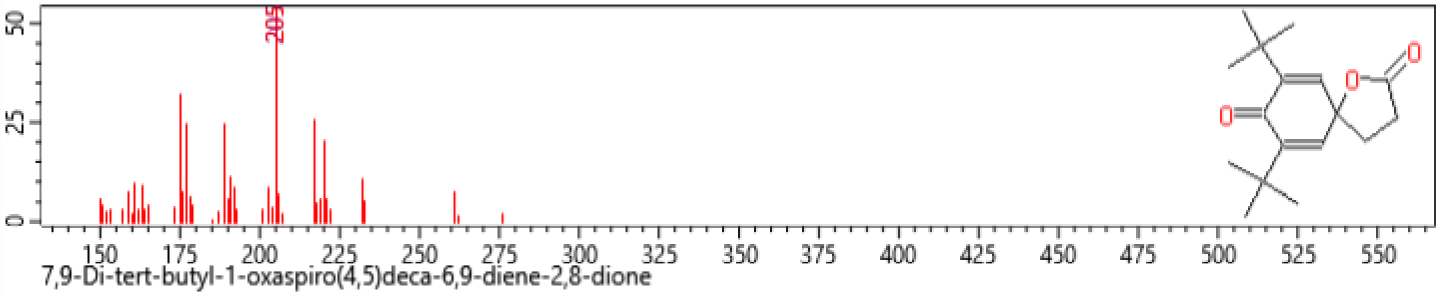


7,9-Di-tert-butyl-1-oxaspiro(4,5)deca-6,9-diene-2,8-dione

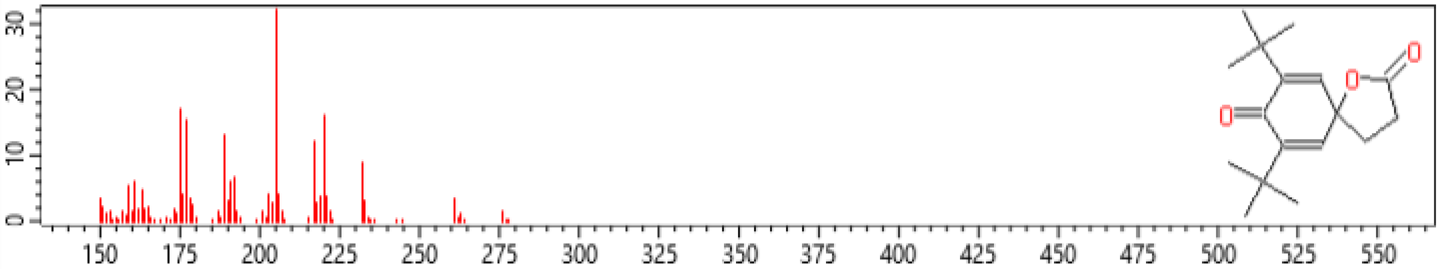

7,9-Di-tert-butyl-1-oxaspiro(4,5)deca-6,9-diene-2,8-dione

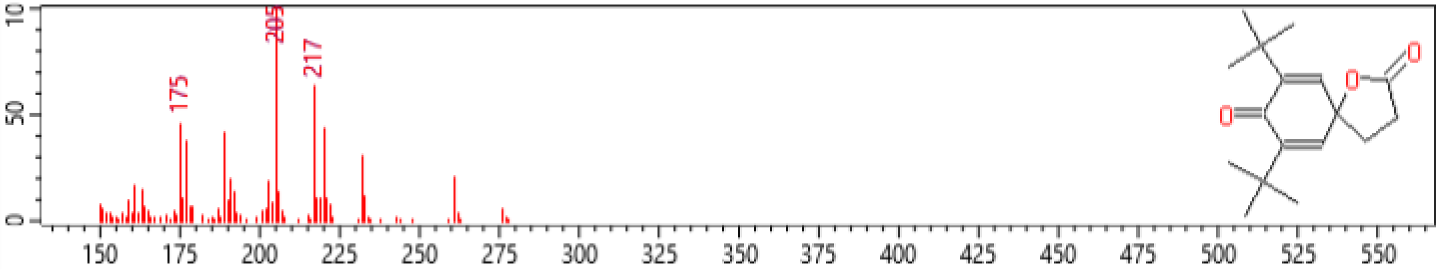


1-Methyl-1-phenylmethoxy-1-silacyclohexane

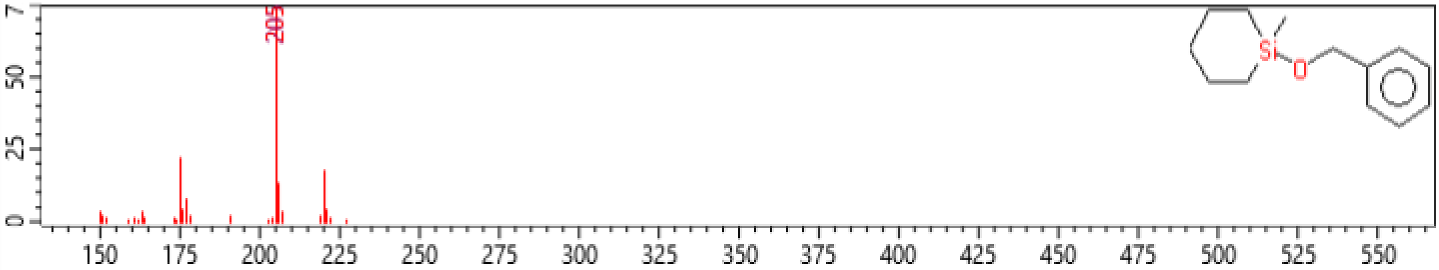


4H-1-Benzopyran-4-one, 5-hydroxy-2-methyl-7-(2-propenyloxy)-

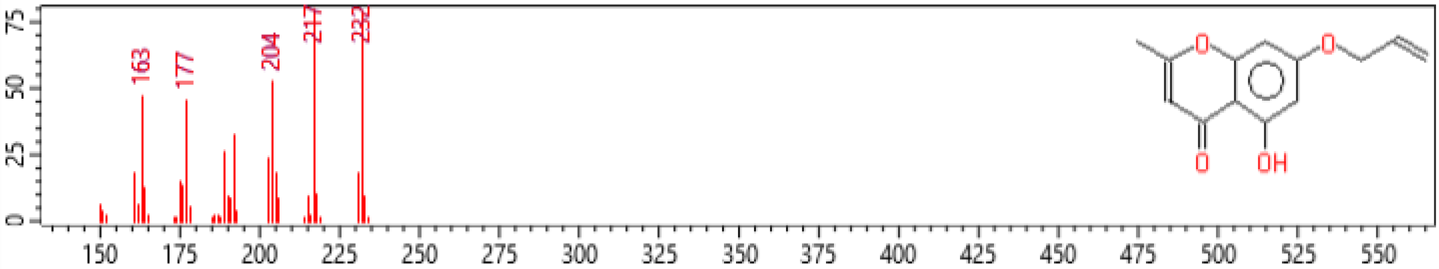

**Pyrano[4,3-b]pyrane-3-carbonitrile, 4,5-dihydro-2-amino-7-methyl-5-oxo-4-phenyl-**

Peak: 14.58 - 14.62 min (scan #2115 - #2125) Subtracted noise: 14.52 - 14.55 min (scan #2098 - #2107), 14.62 - 14.66 min (scan #21

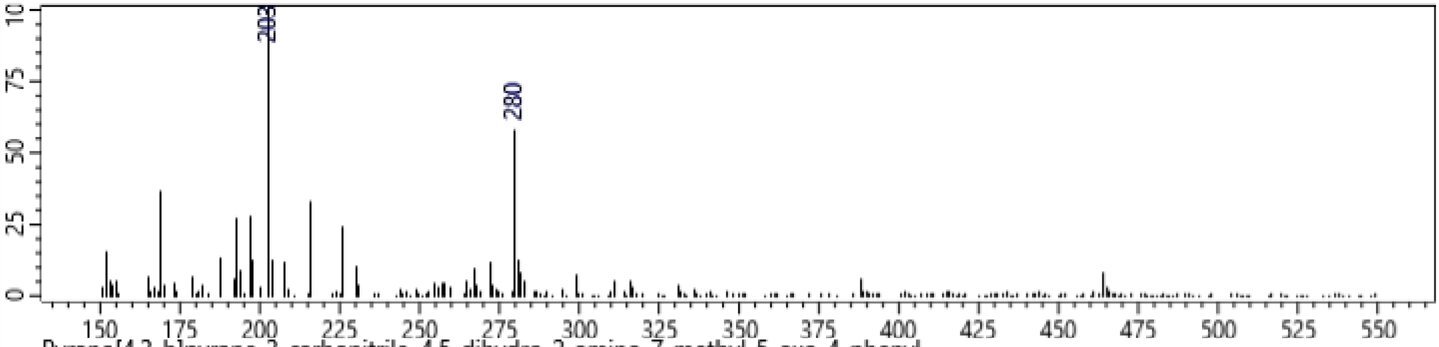


Pyrano[4,3-b]pyrane-3-carbonitrile, 4,5-dihydro-2-amino-7-methyl-5-oxo-4-phenyl-

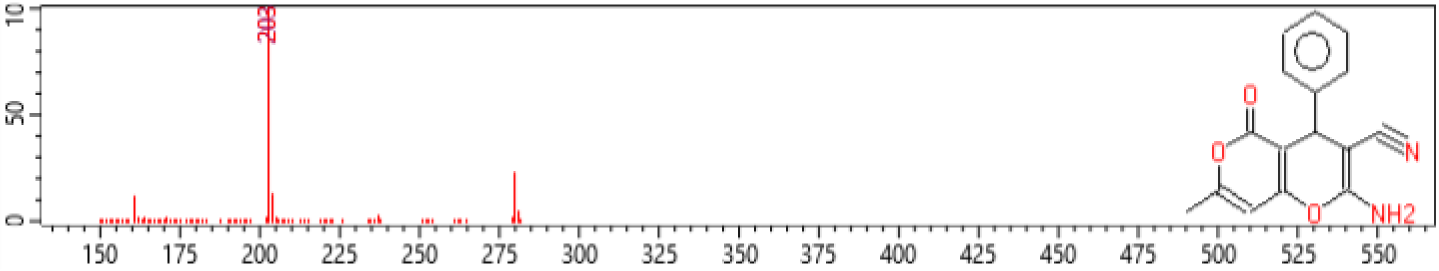


2-Thienyl-diphenylmethylsilane

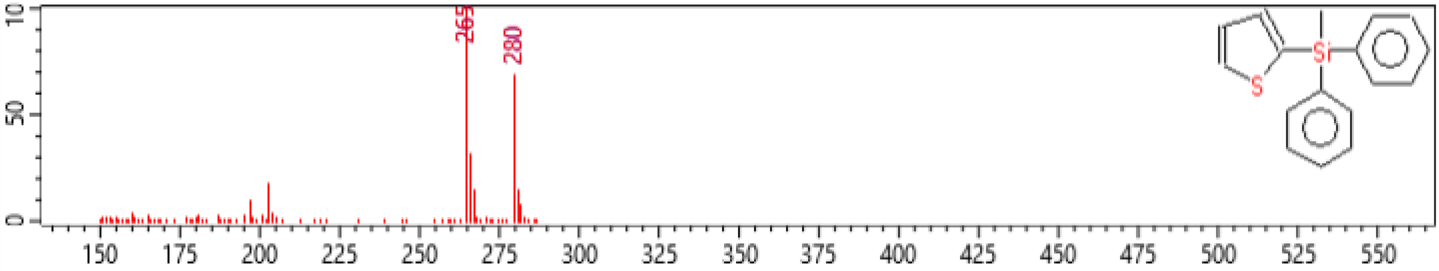


2-Oxo-5-methoxy-4,6-diphenyl-1,2,3,4-tetrahydropyrimidine

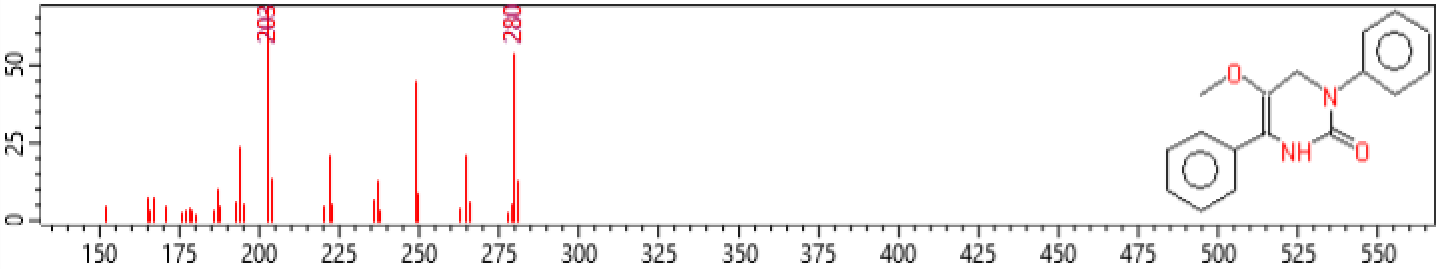


3,5-Pyrazolidinedione, 1-phenyl-4-(phenylazo)-

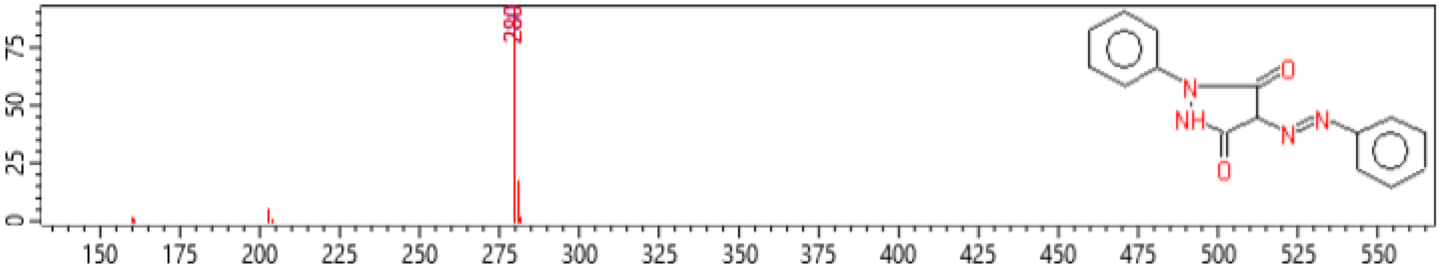

8-Amino-6-methoxy-4-methyl-5-phenoxyquinoline

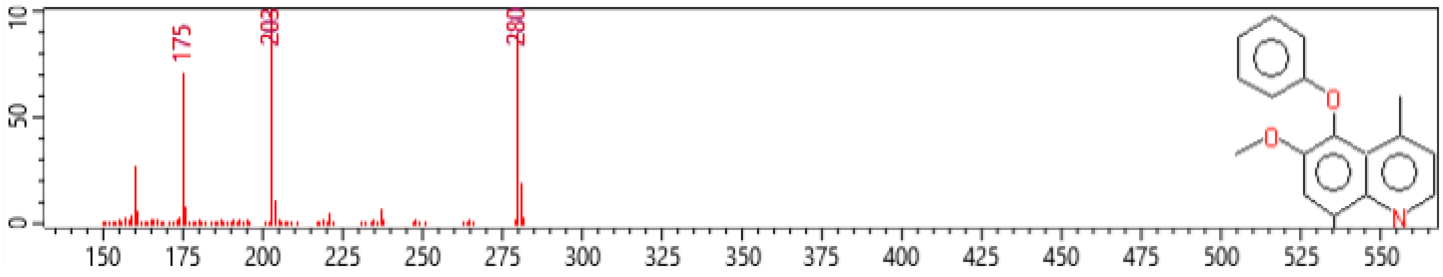


**(5R,6R,7R,8R)-2,2,11,11-Tetramethyl-6,8-bis((trimethylsilyl)oxy)-3,10-dioxa-2,11-disiladodecane-5,7-diol**
Peak: 14.66 - 14.69 min (scan #2137 - #2147) Subtracted noise: 14.59 - 14.62 min (scan #2117 - #2126), 14.70 - 14.74 min (scan #21

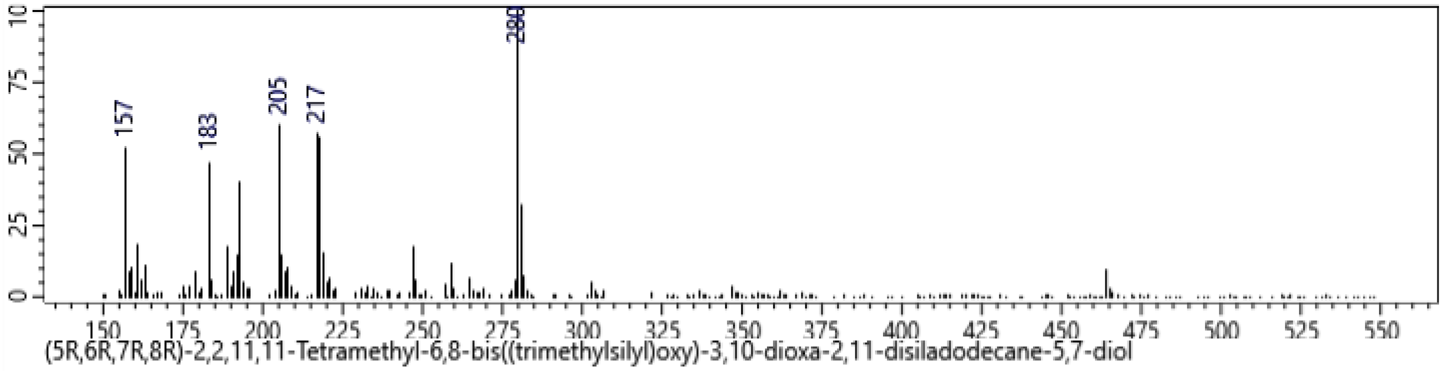


(5R,6R,7R,8R)-2,2,11,11-Tetramethyl-6,8-bis((trimethylsilyl)oxy)-3,10-dioxa-2,11-disiladodecane-5,7-diol

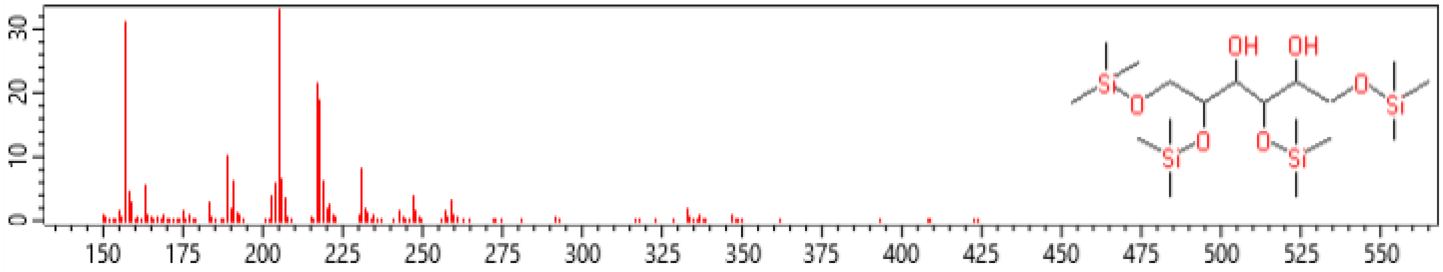


(2R,3R,4R,5S)-Hexane-1,2,3,4,5,6-hexaol, 4TMS

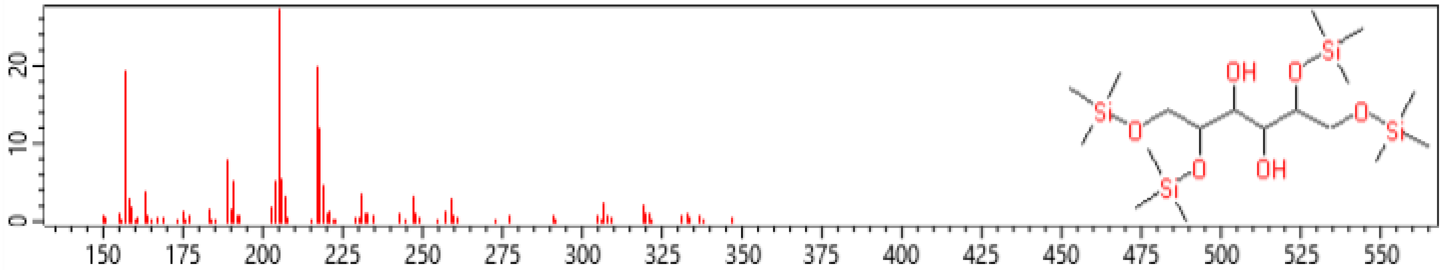


L-Tyrosinamide, 3TMS derivative

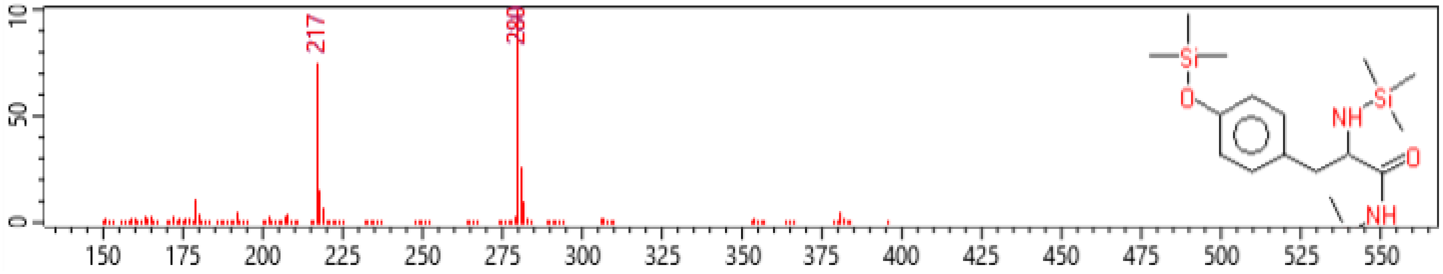

5-(3-Methoxyphenyl)-1,3,4-oxadiazole-2-thiol, TMS derivative

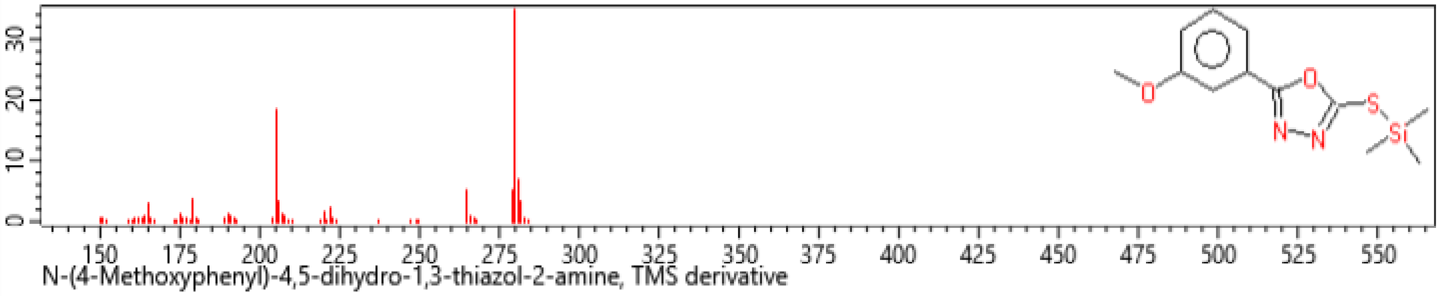


N-(4-Methoxyphenyl)-4,5-dihydro-1,3-thiazol-2-amine, TMS derivative

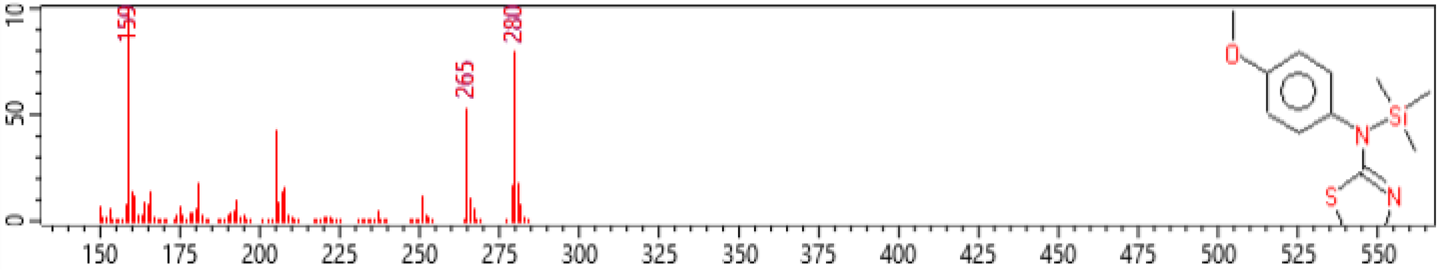


**3-(4-Bromophenyl)-9-phenyl-9H-carbazole**

Peak: 14.71 - 14.74 min (scan #2152 - #2160) Subtracted noise: 14.67 - 14.70 min (scan #2141 - #2148), 14.76 - 14.79 min (scan #21

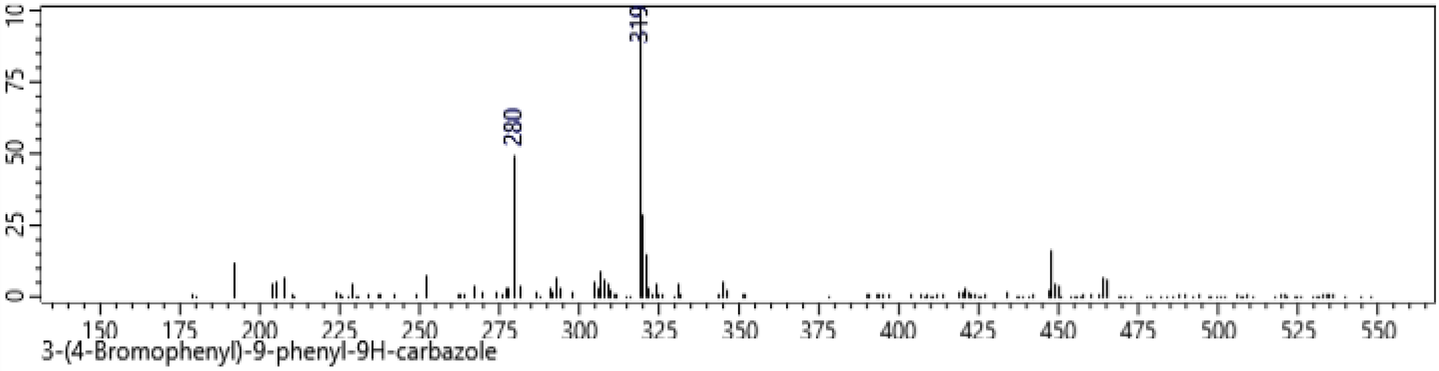


3-(4-Bromophenyl)-9-phenyl-9H-carbazole

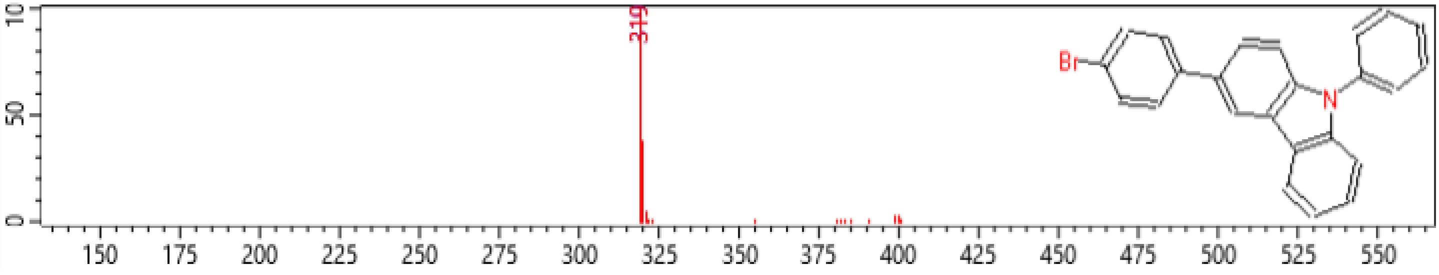


3-(4-Bromophenyl)-9-phenyl-9H-carbazole

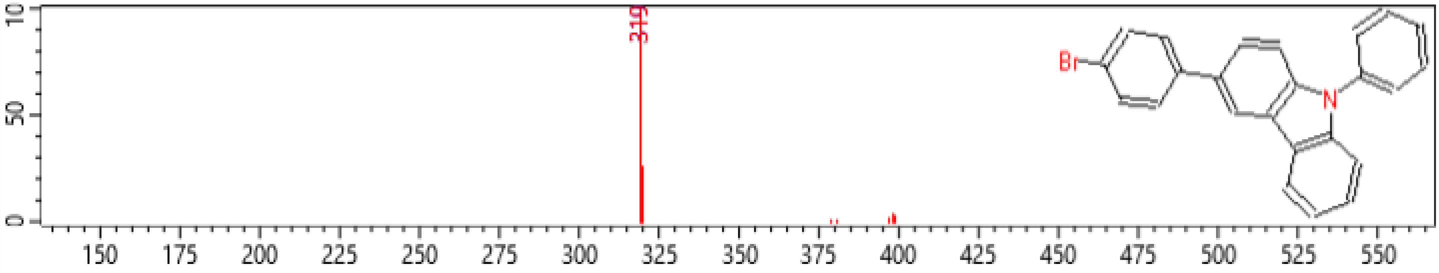

3-Bromo-6,9-diphenyl-9H-carbazole

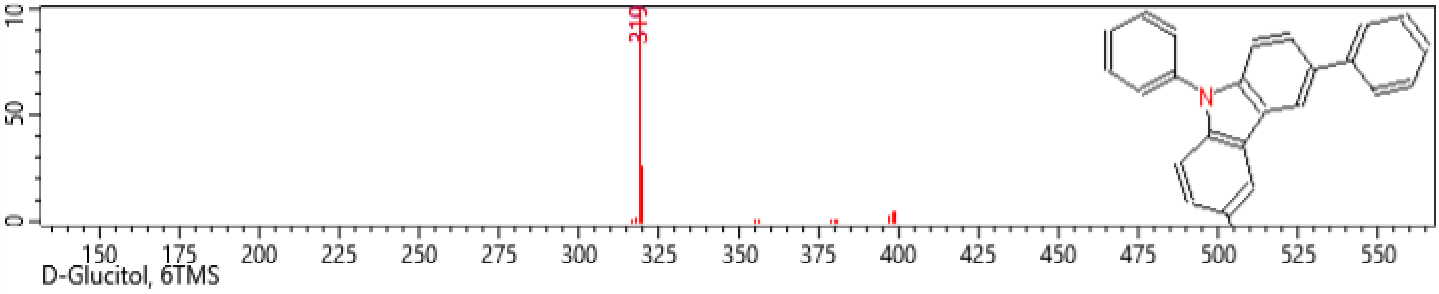


D-Glucitol, 6TMS

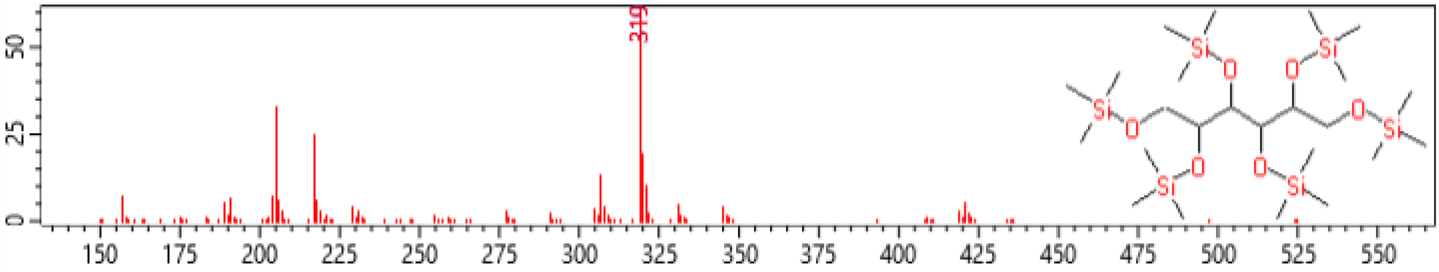


Lonafarnib

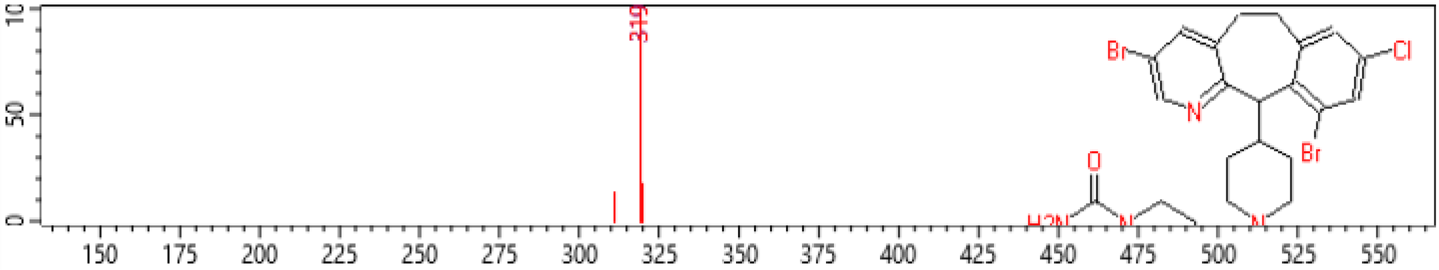


**Octasiloxane, 1,1,3,3,5,5,7,7,9,9,11,11,13,13,15,15-hexadecamethyl-**

Peak: 14.74 - 14.80 min (scan #2161 - #2177) Subtracted noise: 14.70 - 14.76 min (scan #2150 - #2165), 14.88 - 14.93 min (scan #21[illegible]

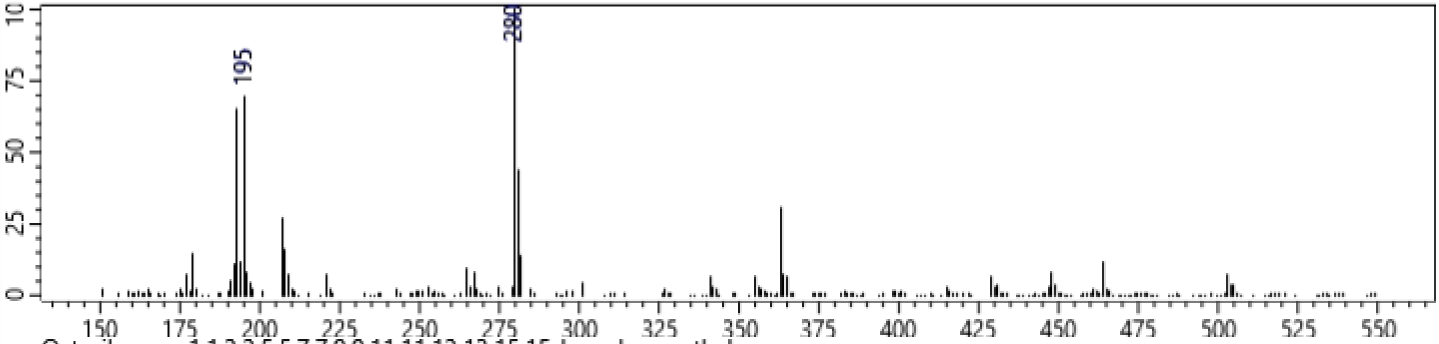


Octasiloxane, 1,1,3,3,5,5,7,7,9,9,11,11,13,13,15,15-hexadecamethyl-

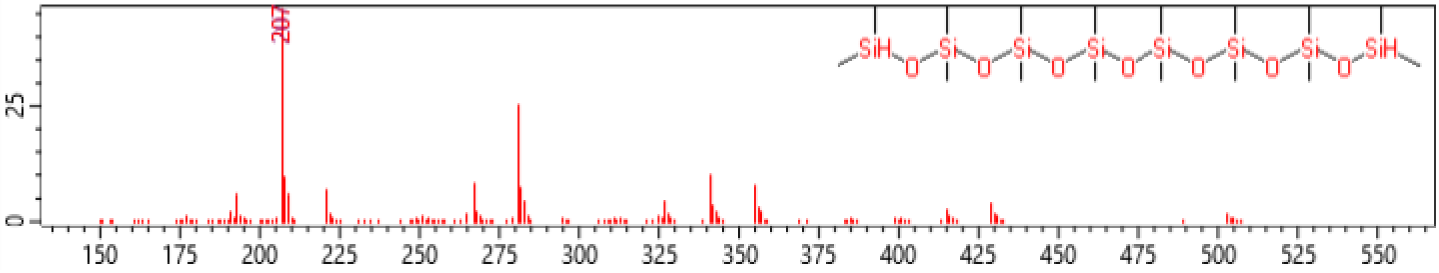

3,4',5-Trihydroxy-7-methoxyflavanone, 3TMS

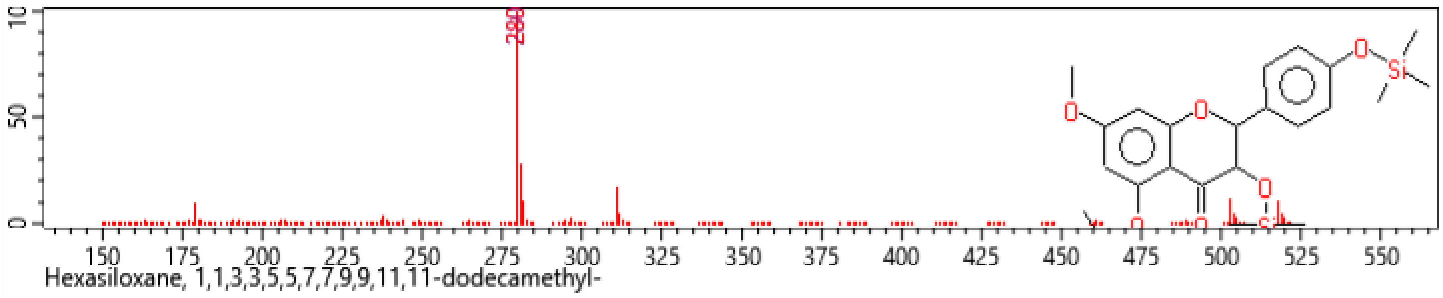


Hexasiloxane, 1,1,3,3,5,5,7,7,9,9,11,11-dodecamethyl-

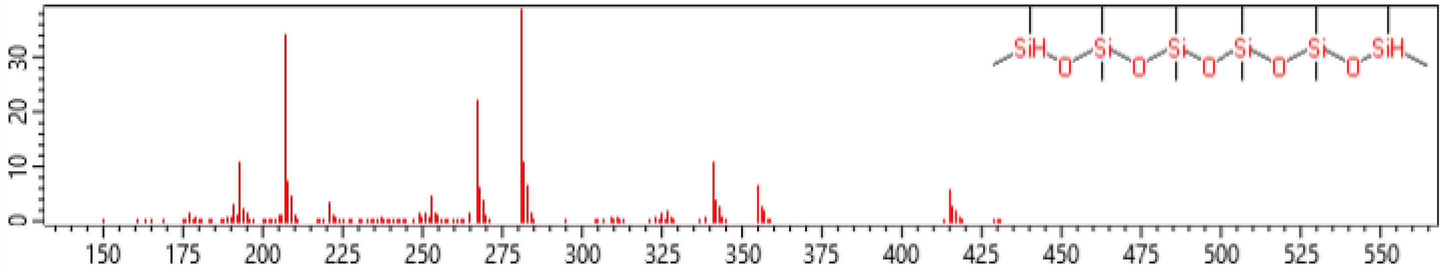

Heptasiloxane, 1,1,3,3,5,5,7,7,9,9,11,11,13,13-tetradecamethyl-

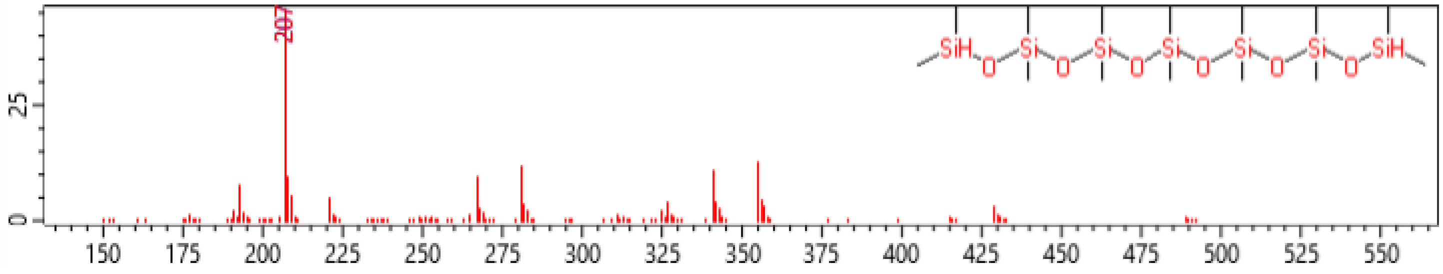


N-[2-(3,4-Dihydroxyphenyl)ethyl]-1-adamantanecarboxamide, 2TMS

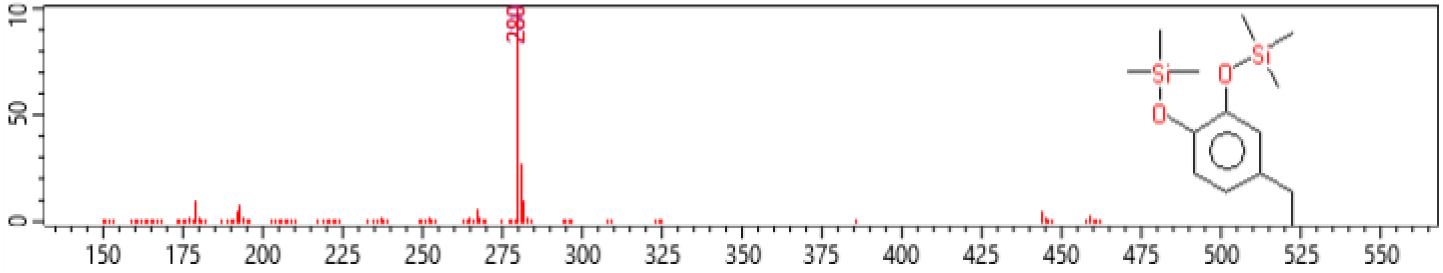


**1-Hexadecanol, TMS derivative**

Peak: 14.89 - 14.92 min (scan #2201 - #2211) Subtracted noise: 14.84 - 14.88 min (scan #2187 - #2198), 14.96 - 14.99 min (scan #22

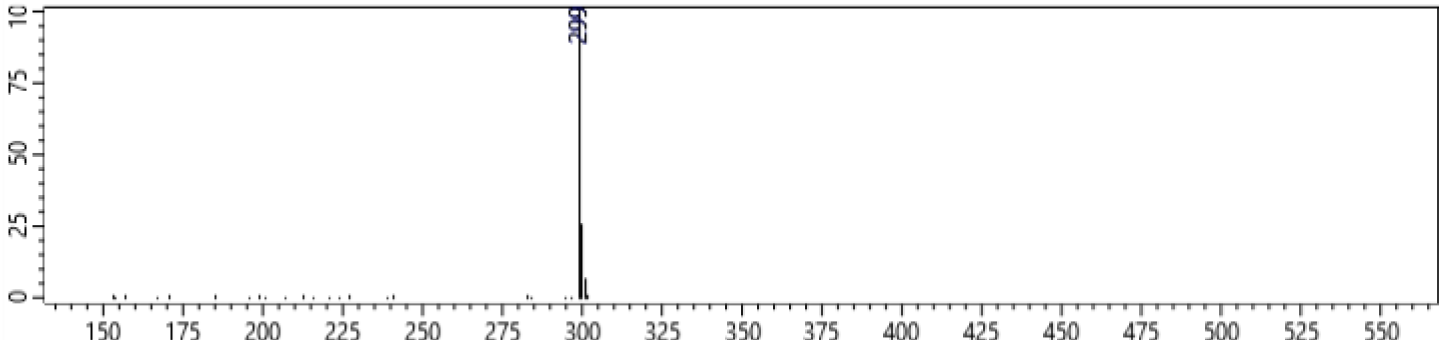

1-Hexadecanol, TMS derivative

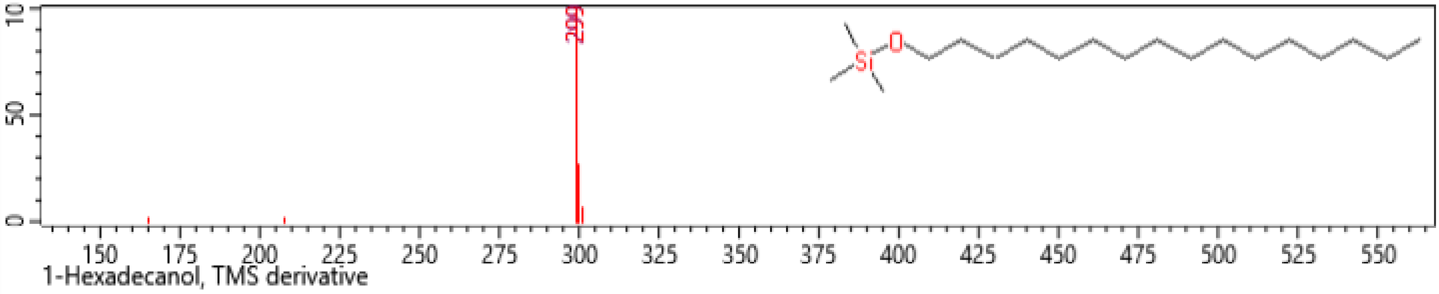


1-Hexadecanol, TMS derivative

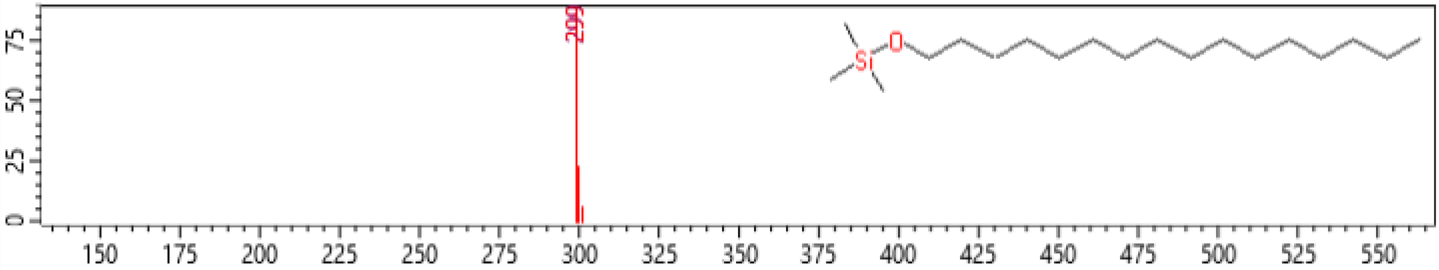


1-Hexadecanol, TMS derivative

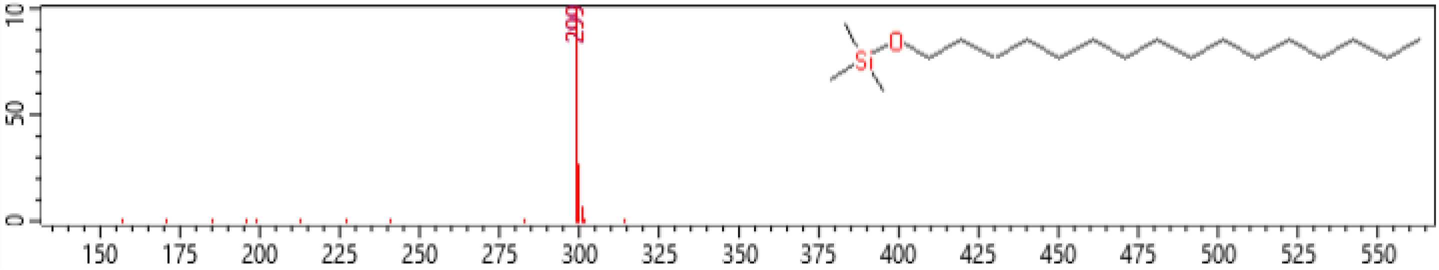


1-Hexadecanol, TBDMS derivative

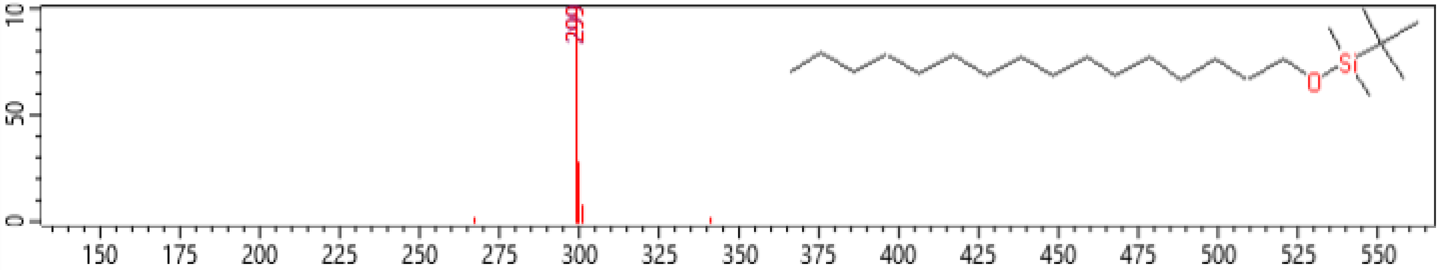


1-Dimethyl(chloromethyl)silyloxyhexadecane

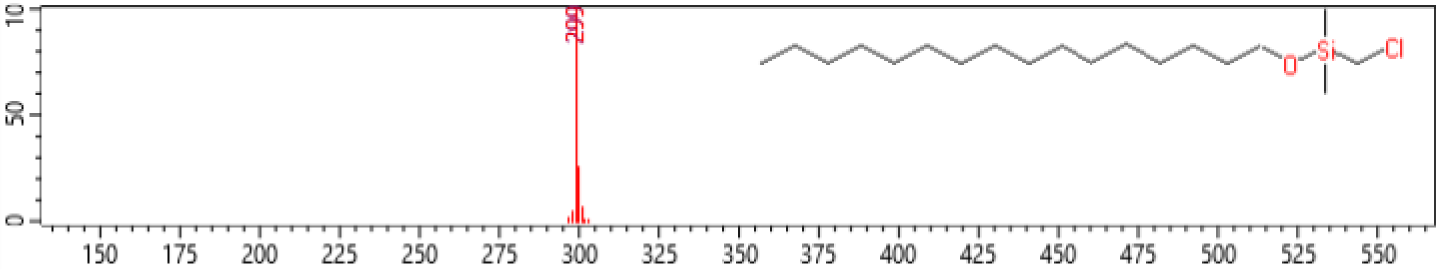

**Zolpidem phenyl-4-carboxylic acid**

Peak: 15.00 - 15.04 min (scan #2232 - #2244) Subtracted noise: 14.91 - 14.95 min (scan #2207 - #2219), 15.05 - 15.10 min (scan #22

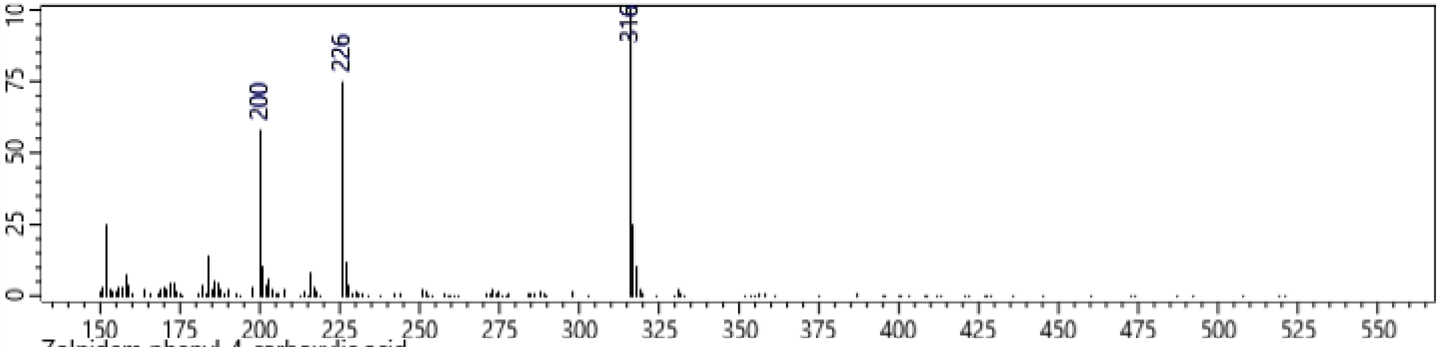


Zolpidem phenyl-4-carboxylic acid

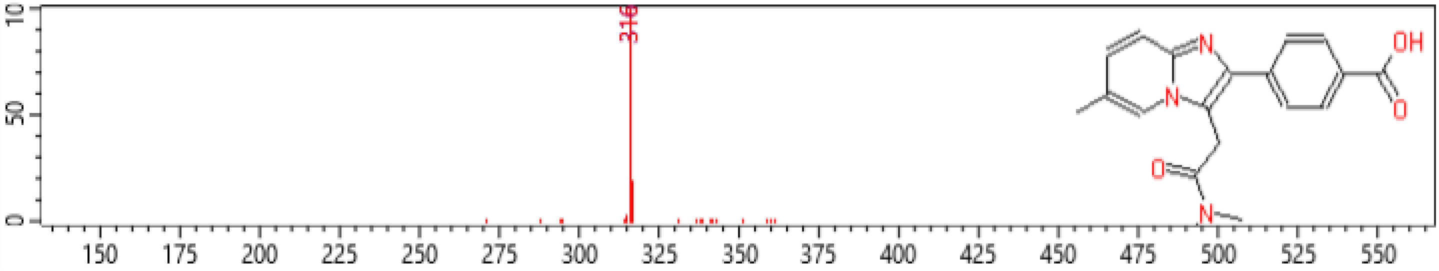


Clonazepam

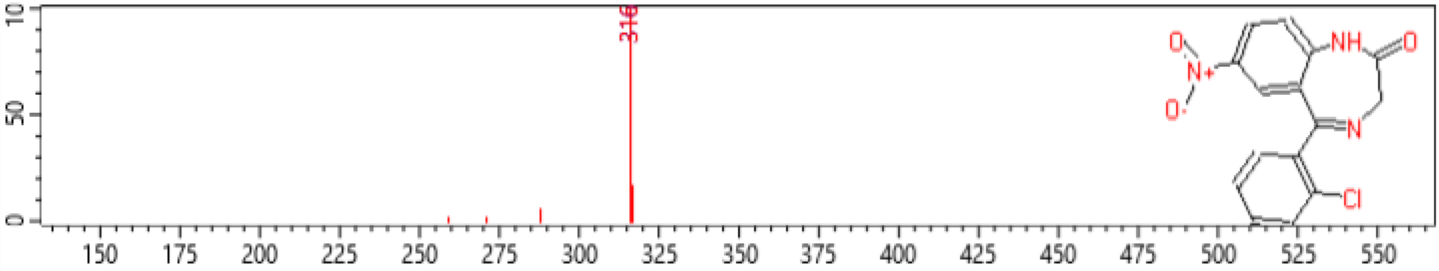


Clonazepam

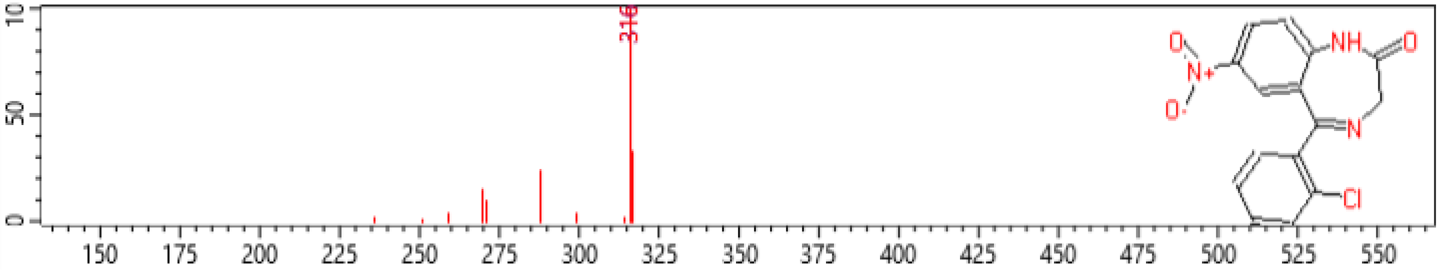


4-[[3-[(2S)-2-Amino-2-carboxyethyl]-2,6-diketo-pyrimidin-1-yl]methyl]benzoic acid

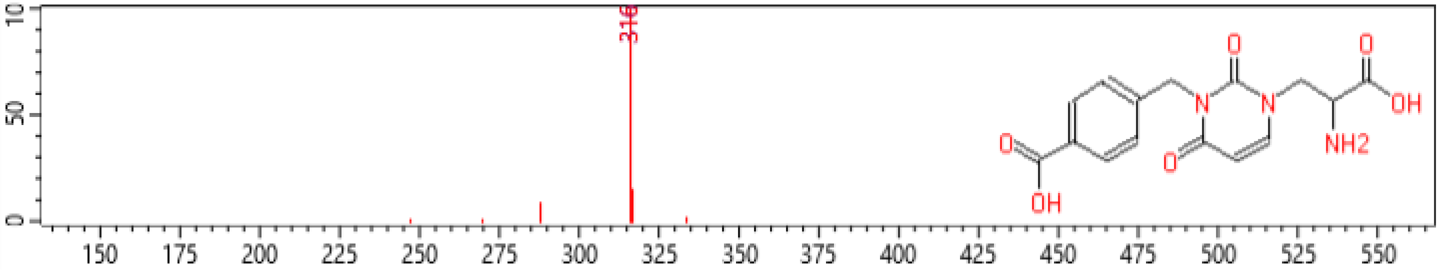

Jaceoside

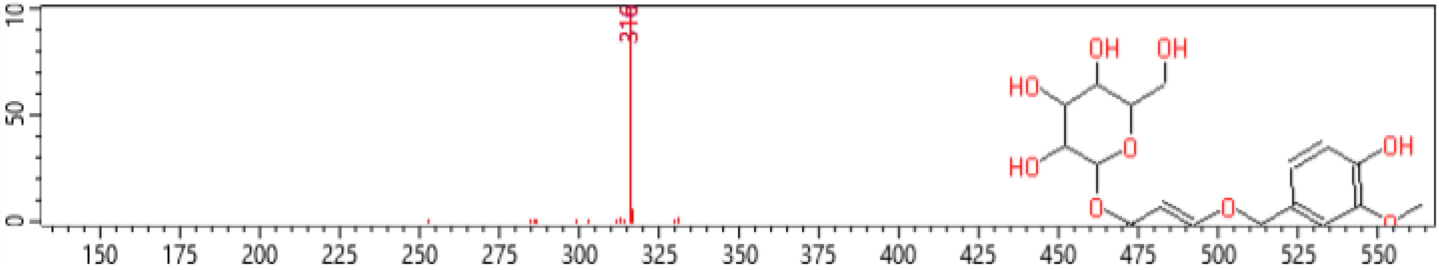


**Uracil, 1,3-dimethyl-5-(2'-trimethylsilyloxyethyl)-6-(trimethylsilyloxymethyl)-**

Peak: 15.06 - 15.09 min (scan #2250 - #2258) Subtracted noise: 15.02 - 15.05 min (scan #2237 - #2245), 15.12 - 15.15 min (scan #22[illegible]

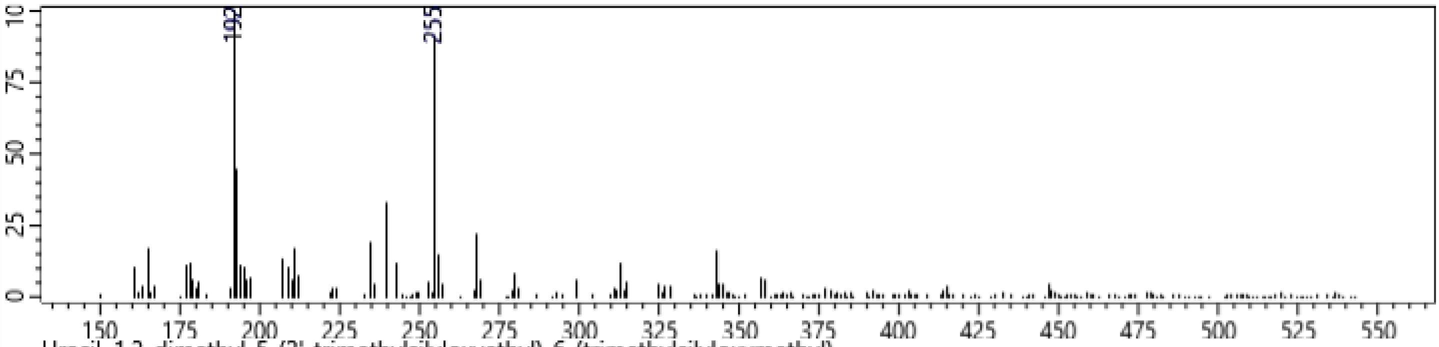


Uracil, 1,3-dimethyl-5-(2'-trimethylsilyloxyethyl)-6-(trimethylsilyloxymethyl)-

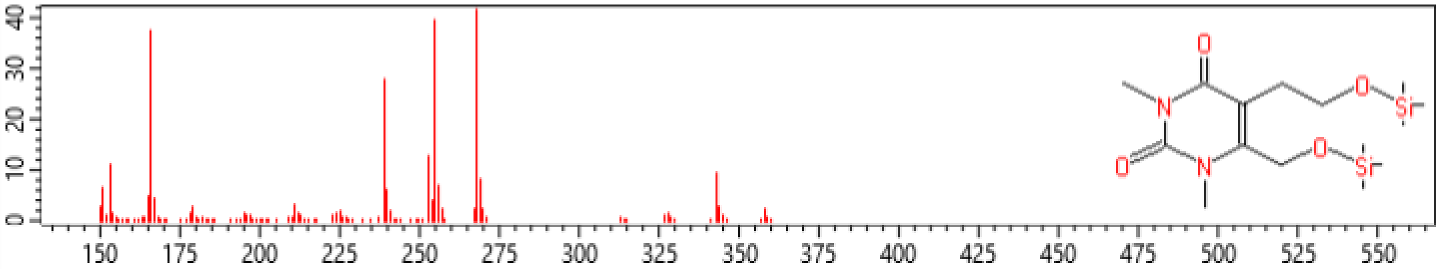

Isoquinoline, 1,2,3,4-tetrahydro-6,7-dimethoxy-1-(4-nitrophenyl)-

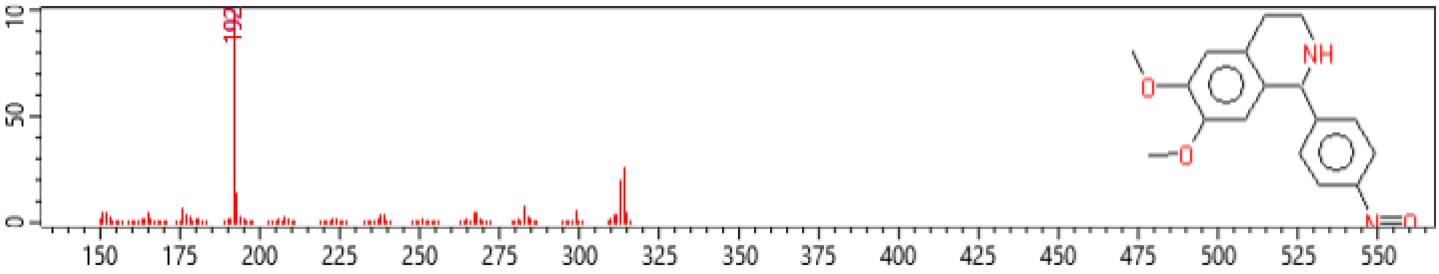


Benzilic acid, 2TMS

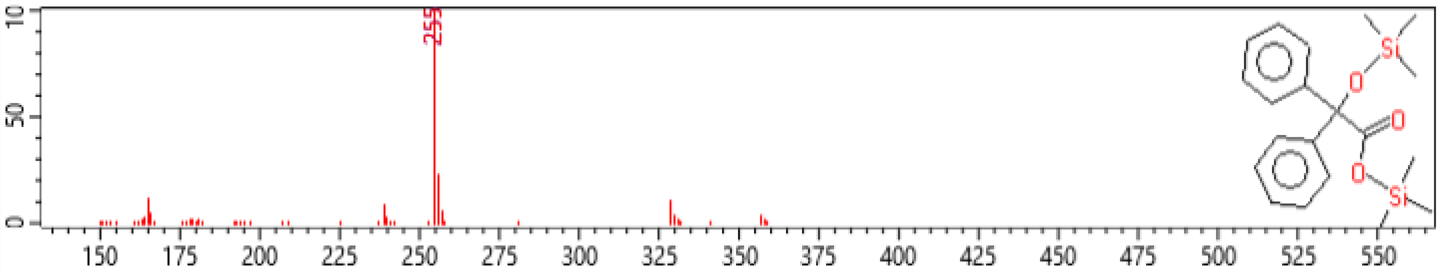

6-Cyclopropyl-4-furan-2-yl-2-methylsulfanyl-nicotinonitrile

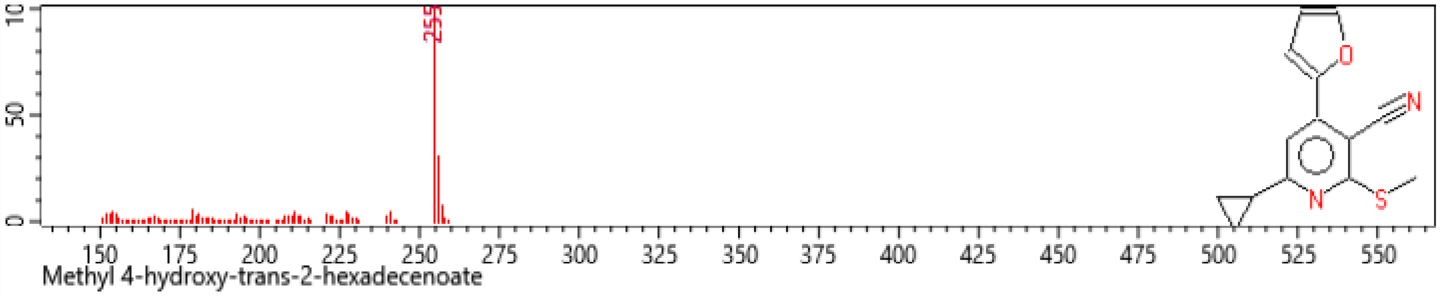


Methyl 4-hydroxy-trans-2-hexadecenoate

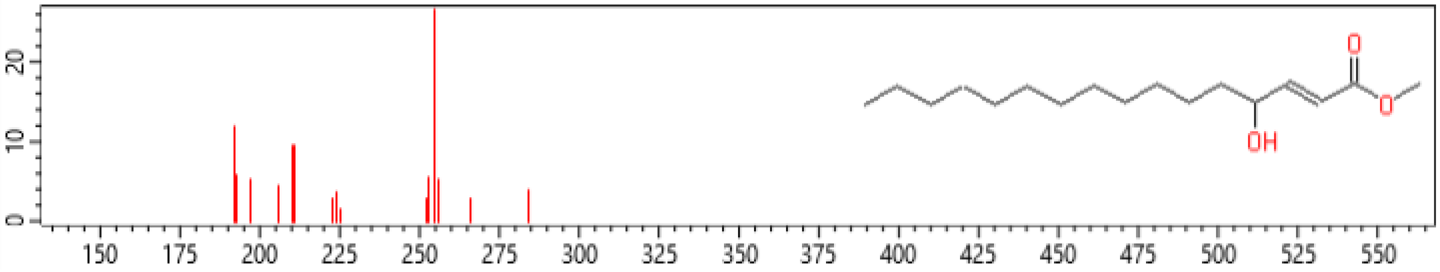


**Hexadecanoic acid, ethyl ester**

Peak: 15.13 - 15.15 min (scan #2269 - #2275) Subtracted noise: 15.10 - 15.11 min (scan #2259 - #2264), 15.16 - 15.18 min (scan #22

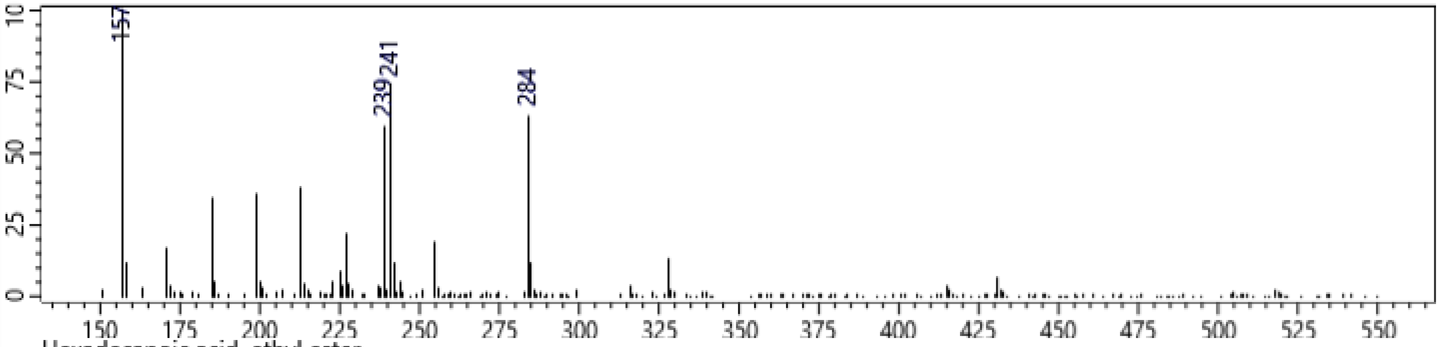


Hexadecanoic acid, ethyl ester

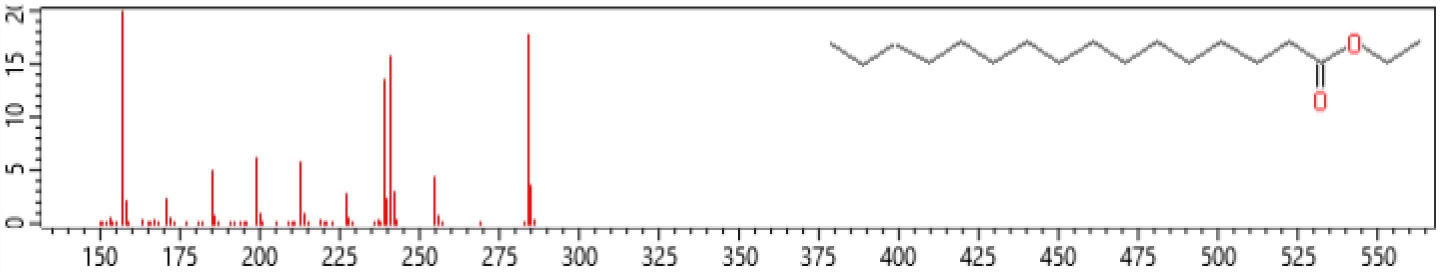


Hexadecanoic acid, ethyl ester

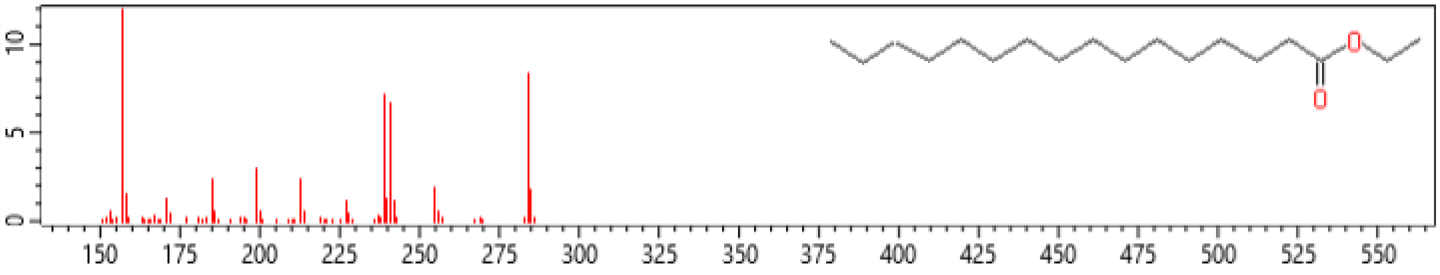

Hexadecanoic acid, ethyl ester

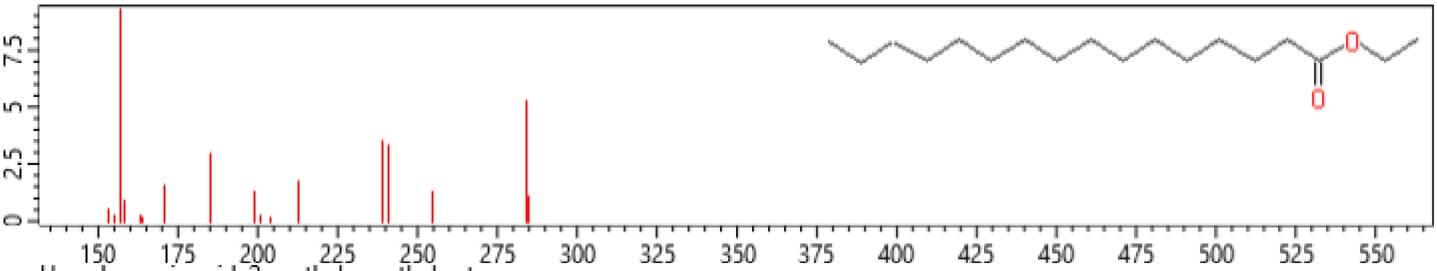

Hexadecanoic acid, 2-methyl-, methyl ester

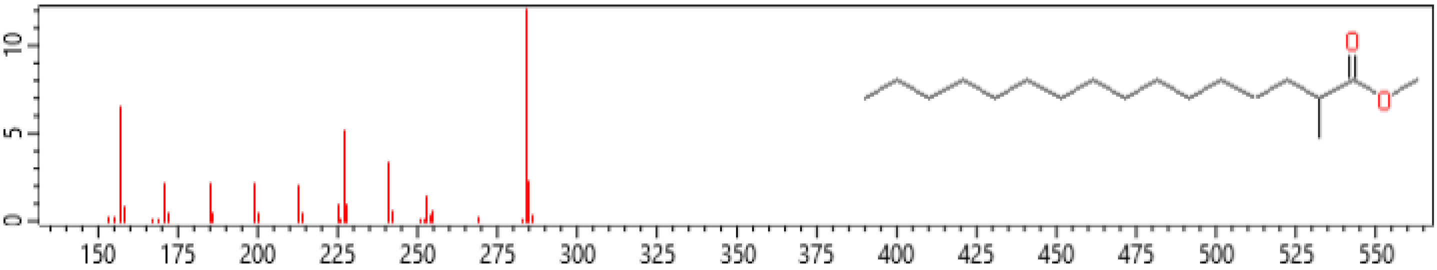

Hexadecanoic acid, 15-methyl-, methyl ester

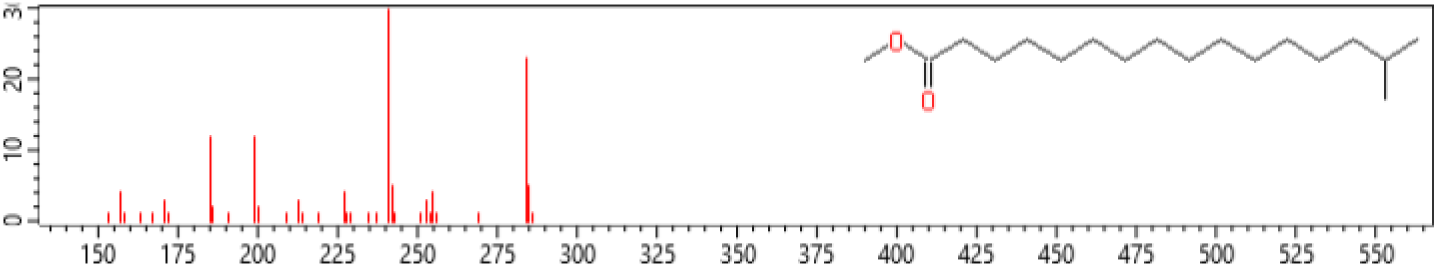

**Thiazole, 2-(phenylthio)-**

Peak: 15.24 - 15.31 min (scan #2300 - #2319) Subtracted noise: 15.09 - 15.16 min (scan #2258 - #2277), 15.31 - 15.37 min (scan #23

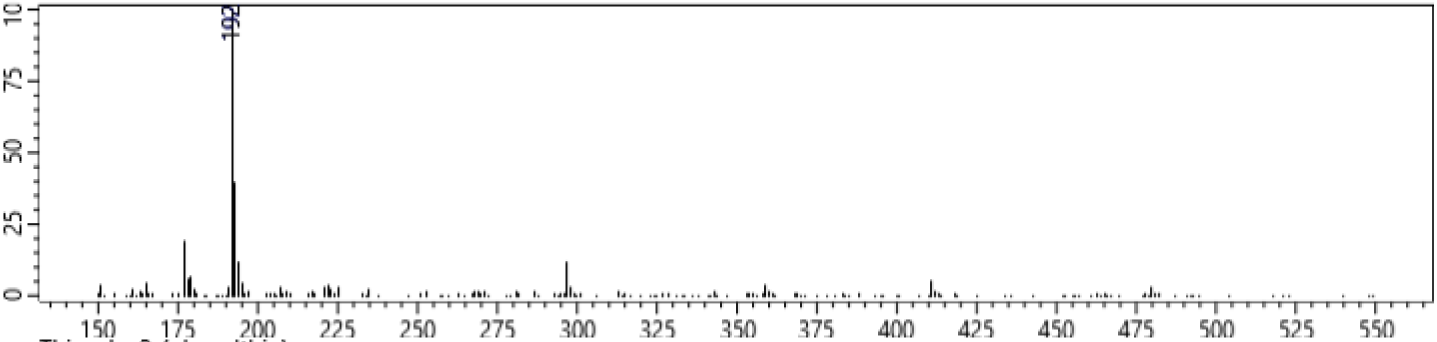


Thiazole, 2-(phenylthio)-

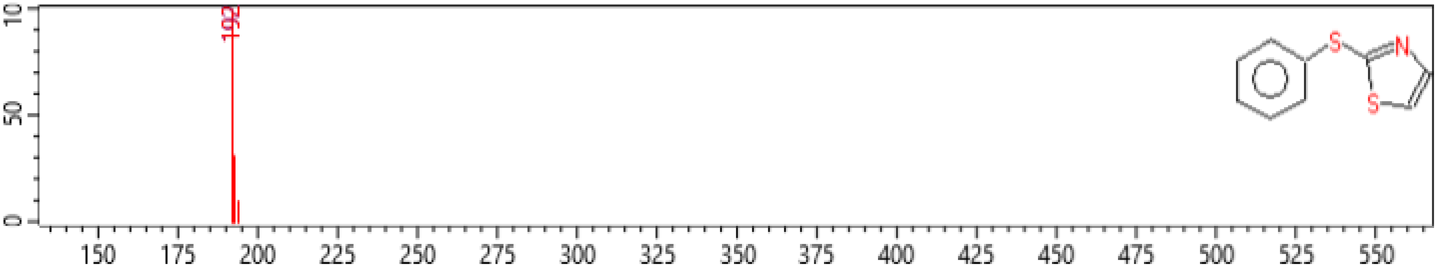

4-((Trimethylsilyl)oxy)phenethyl acetate

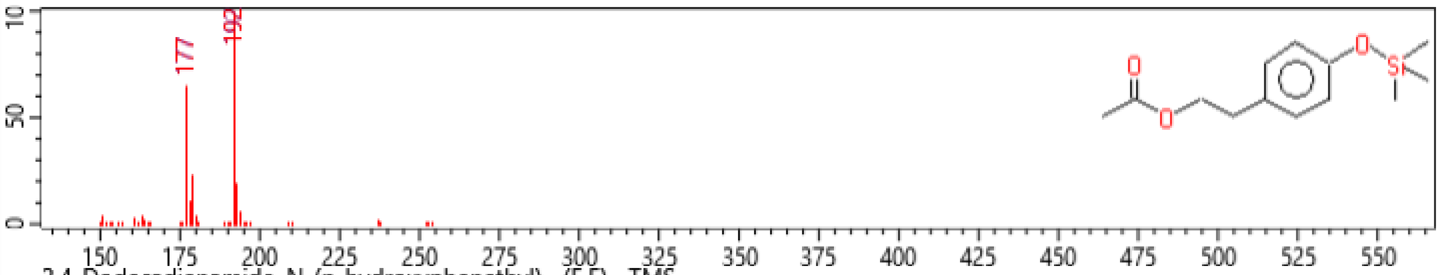


2,4-Dodecadienamide, N-(p-hydroxyphenethyl)-, (E,E)-, TMS

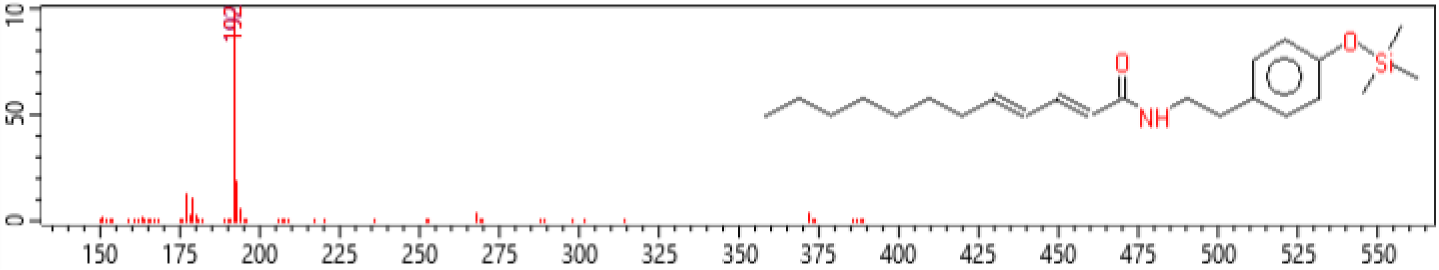


2-(2,5-Dimethoxy-4-ethylphenyl)ethylamine, N-acetyl-

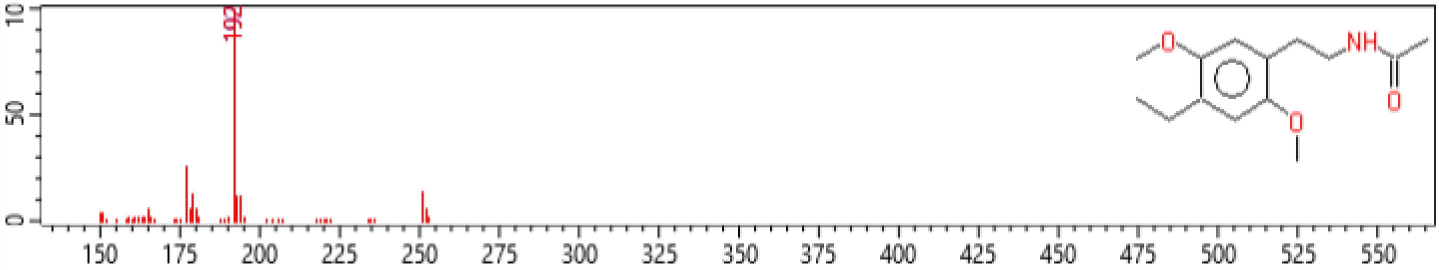


Phloretin, tris(trimethylsilyl) ether

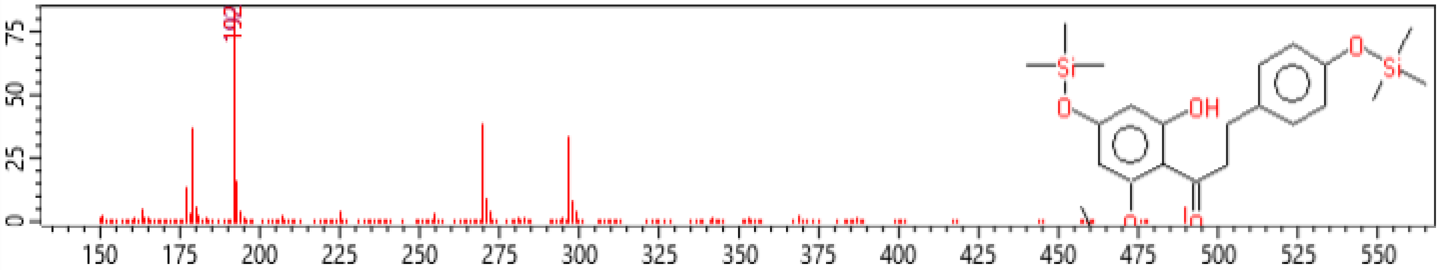


**Thiazole, 2-(phenylthio)-**

Peak: 15.34 - 15.41 min (scan #2326 - #2348) Subtracted noise: 15.23 - 15.30 min (scan #2296 - #2317), 15.47 - 15.55 min (scan #23

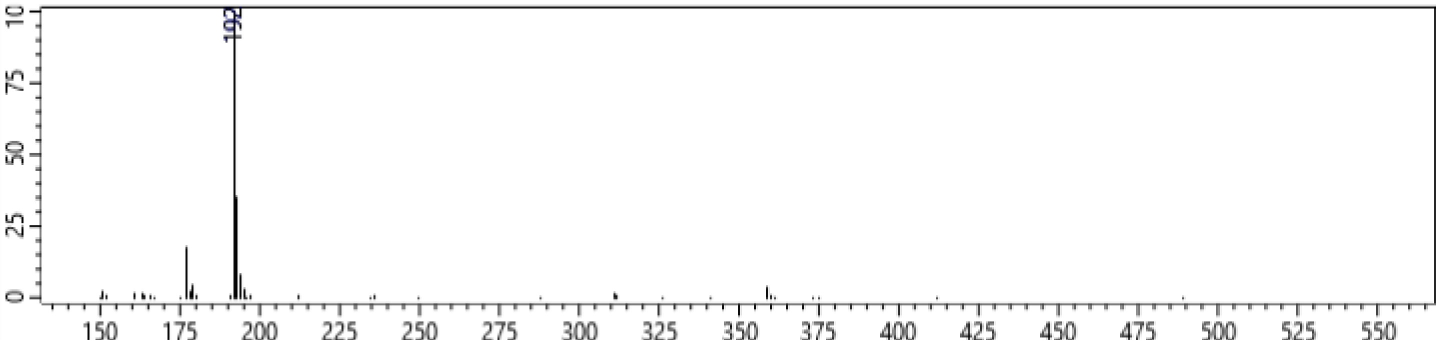

Thiazole, 2-(phenylthio)-

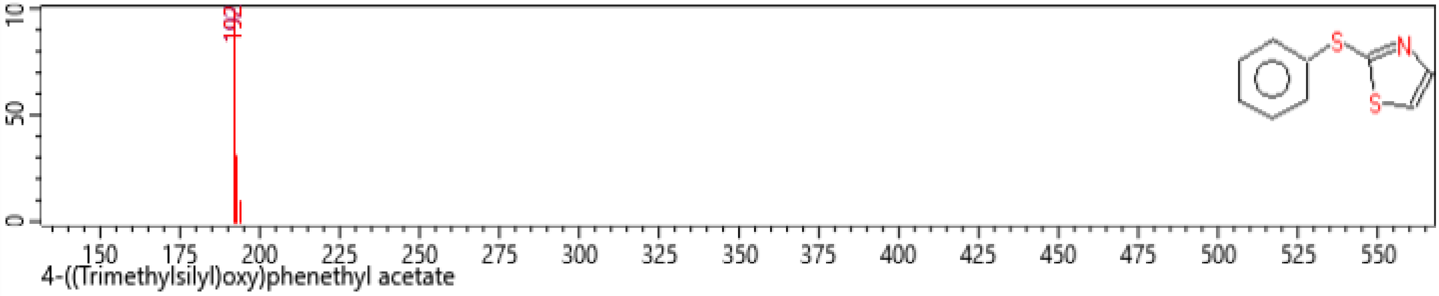


4-((Trimethylsilyl)oxy)phenethyl acetate

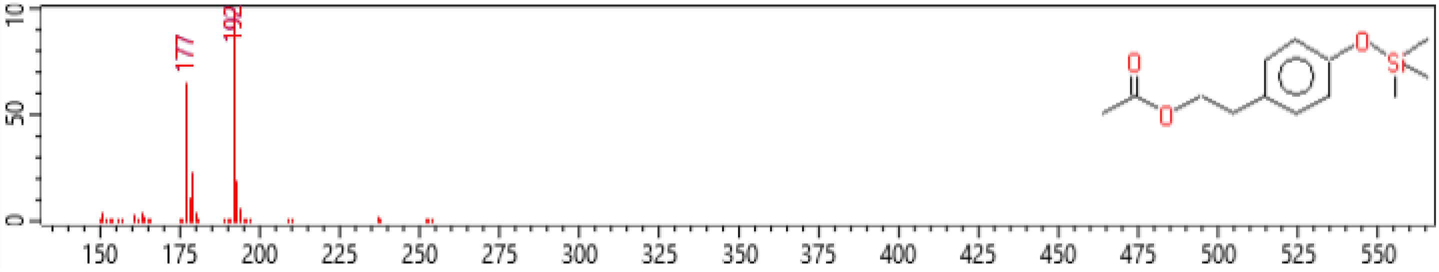


2,4-Dodecadienamide, N-(p-hydroxyphenethyl)-, (E,E)-, TMS

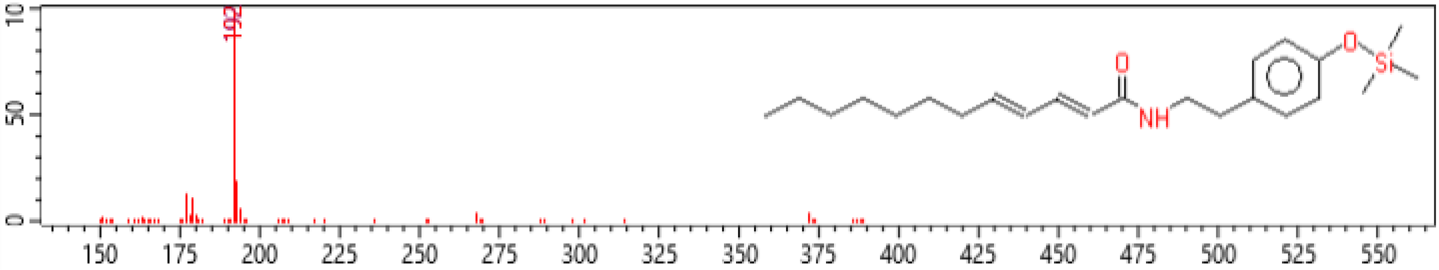


7-Isoquinolinol, 1,2,3,4-tetrahydro-1-[(3-hydroxy-4-methoxyphenyl)methyl]-6-methoxy-2-methyl-, (S)-

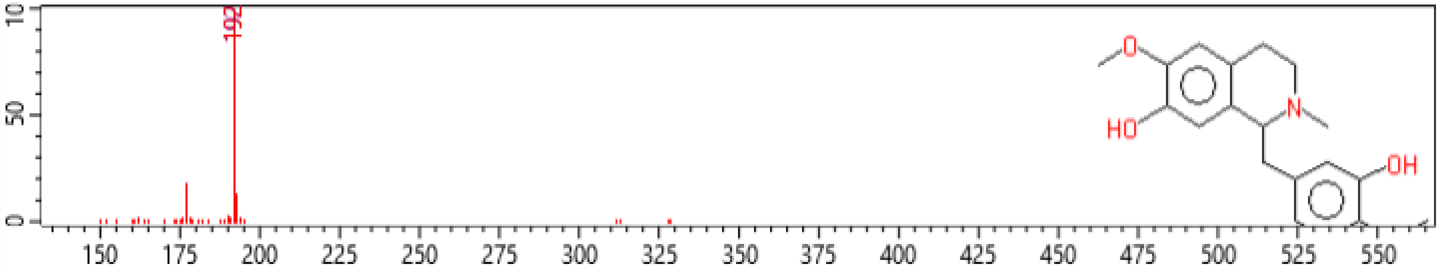


3-(1-Methyl-1-silacyclobutyl)benzoic acid, methyl ester

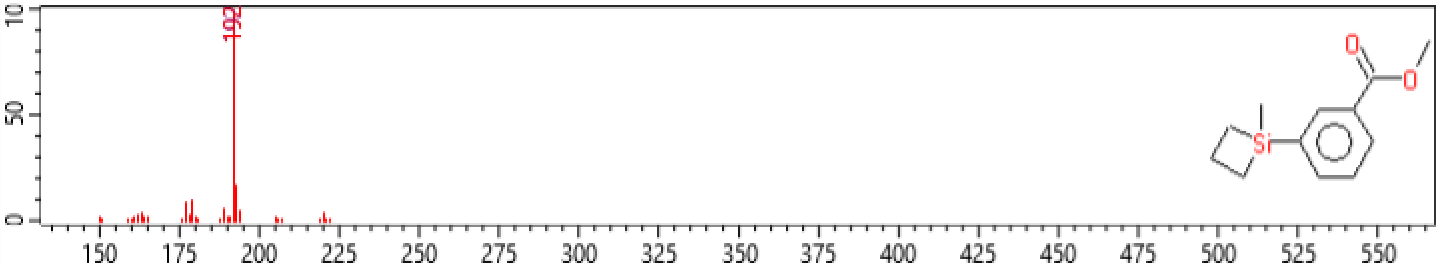

**Hispidulin**

Peak: 15.48 - 15.50 min (scan #2366 - #2371) Subtracted noise: 15.45 - 15.46 min (scan #2358 - #2362), 15.51 - 15.53 min (scan #23

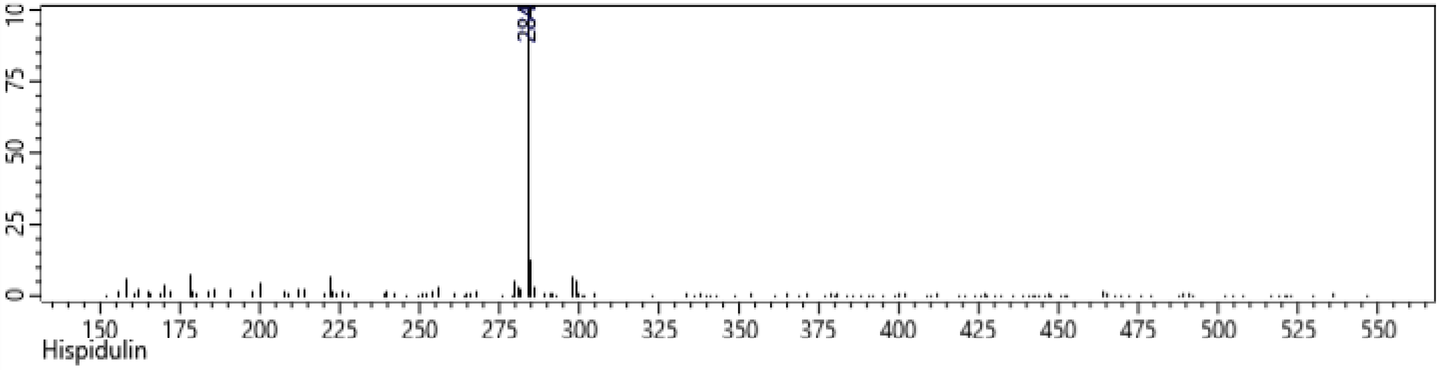


Hispidulin

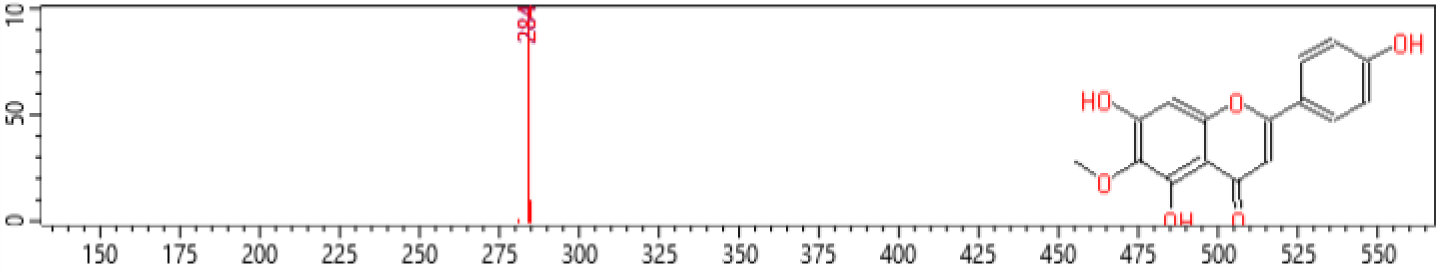


2-(5-Iodo-2-methyl-1H-indol-3-yl)ethanamine

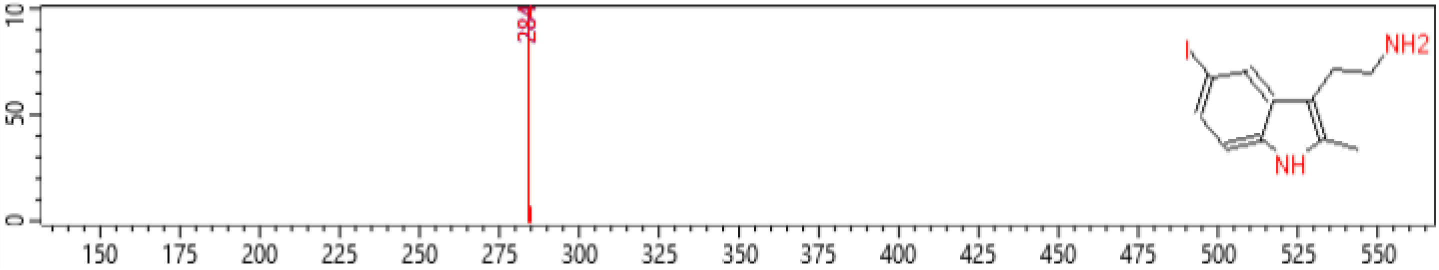


Diosmetin

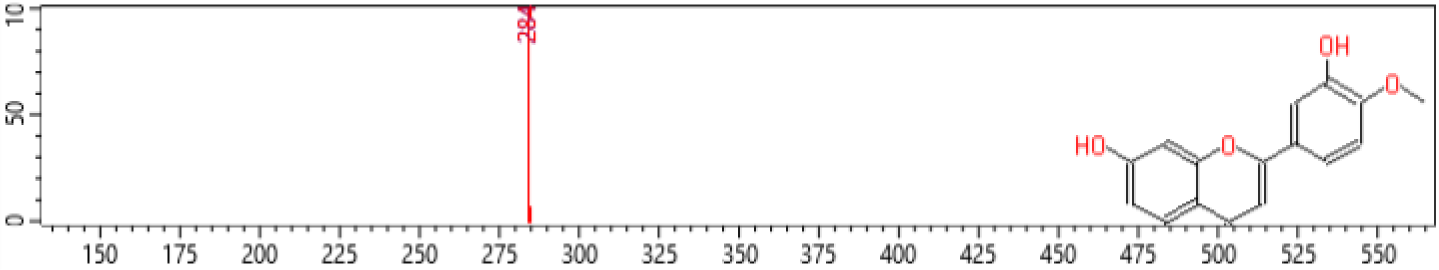


Norflurazon

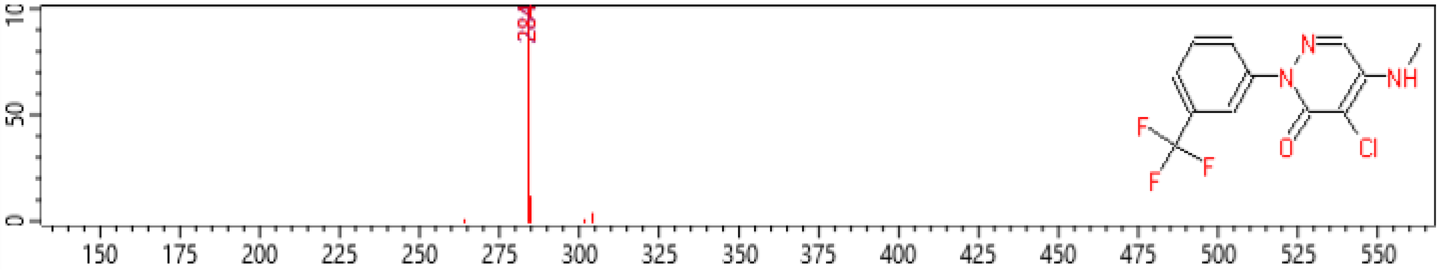

Mesoporphyrin IX

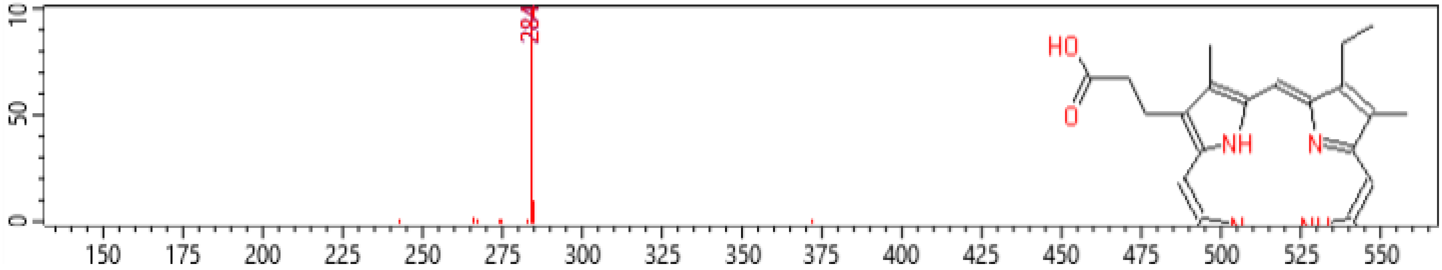


**Palmitic Acid, TMS derivative**

Peak: 15.52 - 15.57 min (scan #2377 - #2391) Subtracted noise: 15.46 - 15.51 min (scan #2360 - #2375), 15.62 - 15.67 min (scan #24

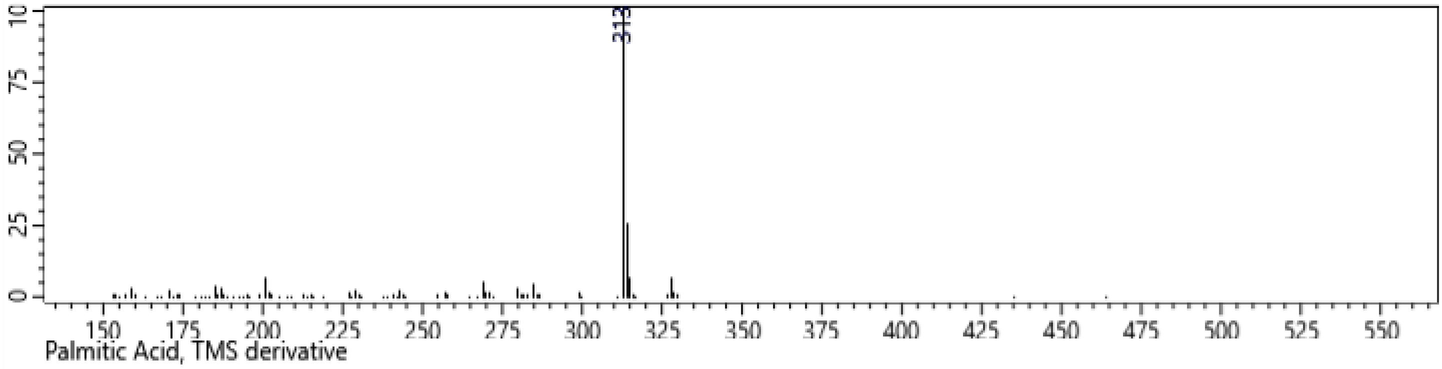


Palmitic Acid, TMS derivative

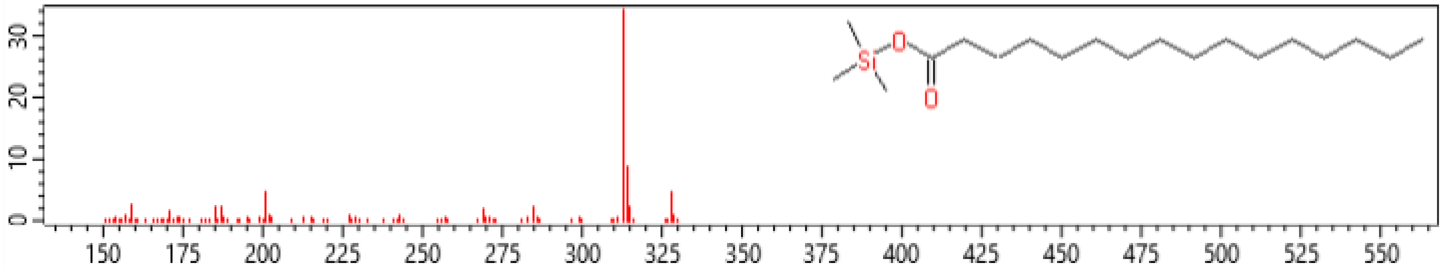


Palmitic Acid, TMS derivative

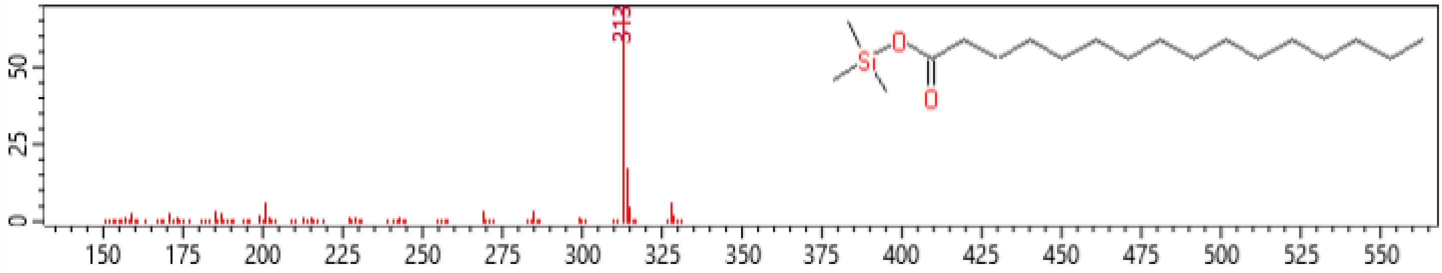


Palmitic Acid, TMS derivative

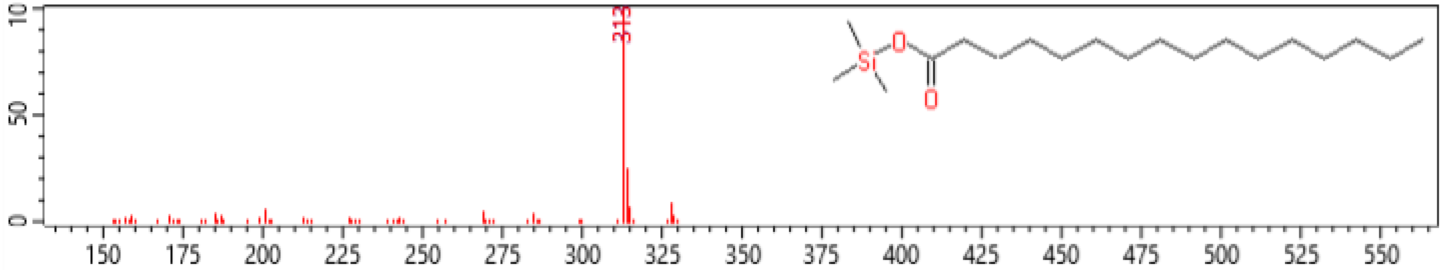

Palmitic Acid, TMS derivative

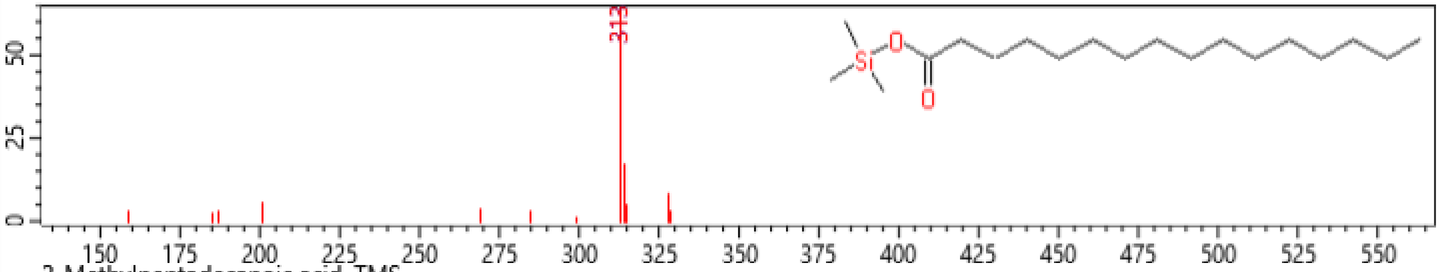


2-Methylpentadecanoic acid, TMS

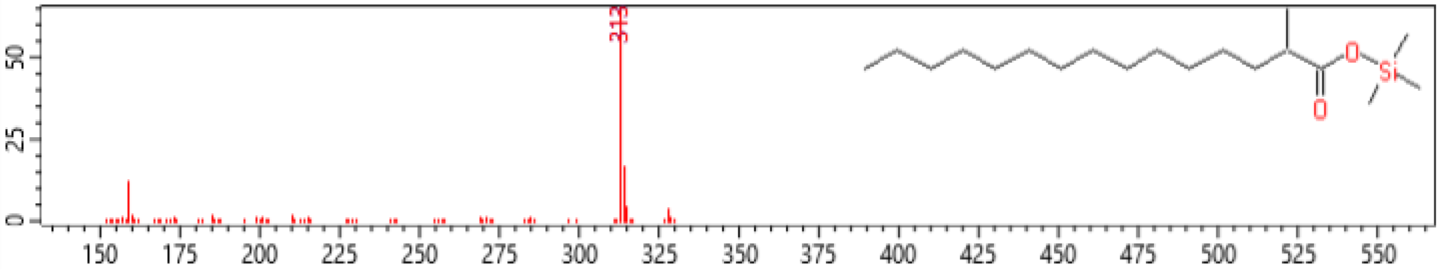


**Momordin Ic**

Peak: 15.61 - 15.67 min (scan #2402 - #2420) Subtracted noise: 15.55 - 15.61 min (scan #2385 - #2404), 15.76 - 15.83 min (scan #24

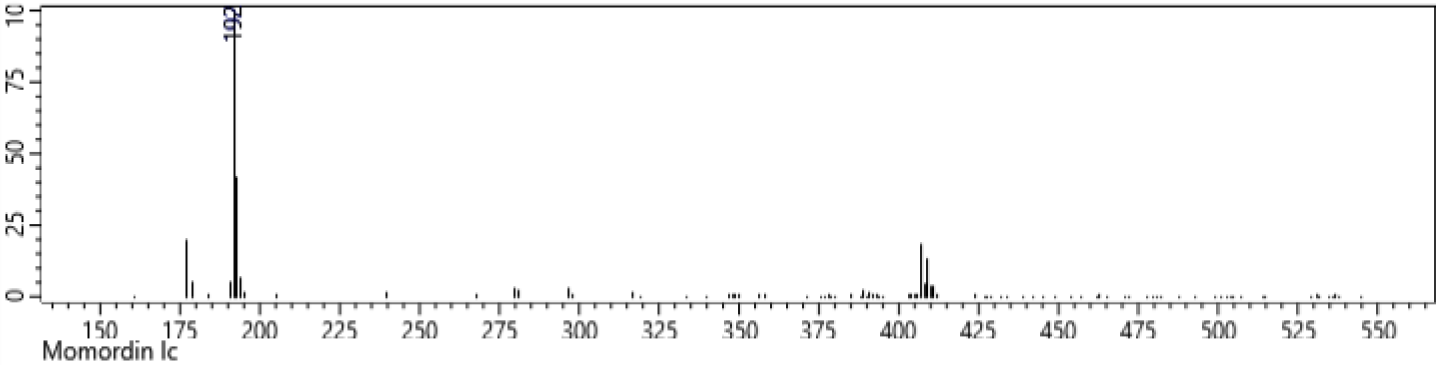


Momordin Ic

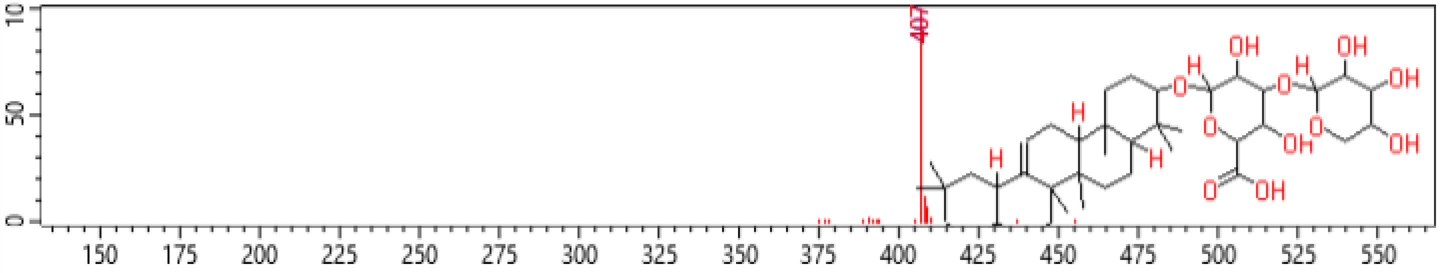


Thiazole, 2-(phenylthio)-

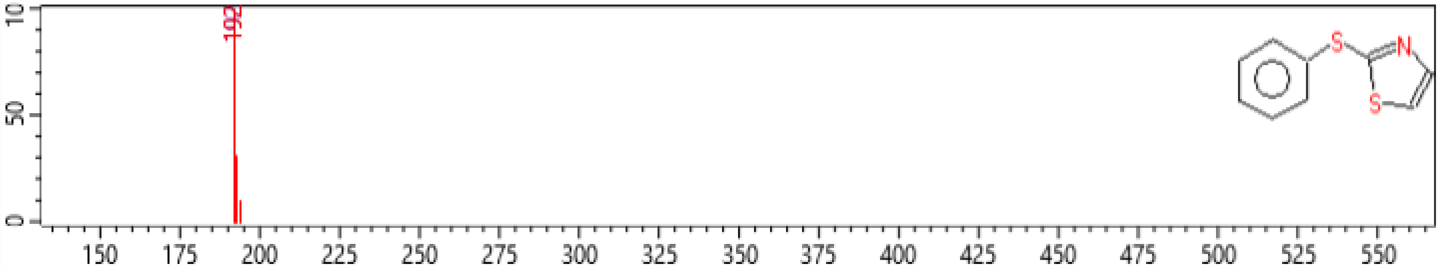

N-[1,1'-Biphenyl]-2-yl-2-chloroacetamide

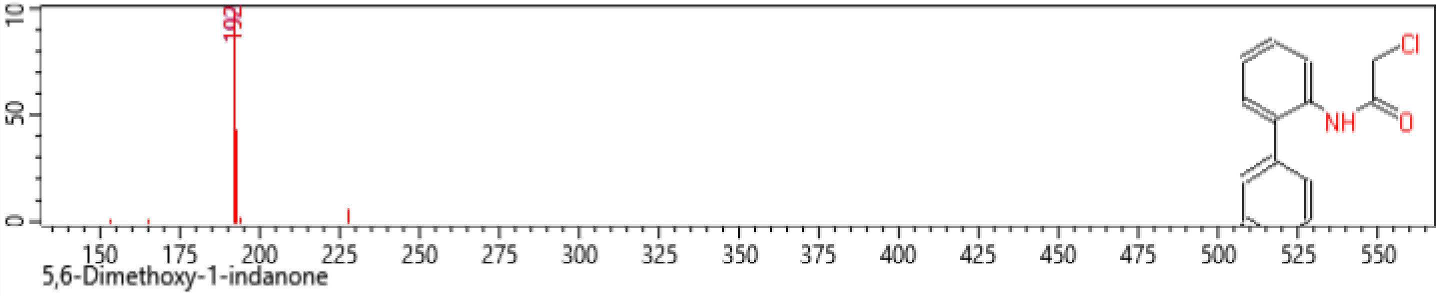


5,6-Dimethoxy-1-indanone

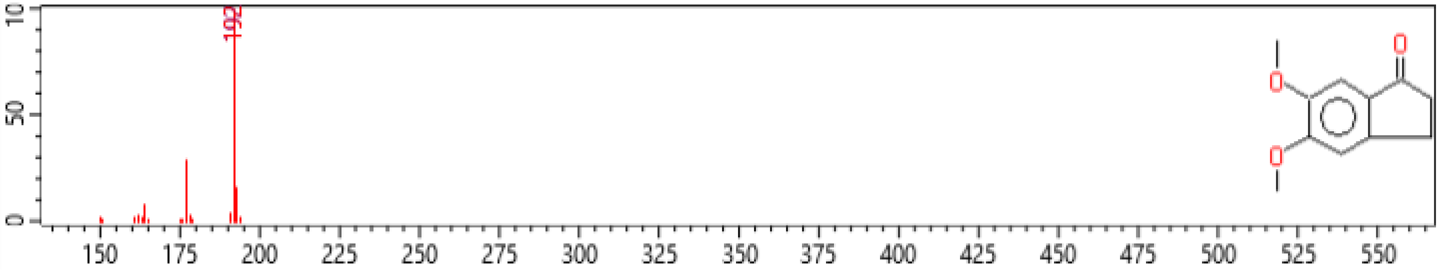


N-Methylcoclaurine

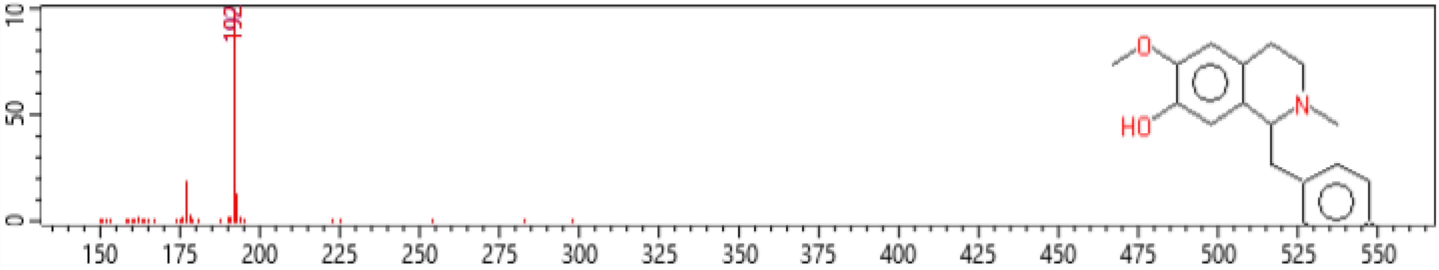


**6-Bromo-1,3-benzothiazol-2-amine, N,N-bis(trimethylsilyl)-**

Peak: 15.79 - 15.85 min (scan #2454 - #2469) Subtracted noise: 15.70 - 15.76 min (scan #2429 - #2444), 15.88 - 15.93 min (scan #24

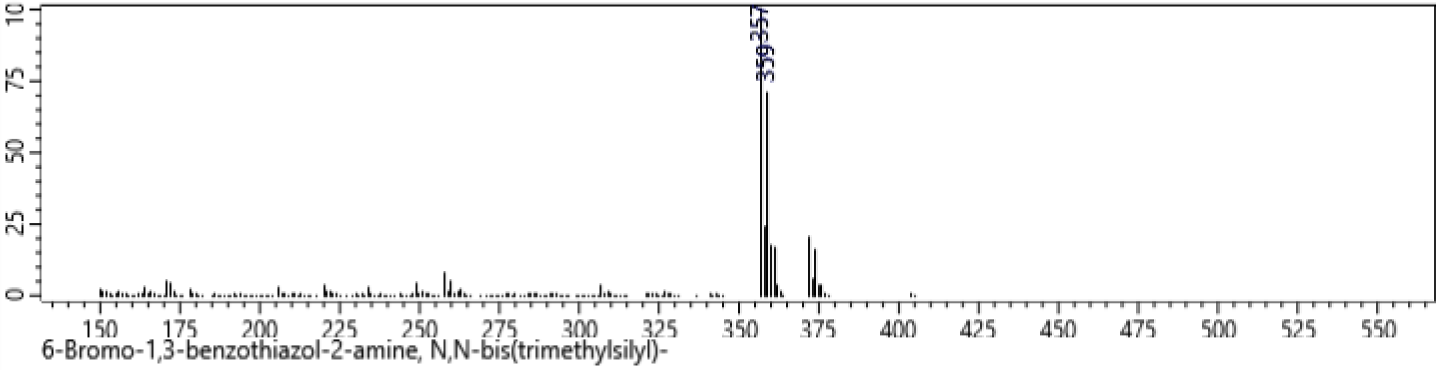


6-Bromo-1,3-benzothiazol-2-amine, N,N-bis(trimethylsilyl)-

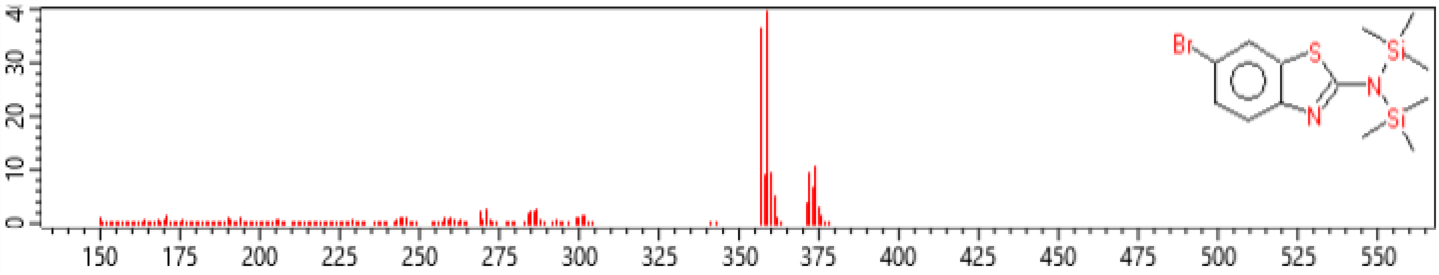

2-Bromo-5-nitro-4-trifluoromethoxyaniline, TMS

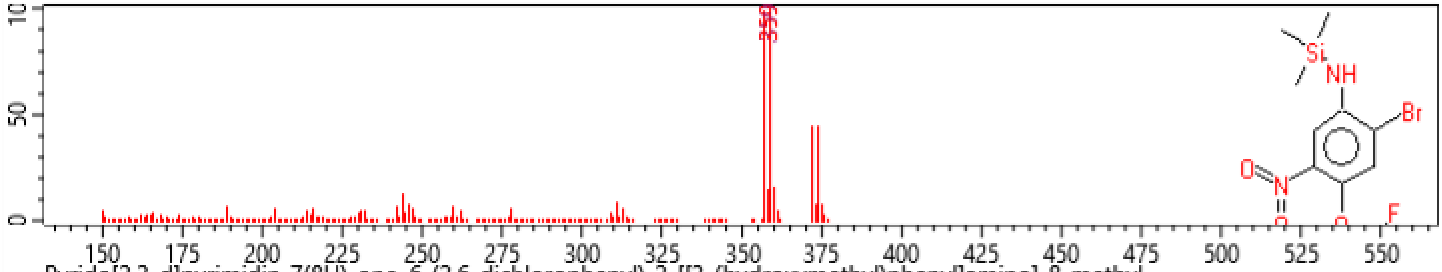


Pyrido[2,3-d]pyrimidin-7(8H)-one, 6-(2,6-dichlorophenyl)-2-[[3-(hydroxymethyl)phenyl]amino]-8-methyl-

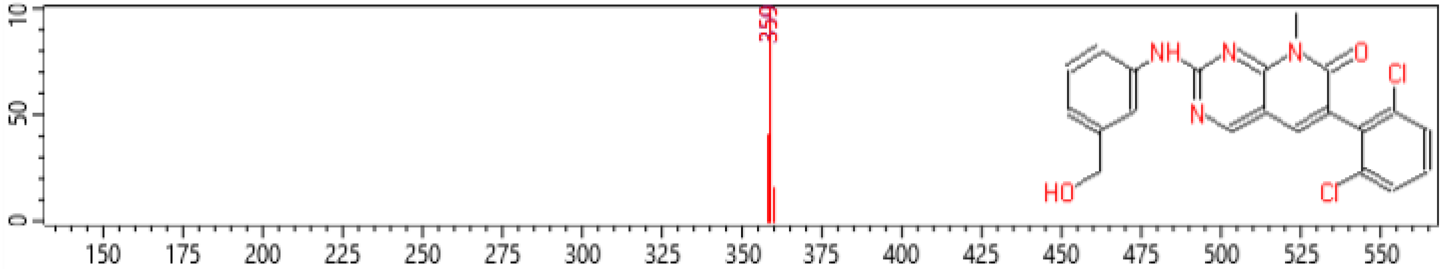


3-Bromo-4-methylbenzene-1-carbohydrazonic acid, 2 TMS derivative

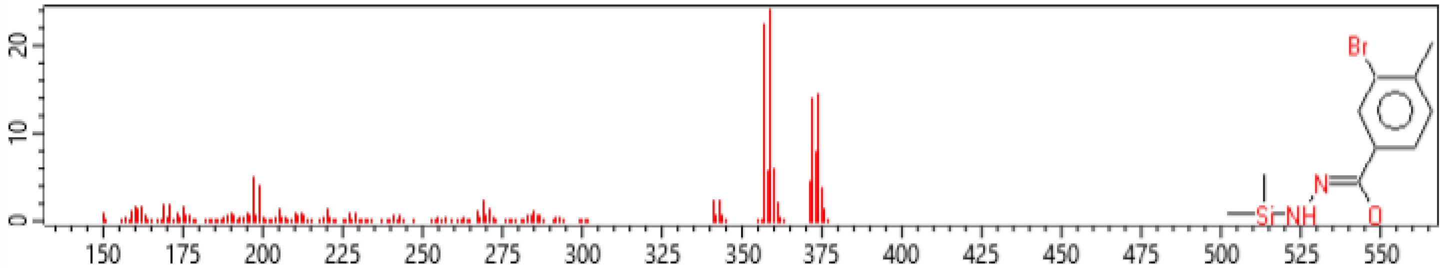


2-(4-Bromophenyl)acetohydrazide, di-TMS

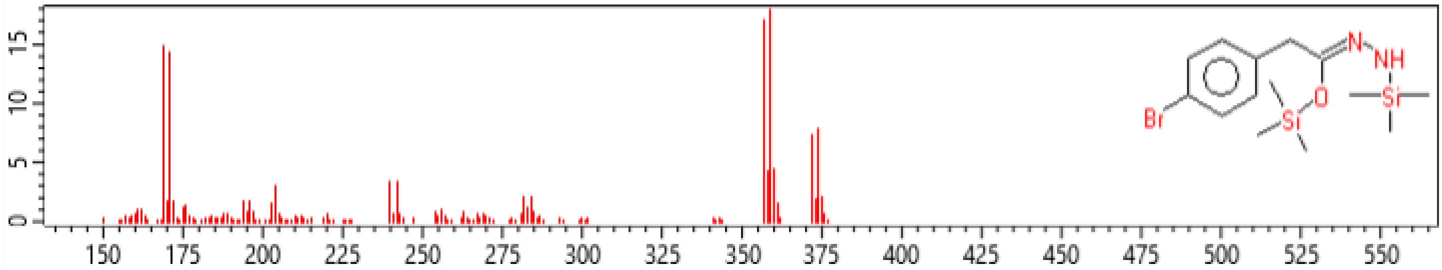


**Cyclodecasiloxane, eicosamethyl-**

Peak: 15.88 - 15.91 min (scan #2479 - #2485) Subtracted noise: 15.85 - 15.87 min (scan #2471 - #2476), 15.92 - 15.94 min (scan #24

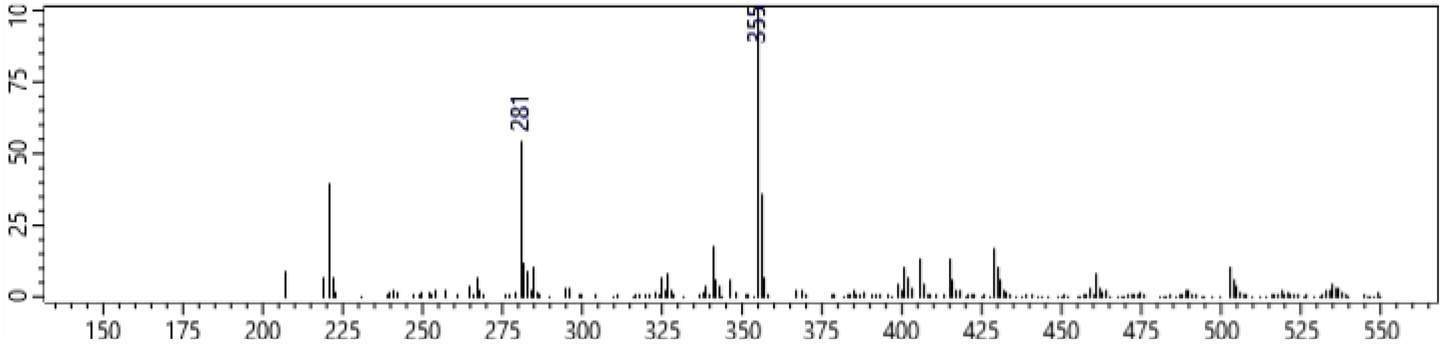

Cyclodecasiloxane, eicosamethyl-

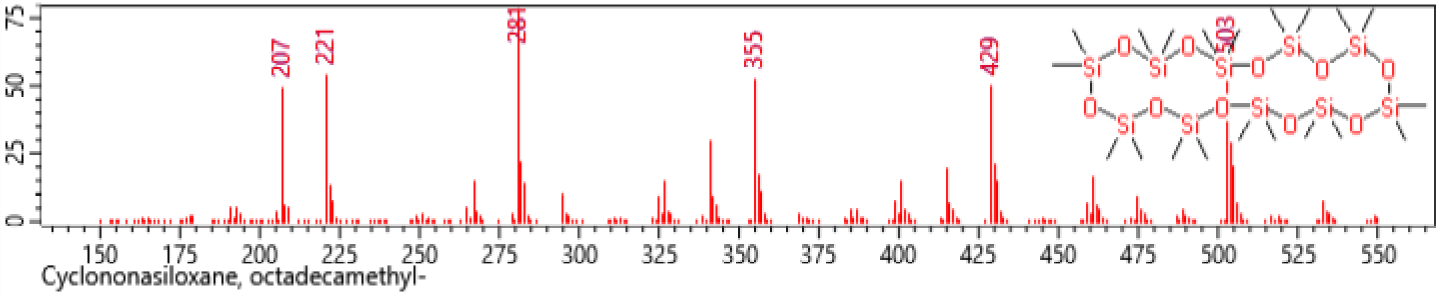


Cyclononasiloxane, octadecamethyl-

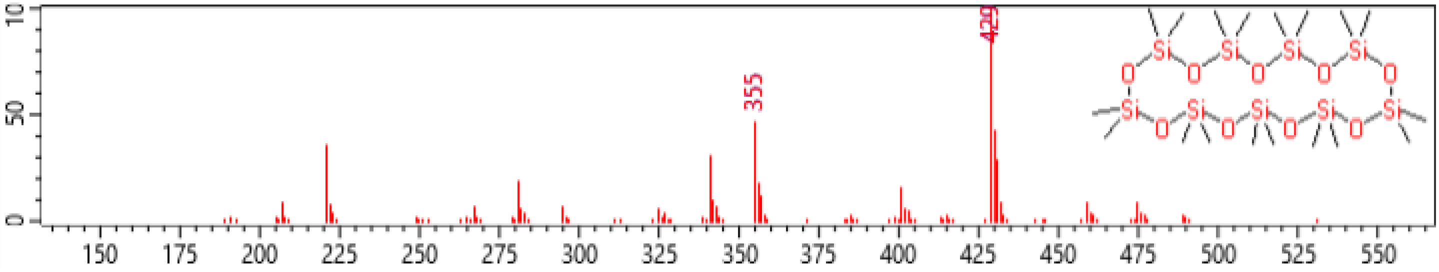


Cyclononasiloxane, octadecamethyl-

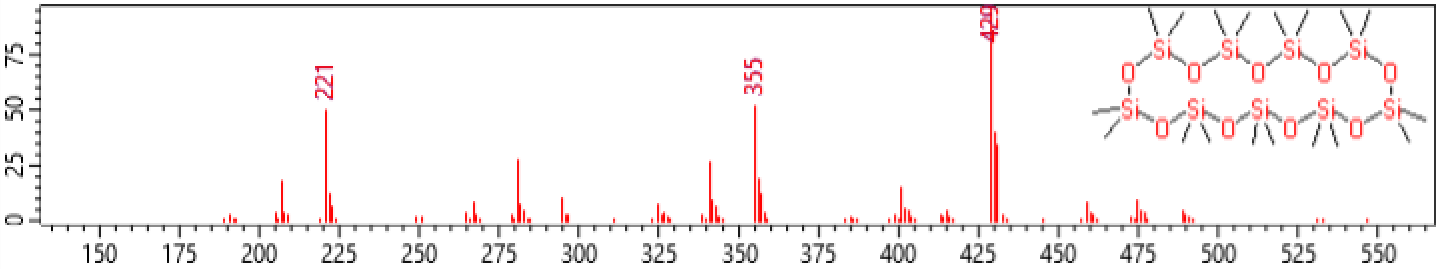


Octasiloxane, 1,1,3,3,5,5,7,7,9,9,11,11,13,13,15,15-hexadecamethyl-

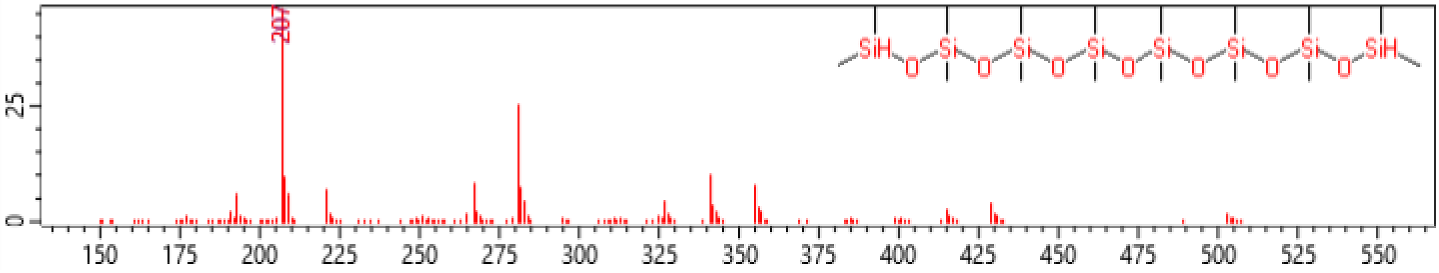


Cyclooctasiloxane, hexadecamethyl-

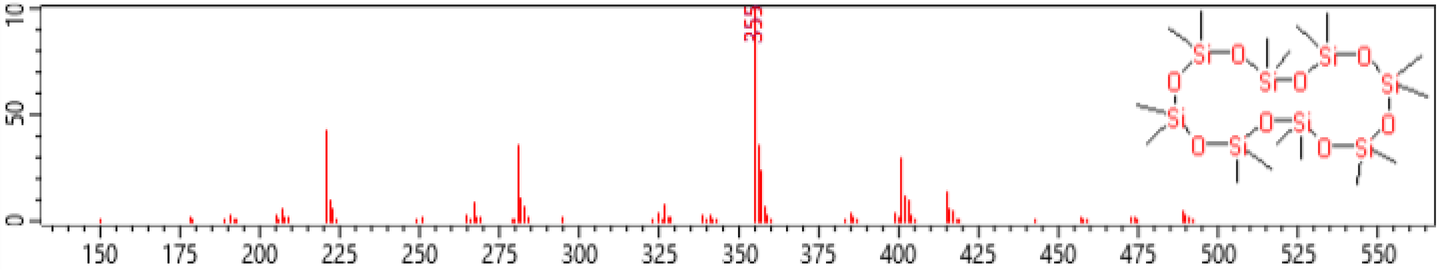

**9,11-Octadecadienoic acid, methyl ester, (E,E)-**

Peak: 15.93 - 15.95 min (scan #2493 - #2498) Subtracted noise: 15.90 - 15.92 min (scan #2483 - #2488), 15.96 - 15.98 min (scan #25[illegible]

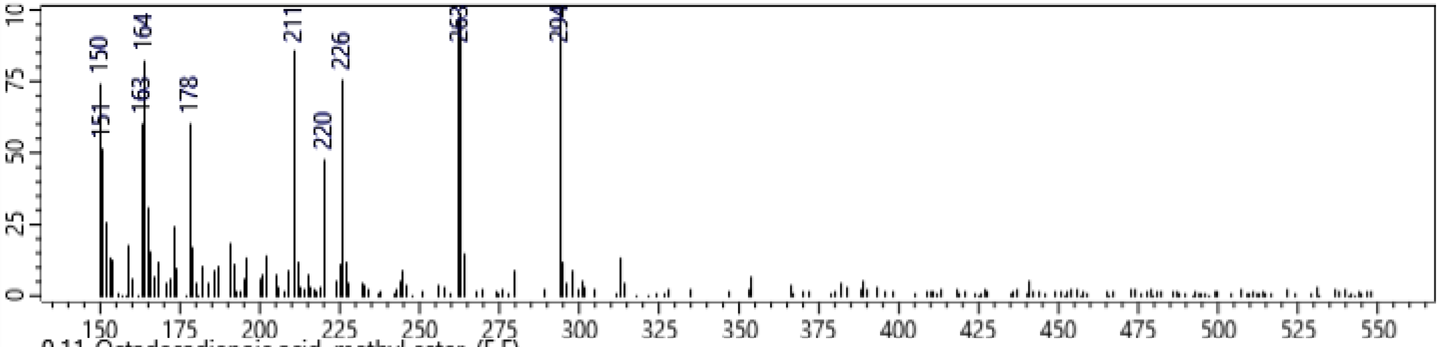


9,11-Octadecadienoic acid, methyl ester, (E,E)-

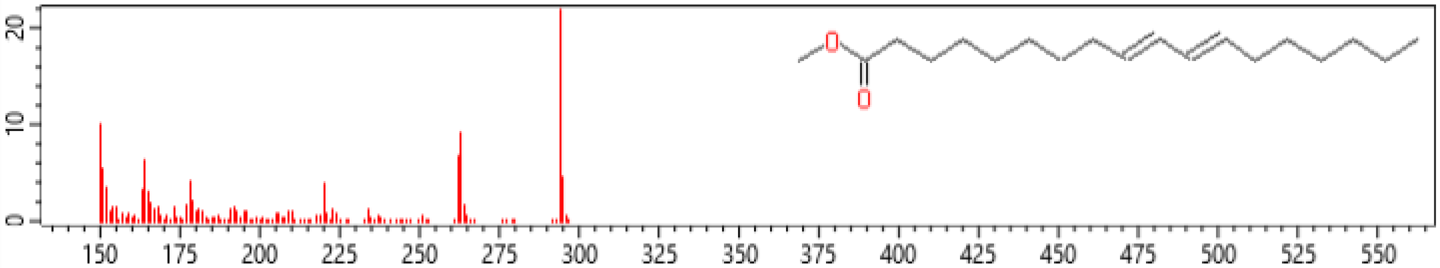

9,12-Octadecadienoic acid (Z,Z)-, methyl ester

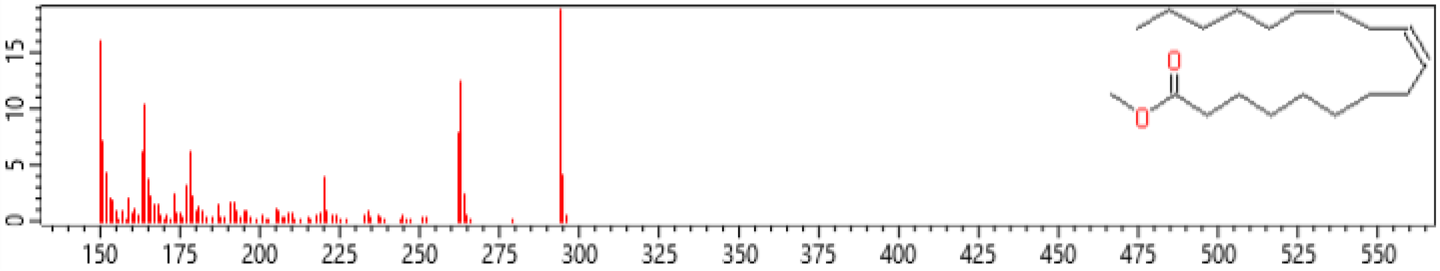

9,12-Octadecadienoic acid (Z,Z)-, methyl ester

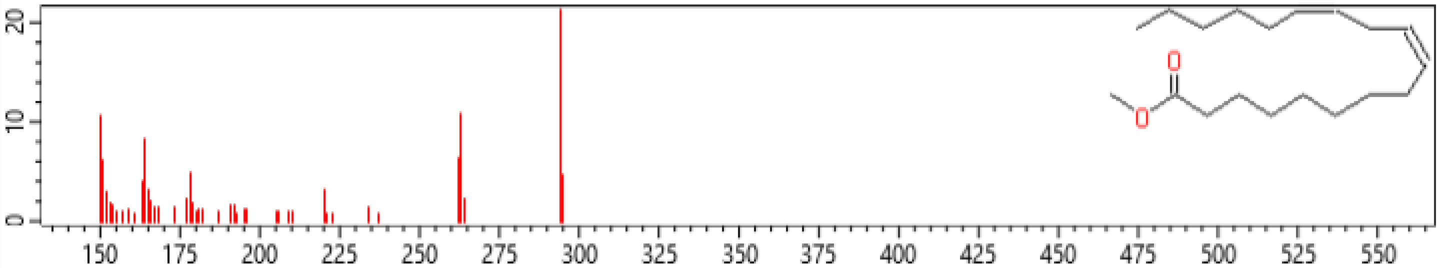

8,11-Octadecadienoic acid, methyl ester

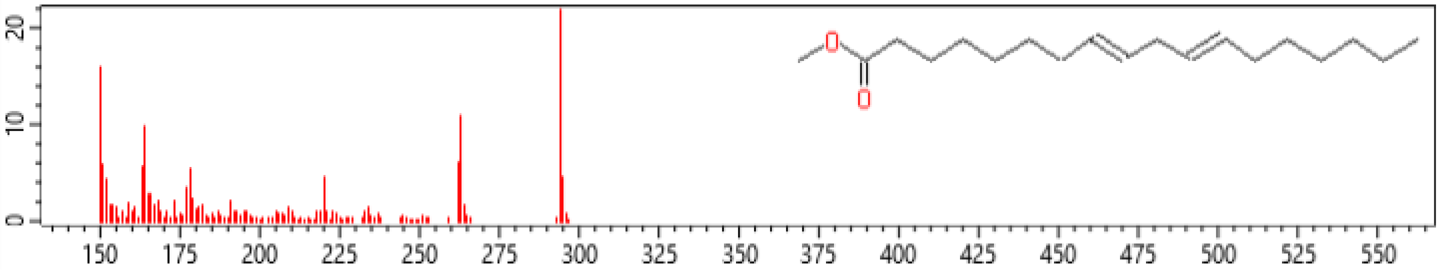

10,13-Octadecadienoic acid, methyl ester

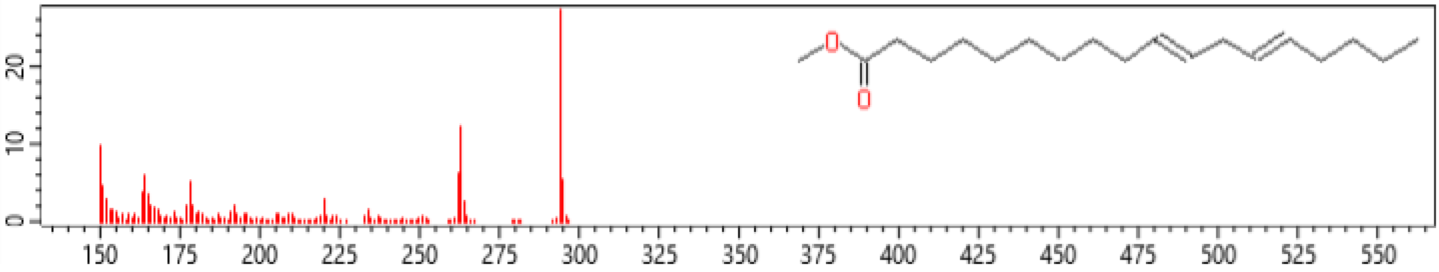

**4-Androsten-9α-fluoro-17α-methyl-3α,6β,11β,17β-tetra-ol, tetra-trimethylsilyl**
Peak: 15.98 - 16.00 min (scan #2506 - #2512) Subtracted noise: 15.94 - 15.96 min (scan #2494 - #2500), 16.02 - 16.03 min (scan #25

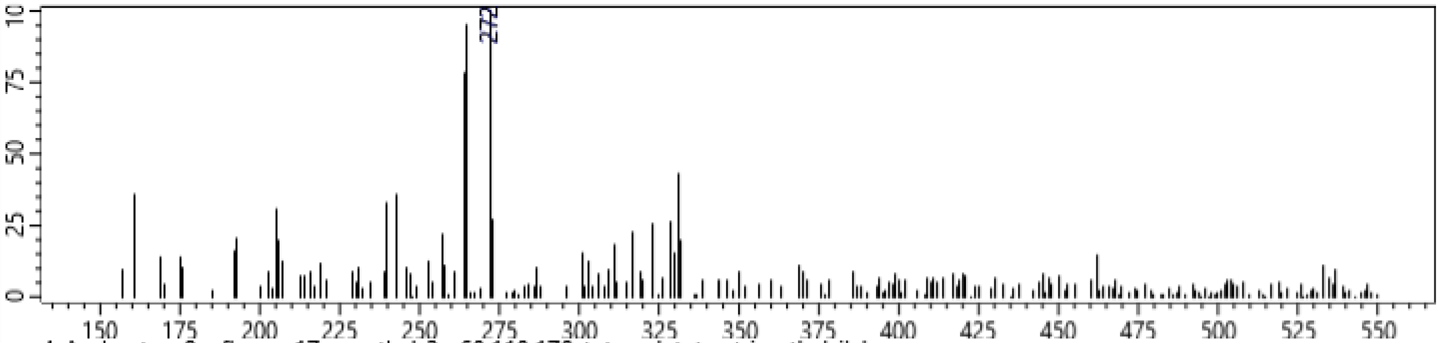


4-Androsten-9α-fluoro-17α-methyl-3α,6β,11β,17β-tetra-ol, tetra-trimethylsilyl

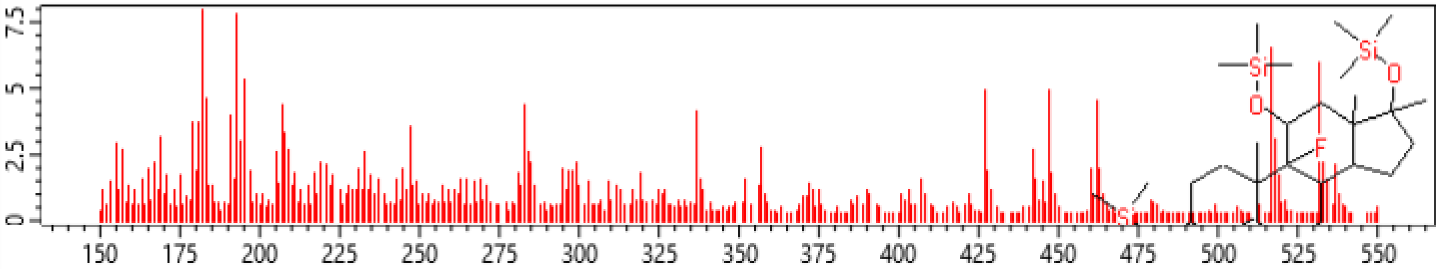

Oleyl oleate

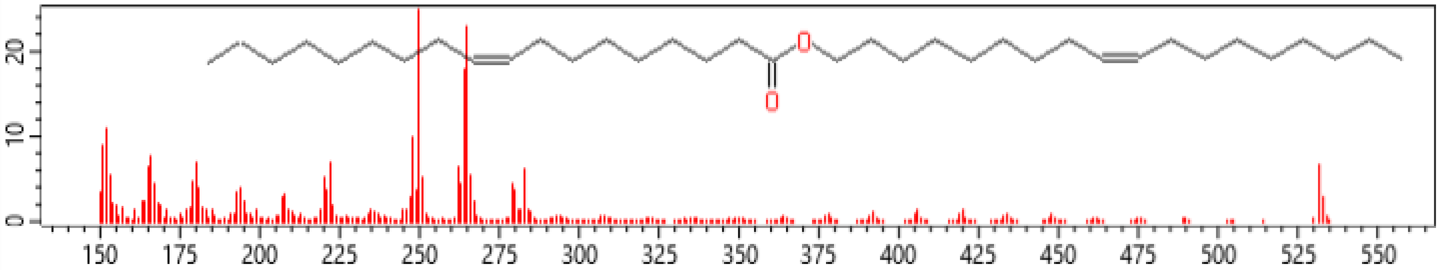

ACTH (5-10)

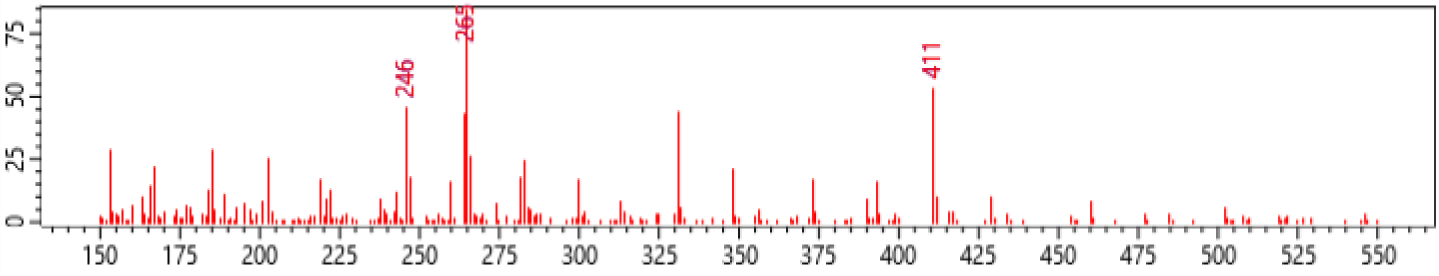

4-[5-(7-Hydroxy-5,5,8a-trimethyl-2-methylidene-3,4,4a,6,7,8-hexahydro-1H-naphthalen-1-yl)-3-methylpentoxy]-4-oxobutanoic acid,

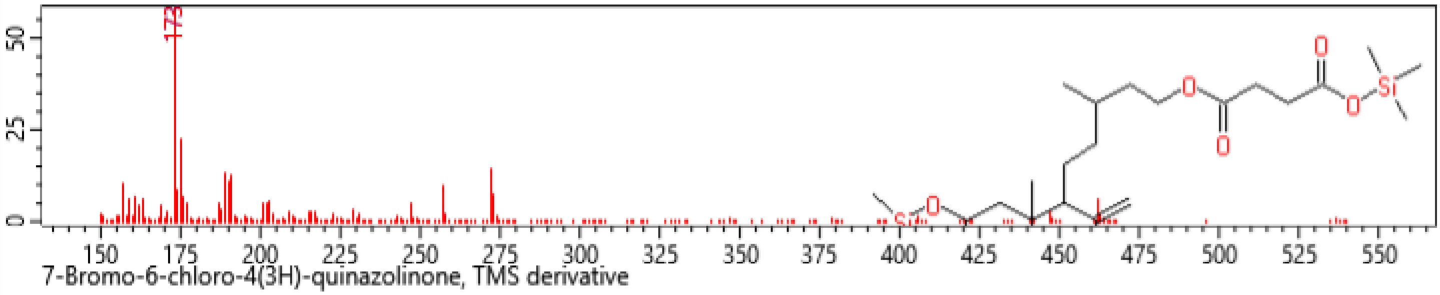


7-Bromo-6-chloro-4(3H)-quinazolinone, TMS derivative

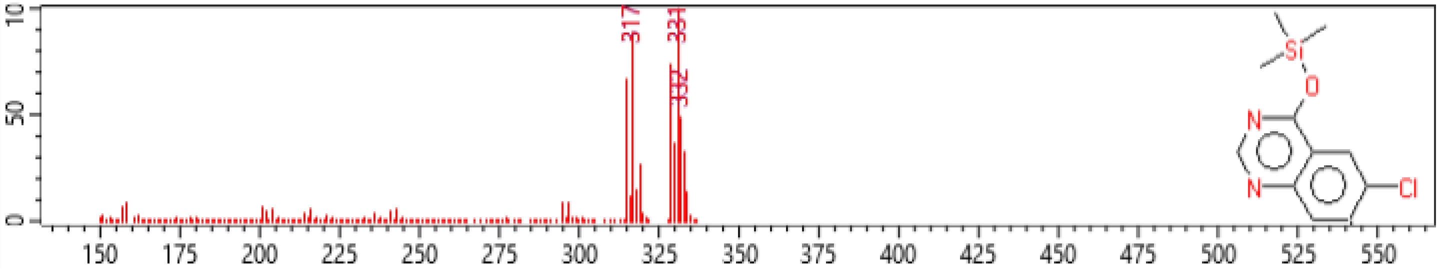


**Octadecanenitrile**

Peak: 16.02 - 16.06 min (scan #2517 - #2527) Subtracted noise: 15.98 - 16.01 min (scan #2505 - #2514), 16.09 - 16.12 min (scan #25

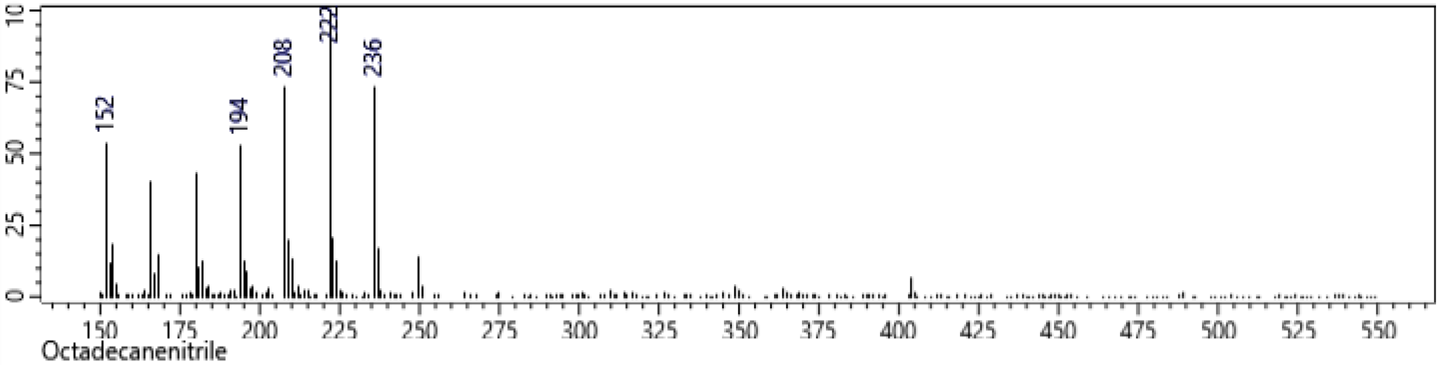


Octadecanenitrile

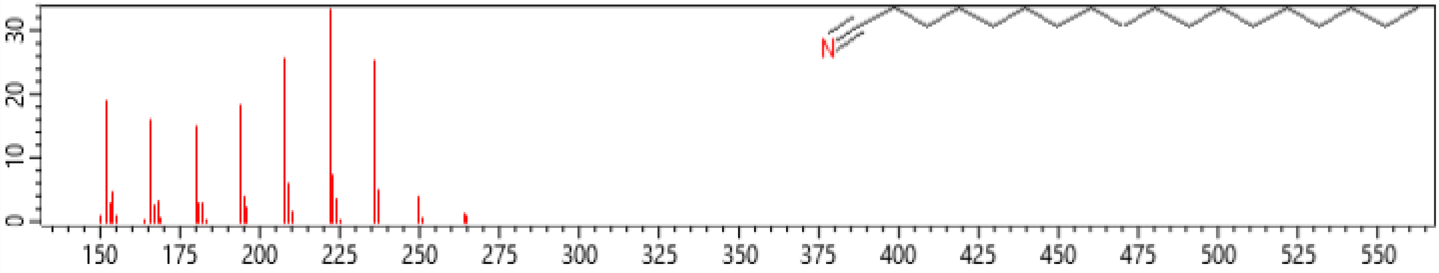

Octadecanenitrile

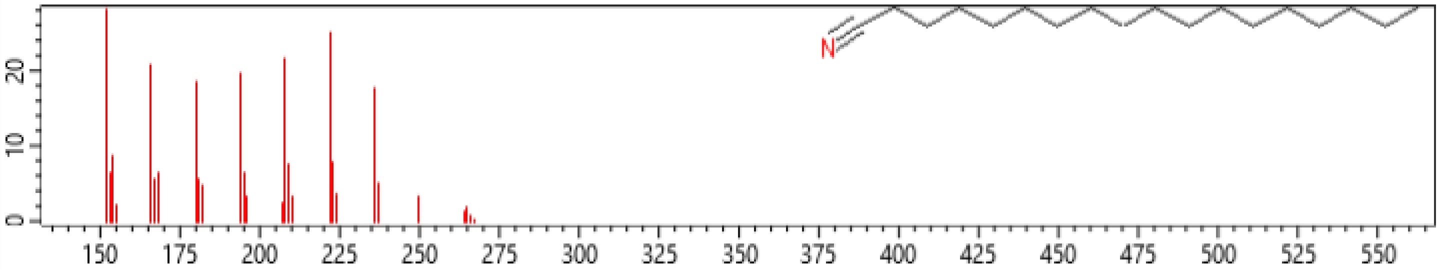

Octadecanenitrile

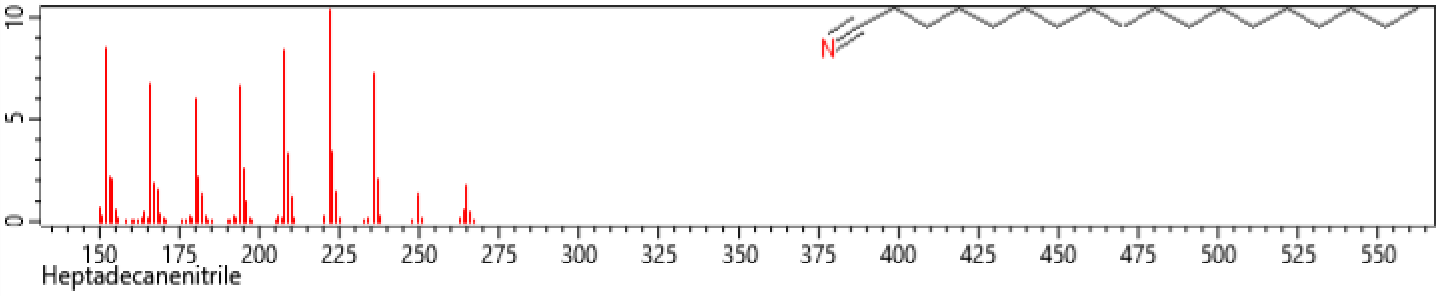

Heptadecanenitrile

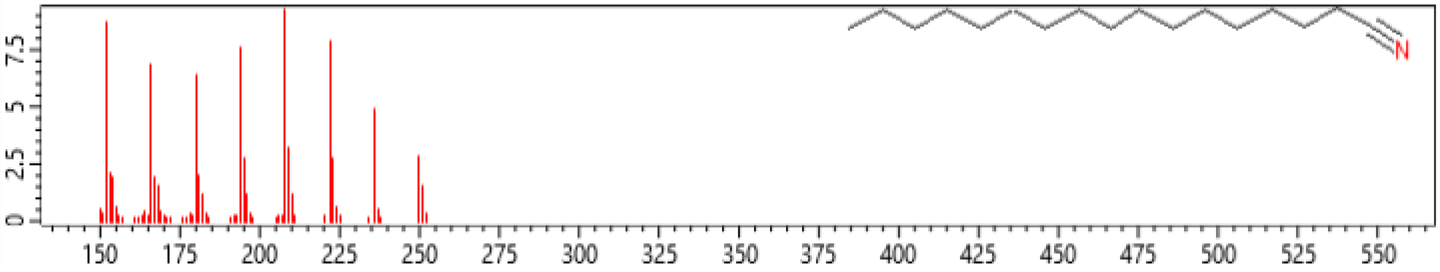

Heptadecanenitrile

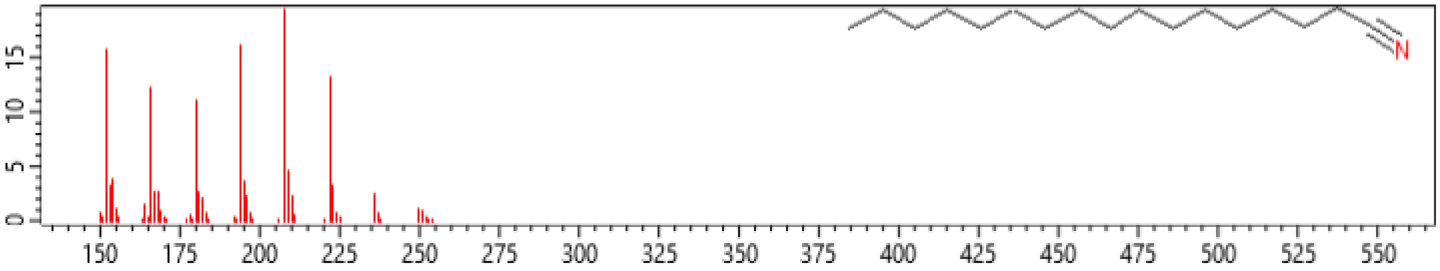

**Deoxyschizandrin**

Peak: 16.18 - 16.22 min (scan #2563 - #2573) Subtracted noise: 16.14 - 16.17 min (scan #2550 - #2559), 16.25 - 16.29 min (scan #25

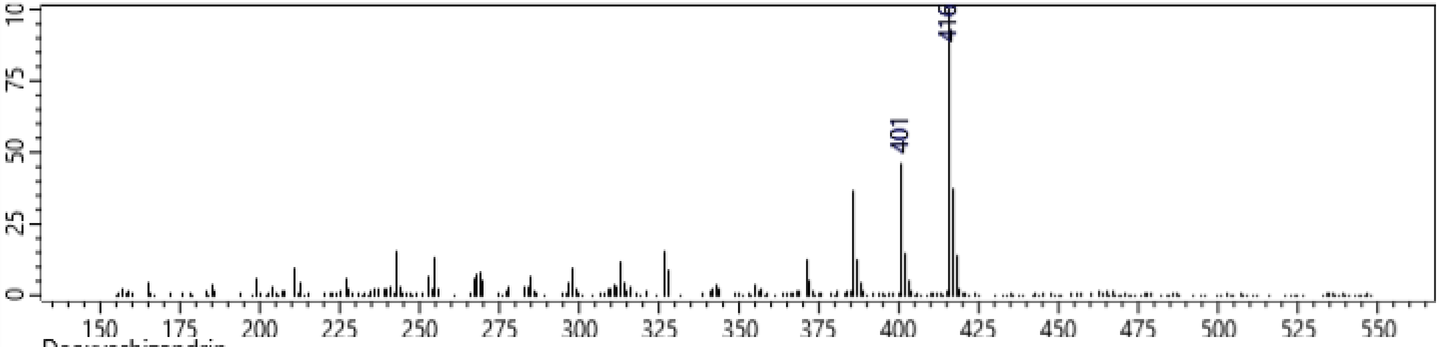

Deoxyschizandrin

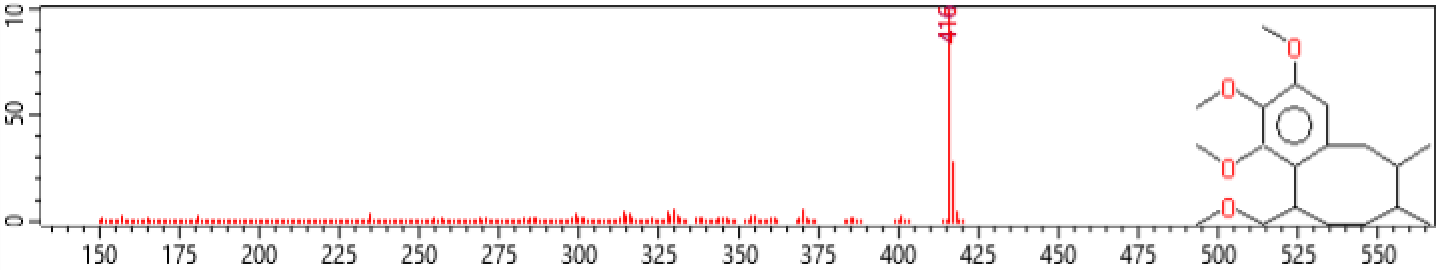

2'-(Trimethylsilyl)oxy-3,4,5,6'-tetramethoxychalcone

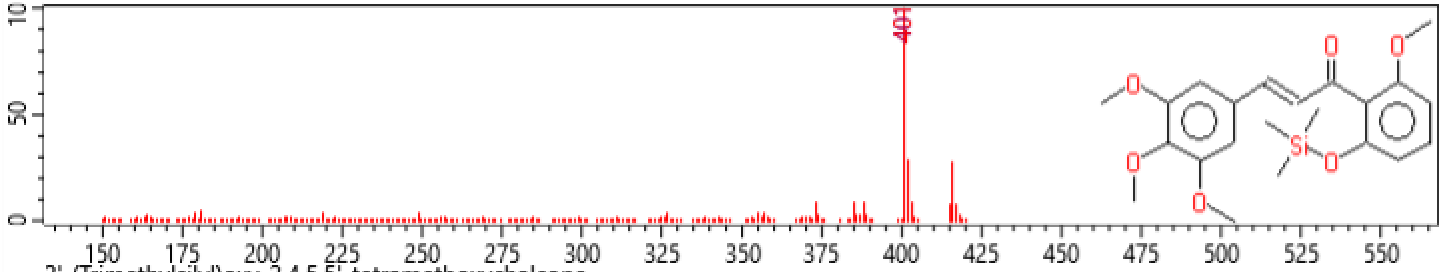


2'-(Trimethylsilyl)oxy-2,4,5,5'-tetramethoxychalcone

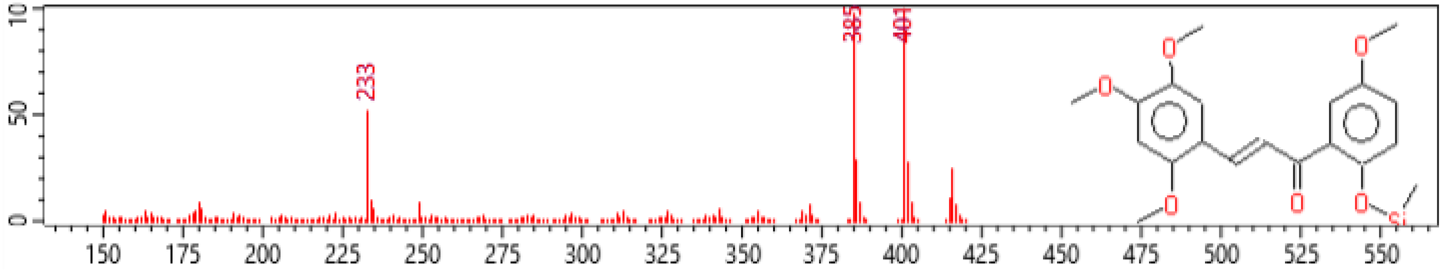


2'-(Trimethylsilyl)oxy-3,4,4',5-tetramethoxychalcone (isomer 2)

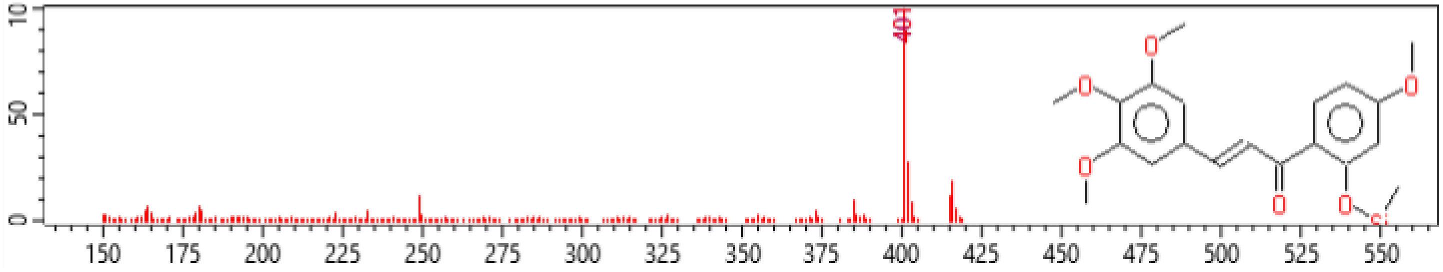


2-(4-Chlorophenyl)-1H-benzimidazole-5-carboxylic acid, 2TMS (isomer 1)

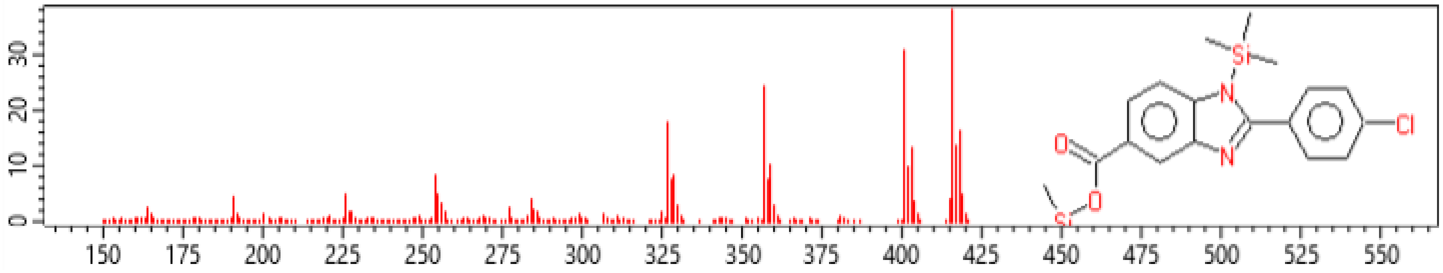

**1,3-Dioxane, 5-(hexadecyloxy)-2-pentadecyl-, trans-**

Peak: 16.35 - 16.39 min (scan #2609 - #2619) Subtracted noise: 16.27 - 16.30 min (scan #2587 - #2596), 16.39 - 16.42 min (scan #26

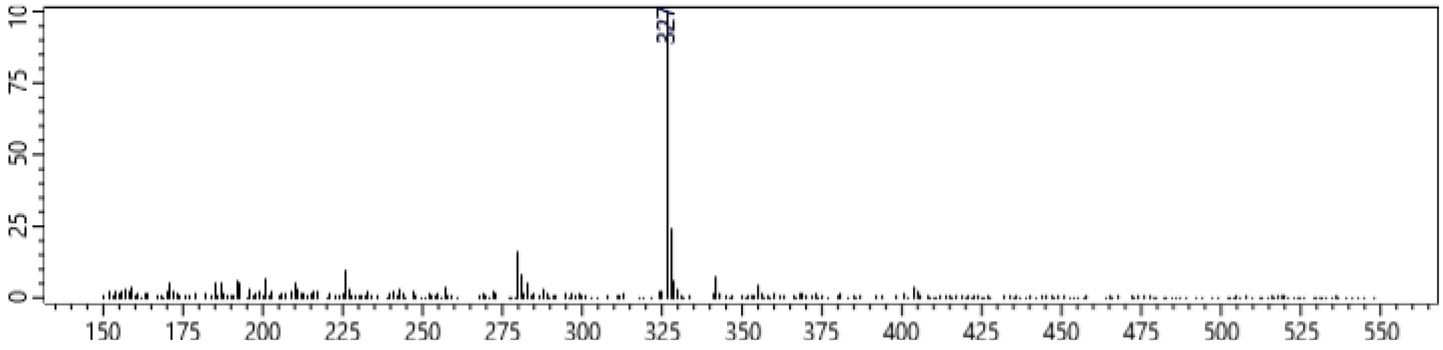

1,3-Dioxane, 5-(hexadecyloxy)-2-pentadecyl-, trans-

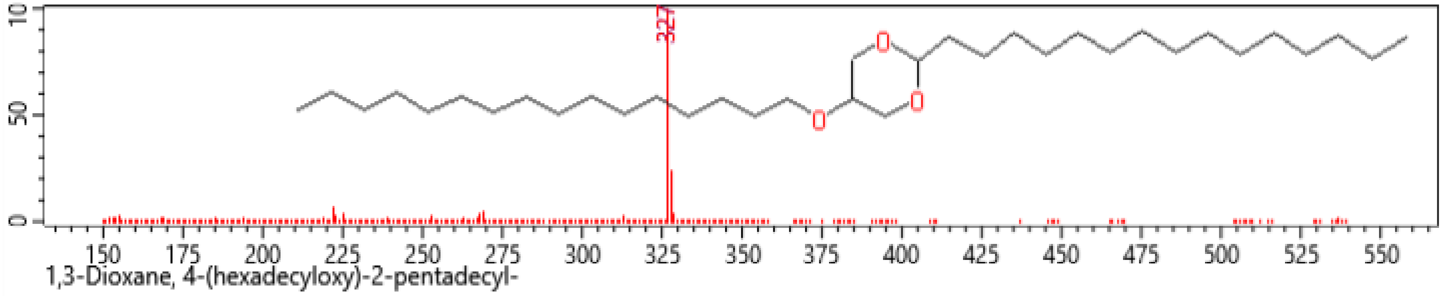


1,3-Dioxane, 4-(hexadecyloxy)-2-pentadecyl-

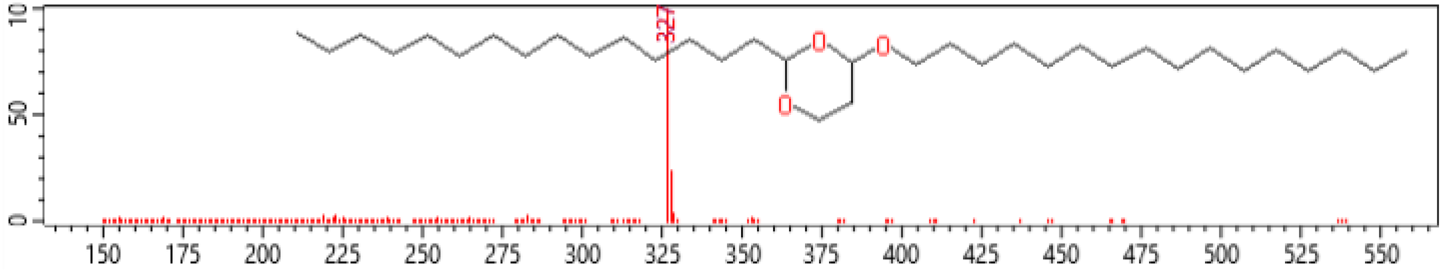


Irisolidone, methyl ether

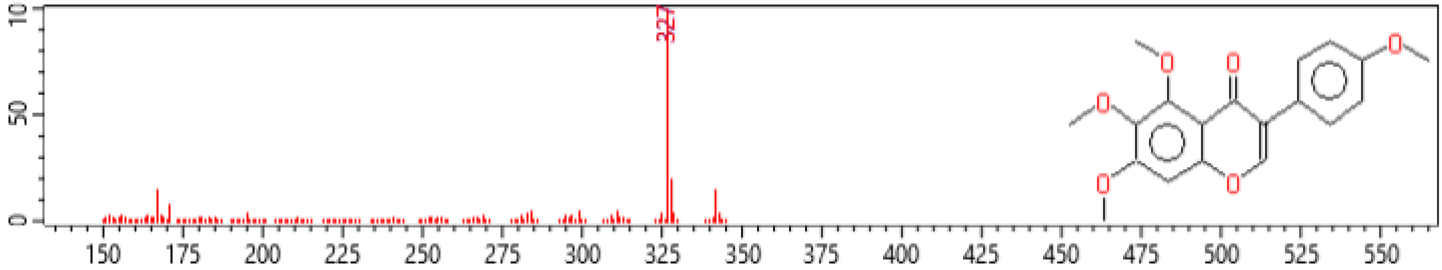


Heptadecanoic acid, TMS derivative

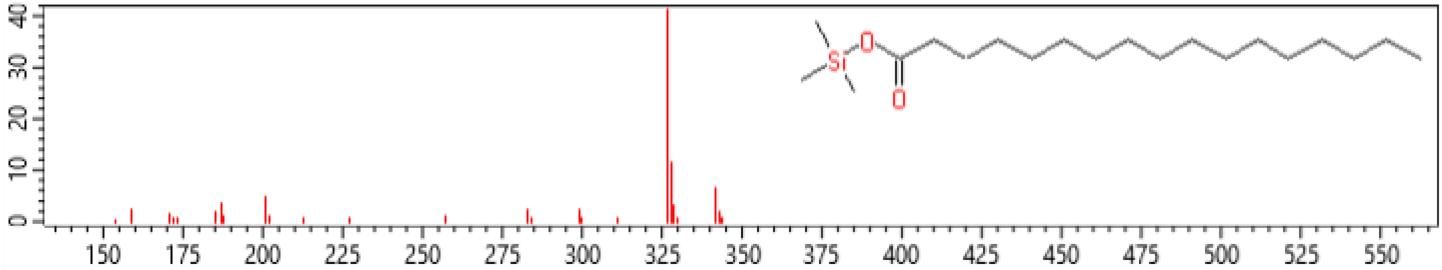


5,6-Difluoro-1H-indazole-3-carboxylic acid, 2TMS

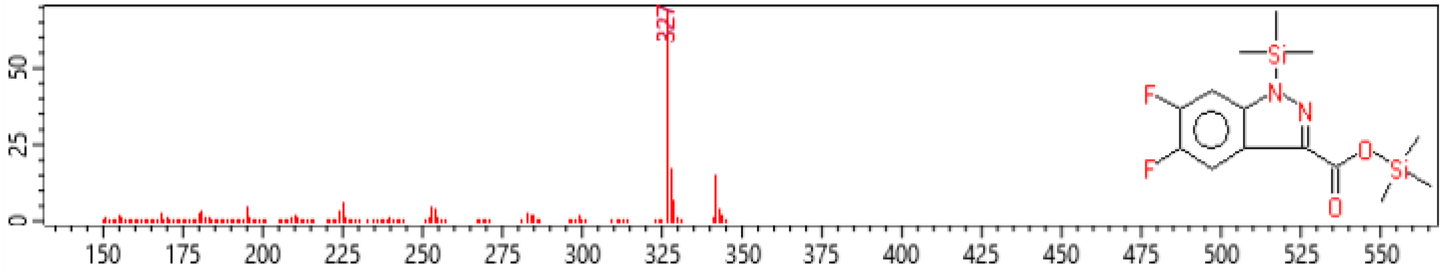

**Silane, dimethyl(octadecyloxy)propyl-**

Peak: 16.48 - 16.51 min (scan #2645 - #2653) Subtracted noise: 16.42 - 16.45 min (scan #2630 - #2637), 16.52 - 16.54 min (scan #26

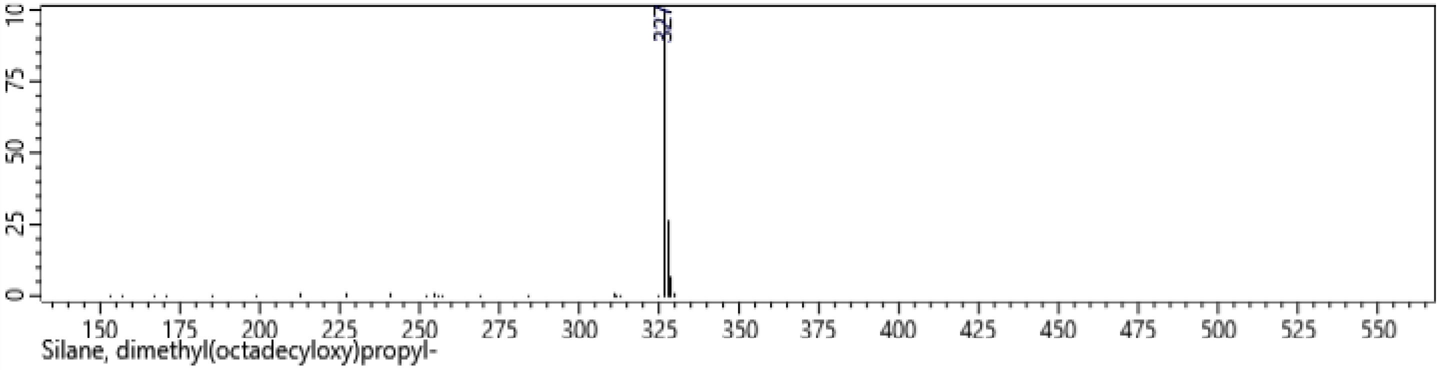


Silane, dimethyl(octadecyloxy)propyl-

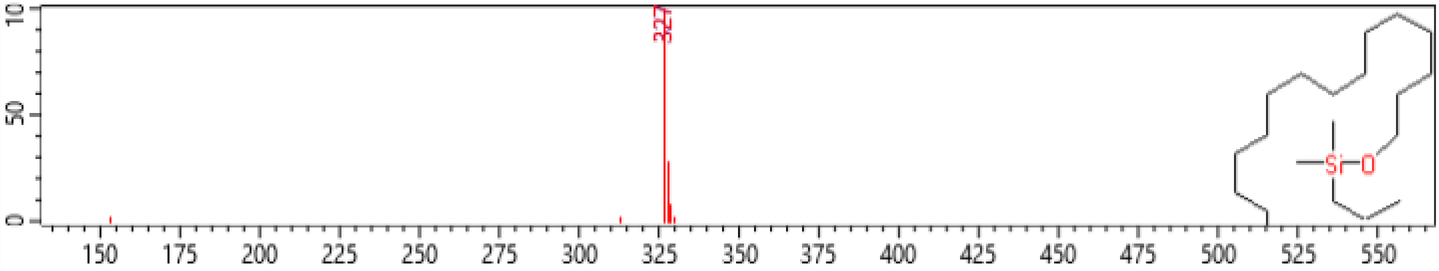


1-Octadecanol, TMS derivative

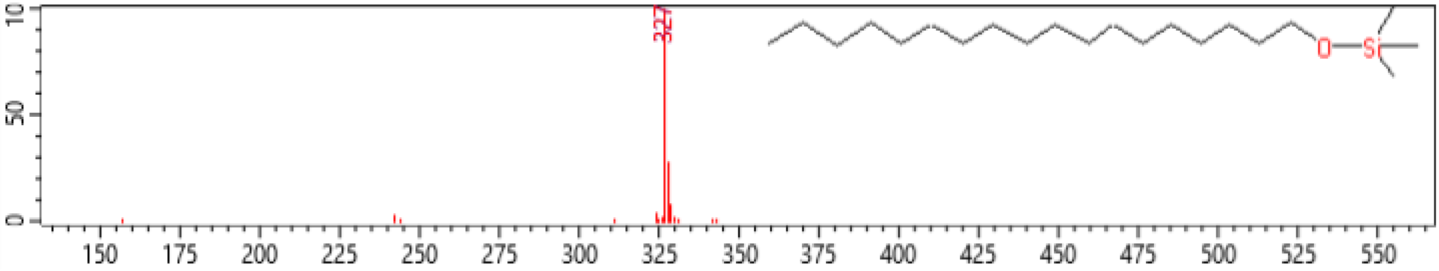


Octadecane-1,2-diol, 2TMS derivative

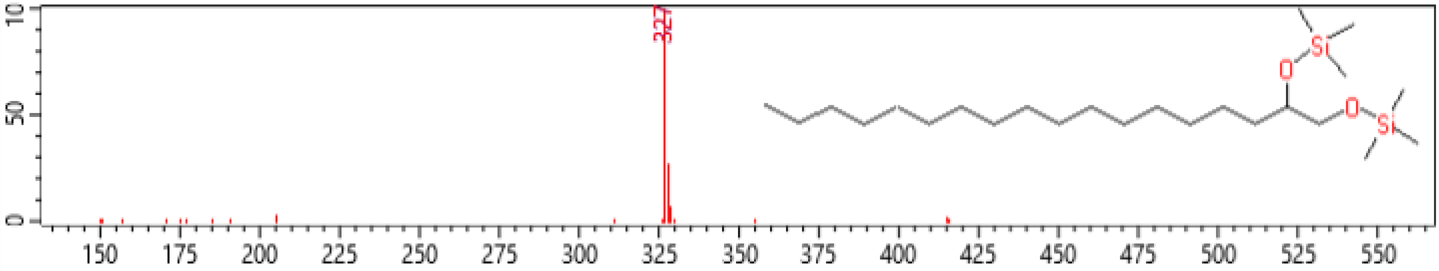


1-Dichloromethyl(dimethyl)silyloxyoctadecane

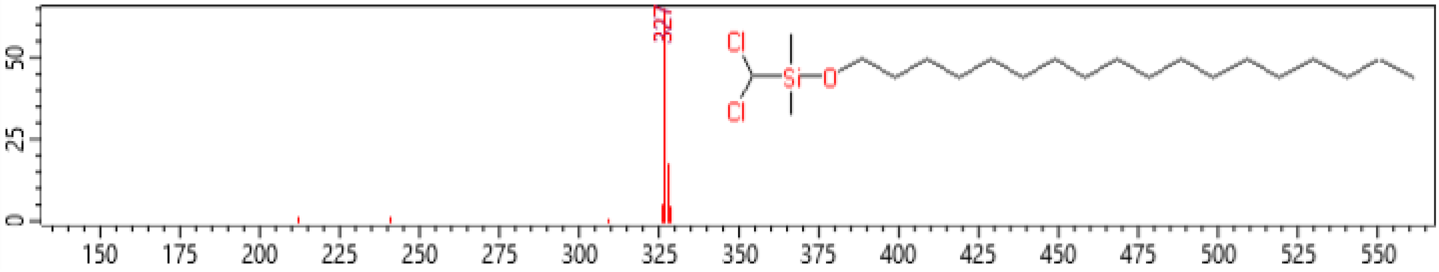

1-Dimethylthexylsilyloxyoctadecane

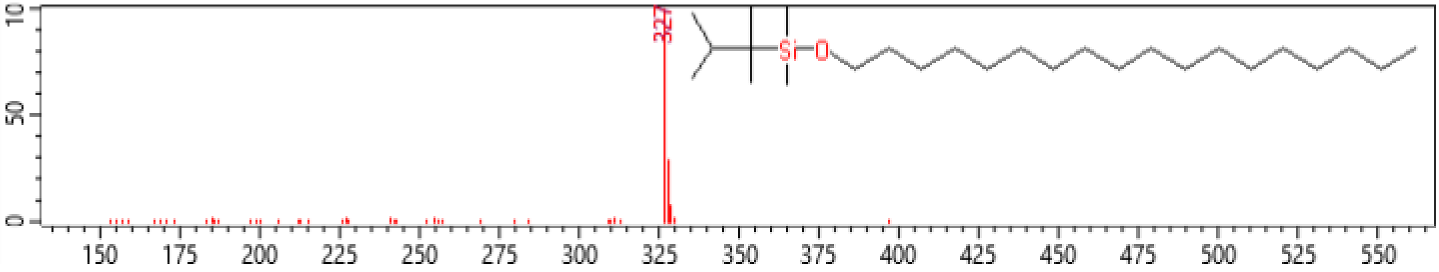


**Linoleic acid ethyl ester**

Peak: 16.51 - 16.54 min (scan #2655 - #2661) Subtracted noise: 16.49 - 16.51 min (scan #2648 - #2654), 16.57 - 16.59 min (scan #26

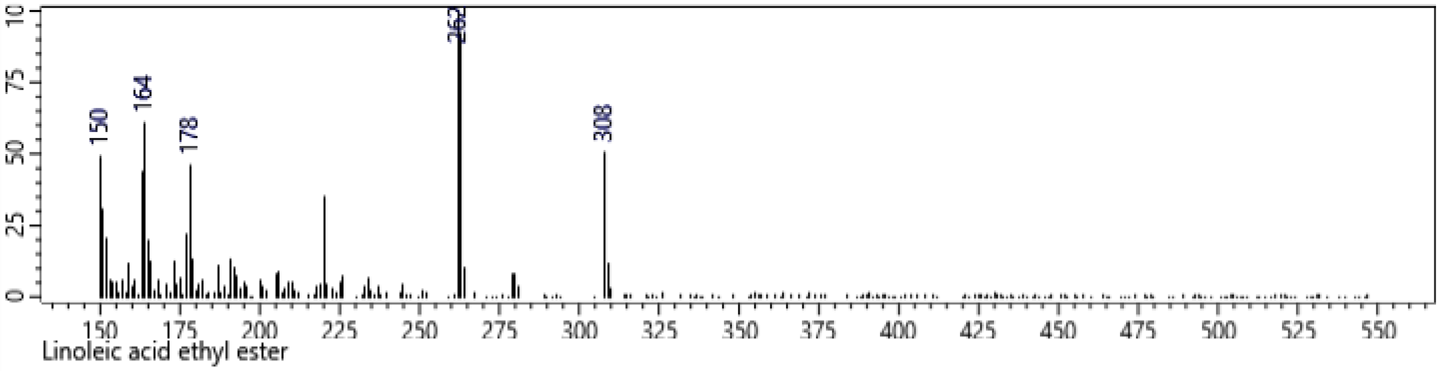


Linoleic acid ethyl ester

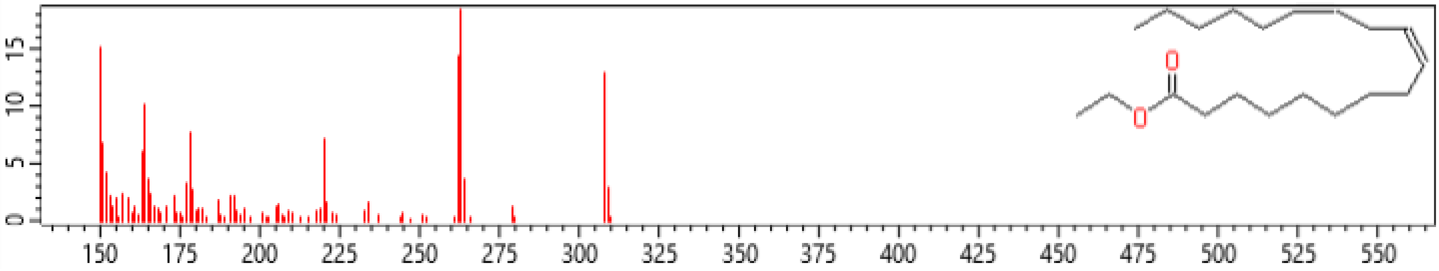


Linoleic acid ethyl ester

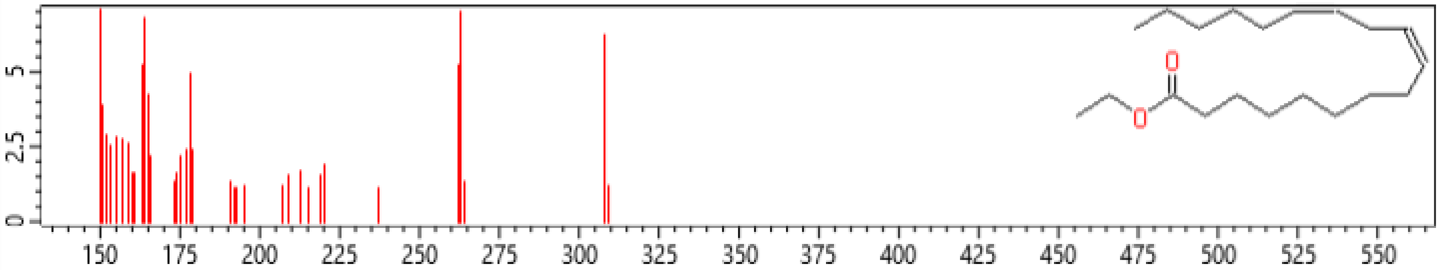


Linoleic acid ethyl ester

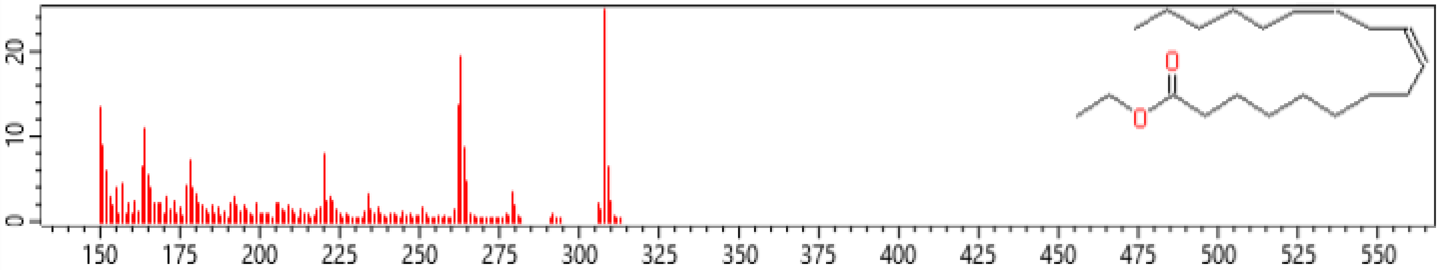

9,12-Octadecadienoic acid, ethyl ester

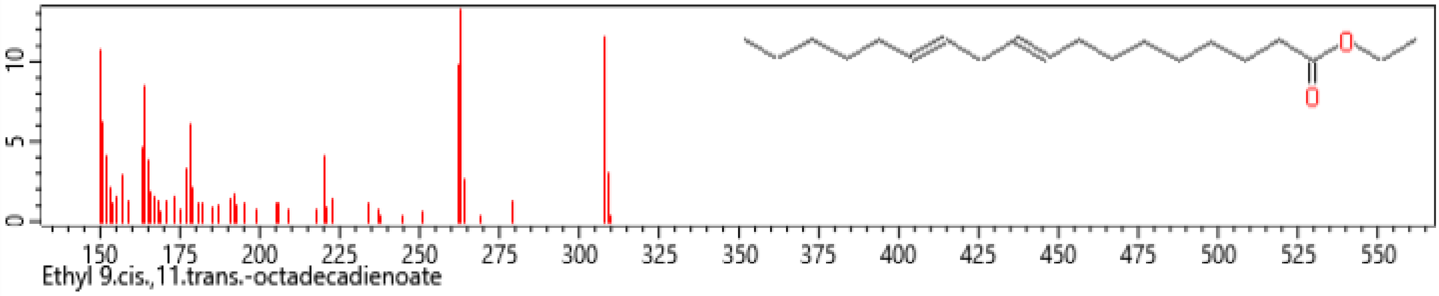

Ethyl 9.cis.,11.trans.-octadecadienoate

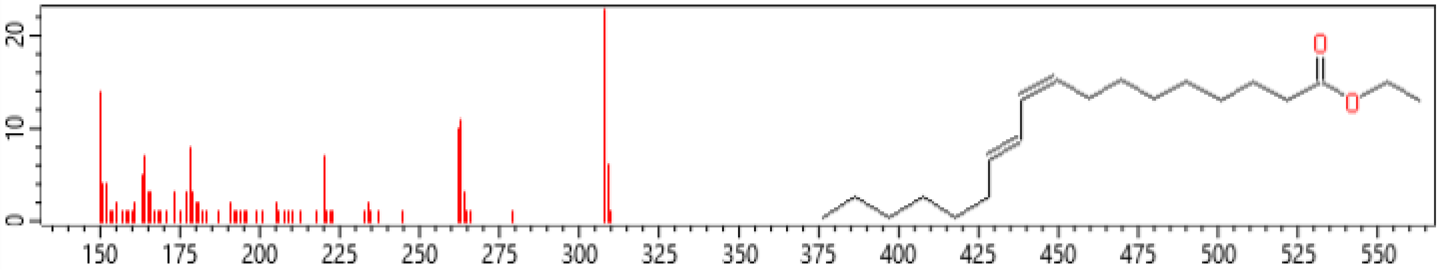

**Silane, diethylpentyloxypropoxy-**

Peak: 16.60 - 16.63 min (scan #2678 - #2688) Subtracted noise: 16.53 - 16.56 min (scan #2658 - #2669), 16.64 - 16.68 min (scan #26[illegible]

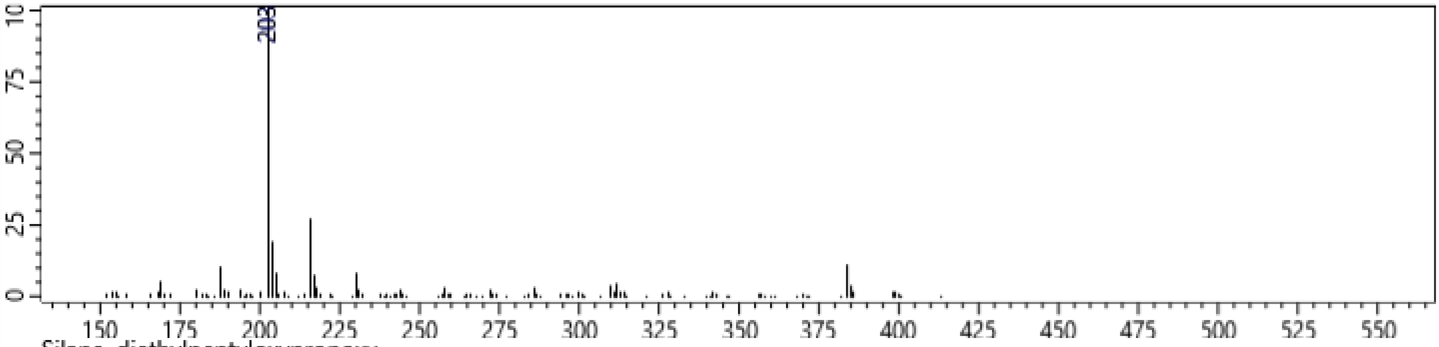


Silane, diethylpentyloxypropoxy-

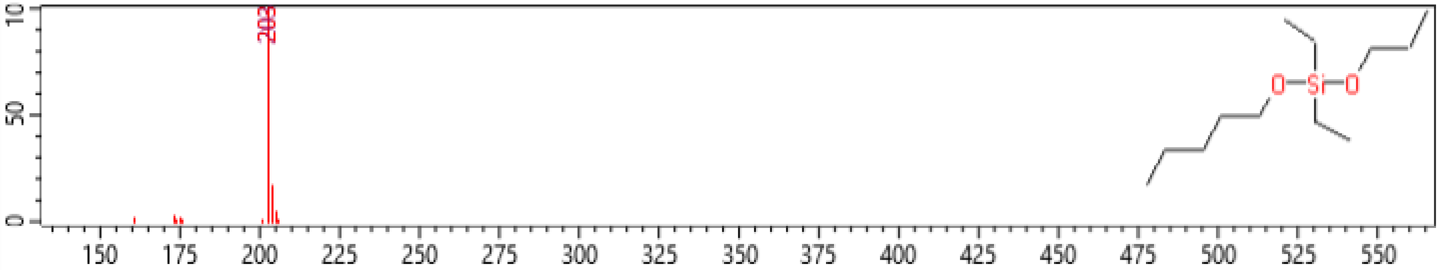


Silane, diethylethoxy(2-methylpent-3-yloxy)-

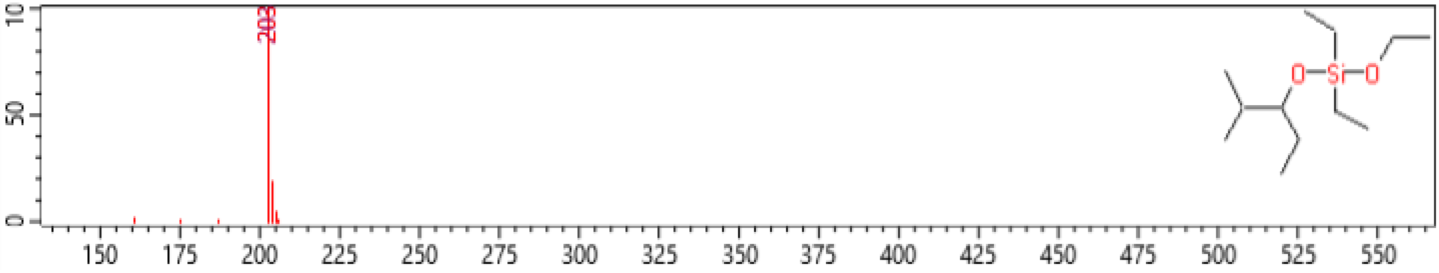

Pro-Ser

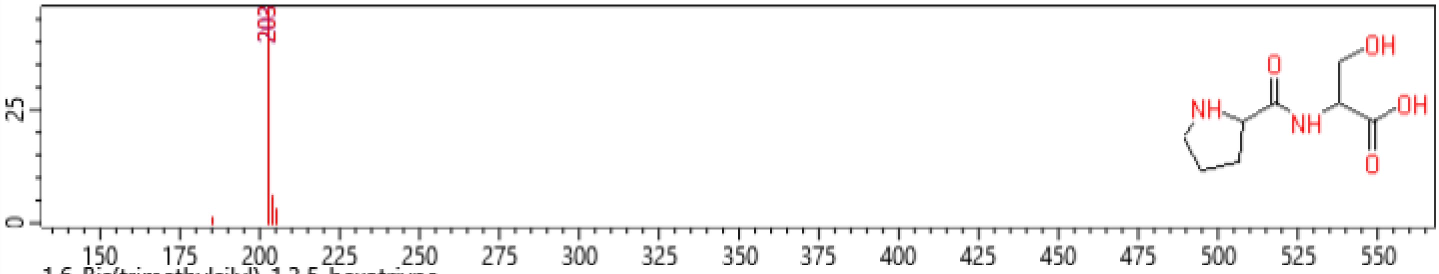


1,6-Bis(trimethylsilyl)-1,3,5-hexatriyne

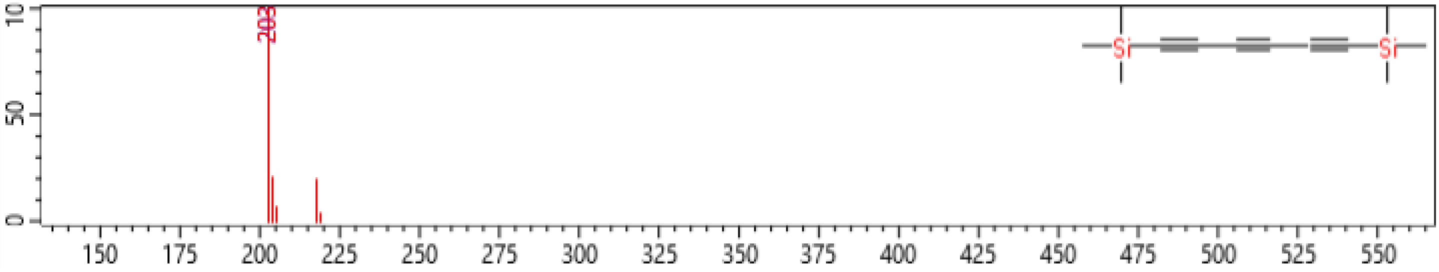


1-Benzyl-4-(2-((4-fluorophenyl)sulfonyl)ethyl)piperazine

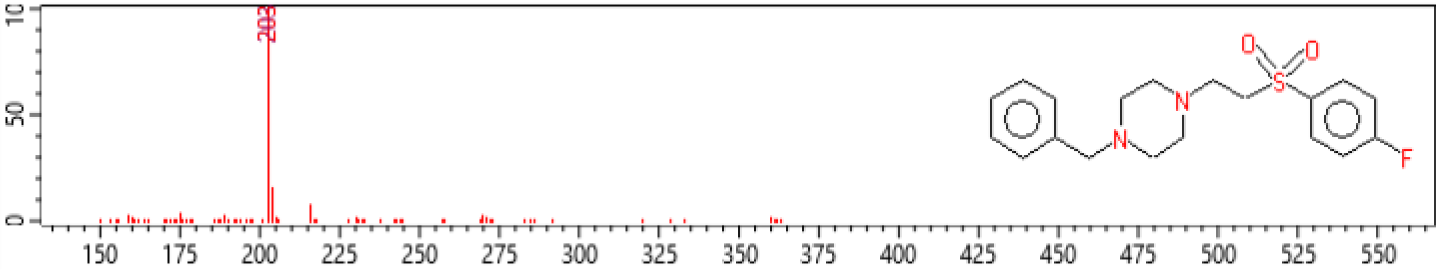


**Octadecanoic acid, ethyl ester**

Peak: 16.83 - 16.85 min (scan #2744 - #2748) Subtracted noise: 16.79 - 16.81 min (scan #2733 - #2737), 16.85 - 16.87 min (scan #27

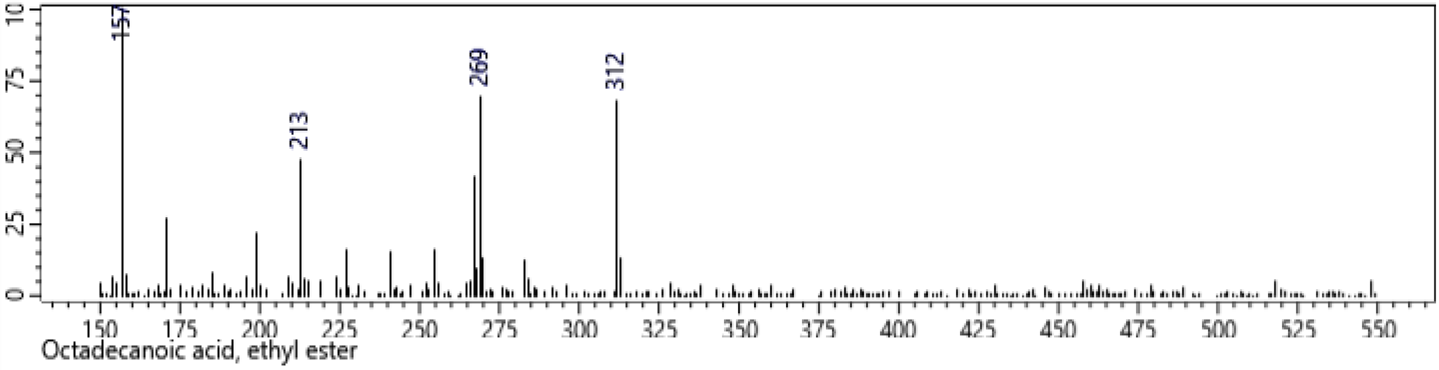


Octadecanoic acid, ethyl ester

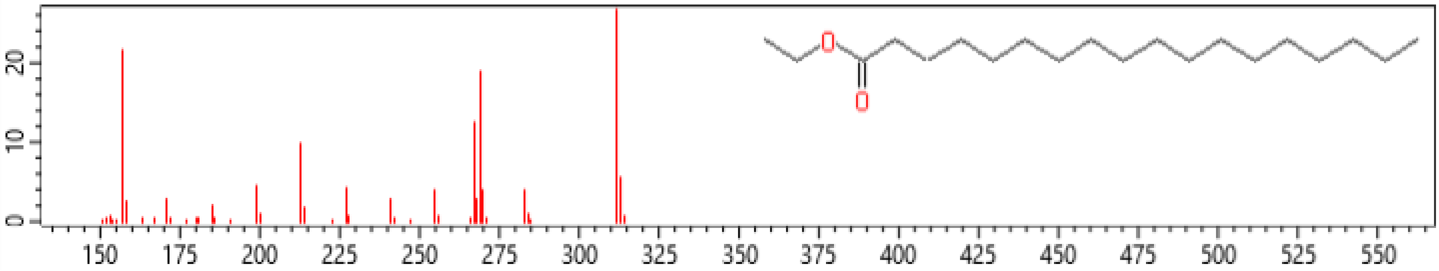

Octadecanoic acid, ethyl ester

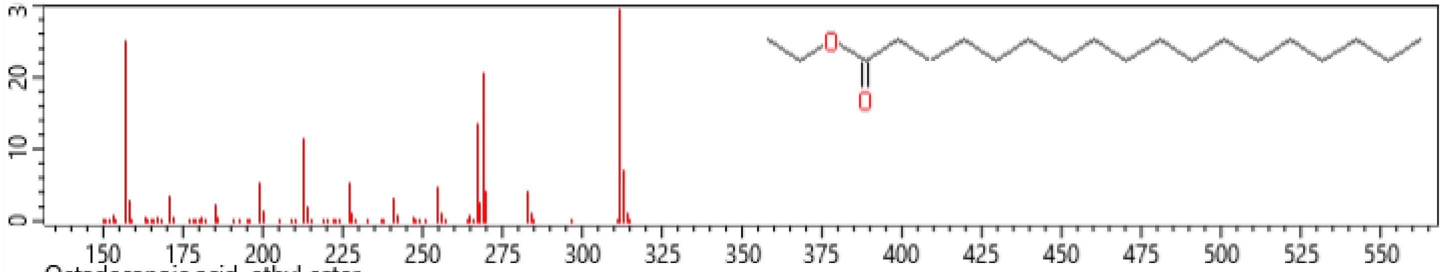


Octadecanoic acid, ethyl ester

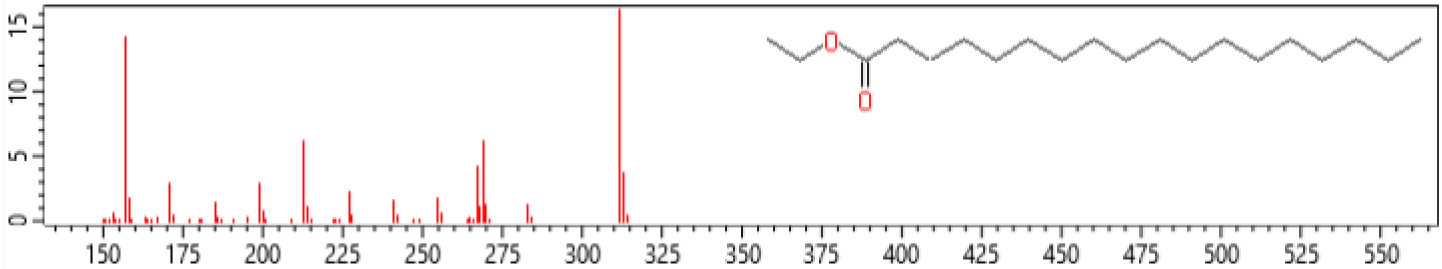


Octadecanoic acid, ethyl ester

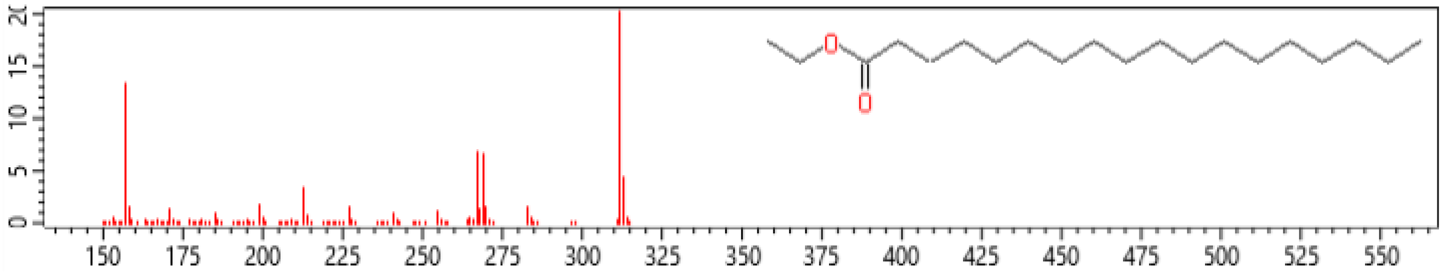


Eicosanoic acid

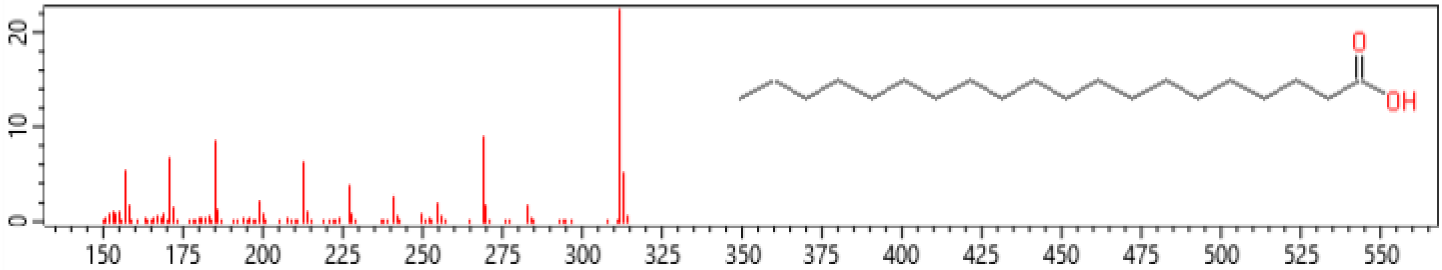


**Monolaurin, 2TMS derivative**

Peak: 16.92 - 16.95 min (scan #2768 - #2776) Subtracted noise: 16.88 - 16.90 min (scan #2756 - #2764), 16.97 - 17.00 min (scan #27

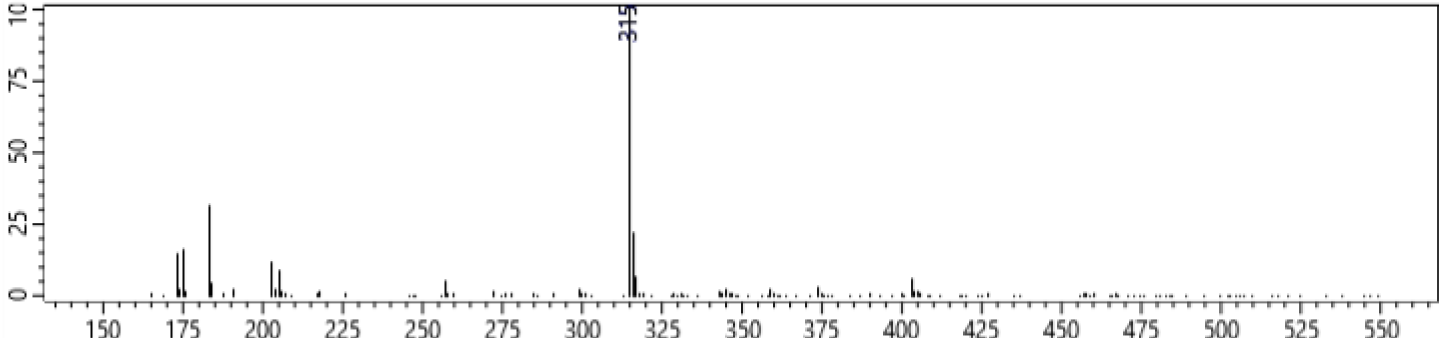

Monolaurin, 2TMS derivative

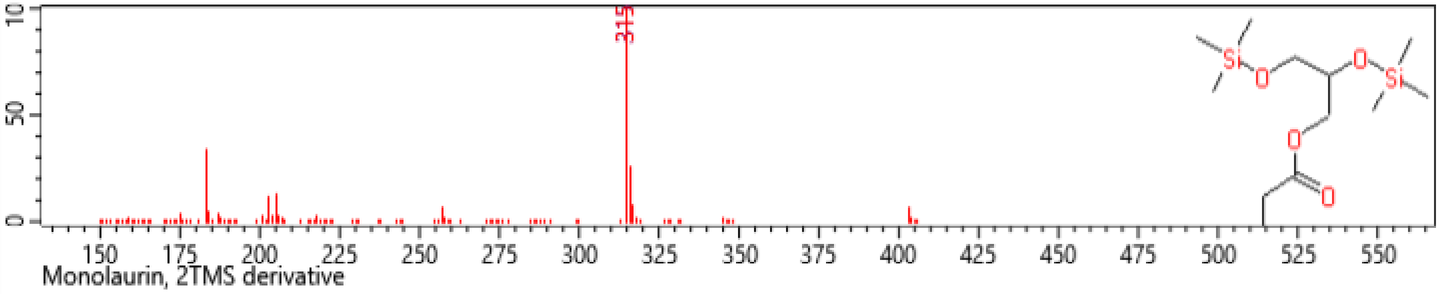


Monolaurin, 2TMS derivative

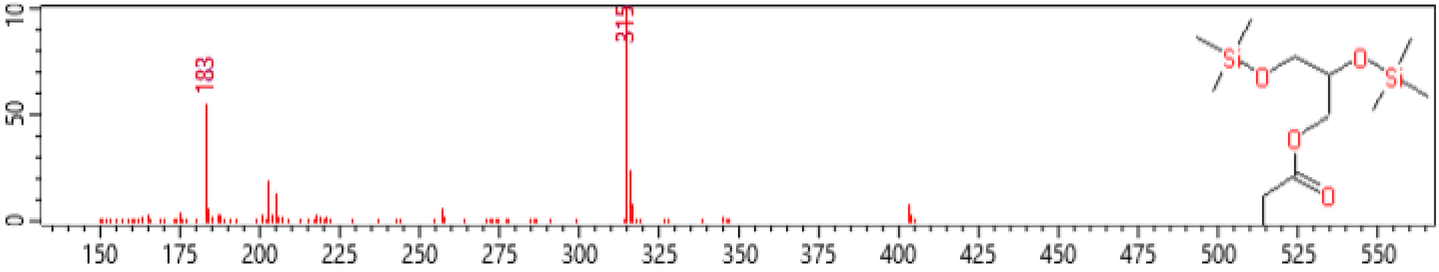


Monolaurin, 2TMS derivative

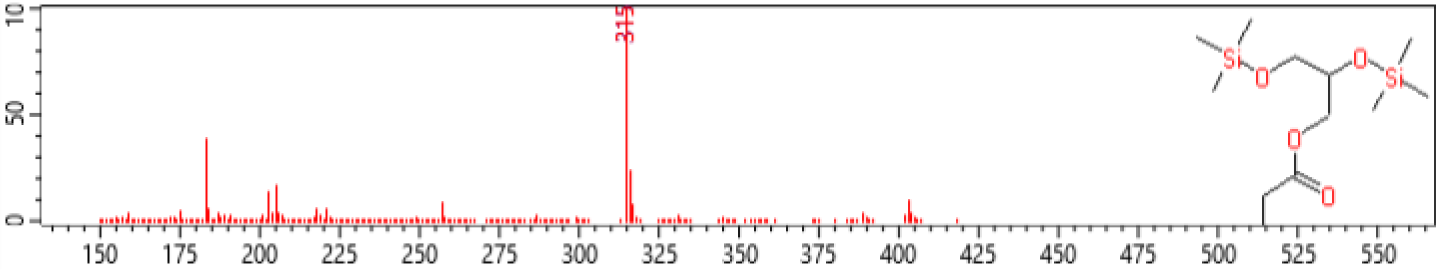


4-[3-(3,4-Difluorophenyl)-[1,2,4]triazolo[3,4-b][1,3,4]thiadiazol-6-yl]pyridine

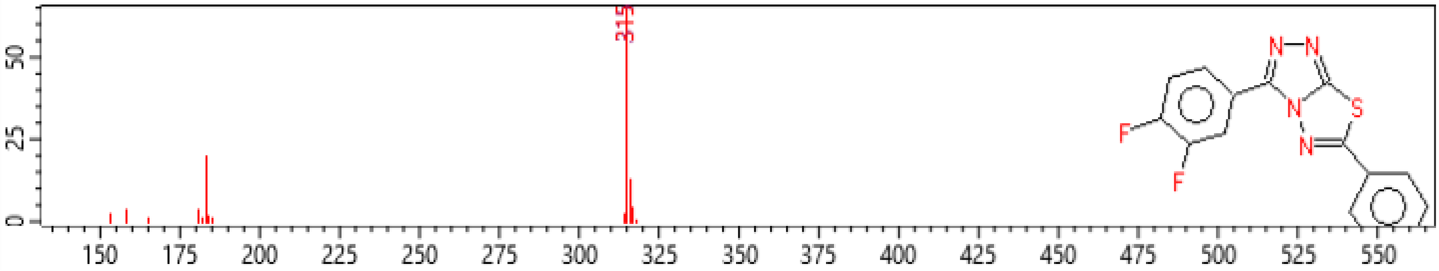


Silane, dimethyl(3,7-dimethyloct-3-yloxy)heptyloxy-

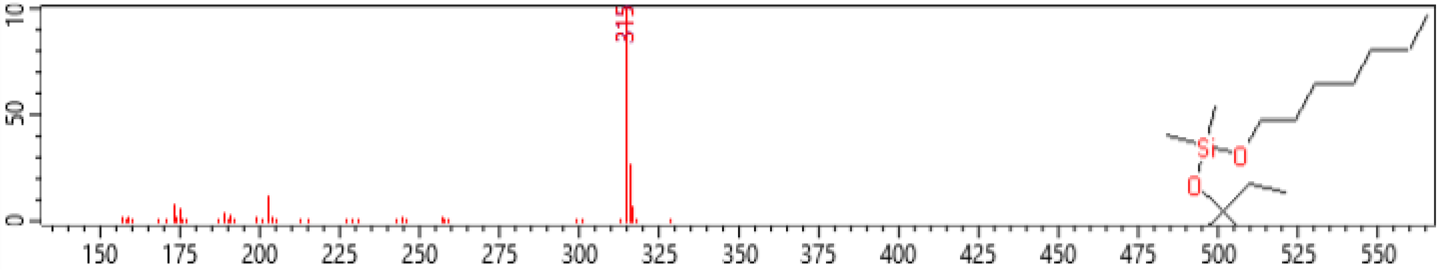

**1,1,3-Tris(2-methyl-4-hydroxy-5-tert-butylphenyl)butane**

Peak: 17.01 - 17.04 min (scan #2793 - #2803) Subtracted noise: 16.94 - 16.97 min (scan #2773 - #2782), 17.06 - 17.09 min (scan #28

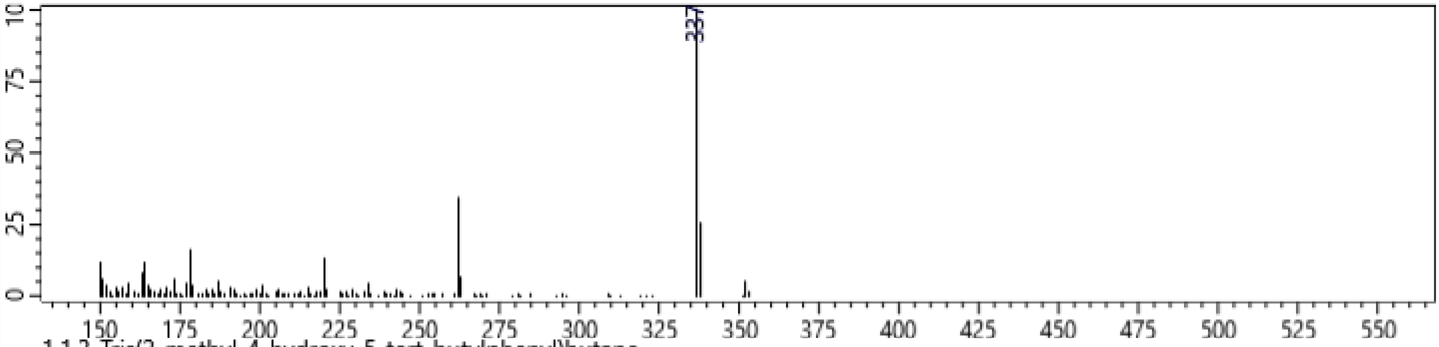


1,1,3-Tris(2-methyl-4-hydroxy-5-tert-butylphenyl)butane

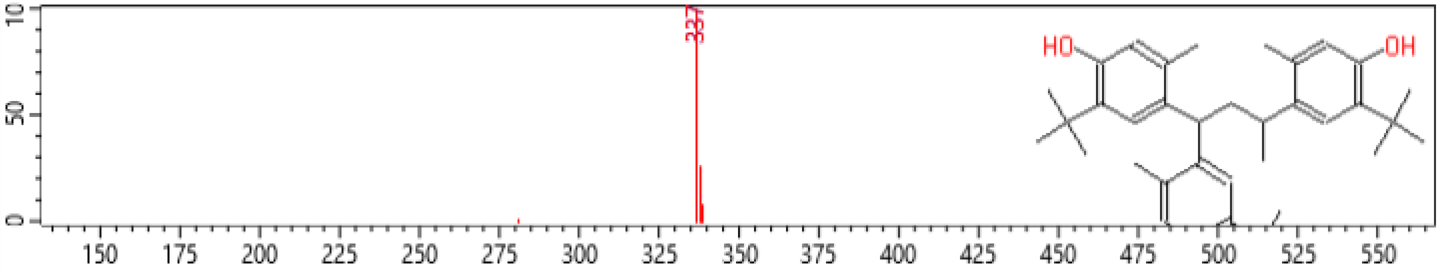


9,12-Octadecadienoic acid (Z,Z)-, TMS derivative

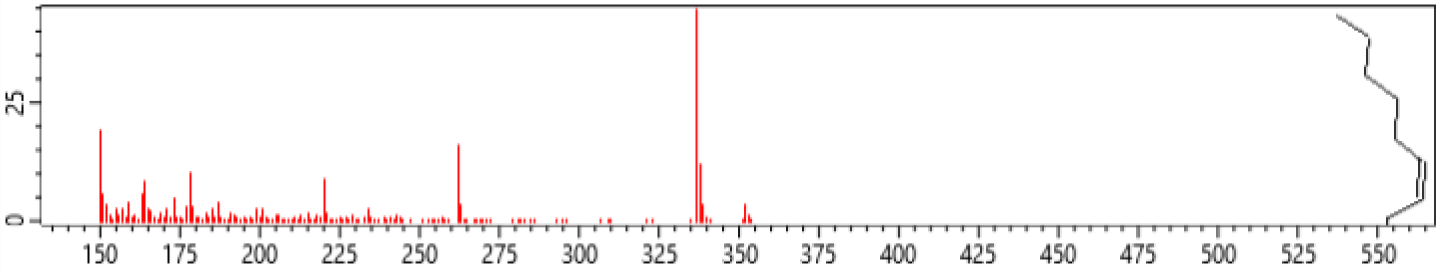

9,12-Octadecadienoic acid (Z,Z)-, TMS derivative

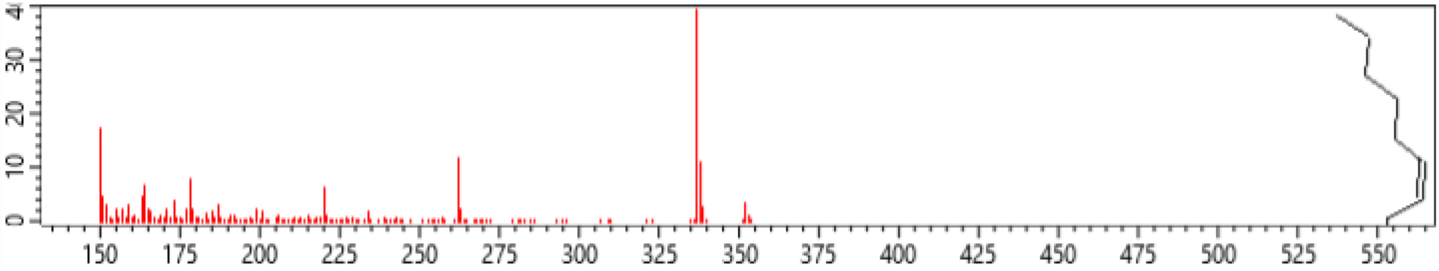

9,12-Octadecadienoic acid (Z,Z)-, TMS derivative

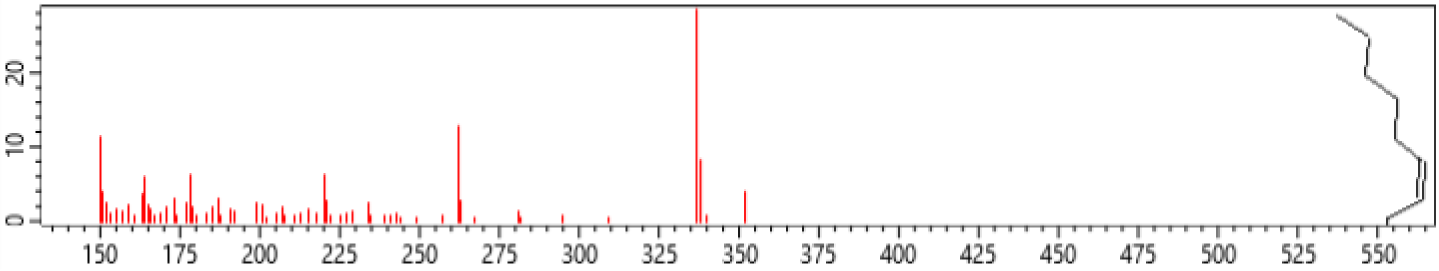

9(E),11(E)-Conjugated linoleic acid, trimethylsilyl ester

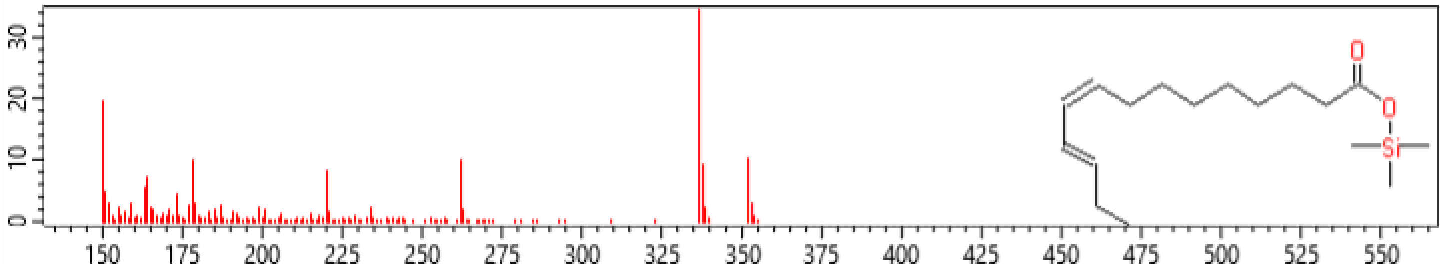


**D-myo-Inositol-1,4-diphosphate**
Peak: 17.06 - 17.10 min (scan #2808 - #2818) Subtracted noise: 17.01 - 17.05 min (scan #2794 - #2804), 17.13 - 17.17 min (scan #28

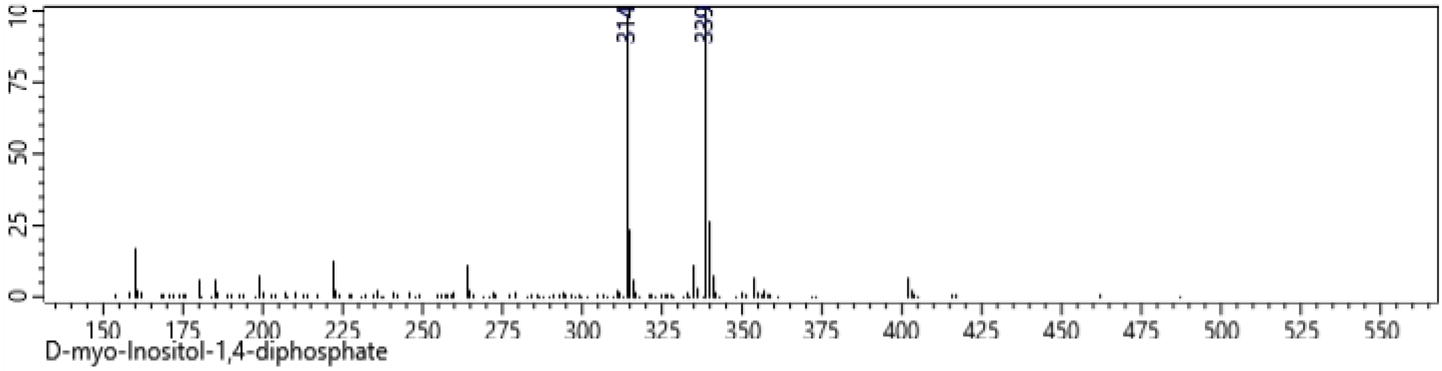


D-myo-Inositol-1,4-diphosphate

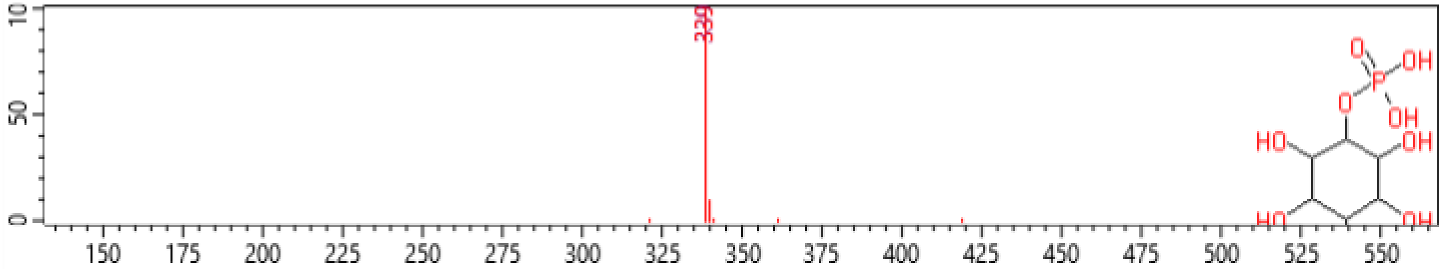


Oleic Acid, (Z)-, TMS derivative

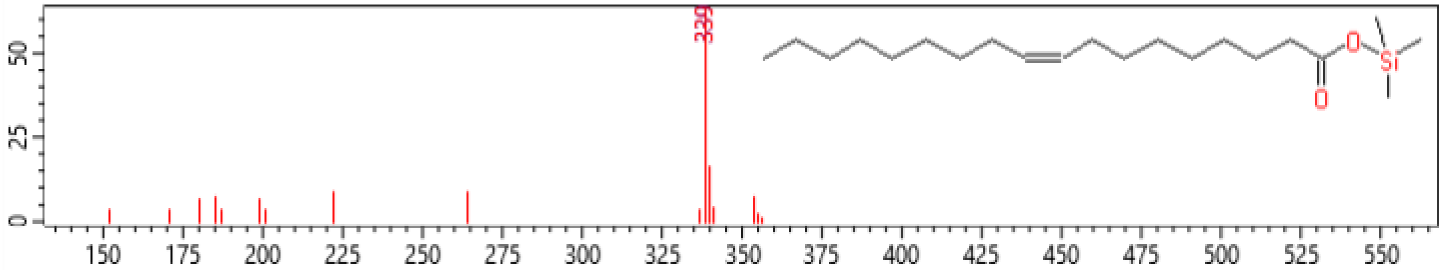


Oleic Acid, (Z)-, TMS derivative

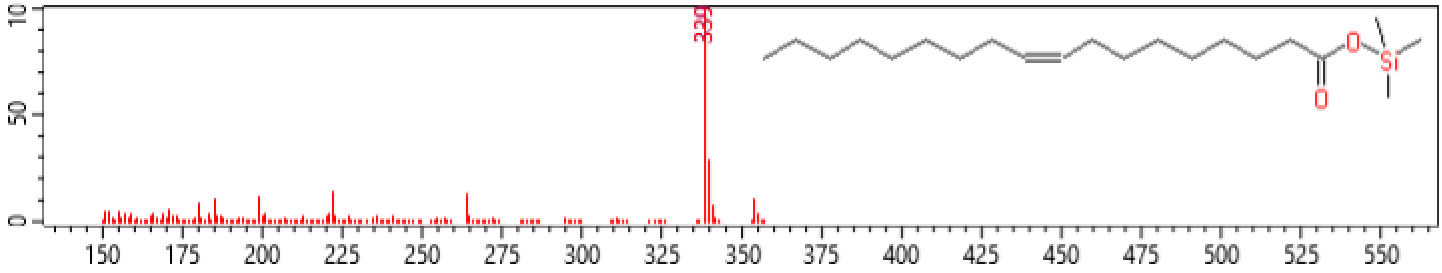

Pinoxaden

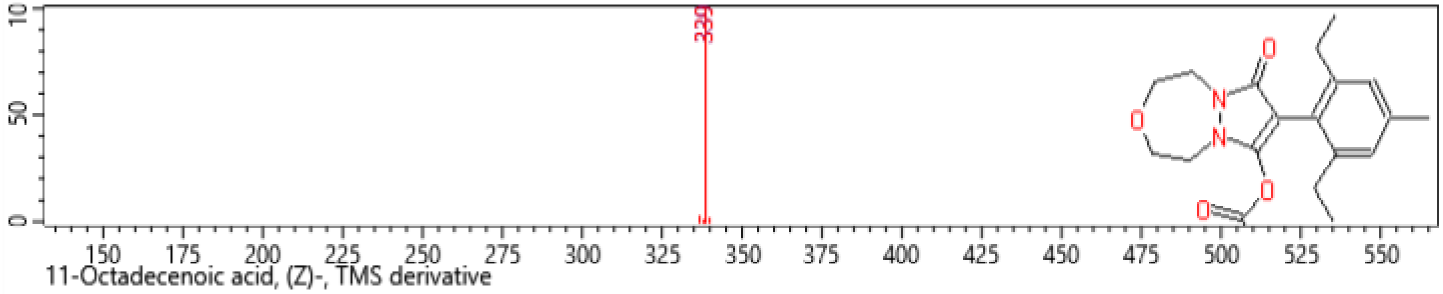


11-Octadecenoic acid, (Z)-, TMS derivative

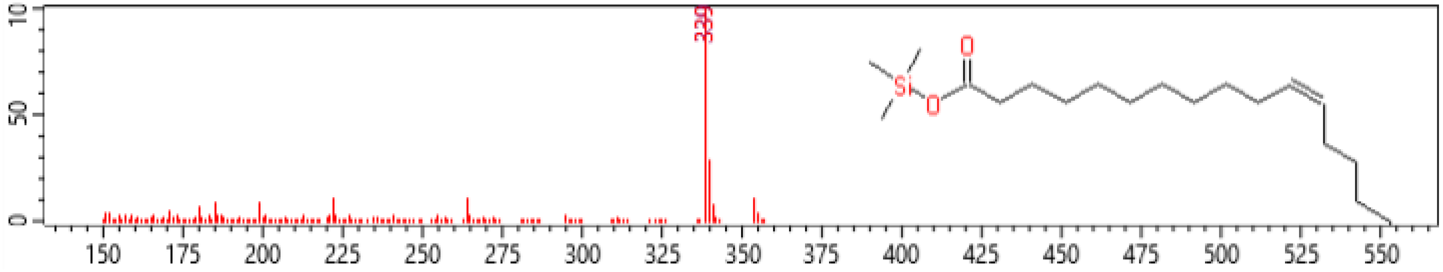


**13-Octadecenoic acid, (Z)-, TMS derivative**

Peak: 17.14 - 17.16 min (scan #2830 - #2835) Subtracted noise: 17.12 - 17.13 min (scan #2823 - #2827), 17.18 - 17.20 min (scan #28

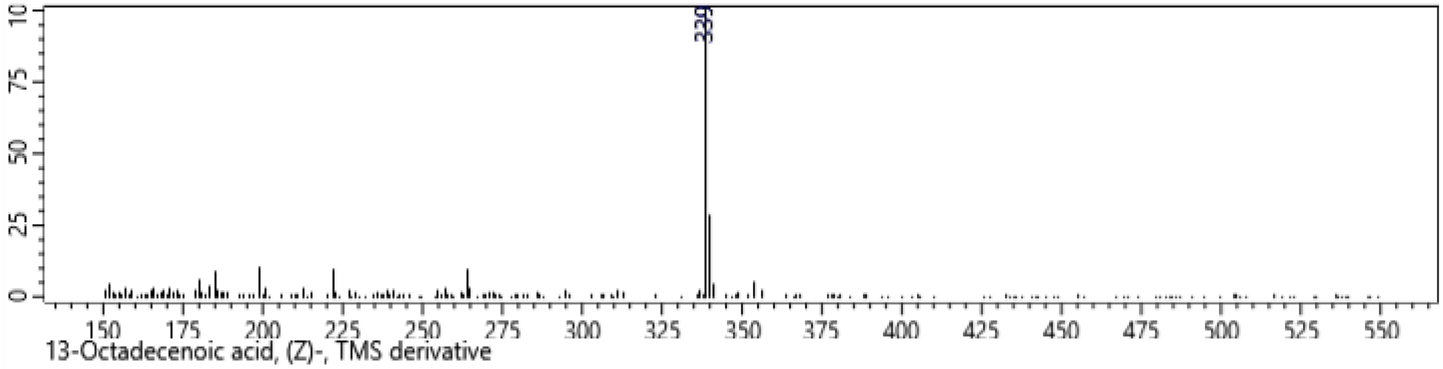


13-Octadecenoic acid, (Z)-, TMS derivative

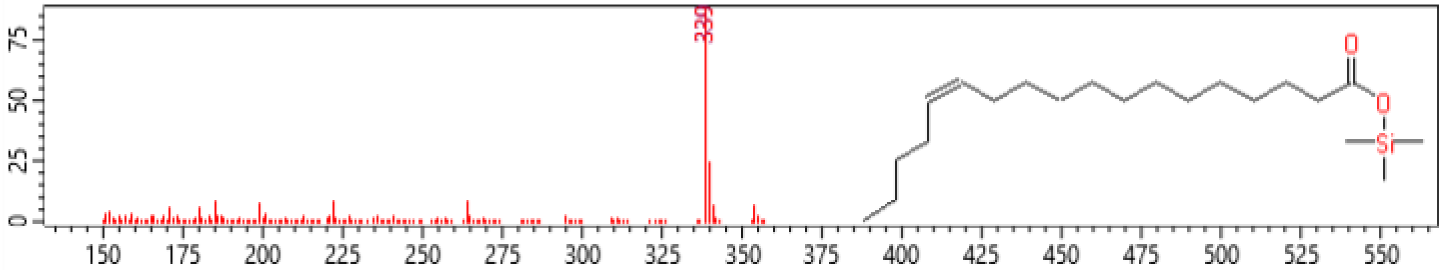


11-Octadecenoic acid, (Z)-, TMS derivative

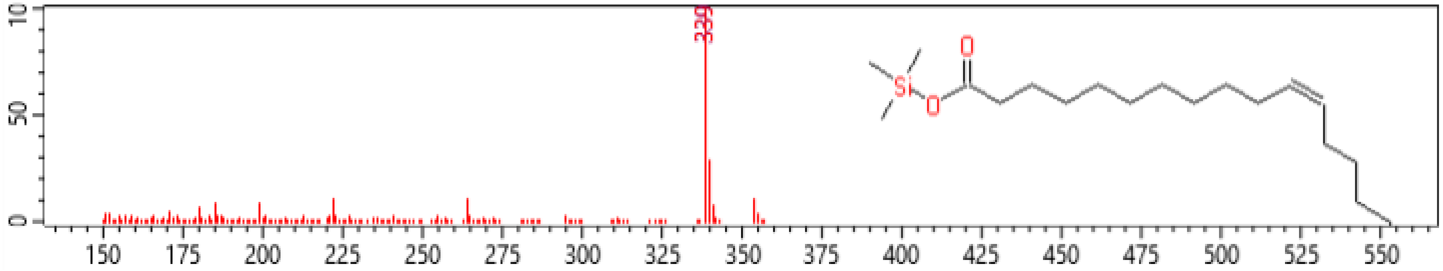

11-Octadecenoic acid, (E)-, TMS derivative

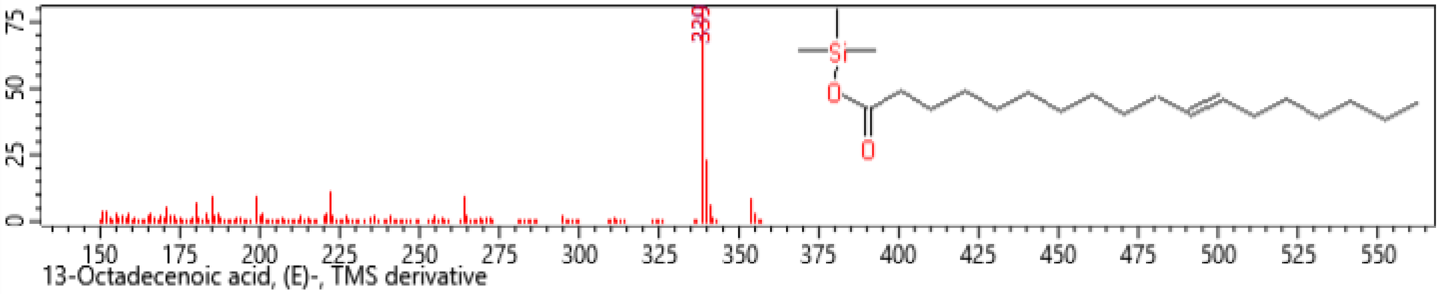


13-Octadecenoic acid, (E)-, TMS derivative

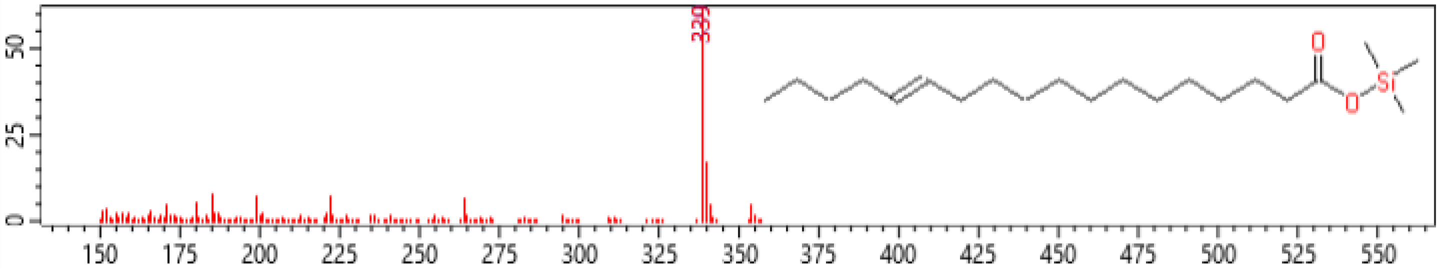


Oleic Acid, (Z)-, TMS derivative

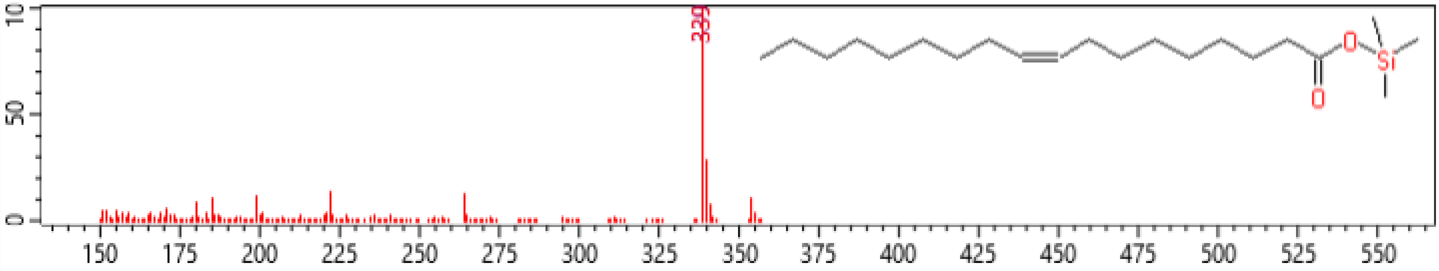


**Cyclononasiloxane, octadecamethyl-**

Peak: 17.20 - 17.24 min (scan #2845 - #2857) Subtracted noise: 17.13 - 17.17 min (scan #2828 - #2839), 17.27 - 17.32 min (scan #28

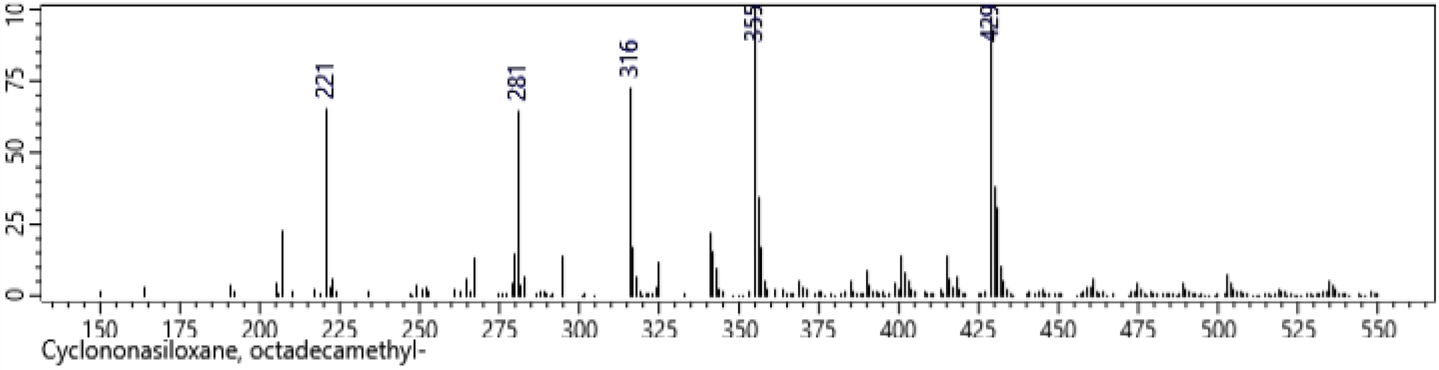


Cyclononasiloxane, octadecamethyl-

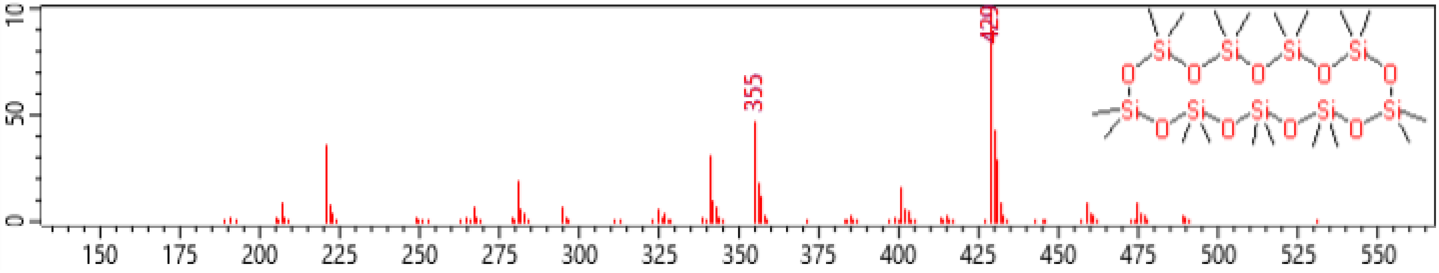

Cyclononasiloxane, octadecamethyl-

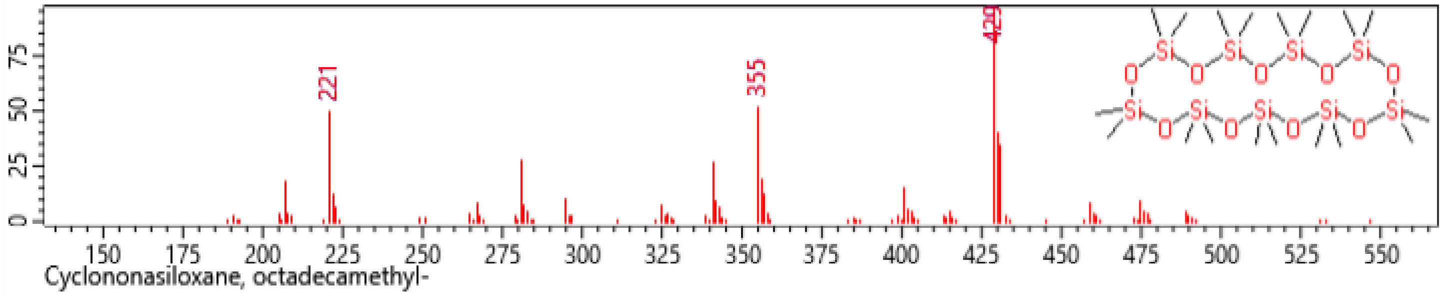


Cyclononasiloxane, octadecamethyl-

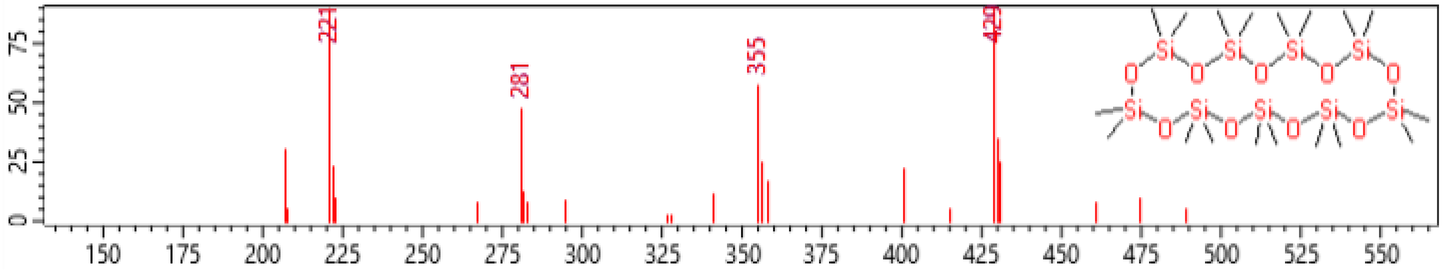


Cyclodecasiloxane, eicosamethyl-

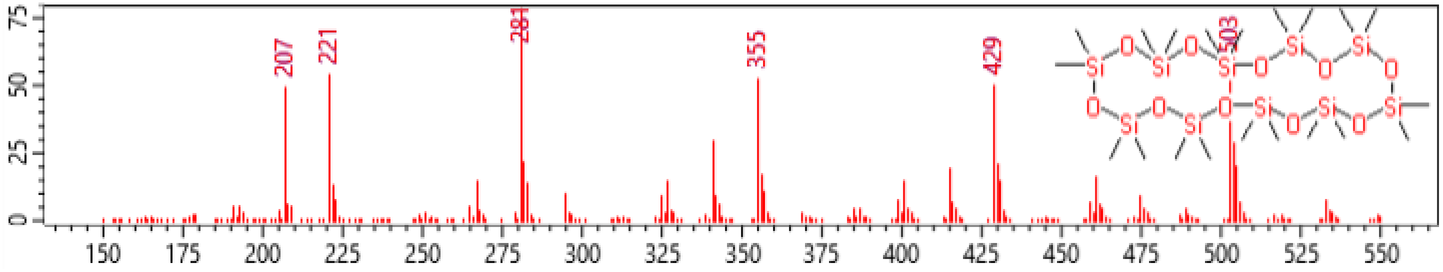


Tetracosamethyl-cyclododecasiloxane

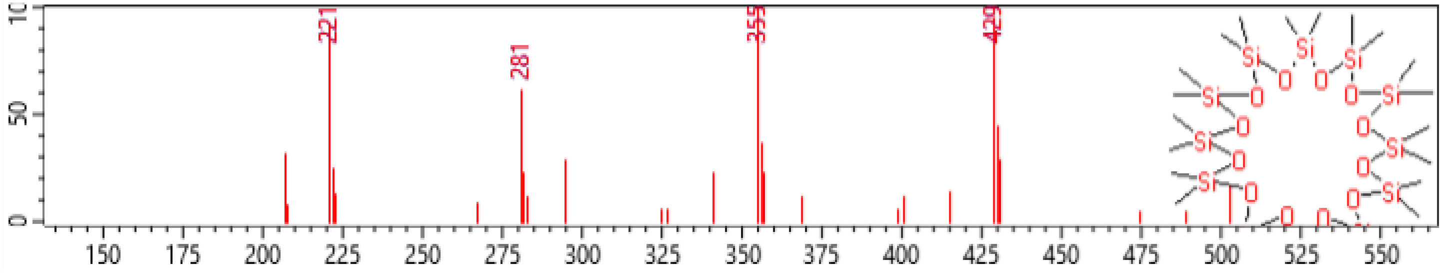


**4-Chloro-2-trichloromethyl-6-trifluoromethylquinoline**

Peak: 17.31 - 17.34 min (scan #2876 - #2886) Subtracted noise: 17.23 - 17.27 min (scan #2856 - #2866), 17.35 - 17.39 min (scan #28

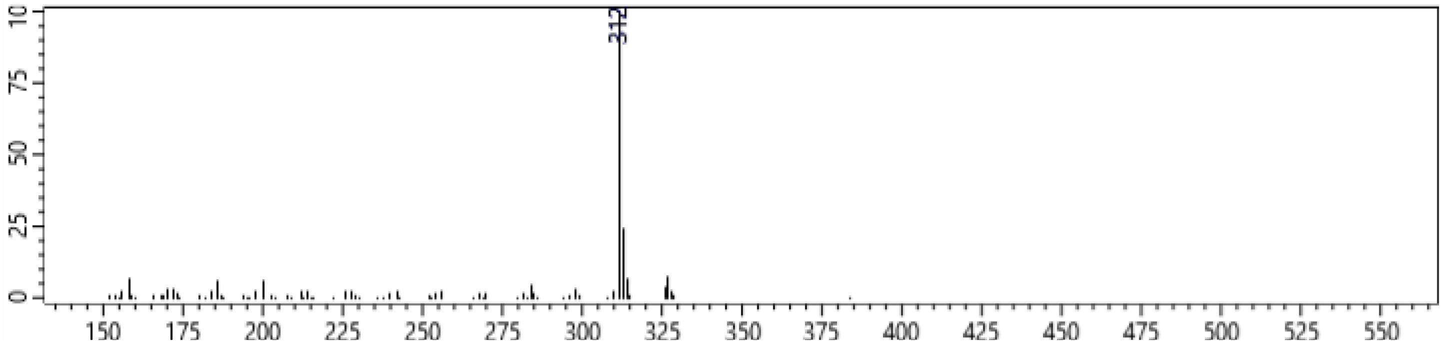

4-Chloro-2-trichloromethyl-6-trifluoromethylquinoline

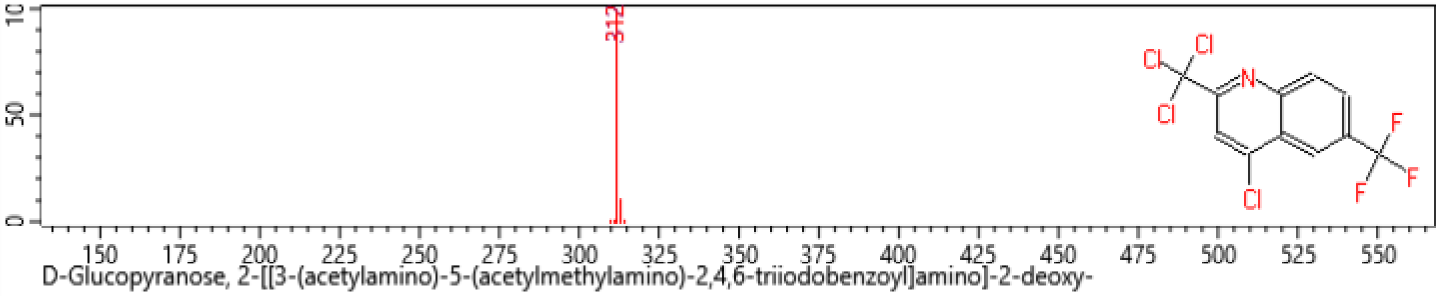


D-Glucopyranose, 2-[[3-(acetylamino)-5-(acetylmethylamino)-2,4,6-triiodobenzoyl]amino]-2-deoxy-

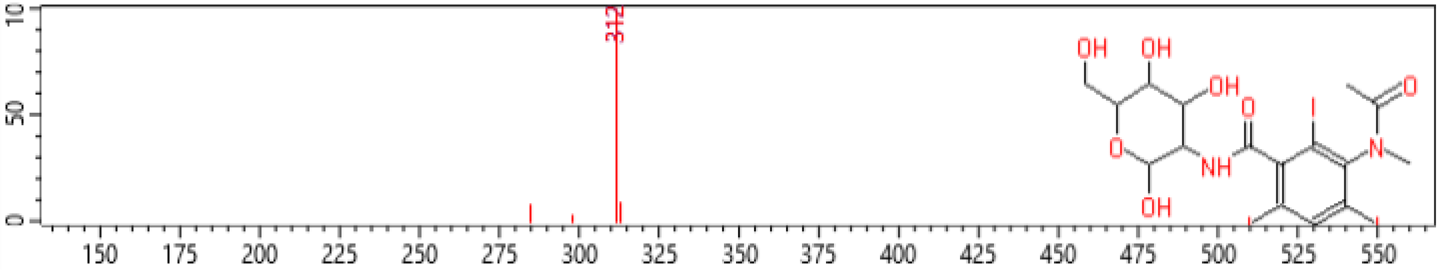


Palmitoylserinol

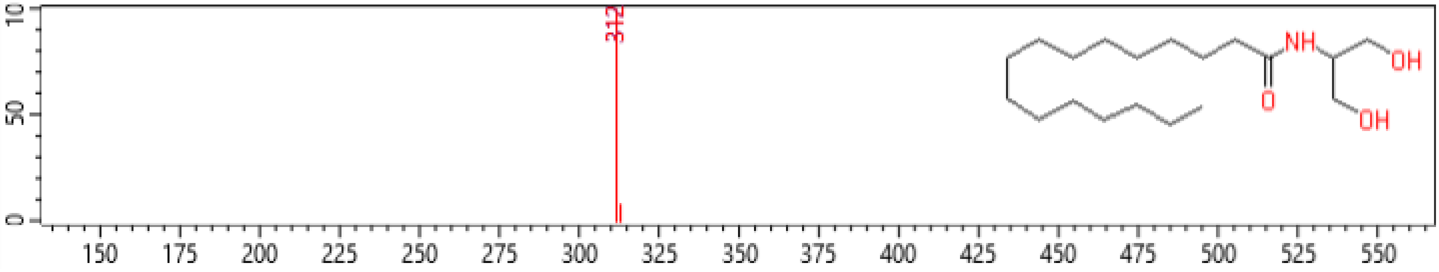


1-[(4,8-Dimethoxyfuro[2,3-b]quinolin-7-yl)oxy]-3-methylbutane-2,3-diol

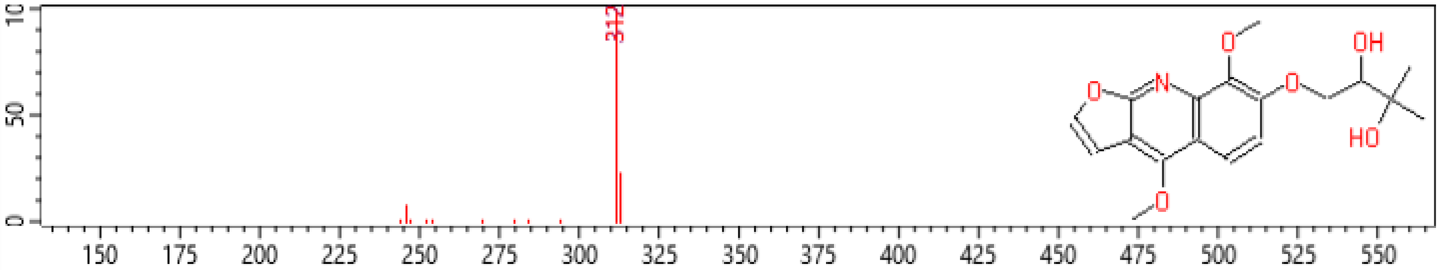


3-Pyridinecarbonitrile, 2-amino-5-butyl-4-(2-furanyl)-6-pentyl-

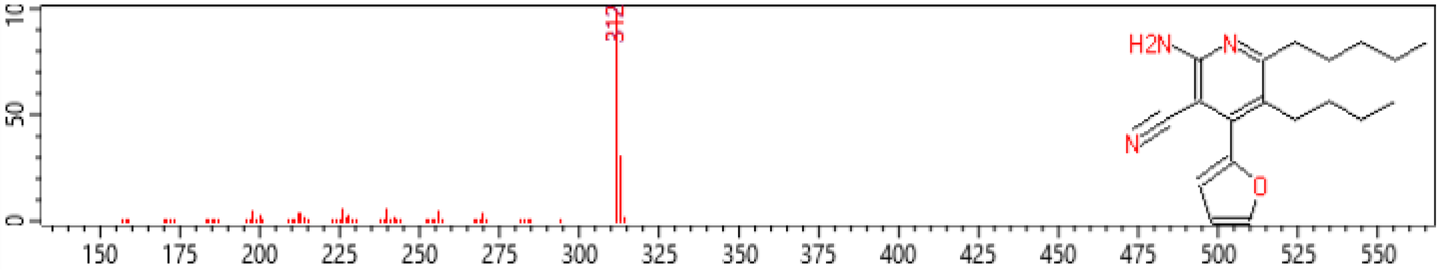

**Stearic acid, TMS derivative**

Peak: 17.33 - 17.40 min (scan #2882 - #2902) Subtracted noise: 17.28 - 17.35 min (scan #2868 - #2888), 17.49 - 17.56 min (scan #29

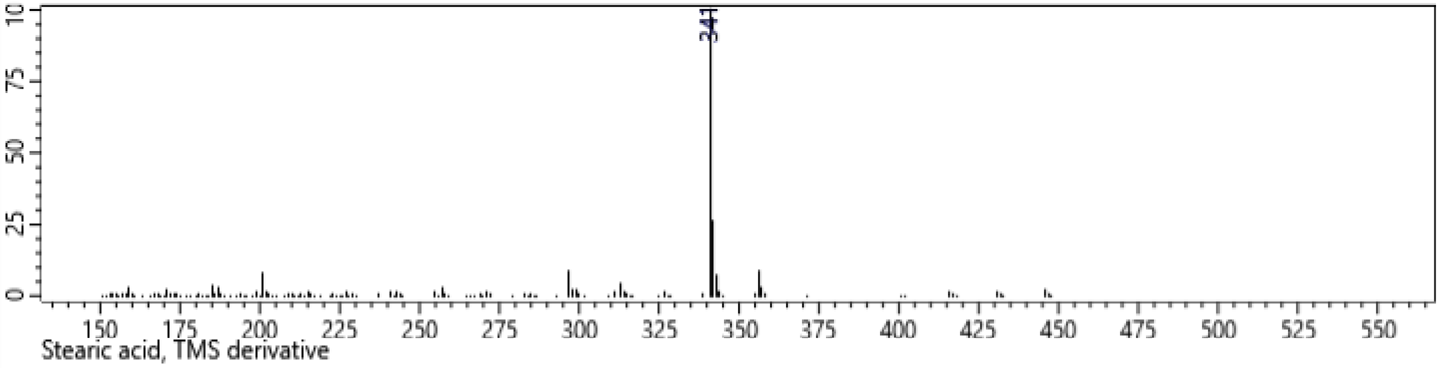


Stearic acid, TMS derivative

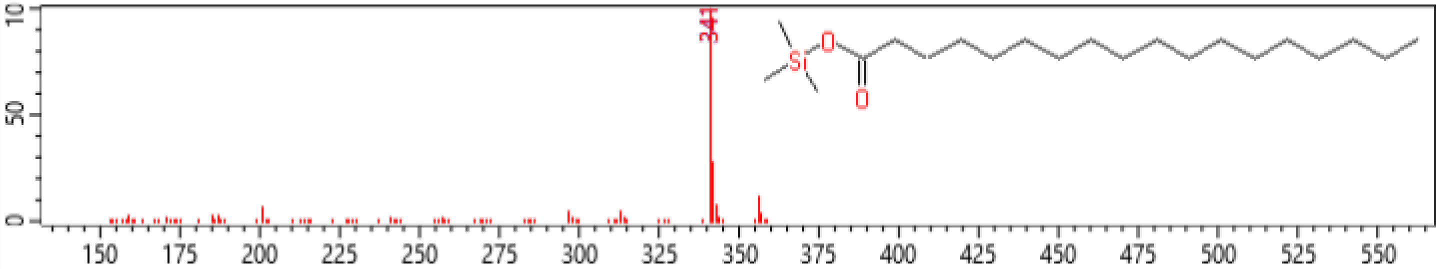


Stearic acid, TMS derivative

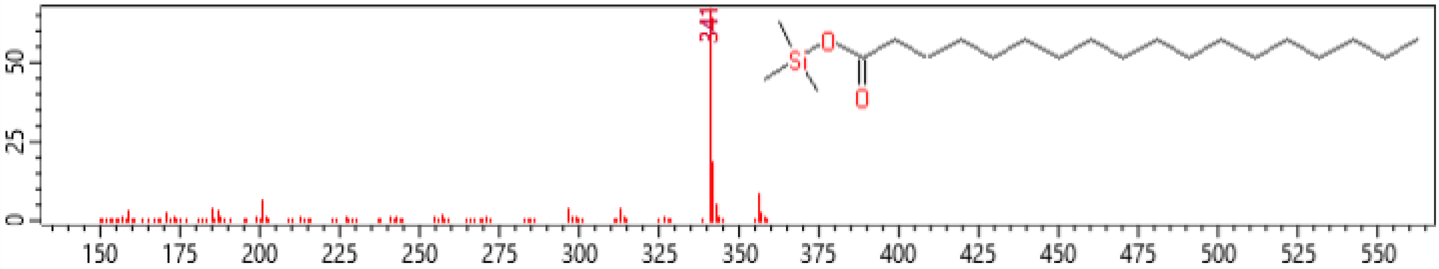


Stearic acid, TMS derivative

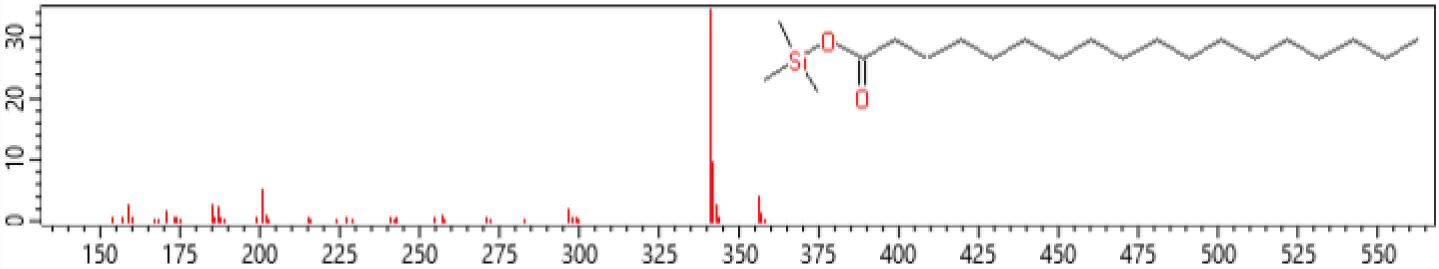


Stearic acid, TMS derivative

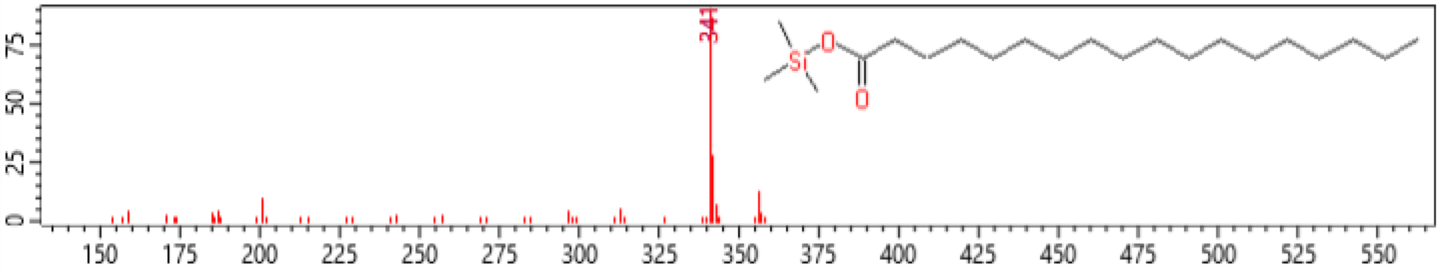

Spironolactone

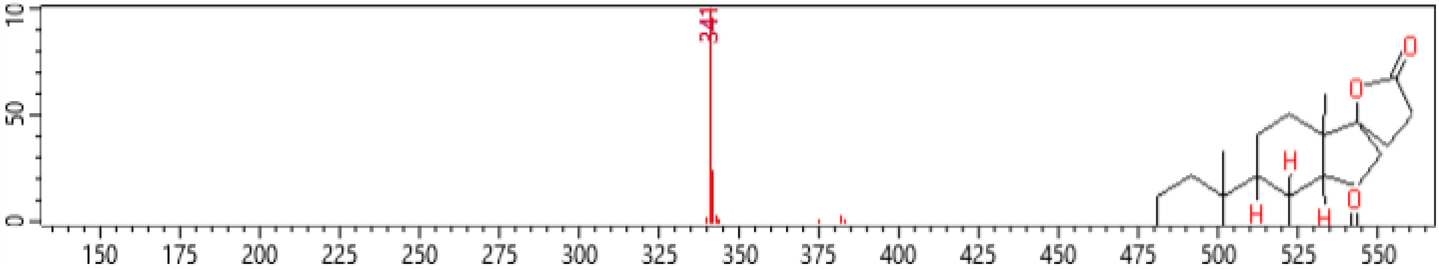


**Methanone, [1-(5-fluoropentyl)-2-hydroxy-1H-indol-3-yl]-1-naphthalenyl-**

Peak: 17.56 - 17.59 min (scan #2946 - #2954) Subtracted noise: 17.50 - 17.54 min (scan #2931 - #2940), 17.61 - 17.64 min (scan #29

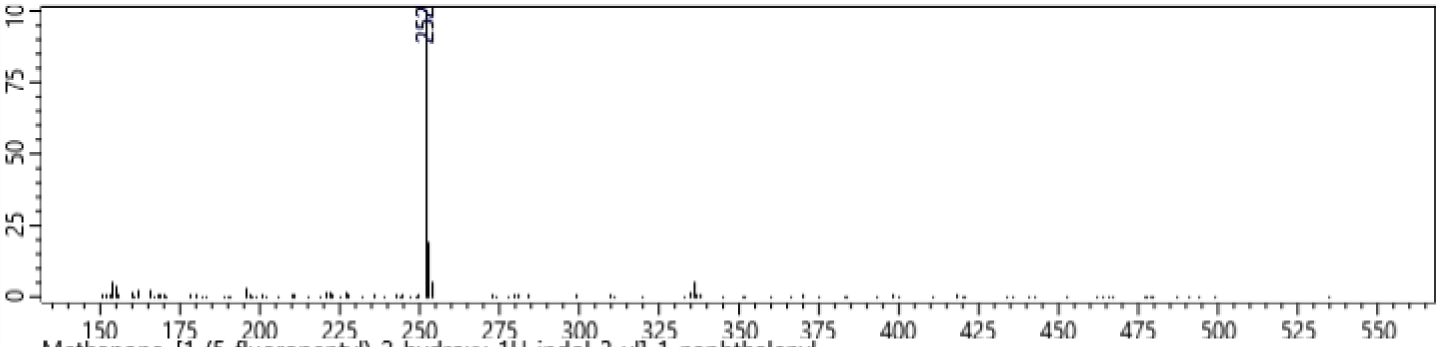


Methanone, [1-(5-fluoropentyl)-2-hydroxy-1H-indol-3-yl]-1-naphthalenyl-

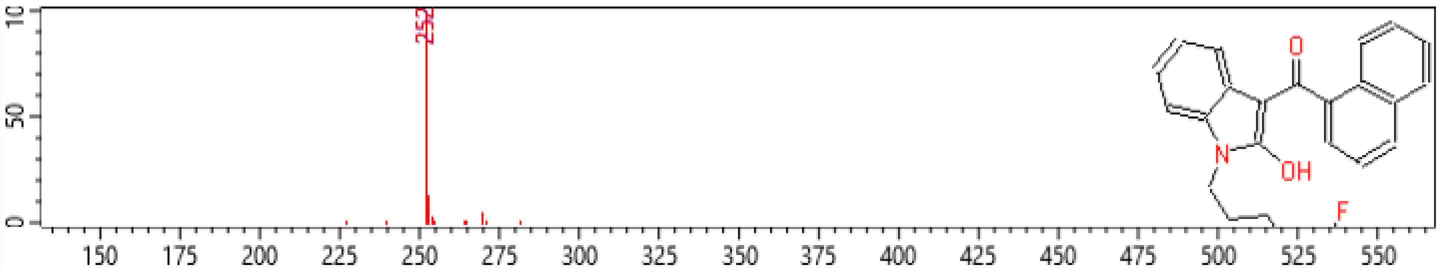


7-Aminonitrazepam

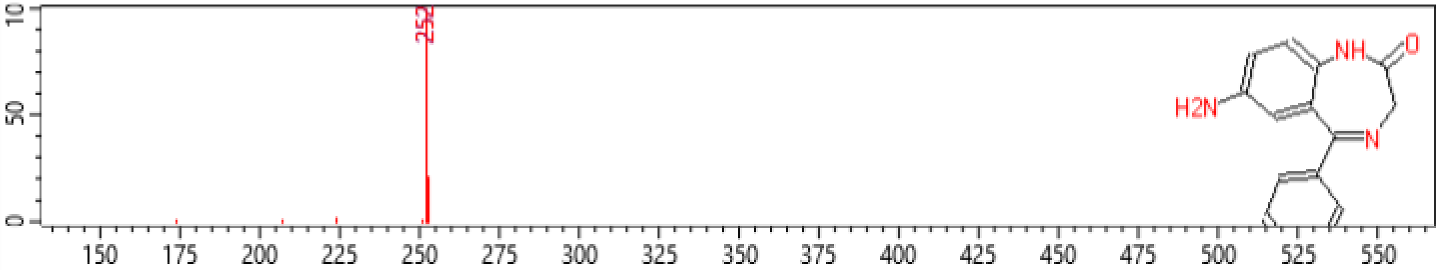


2-Ethyl-5-tridecylpyrrolidine

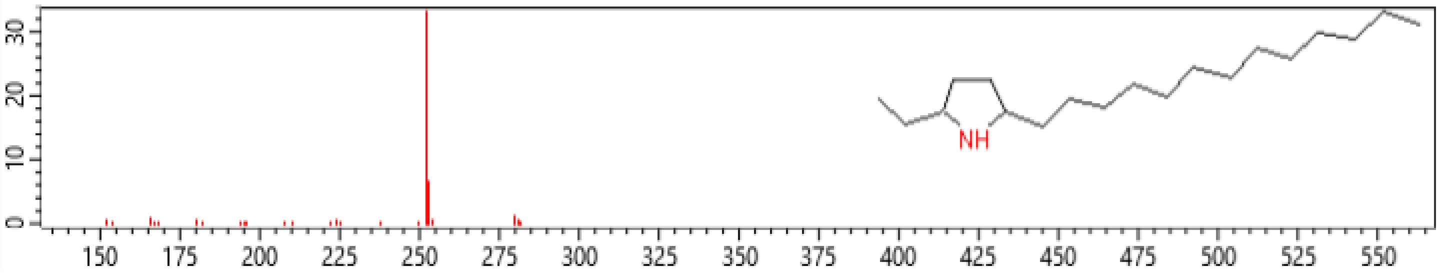

Benzo(a)pyrene

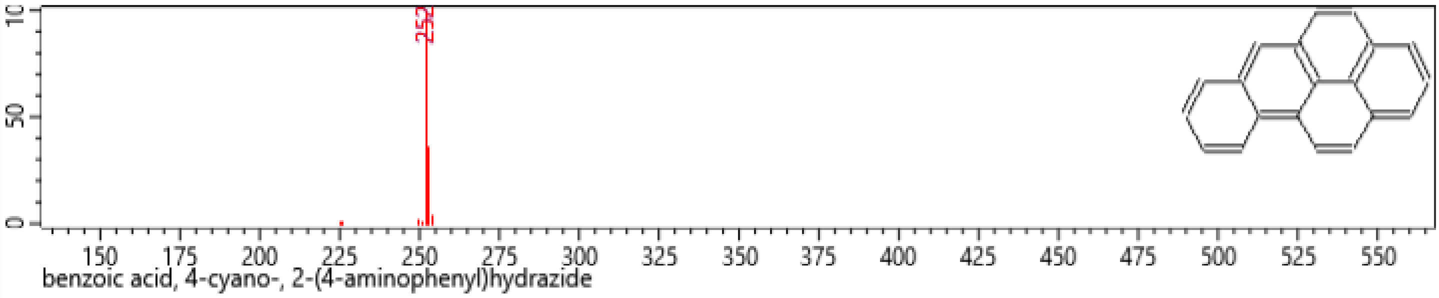


benzoic acid, 4-cyano-, 2-(4-aminophenyl)hydrazide

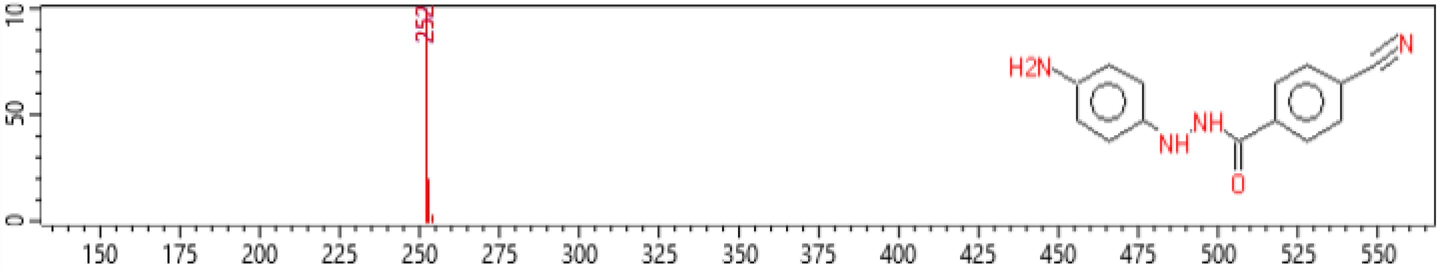


**3-Pyridinecarboxamide, N-(2,3-dihydro-7,8-dimethoxyimidazo[1,2-c]quinazolin-5-yl)-**

Peak: 17.63 - 17.66 min (scan #2965 - #2976) Subtracted noise: 17.56 - 17.60 min (scan #2947 - #2958), 17.69 - 17.73 min (scan #29

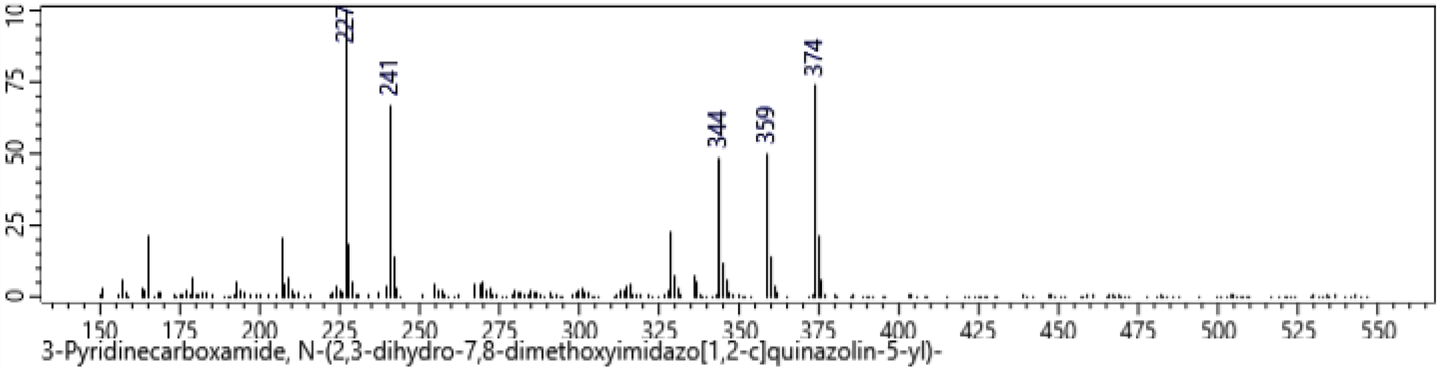


3-Pyridinecarboxamide, N-(2,3-dihydro-7,8-dimethoxyimidazo[1,2-c]quinazolin-5-yl)-

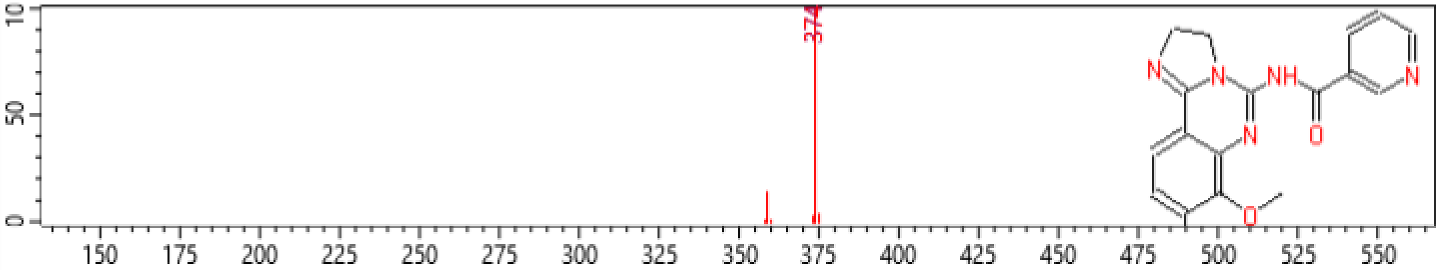


4H-1-Benzopyran-4-one, 2-(3,4-dimethoxyphenyl)-5,6-dihydroxy-7,8-dimethoxy-

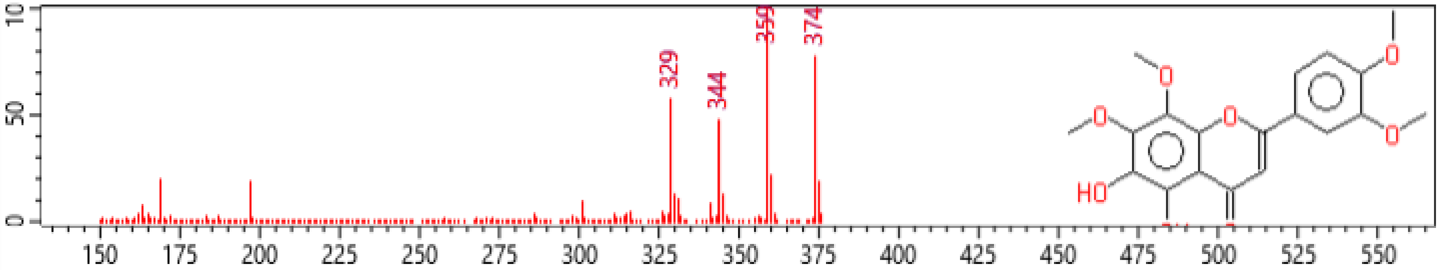

2,3,8,9,10-Pentamethoxy-6a,11a-dihydro-6H-benzofuro[3,2-c]chromene

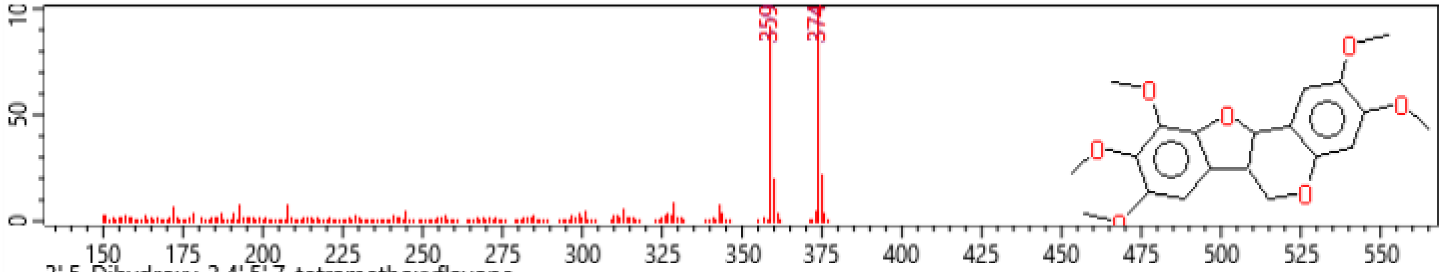


3',5-Dihydroxy-3,4',5',7-tetramethoxyflavone

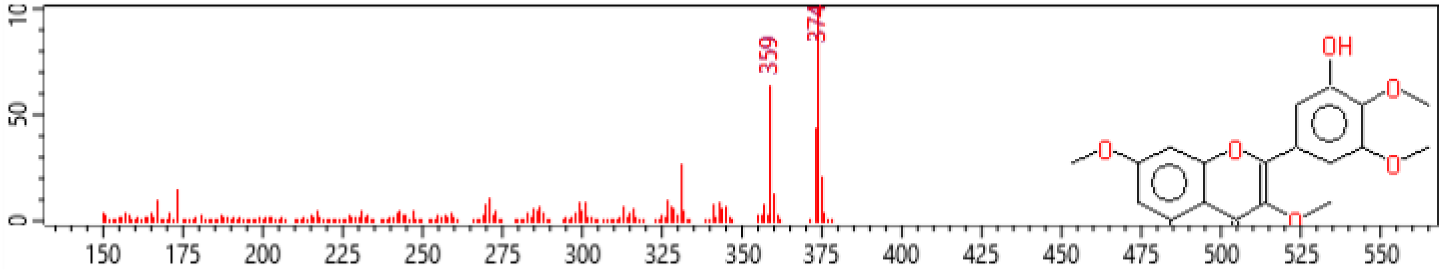


10a-[(E)-2-[4-(Dimethylamino)phenyl]ethenyl]-10,10-dimethyl-3,4,10,10a-tetrahydropyrimido[1,2-a]indol-2(1H)-one, N-methyl

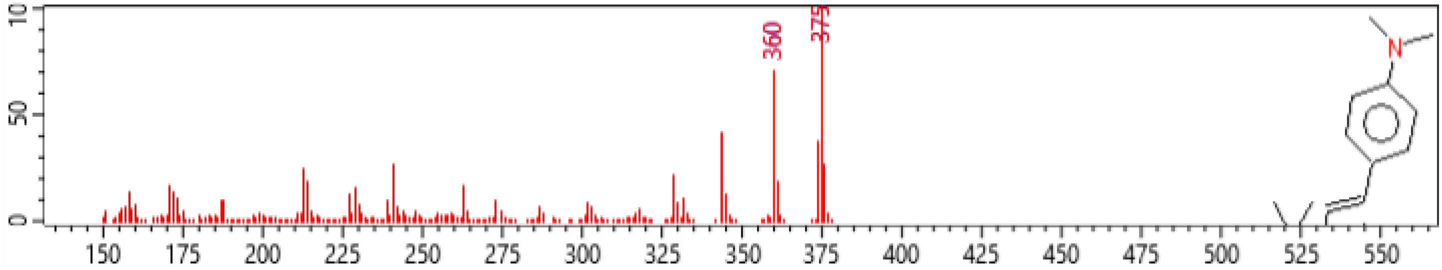


**Formononetin**

Peak: 17.73 - 17.76 min (scan #2993 - #3004) Subtracted noise: 17.65 - 17.69 min (scan #2972 - #2982), 17.78 - 17.82 min (scan #30

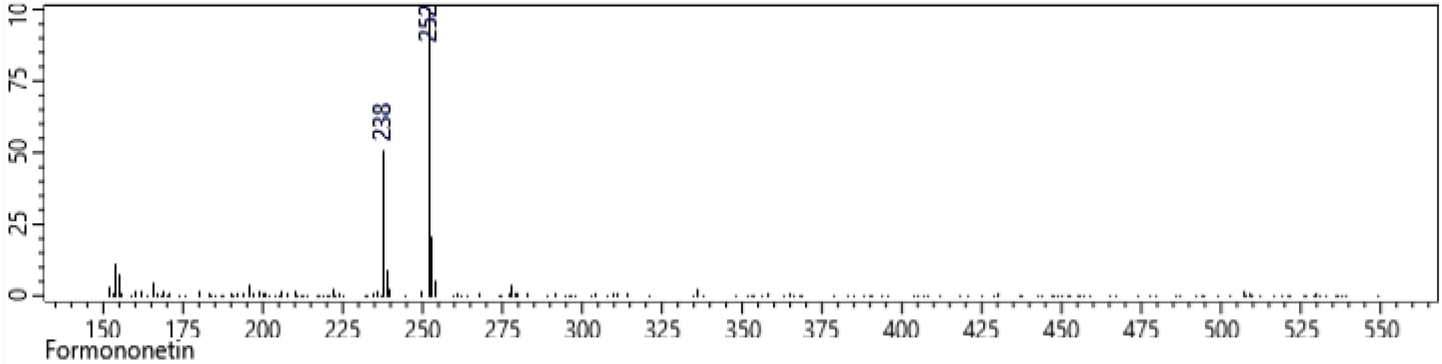


Formononetin

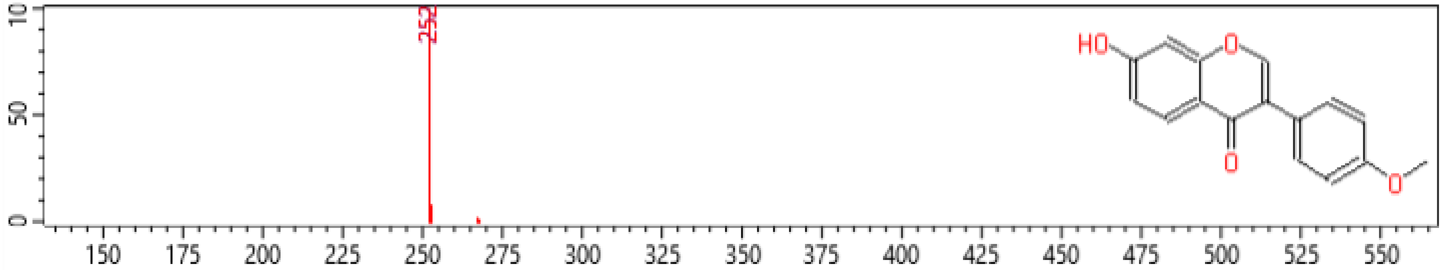

7-Hydroxy-4'-methoxyflavone

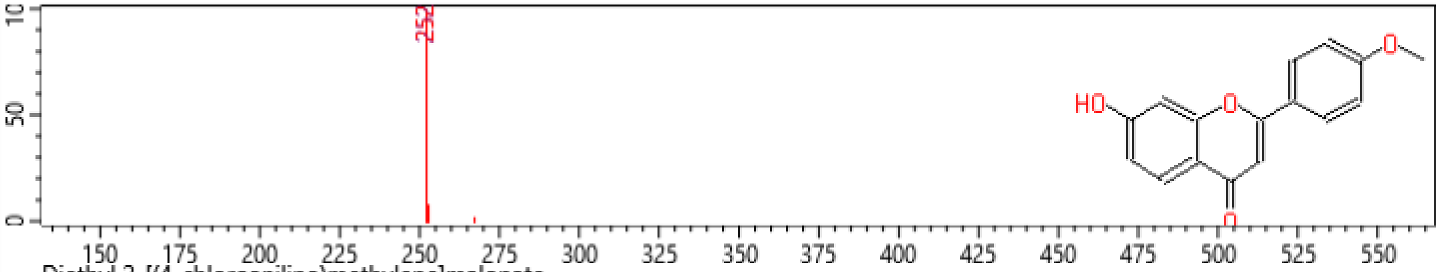


Diethyl 2-[(4-chloroanilino)methylene]malonate

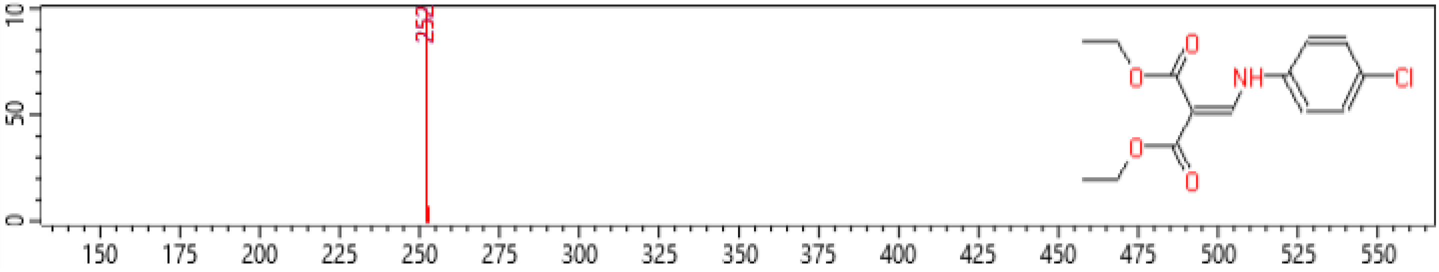


Methanone, [1-(5-fluoropentyl)-2-hydroxy-1H-indol-3-yl]-1-naphthalenyl-

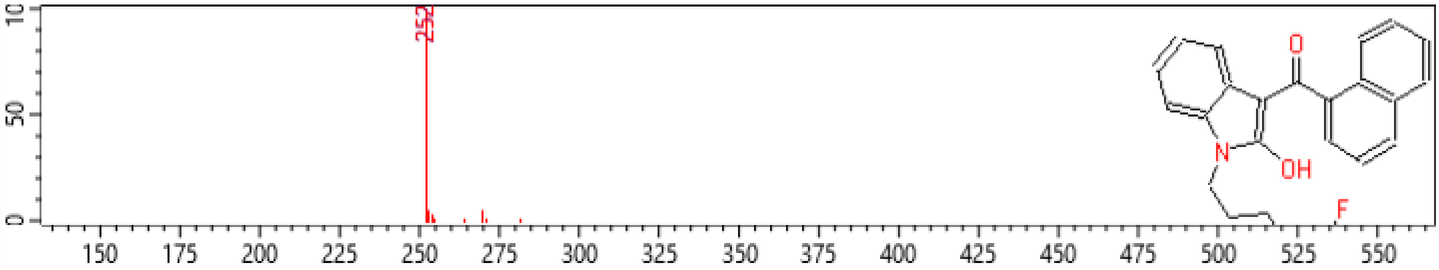


Thiophen-2-methylamine, N,N-dinonyl-

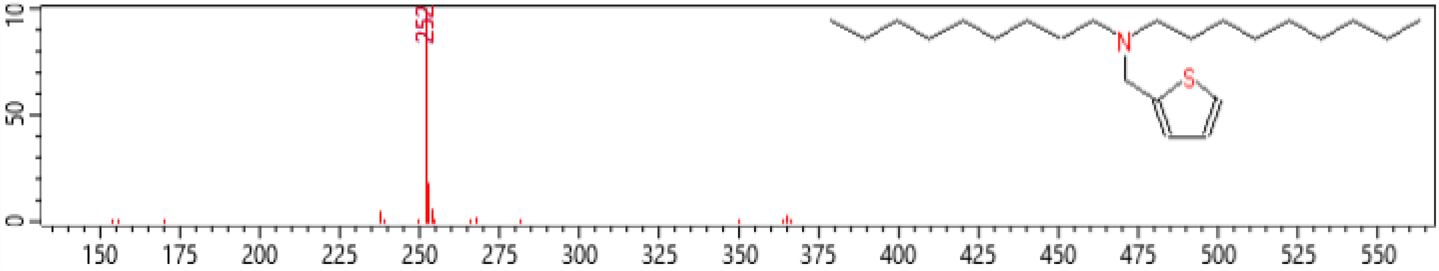


**trimethylsilyl 9-(3-methyl-5-pentylfuran-2-yl)nonanoate**

Peak: 18.23 - 18.25 min (scan #3134 - #3140) Subtracted noise: 18.19 - 18.21 min (scan #3123 - #3128), 18.26 - 18.29 min (scan #31[illegible]

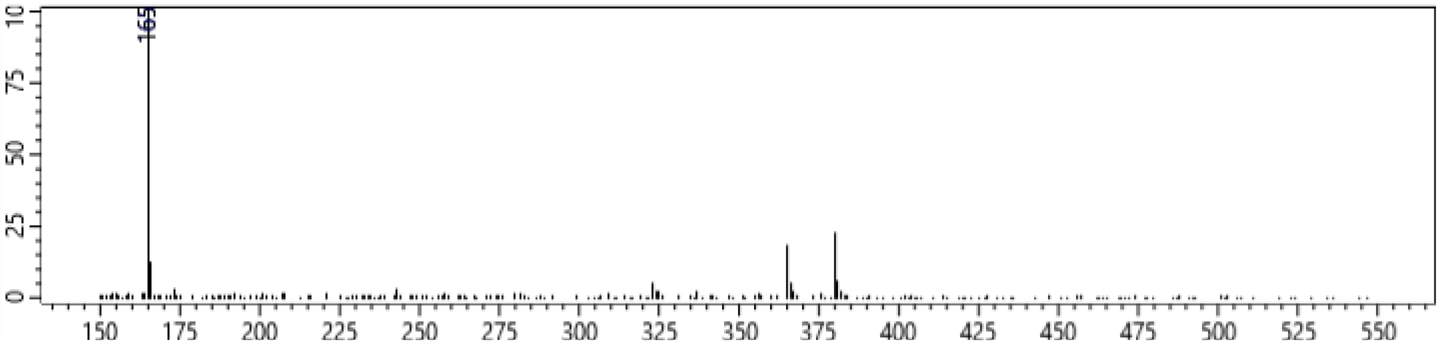

trimethylsilyl 9-(3-methyl-5-pentylfuran-2-yl)nonanoate

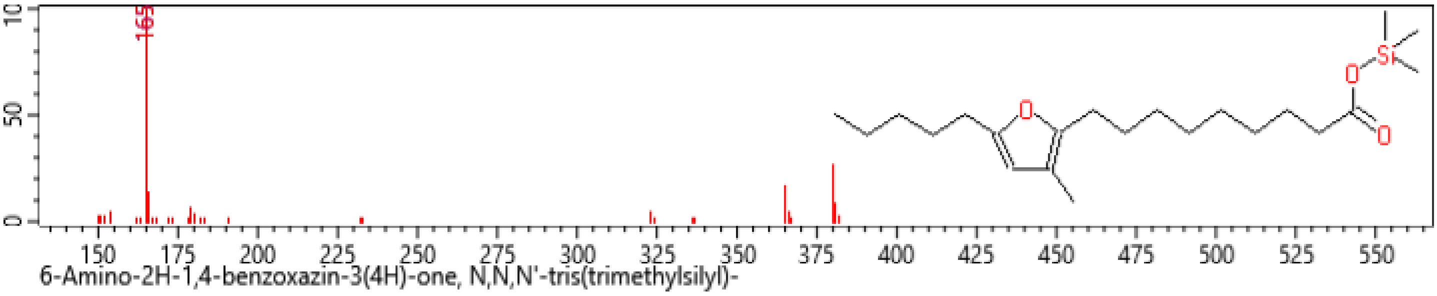


6-Amino-2H-1,4-benzoxazin-3(4H)-one, N,N,N'-tris(trimethylsilyl)-

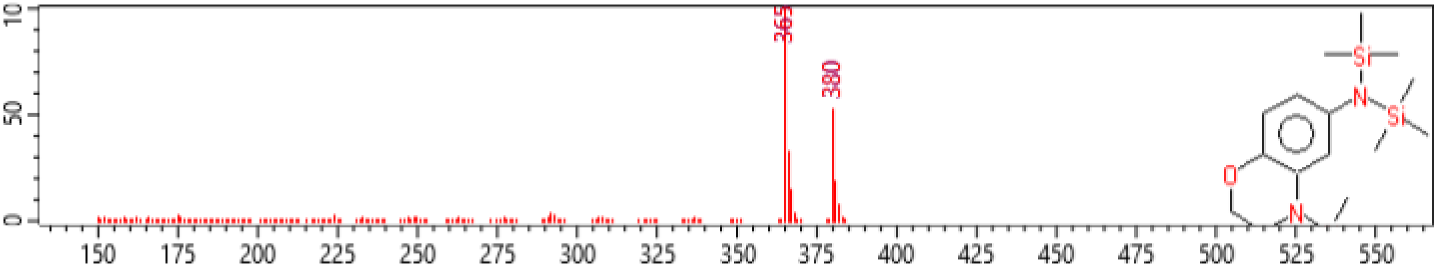


4-(3,4-Dimethoxyphenyl)-5-isopropyl-1-(2-methoxyphenethyl)-1H-imidazole

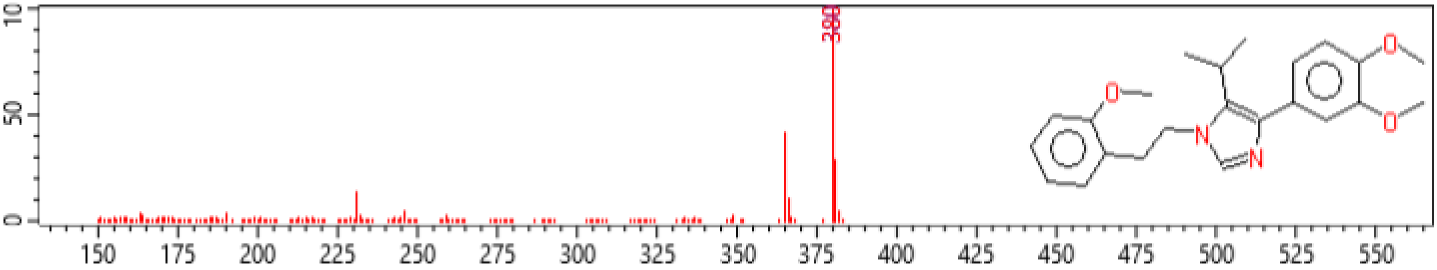


Tangeritin

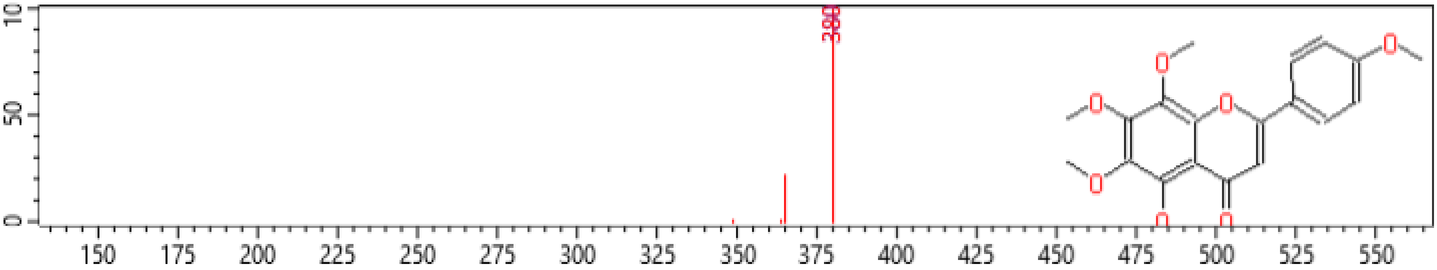


Quercetin 3,5,7,3',4'-pentamethyl ether

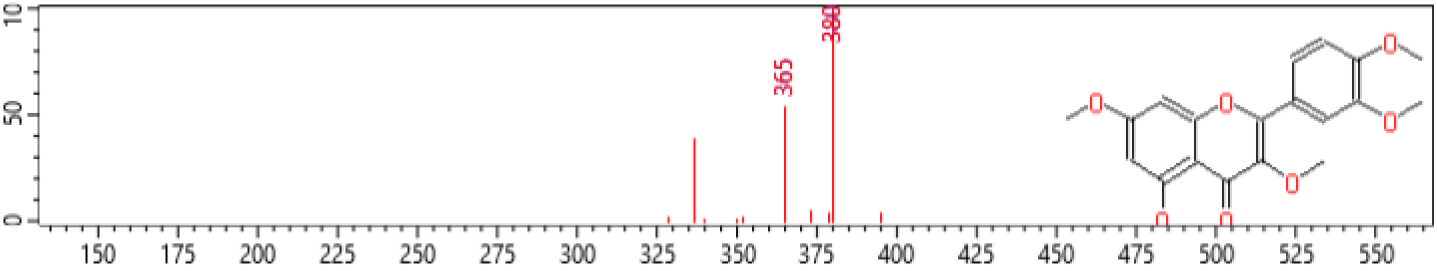

**Benzamide, N-(3-bromophenyl)-3-[(hexahydro-1H-azepin-1-yl)sulfonyl]-**

Peak: 18.40 - 18.43 min (scan #3182 - #3190) Subtracted noise: 18.35 - 18.38 min (scan #3166 - #3175), 18.44 - 18.47 min (scan #31

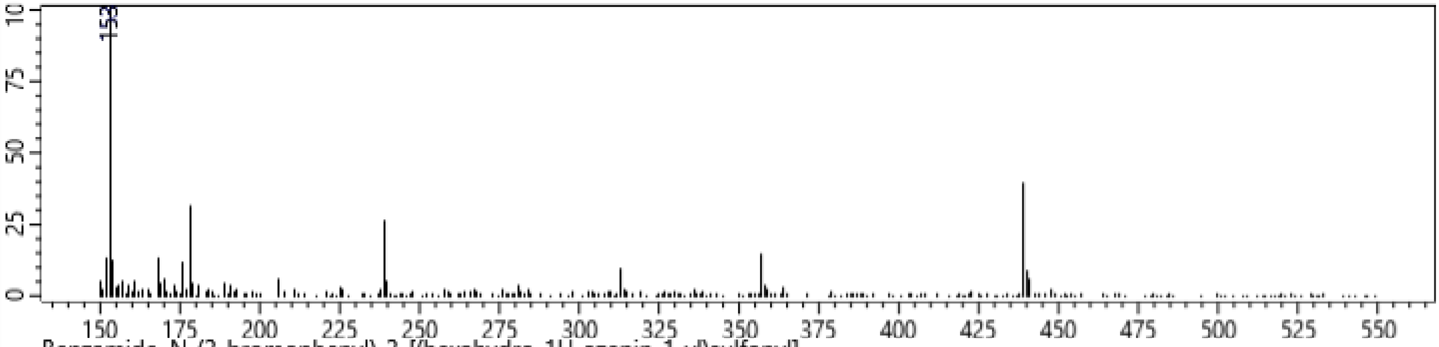


Benzamide, N-(3-bromophenyl)-3-[(hexahydro-1H-azepin-1-yl)sulfonyl]-

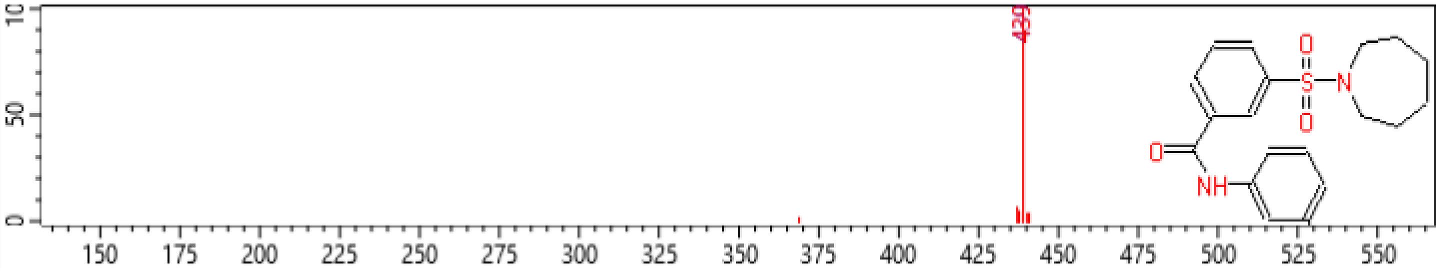


2'-Trifluoroacetyloxy-2,4,4',5,6'-pentamethoxychalcone

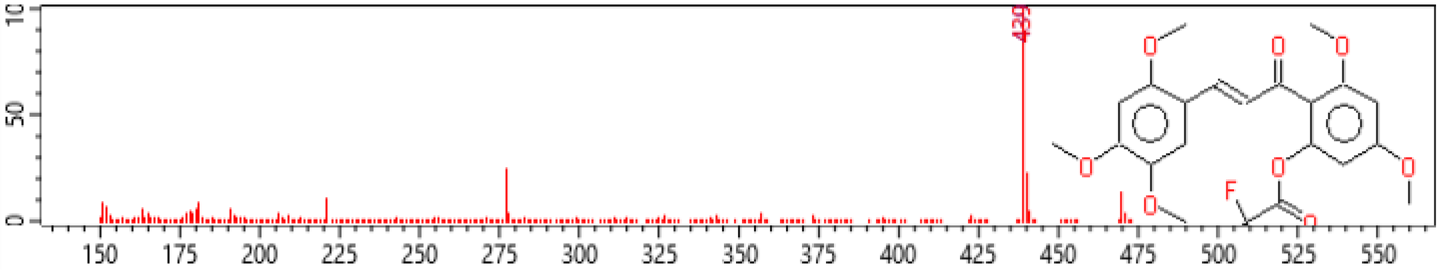


4,5,6,7-Tetrabromo-3H-2,1-benzoxathiol-3-one 1,1-dioxide

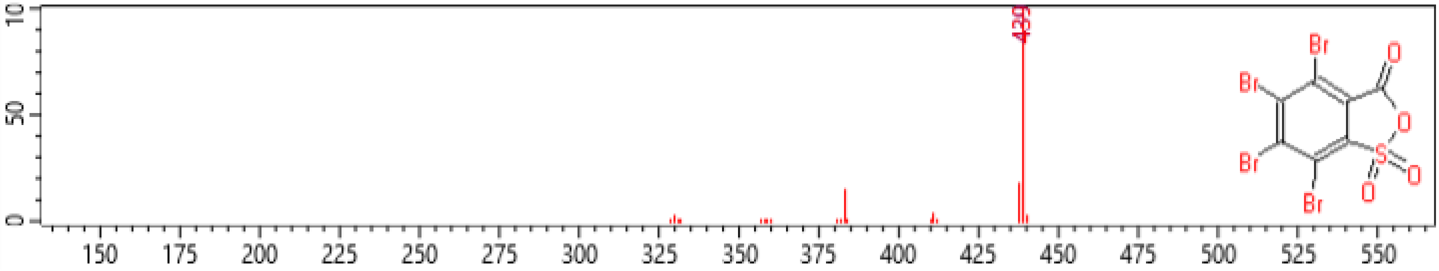


5-(4-Chlorophenyl)-6-ethylpyrimidine-2,4-diamine, N,N'-bis(trifluoroacetyl)-

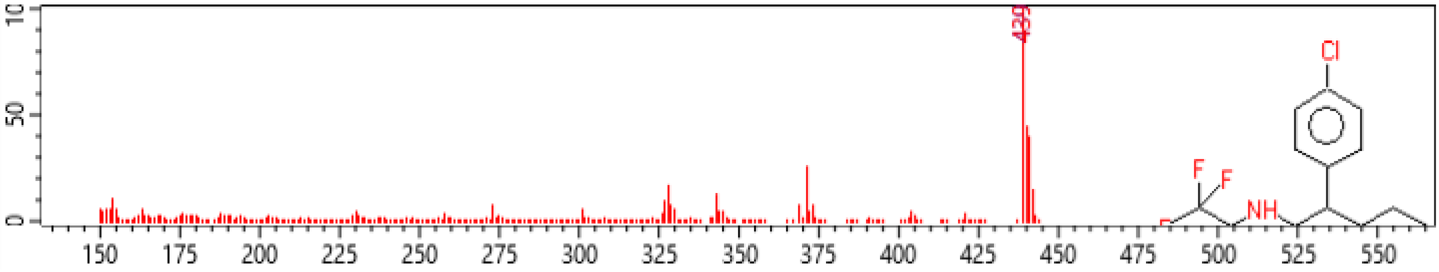

Androsta[17-16-b]furan-5'-imine, 4'-methylene-3-methoxy-N-cyclohexyl-

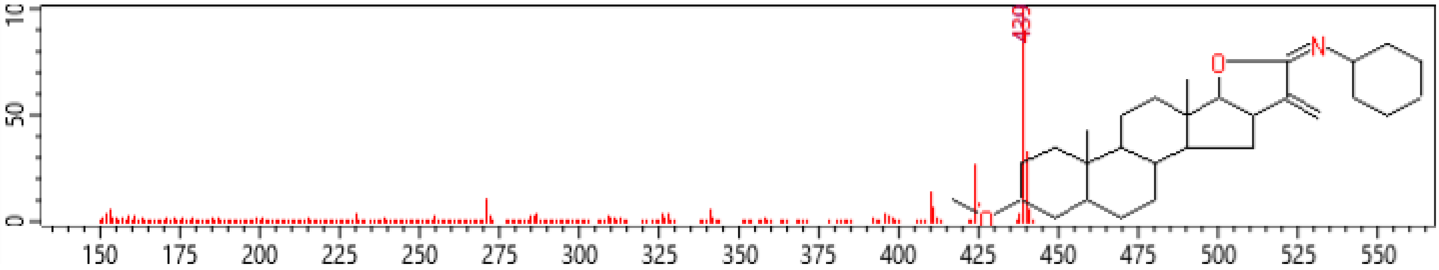


**4-Hydroxybutanamide, 3TMS**

Peak: 18.56 - 18.61 min (scan #3226 - #3240) Subtracted noise: 18.47 - 18.52 min (scan #3201 - #3215), 18.63 - 18.69 min (scan #32

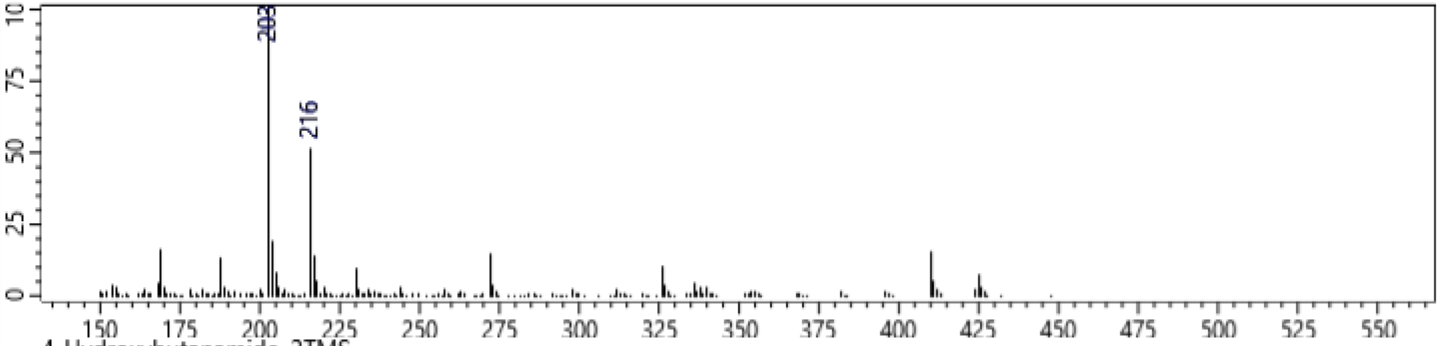


4-Hydroxybutanamide, 3TMS

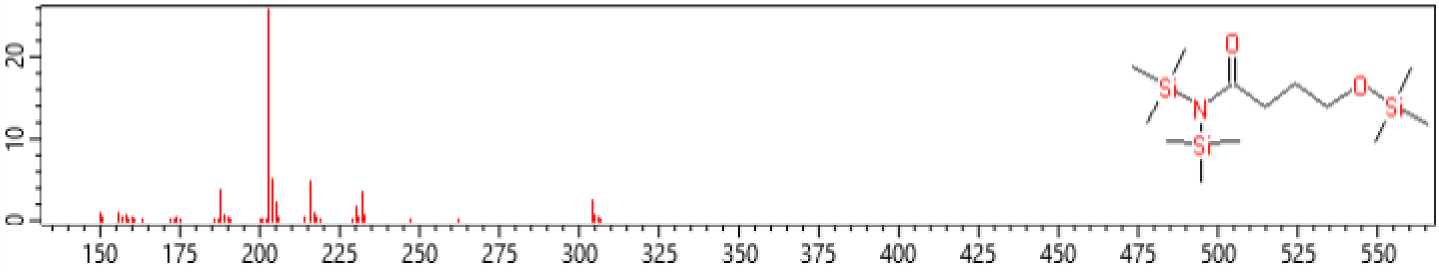

4-(3-Hexyl-4-methyl-2,5-dioxopyrrol-1-yl)butanamide, 2TMS

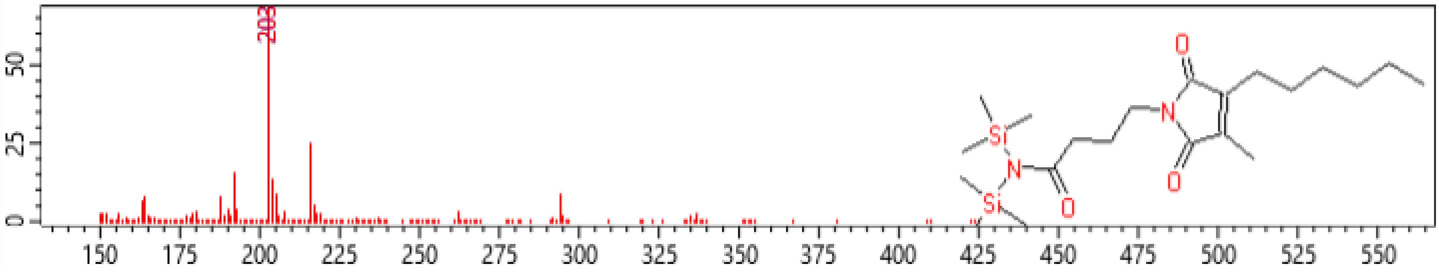


5-Amino-2-(3-hexyl-4-methyl-2,5-dioxopyrrol-1-yl)-5-oxopentanoic acid, 3TMS

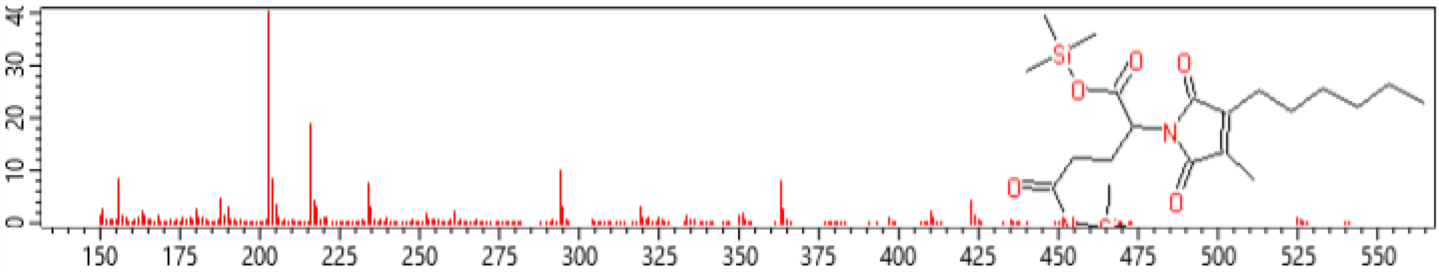

4,5,6,7-Tetrahydroindoxazen-4-one, 3-undecyl-6-[phenyl-dimethylsilyl]-

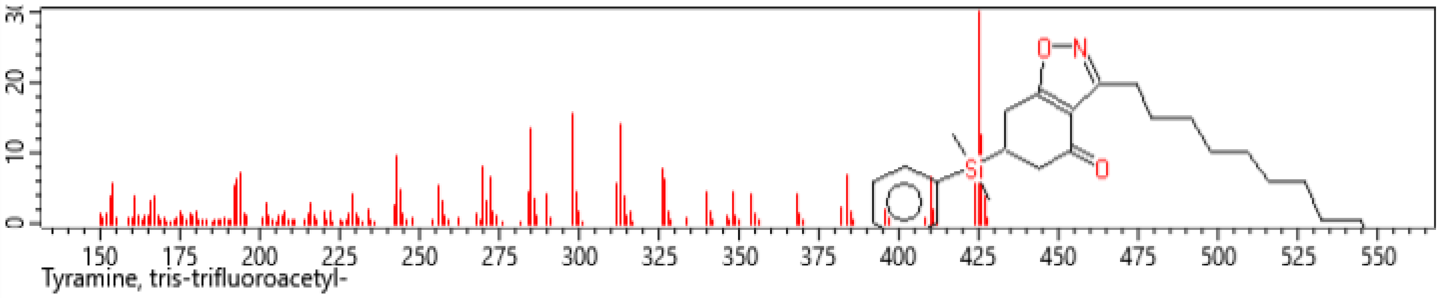

Tyramine, tris-trifluoroacetyl-

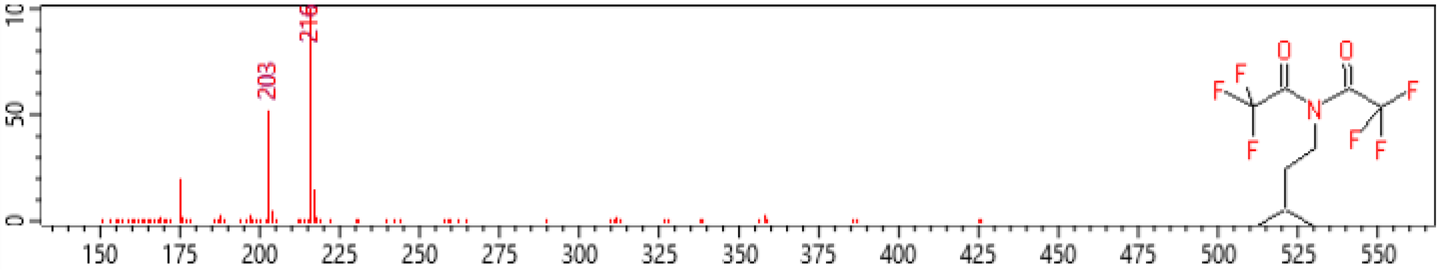


**Phthalic acid, cyclohexyl heptyl ester**

Peak: 18.66 - 18.70 min (scan #3255 - #3265) Subtracted noise: 18.60 - 18.64 min (scan #3237 - #3247), 18.72 - 18.75 min (scan #32

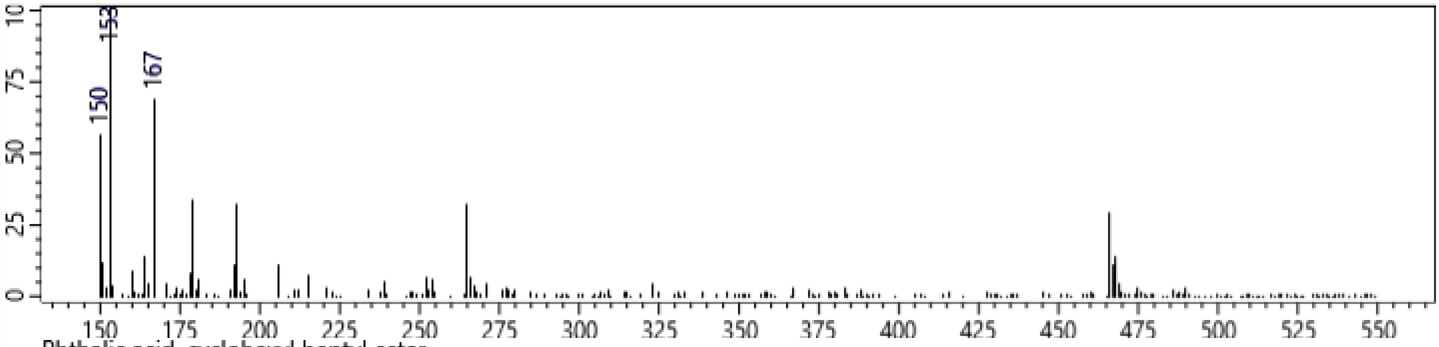


Phthalic acid, cyclohexyl heptyl ester

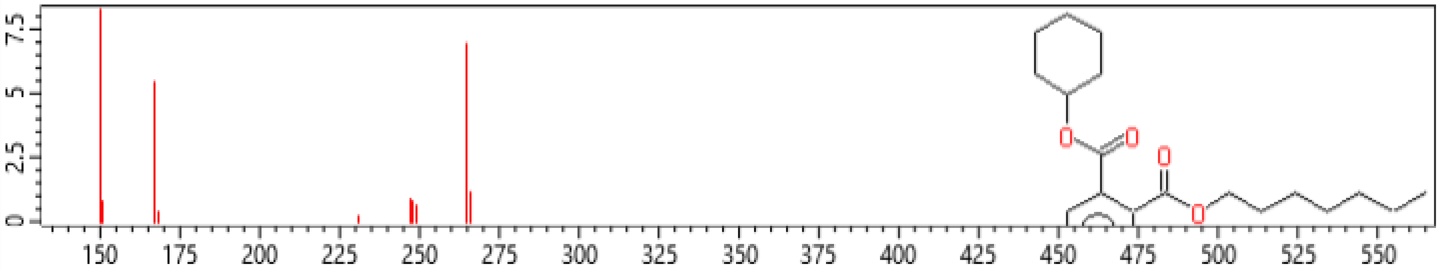

Butylphosphonic acid, neopentyl nonyl ester

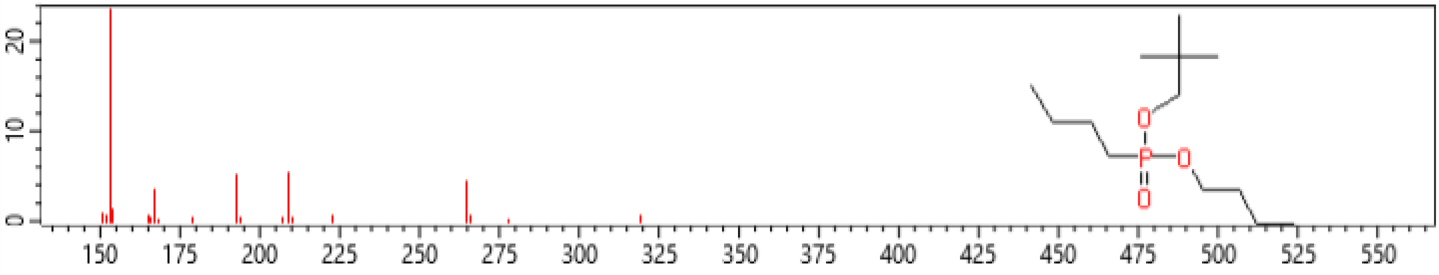

Phthalic acid, cycloheptyl heptyl ester

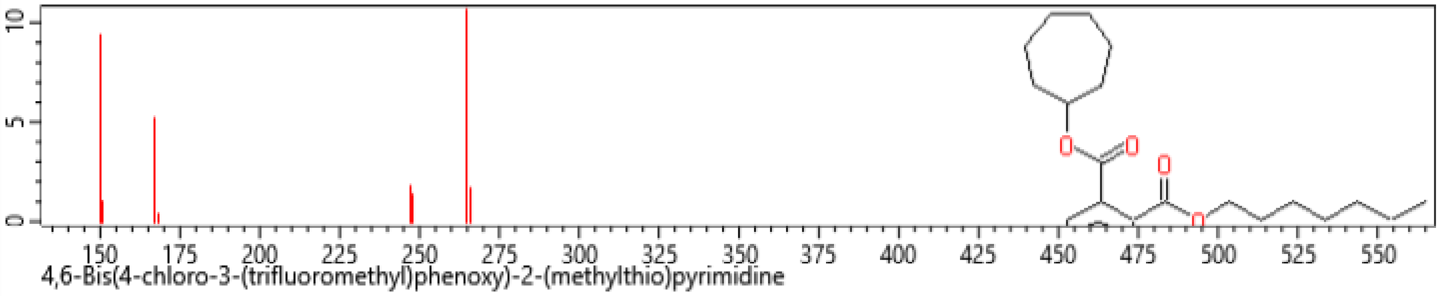

4,6-Bis(4-chloro-3-(trifluoromethyl)phenoxy)-2-(methylthio)pyrimidine

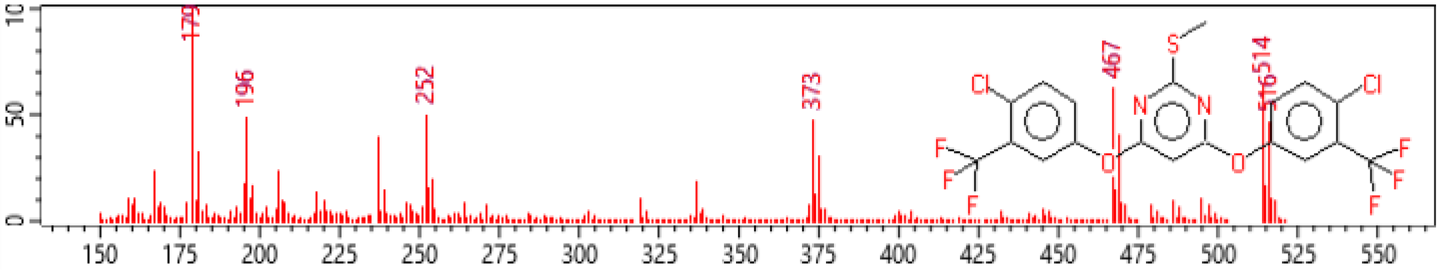


N-(4-Hexanoyl-1,2,2-trimethyl-5-oxo-3-pyrrolin-3-yl)butyramide

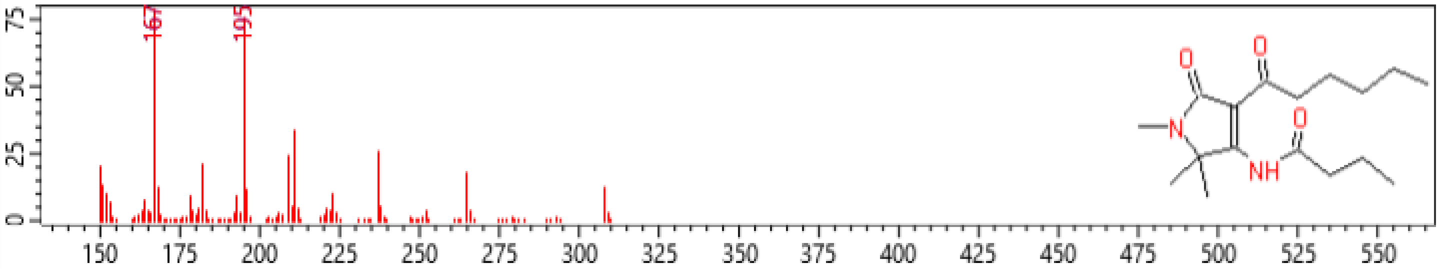


**Glycine, N-[(3α,5β)-24-oxo-3-[(trimethylsilyl)oxy]cholan-24-yl]-, methyl ester**

Peak: 18.91 - 18.99 min (scan #3323 - #3345) Subtracted noise: 18.79 - 18.87 min (scan #3291 - #3313), 19.04 - 19.11 min (scan #33

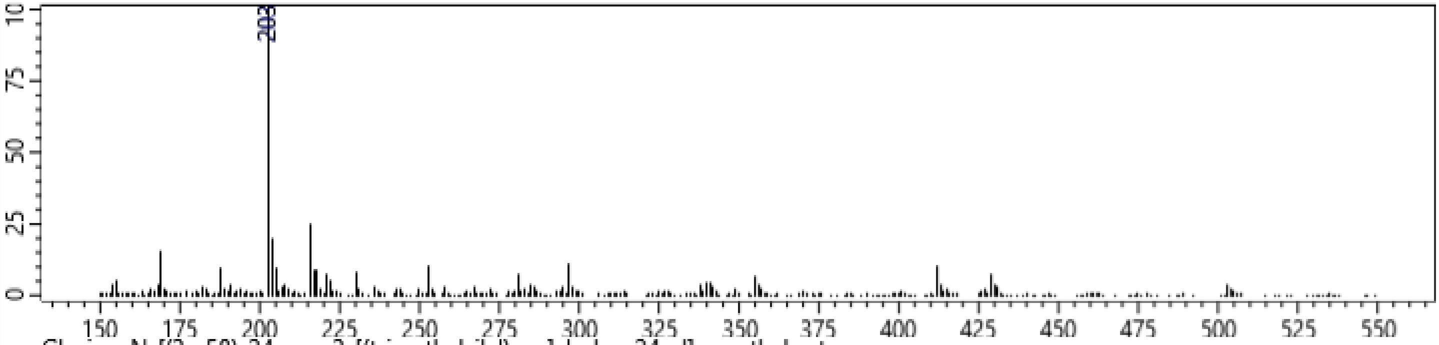


Glycine, N-[(3α,5β)-24-oxo-3-[(trimethylsilyl)oxy]cholan-24-yl]-, methyl ester

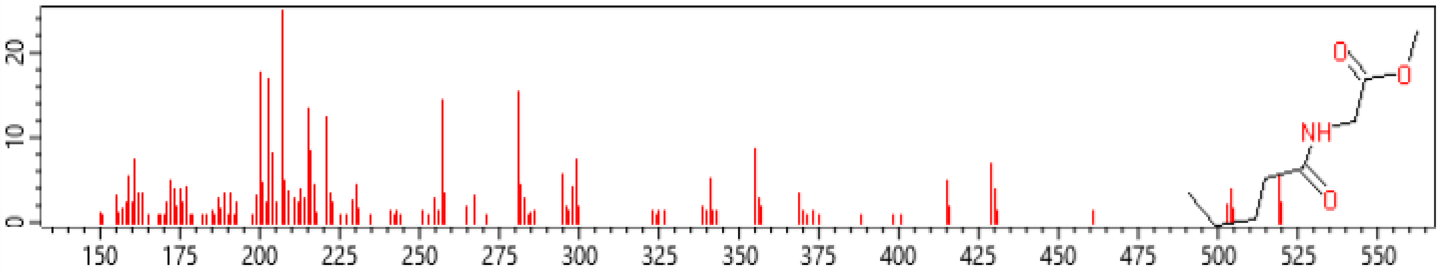

Gitoxigenin

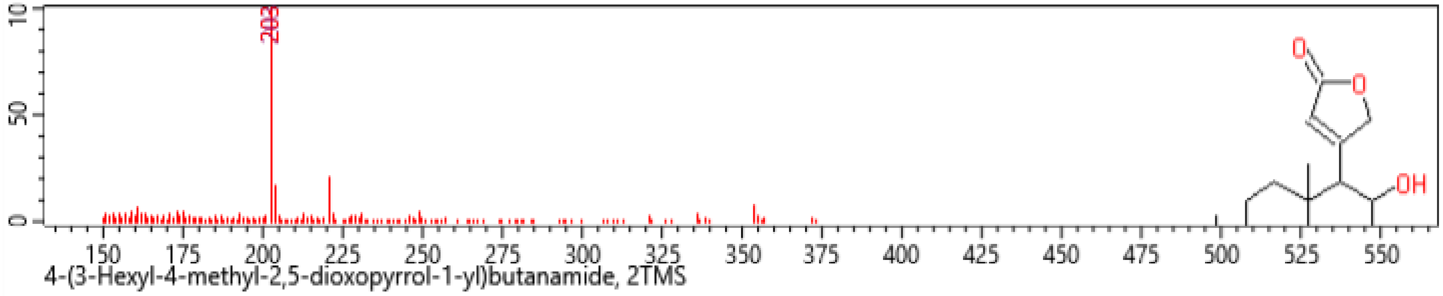


4-(3-Hexyl-4-methyl-2,5-dioxopyrrol-1-yl)butanamide, 2TMS

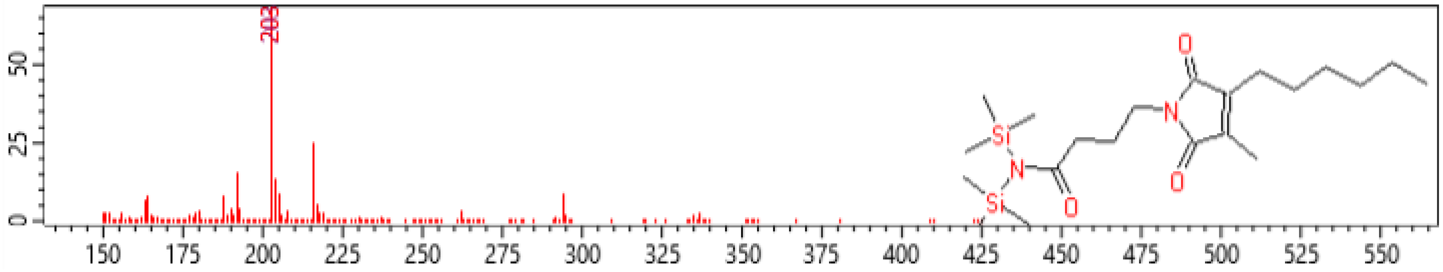


Olean-12-ene-3,28-diol, (3β)-

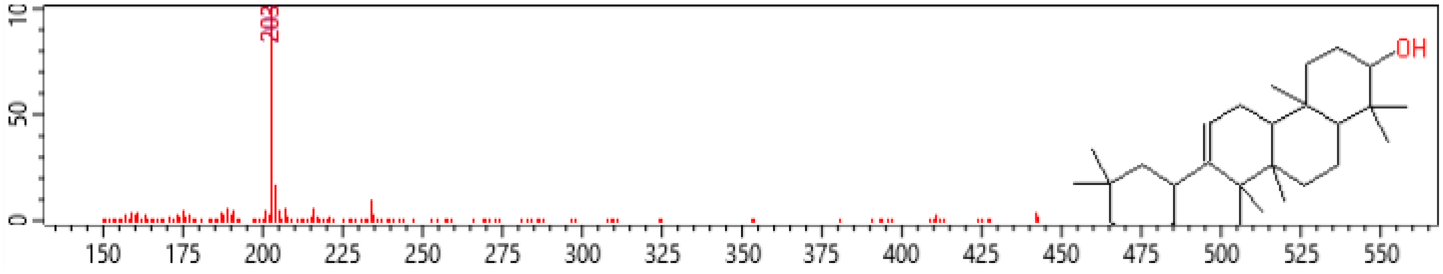


Cyclodecasiloxane, eicosamethyl-

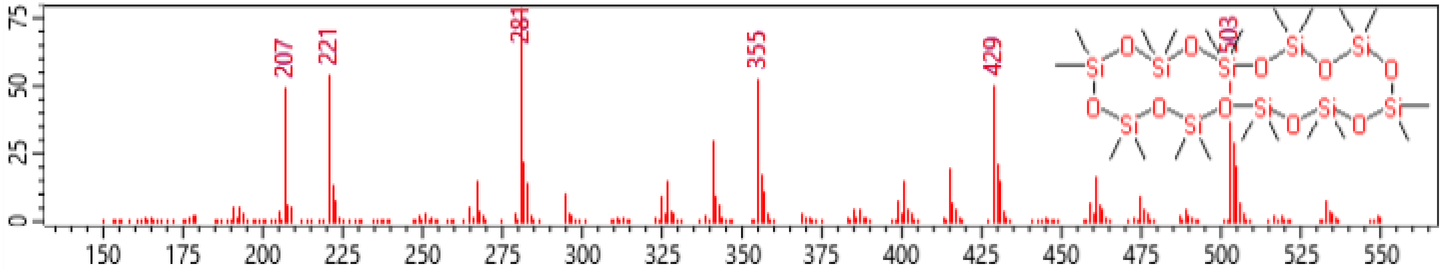


**Dehydroabietic acid, TMS derivative**

Peak: 19.32 - 19.36 min (scan #3439 - #3449) Subtracted noise: 19.27 - 19.30 min (scan #3423 - #3432), 19.37 - 19.41 min (scan #34 [illegible]

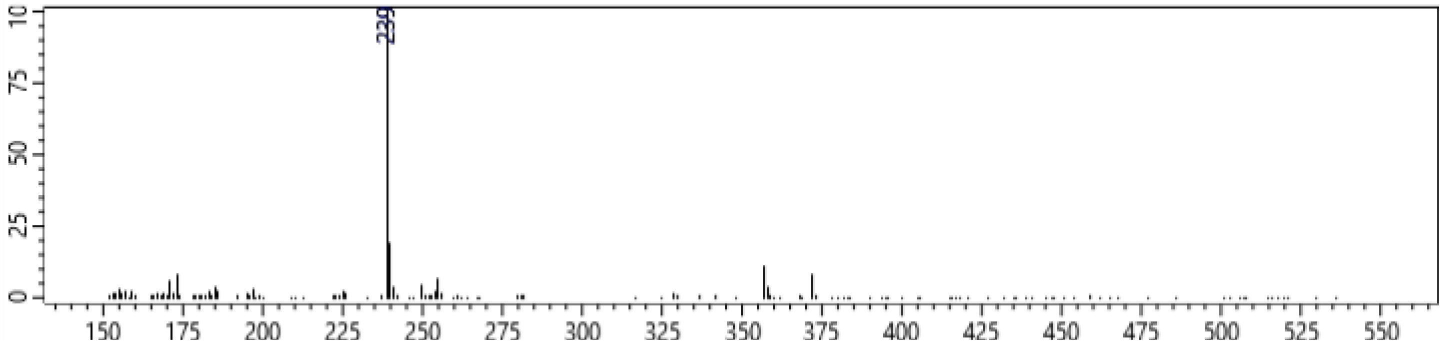

Dehydroabietic acid, TMS derivative

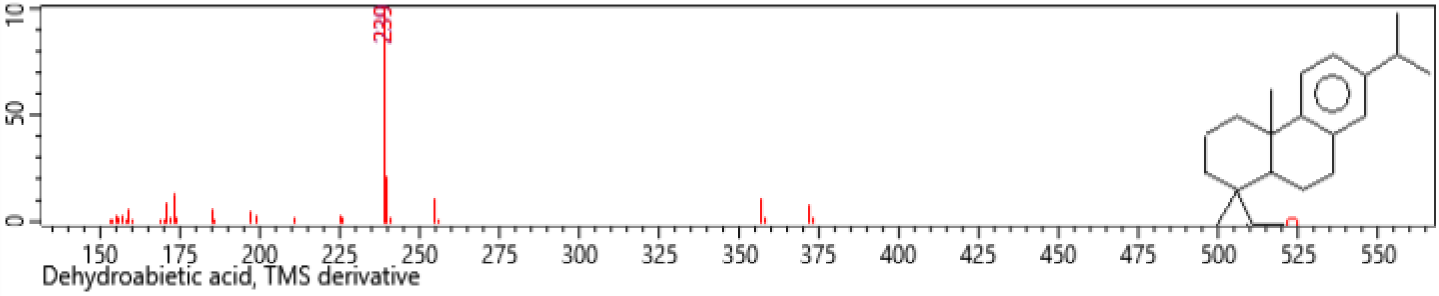


Dehydroabietic acid, TMS derivative

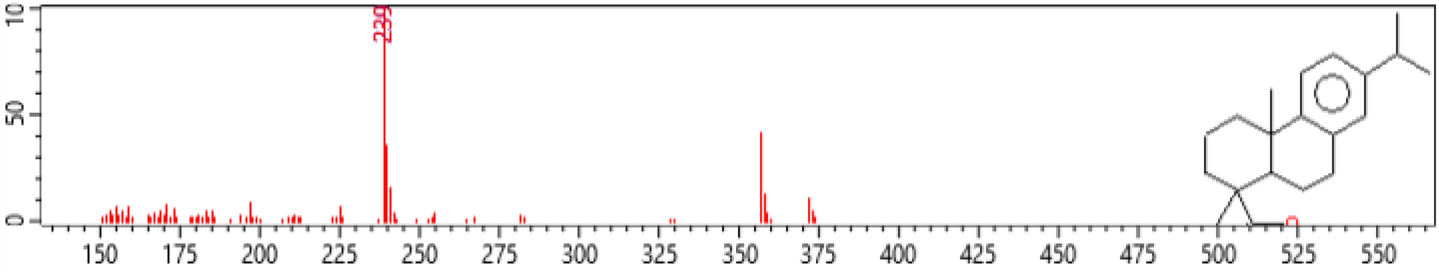


Dehydroabietic acid, TMS derivative

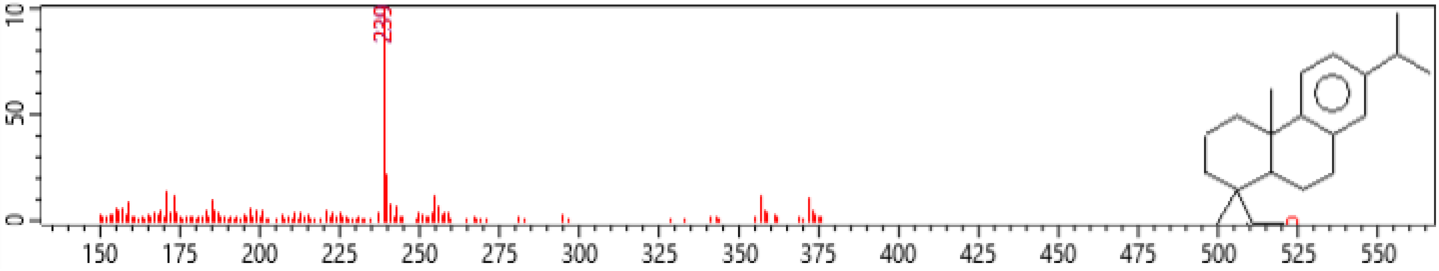


4-Hydroxy-2,2',3',5',6'-pentachlorobiphenyl

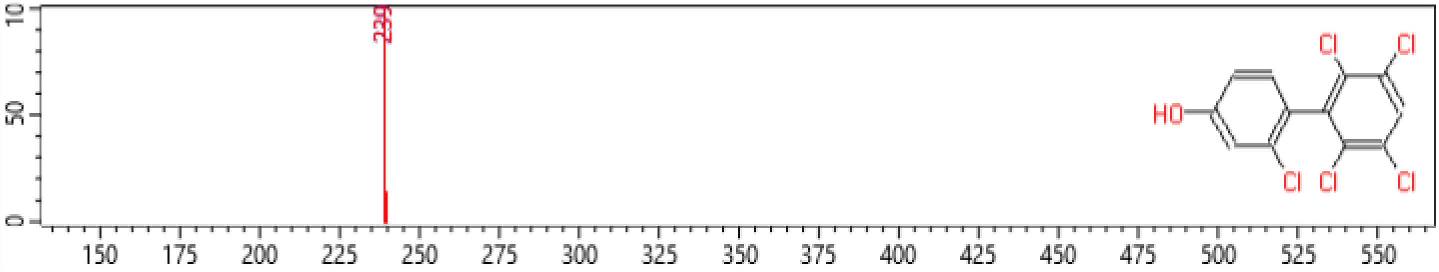


Methyl dehydroabietate

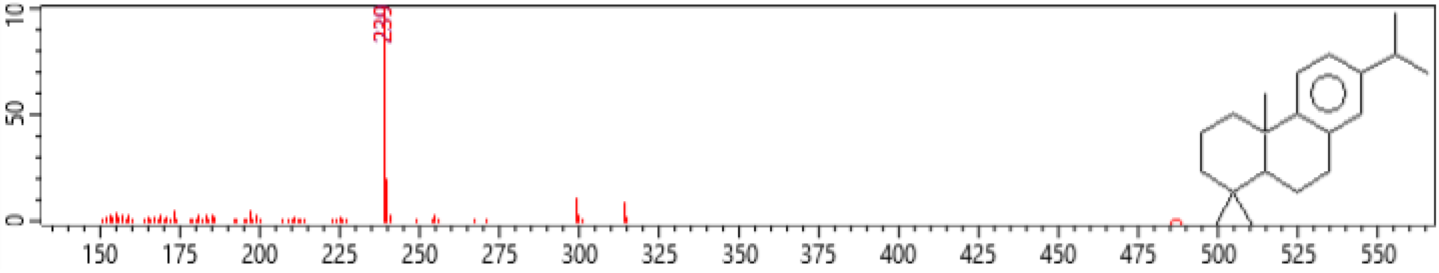

**1-Monomyristin, 2TMS derivative**

Peak: 19.40 - 19.45 min (scan #3460 - #3474) Subtracted noise: 19.32 - 19.37 min (scan #3439 - #3452), 19.48 - 19.53 min (scan #34

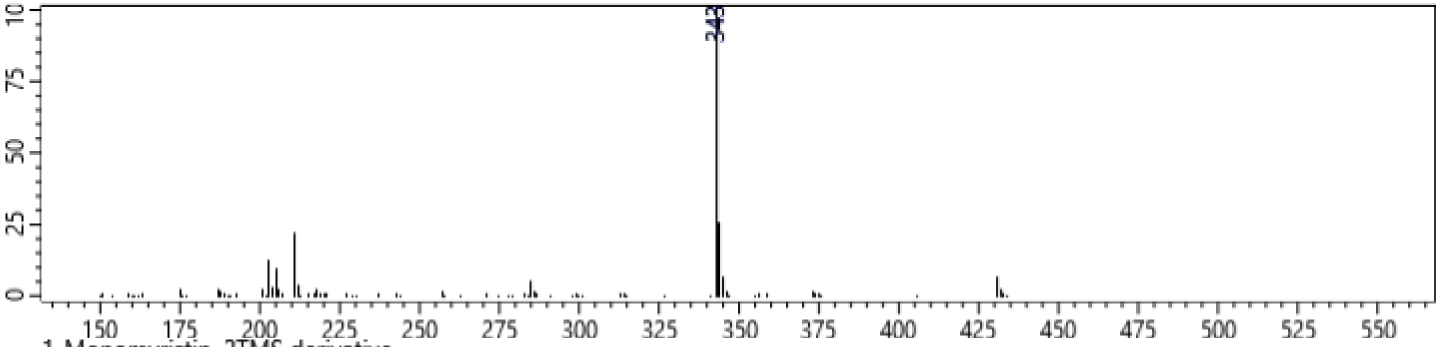


1-Monomyristin, 2TMS derivative

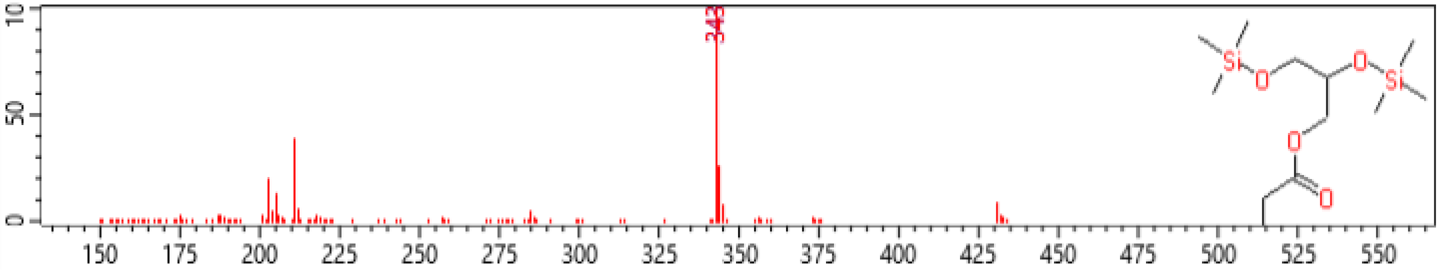


1-Monomyristin, 2TMS derivative

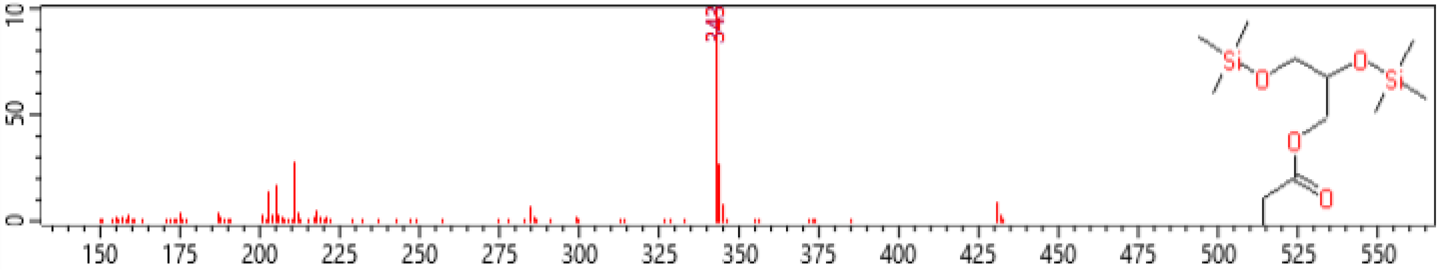


2,3-Dihydroxypropyl 12-methyltridecanoate, 2TMS derivative

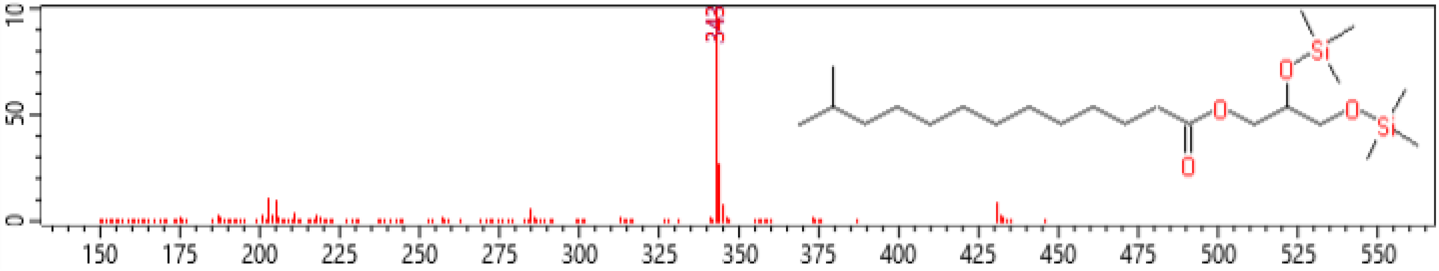


5,7,3',4'-Tetramethoxyisoflavone

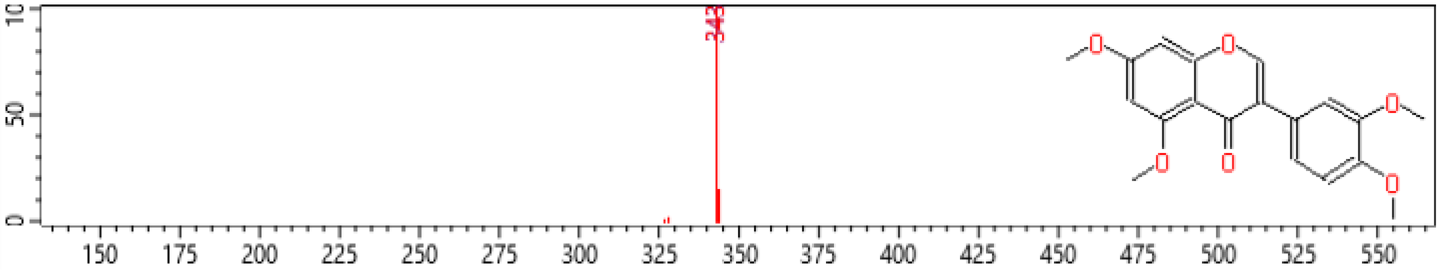

3-[2-Hydroxy-5-(trifluoromethyl)phenyl]-6-(trifluoromethyl)-1H-benzimidazol-2-one

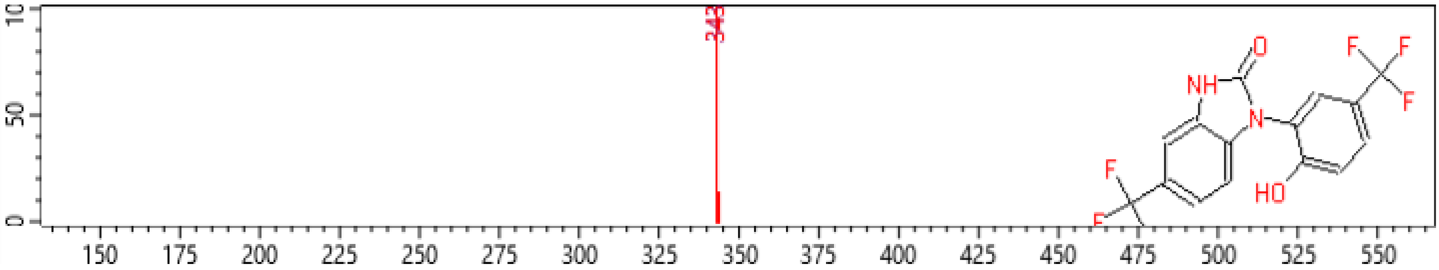


**Cyclopentene, 2,3,3-trimethyl-4,4-diphenyl-5-phenylimino-**

Peak: 19.54 - 19.59 min (scan #3500 - #3512) Subtracted noise: 19.47 - 19.51 min (scan #3479 - #3490), 19.60 - 19.64 min (scan #35

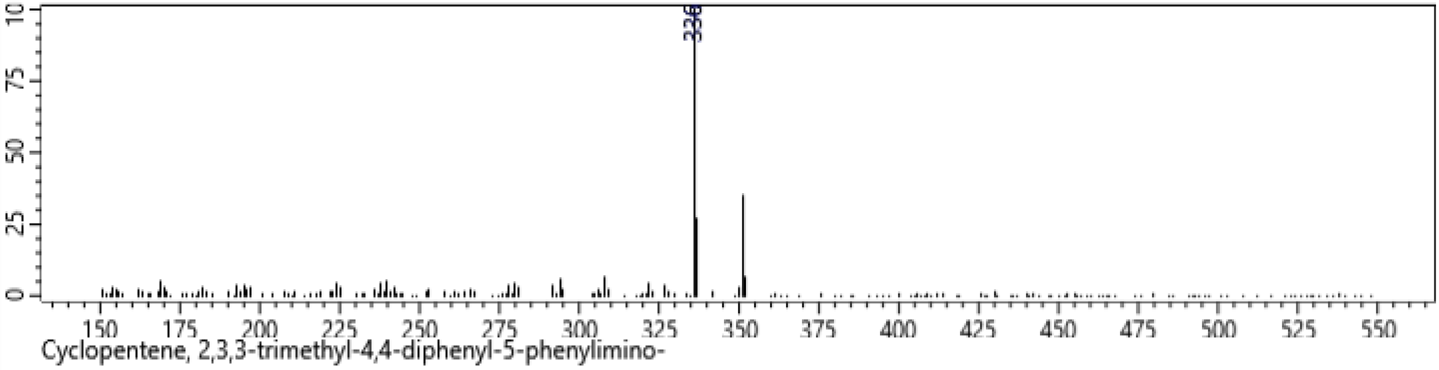


Cyclopentene, 2,3,3-trimethyl-4,4-diphenyl-5-phenylimino-

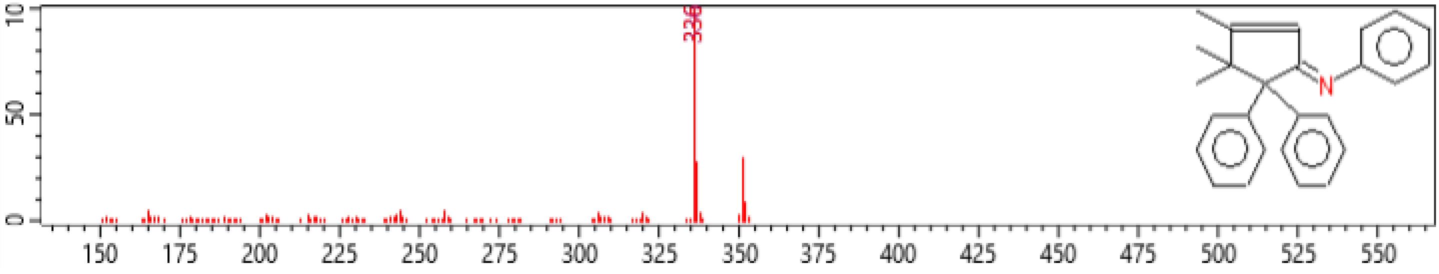


Benzamide, 2-methyl-N-benzyl-N-nonyl-

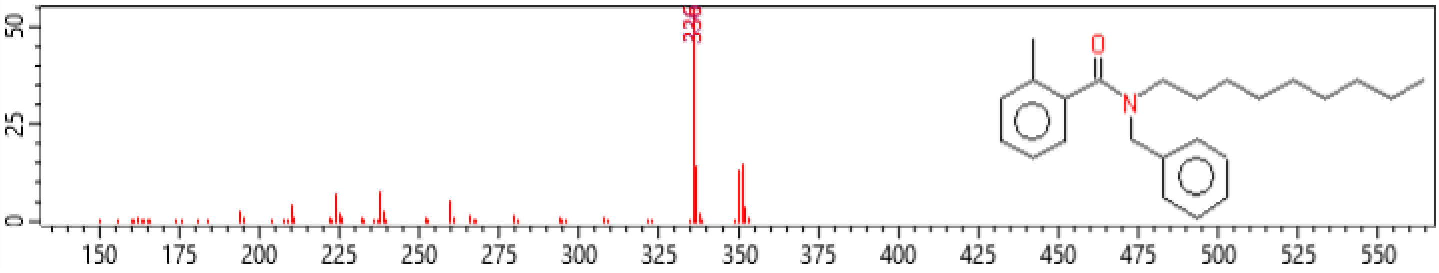


1H-isoindole-1,3(2H)-dione, 2-[4-(diethylamino)-2-methylphenyl]-4-(dimethylamino)-

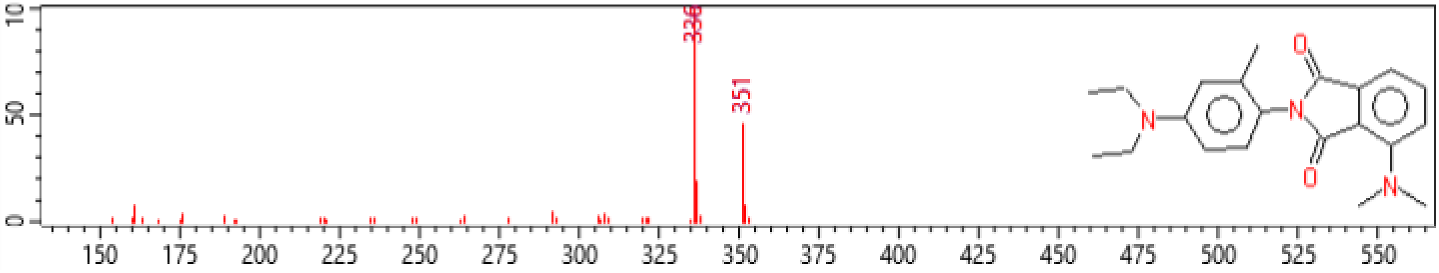

2,5-Di-tert-butylaniline, N-pentafluoropropionyl-

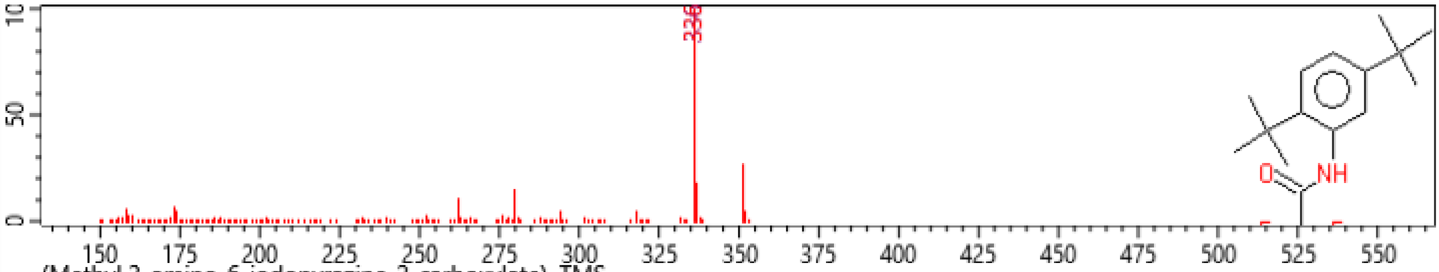


(Methyl 3-amino-6-iodopyrazine-2-carboxylate), TMS

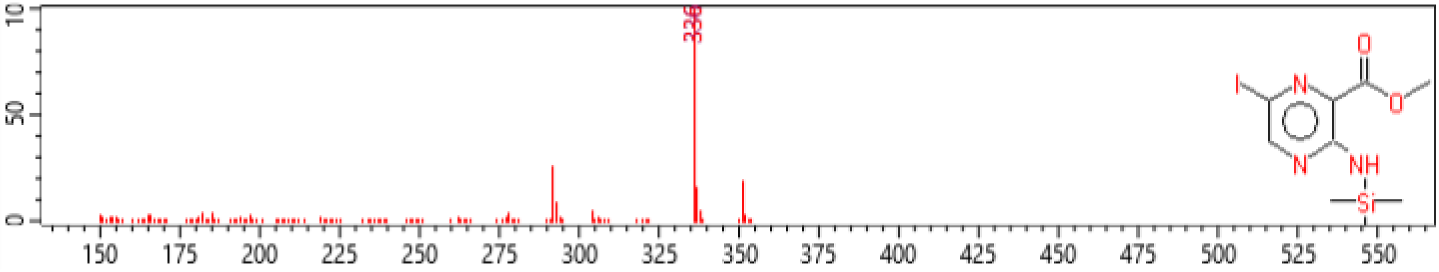


**Oleamide, TMS derivative**

Peak: 19.68 - 19.76 min (scan #3538 - #3560) Subtracted noise: 19.52 - 19.60 min (scan #3494 - #3515), 19.76 - 19.84 min (scan #35

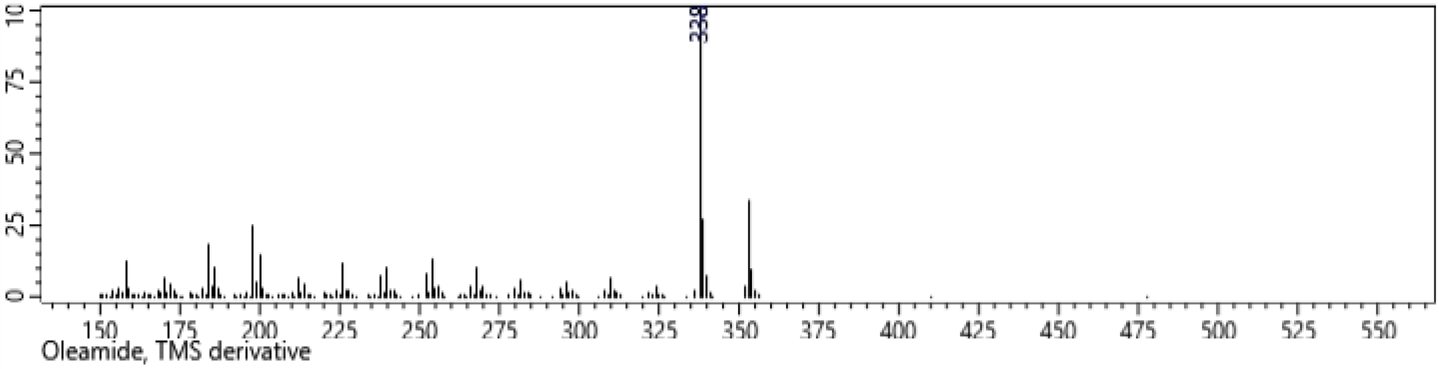


Oleamide, TMS derivative

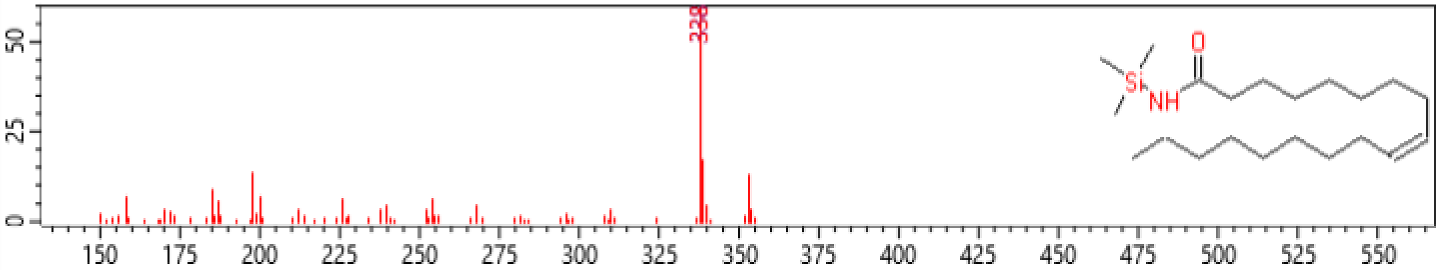


7-Chloro-1-cyclopropyl-6-fluoro-4-oxo-1,4-dihydro-3-quinolinecarboxylic acid, TMS derivative

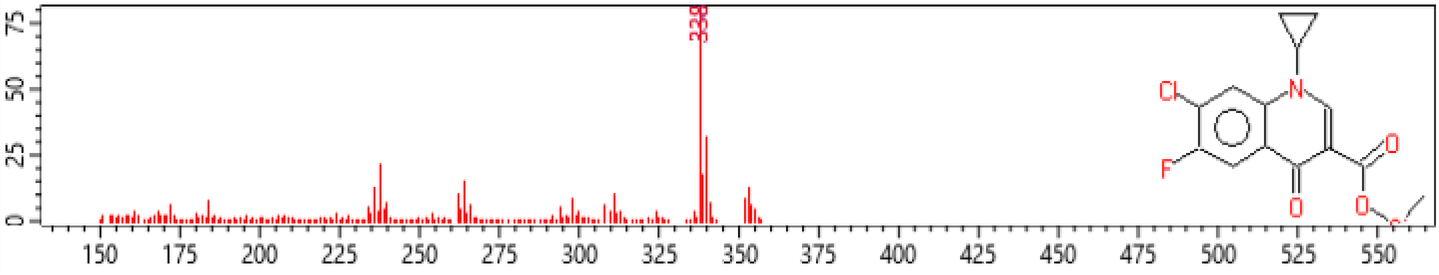

Stearamide, N-(3-methylbutyl)-

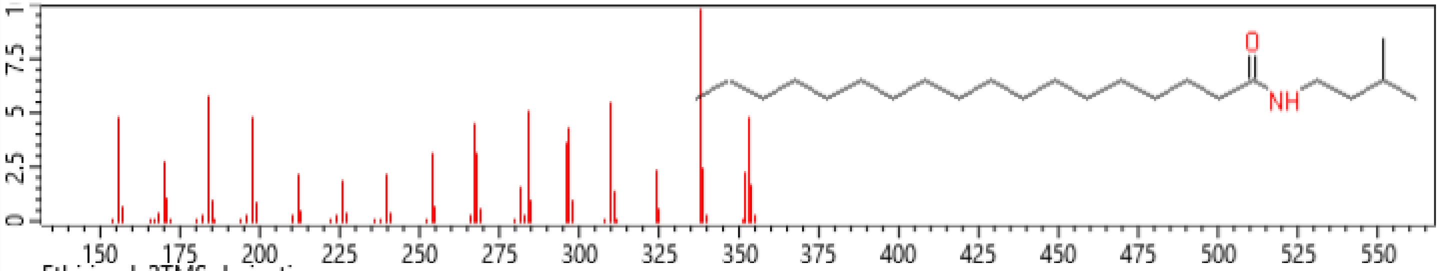


Ethirimol, 2TMS derivative

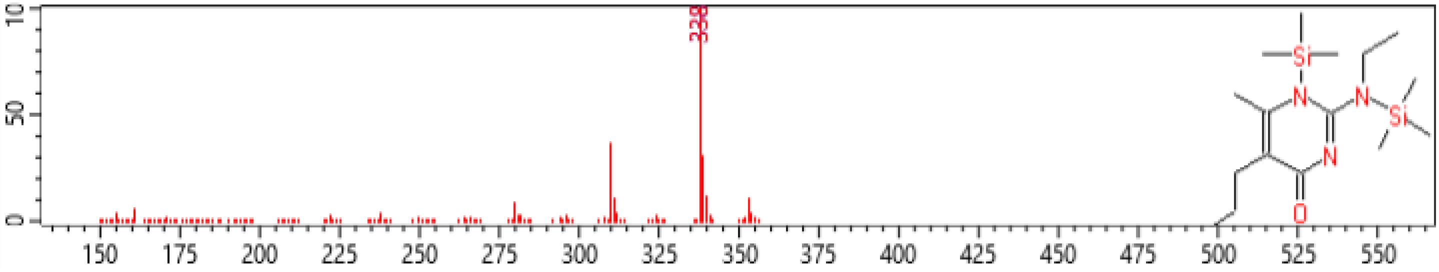


Butyramide, 3-methyl-N-octadecyl-

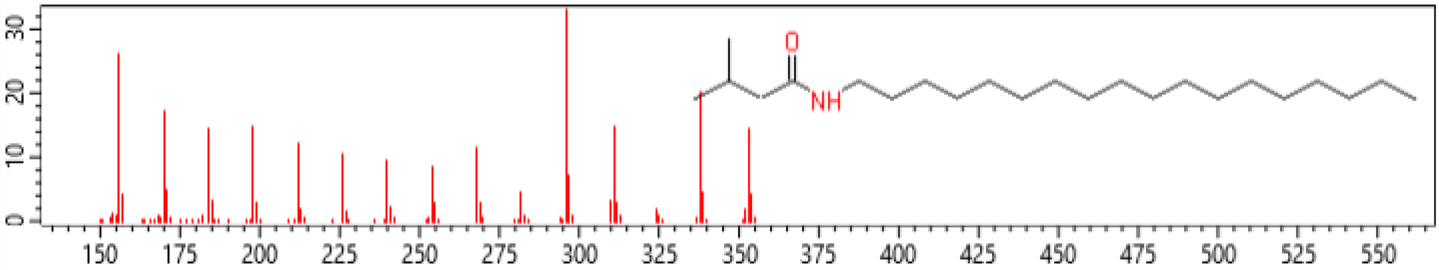


**Benzenecarbohydrazonic acid, N-phenyl-, 3-methylphenyl ester**

Peak: 19.78 - 19.82 min (scan #3565 - #3577) Subtracted noise: 19.71 - 19.76 min (scan #3548 - #3560), 19.86 - 19.90 min (scan #35

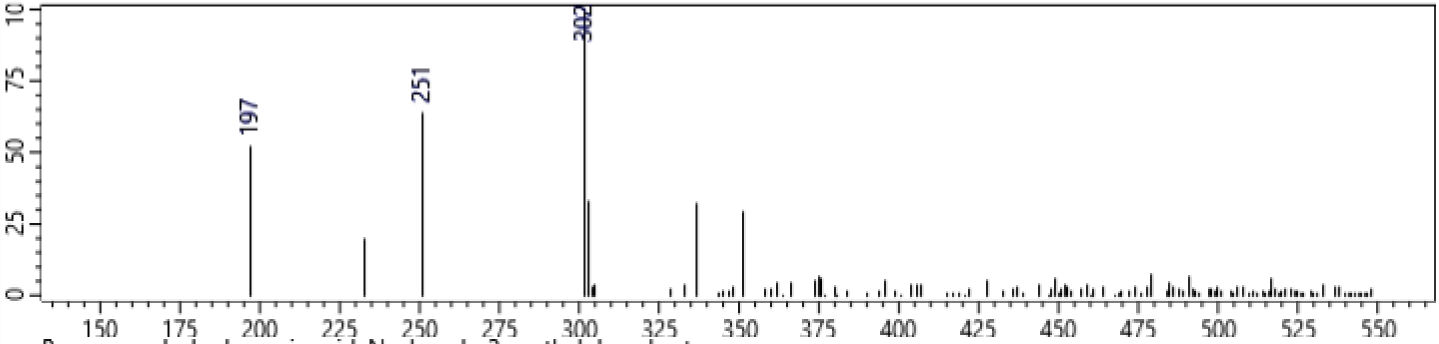


Benzenecarbohydrazonic acid, N-phenyl-, 3-methylphenyl ester

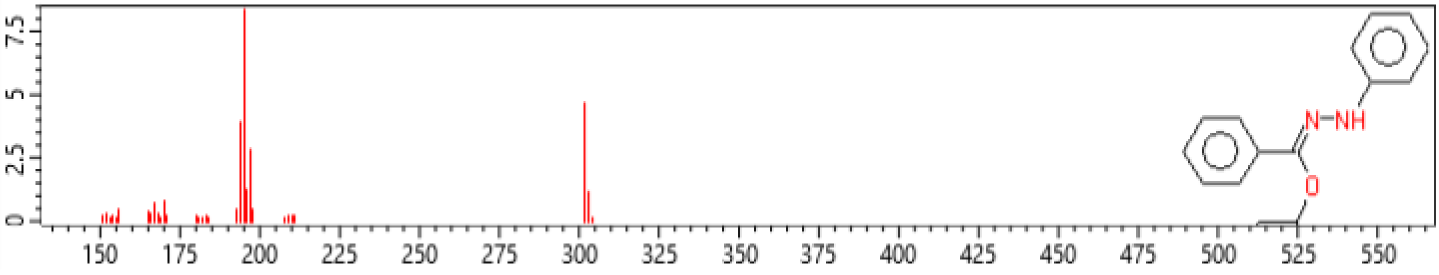

2,3,4-Trifluorobenzoic acid, 2-naphthyl ester

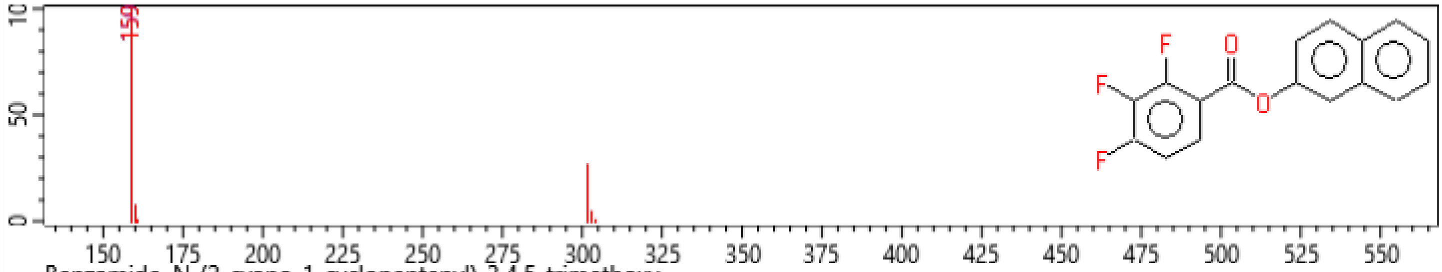


Benzamide, N-(2-cyano-1-cyclopentenyl)-3,4,5-trimethoxy-

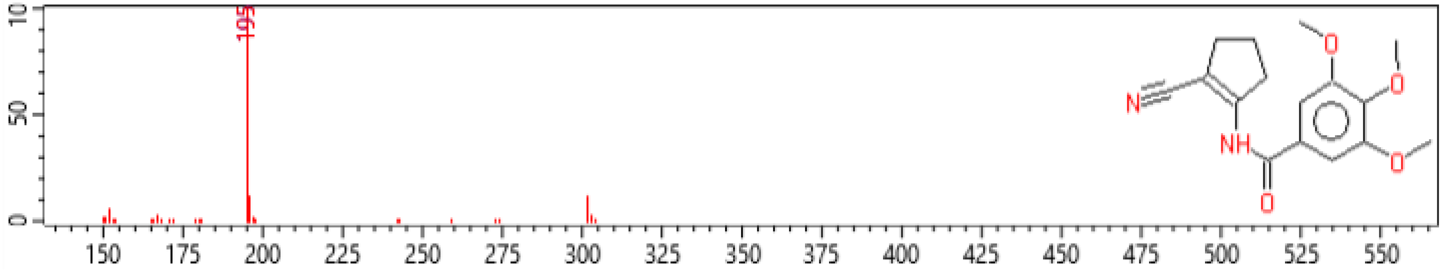


2-Amino-4-(4-methoxyphenyl)-6-phenylnicotinonitrile

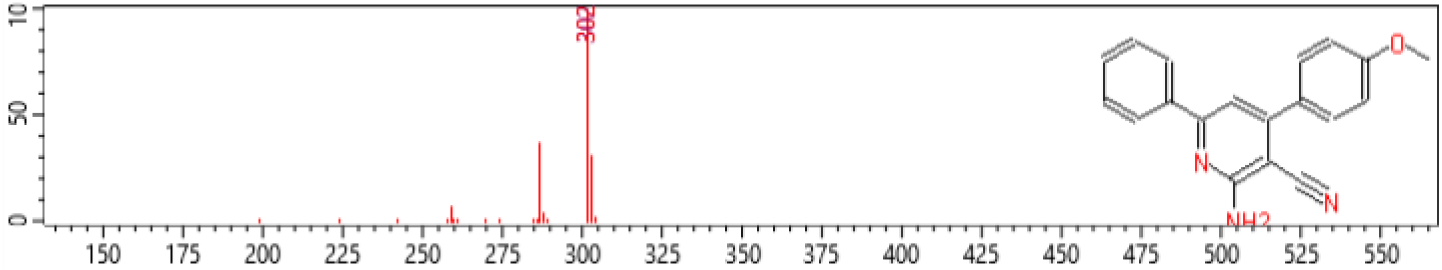


Sanguinarine cation

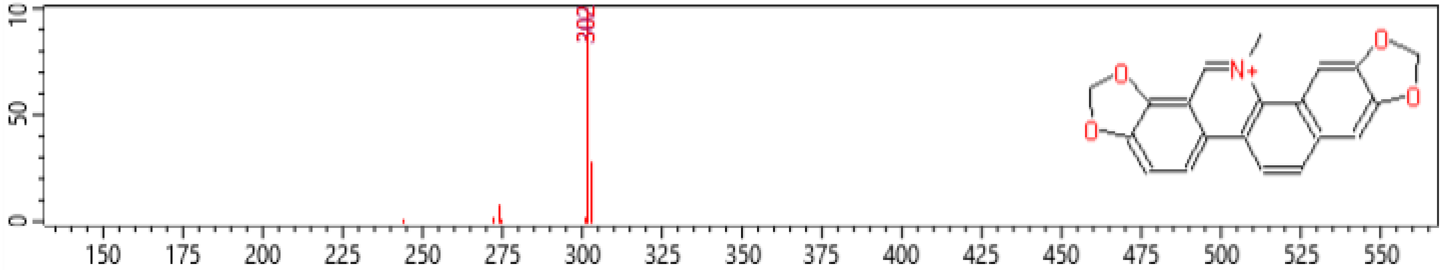


**6,6'-Thiobis(2-bromo-4-chlorophenol)**

Peak: 19.88 - 19.92 min (scan #3593 - #3605) Subtracted noise: 19.81 - 19.85 min (scan #3575 - #3587), 19.95 - 19.99 min (scan #36

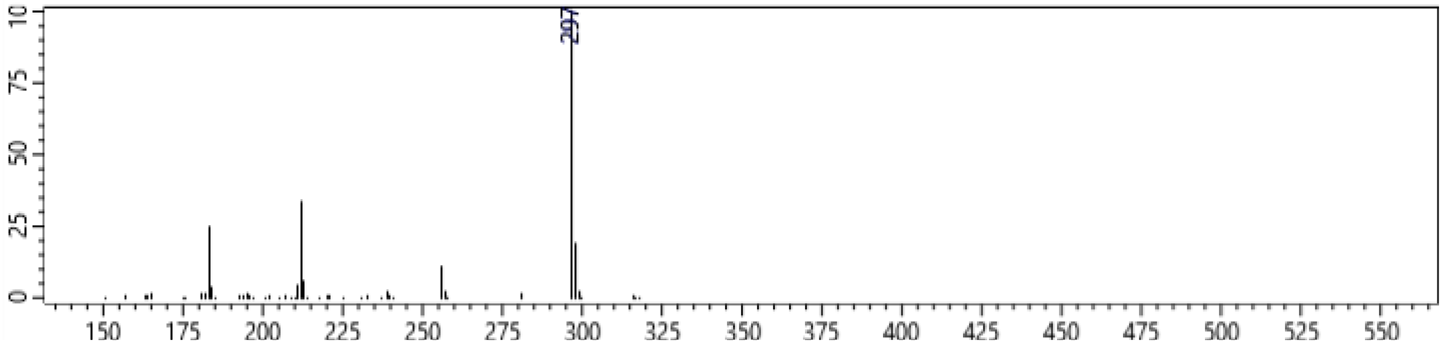

6,6'-Thiobis(2-bromo-4-chlorophenol)

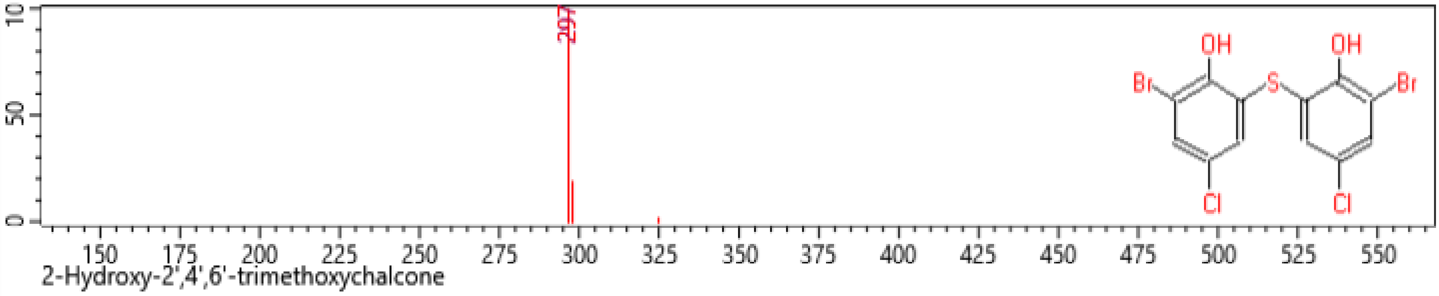


2-Hydroxy-2',4',6'-trimethoxychalcone

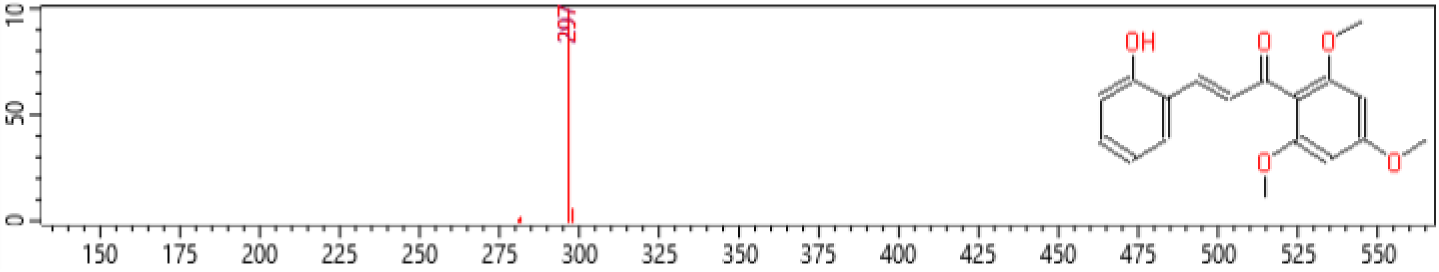


5-[(7-Chloro-4-quinolinyl)amino]-2-hydroxybenzoic acid

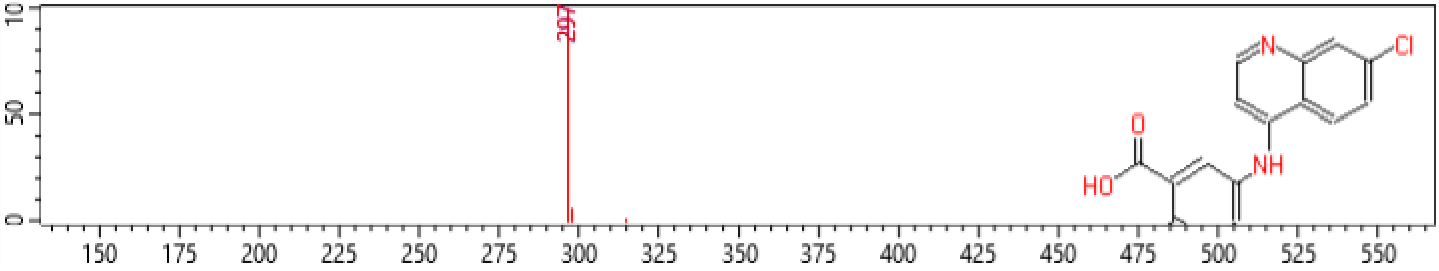


5,5'-Methylenebis(2-hydroxy-3-methylbenzoic acid)

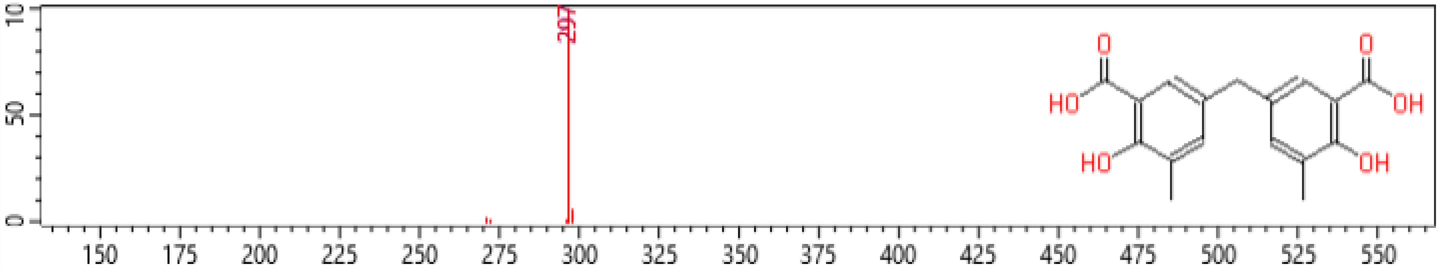


7,4'-Dimethoxy-5-hydroxyisoflavone

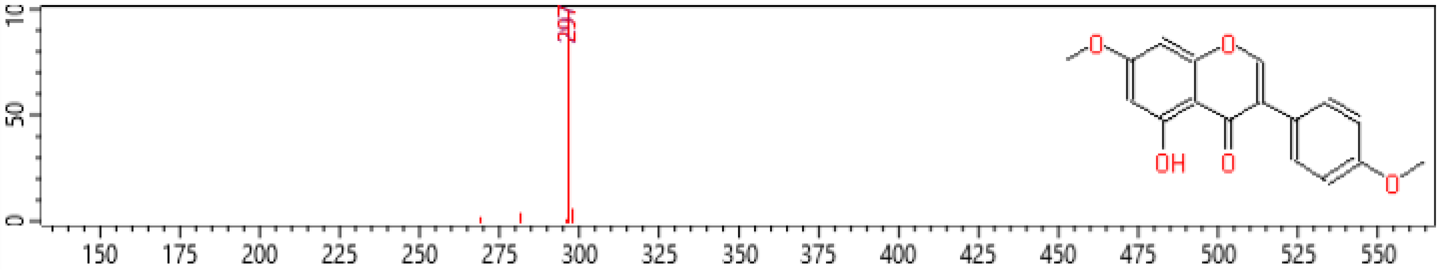

**N-Trimethylsilylstearamide**

Peak: 20.12 - 20.19 min (scan #3660 - #3680) Subtracted noise: 20.00 - 20.07 min (scan #3627 - #3646), 20.22 - 20.29 min (scan #36

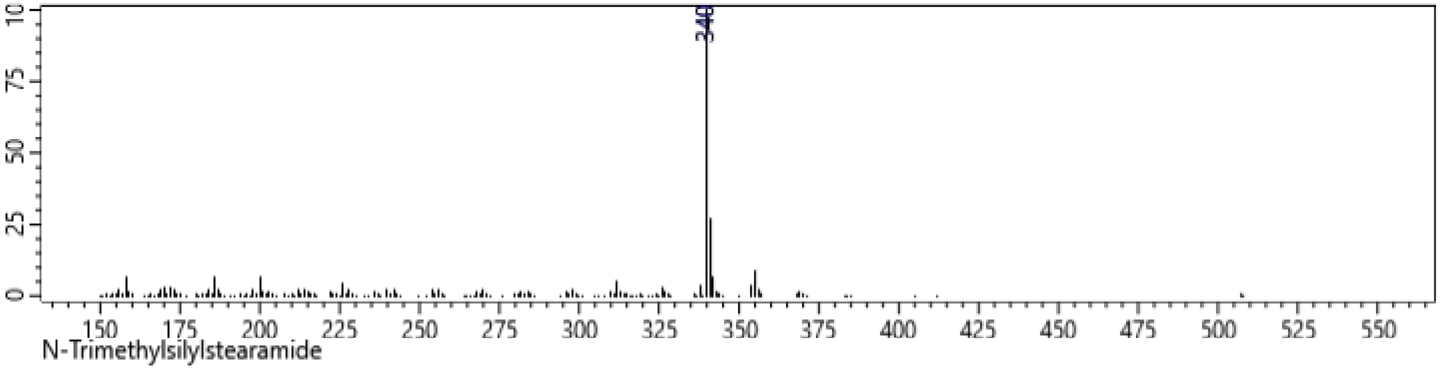


N-Trimethylsilylstearamide

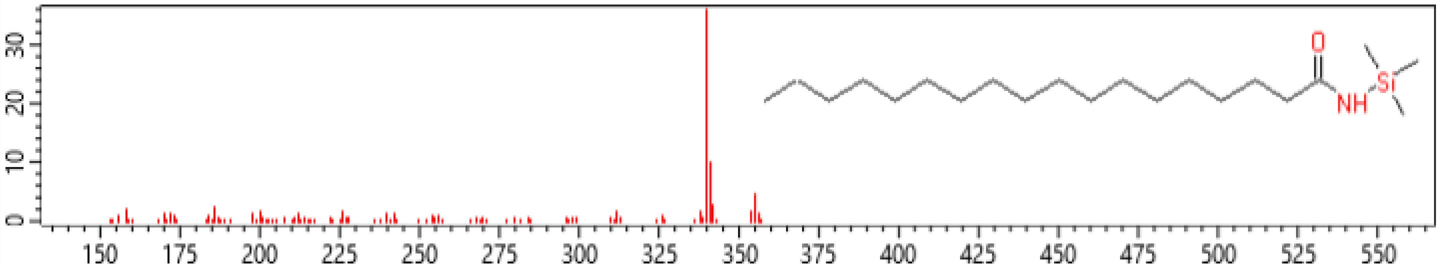


Carbamic acid, N-(2-butyl)-N-tetradecyl-, butyl ester

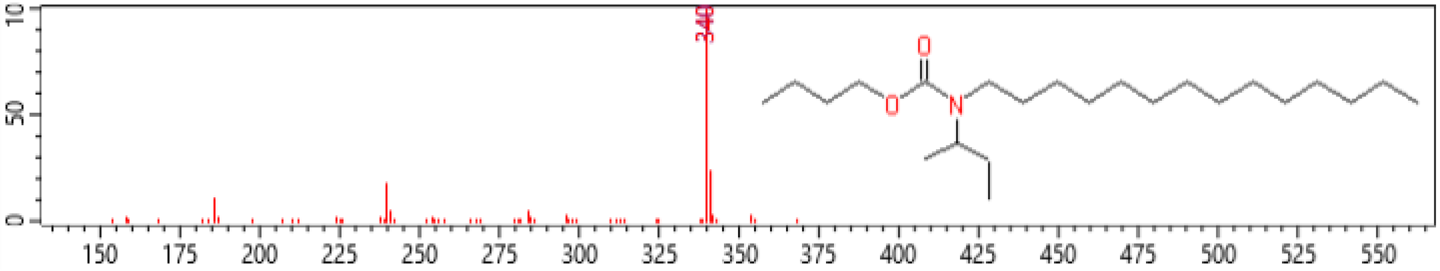


β-Alanine, N-[2-(2-pyridinyl)-6-(1,2,4,5-tetrahydro-3H-3-benzazepin-3-yl)-4-pyrimidinyl]-

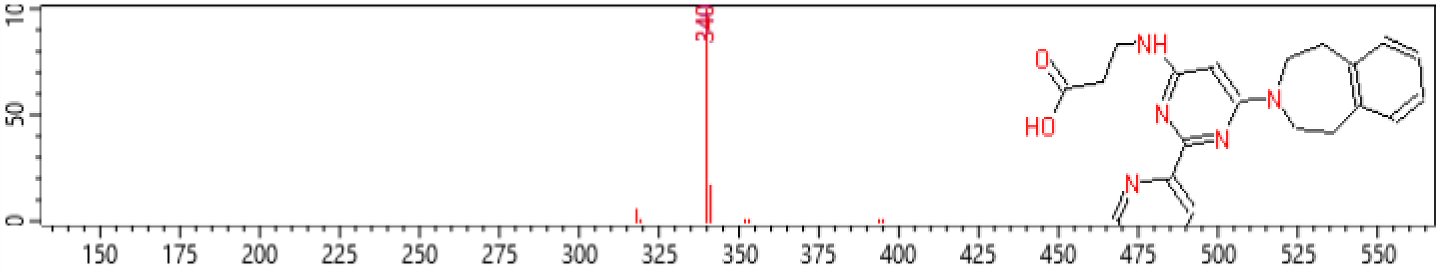


Carbamic acid, N-(2-butyl)-N-pentadecyl-, propyl ester

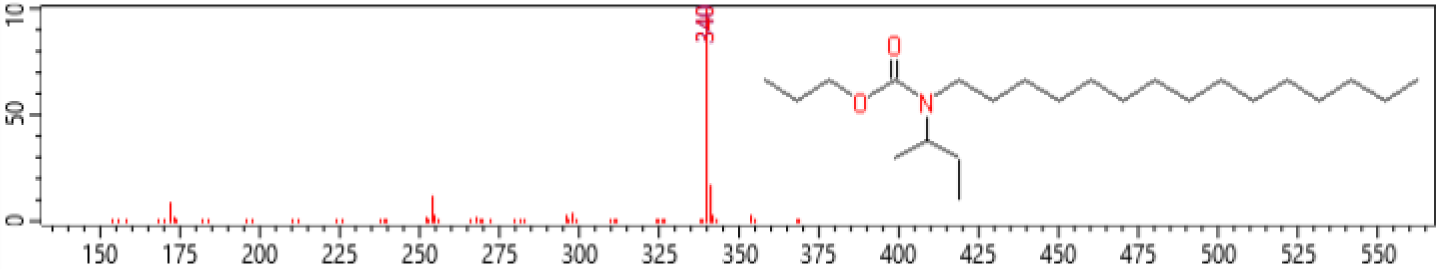

Carbonic acid, monoamide, N-(2-pentyl)-N-tetradecyl-, butyl ester

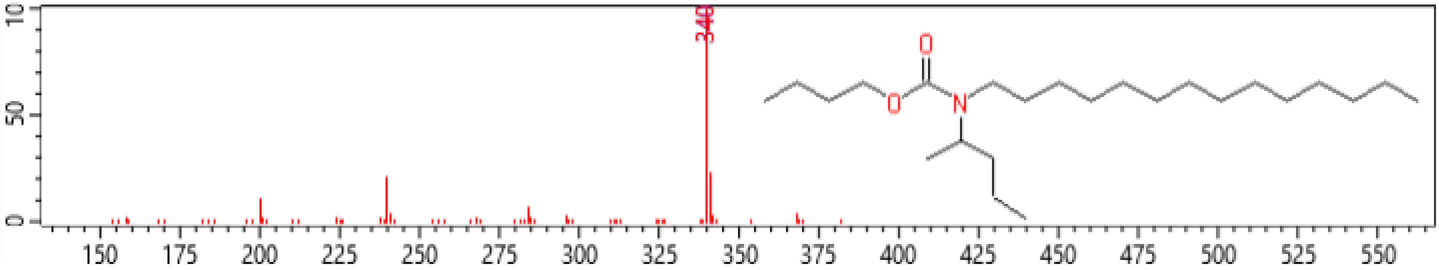


**JWH 149**

Peak: 21.17 - 21.21 min (scan #3954 - #3966) Subtracted noise: 21.10 - 21.14 min (scan #3934 - #3946), 21.23 - 21.27 min (scan #39

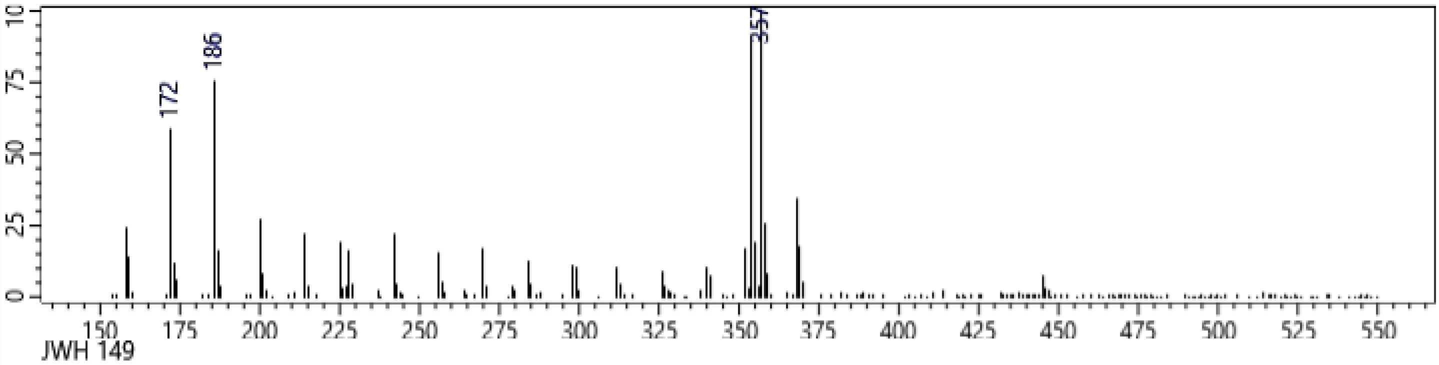


JWH 149

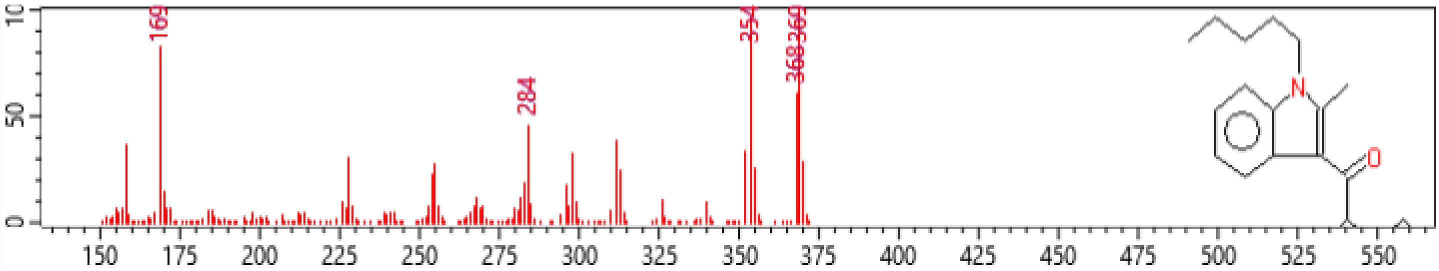


Quinolin-4(1H)-one, 3-ethyl-2-tridecyl-

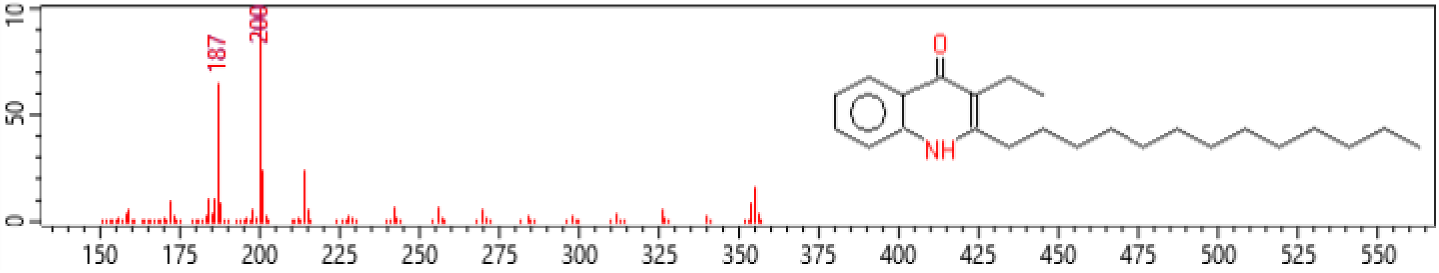


JWH 116

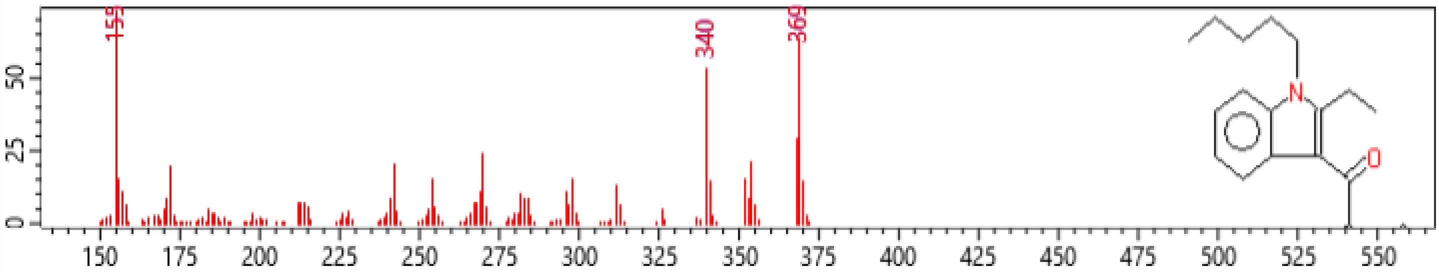

1-Naphthaleneacetic acid, dodecyl ester

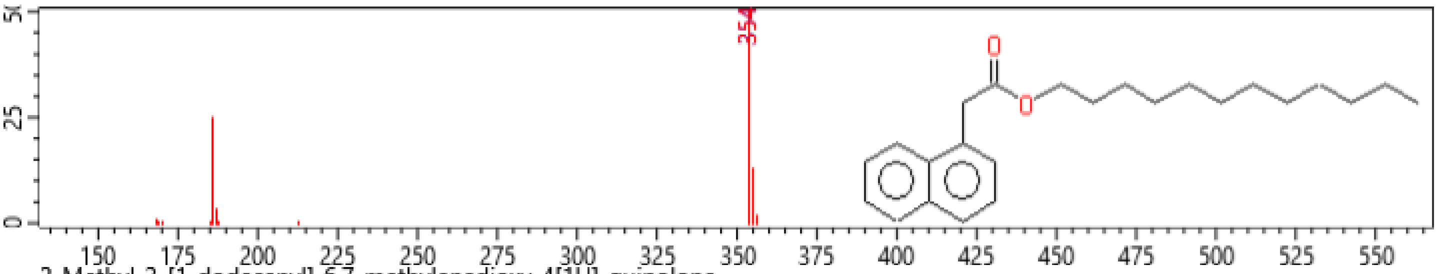


2-Methyl-3-[1-dodecenyl]-6,7-methylenedioxy-4[1H]-quinolone

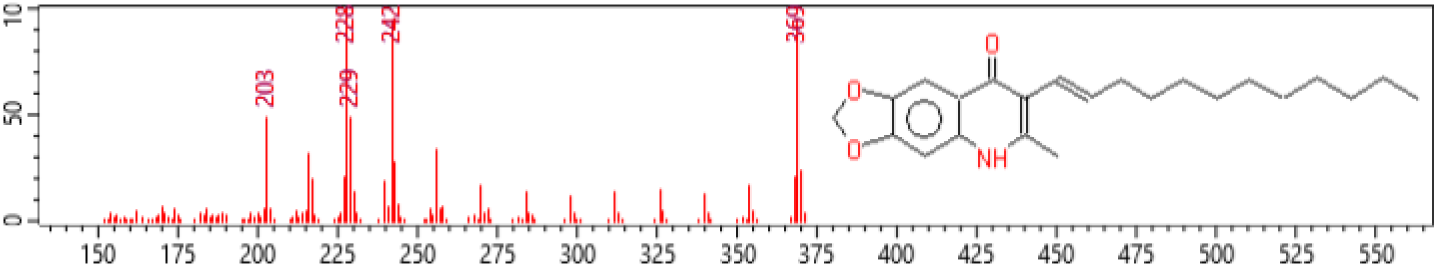


**2,2'-Methylenebis(6-tert-butyl-4 methylphenol), O,O'-bis(trimethylsilyl)-**

Peak: 21.27 - 21.34 min (scan #3982 - #4002) Subtracted noise: 21.15 - 21.23 min (scan #3950 - #3971), 21.38 - 21.46 min (scan #40

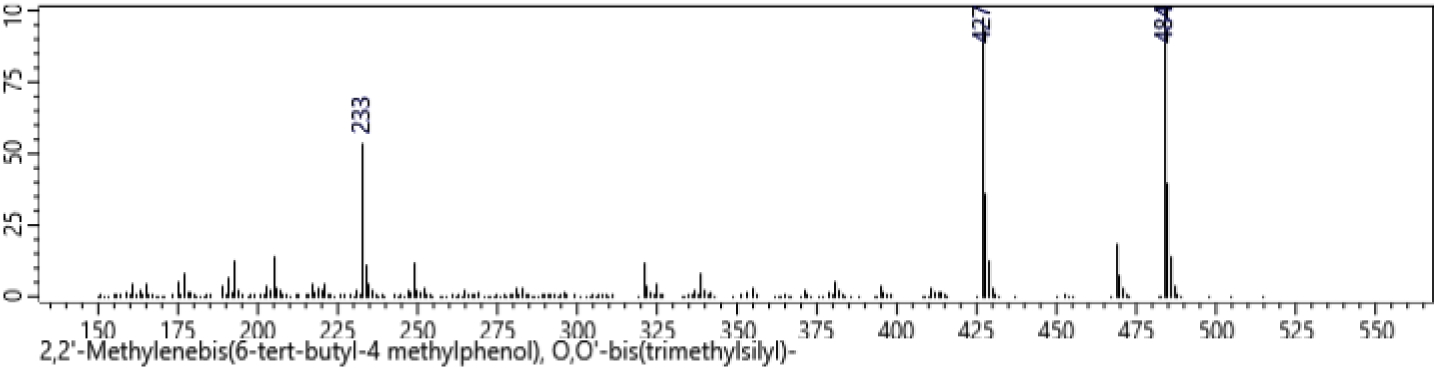


2,2'-Methylenebis(6-tert-butyl-4 methylphenol), O,O'-bis(trimethylsilyl)-

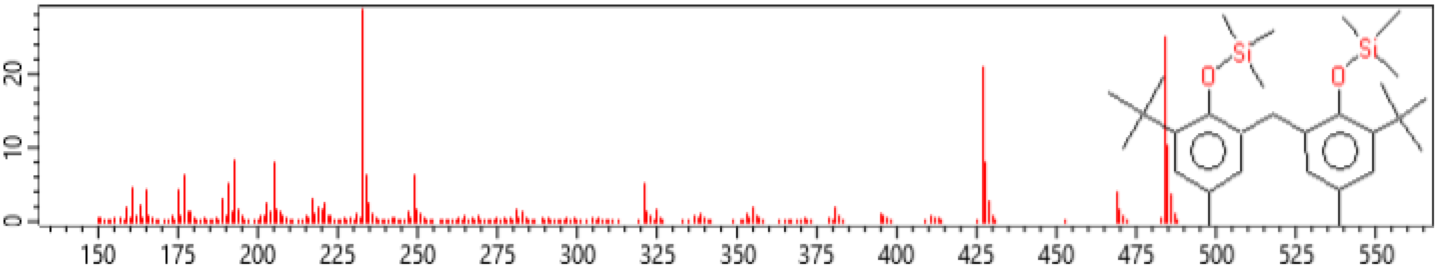


benzene, 1,1'-methylenebis[3-(1,1-dimethylethyl)-5-methyl-4-[(trimethylsilyl)oxy]-

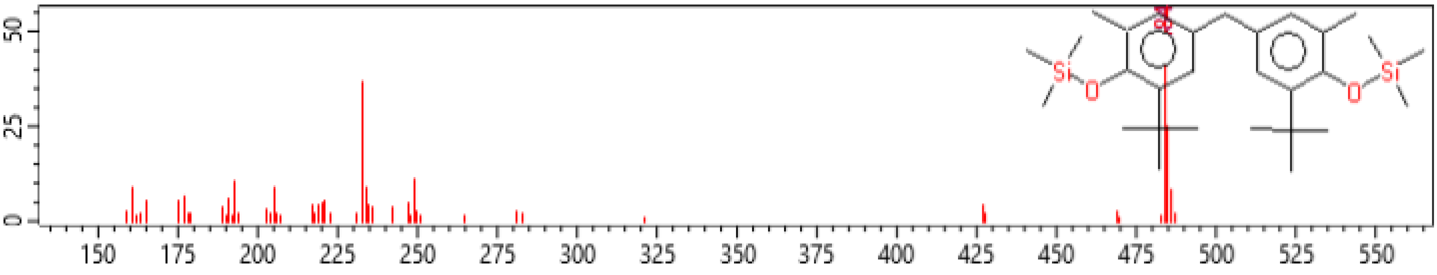

6,2'-Bis(tert.-butyldimethylsilyl)oxyflavanone

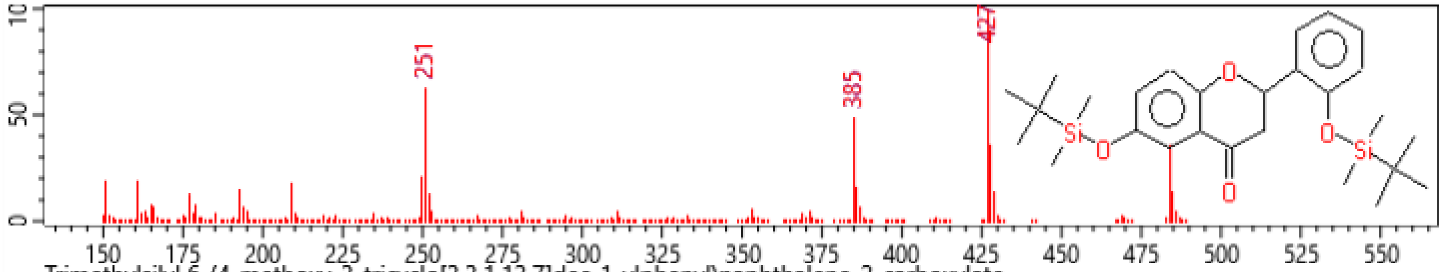


Trimethylsilyl 6-(4-methoxy-3-tricyclo[3.3.1.13,7]dec-1-ylphenyl)naphthalene-2-carboxylate

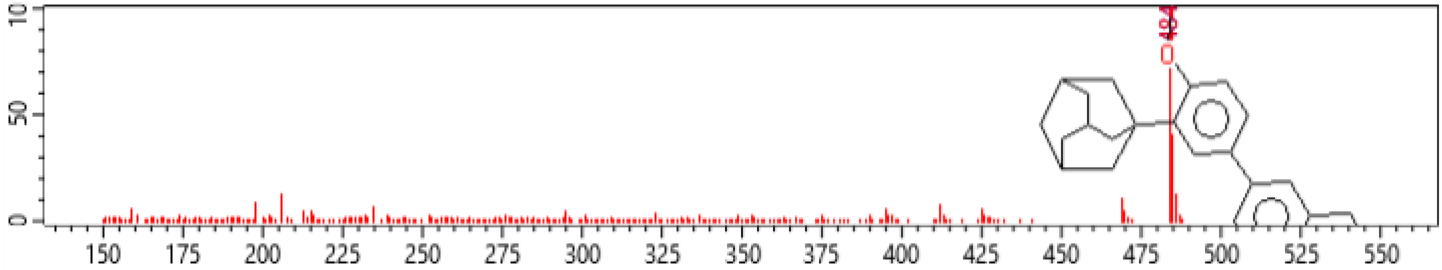

2-(2-Chloro-5-iodophenyl)-1,3-benzoxazol-5-amine, N-(tert-butyldimethylsilyl)-

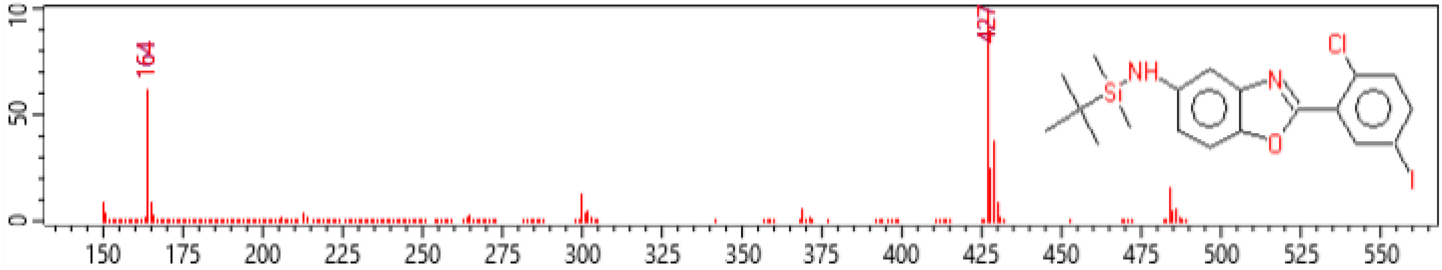


**Cyclodecasiloxane, eicosamethyl-**

Peak: 21.50 - 21.56 min (scan #4046 - #4062) Subtracted noise: 21.40 - 21.46 min (scan #4019 - #4035), 21.59 - 21.65 min (scan #40

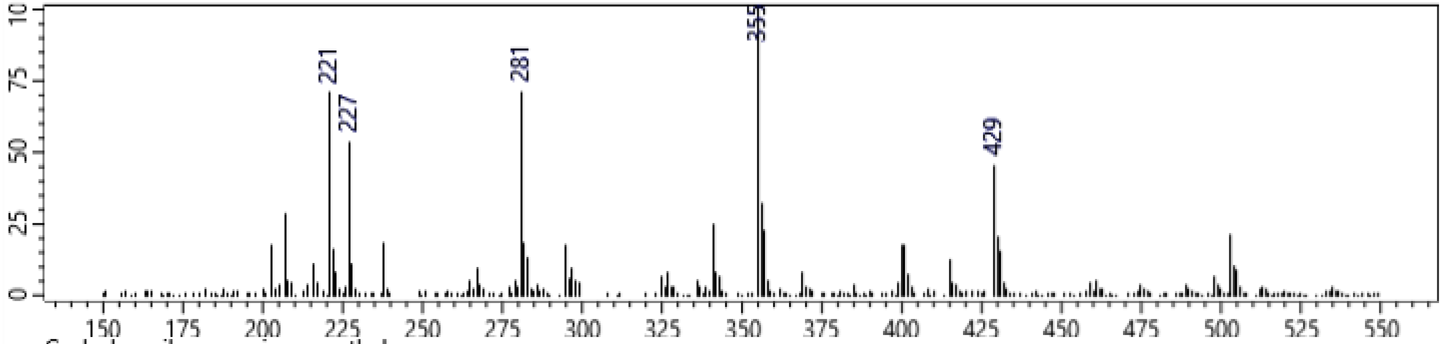


Cyclodecasiloxane, eicosamethyl-

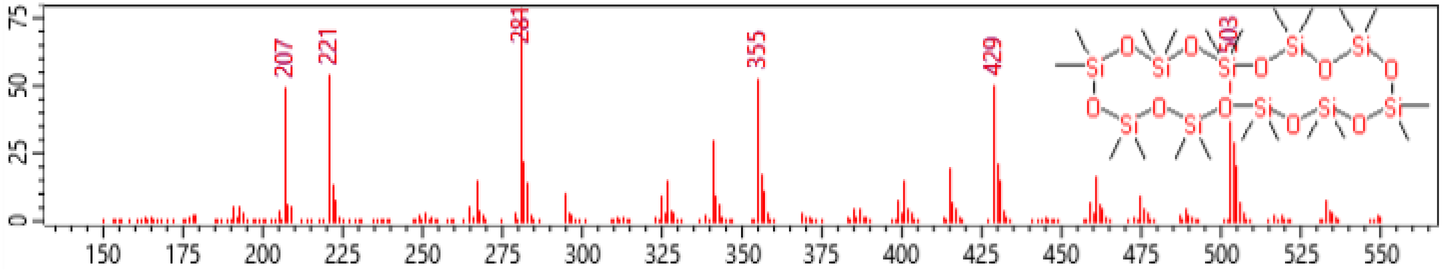

Cyclononasiloxane, octadecamethyl-

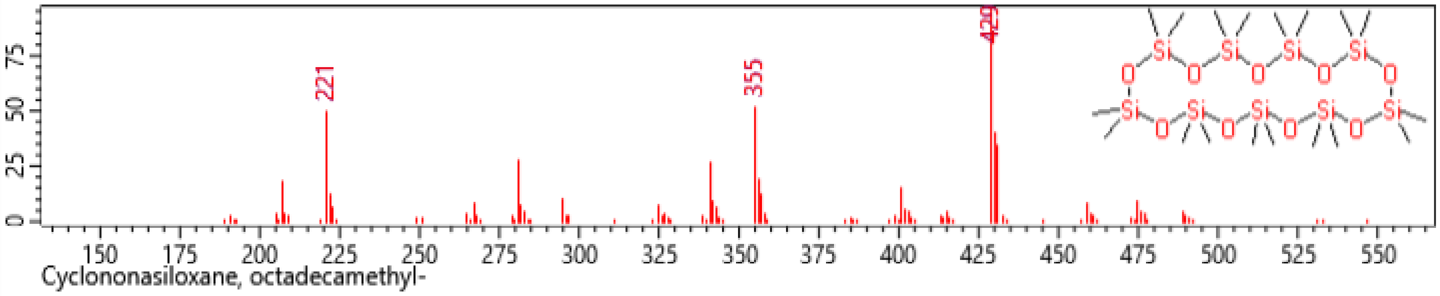


Cyclononasiloxane, octadecamethyl-

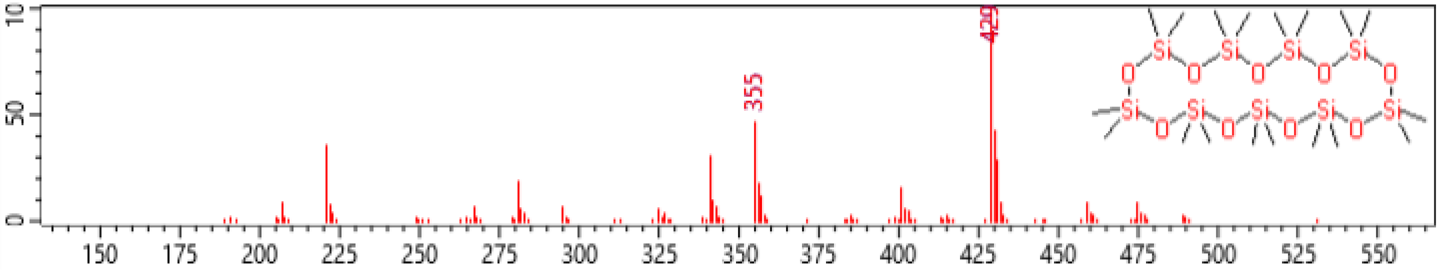


1,1,1,3,3,5,5,7,7,9,9,11,11,13,13,15,15,17,17,19,19,19-docosamethyldecasiloxane

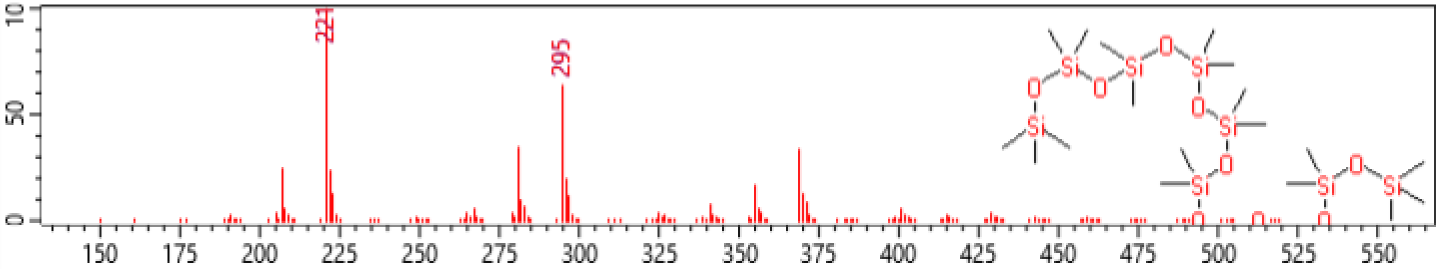


1,1,1,3,3,5,5,7,7,9,9,11,11,13,13,15,15,17,17,17-icosamethylnonasiloxane

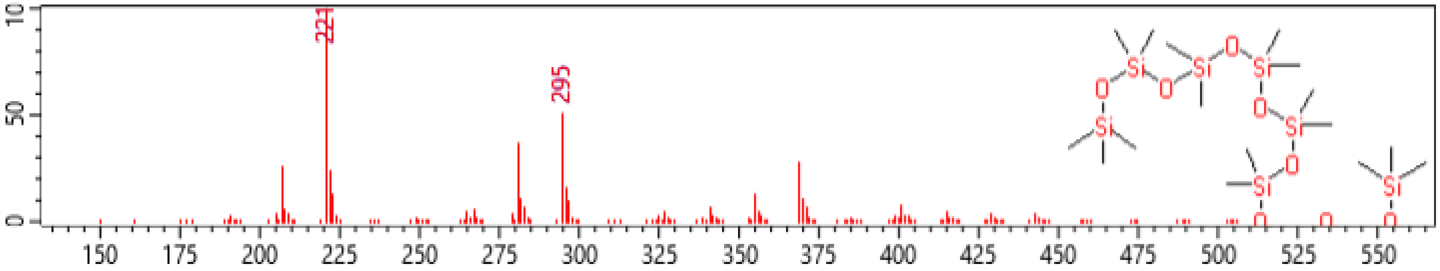


**4H-3,8a-Methanofuro[2',3':6,7]azonino[5,4-b]indol-14-one, 13-acetyl-2,3,3a,5,6,7,8,13,13a,13b-decahydro-11,12-**

Peak: 21.64 - 21.67 min (scan #4086 - #4094) Subtracted noise: 21.61 - 21.64 min (scan #4077 - #4084), 21.70 - 21.73 min (scan #41

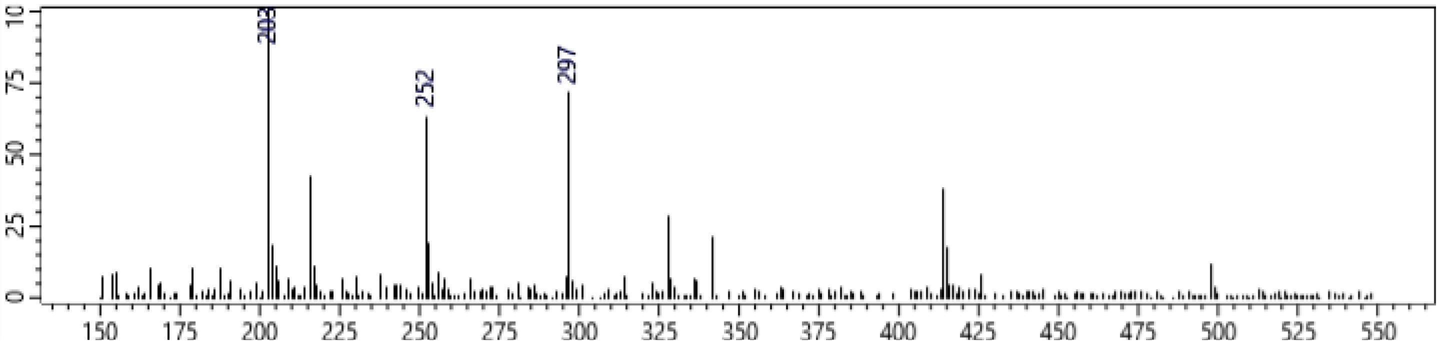

4H-3,8a-Methanofuro[2',3':6,7]azonino[5,4-b]indol-14-one, 13-acetyl-2,3,3a,5,6,7,8,13,13a,13b-decahydro-11,12-dimethoxy-2,6-dime

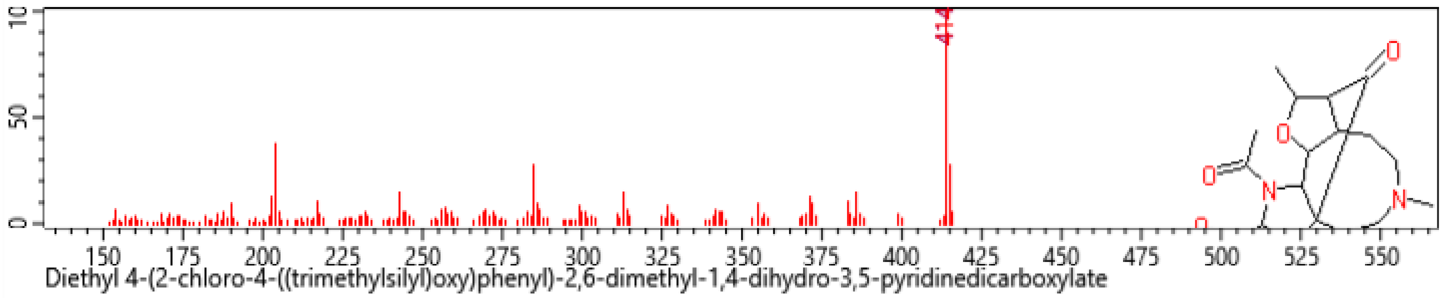


Diethyl 4-(2-chloro-4-((trimethylsilyl)oxy)phenyl)-2,6-dimethyl-1,4-dihydro-3,5-pyridinedicarboxylate

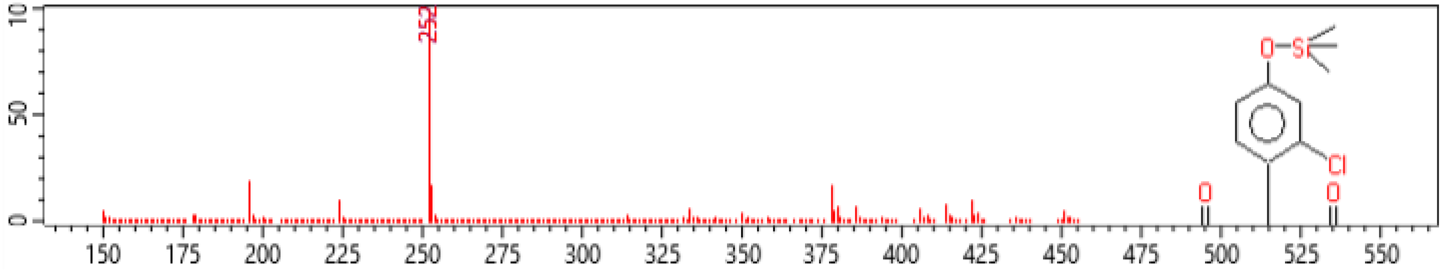


(3,4-Dimethyl-5-oxo-2,5-dihydro-1H-pyrrol-2-yl)-[4,4-dimethyl-5-(2,3,3-trimethyl-5-methylthio-3,4-dihydro-2H-pyrrol-2-ylmethylen

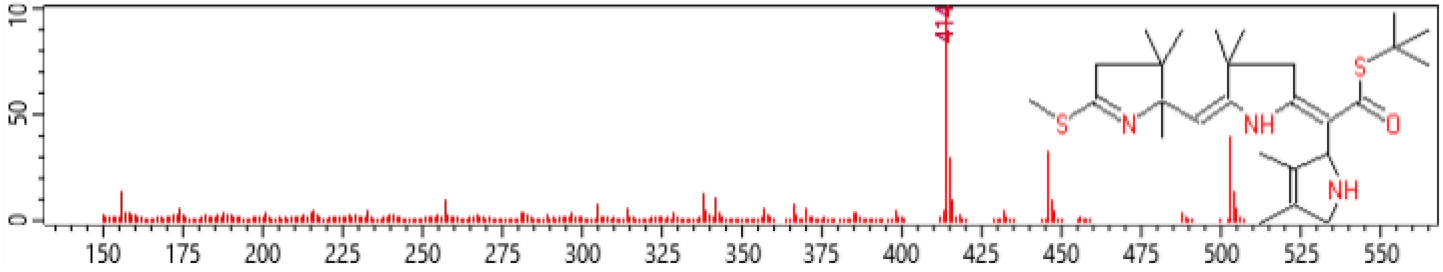


2-{[(2-Bromophenoxy)acetyl]amino}benzoic acid, 2TMS derivative

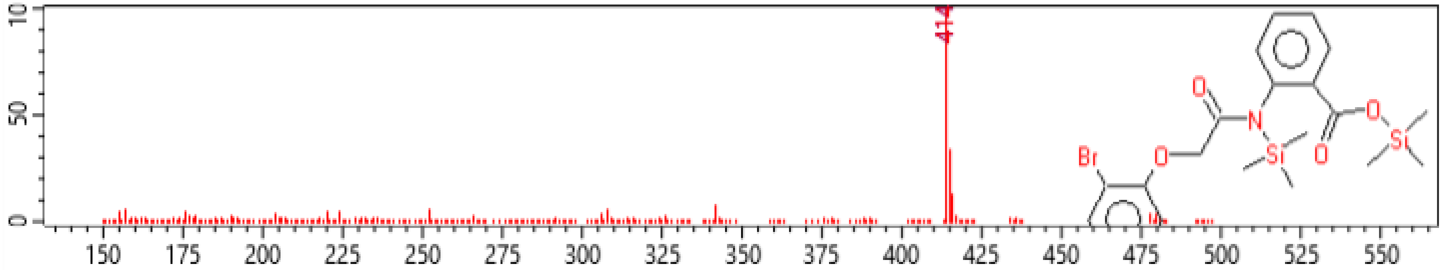


Interiotherin B

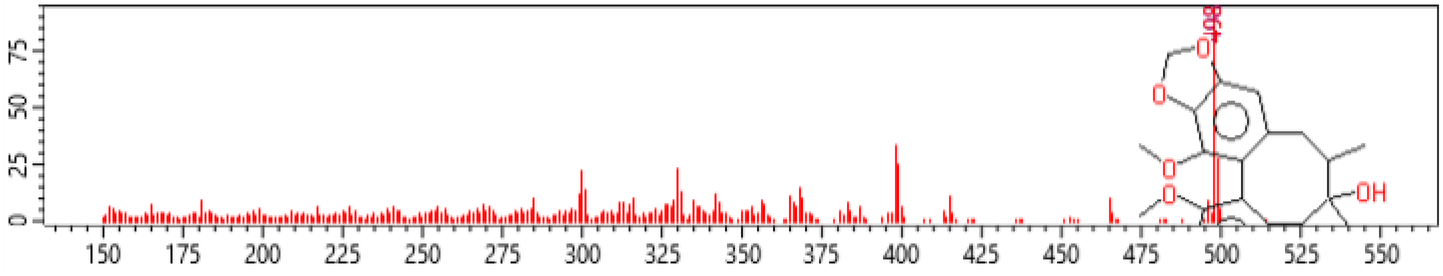

**Bis(2-ethylhexyl) phthalate**

Peak: 21.93 - 22.02 min (scan #4167 - #4191) Subtracted noise: 21.79 - 21.88 min (scan #4128 - #4152), 22.07 - 22.15 min (scan #42[illegible]

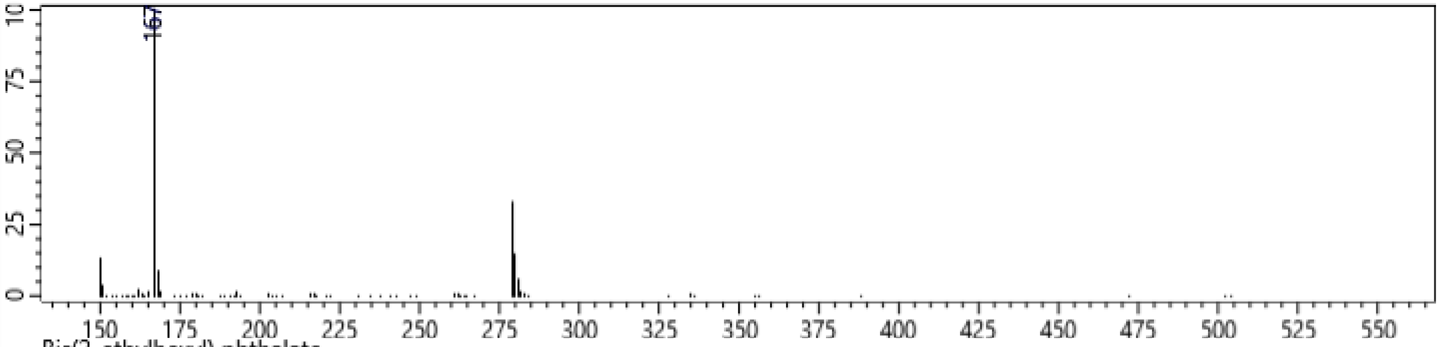


Bis(2-ethylhexyl) phthalate

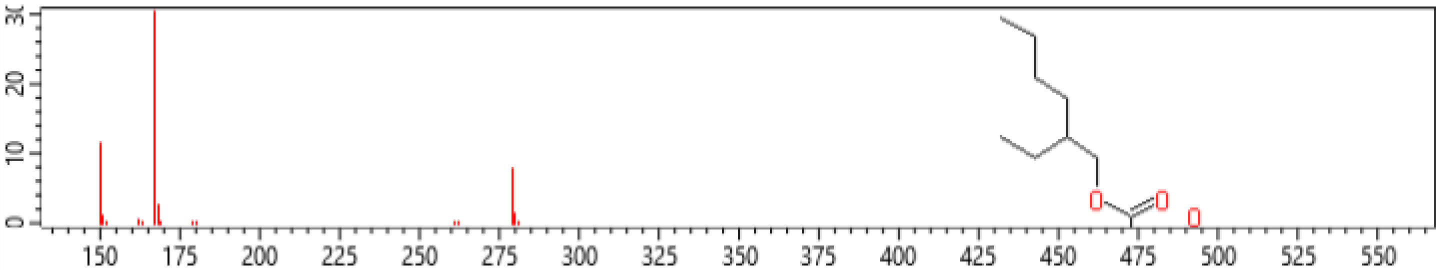


Bis(2-ethylhexyl) phthalate

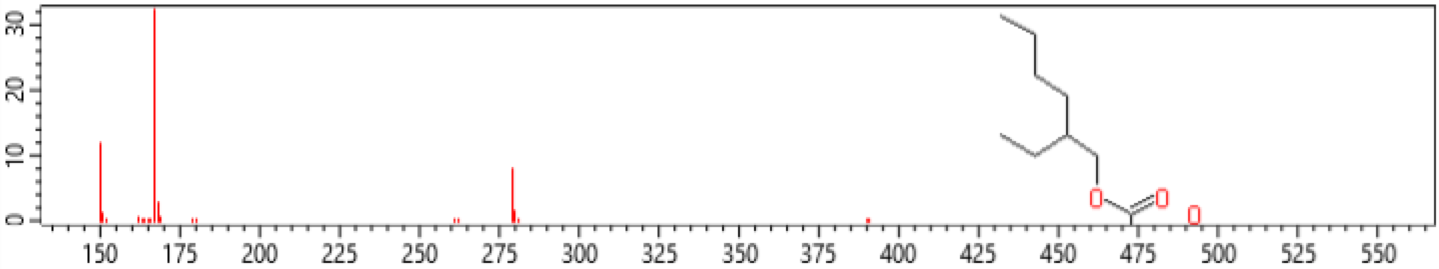


Bis(2-ethylhexyl) phthalate

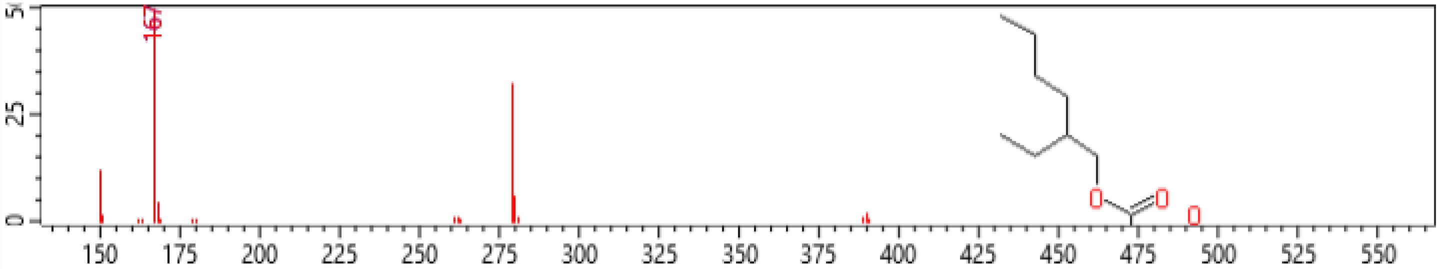


Bis(2-ethylhexyl) phthalate

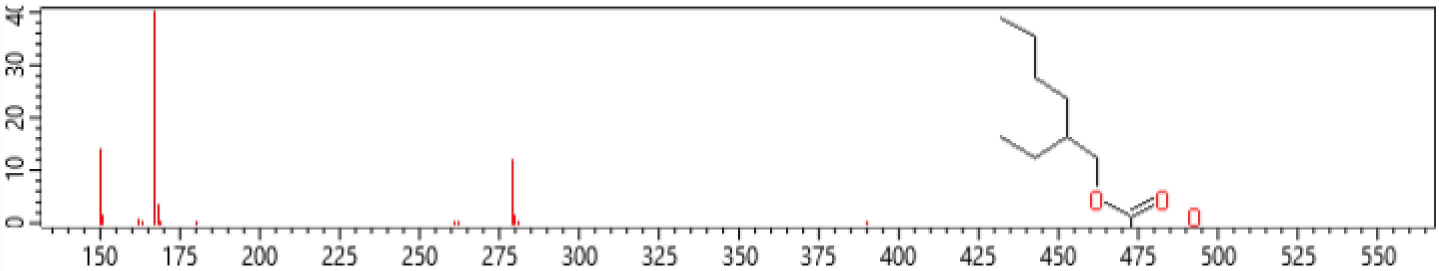

Phthalic acid, di(2-propylpentyl) ester

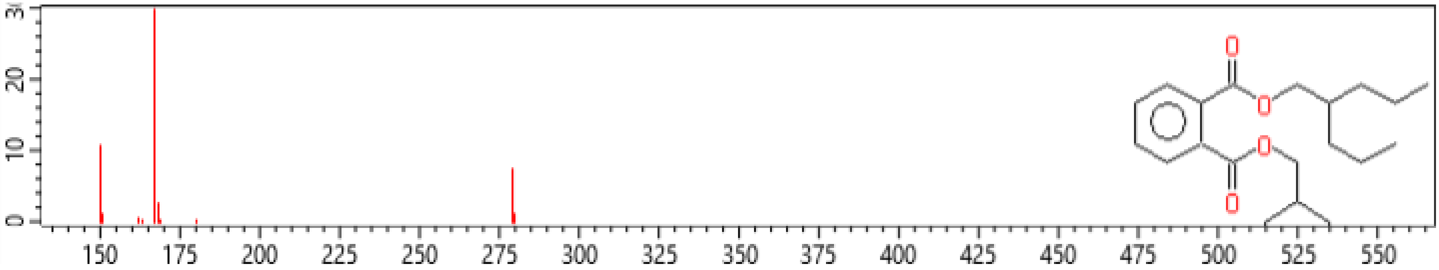


**2-Palmitoylglycerol, 2TMS derivative**

Peak: 22.60 - 22.69 min (scan #4353 - #4379) Subtracted noise: 22.45 - 22.55 min (scan #4312 - #4338), 22.75 - 22.84 min (scan #43

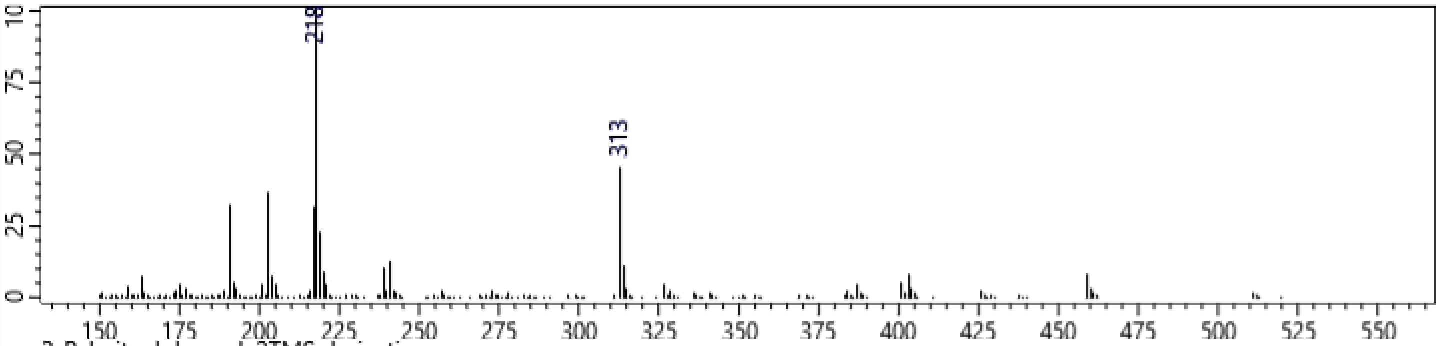


2-Palmitoylglycerol, 2TMS derivative

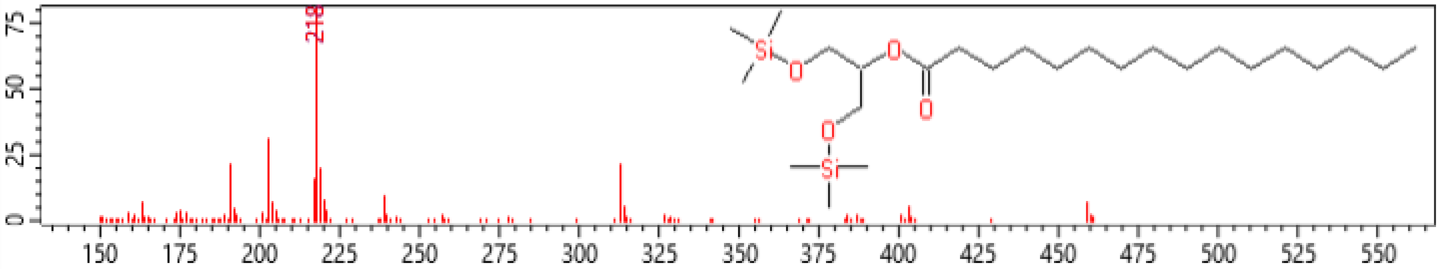


2-Palmitoylglycerol, 2TMS derivative

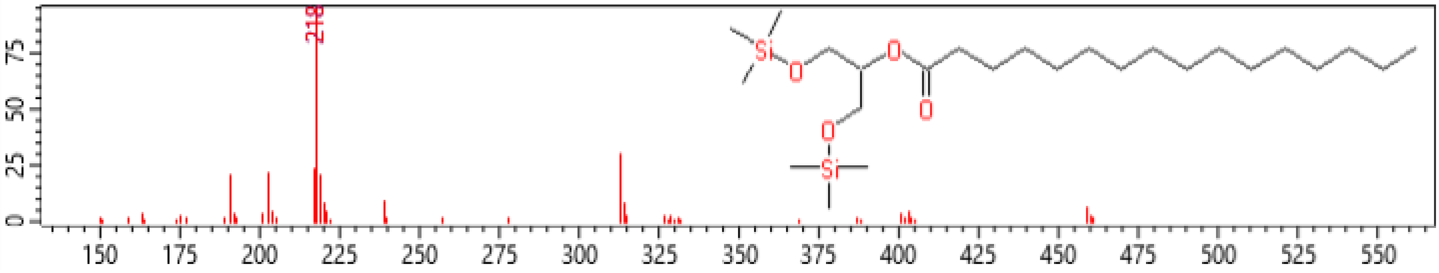


2-Palmitoylglycerol, 2TMS derivative

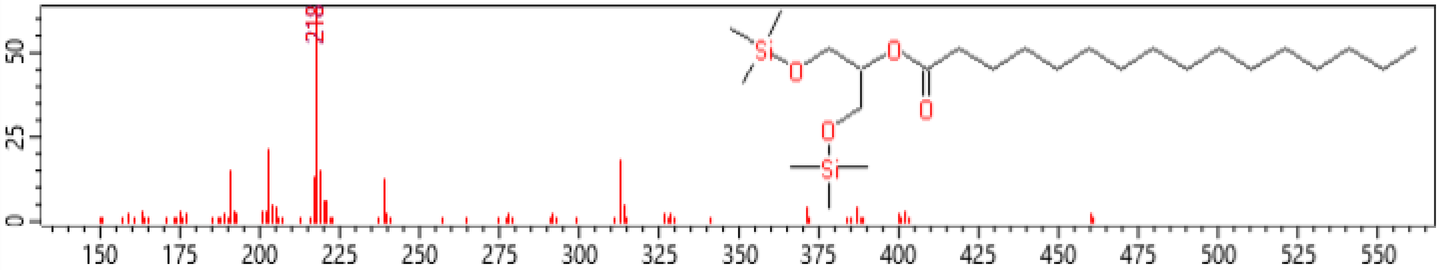

Acetin, bis-1,3-trimethylsilyl ether

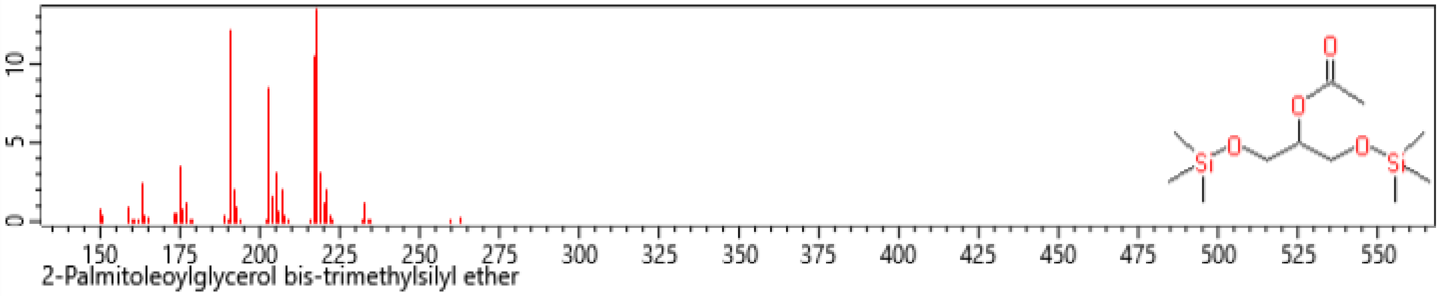

2-Palmitoleoylglycerol bis-trimethylsilyl ether

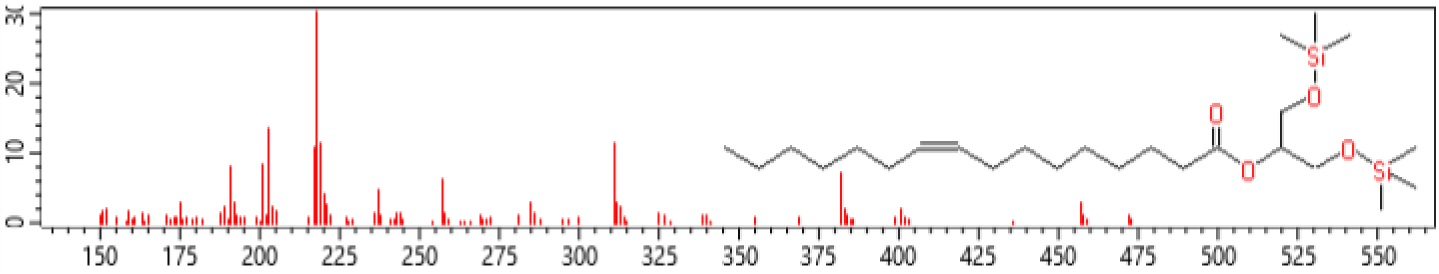

**Sulpiride**

Peak: 22.88 - 22.99 min (scan #4431 - #4461) Subtracted noise: 22.72 - 22.84 min (scan #4388 - #4419), 23.06 - 23.17 min (scan #44

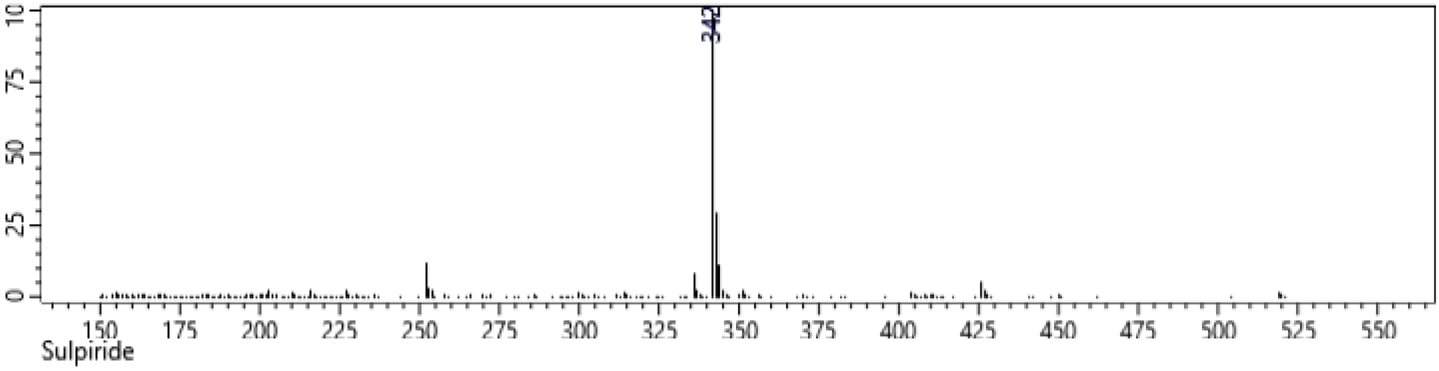


Sulpiride

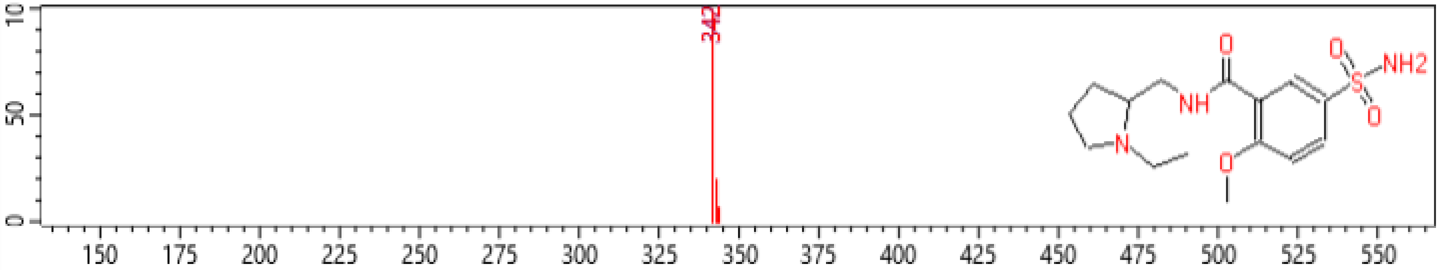


Sulpiride

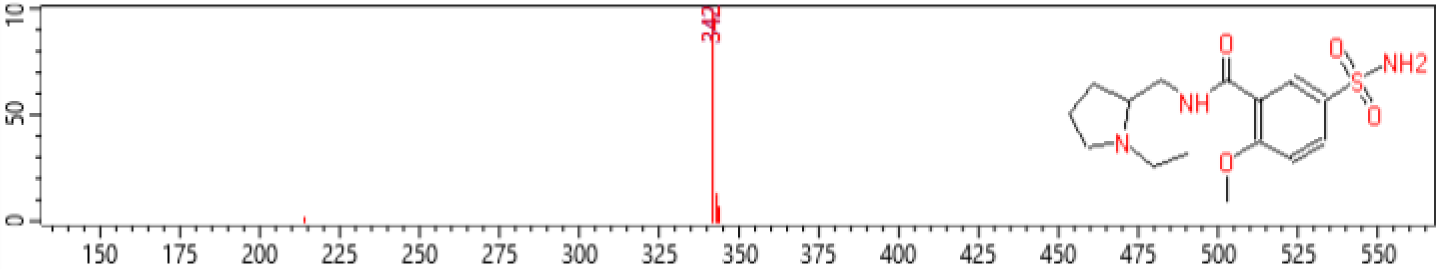

Sulpiride

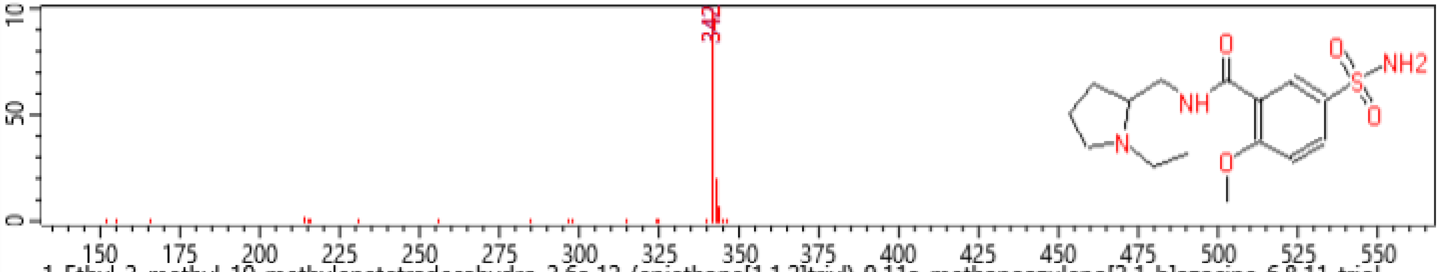


1-Ethyl-3-methyl-10-methylenetetradecahydro-3,6a,12-(epiethane[1,1,2]triyl)-9,11a-methanoazuleno[2,1-b]azocine-6,8,11-triol

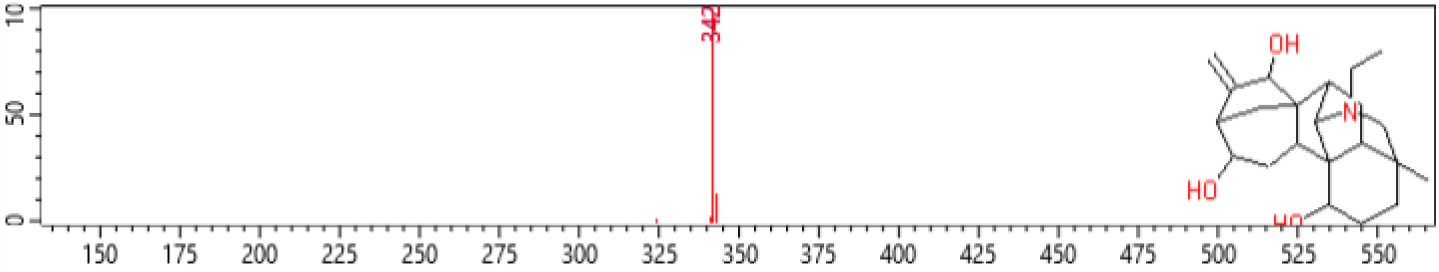


Posaconazole

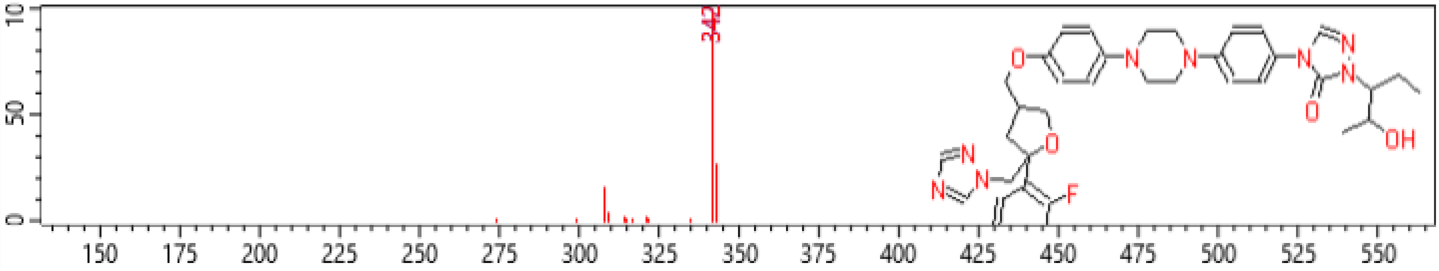


**Coproporphyrin I**

Peak: 23.22 - 23.30 min (scan #4526 - #4548) Subtracted noise: 23.09 - 23.17 min (scan #4491 - #4513), 23.34 - 23.41 min (scan #45

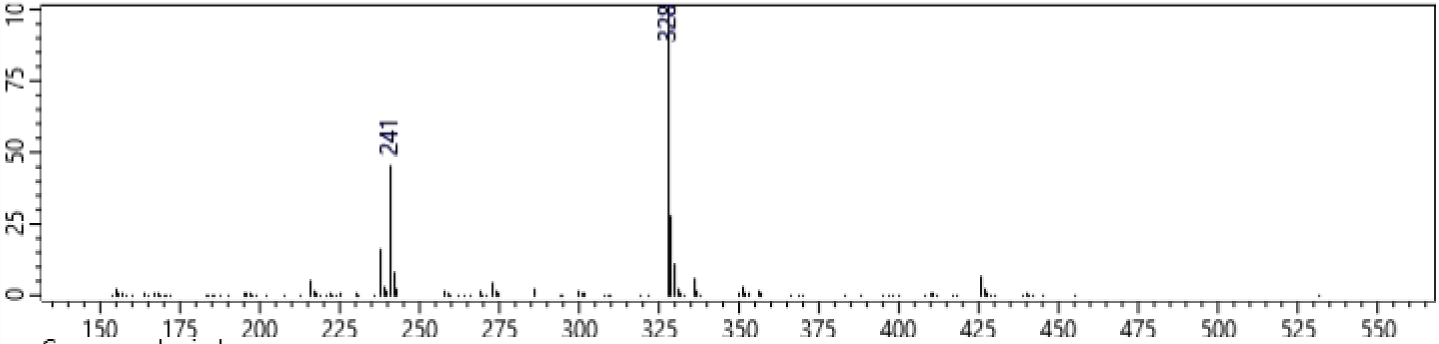


Coproporphyrin I

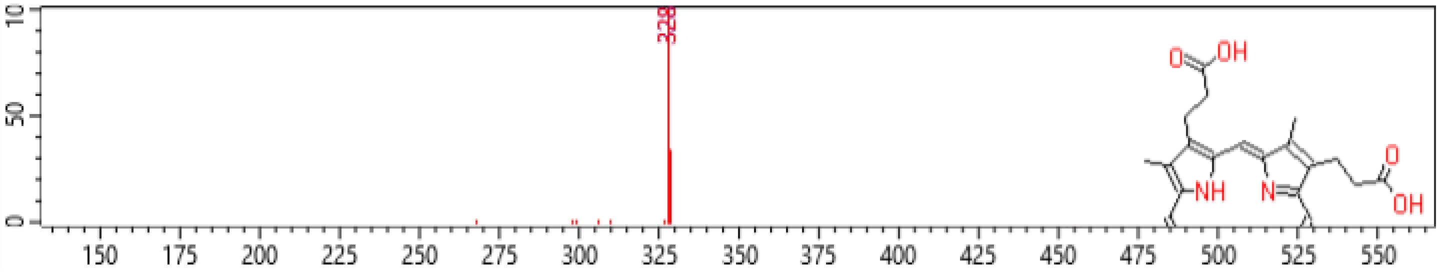

Coproporphyrin I

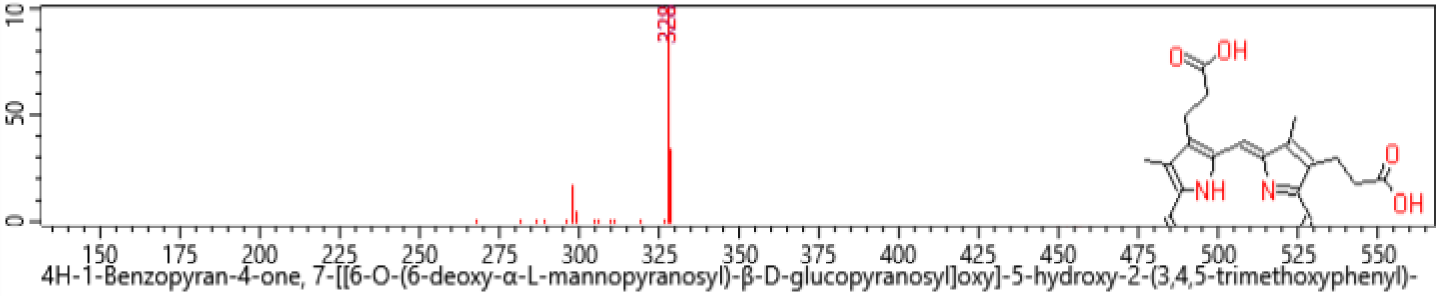


4H-1-Benzopyran-4-one, 7-[[6-O-(6-deoxy-α-L-mannopyranosyl)-β-D-glucopyranosyl]oxy]-5-hydroxy-2-(3,4,5-trimethoxyphenyl)-

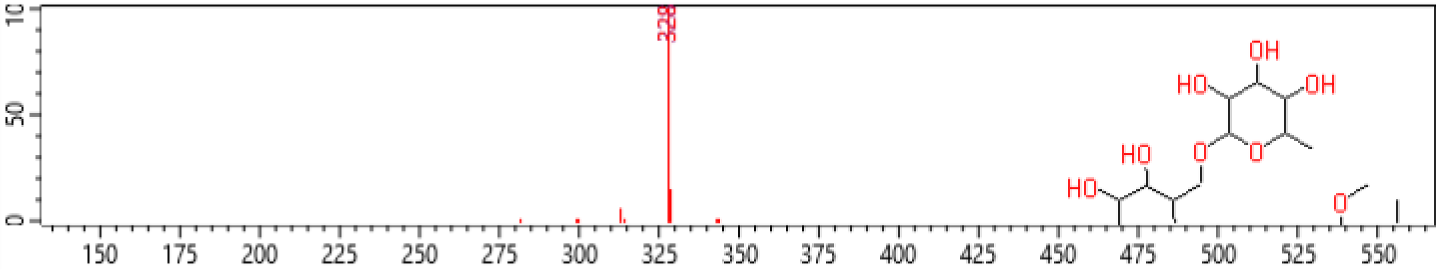


N-(1-Naphthyl)-m-nitrobenzenesulfonamide

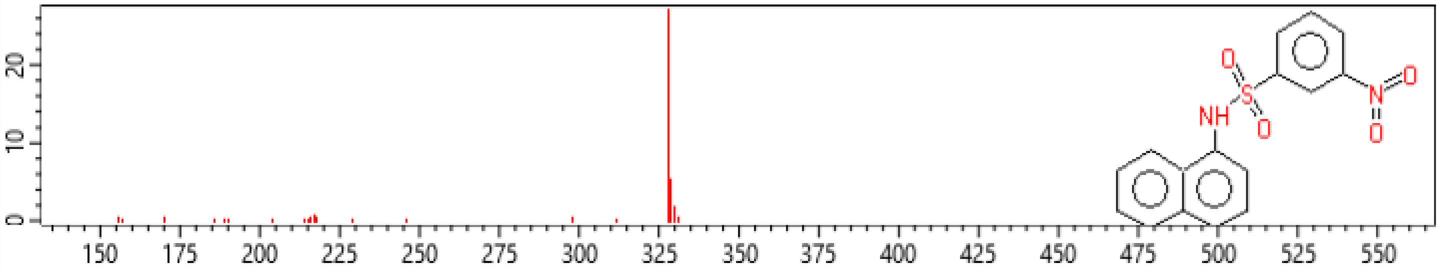

5-Hydroxy-3-(5-hydroxy-2,4-dimethoxyphenyl)-6-methoxy-4-oxo-4H-chromen-7-yl hexopyranoside

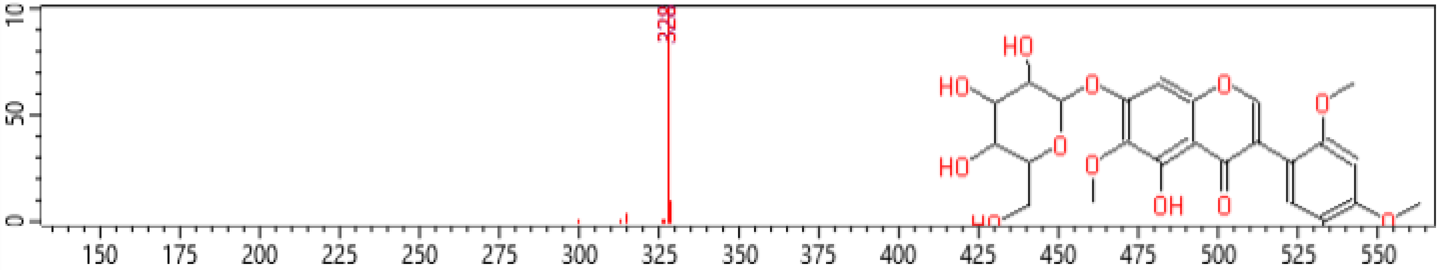


**1-Monopalmitin, 2TMS derivative**

Peak: 23.53 - 23.70 min (scan #4613 - #4661) Subtracted noise: 23.16 - 23.33 min (scan #4509 - #4557), 23.69 - 23.86 min (scan #46

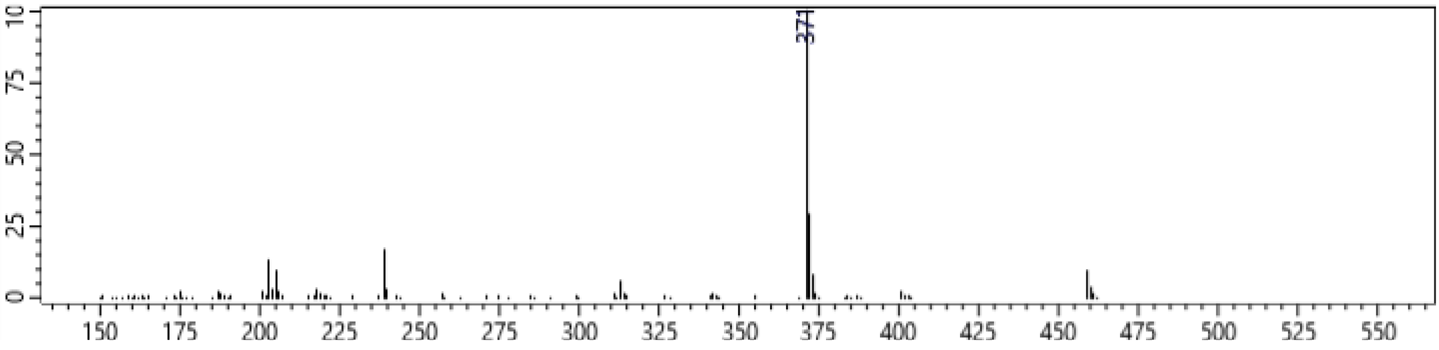

1-Monopalmitin, 2TMS derivative

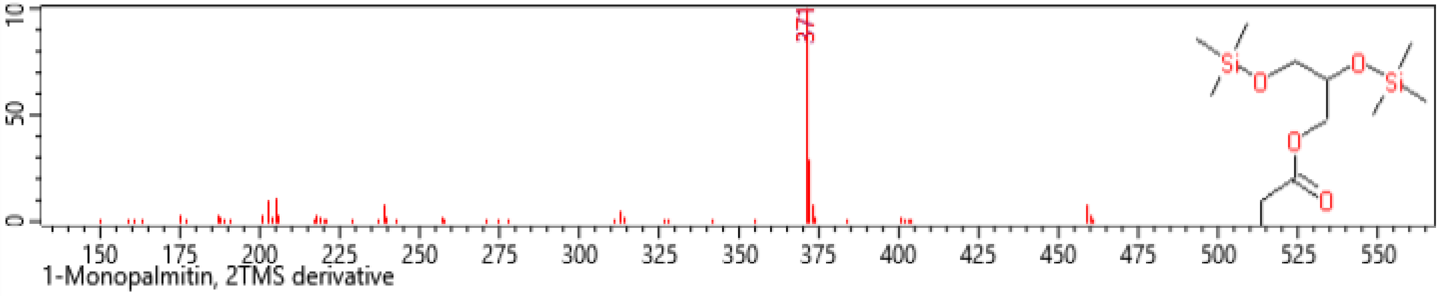


1-Monopalmitin, 2TMS derivative

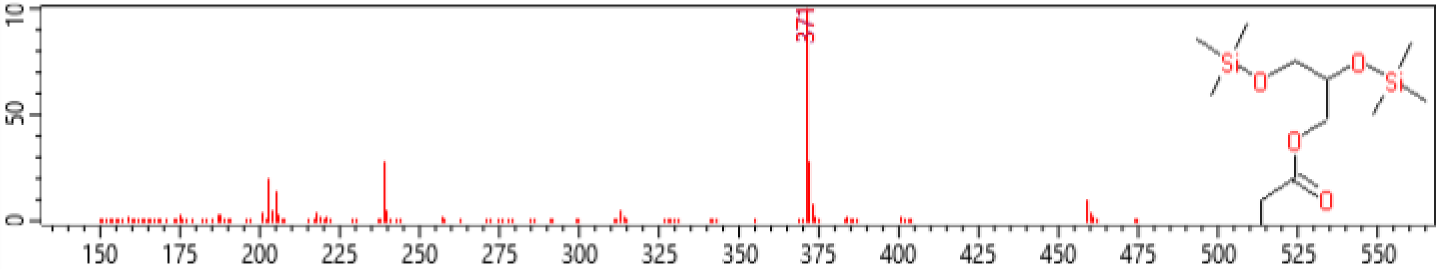


1-Monopalmitin, 2TMS derivative

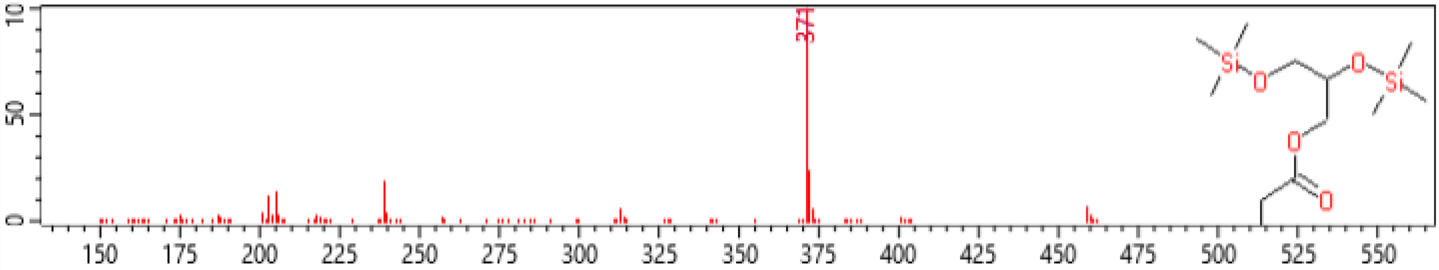


1-Monopalmitin, 2TMS derivative

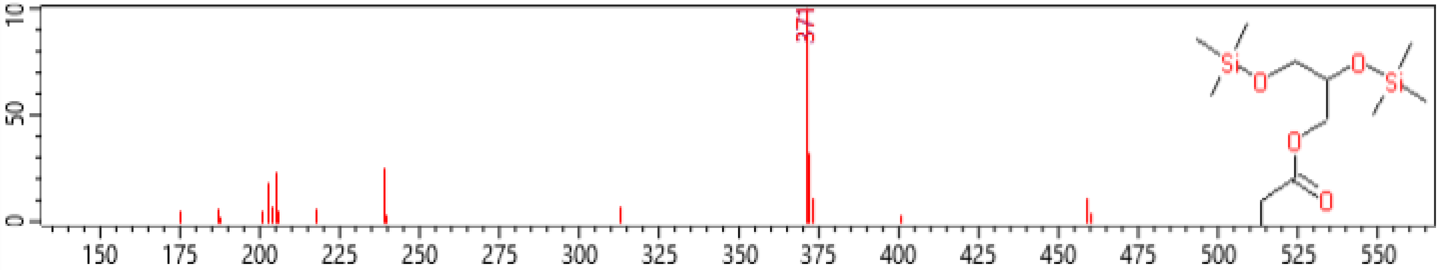


1-Monopalmitin, 2TMS derivative

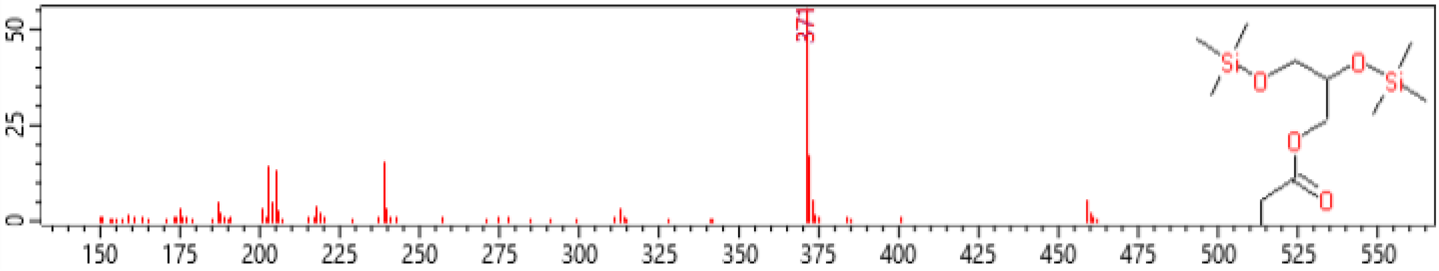

**Pyrazolidinetrione, phenyl-, 4-(phenylhydrazone)**
Peak: 23.77 - 23.87 min (scan #4679 - #4707) Subtracted noise: 23.62 - 23.73 min (scan #4639 - #4668), 23.95 - 24.05 min (scan #47

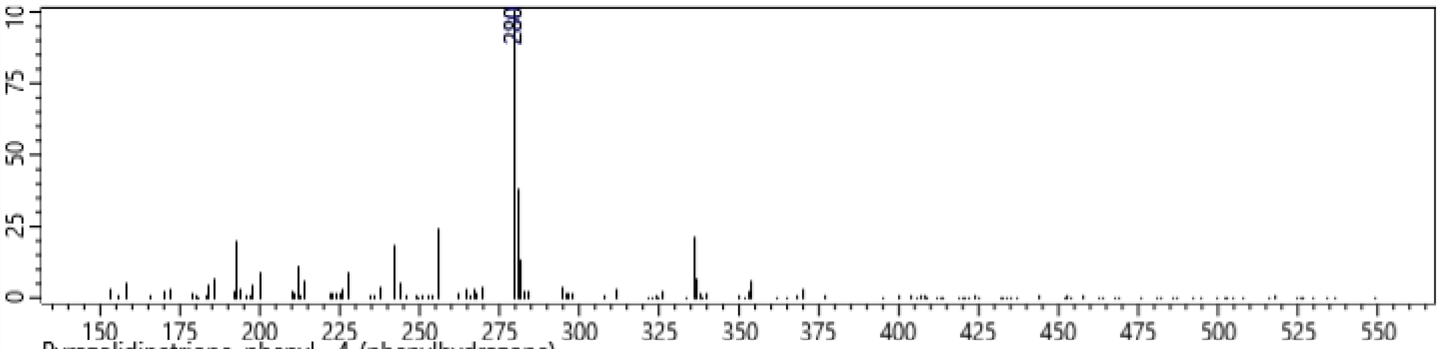


Pyrazolidinetrione, phenyl-, 4-(phenylhydrazone)

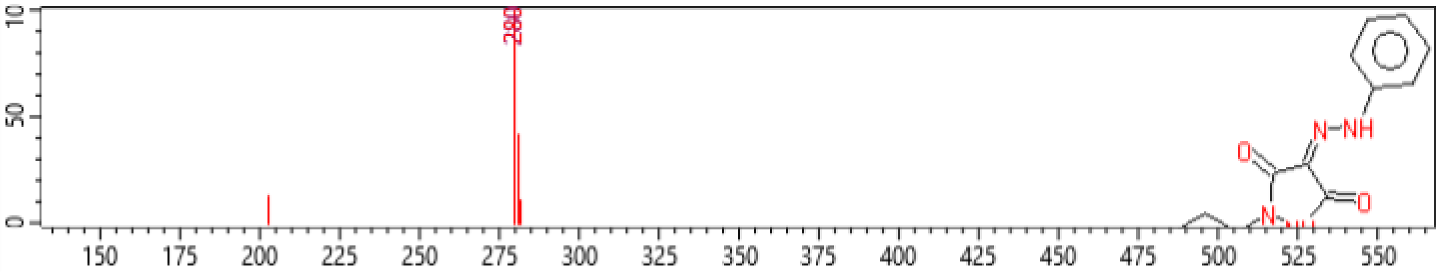


Psoralidin

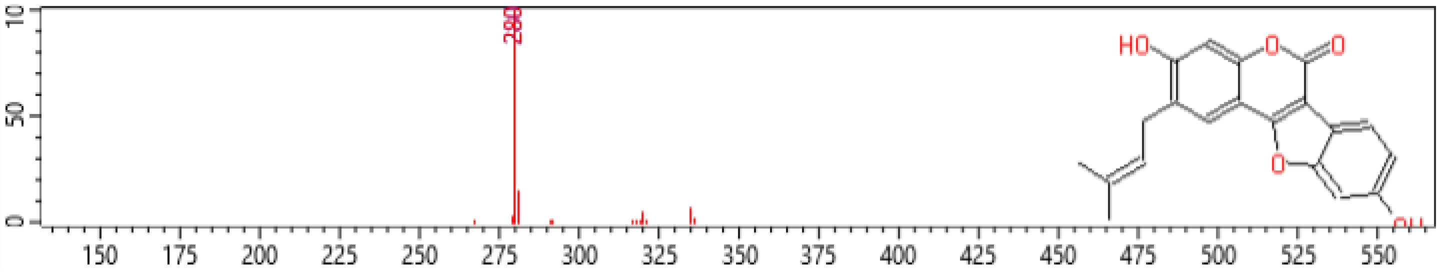


7-[2-(Ethoxycarbonyl)-3α,5β-dimethoxycyclopentyl-1]-heptanoic acid, ethyl ester

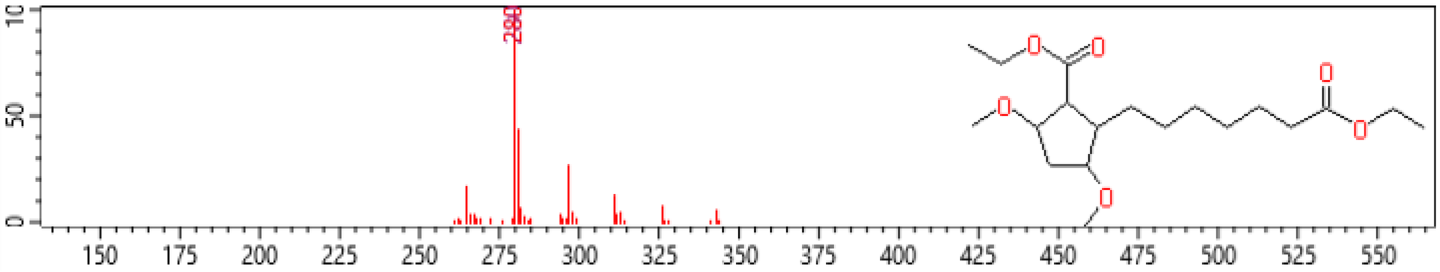


2-Benzamido-4-phenylthiazole

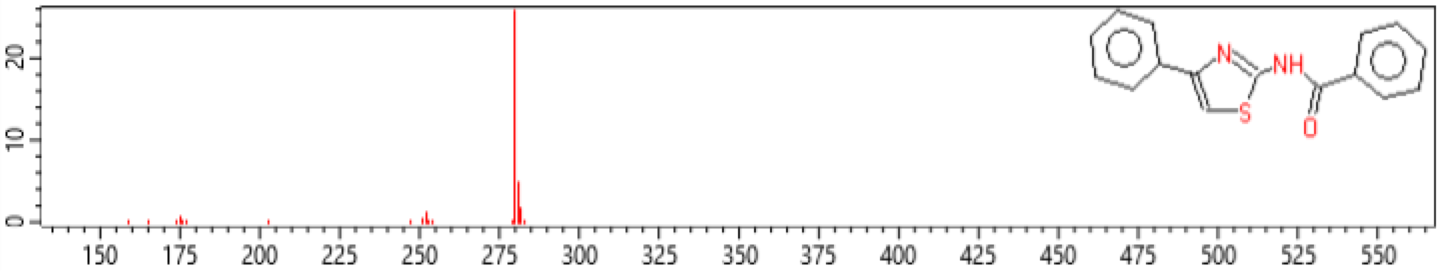

Pyrido[2,3-b]isoquinolino[3,4-d]furan-5(6H)-one, 7,9-dimethyl-

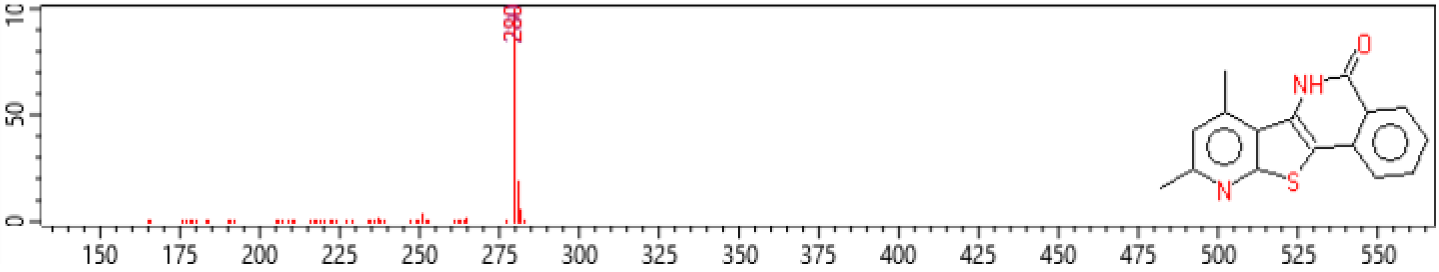


**Cyclodecasiloxane, eicosamethyl-**

Peak: 25.24 - 25.28 min (scan #5089 - #5101) Subtracted noise: 25.16 - 25.20 min (scan #5067 - #5079), 25.30 - 25.35 min (scan #51

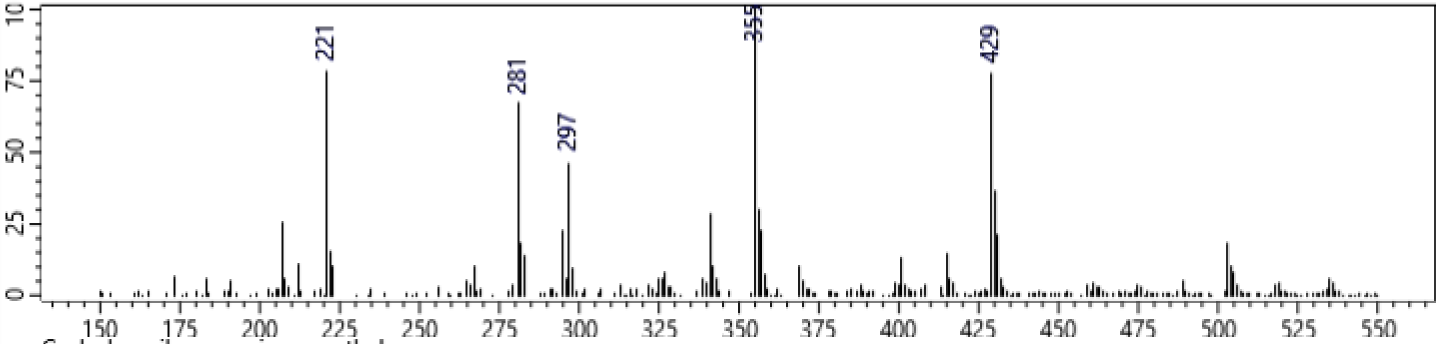


Cyclodecasiloxane, eicosamethyl-

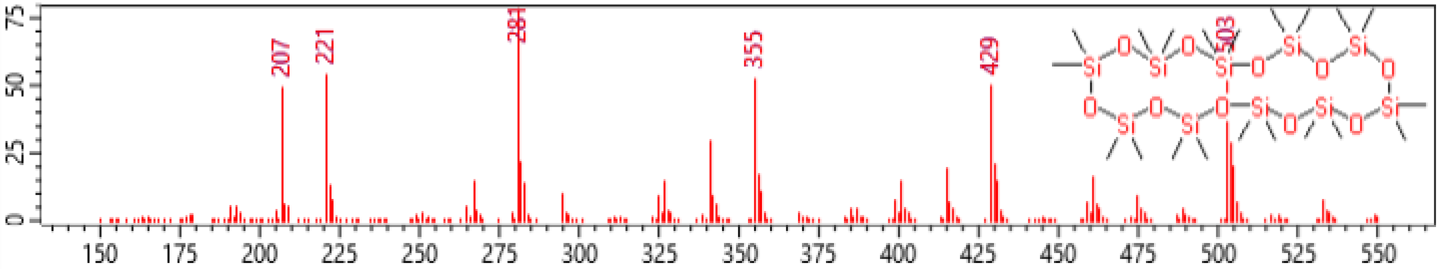


Cyclononasiloxane, octadecamethyl-

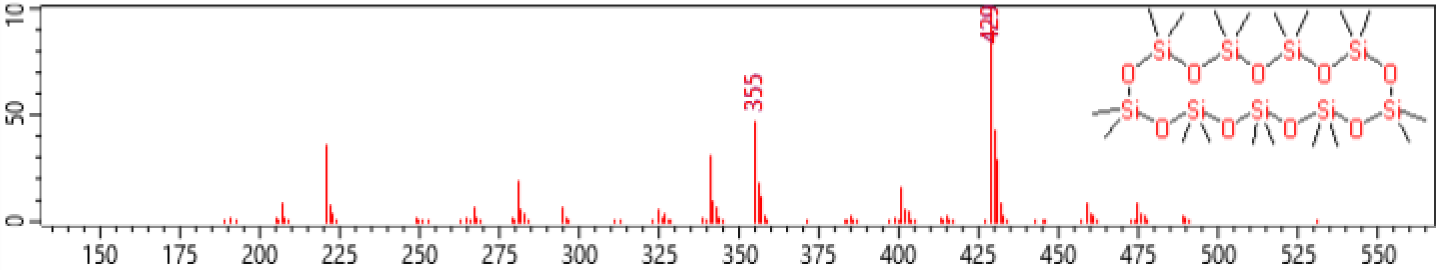


Cyclononasiloxane, octadecamethyl-

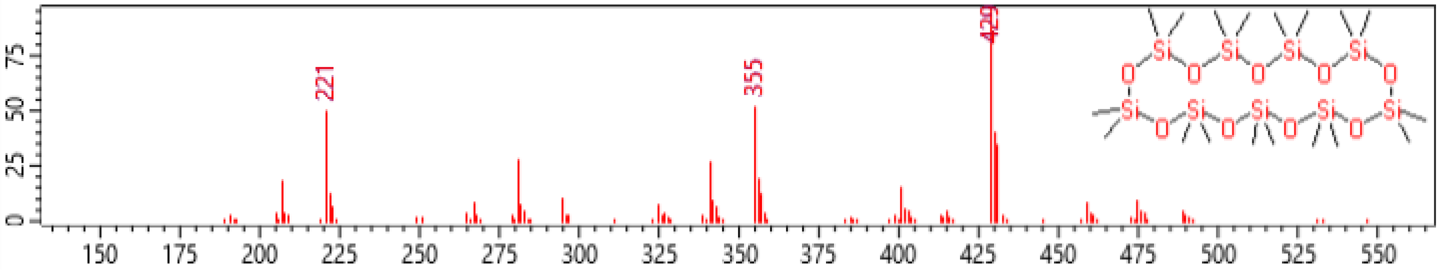

Tetracosamethyl-cyclododecasiloxane

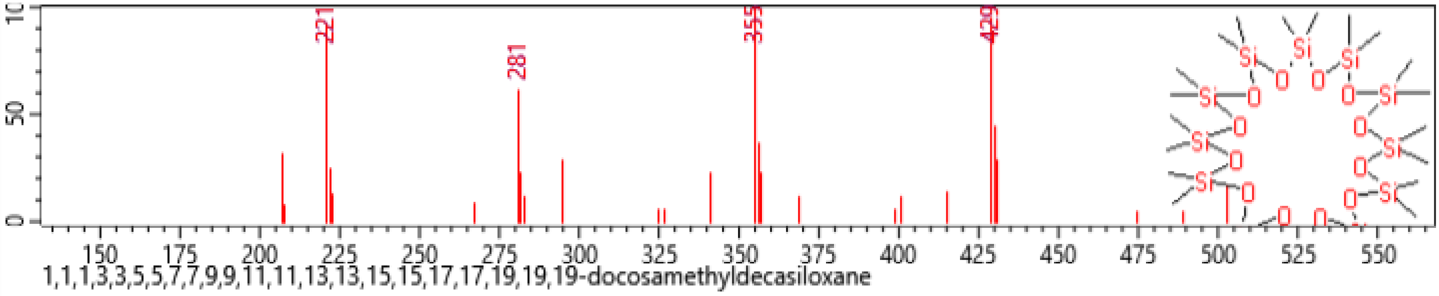


1,1,1,3,3,5,5,7,7,9,9,11,11,13,13,15,15,17,17,19,19,19-docosamethyldecasiloxane

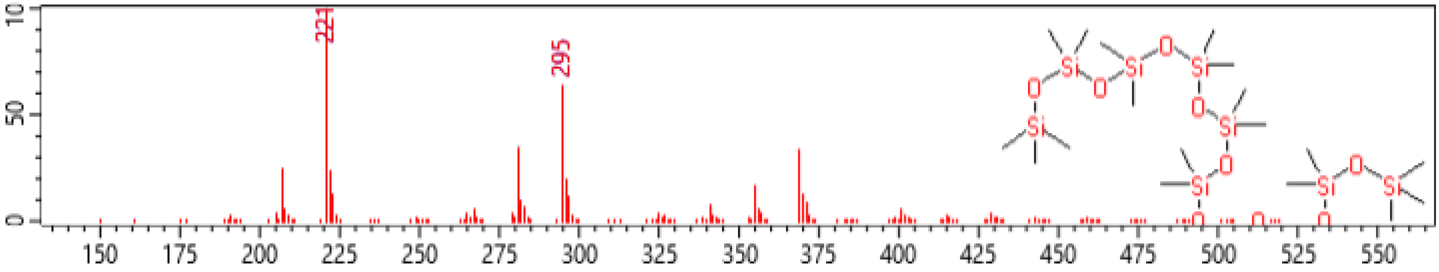


**2,5-Furandione, dihydro-3-isooctadecyl-**

Peak: 25.46 - 25.56 min (scan #5151 - #5179) Subtracted noise: 25.31 - 25.41 min (scan #5110 - #5137), 25.62 - 25.72 min (scan #51

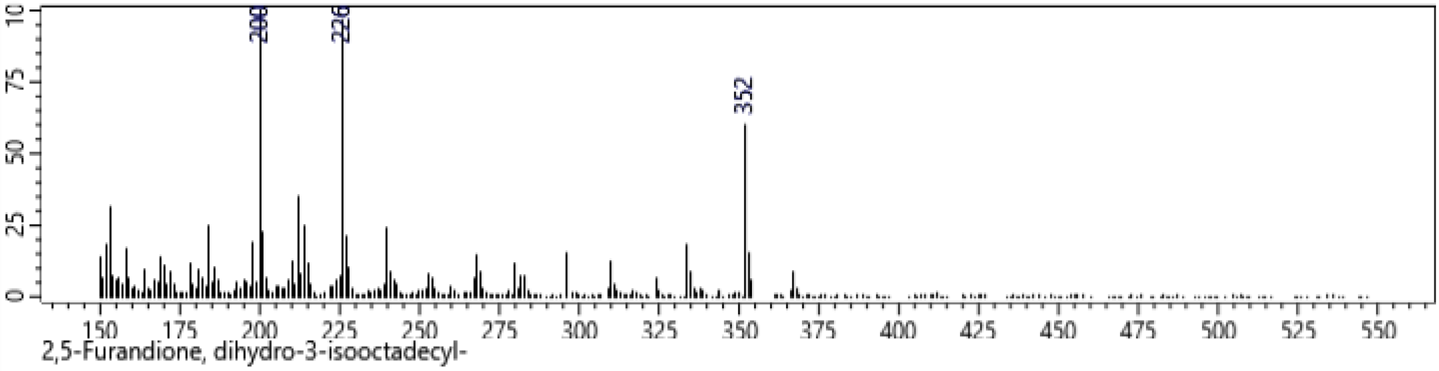


2,5-Furandione, dihydro-3-isooctadecyl-

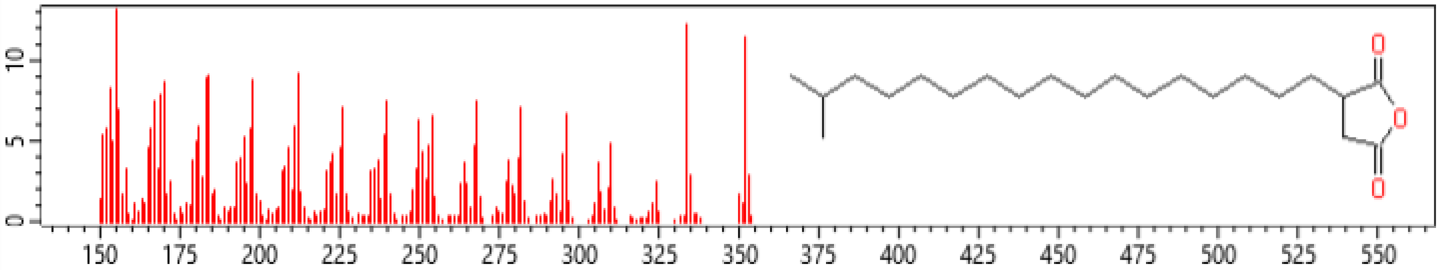

4,5,6,7-Tetrahydro-indoxazen-4-one, 3-undecyl-6-[fluoro-dimethylsilyl]-

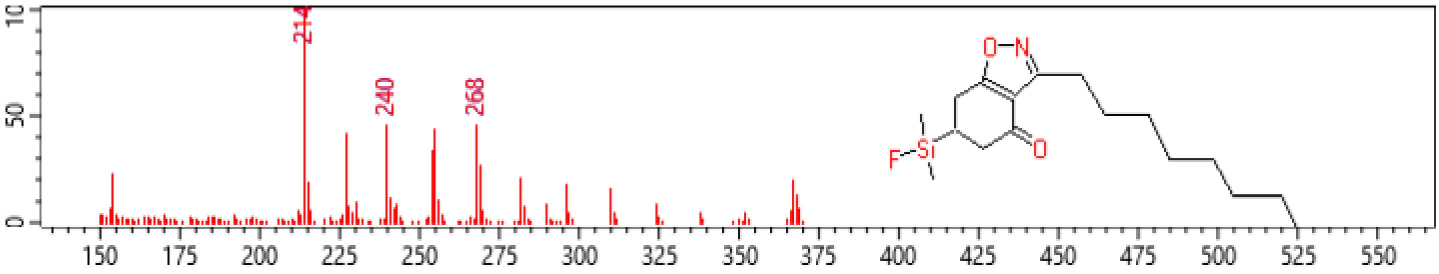

JWH 122 N-(4-pentenyl) analog

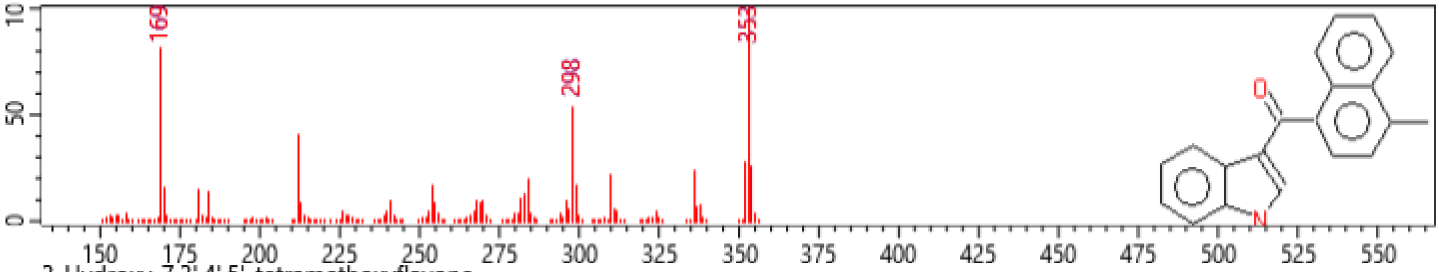


3-Hydroxy-7,3',4',5'-tetramethoxyflavone

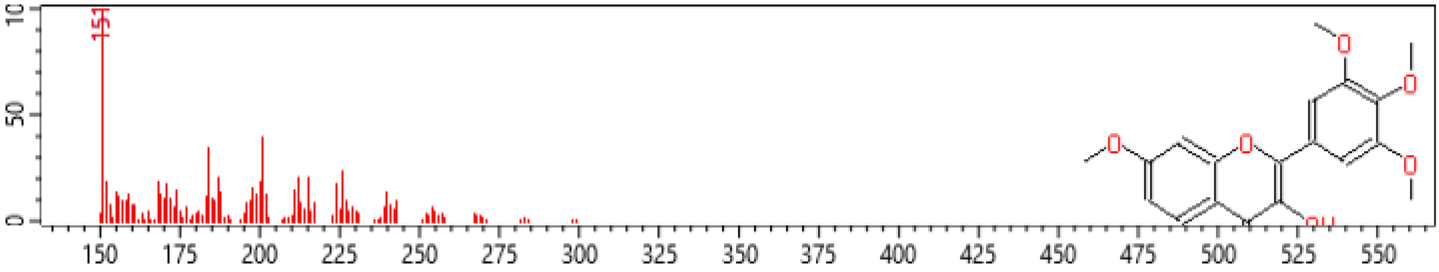


1H,5H-Benzo[ij]quinolizin-5-one, 2,3-dihydro-1,3,3,7,9-pentamethyl-

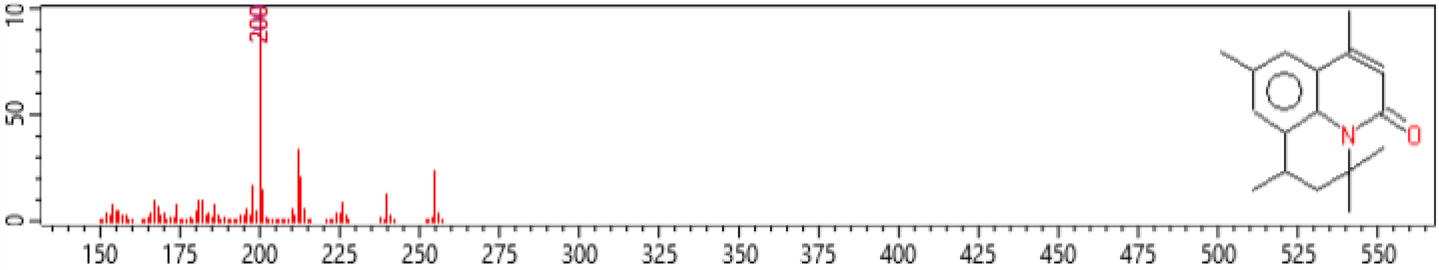


**Gangaleoidin**

Peak: 26.88 - 26.93 min (scan #5547 - #5562) Subtracted noise: 26.80 - 26.85 min (scan #5525 - #5539), 26.97 - 27.02 min (scan #55

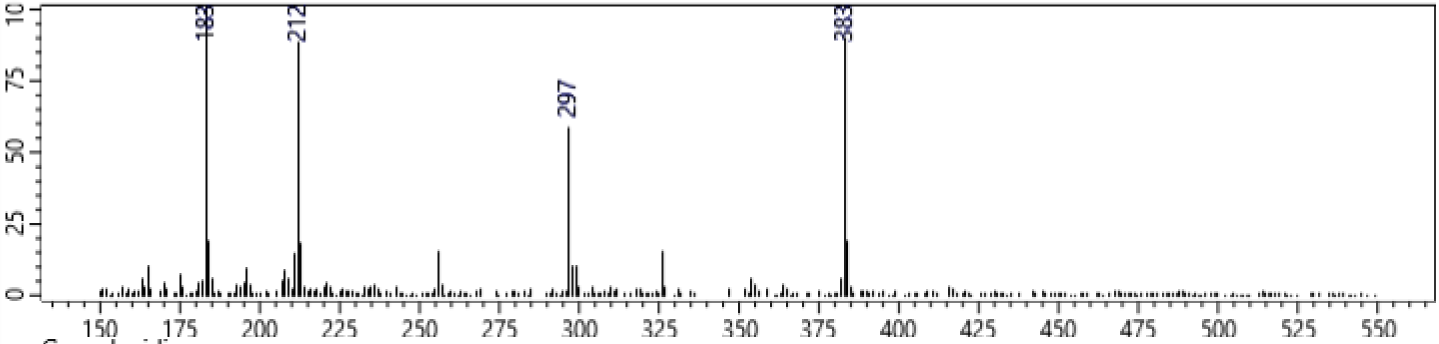


Gangaleoidin

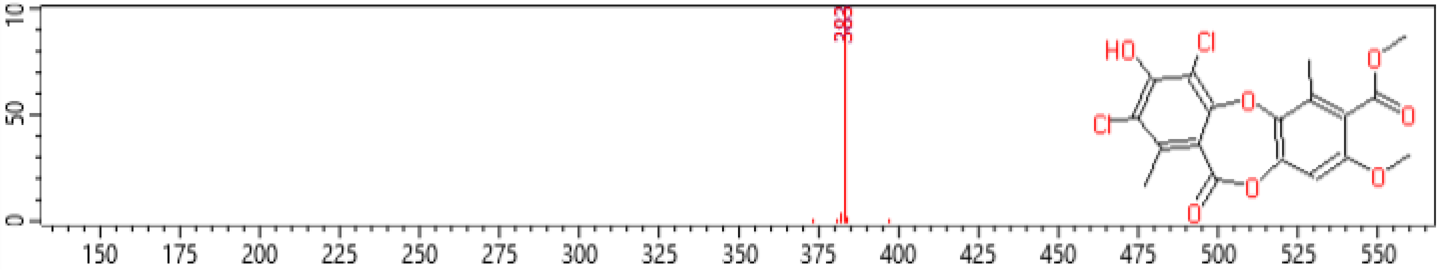

4',6'-Dimethoxy-4-dimethylamino-2'-(2-methylpropyl)oxychalcone (isomer 1)

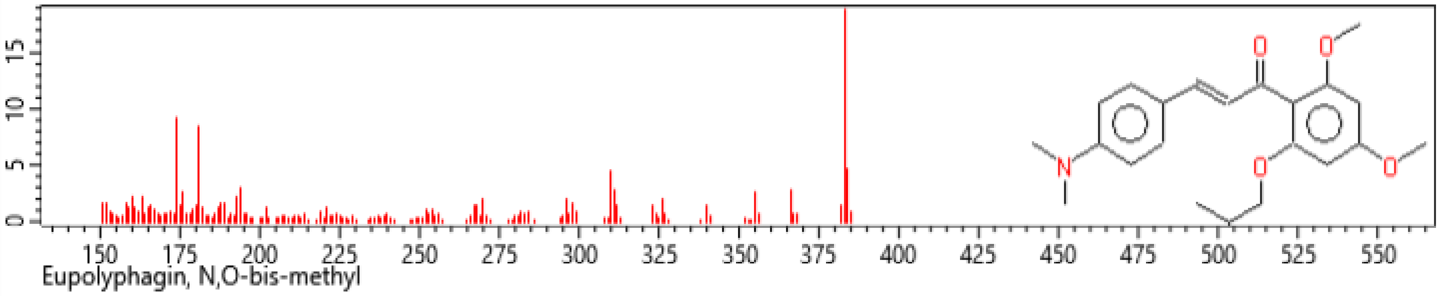

Eupolyphagin, N,O-bis-methyl

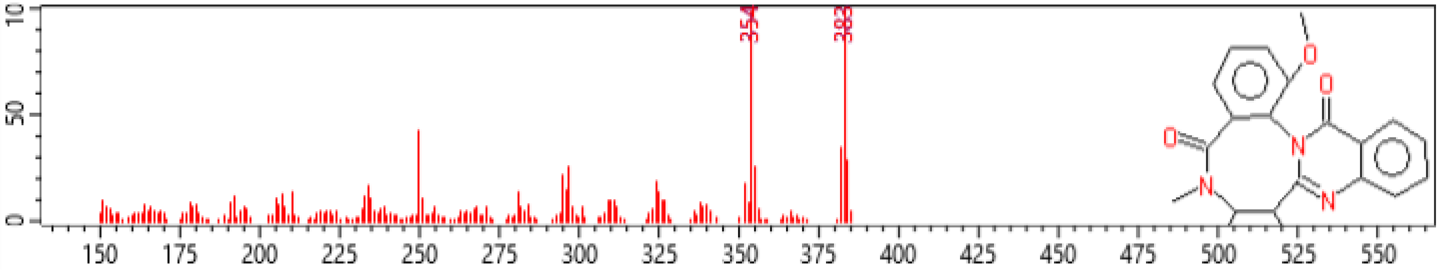


JWH 213

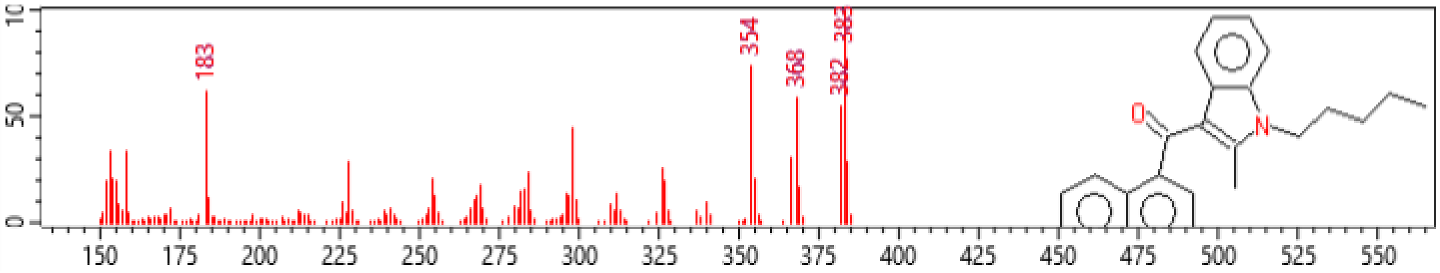


4-Hydroxy-3,5-dimethyl-6-(4-(2-methyl-3-(p-nitrophenyl)-2-propenylidene)tetrahydro-2-furyl)-2-pyranone

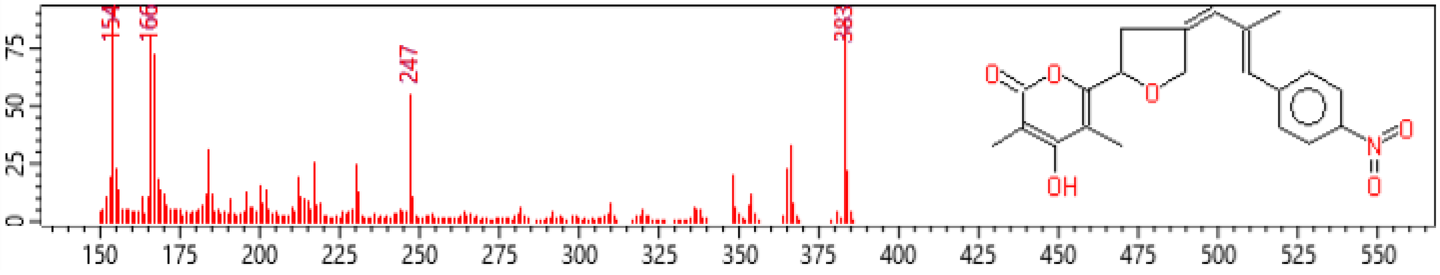


**N-[2-(3,4-Dihydroxyphenyl)ethyl]-1-adamantanecarboxamide, 2TMS**

Peak: 28.14 - 28.50 min (scan #5899 - #6001) Subtracted noise: 27.50 - 27.87 min (scan #5722 - #5824), 28.62 - 28.98 min (scan #60

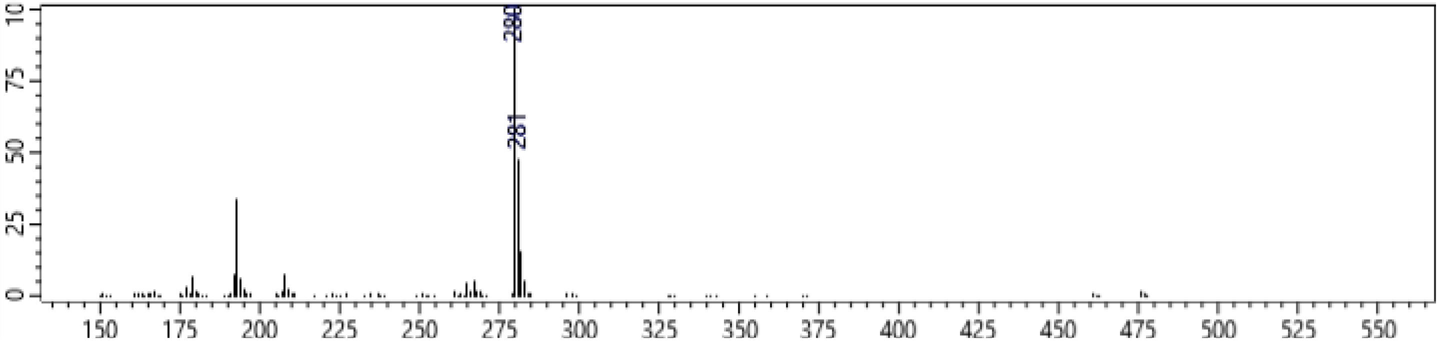

N-[2-(3,4-Dihydroxyphenyl)ethyl]-1-adamantanecarboxamide, 2TMS

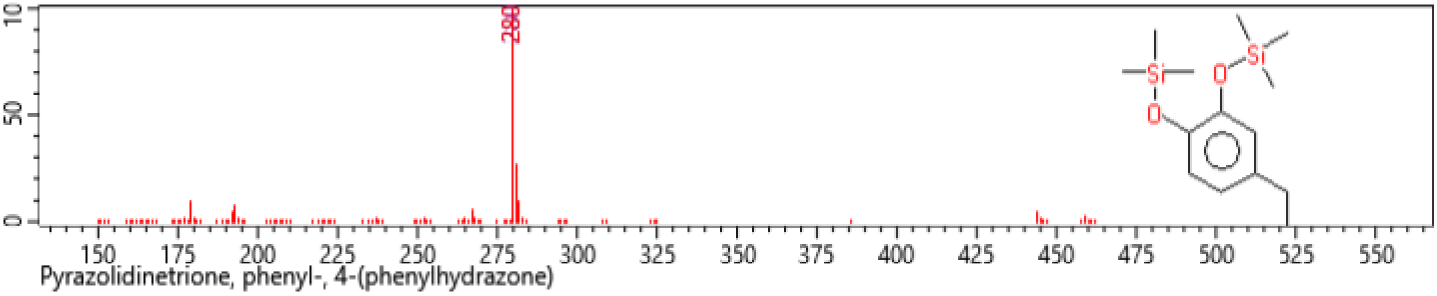


Pyrazolidinetrione, phenyl-, 4-(phenylhydrazone)

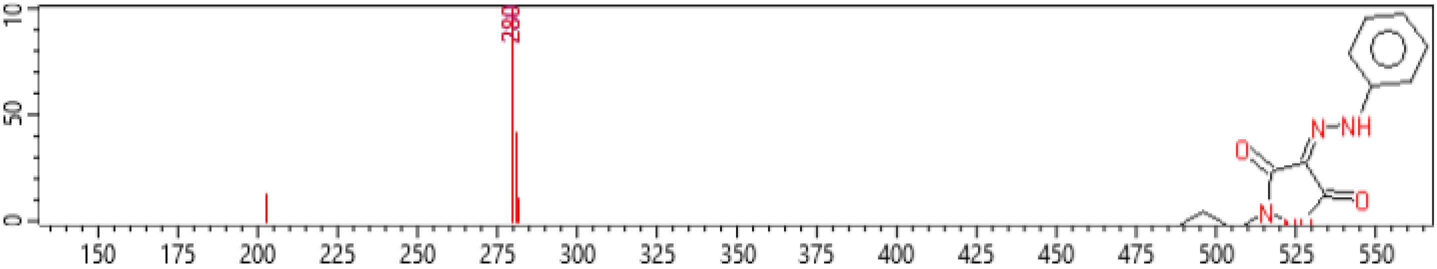


(2R,3R)-3,5-Dihydroxy-2-(4-hydroxyphenyl)-7-(3-methylbut-2-enoxy)-2,3-dihydrochromen-4-one, 3TMS

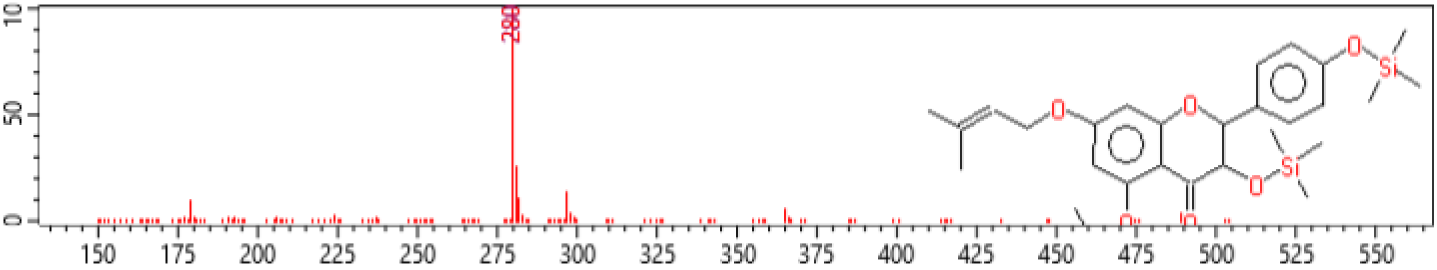


Phthalazine-1,4(2H,3H)-dione, 2-(2-ethyl-6-methylphenyl)-

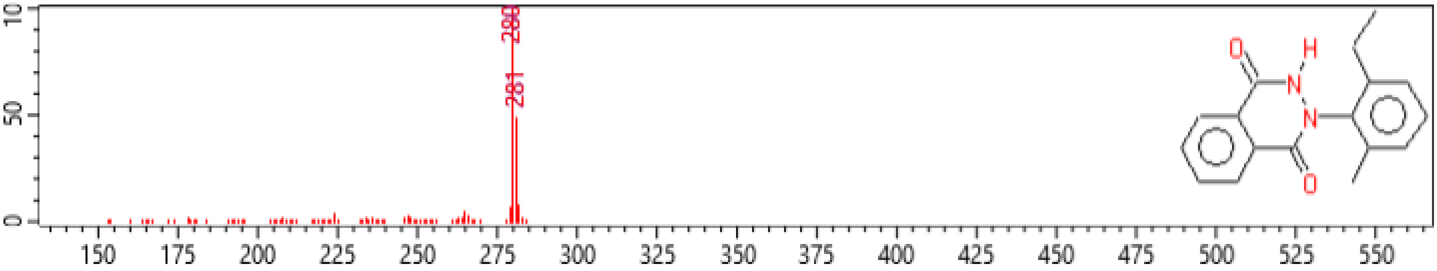


4,5-Bis(4-methylphenyl)-4H-1,2,4-triazole-3-thiol

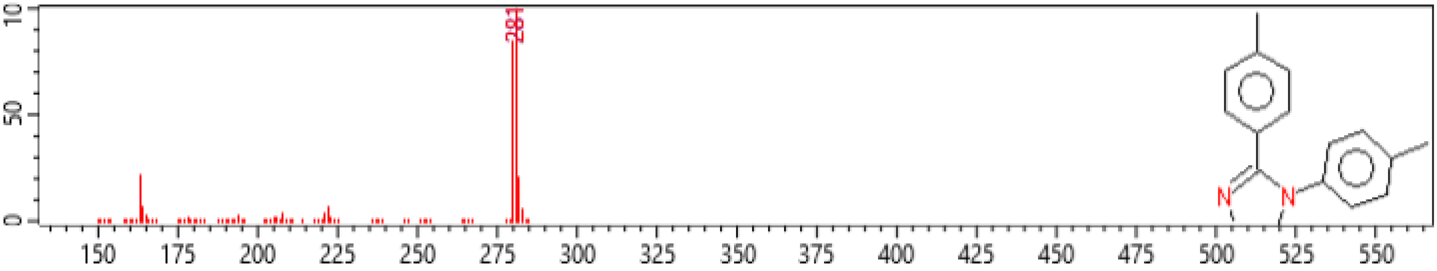

**2-Monostearin, 2TMS derivative**

Peak: 29.00 - 29.12 min (scan #6138 - #6173) Subtracted noise: 28.80 - 28.93 min (scan #6084 - #6119), 29.19 - 29.31 min (scan #61

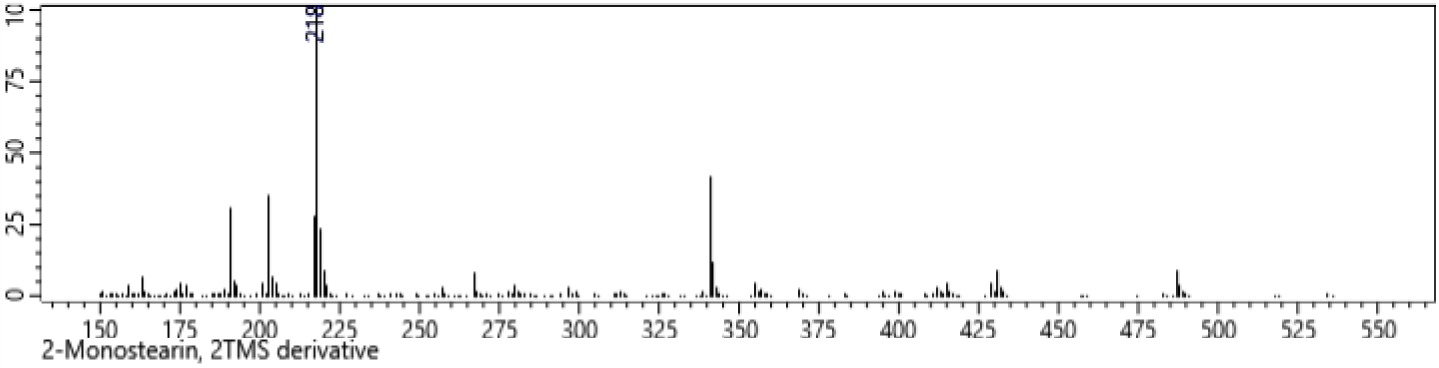


2-Monostearin, 2TMS derivative

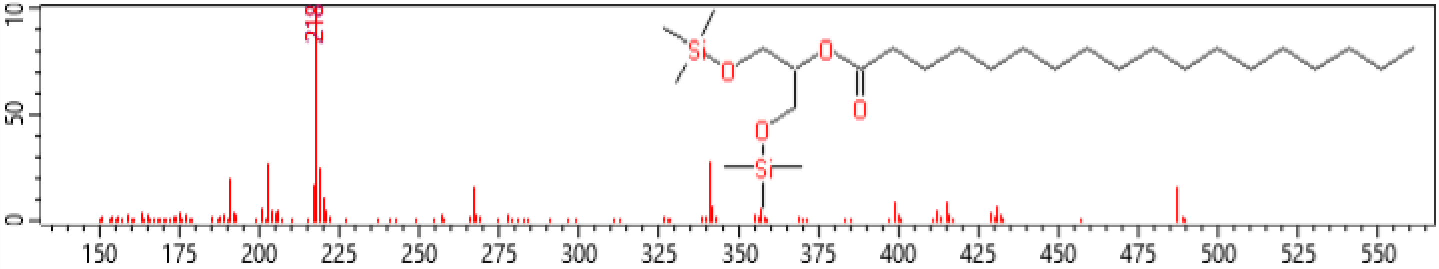


2-Monostearin, 2TMS derivative

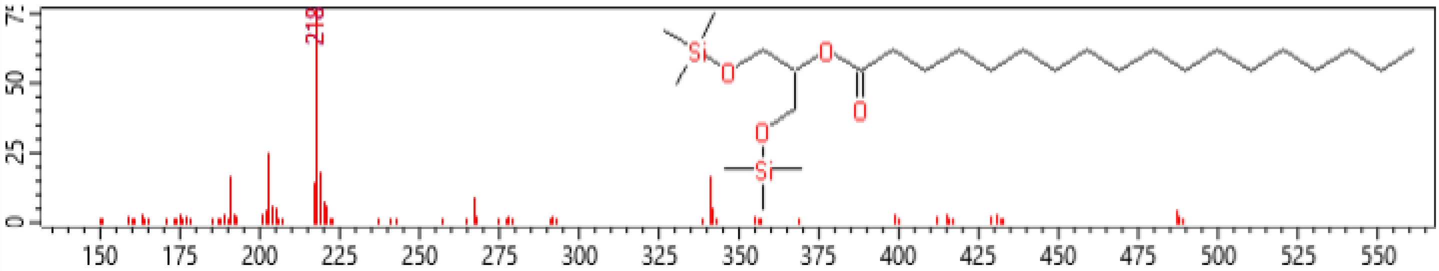


2-Palmitoylglycerol, 2TMS derivative

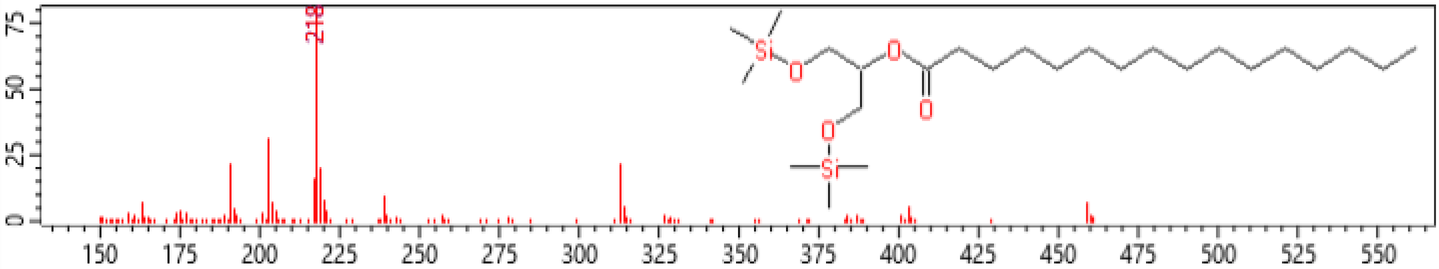


Acetin, bis-1,3-trimethylsilyl ether

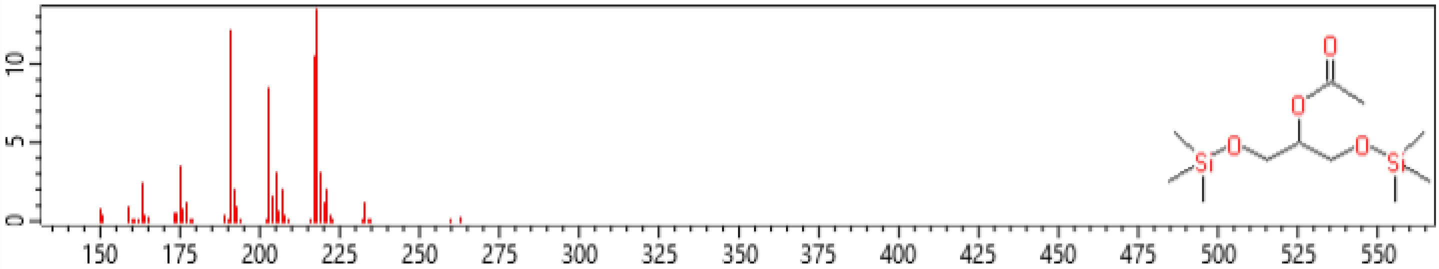

2-Monomyristin, 2TMS derivative

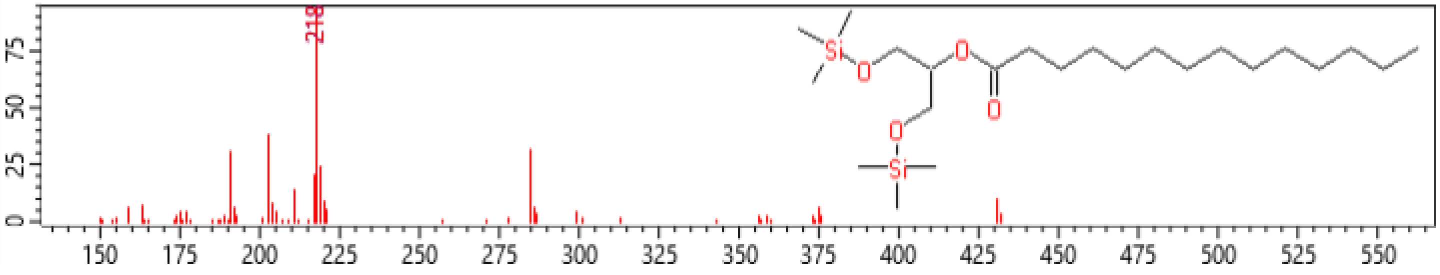


**Glycerol monostearate, 2TMS derivative**

Peak: 30.60 - 30.81 min (scan #6585 - #6643) Subtracted noise: 30.21 - 30.41 min (scan #6476 - #6533), 30.84 - 31.04 min (scan #66[illegible]

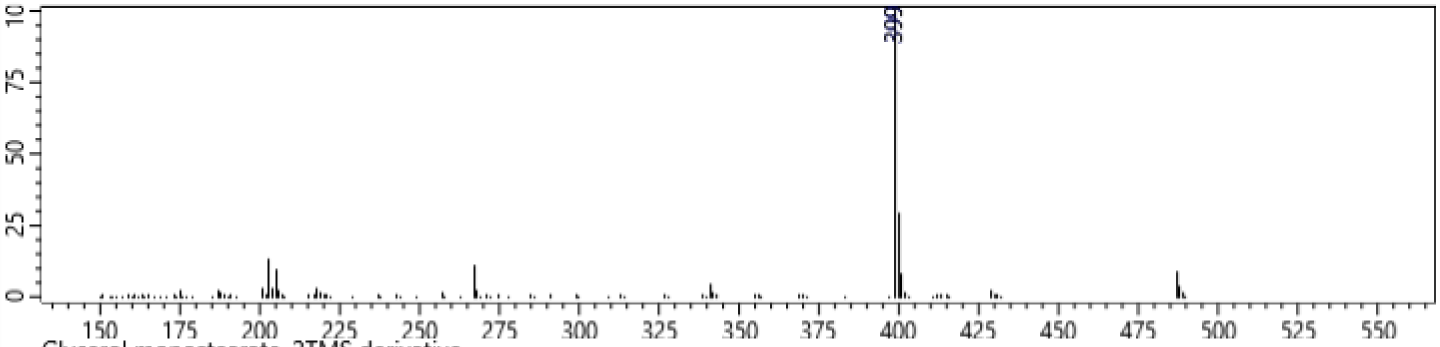


Glycerol monostearate, 2TMS derivative

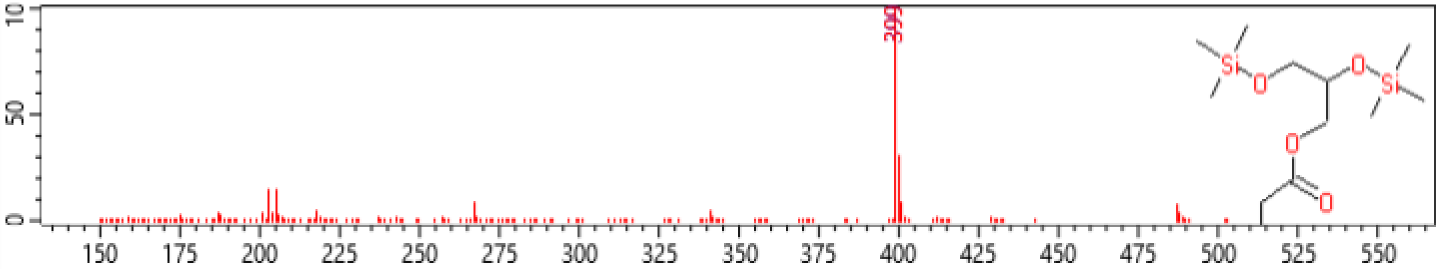


Glycerol monostearate, 2TMS derivative

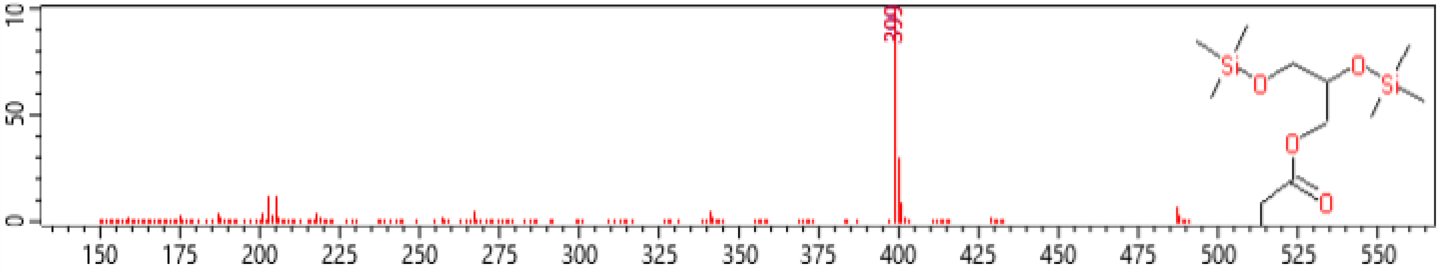


Glycerol monostearate, 2TMS derivative

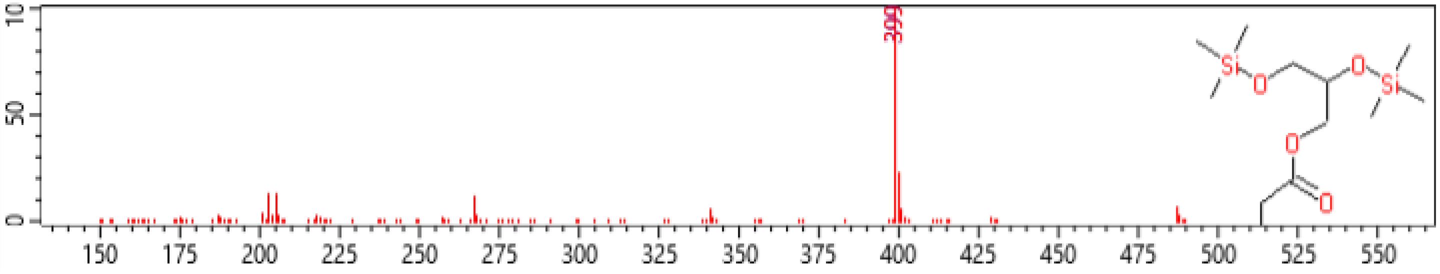

Glycerol monostearate, 2TMS derivative

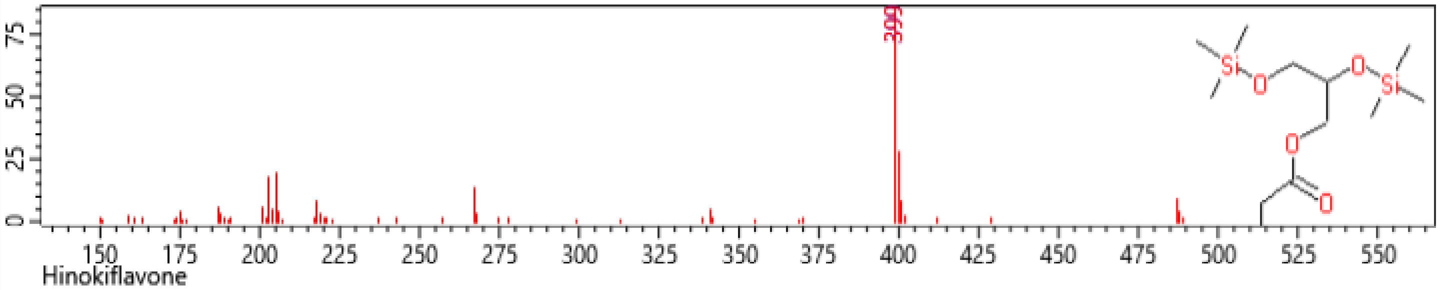


Hinokiflavone

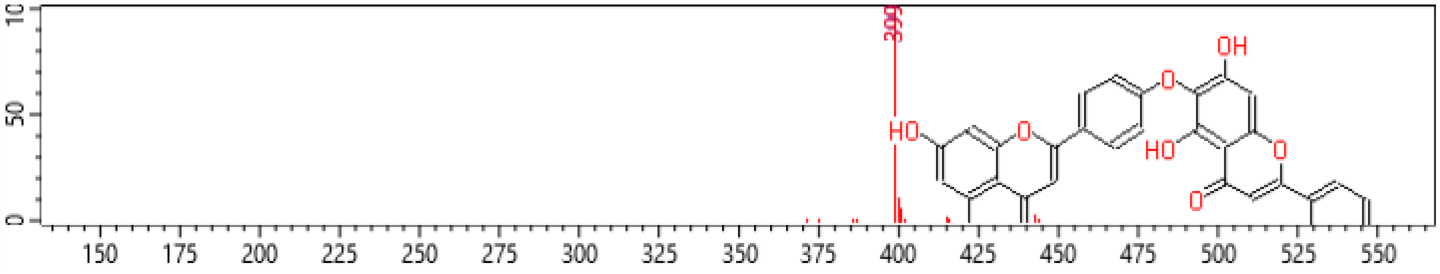


**Cycloolivil, 5TMS derivative**

Peak: 30.95 - 31.16 min (scan #6684 - #6742) Subtracted noise: 30.67 - 30.88 min (scan #6605 - #6663), 31.30 - 31.51 min (scan #67

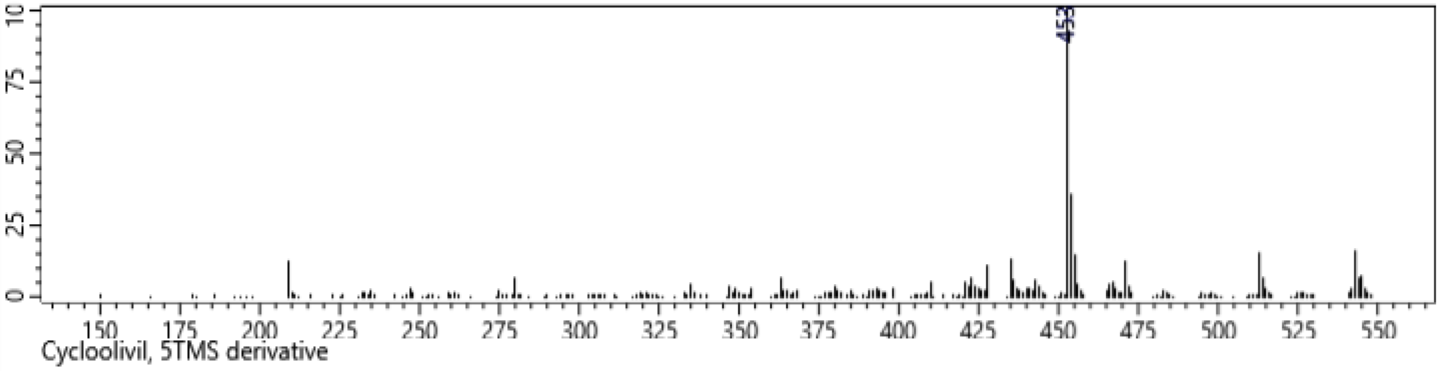


Cycloolivil, 5TMS derivative

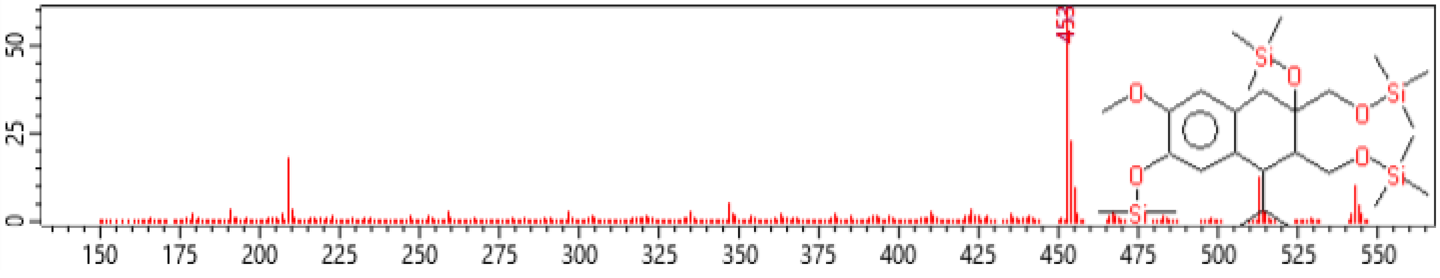


27-(Acetyloxy)-3-hydroxylup-20(29)-en-28-oic acid

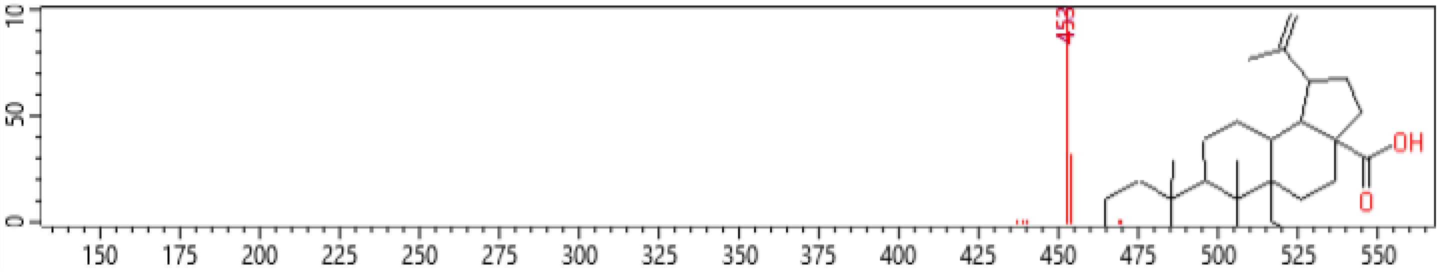

(2S)-5,7-Dihydroxy-6-(3-methylbut-2-enyl)-2-phenylchroman-4-one, 2TMS

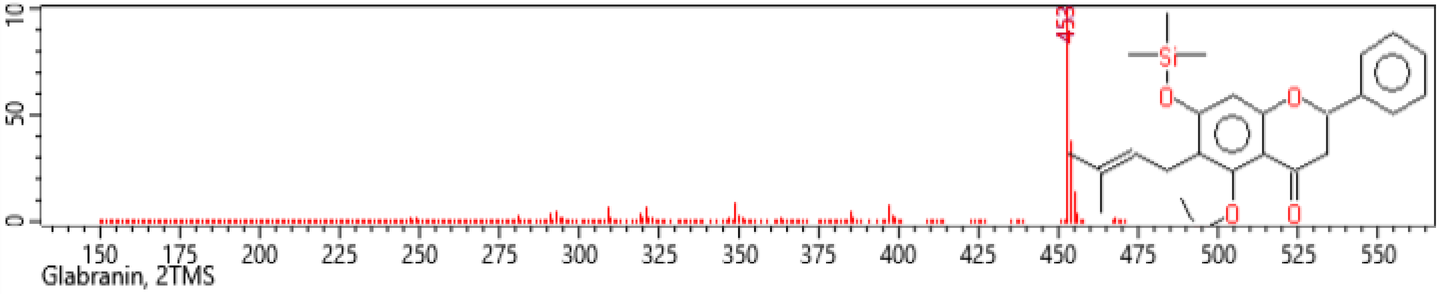


Glabranin, 2TMS

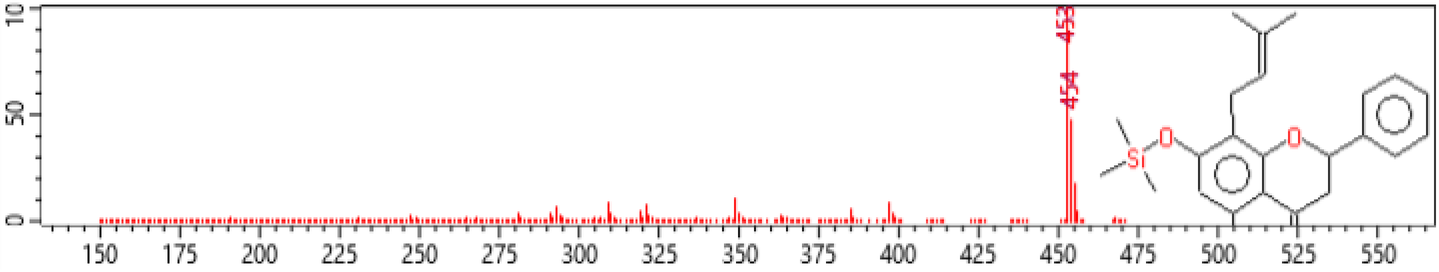


4,7-Benzofurandione, 3-acetyl-3a,7a-dihydro-3a,5,6,7a-tetrahydroxy-2-methyl-, 4TMS derivative

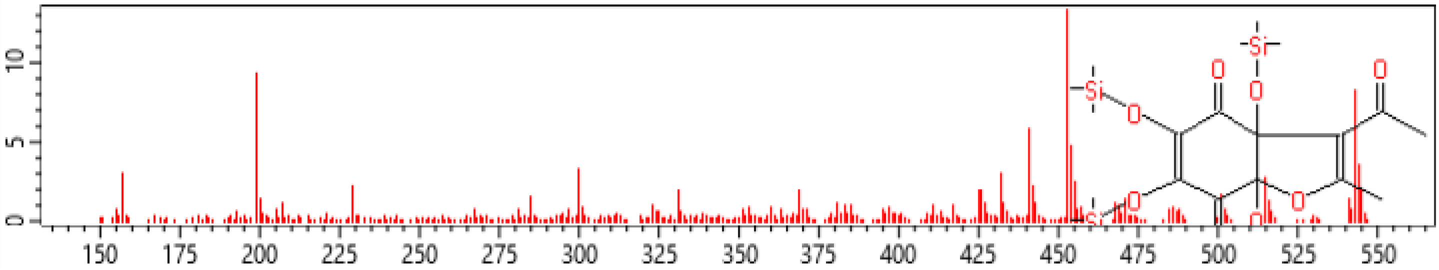


# Component - spectrum
