# Supplementary material for: Effective Detoxification of Olive Mill Wastewater Using Multi-Step Surfactant-Based Treatment: Assessment of Environmental and Health Impact
Source: Molecules. 2024 Sep 10;29(18):4284. doi: 10.3390/molecules29184284 (PMC11434139; doi:10.3390/molecules29184284)
Supplement: Supplementary file 1 [file molecules-29-04284-s001.zip › filt-1micro.pdf]

Analysis report

Sample Info

Analyst:	nora
Chromatogram:	№308 2023-01-23 14:58:37
Data file:	G:\Analytic 3\Projects\2291421+МСД 2160148\.chromatograms\2023-01-23 14-58-37 0308.chrx
Instrument:	Chromatec-Crystal 5000 S/N: 2291421 Firmware version: v 03.21.17.732
Method:	C:\ProgramData\Chromatec\Chromatec.Panel.UI\TempMethods\صيدلة_Scheduler_b9967dd176d34d258e241a8f97a80c7c.mthx
Sample name:	filt-1micro
Sample volume:	1
Dilution:	1
Column:	
Comments:	

Instrument Info

```
Instrument method: صيدلة.mthx
Channel - 1
Run time, min: 35.3077
PostRun, min: 0
Equilibration time, min: 0
Oven - 1
Temperature, °C: (80; 3)(13; 240; 20)
Inlet - 1
Mode: Splitless [+ gas saver]
PrepRun: Off
Split flow, ml/min: (0; 1)(0; 30; 3)(200; 10; 0.0)
Septum purge flow, ml/min: (0; 1)(0; 3; 0.0)
Temperature, °C: 250.0
Column - 1
Control mode: Constant capillary flow
Capillary flow, ml/min: 1.000
Solenoid valve - 1
PrepRun: On
Switching time: (On; 1)
MSD
Runtime, min = 35.3077
Ion source temperature, ℃ = 280
Transfer line temperature, ℃ = 200
Calibration gas = Off
Ionization type = Electron ionization
Data collection type = Centroid
Segments count = 1
Segment number = 1
Start time, min = 7
Filament = On
Emmission current = 100
Gain = 300000
Scans count (in segment) = 1
Scan number = 1
Acquisition type = Mass range
Mass range = 150 - 550
Dwell time, sec = 0.2
Settings = Current tune

Vial number : 2
Sampling type : Simple
Vial needle depth, mm : 28
Solvent washing
Vials for washing : A
Solvent volume, ul : 5
Number of washing cycles : 3
Drain speed, µl/min : 300
Sample washing
Number of washing cycles : 3
Injection speed, µl/min : 2
Sample volume, ul : 3
Sampling
Sampling speed, µl/min : 2
Number of strokes : 0
Viscosity delay, sec : 0
Sample volume, ul : 1
Injection
Pre injection delay, sec : 0
Post injection delay, sec : 0
Injection speed, µl/min : 4500
Solvent washing
Vials for washing : A
Solvent volume, ul : 3
Number of washing cycles : 3
Drain speed, µl/min : 2
```

Chromatogram

MSD-1 : TIC

Range from 7.000 min. to 35.274 min.

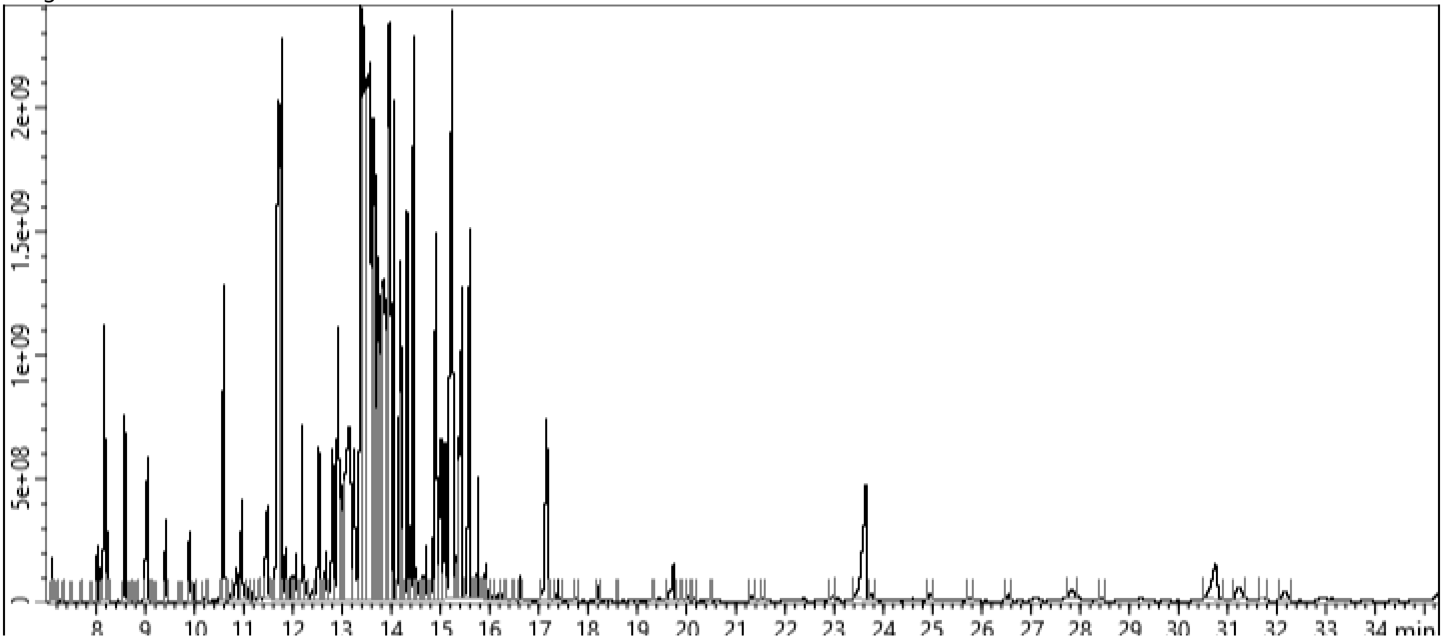

Spectra

Benzyl alcohol, TMS derivative

Peak: 7.08 - 7.11 min (scan #23 - #31) Subtracted noise: 7.03 - 7.06 min (scan #9 - #17), 7.12 - 7.15 min (scan #34 - #42)

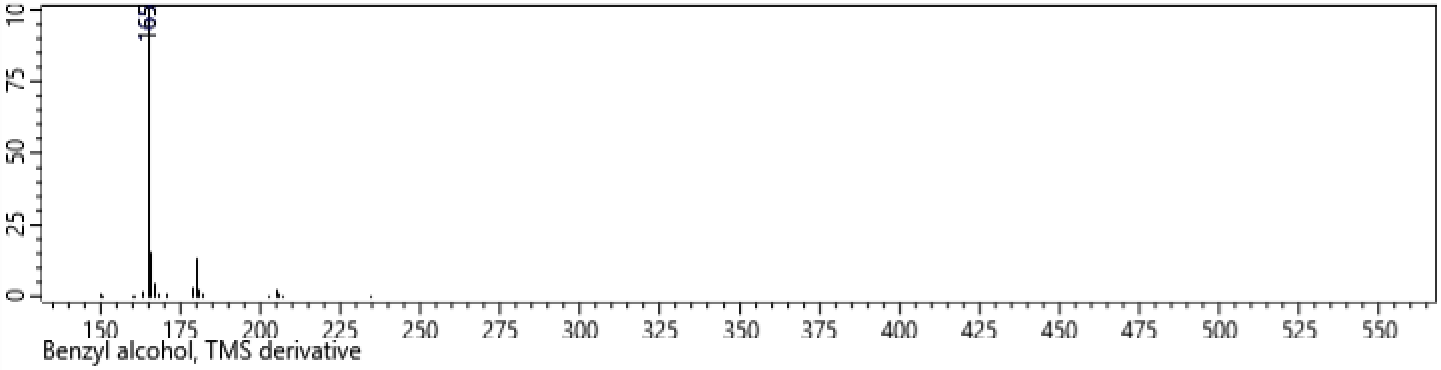

Benzyl alcohol, TMS derivative

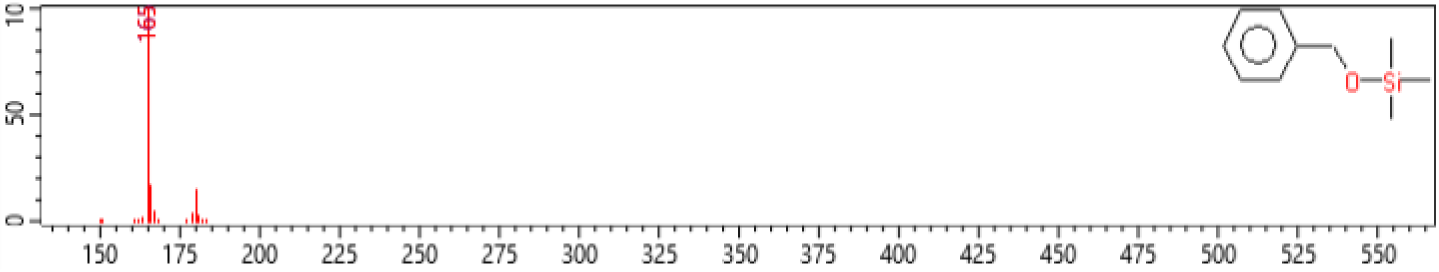

m-Cresol, TMS derivative

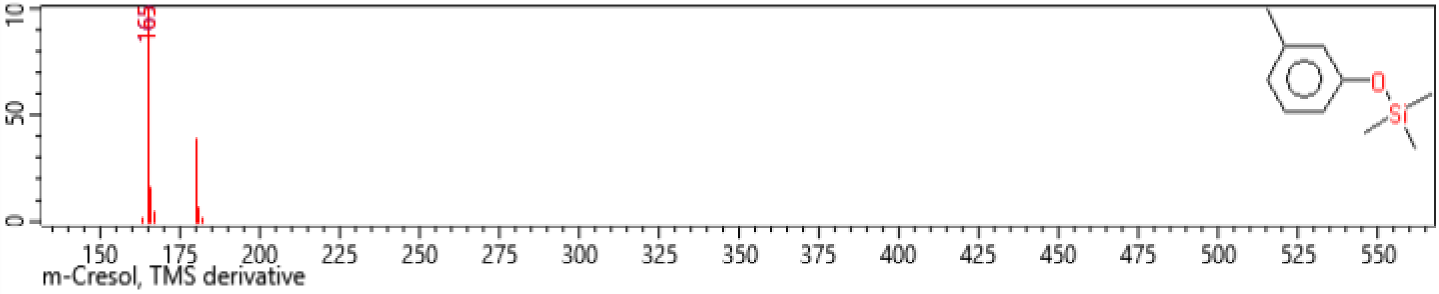

m-Cresol, TMS derivative

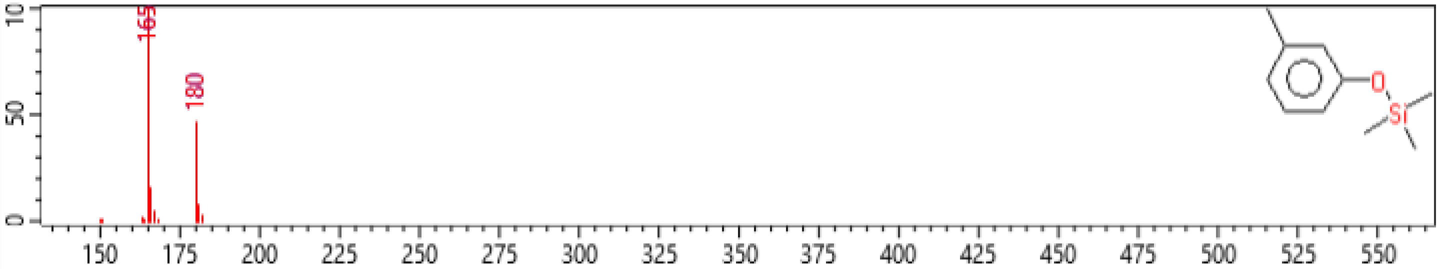

m-Cresol, TMS derivative

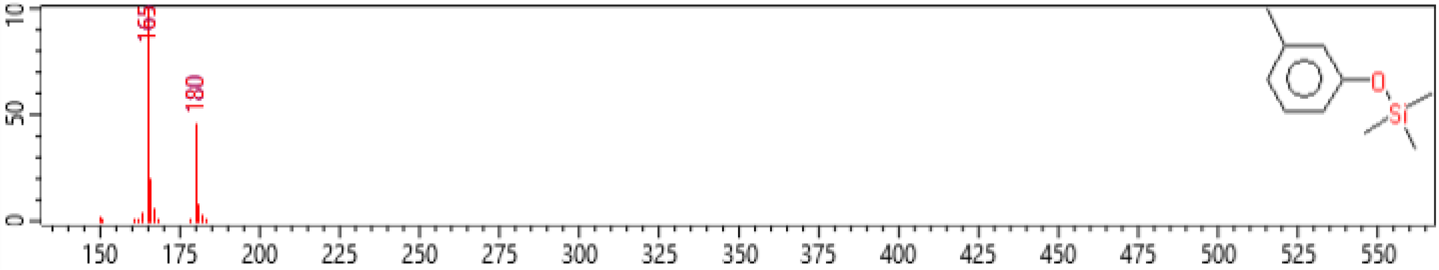

5-Methyl-N-(trimethylsilyl)pyridin-2-amine

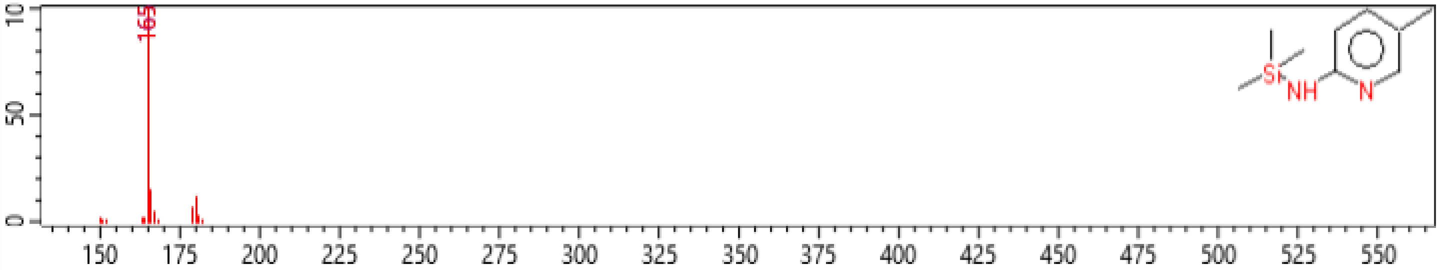

3-Hydroxybutyric acid, 2TMS derivative

Peak: 7.15 - 7.18 min (scan #43 - #49) Subtracted noise: 7.12 - 7.14 min (scan #34 - #39), 7.19 - 7.21 min (scan #54 - #59)

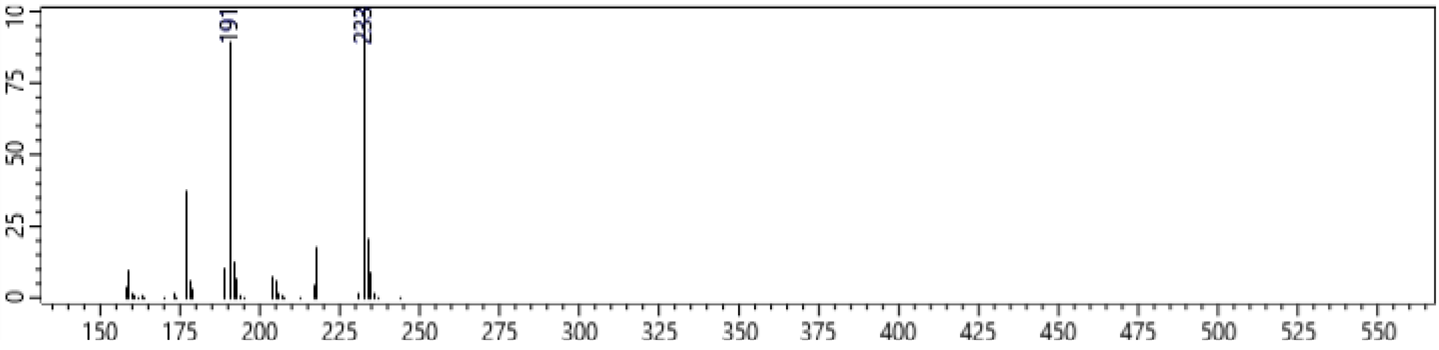

3-Hydroxybutyric acid, 2TMS derivative

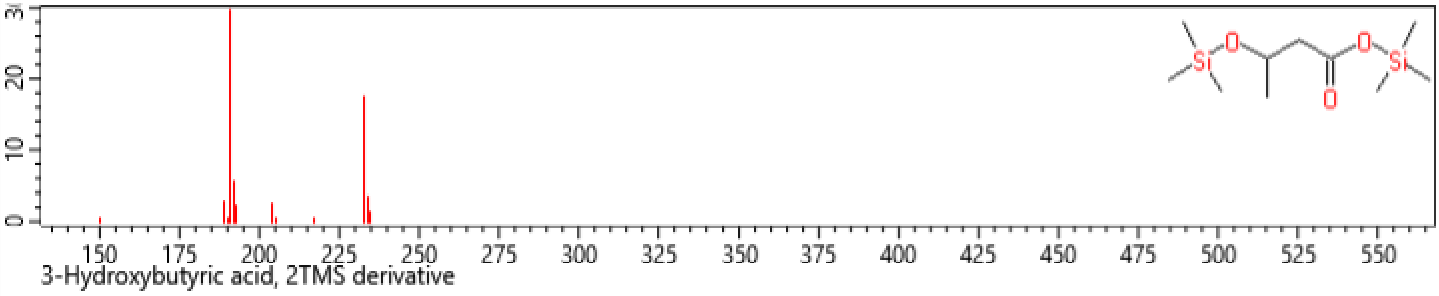

3-Hydroxybutyric acid, 2TMS derivative

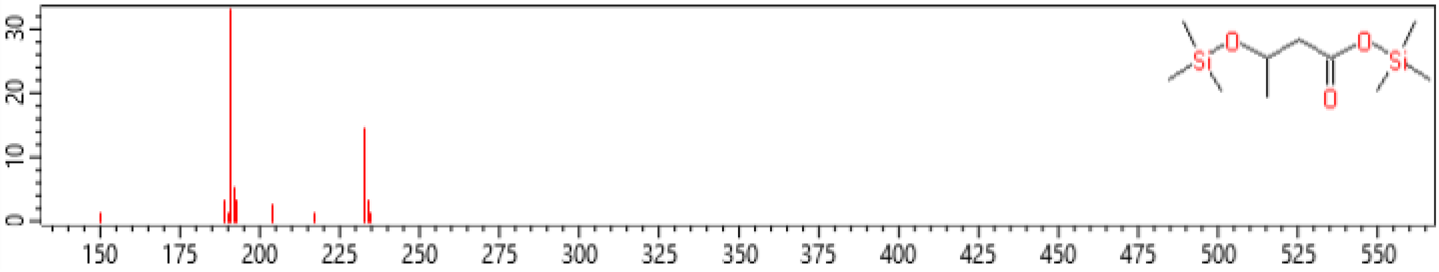

3-Hydroxybutyric acid, 2TMS derivative

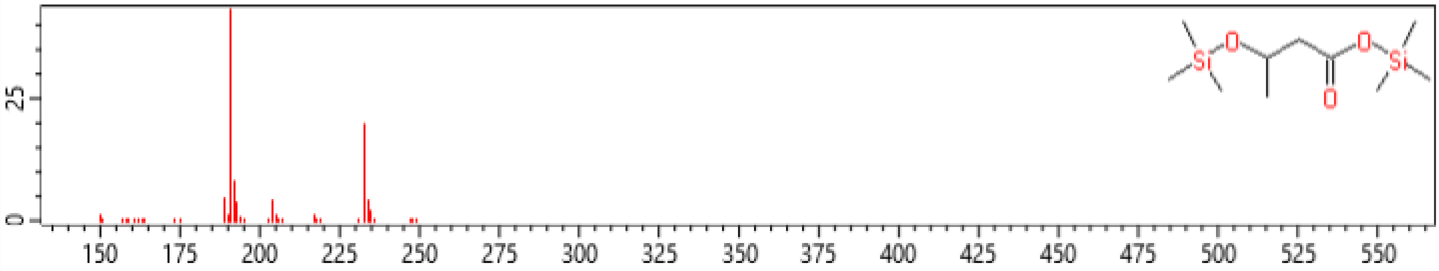

3,5-di-tert-butyl-4-hydroxybenzaldehyde

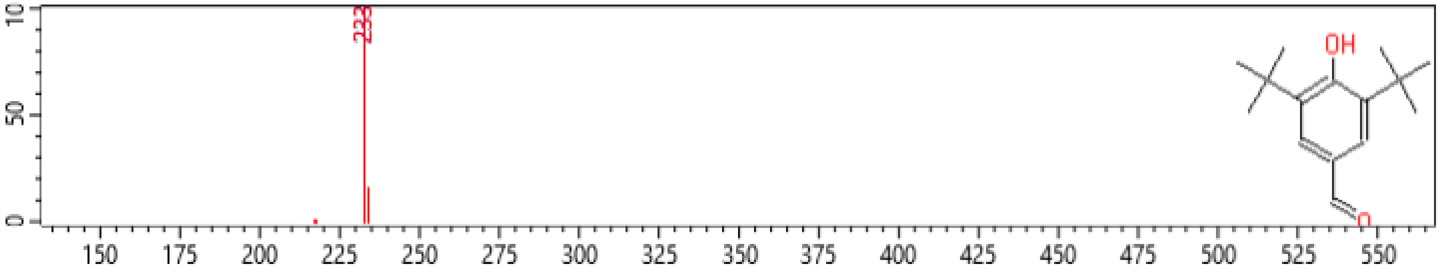

2-propanone, 1-[5-(phenylamino)-1,2,4-thiadiazol-3-yl]-

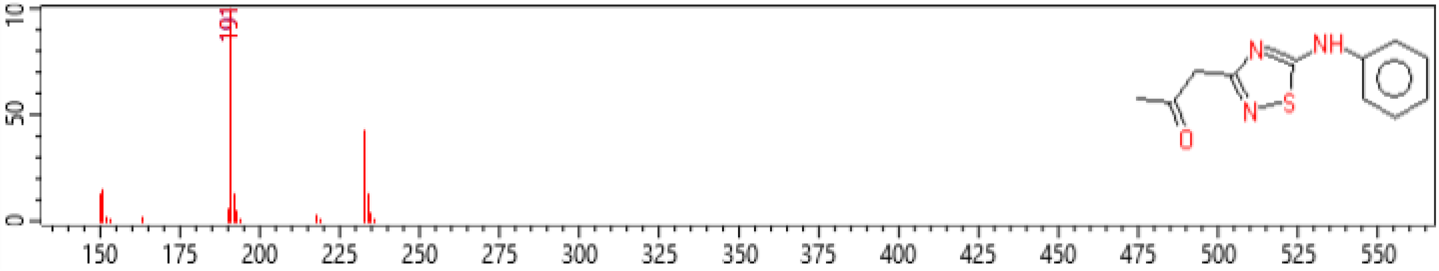

Imatinib

Peak: 7.21 - 7.23 min (scan #59 - #65) Subtracted noise: 7.17 - 7.19 min (scan #47 - #53), 7.24 - 7.26 min (scan #67 - #73)

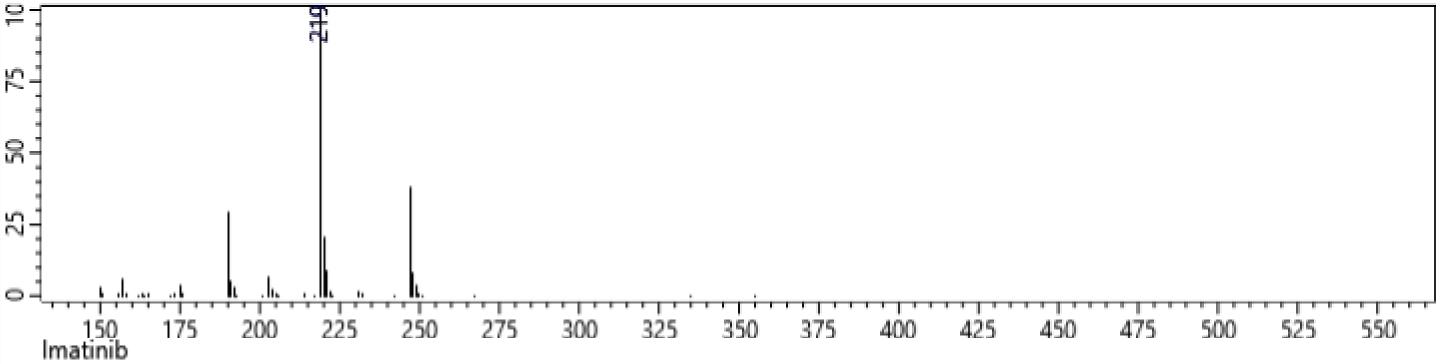

Imatinib

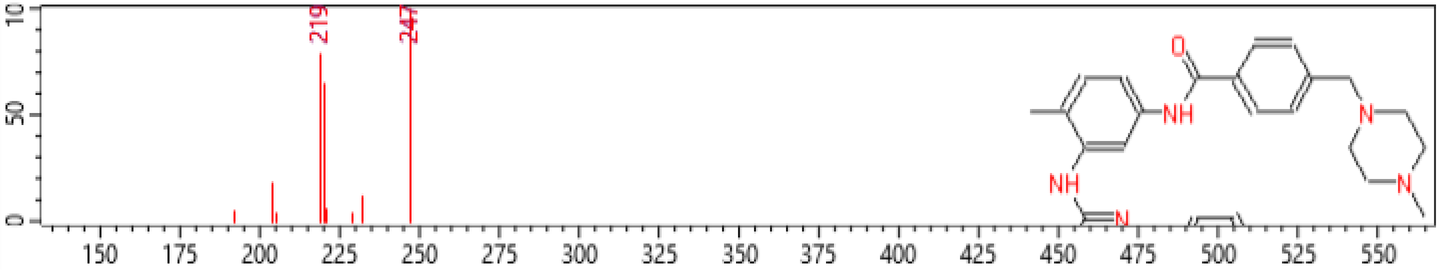

Ethyl 2(2-pyridyl)-4-(bromomethyl)thiazole-5-carboxylate

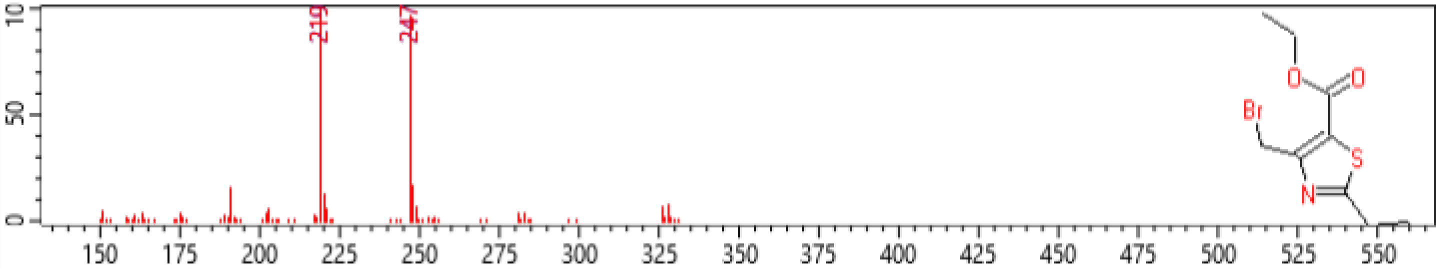

Benzo[c]phenoxazin-10-one

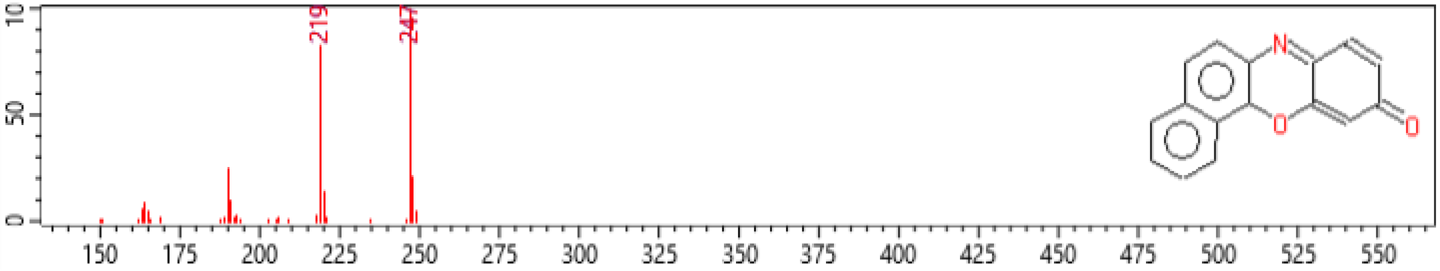

4-(2-Methyl-2H-tetraazol-5-yl)aniline, TMS derivative

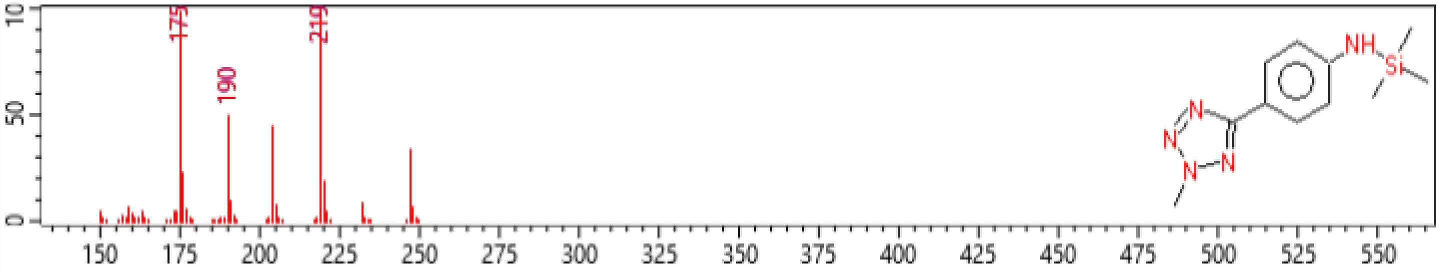

Diclofop-methyl

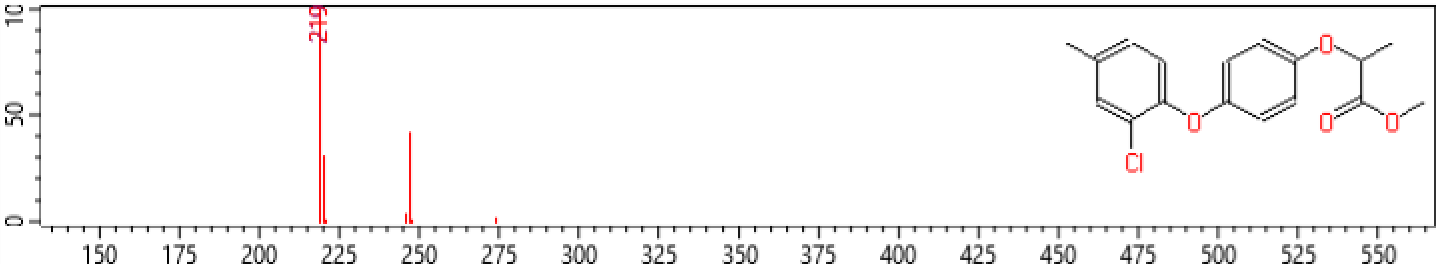

Monomethyl succinate, trimethylsilyl ester
Peak: 7.34 - 7.35 min (scan #95 - #99) Subtracted noise: 7.30 - 7.32 min (scan #85 - #89), 7.37 - 7.38 min (scan #102 - #106)

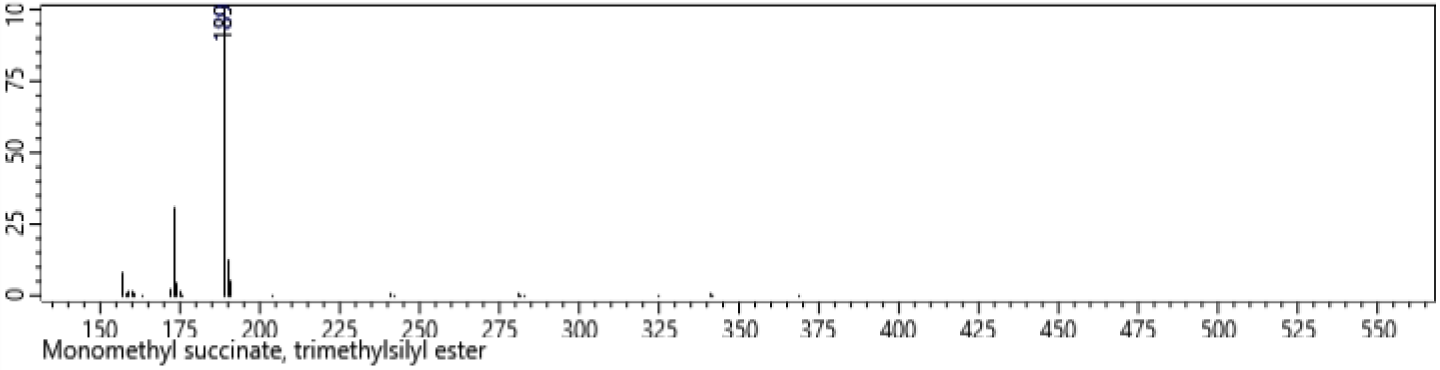

Monomethyl succinate, trimethylsilyl ester

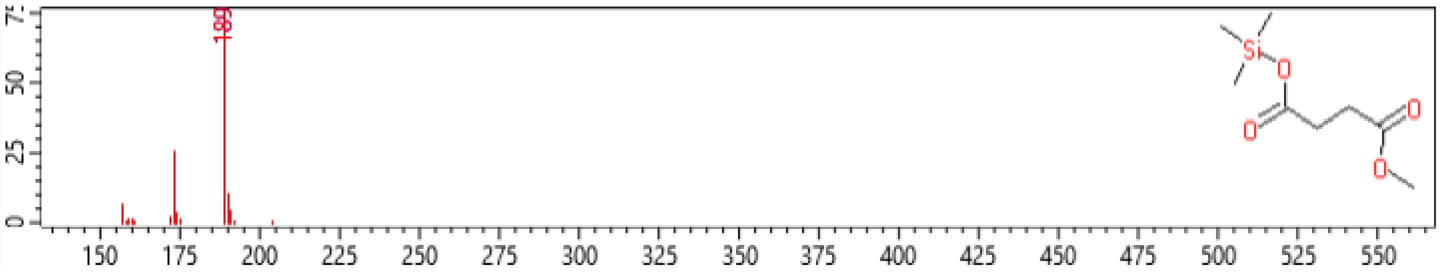

Monomethyl succinate, trimethylsilyl ester

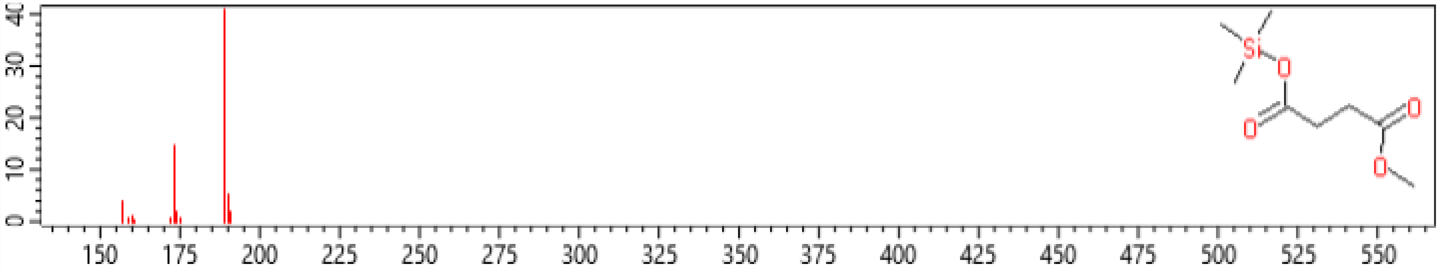

Monomethyl succinate, trimethylsilyl ester

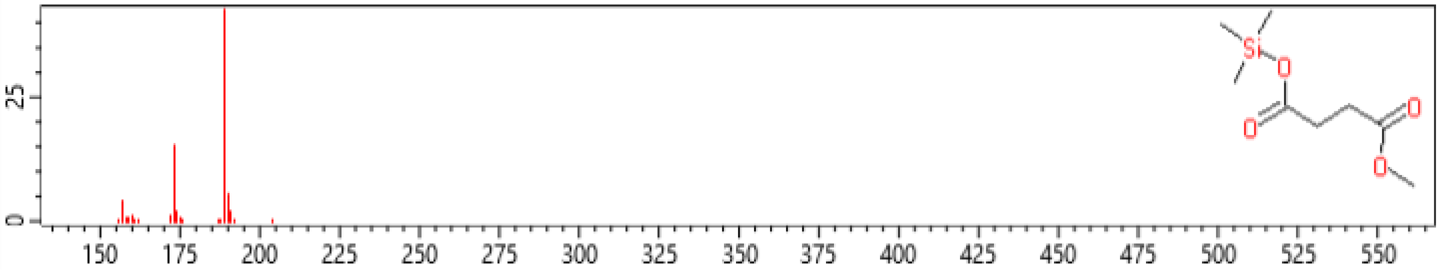

3,5-Di-tert-butyl-4-hydroxybenzoic acid

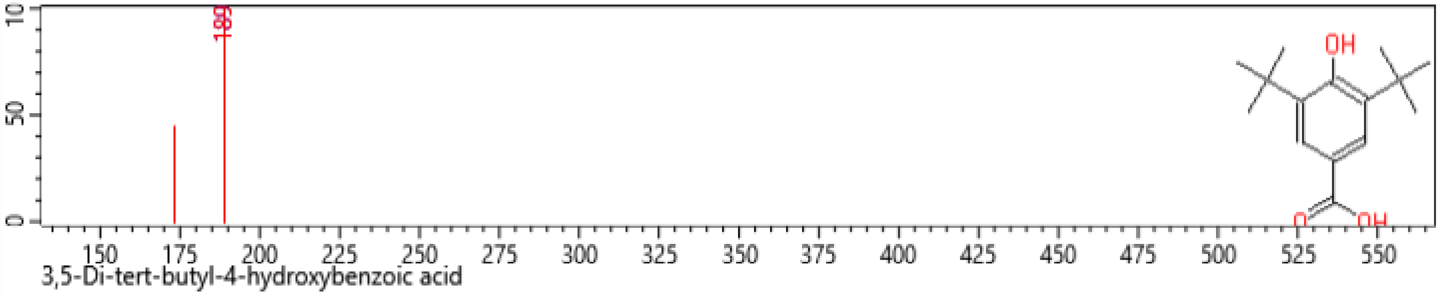

3,5-Di-tert-butyl-4-hydroxybenzoic acid

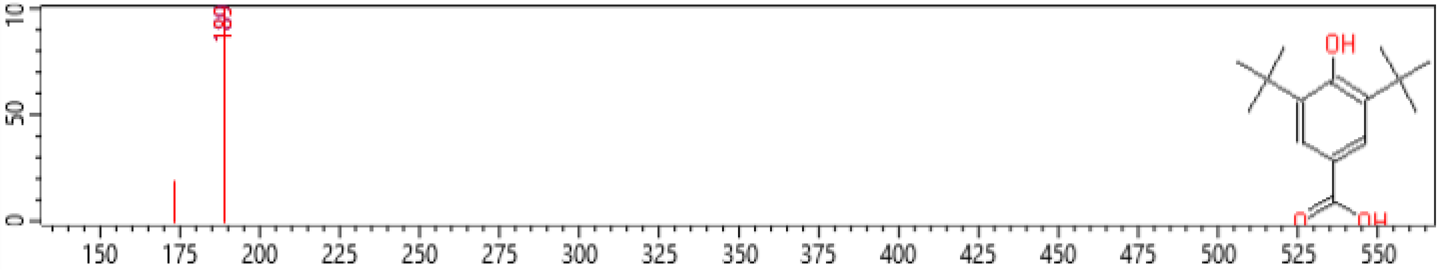

Pro-Ala

Peak: 7.49 - 7.50 min (scan #137 - #140) Subtracted noise: 7.47 - 7.48 min (scan #130 - #133), 7.51 - 7.52 min (scan #143 - #146)

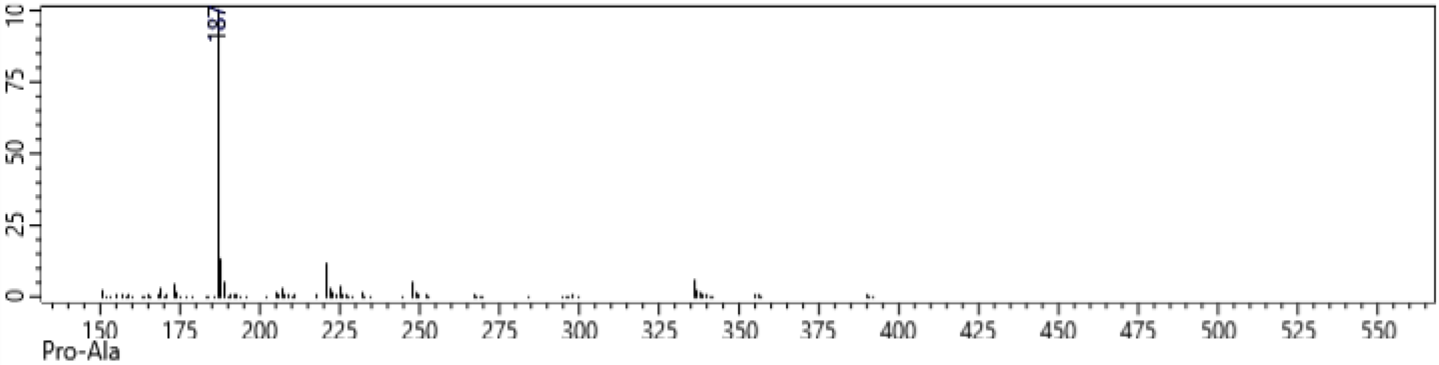

Pro-Ala

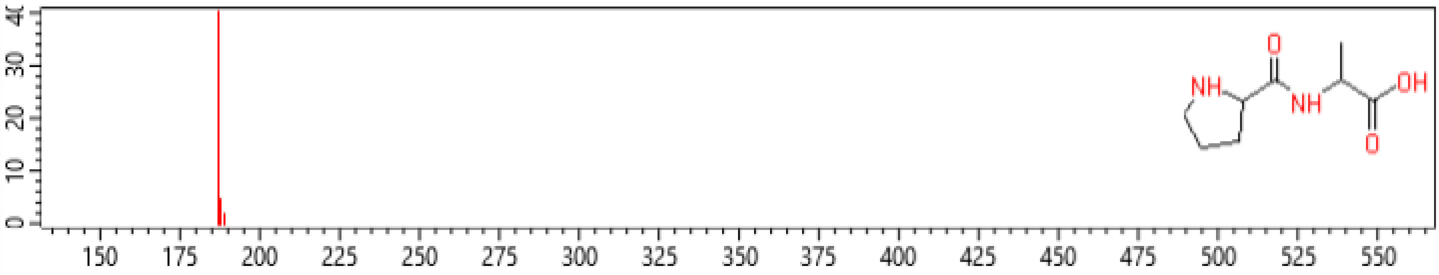

2-Ethylhexanol, TMS derivative

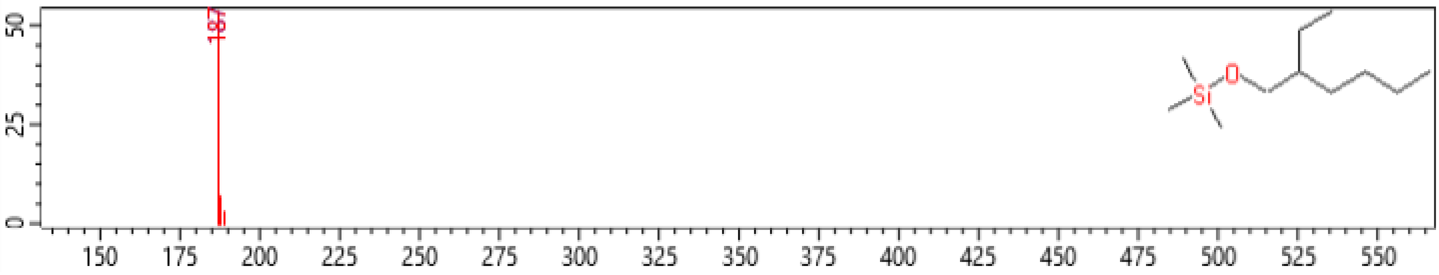

2,3-Dimethylpentanoic acid, TMS

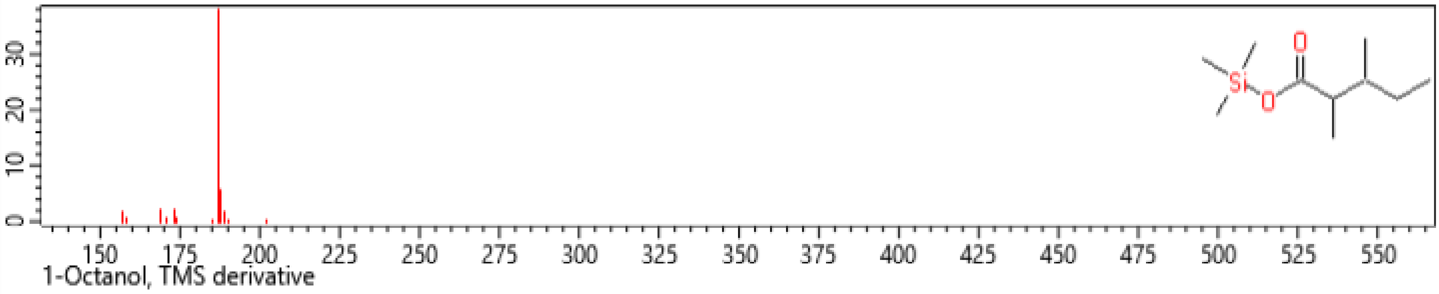

1-Octanol, TMS derivative

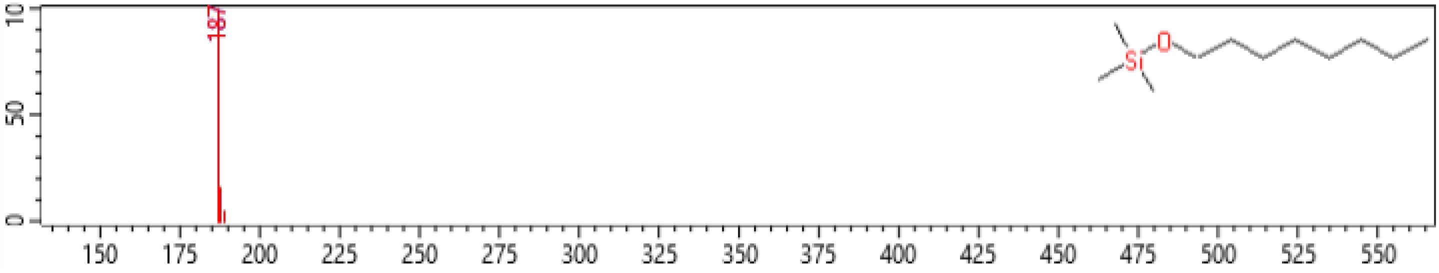

3-Hydroxycapric acid

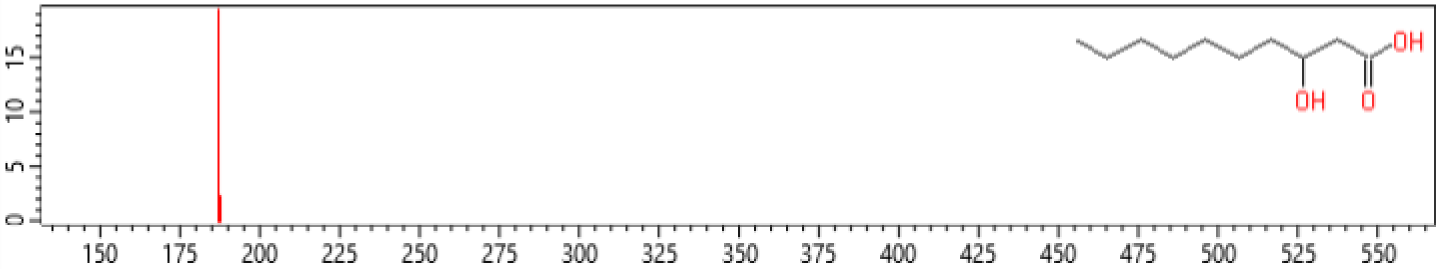

3,5-di-tert-butyl-4-hydroxybenzaldehyde

Peak: 7.69 - 7.71 min (scan #192 - #197) Subtracted noise: 7.66 - 7.67 min (scan #183 - #188), 7.72 - 7.73 min (scan #201 - #205)

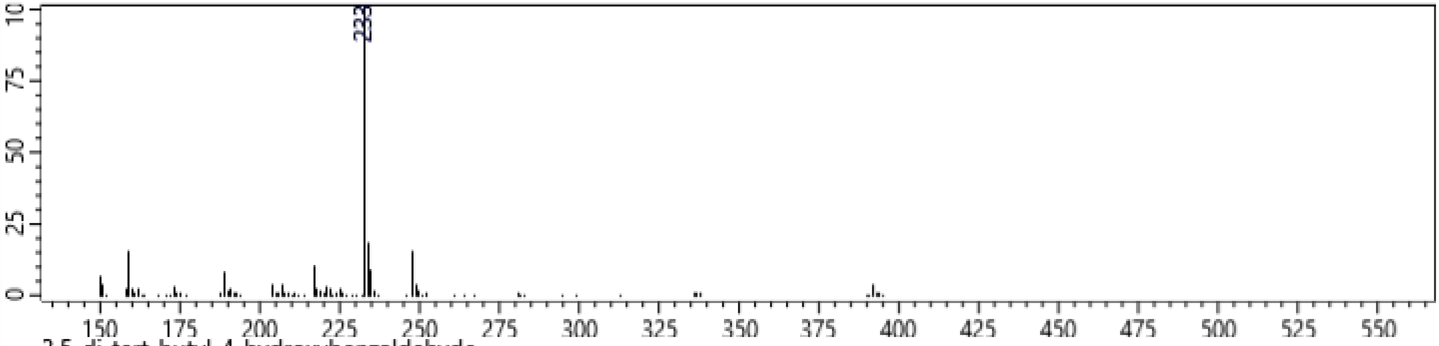

3,5-di-tert-butyl-4-hydroxybenzaldehyde

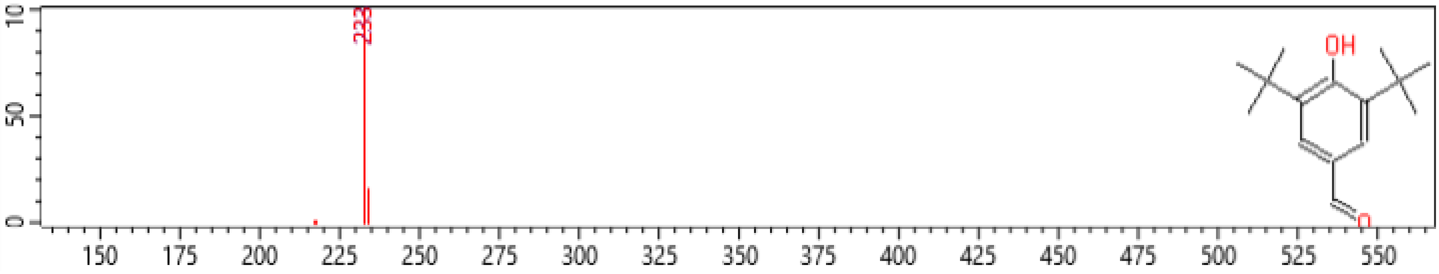

3,5-di-tert-butyl-4-hydroxybenzaldehyde

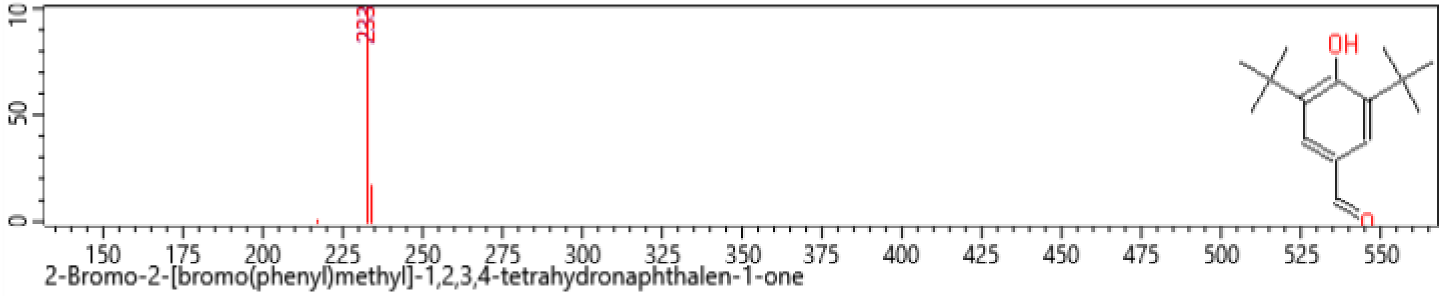

2-Bromo-2-[bromo(phenyl)methyl]-1,2,3,4-tetrahydronaphthalen-1-one

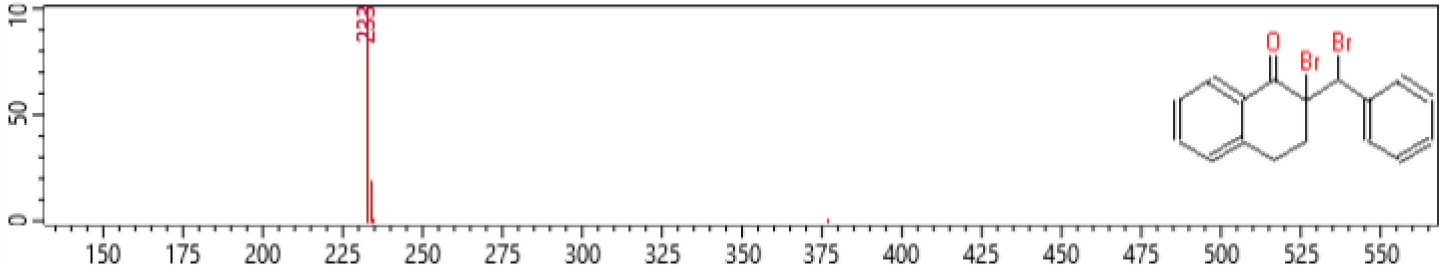

3,5-di-tert-Butyl-4-hydroxyacetophenone

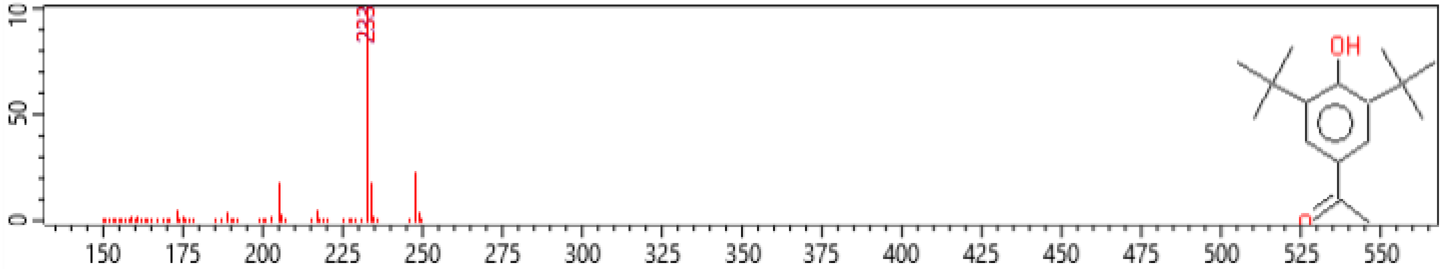

4-(Trimethylsilyl)oxy-6-methylcoumarin

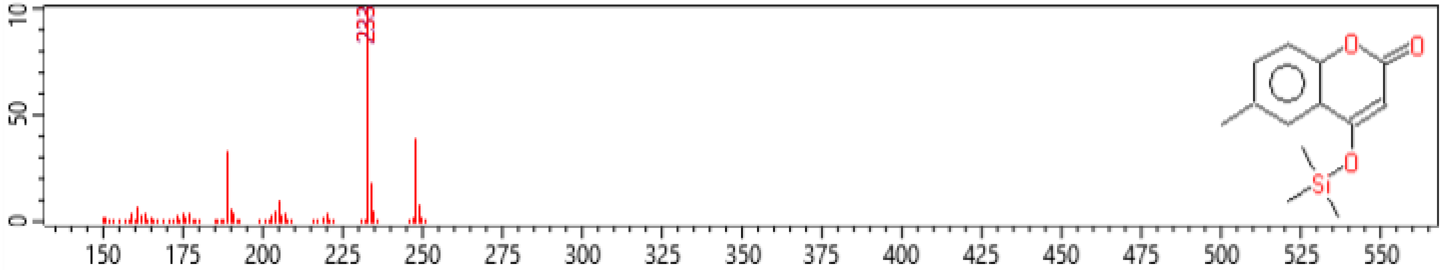

4-(4-tert-Butylphenyl)-1,3-thiazol-2-ylamine

Peak: 7.90 - 7.91 min (scan #252 - #254) Subtracted noise: 7.88 - 7.89 min (scan #246 - #248), 7.92 - 7.93 min (scan #257 - #259)

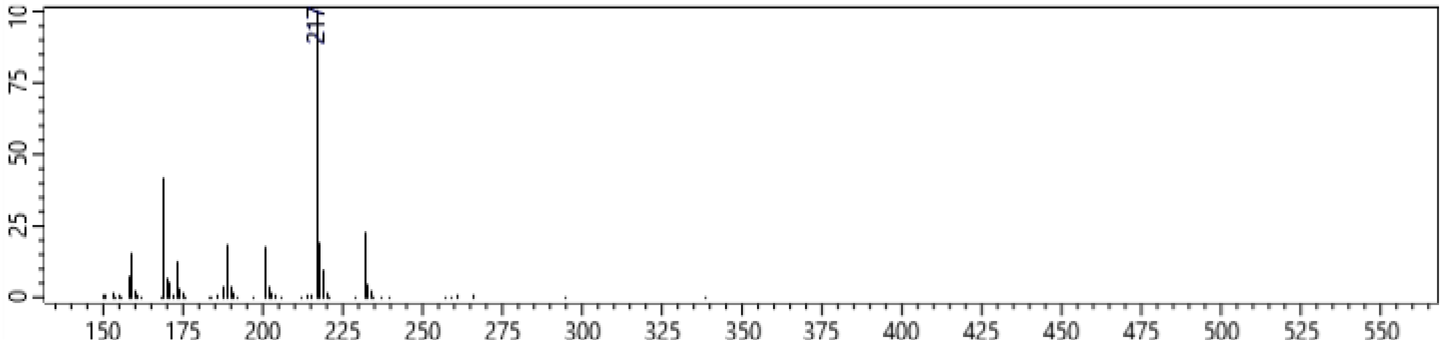

4-(4-tert-Butylphenyl)-1,3-thiazol-2-ylamine

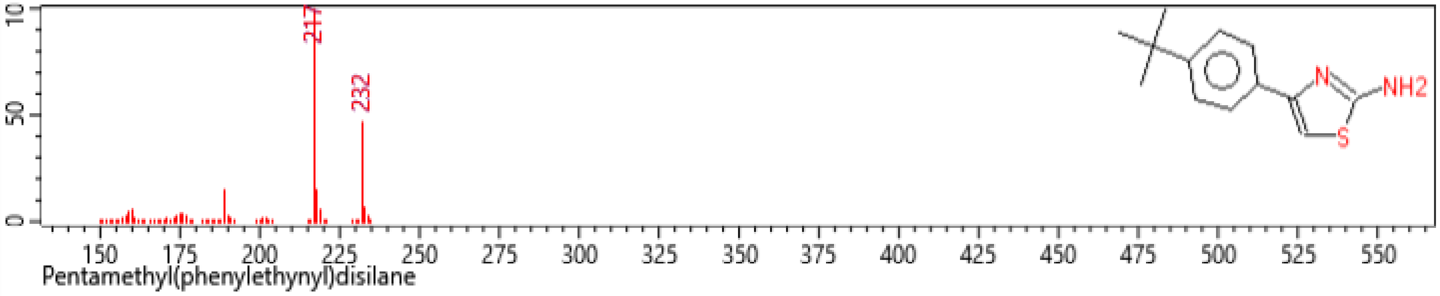

Pentamethyl(phenylethynyl)disilane

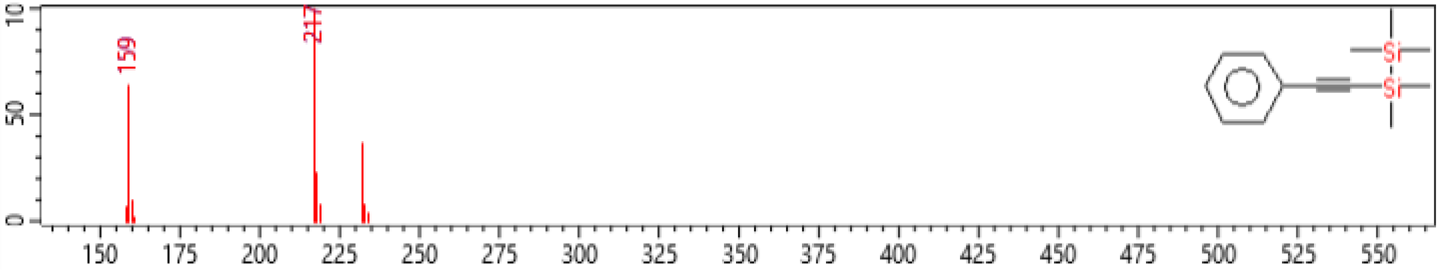

(3,4-Difluorophenyl)methanethiol, S-trimethylsilyl-

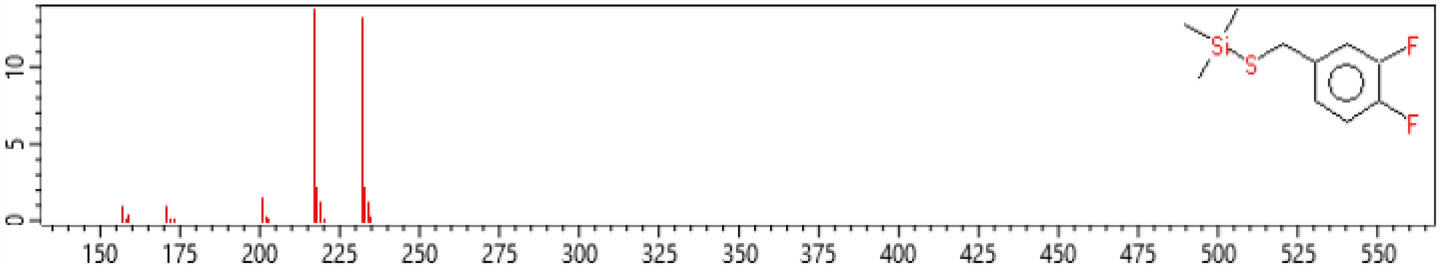

Glyoxime, 2TMS derivative

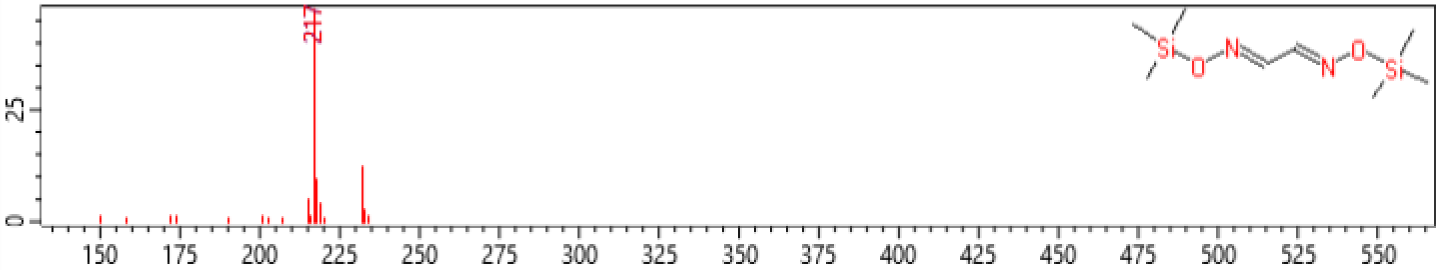

3,5-Difluorobenzyl mercaptan, S-trimethylsilyl-

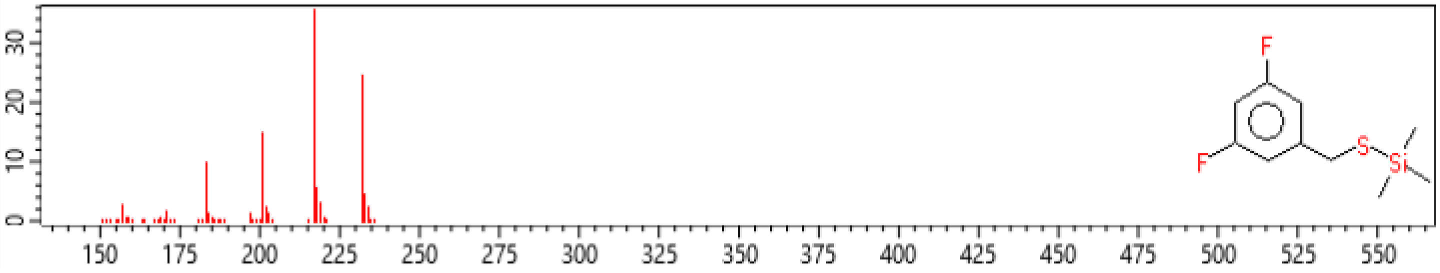

Phenylethyl Alcohol, TMS derivative

Peak: 8.01 - 8.04 min (scan #281 - #291) Subtracted noise: 7.95 - 7.98 min (scan #264 - #274), 8.07 - 8.11 min (scan #299 - #309)

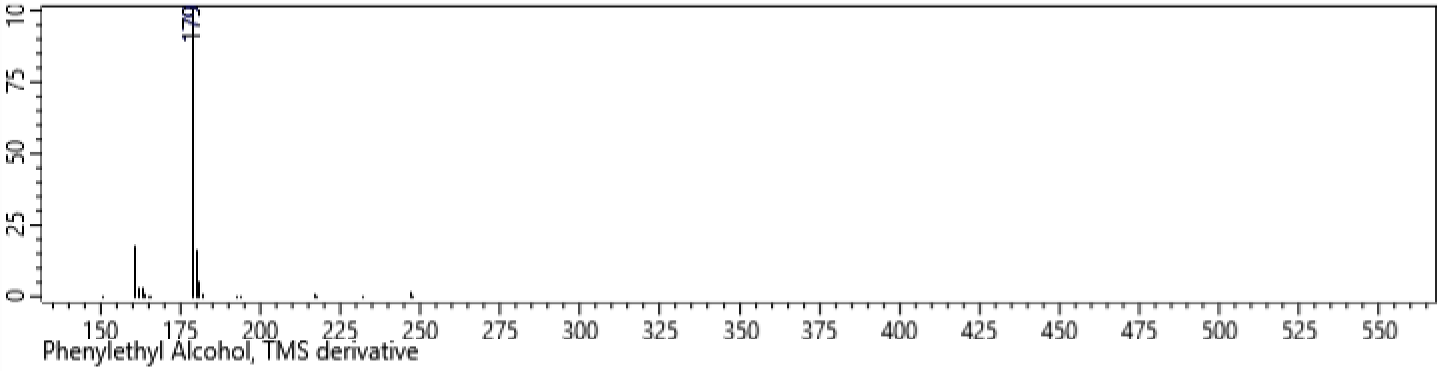

Phenylethyl Alcohol, TMS derivative

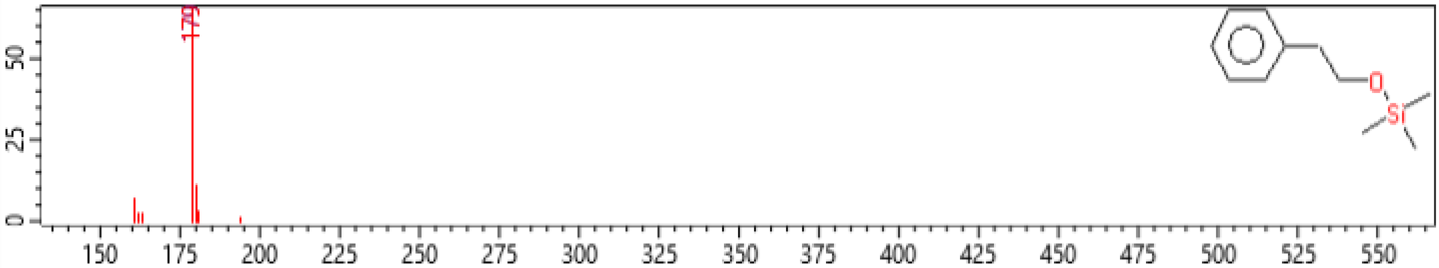

1-Cyclohexyldimethylsilyloxy-2-phenylethane

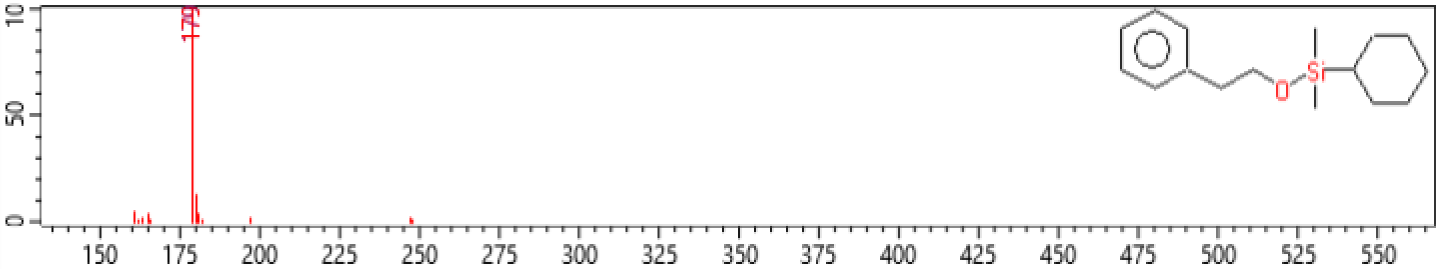

Salicylaldehyde, TMS derivative

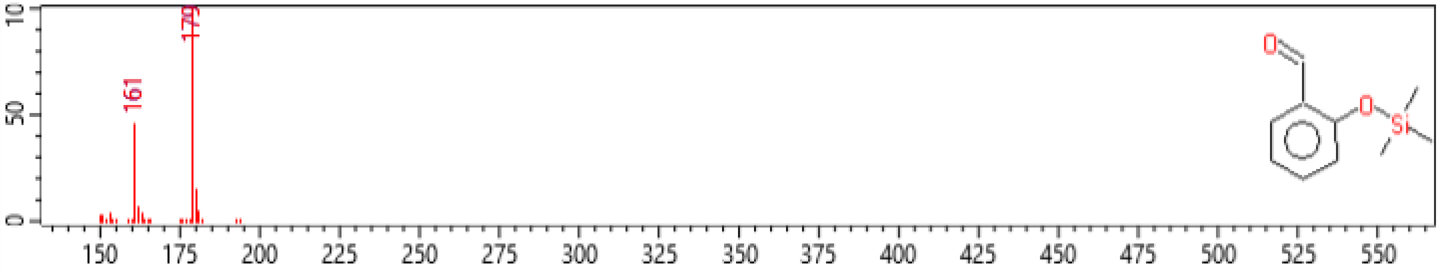

Salicylaldehyde, TMS derivative

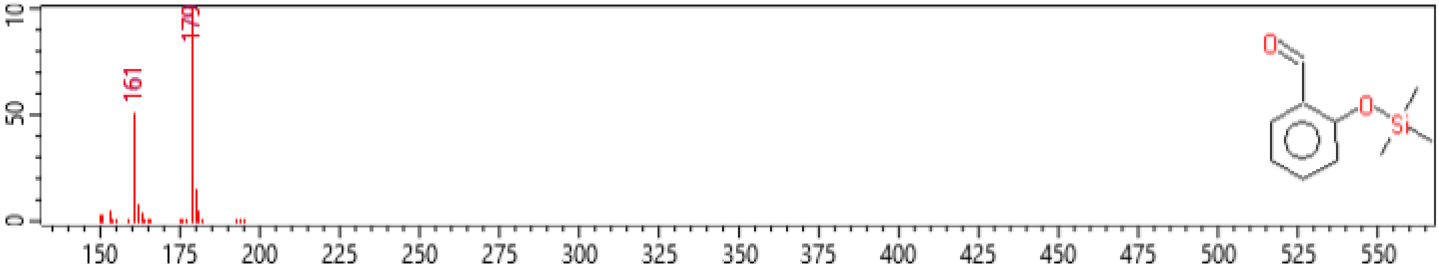

1-Ethyl(dimethyl)silyloxy-2-phenylethane

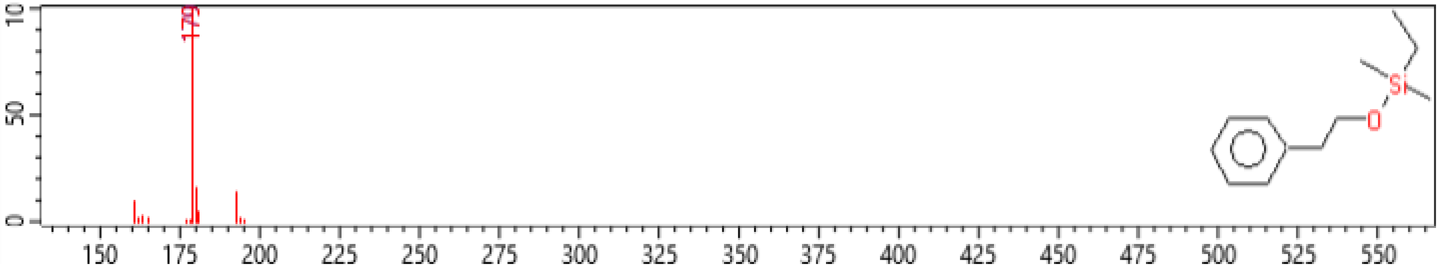

2-Hydroxyisocaproic acid, 2TMS derivative

Peak: 8.09 - 8.11 min (scan #303 - #309) Subtracted noise: 8.04 - 8.07 min (scan #291 - #298), 8.13 - 8.15 min (scan #314 - #320)

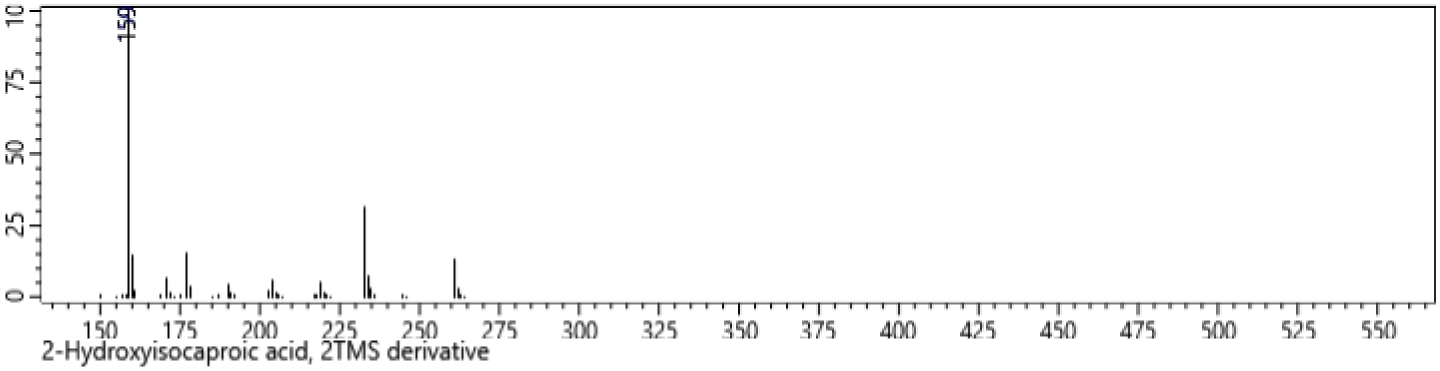

2-Hydroxyisocaproic acid, 2TMS derivative

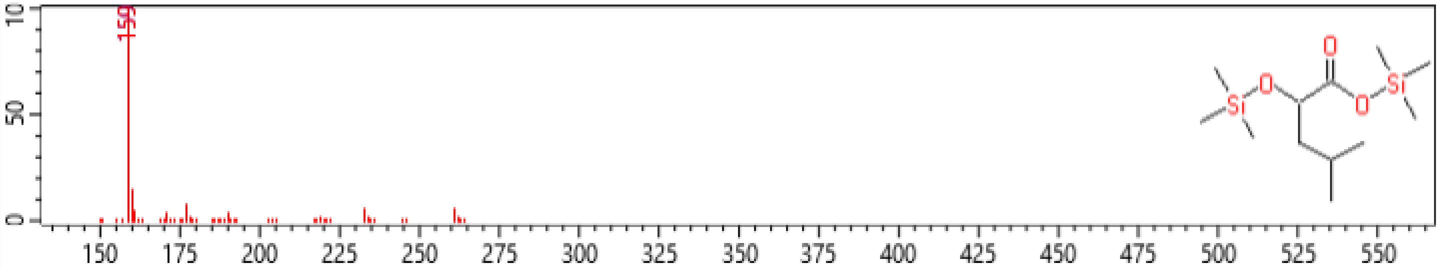

2-Hydroxyisocaproic acid, 2TMS derivative

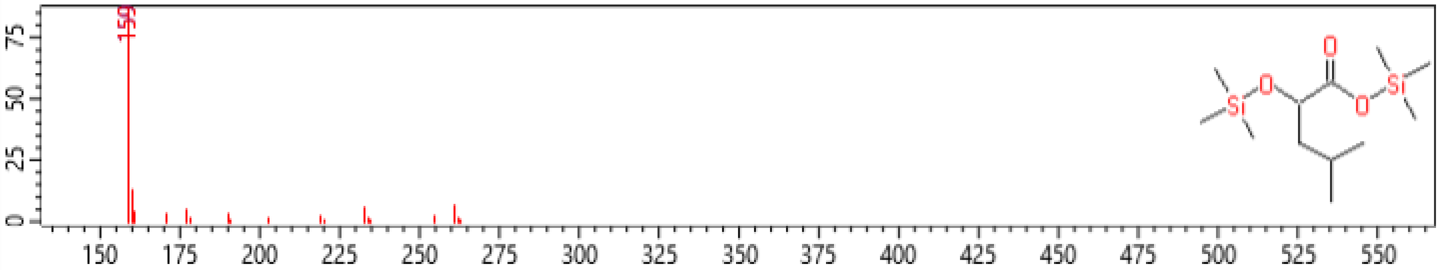

2-Hydroxyisocaproic acid, 2TMS derivative

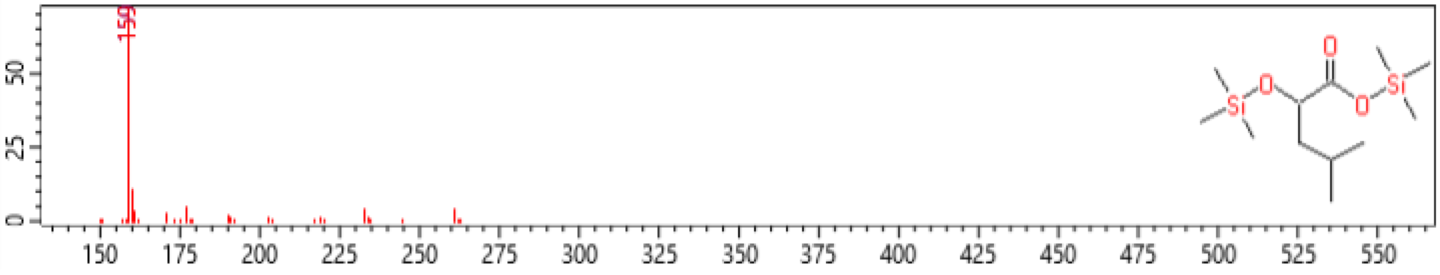

Pentanoic acid, 3-methyl-2-[(trimethylsilyl)oxy]-, trimethylsilyl ester

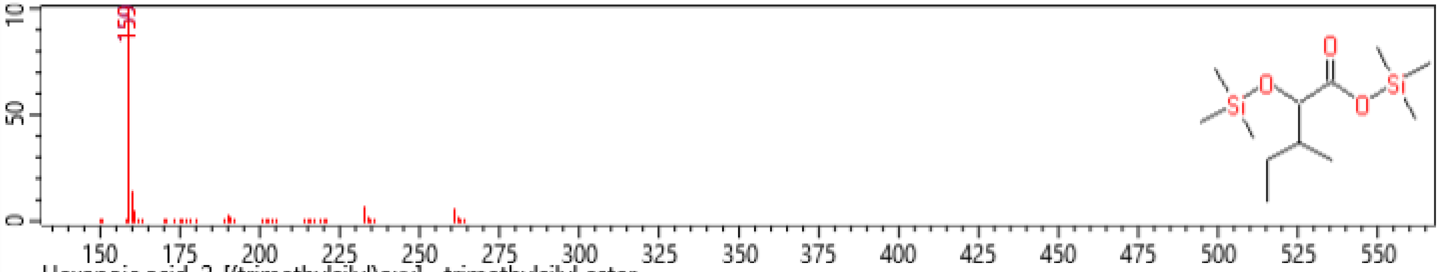

Hexanoic acid, 2-[(trimethylsilyl)oxy]-, trimethylsilyl ester

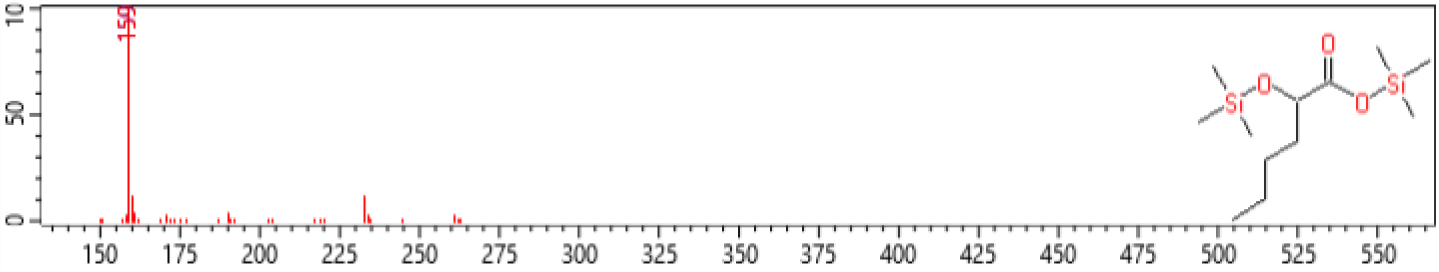

3-Ethylphenol, TMS derivative

Peak: 8.16 - 8.19 min (scan #323 - #333) Subtracted noise: 8.08 - 8.12 min (scan #302 - #312), 8.21 - 8.24 min (scan #337 - #347)

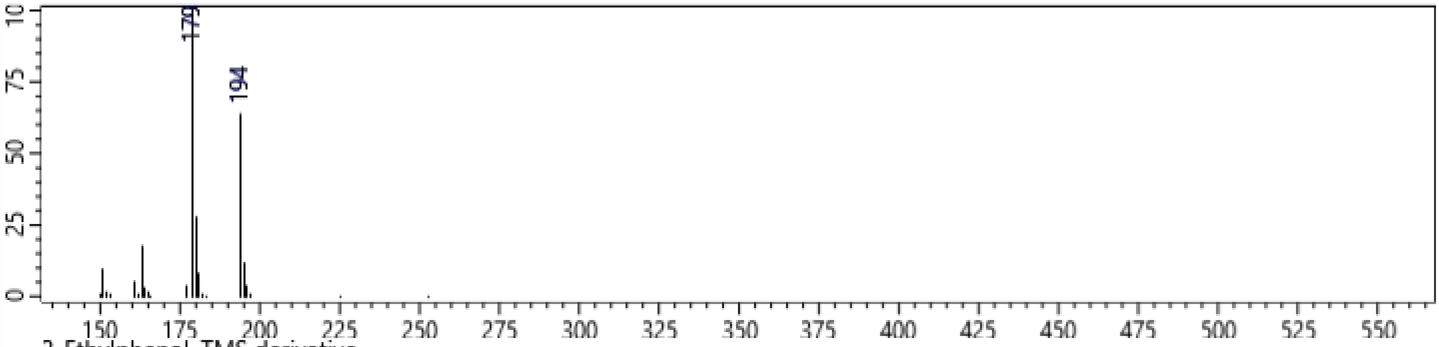

3-Ethylphenol, TMS derivative

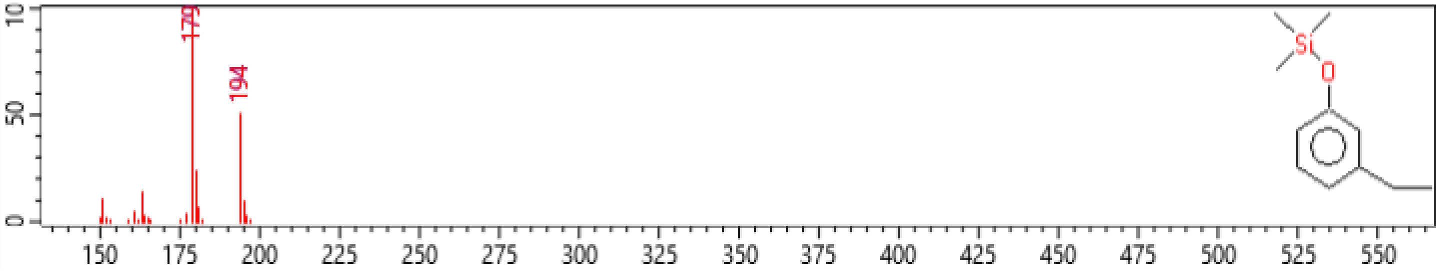

2-Ethylphenol, TMS derivative

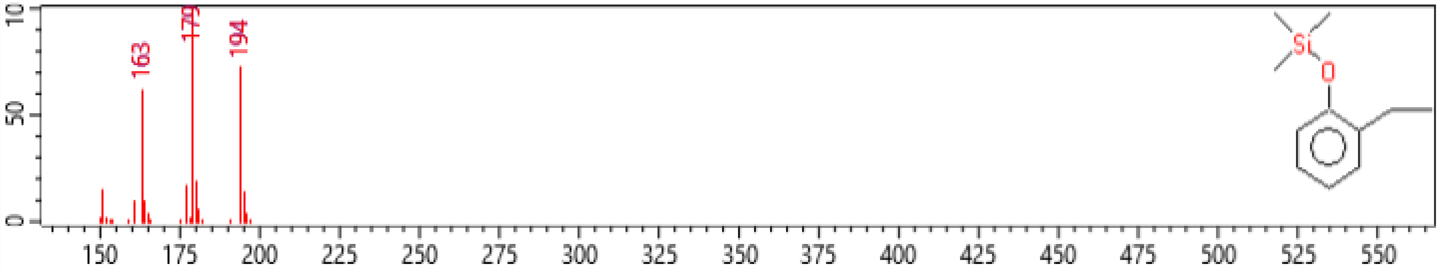

2-Ethylphenol, TMS derivative

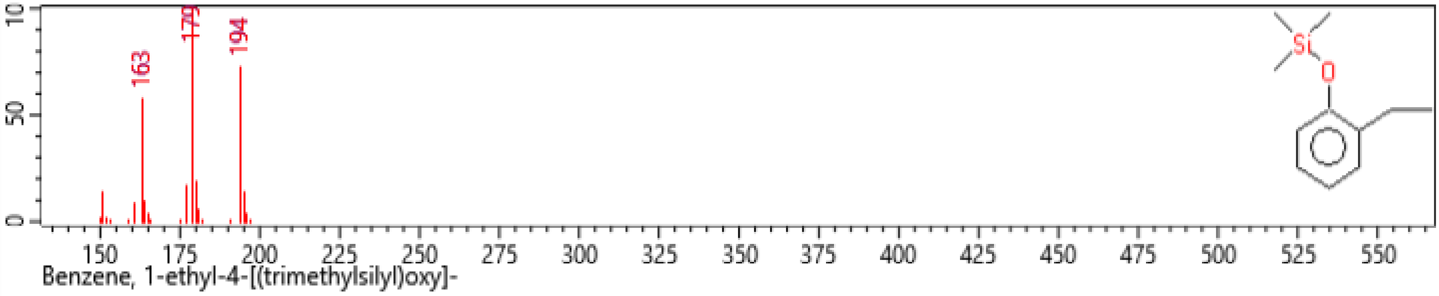

Benzene, 1-ethyl-4-[(trimethylsilyl)oxy]-

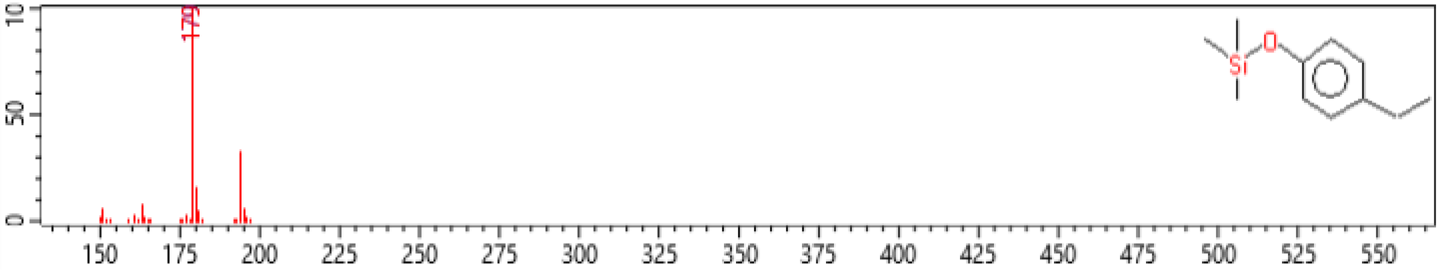

3,5-Dimethylphenol, TMS derivative

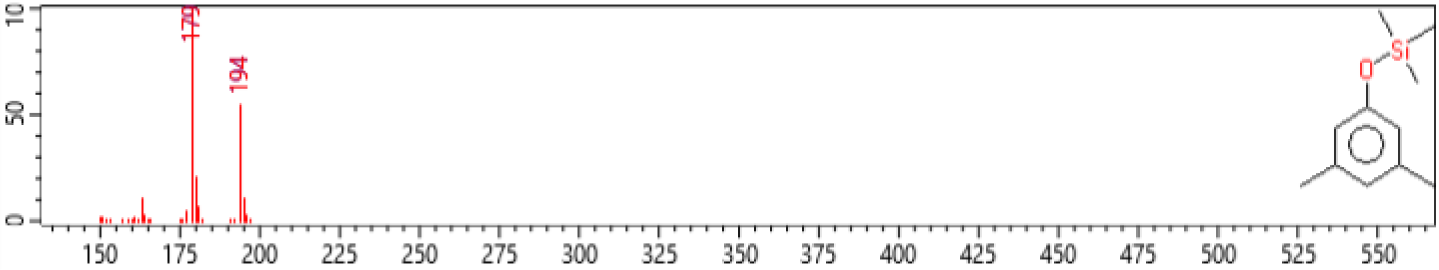

Succinic acid, monoethyl ester-, (TMS)

Peak: 8.21 - 8.23 min (scan #339 - #343) Subtracted noise: 8.19 - 8.20 min (scan #331 - #336), 8.25 - 8.26 min (scan #348 - #352)

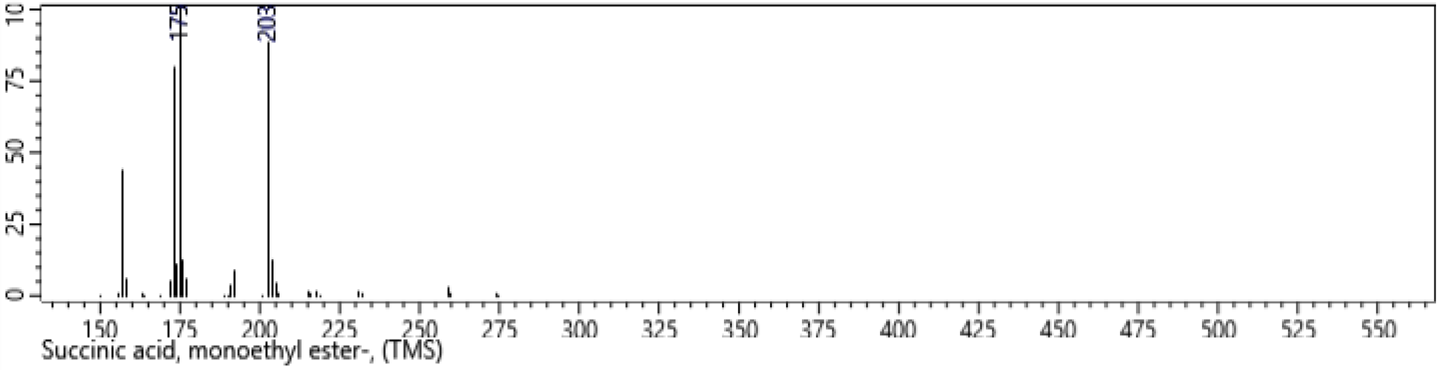

Succinic acid, monoethyl ester-, (TMS)

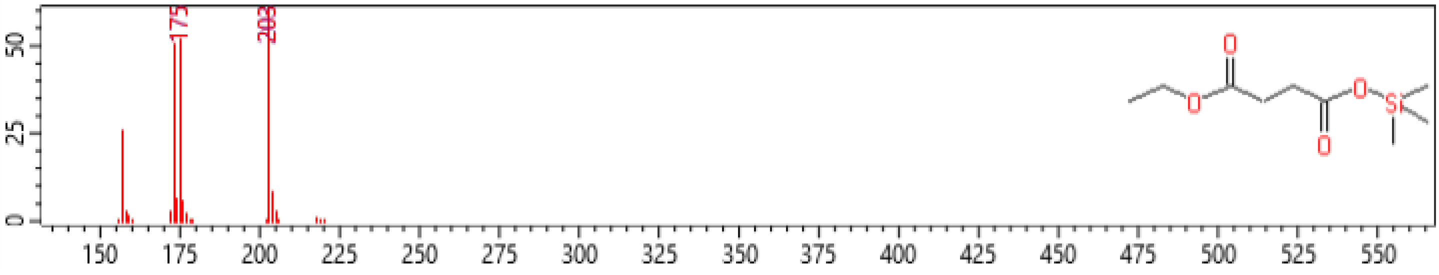

Succinic acid, monoethyl ester-, (TMS)

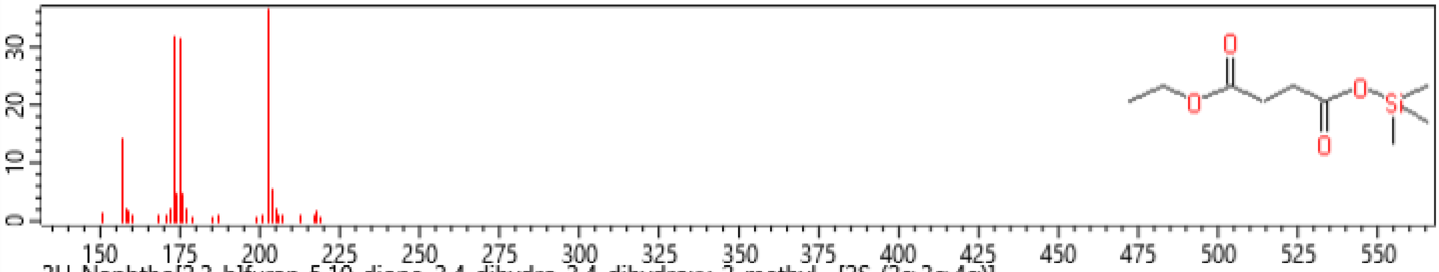

2H-Naphtho[2,3-b]furan-5,10-dione, 3,4-dihydro-3,4-dihydroxy-2-methyl-, [2S-(2α,3α,4α)]-

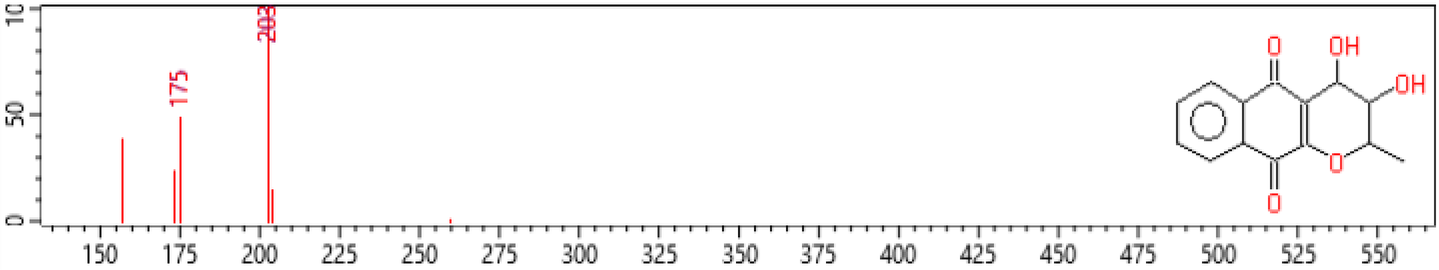

Silane, dimethyl(2-pentyloxy)butoxy-

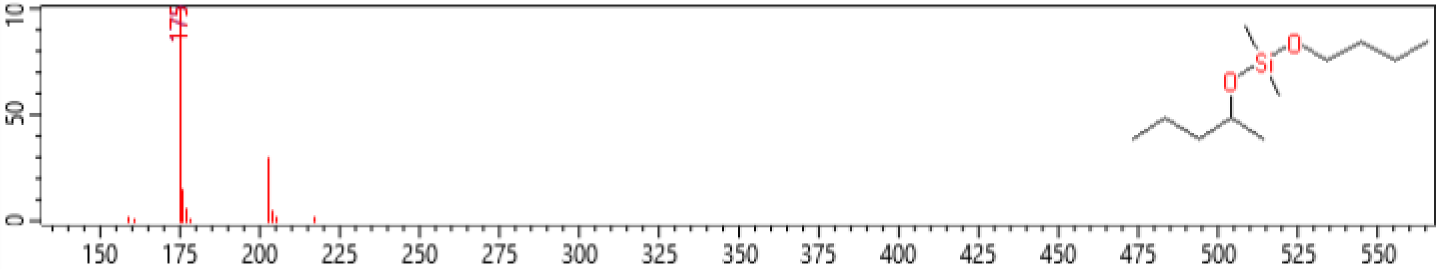

Disiloxane, pentaethylmethyl-

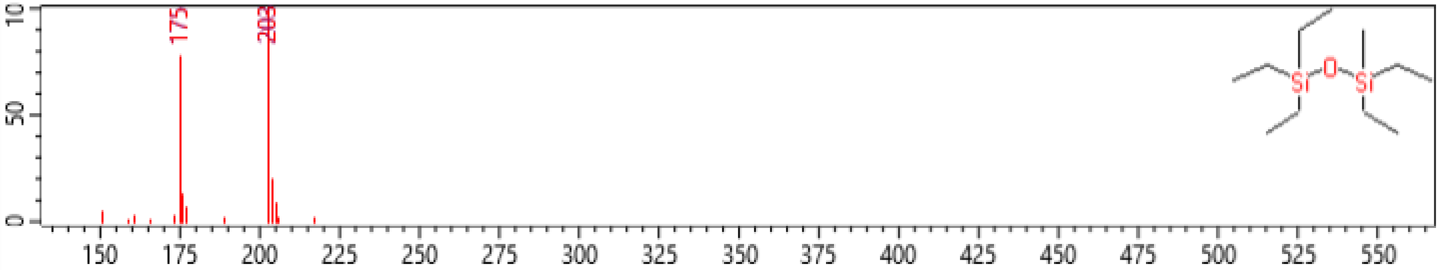

Silacycloundec-6-yne, 1,1-dimethyl-

Peak: 8.24 - 8.27 min (scan #347 - #353) Subtracted noise: 8.22 - 8.24 min (scan #341 - #347), 8.30 - 8.32 min (scan #362 - #368)

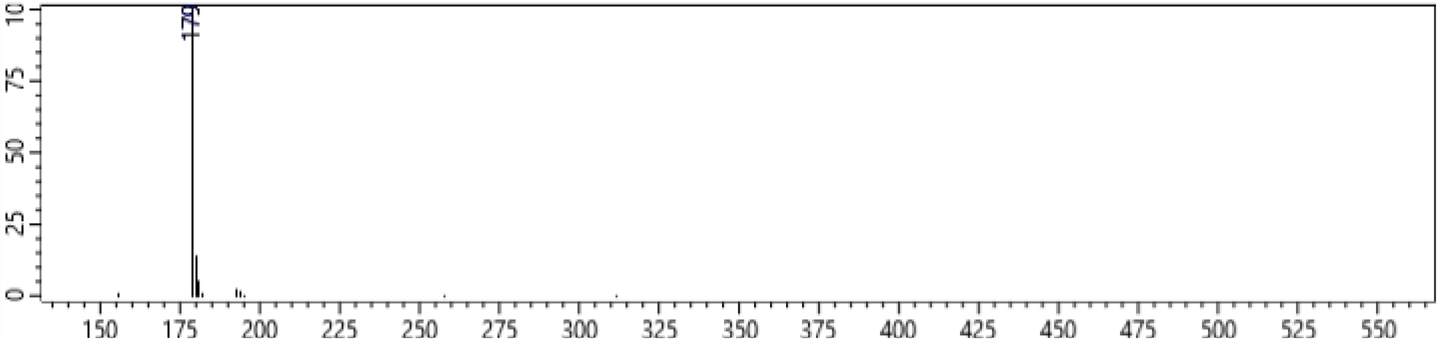

Silacycloundec-6-yne, 1,1-dimethyl-

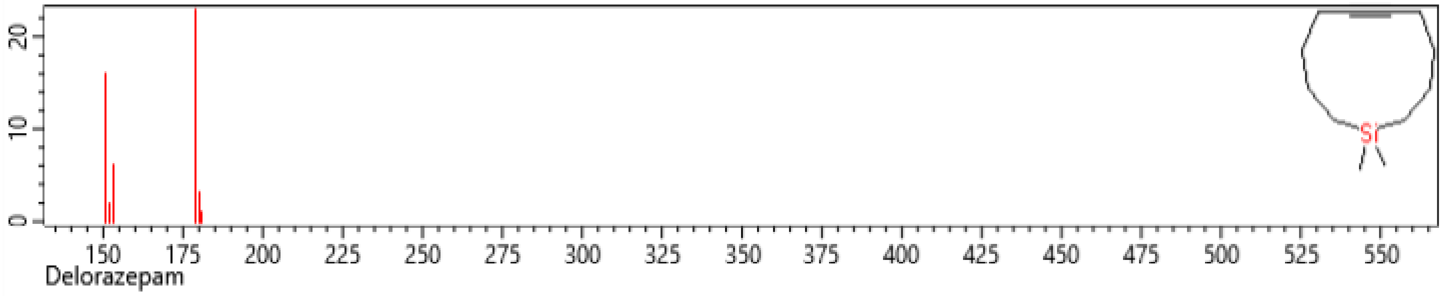

Delorazepam

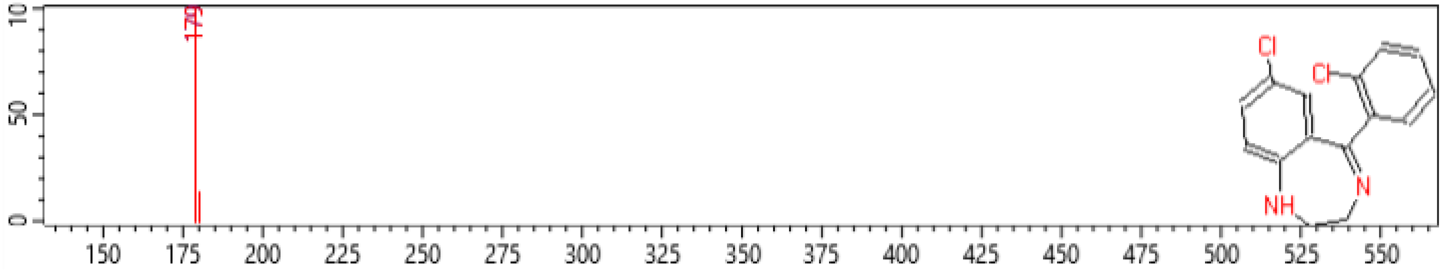

Benzoic Acid, TBDMS derivative

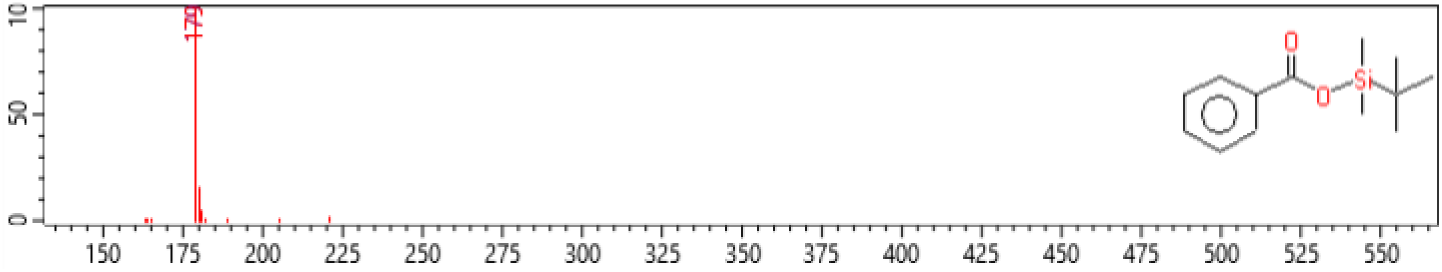

N,N-Dimethyl phenyl(thioacetamide)

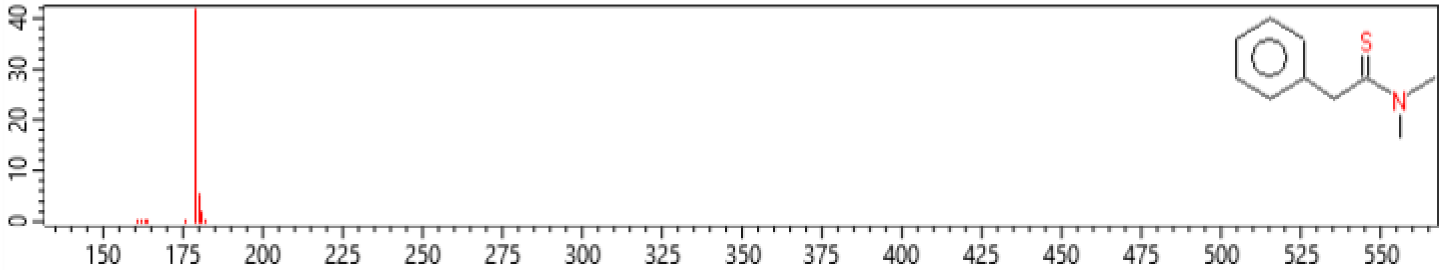

Benzene, 1-ethoxy-4-isothiocyanato-

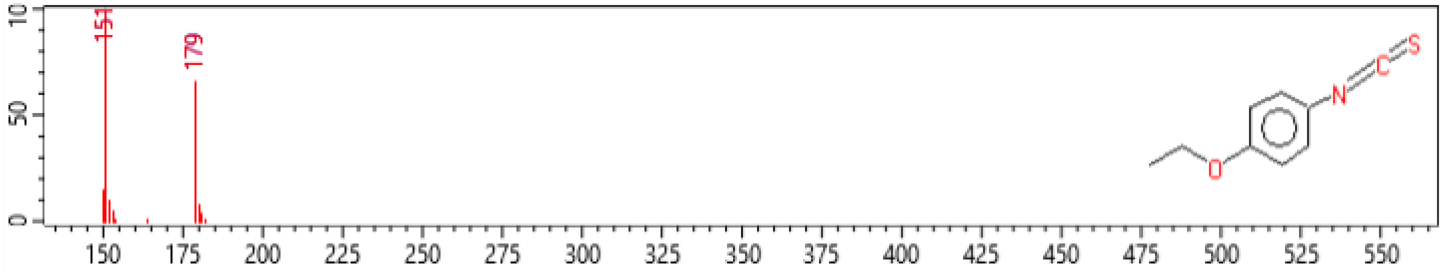

Glycerol, 3TMS derivative

Peak: 8.55 - 8.62 min (scan #433 - #451) Subtracted noise: 8.44 - 8.51 min (scan #403 - #420), 8.64 - 8.71 min (scan #458 - #476)

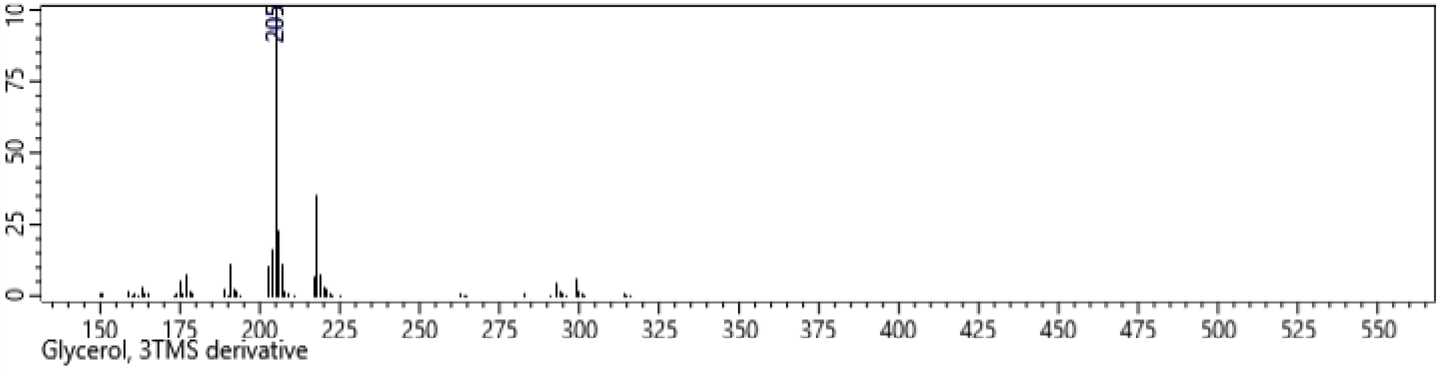

Glycerol, 3TMS derivative

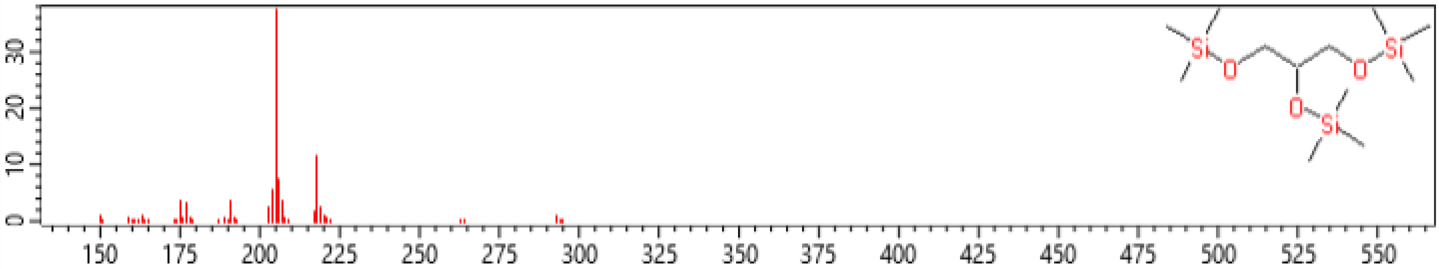

Glycerol, 3TMS derivative

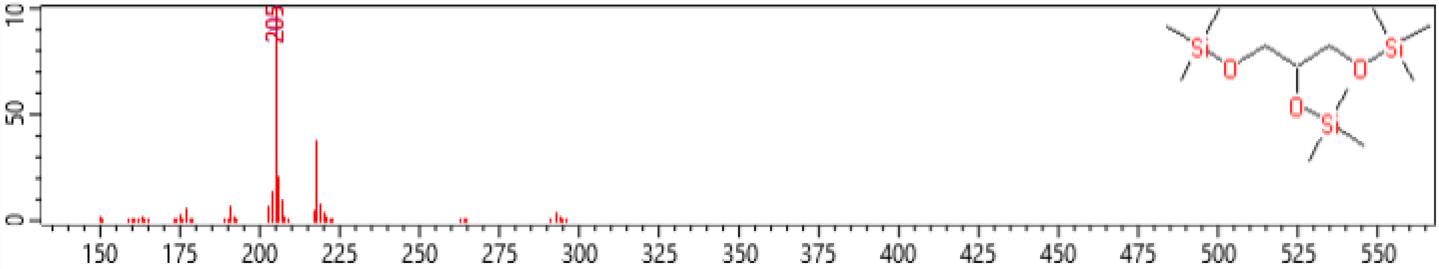

Glycerol, 3TMS derivative

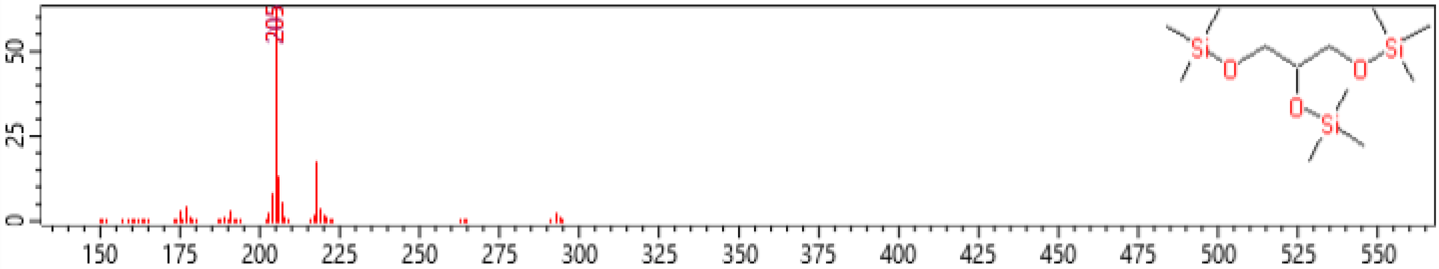

Glycerol, 3TMS derivative

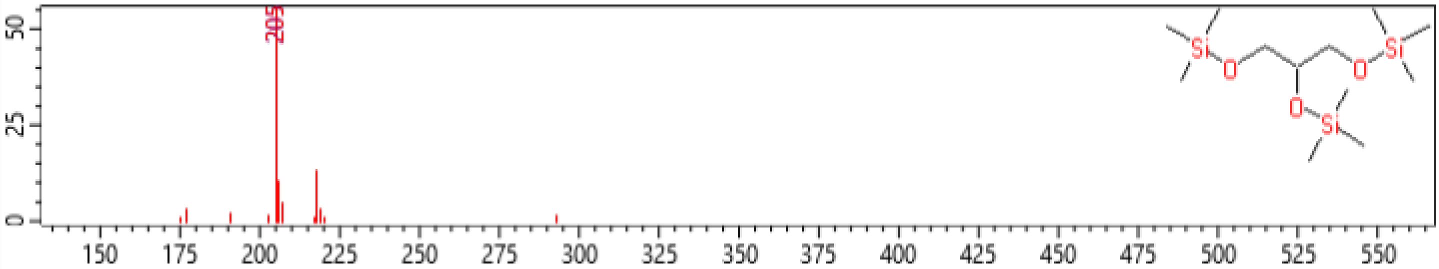

2-Thiophenecarboxaldehyde, 4-bromo-5-ethyl-

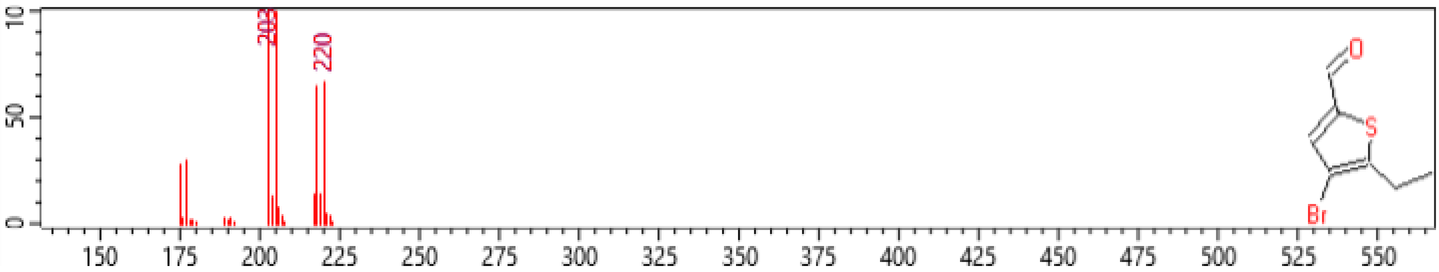

Maltol, TMS derivative

Peak: 8.69 - 8.71 min (scan #472 - #476) Subtracted noise: 8.66 - 8.67 min (scan #464 - #467), 8.72 - 8.73 min (scan #479 - #483)

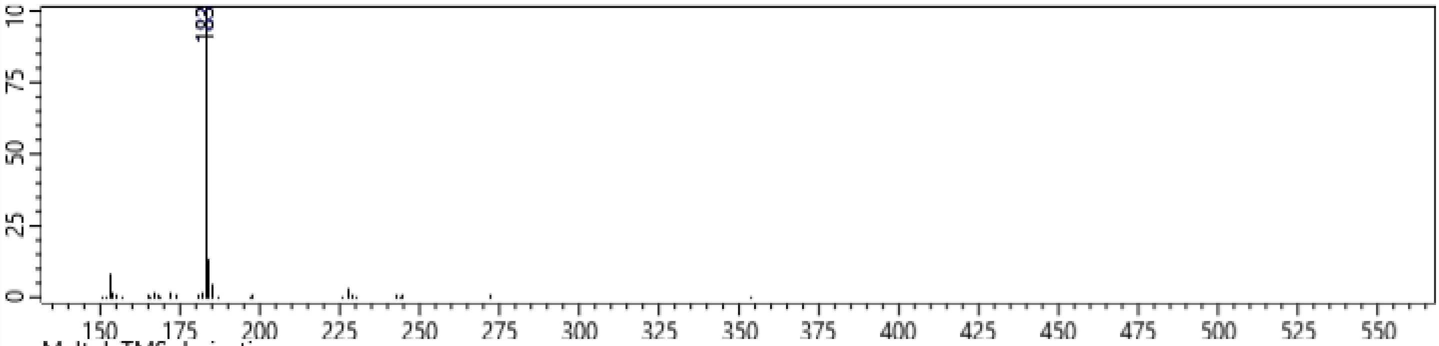

Maltol, TMS derivative

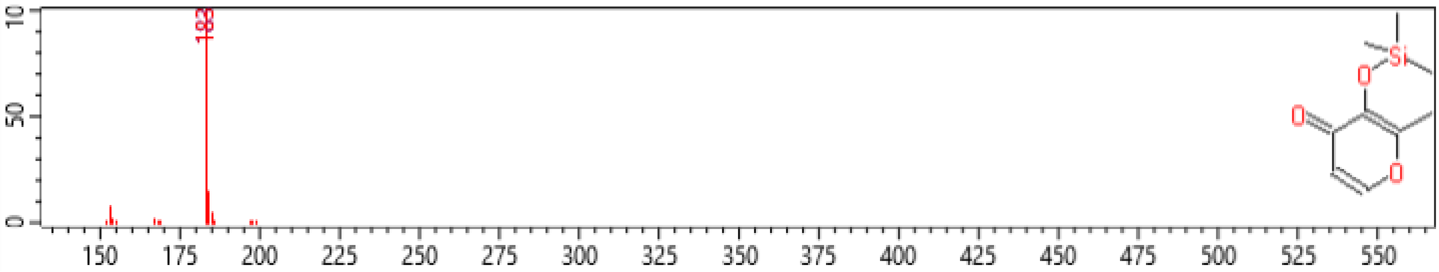

2-Methyl-4-oxo-3,4-dihydro-2H-pyran-3-yl isobutyrate

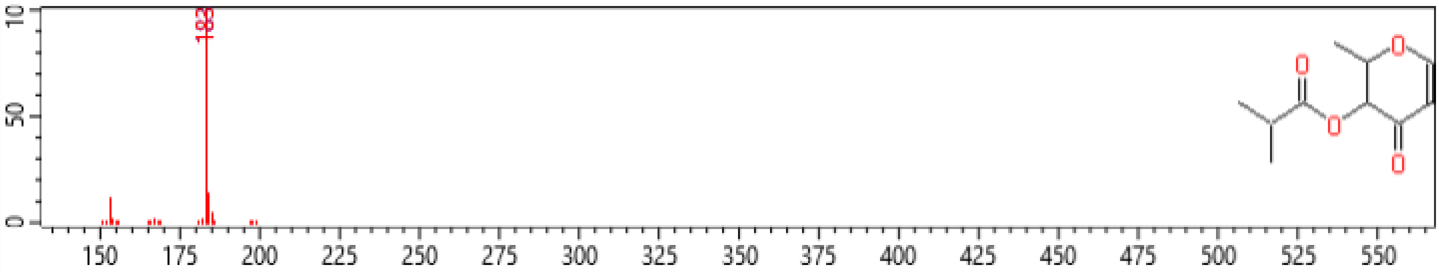

Maltol, TBDMS derivative

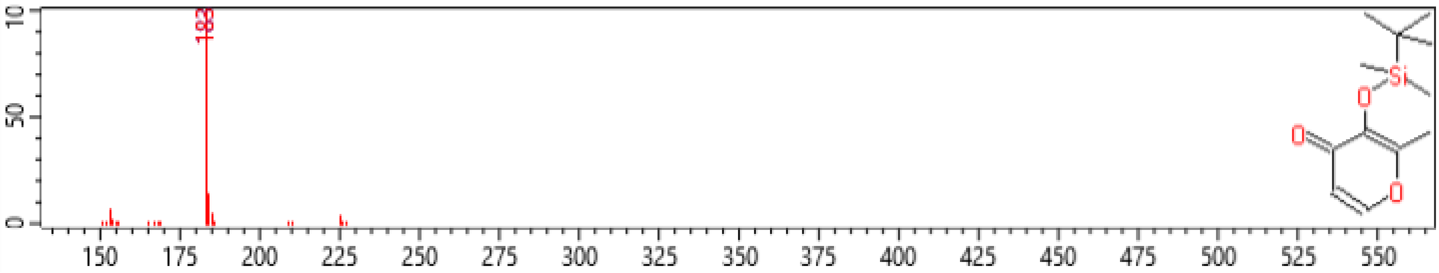

5,8-Methano-4H-3,1-benzoxazine-2-thione, 1,2,4a-rel,5-trans,6,7,8-trans,8a-cis-octahydro-

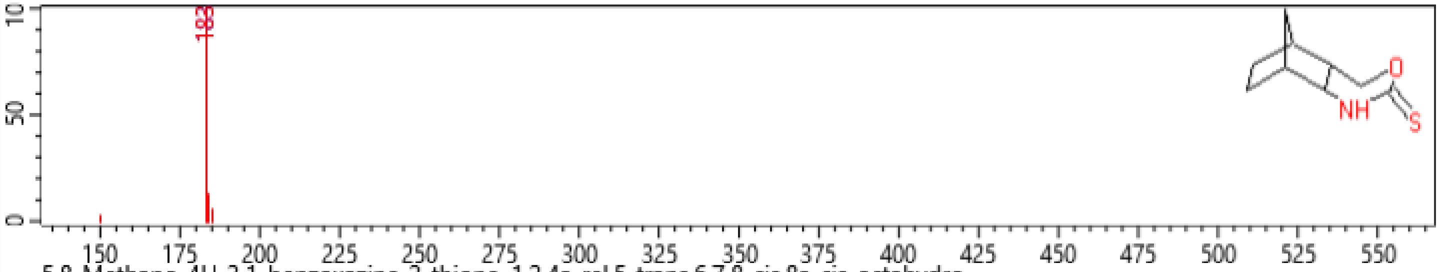

5,8-Methano-4H-3,1-benzoxazine-2-thione, 1,2,4a-rel,5-trans,6,7,8-cis,8a-cis-octahydro-

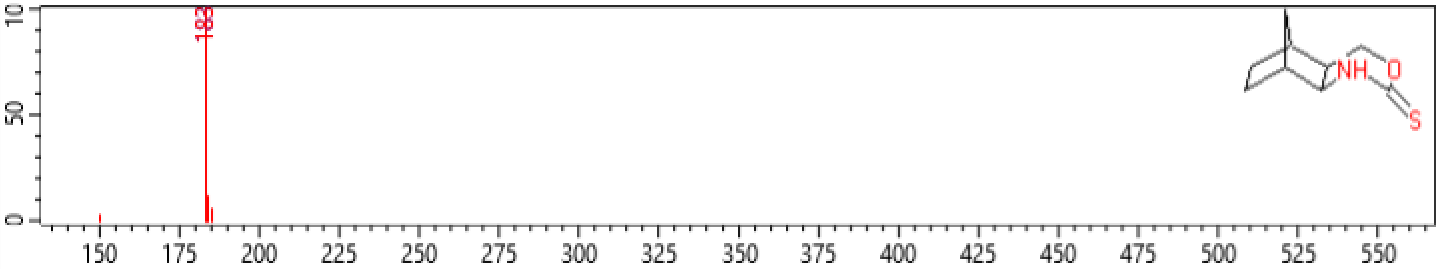

Pyrazine-2-carboxamide, N-tert-butyldimethylsilyl

Peak: 8.77 - 8.79 min (scan #494 - #500) Subtracted noise: 8.74 - 8.76 min (scan #485 - #490), 8.81 - 8.83 min (scan #505 - #510)

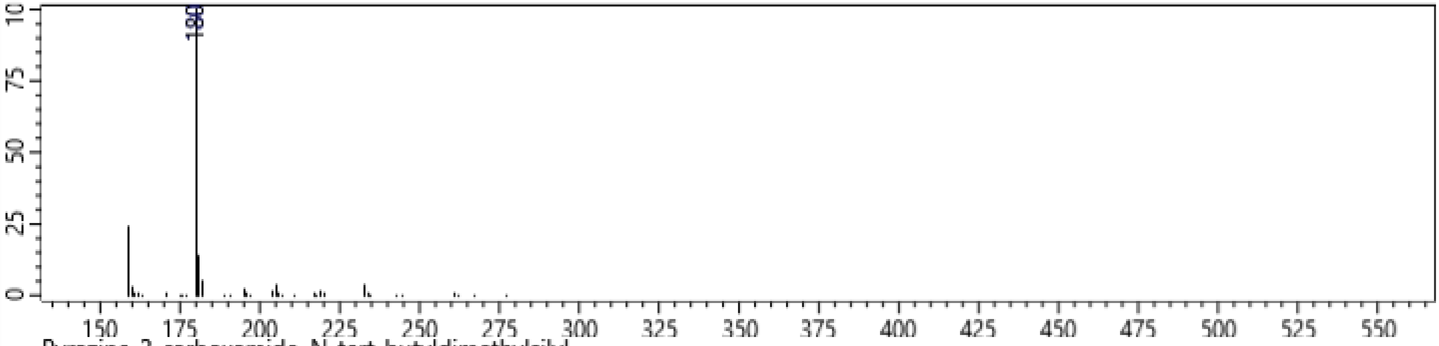

Pyrazine-2-carboxamide, N-tert-butyldimethylsilyl

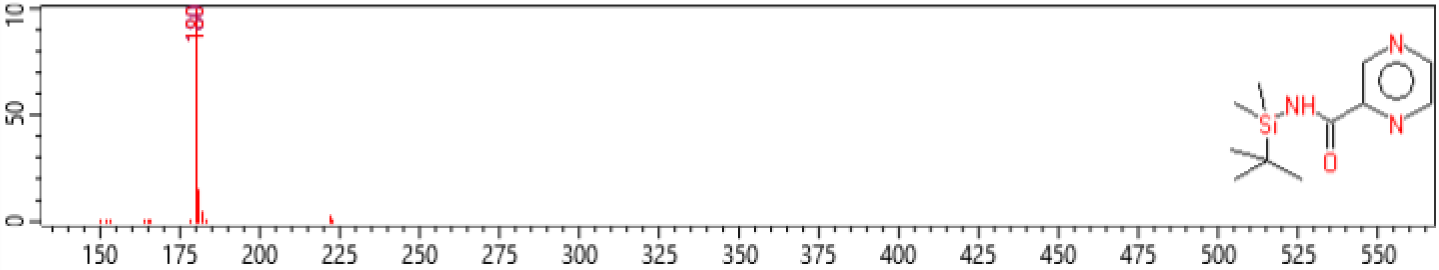

4-Chloro-7-hydroxy-3-methyl-1-indanone

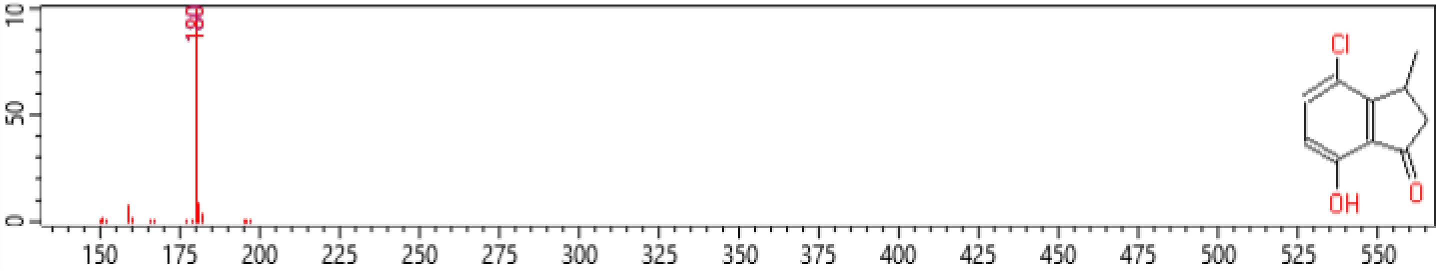

(2-Bromo-1-phenylvinyl)benzene

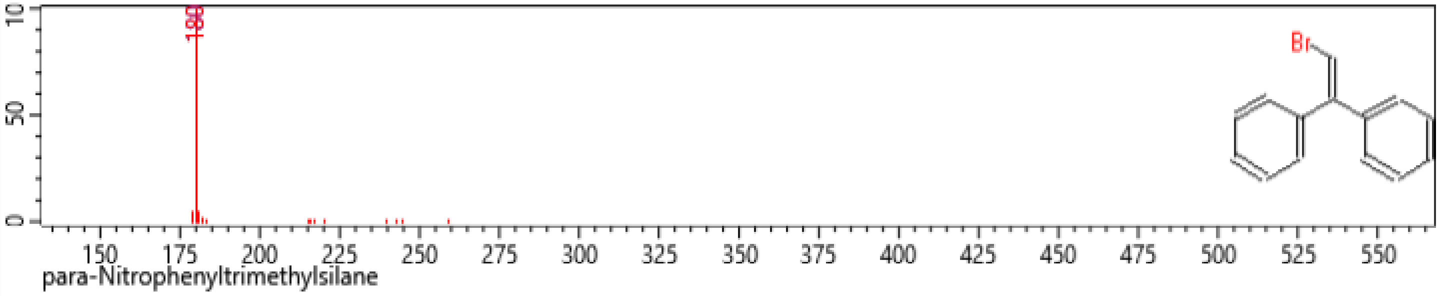

para-Nitrophenyltrimethylsilane

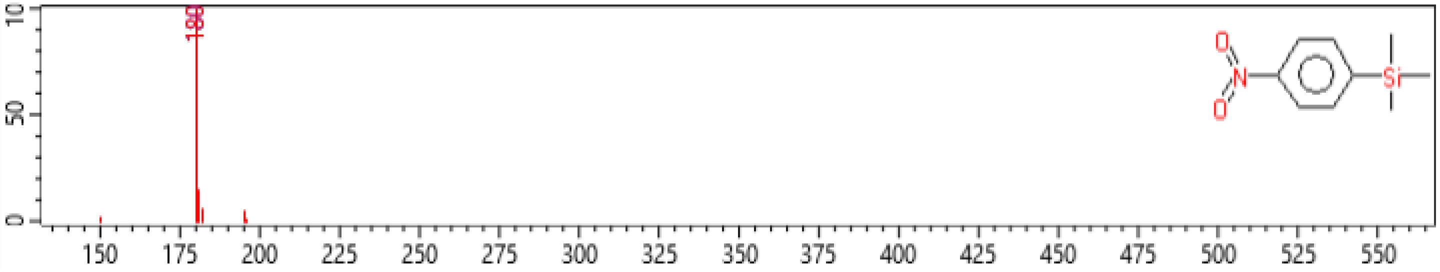

Niacin, TBDMS derivative

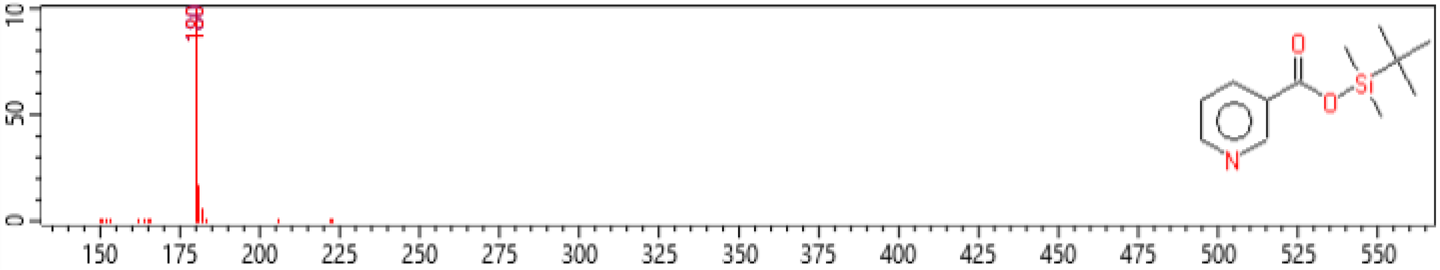

Benzeneacetic acid, TMS derivative

Peak: 8.82 - 8.83 min (scan #508 - #512) Subtracted noise: 8.79 - 8.81 min (scan #499 - #504), 8.85 - 8.86 min (scan #516 - #520)

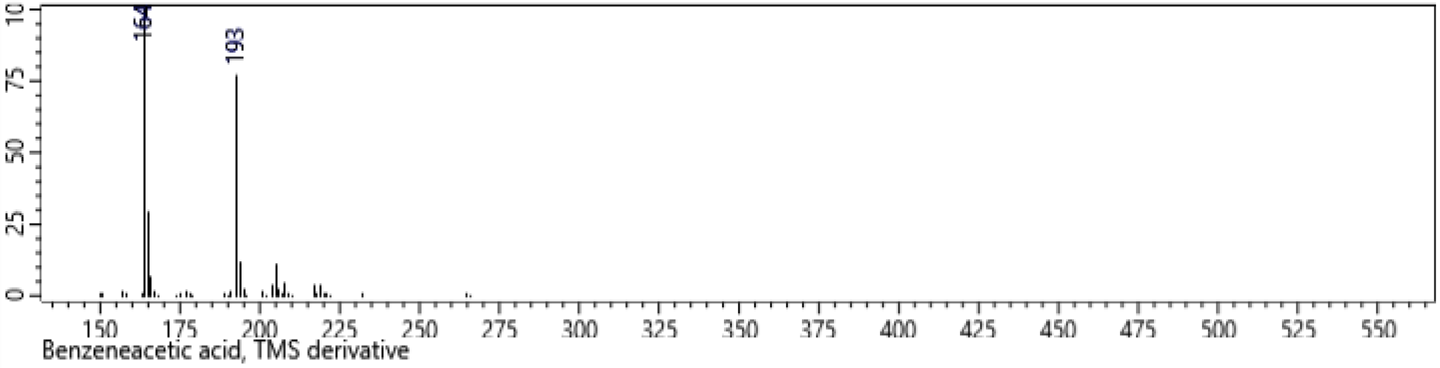

Benzeneacetic acid, TMS derivative

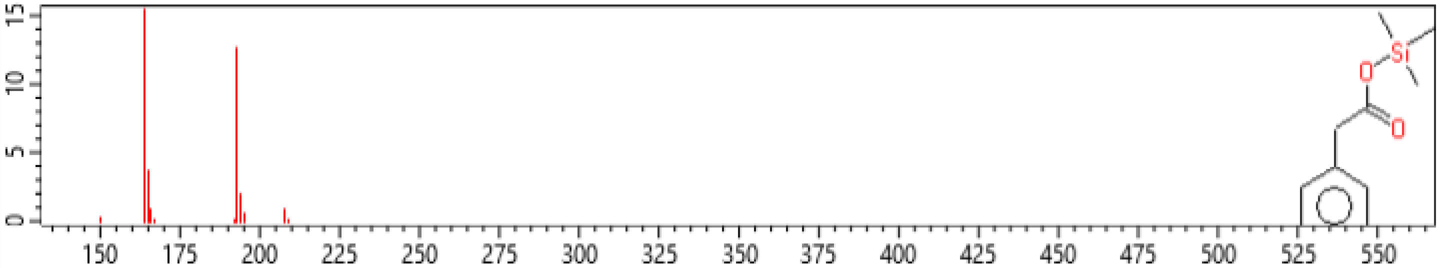

Benzeneacetic acid, TMS derivative

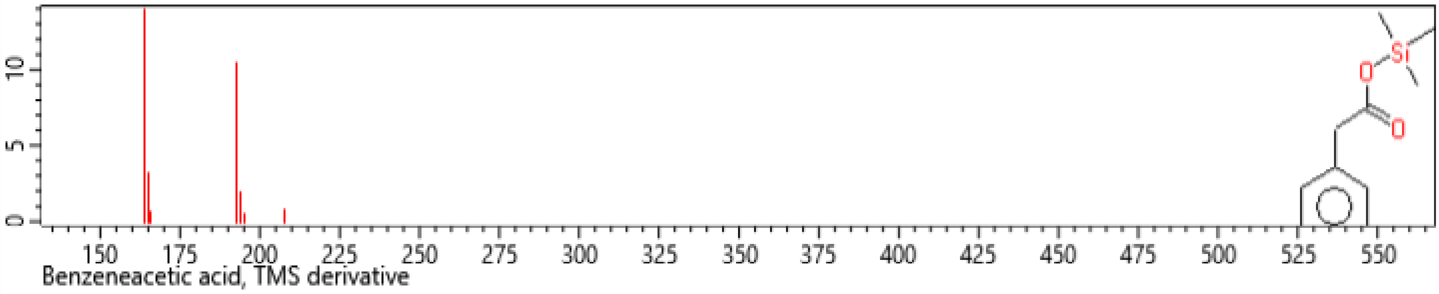

Benzeneacetic acid, TMS derivative

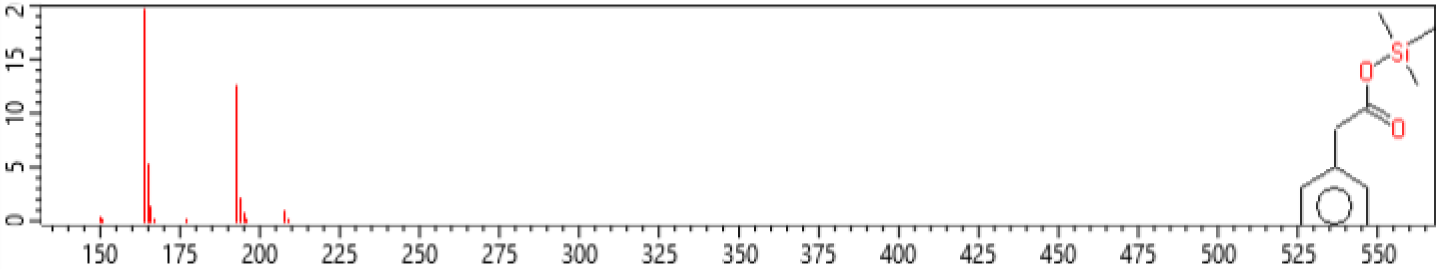

Benzeneacetic acid, TMS derivative

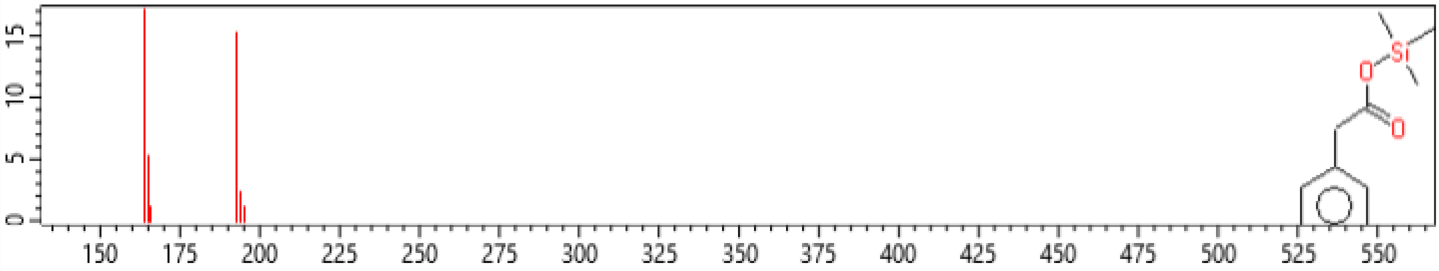

Benzeneacetic acid, TMS derivative

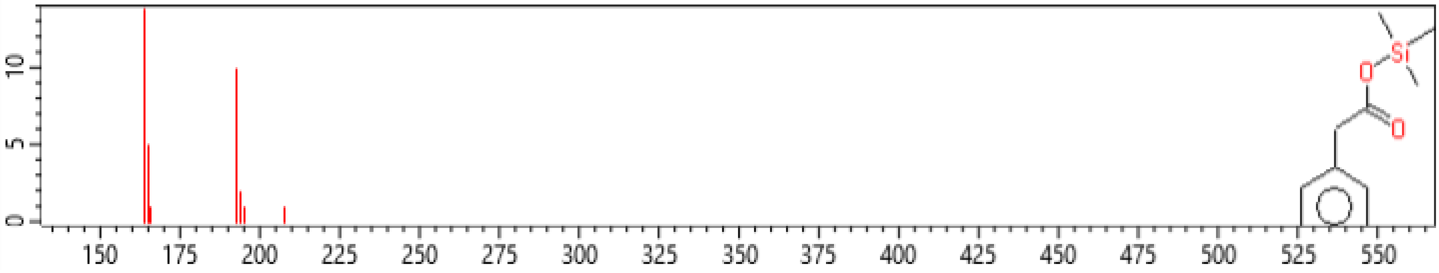

Butanedioic acid, 2TMS derivative

Peak: 9.03 - 9.09 min (scan #566 - #582) Subtracted noise: 8.91 - 8.96 min (scan #533 - #548), 9.09 - 9.14 min (scan #582 - #598)

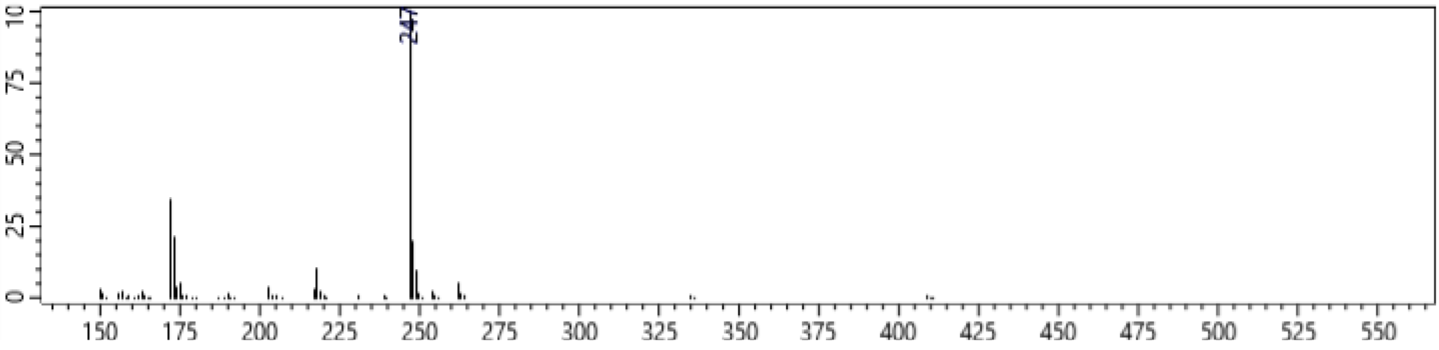

Butanedioic acid, 2TMS derivative

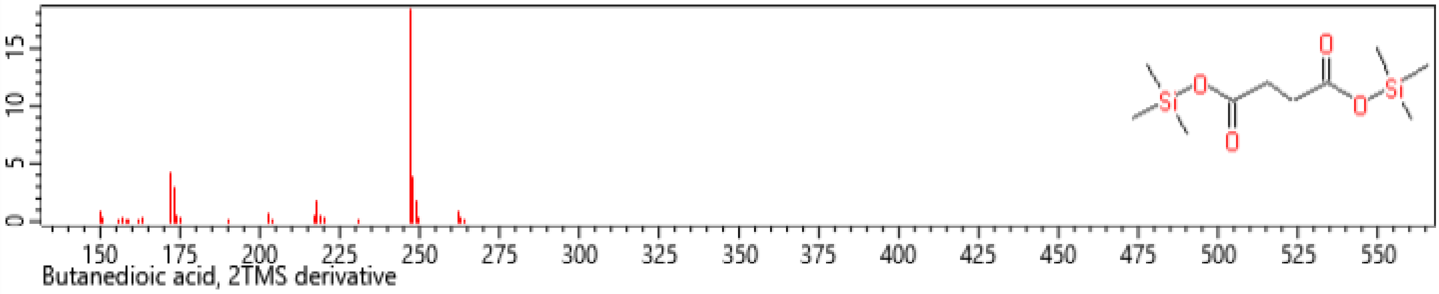

Butanedioic acid, 2TMS derivative

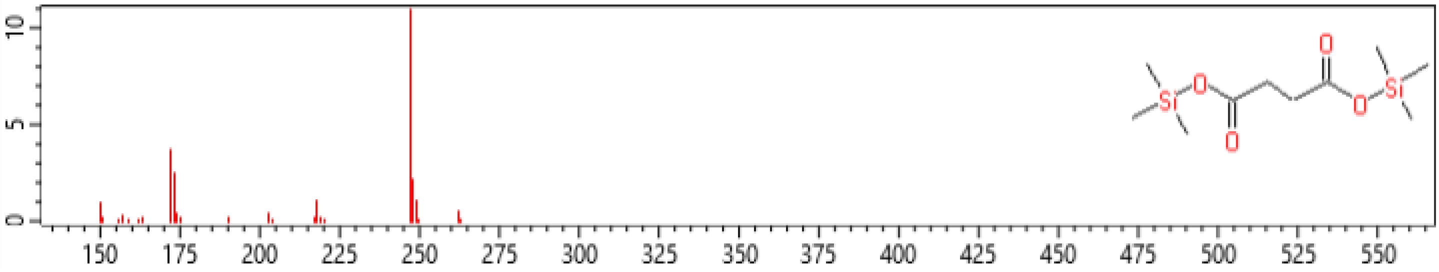

Butanedioic acid, 2TMS derivative

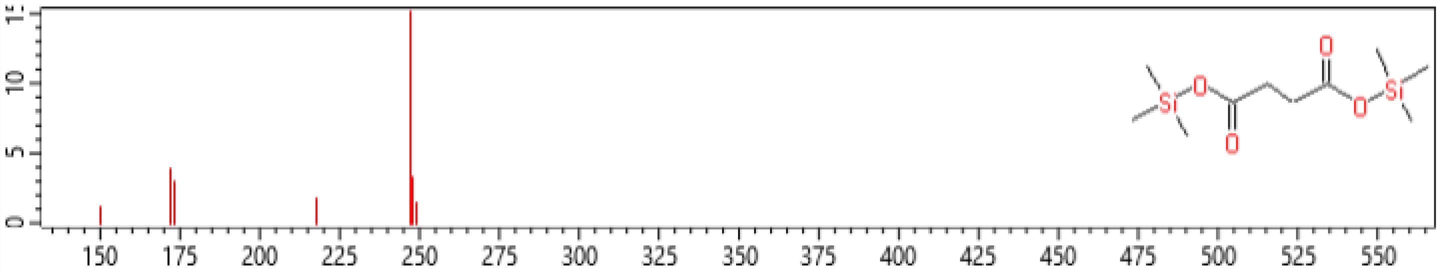

Butanedioic acid, 2TMS derivative

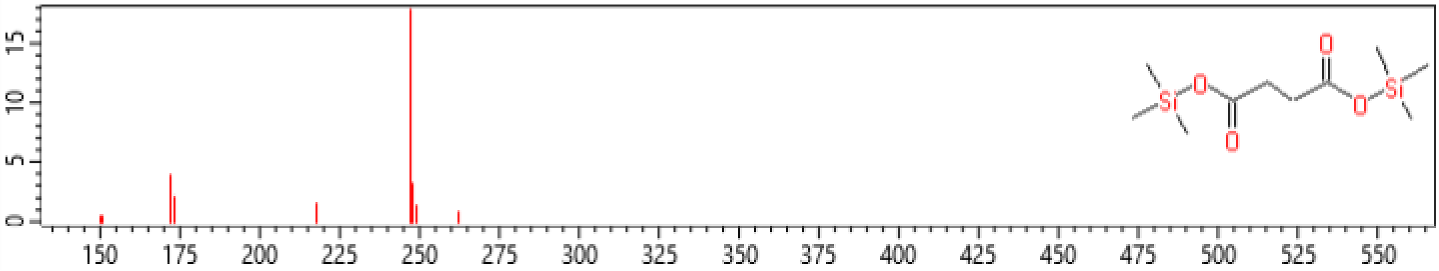

Butanedioic acid, 2TMS derivative

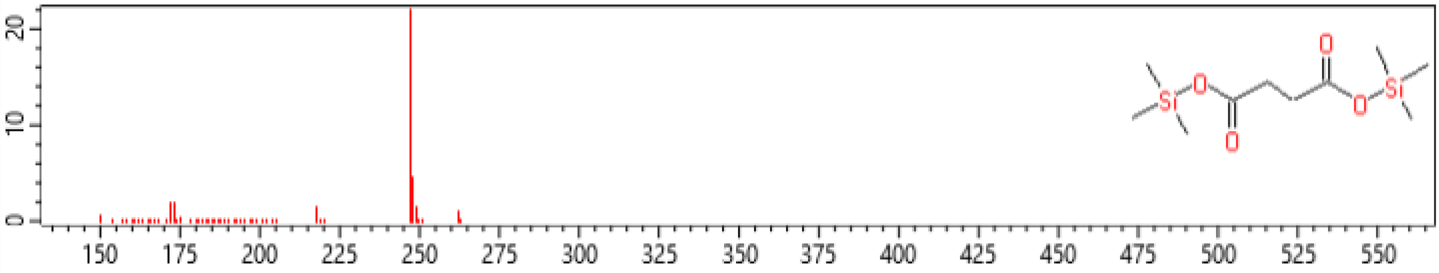

Methylsuccinic acid, 2TMS derivative

Peak: 9.14 - 9.15 min (scan #597 - #599) Subtracted noise: 9.13 - 9.13 min (scan #593 - #595), 9.15 - 9.16 min (scan #600 - #602)

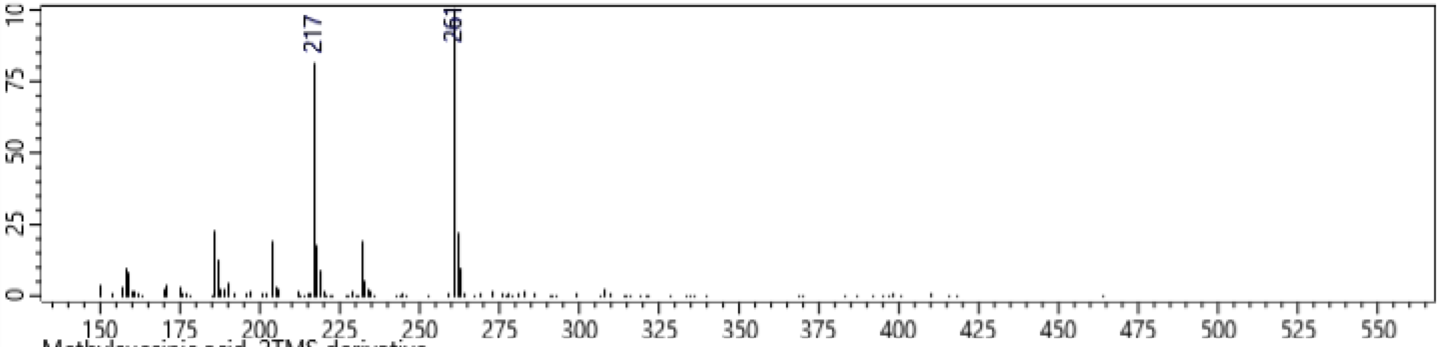

Methylsuccinic acid, 2TMS derivative

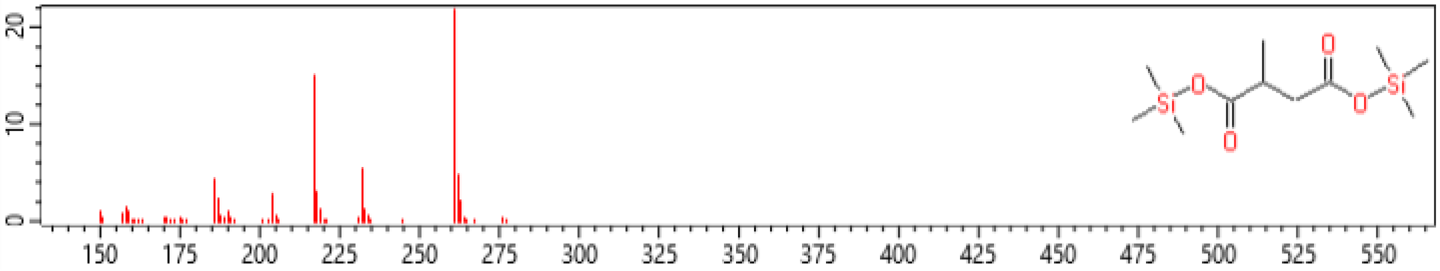

Methylsuccinic acid, 2TMS derivative

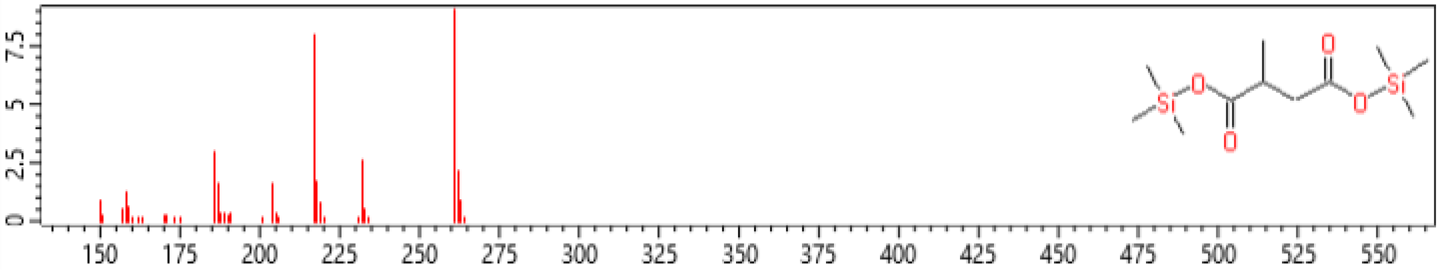

Methylsuccinic acid, 2TMS derivative

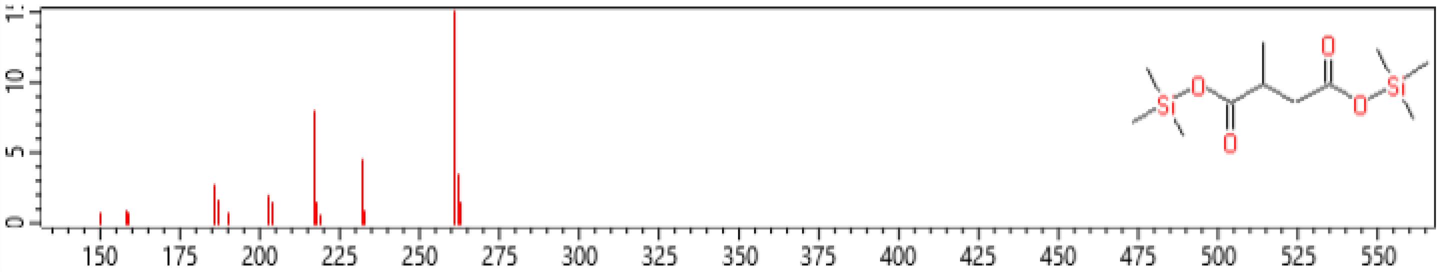

Ethylmalonic acid, 2TMS derivative

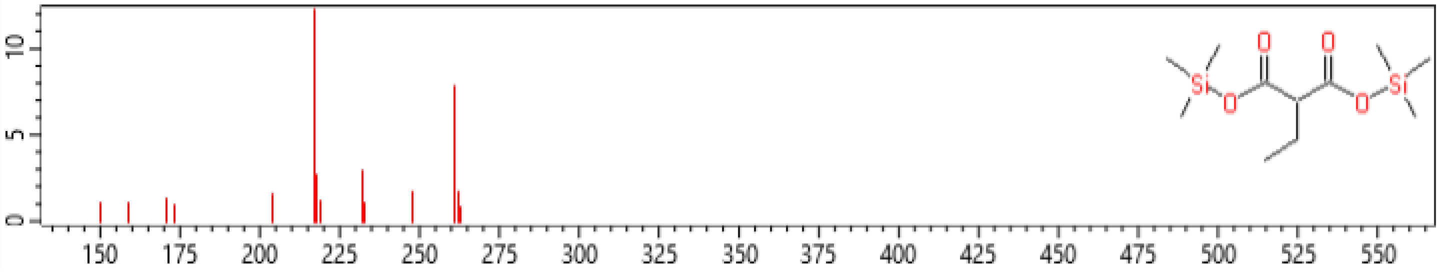

Ethylmalonic acid, 2TMS derivative

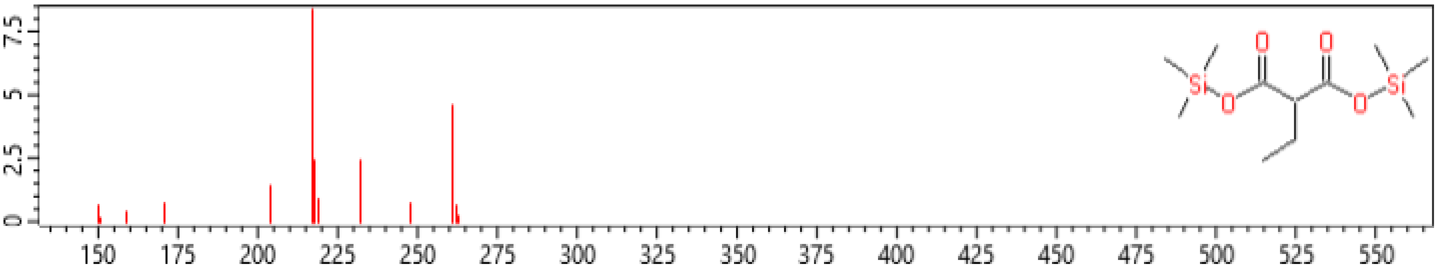

Glyceric acid, 3TMS derivative

Peak: 9.19 - 9.21 min (scan #611 - #617) Subtracted noise: 9.16 - 9.18 min (scan #602 - #608), 9.23 - 9.25 min (scan #622 - #627)

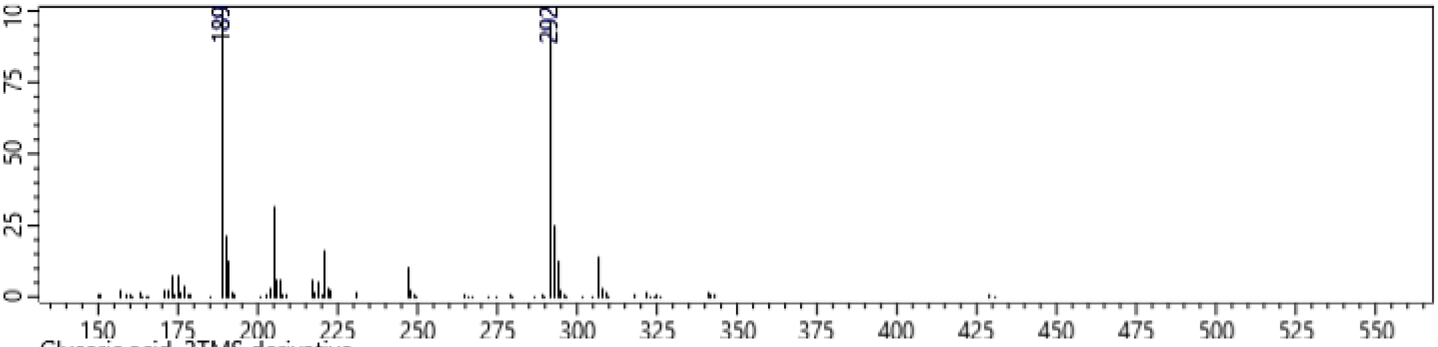

Glyceric acid, 3TMS derivative

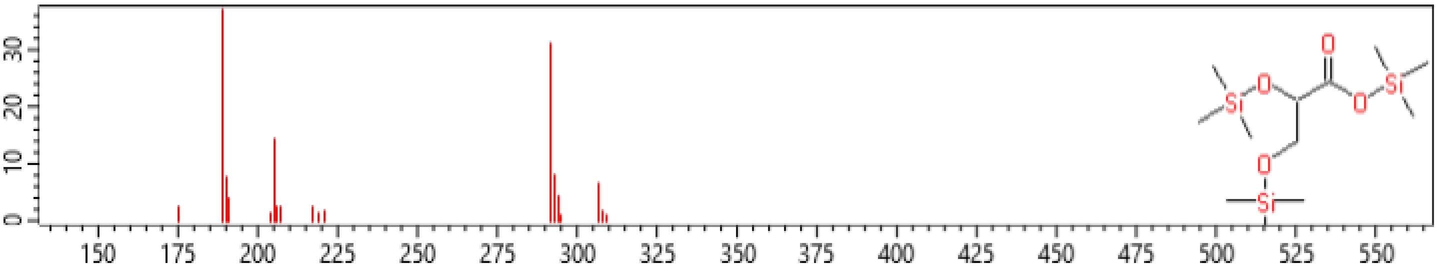

Glyceric acid, 3TMS derivative

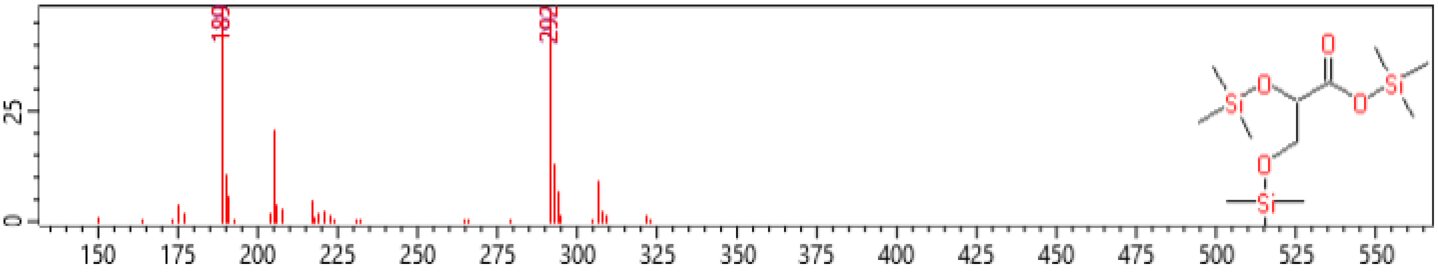

Glyceric acid, 3TMS derivative

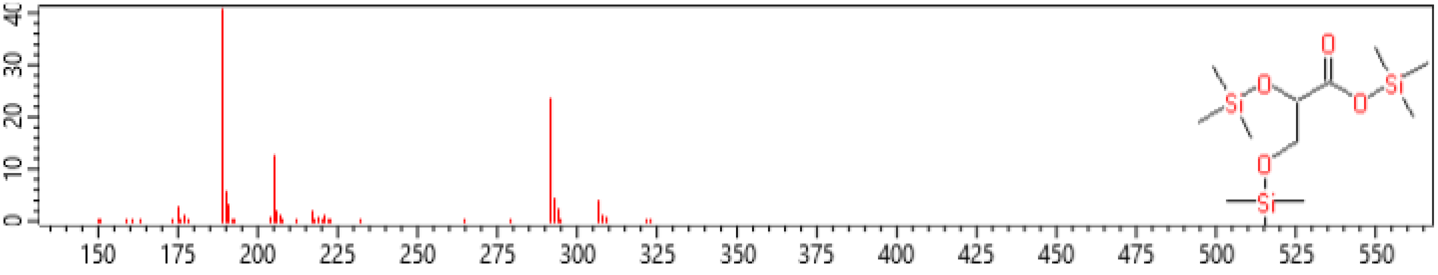

Glyceric acid, 3TMS derivative

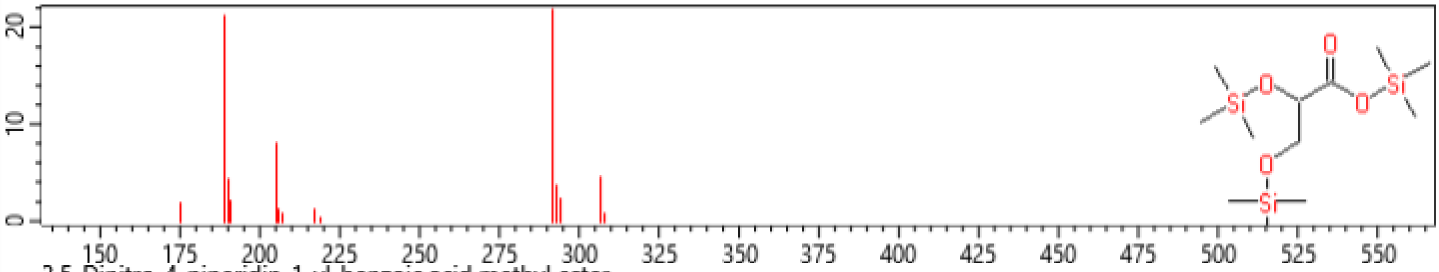

3,5-Dinitro-4-piperidin-1-yl-benzoic acid methyl ester

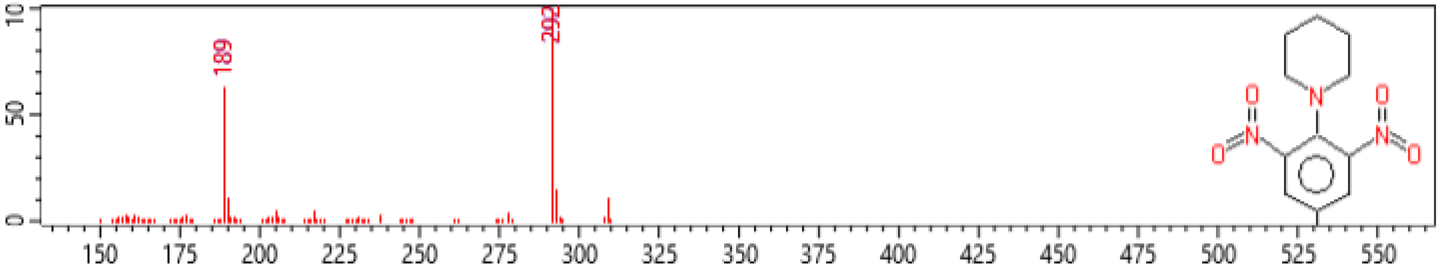

2-Butenedioic acid, (E)-, 2TMS derivative

Peak: 9.39 - 9.43 min (scan #667 - #679) Subtracted noise: 9.33 - 9.37 min (scan #650 - #662), 9.46 - 9.50 min (scan #687 - #699)

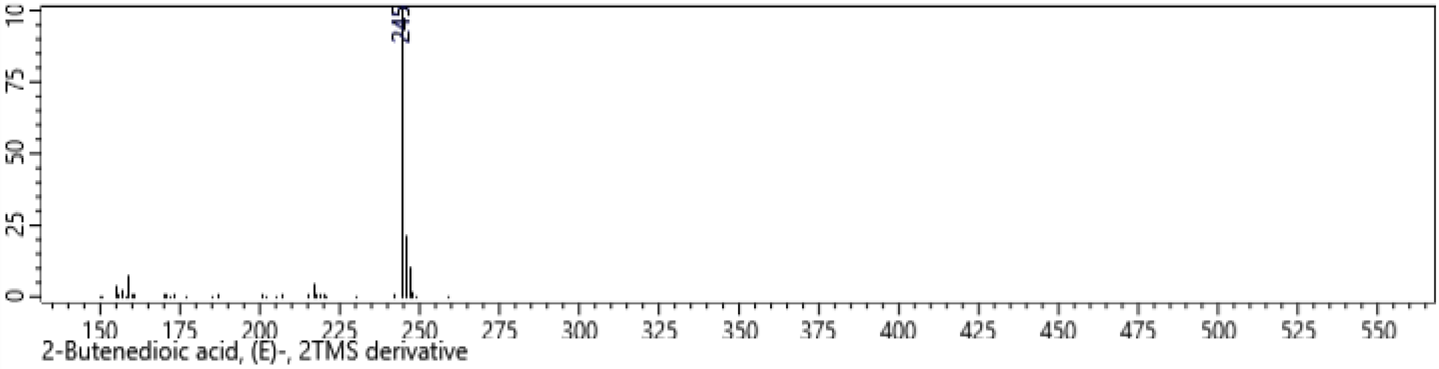

2-Butenedioic acid, (E)-, 2TMS derivative

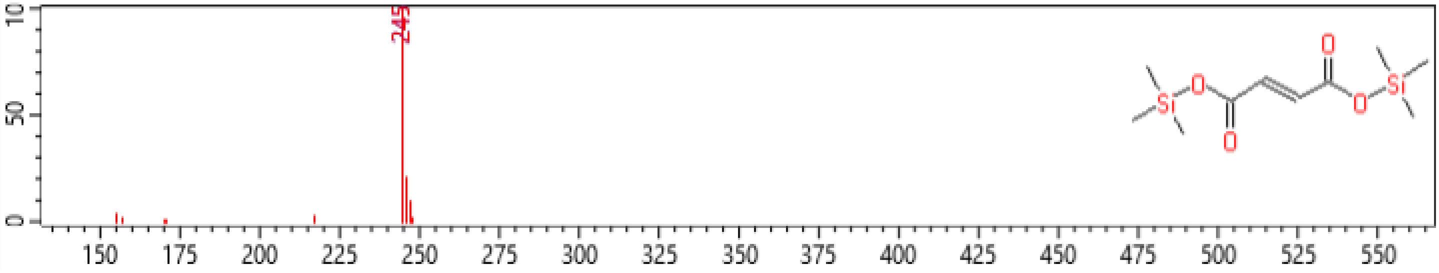

2-Butenedioic acid, (E)-, 2TMS derivative

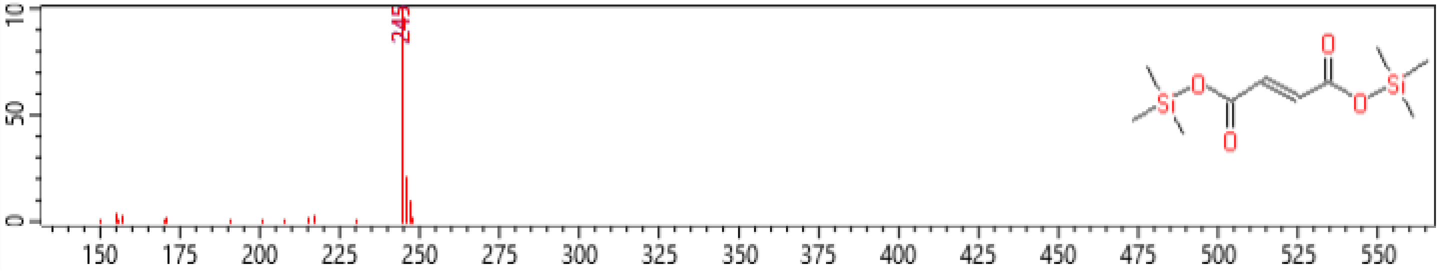

2-Butenedioic acid, (E)-, 2TMS derivative

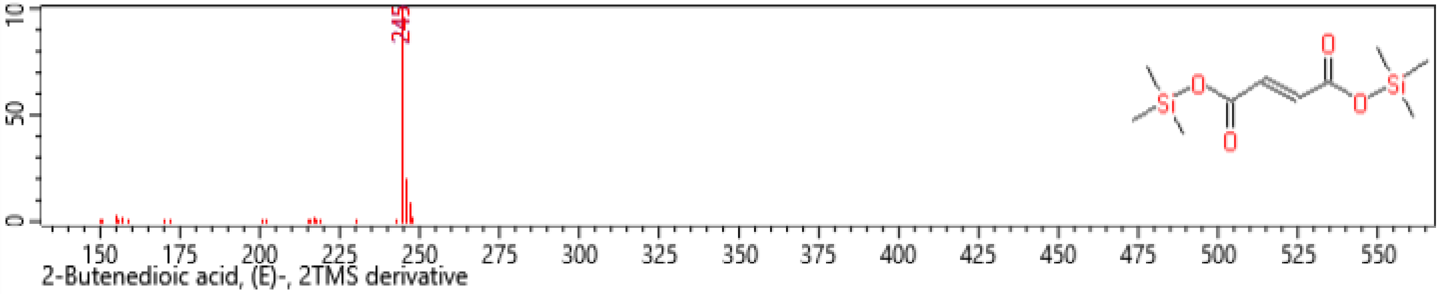

2-Butenedioic acid, (E)-, 2TMS derivative

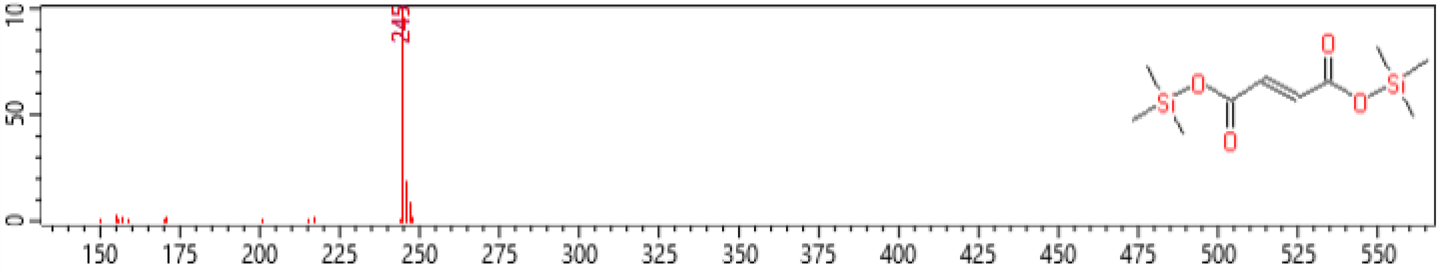

2-Butenedioic acid, (E)-, 2TMS derivative

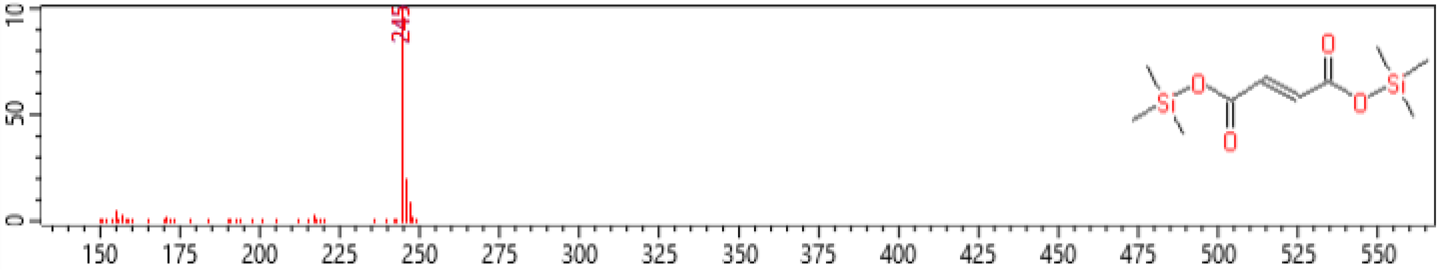

2,3-Dihydroxy-2-methylbutanoic acid, 3TMS

Peak: 9.68 - 9.71 min (scan #749 - #755) Subtracted noise: 9.65 - 9.67 min (scan #739 - #744), 9.72 - 9.74 min (scan #758 - #764)

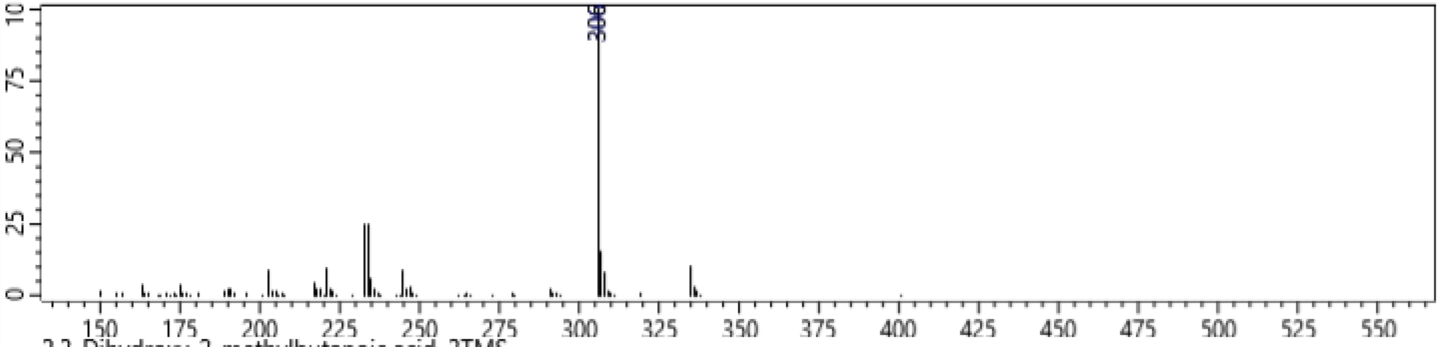

2,3-Dihydroxy-2-methylbutanoic acid, 3TMS

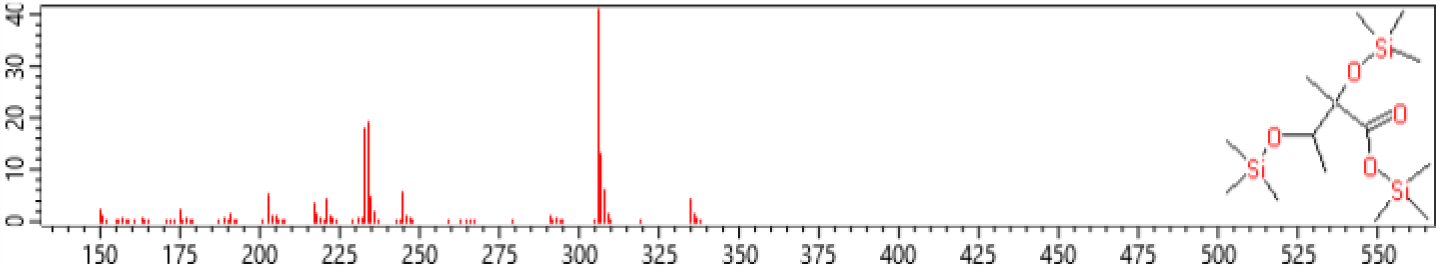

(3S)-3-Benzyloxycarbonylamino-1-chloro-4-(p-fluorophenyl)-2-butanone

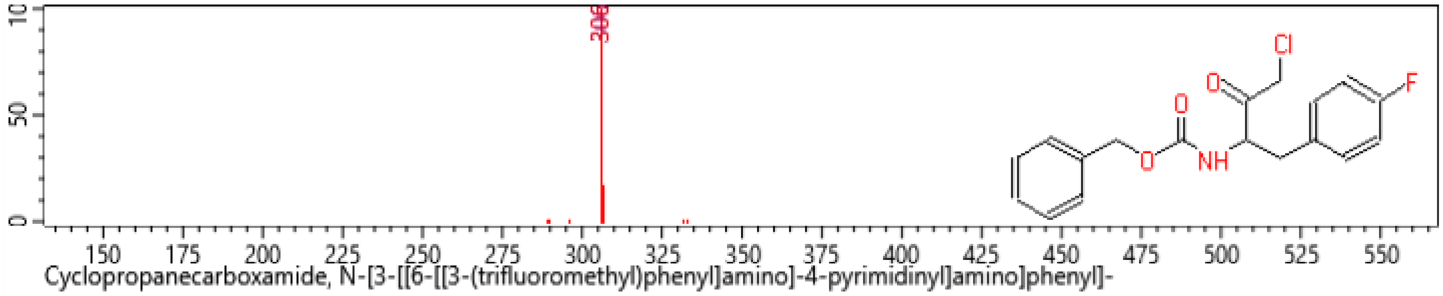

Cyclopropanecarboxamide, N-[3-[[6-[[3-(trifluoromethyl)phenyl]amino]-4-pyrimidinyl]amino]phenyl]-

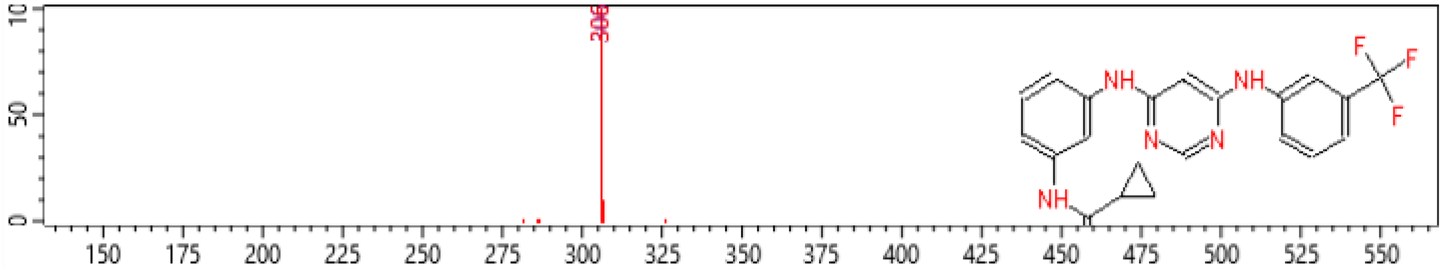

2-(3-Methoxyphenyl)-3-oxo-1-(prop-2-en-1-yl)-2,3-dihydro-1H-isoindole-4-carboxylic acid

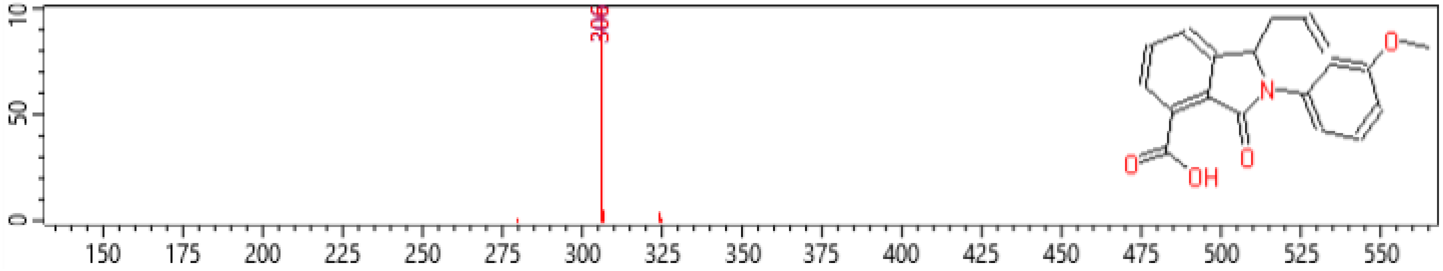

2-{[2-Chloro-5-(trifluoromethyl)anilino]carbonyl}benzoic acid

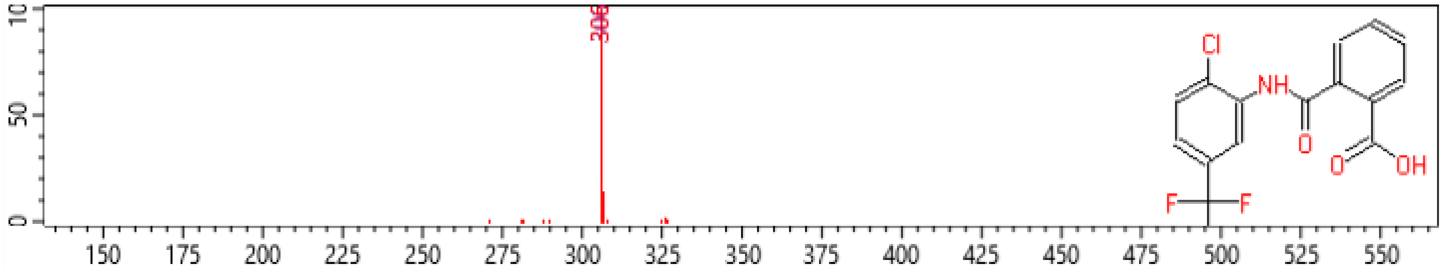

2,3-Dihydroxy-2-methylbutanoic acid, 3TMS

Peak: 9.73 - 9.75 min (scan #761 - #767) Subtracted noise: 9.69 - 9.71 min (scan #751 - #757), 9.77 - 9.79 min (scan #773 - #779)

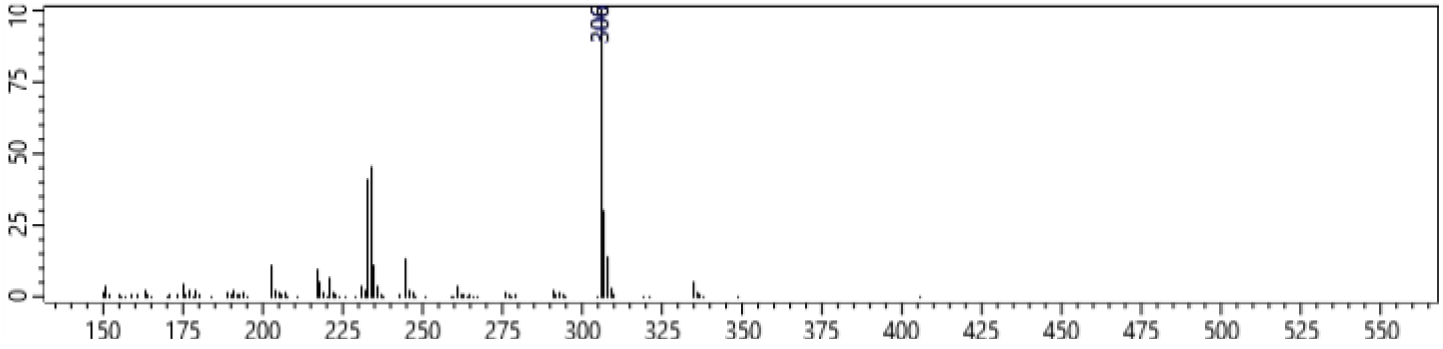

2,3-Dihydroxy-2-methylbutanoic acid, 3TMS

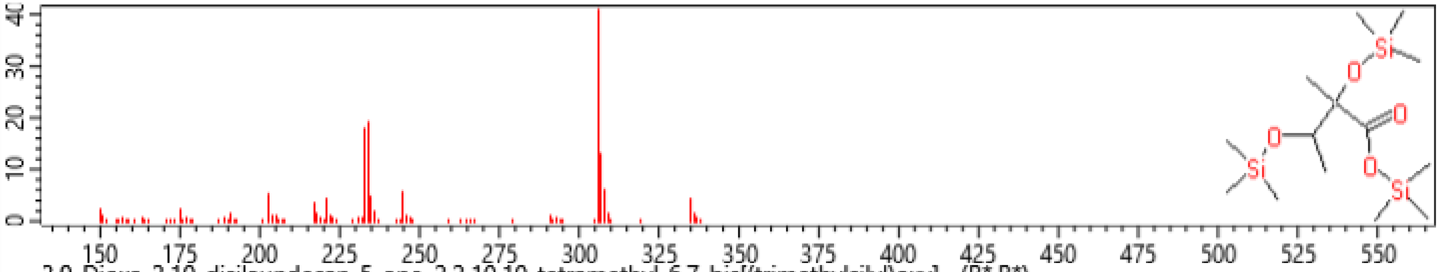

3,9-Dioxa-2,10-disilaundecan-5-one, 2,2,10,10-tetramethyl-6,7-bis[(trimethylsilyl)oxy]-, (R*,R*)-

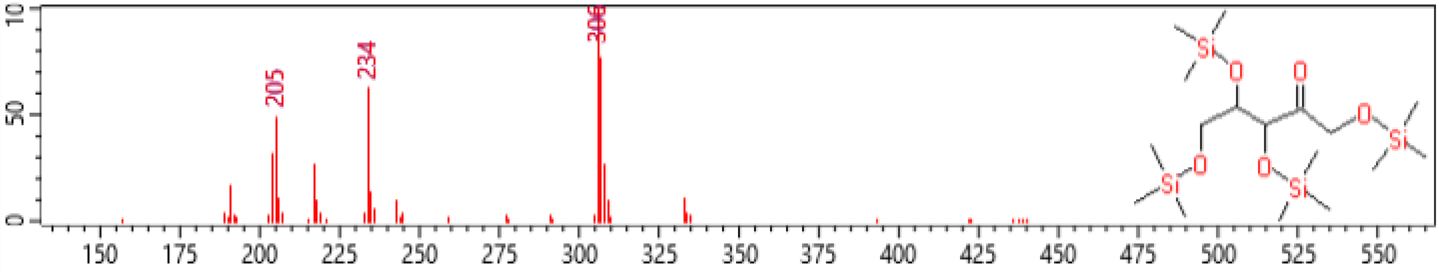

4-Chloro-5,7-diphenyl-7H-pyrrolo[2,3-d]pyrimidine

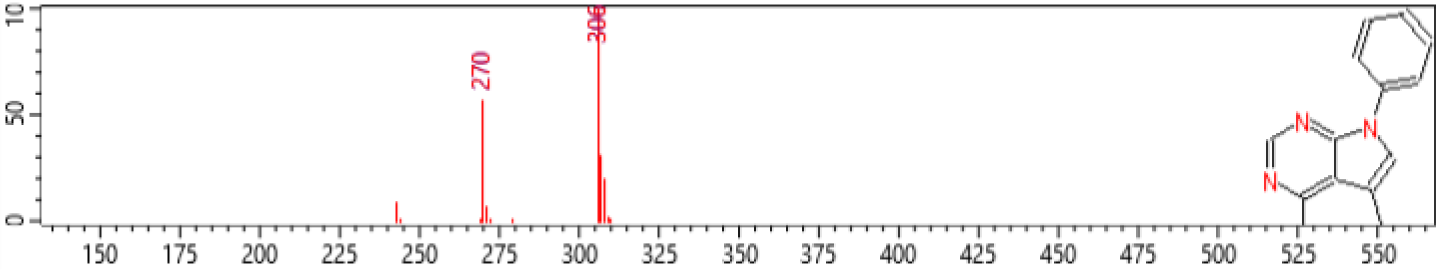

Dextromoramide

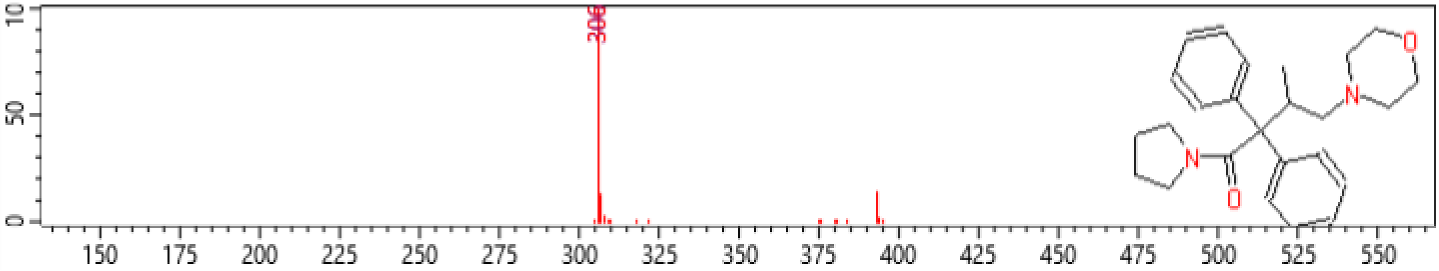

Tromethamine, 4TMS derivative

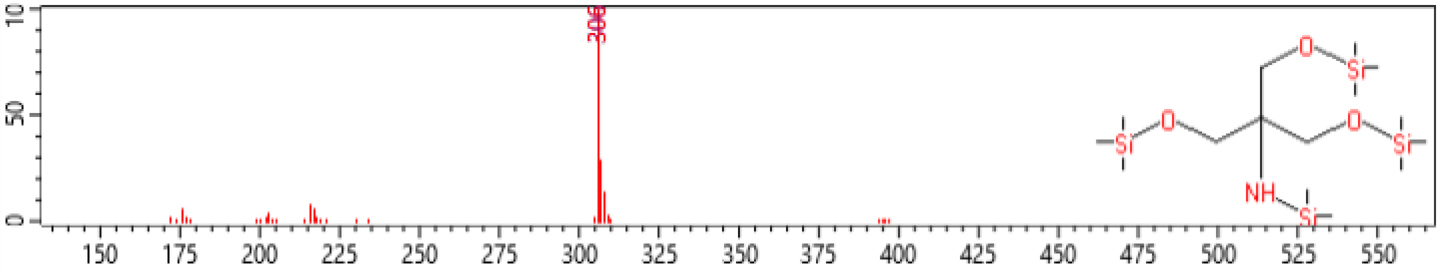

Vanillin, TMS derivative

Peak: 9.87 - 9.92 min (scan #802 - #814) Subtracted noise: 9.82 - 9.86 min (scan #787 - #798), 9.96 - 10.00 min (scan #826 - #837)

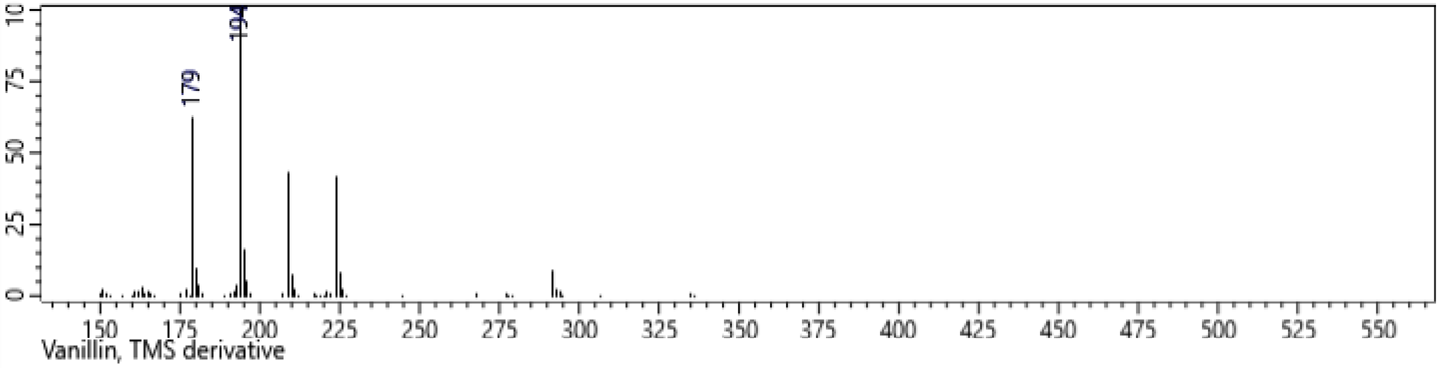

Vanillin, TMS derivative

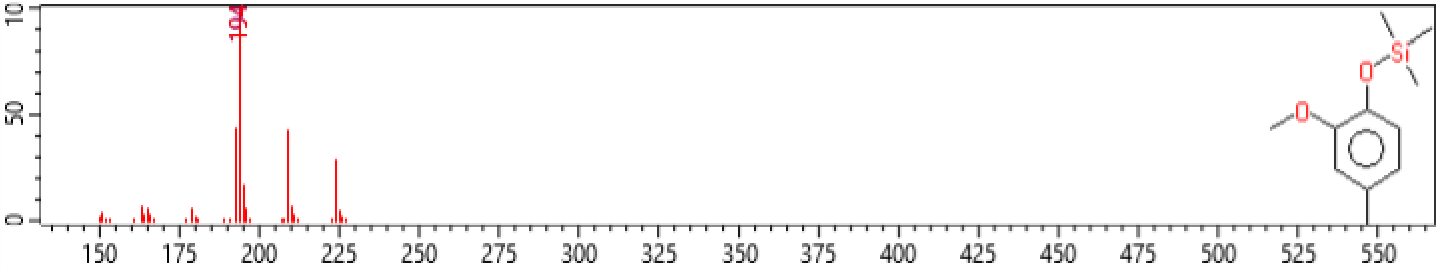

Vanillin, TMS derivative

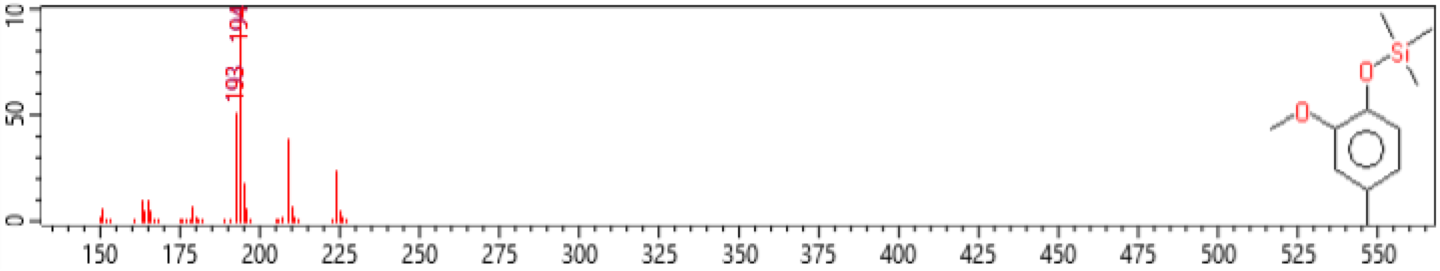

Vanillin, TMS derivative

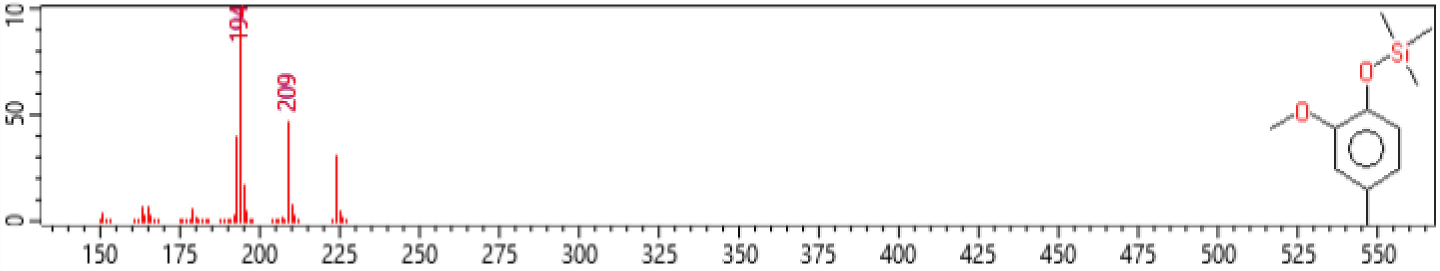

3-Hydroxy-4-methoxybenzaldehyde, TMS

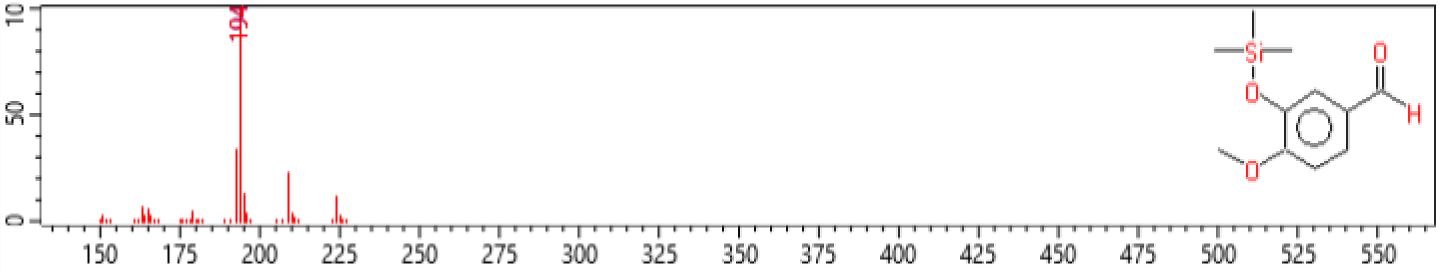

o-Anisic acid, TMS derivative

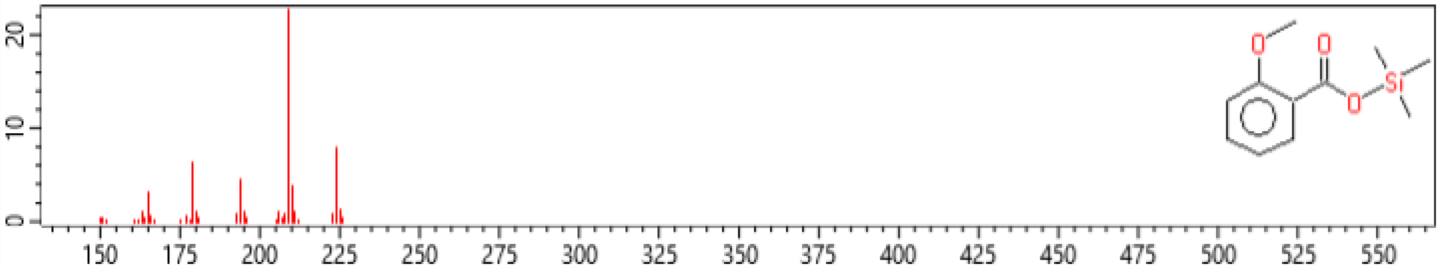

Resorcinol, 2TMS derivative

Peak: 9.97 - 10.00 min (scan #829 - #837) Subtracted noise: 9.92 - 9.96 min (scan #816 - #825), 10.02 - 10.05 min (scan #843 - #852)

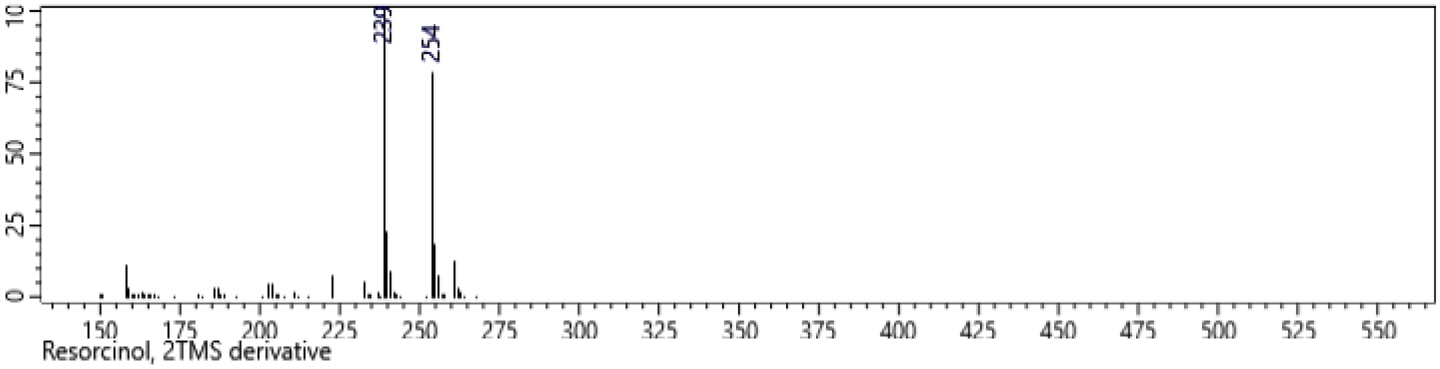

Resorcinol, 2TMS derivative

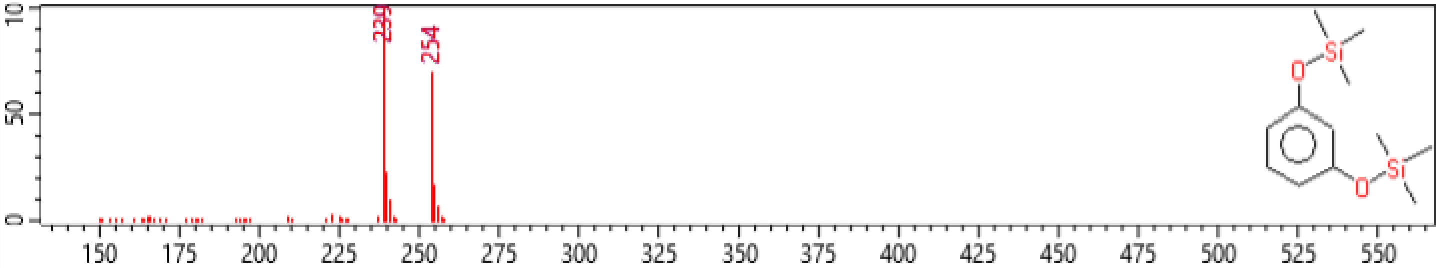

Resorcinol, 2TMS derivative

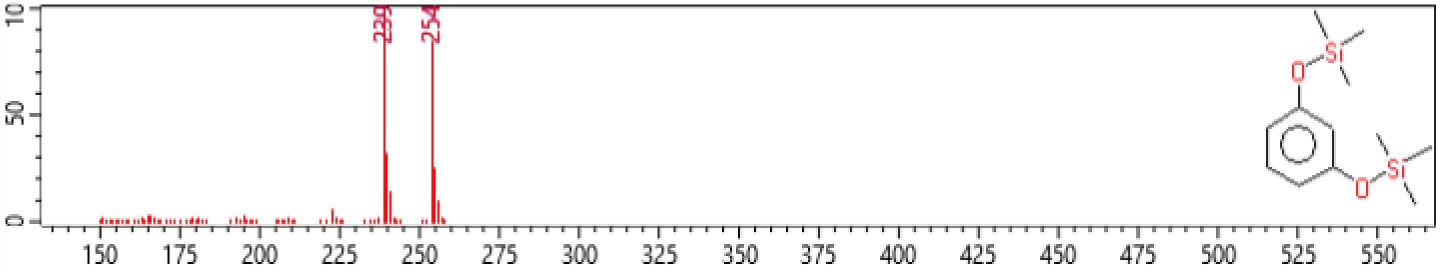

Hydroquinone, 2TMS derivative

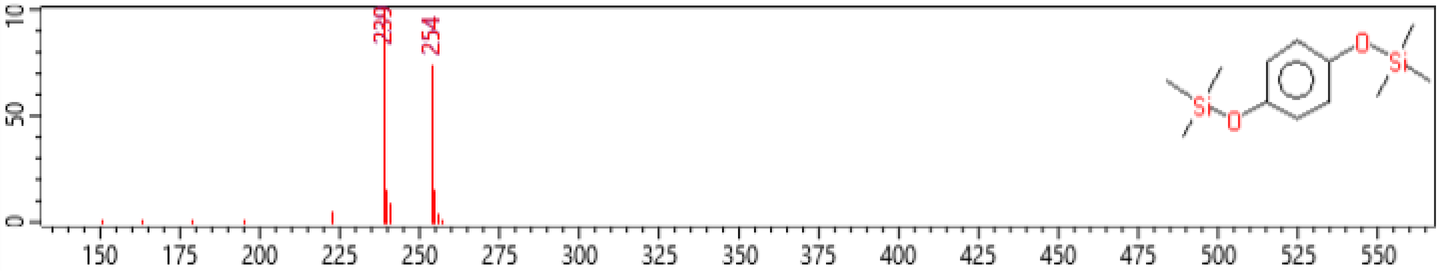

Hydroquinone, 2TMS derivative

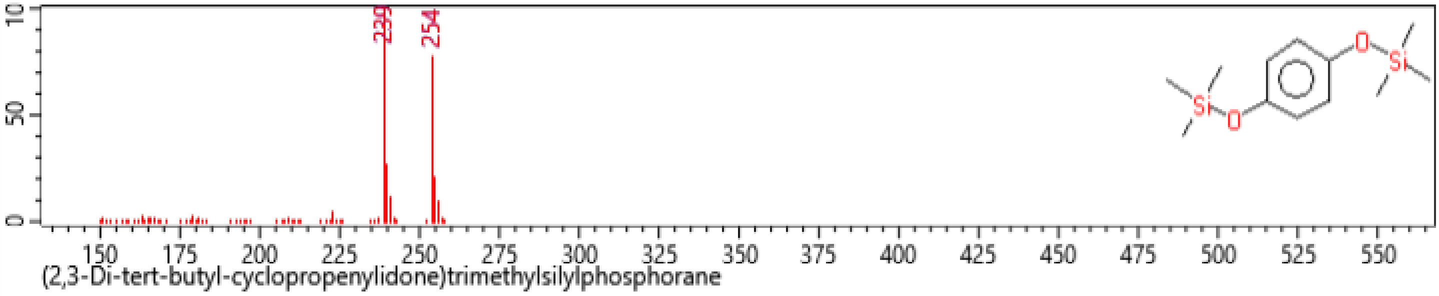

(2,3-Di-tert-butyl-cyclopropenylidone)trimethylsilylphosphorane

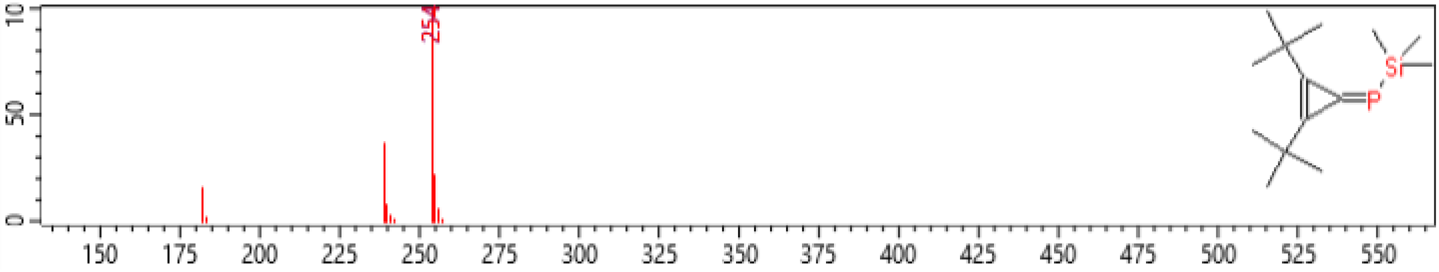

Propanedioic acid, (trimethylsilyl)-, bis(trimethylsilyl) ester

Peak: 10.19 - 10.21 min (scan #890 - #896) Subtracted noise: 10.15 - 10.17 min (scan #878 - #884), 10.22 - 10.24 min (scan #899 - #

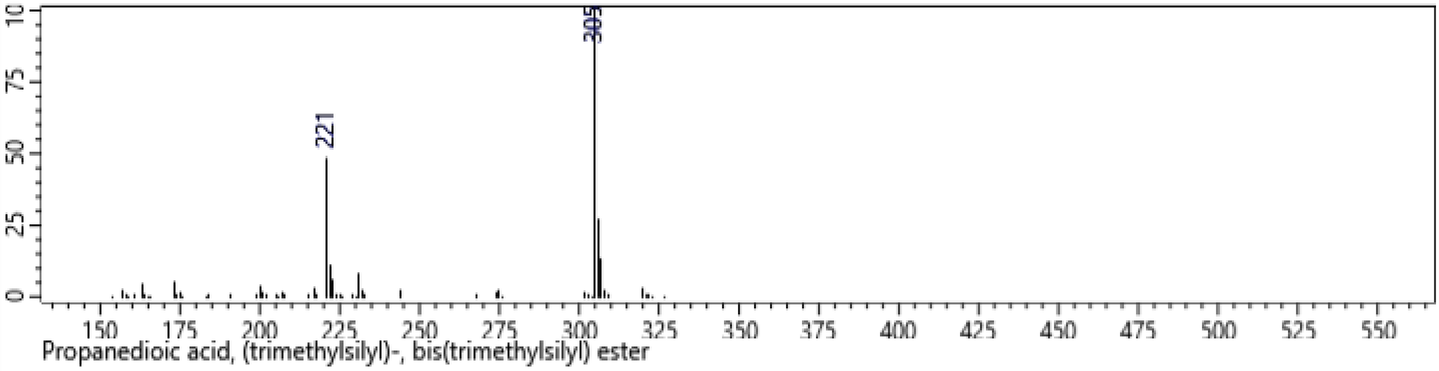

Propanedioic acid, (trimethylsilyl)-, bis(trimethylsilyl) ester

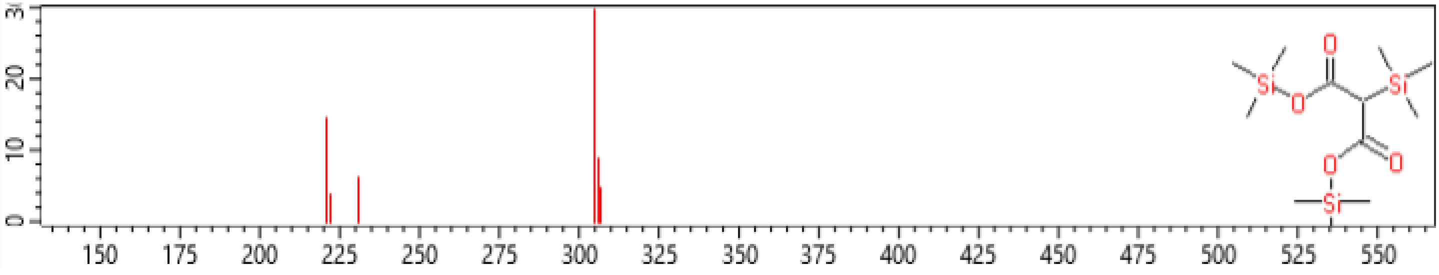

O,O,O'-Tris-trimethylsilylmalonate

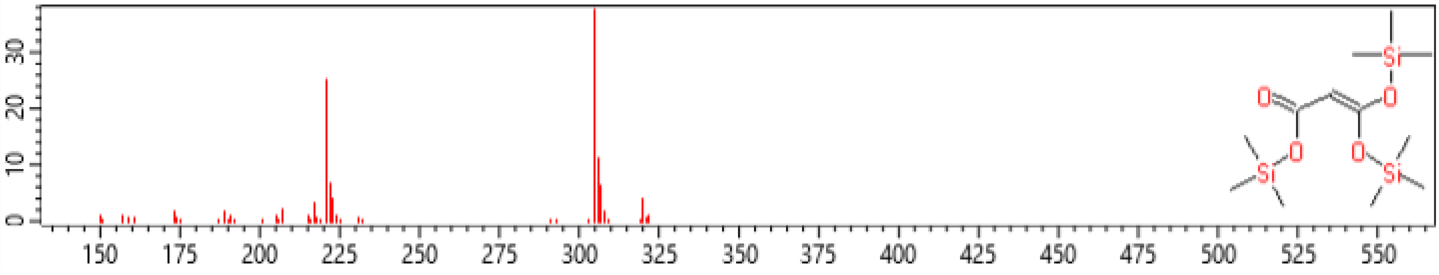

Acrylic acid, 2,3-bis[(trimethylsilyl)oxy]-, trimethylsilyl ester

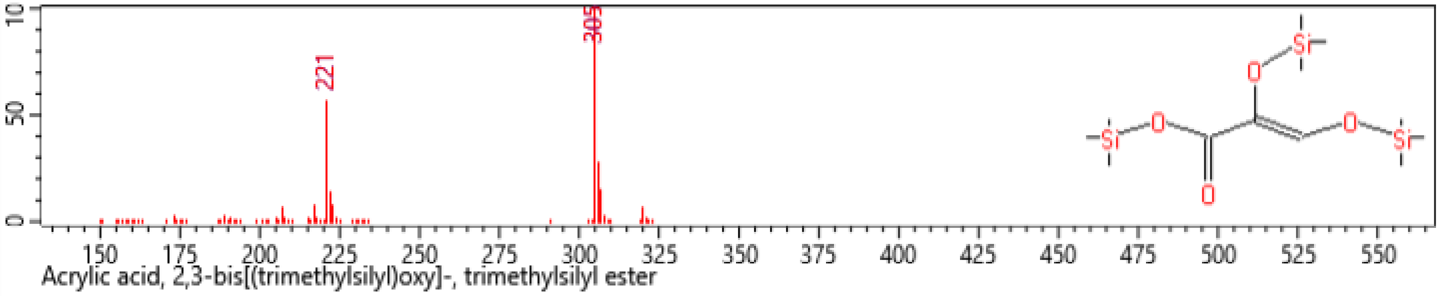

Acrylic acid, 2,3-bis[(trimethylsilyl)oxy]-, trimethylsilyl ester

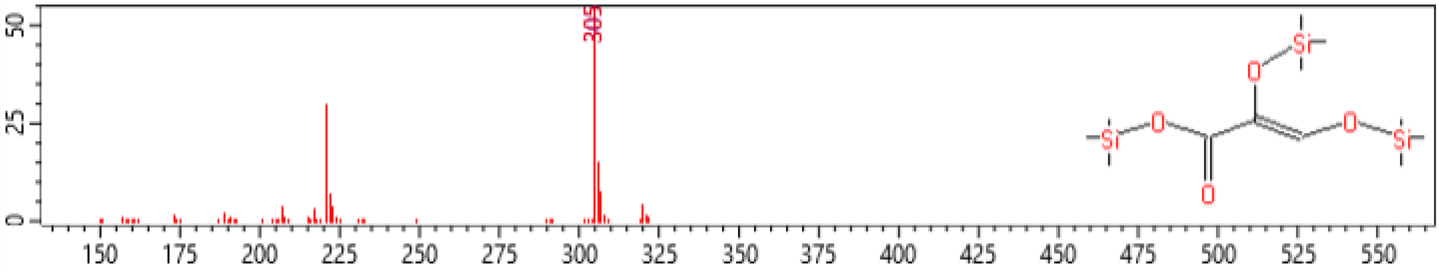

1,7'-Dimethyl-2'-propyl-1H,1'H-2,5'-bibenzo[d]imidazole

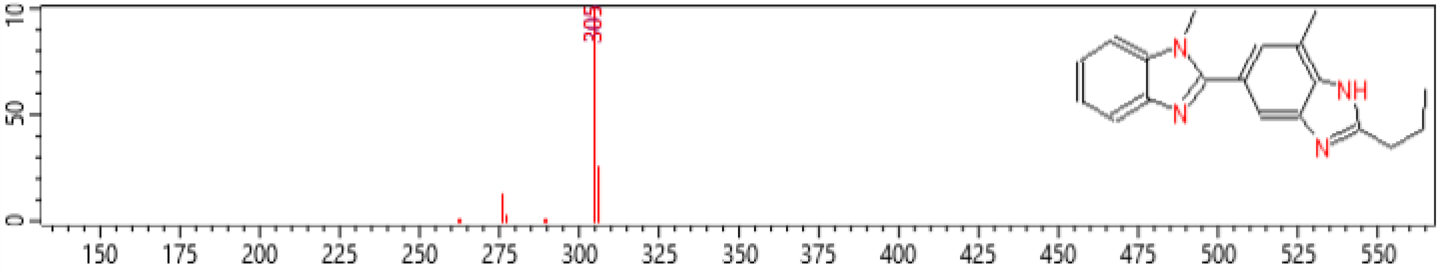

Bis(trimethylsilyl) 2-methylbicyclo[2.2.1]hept-5-ene-2,3-dicarboxylate (isomer 2)

Peak: 10.23 - 10.25 min (scan #902 - #907) Subtracted noise: 10.20 - 10.22 min (scan #893 - #898), 10.26 - 10.28 min (scan #911 - #

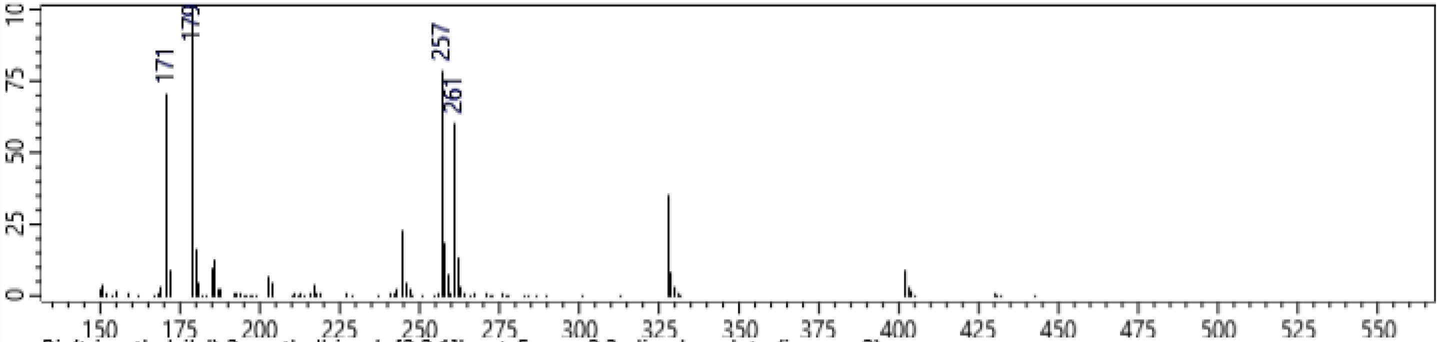

Bis(trimethylsilyl) 2-methylbicyclo[2.2.1]hept-5-ene-2,3-dicarboxylate (isomer 2)

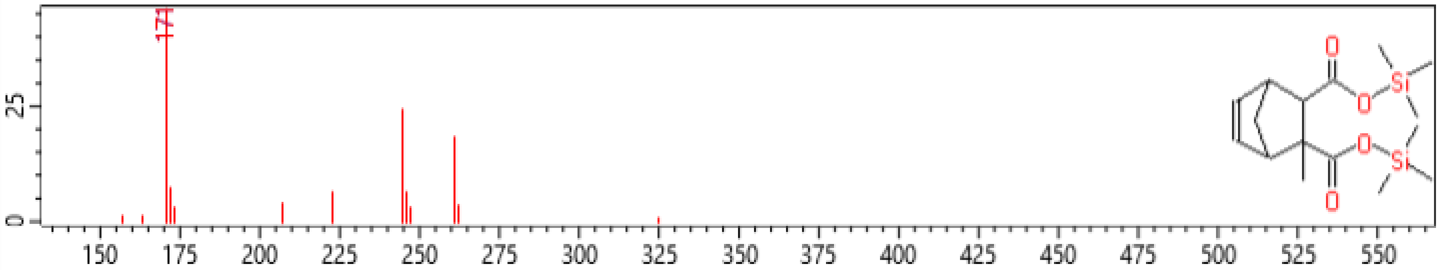

6-Hydroxyhexanoic acid, 2TMS derivative

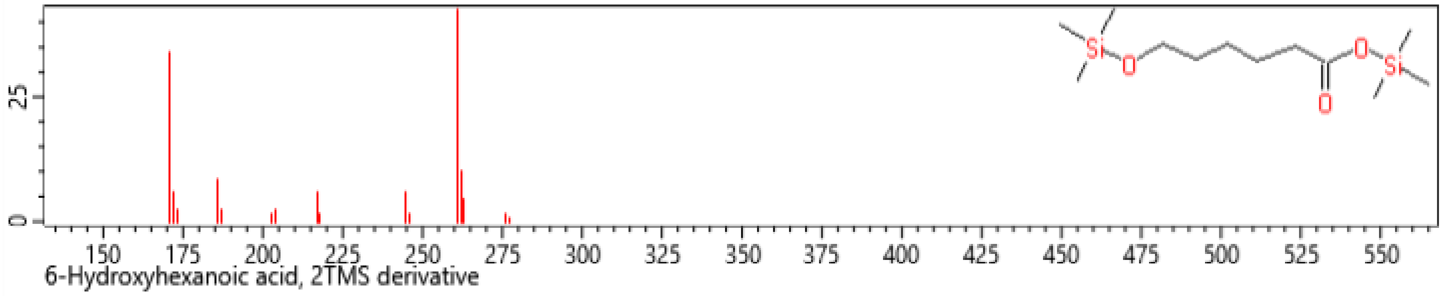

6-Hydroxyhexanoic acid, 2TMS derivative

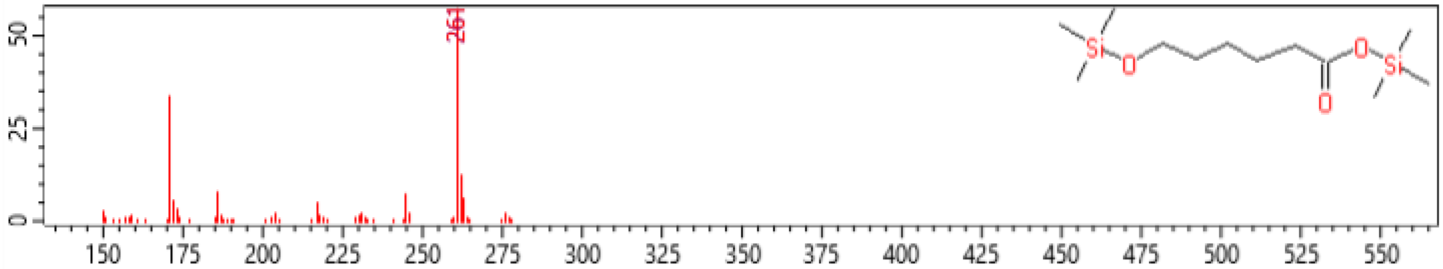

5-Bromo-5'-hexyl-2,2'-bithiophene

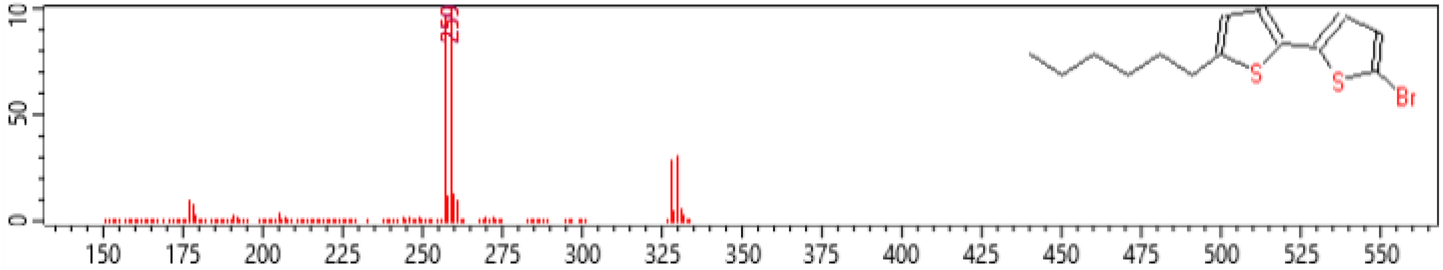

2-Cyclohexyl-N-(4-methoxyphenyl)acetamide, N-methyl

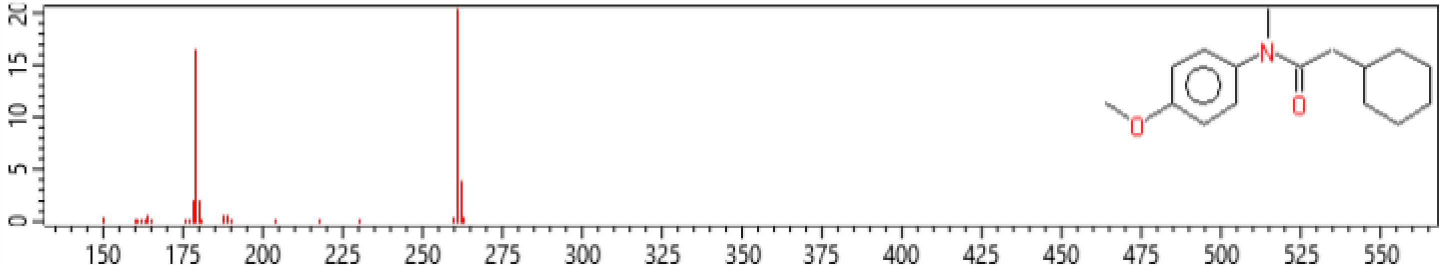

α-Ketoglutaric acid, TMS

Peak: 10.53 - 10.55 min (scan #986 - #990) Subtracted noise: 10.50 - 10.52 min (scan #977 - #981), 10.55 - 10.57 min (scan #991 - #

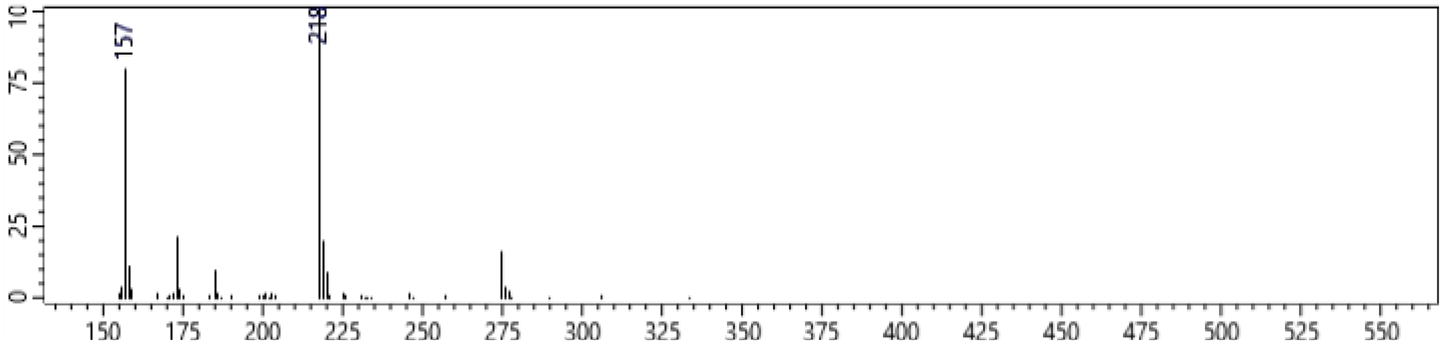

α-Ketoglutaric acid, TMS

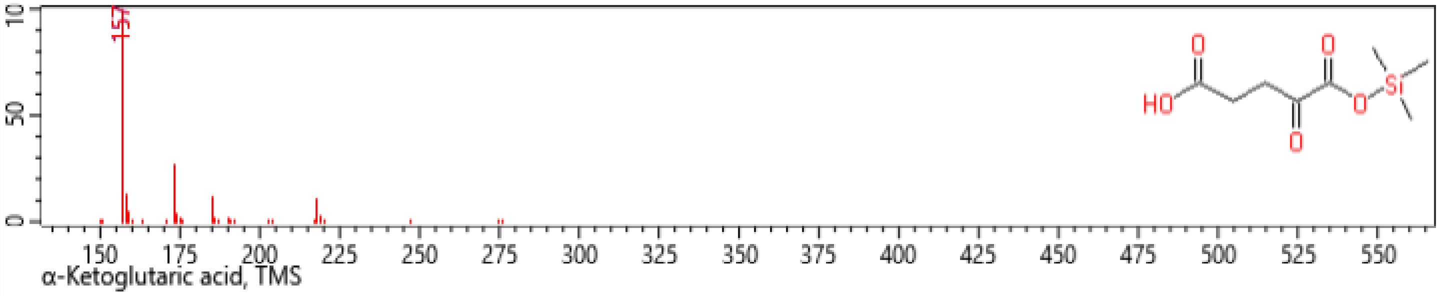

α-Ketoglutaric acid, TMS

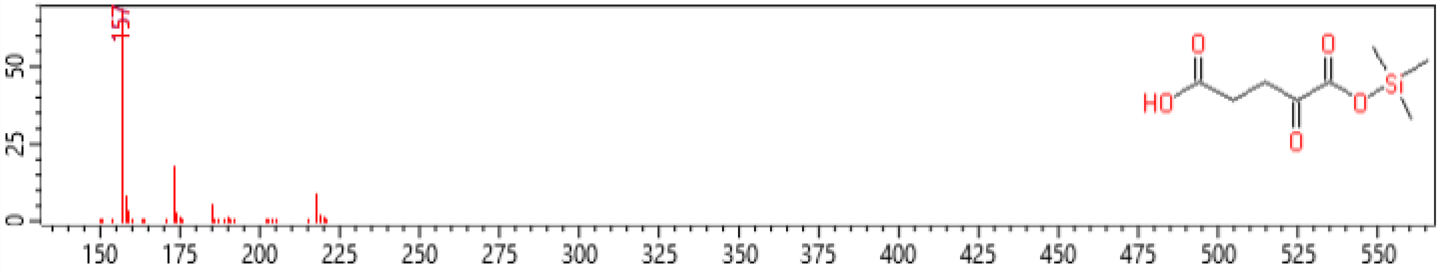

Phenol, 4,4'-thiobis-

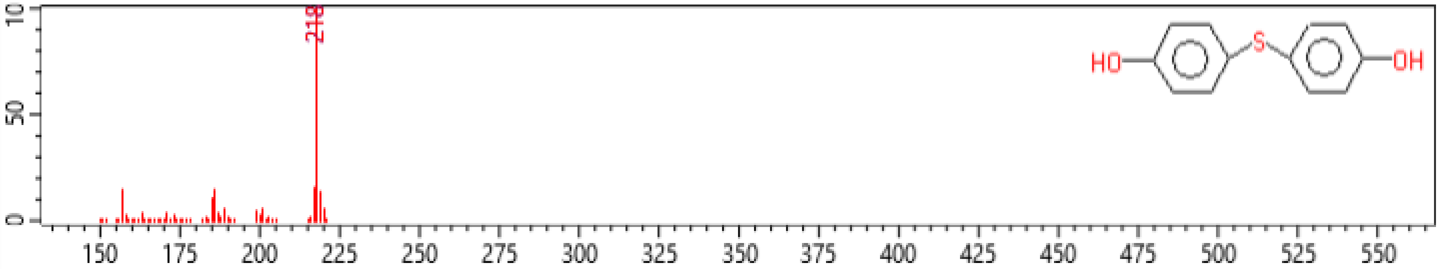

Phenol, 4,4'-thiobis-

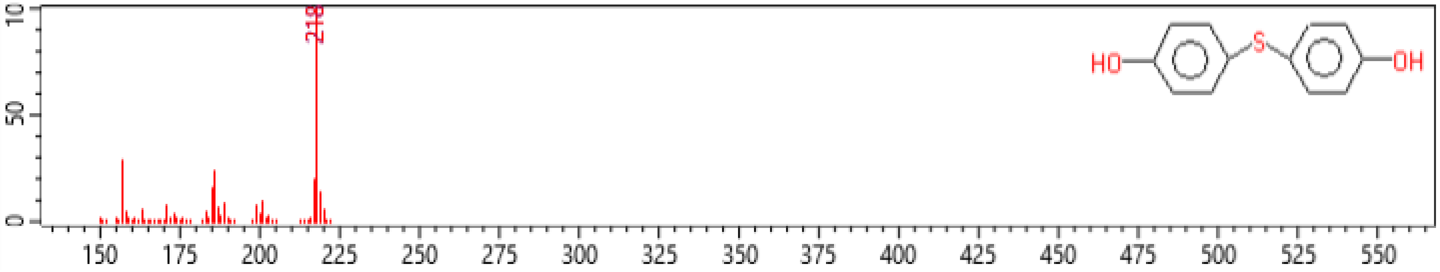

Bis(2-hydroxyphenyl)sulfide

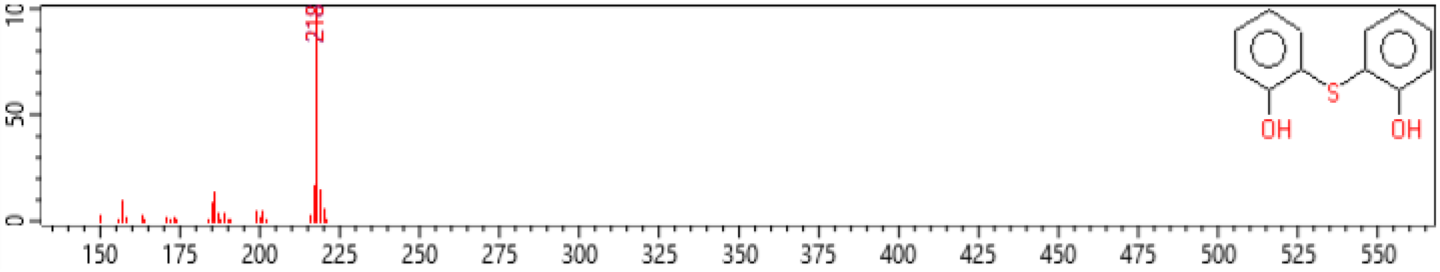

3,4-Dihydroxybenzaldehyde, bis(trimethylsilyl) ether

Peak: 10.59 - 10.63 min (scan #1001 - #1012) Subtracted noise: 10.51 - 10.55 min (scan #979 - #990), 10.64 - 10.68 min (scan #1015

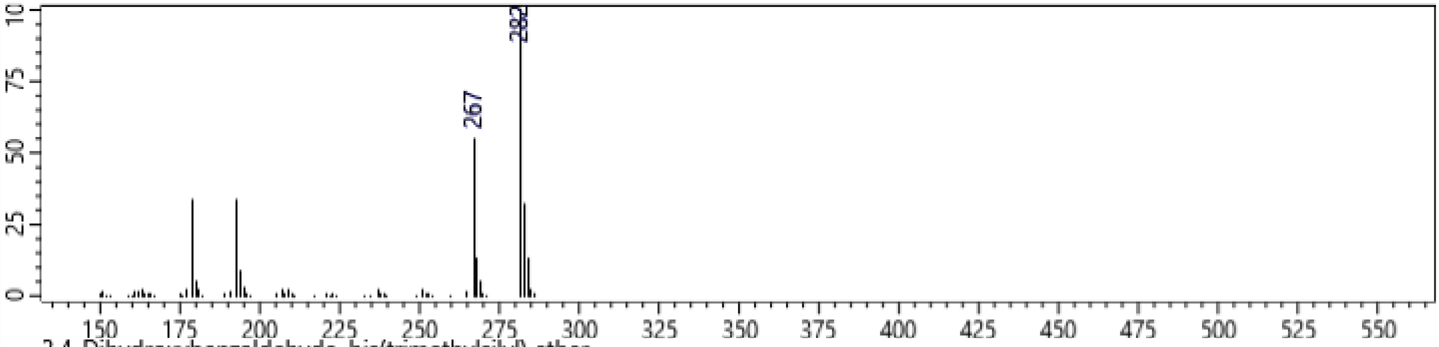

3,4-Dihydroxybenzaldehyde, bis(trimethylsilyl) ether

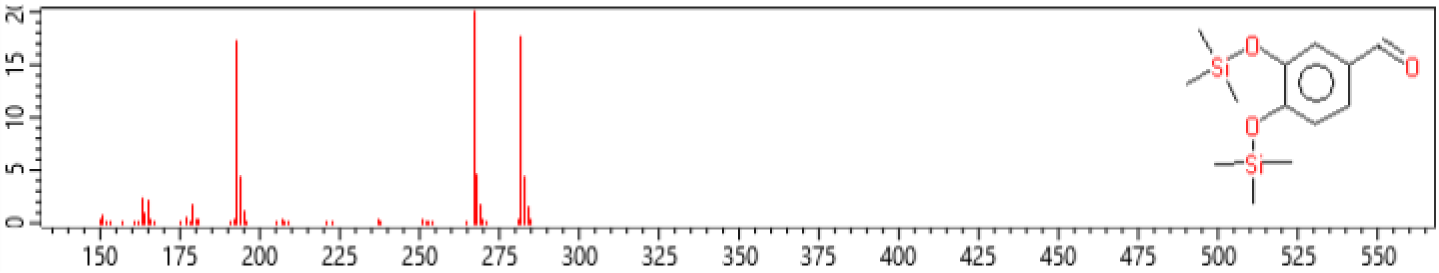

2-Ethylhydroquinone, bis(trimethylsilyl) ether

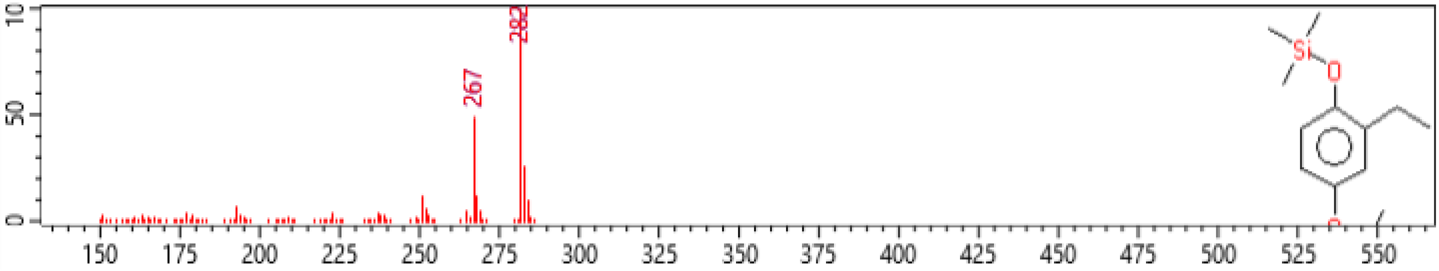

4,5-Dimethyl-1,3-benzenediol, 2TMS

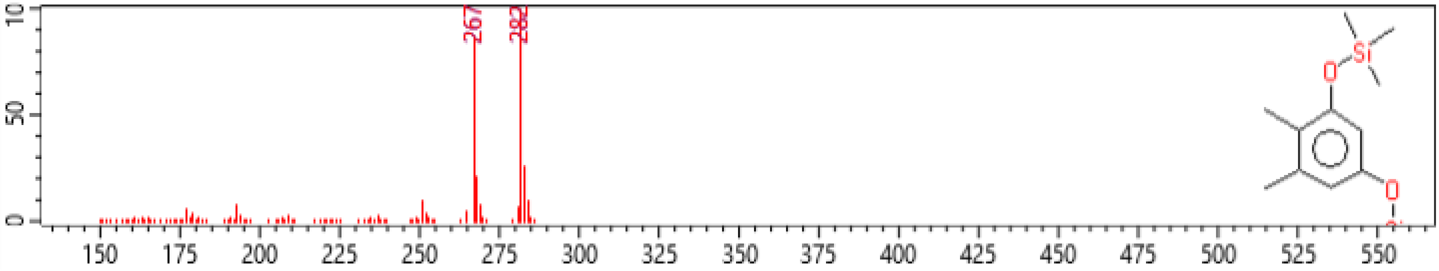

2,3-Dimethylhydroquinone, bis(trimethylsilyl) ether

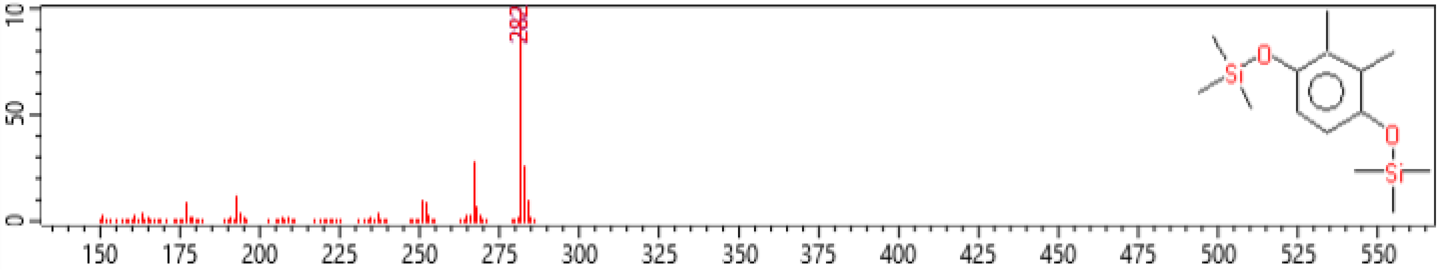

2-Hydroxyphenethyl alcohol, 2TMS derivative

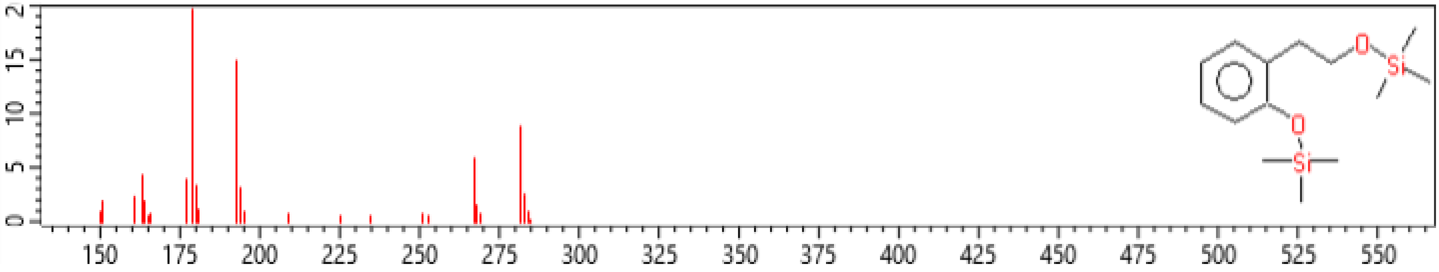

D-(-)-Citramalic acid, 3TMS derivative
Peak: 10.69 - 10.74 min (scan #1031 - #1043) Subtracted noise: 10.62 - 10.66 min (scan #1011 - #1022), 10.76 - 10.81 min (scan #10

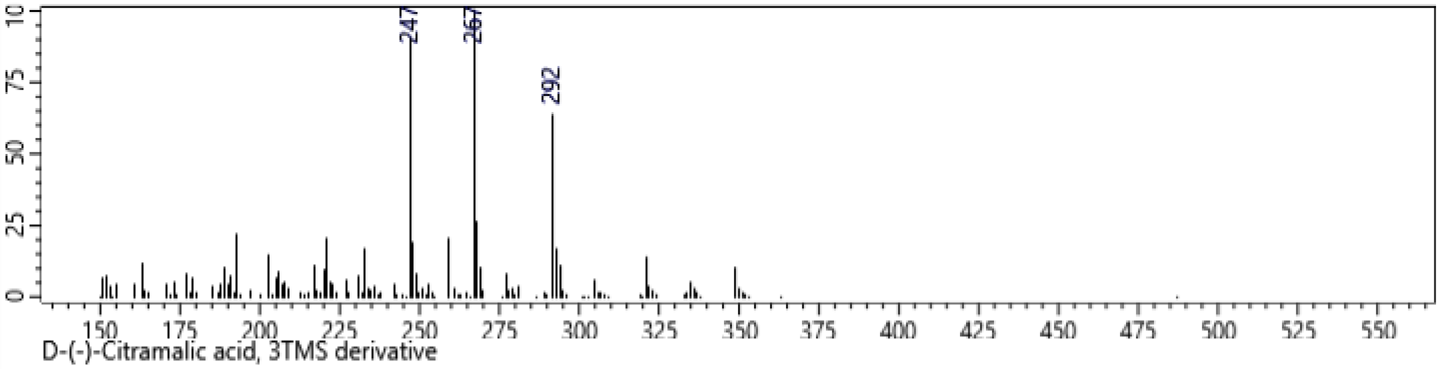

D-(-)-Citramalic acid, 3TMS derivative

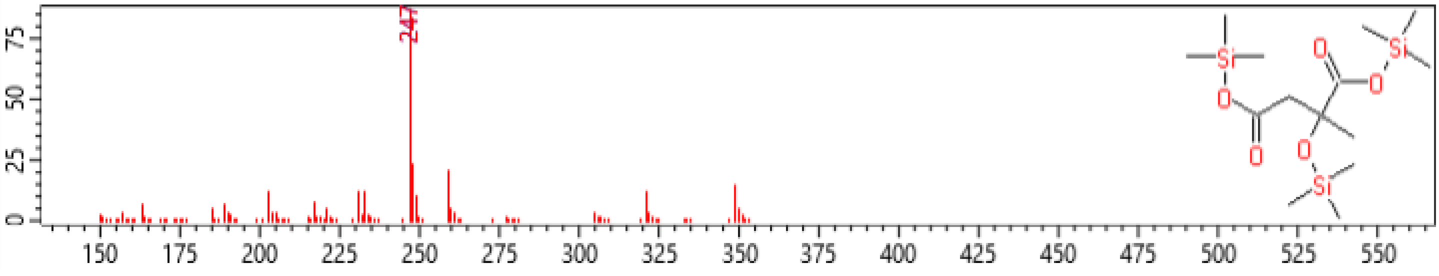

Tartronic acid, 3TMS derivative

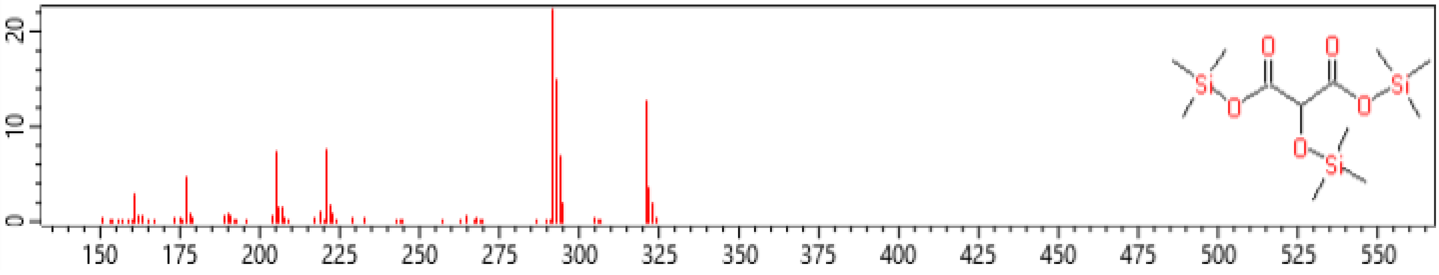

Cyclopentyl(hydroxy)phenylacetic acid, O-trimethylsilyl-, trimethylsilyl ester

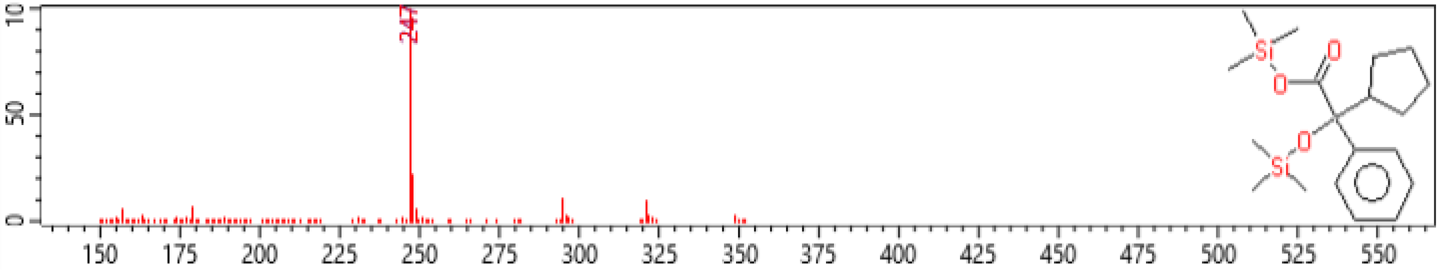

1H-Indole-2,3-dione, 5-methoxy-1-(trimethylsilyl)-, 3-(O-ethyloxime)

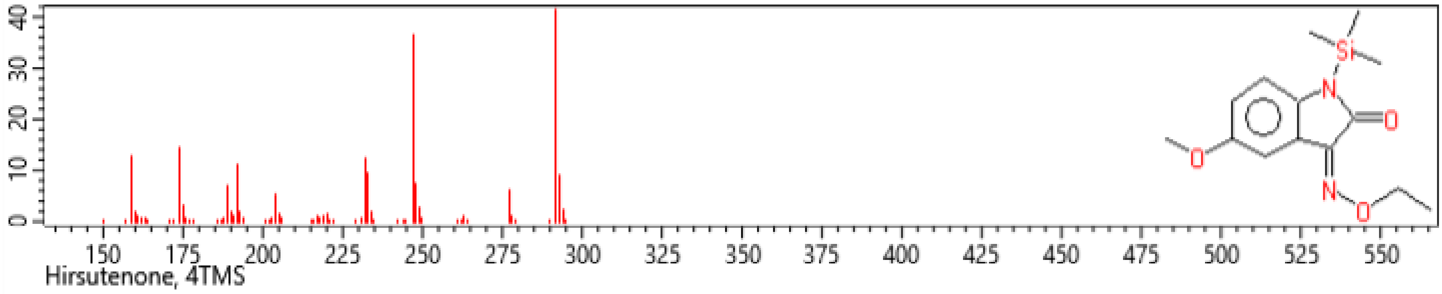

Hirsutenone, 4TMS

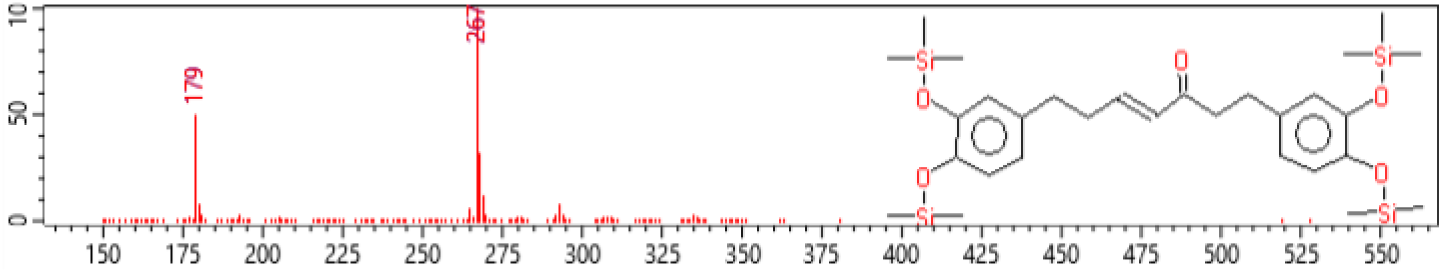

Malic acid, 3TMS derivative

Peak: 10.82 - 10.89 min (scan #1066 - #1086) Subtracted noise: 10.69 - 10.76 min (scan #1029 - #1048), 10.91 - 10.98 min (scan #10

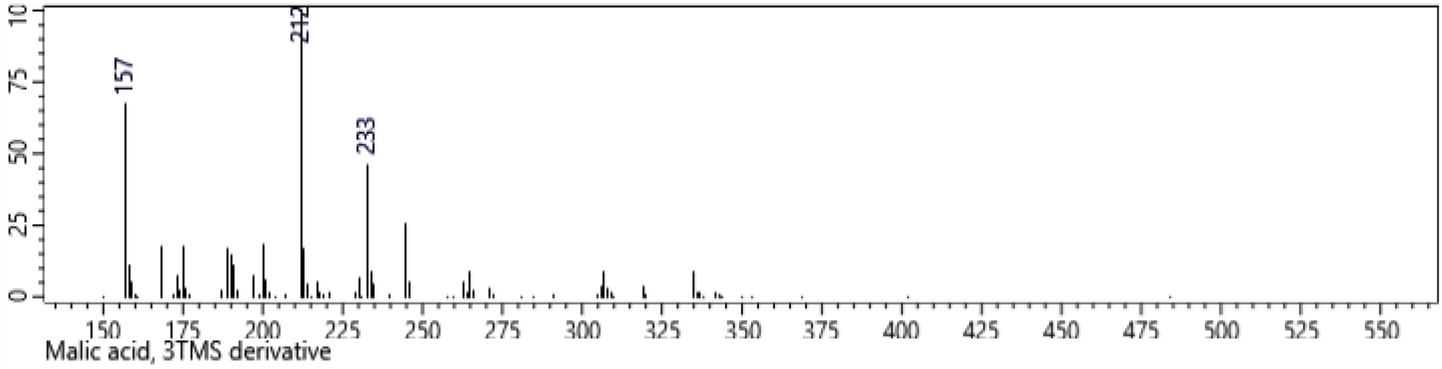

Malic acid, 3TMS derivative

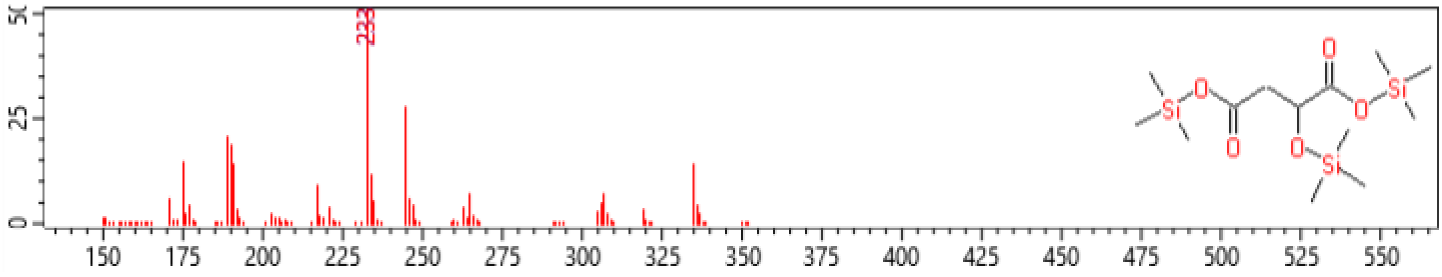

Malic acid, 3TMS derivative

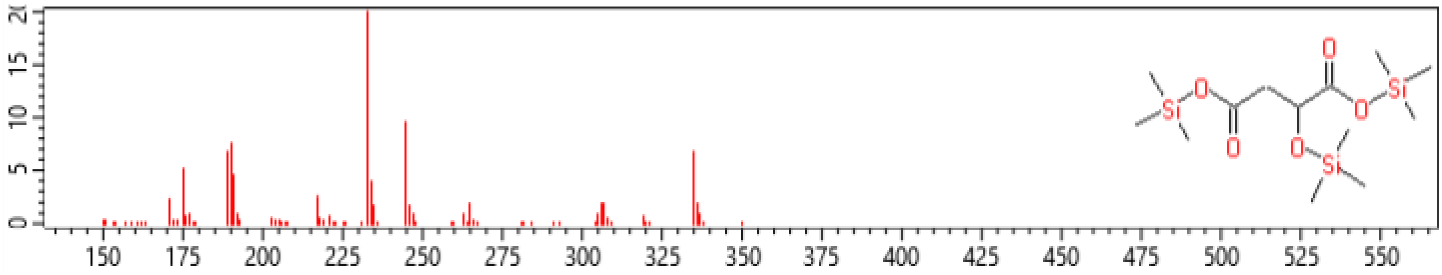

Malic acid, 3TMS derivative

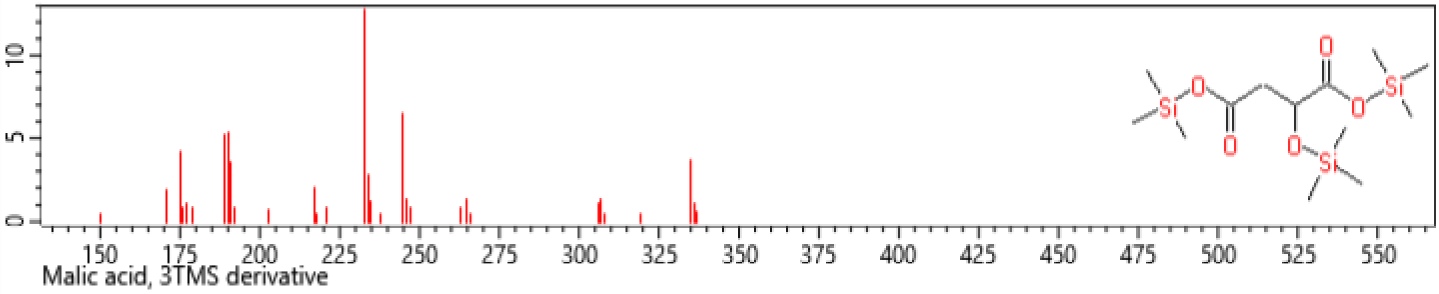

Malic acid, 3TMS derivative

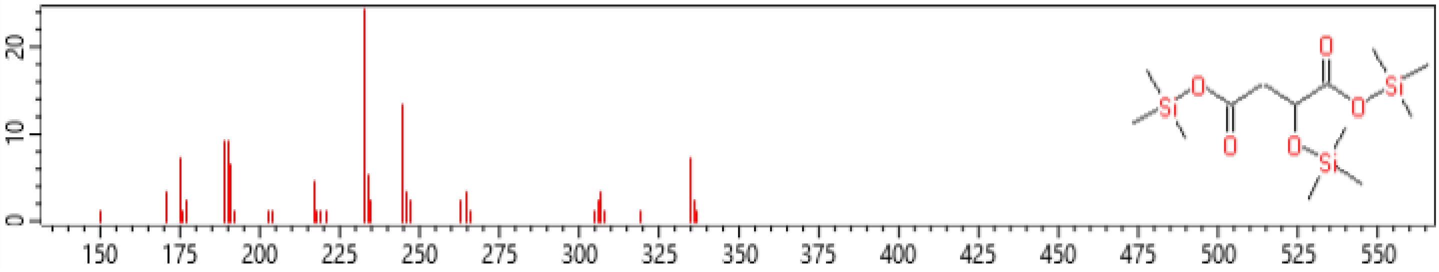

Malic acid, 3TMS derivative

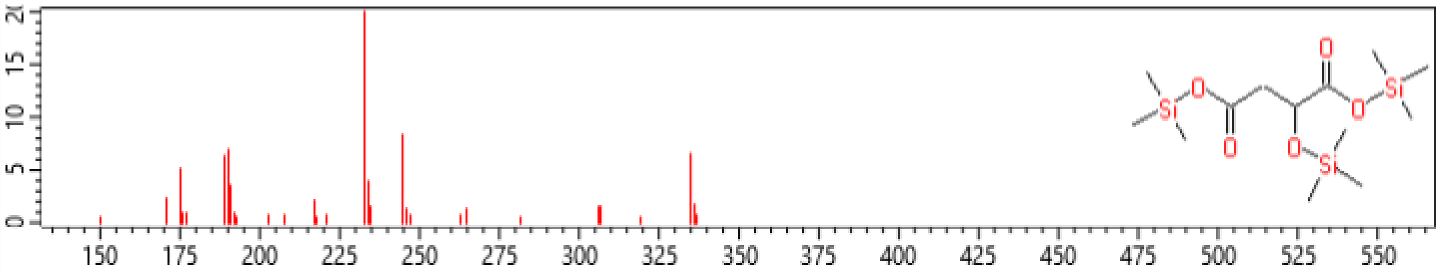

2-(Cyclopropylamino)-1,3-thiazole-4-carboxylic acid

Peak: 10.93 - 11.00 min (scan #1098 - #1116) Subtracted noise: 10.84 - 10.91 min (scan #1071 - #1090), 11.05 - 11.11 min (scan #11

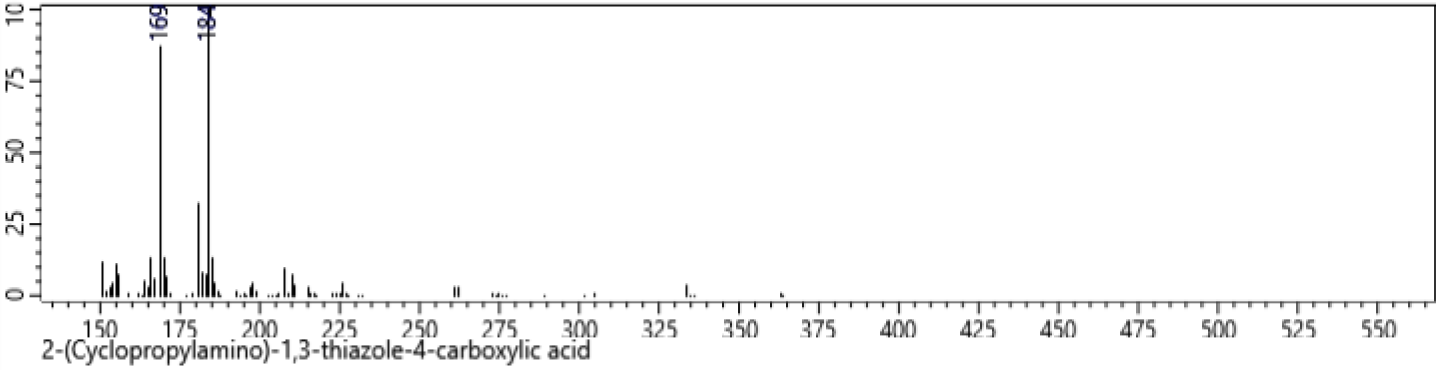

2-(Cyclopropylamino)-1,3-thiazole-4-carboxylic acid

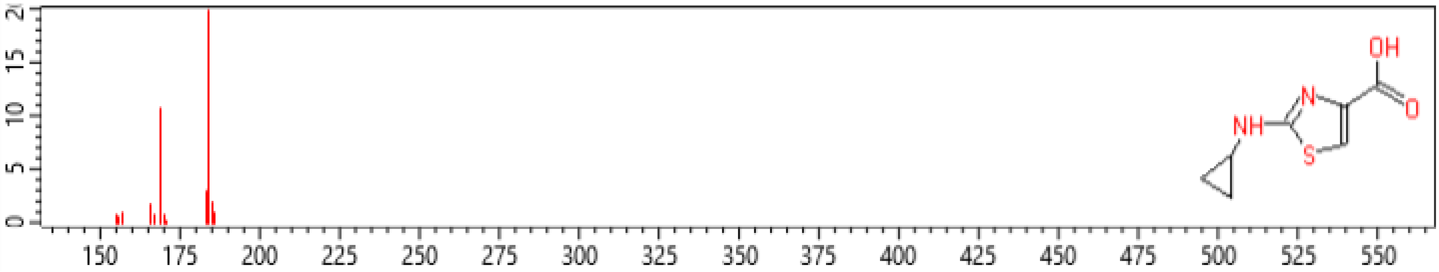

3,4,4-Trimethyl-5-methylene-2-ethylimino-1,3-thiazolidine

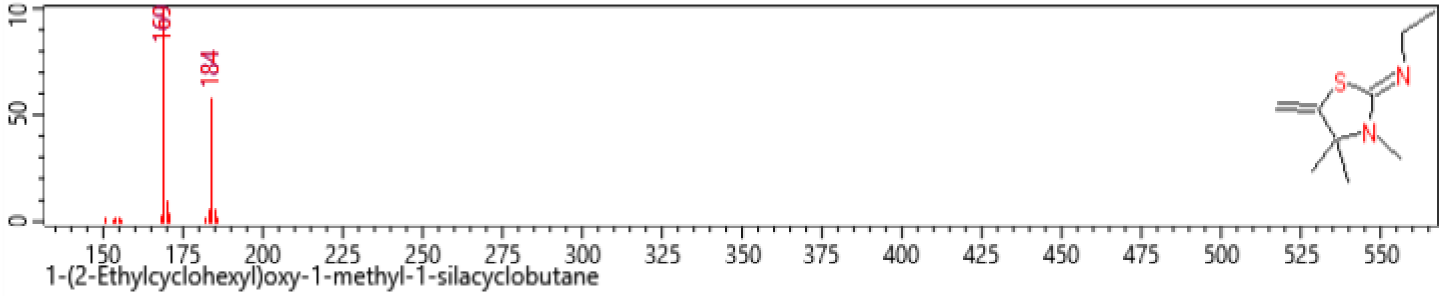

1-(2-Ethylcyclohexyl)oxy-1-methyl-1-silacyclobutane

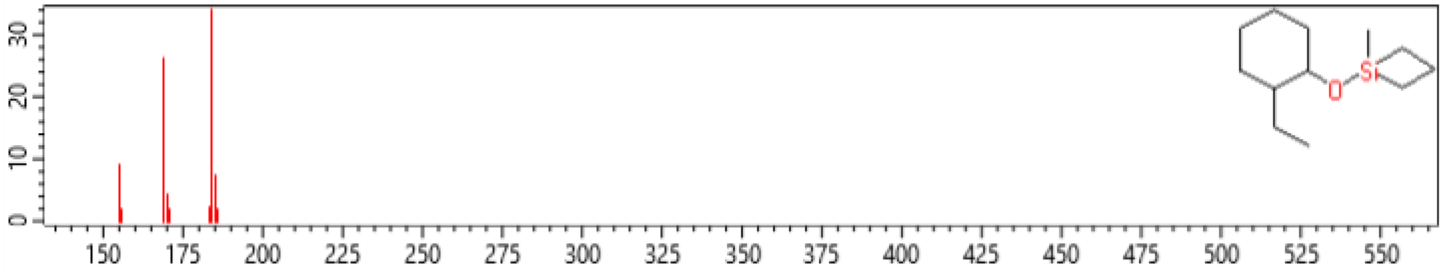

1,6-Dimethyl-4-ethylnaphthalene (Norcadalene)

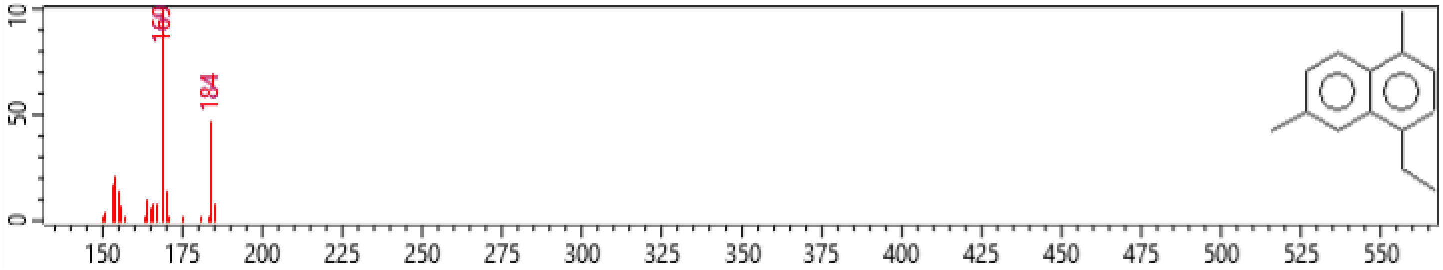

Silane, tetra-1-propynyl-

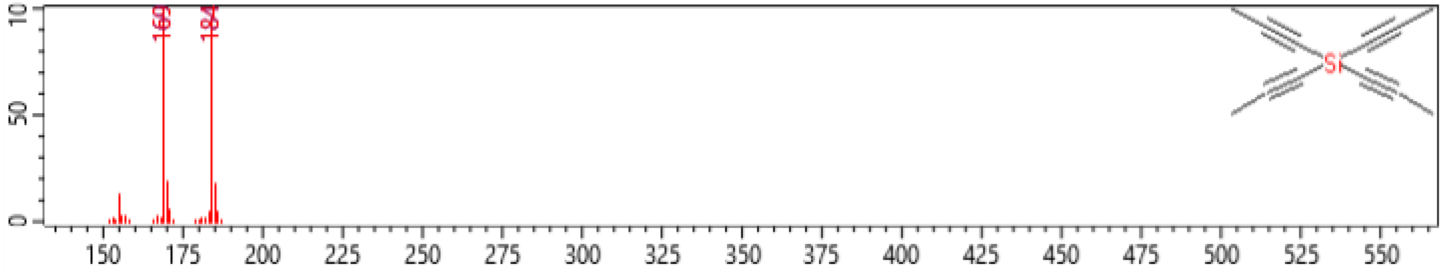

Salicylic acid, 2TMS derivative

Peak: 11.06 - 11.10 min (scan #1134 - #1144) Subtracted noise: 11.01 - 11.04 min (scan #1119 - #1128), 11.12 - 11.16 min (scan #11

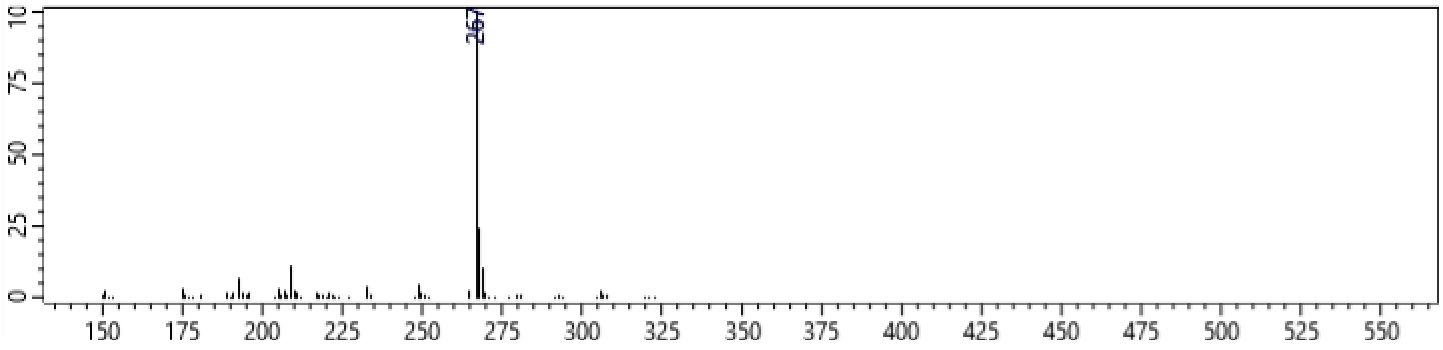

Salicylic acid, 2TMS derivative

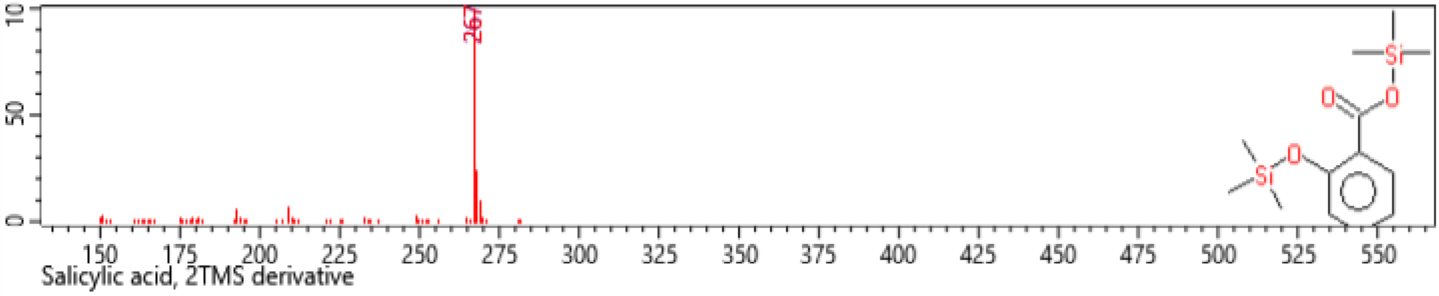

Salicylic acid, 2TMS derivative

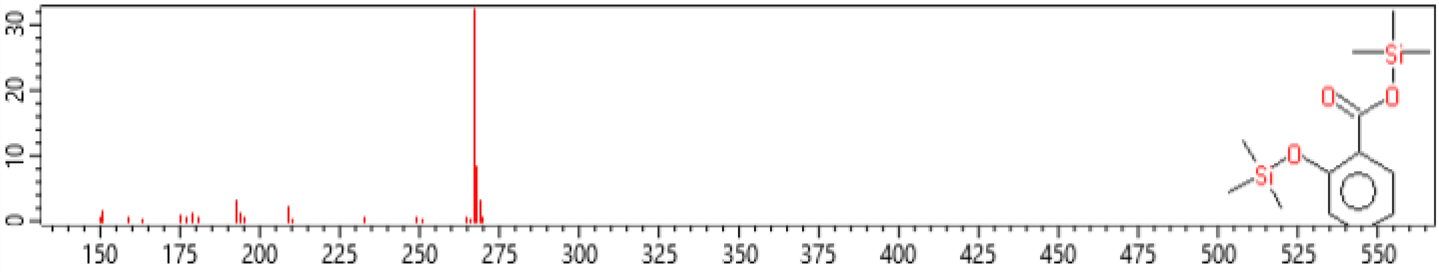

Salicylic acid, 2TMS derivative

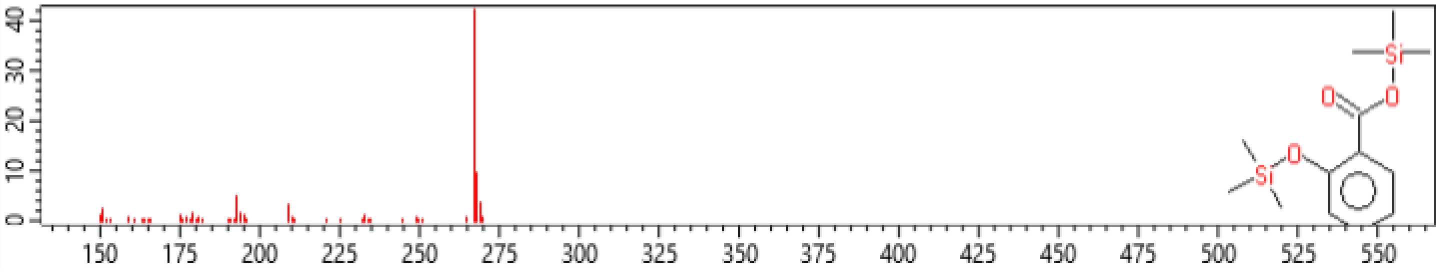

Salicylic acid, 2TMS derivative

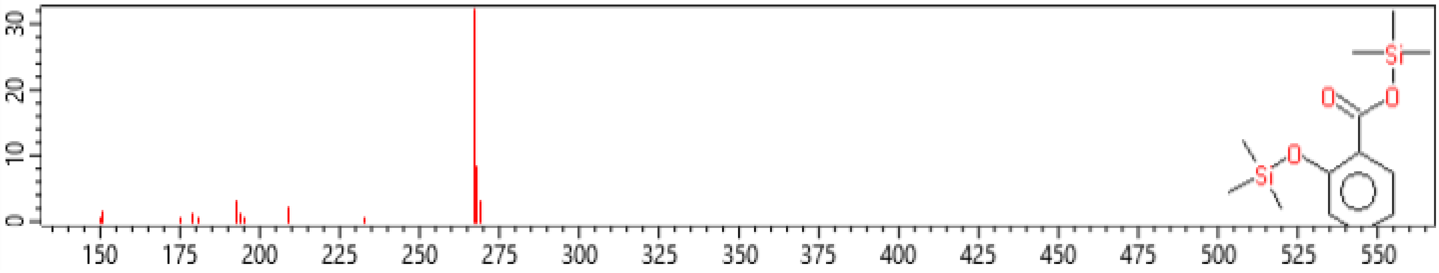

Salicylic acid, 2TMS derivative

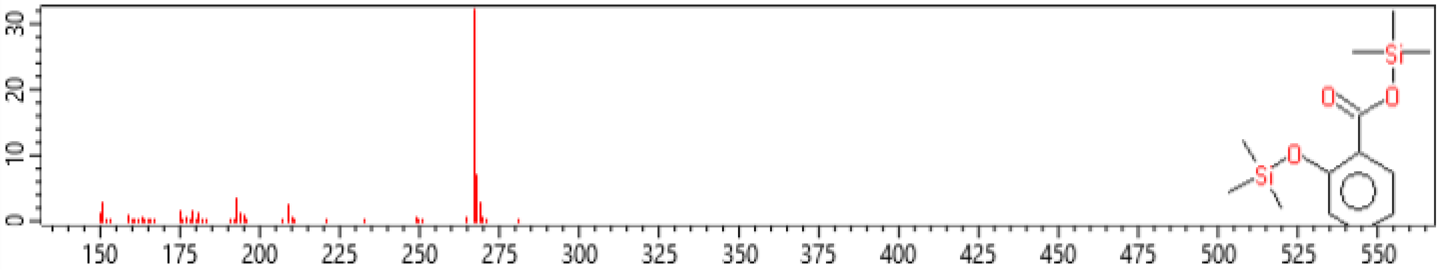

6-(2-Methoxy-ethyl)-2,3-dimethyl-1,4-dioxa-spiro[4.5]decane

Peak: 11.17 - 11.21 min (scan #1165 - #1174) Subtracted noise: 11.11 - 11.14 min (scan #1147 - #1156), 11.22 - 11.25 min (scan #11

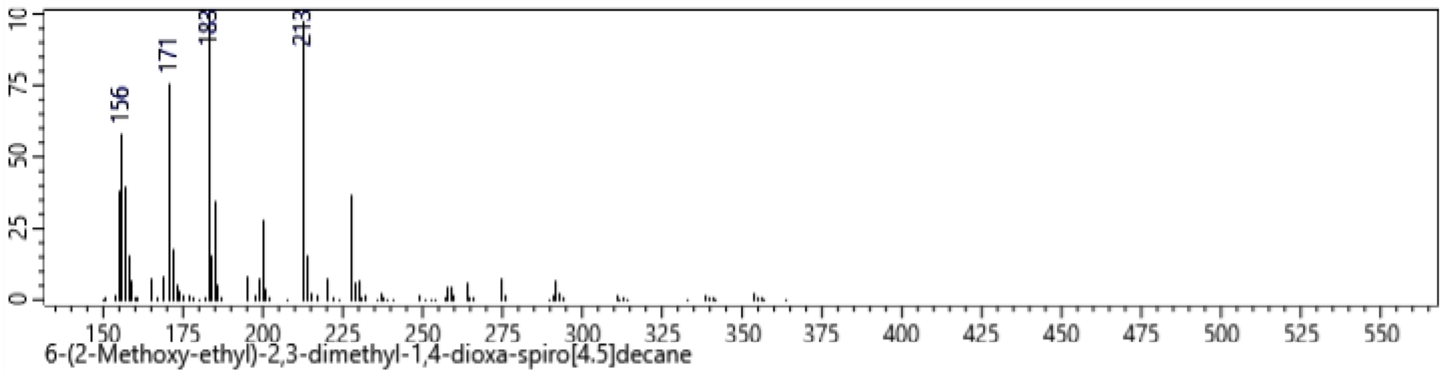

6-(2-Methoxy-ethyl)-2,3-dimethyl-1,4-dioxa-spiro[4.5]decane

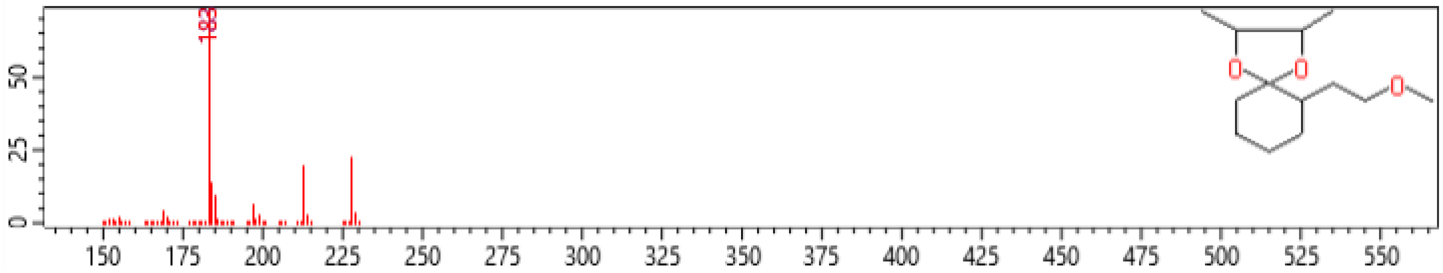

Silane, methylvinyl(trans-2-methylcyclohexyloxy)ethoxy-

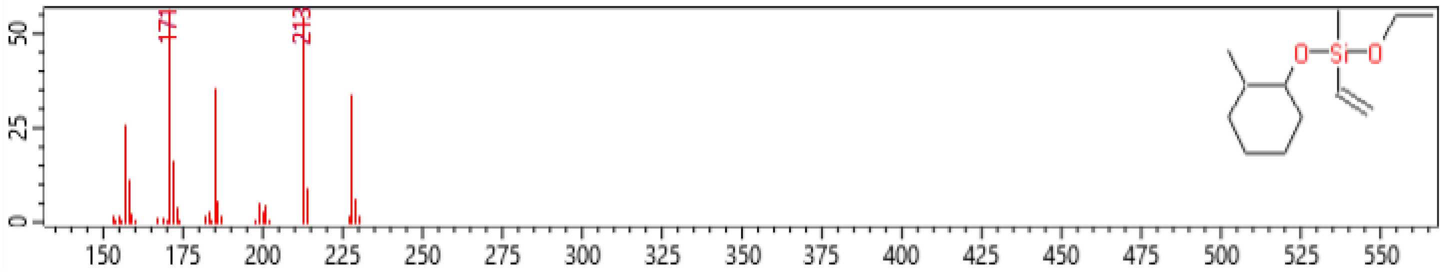

Silane, methylvinyl(cis-2-methylcyclohexyloxy)ethoxy-

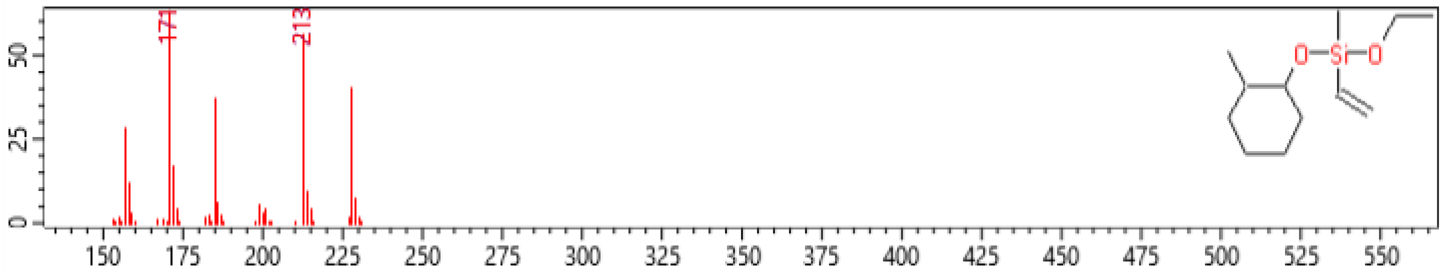

Silane, methylvinyl(cycloheptyloxy)ethoxy-

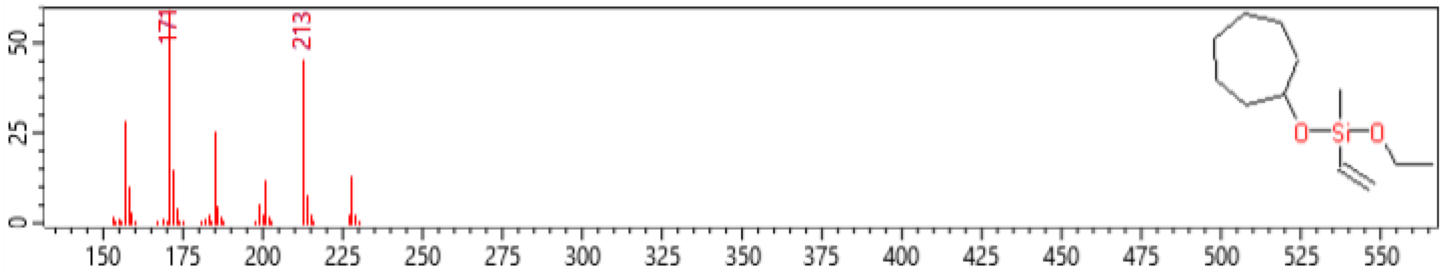

2-Isopropenyl-2,3-dihydrofuro[3,2-g]chromen-7-one

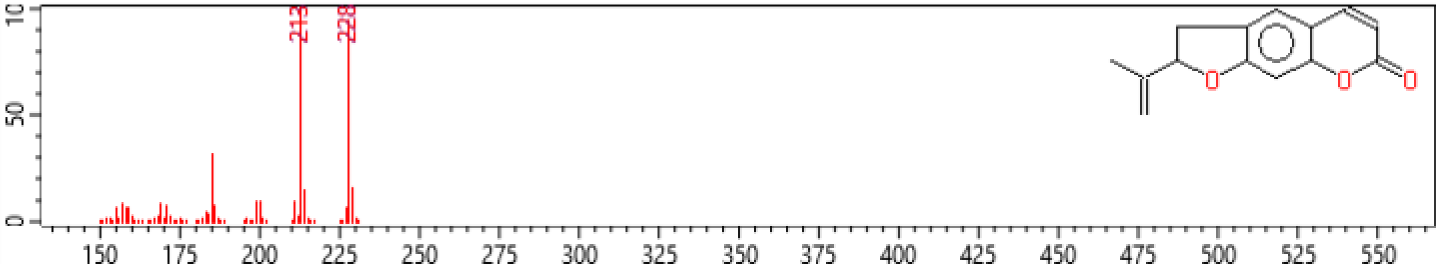

Hydroxychavicol, bis-O-TMS
Peak: 11.30 - 11.34 min (scan #1201 - #1211) Subtracted noise: 11.24 - 11.27 min (scan #1183 - #1192), 11.35 - 11.39 min (scan #12

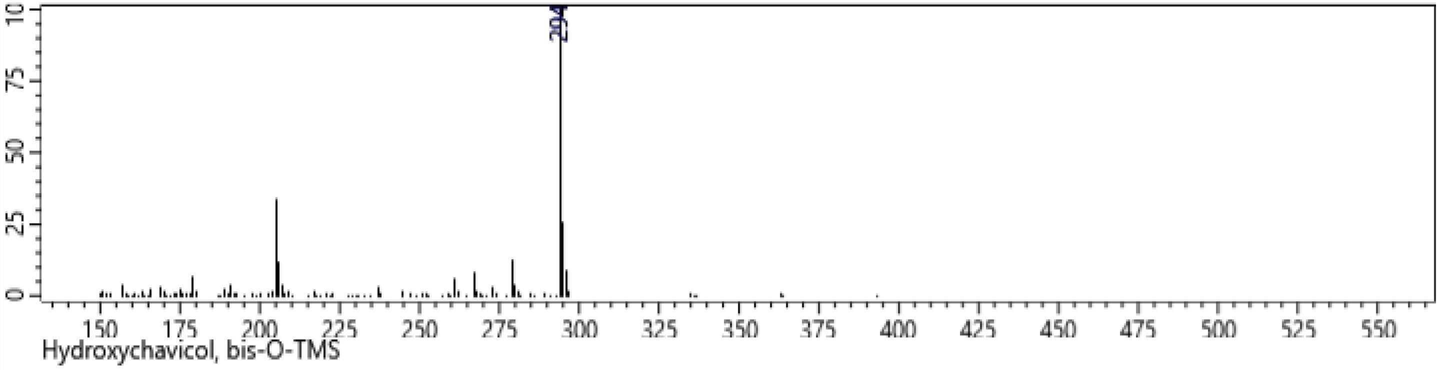

Hydroxychavicol, bis-O-TMS

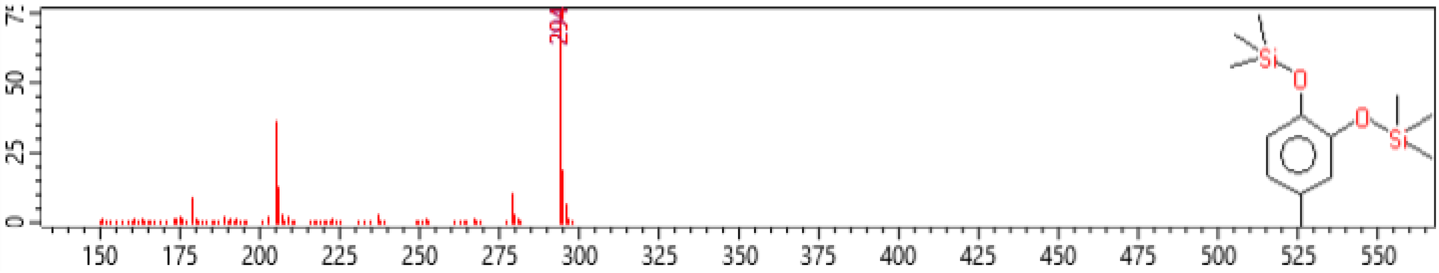

Aristolactam

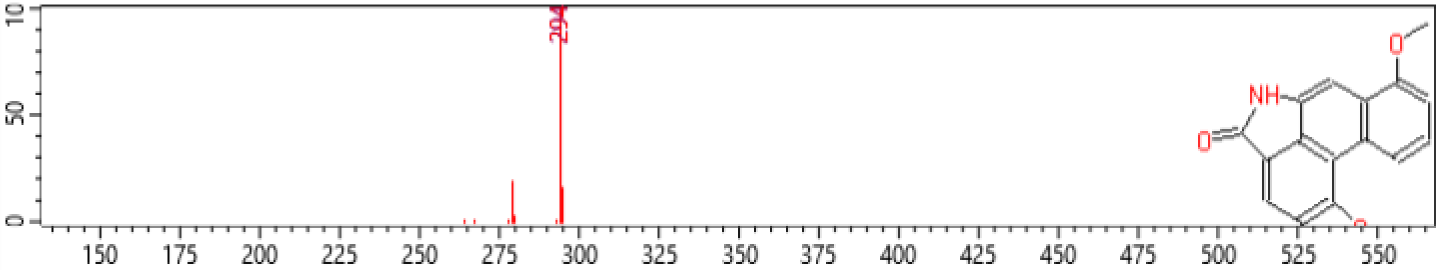

Aristolactam

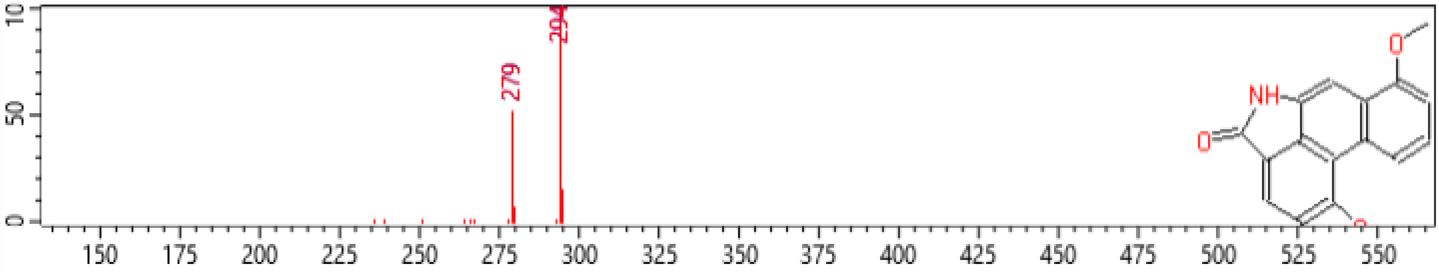

1-(1Z-Octadecenyl)-2-(5Z,8Z,11Z,14Z-eicosatetraenoyl)-sn-glycero-3-phosphoethanolamine

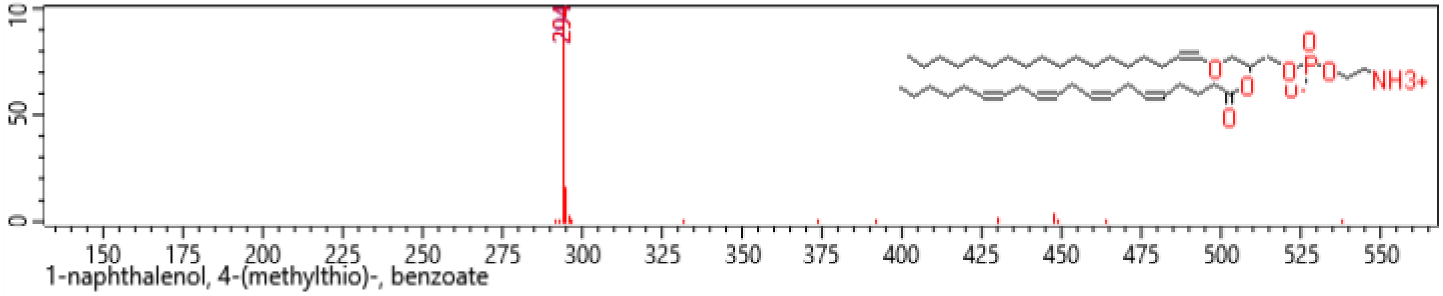

1-naphthalenol, 4-(methylthio)-, benzoate

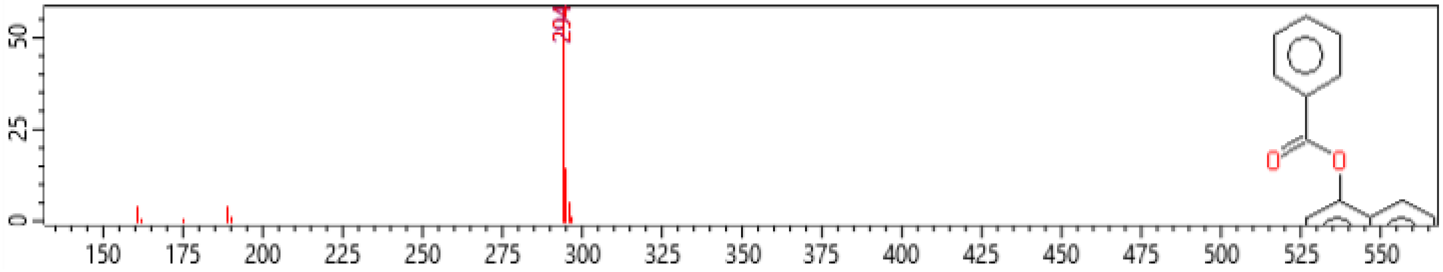

4-Ethyl-2,6-dimethoxyphenol, TMS

Peak: 11.45 - 11.51 min (scan #1242 - #1258) Subtracted noise: 11.34 - 11.40 min (scan #1212 - #1228), 11.53 - 11.59 min (scan #12

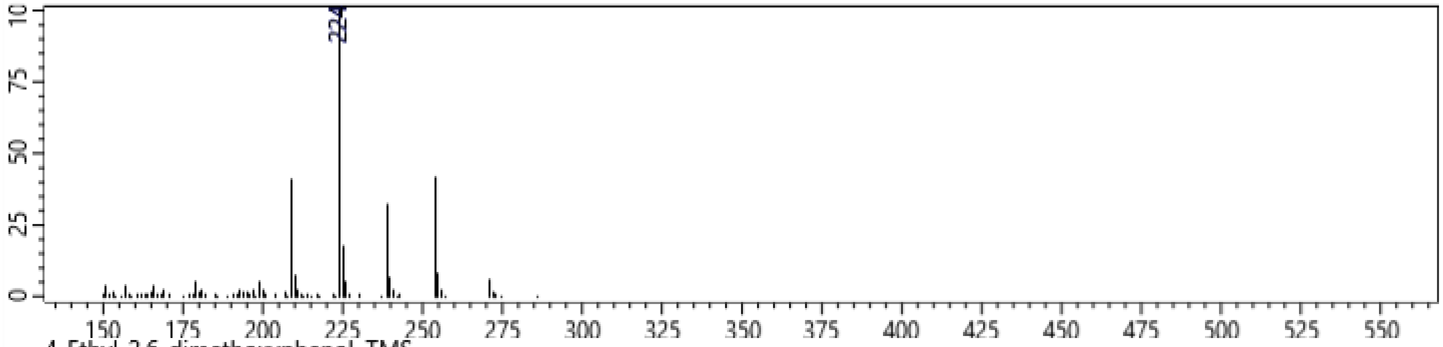

4-Ethyl-2,6-dimethoxyphenol, TMS

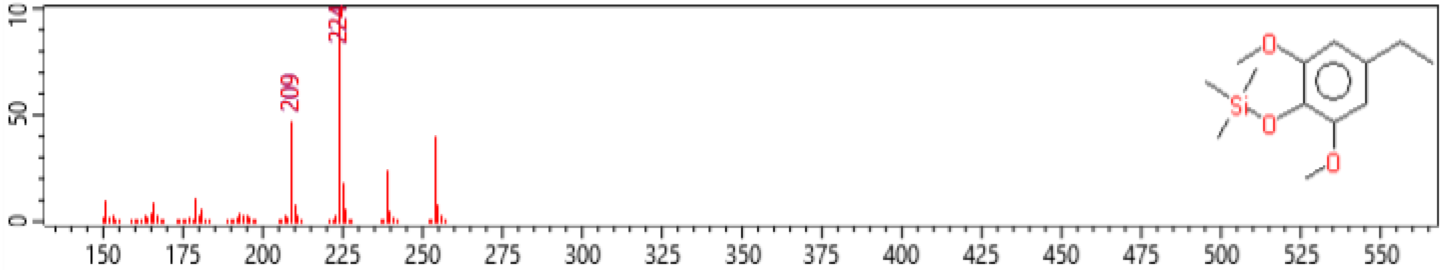

Syringaldehyde, TMS derivative

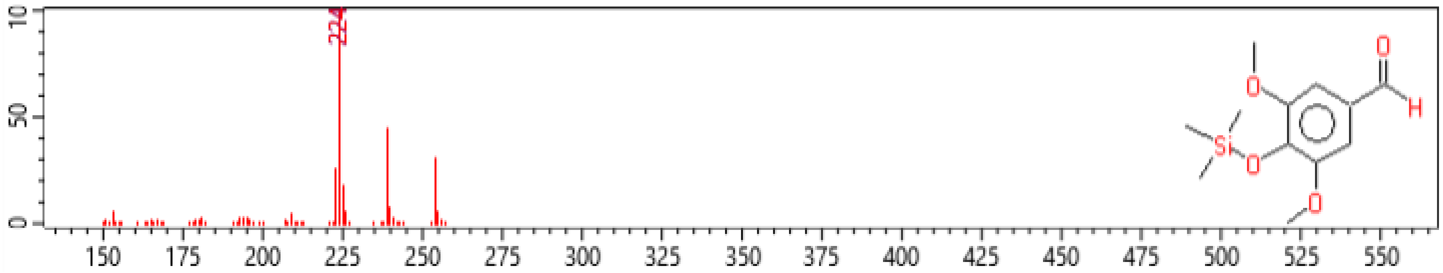

Syringaldehyde, TMS derivative

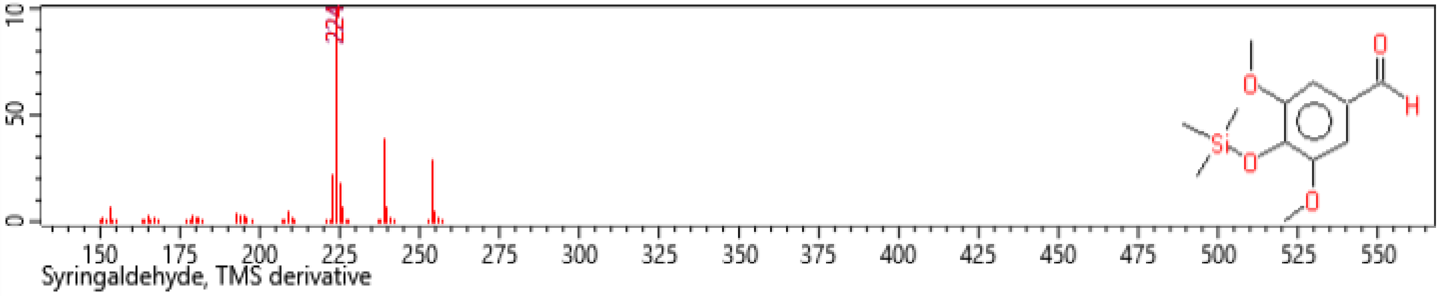

Syringaldehyde, TMS derivative

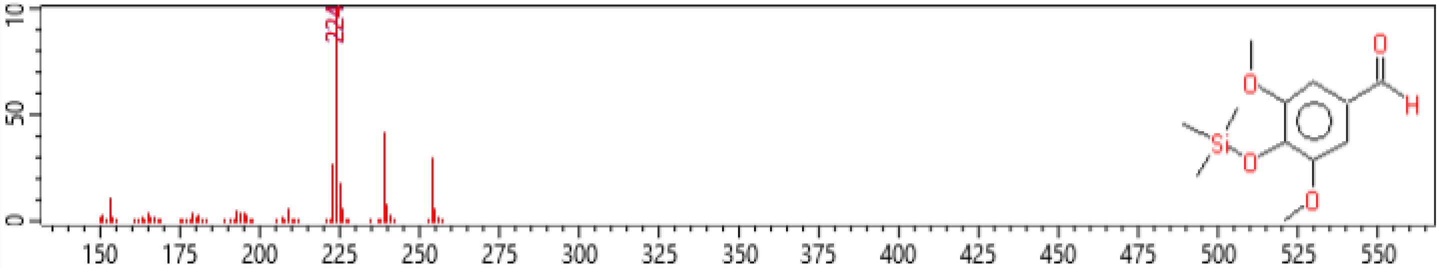

4-(Ethoxymethyl)-2-methoxyphenol, TMS

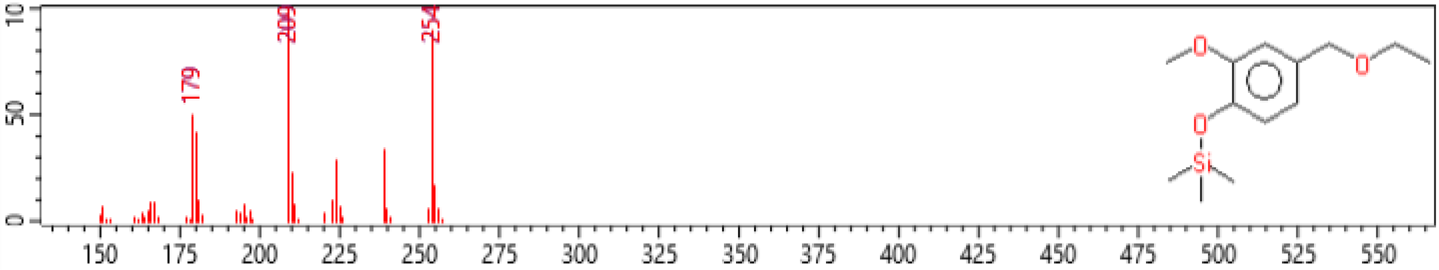

4-(4-Bromophenoxy)-6-chloro-2-pyrimidinylamine

Peak: 11.55 - 11.57 min (scan #1270 - #1276) Subtracted noise: 11.51 - 11.53 min (scan #1258 - #1263), 11.58 - 11.60 min (scan #12

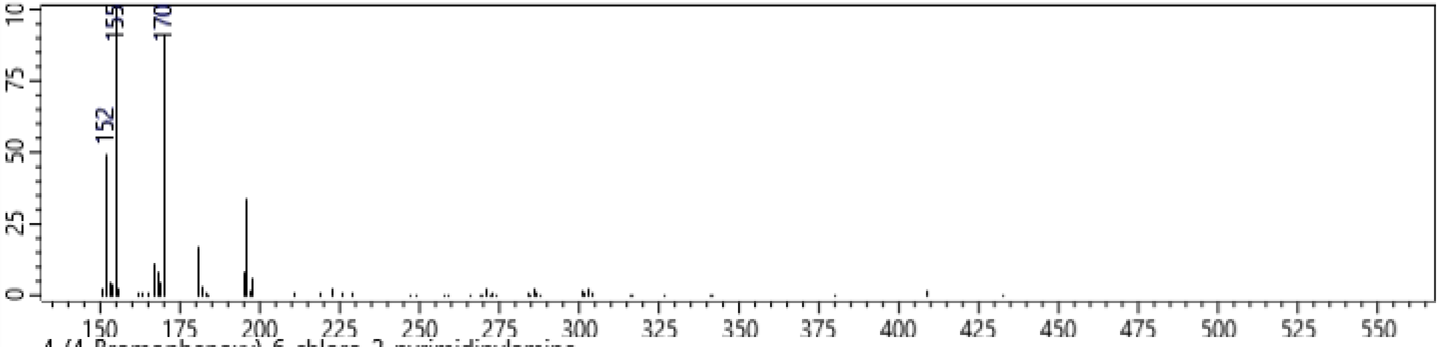

4-(4-Bromophenoxy)-6-chloro-2-pyrimidinylamine

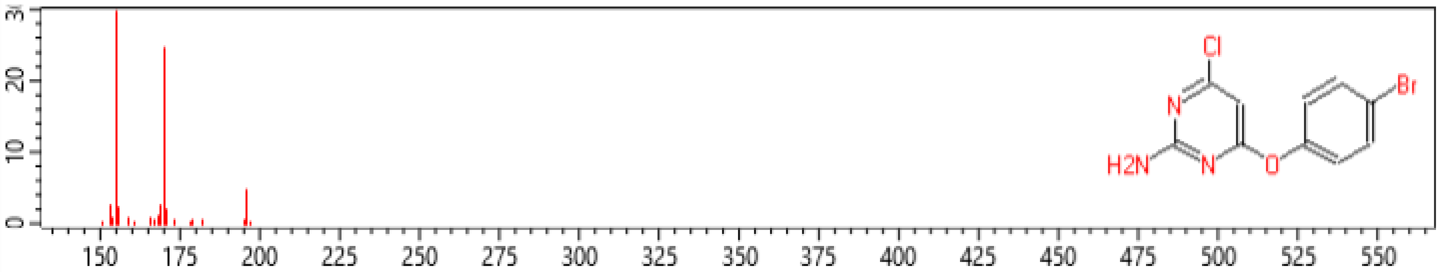

Scriptaid

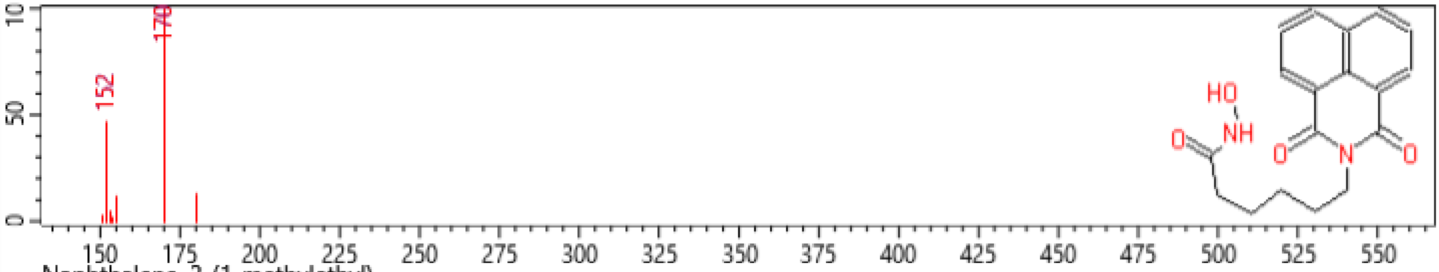

Naphthalene, 2-(1-methylethyl)-

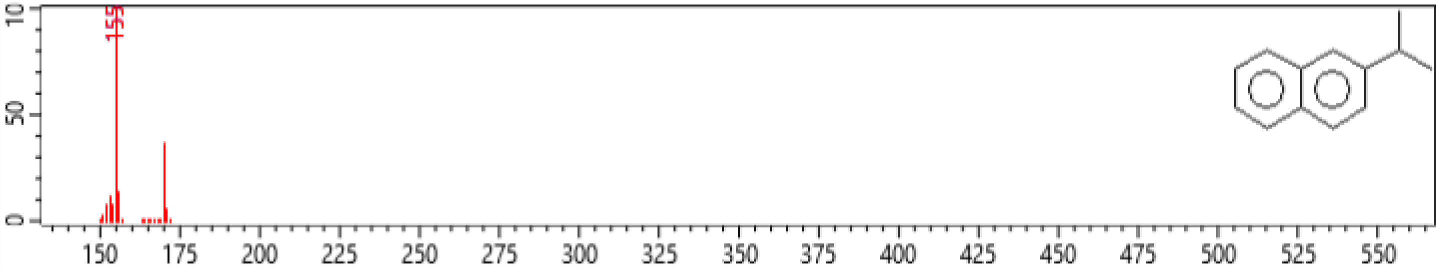

(S)-2-Ureidopentanedioic acid

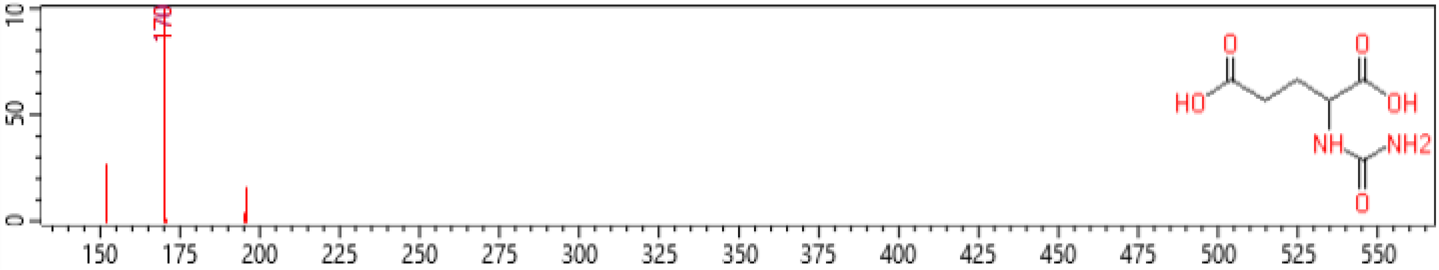

Naphthalene, 2,3,6-trimethyl-

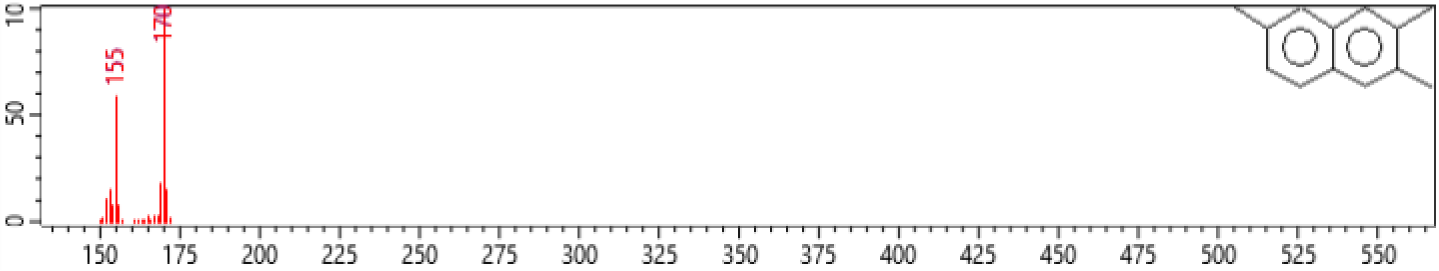

2,3,4-Trihydroxybutyric acid tetrakis(trimethylsilyl) deriv., (, (R*,R*)-)

Peak: 11.59 - 11.60 min (scan #1280 - #1284) Subtracted noise: 11.56 - 11.57 min (scan #1272 - #1276), 11.62 - 11.63 min (scan #12

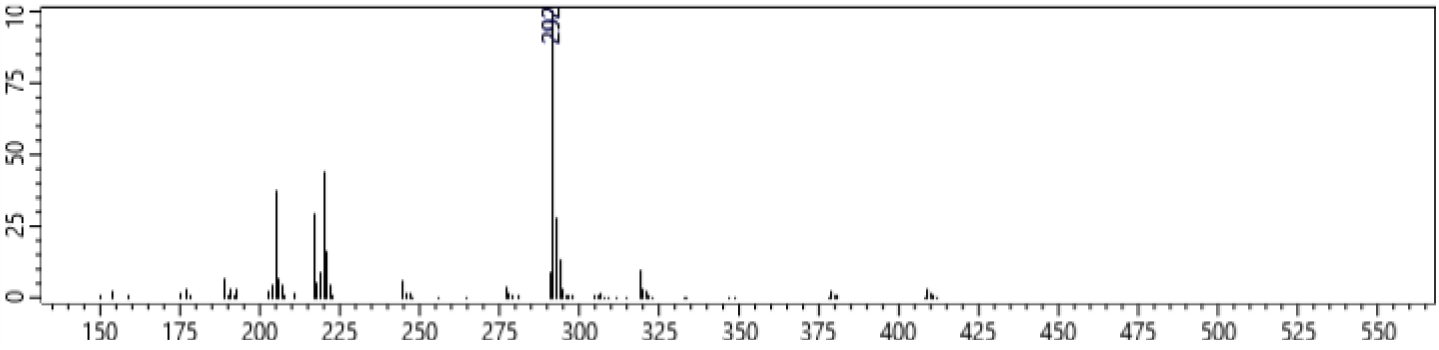

2,3,4-Trihydroxybutyric acid tetrakis(trimethylsilyl) deriv., (, (R*,R*)-)

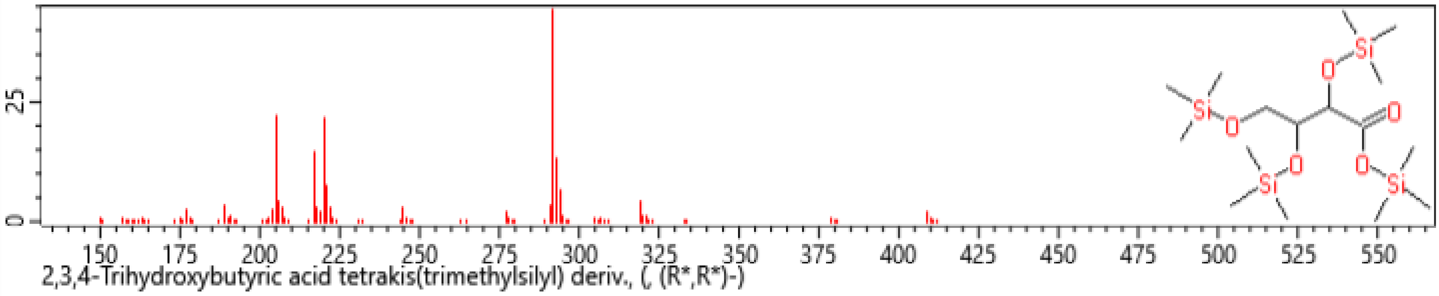

2,3,4-Trihydroxybutyric acid tetrakis(trimethylsilyl) deriv., (, (R*,R*)-)

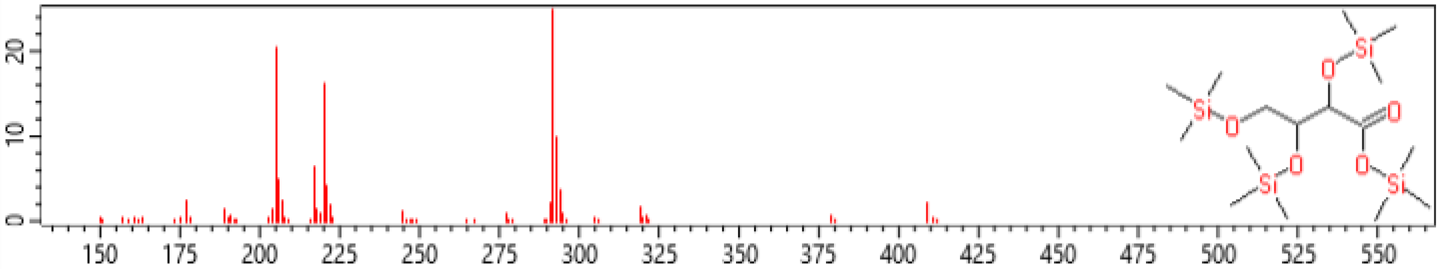

2,3,4-Trihydroxybutyric acid tetrakis(trimethylsilyl) deriv., (, (R*,R*)-)

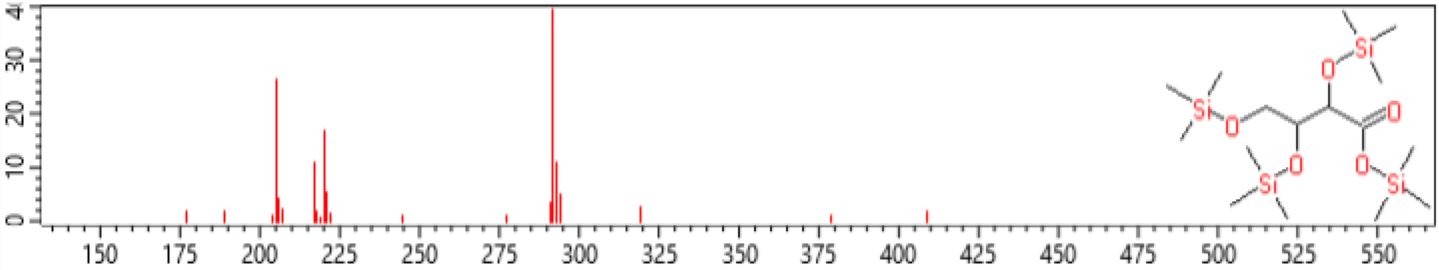

2,3,4-Trihydroxybutyric acid tetrakis(trimethylsilyl) deriv., (, (R*,R*)-)

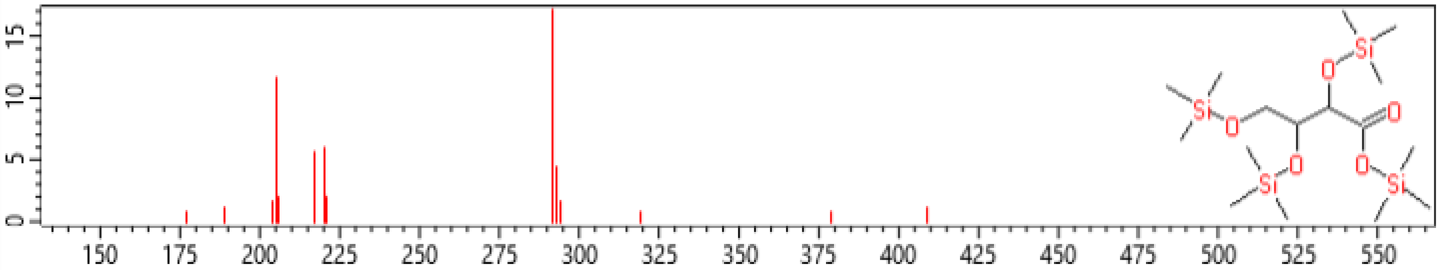

(R*,S*)-2,3-Dihydroxybutanoic acid, tris(trimethylsilyl) deriv.

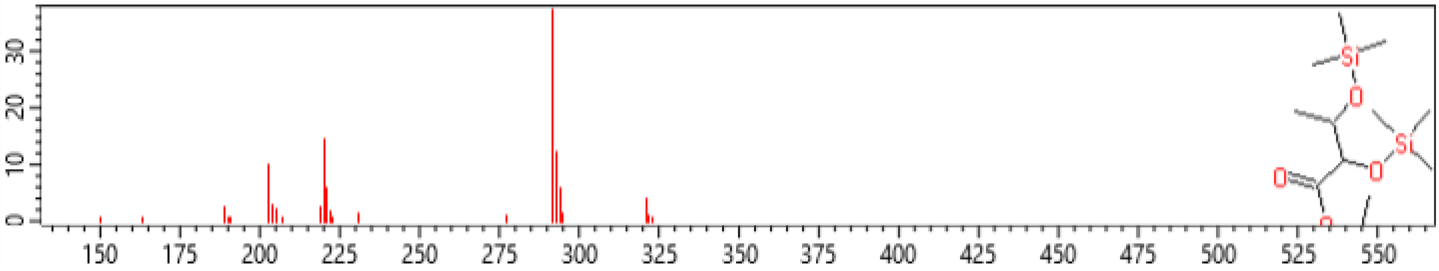

Tyrosol, 2TMS derivative

Peak: 11.68 - 11.75 min (scan #1307 - #1325) Subtracted noise: 11.55 - 11.61 min (scan #1269 - #1287), 11.76 - 11.82 min (scan #13

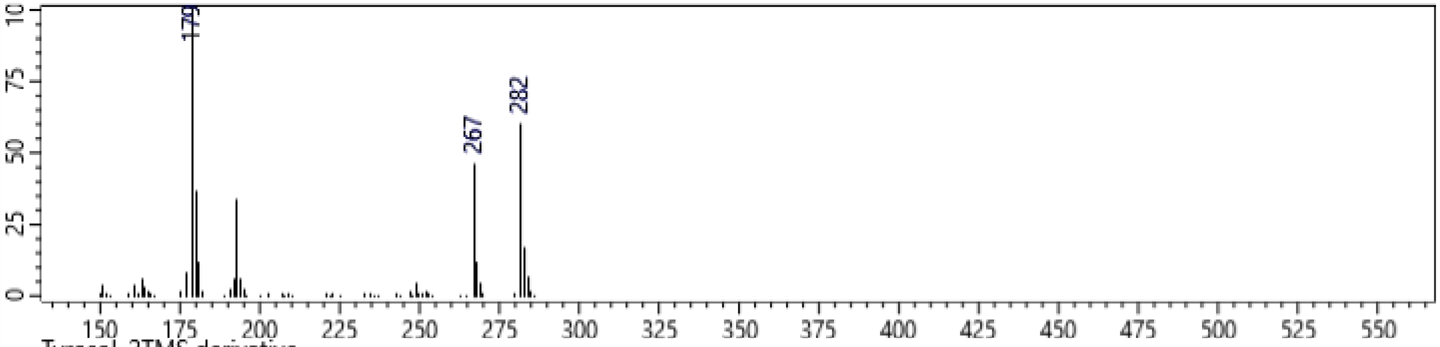

Tyrosol, 2TMS derivative

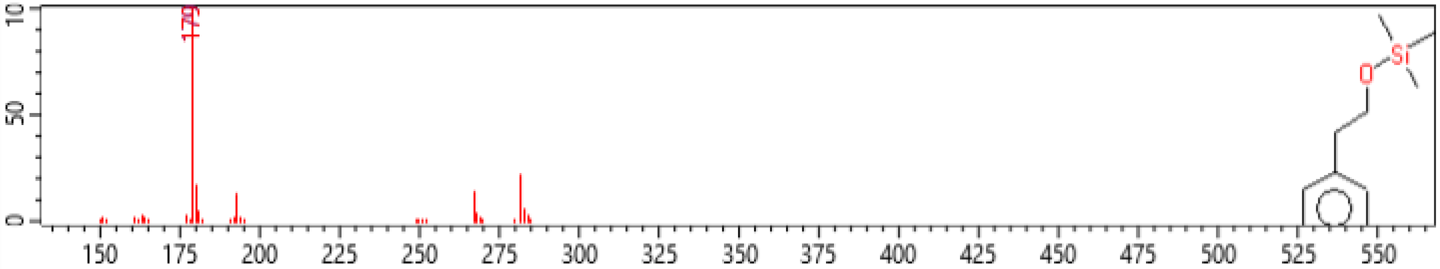

Tyrosol, 2TMS derivative

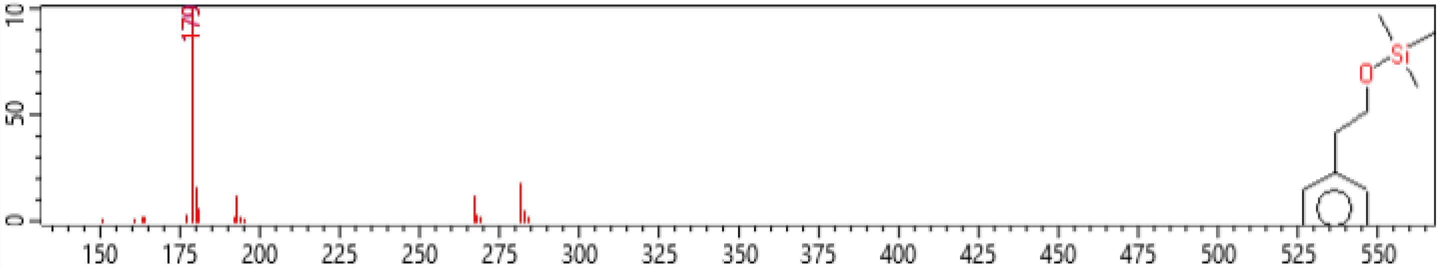

Tyrosol, 2TMS derivative

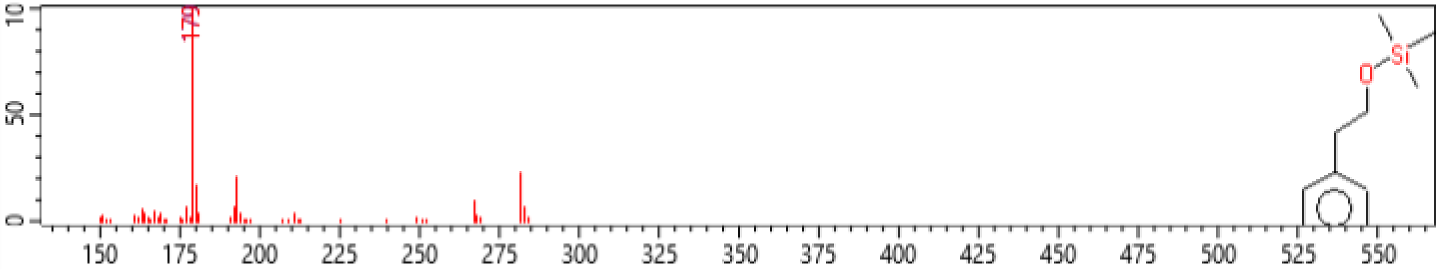

2-Hydroxyphenethyl alcohol, 2TMS derivative

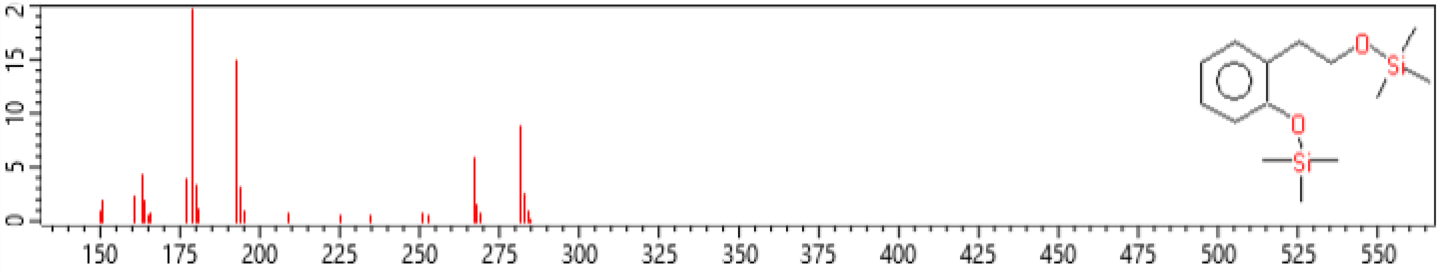

3-(2-Hydroxyethyl)phenol, 2TMS derivative

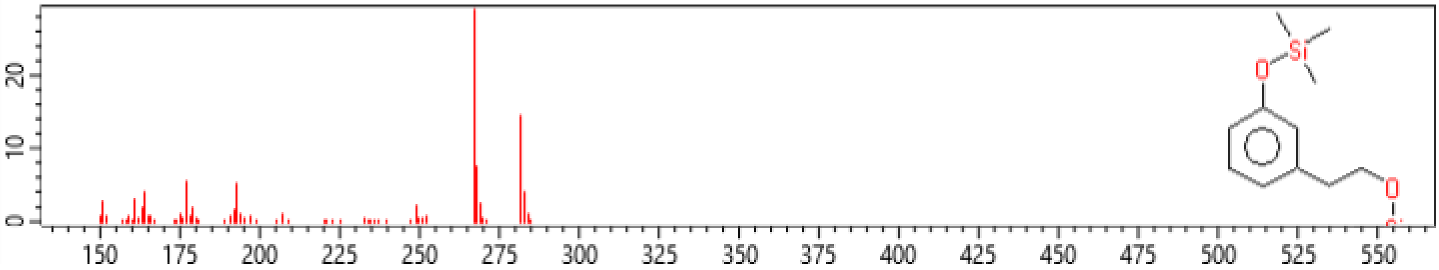

2-Isopropylmalic acid, O-trimethylsilyl-, bis(trimethylsilyl) ester

Peak: 11.76 - 11.78 min (scan #1328 - #1333) Subtracted noise: 11.73 - 11.75 min (scan #1321 - #1326), 11.79 - 11.81 min (scan #13

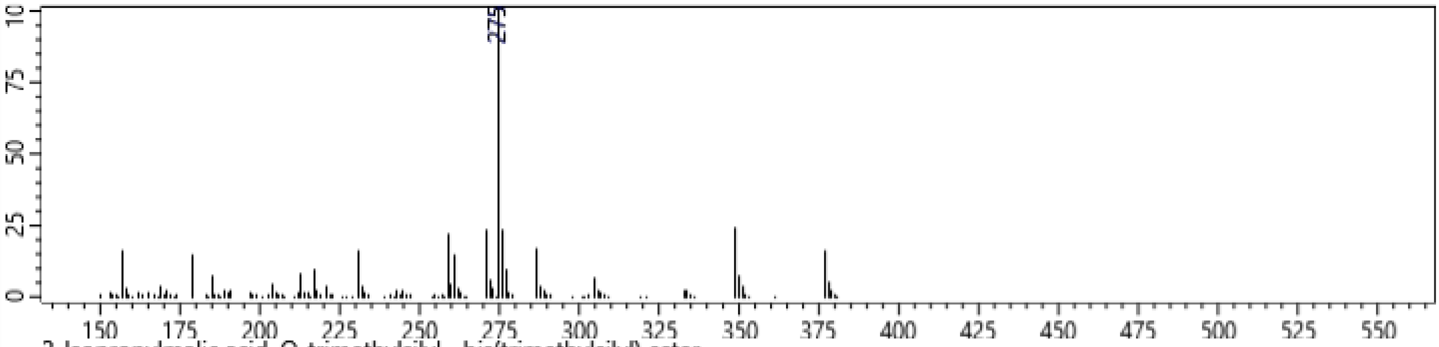

2-Isopropylmalic acid, O-trimethylsilyl-, bis(trimethylsilyl) ester

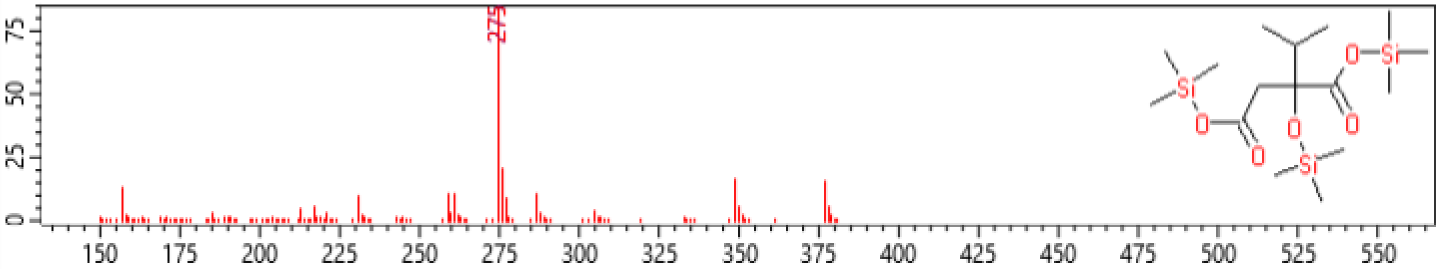

2-Carboxy-3-methyl-succinate, tris(trimethylsilyl)-

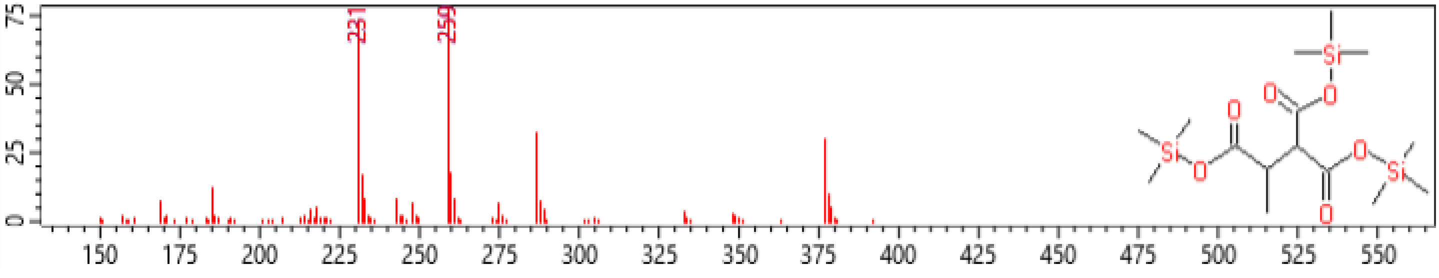

2-[(1,3-Thiazol-2-ylamino)carbonyl]benzoic acid, 2TMS derivative

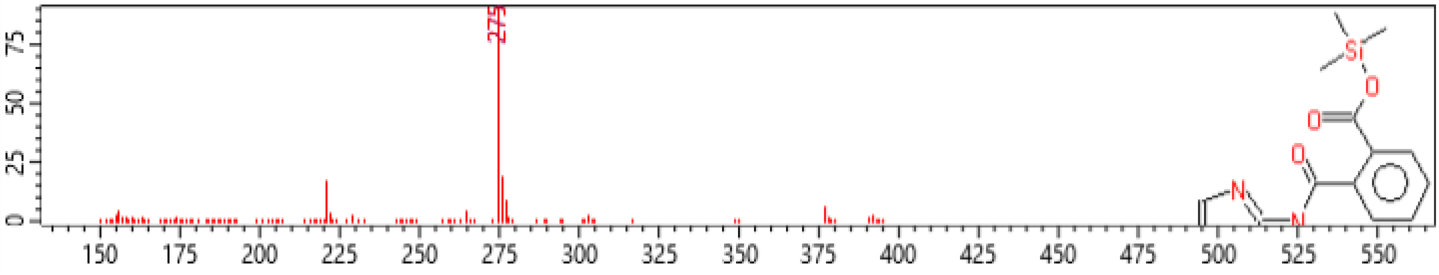

4-(4-Methylquinolin-2-yl)-3,4-dihydro-2H-benzo[b][1,4]oxazine

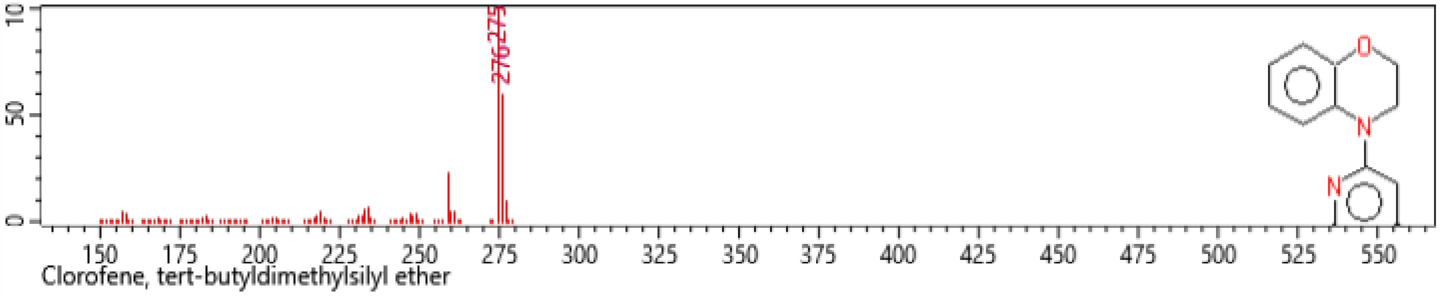

Clorofene, tert-butyldimethylsilyl ether

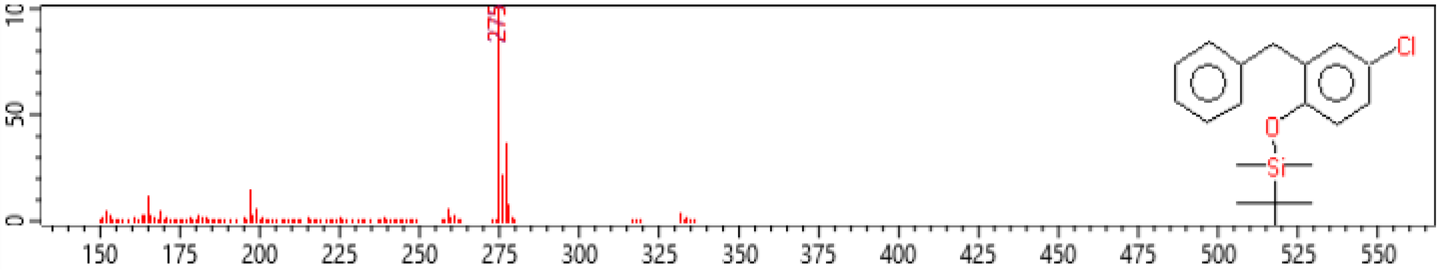

N-(1-Hydroxy-3-phenylpropan-2-yl)-3-(4-hydroxyquinazolin-2-yl)propanamide

Peak: 11.83 - 11.86 min (scan #1347 - #1355) Subtracted noise: 11.77 - 11.79 min (scan #1330 - #1337), 11.86 - 11.88 min (scan #13

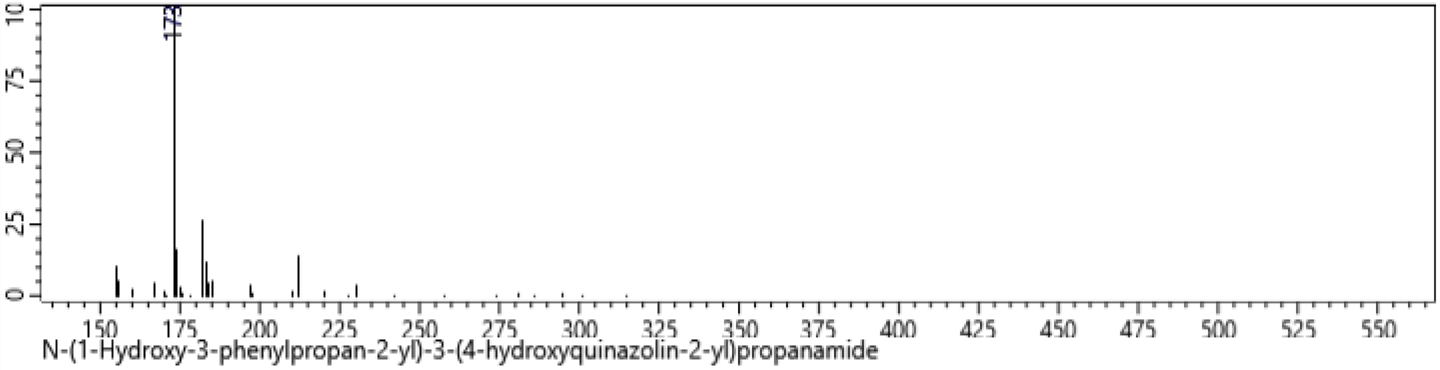

N-(1-Hydroxy-3-phenylpropan-2-yl)-3-(4-hydroxyquinazolin-2-yl)propanamide

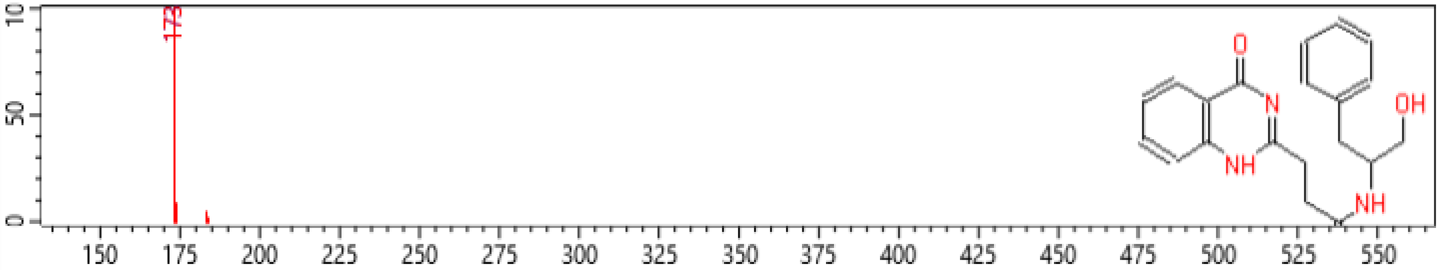

2,6-Diaminopimelic acid

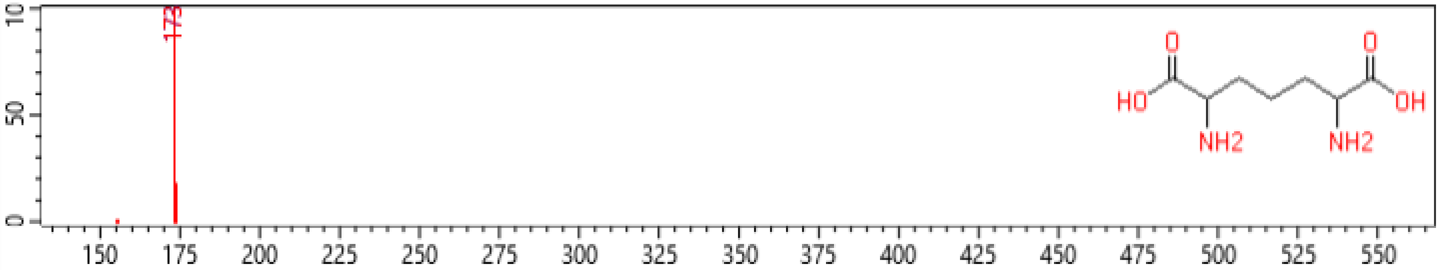

Leu-Ser

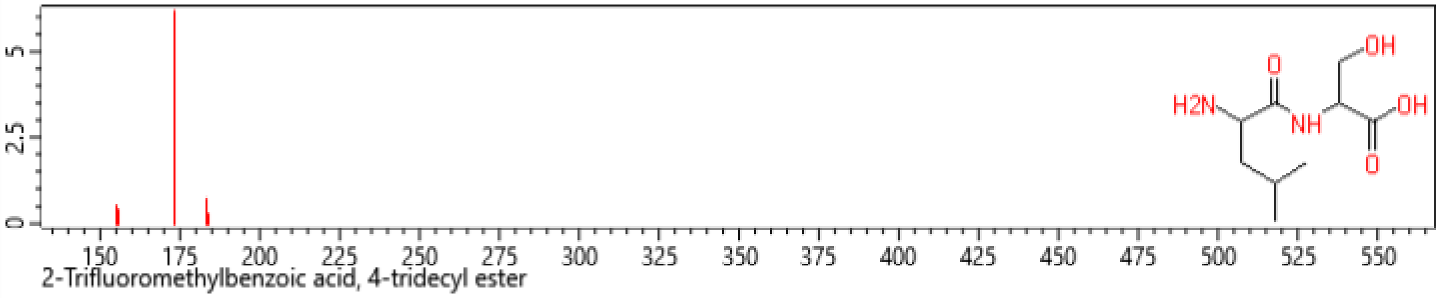

2-Trifluoromethylbenzoic acid, 4-tridecyl ester

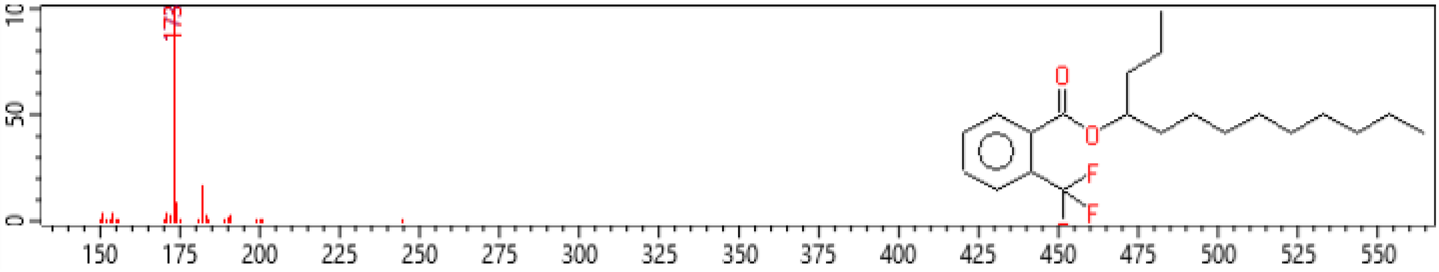

2-Trifluoromethylbenzoic acid, pentafluorophenyl ester

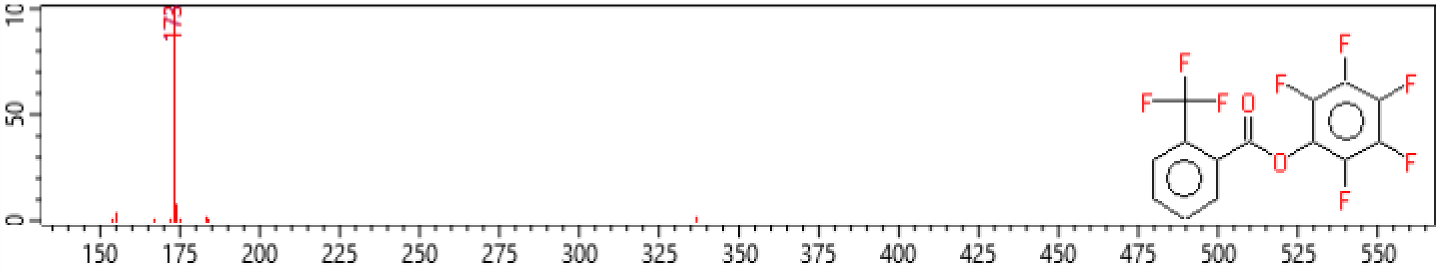

3a,9b-Dimethyl-1,2,3a,4,5,9b-hexahydrocyclopenta[a]naphthalen-3-one

Peak: 11.86 - 11.88 min (scan #1357 - #1362) Subtracted noise: 11.83 - 11.86 min (scan #1349 - #1355), 11.90 - 11.92 min (scan #13

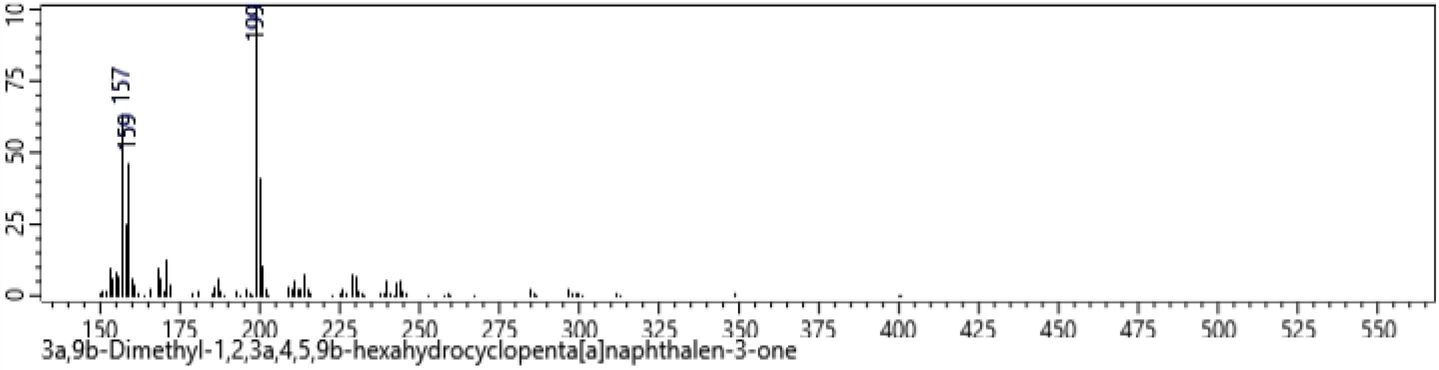

3a,9b-Dimethyl-1,2,3a,4,5,9b-hexahydrocyclopenta[a]naphthalen-3-one

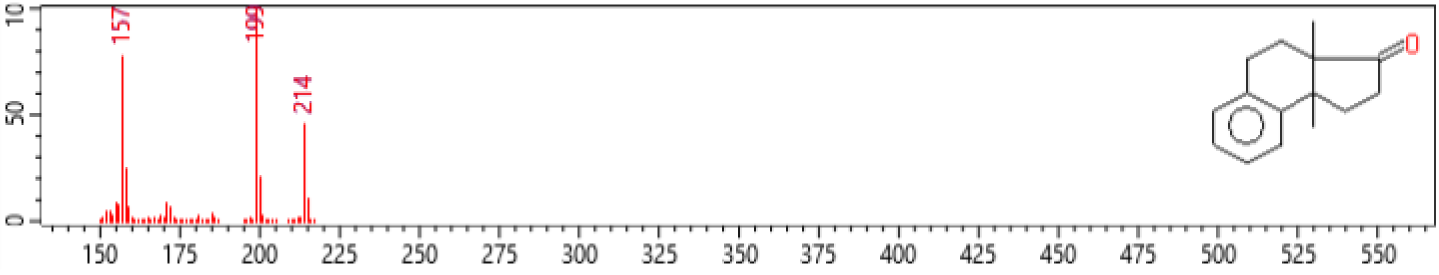

2,3-Dihydroxypropyl 9-hydroxynonanoate, triacetate

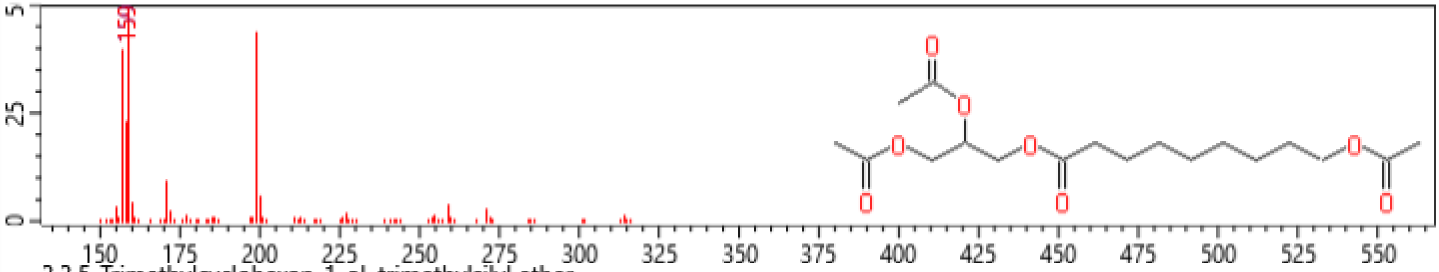

3,3,5-Trimethylcyclohexan-1-ol, trimethylsilyl ether

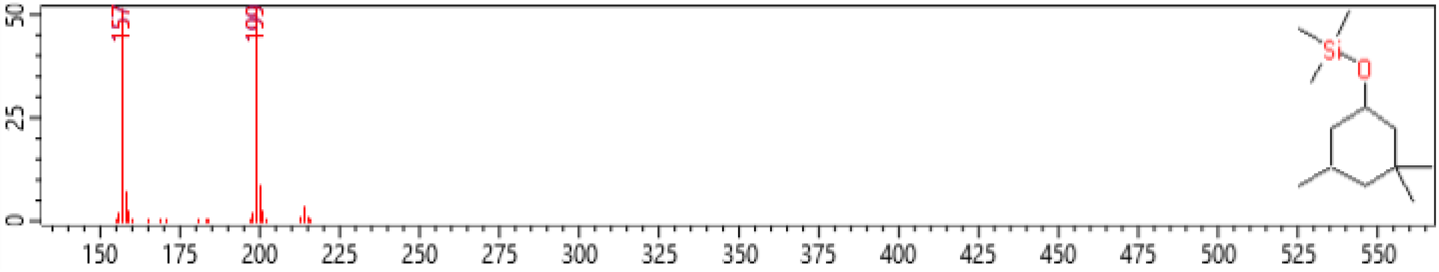

Acetamide, N-(4-chloro-2-methoxyphenyl)-

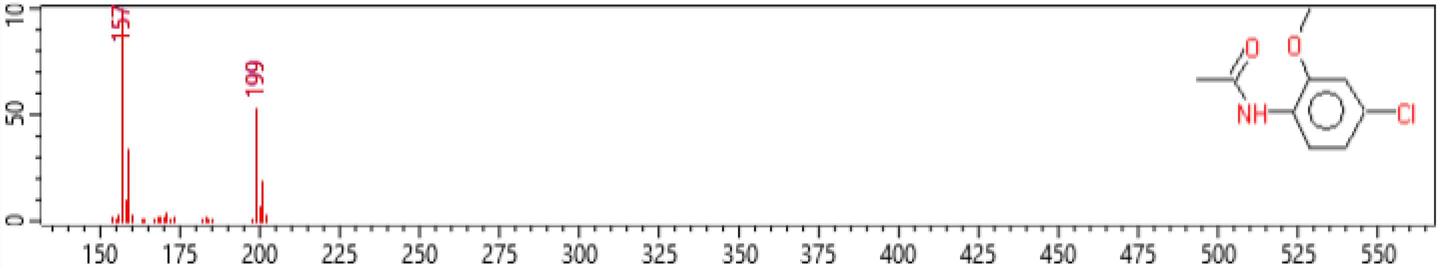

Acetamide, N-(3-chloro-4-methoxyphenyl)-

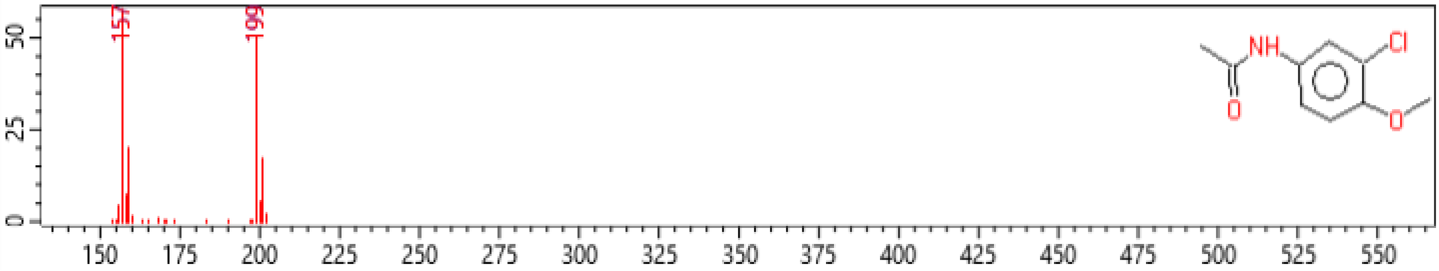

Pimelic acid, 2TMS derivative

Peak: 11.93 - 11.96 min (scan #1376 - #1384) Subtracted noise: 11.87 - 11.90 min (scan #1358 - #1366), 11.97 - 12.00 min (scan #13

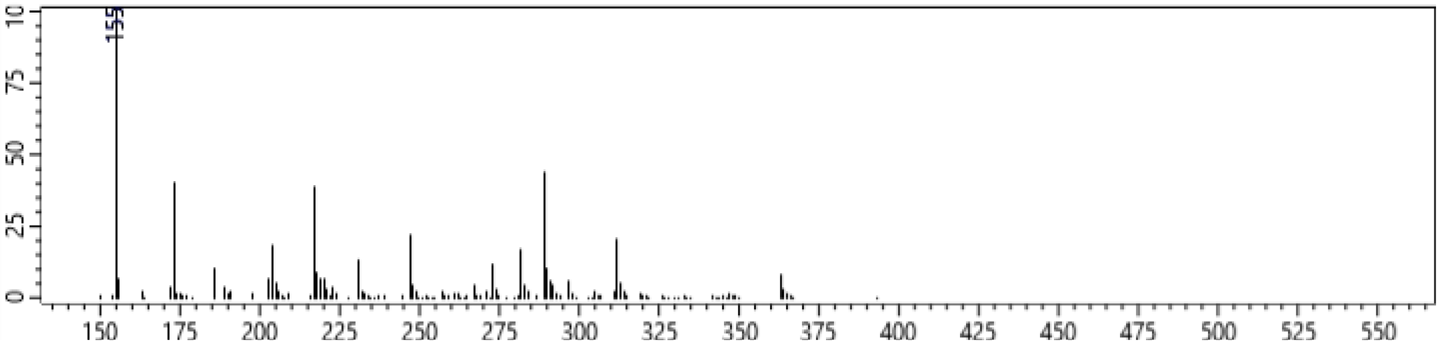

Pimelic acid, 2TMS derivative

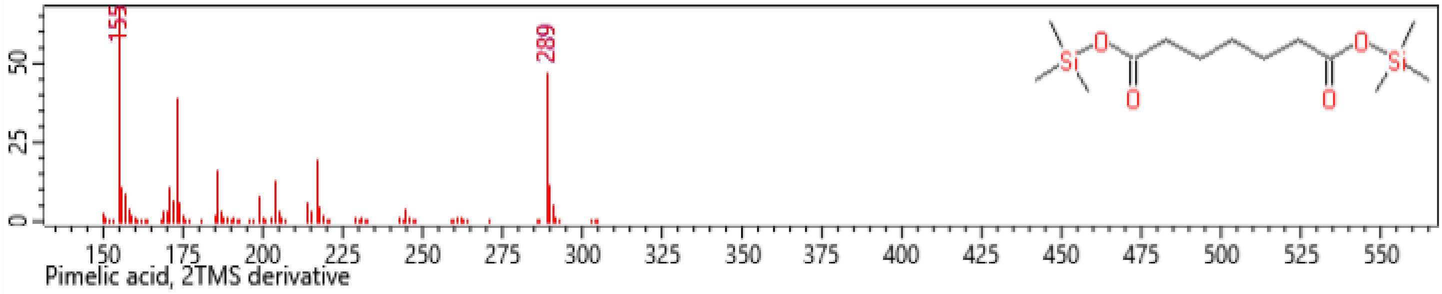

Pimelic acid, 2TMS derivative

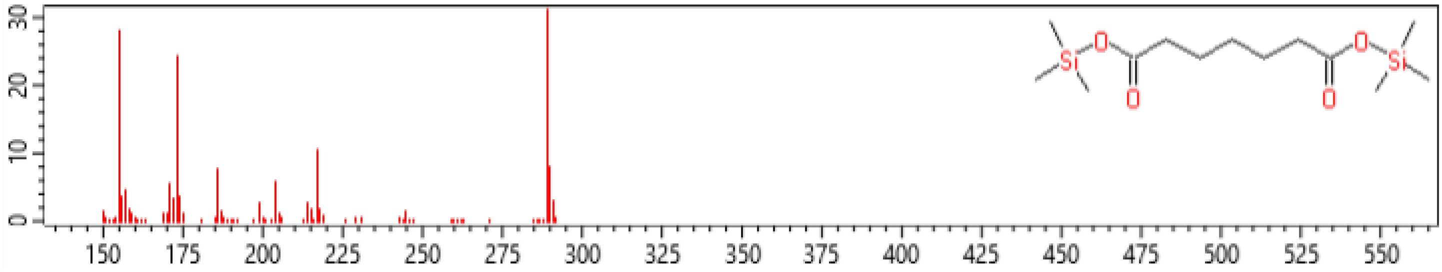

Pimelic acid, 2TMS derivative

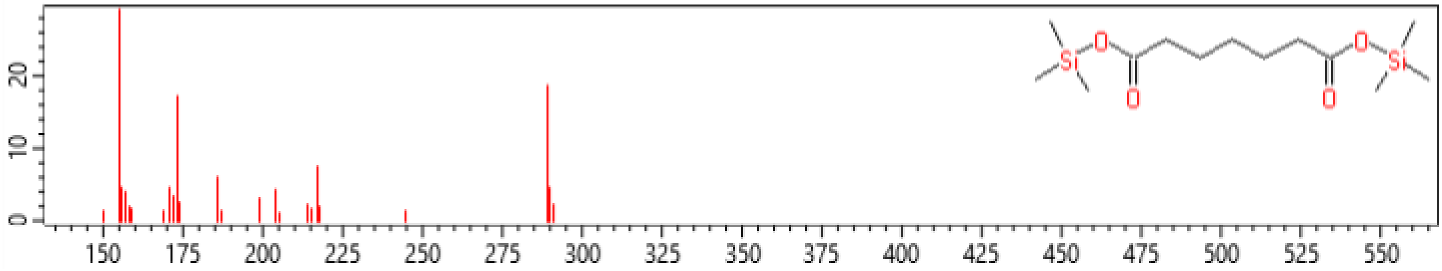

3-Methyladipic acid, 2TMS derivative

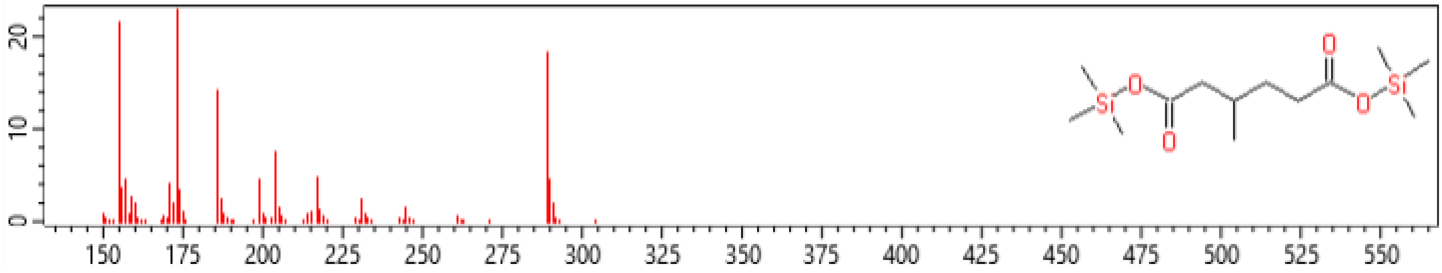

3-Methyladipic acid, 2TMS derivative

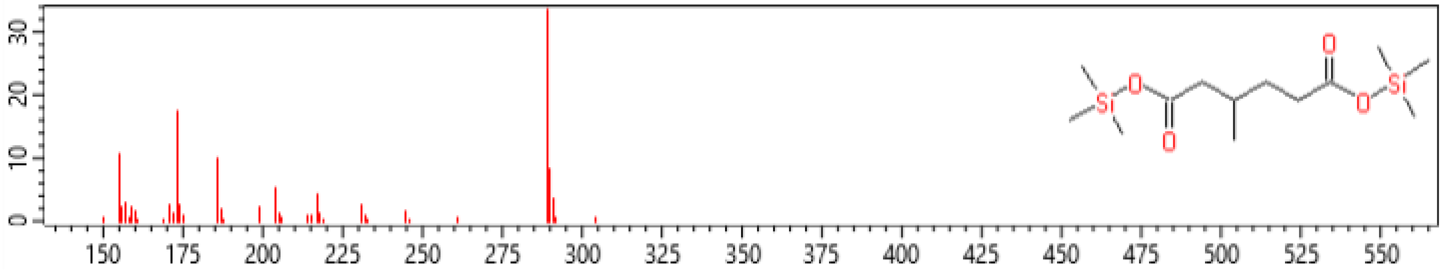

3,3,5-Trimethylcyclohexan-1-ol, trimethylsilyl ether

Peak: 11.99 - 12.01 min (scan #1393 - #1399) Subtracted noise: 11.94 - 11.96 min (scan #1379 - #1385), 12.02 - 12.05 min (scan #14

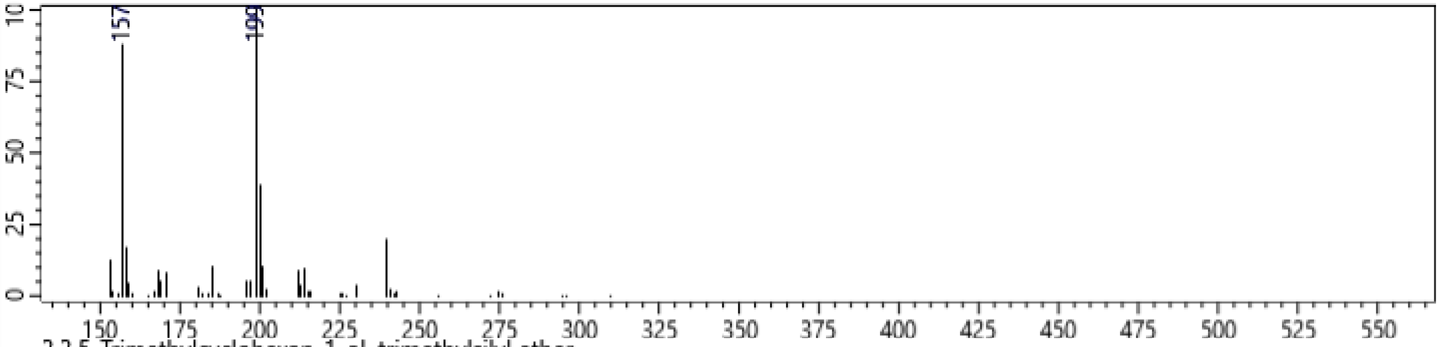

3,3,5-Trimethylcyclohexan-1-ol, trimethylsilyl ether

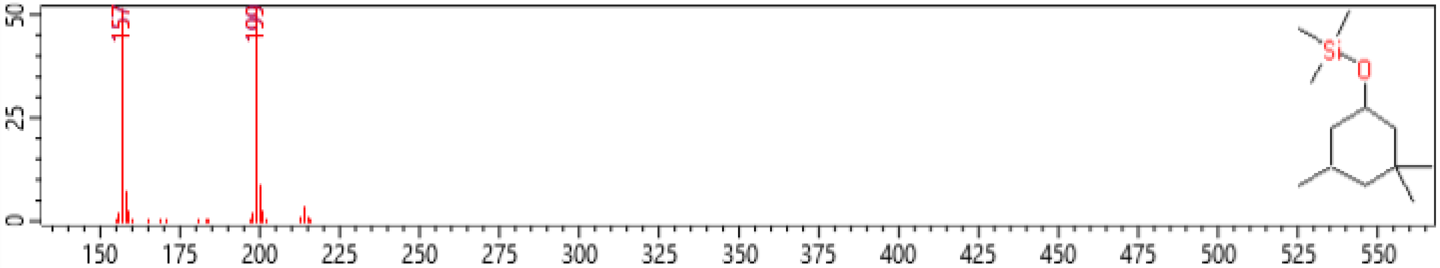

3a,9b-Dimethyl-1,2,3a,4,5,9b-hexahydrocyclopenta[a]naphthalen-3-one

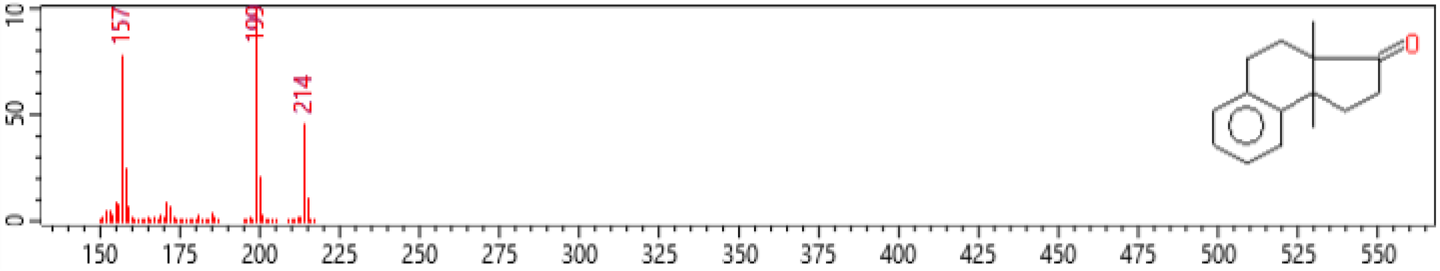

N-(4-Imidazo[2,1-b][1,3]thiazol-6-ylphenyl)acetamide

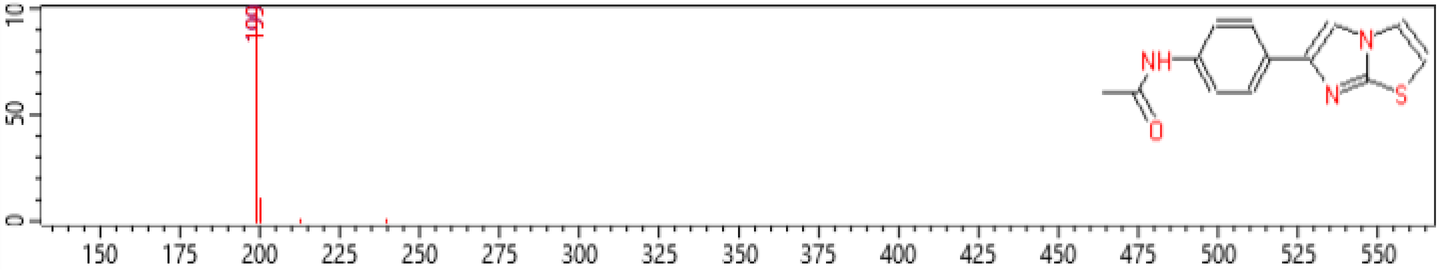

N-(4-Imidazo[2,1-b][1,3]thiazol-6-ylphenyl)acetamide

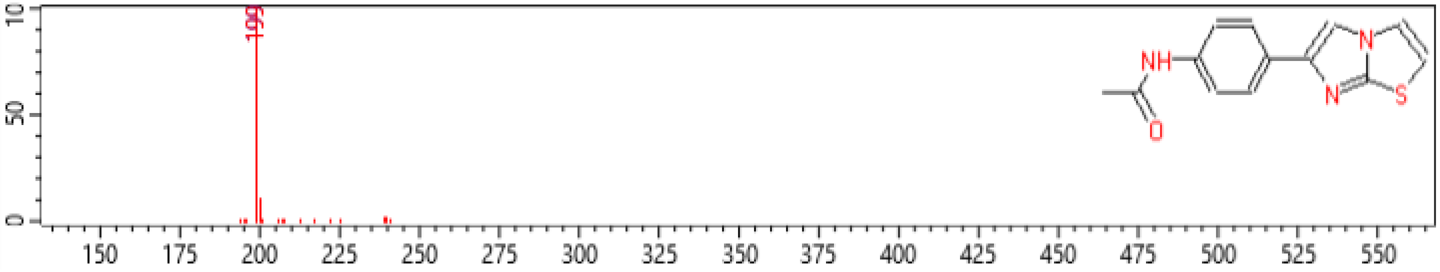

Butyl(dimethyl)silyloxycyclohexane

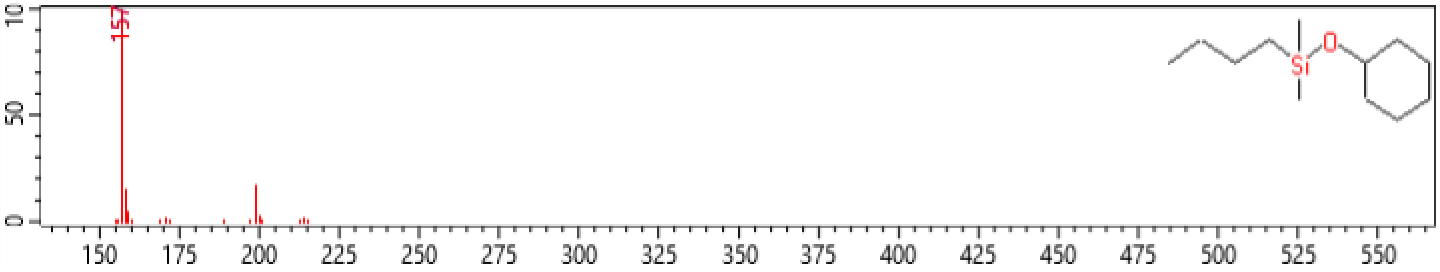

Salidroside, 5O-TMS

Peak: 12.05 - 12.09 min (scan #1409 - #1419) Subtracted noise: 11.98 - 12.02 min (scan #1390 - #1401), 12.11 - 12.14 min (scan #14

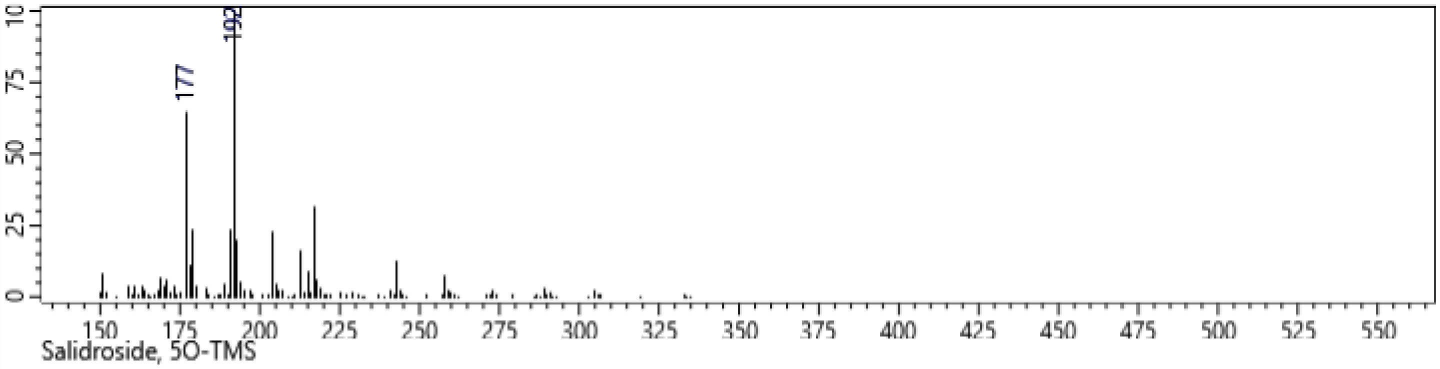

Salidroside, 5O-TMS

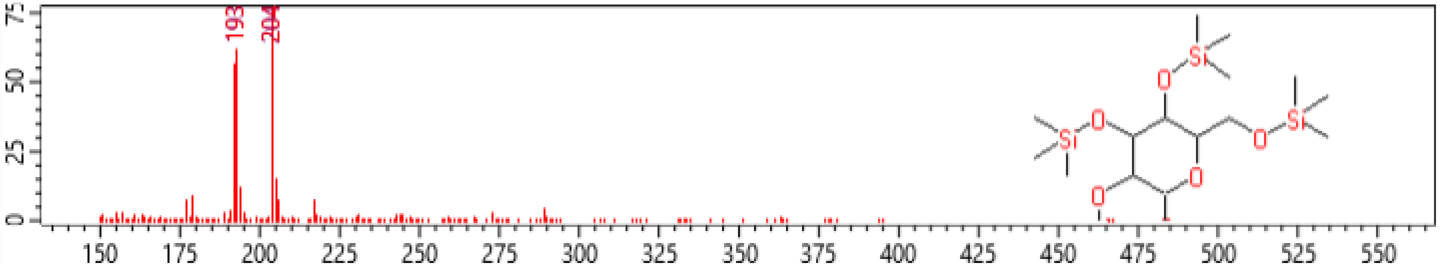

4-((Trimethylsilyl)oxy)phenethyl acetate

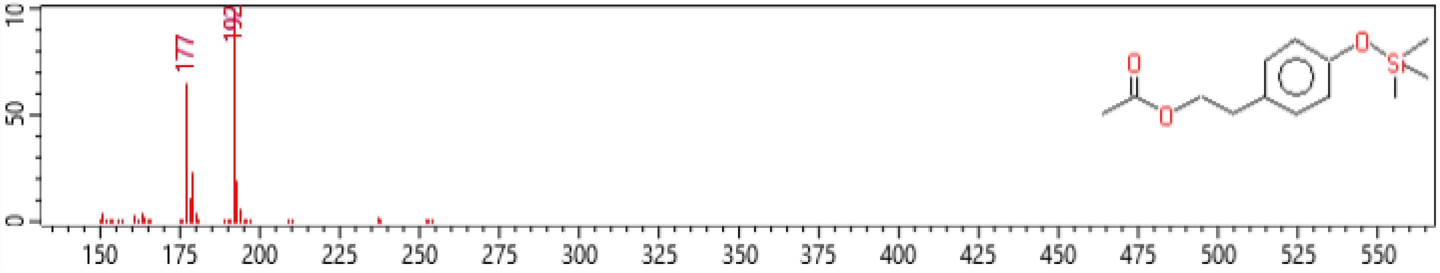

(1S,2S,8aR)-1-((Z)-5-Hydroxy-3-methylpent-3-en-1-yl)-2,5,5,8a-tetramethyldecahydronaphthalen-2-ol

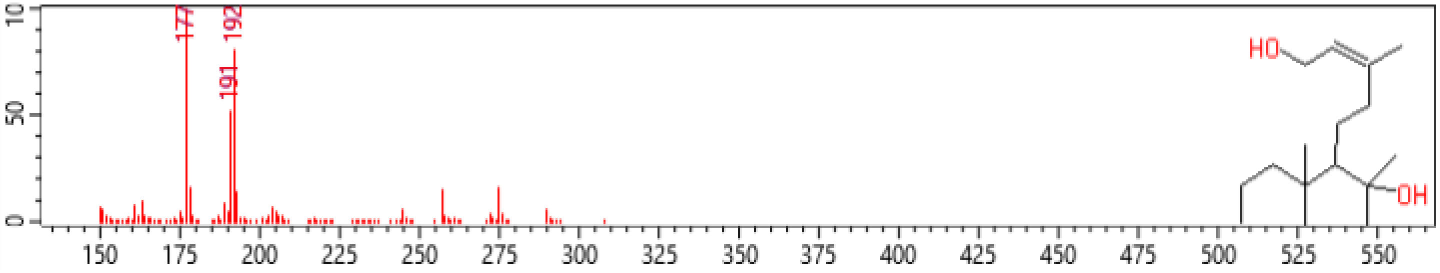

2-O-(2-(4-hydroxyphenyl)-ethyl)-d-β-glucopyranose, 5TMS

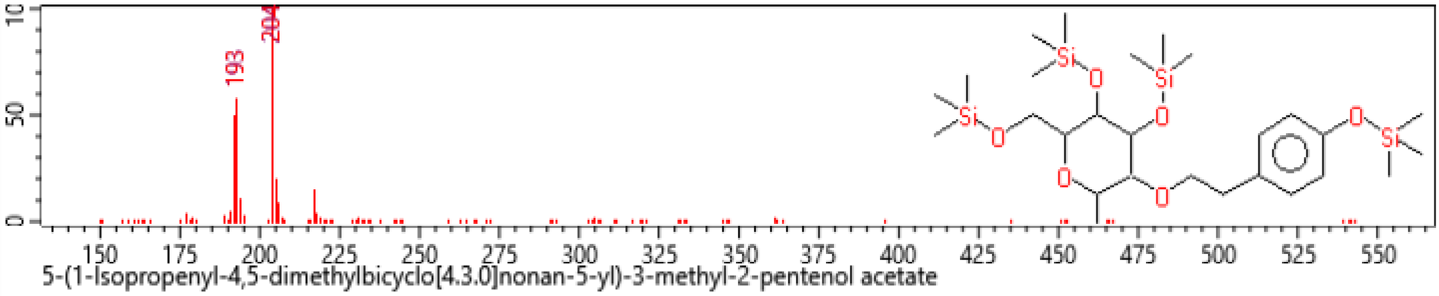

5-(1-Isopropenyl-4,5-dimethylbicyclo[4.3.0]nonan-5-yl)-3-methyl-2-pentenol acetate

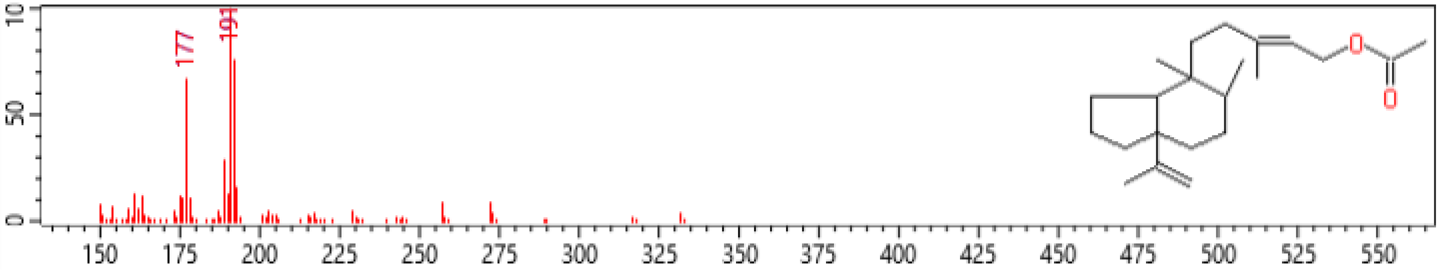

N-Acetyl-2,4,5-trimethoxyamphetamine

Peak: 12.11 - 12.13 min (scan #1427 - #1431) Subtracted noise: 12.09 - 12.10 min (scan #1419 - #1424), 12.14 - 12.16 min (scan #14

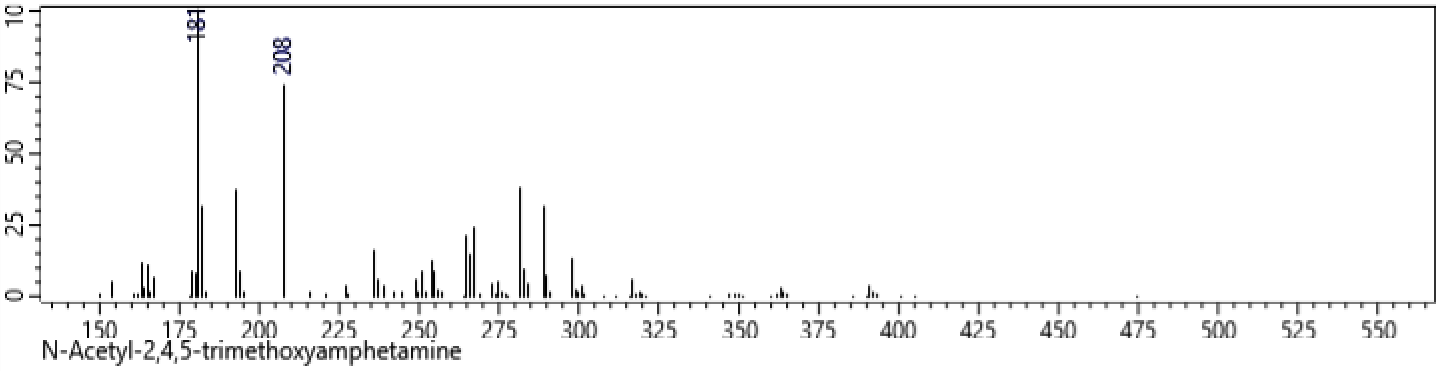

N-Acetyl-2,4,5-trimethoxyamphetamine

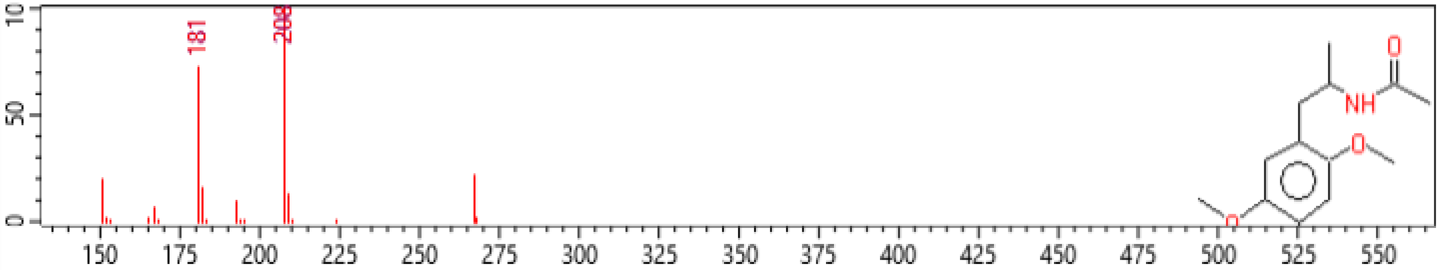

3,4,5-Trimethoxyphenylprop-2-amine, N-acetyl-

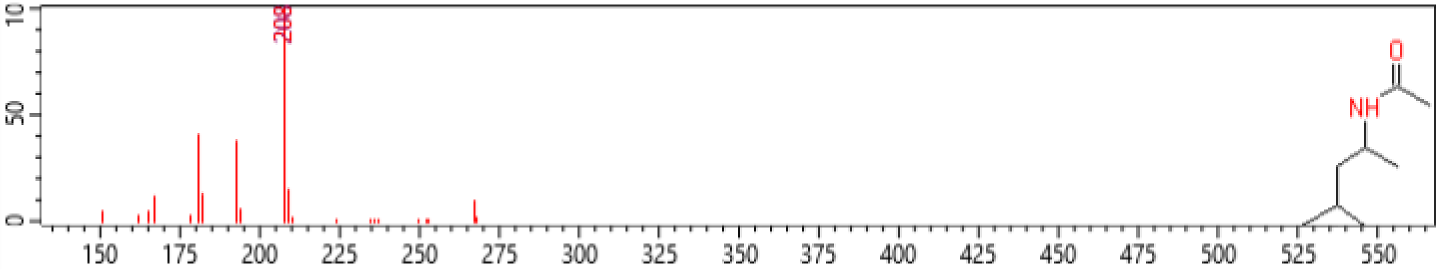

Sarpagan-17-ol, 16-[(acetyloxy)methyl]-, acetate (ester)

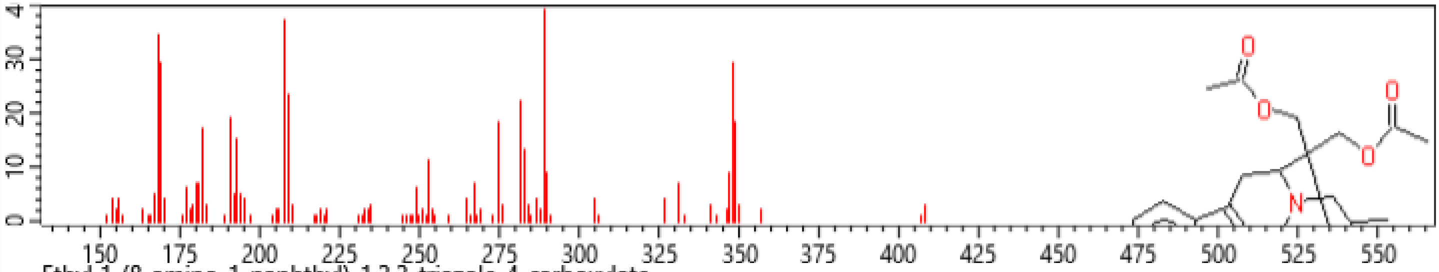

Ethyl 1-(8-amino-1-naphthyl)-1,2,3-triazole-4-carboxylate

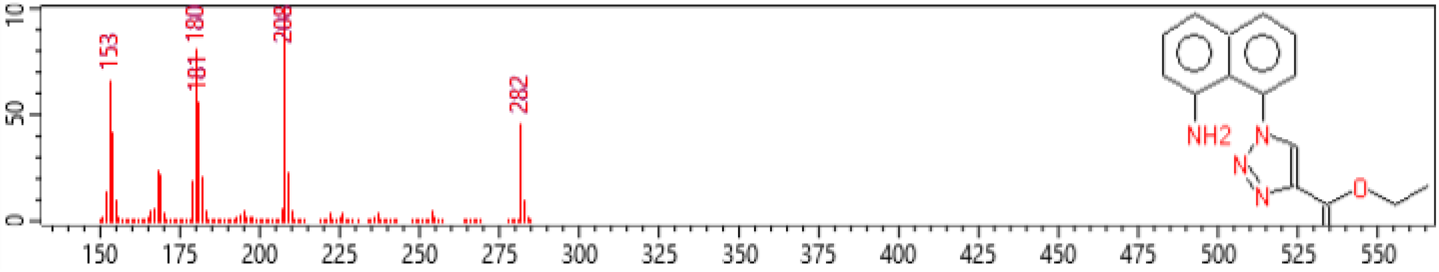

7-Nitro-2,3-dihydro-1,4-benzodioxine-6-carboxylic acid, TMS derivative

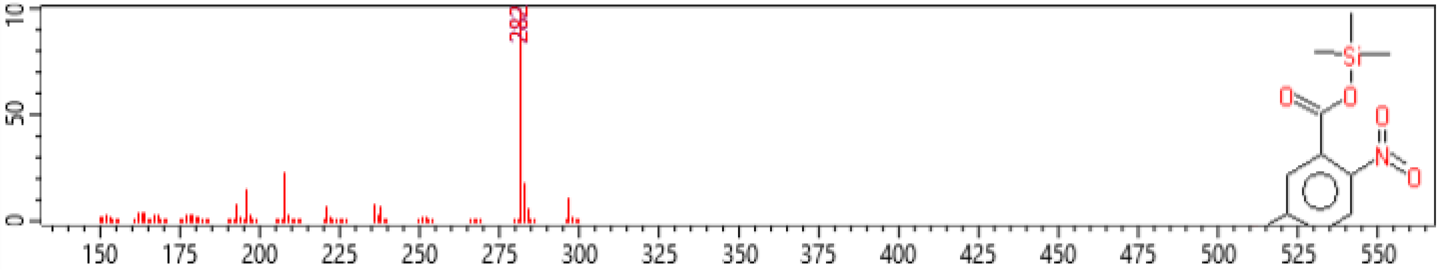

4-Hydroxybenzoic acid, 2TMS derivative

Peak: 12.17 - 12.22 min (scan #1444 - #1457) Subtracted noise: 12.09 - 12.14 min (scan #1421 - #1434), 12.24 - 12.29 min (scan #14

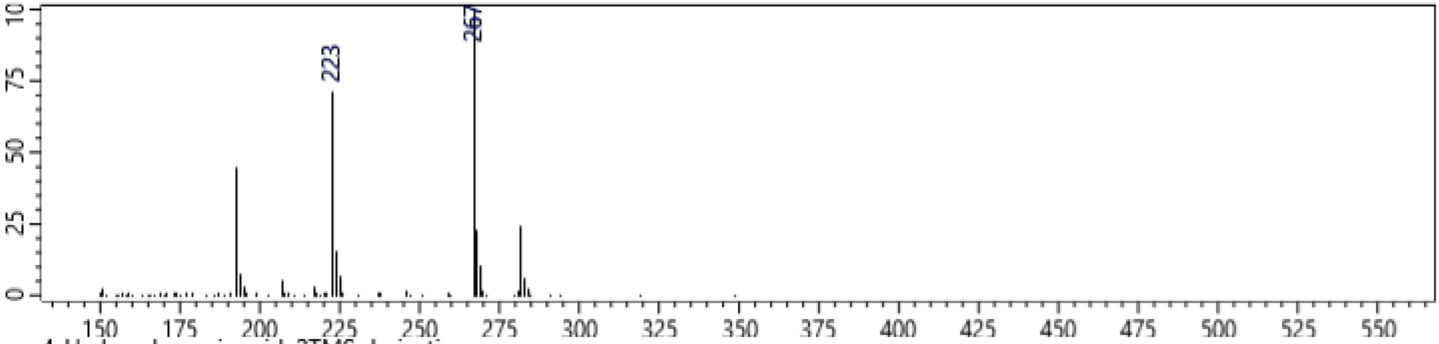

4-Hydroxybenzoic acid, 2TMS derivative

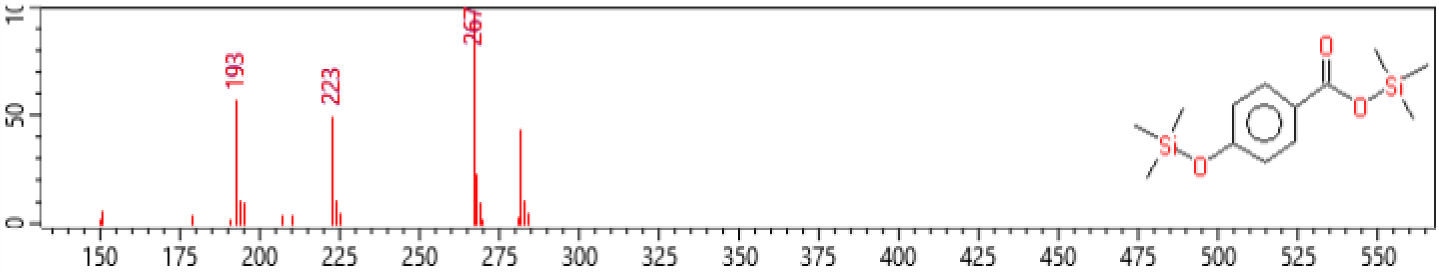

4-Hydroxybenzoic acid, 2TMS derivative

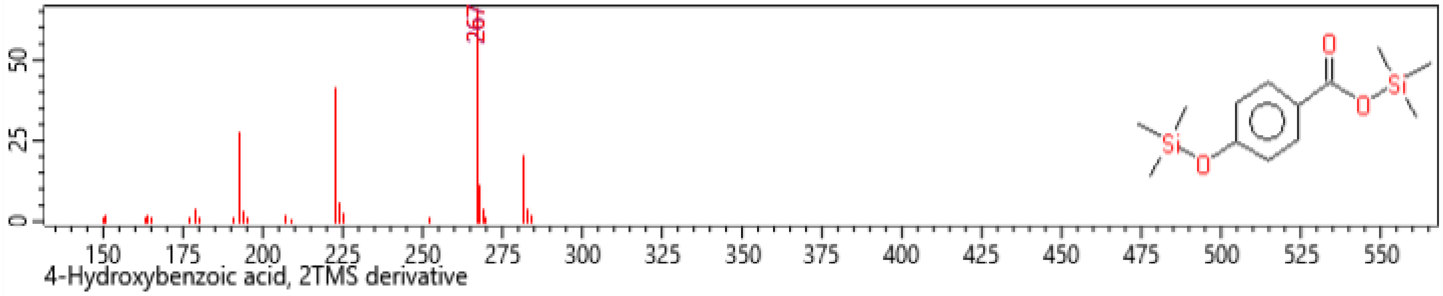

4-Hydroxybenzoic acid, 2TMS derivative

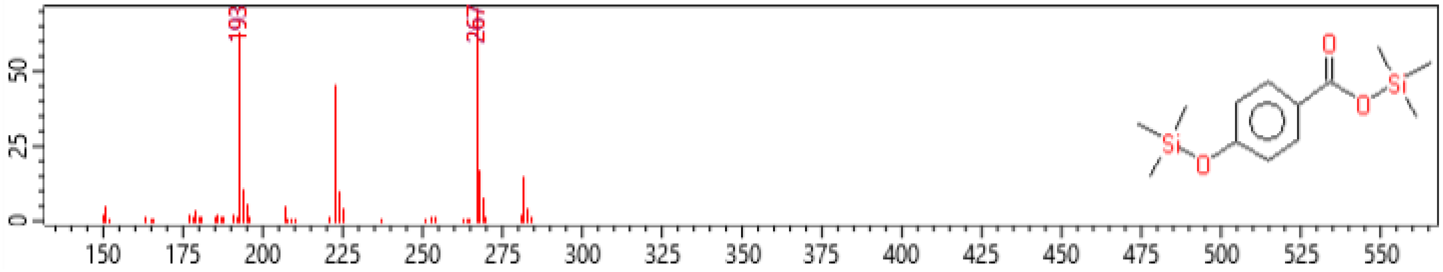

3-Hydroxybenzoic acid, 2TMS derivative

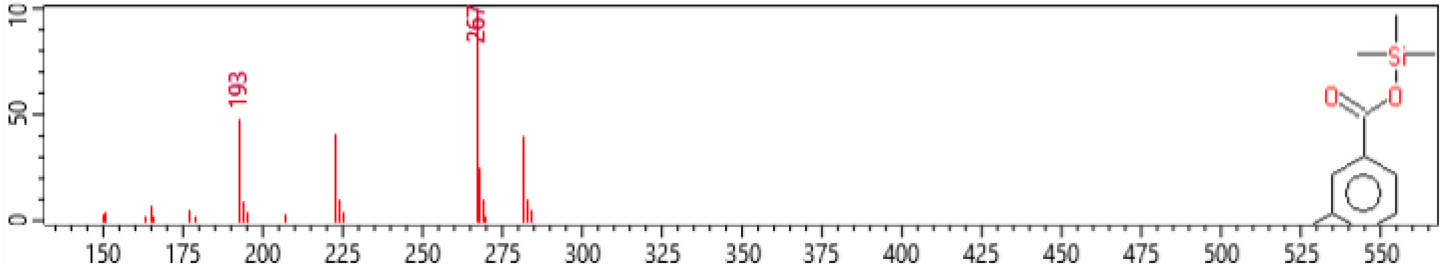

3-Hydroxybenzoic acid, 2TMS derivative

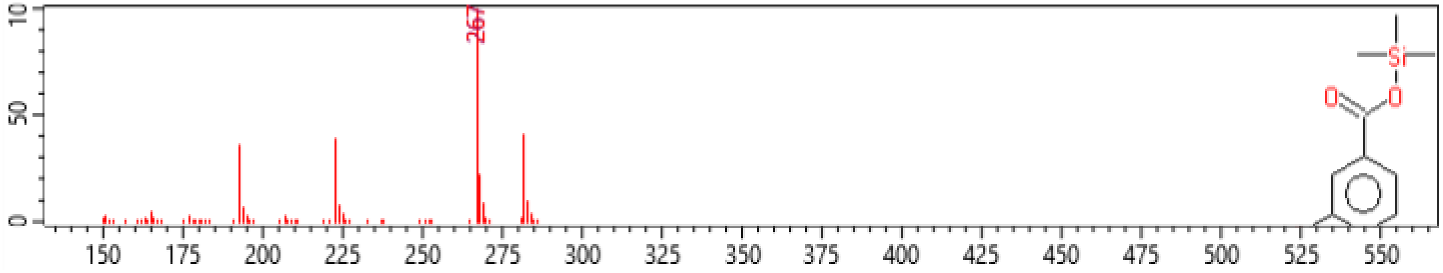

4-Hydroxybenzeneacetic acid, 2TMS derivative

Peak: 12.26 - 12.30 min (scan #1469 - #1479) Subtracted noise: 12.21 - 12.24 min (scan #1453 - #1462), 12.32 - 12.35 min (scan #14

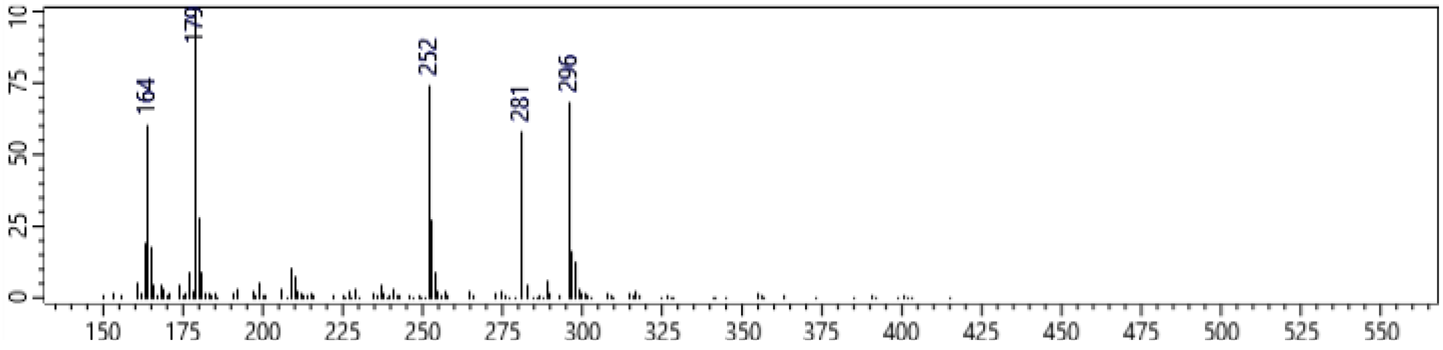

4-Hydroxybenzeneacetic acid, 2TMS derivative

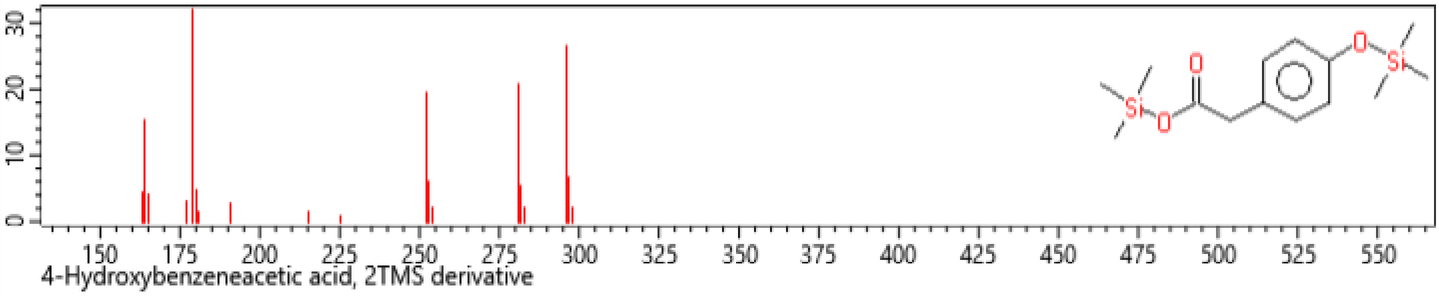

4-Hydroxybenzeneacetic acid, 2TMS derivative

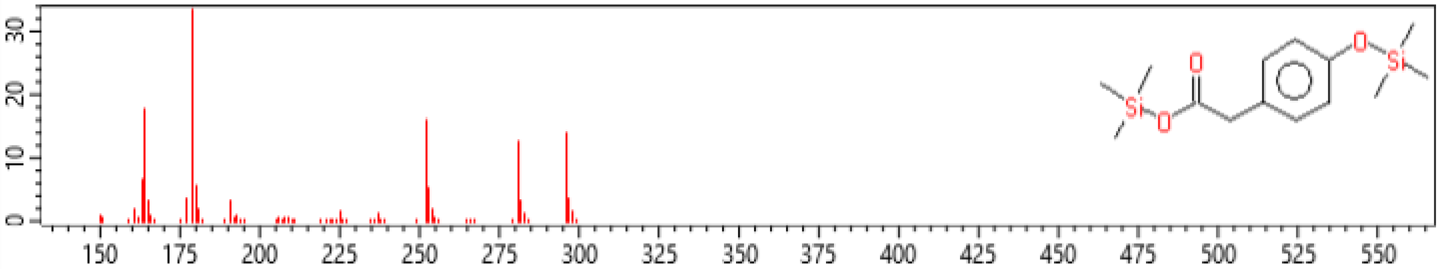

4-Hydroxybenzeneacetic acid, 2TMS derivative

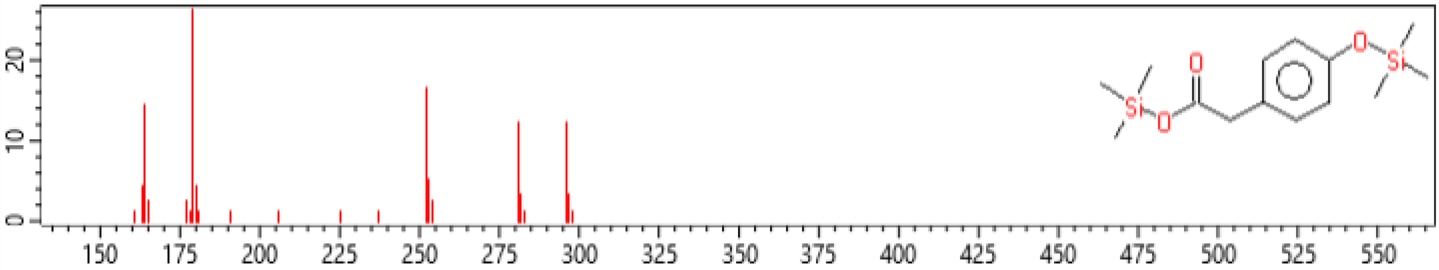

4-Hydroxybenzeneacetic acid, 2TMS derivative

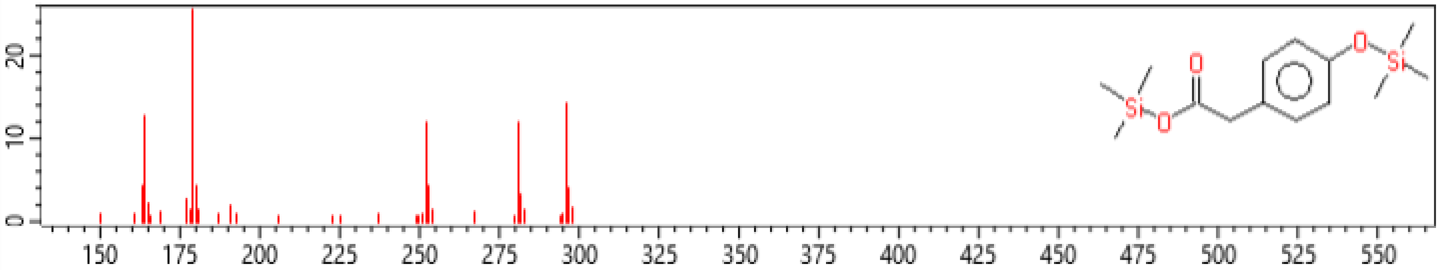

3-Hydroxyphenylacetic acid, 2TMS derivative

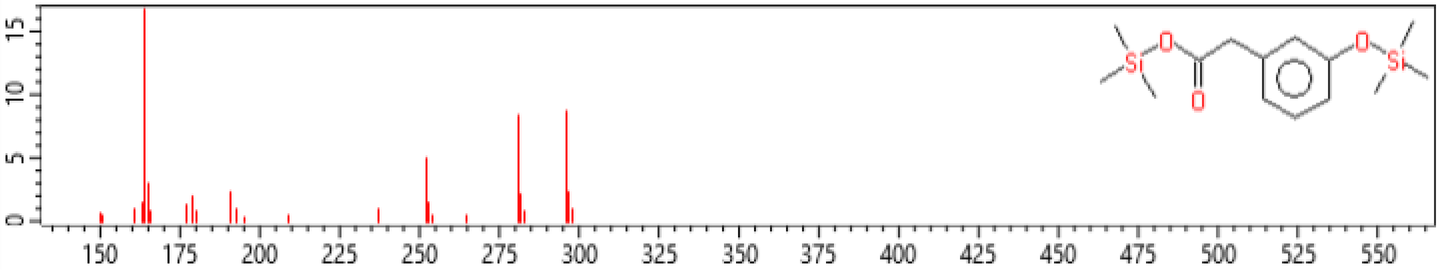

5-Amino-3-(methylsulfanyl)-1H-pyrazole-4-carbonitrile, 3TMS derivative

Peak: 12.38 - 12.43 min (scan #1500 - #1514) Subtracted noise: 12.26 - 12.32 min (scan #1469 - #1484), 12.43 - 12.48 min (scan #15

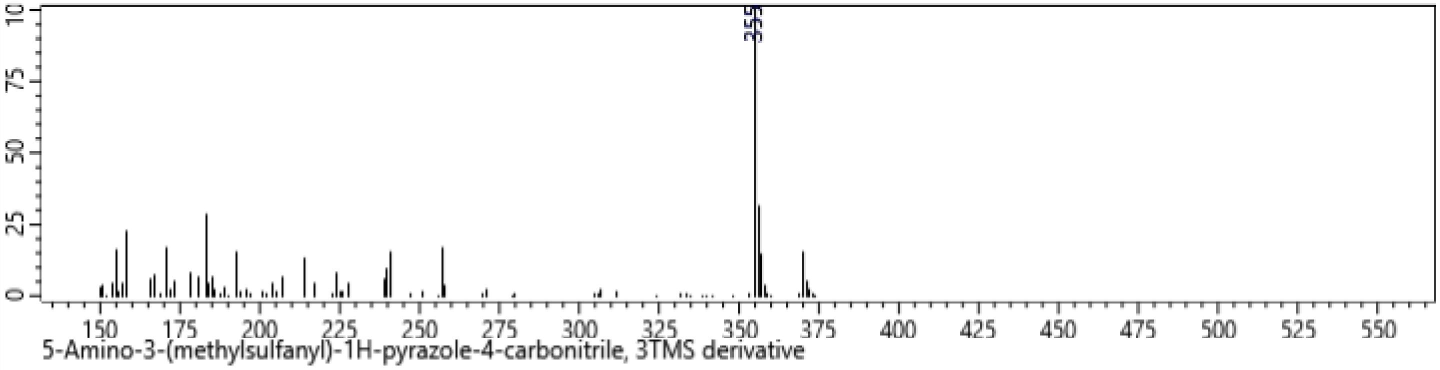

5-Amino-3-(methylsulfanyl)-1H-pyrazole-4-carbonitrile, 3TMS derivative

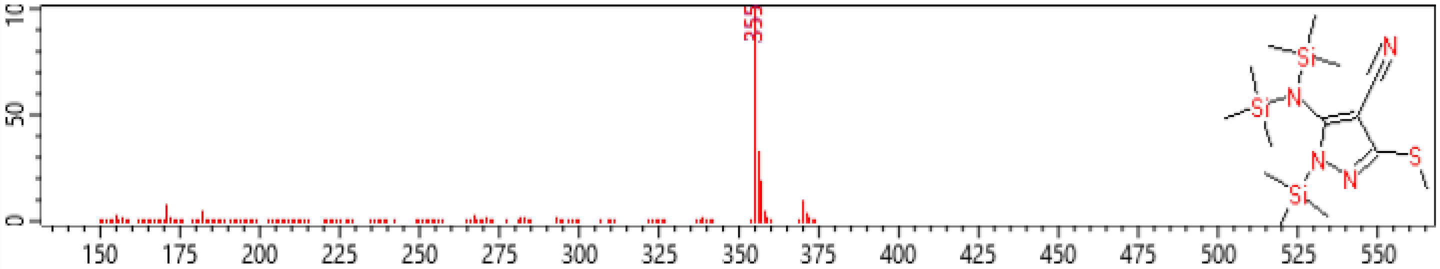

Nonadecanoic acid, TMS derivative

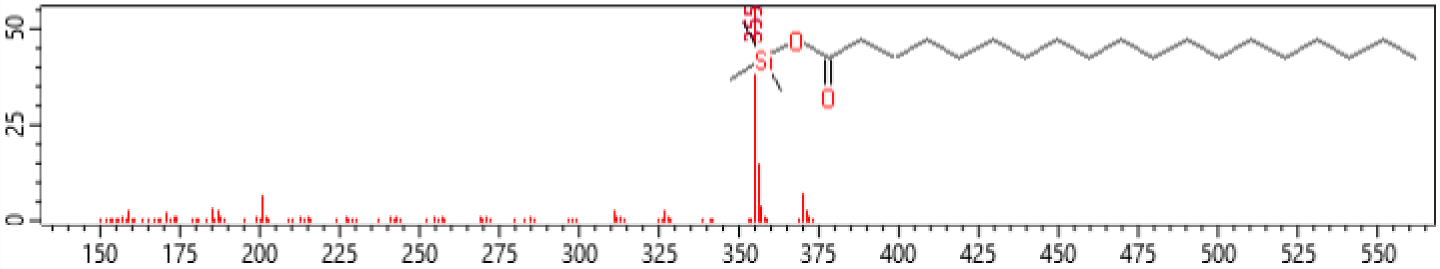

2-Methyloctadecanoic acid, TMS

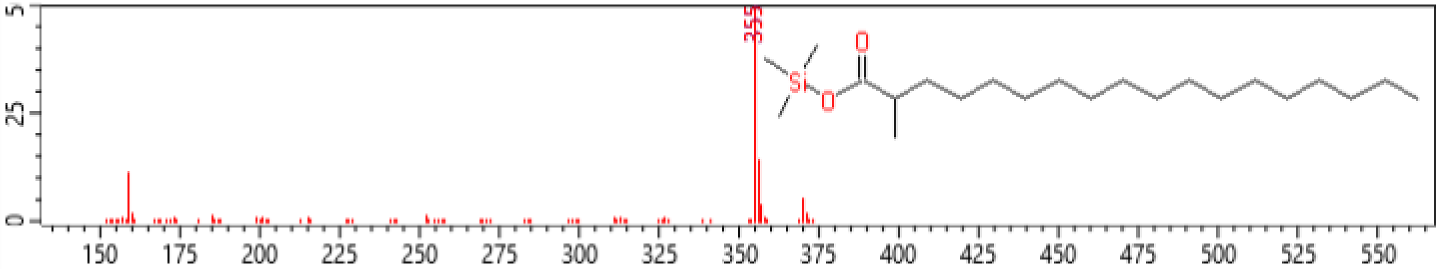

Silane, dimethyl(4-(2-phenylprop-2-yl)phenoxy)hexyloxy-

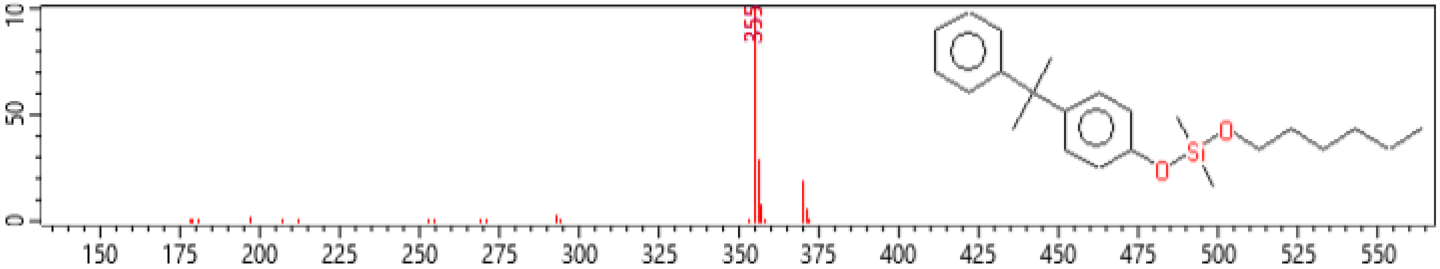

Silane, dimethyl(2-chlorophenoxy)dodecyloxy-

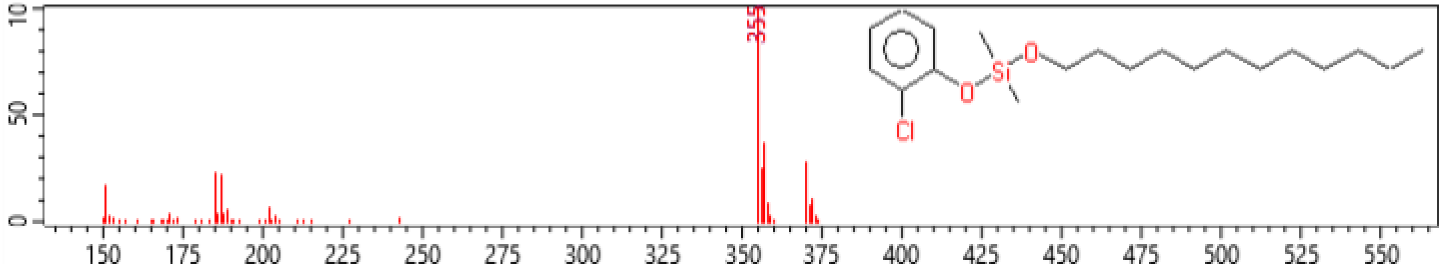

Triphenylene, 1,2,3,4,5,6,7,8,9,10,11,12-dodecahydro-

Peak: 12.50 - 12.56 min (scan #1536 - #1552) Subtracted noise: 12.37 - 12.43 min (scan #1498 - #1514), 12.56 - 12.62 min (scan #15

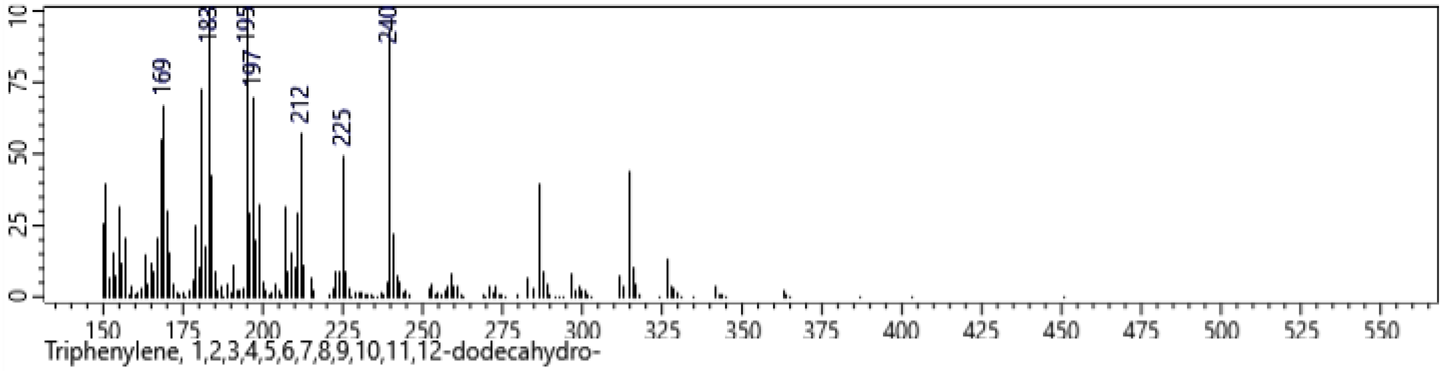

Triphenylene, 1,2,3,4,5,6,7,8,9,10,11,12-dodecahydro-

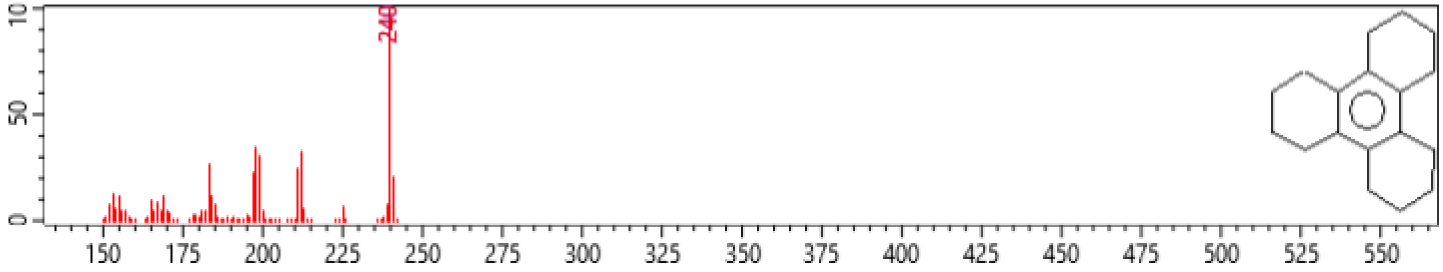

Triphenylene, 1,2,3,4,5,6,7,8,9,10,11,12-dodecahydro-

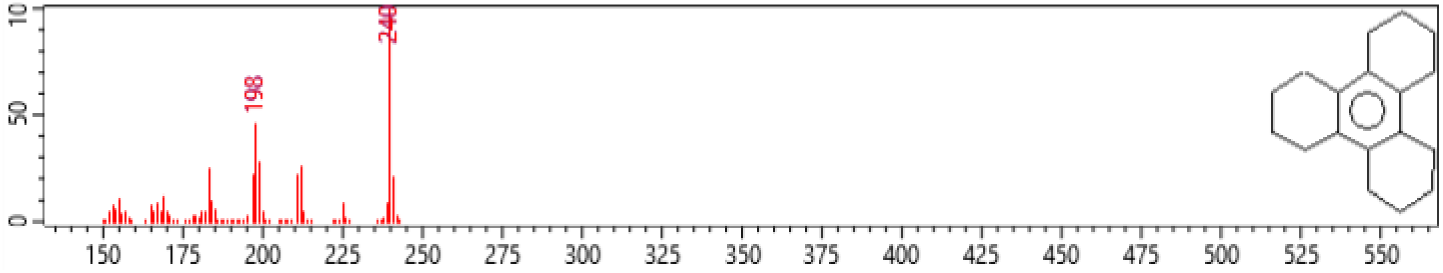

Cyclohexane, hexaethylidene- (isomer 1)

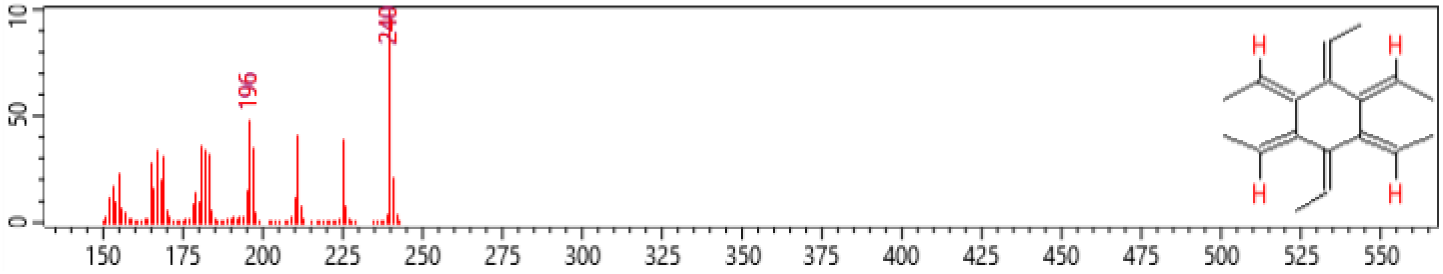

Cyclohexane, hexaethylidene-

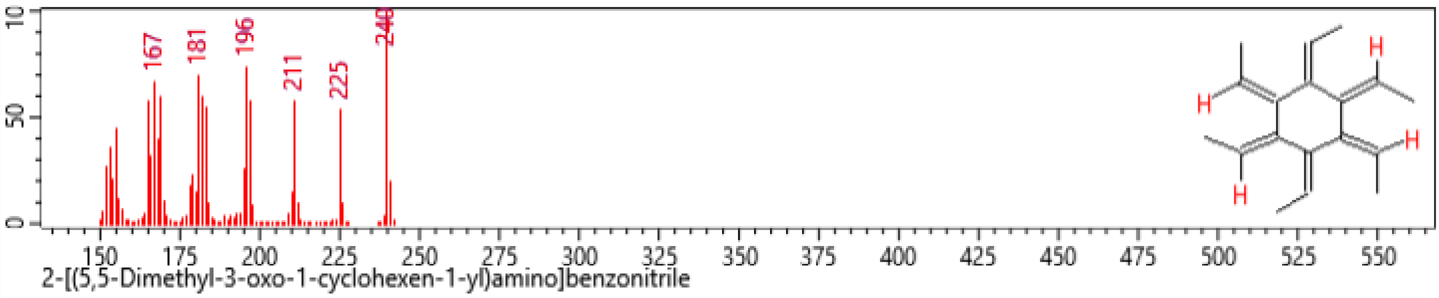

2-[(5,5-Dimethyl-3-oxo-1-cyclohexen-1-yl)amino]benzonitrile

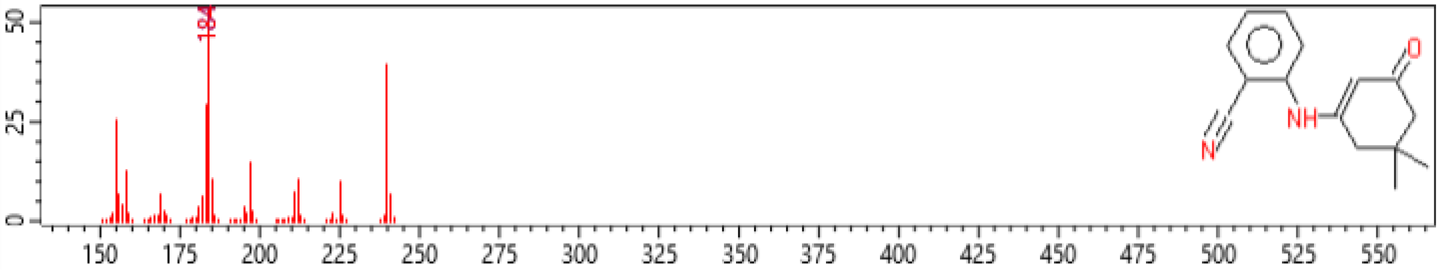

18-Chloro-17-hydroxy-5,15-dimethoxy-11-methyl-12-oxabicyclo[12.4.0]octadeca-1(18),14,16-triene-3,8,13-trione

Peak: 12.56 - 12.57 min (scan #1552 - #1554) Subtracted noise: 12.54 - 12.55 min (scan #1547 - #1550), 12.59 - 12.59 min (scan #15

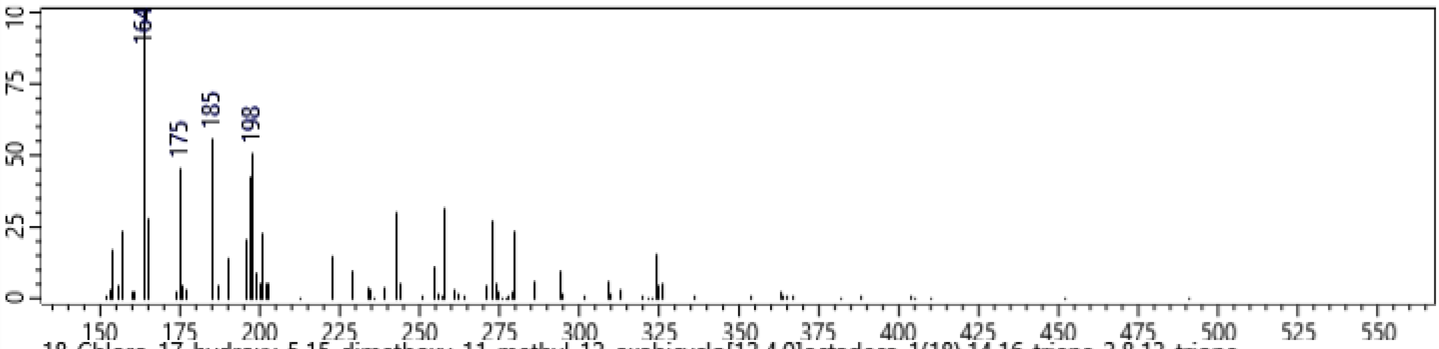

18-Chloro-17-hydroxy-5,15-dimethoxy-11-methyl-12-oxabicyclo[12.4.0]octadeca-1(18),14,16-triene-3,8,13-trione

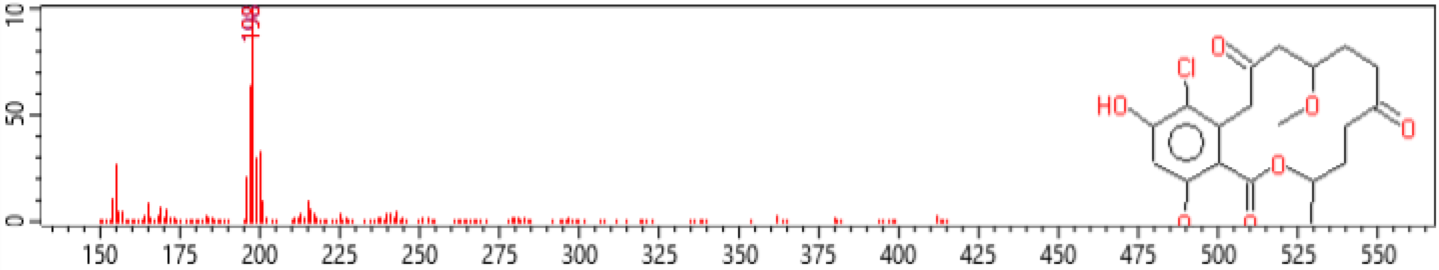

2',6'-Dihydroxy-4-methoxychalcone-4'-O-neohesperidoside

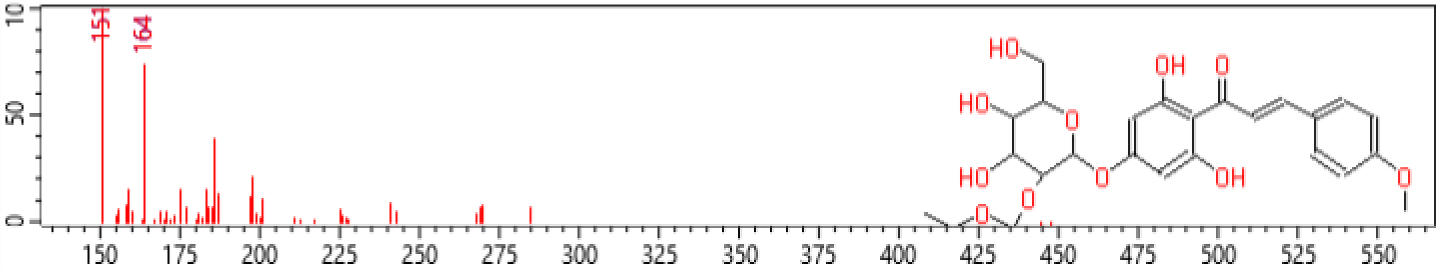

Pyrrolizidine-3-one, 5-[2-methyl-2-ethylenedithioethyl]-

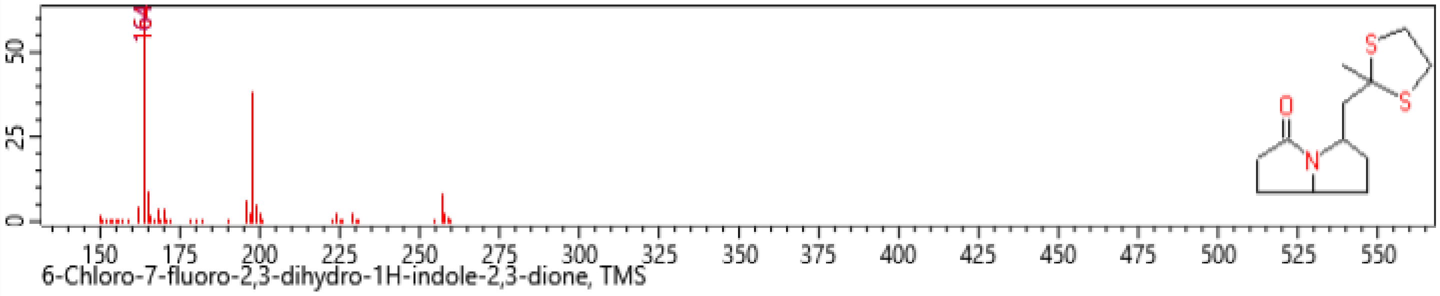

6-Chloro-7-fluoro-2,3-dihydro-1H-indole-2,3-dione, TMS

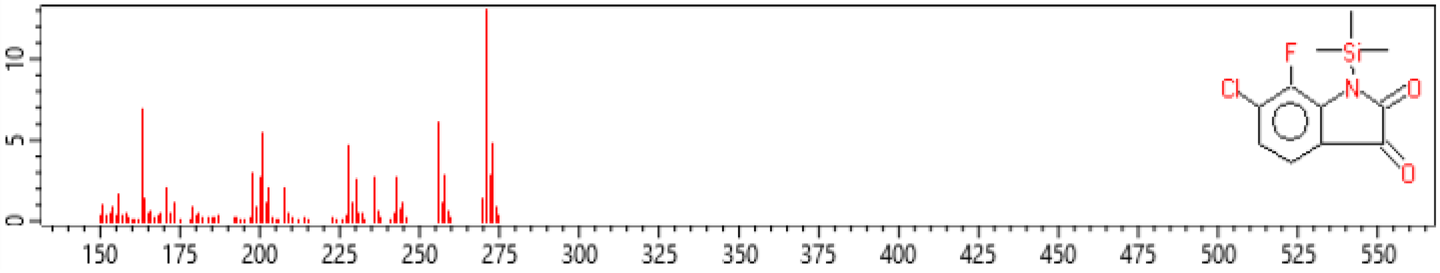

4H-Cyclopenta[5,6]naphtho[1,2-b]thiophene, 3b,5,7,8,8a,8b,9,10-octahydro-6,10-dimethyl-

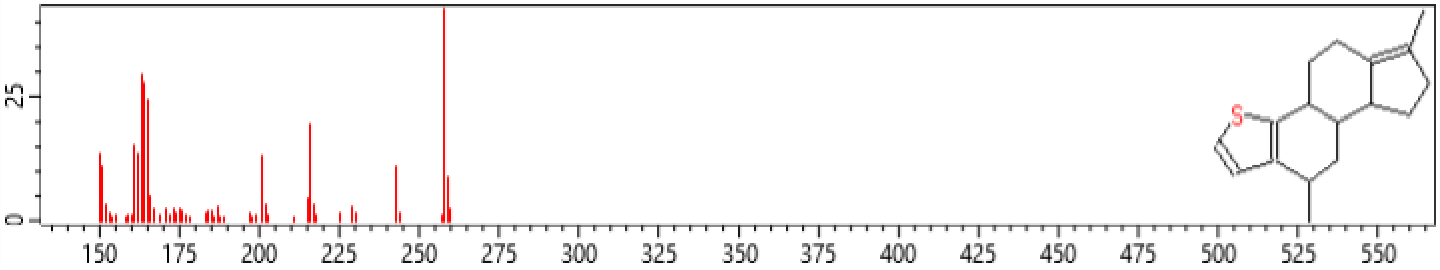

(3-Nitrophenyl)methanamine, N, N, di-TMS

Peak: 12.62 - 12.64 min (scan #1567 - #1575) Subtracted noise: 12.55 - 12.58 min (scan #1549 - #1558), 12.65 - 12.68 min (scan #15

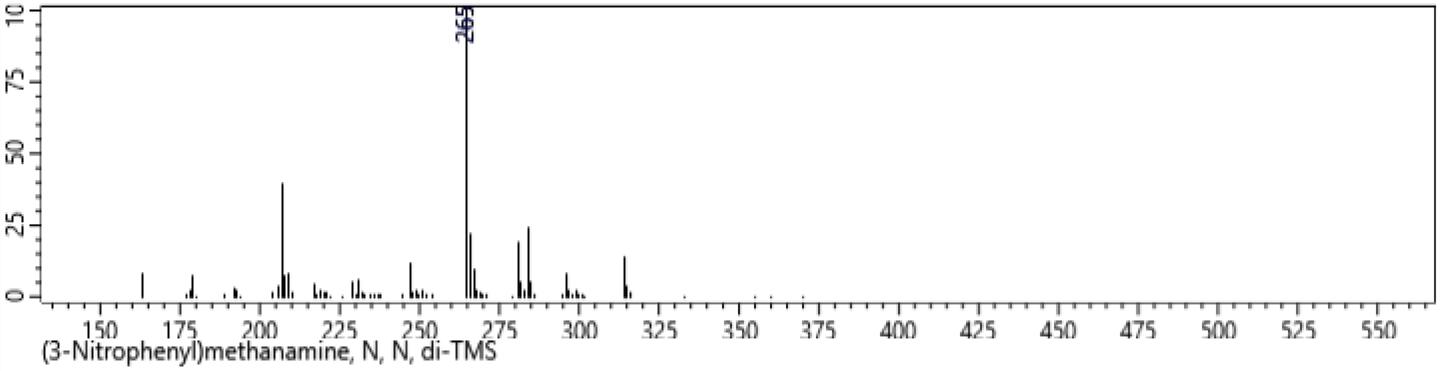

(3-Nitrophenyl)methanamine, N, N, di-TMS

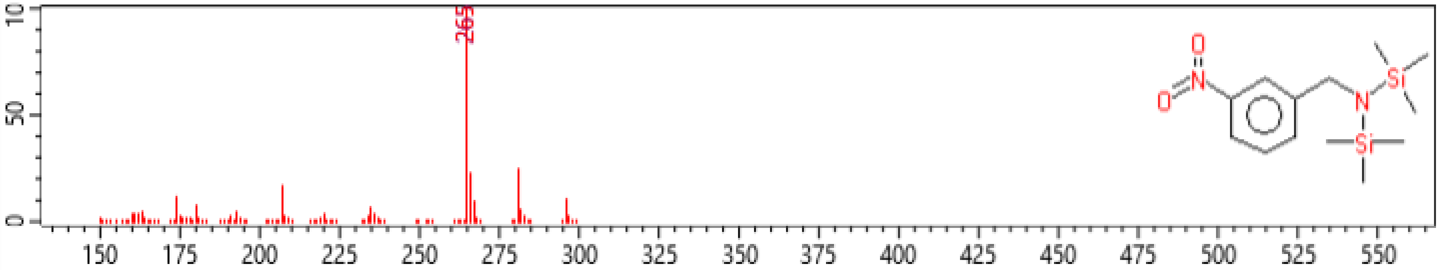

2,2,3,5,6,6,7-Heptamethyl[1,4,2,3,5,6,7] dioxapentasilepane

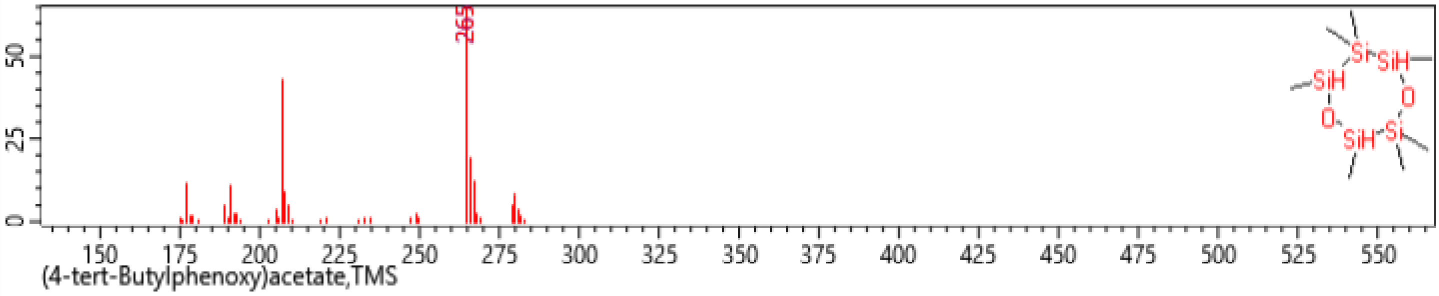

(4-tert-Butylphenoxy)acetate,TMS

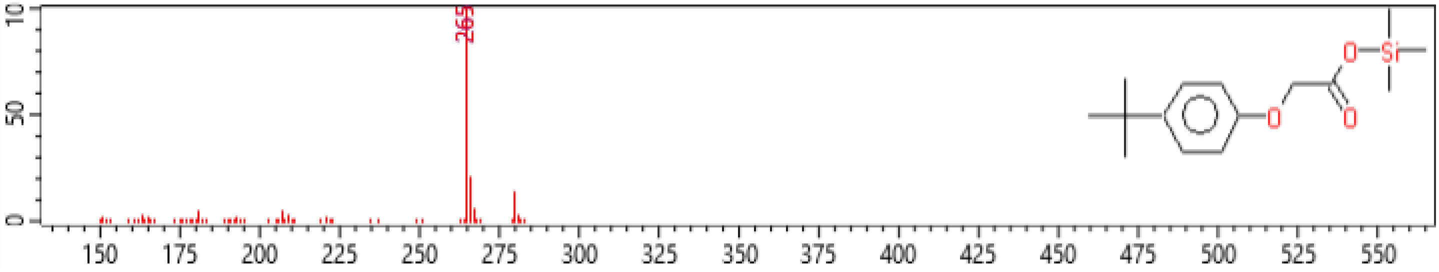

benzoic acid, 4-[[(trimethylsilyl)oxy]methyl]-, trimethylsilyl ester

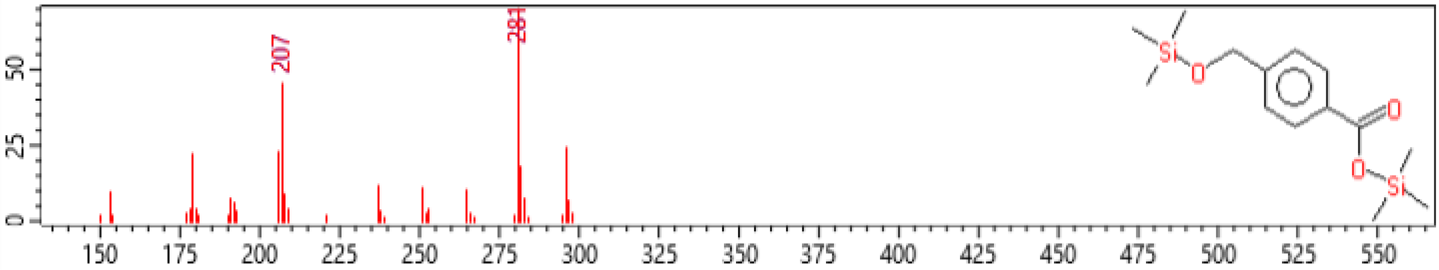

(4-tert-Butylphenoxy)acetate,TMS

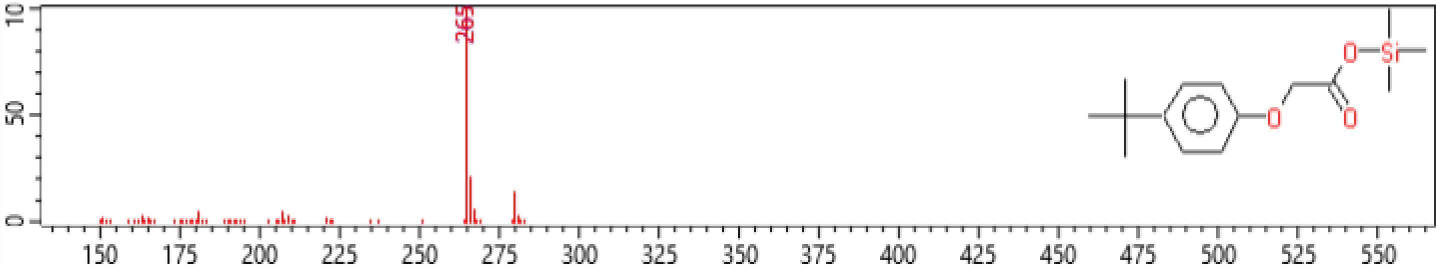

Brefeldin-C, TMS

Peak: 12.66 - 12.69 min (scan #1580 - #1587) Subtracted noise: 12.63 - 12.65 min (scan #1570 - #1576), 12.71 - 12.73 min (scan #15

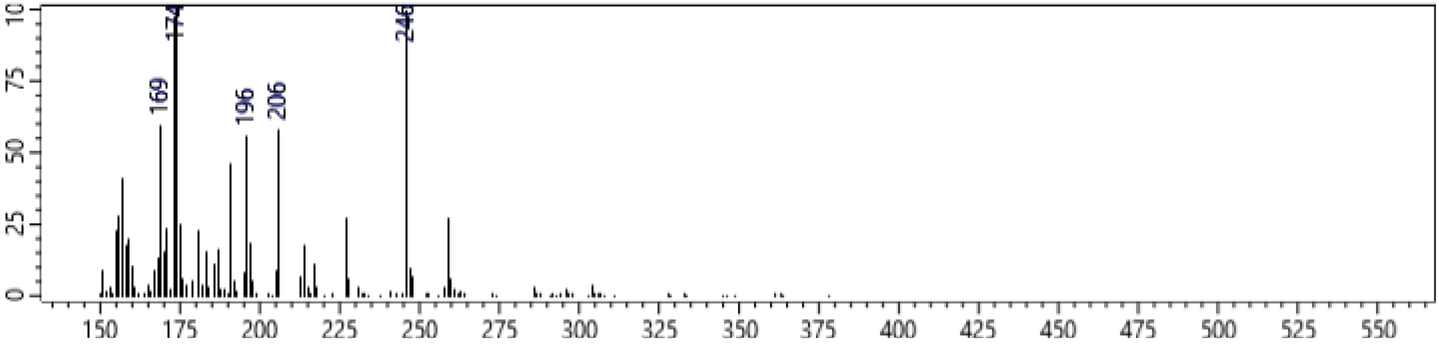

Brefeldin-C, TMS

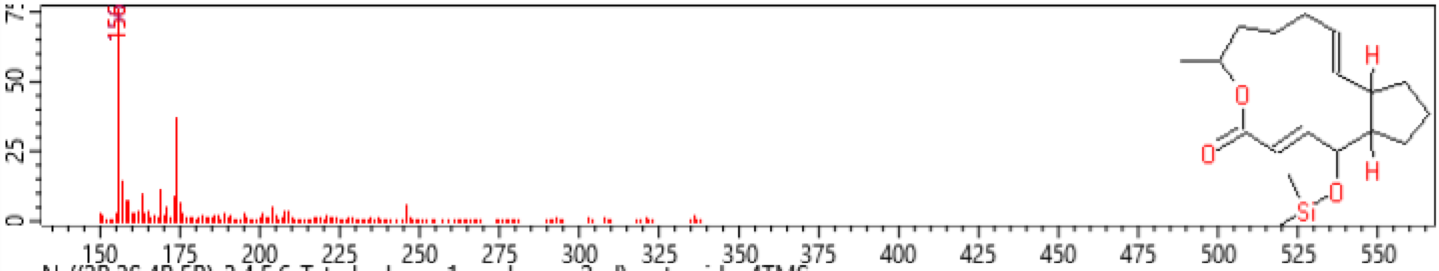

N-((2R,3S,4R,5R)-3,4,5,6-Tetrahydroxy-1-oxohexan-2-yl)acetamide, 4TMS

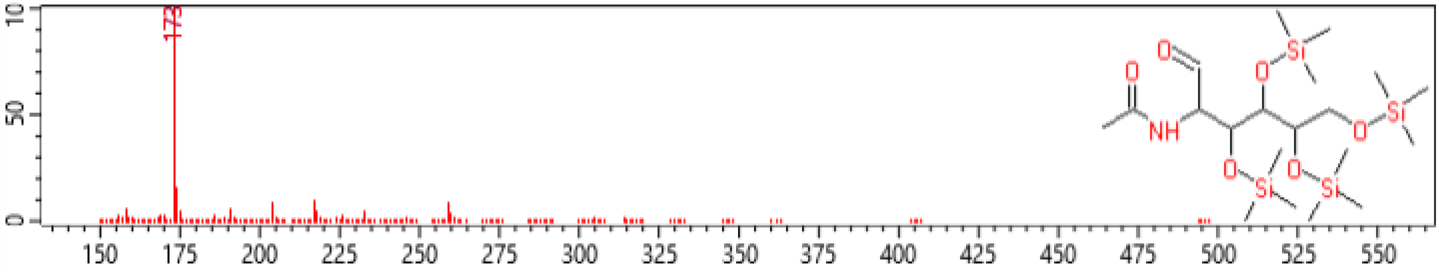

N-((2R,3S,4R,5R)-3,4,5,6-Tetrahydroxy-1-oxohexan-2-yl)acetamide, 4TMS

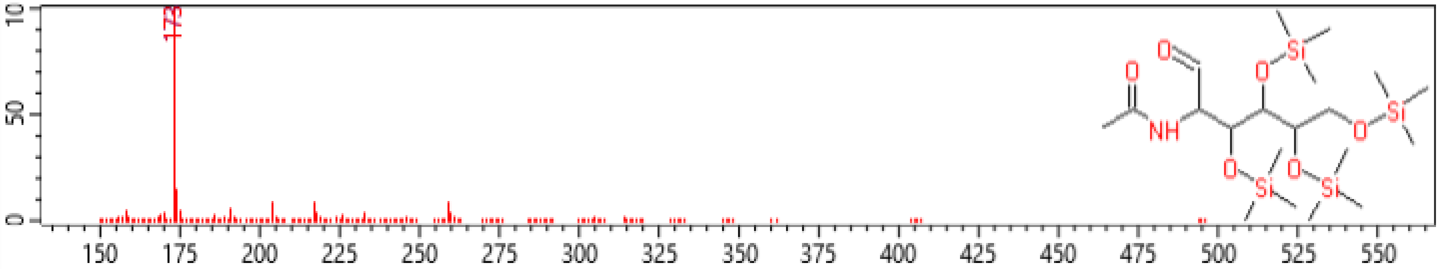

N-Acetyl-D-galactosamine, (isomer 2), 4TMS derivative

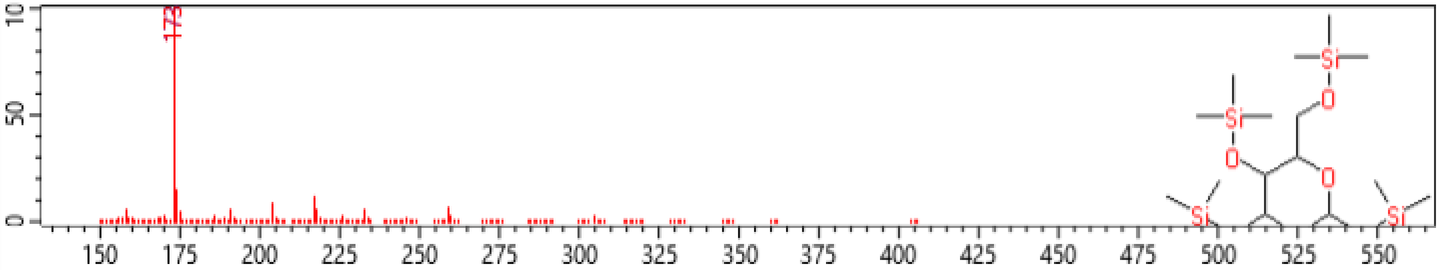

N-Acetyl-D-galactosamine, (isomer 1), 4TMS derivative

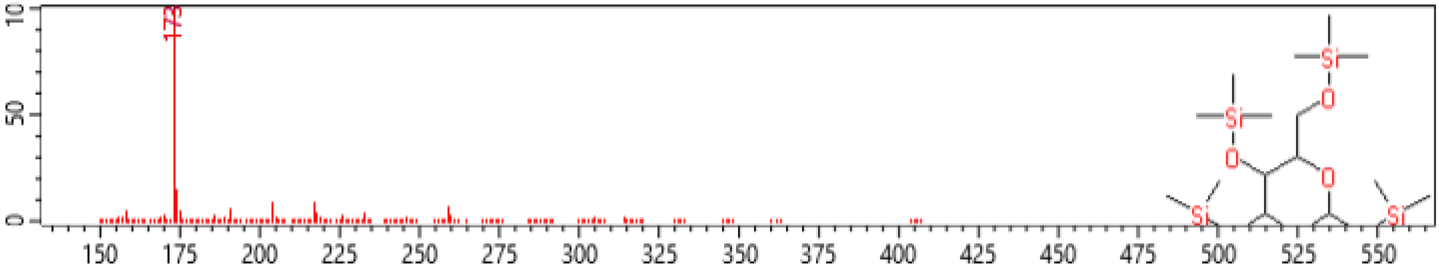

2,2-Dimethyl-1-oxa-2-silacyclotridecanone-13

Peak: 12.72 - 12.73 min (scan #1596 - #1598) Subtracted noise: 12.70 - 12.71 min (scan #1590 - #1593), 12.74 - 12.74 min (scan #16

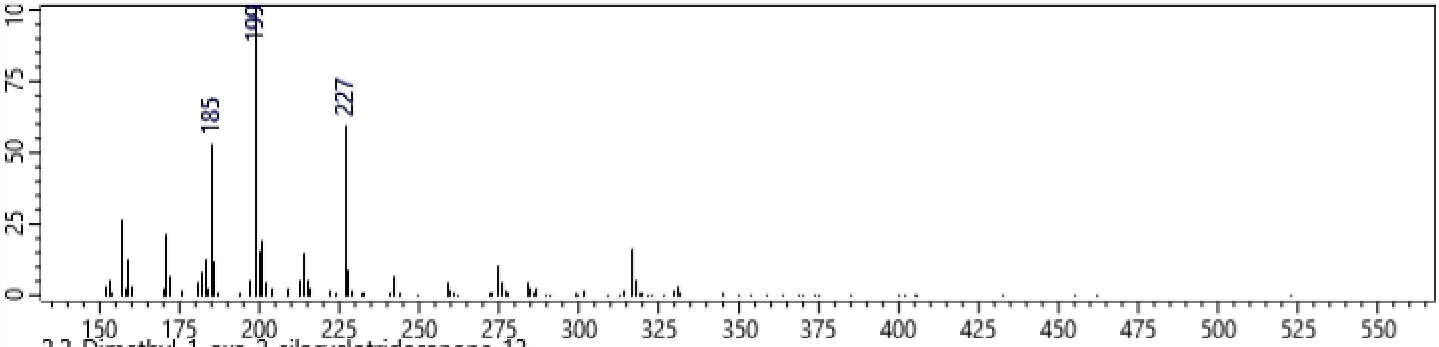

2,2-Dimethyl-1-oxa-2-silacyclotridecanone-13

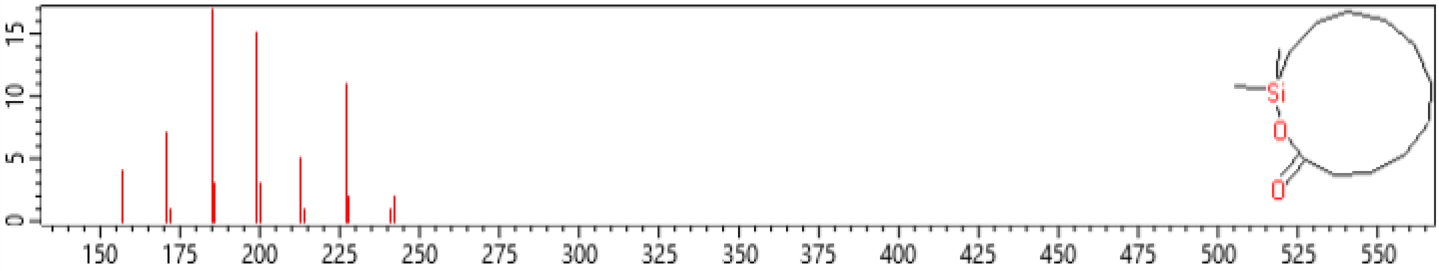

3-Methyl-1-tripropylsilyloxybut-2-ene

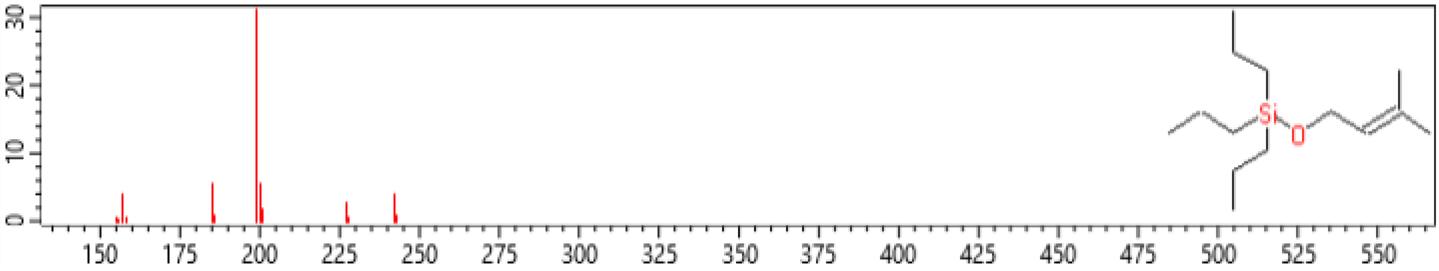

Succinic acid, 1-(3-bromophenyl)ethyl nonyl ester

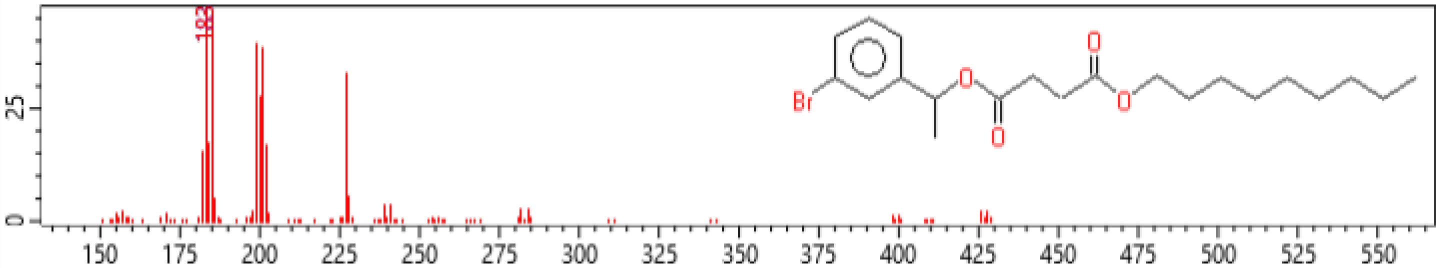

Silane, (iodomethyl)dimethylpropyl-

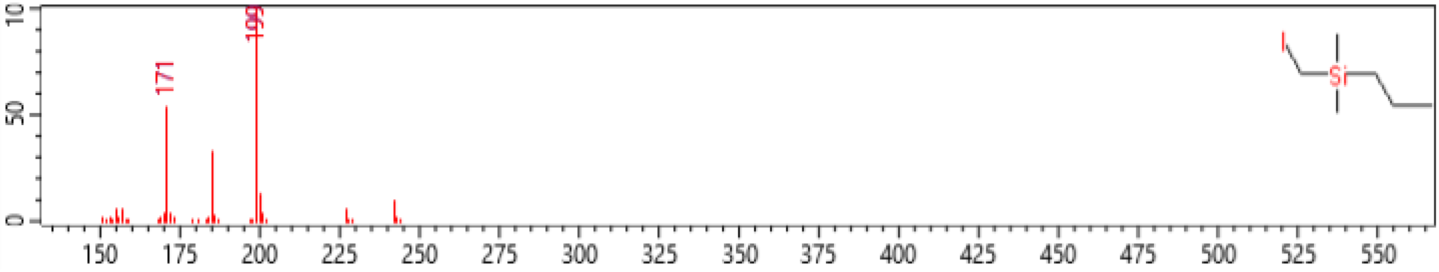

Podocarpa-8,11,13-triene-7β,13-diol, 14-isopropyl-

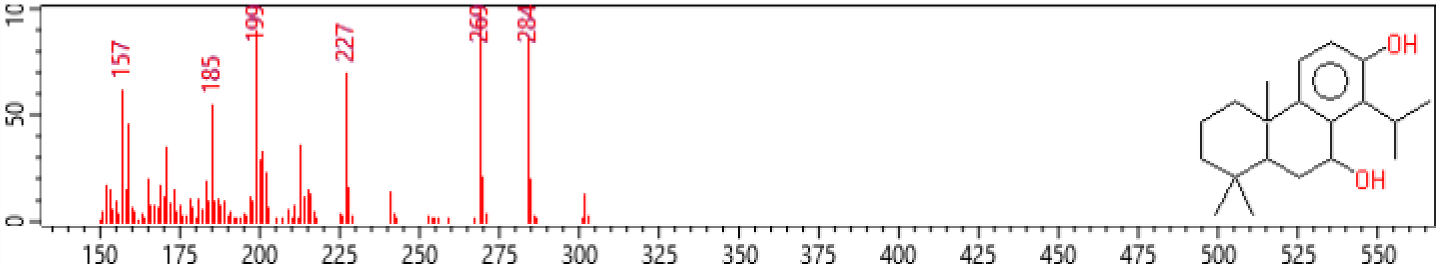

Triphenylene, 1,2,3,4,5,6,7,8,9,10,11,12-dodecahydro-

Peak: 12.78 - 12.84 min (scan #1614 - #1630) Subtracted noise: 12.68 - 12.73 min (scan #1584 - #1600), 12.86 - 12.92 min (scan #16

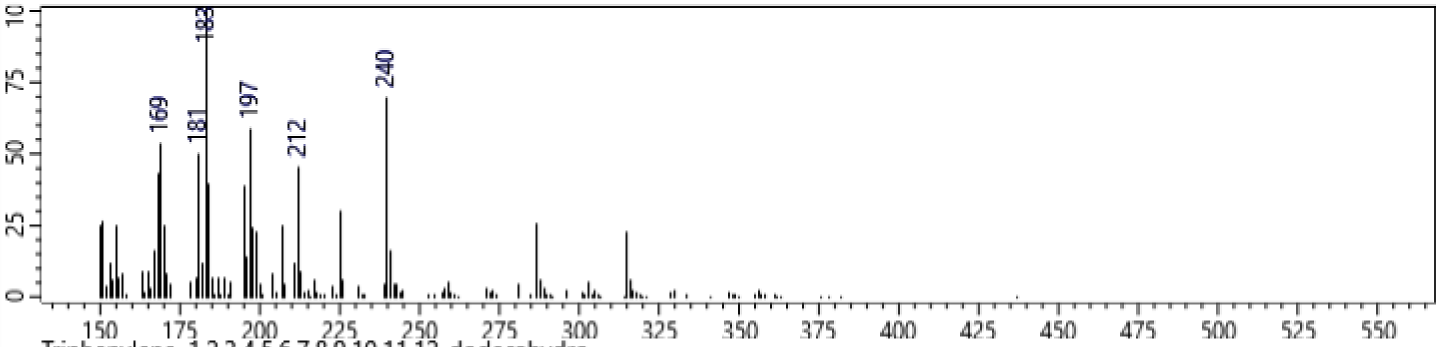

Triphenylene, 1,2,3,4,5,6,7,8,9,10,11,12-dodecahydro-

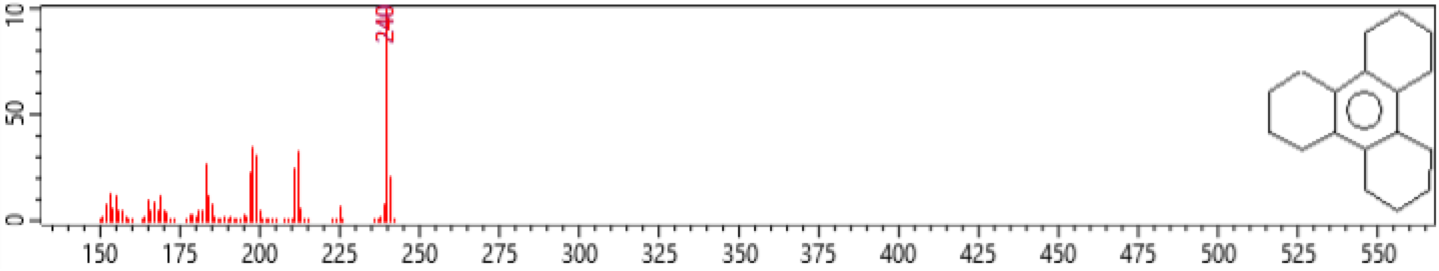

Triphenylene, 1,2,3,4,5,6,7,8,9,10,11,12-dodecahydro-

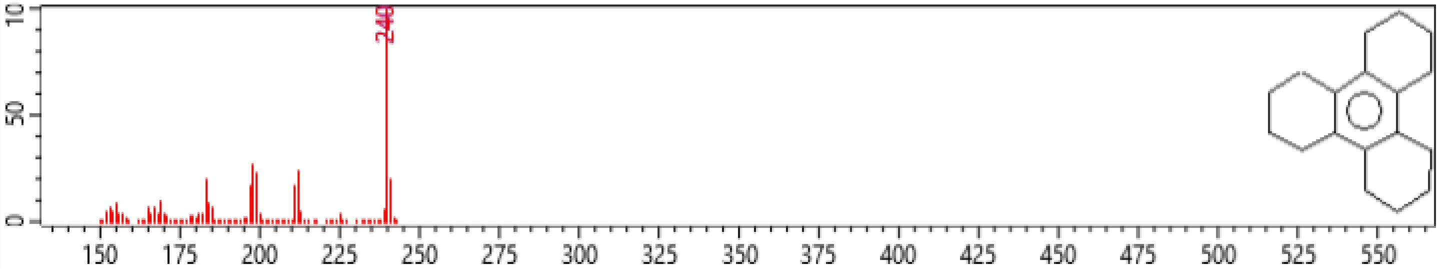

Triphenylene, 1,2,3,4,5,6,7,8,9,10,11,12-dodecahydro-

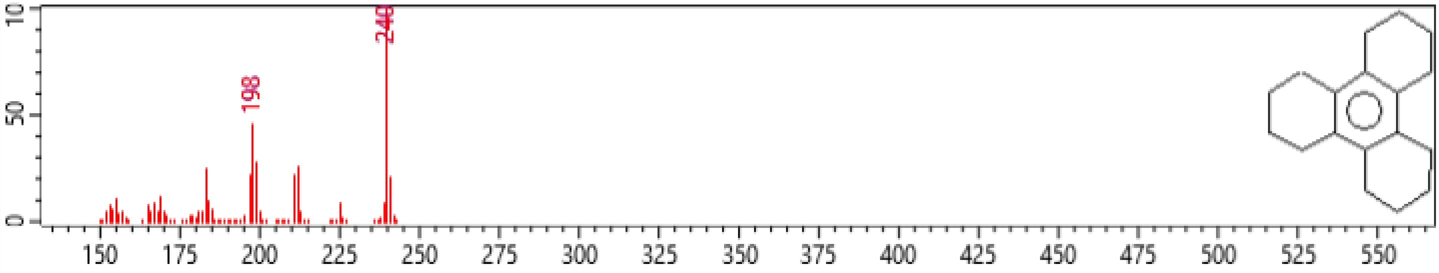

2-[(5,5-Dimethyl-3-oxo-1-cyclohexen-1-yl)amino]benzonitrile

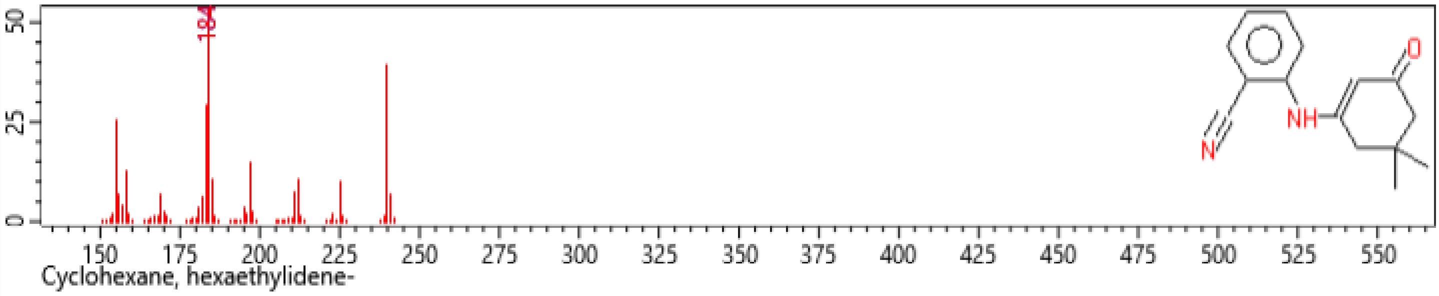

Cyclohexane, hexaethylidene-

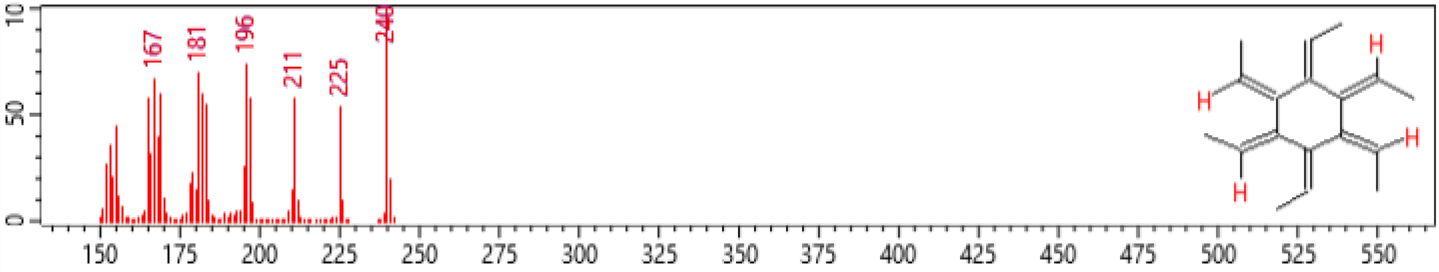

Homovanillyl alcohol, 2TMS derivative

Peak: 12.89 - 12.95 min (scan #1645 - #1659) Subtracted noise: 12.81 - 12.85 min (scan #1620 - #1633), 12.96 - 13.01 min (scan #16

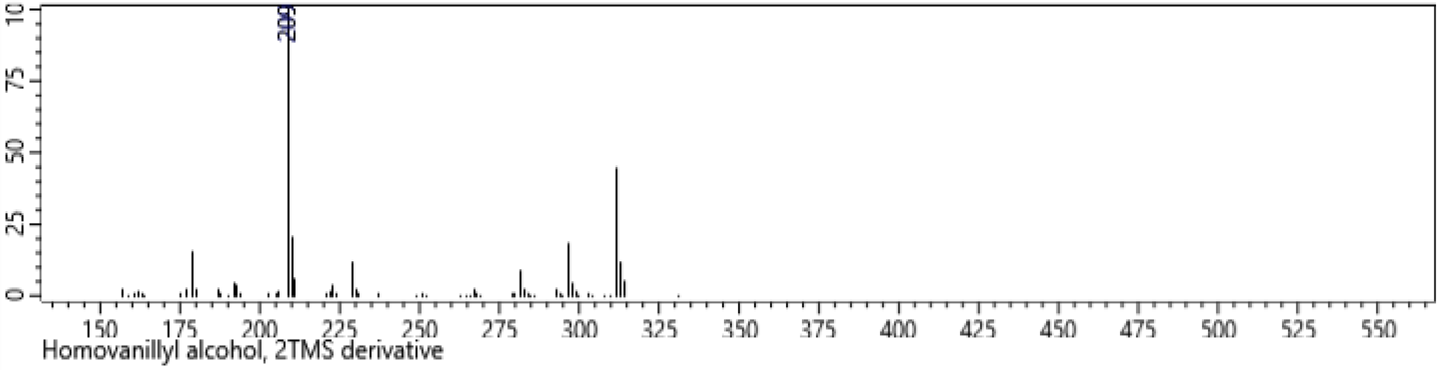

Homovanillyl alcohol, 2TMS derivative

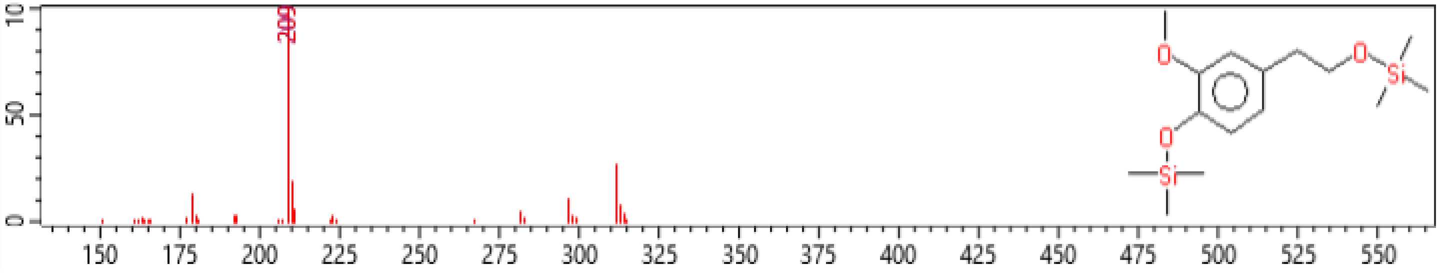

Homovanillyl alcohol, 2TMS derivative

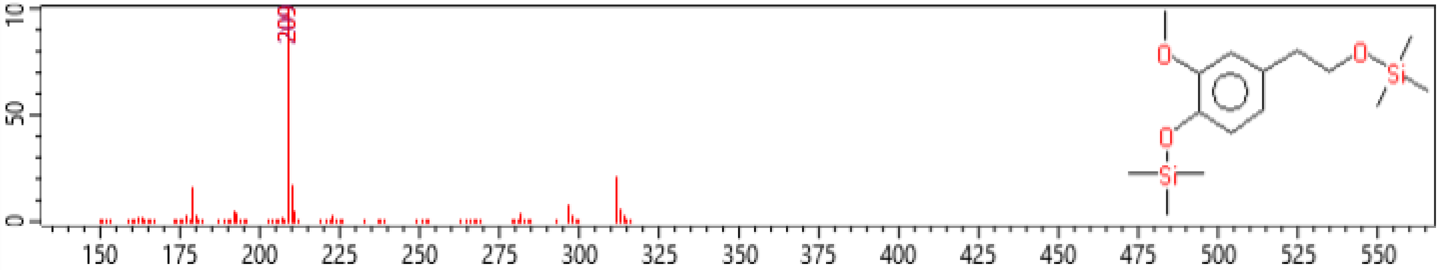

Homovanillyl alcohol, 2TMS derivative

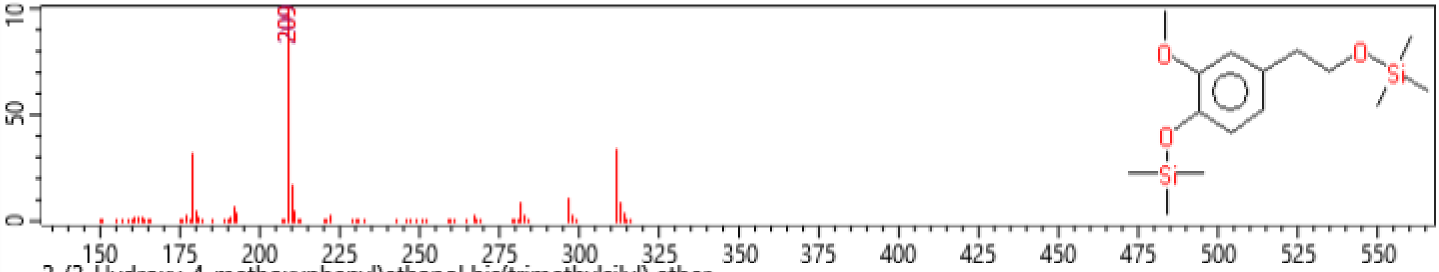

2-(3-Hydroxy-4-methoxyphenyl)ethanol bis(trimethylsilyl) ether

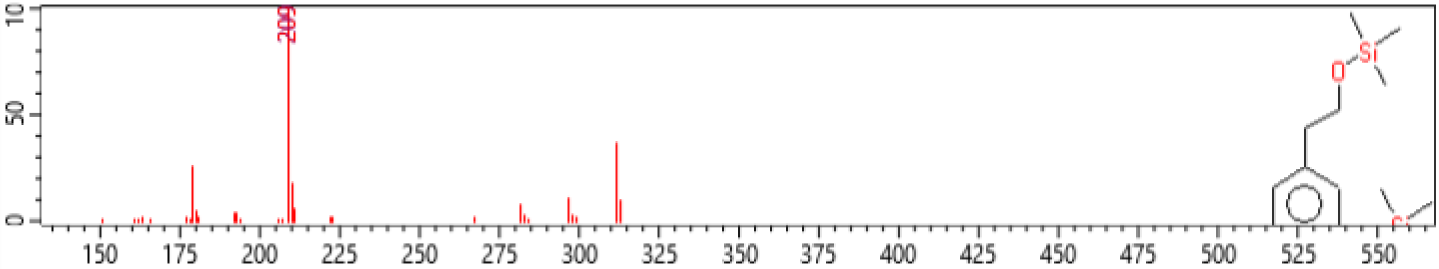

7,8-Dimethoxy-3-(4-methoxyphenyl)-4-oxo-4H-chromen-5-yl 2-O-pentopyranosylhexopyranoside

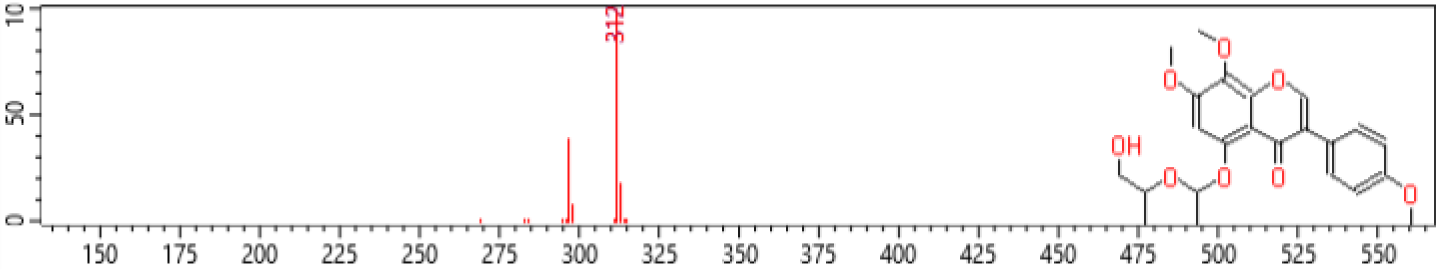

Octanoic acid, di(tert-butyl)silyl ester

Peak: 12.97 - 12.99 min (scan #1666 - #1672) Subtracted noise: 12.94 - 12.96 min (scan #1658 - #1663), 13.01 - 13.03 min (scan #16

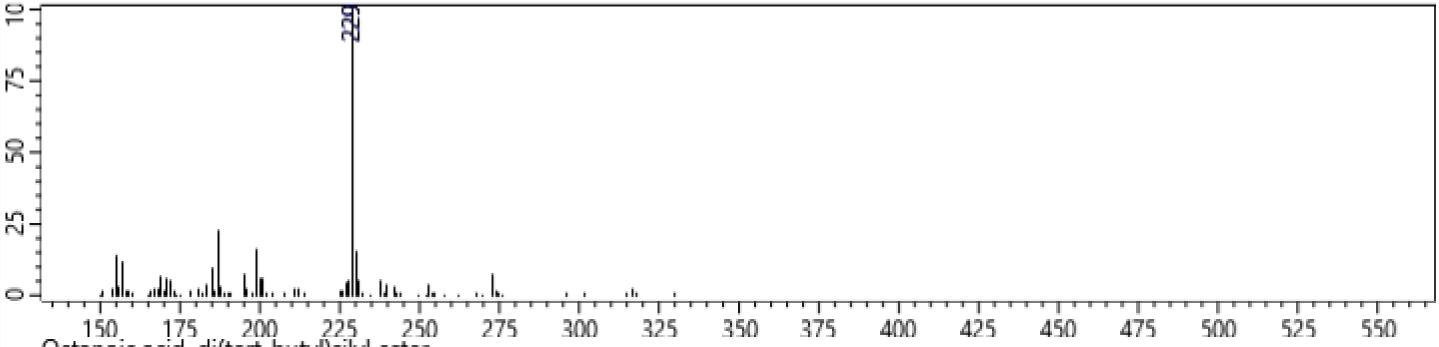

Octanoic acid, di(tert-butyl)silyl ester

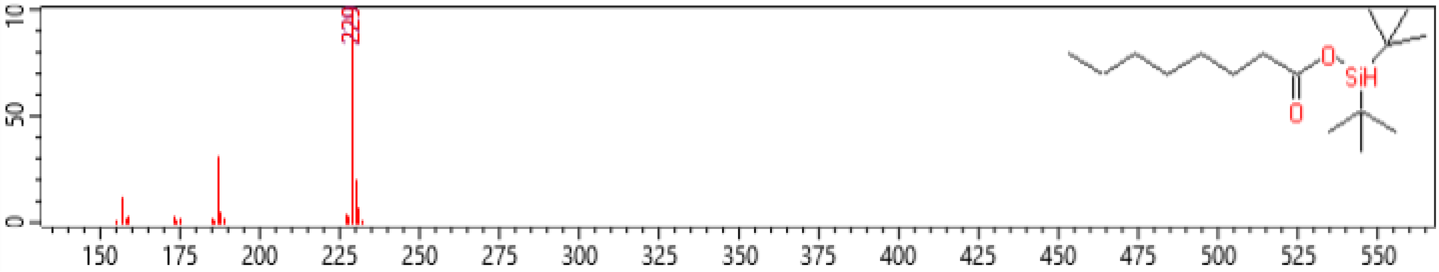

Naphthalene, 6,7-diethyl-1,2,3,4-tetrahydro-1,1,4,4-tetramethyl-

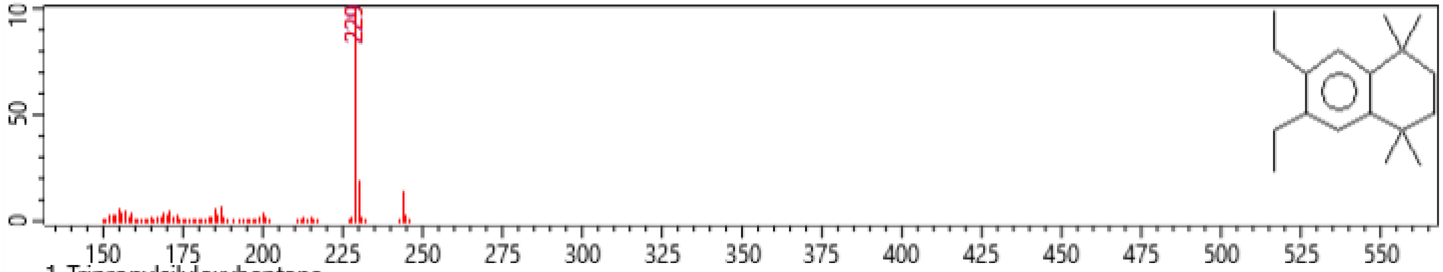

1-Tripropylsilyloxyheptane

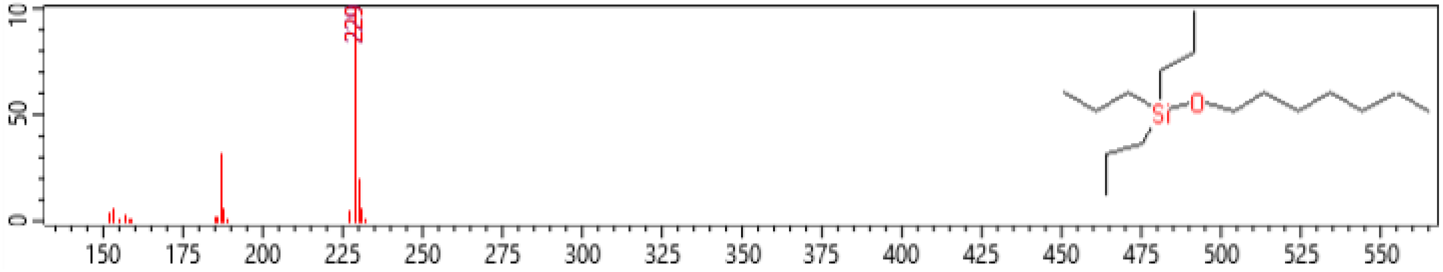

2-Propenoic acid, 2-cyano-3-[4-(diethylamino)phenyl]-

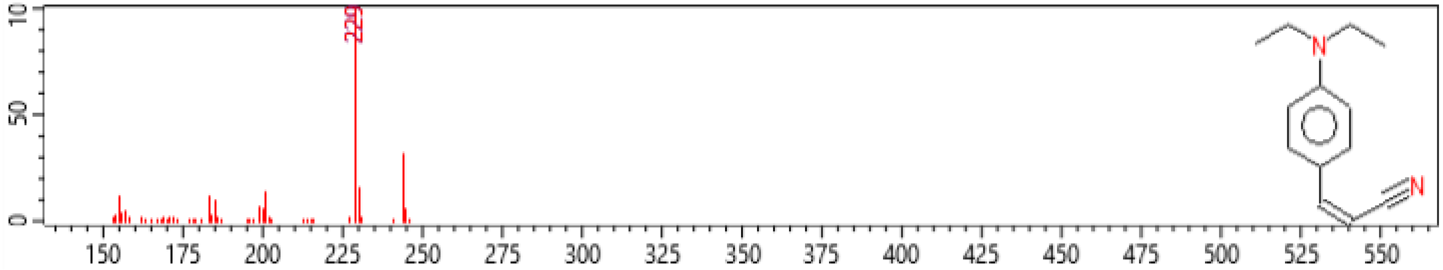

Silane, methylvinyl(pentyloxy)pentyloxy-

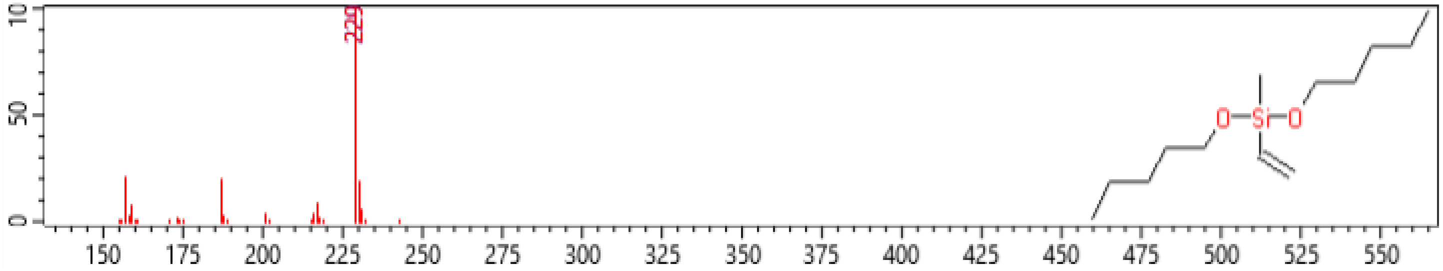

Lapachol

Peak: 13.03 - 13.06 min (scan #1684 - #1690) Subtracted noise: 12.99 - 13.01 min (scan #1671 - #1677), 13.06 - 13.08 min (scan #16

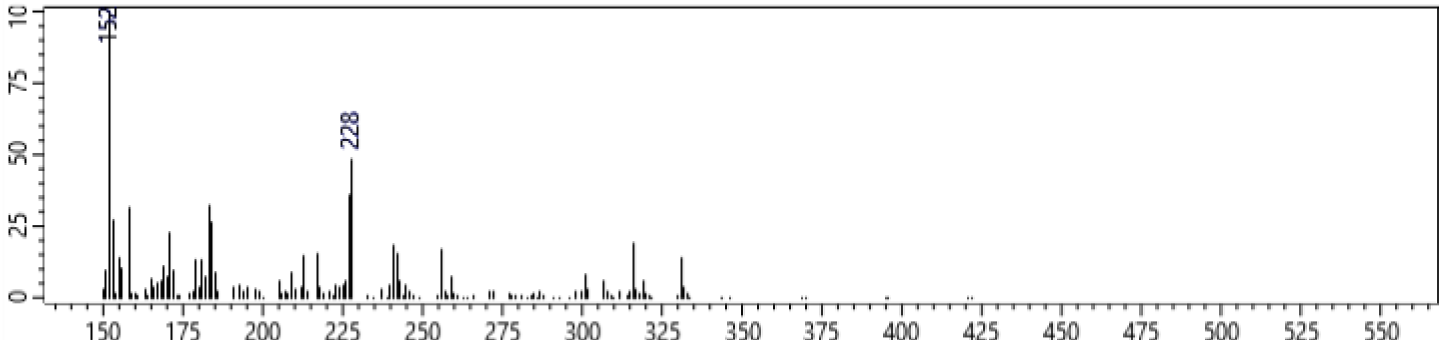

Lapachol

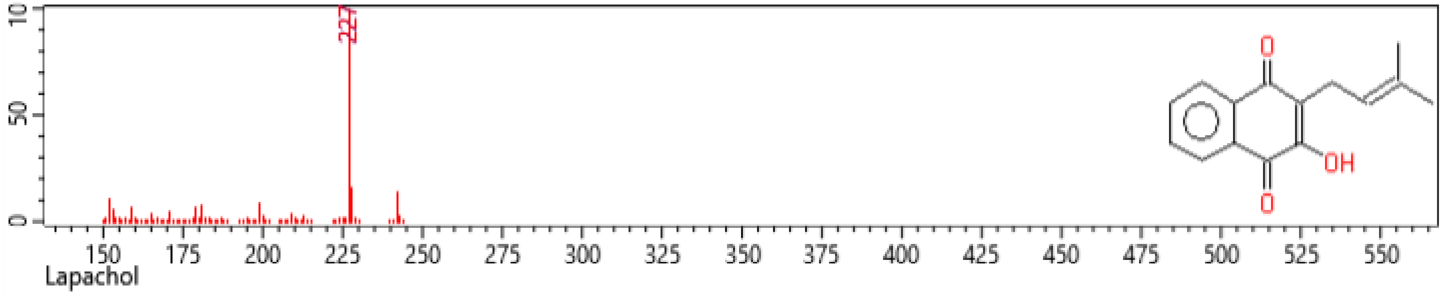

Lapachol

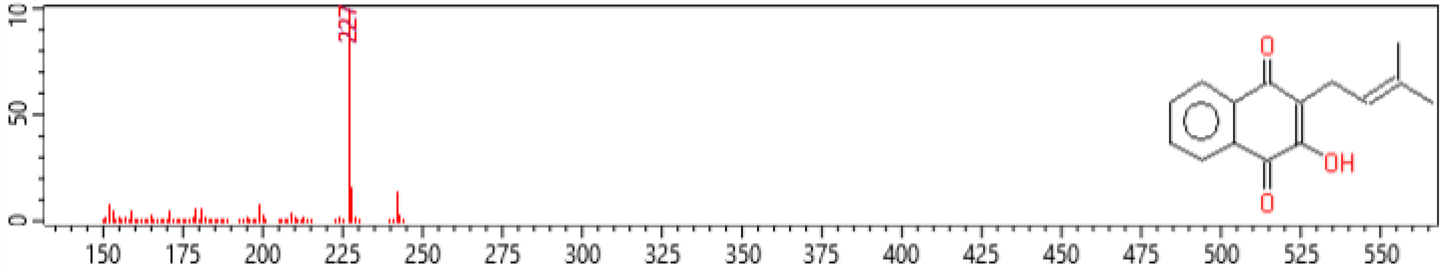

Nonanedioic acid, dibutyl ester

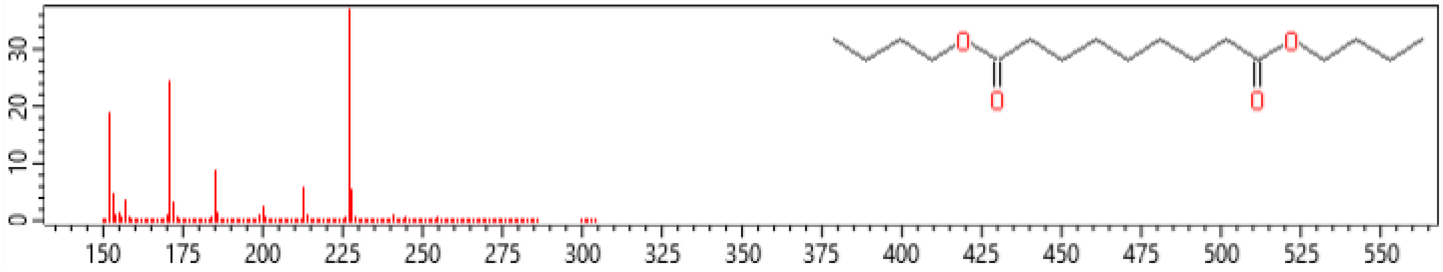

Dicyclopenta[a,d]benzene, 4,8-diethyl-1,5-dimethyl-

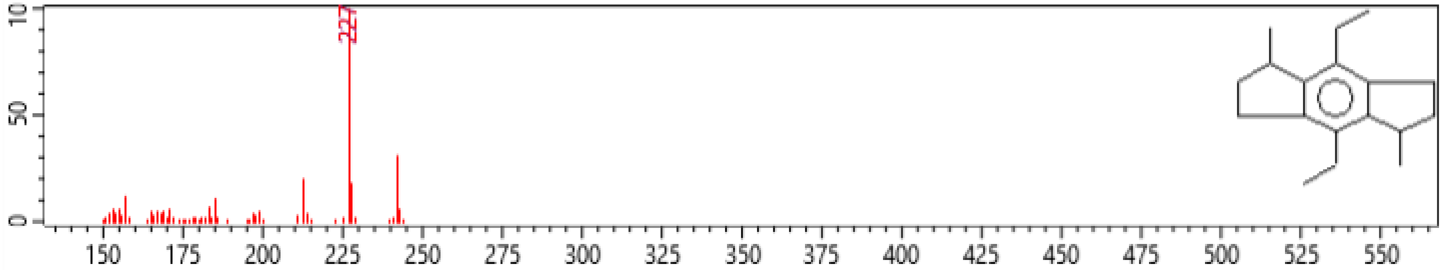

2-Hydroxy-1,3-dimethyl-9H-thioxanthen-9-one

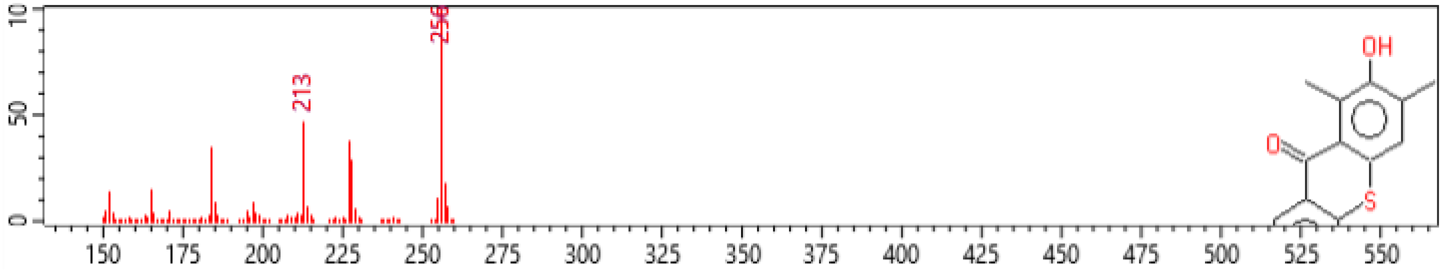

Styrene, 2,3,5,6-tetraethyl-4-vinyl-

Peak: 13.11 - 13.18 min (scan #1704 - #1724) Subtracted noise: 12.98 - 13.05 min (scan #1670 - #1689), 13.21 - 13.27 min (scan #17

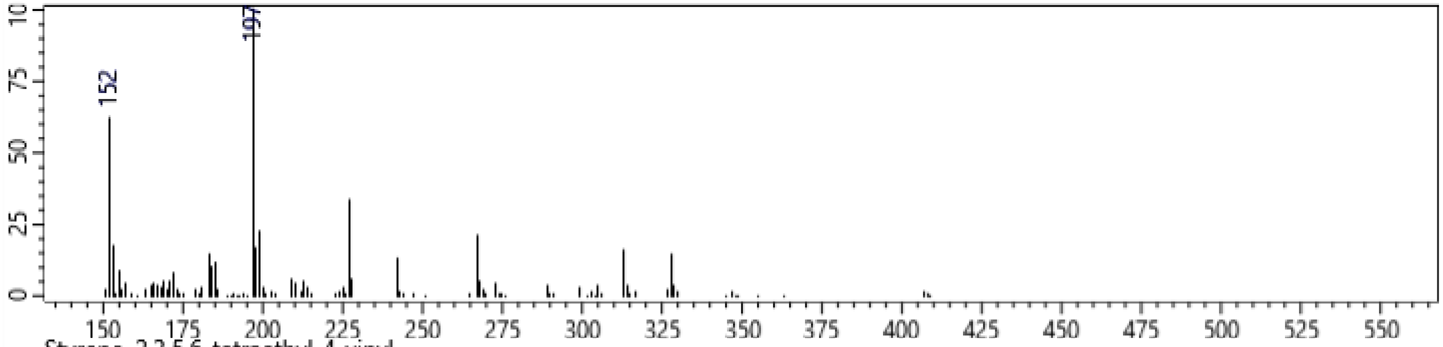

Styrene, 2,3,5,6-tetraethyl-4-vinyl-

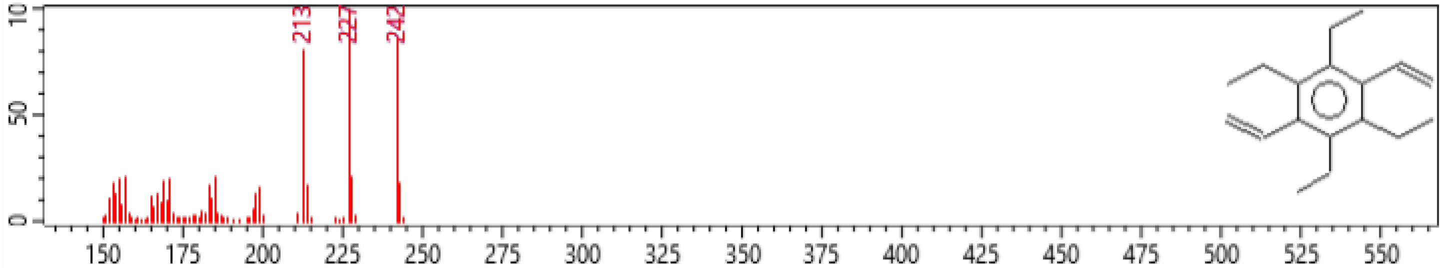

Benzocyclopentene, 4,6,7-triethyl-1-methyl-5-vinyl-

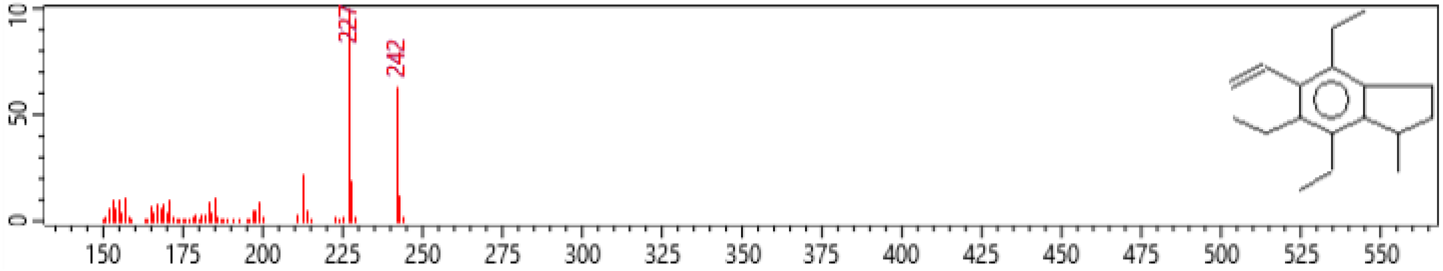

Schizandrin A

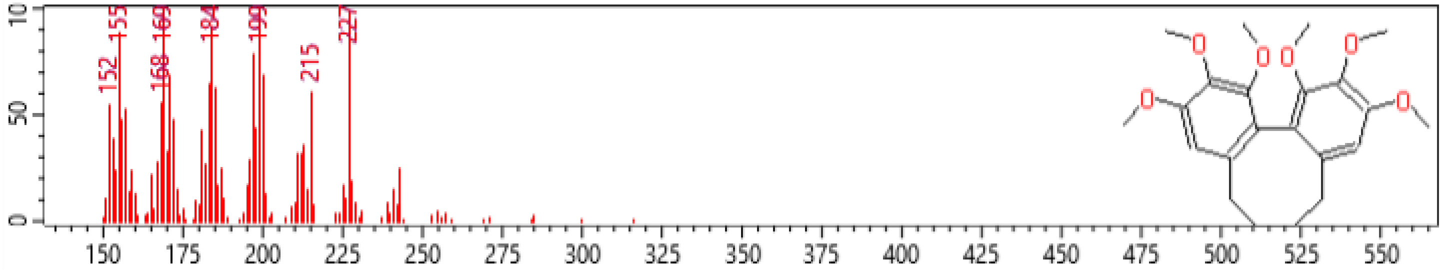

Tricin methyl ether

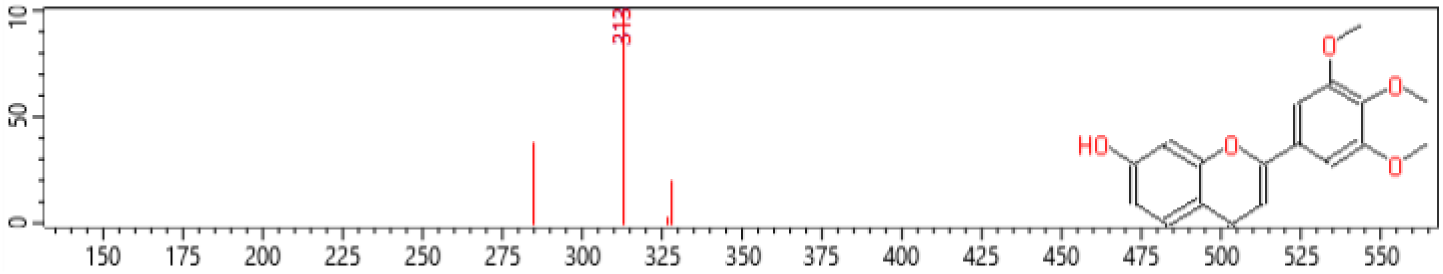

5,19-Cycloandrost-6-en-3-one, 17,17-ethylenedioxy-

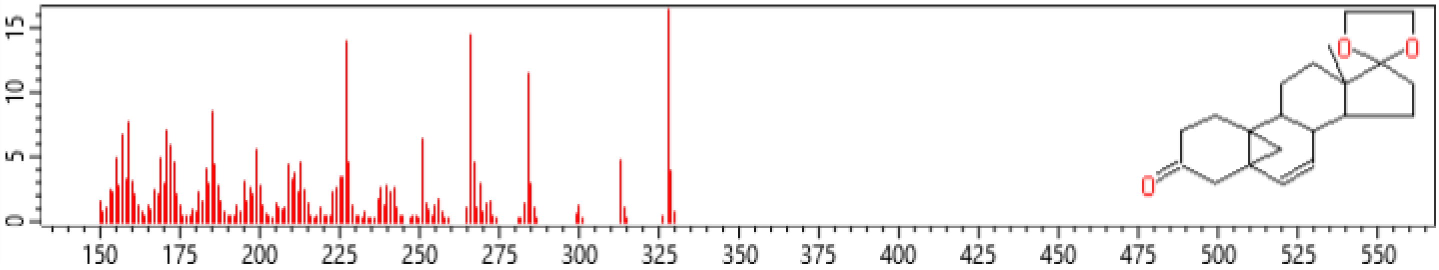

2-(2-Hydroxy-5-((1E)-3-hydroxyprop-1-en-1-yl)-3-methoxyphenyl)-3-(4-hydroxy-3-methoxyphenyl)propyl hexopyranoside

Peak: 13.22 - 13.27 min (scan #1735 - #1751) Subtracted noise: 13.15 - 13.20 min (scan #1715 - #1731), 13.32 - 13.39 min (scan #17

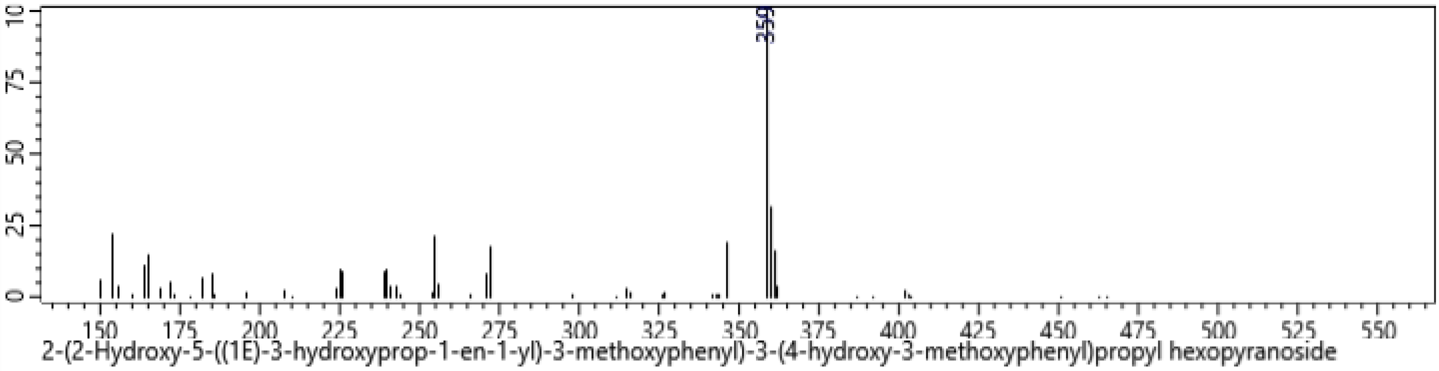

2-(2-Hydroxy-5-((1E)-3-hydroxyprop-1-en-1-yl)-3-methoxyphenyl)-3-(4-hydroxy-3-methoxyphenyl)propyl hexopyranoside

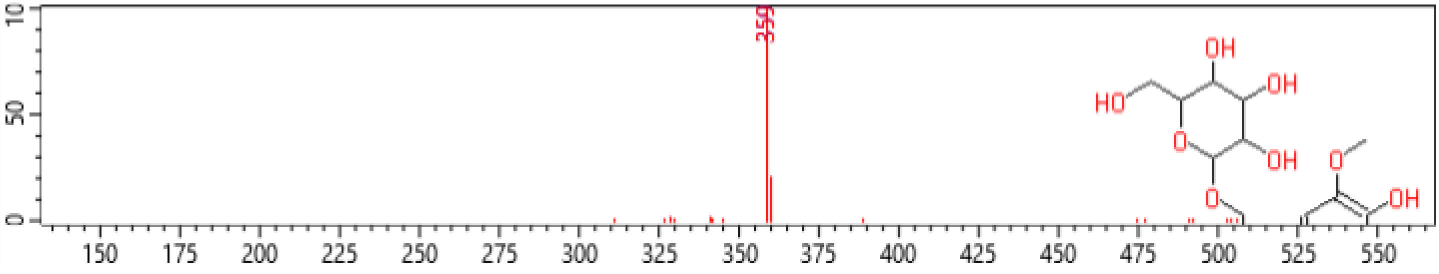

Pyrido[2,3-d]pyrimidin-7(8H)-one, 6-(2,6-dichlorophenyl)-2-[[3-(hydroxymethyl)phenyl]amino]-8-methyl-

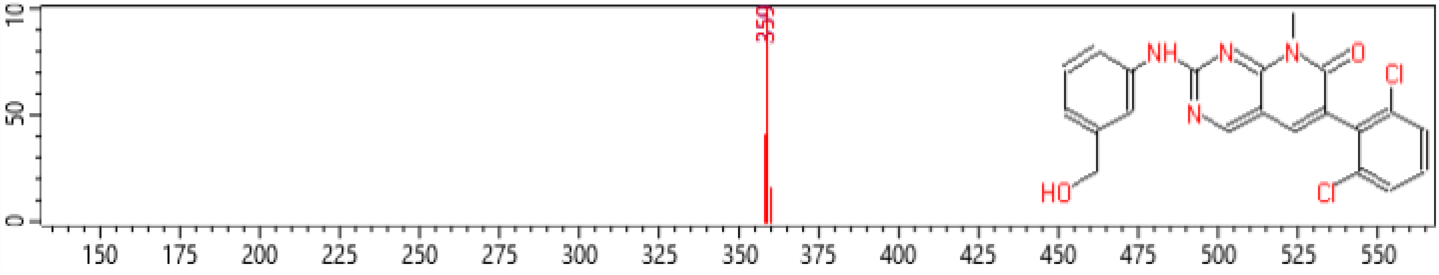

6-[(3,4-Dimethoxyphenyl)(4-morpholinyl)methyl]-1,3-benzodioxol-5-ol, trimethylsilyl ether

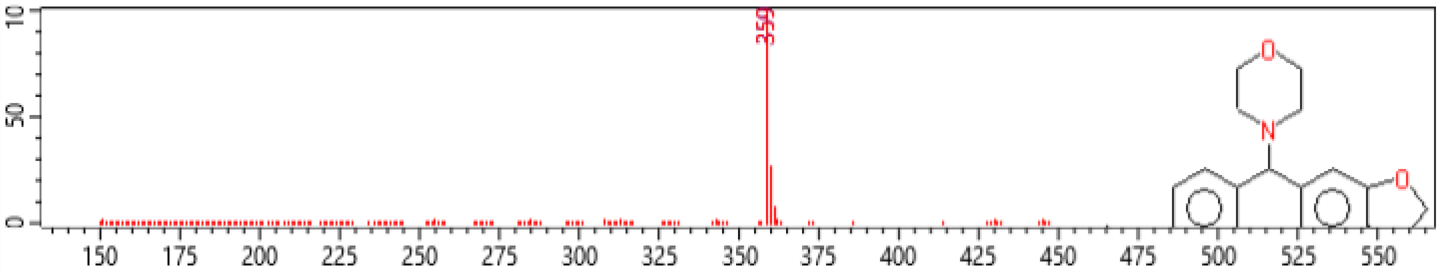

Silane, diethyl(2-ethoxyethyloxy)tetradecyloxy-

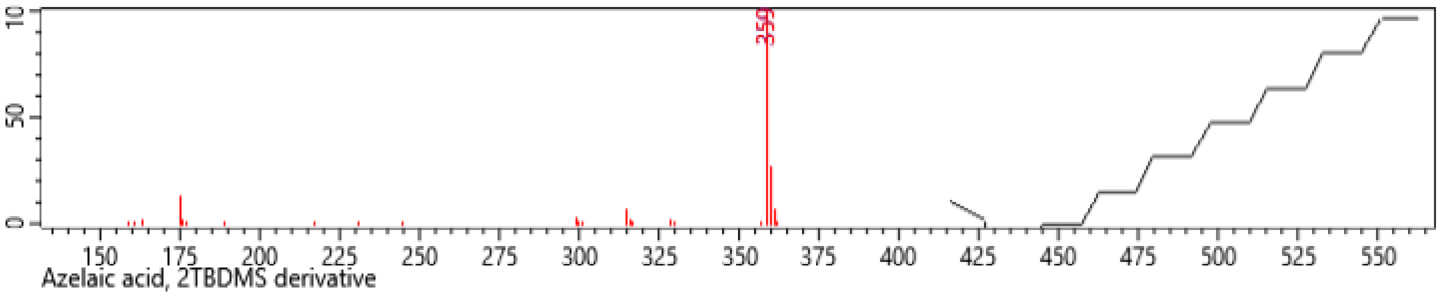

Azelaic acid, 2TBDMS derivative

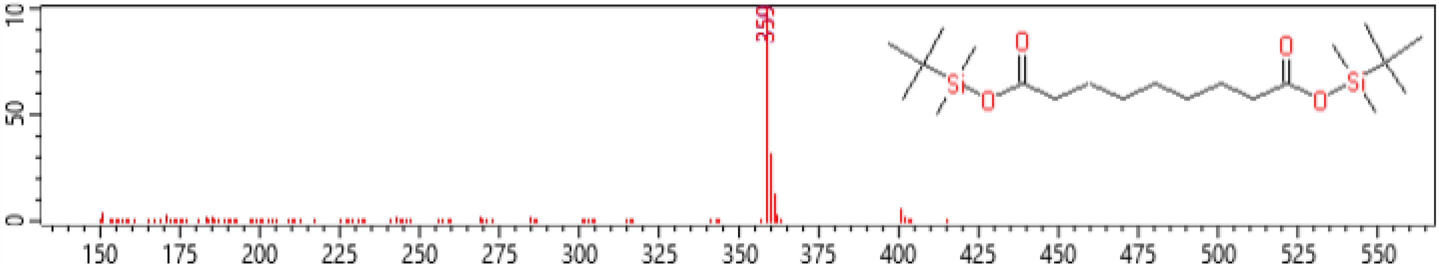

Aconitic acid, (E)-, 3TMS derivative

Peak: 13.37 - 13.40 min (scan #1777 - #1786) Subtracted noise: 13.29 - 13.32 min (scan #1755 - #1764), 13.40 - 13.43 min (scan #17[illegible]

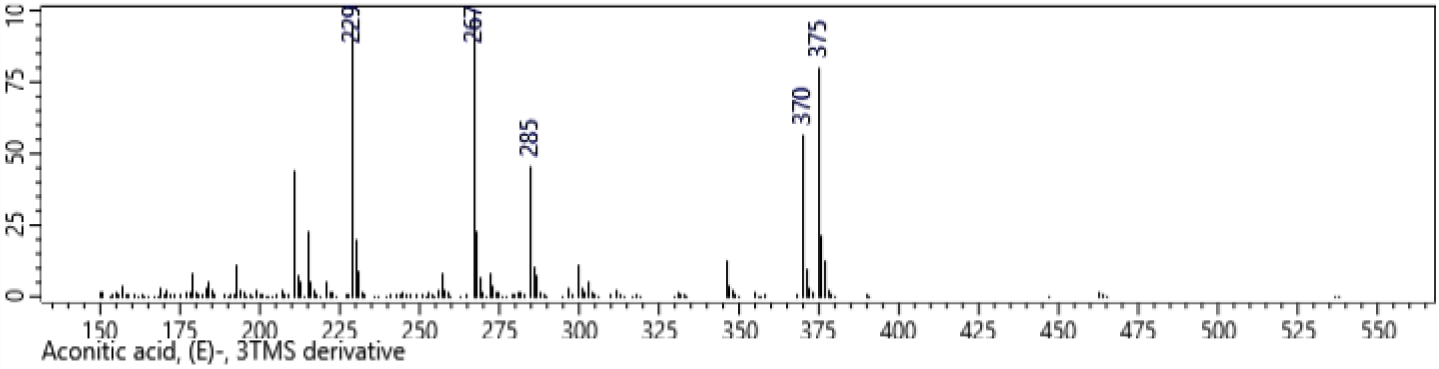

Aconitic acid, (E)-, 3TMS derivative

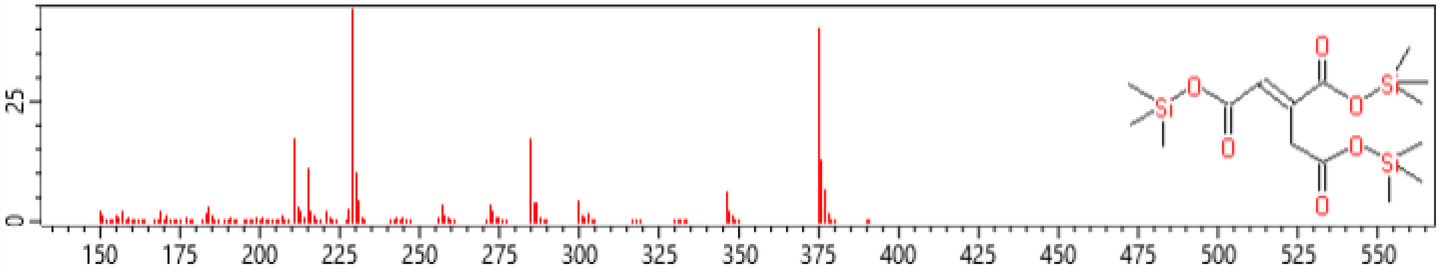

Aconitic acid, (E)-, 3TMS derivative

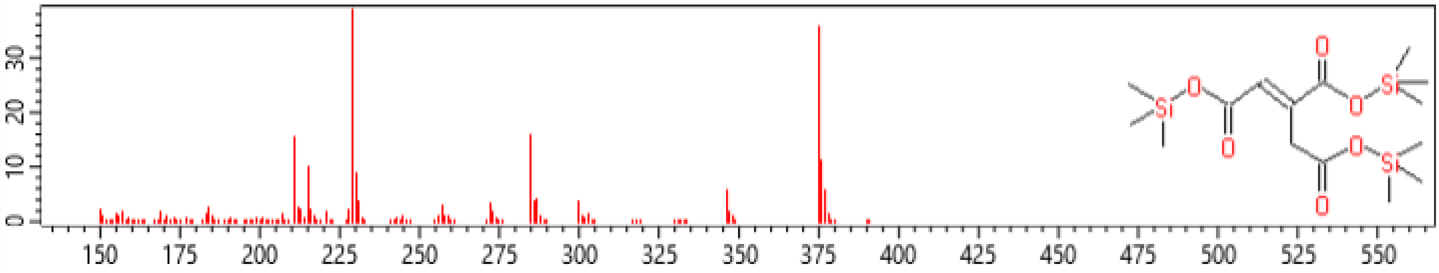

Aconitic acid, (Z)-, 3TMS derivative

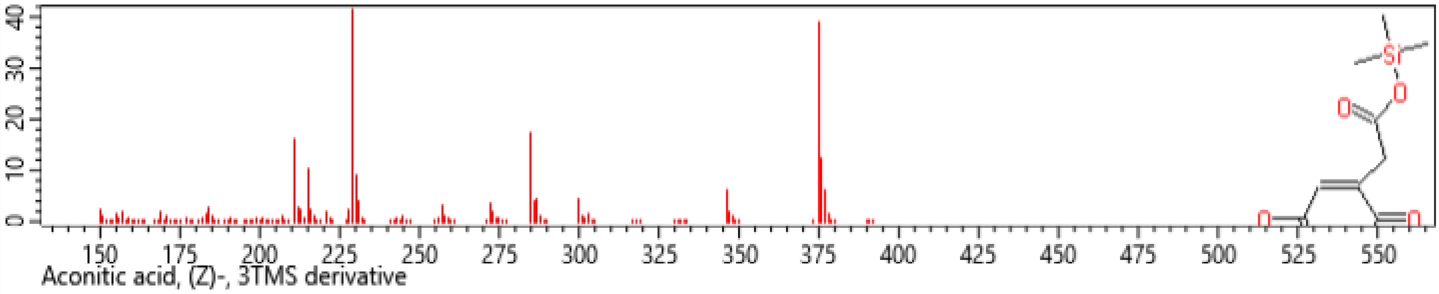

Aconitic acid, (Z)-, 3TMS derivative

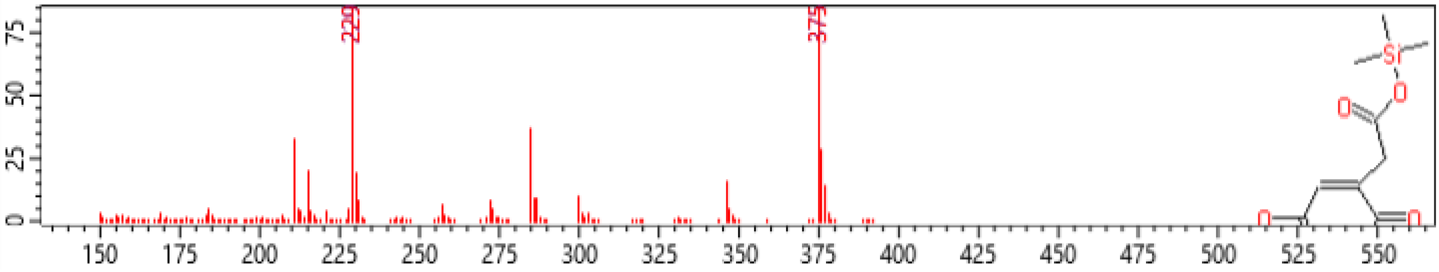

Aconitic acid, (Z)-, 3TMS derivative

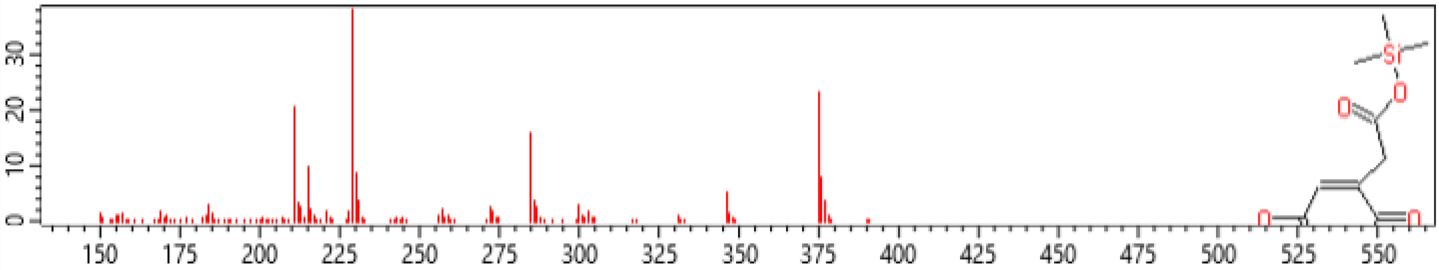

Ethanol, (2-(3,4-dihydroxyphenyl)-, tris(trimethylsilyl)-

Peak: 13.39 - 13.43 min (scan #1784 - #1794) Subtracted noise: 13.36 - 13.40 min (scan #1774 - #1785), 13.48 - 13.52 min (scan #18

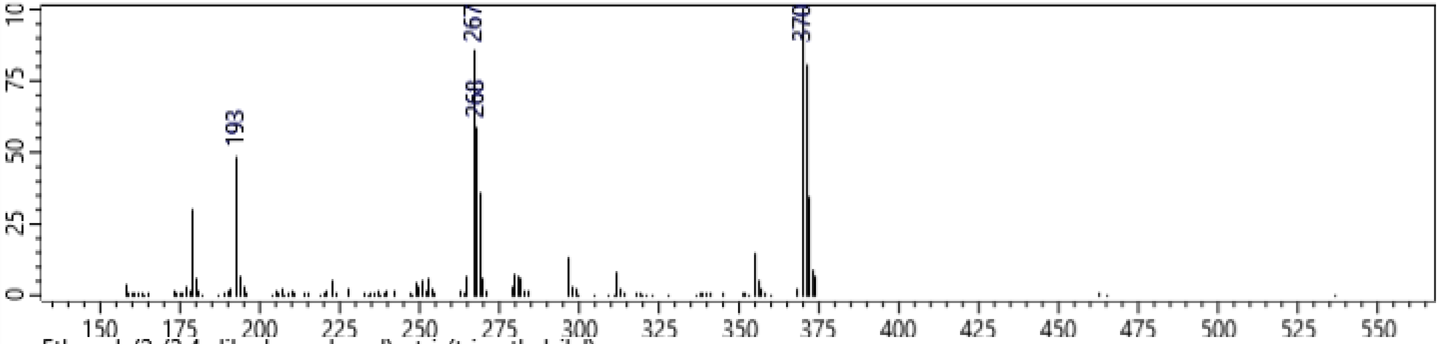

Ethanol, (2-(3,4-dihydroxyphenyl)-, tris(trimethylsilyl)-

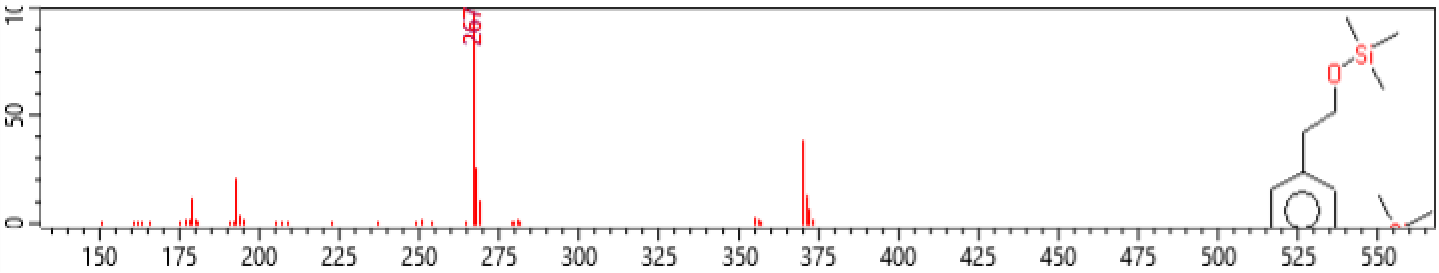

Ethanol, (2-(3,4-dihydroxyphenyl)-, tris(trimethylsilyl)-

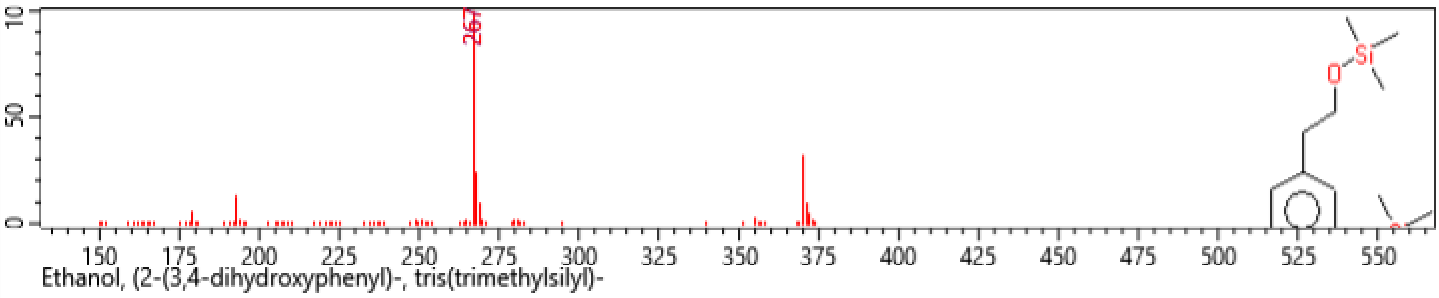

Ethanol, (2-(3,4-dihydroxyphenyl)-, tris(trimethylsilyl)-

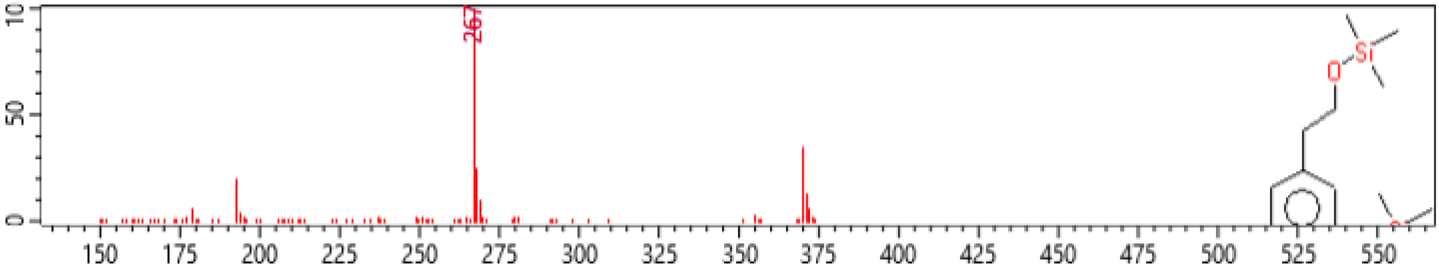

Ethanol, (2-(3,4-dihydroxyphenyl)-, tris(trimethylsilyl)-

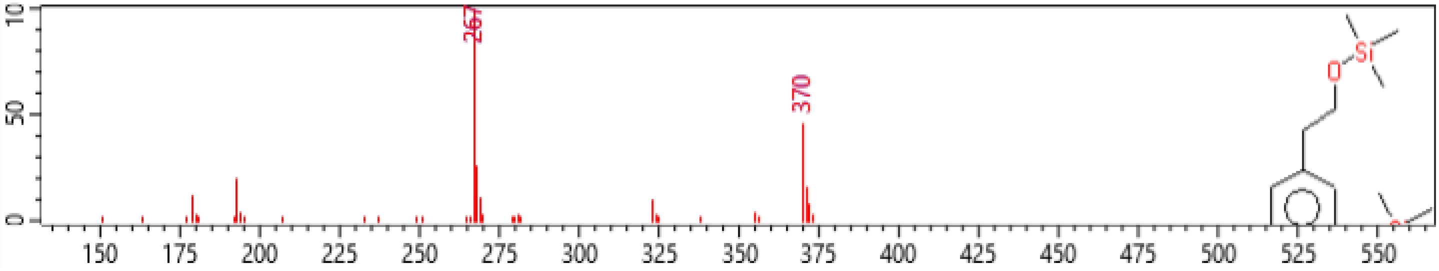

Rhodamine B cation

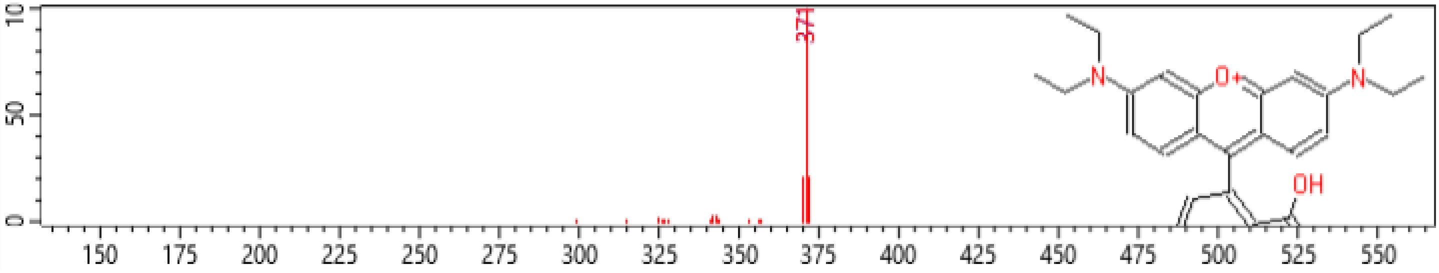

Rhodamine B cation

Peak: 13.49 - 13.50 min (scan #1812 - #1814) Subtracted noise: 13.47 - 13.48 min (scan #1805 - #1808), 13.51 - 13.51 min (scan #18

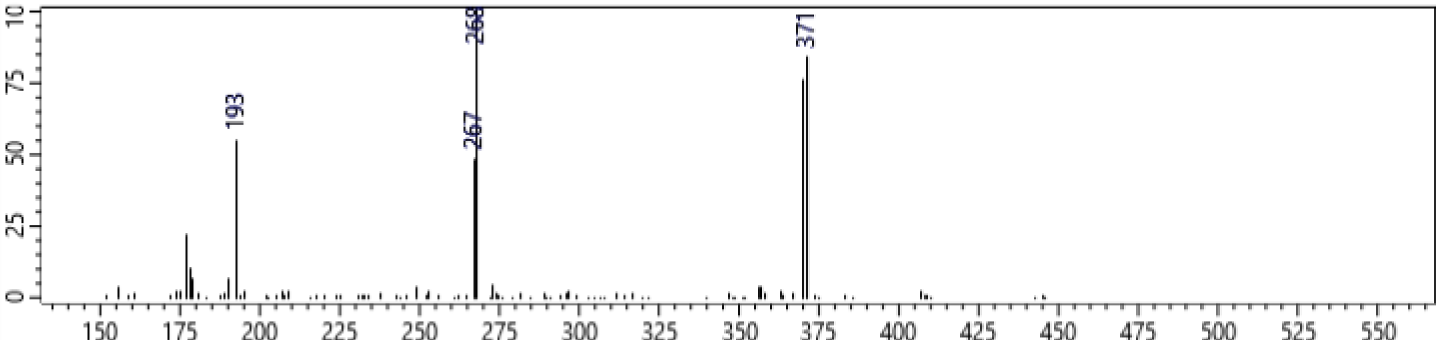

Rhodamine B cation

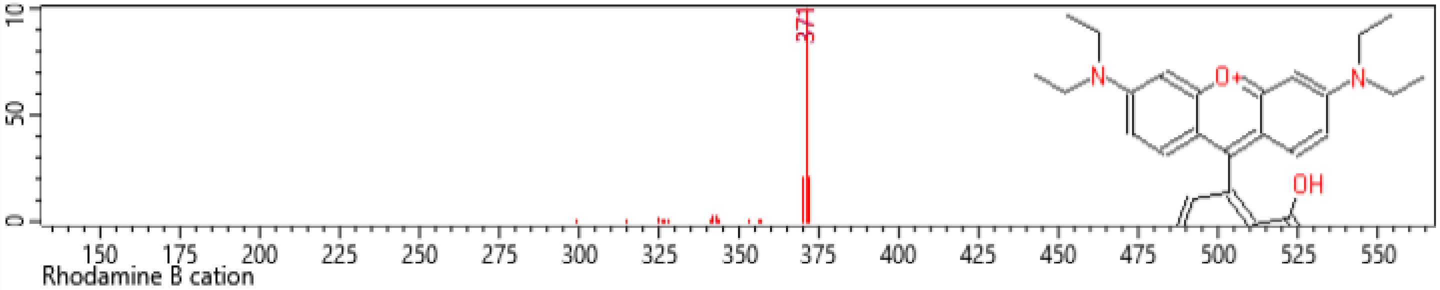

Rhodamine B cation

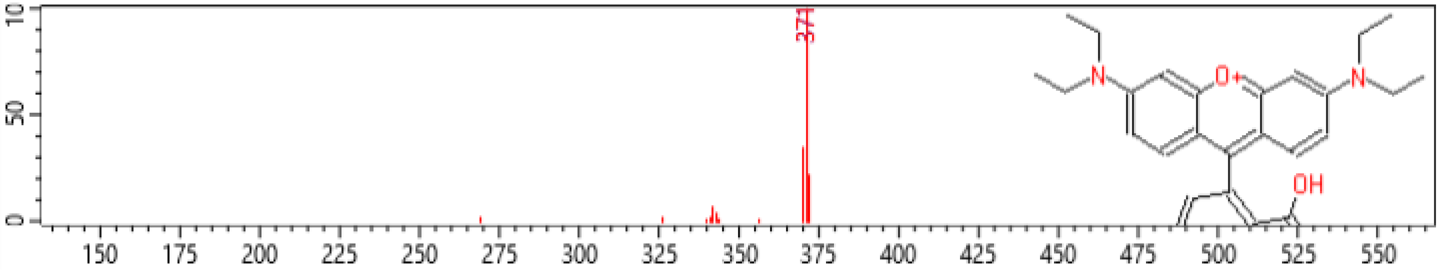

Ethanol, (2-(3,4-dihydroxyphenyl)-, tris(trimethylsilyl)-

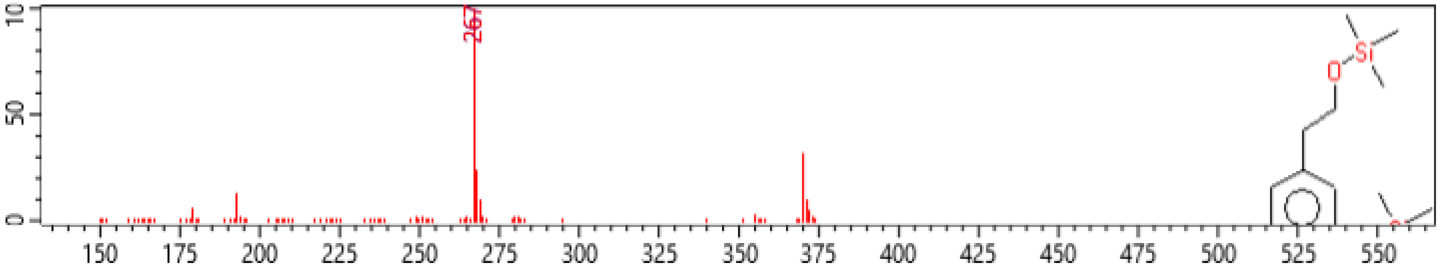

Ethanol, (2-(3,4-dihydroxyphenyl)-, tris(trimethylsilyl)-

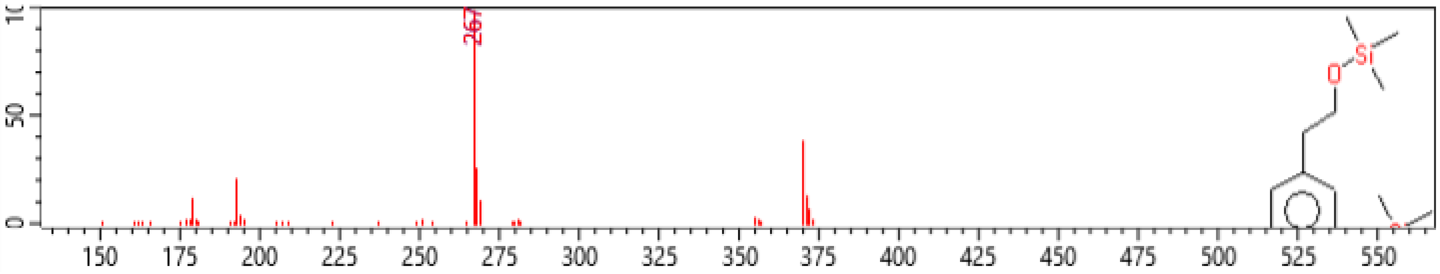

Ethanol, (2-(3,4-dihydroxyphenyl)-, tris(trimethylsilyl)-

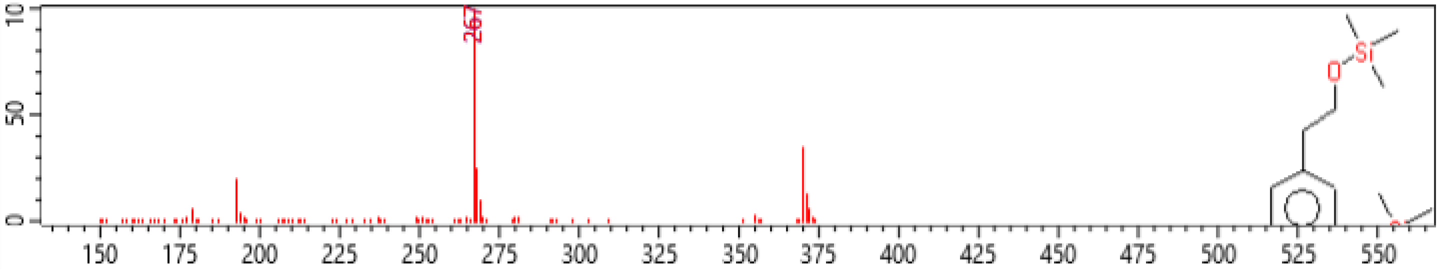

Ethanol, (2-(3,4-dihydroxyphenyl)-, tris(trimethylsilyl)-

Peak: 13.54 - 13.58 min (scan #1825 - #1837) Subtracted noise: 13.46 - 13.50 min (scan #1802 - #1815), 13.60 - 13.64 min (scan #18

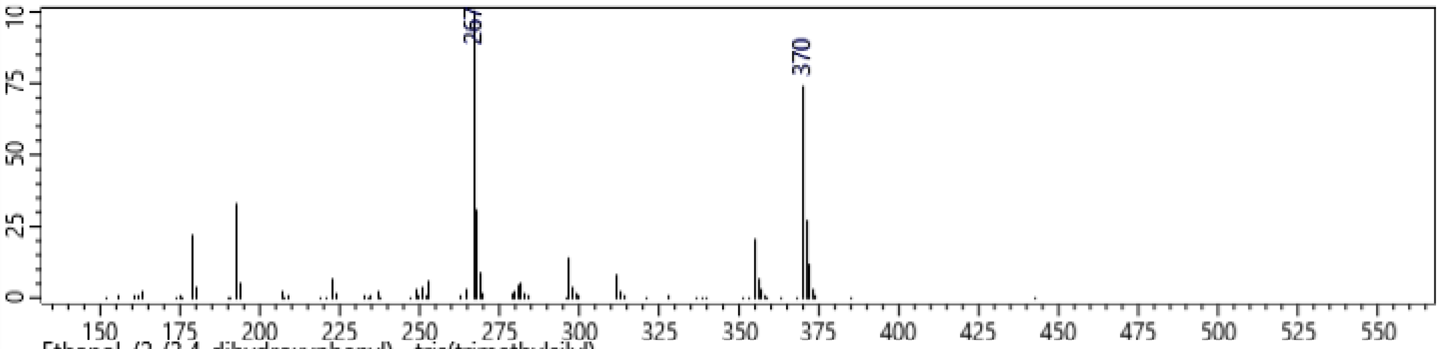

Ethanol, (2-(3,4-dihydroxyphenyl)-, tris(trimethylsilyl)-

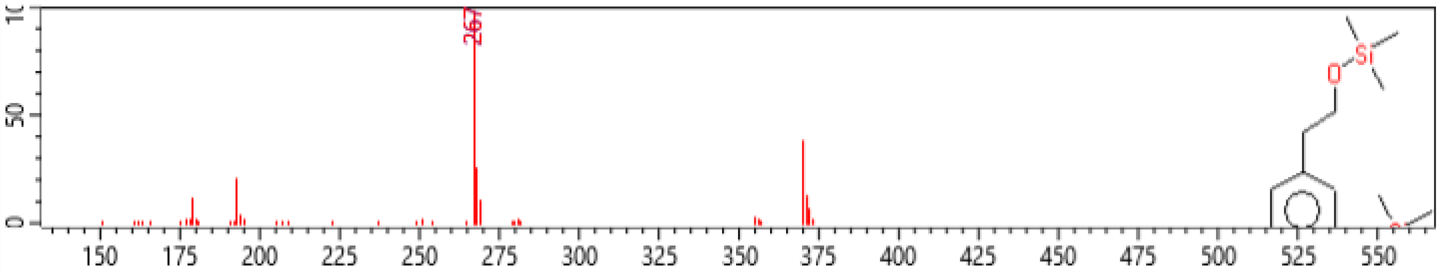

Ethanol, (2-(3,4-dihydroxyphenyl)-, tris(trimethylsilyl)-

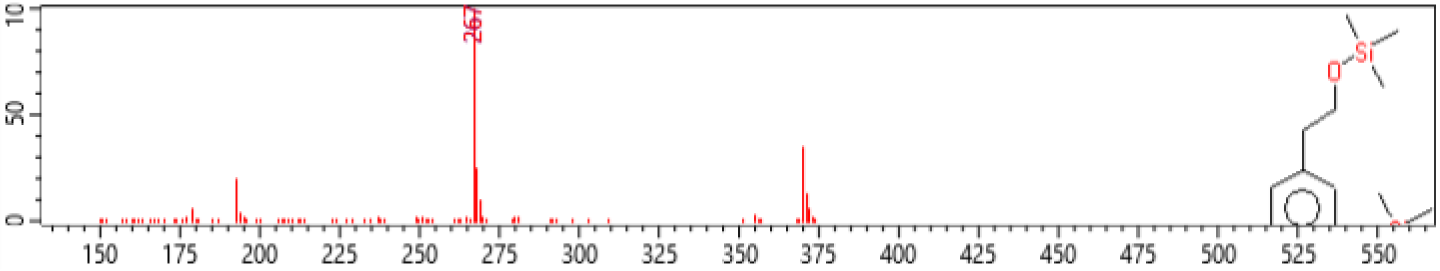

Ethanol, (2-(3,4-dihydroxyphenyl)-, tris(trimethylsilyl)-

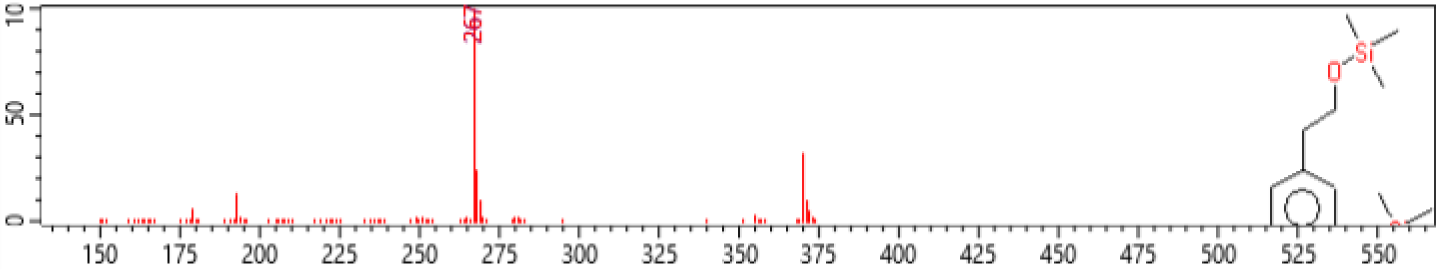

Ethanol, (2-(3,4-dihydroxyphenyl)-, tris(trimethylsilyl)-

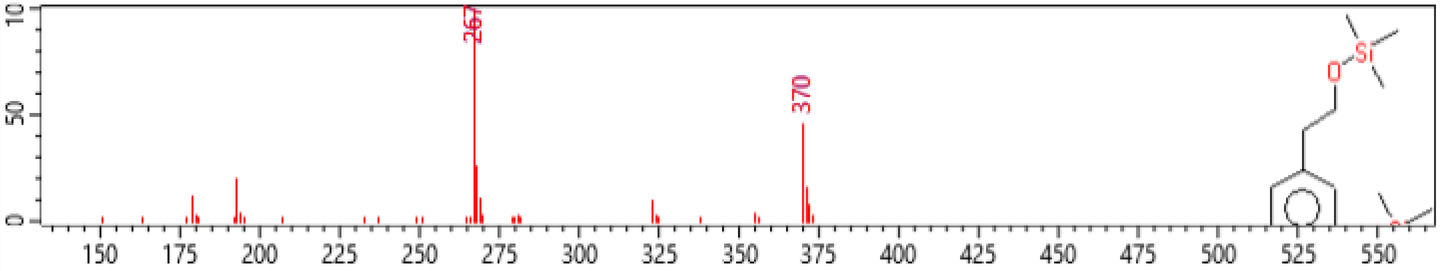

Protocatechoic acid, 3TMS derivative

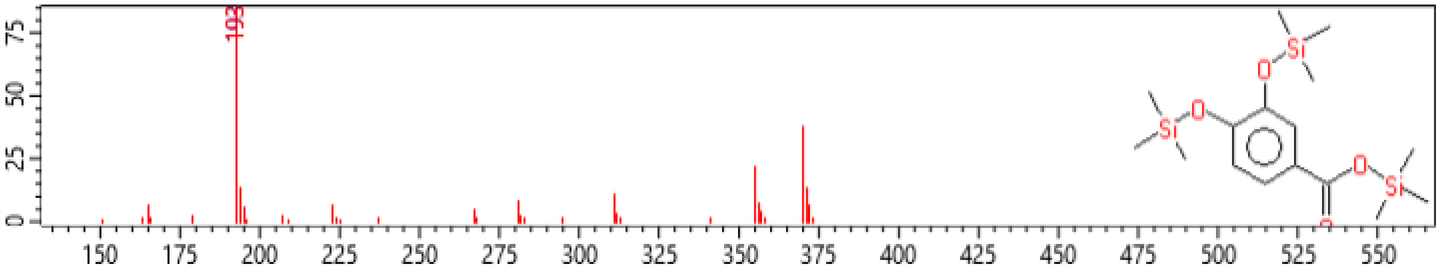

Auranticin A

Peak: 13.62 - 13.65 min (scan #1847 - #1855) Subtracted noise: 13.57 - 13.59 min (scan #1833 - #1840), 13.66 - 13.69 min (scan #18

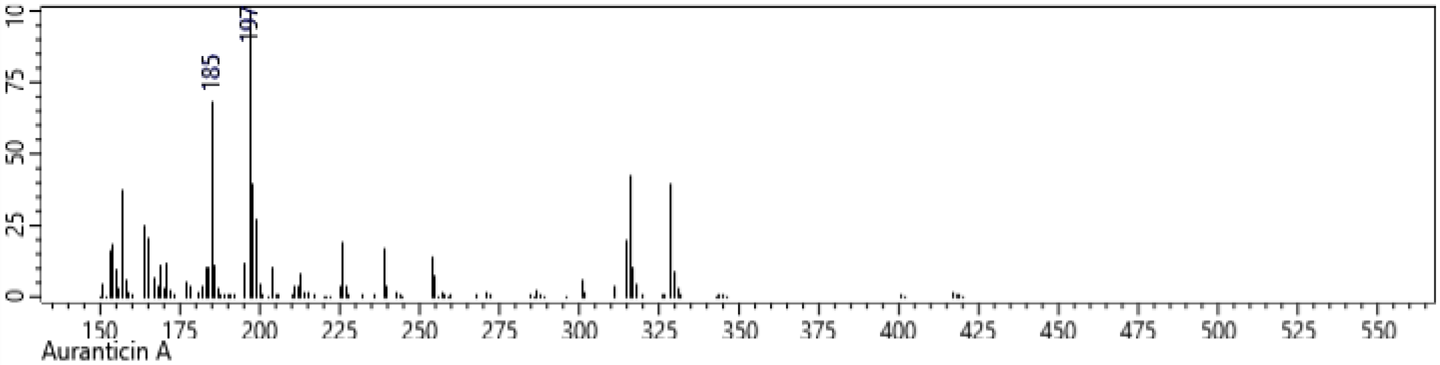

Auranticin A

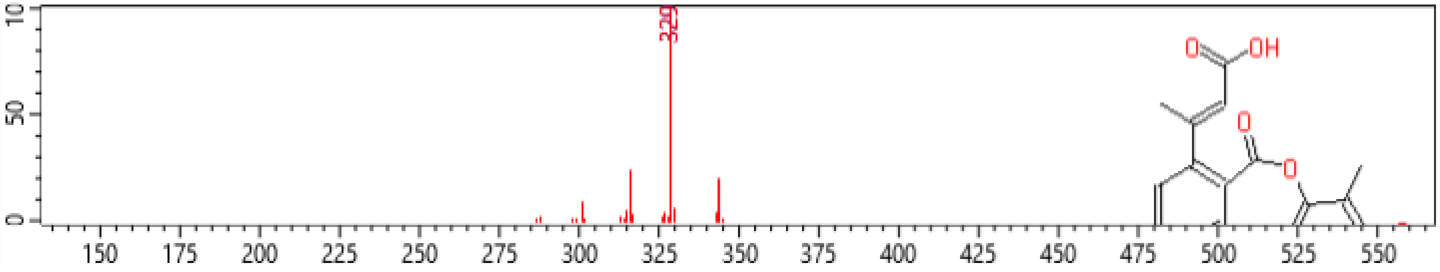

Nitrendipine

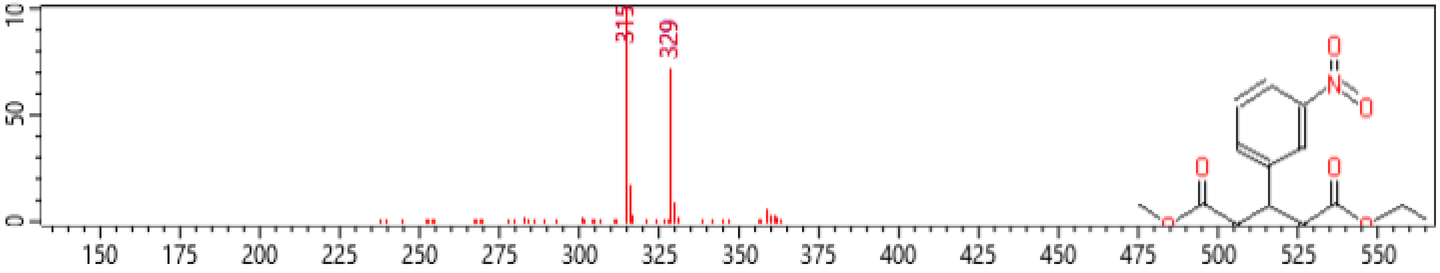

Nitrendipine

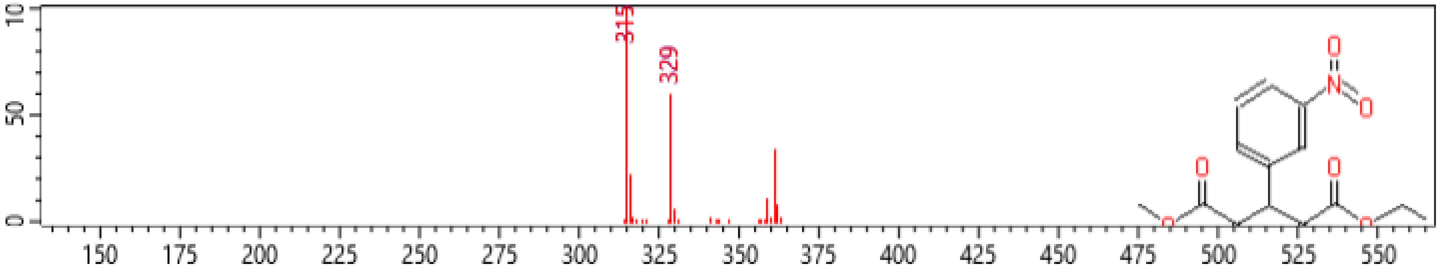

Androst-1-en-3-one, 17-(acetyloxy)-4,5-epoxy-, (4β,5β,17β)-

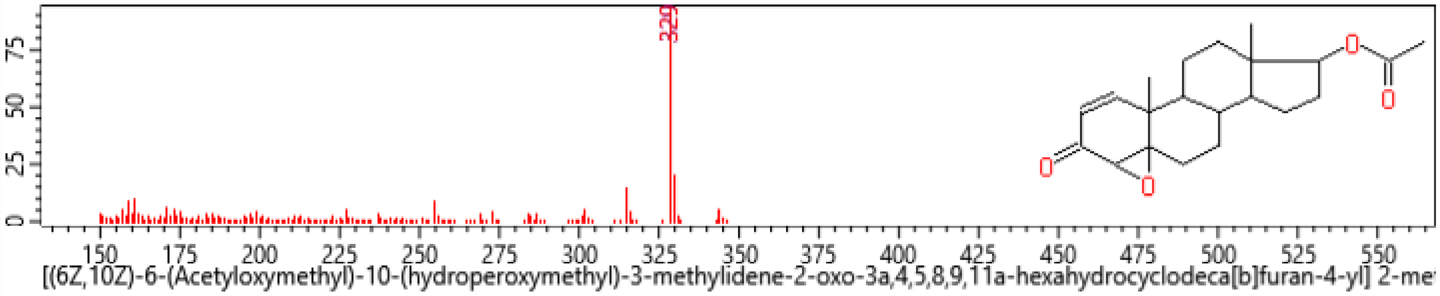

[(6Z,10Z)-6-(Acetyloxymethyl)-10-(hydroperoxymethyl)-3-methylidene-2-oxo-3a,4,5,8,9,11a-hexahydrocyclodeca[b]furan-4-yl] 2-me[illegible]

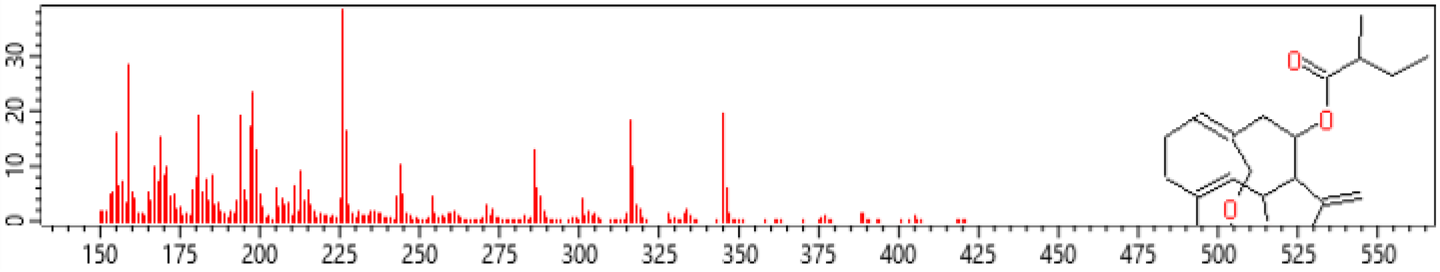

2-Ethyl 1,3-bis(trimethylsilyl) 2-((trimethylsilyl)oxy)propane-1,2,3-tricarboxylate

Peak: 13.68 - 13.69 min (scan #1863 - #1866) Subtracted noise: 13.65 - 13.65 min (scan #1855 - #1857), 13.69 - 13.70 min (scan #18[illegible]

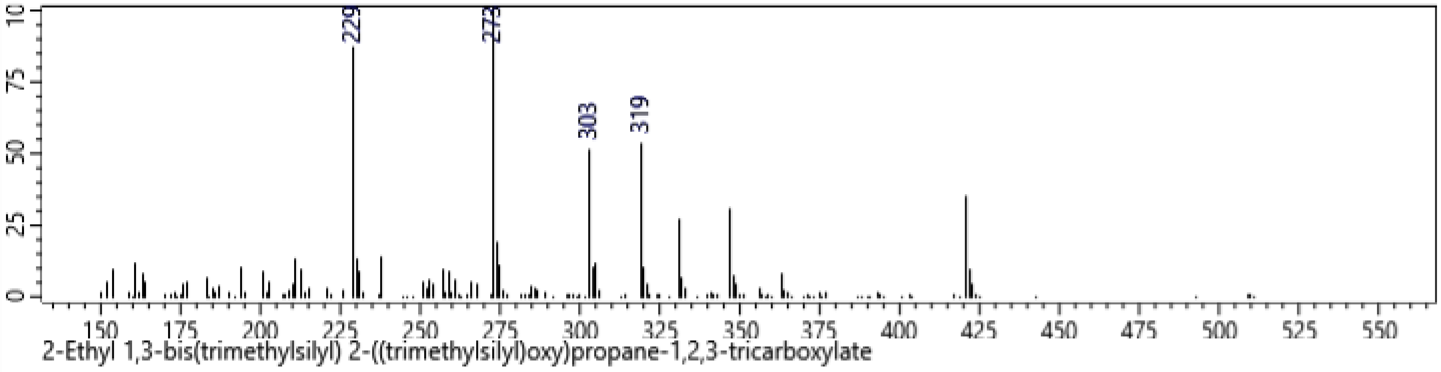

2-Ethyl 1,3-bis(trimethylsilyl) 2-((trimethylsilyl)oxy)propane-1,2,3-tricarboxylate

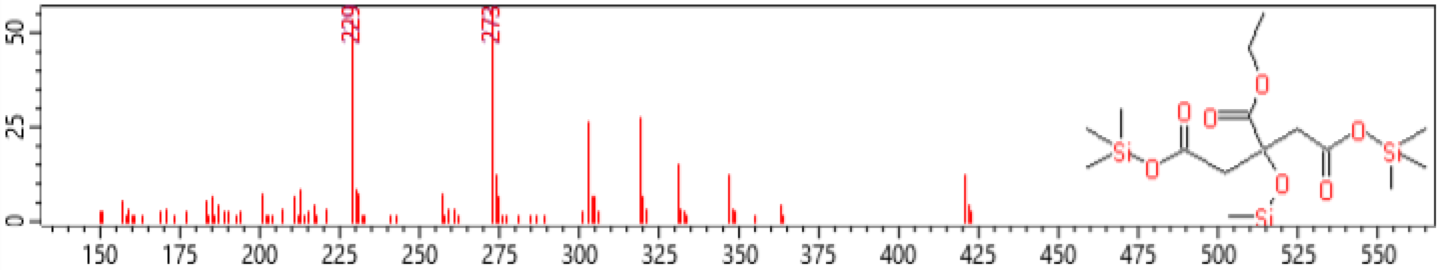

2-Ethyl 1,3-bis(trimethylsilyl) 2-((trimethylsilyl)oxy)propane-1,2,3-tricarboxylate

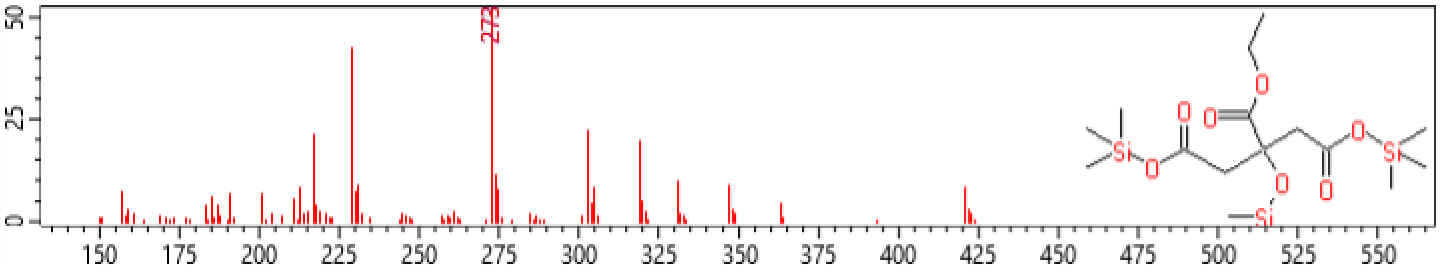

Citric acid, 4TMS derivative

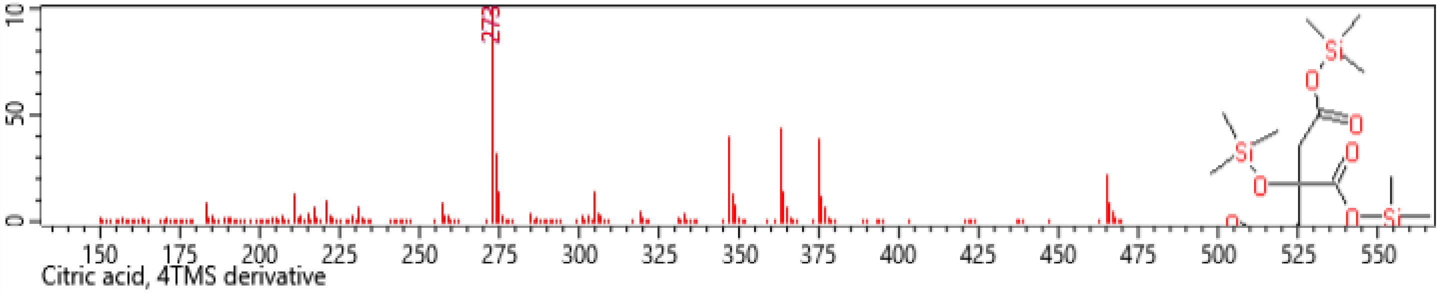

Citric acid, 4TMS derivative

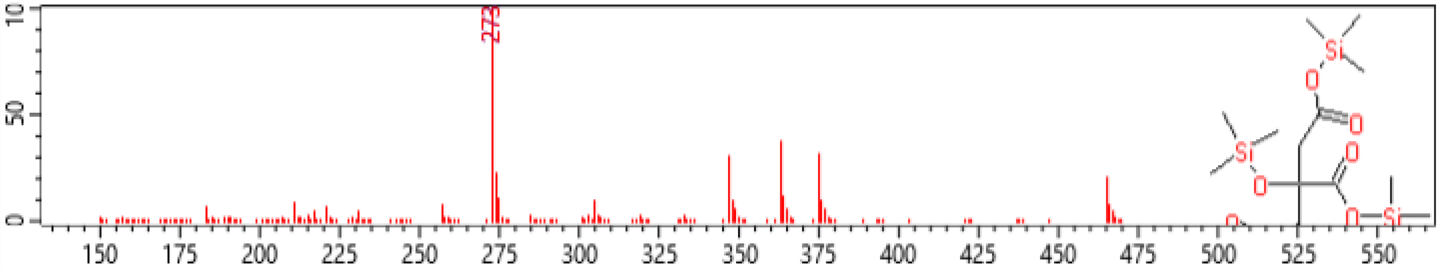

Diethyl 2-(2-furyl)-4-hydroxy-4-methyl-6-oxo-1,3-cyclohexanedicarboxylate tms pk2

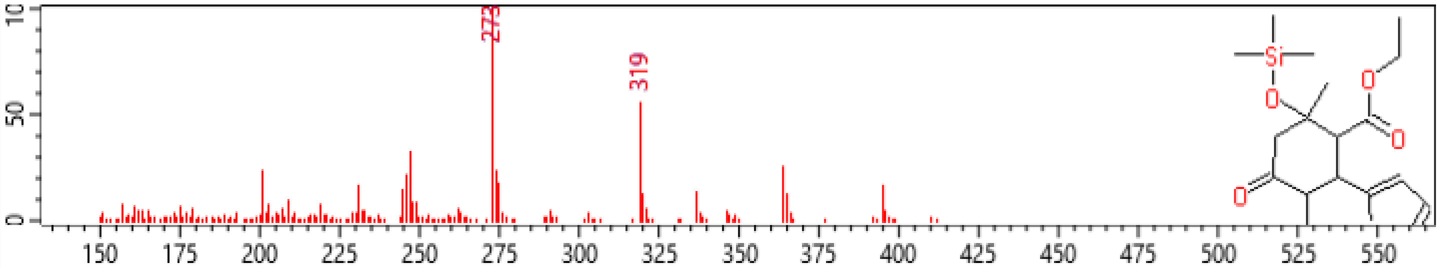

4-Coumaric acid, 2TMS

Peak: 13.70 - 13.73 min (scan #1871 - #1877) Subtracted noise: 13.67 - 13.69 min (scan #1860 - #1866), 13.74 - 13.76 min (scan #18

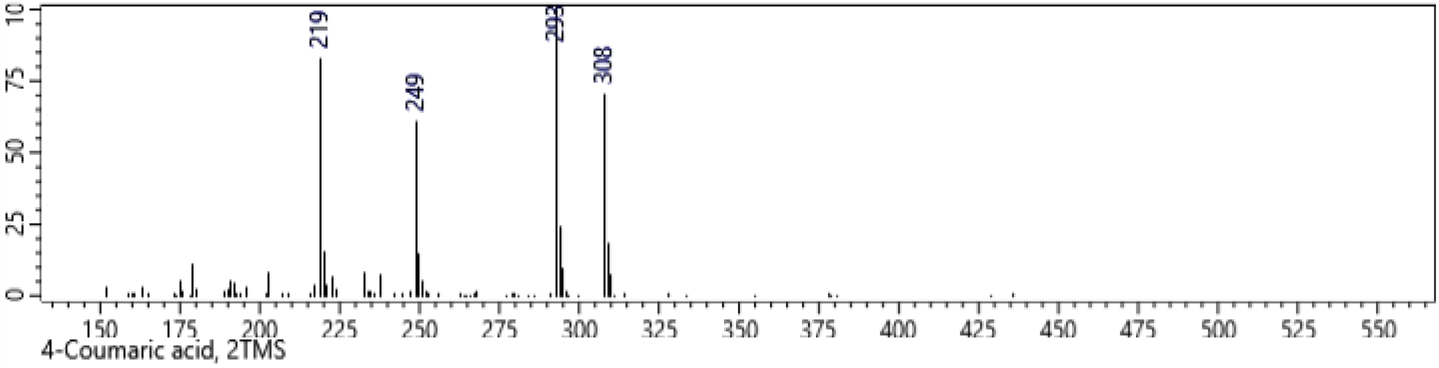

4-Coumaric acid, 2TMS

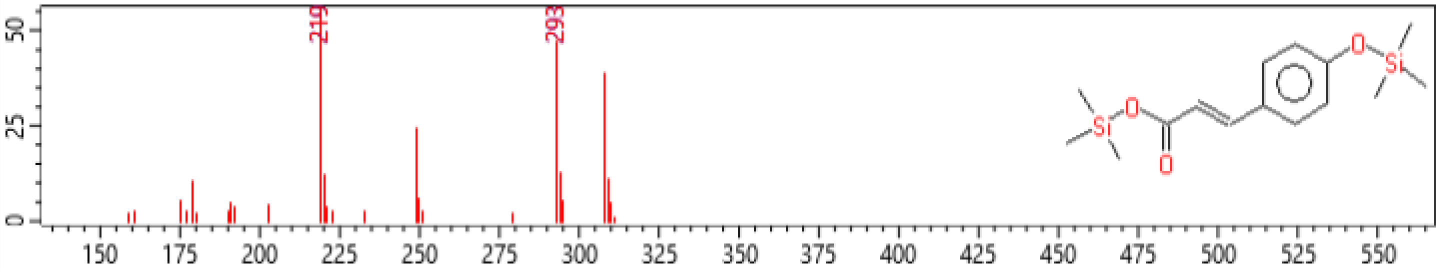

4-Coumaric acid, 2TMS

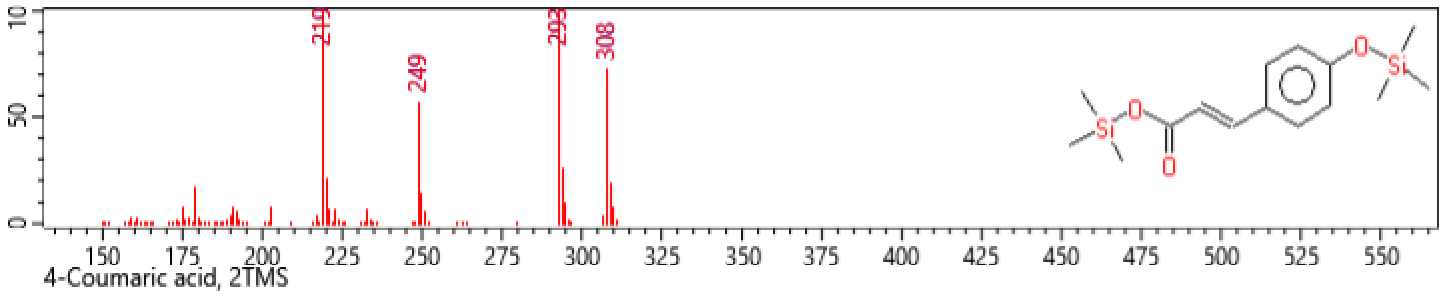

4-Coumaric acid, 2TMS

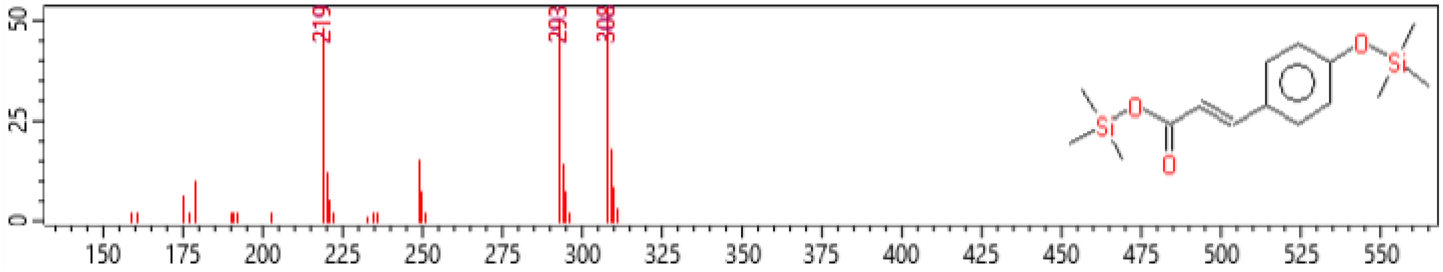

4-Coumaric acid, 2TMS

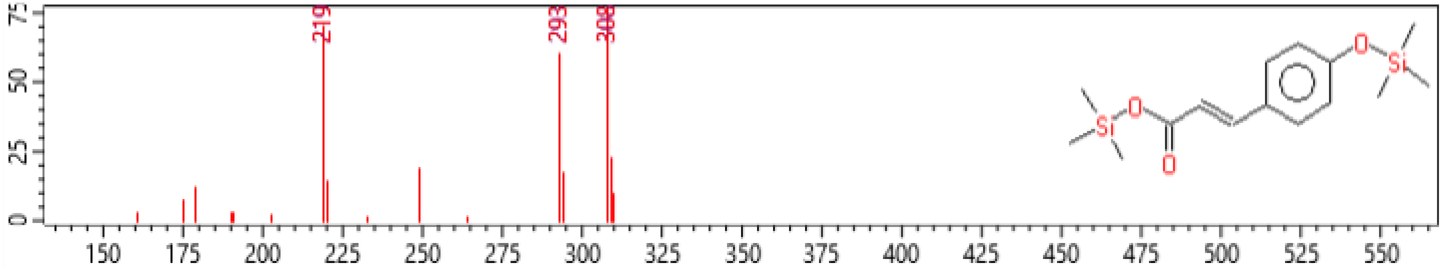

m-Coumaric acid, 2TMS derivative

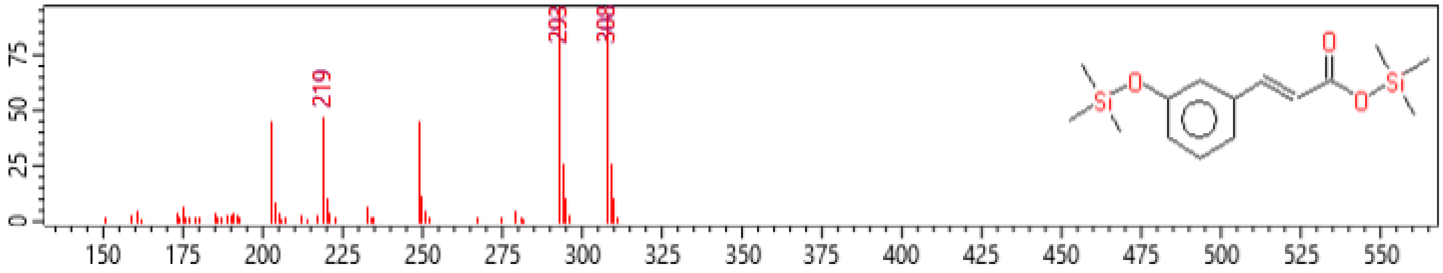

Isoquinoline-4-carbonitrile, 1-ethyl-6,7-dimethoxy-3-methyl-

Peak: 13.75 - 13.76 min (scan #1883 - #1887) Subtracted noise: 13.72 - 13.74 min (scan #1876 - #1880), 13.77 - 13.78 min (scan #18

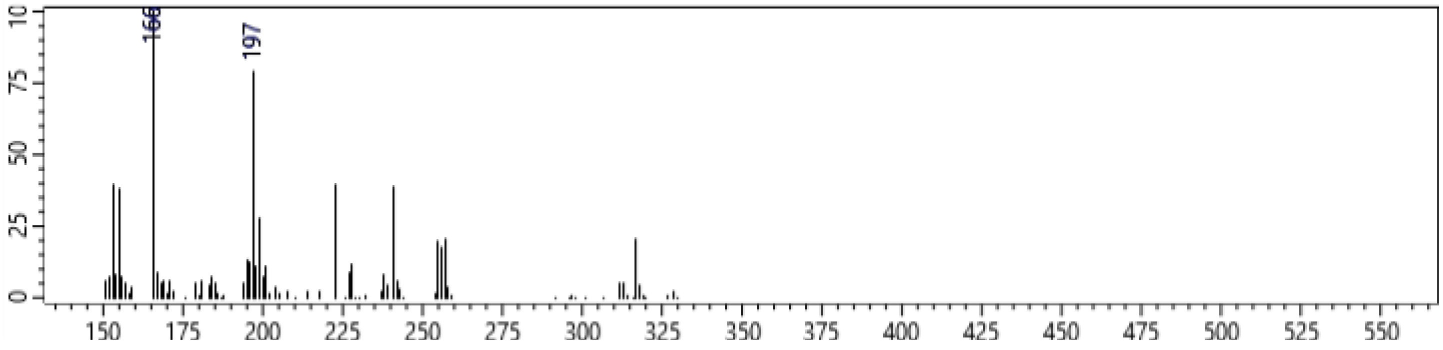

Isoquinoline-4-carbonitrile, 1-ethyl-6,7-dimethoxy-3-methyl-

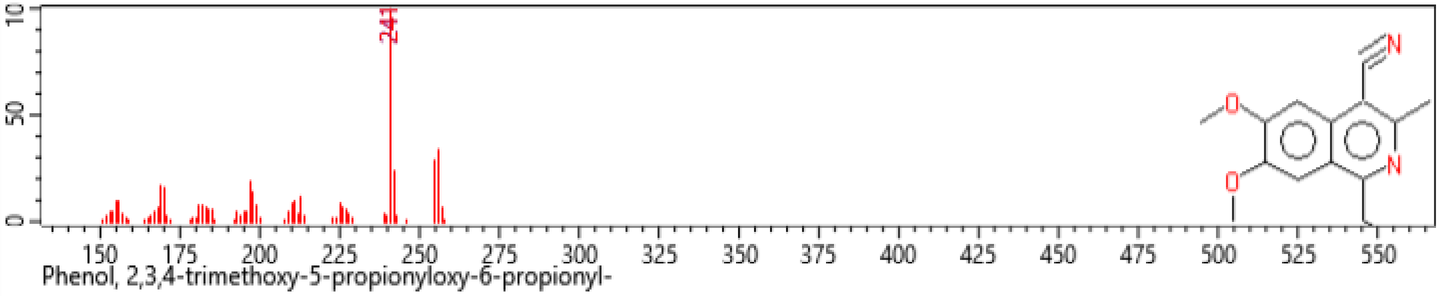

Phenol, 2,3,4-trimethoxy-5-propionyloxy-6-propionyl-

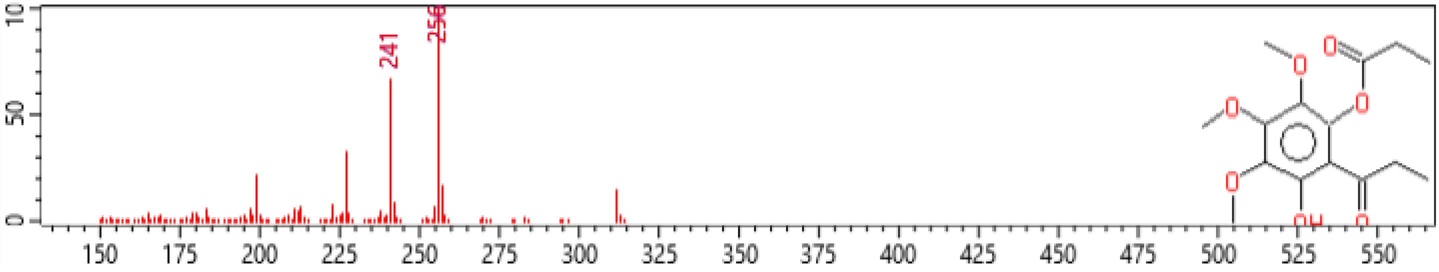

Phen-1,3-diol, 4,5,6-trimethoxy-2-propionyl-

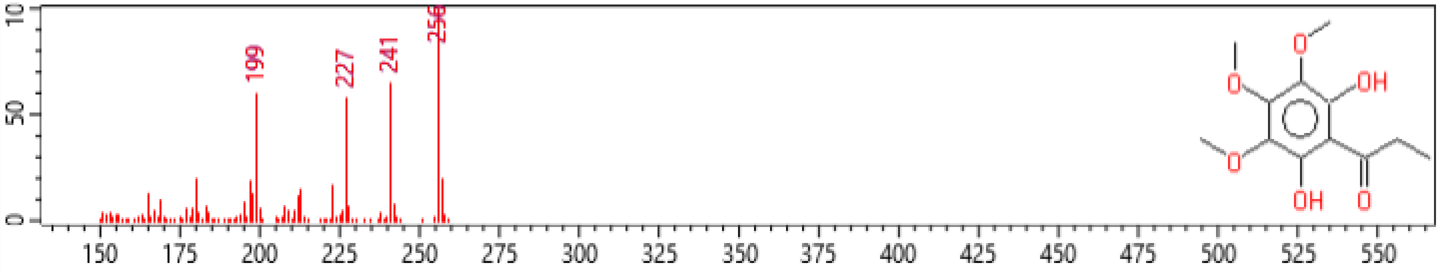

5,6-dihydroimidazo[2,1-b][1,3]thiazol-3-ylacetic acid, trimethylsilyl ester

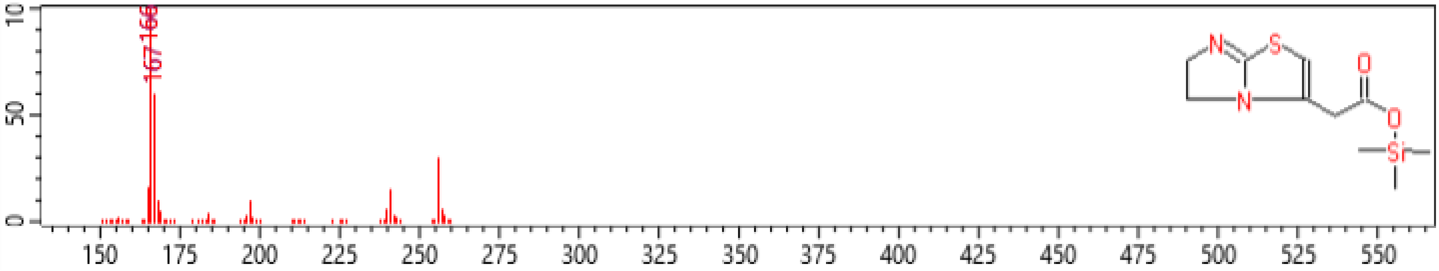

Quercetin 3,5,7,3',4'-pentamethyl ether

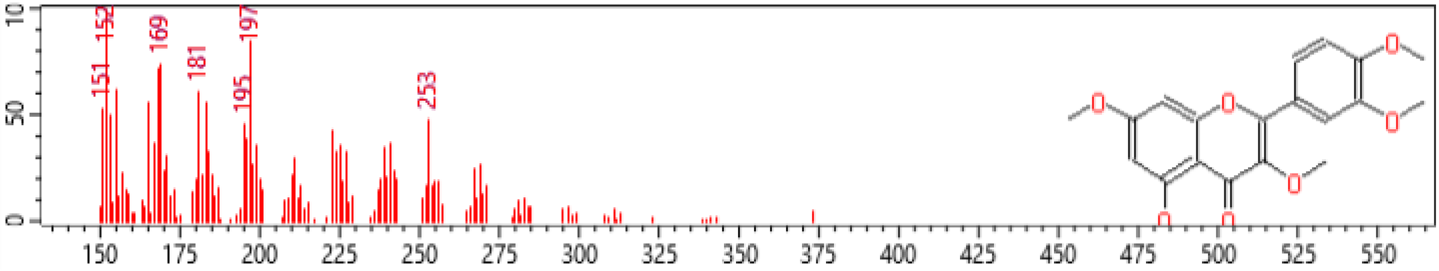

5-Methyl-4-[2-sulfanylprop-1-en-1-yl]-1H,2H,3H-cyclopenta[a]naphthalen-1-one

Peak: 13.80 - 13.83 min (scan #1897 - #1905) Subtracted noise: 13.74 - 13.77 min (scan #1881 - #1888), 13.83 - 13.86 min (scan #19

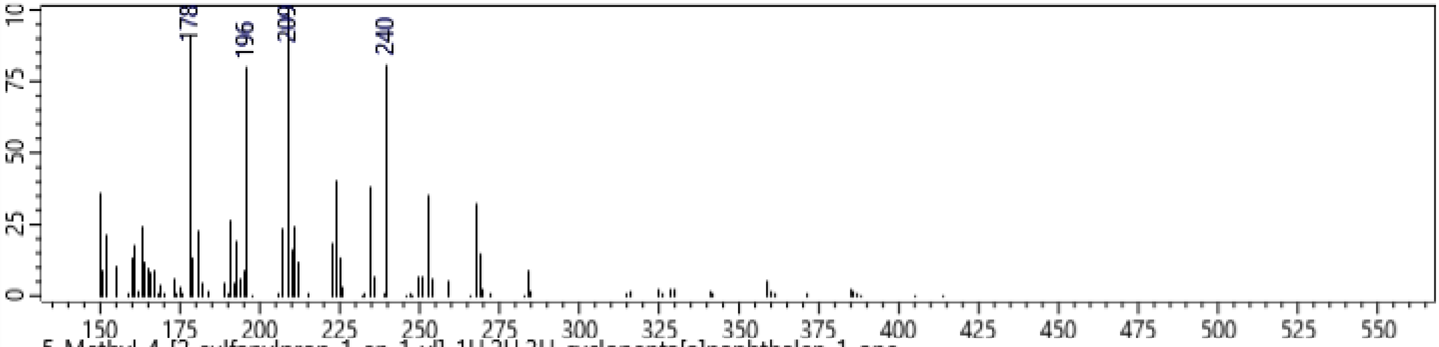

5-Methyl-4-[2-sulfanylprop-1-en-1-yl]-1H,2H,3H-cyclopenta[a]naphthalen-1-one

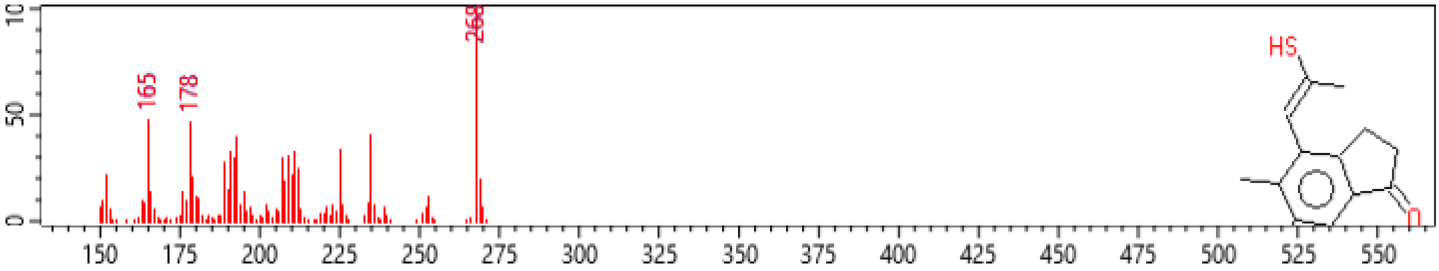

Estra-1,3,5,7,9-pentaene-3,17-diol, (17β)-

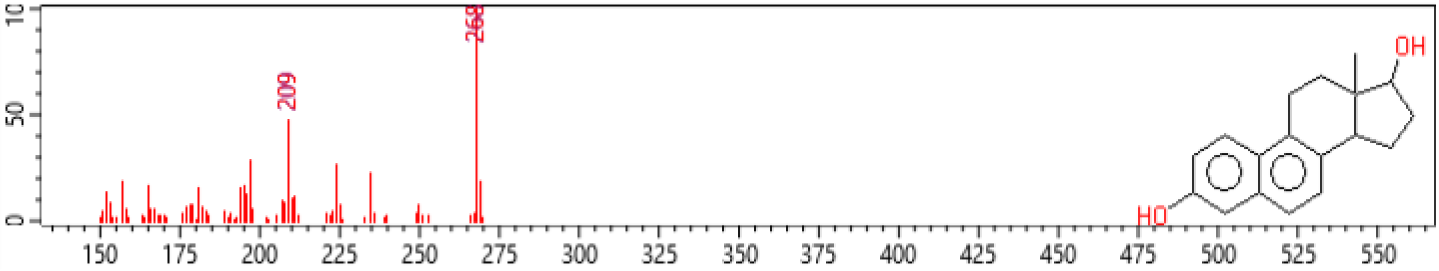

Estra-1,3,5,7,9-pentaene-3,17-diol, (17β)-

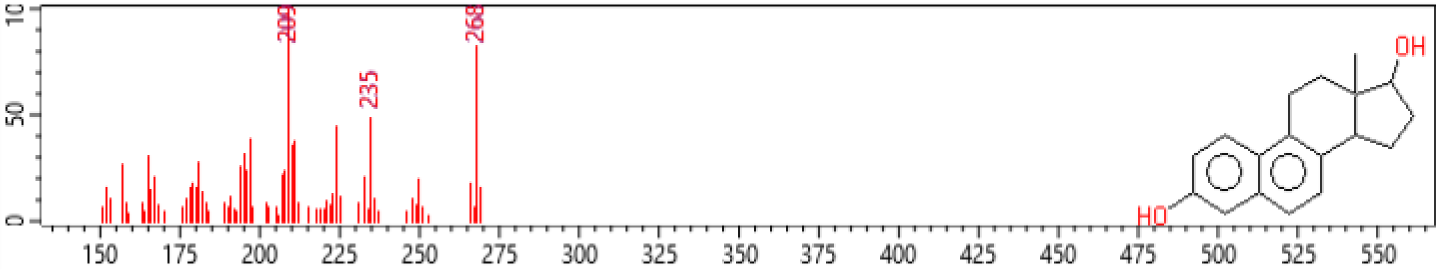

Estra-1,3,5(10),9(11)-tetraen-17-one, 3-hydroxy-

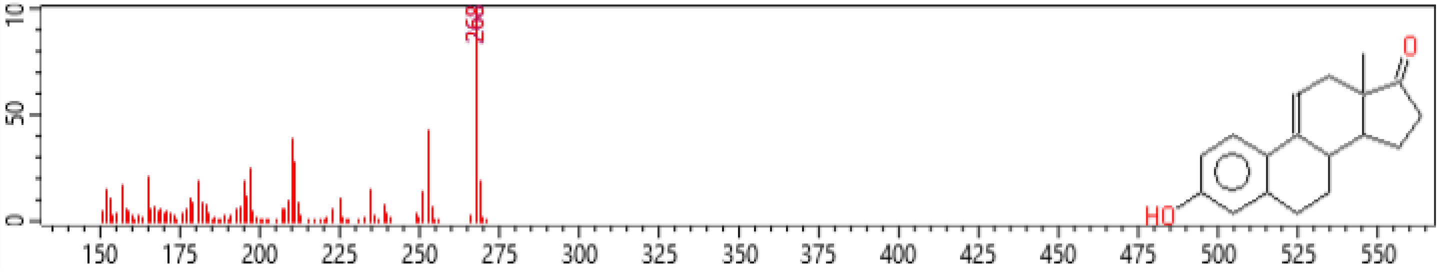

9,10-Anthracenedione, 1,8-dimethoxy-

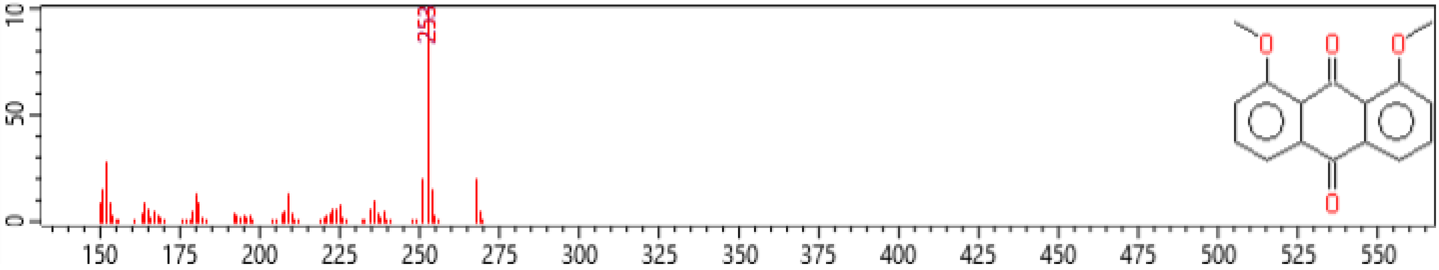

Isobenzofuran-4,7-imine-1,3-dione, hexahydro-8-[(4-methylphenyl)sulfonyl]-, (3aα,4α,7α,7aα)-

Peak: 13.83 - 13.86 min (scan #1906 - #1915) Subtracted noise: 13.79 - 13.83 min (scan #1896 - #1905), 13.91 - 13.93 min (scan #19

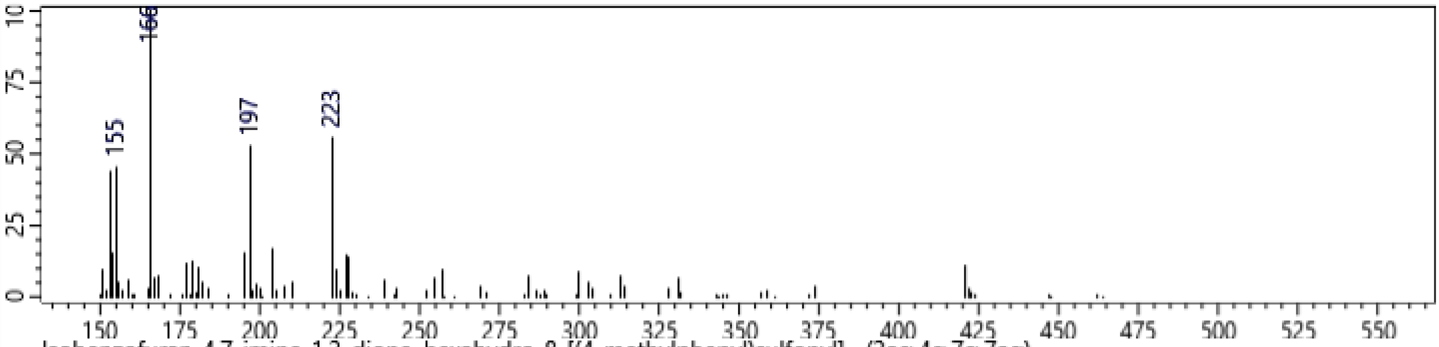

Isobenzofuran-4,7-imine-1,3-dione, hexahydro-8-[(4-methylphenyl)sulfonyl]-, (3aα,4α,7α,7aα)-

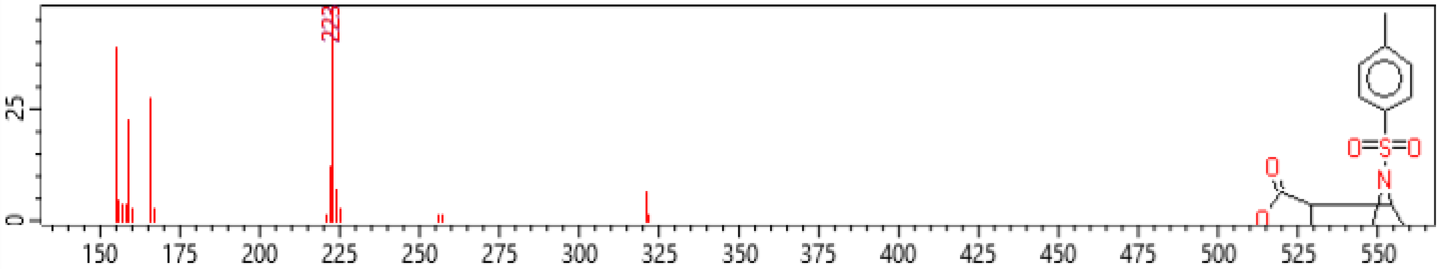

6-Ethyl-5-[1-piperidinyl]-2,4(1H,3H)-pyrimidinedione

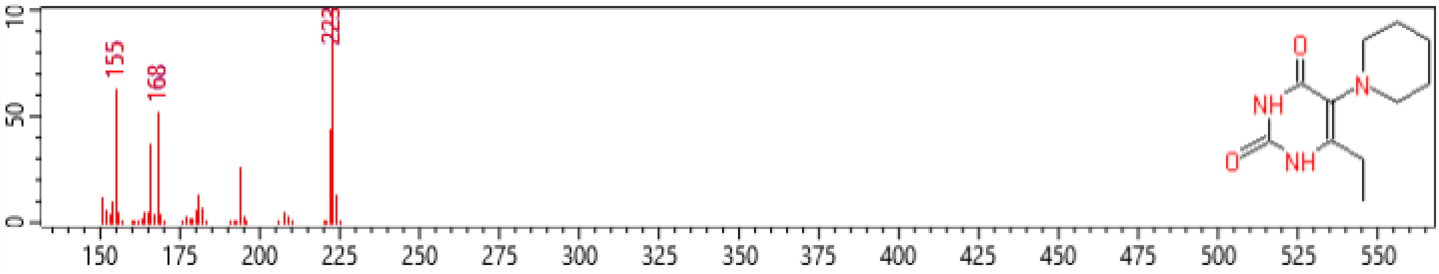

N-[5-(2,5-Dioxopyrrolidin-1-yl)pentyl]-N-hydroxyacetamide

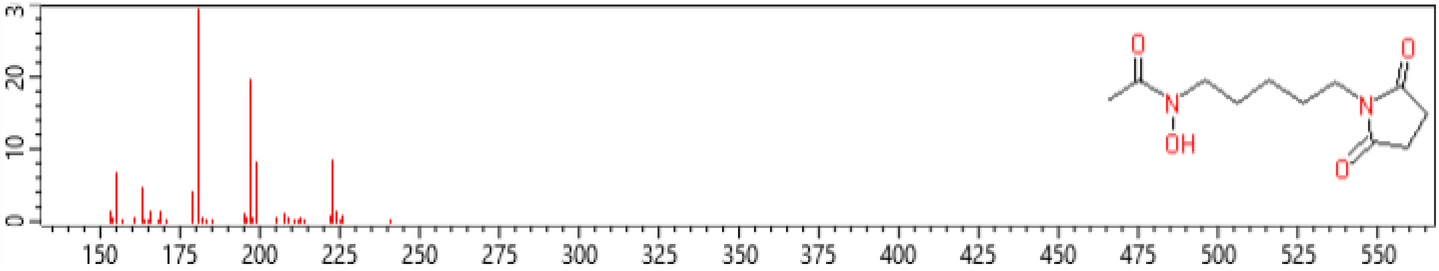

3-Chloro-2-(4-methyl-1-piperidinyl)aniline

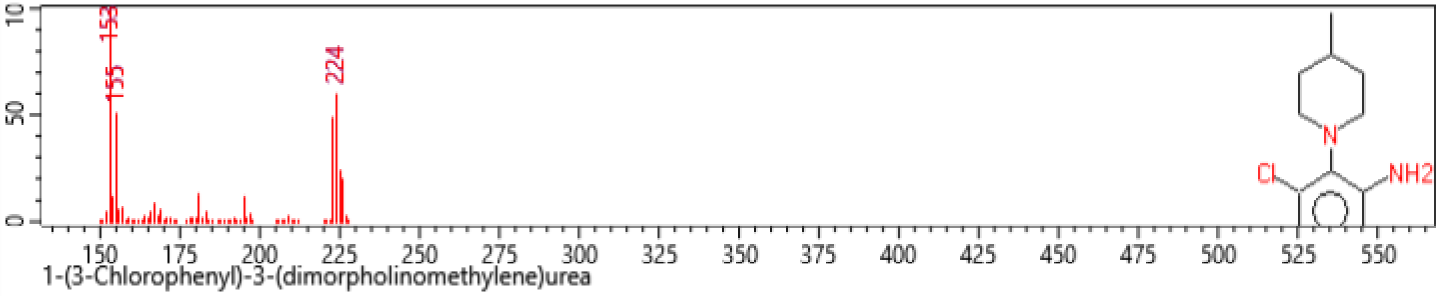

1-(3-Chlorophenyl)-3-(dimorpholinomethylene)urea

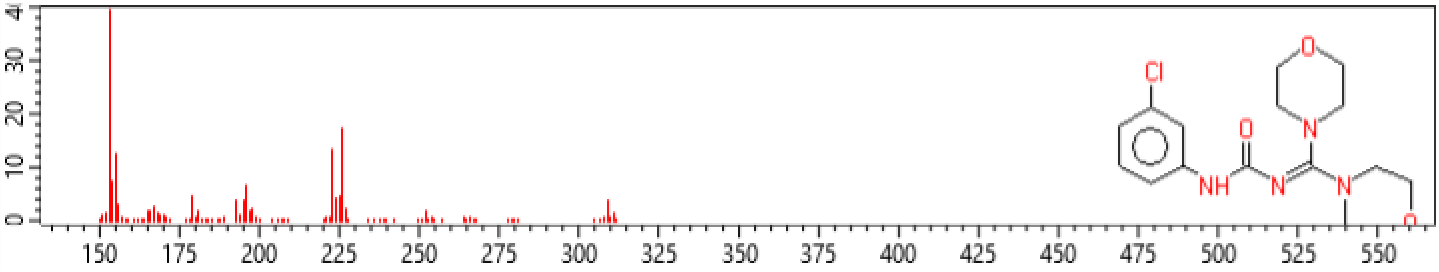

Citric acid, 4TMS derivative

Peak: 13.93 - 13.95 min (scan #1933 - #1939) Subtracted noise: 13.88 - 13.90 min (scan #1920 - #1926), 13.95 - 13.97 min (scan #19

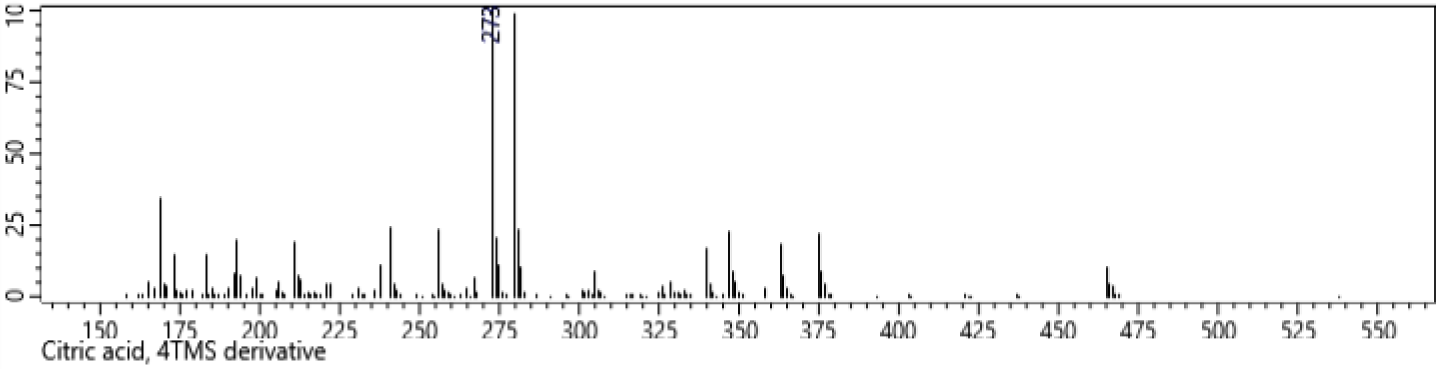

Citric acid, 4TMS derivative

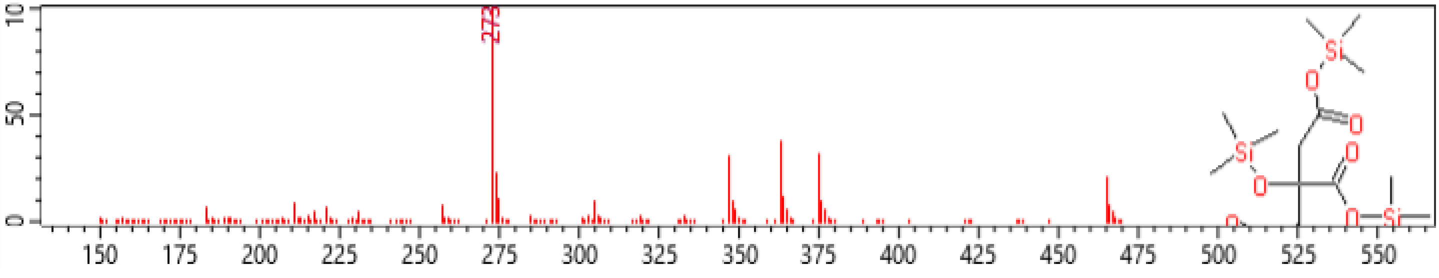

Citric acid, 4TMS derivative

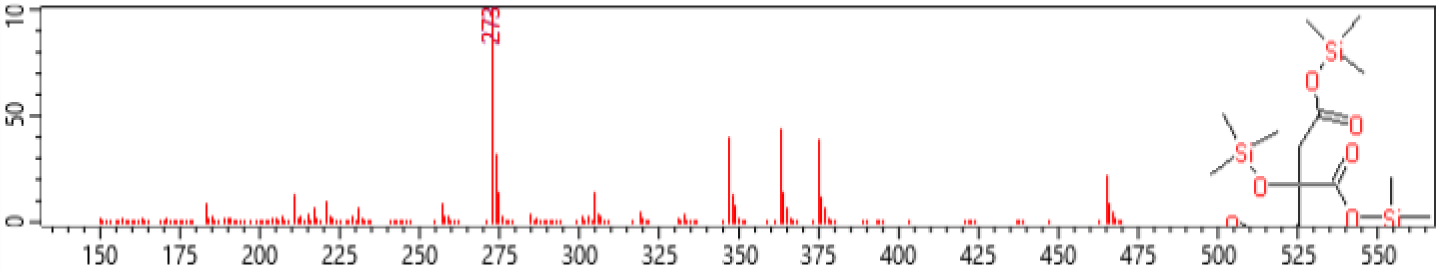

Citric acid, 4TMS derivative

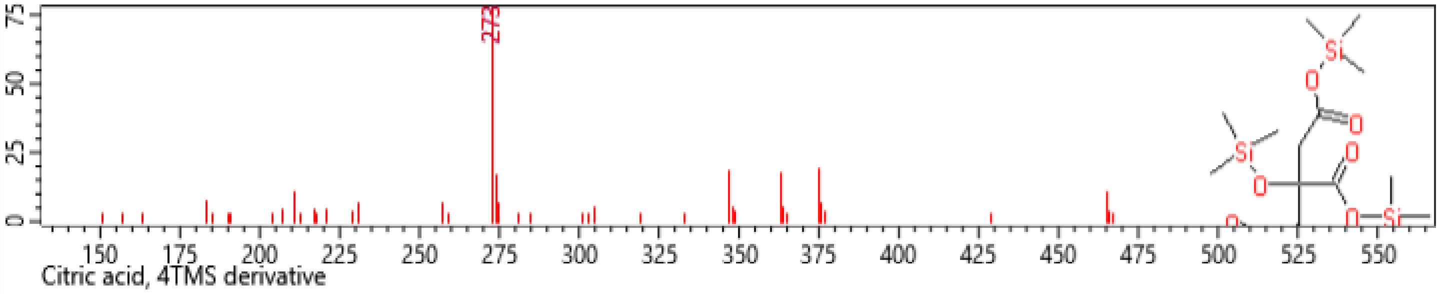

Citric acid, 4TMS derivative

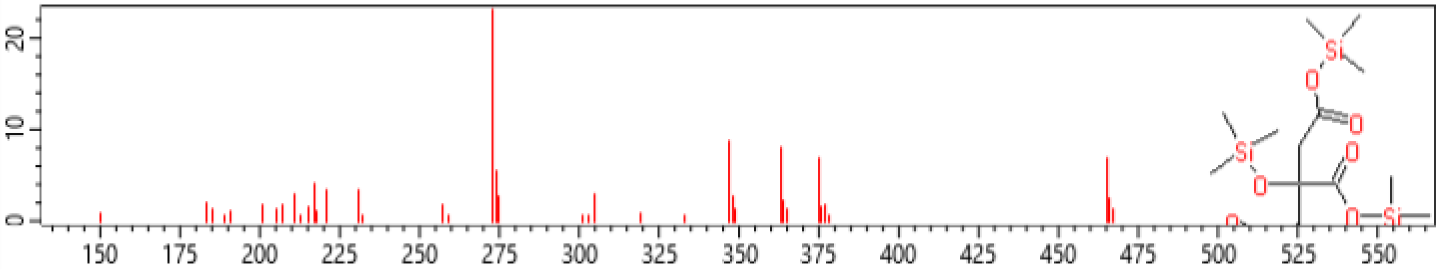

Citric acid, 4TMS derivative

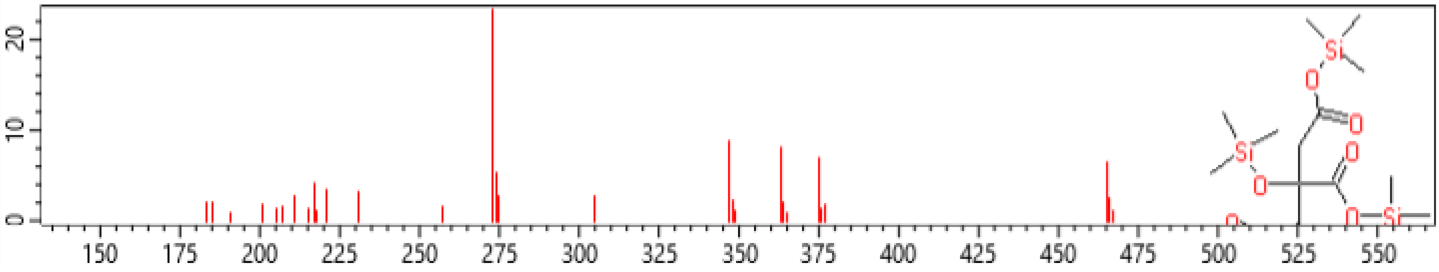

Citric acid, 4TMS derivative

Peak: 13.96 - 13.99 min (scan #1942 - #1950) Subtracted noise: 13.92 - 13.94 min (scan #1930 - #1938), 14.01 - 14.05 min (scan #19

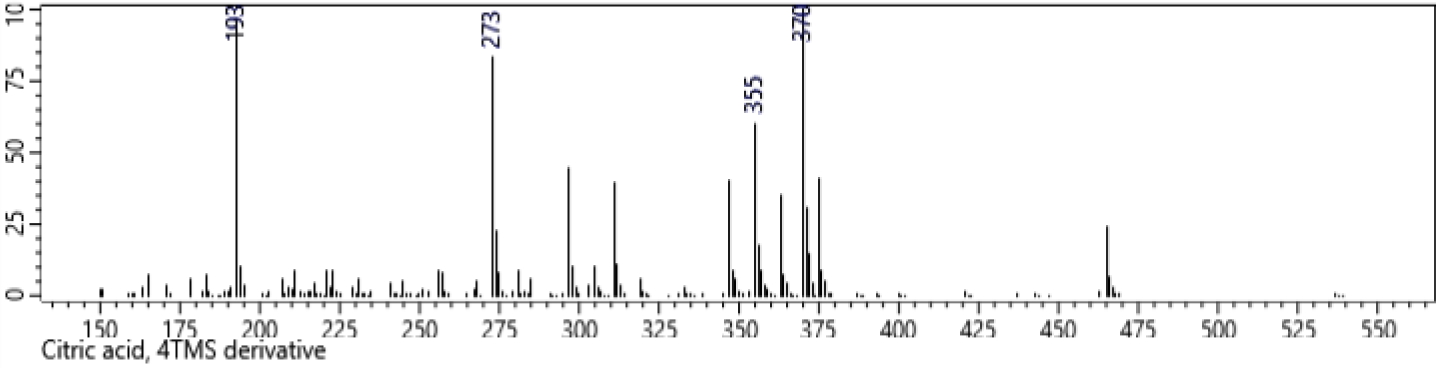

Citric acid, 4TMS derivative

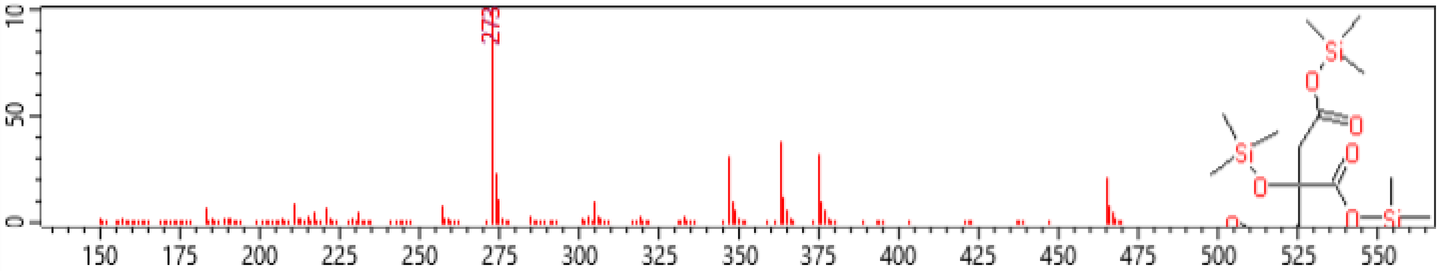

Citric acid, 4TMS derivative

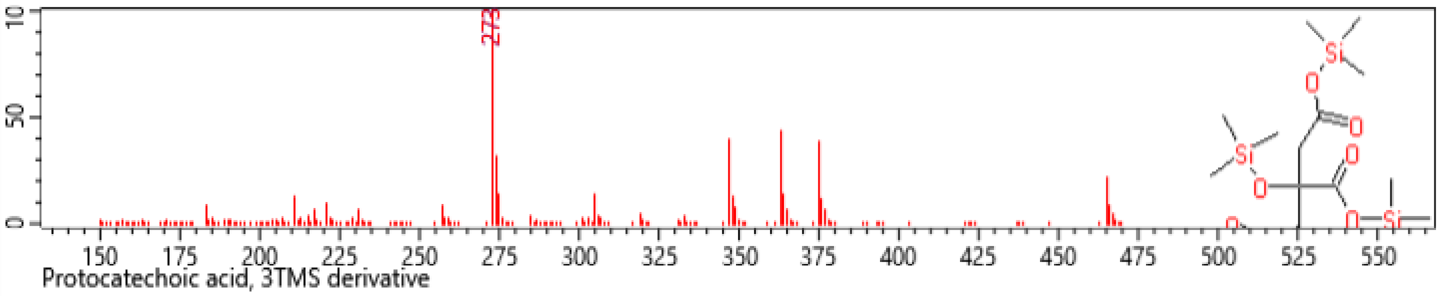

Protocatechoic acid, 3TMS derivative

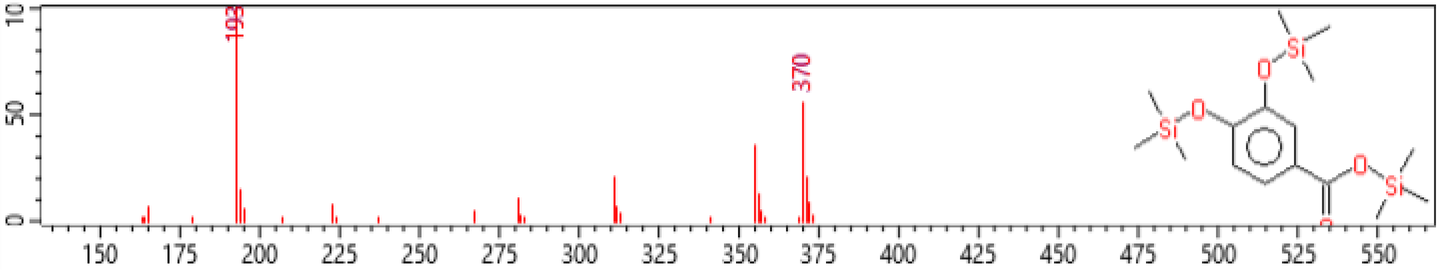

Protocatechoic acid, 3TMS derivative

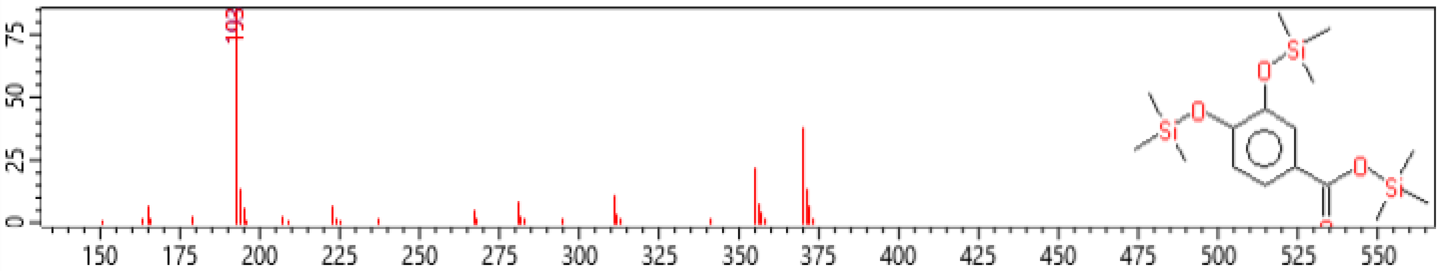

Protocatechoic acid, 3TMS derivative

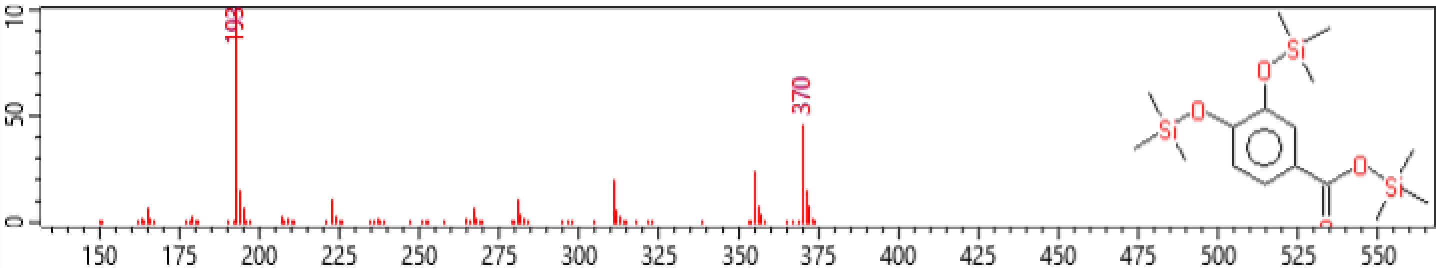

Tranexamic acid, 2TBDMS derivative

Peak: 14.04 - 14.07 min (scan #1964 - #1974) Subtracted noise: 13.98 - 14.01 min (scan #1947 - #1956), 14.09 - 14.13 min (scan #19

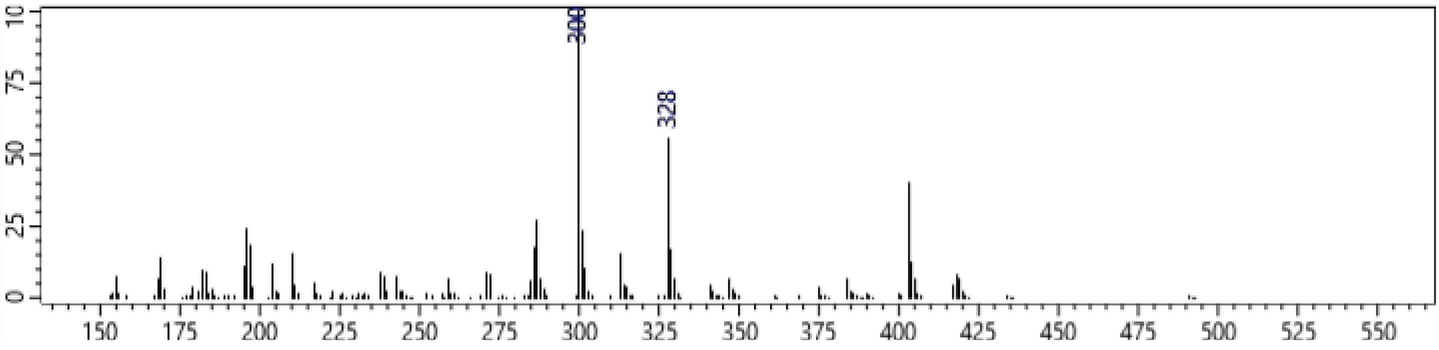

Tranexamic acid, 2TBDMS derivative

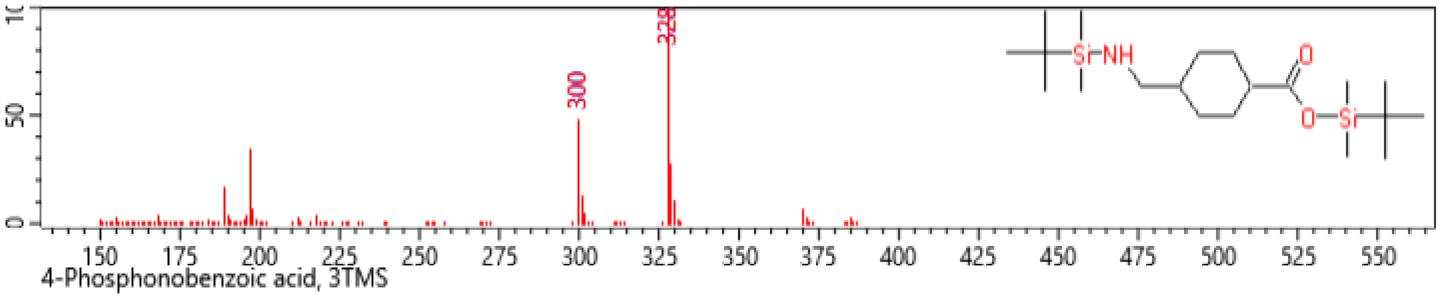

4-Phosphonobenzoic acid, 3TMS

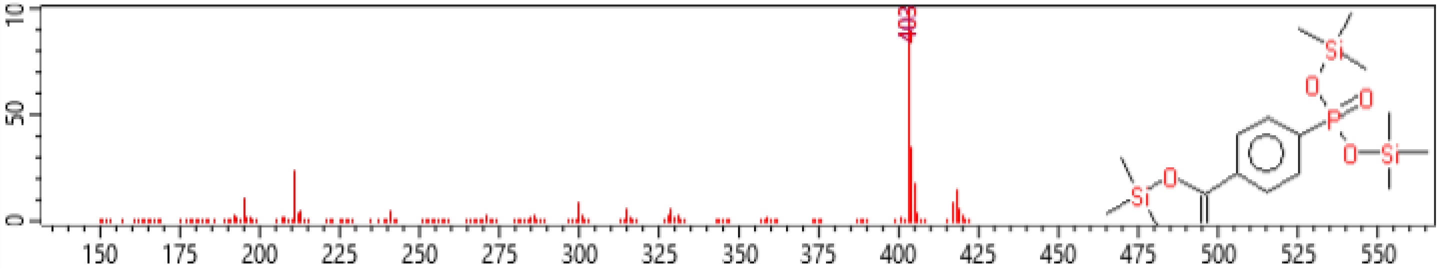

6-Methoxyluteolin

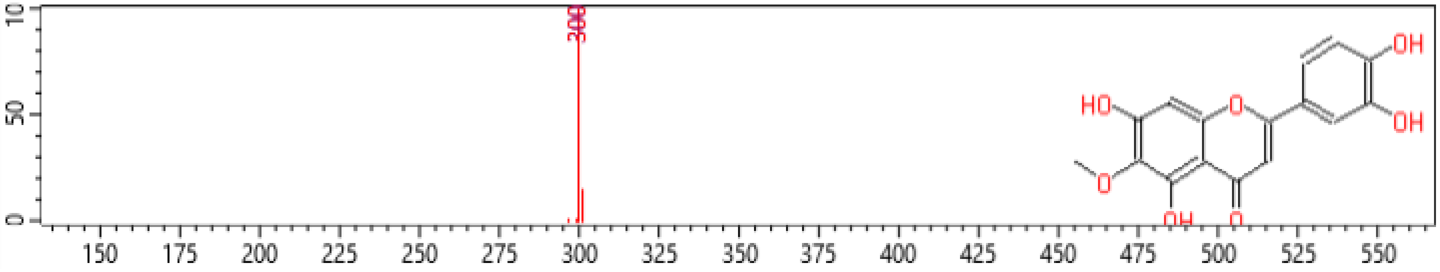

Tranexamic acid, 2TBDMS derivative

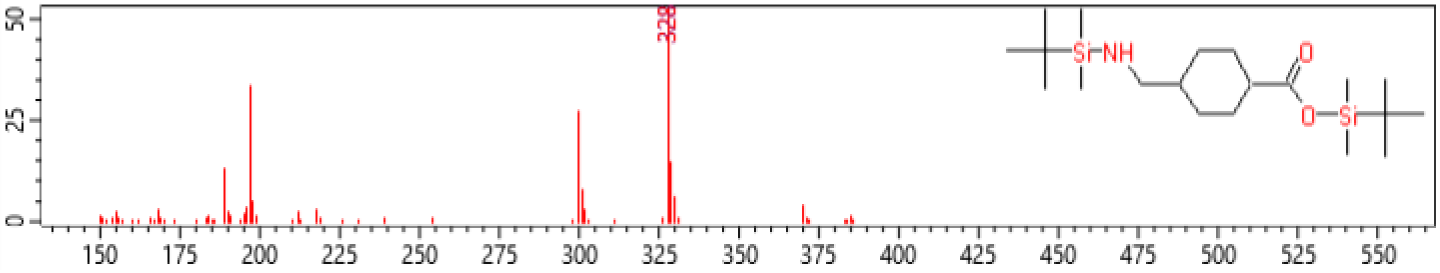

Colchiceine, 7-deacetoamino-

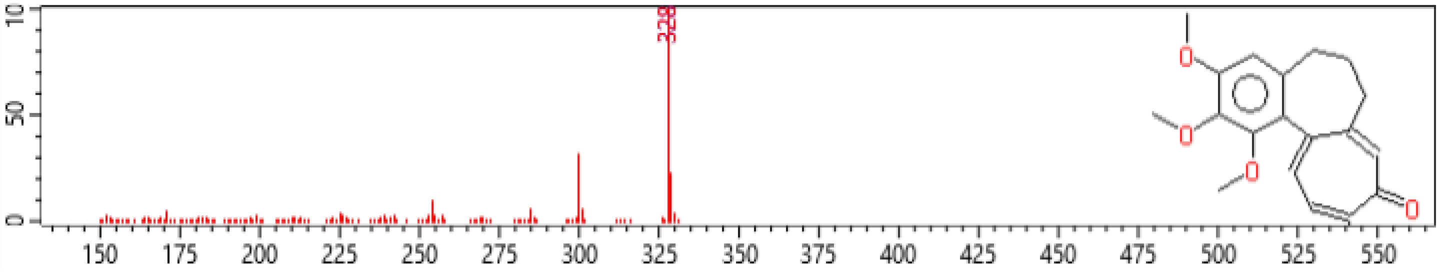

1-Methyl-3,4-bis(indol-3-yl)maleimide

Peak: 14.15 - 14.20 min (scan #1996 - #2008) Subtracted noise: 14.05 - 14.09 min (scan #1967 - #1978), 14.19 - 14.23 min (scan #20

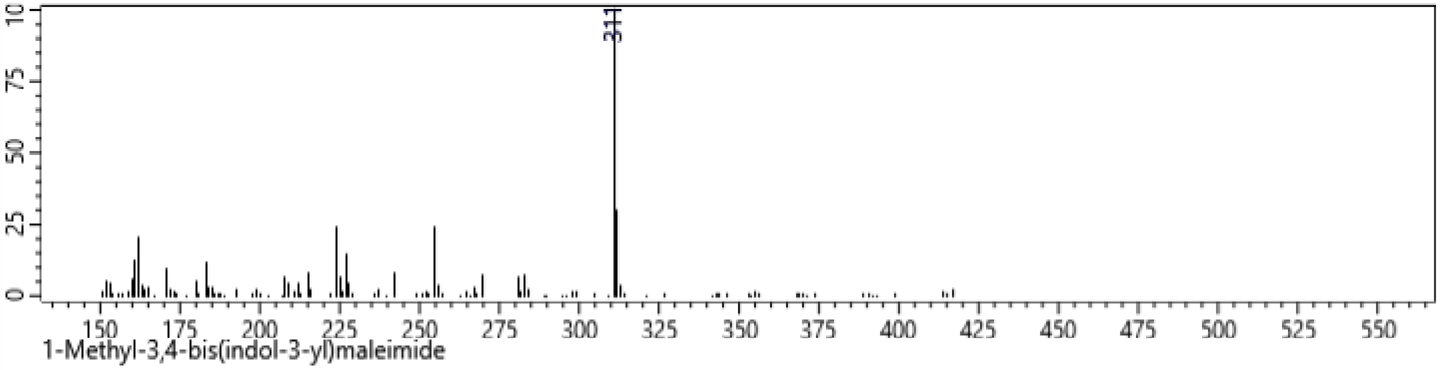

1-Methyl-3,4-bis(indol-3-yl)maleimide

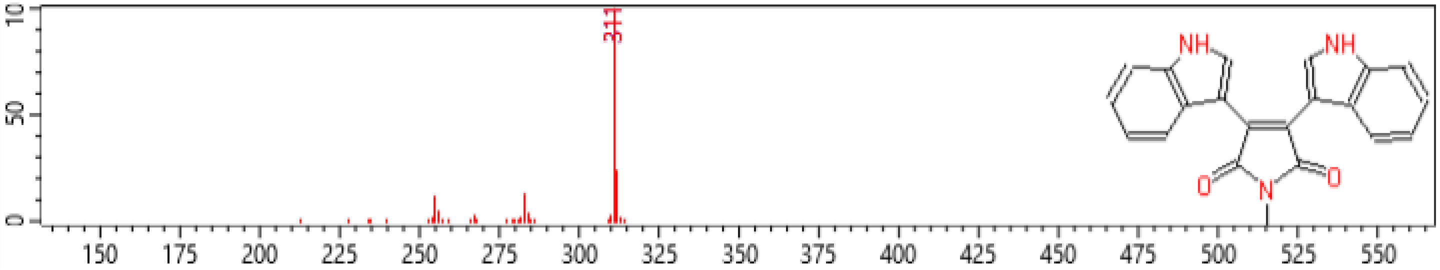

1-Methyl-3,4-bis(indol-3-yl)maleimide

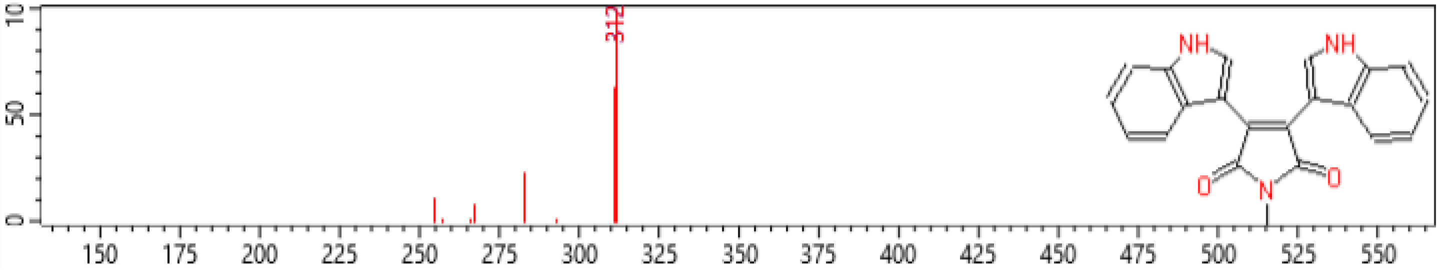

Unguinol

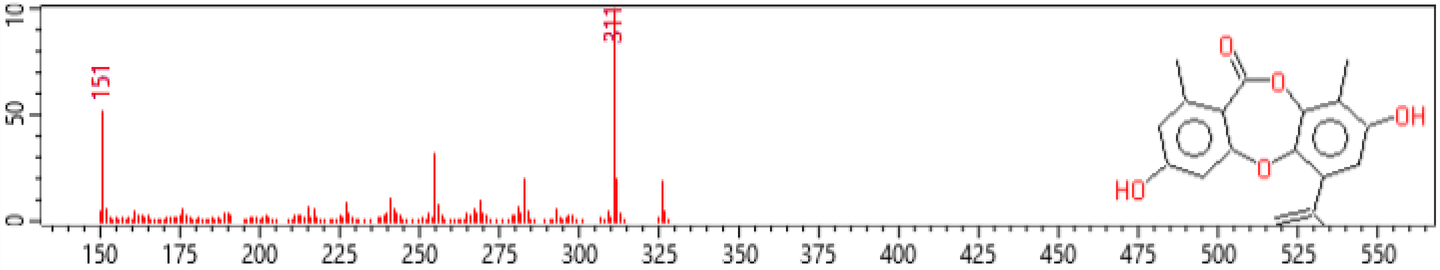

1-Dimethyl(pentafluorophenyl)silyloxyhexane

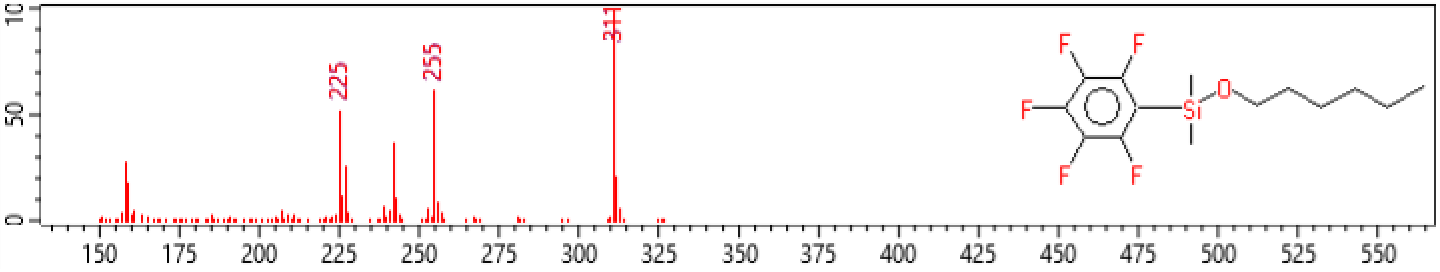

Pyrido[2,3-j]indole-2-carboxylic acid, 5-methoxy-3,7,8-trimethyl-, ethyl ester

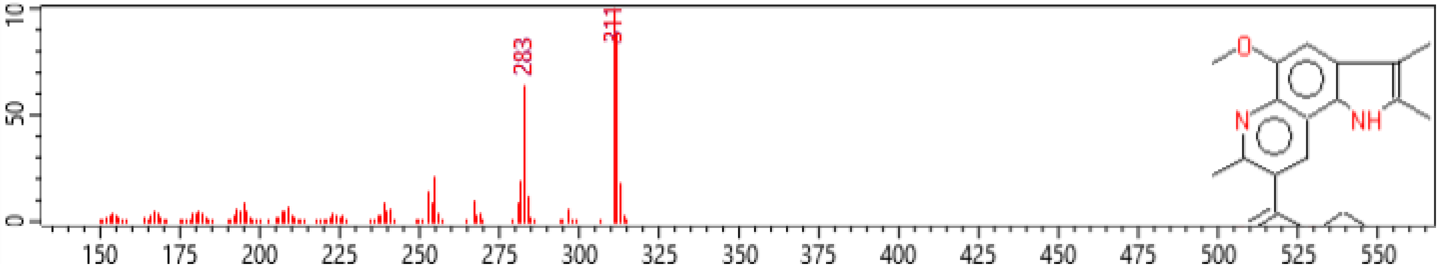

Gibbane-1,10-dicarboxylic acid, 2,4a-dihydroxy-1-methyl-8-methylene-, 1,4a-lactone, (1α,2β,4aα,4bβ,10β)-

Peak: 14.20 - 14.20 min (scan #2008 - #2010) Subtracted noise: 14.17 - 14.18 min (scan #2002 - #2004), 14.21 - 14.22 min (scan #20

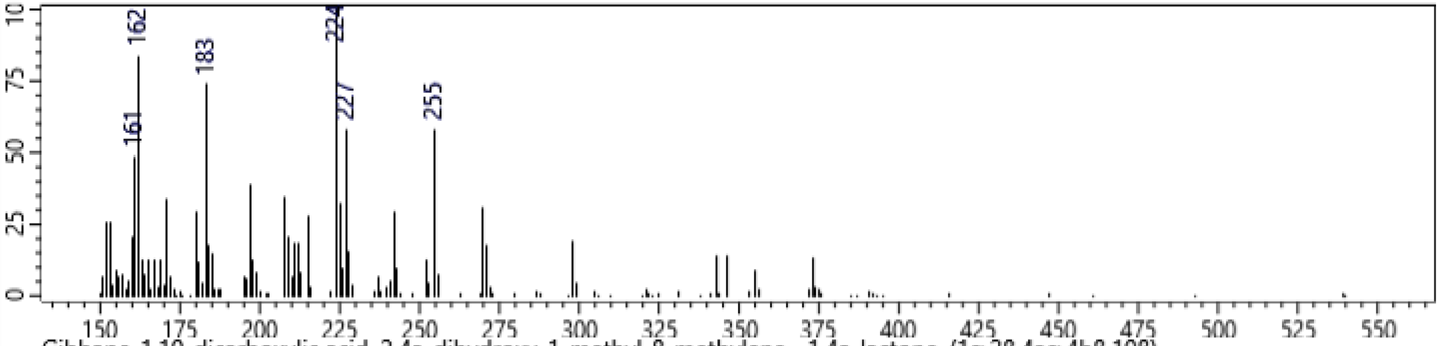

Gibbane-1,10-dicarboxylic acid, 2,4a-dihydroxy-1-methyl-8-methylene-, 1,4a-lactone, (1α,2β,4aα,4bβ,10β)-

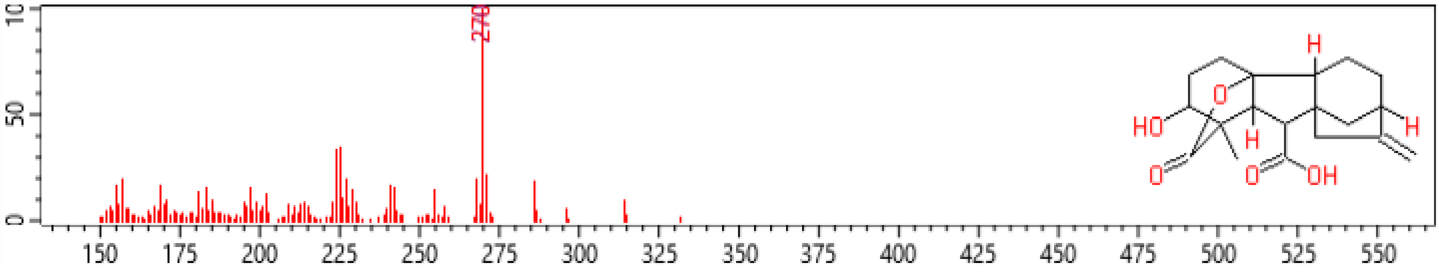

1-Cyclohexyl-3-ethoxy-3-(trimethylsilyl)oxyprop-2-en-1-one (isomer 2)

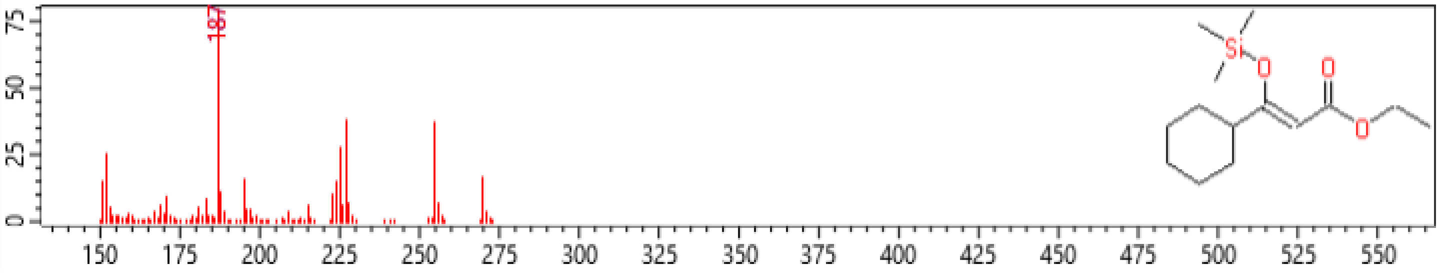

3',4',6-Trimethoxyisoflavone-7-O-β-D-glucopyranoside

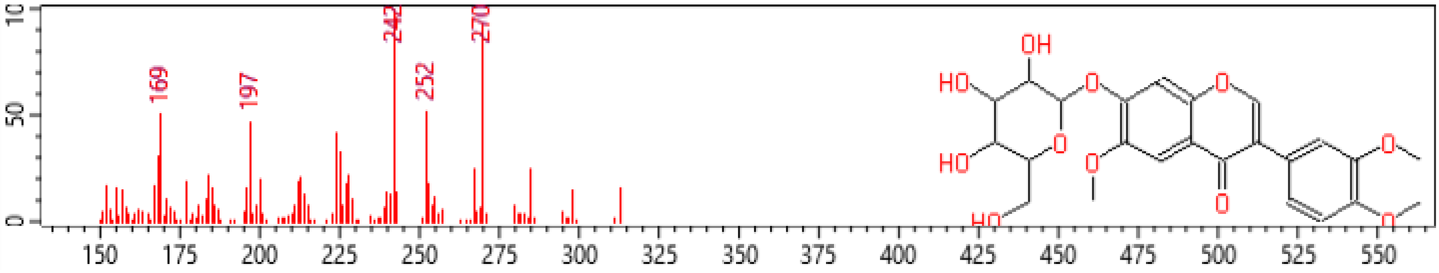

1-Cyclohexyl-3-ethoxy-3-(trimethylsilyl)oxyprop-2-en-1-one (isomer 1)

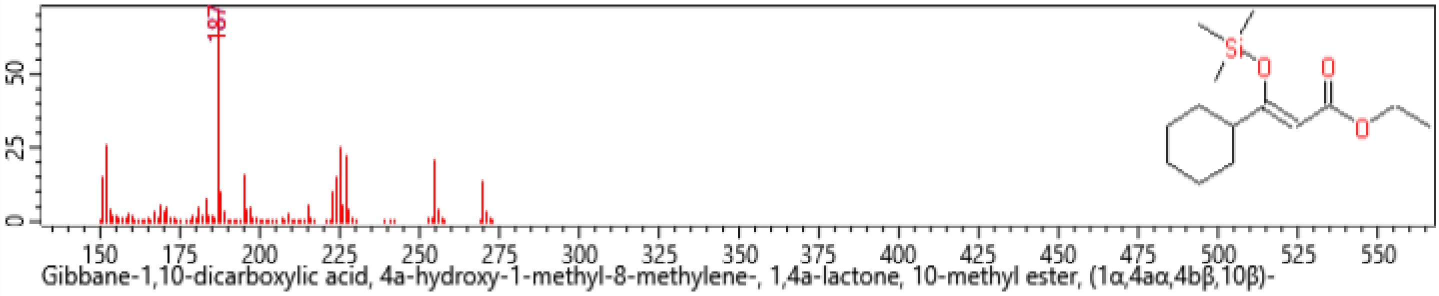

Gibbane-1,10-dicarboxylic acid, 4a-hydroxy-1-methyl-8-methylene-, 1,4a-lactone, 10-methyl ester, (1α,4aα,4bβ,10β)-

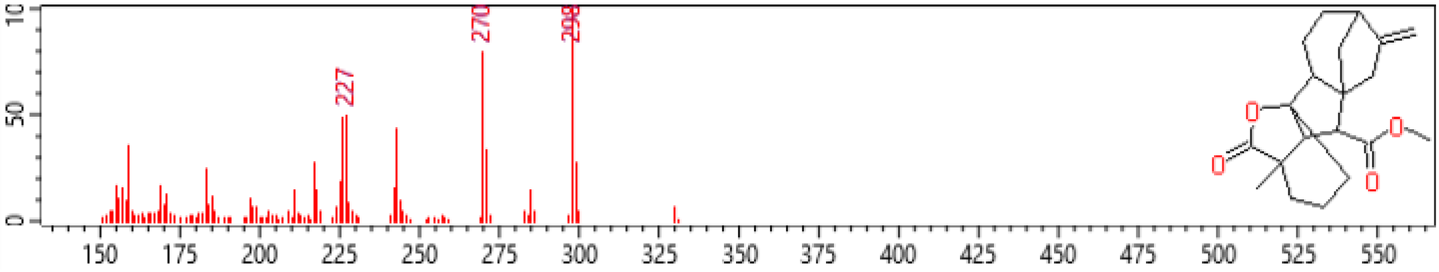

Quininic acid (5TMS)

Peak: 14.20 - 14.23 min (scan #2010 - #2018) Subtracted noise: 14.18 - 14.21 min (scan #2004 - #2011), 14.27 - 14.30 min (scan #20

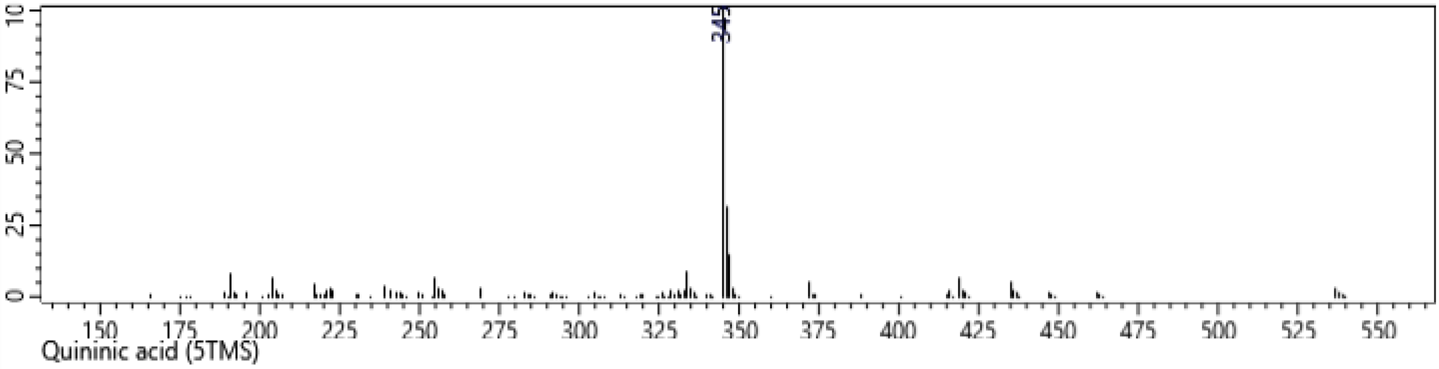

Quininic acid (5TMS)

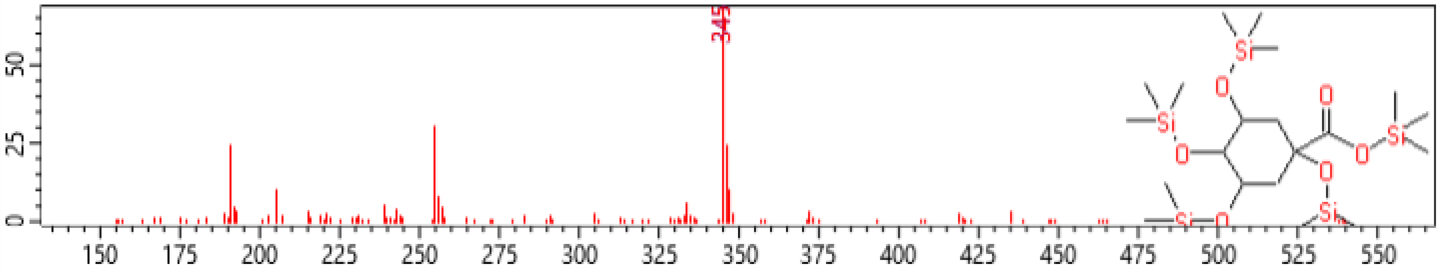

Rhodamine 123 cation

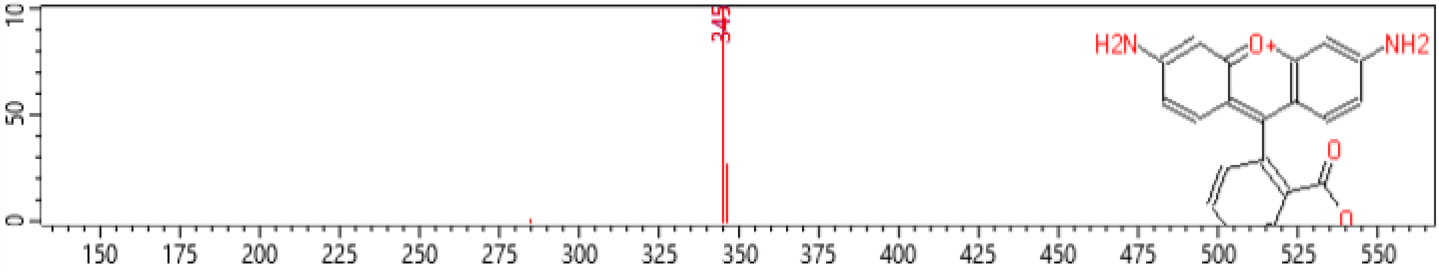

5-O-Coumaroyl-D-quinic acid, 5TMS, (1R,3R,4S,5R)-

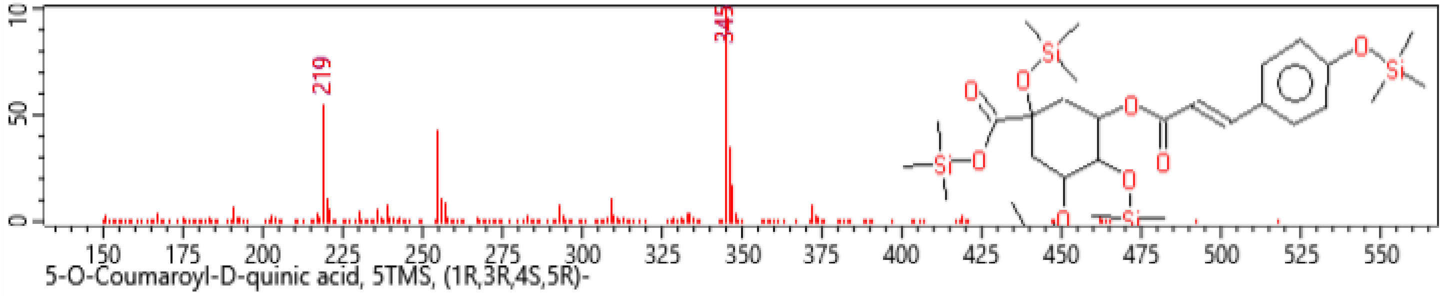

5-O-Coumaroyl-D-quinic acid, 5TMS, (1R,3R,4S,5R)-

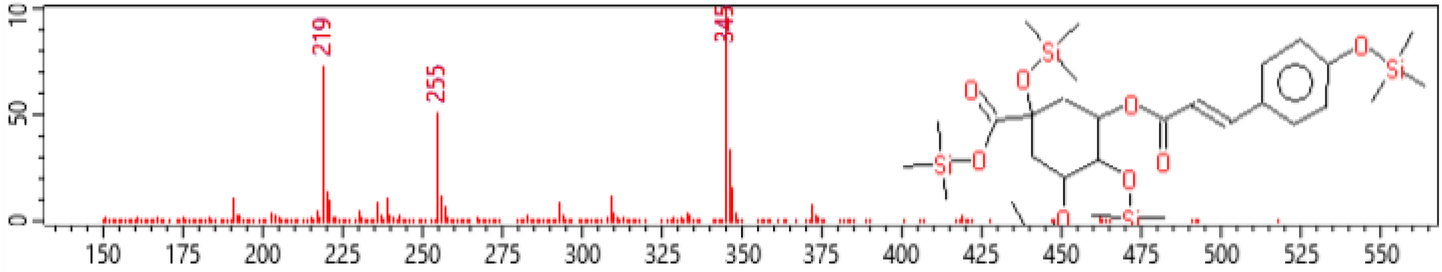

3-O-Coumaroyl-D-quinic acid, 5TMS, (1S,3R,4R,5R)

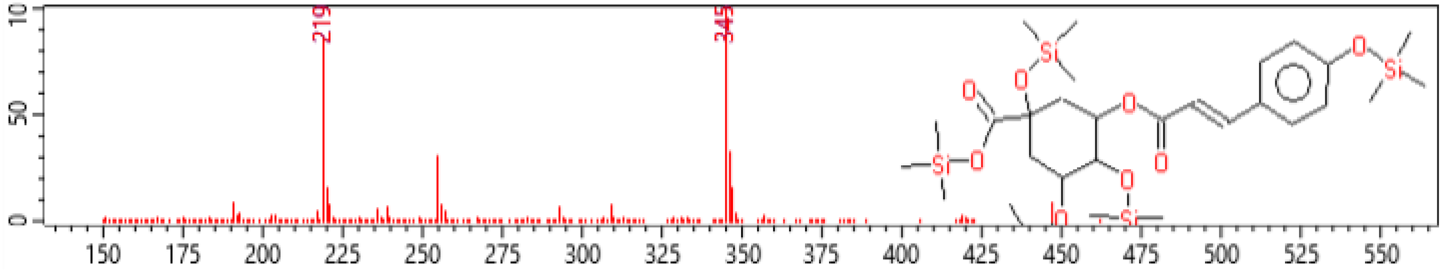

3,4-Dihydroxyphenylglycol, 4TMS derivative

Peak: 14.30 - 14.34 min (scan #2038 - #2048) Subtracted noise: 14.24 - 14.27 min (scan #2019 - #2028), 14.35 - 14.38 min (scan #20

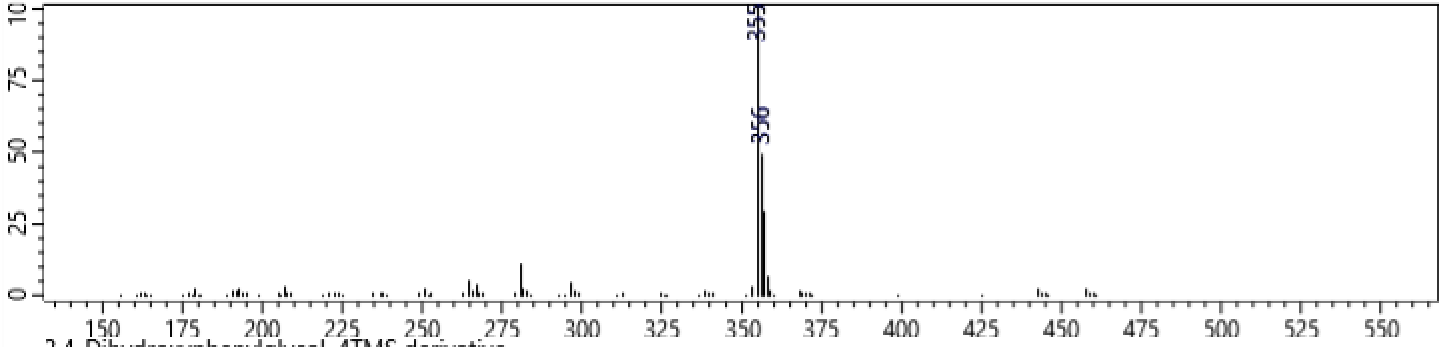

3,4-Dihydroxyphenylglycol, 4TMS derivative

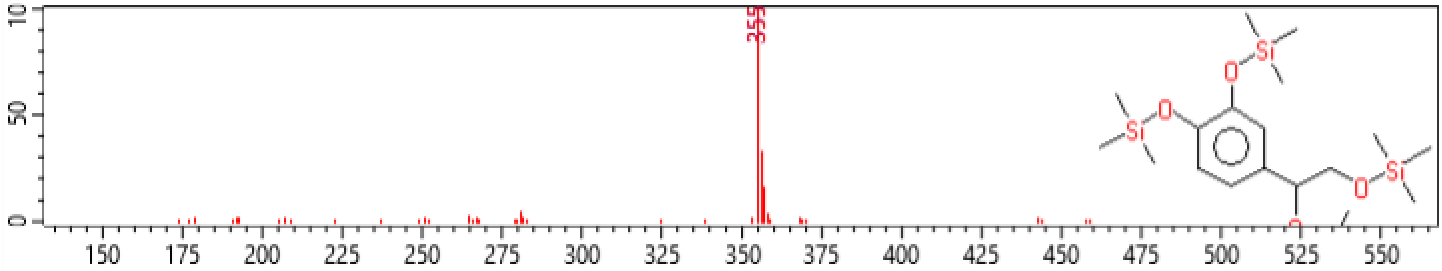

3,4-Dihydroxyphenylglycol, 4TMS derivative

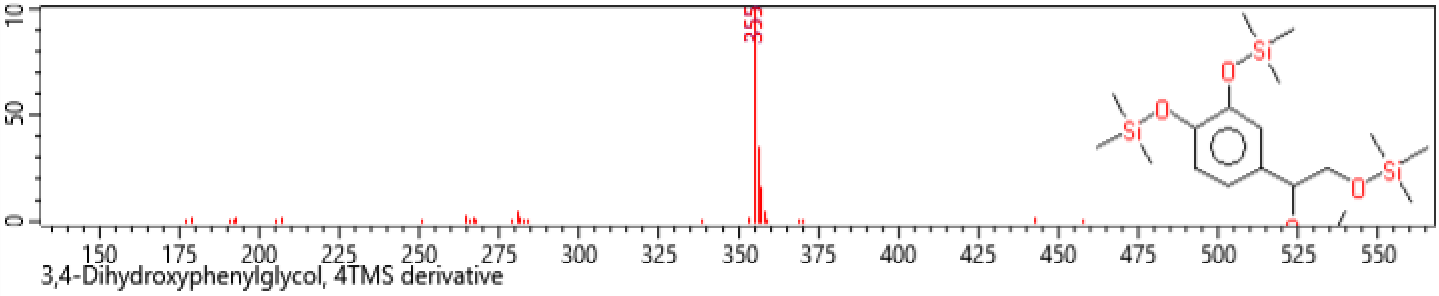

3,4-Dihydroxyphenylglycol, 4TMS derivative

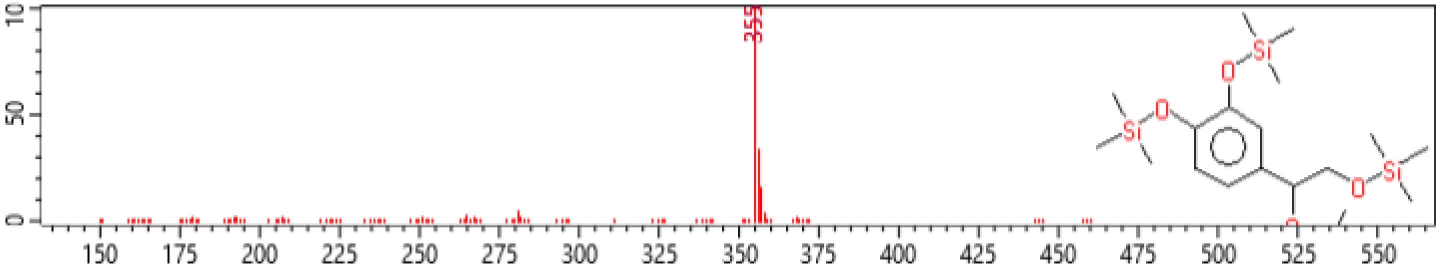

3,4-Dihydroxyphenylglycol, 4TMS derivative

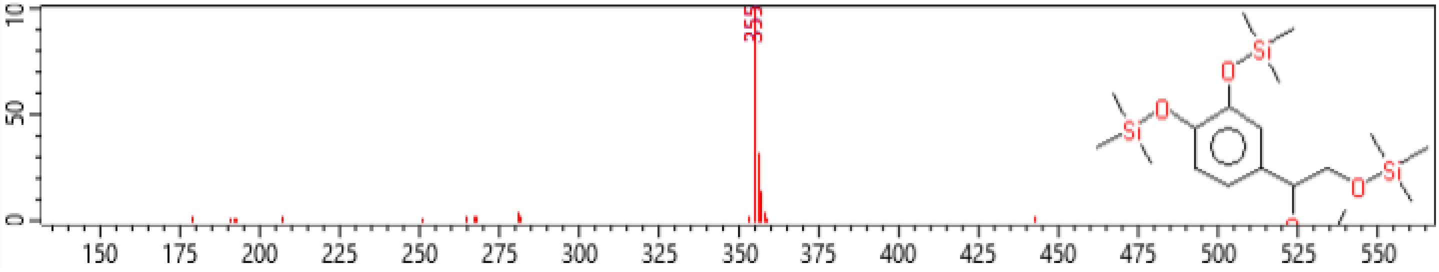

N-(Trifluoracetyl)-O,O',O''-tris(trimethylsilyl)epinephrine

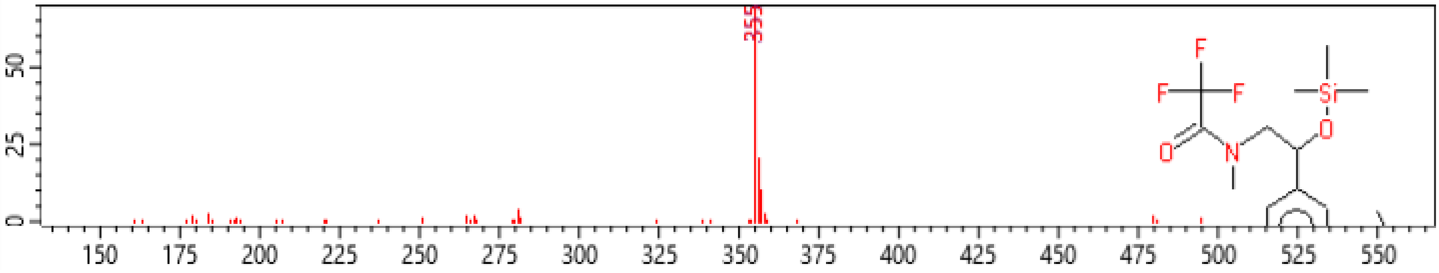

Labdanolic acid

Peak: 14.37 - 14.40 min (scan #2056 - #2064) Subtracted noise: 14.32 - 14.35 min (scan #2043 - #2050), 14.40 - 14.43 min (scan #20

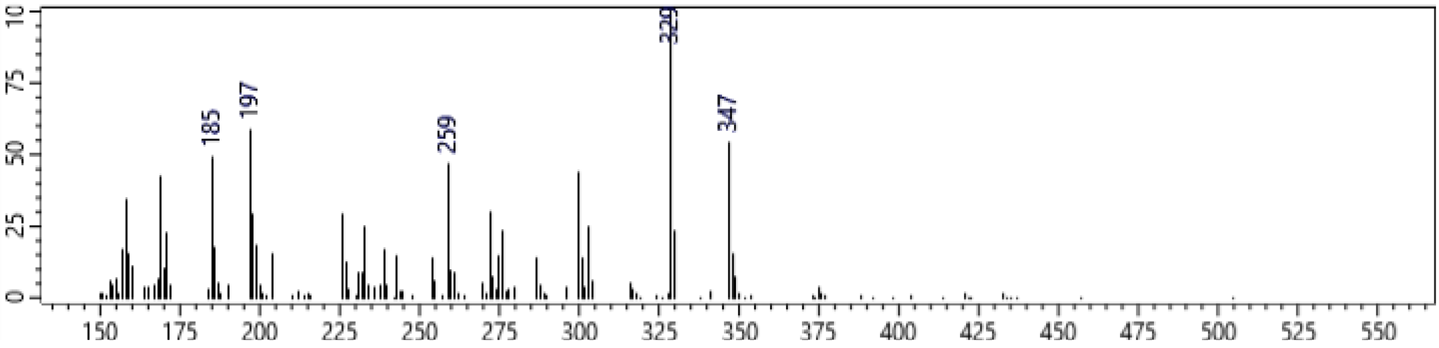

Labdanolic acid

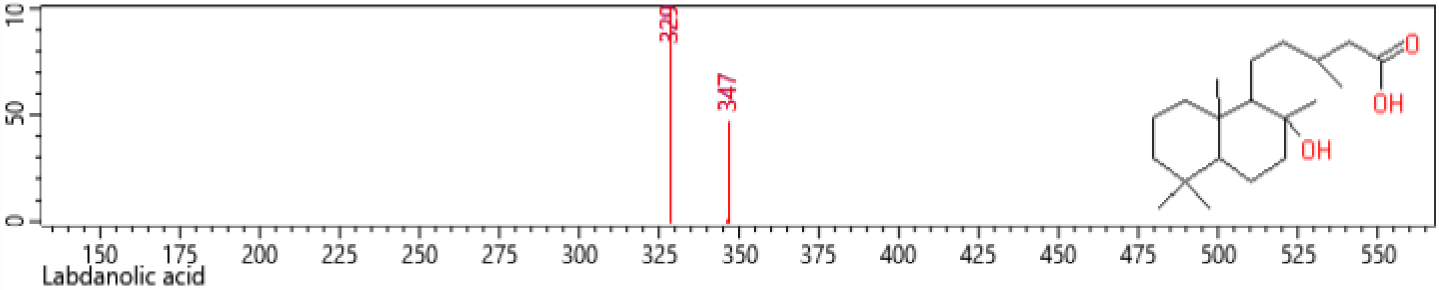

Labdanolic acid

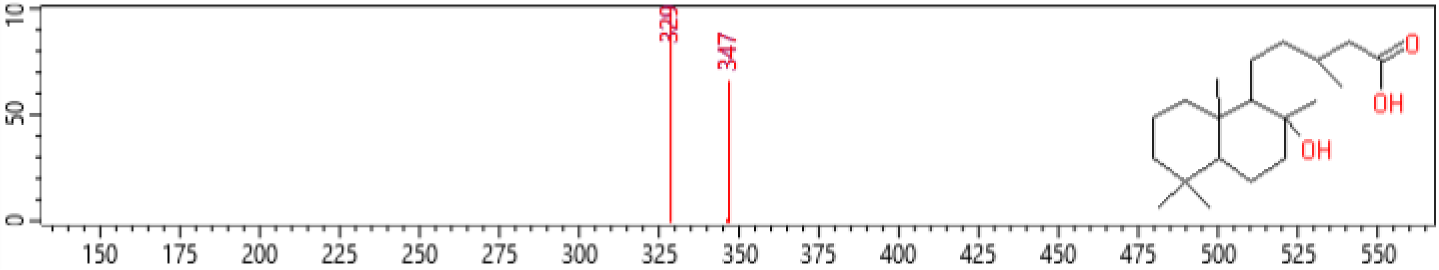

Labdanolic acid

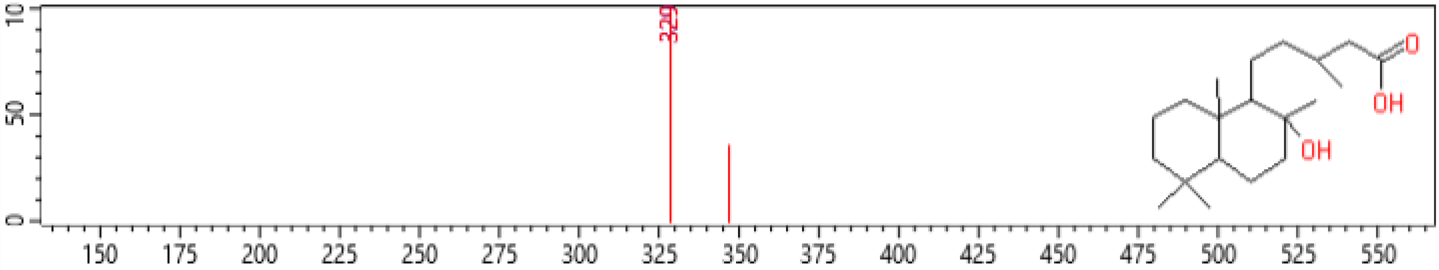

Labdanolic acid

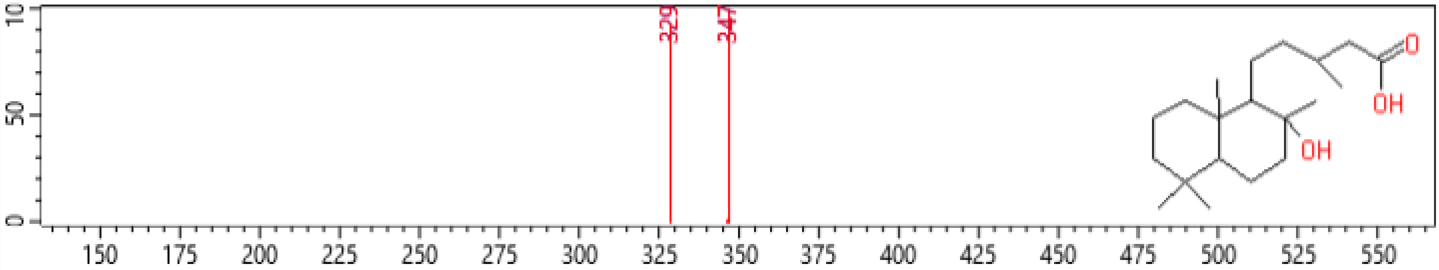

Labdanolic acid

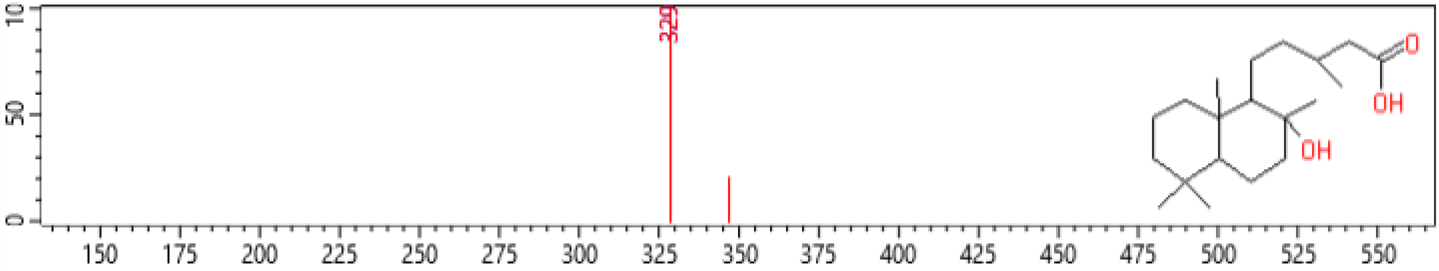

Syringaresinol diglucoside

Peak: 14.44 - 14.48 min (scan #2076 - #2086) Subtracted noise: 14.36 - 14.40 min (scan #2055 - #2065), 14.49 - 14.53 min (scan #20

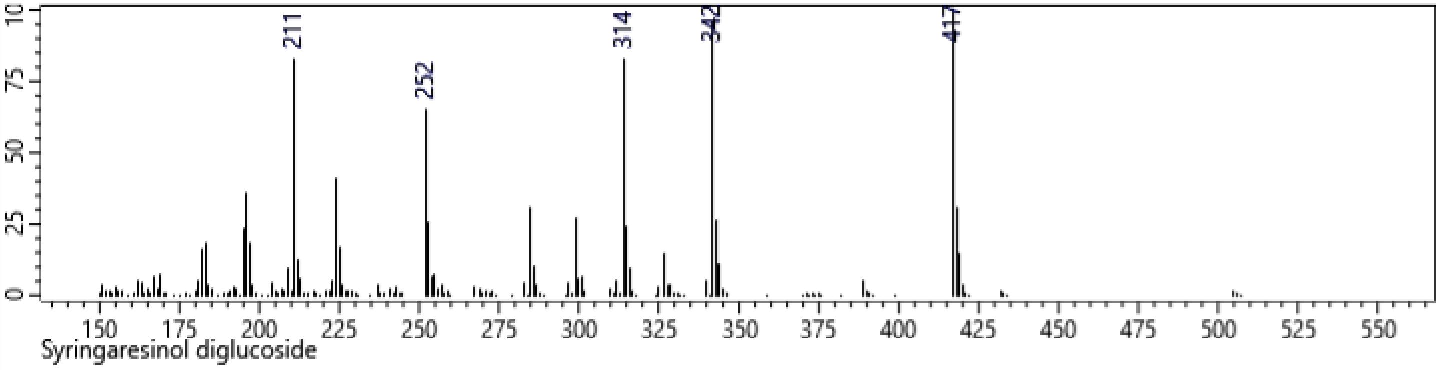

Syringaresinol diglucoside

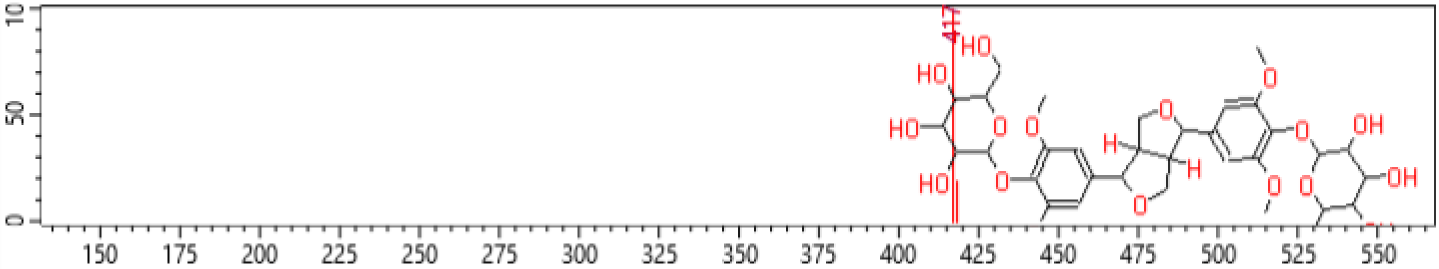

Acetic acid, 2-[[(2Z)-4-[(1R,2R,3R,5R)-5-chloro-2-[(3R)-3-cyclohexyl-3-hydroxypropyl]-3-hydroxycyclopentyl]-2-buten-1-yl]oxy]-, 1-m

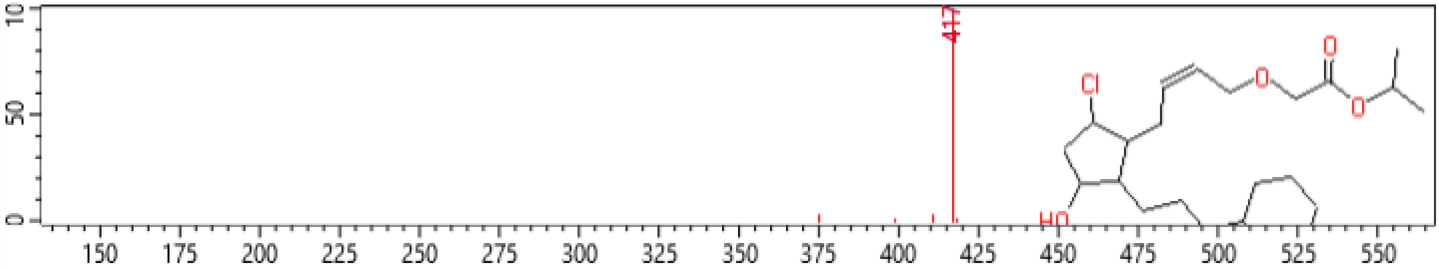

Ac-Asp-Tyr(2-malonyl)-Val-Pro-Met-Leu-NH2

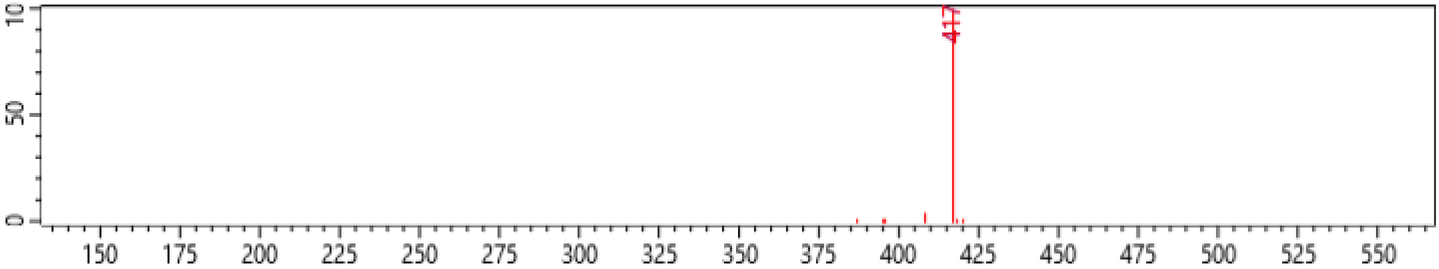

7-Deacetamidocolchicine

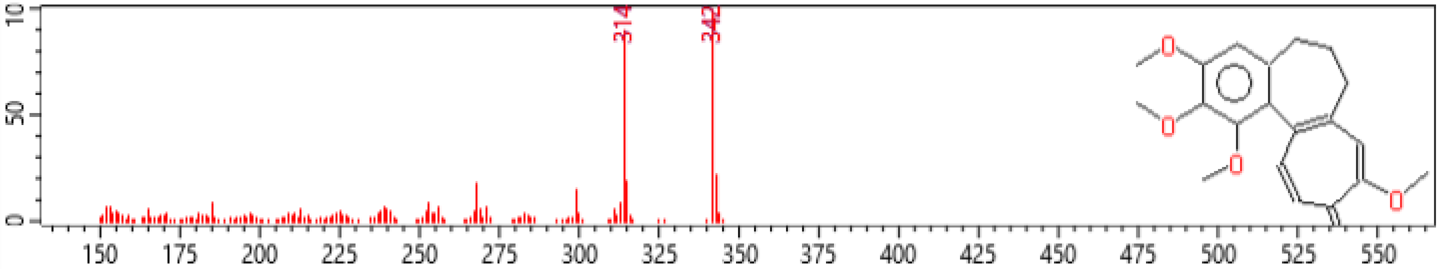

Gomisin A

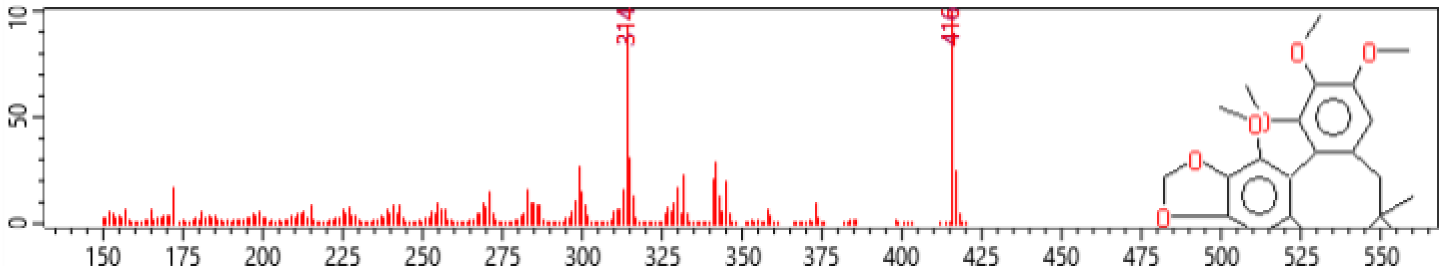

4-Hydroxyphenyllactic acid, 3TMS derivative

Peak: 14.50 - 14.51 min (scan #2092 - #2096) Subtracted noise: 14.47 - 14.48 min (scan #2085 - #2088), 14.52 - 14.53 min (scan #20[illegible]

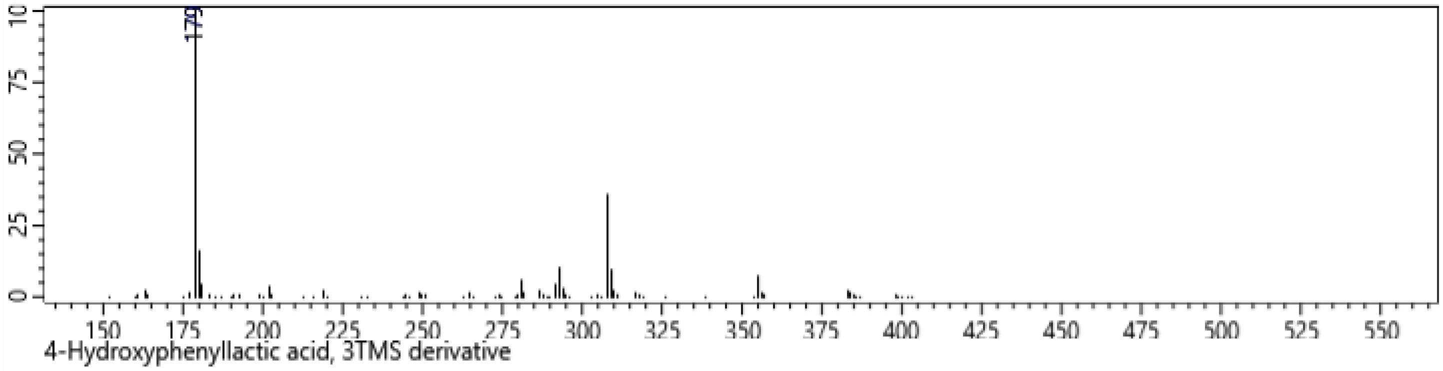

4-Hydroxyphenyllactic acid, 3TMS derivative

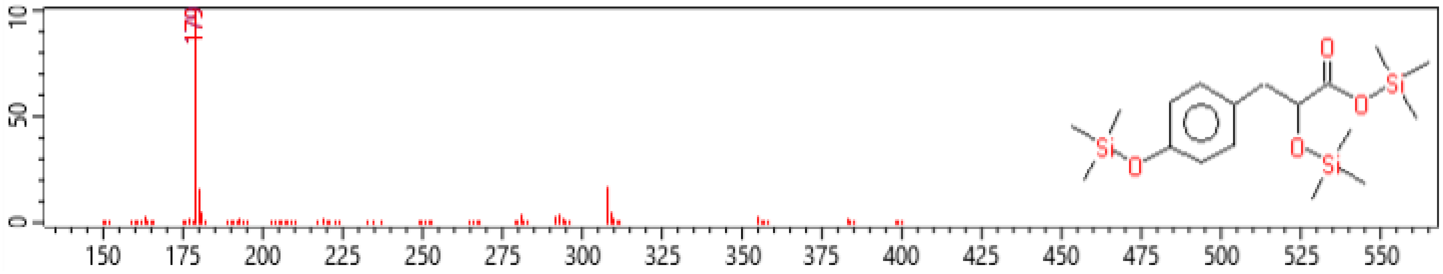

4-Hydroxyphenyllactic acid, 3TMS derivative

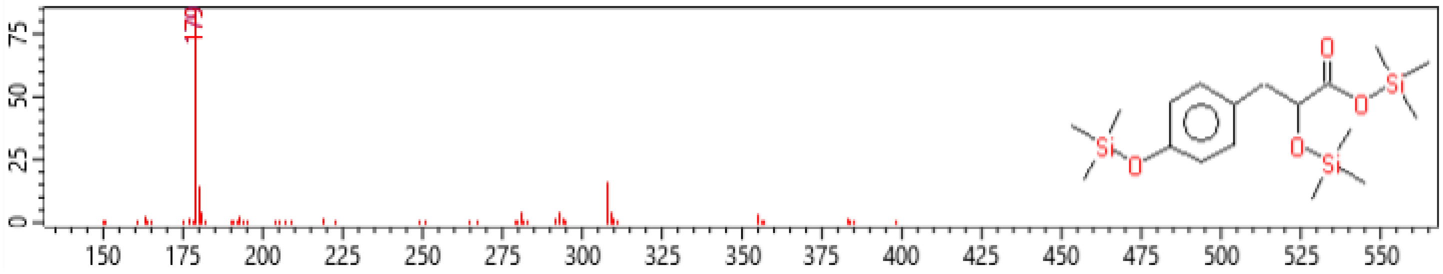

4-Hydroxyphenyllactic acid, 3TMS derivative

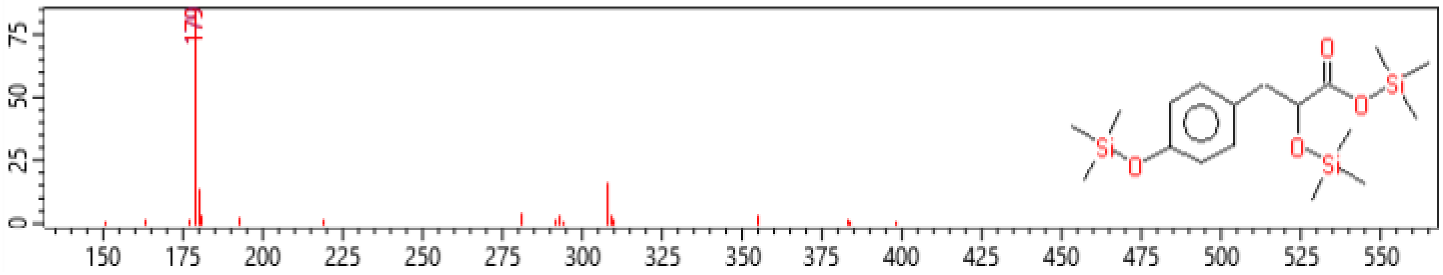

N-Acetyltyrosine, 2TMS

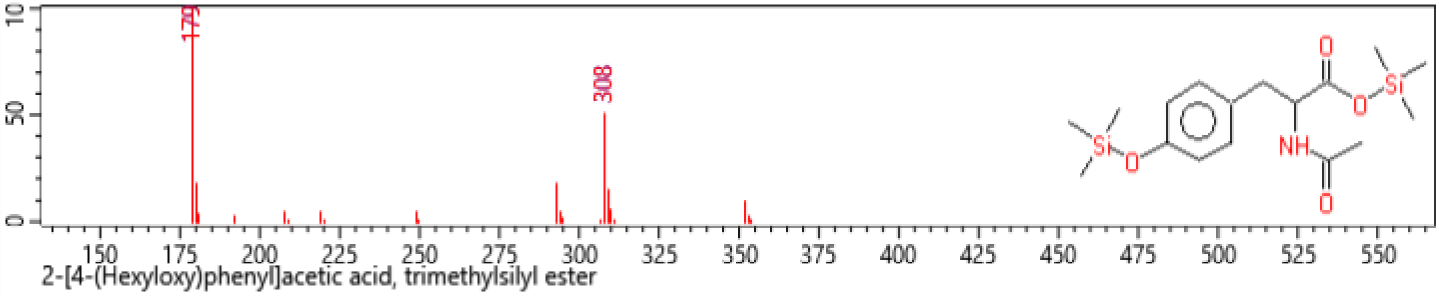

2-[4-(Hexyloxy)phenyl]acetic acid, trimethylsilyl ester

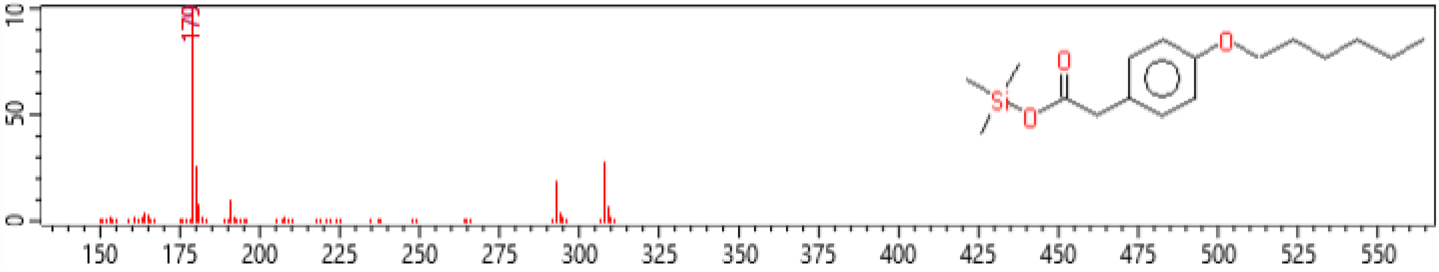

Chlamydospordiol, 2TMS

Peak: 14.52 - 14.54 min (scan #2099 - #2105) Subtracted noise: 14.49 - 14.52 min (scan #2091 - #2098), 14.58 - 14.60 min (scan #21

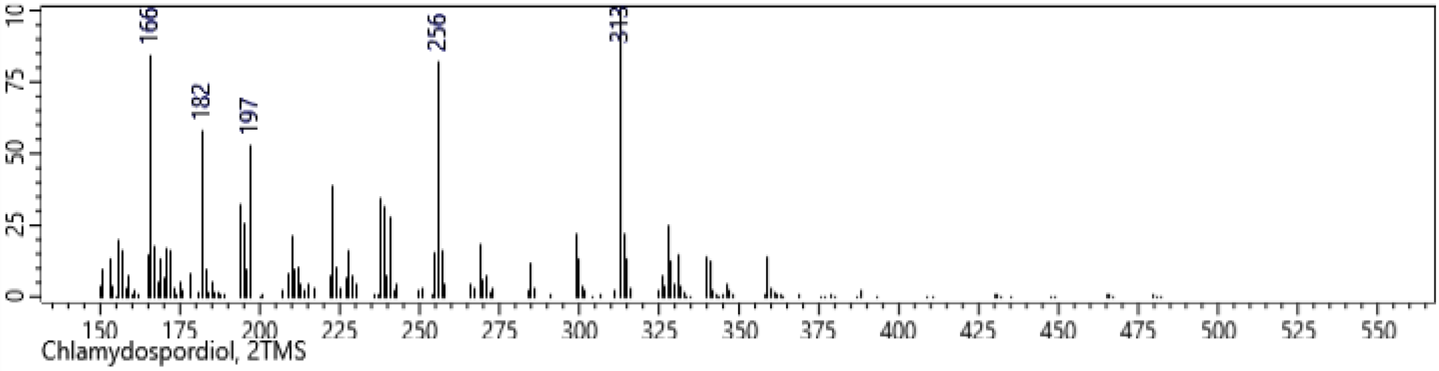

Chlamydospordiol, 2TMS

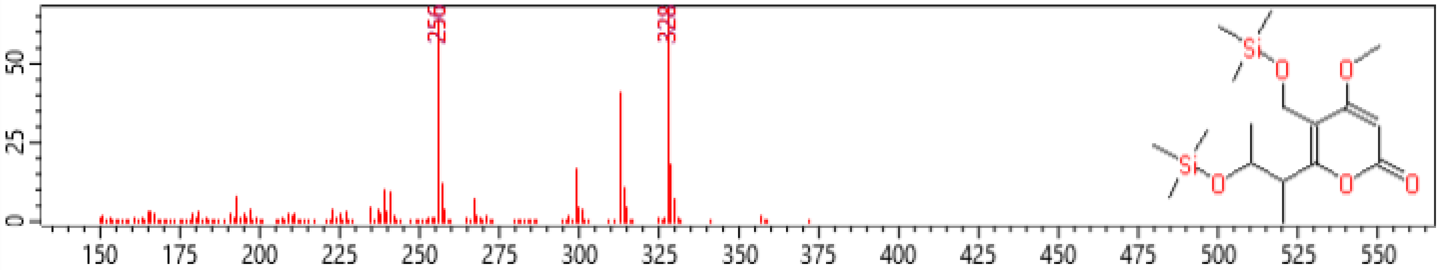

Hexadecanoic acid, 1-[[[(2-aminoethoxy)hydroxyphosphinyl]oxy]methyl]-1,2-ethanediyl ester

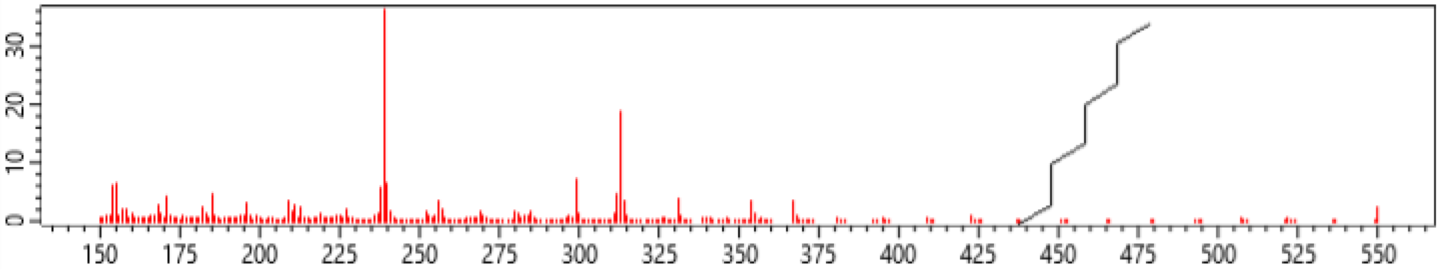

N-(3-Nitro[1,1'-biphenyl]-4-yl)acetamide, TMS derivative

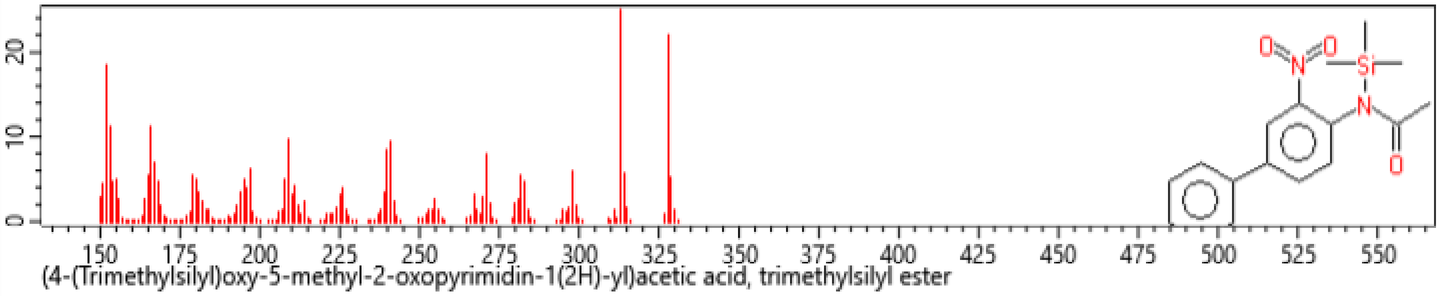

(4-(Trimethylsilyl)oxy-5-methyl-2-oxopyrimidin-1(2H)-yl)acetic acid, trimethylsilyl ester

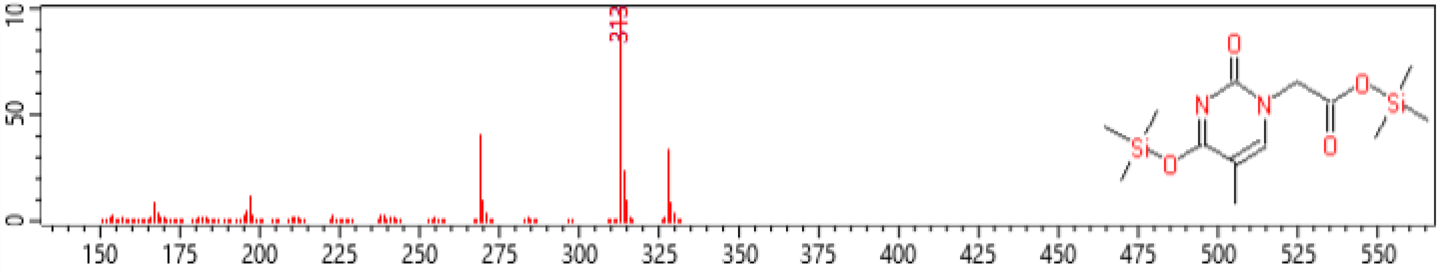

4-Amino-2-(methylsulfanyl)pyrimidine-5-carboxamide, 2TMS

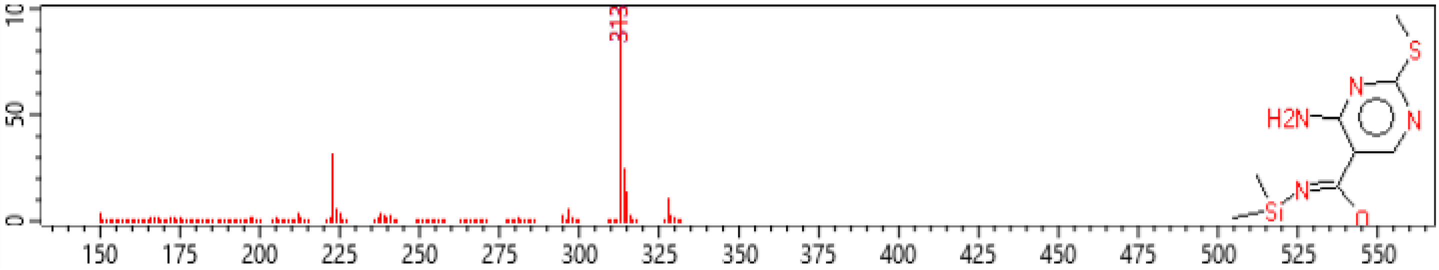

6,6'-Thiobis(2-bromo-4-chlorophenol)

Peak: 14.58 - 14.60 min (scan #2116 - #2120) Subtracted noise: 14.56 - 14.57 min (scan #2109 - #2113), 14.61 - 14.63 min (scan #21

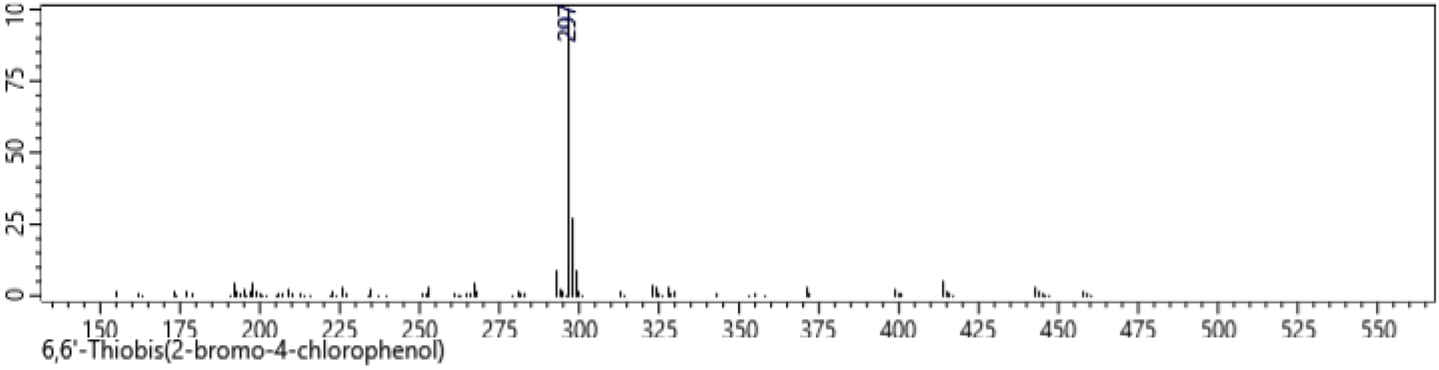

6,6'-Thiobis(2-bromo-4-chlorophenol)

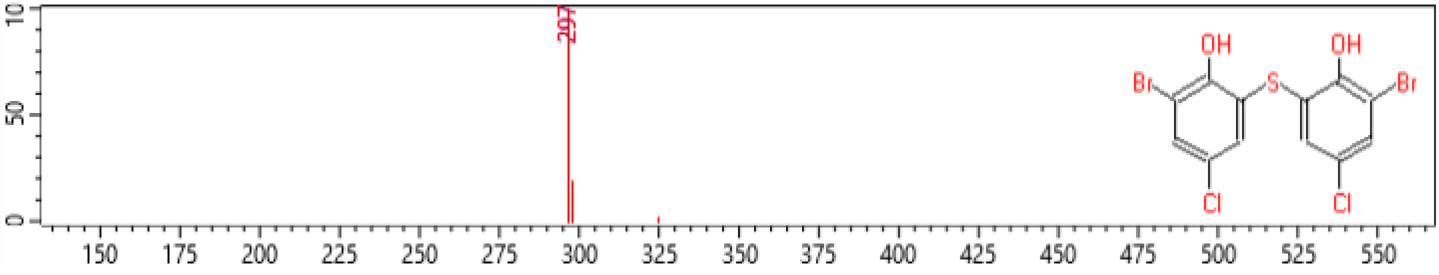

Vanillylmandelic acid, 3TMS derivative

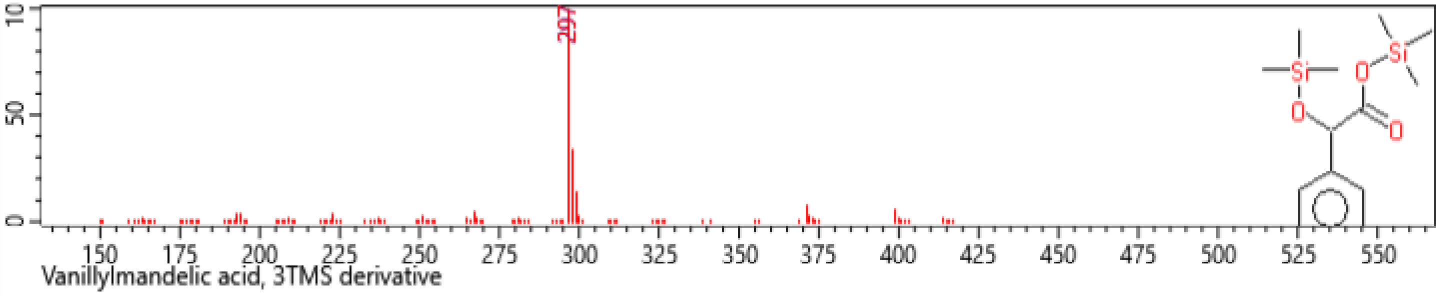

Vanillylmandelic acid, 3TMS derivative

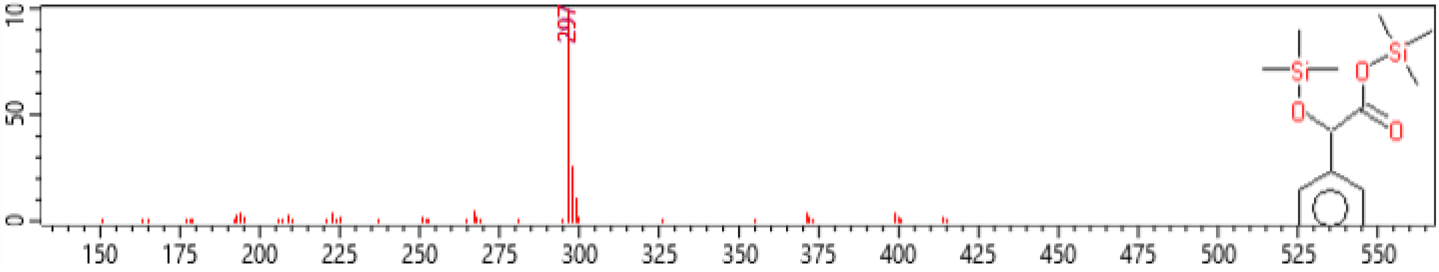

Vanillylmandelic acid, 3TMS derivative

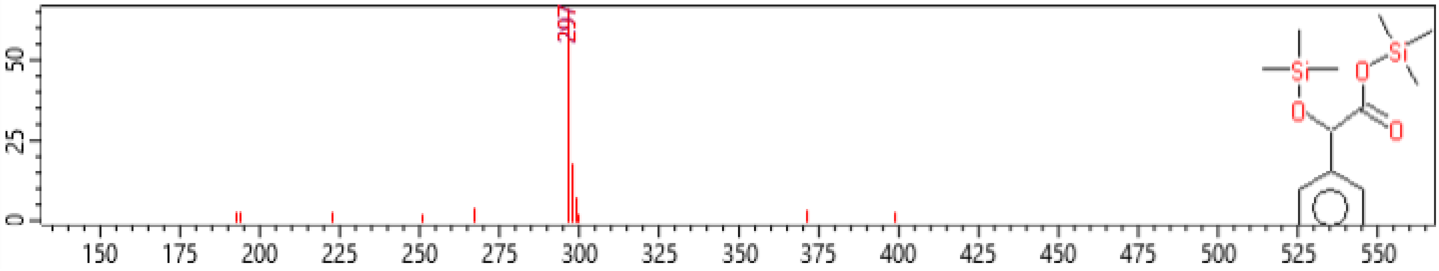

Vanillylmandelic acid, 3TMS derivative

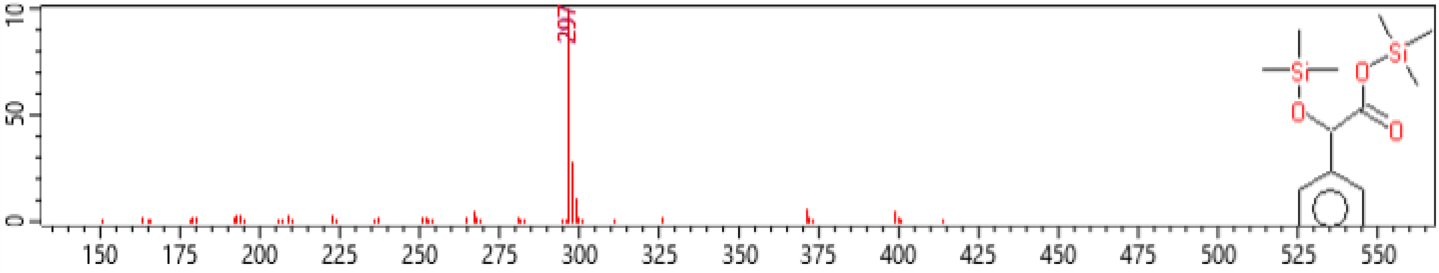

Ascochlorin, 2TMS

Peak: 14.64 - 14.67 min (scan #2131 - #2139) Subtracted noise: 14.58 - 14.61 min (scan #2116 - #2123), 14.67 - 14.70 min (scan #21

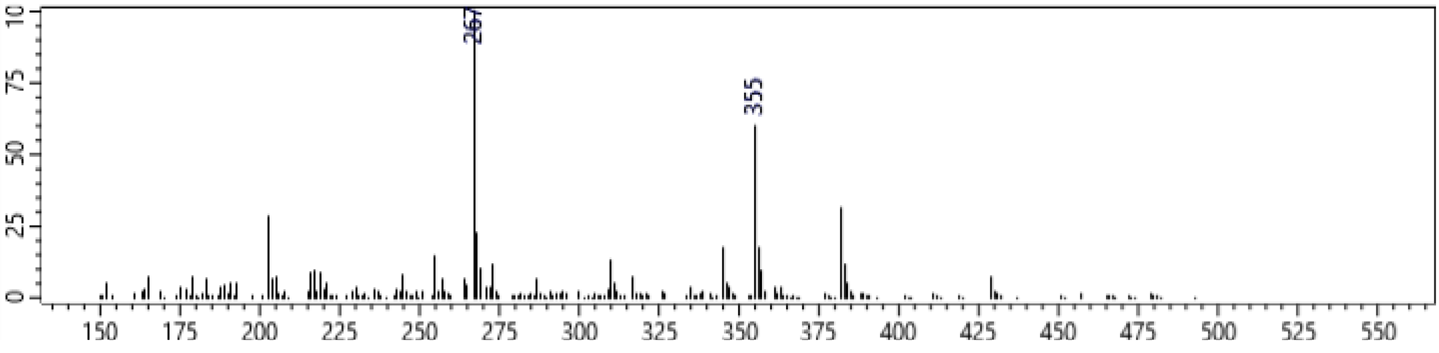

Ascochlorin, 2TMS

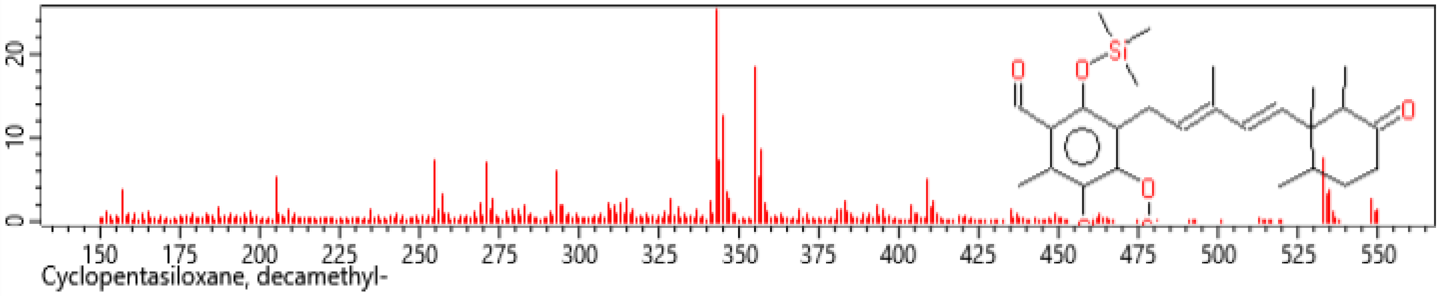

Cyclopentasiloxane, decamethyl-

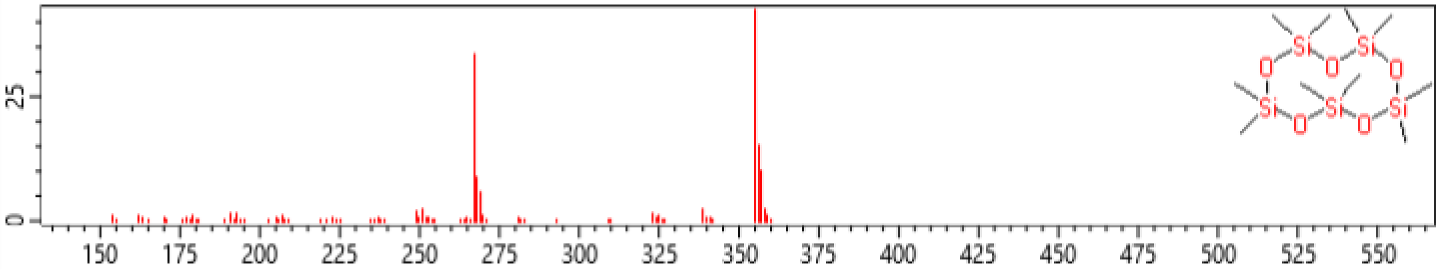

Cyclopentasiloxane, decamethyl-

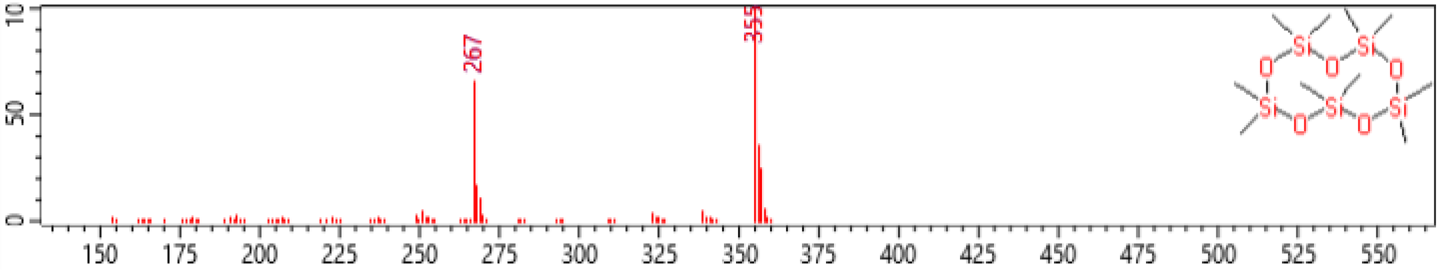

5-Chloro-2,4-dihydroxy-6-methyl-3-[(2E,4E)-3-methyl-5-(1,2,6-trimethyl-5-oxocyclohex-3-en-1-yl)penta-2,4-dienyl]benzaldehyde, 2

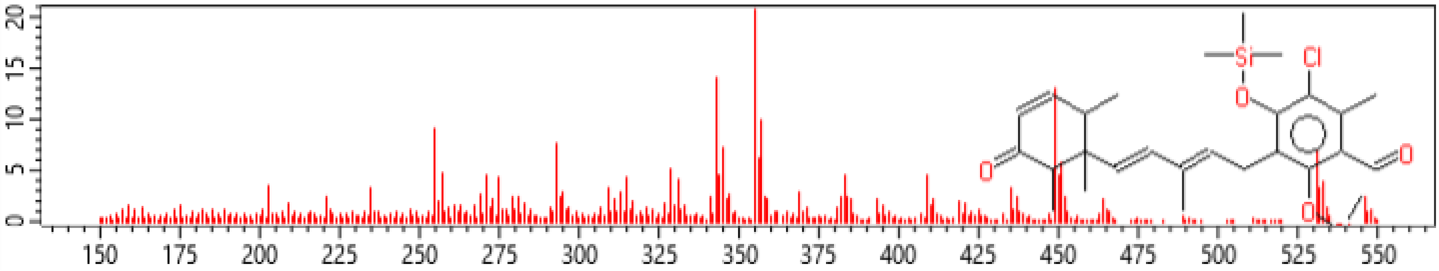

2-Hydrazinyl-5-nitropyridine, 3TMS derivative

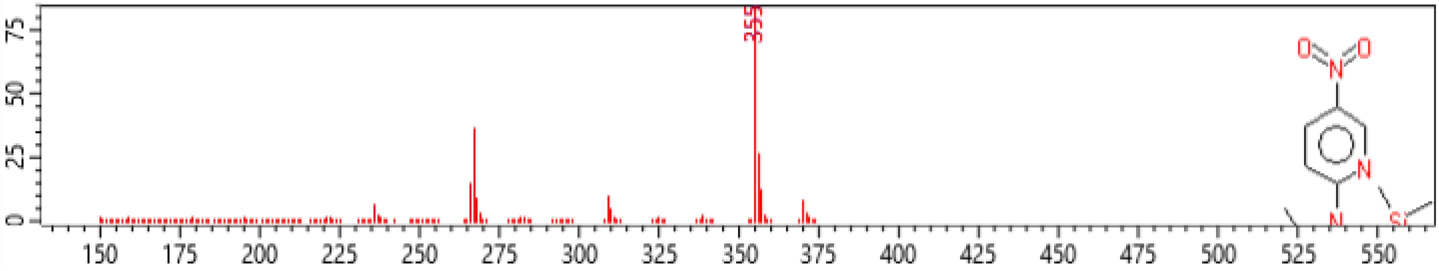

7,11-Dioxapentacyclo[15.3.0.0(4,16).0(5,13).0(5,10)]eicos-13-en-20-ol-8-one, 1β,12,12-trimethyl-

Peak: 14.70 - 14.73 min (scan #2149 - #2157) Subtracted noise: 14.64 - 14.67 min (scan #2132 - #2140), 14.74 - 14.77 min (scan #21

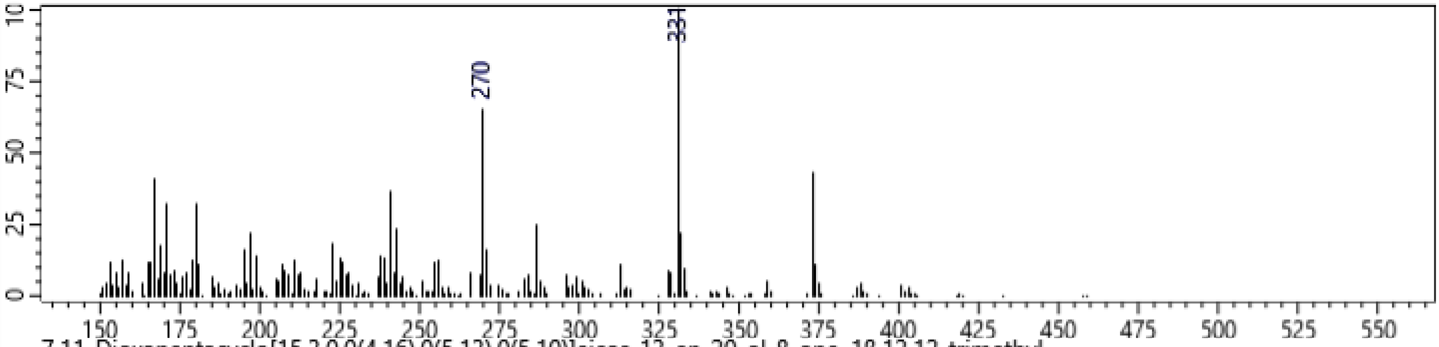

7,11-Dioxapentacyclo[15.3.0.0(4,16).0(5,13).0(5,10)]eicos-13-en-20-ol-8-one, 1β,12,12-trimethyl-

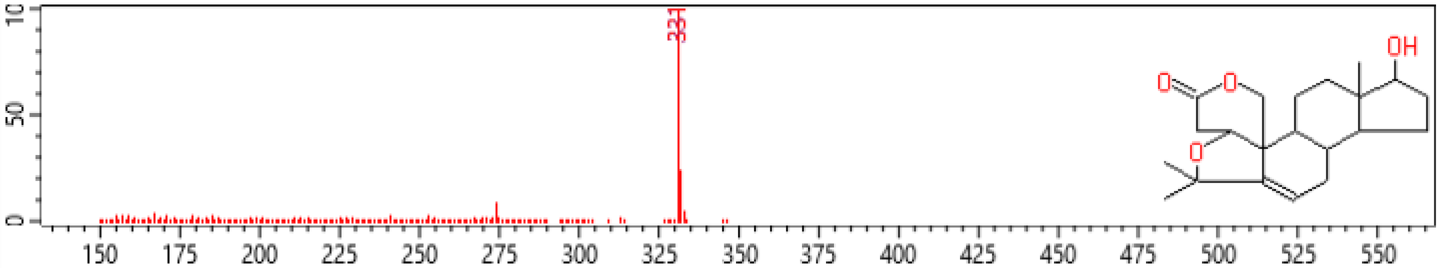

Triptonide

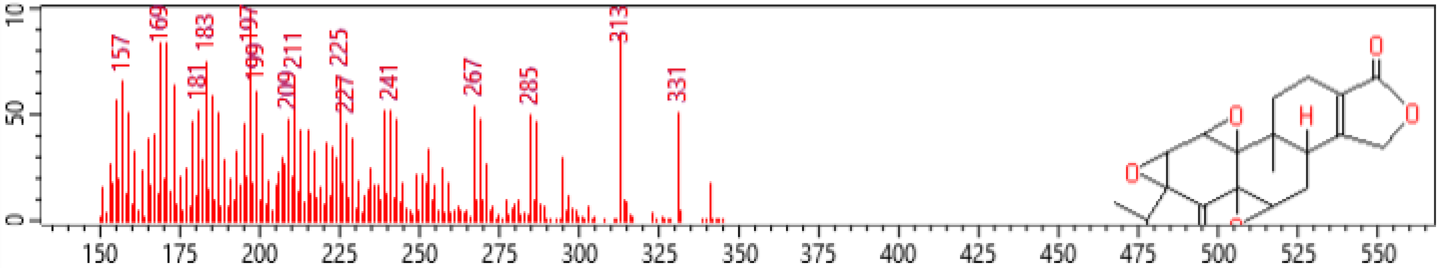

Cephalotaxine, 11-hydroxy-, (11α)-

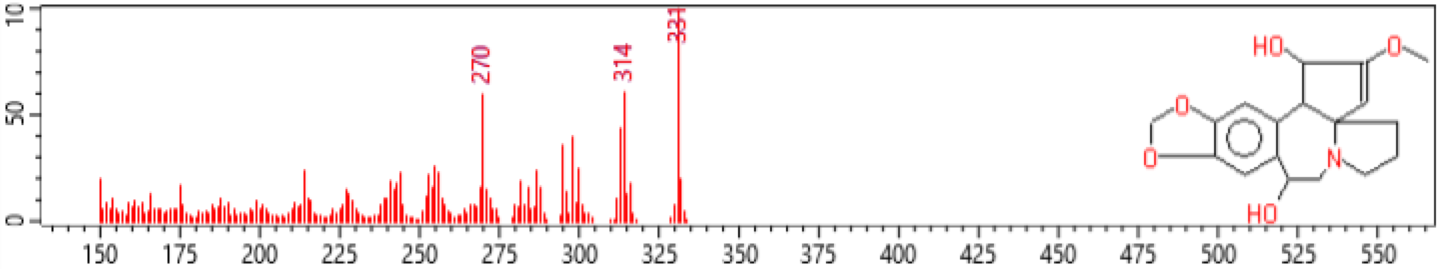

Montanin D, 2Ac derivative

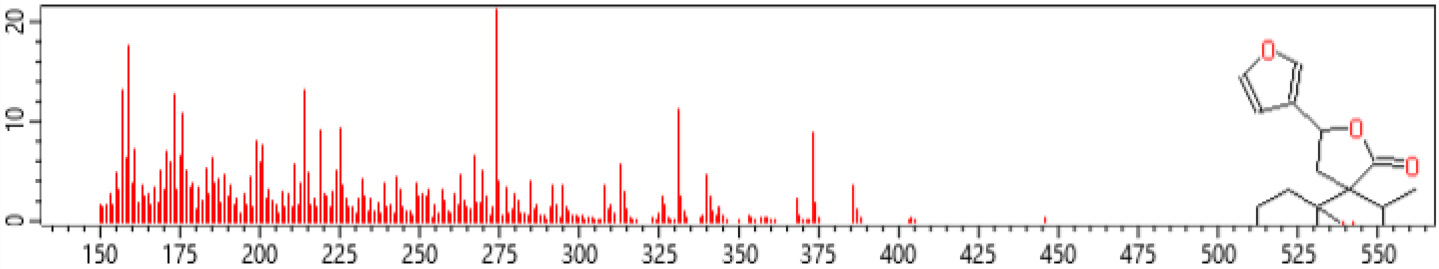

Acetic acid, 3,4-diacetoxy-7-cyano-1,4a,7-trimethyl-8-(2-oxoethyl)tetradecahydrophenanthren-2-yl ester

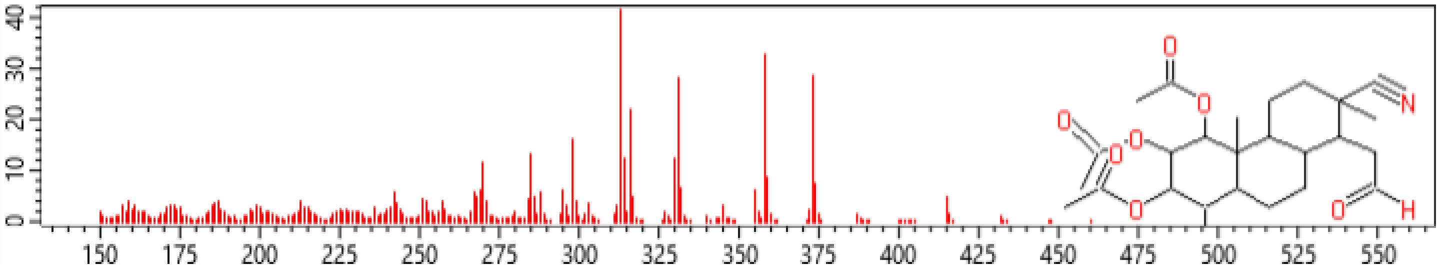

Isorhamnetin 3-neohesperidoside

Peak: 14.74 - 14.77 min (scan #2160 - #2168) Subtracted noise: 14.70 - 14.73 min (scan #2150 - #2158), 14.80 - 14.83 min (scan #21

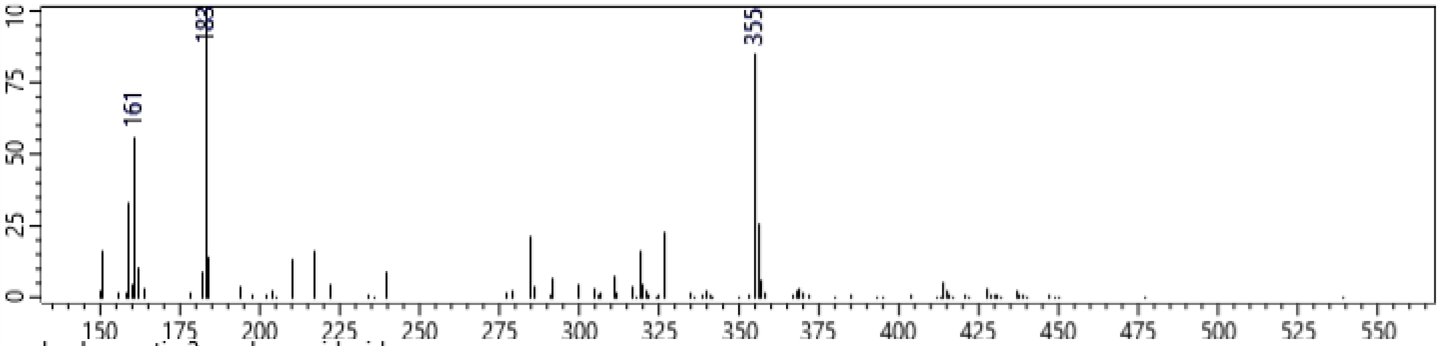

Isorhamnetin 3-neohesperidoside

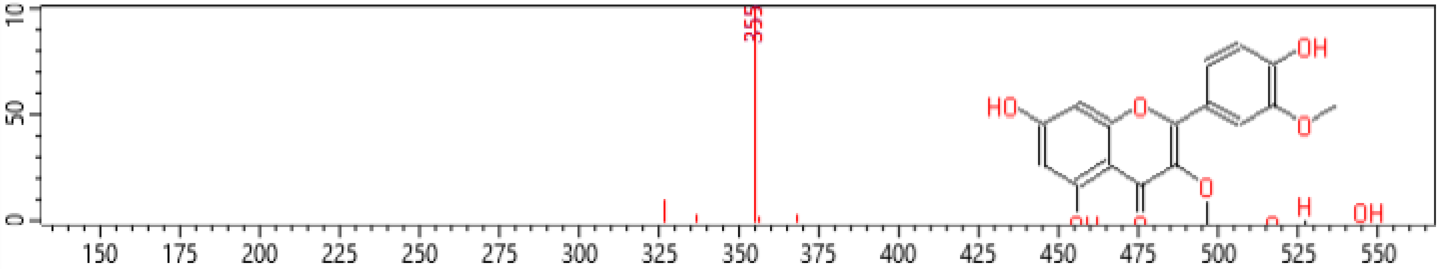

Prostaglandin F1α

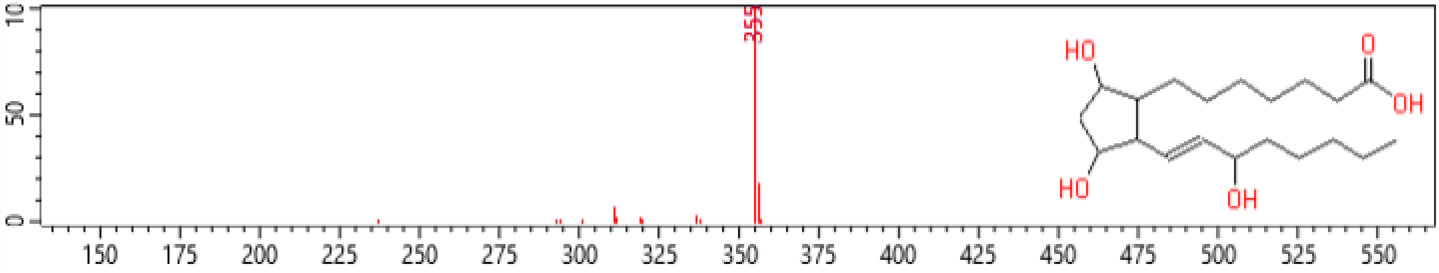

Ethanone, 1-[5-ethyl-2-hydroxy-4-[[6-methyl-6-(2H-tetrazol-5-yl)heptyl]oxy]phenyl]-

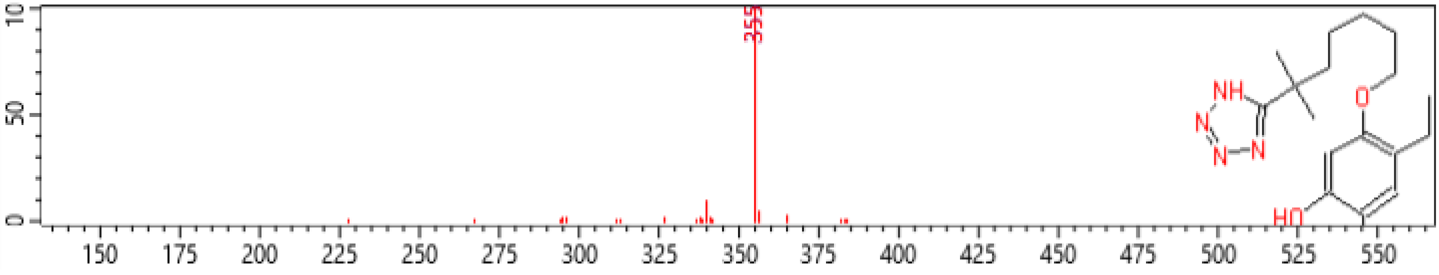

6H-Thieno[3,2-f][1,2,4]triazolo[4,3-a][1,4]diazepine-6-acetamide, 4-(4-chlorophenyl)-2,3,9-trimethyl-, (6S)-

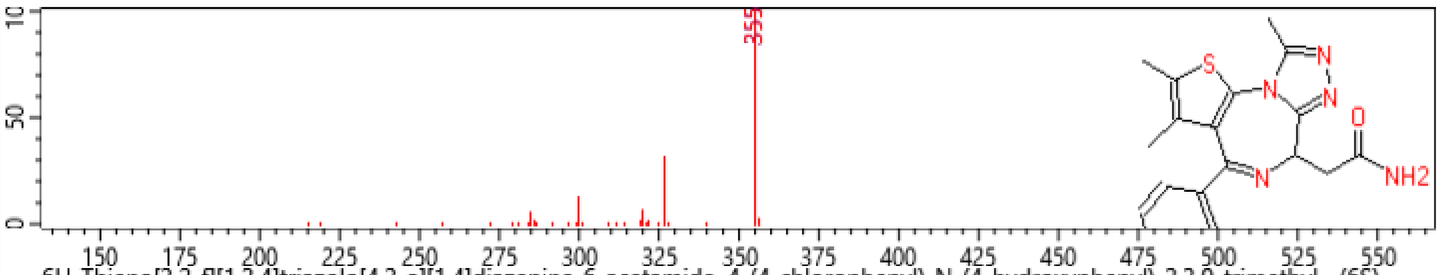

6H-Thieno[3,2-f][1,2,4]triazolo[4,3-a][1,4]diazepine-6-acetamide, 4-(4-chlorophenyl)-N-(4-hydroxyphenyl)-2,3,9-trimethyl-, (6S)-

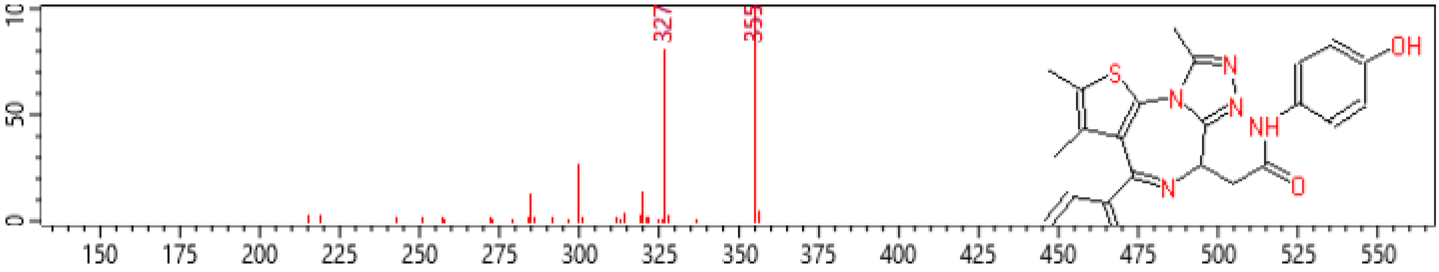

3,4-Dihydroxyhydrocinnamic acid, 3TMS derivative

Peak: 14.82 - 14.85 min (scan #2183 - #2191) Subtracted noise: 14.77 - 14.80 min (scan #2169 - #2177), 14.86 - 14.89 min (scan #21

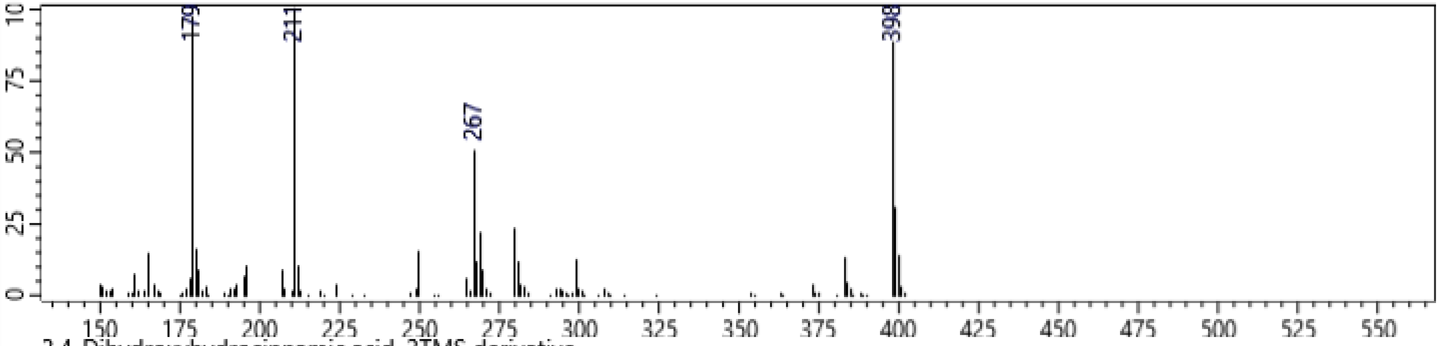

3,4-Dihydroxyhydrocinnamic acid, 3TMS derivative

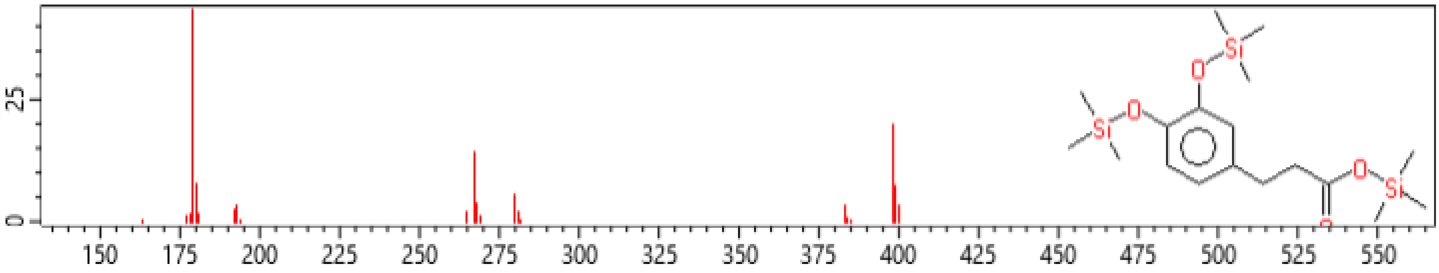

3,4-Dihydroxyhydrocinnamic acid, 3TMS derivative

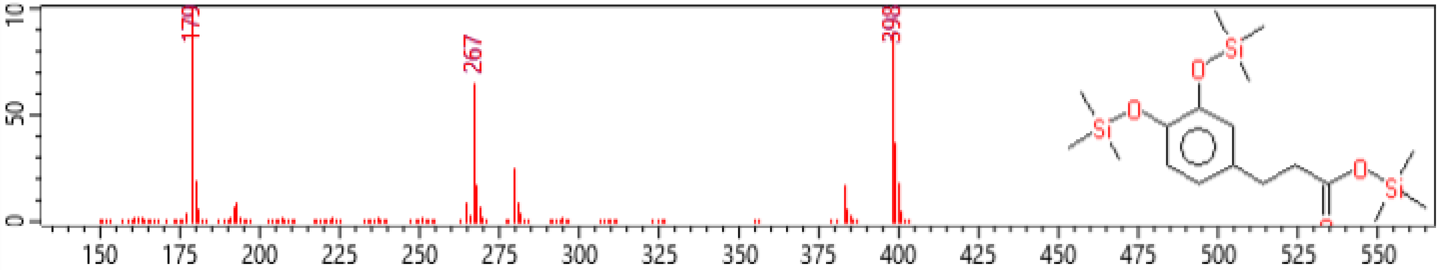

3-(3-Hydroxyphenyl)-3-hydroxypropionic acid, tris-(O-trimethylsilyl)-

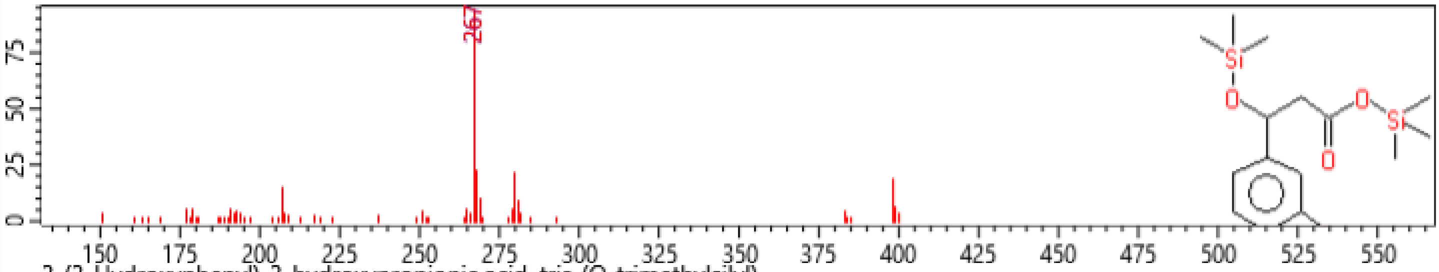

3-(3-Hydroxyphenyl)-3-hydroxypropionic acid, tris-(O-trimethylsilyl)-

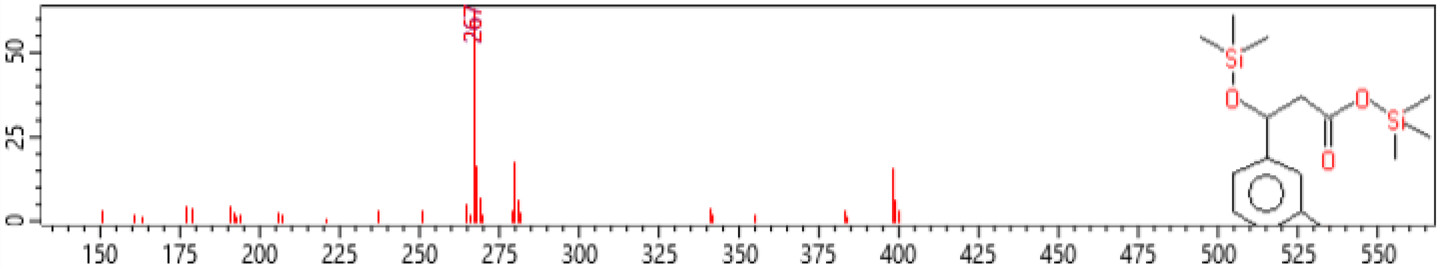

3-(3-Hydroxyphenyl)-3-hydroxypropionic acid, tris-(O-trimethylsilyl)-

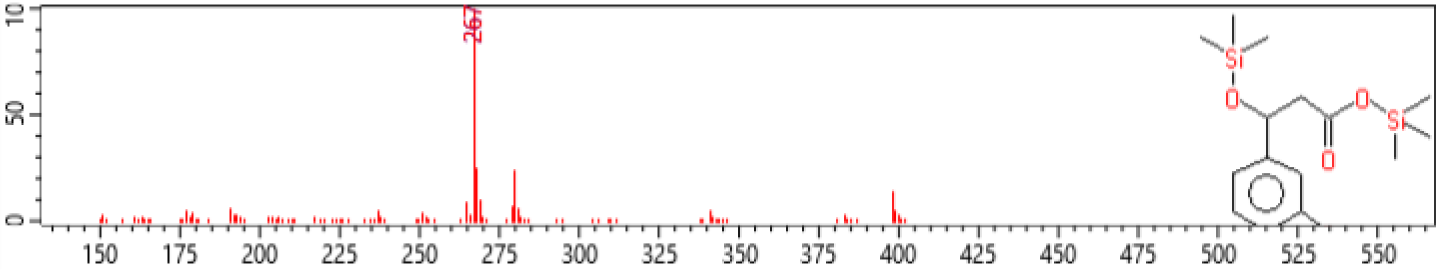

Biochanin A, trimethylsilyl ether

Peak: 14.88 - 14.93 min (scan #2200 - #2212) Subtracted noise: 14.82 - 14.86 min (scan #2182 - #2193), 14.95 - 14.99 min (scan #22

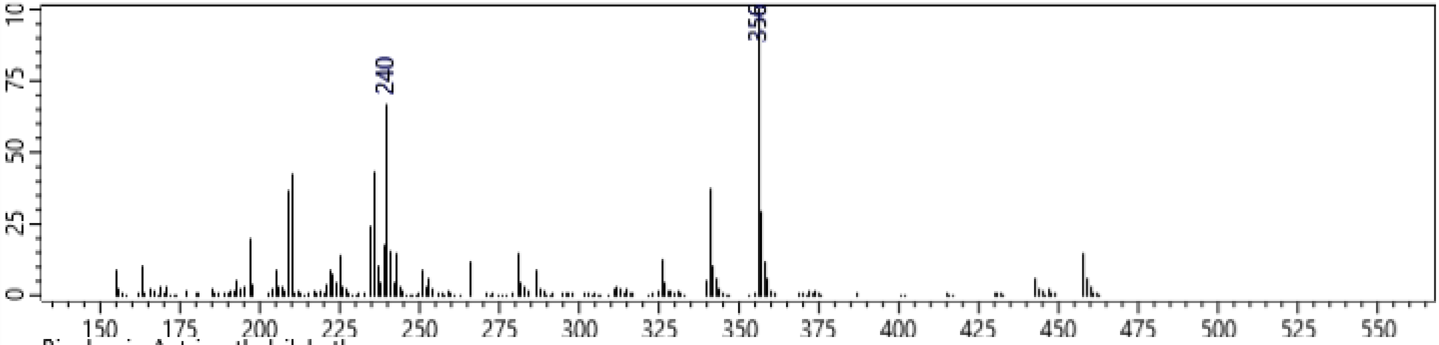

Biochanin A, trimethylsilyl ether

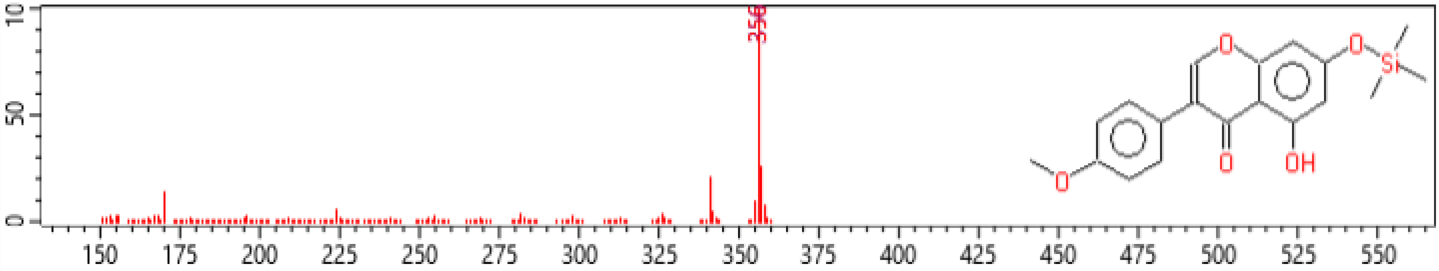

o-Tolidine, N,N'-bis(trimethylsilyl)-

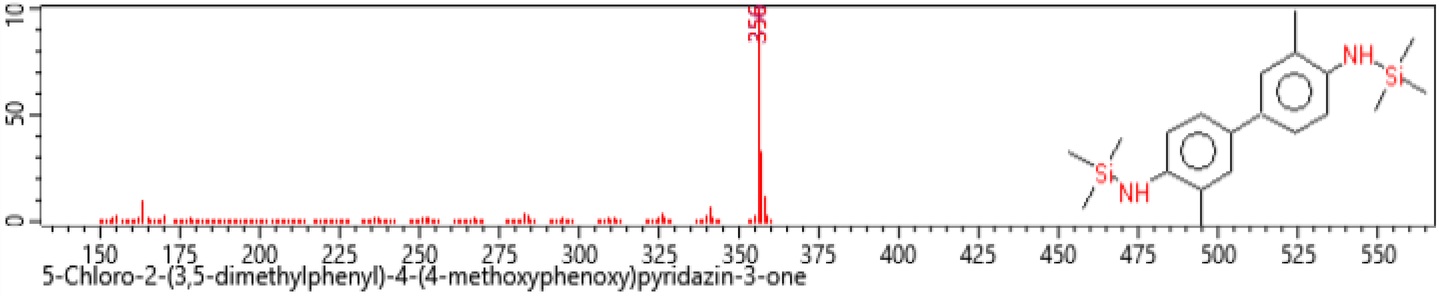

5-Chloro-2-(3,5-dimethylphenyl)-4-(4-methoxyphenoxy)pyridazin-3-one

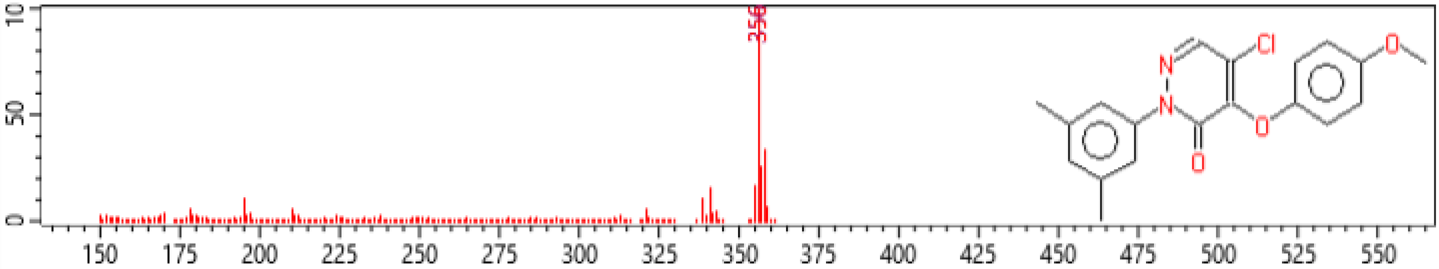

Pinosylvin, bis(trimethylsilyl) ether

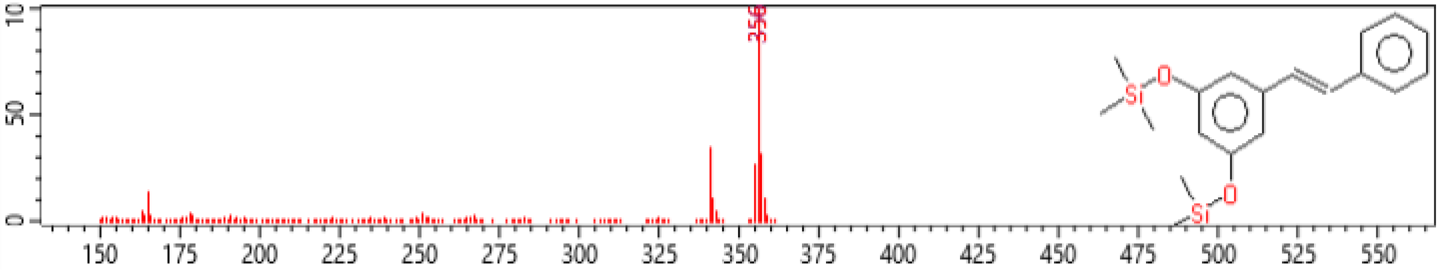

(Z)-4-(Phenyldiazenyl)-N1,N3-bis(trimethylsilyl)benzene-1,3-diamine

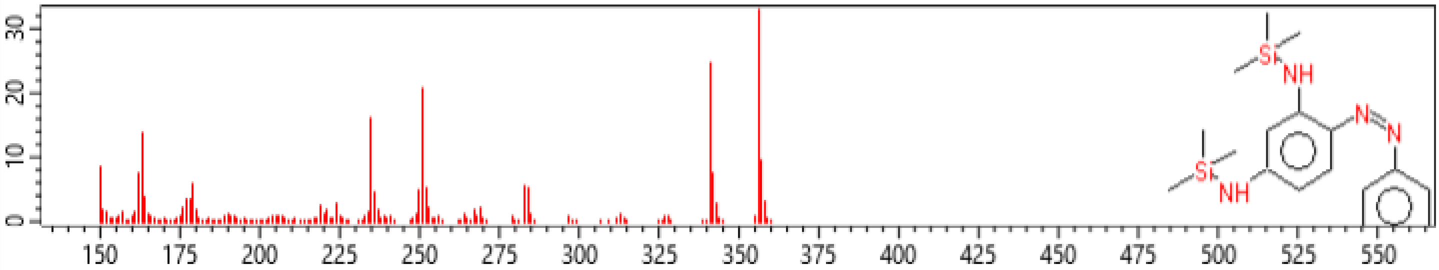

Fumaric acid, dodecyl propyl ester

Peak: 14.99 - 15.04 min (scan #2231 - #2243) Subtracted noise: 14.91 - 14.95 min (scan #2207 - #2218), 15.04 - 15.08 min (scan #22

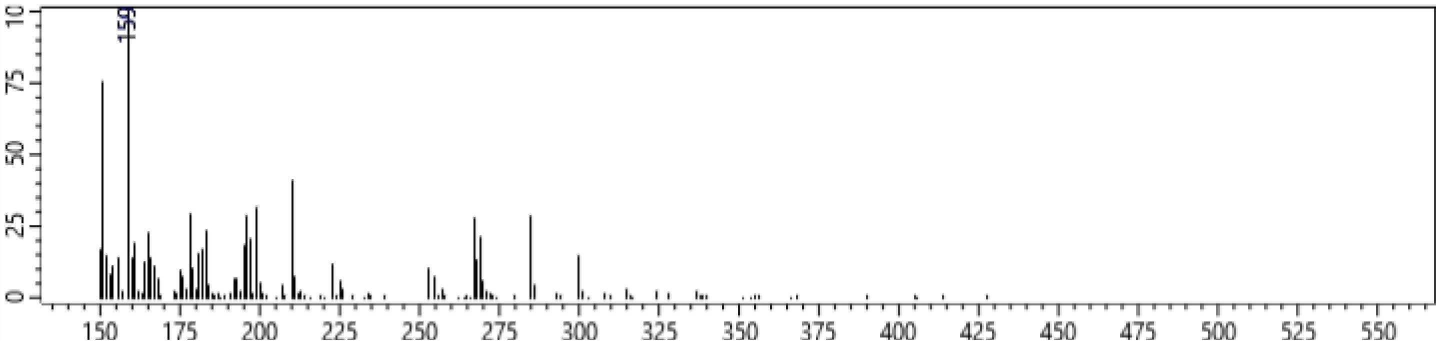

Fumaric acid, dodecyl propyl ester

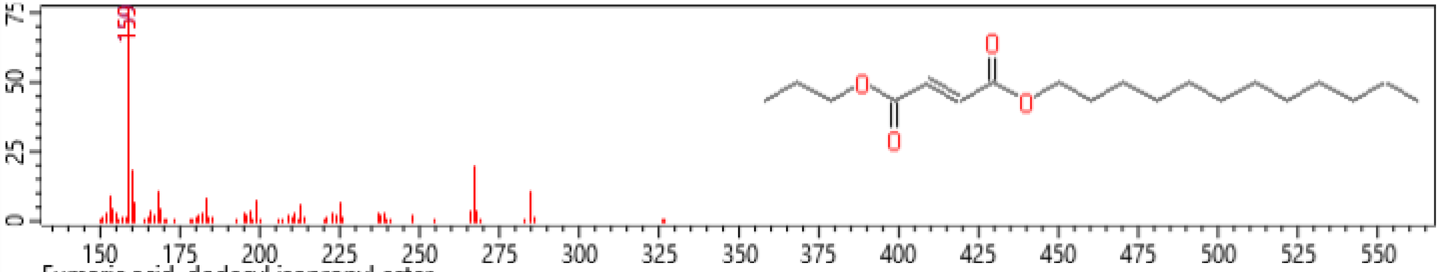

Fumaric acid, dodecyl isopropyl ester

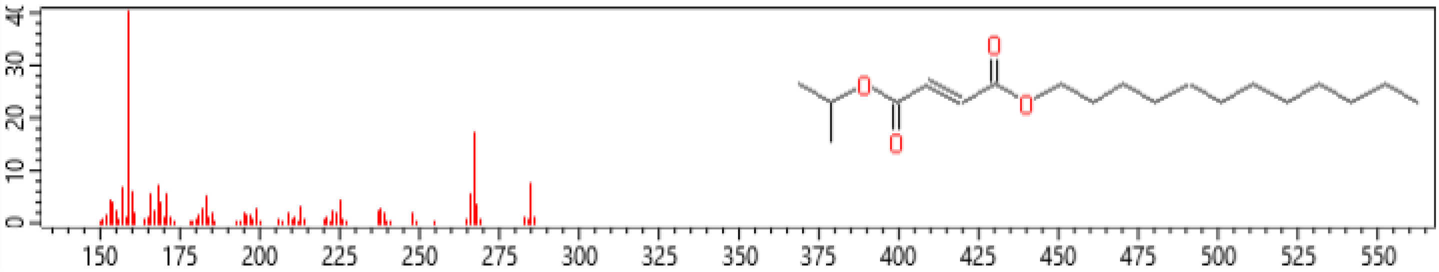

Isorhoifolin

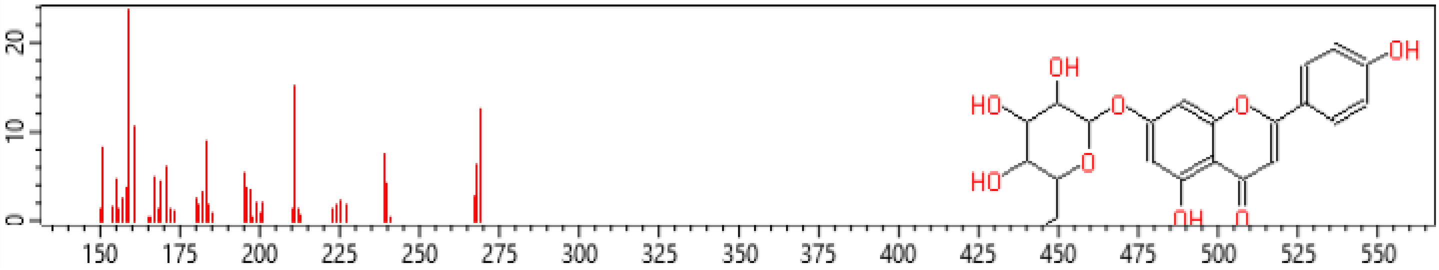

Retinal, 9-cis-

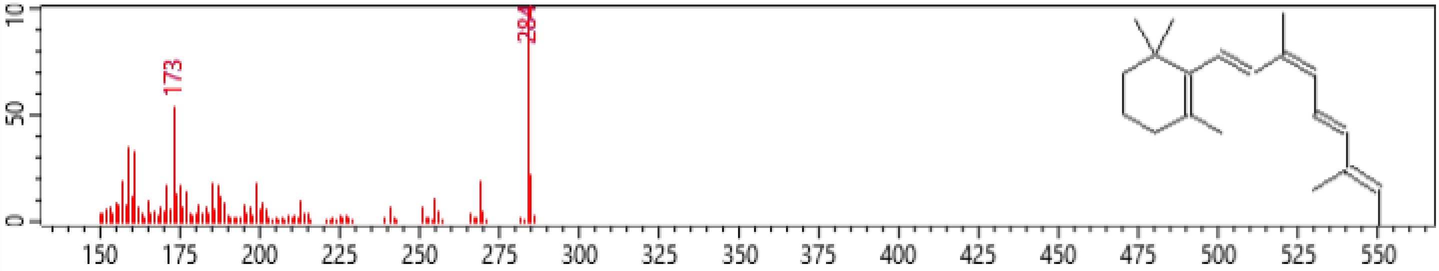

Oxirane-2-carboxylic acid, 3-(3,4,5-trimethoxyphenyl)-, methyl ester

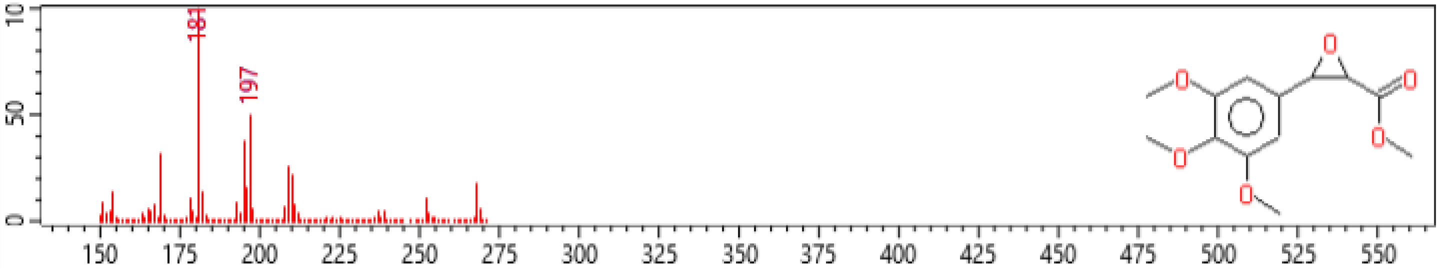

Sufentanyl

Peak: 15.08 - 15.12 min (scan #2254 - #2266) Subtracted noise: 15.00 - 15.04 min (scan #2232 - #2243), 15.13 - 15.17 min (scan #22

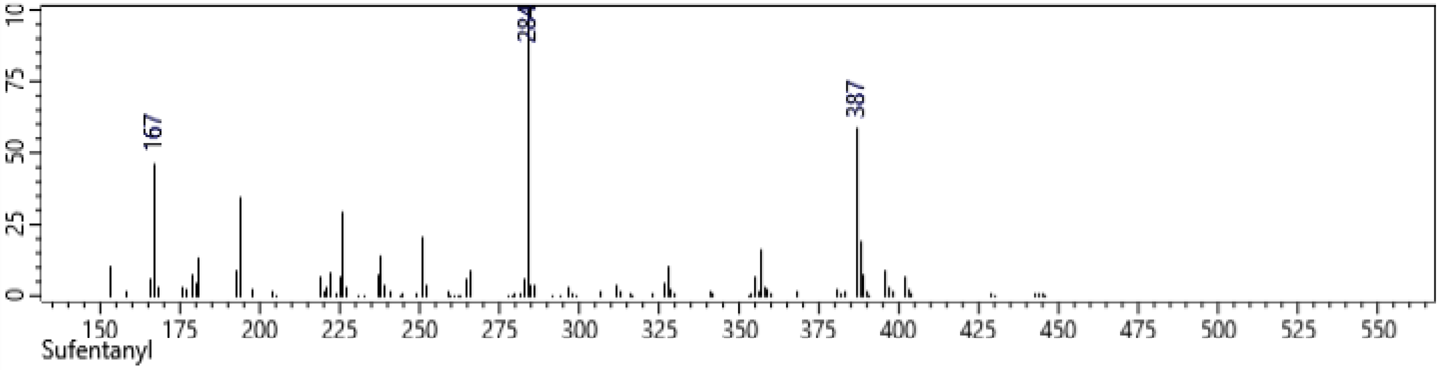

Sufentanyl

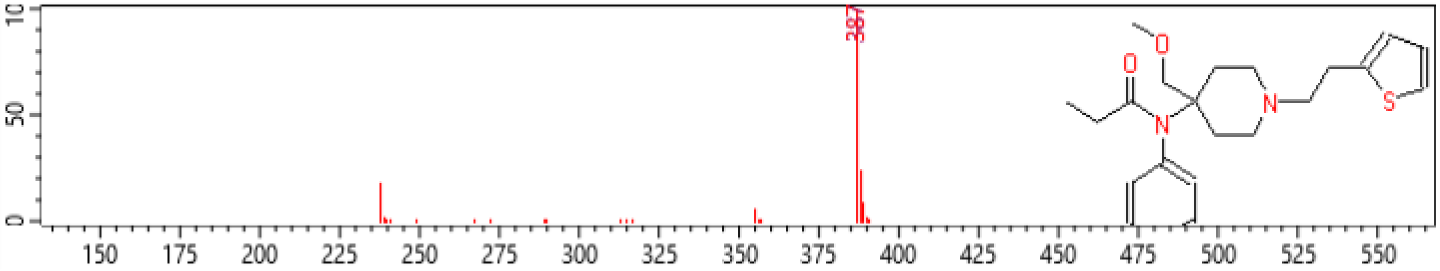

Irigenin, trimethyl ether

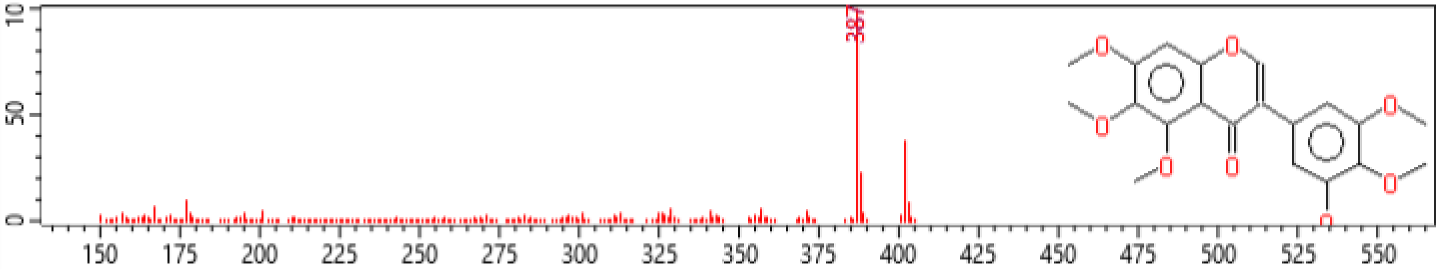

Buchanaxanthone, 2TMS

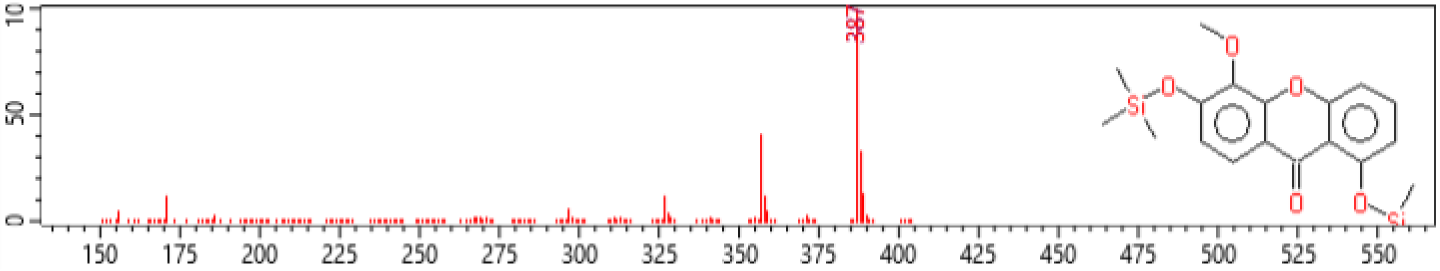

15(R)-15-Methylprostaglandin F2α methyl ester

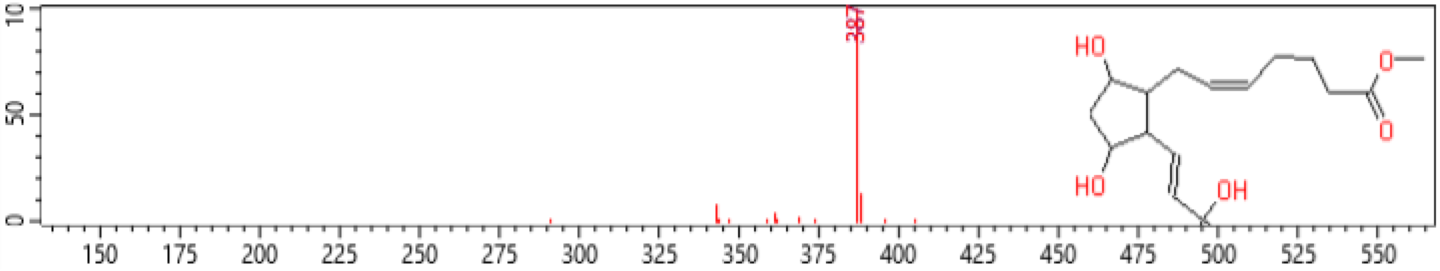

Irisflorentin

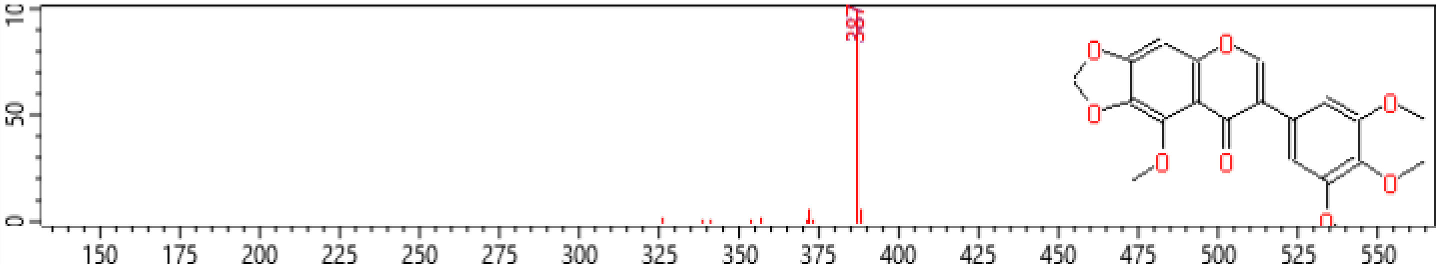

Uracil, 1,3-dimethyl-5-(2'-trimethylsilyloxyethyl)-6-(trimethylsilyloxymethyl)-

Peak: 15.20 - 15.26 min (scan #2288 - #2306) Subtracted noise: 15.06 - 15.13 min (scan #2250 - #2268), 15.27 - 15.34 min (scan #23

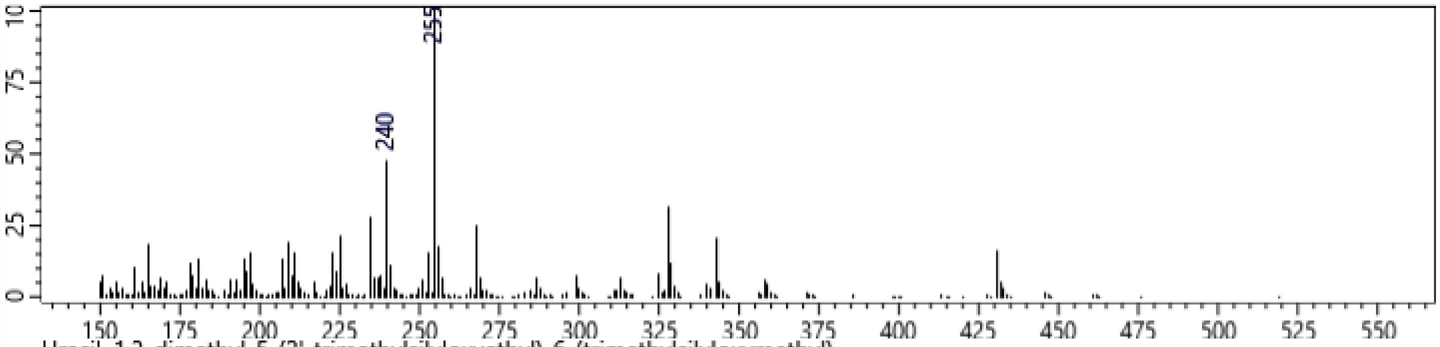

Uracil, 1,3-dimethyl-5-(2'-trimethylsilyloxyethyl)-6-(trimethylsilyloxymethyl)-

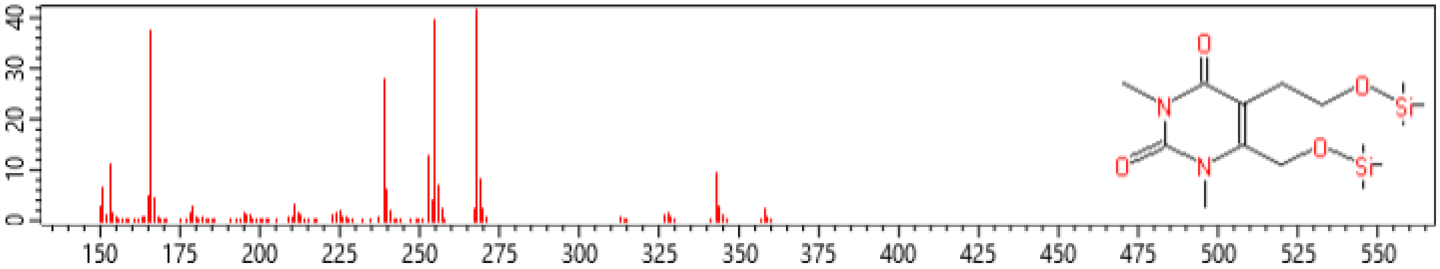

Glycodeoxycholic acid

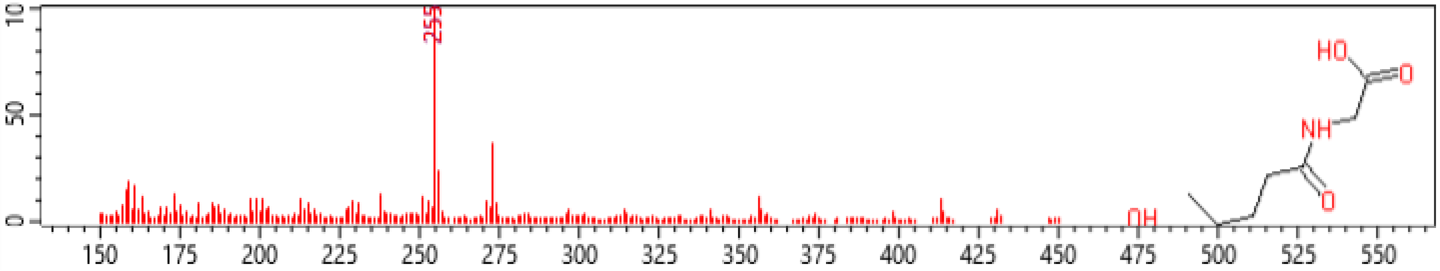

Deoxycholic Acid, 2TMS derivative

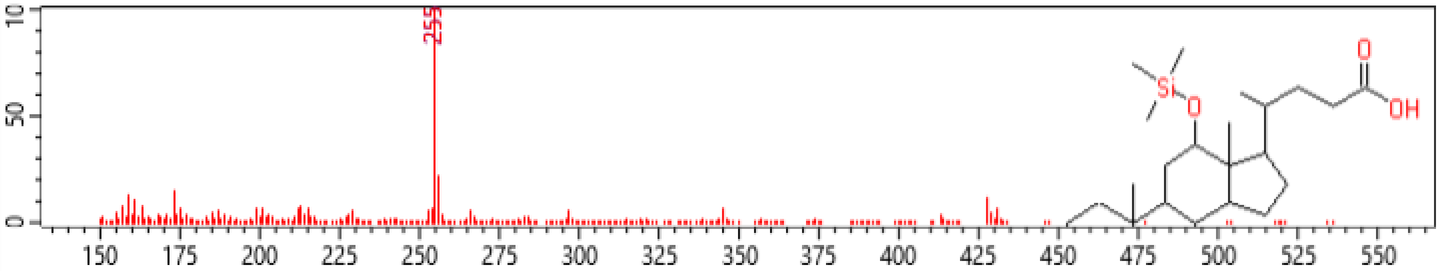

12-O-Tigloylphorbol-13-(2-methylbutyrate)

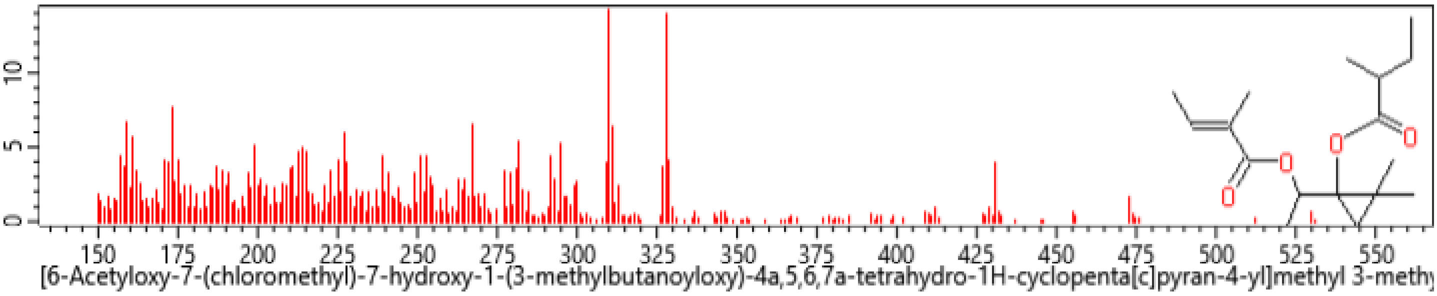

[6-Acetyloxy-7-(chloromethyl)-7-hydroxy-1-(3-methylbutanoyloxy)-4a,5,6,7a-tetrahydro-1H-cyclopenta[c]pyran-4-yl]methyl 3-methy

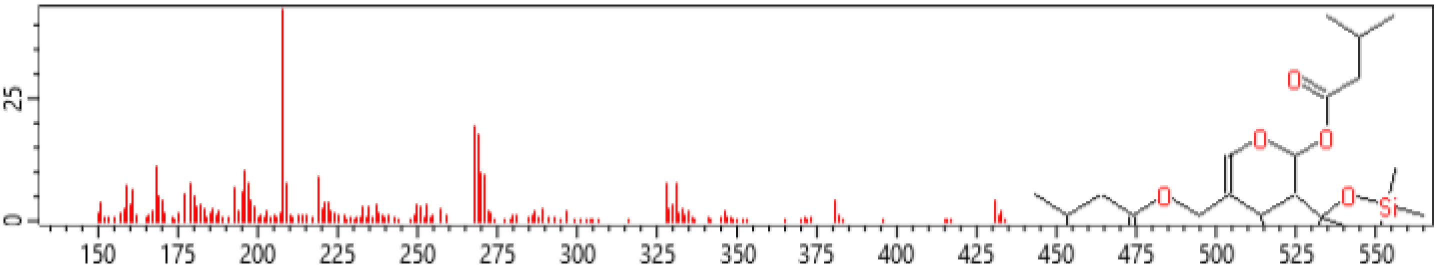

3,6,3'-Trimethoxyflavone

Peak: 15.29 - 15.30 min (scan #2313 - #2317) Subtracted noise: 15.26 - 15.27 min (scan #2304 - #2307), 15.31 - 15.32 min (scan #23

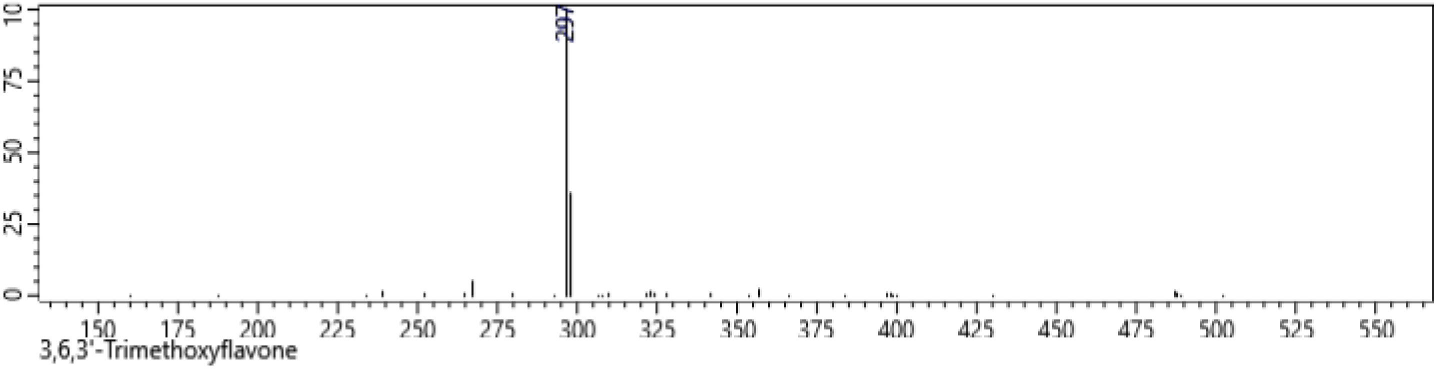

3,6,3'-Trimethoxyflavone

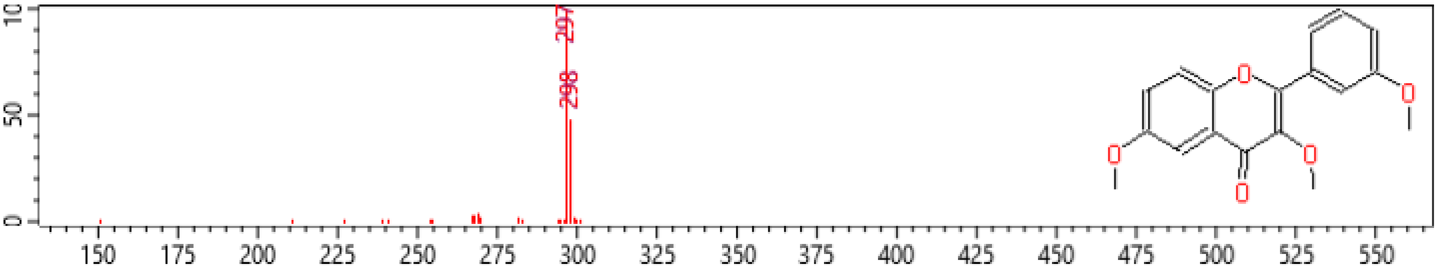

3,6,3'-Trimethoxyflavone

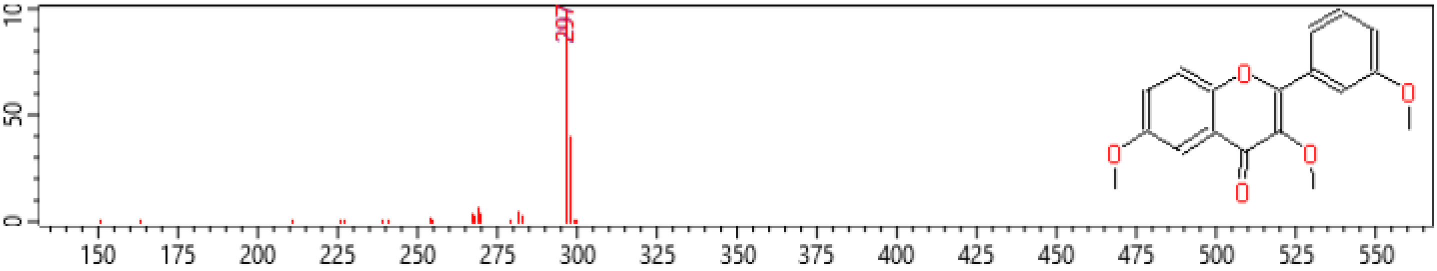

6,6'-Thiobis(2-bromo-4-chlorophenol)

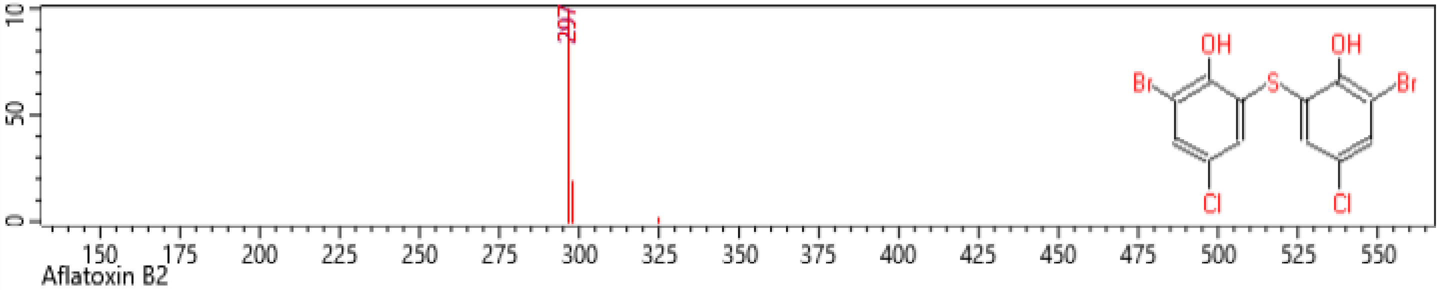

Aflatoxin B2

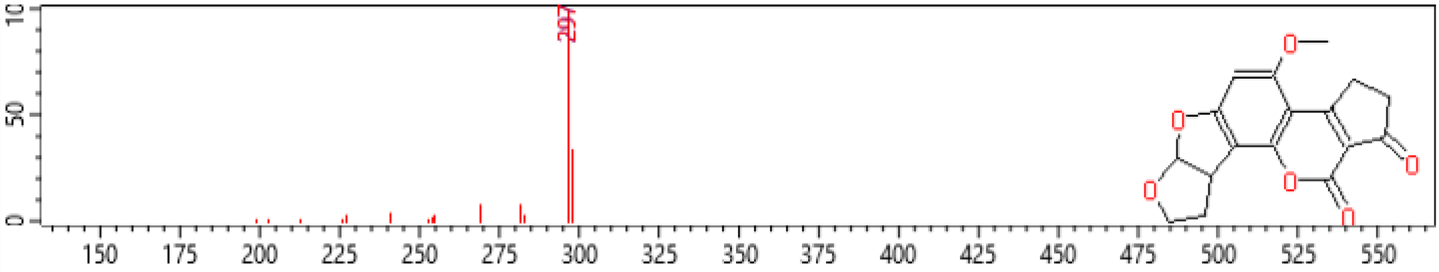

2,6-Difluoro-3-methylbenzamide, N-(1-naphthyl)-

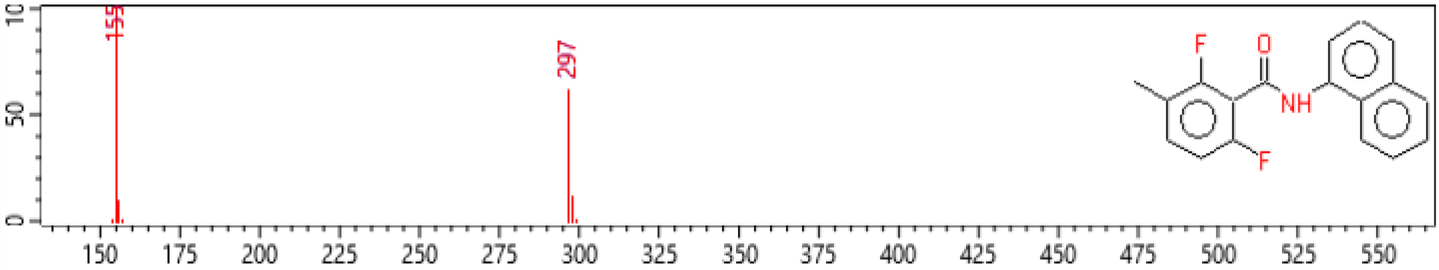

1-(2-Hydroxy-4,5-bis((trimethylsilyl)oxy)phenyl)butan-1-one

Peak: 15.31 - 15.32 min (scan #2319 - #2323) Subtracted noise: 15.29 - 15.30 min (scan #2314 - #2317), 15.34 - 15.35 min (scan #23

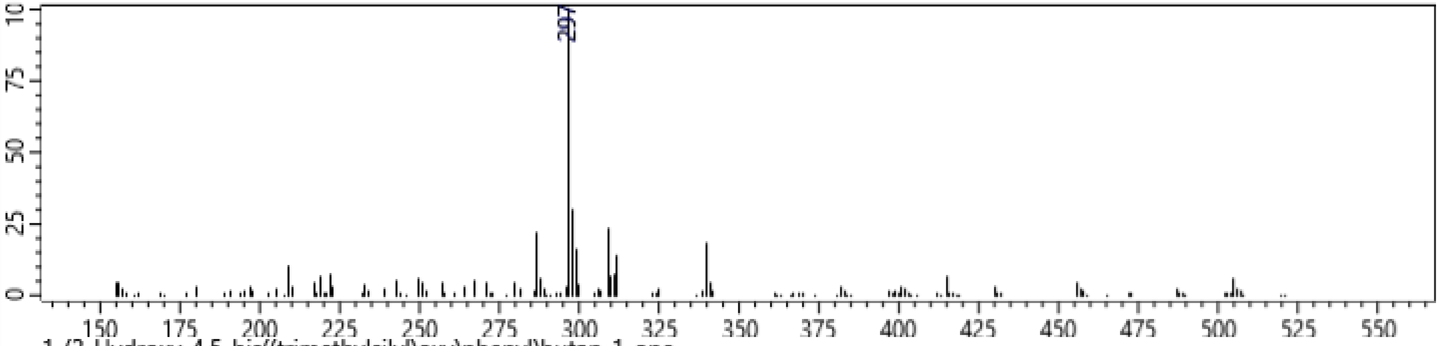

1-(2-Hydroxy-4,5-bis((trimethylsilyl)oxy)phenyl)butan-1-one

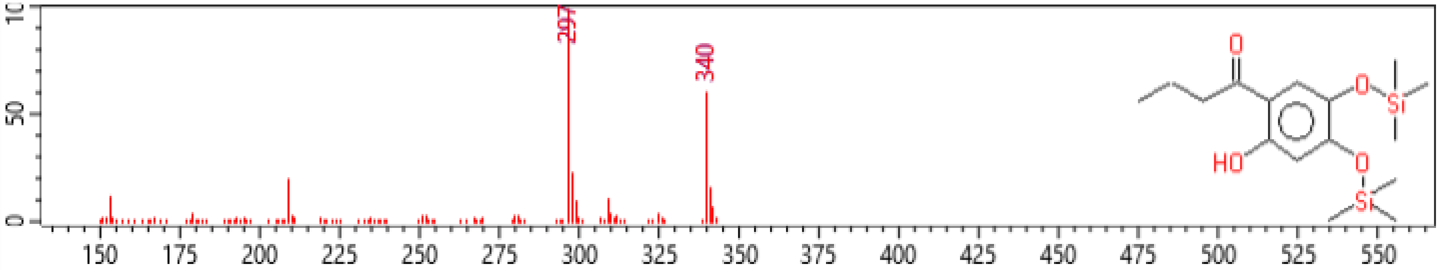

1,3,5,7-Tetraethyl-1-methoxycyclotetrasiloxane

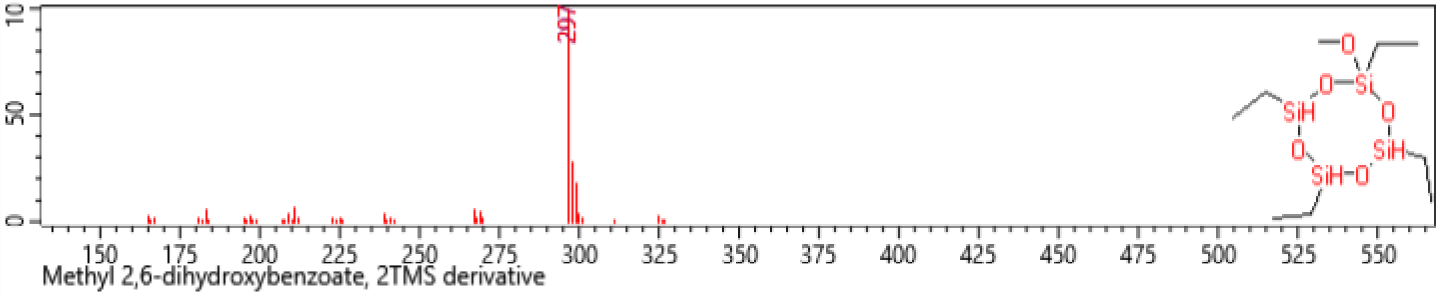

Methyl 2,6-dihydroxybenzoate, 2TMS derivative

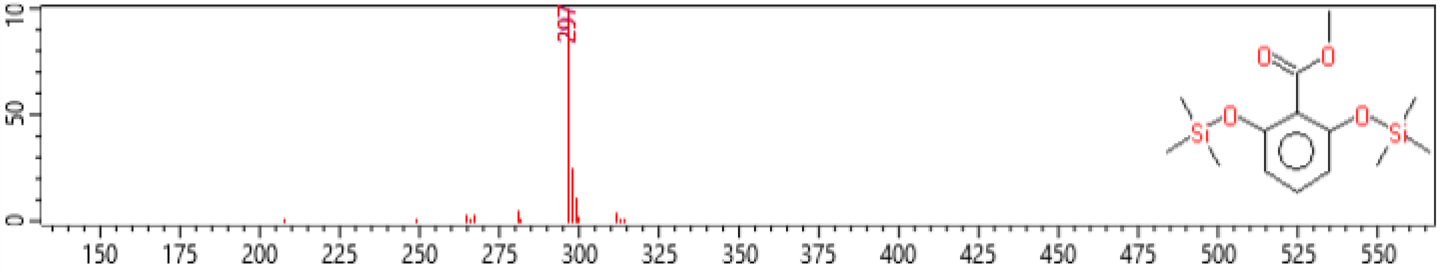

Vanillylmandelic acid, 3TMS derivative

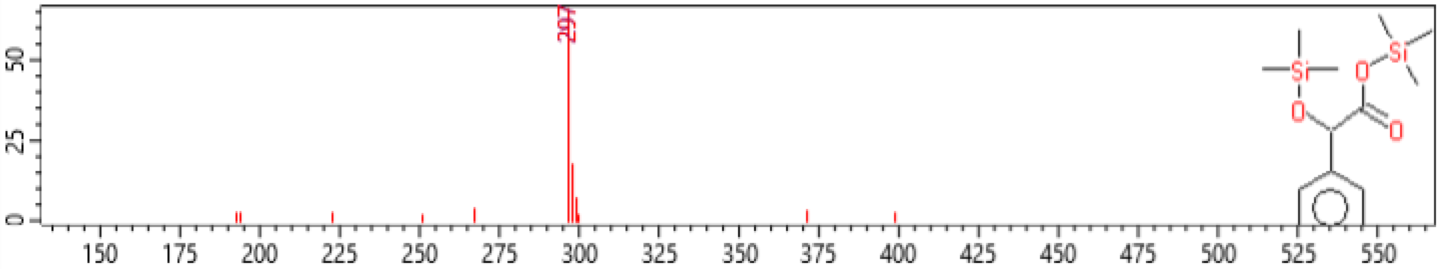

Normetanephrine, 3TMS derivative

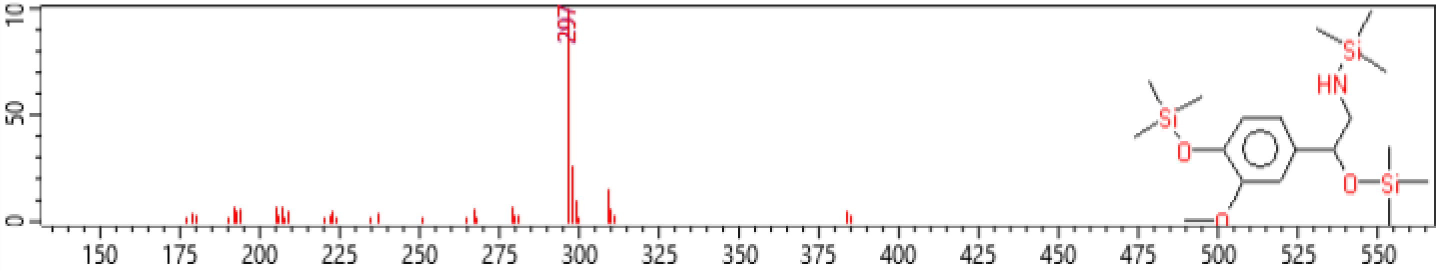

7,18-Dihydroxykaurenolide, 2TMS derivative

Peak: 15.35 - 15.37 min (scan #2331 - #2335) Subtracted noise: 15.32 - 15.34 min (scan #2323 - #2327), 15.37 - 15.39 min (scan #23

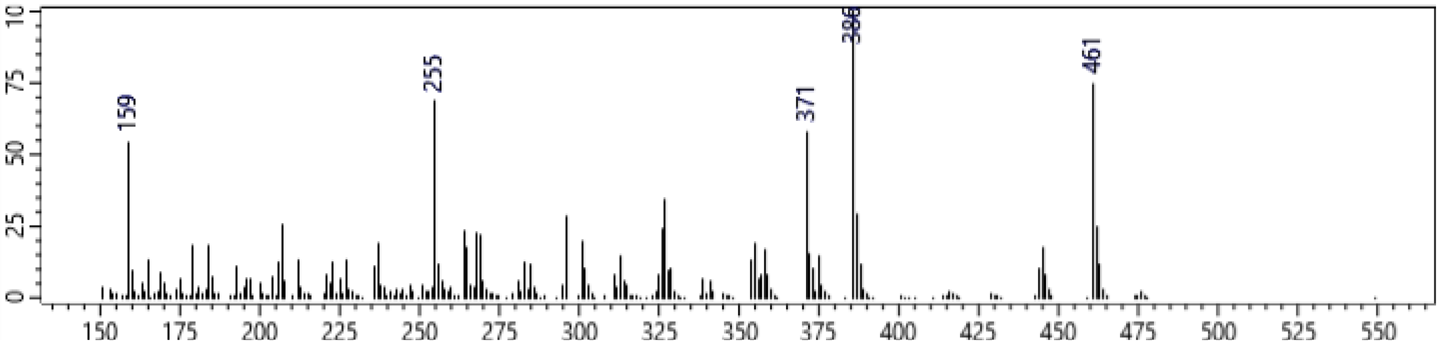

7,18-Dihydroxykaurenolide, 2TMS derivative

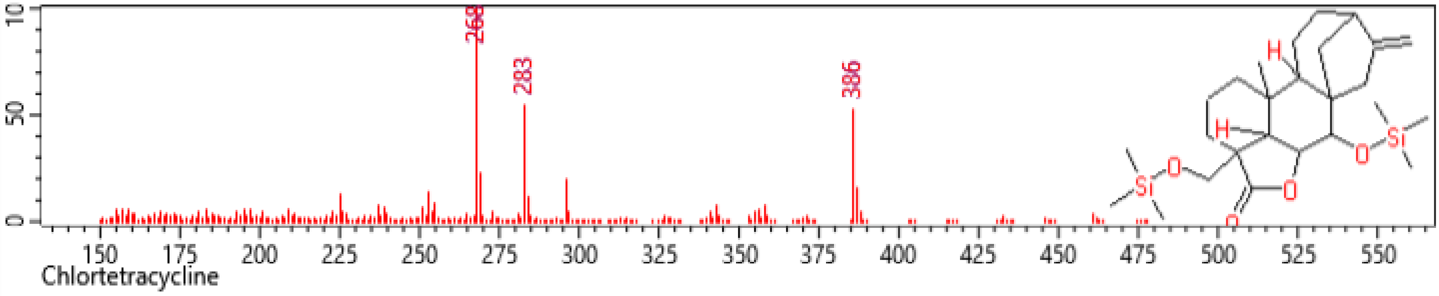

Chlortetracycline

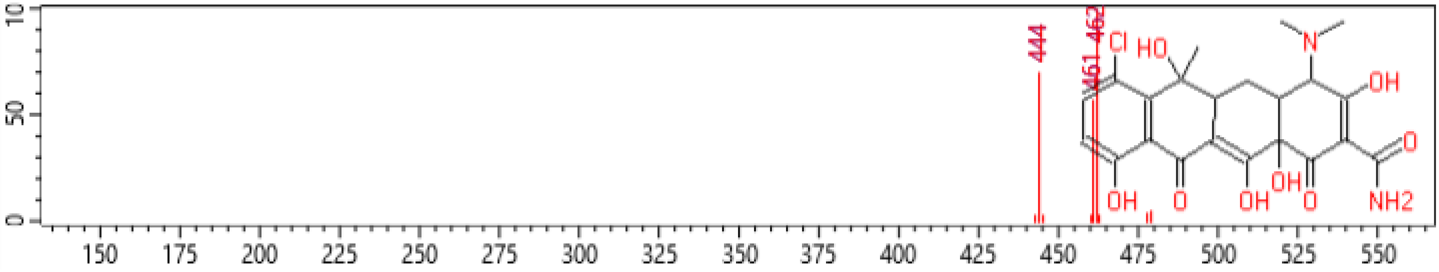

Pregn-5-en-20-one, 3,16-bis[(trimethylsilyl)oxy]-, (3β,16α)-

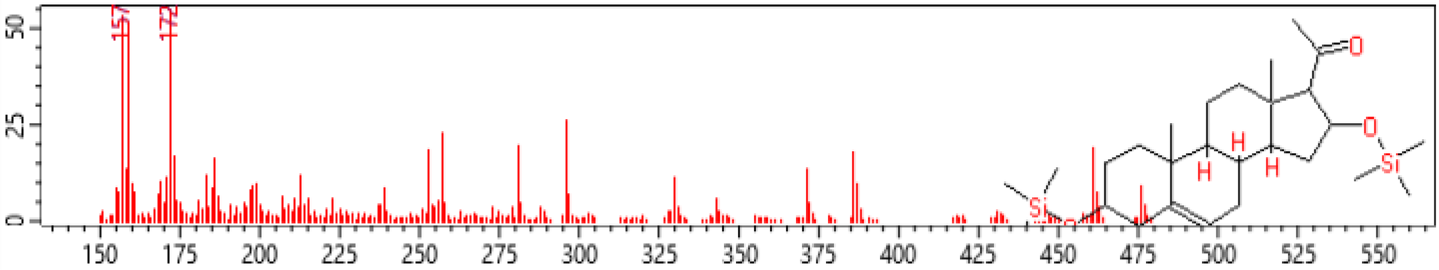

17-Trifluoromethylphenyltrinorprostaglandin F2α

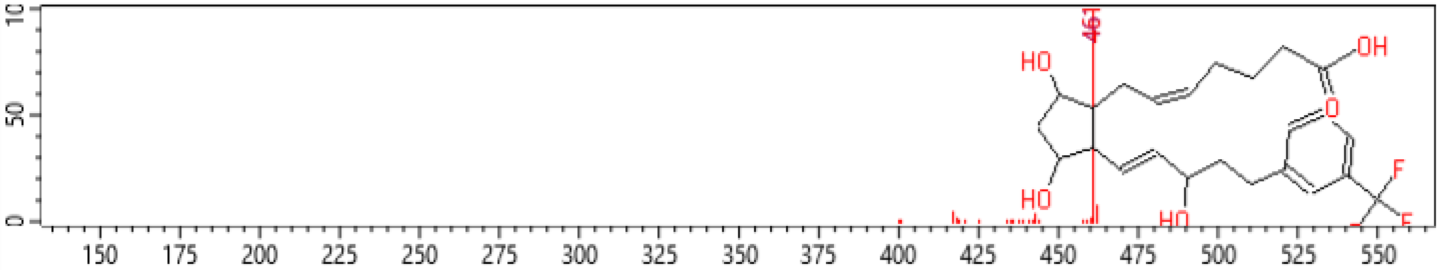

Chagreslactone, TMS derivative

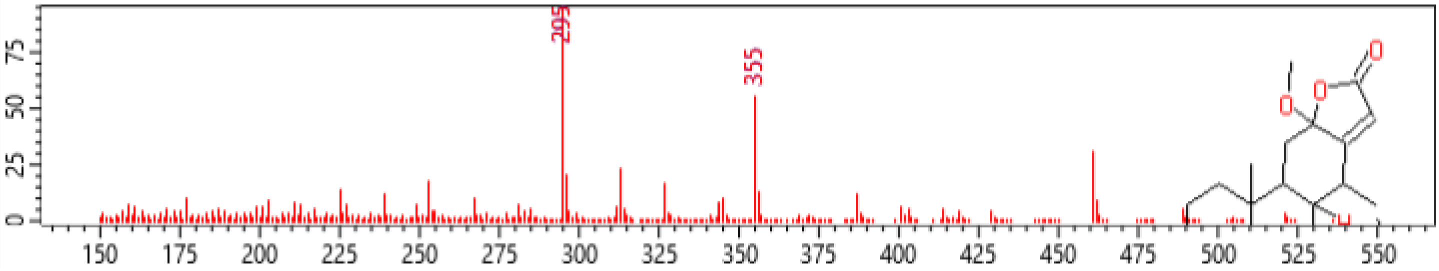

5-Hydroxy-3-(5-hydroxy-2,4-dimethoxyphenyl)-6-methoxy-4-oxo-4H-chromen-7-yl hexopyranoside

Peak: 15.41 - 15.46 min (scan #2348 - #2360) Subtracted noise: 15.33 - 15.37 min (scan #2324 - #2336), 15.47 - 15.51 min (scan #23

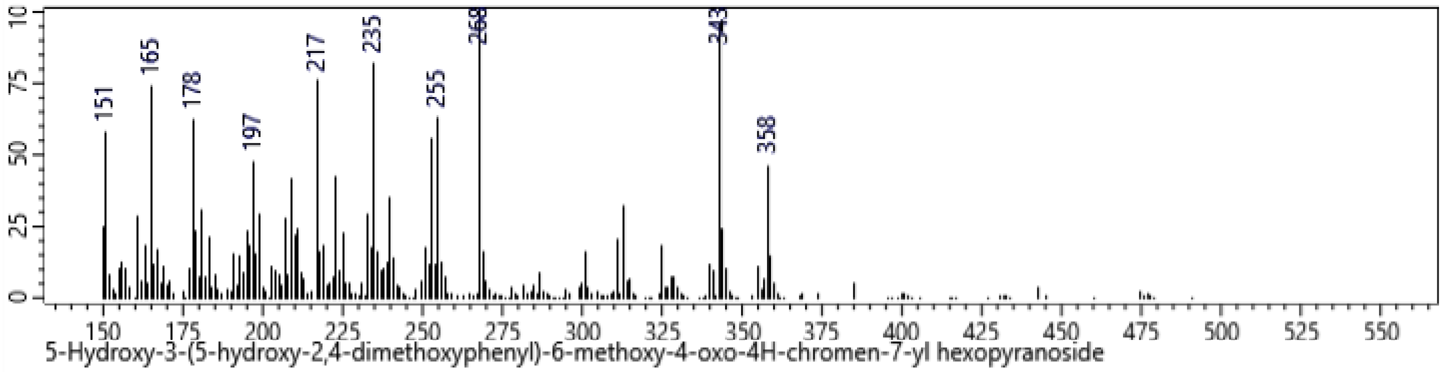

5-Hydroxy-3-(5-hydroxy-2,4-dimethoxyphenyl)-6-methoxy-4-oxo-4H-chromen-7-yl hexopyranoside

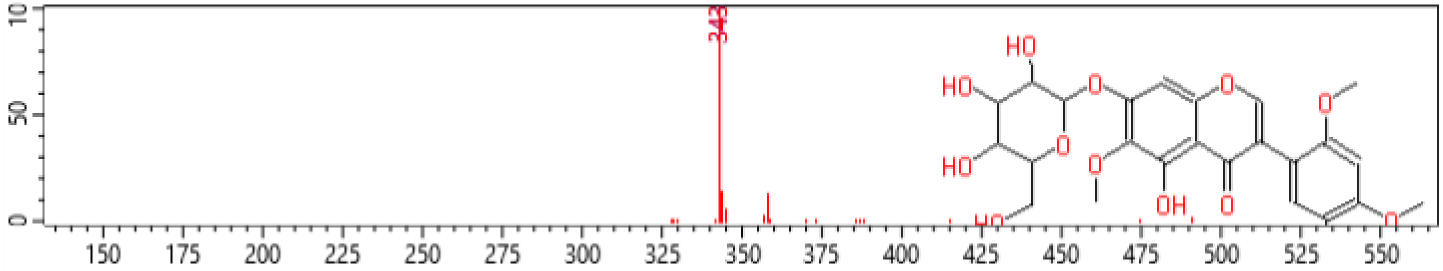

Androstan-4,6-diene-17β-ol-3-one, trimethylsilyl ether

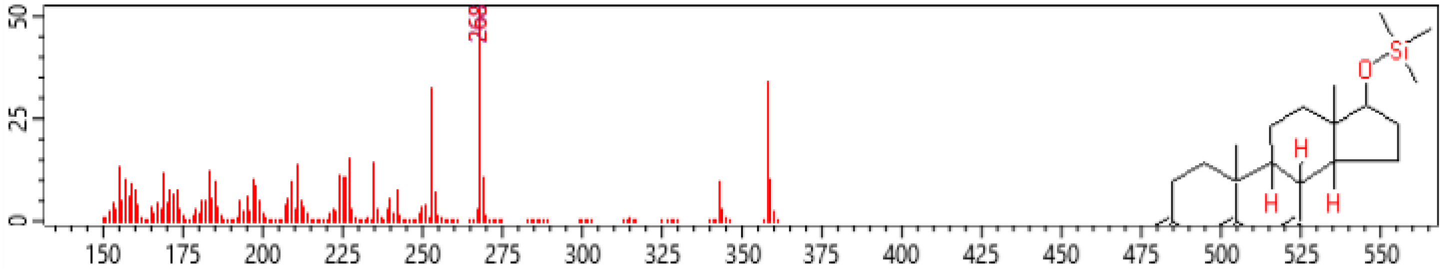

Boldenone, TMS derivative

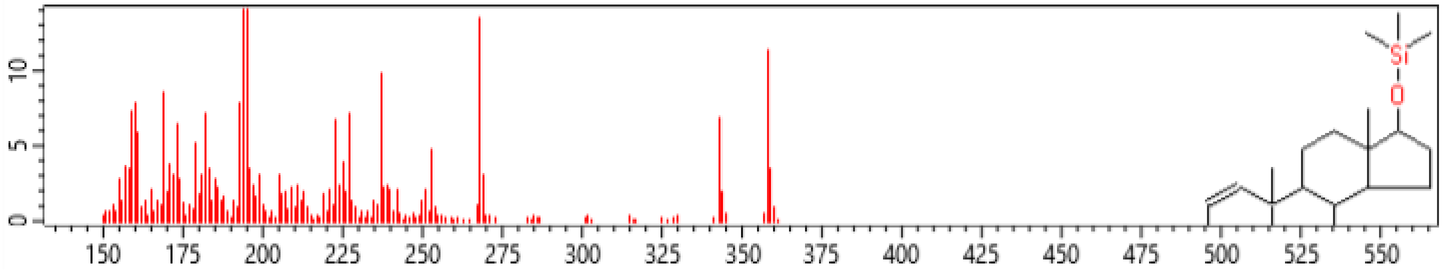

4-Androsten-7α, 17β-bis[(trimethylsilyl)oxy]-3-one

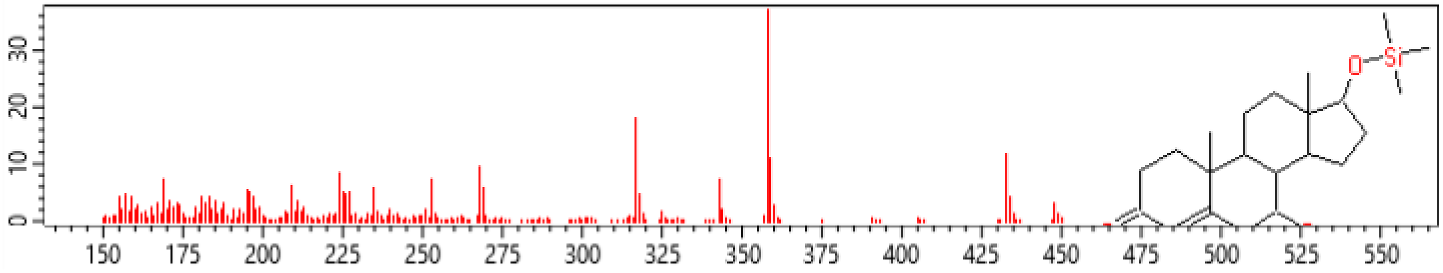

N-(3-Methoxyphenyl)-2-methyl-3-nitrobenzamide, TMS derivative

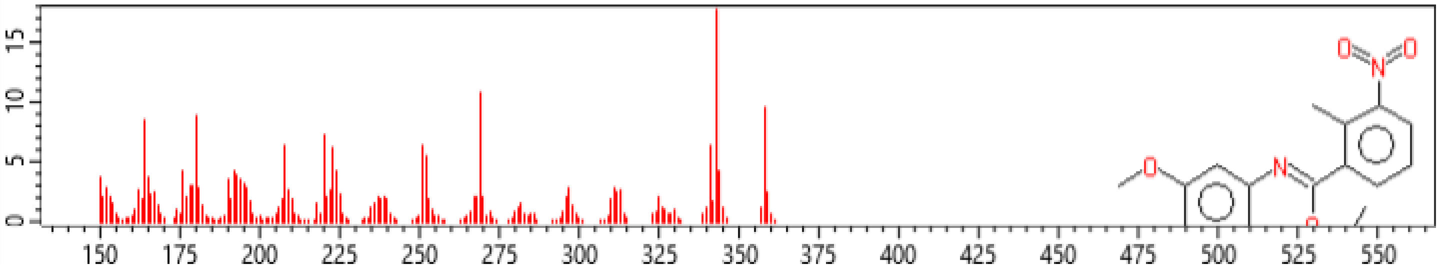

Prostaglandin A3

Peak: 15.56 - 15.64 min (scan #2388 - #2410) Subtracted noise: 15.39 - 15.46 min (scan #2341 - #2362), 15.63 - 15.71 min (scan #24

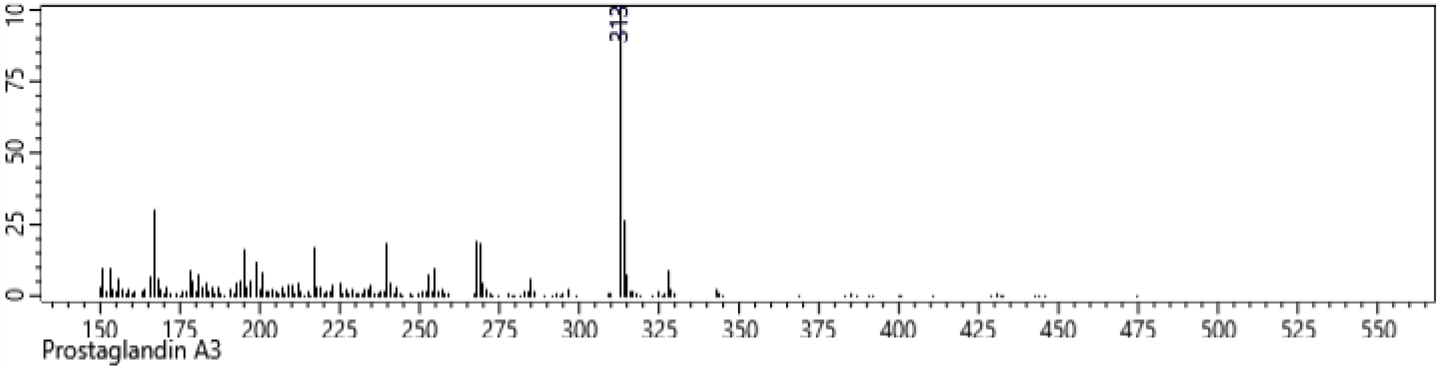

Prostaglandin A3

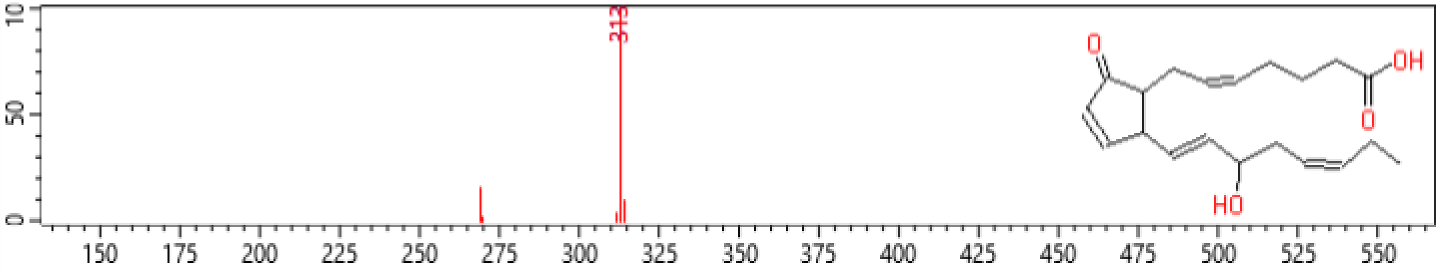

Prostaglandin A3

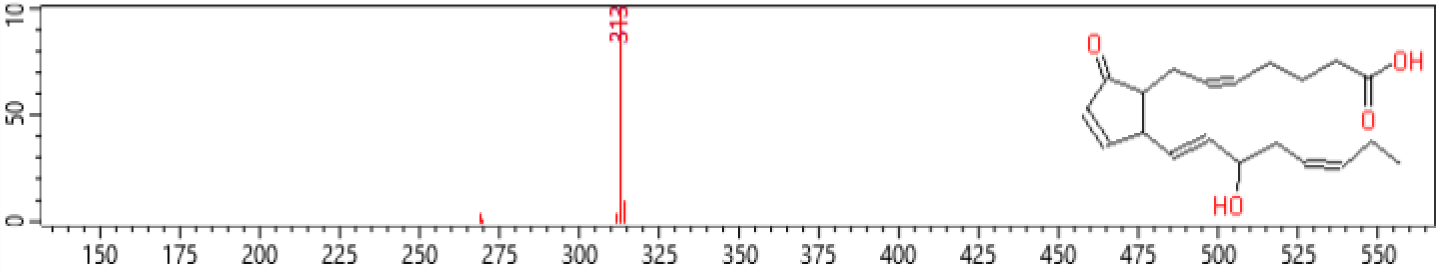

Prostaglandin B3

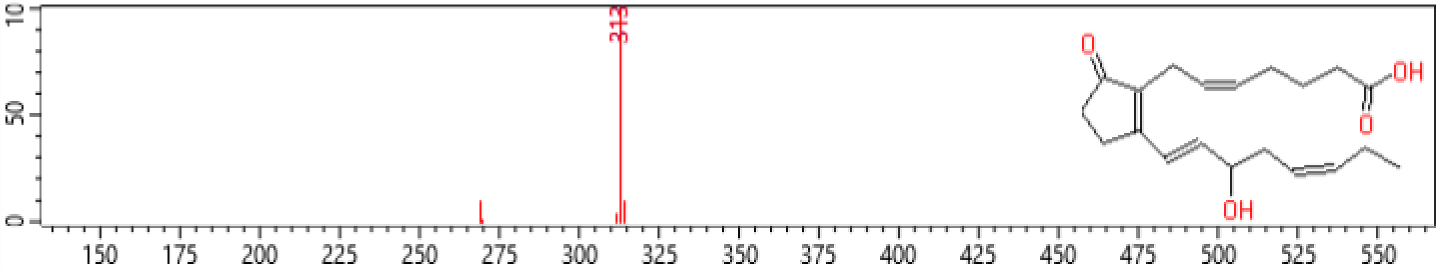

Prostaglandin B3

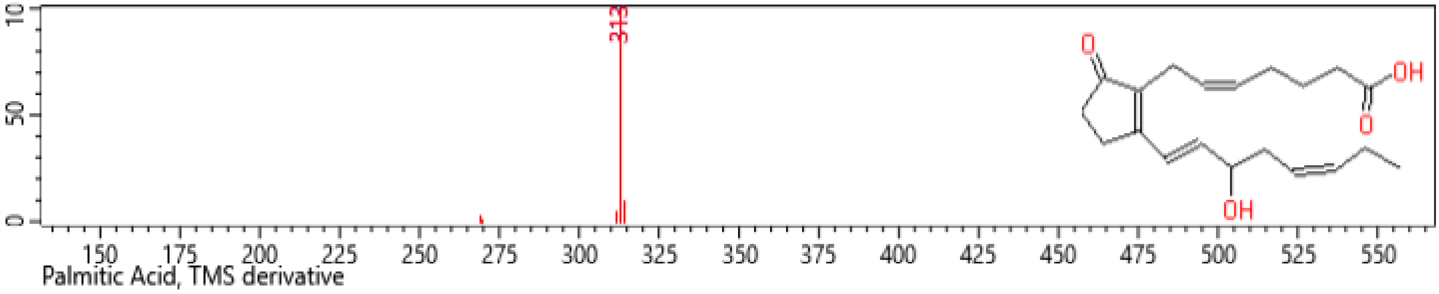

Palmitic Acid, TMS derivative

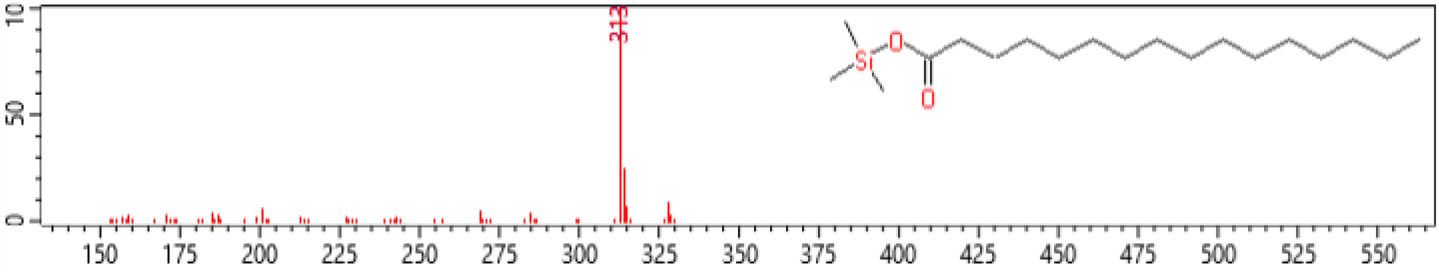

Ribavirin

Peak: 15.65 - 15.67 min (scan #2414 - #2419) Subtracted noise: 15.61 - 15.63 min (scan #2402 - #2408), 15.68 - 15.69 min (scan #24

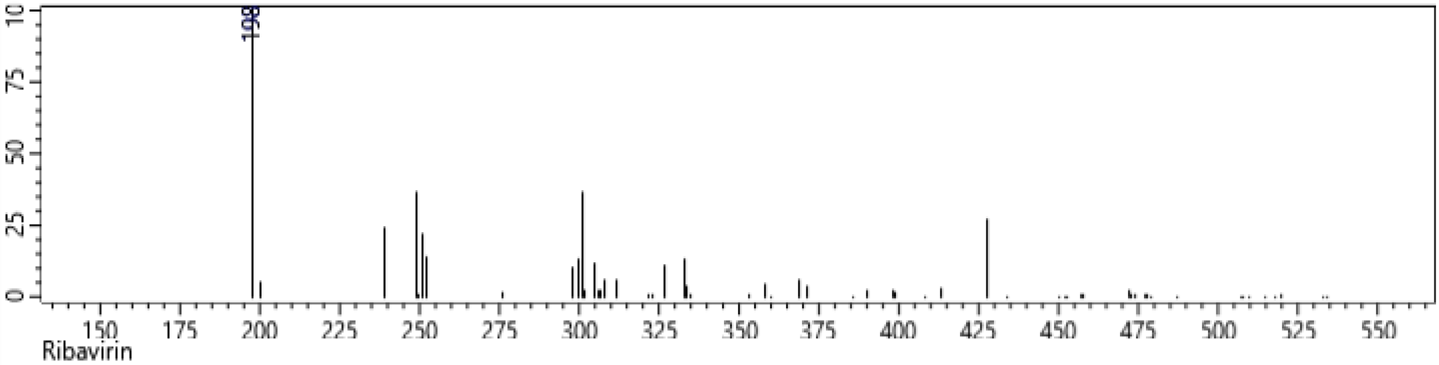

Ribavirin

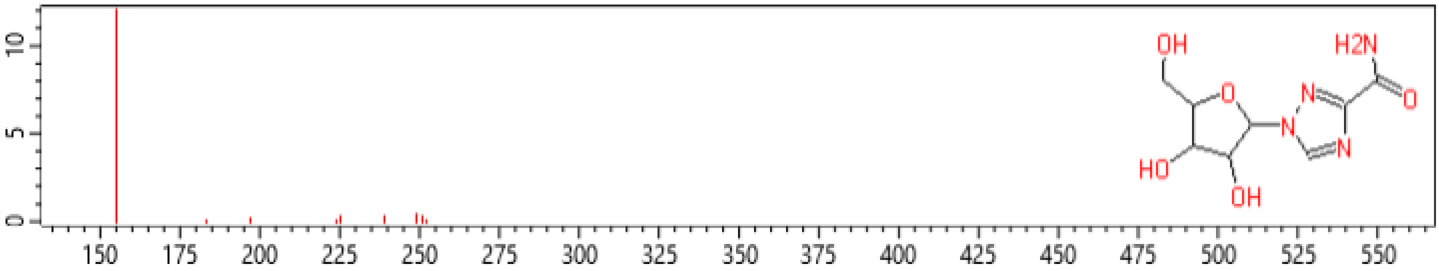

Bifenazate

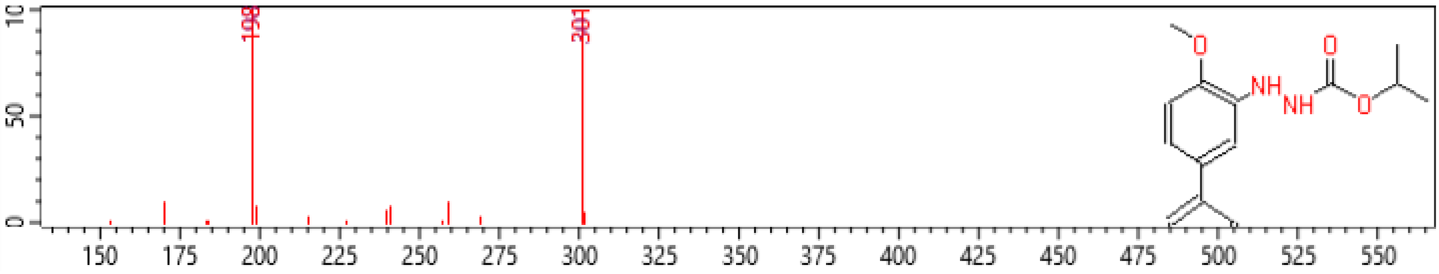

Benzo[1,2-b:4,5-b']difuran-4-ethanamine, 8-bromo-α-methyl-

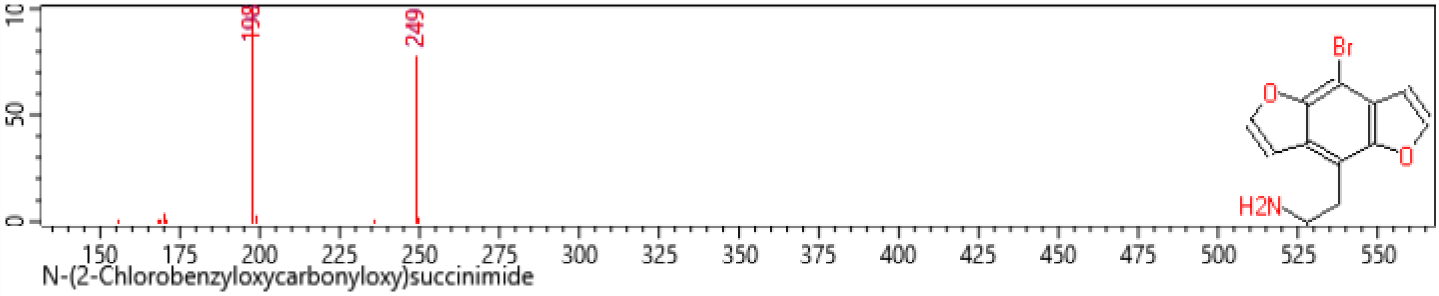

N-(2-Chlorobenzyloxycarbonyloxy)succinimide

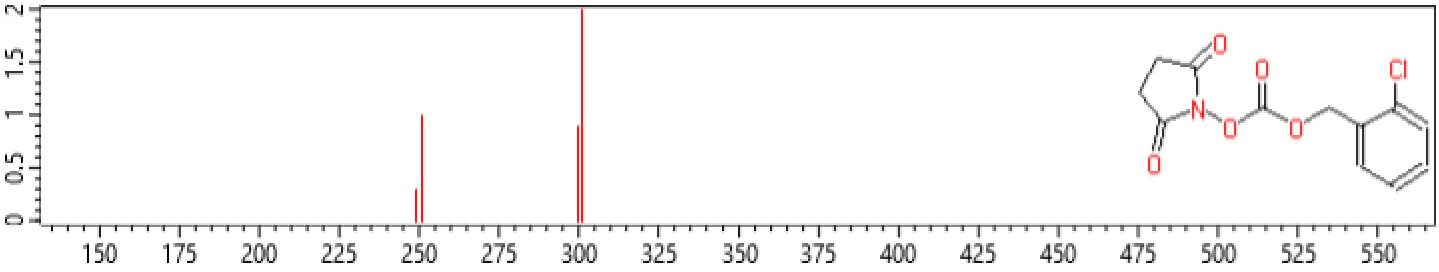

4,6'-Dihydroxy-2,2',4',6-tetrachlorobiphenyl

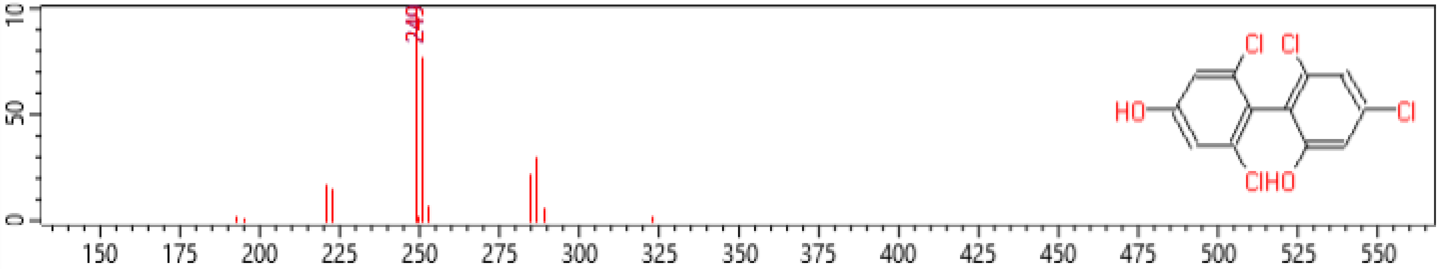

7-Ethenyl-1,1,7-trimethyl-9-oxo-2,3,4,4b,5,6,10,10a-octahydrophenanthrene-4a-carboxylic acid, 2TMS

Peak: 15.68 - 15.70 min (scan #2423 - #2427) Subtracted noise: 15.65 - 15.67 min (scan #2415 - #2420), 15.71 - 15.73 min (scan #24

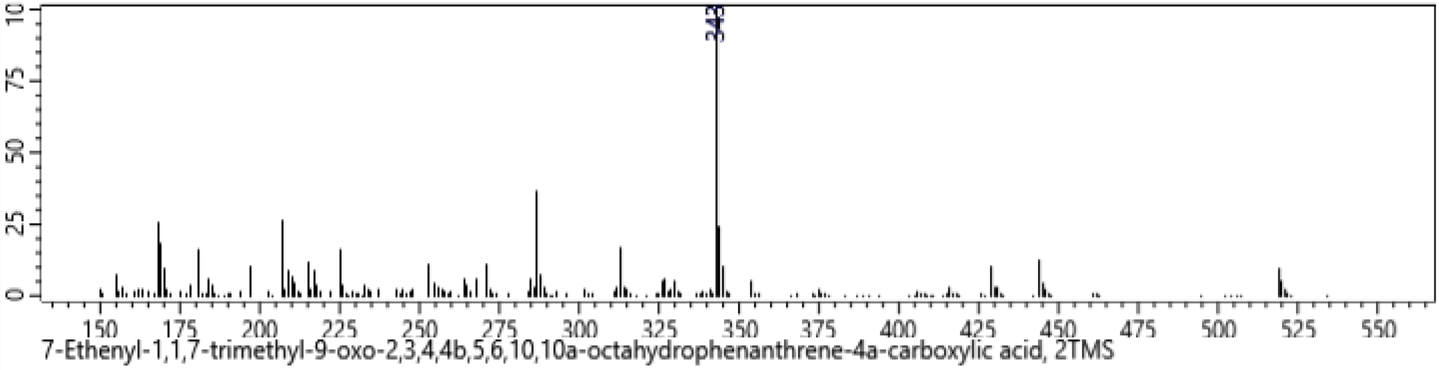

7-Ethenyl-1,1,7-trimethyl-9-oxo-2,3,4,4b,5,6,10,10a-octahydrophenanthrene-4a-carboxylic acid, 2TMS

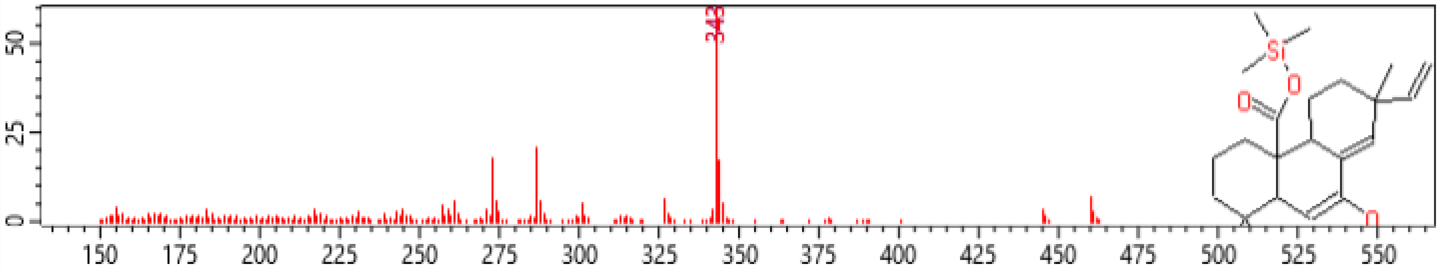

Bis(4-hydroxyphenyl)acetic acid, 3TMS

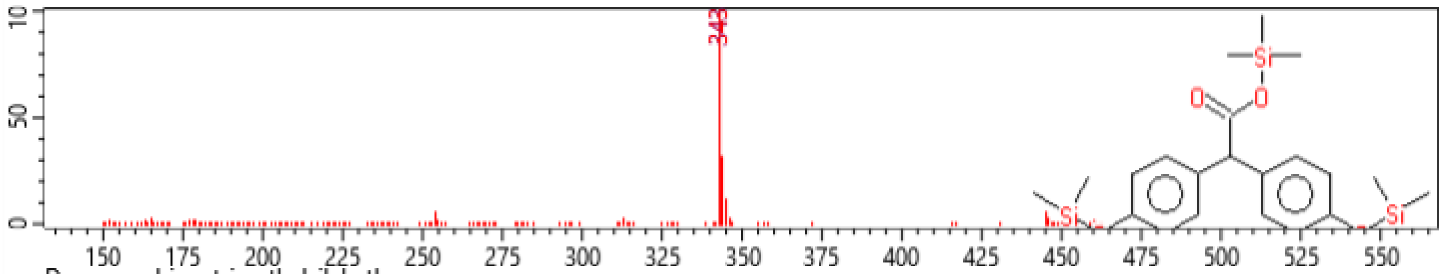

Desomorphine, trimethylsilyl ether

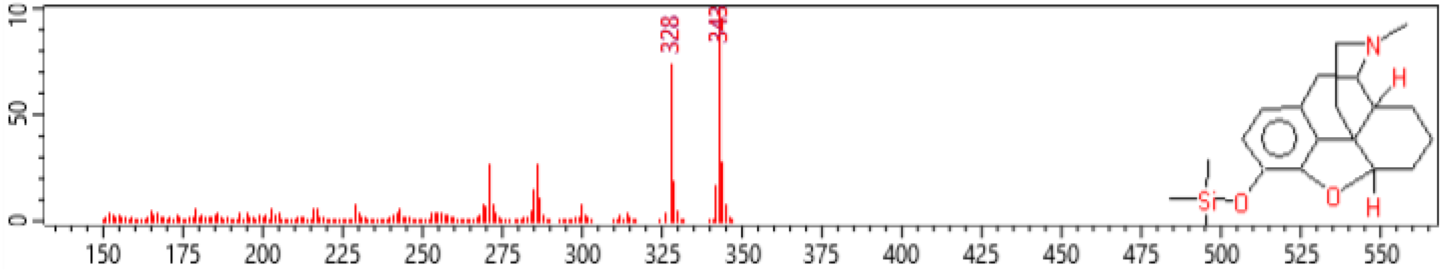

Austalide A

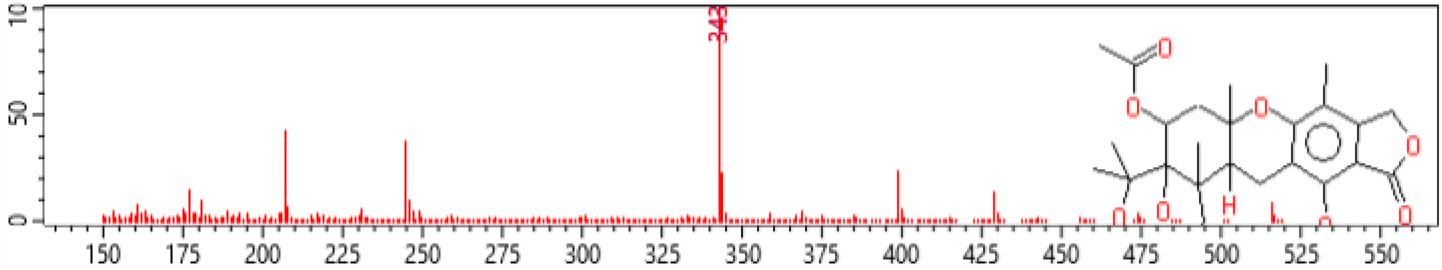

2,4'-Dihydroxybenzophenone, bis(trimethylsilyl) ether

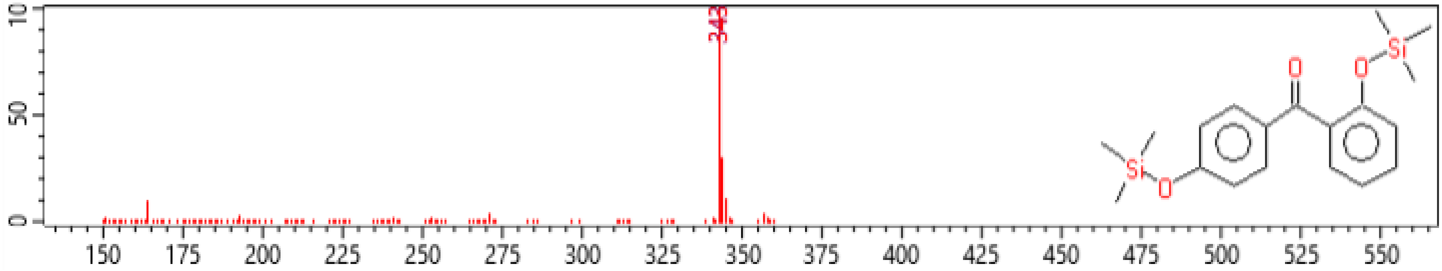

5-Hydroxy-7,8-dimethoxyflavanone, TMS derivative

Peak: 15.75 - 15.80 min (scan #2443 - #2455) Subtracted noise: 15.67 - 15.71 min (scan #2420 - #2430), 15.80 - 15.84 min (scan #24

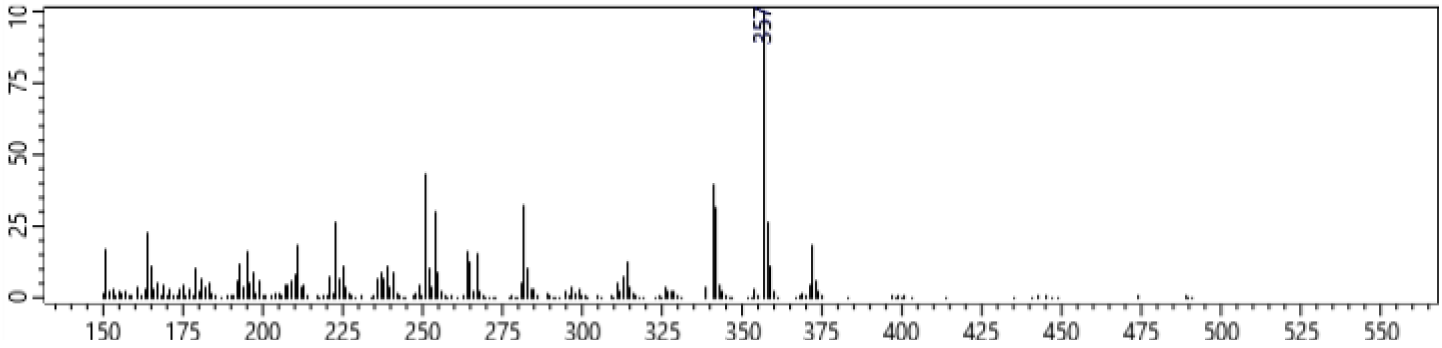

5-Hydroxy-7,8-dimethoxyflavanone, TMS derivative

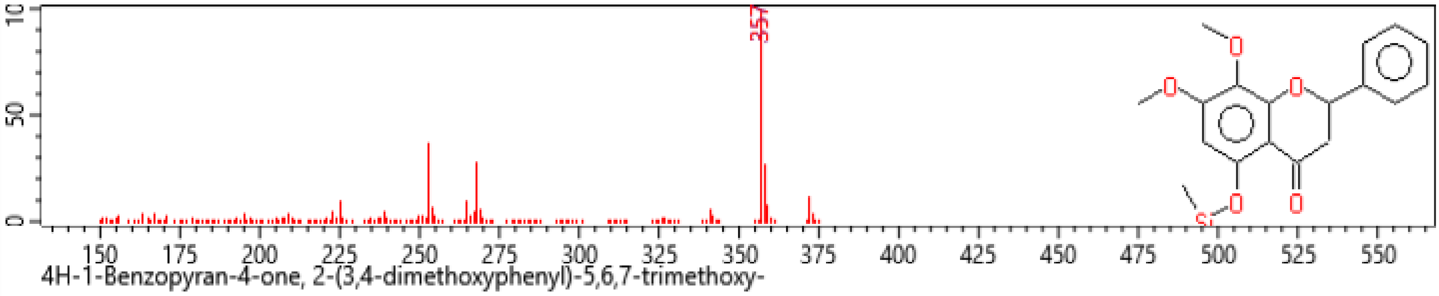

4H-1-Benzopyran-4-one, 2-(3,4-dimethoxyphenyl)-5,6,7-trimethoxy-

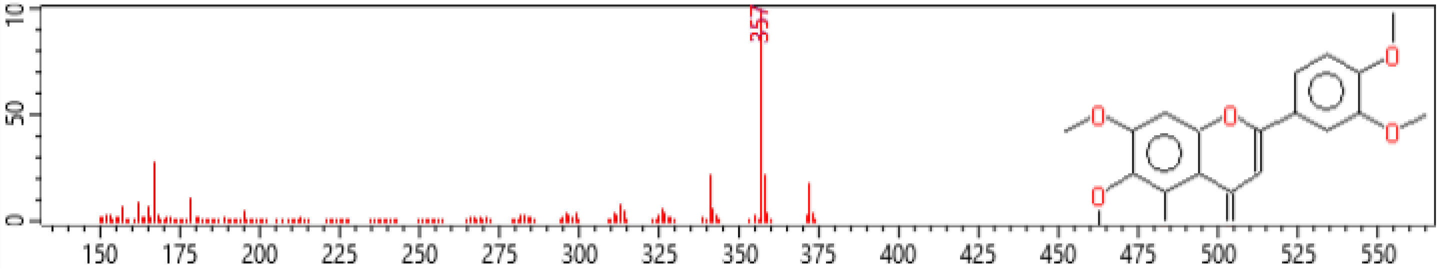

3,2',4',5',6-Pentamethoxyflavone

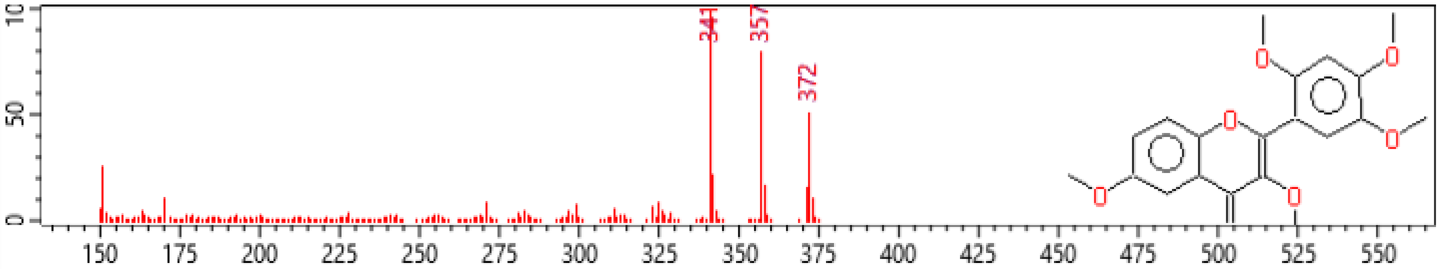

Methyl 4-hydroxy-5-[2-(hydroxymethyl)-3-methoxy-5-methylphenoxy]-2-methoxy-3,6-dimethylbenzoate, 2Me derivative

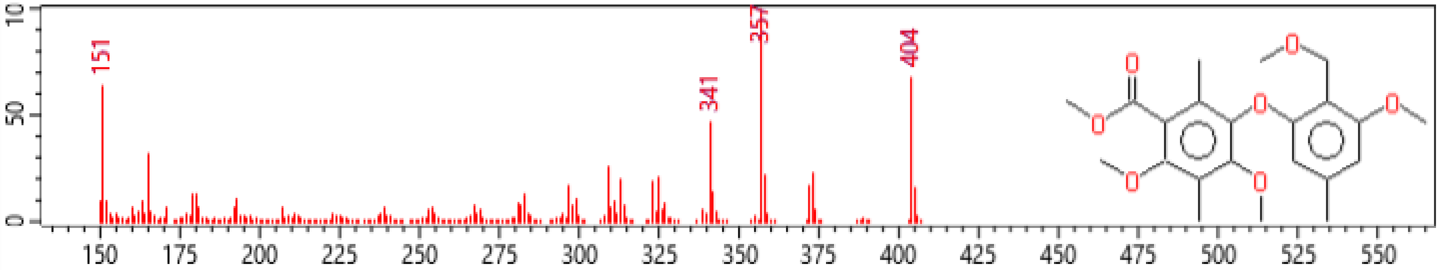

3,7,2',4',5'-Pentamethoxyflavone

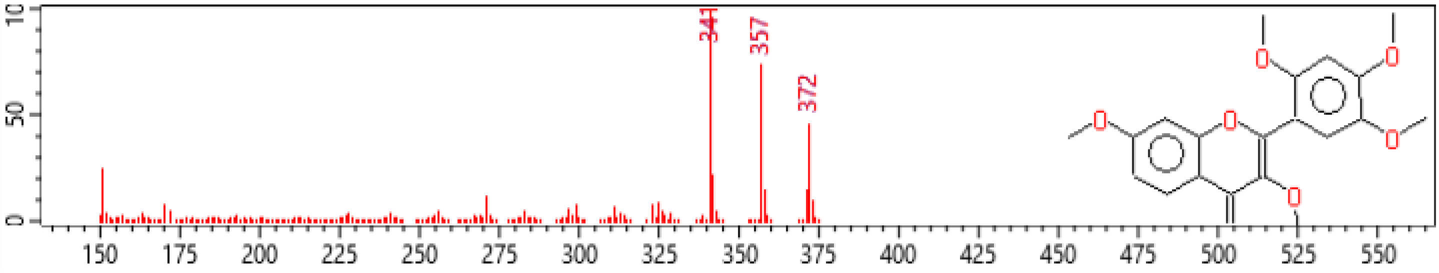

Calphostin C

Peak: 15.85 - 15.87 min (scan #2469 - #2475) Subtracted noise: 15.81 - 15.83 min (scan #2458 - #2464), 15.88 - 15.91 min (scan #24

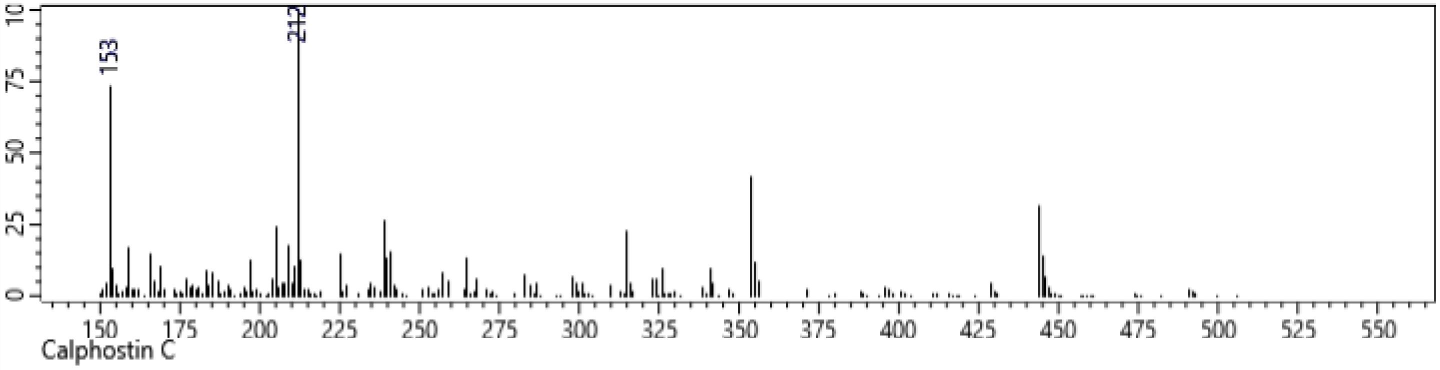

Calphostin C

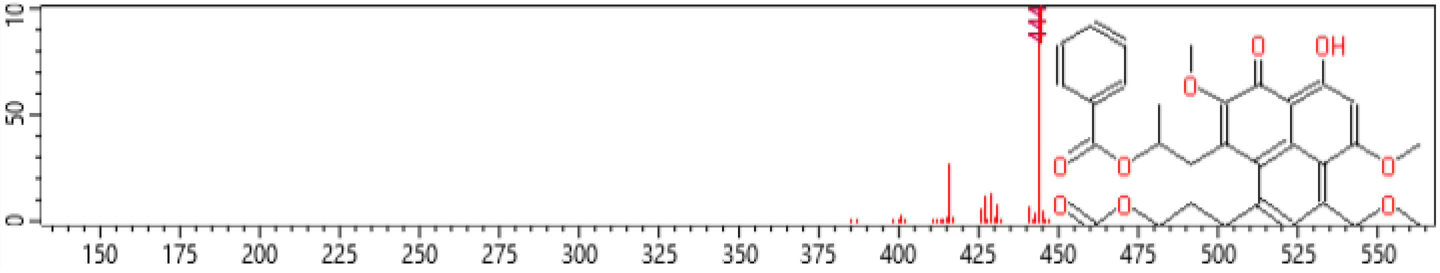

1H-Cyclopropa[3,4]benz[1,2-e]azulene-2,5-dione, 9,9a-bis(acetyloxy)-3-[(acetyloxy)methyl]-1a,1b,4a,7a,7b,8,9,9a-octahydro-4a,7b-di

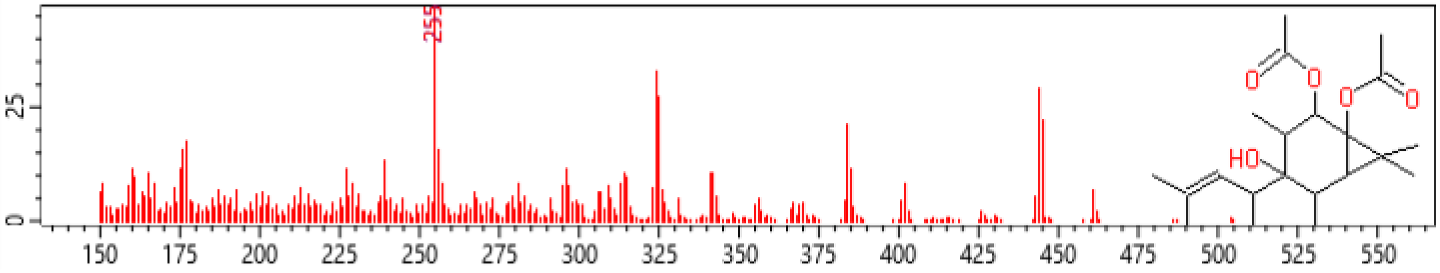

N-(2,6-Dimethylphenyl)-2-(5-methylthiophen-2-yl)quinoline-4-carboxamide, TMS derivative

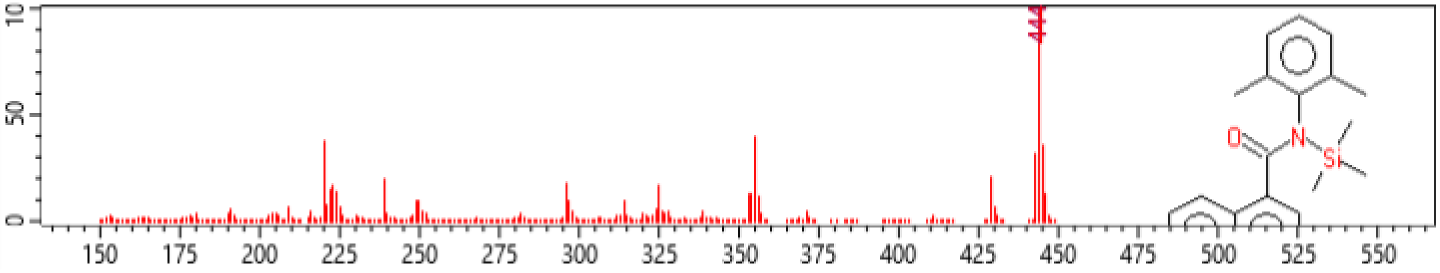

Xysmalogenin, TMS derivative

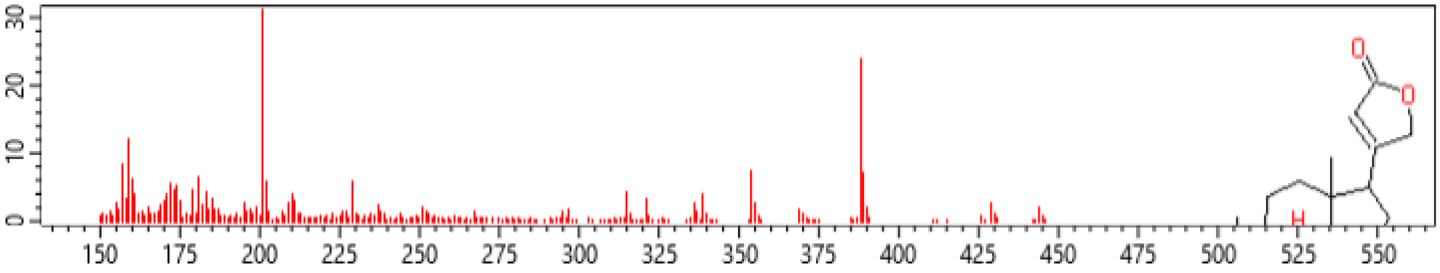

Spiro(1,2,3,4,4a,4b,5,6,7,8,8a,9-dodecahydro-4a-methyl-5-hydroxy-7,7-di(allyl)-phenanthren-8-one)-2,2'-(1',3'-dioxolane), TMS deriv

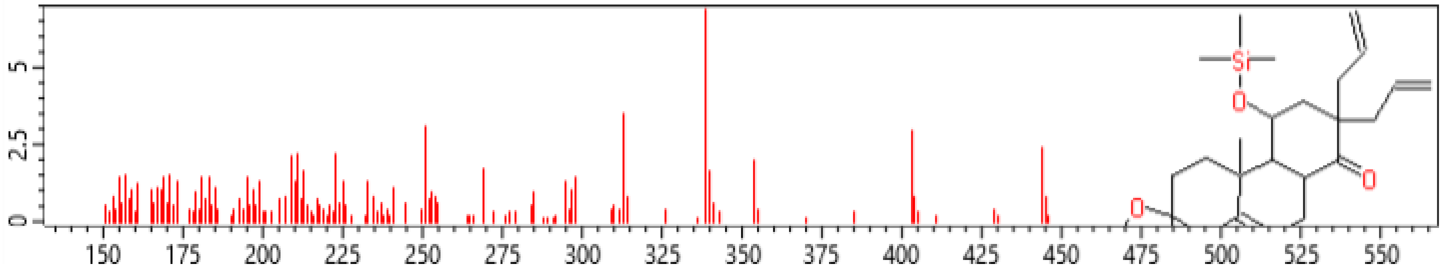

Pyrido[2,3-d]pyrimidin-7(8H)-one, 6-(2,6-dichlorophenyl)-2-[[3-(hydroxymethyl)phenyl]amino]-8-methyl-
Peak: 15.91 - 15.93 min (scan #2485 - #2493) Subtracted noise: 15.85 - 15.88 min (scan #2471 - #2478), 15.94 - 15.97 min (scan #24

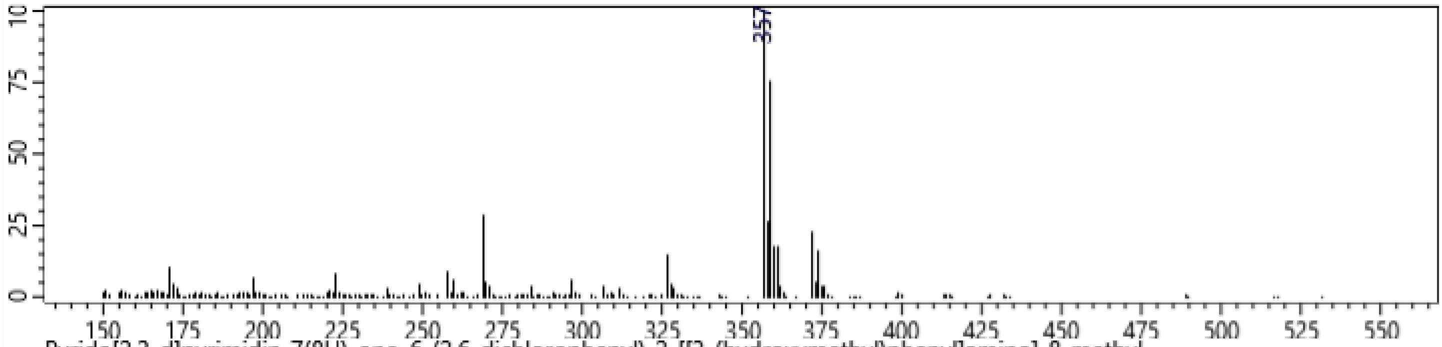

Pyrido[2,3-d]pyrimidin-7(8H)-one, 6-(2,6-dichlorophenyl)-2-[[3-(hydroxymethyl)phenyl]amino]-8-methyl-

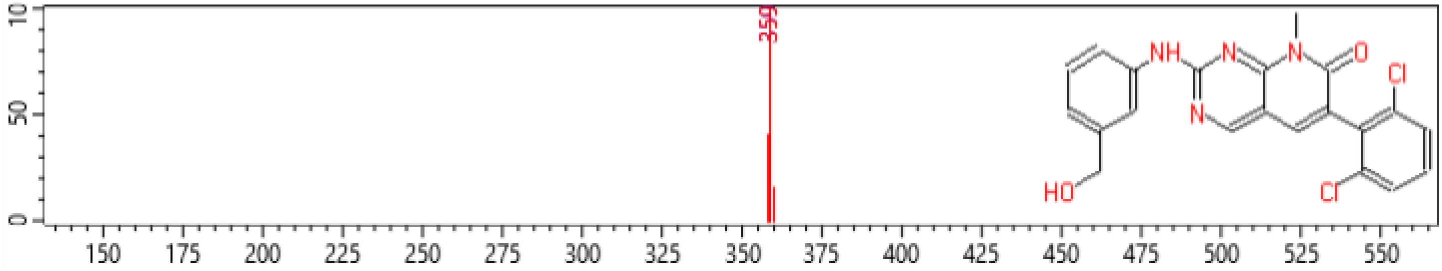

6-Bromo-1,3-benzothiazol-2-amine, N,N-bis(trimethylsilyl)-

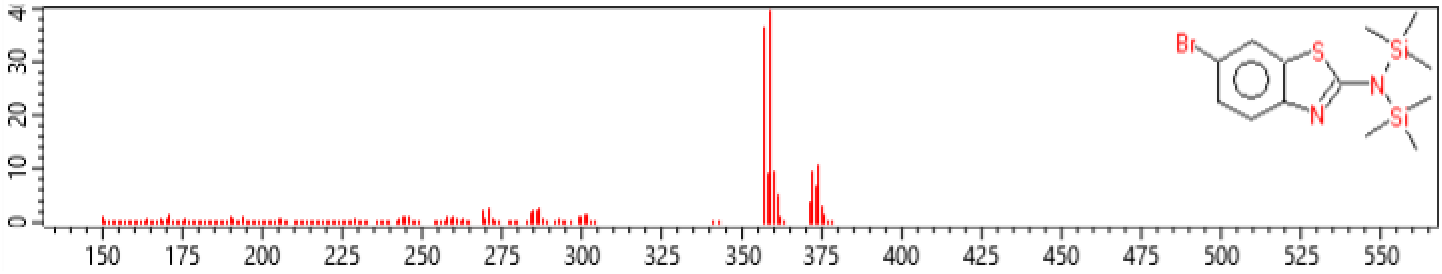

Bisphenol A, 2TMS derivative

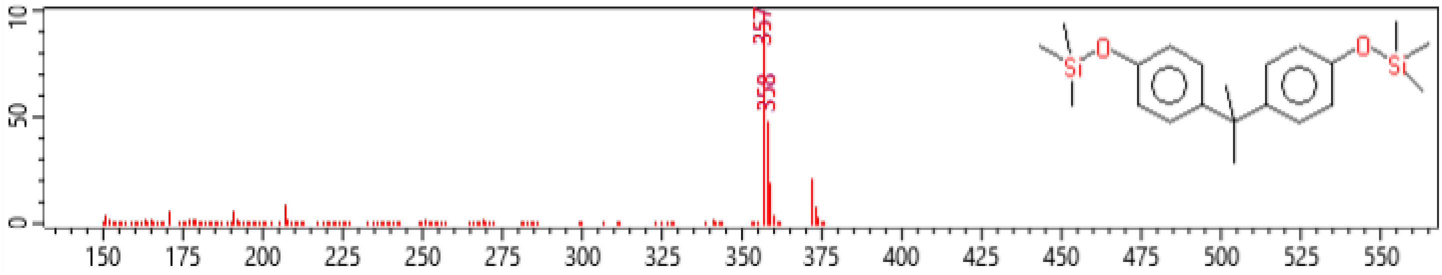

3-Bromo-4-methylbenzene-1-carbohydrazonic acid, 2 TMS derivative

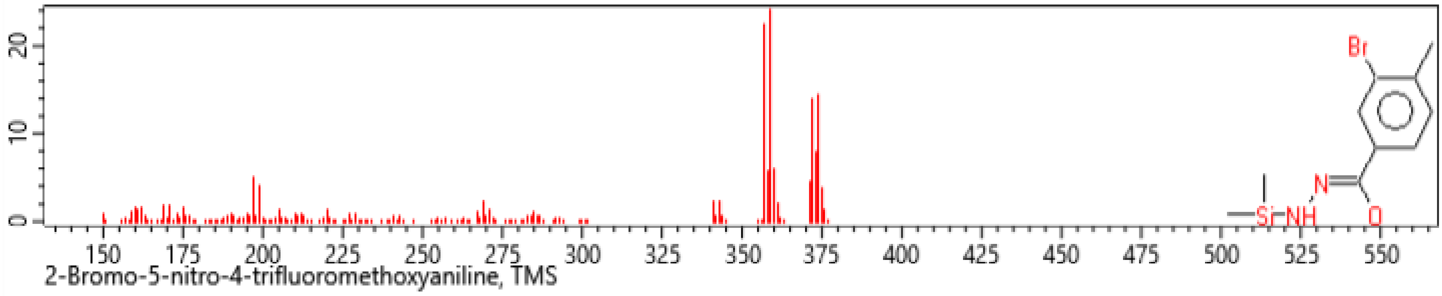

2-Bromo-5-nitro-4-trifluoromethoxyaniline, TMS

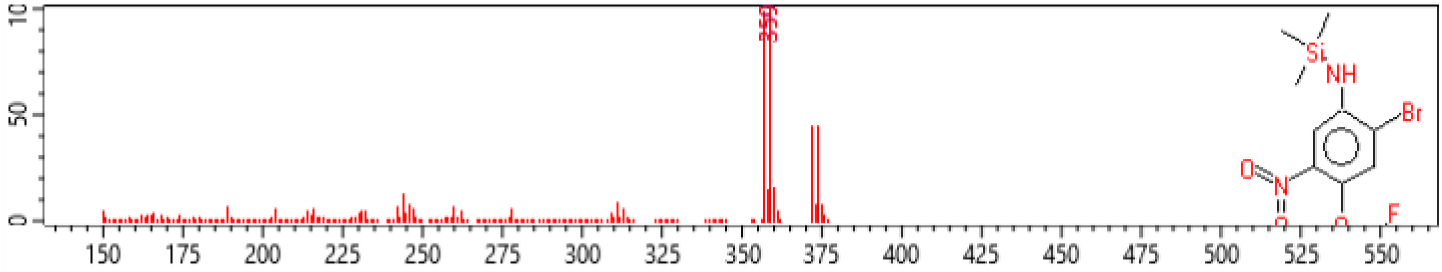

4-epi-Oxytetracycline

Peak: 15.96 - 15.98 min (scan #2500 - #2506) Subtracted noise: 15.92 - 15.94 min (scan #2489 - #2495), 15.99 - 16.02 min (scan #25

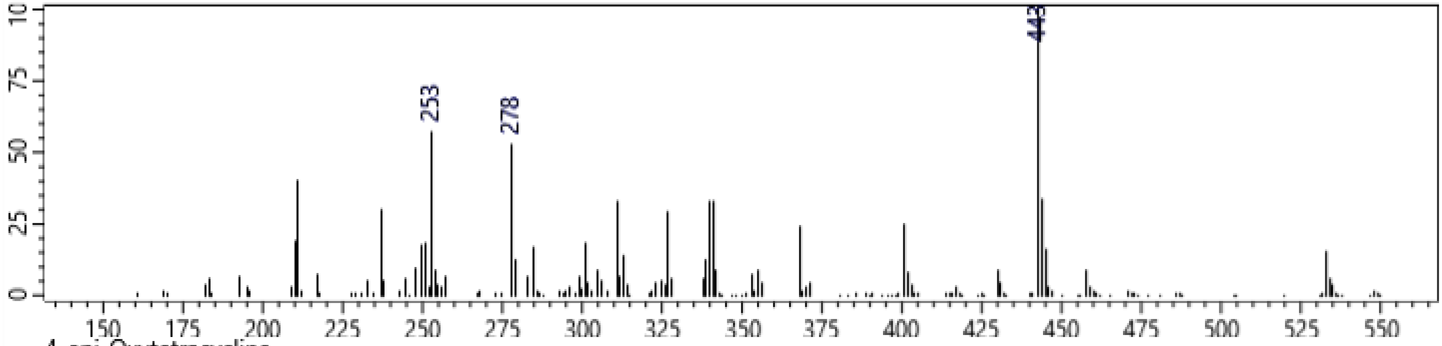

4-epi-Oxytetracycline

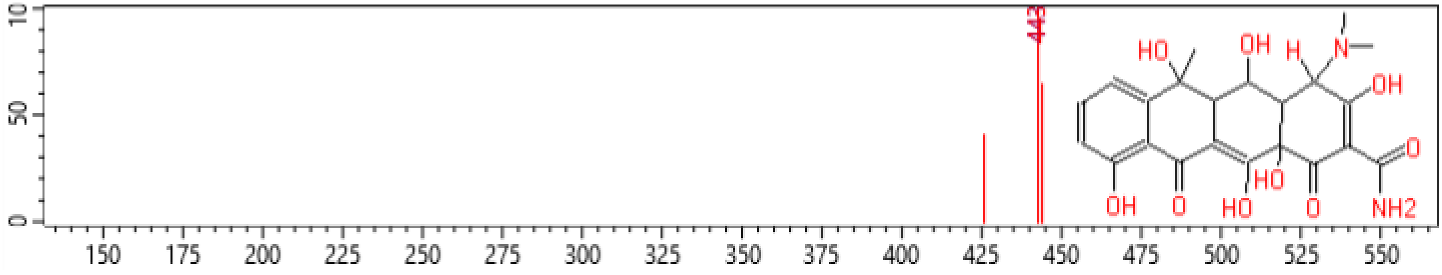

Rhodamine B cation

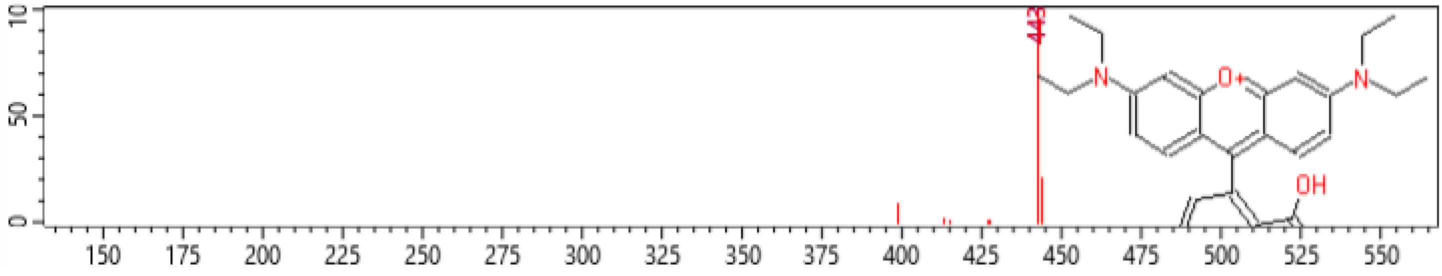

Apixaban

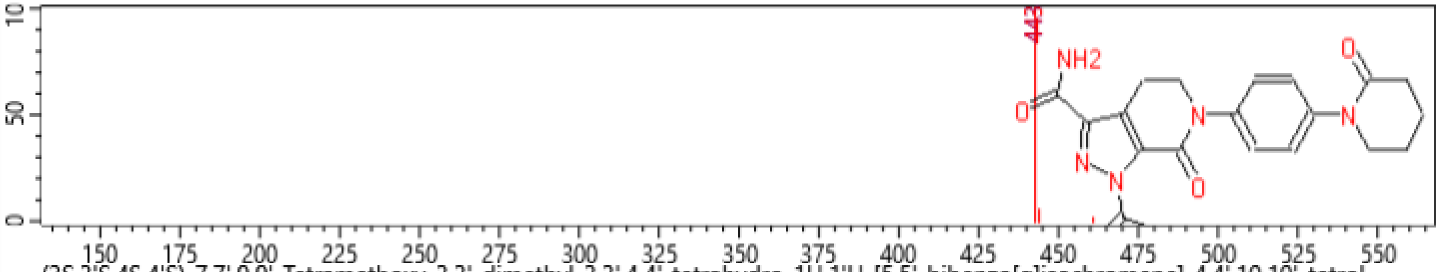

(3S,3'S,4S,4'S)-7,7',9,9'-Tetramethoxy-3,3'-dimethyl-3,3',4,4'-tetrahydro-1H,1'H-[5,5'-bibenzo[g]isochromene]-4,4',10,10'-tetrol

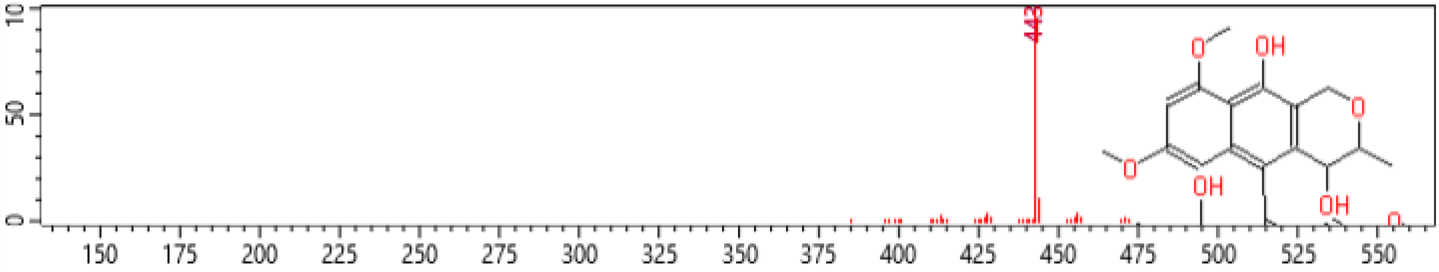

1-(1Z-Hexadecenyl)-sn-glycero-3-phosphocholine

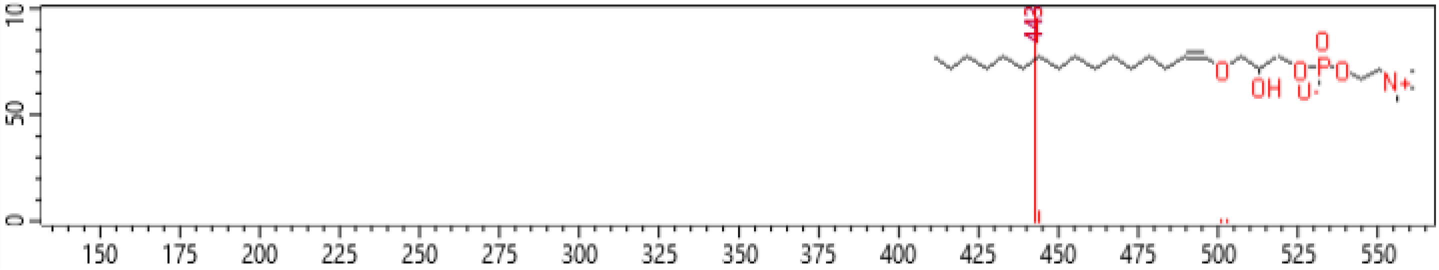

1-Propanamine, N,N-dimethyl-3-[[1-(phenylmethyl)-1H-indazol-3-yl]oxy]-

Peak: 16.01 - 16.06 min (scan #2515 - #2527) Subtracted noise: 15.95 - 15.99 min (scan #2497 - #2509), 16.09 - 16.13 min (scan #25

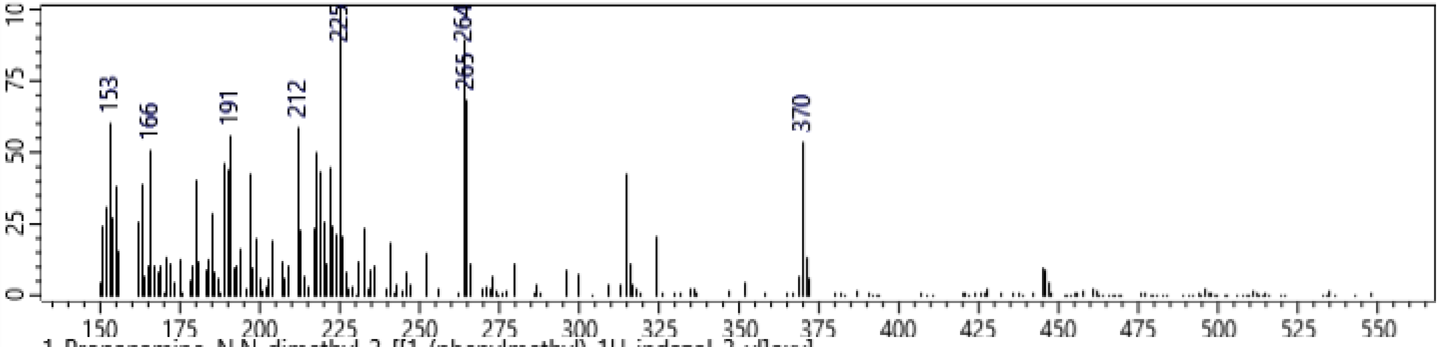

1-Propanamine, N,N-dimethyl-3-[[1-(phenylmethyl)-1H-indazol-3-yl]oxy]-

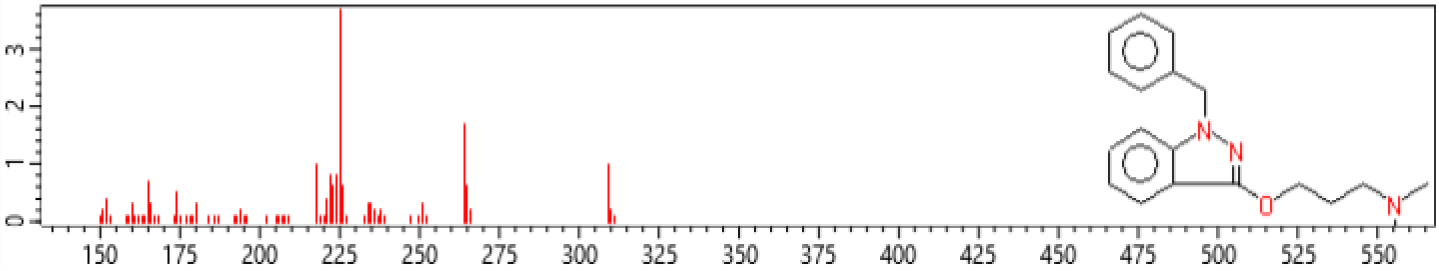

1-Propanamine, N,N-dimethyl-3-[[1-(phenylmethyl)-1H-indazol-3-yl]oxy]-

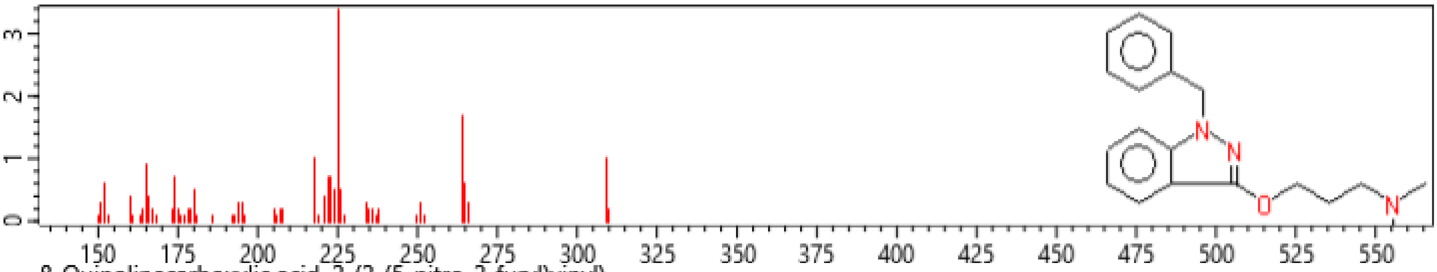

8-Quinolinecarboxylic acid, 2-(2-(5-nitro-2-furyl)vinyl)-

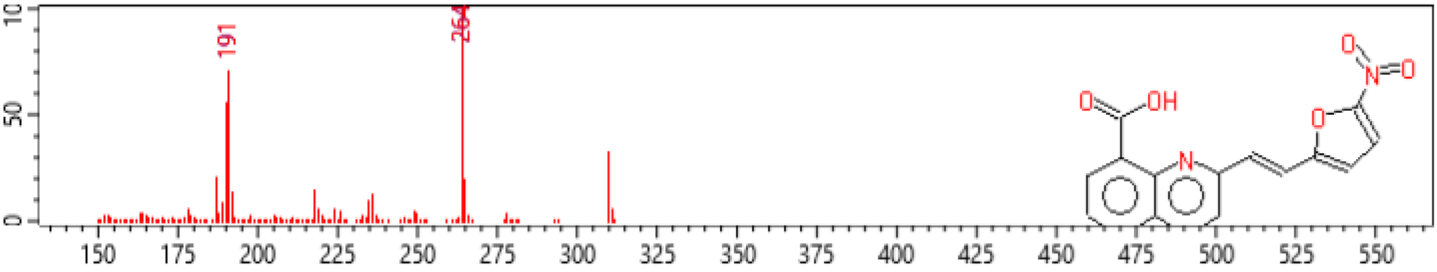

Sericic acid

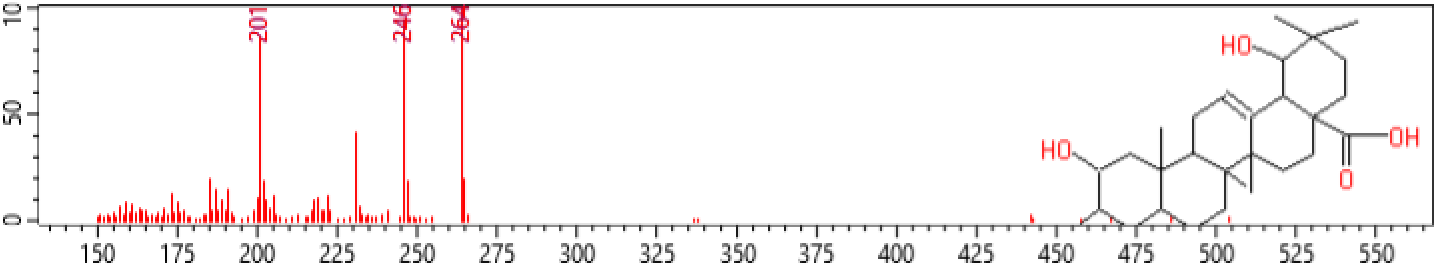

Perhydrobenzo[a]cycloheptene-6,8-dicarboxylic acid, perhydro-7-oxo-, diethyl ester

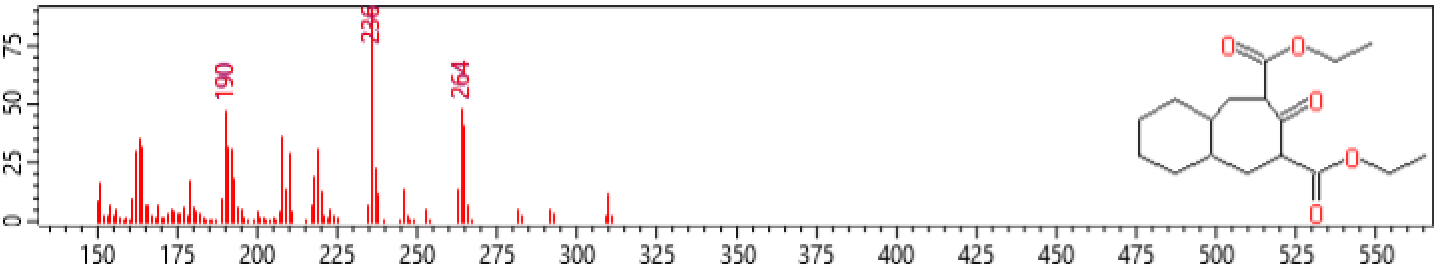

Matairesinoside

Peak: 16.15 - 16.20 min (scan #2554 - #2568) Subtracted noise: 16.04 - 16.09 min (scan #2523 - #2536), 16.20 - 16.25 min (scan #25

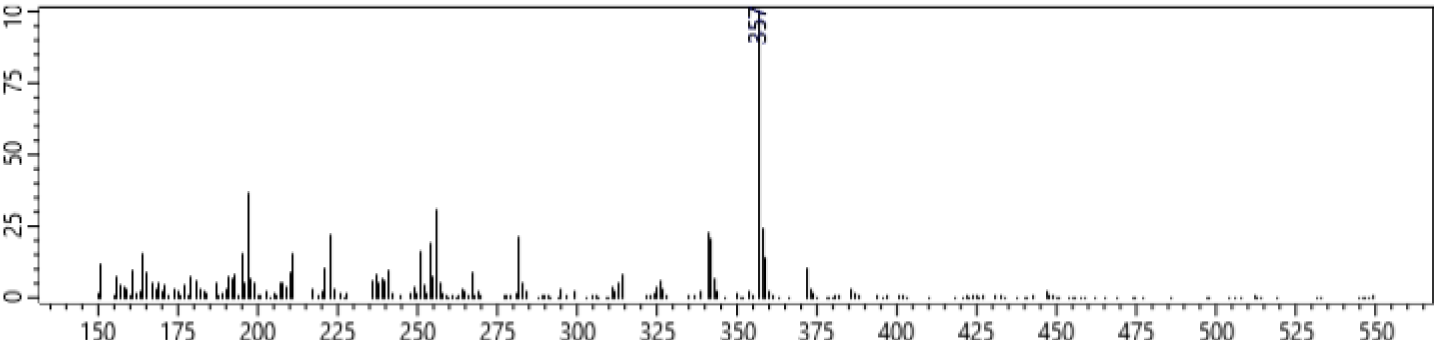

Matairesinoside

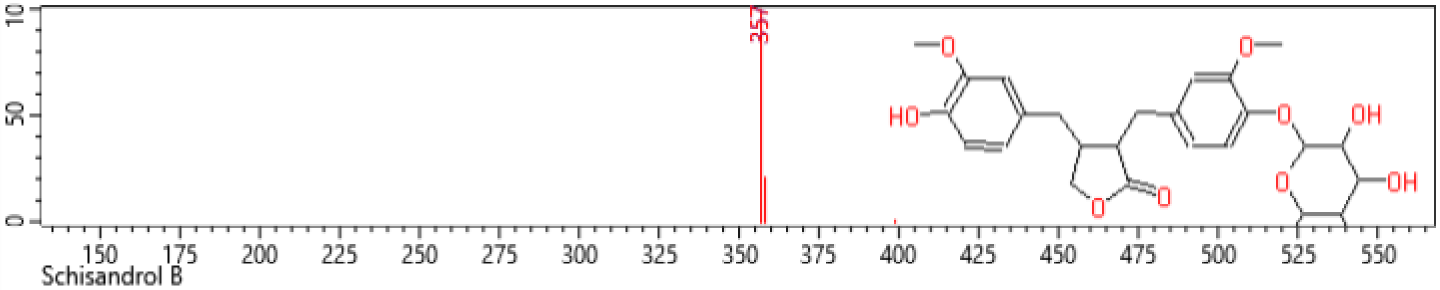

Schisandrol B

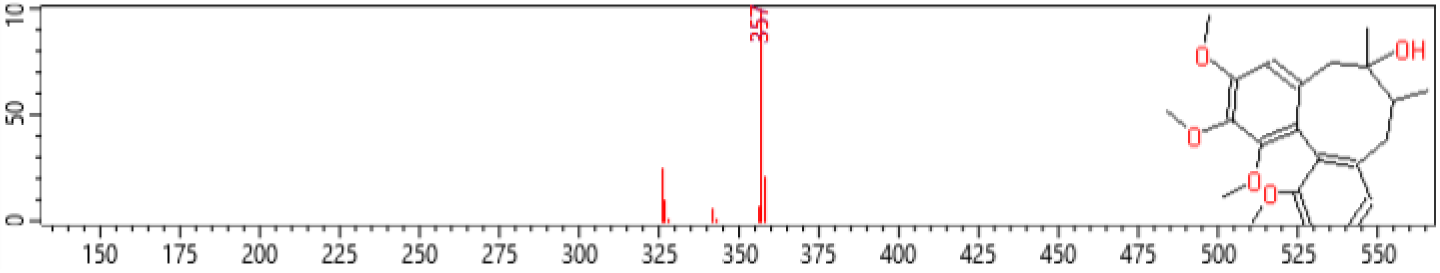

4H-1-Benzopyran-4-one, 2-(3,4-dimethoxyphenyl)-5,6,7-trimethoxy-

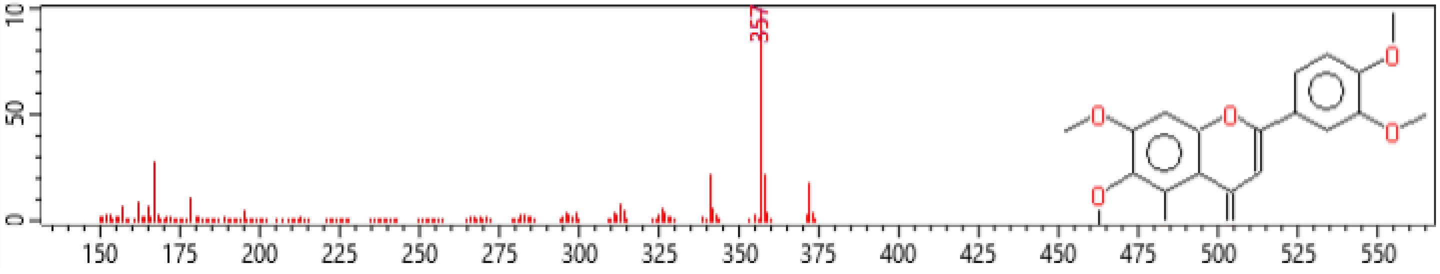

5-Hydroxy-7,8-dimethoxyflavanone, TMS derivative

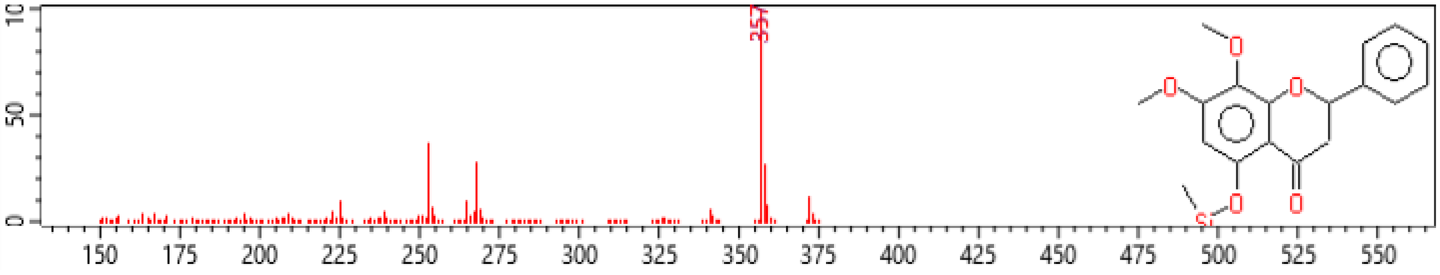

3-Hydroxy-7,3',4',5'-tetramethoxyflavone

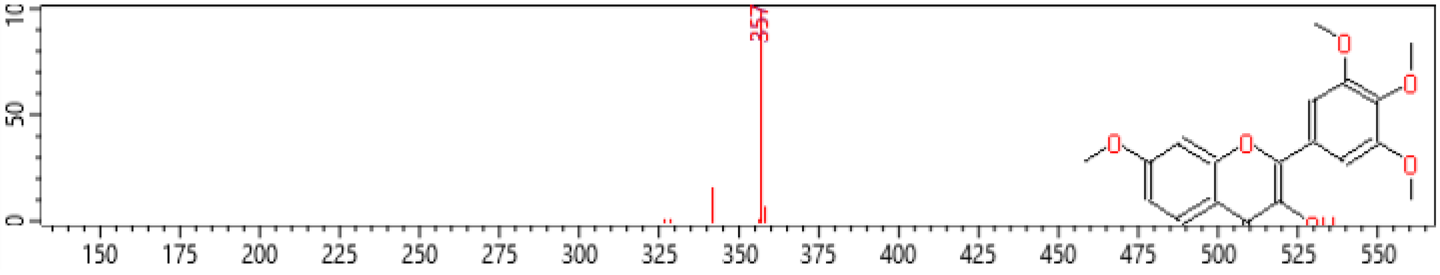

Androst-4-ene-3,17-dione, 7-[(trimethylsilyl)oxy]-, (7α)-

Peak: 16.25 - 16.27 min (scan #2582 - #2588) Subtracted noise: 16.21 - 16.23 min (scan #2569 - #2576), 16.28 - 16.30 min (scan #25

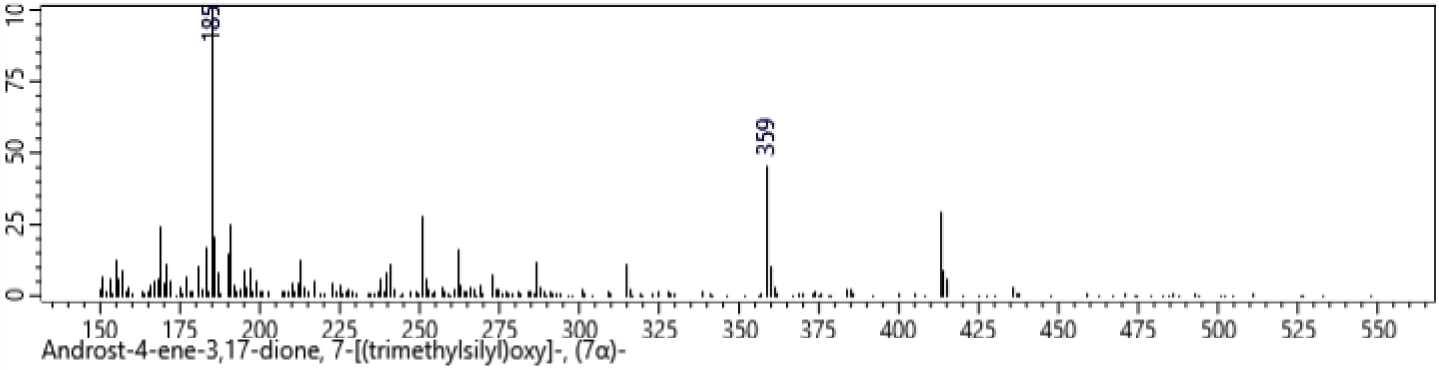

Androst-4-ene-3,17-dione, 7-[(trimethylsilyl)oxy]-, (7α)-

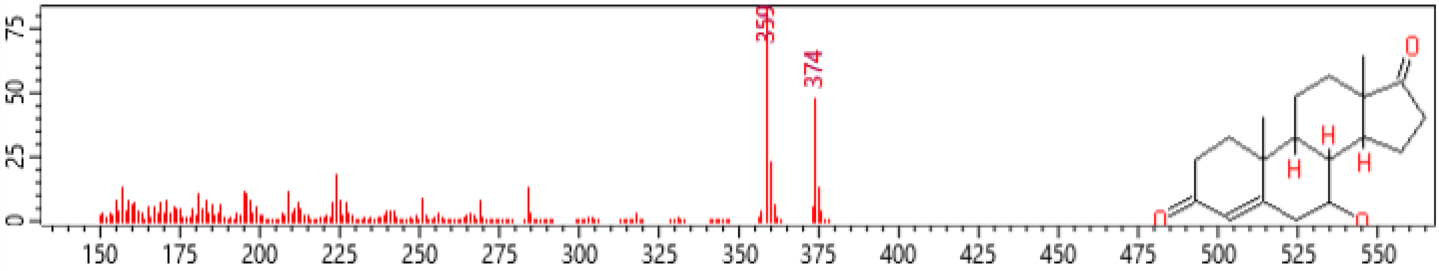

Androst-4-ene-3,17-dione, 7-[(trimethylsilyl)oxy]-, (7α)-

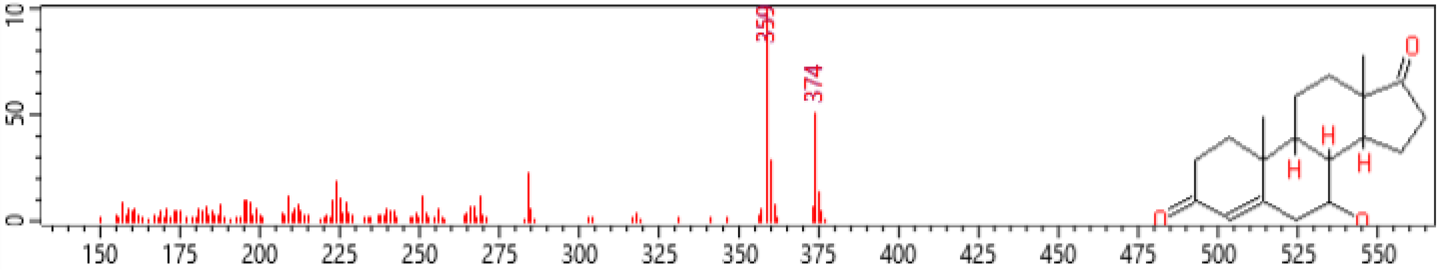

3-Hydroxy-4,4,6a,9,12b-pentamethyl-3,4,4a,6,6a,12,12a,12b-octahydro-2H,11H-benzo[f]pyrano[2,3-b]chromene-1,11(5H)-dione, TM

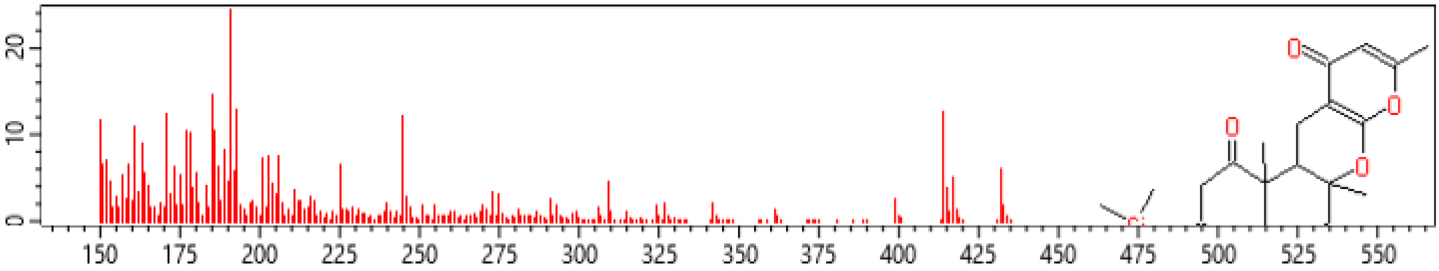

Pregnan-11-one, 17,21-[[(1,1-dimethylethyl)borylene]bis(oxy)]-3,20-dihydroxy-, (3α,5β,20S)-

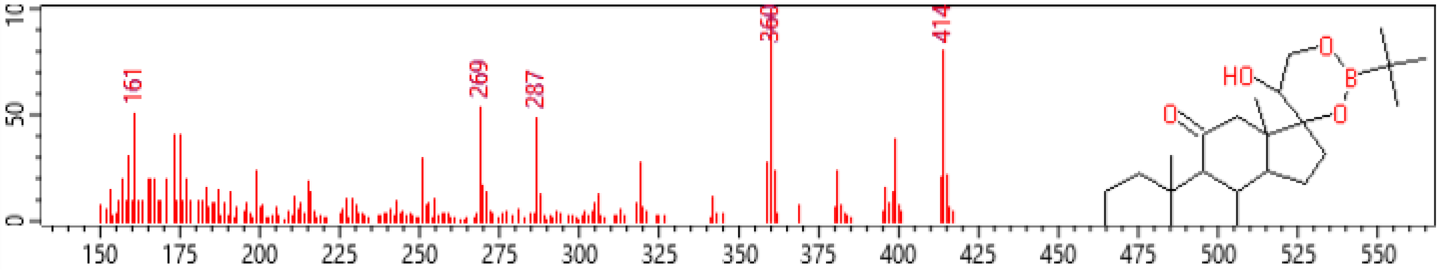

7-Keto-3α,12-α-dihydroxycholanic acid, 3TMS derivative

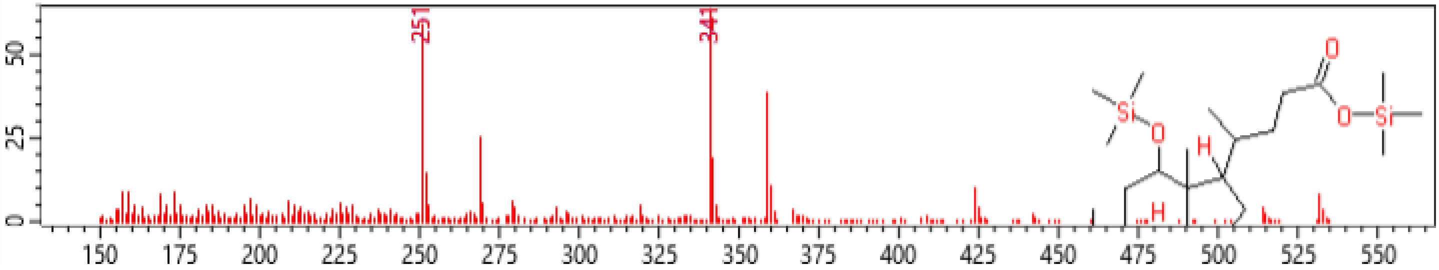

4-Butyl-1-phenyl-pyrazolidine-3,5-diol, O,O'-bis(tert-butyldimethylsilyl) ether

Peak: 16.30 - 16.31 min (scan #2594 - #2598) Subtracted noise: 16.26 - 16.28 min (scan #2585 - #2589), 16.32 - 16.34 min (scan #26

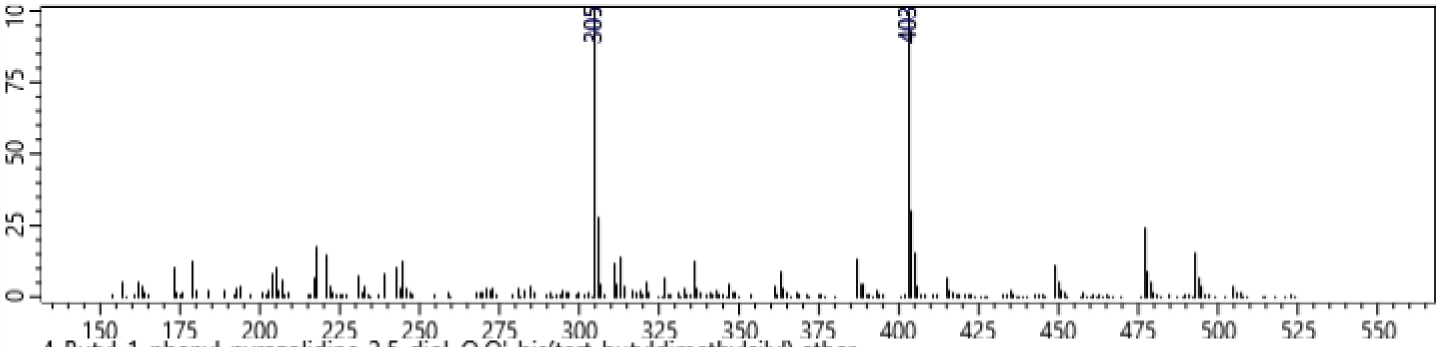

4-Butyl-1-phenyl-pyrazolidine-3,5-diol, O,O'-bis(tert-butyldimethylsilyl) ether

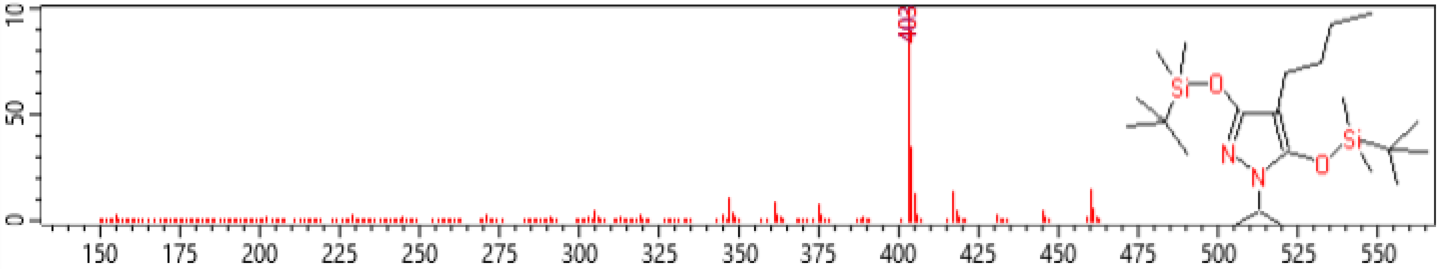

Dimethyl 2-acetyloxy-5'-(furan-3-yl)-1-hydroxy-6-methyl-2'-oxospiro[3,4,4a,8-tetrahydro-2H-naphthalene-5,3'-oxolane]-1,8a-dicarb

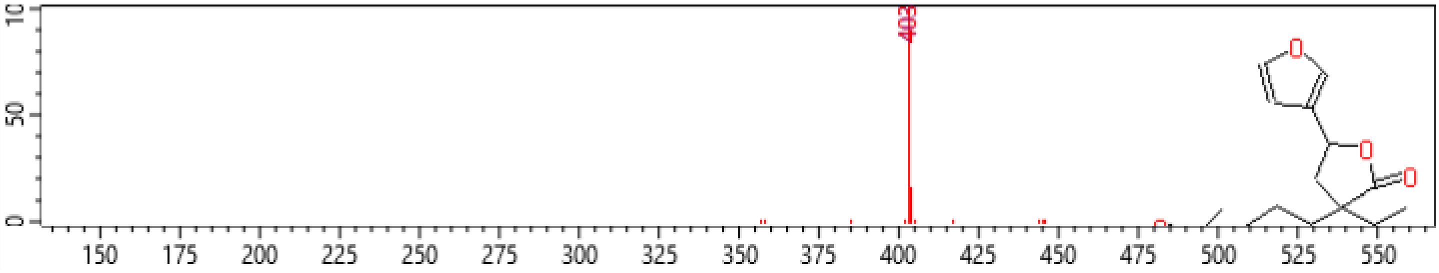

(3-Ethenyl-2-(β-D-glucopyranosyloxy)-5-(methoxycarbonyl)-3,4-dihydro-2H-pyran-4-yl)acetic acid

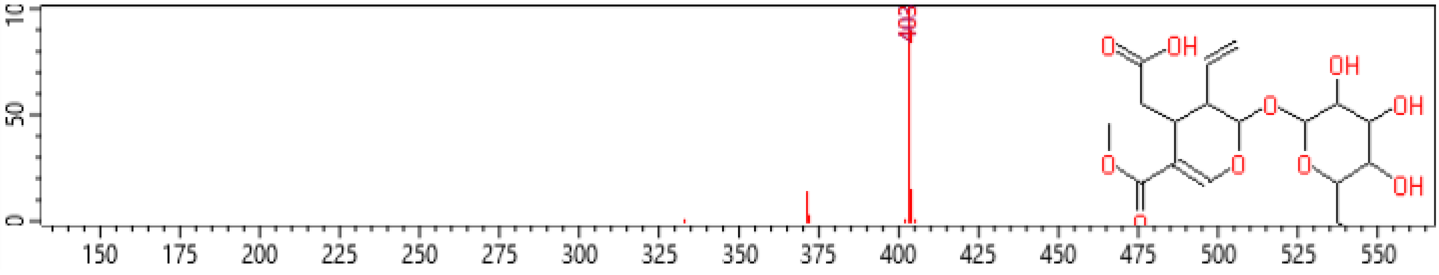

Bis(2-hydroxy-4-methoxyphenyl)methanone, 2TMS derivative

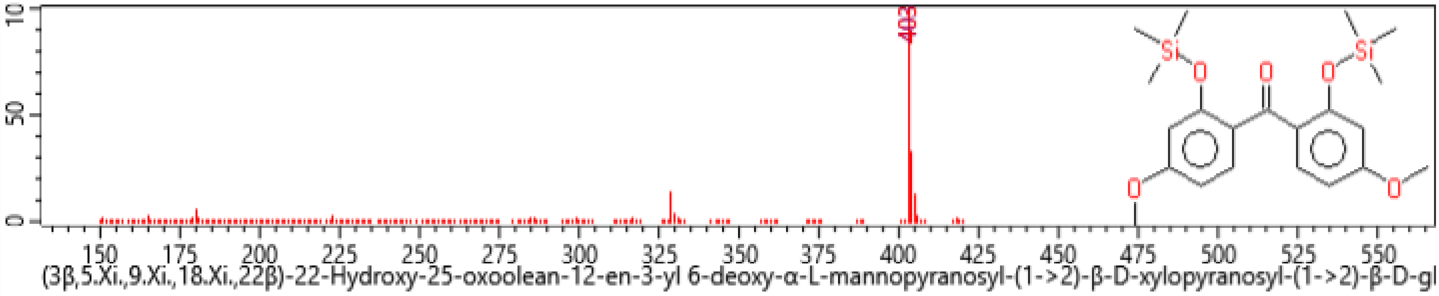

(3β,5.Xi.,9.Xi.,18.Xi.,22β)-22-Hydroxy-25-oxoolean-12-en-3-yl 6-deoxy-α-L-mannopyranosyl-(1->2)-β-D-xylopyranosyl-(1->2)-β-D-gl

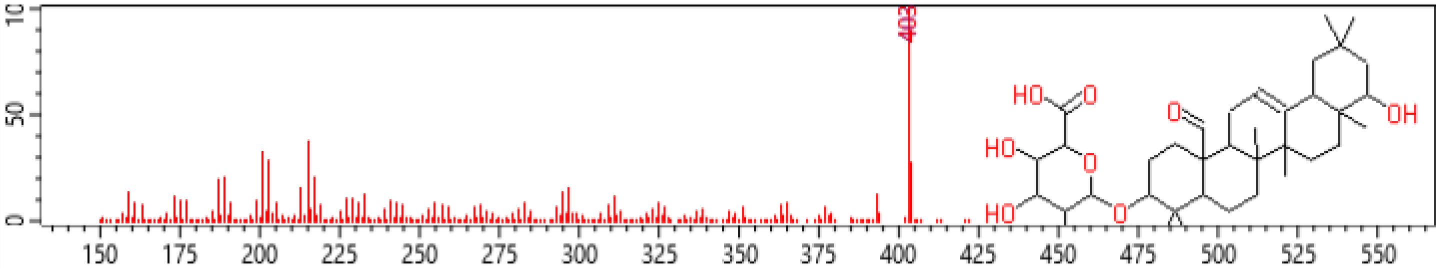

2-Amino-5-phenyl-3-furonitrile, TMS derivative

Peak: 16.47 - 16.49 min (scan #2642 - #2647) Subtracted noise: 16.43 - 16.45 min (scan #2631 - #2636), 16.49 - 16.51 min (scan #26

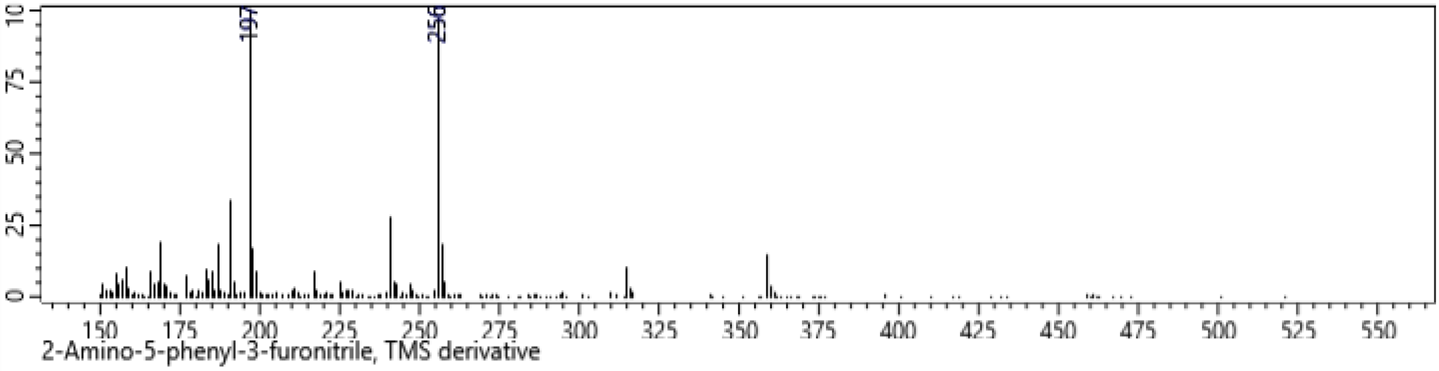

2-Amino-5-phenyl-3-furonitrile, TMS derivative

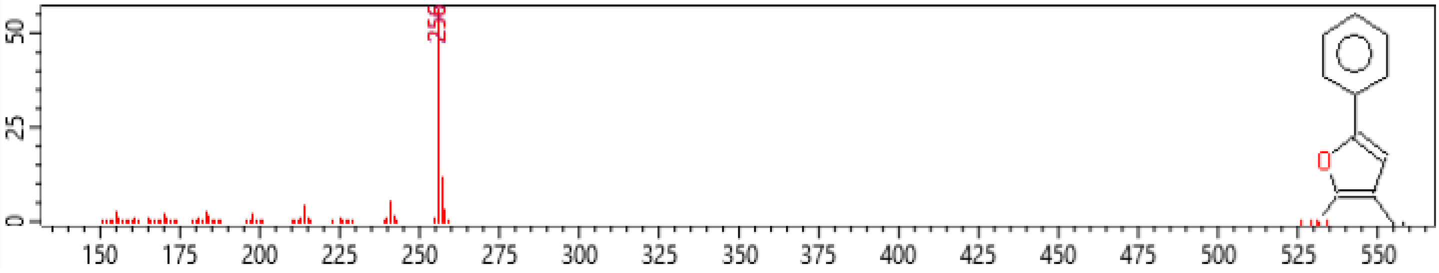

Isopimaric acid, TMS

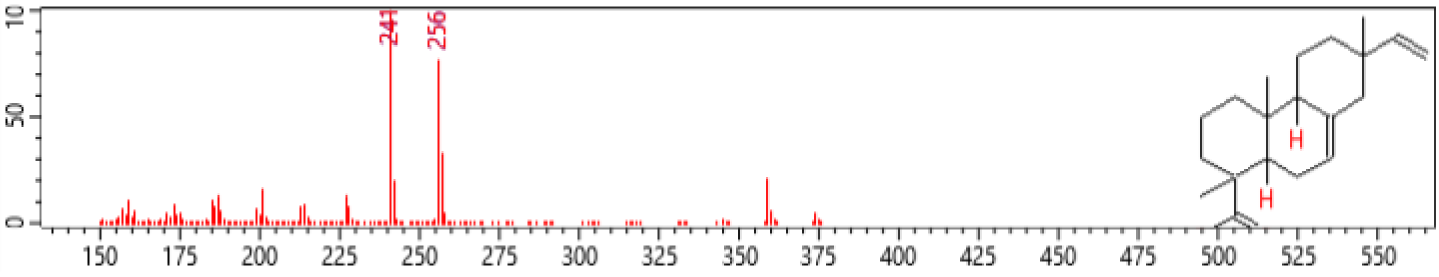

Benzeneacetic acid, α-methyl-3-phenoxy-, methyl ester, (±)-

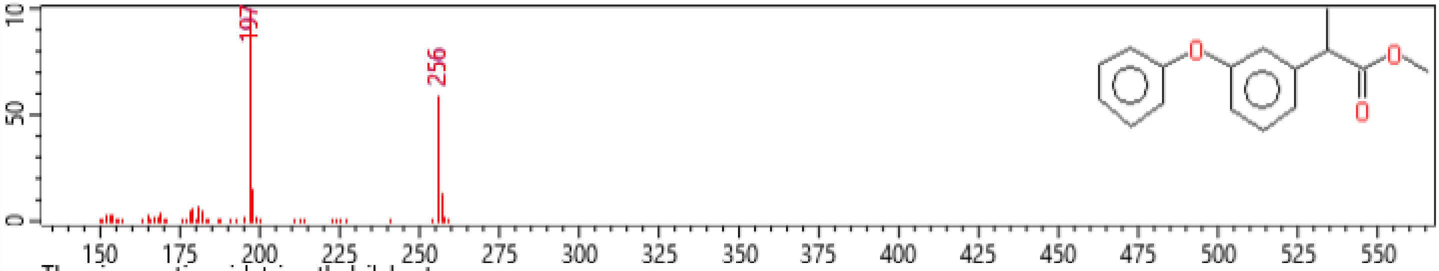

Thymine acetic acid, trimethylsilyl ester

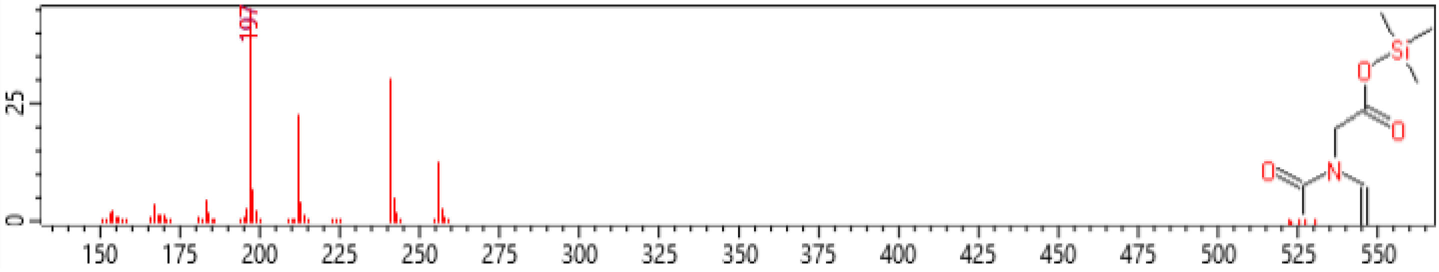

1-Phenyl-3,6-diazahomoadamantan-9-one hydrazone

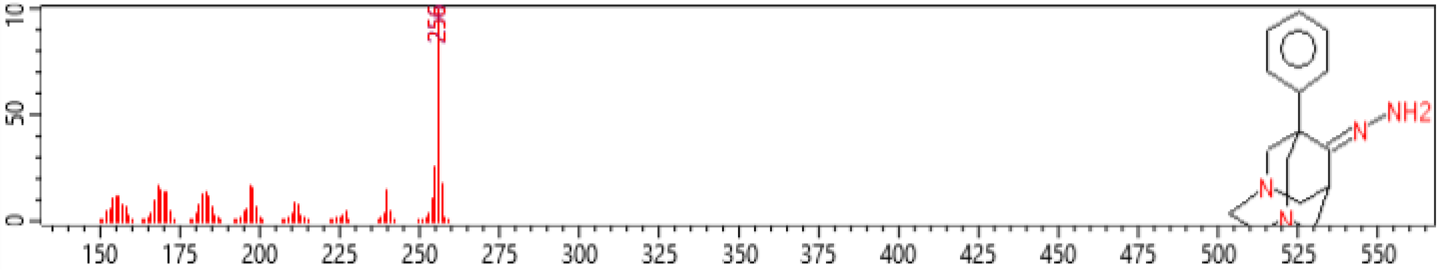

Ethyl Oleate

Peak: 16.62 - 16.65 min (scan #2685 - #2693) Subtracted noise: 16.57 - 16.59 min (scan #2670 - #2677), 16.66 - 16.69 min (scan #26

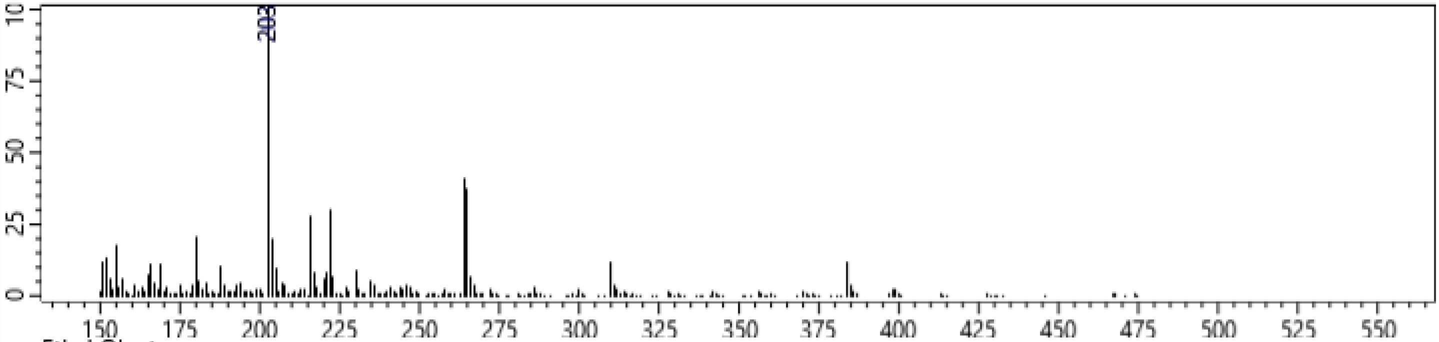

Ethyl Oleate

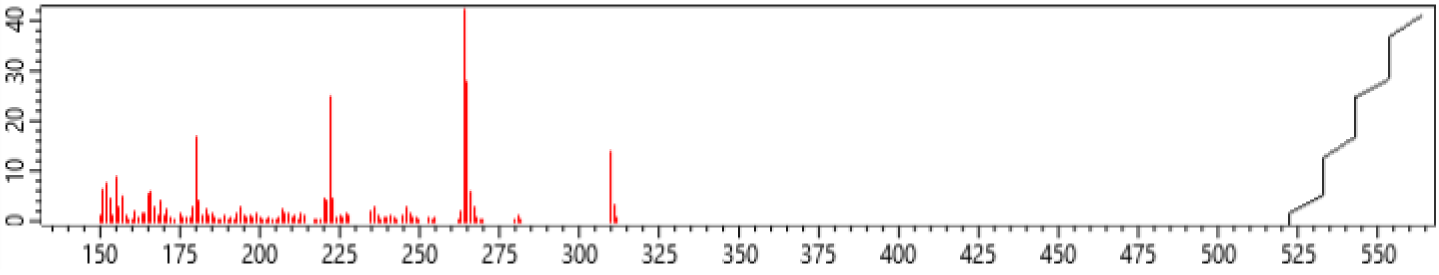

Ethyl Oleate

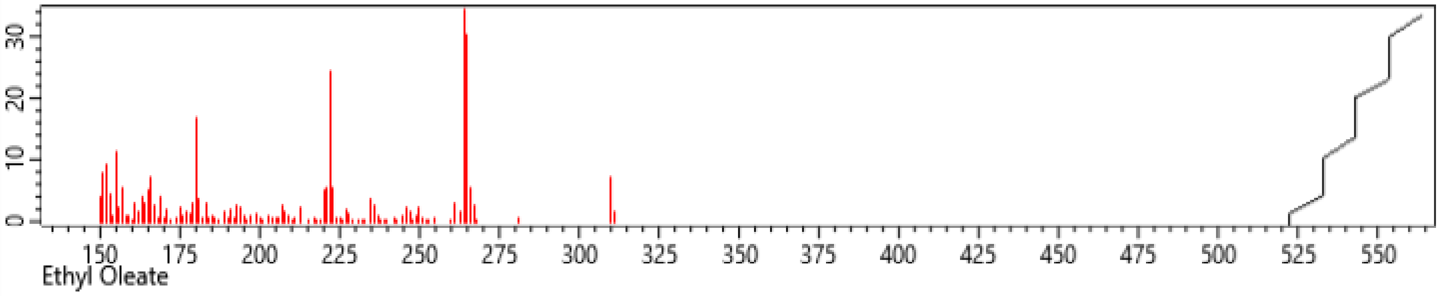

Ethyl Oleate

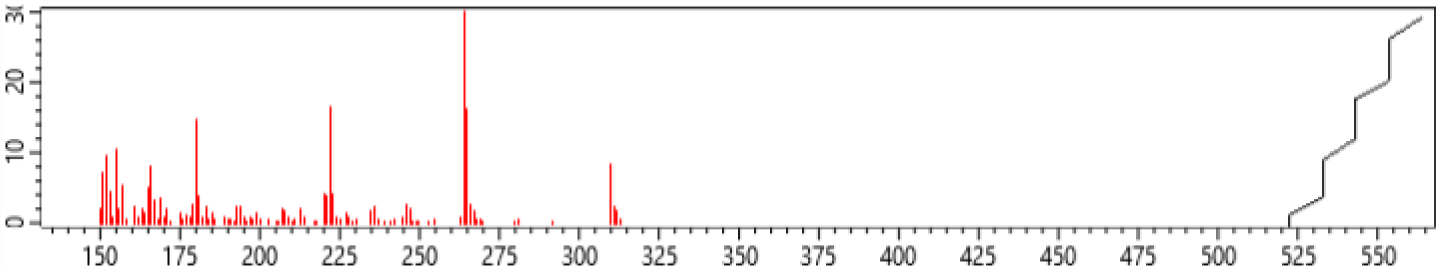

(E)-9-Octadecenoic acid ethyl ester

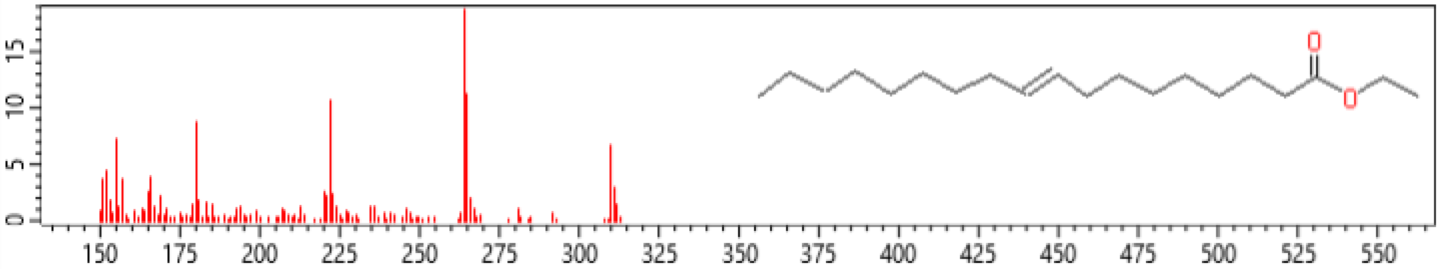

(E)-9-Octadecenoic acid ethyl ester

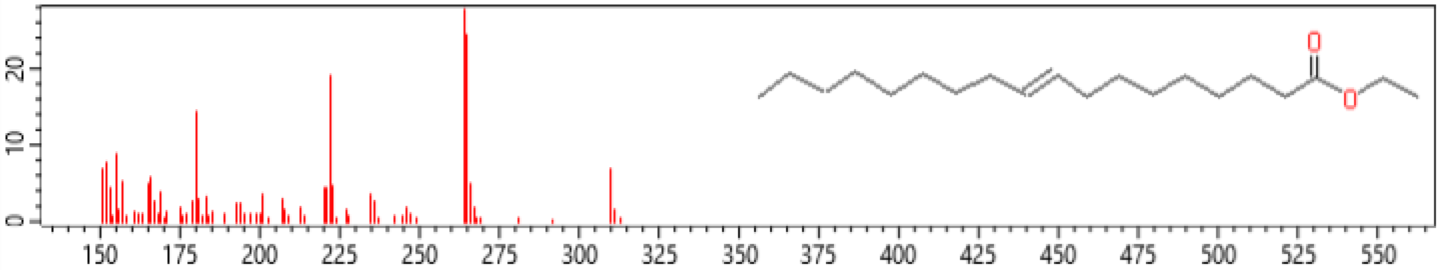

9-Octadecenoic acid, (E)-, TMS derivative

Peak: 17.12 - 17.21 min (scan #2825 - #2848) Subtracted noise: 16.93 - 17.02 min (scan #2771 - #2795), 17.19 - 17.27 min (scan #28

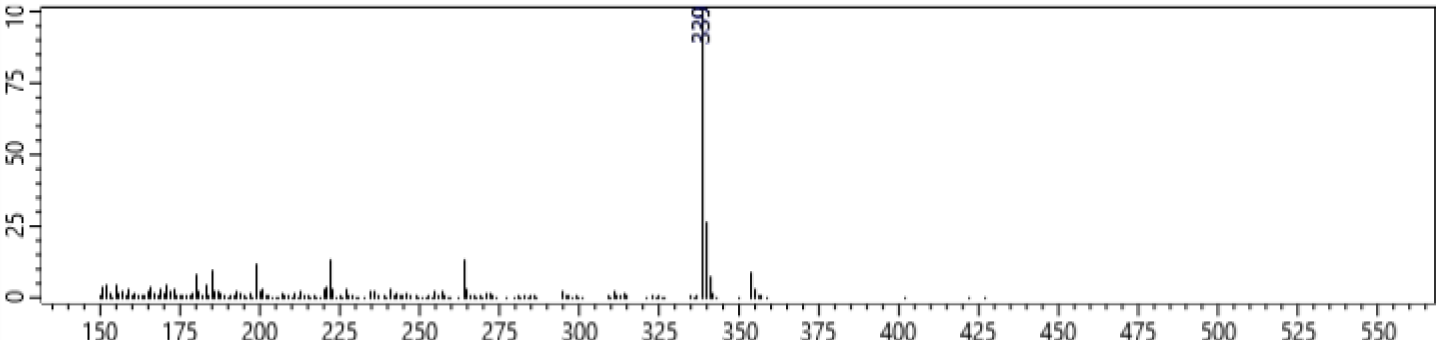

9-Octadecenoic acid, (E)-, TMS derivative

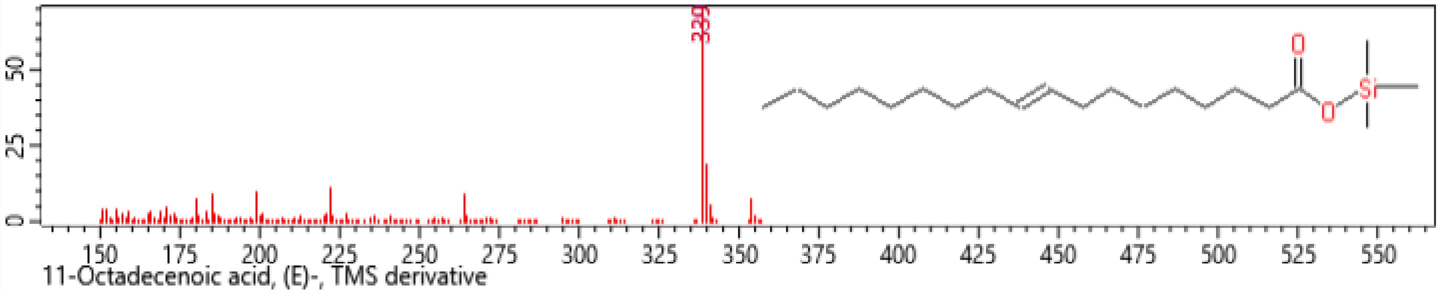

11-Octadecenoic acid, (E)-, TMS derivative

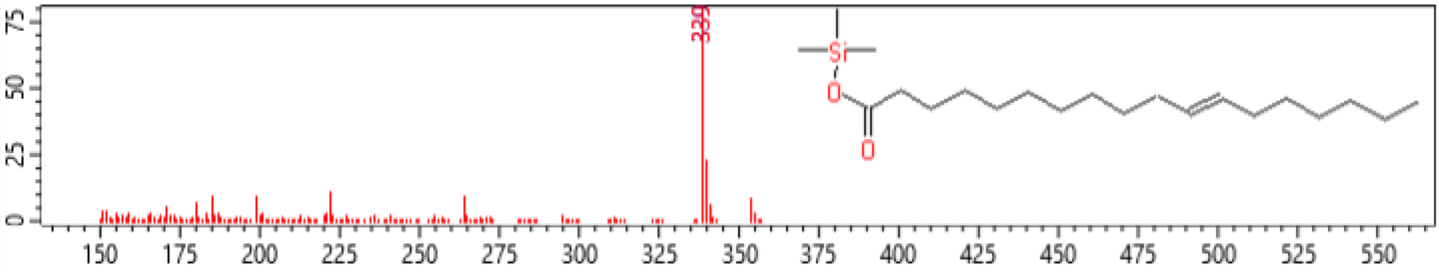

Oleic Acid, (Z)-, TMS derivative

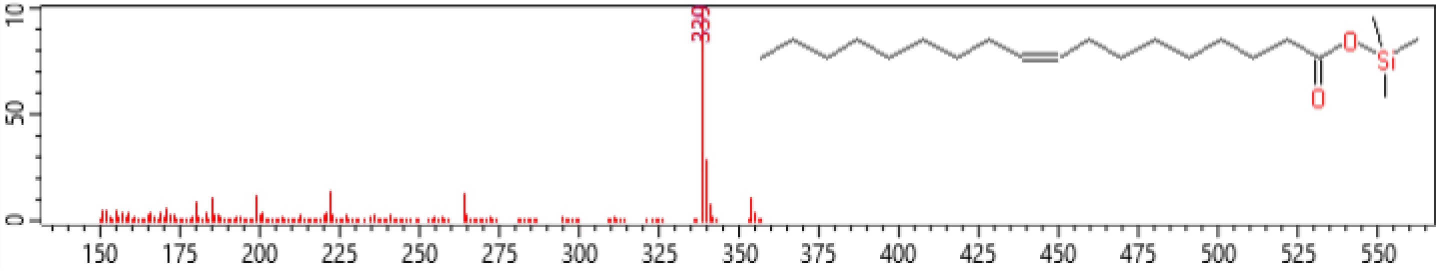

13-Octadecenoic acid, (E)-, TMS derivative

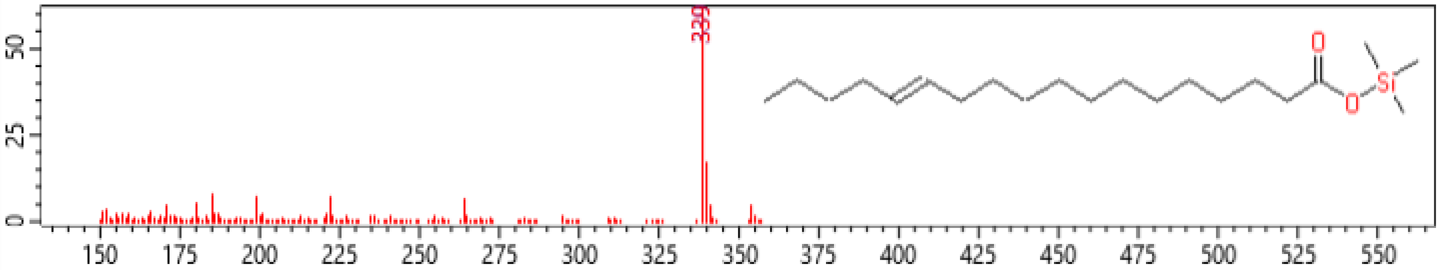

11-Octadecenoic acid, (Z)-, TMS derivative

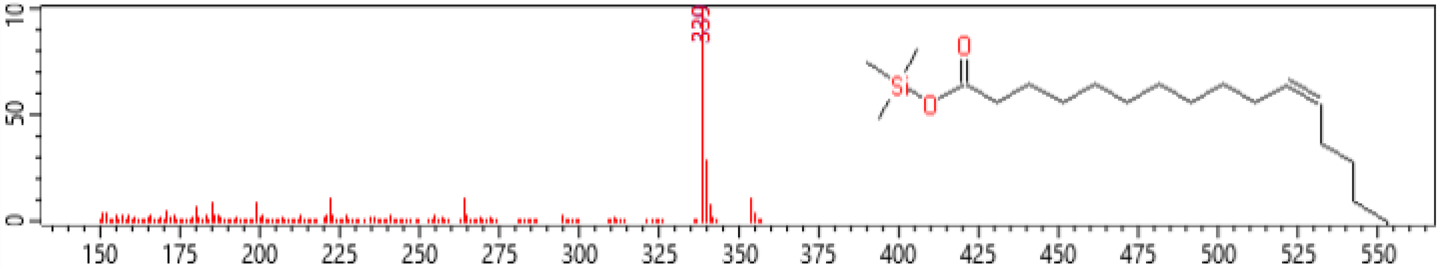

3,8-Dihydroxy-1-methyl-9,10-dioxoanthracene-2-carboxylic acid, 3TMS

Peak: 17.20 - 17.21 min (scan #2845 - #2849) Subtracted noise: 17.17 - 17.19 min (scan #2839 - #2843), 17.22 - 17.24 min (scan #28

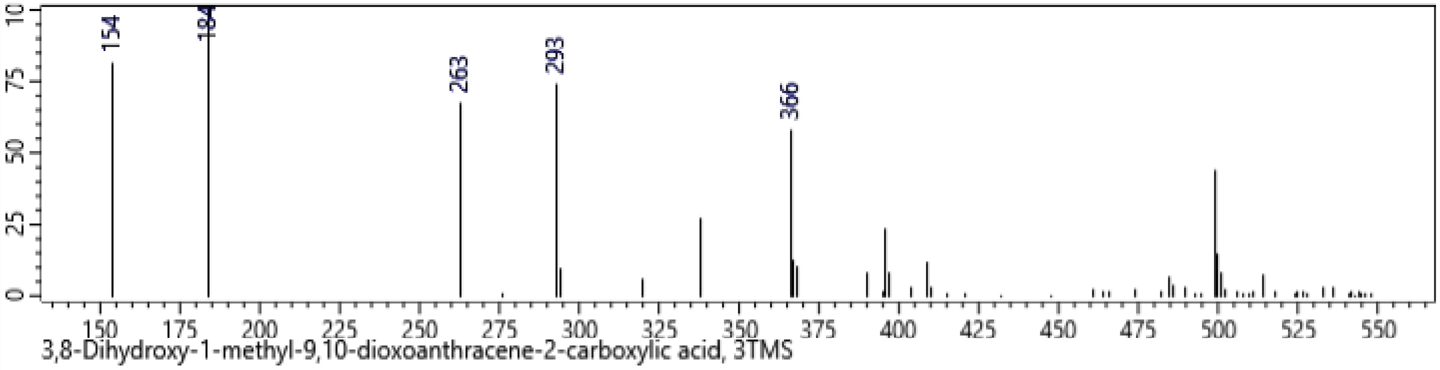

3,8-Dihydroxy-1-methyl-9,10-dioxoanthracene-2-carboxylic acid, 3TMS

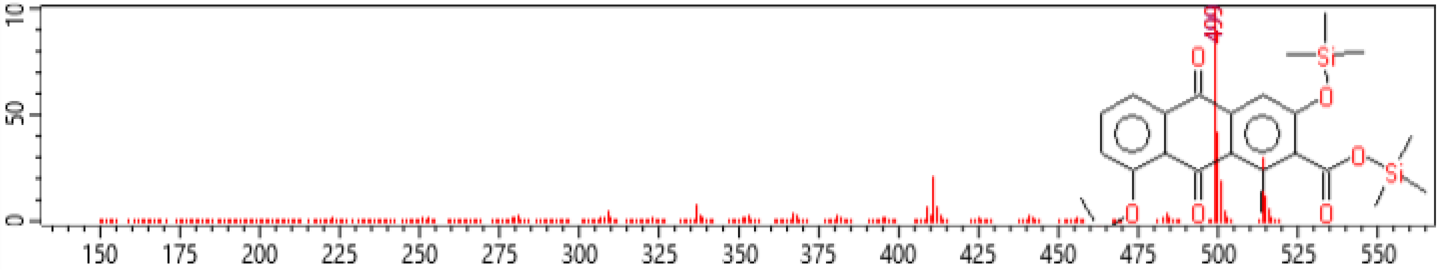

Fumaritine, 2TMS

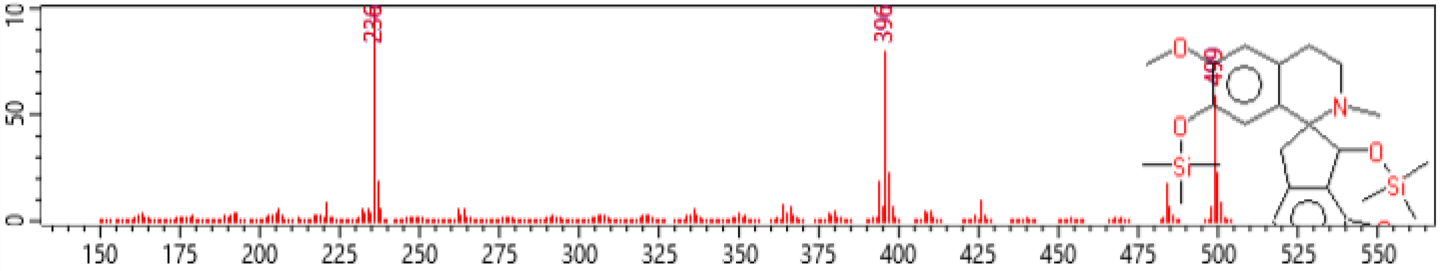

Isonipecotic acid, N-(4-ethylbenzoyl)-, heptadecyl ester

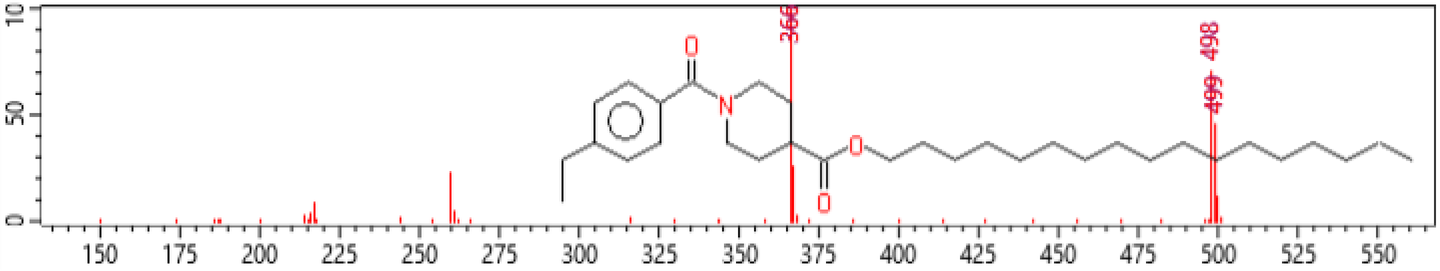

3,27-Dioxa-2,28-disilanonacosane, 2,2,4,28,28-pentamethyl-

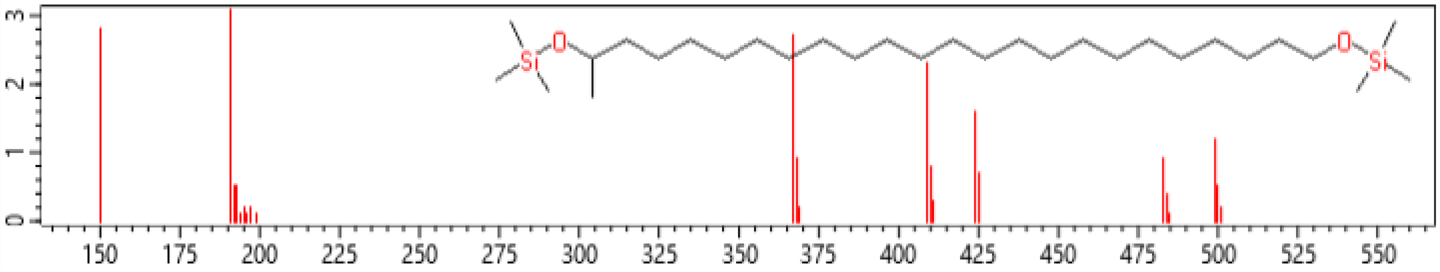

benzenamine, 4,4',4''-methylidynetris[N,N-diethyl-3-methyl-

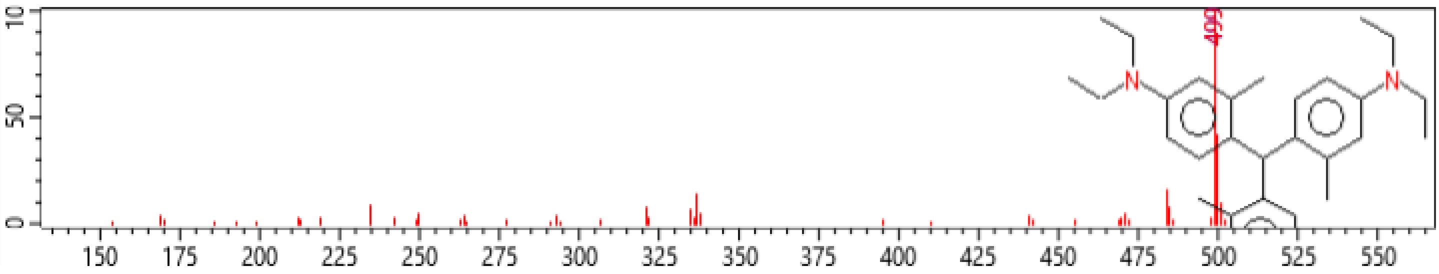

Stearic acid, TMS derivative

Peak: 17.36 - 17.44 min (scan #2892 - #2912) Subtracted noise: 17.25 - 17.32 min (scan #2859 - #2880), 17.47 - 17.55 min (scan #29

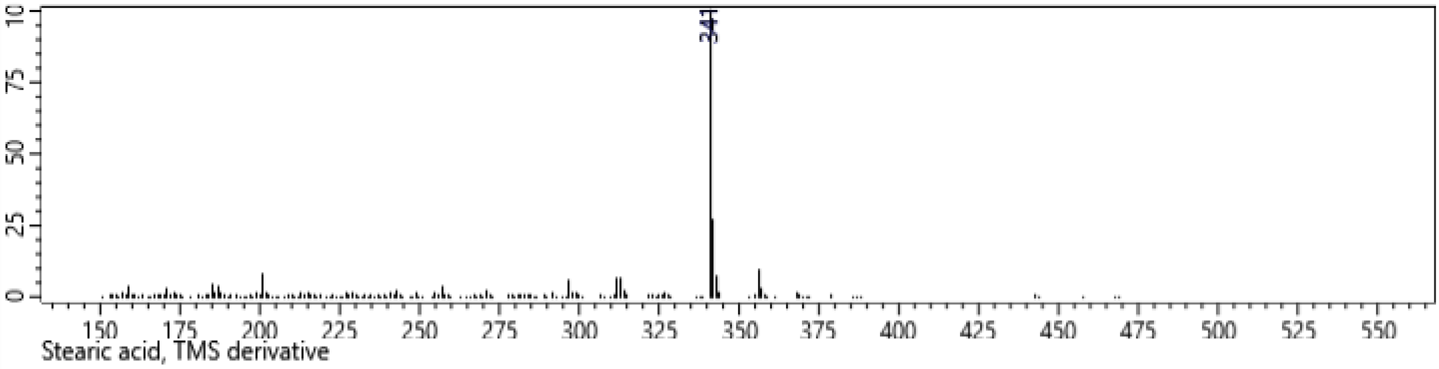

Stearic acid, TMS derivative

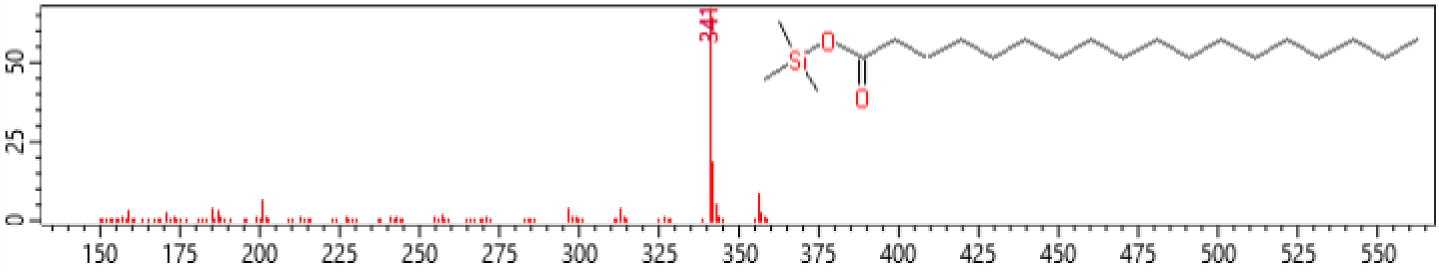

Stearic acid, TMS derivative

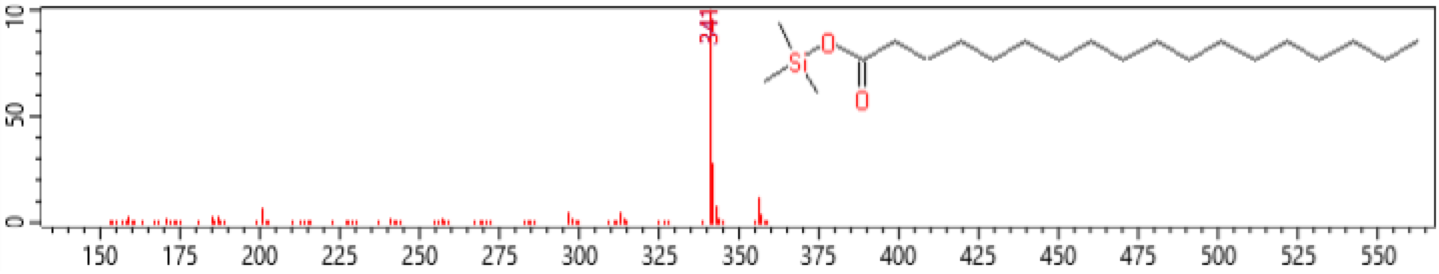

Stearic acid, TMS derivative

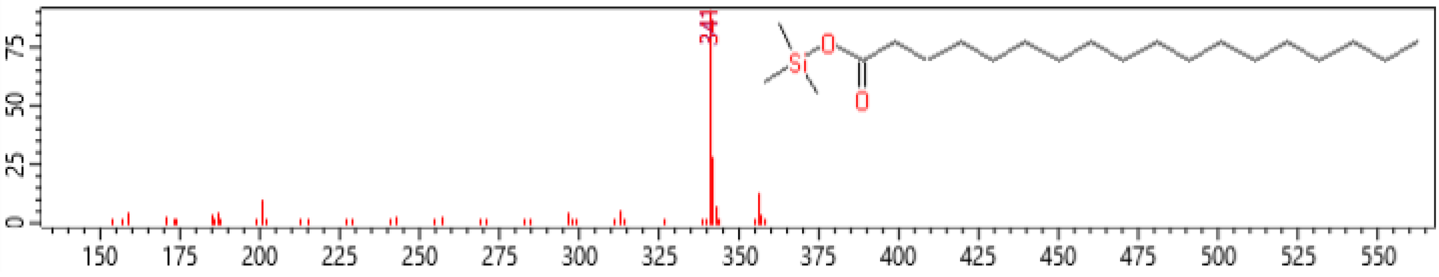

Spironolactone

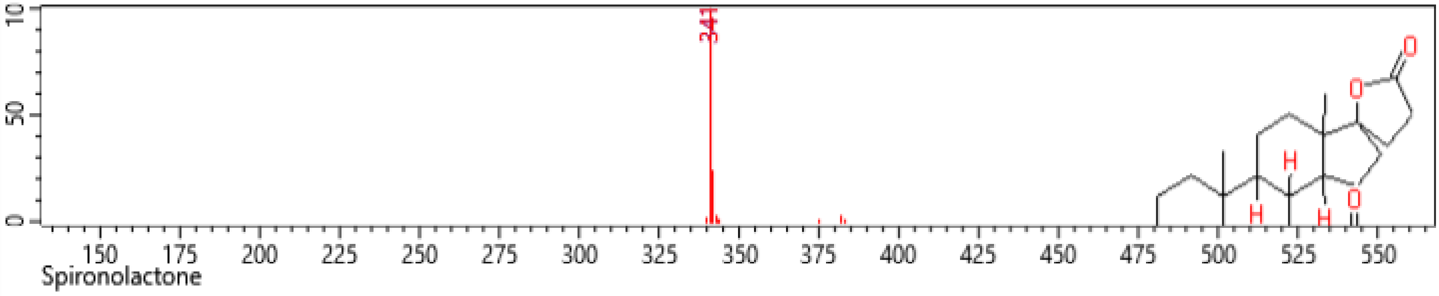

Spironolactone

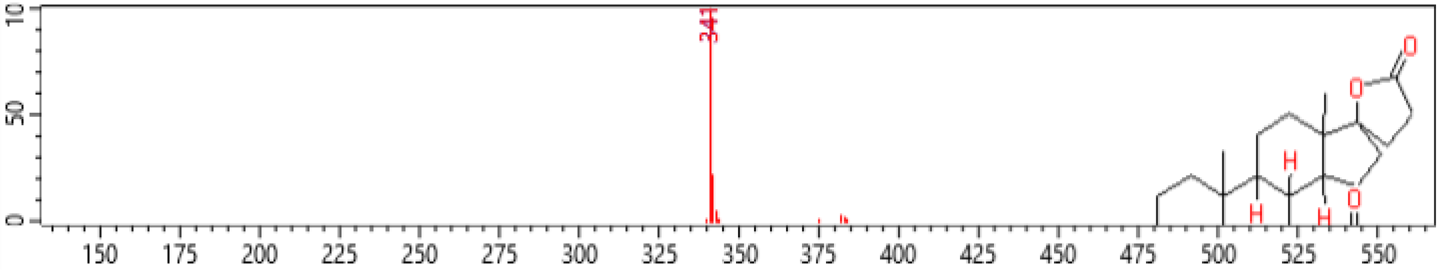

Androstan-3-ol, (3β,5α)-

Peak: 17.71 - 17.76 min (scan #2990 - #3002) Subtracted noise: 17.66 - 17.70 min (scan #2974 - #2987), 17.80 - 17.85 min (scan #30

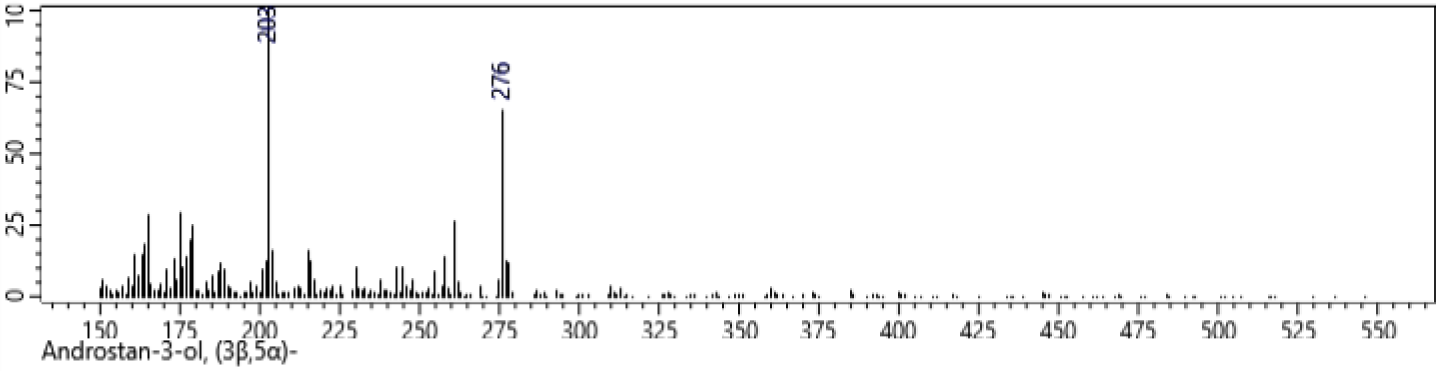

Androstan-3-ol, (3β,5α)-

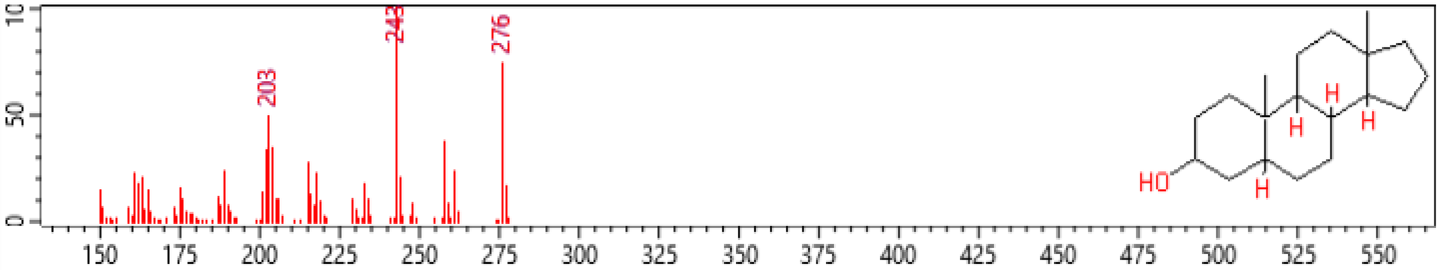

Androstan-3-ol, (3β,5α)-

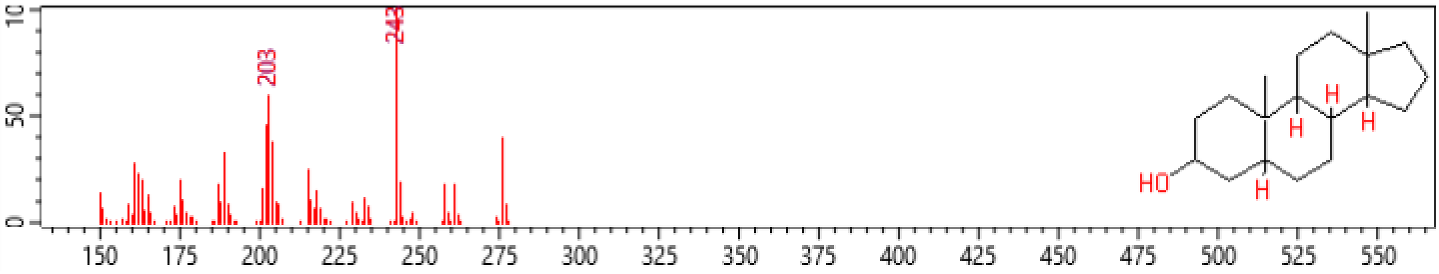

Androstane, 17,18-diiodo-, (5α,17β)-

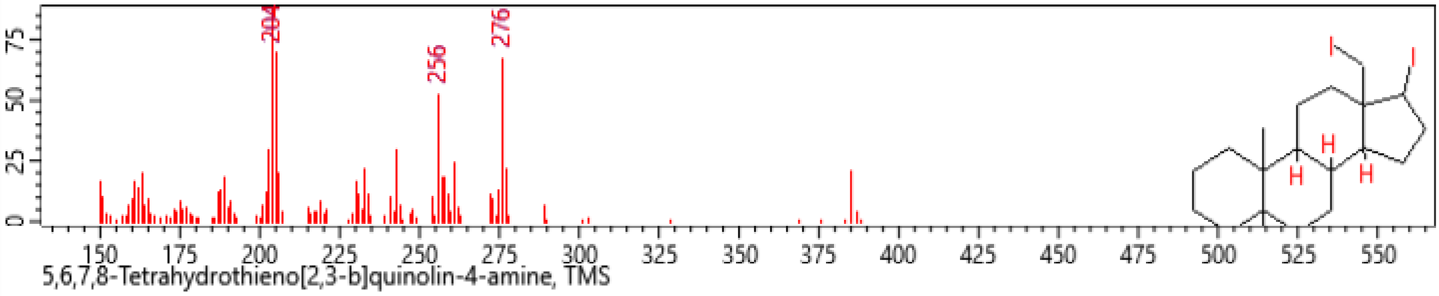

5,6,7,8-Tetrahydrothieno[2,3-b]quinolin-4-amine, TMS

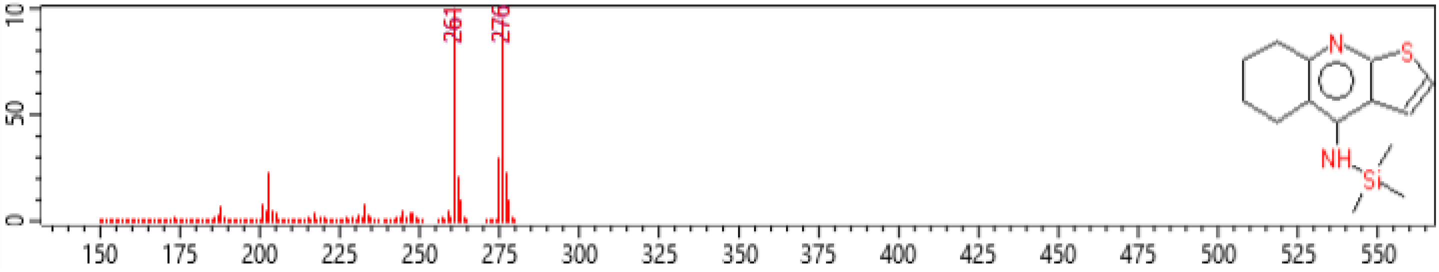

Indophenol blue

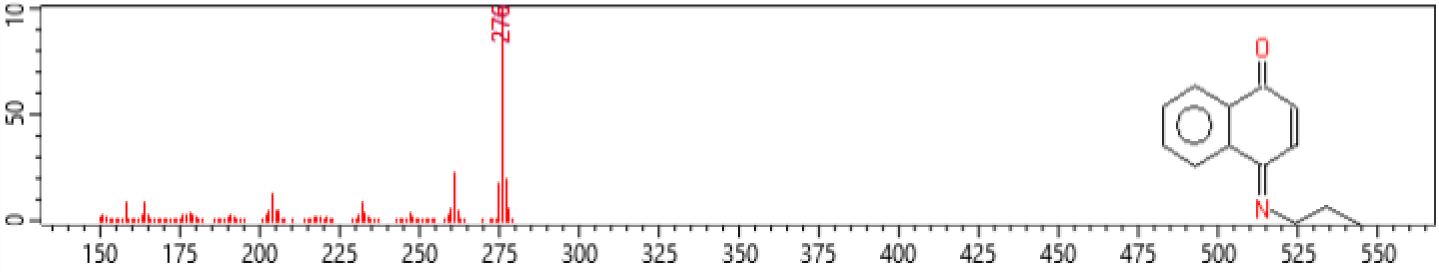

Morphine Modulating Peptide, C-Terminal Fragment

Peak: 18.18 - 18.22 min (scan #3121 - #3131) Subtracted noise: 18.12 - 18.15 min (scan #3102 - #3112), 18.24 - 18.28 min (scan #31

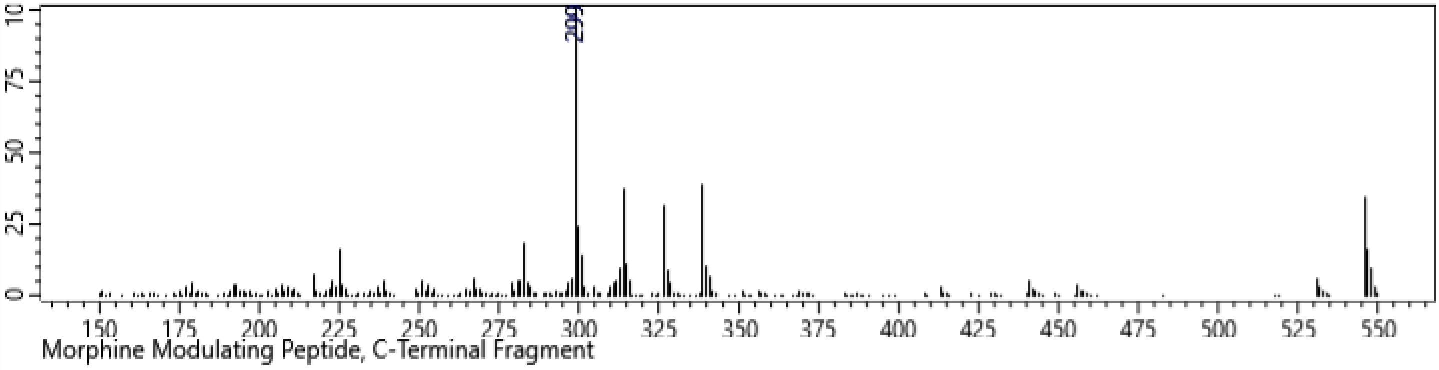

Morphine Modulating Peptide, C-Terminal Fragment

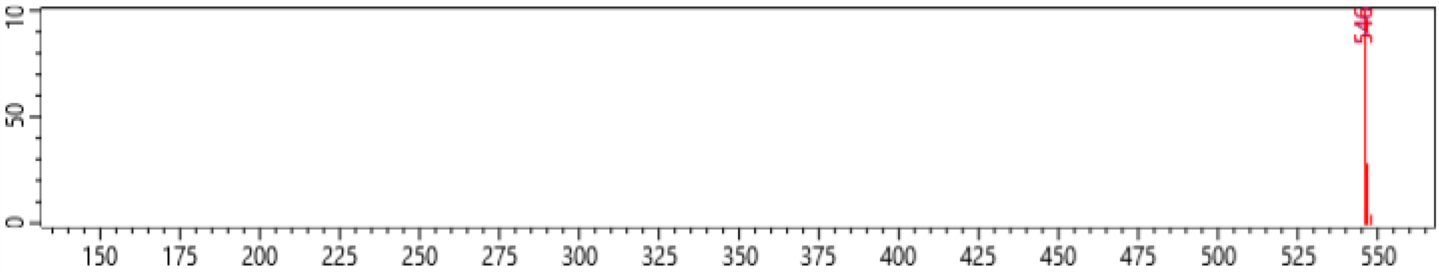

Morphine Modulating Peptide, C-Terminal Fragment

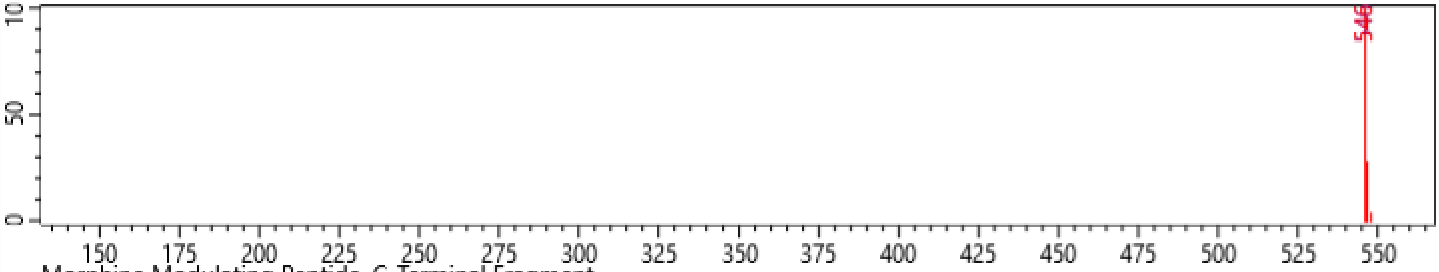

Morphine Modulating Peptide, C-Terminal Fragment

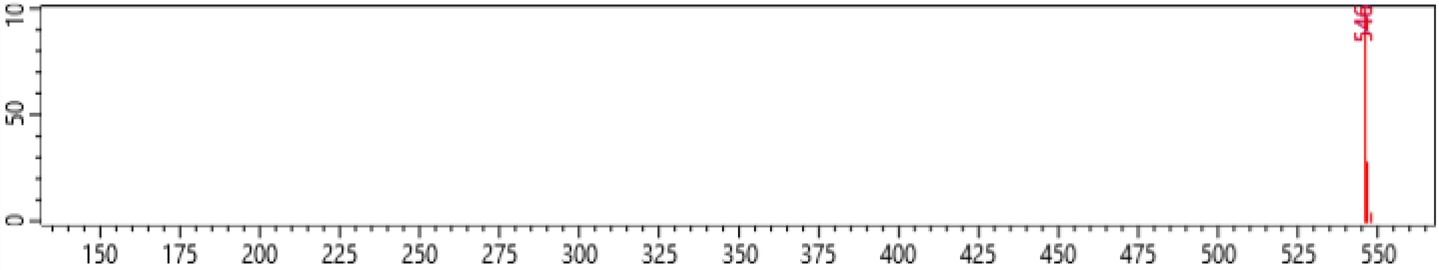

Morphine Modulating Peptide, C-Terminal Fragment

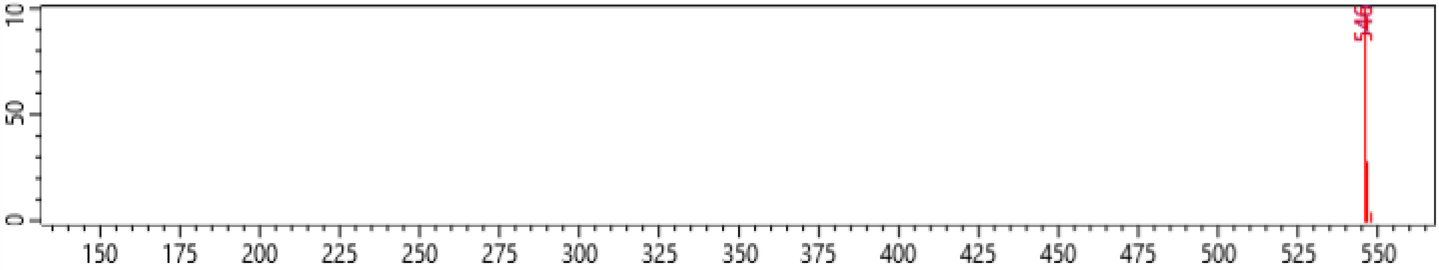

Silanol, trimethyl-, phosphate (3:1)

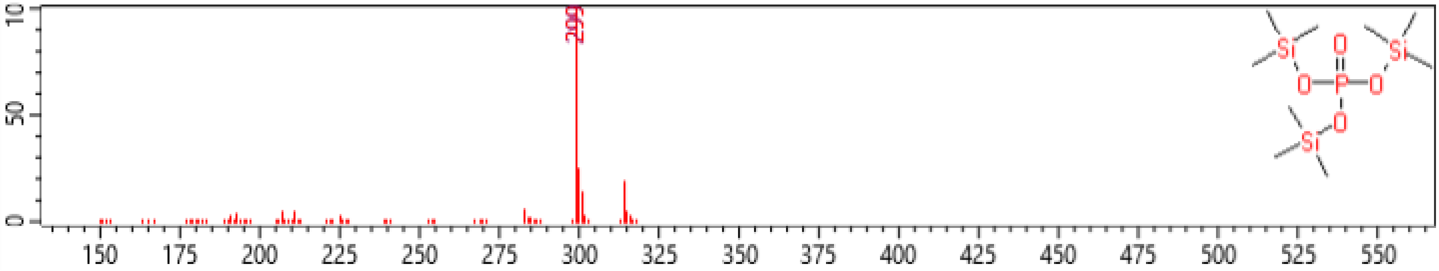

5-Amino-2-(3-hexyl-4-methyl-2,5-dioxopyrrol-1-yl)-5-oxopentanoic acid, 3TMS

Peak: 18.59 - 18.62 min (scan #3234 - #3242) Subtracted noise: 18.54 - 18.57 min (scan #3219 - #3228), 18.64 - 18.67 min (scan #32

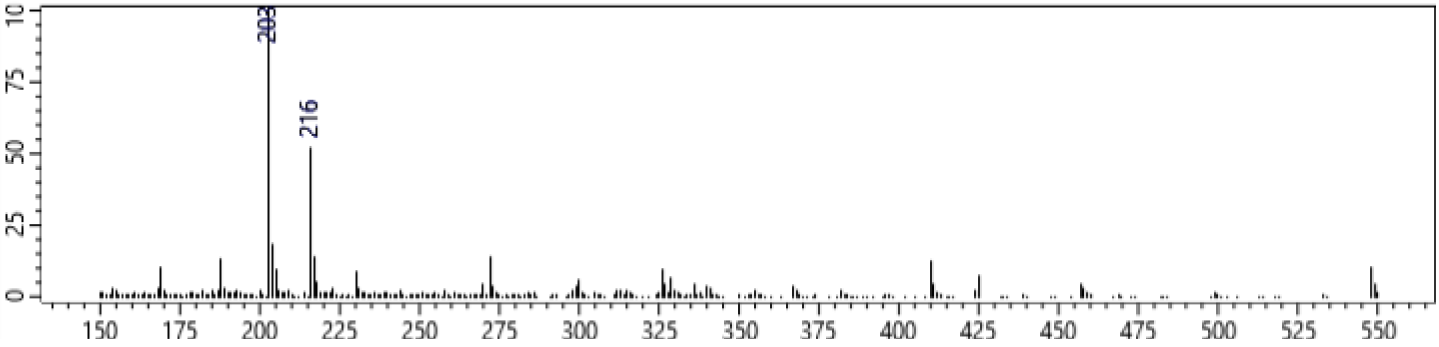

5-Amino-2-(3-hexyl-4-methyl-2,5-dioxopyrrol-1-yl)-5-oxopentanoic acid, 3TMS

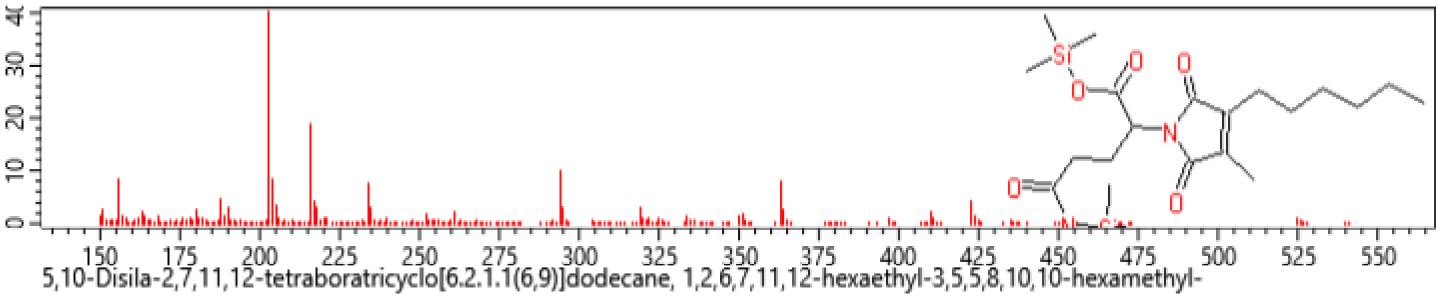

5,10-Disila-2,7,11,12-tetraboratricyclo[6.2.1.1(6,9)]dodecane, 1,2,6,7,11,12-hexaethyl-3,5,5,8,10,10-hexamethyl-

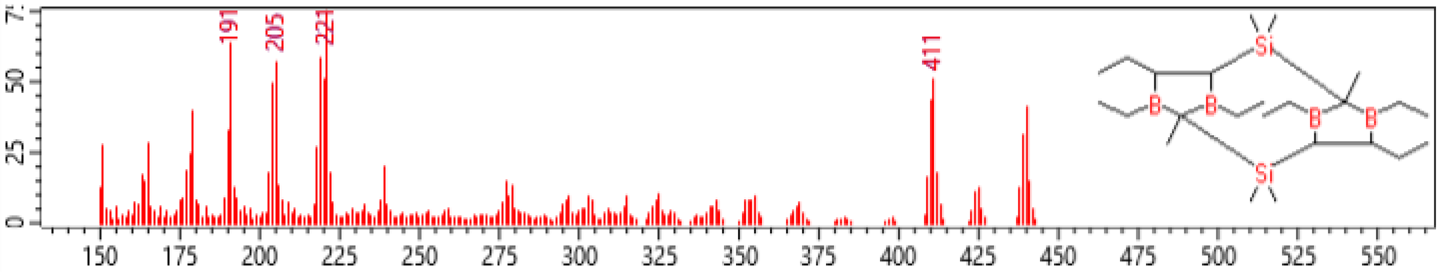

Olean-13(18)-ene

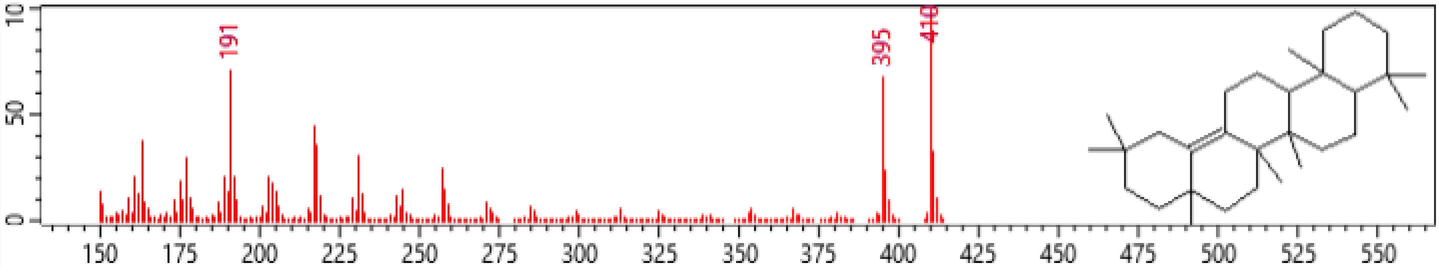

Uvaol

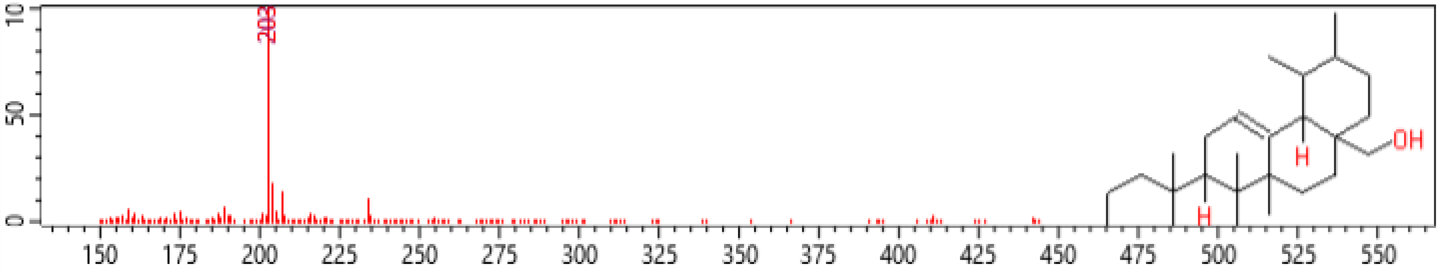

Octadecanamide, N-(2-methylpropyl)-N-nitroso-

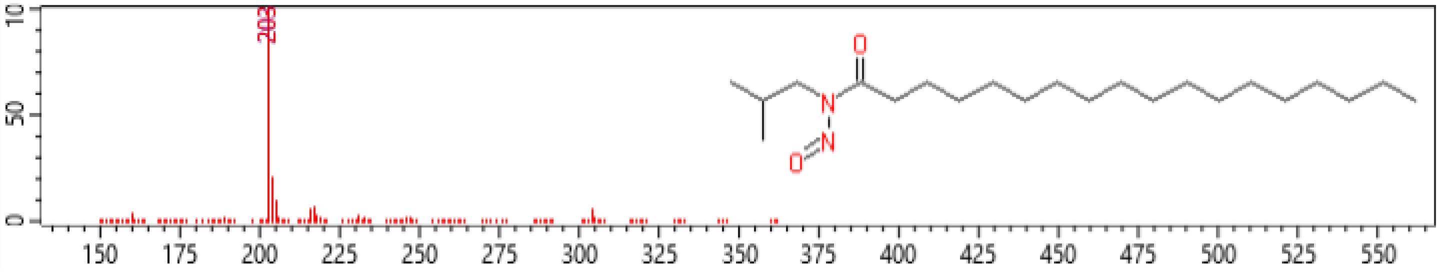

9,10-Anthracenedione, 1,4-bis[(1-methylethyl)amino]-

Peak: 19.32 - 19.34 min (scan #3438 - #3444) Subtracted noise: 19.28 - 19.30 min (scan #3428 - #3433), 19.36 - 19.38 min (scan #34

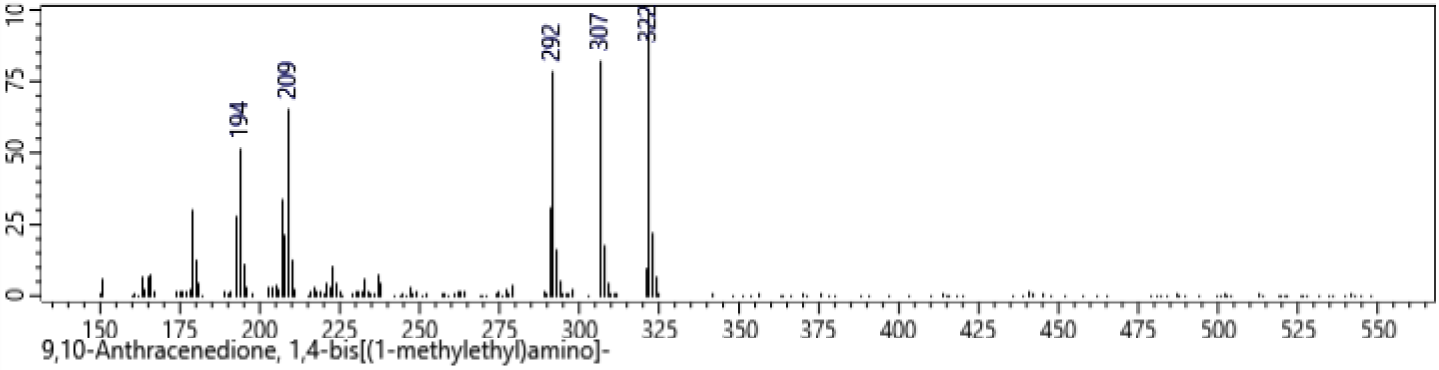

9,10-Anthracenedione, 1,4-bis[(1-methylethyl)amino]-

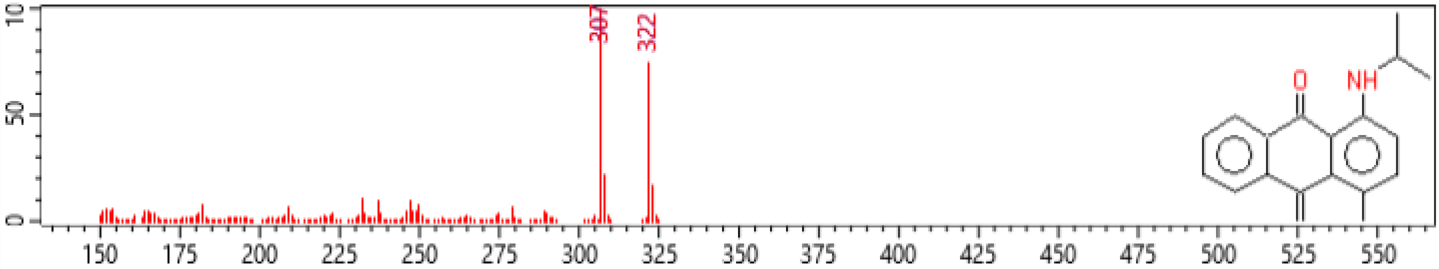

4-Iodo-2-methoxyphenol, TMS

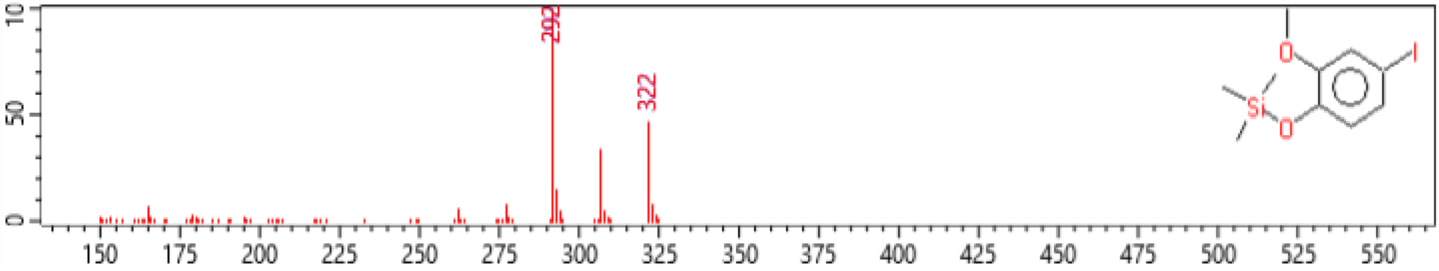

2,4-Dimethyl-6-(1-phenylethyl)phenol, trifluoroacetate

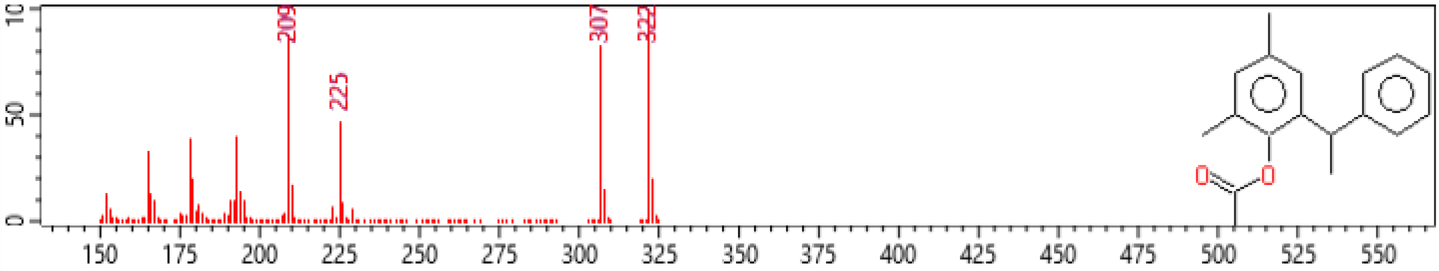

2-Amino-6-methoxy-4-phenyl-3,5-pyridinedicarbonitrile, TMS derivative

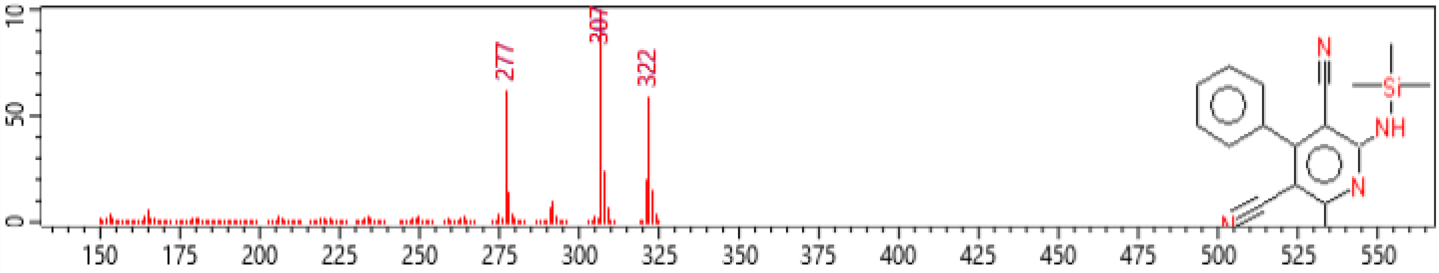

1,4-Naphthoquinone, 2-acetyl-5,8-dihydroxy-3,6,7-trimethoxy-

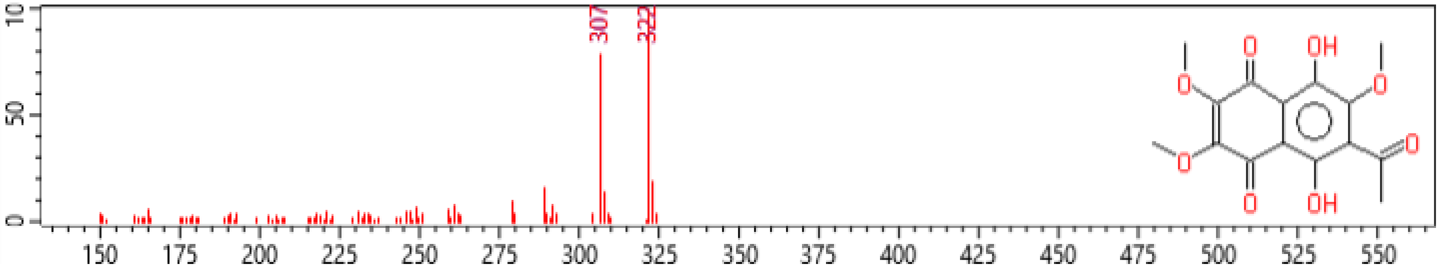

Oleamide, TMS derivative

Peak: 19.69 - 19.79 min (scan #3542 - #3570) Subtracted noise: 19.48 - 19.58 min (scan #3483 - #3510), 19.79 - 19.88 min (scan #35

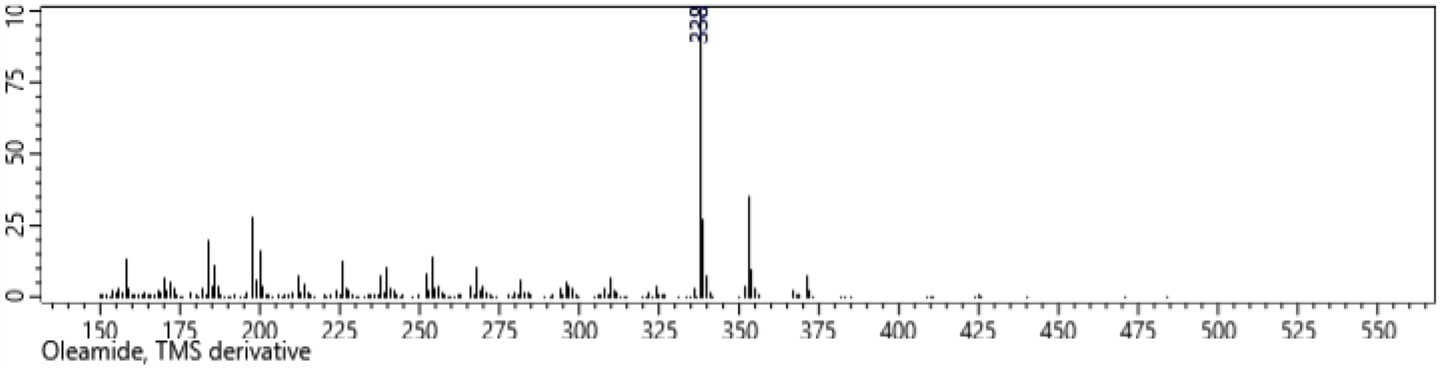

Oleamide, TMS derivative

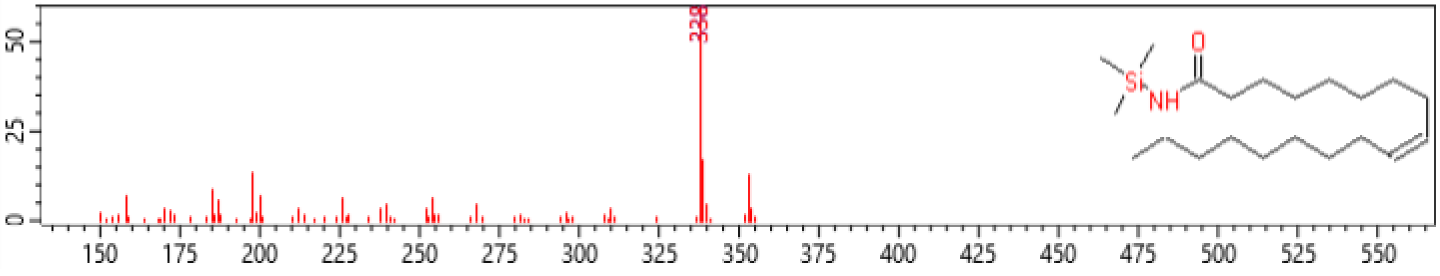

7-Chloro-1-cyclopropyl-6-fluoro-4-oxo-1,4-dihydro-3-quinolinecarboxylic acid, TMS derivative

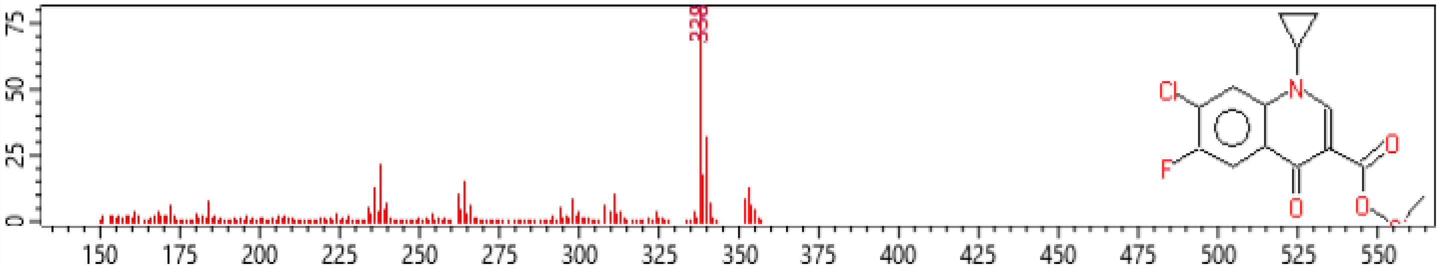

Stearamide, N-(3-methylbutyl)-

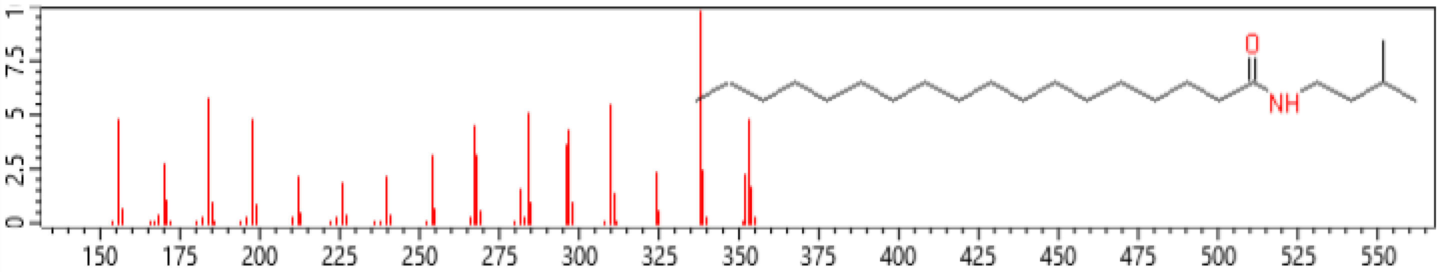

Ethirimol, 2TMS derivative

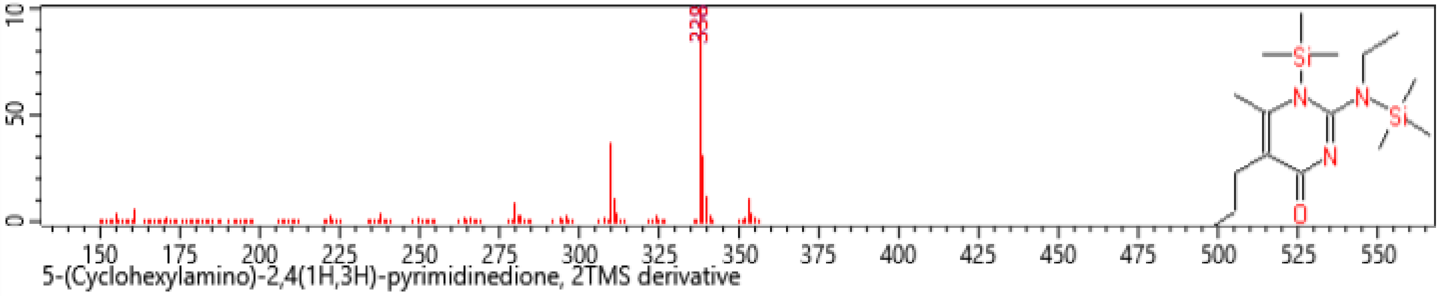

5-(Cyclohexylamino)-2,4(1H,3H)-pyrimidinedione, 2TMS derivative

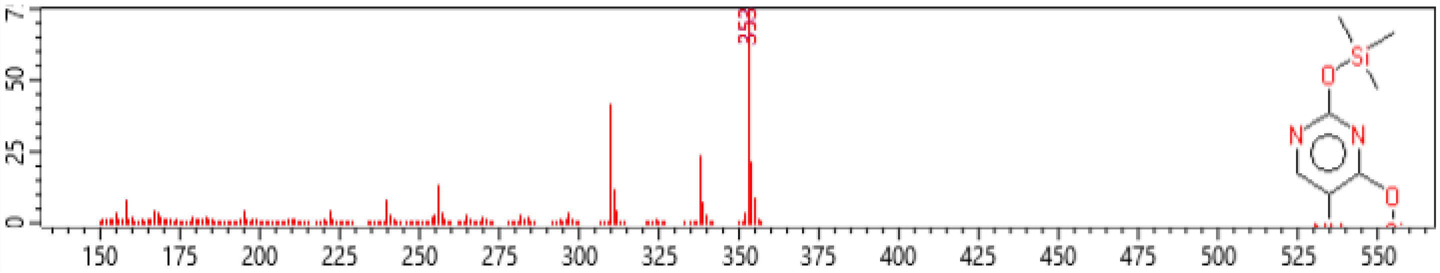

Malvidin 3-O-galactoside cation

Peak: 19.89 - 19.92 min (scan #3597 - #3605) Subtracted noise: 19.85 - 19.87 min (scan #3585 - #3592), 19.94 - 19.97 min (scan #36

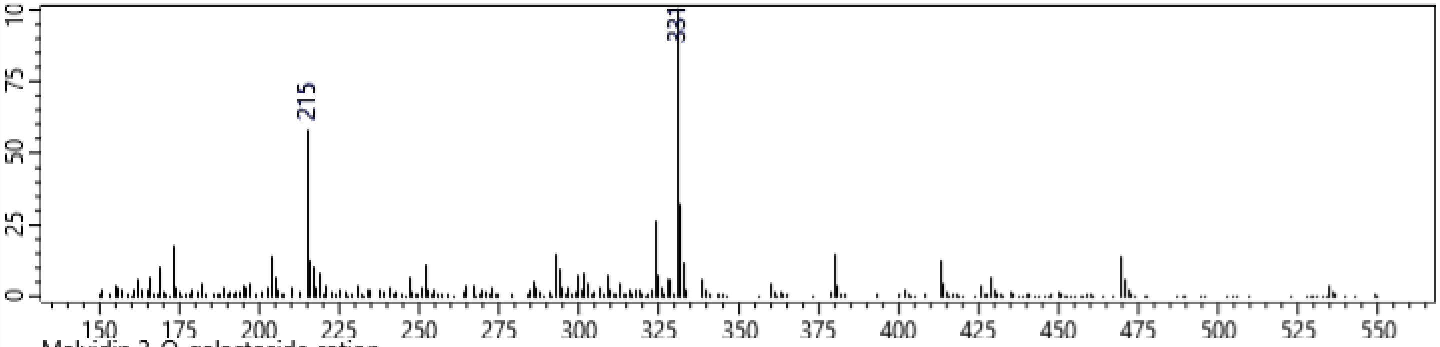

Malvidin 3-O-galactoside cation

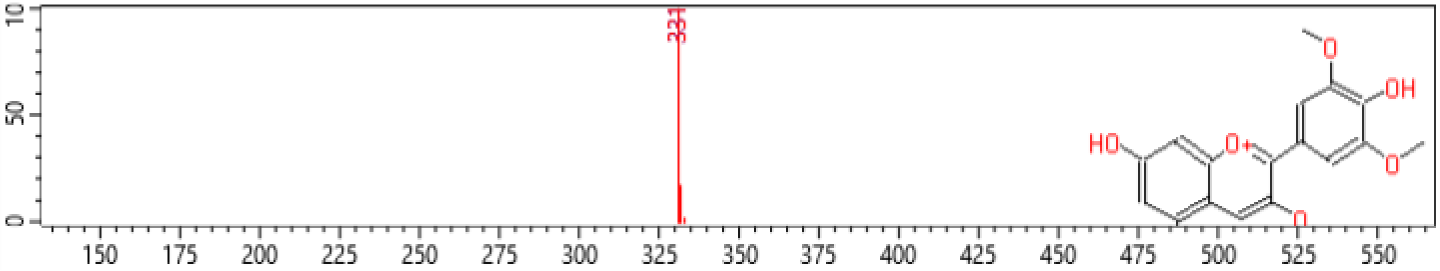

Malvidin 3-O-galactoside cation

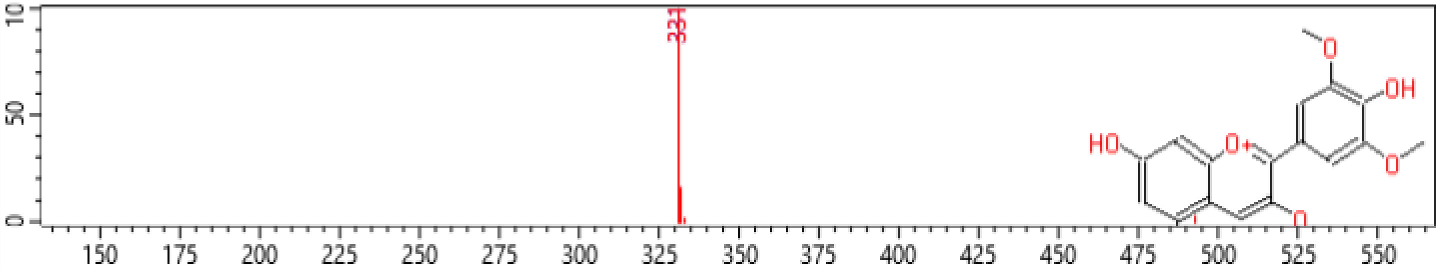

Malvidin 3-O-galactoside cation

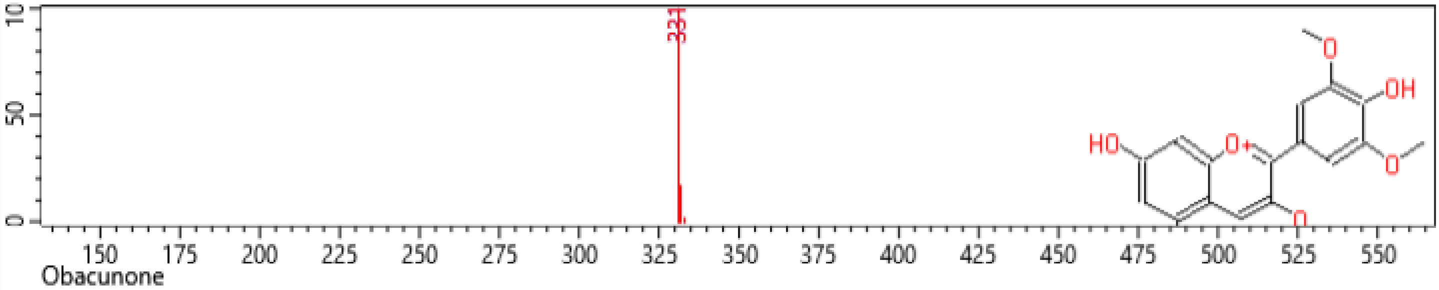

Obacunone

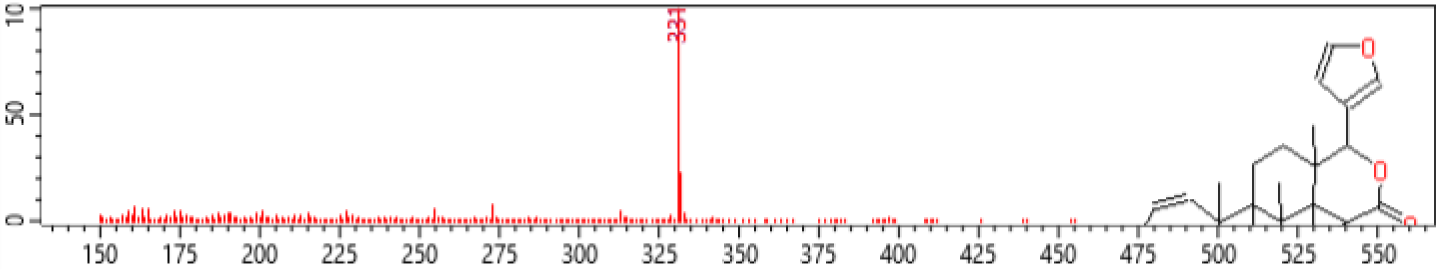

Obacunone

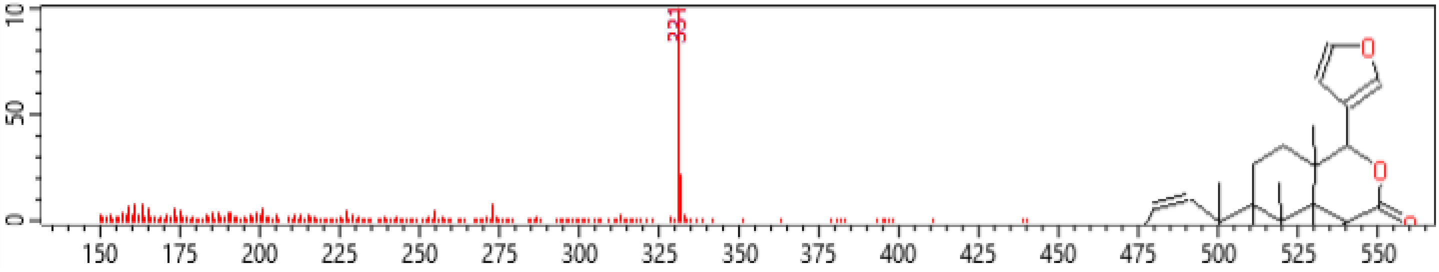

12-Hydroxyoctadecanoic acid, 2TMS derivative

Peak: 20.04 - 20.08 min (scan #3639 - #3649) Subtracted noise: 19.98 - 20.01 min (scan #3621 - #3631), 20.10 - 20.14 min (scan #36

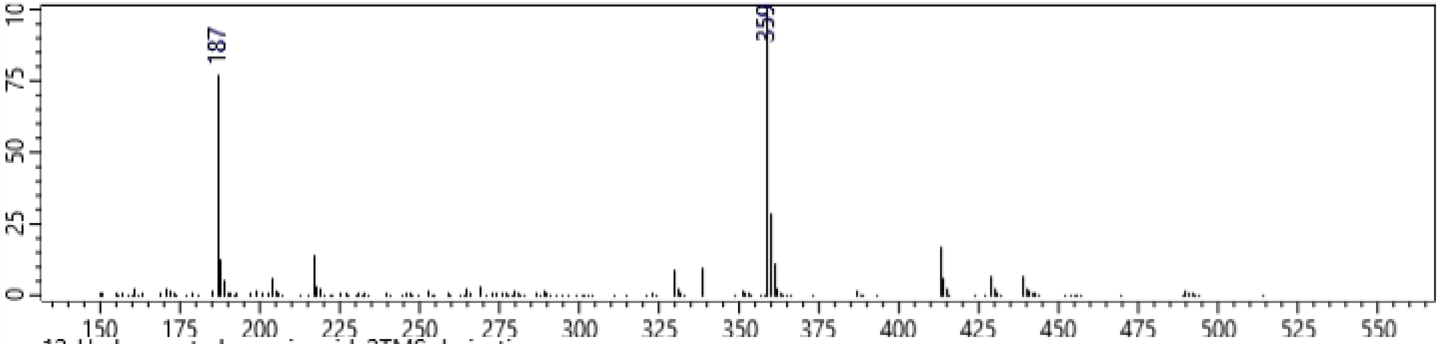

12-Hydroxyoctadecanoic acid, 2TMS derivative

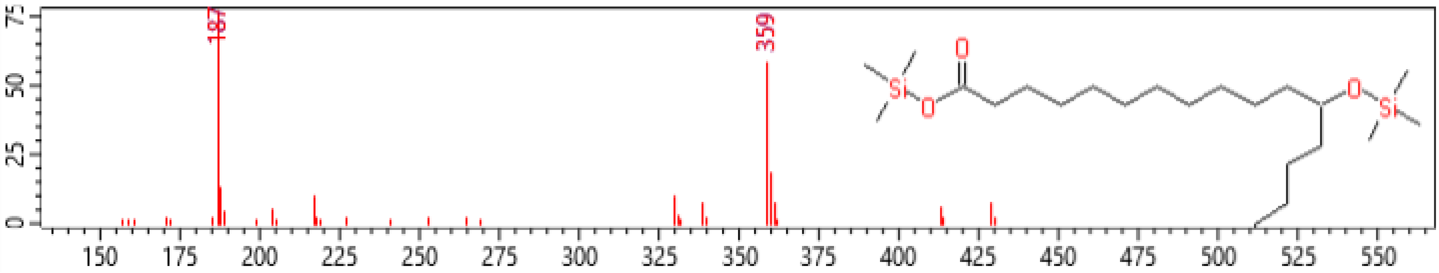

12-Hydroxyoctadecanoic acid, 2TMS derivative

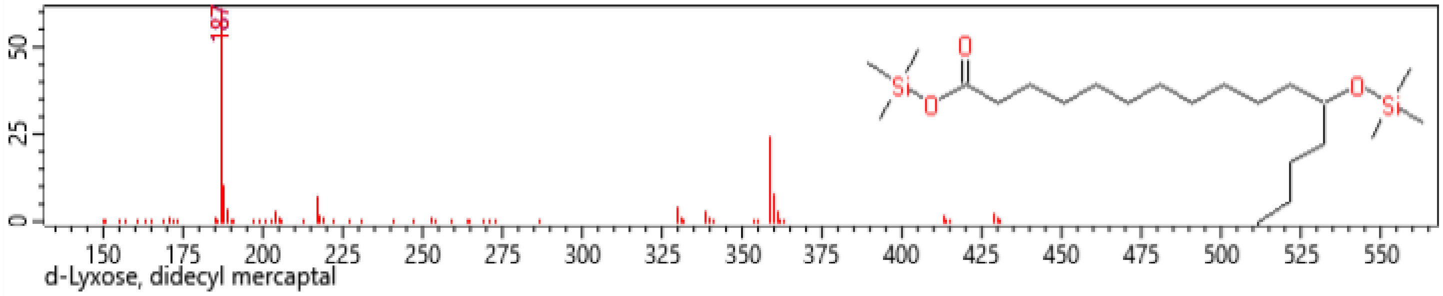

d-Lyxose, didecyl mercaptal

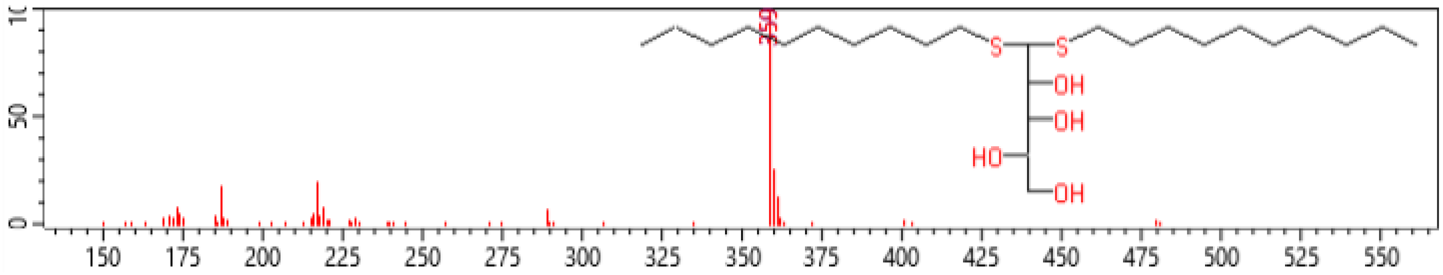

d-Galactose, didecyl mercaptal

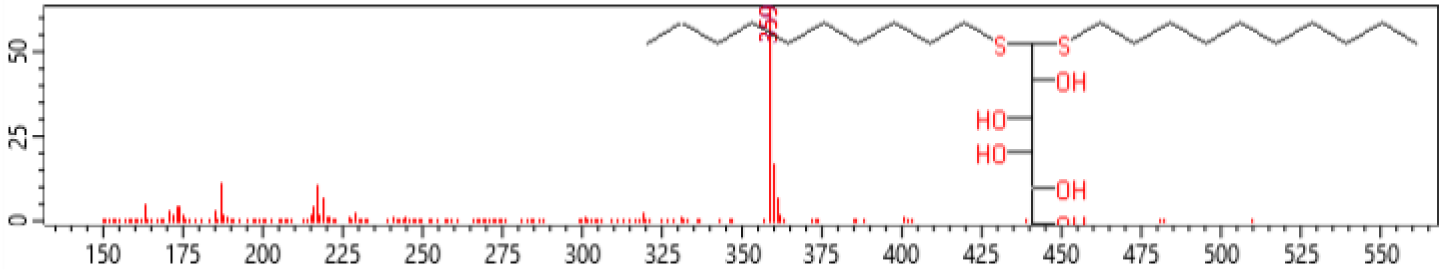

l-Arabinose, didecyl mercaptal

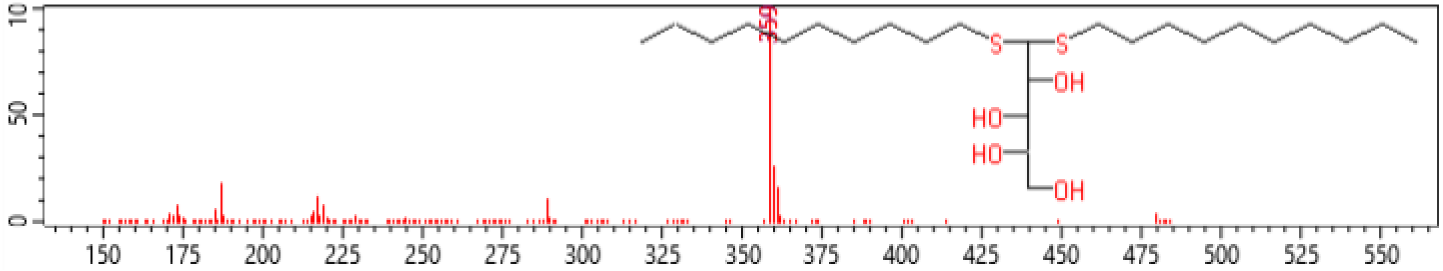

N-Trimethylsilylstearamide

Peak: 20.16 - 20.20 min (scan #3672 - #3684) Subtracted noise: 20.09 - 20.13 min (scan #3653 - #3664), 20.22 - 20.27 min (scan #36

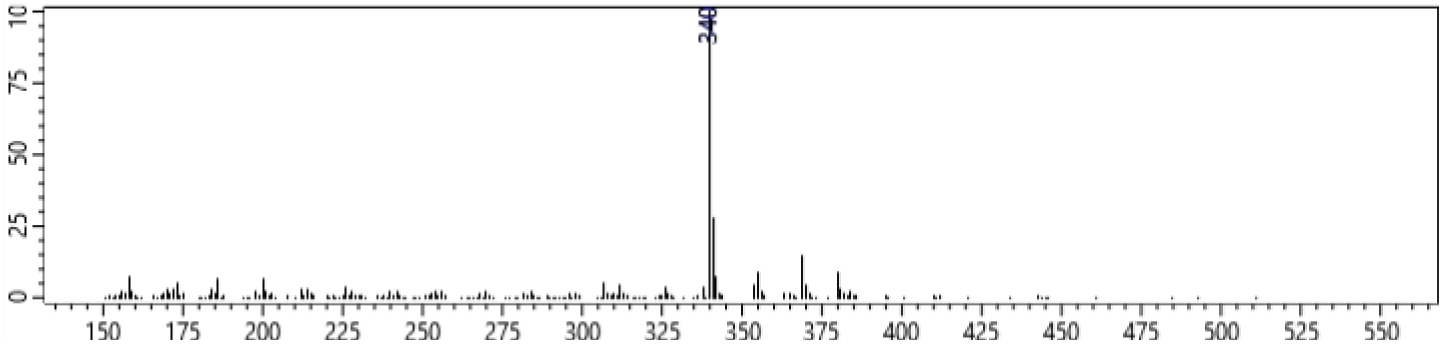

N-Trimethylsilylstearamide

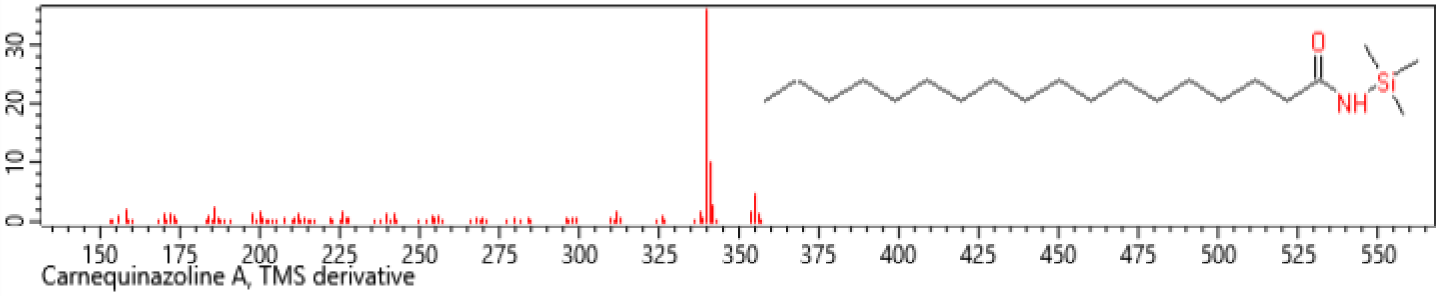

Carnequinazoline A, TMS derivative

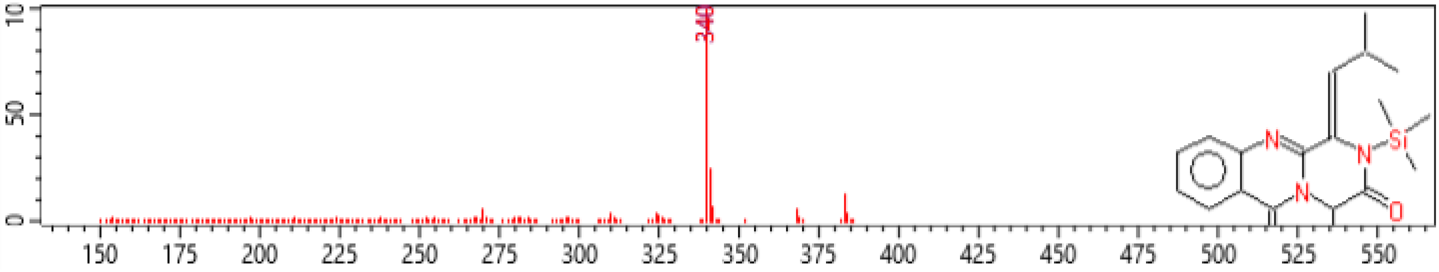

1,1'-Biphenyl, 3,4,4'-trimethoxy-2'-[4,5-dihydro-4,4-dimethyl-2-oxazolyl]-

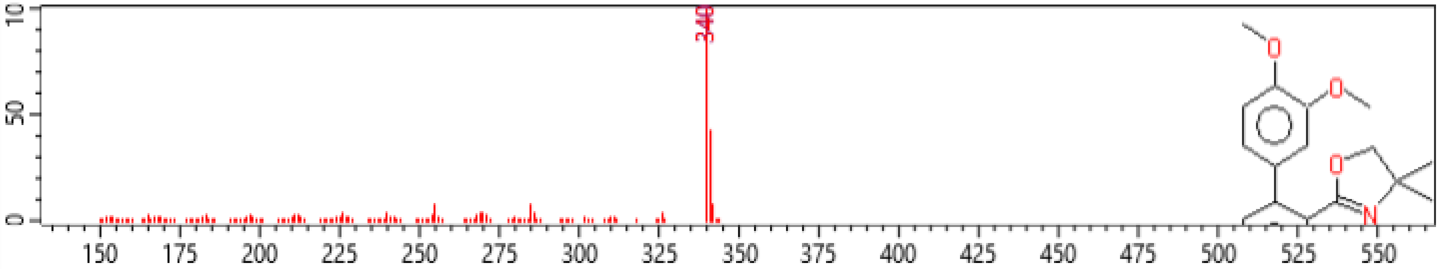

N-Phenyl-1H-pyrazolo[3,4-d]pyrimidin-4-amine, 2TMS derivative

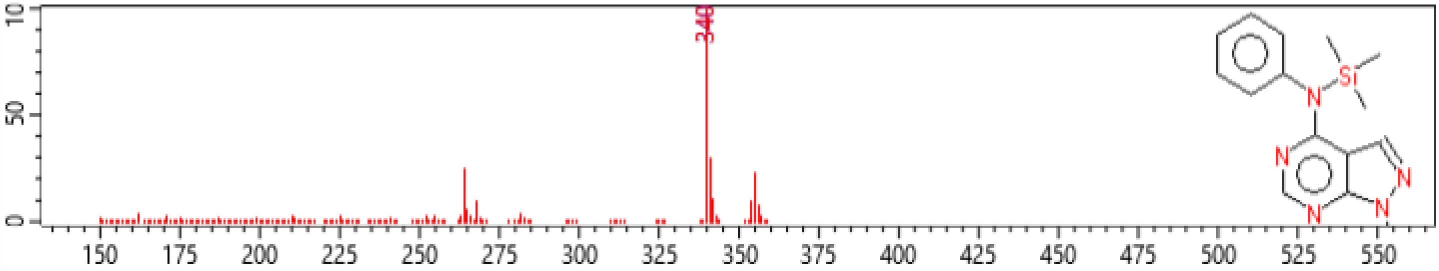

6,7-Dibromo-8-nitro-2,3-dihydro-1,4-benzodioxine-5-carboxylic acid

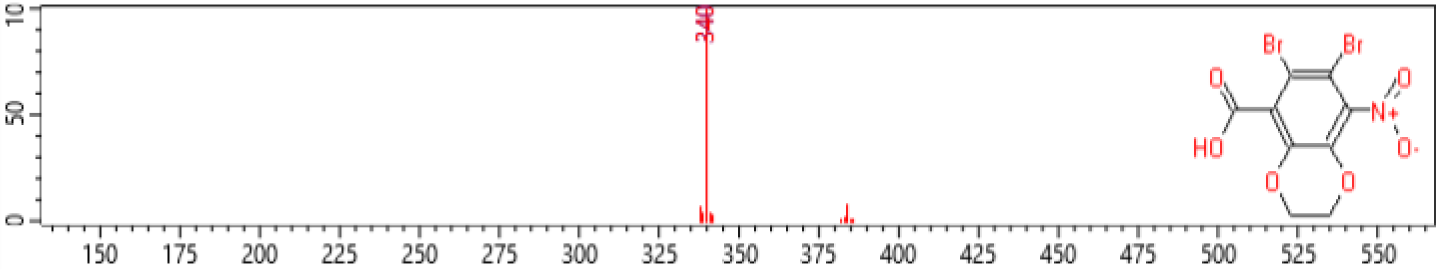

Topotecan

Peak: 20.49 - 20.51 min (scan #3764 - #3770) Subtracted noise: 20.44 - 20.47 min (scan #3751 - #3758), 20.53 - 20.55 min (scan #37

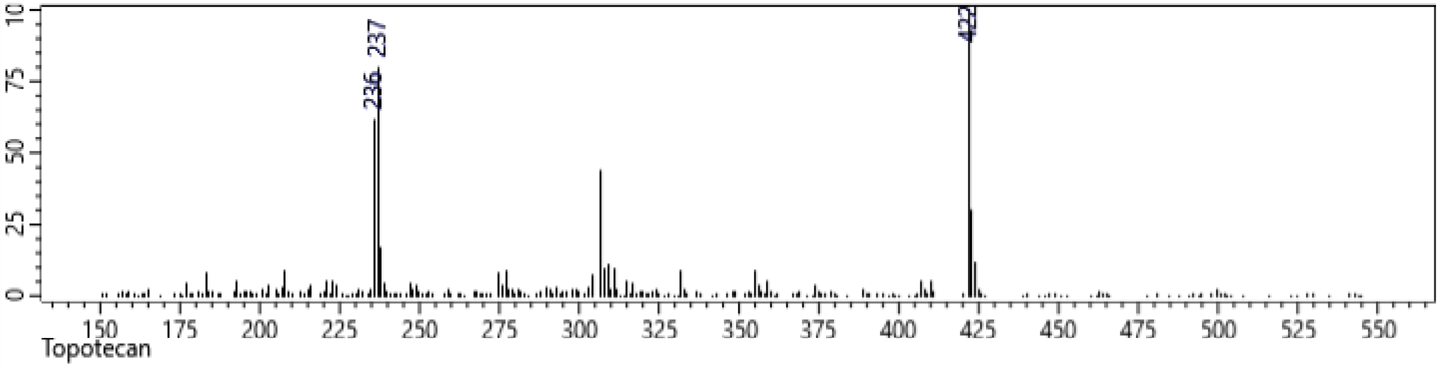

Topotecan

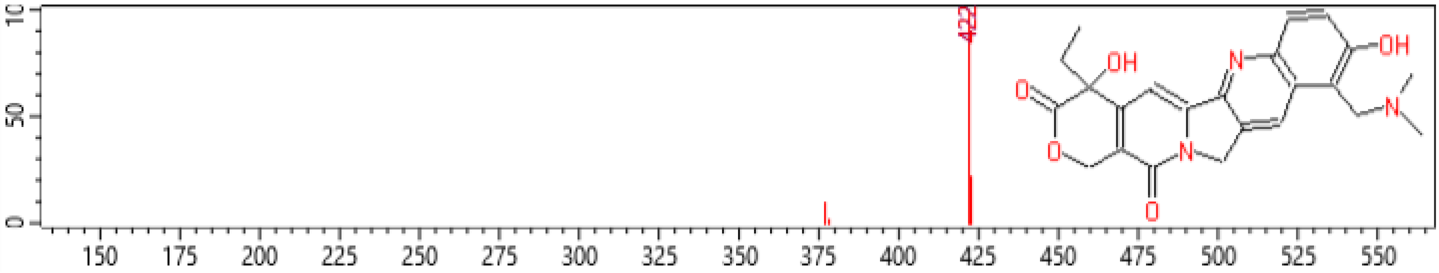

Topotecan

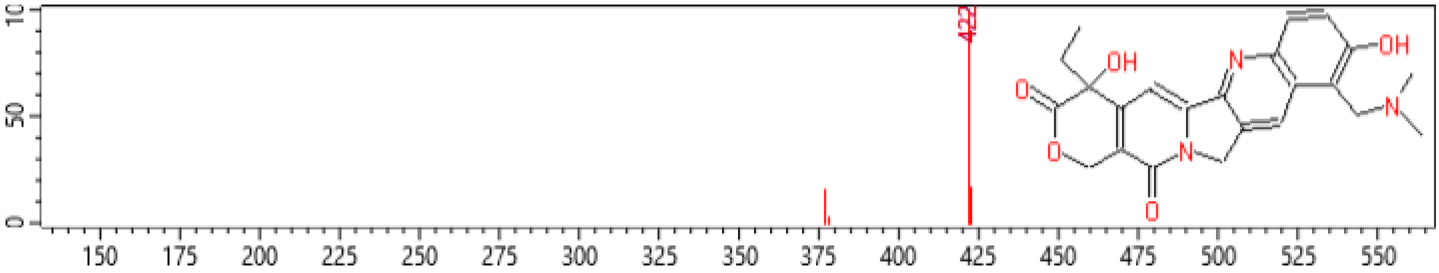

Gemcitabine diphosphate

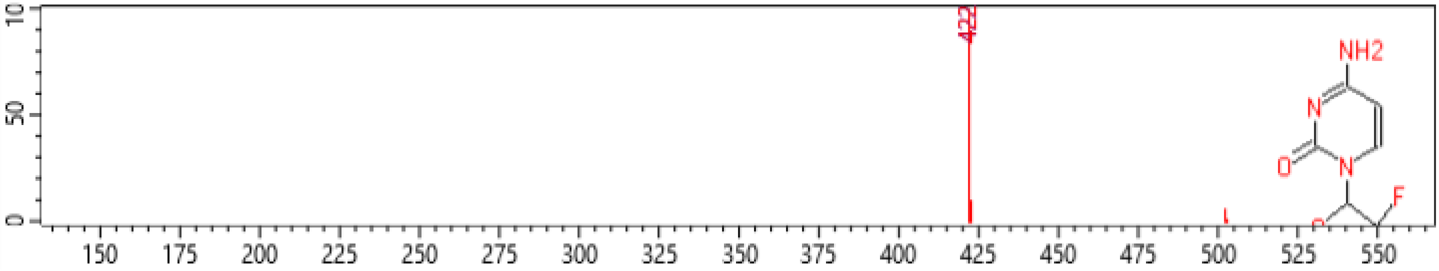

Gemcitabine diphosphate

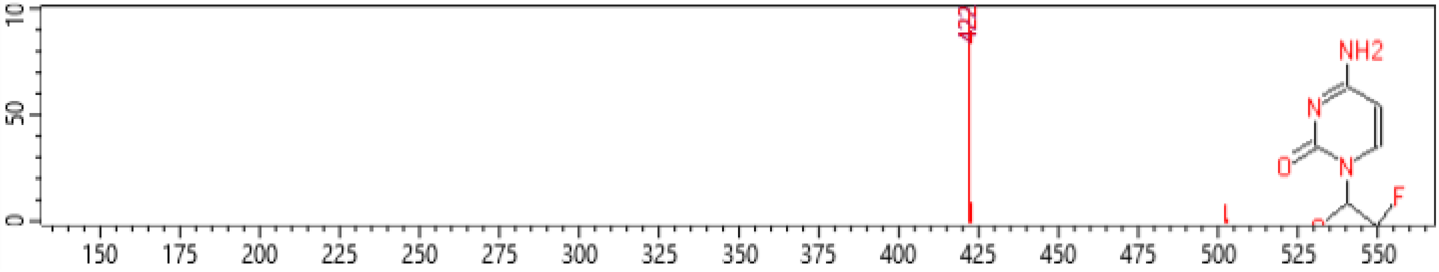

L-Serine, N-(1-oxohexadecyl)-O-phosphono-

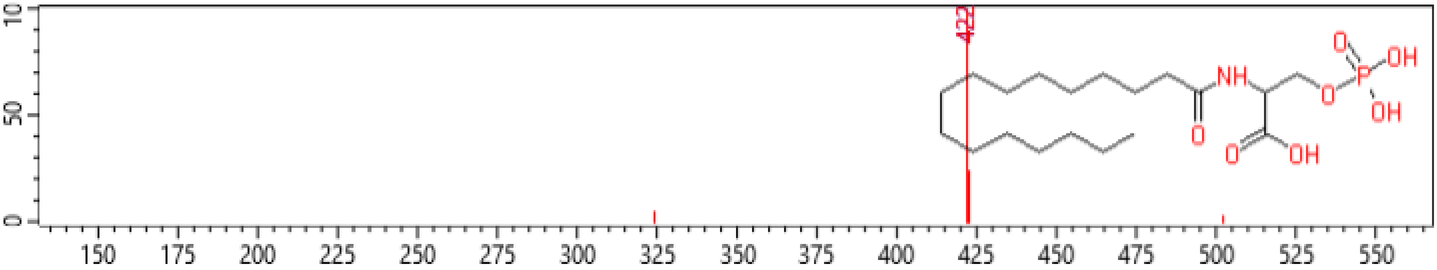

2,2'-Methylenebis(6-tert-butyl-4 methylphenol), O,O'-bis(trimethylsilyl)-

Peak: 21.30 - 21.34 min (scan #3991 - #4003) Subtracted noise: 21.22 - 21.27 min (scan #3969 - #3982), 21.37 - 21.41 min (scan #40

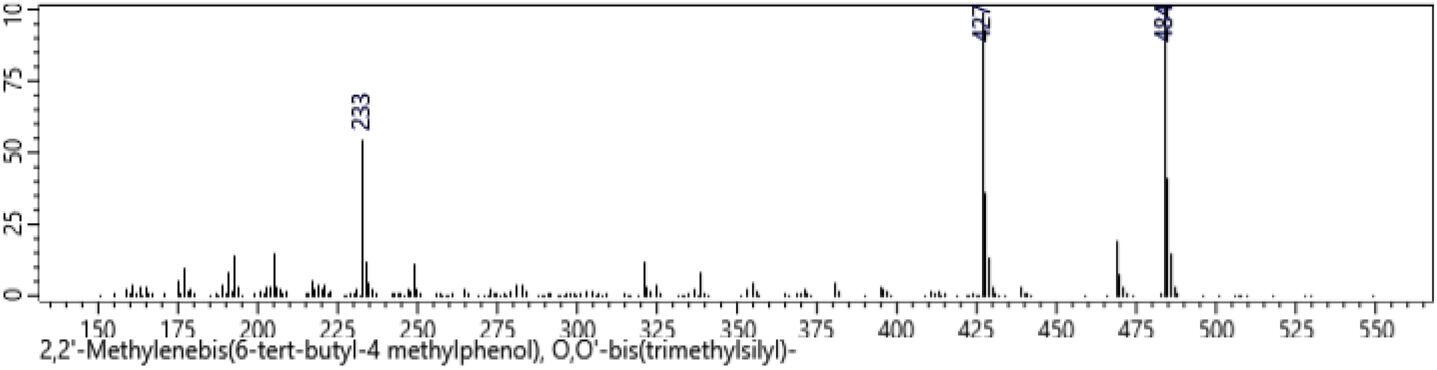

2,2'-Methylenebis(6-tert-butyl-4 methylphenol), O,O'-bis(trimethylsilyl)-

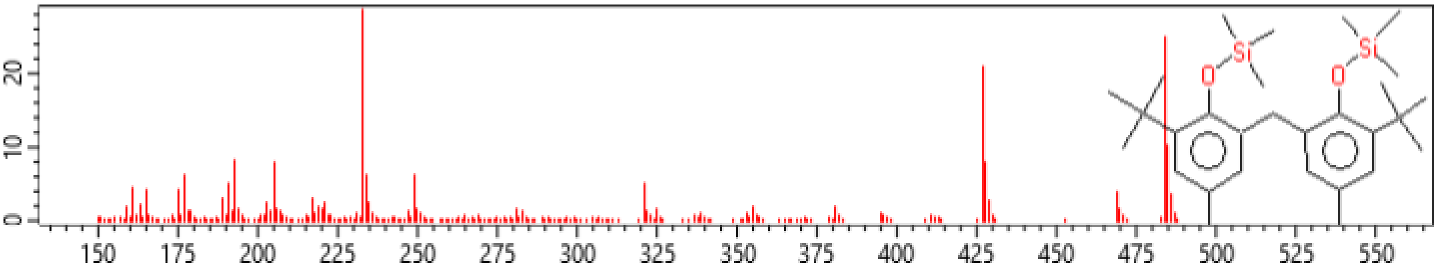

benzene, 1,1'-methylenebis[3-(1,1-dimethylethyl)-5-methyl-4-[(trimethylsilyl)oxy]-

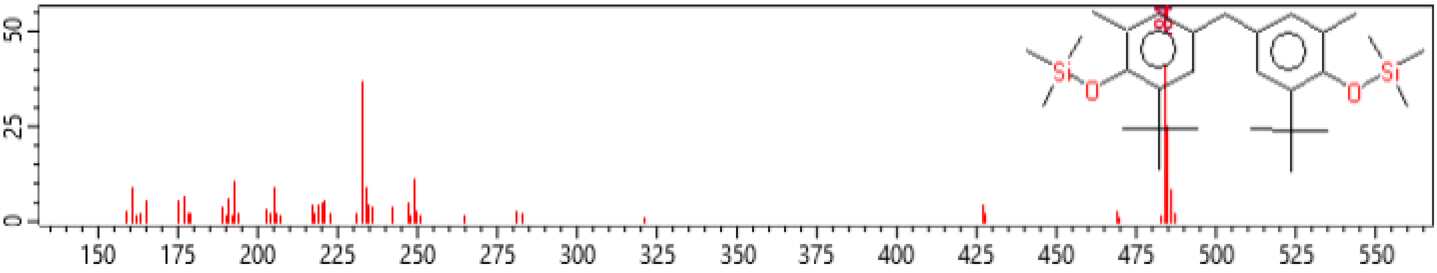

6,2'-Bis(tert.-butyldimethylsilyl)oxyflavanone

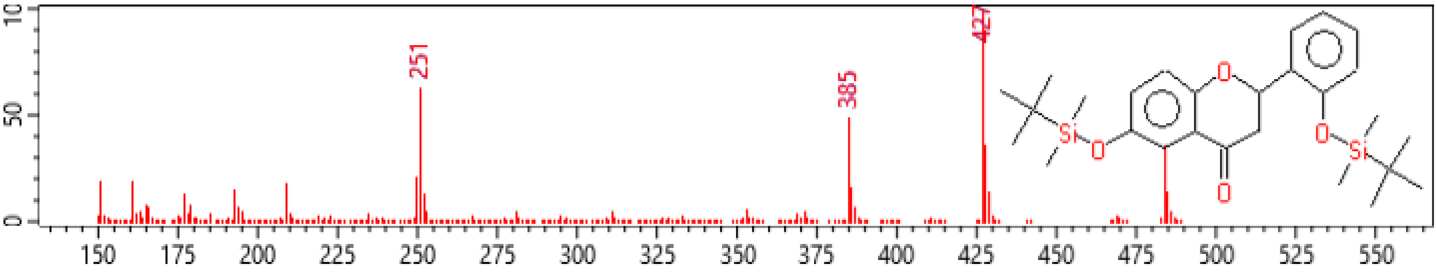

2-(2-Chloro-5-iodophenyl)-1,3-benzoxazol-5-amine, N-(tert-butyldimethylsilyl)-

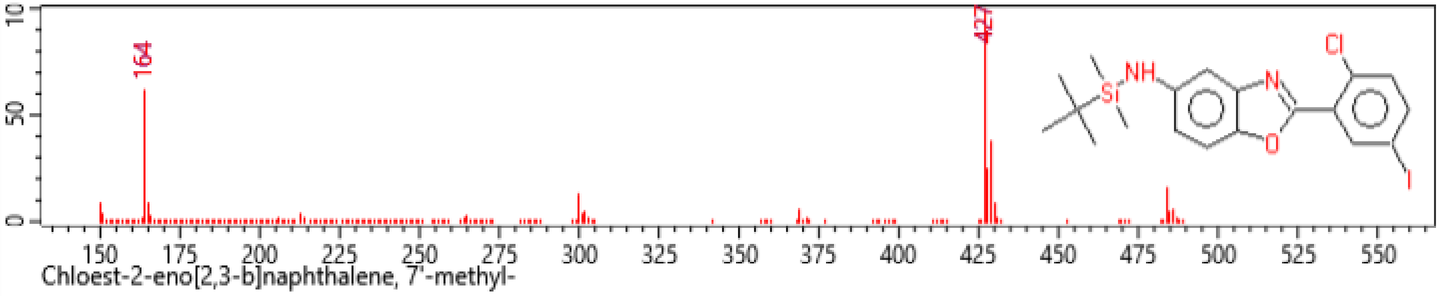

Chloest-2-eno[2,3-b]naphthalene, 7'-methyl-

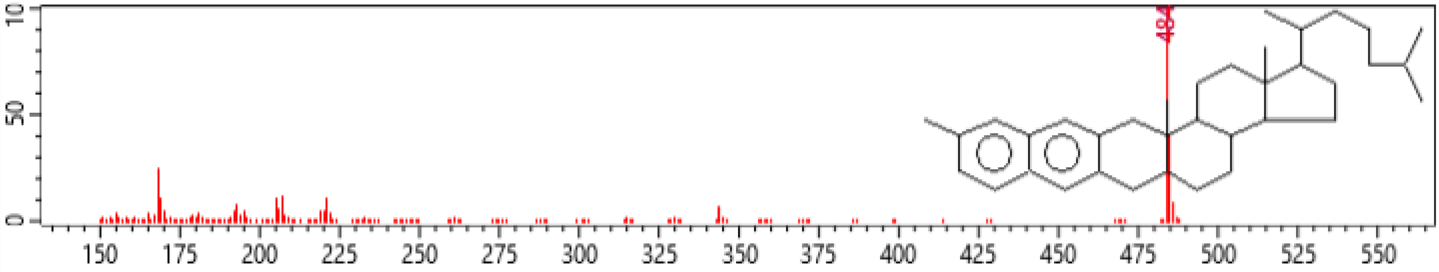

Cyclopentanepropanoic acid, 3,5-bis[(trimethylsilyl)oxy]-2-[3-[(trimethylsilyl)oxy]-1-octenyl]-, methyl ester, [1R-[1α,2β

Peak: 21.52 - 21.56 min (scan #4052 - #4062) Subtracted noise: 21.46 - 21.49 min (scan #4034 - #4044), 21.58 - 21.62 min (scan #40

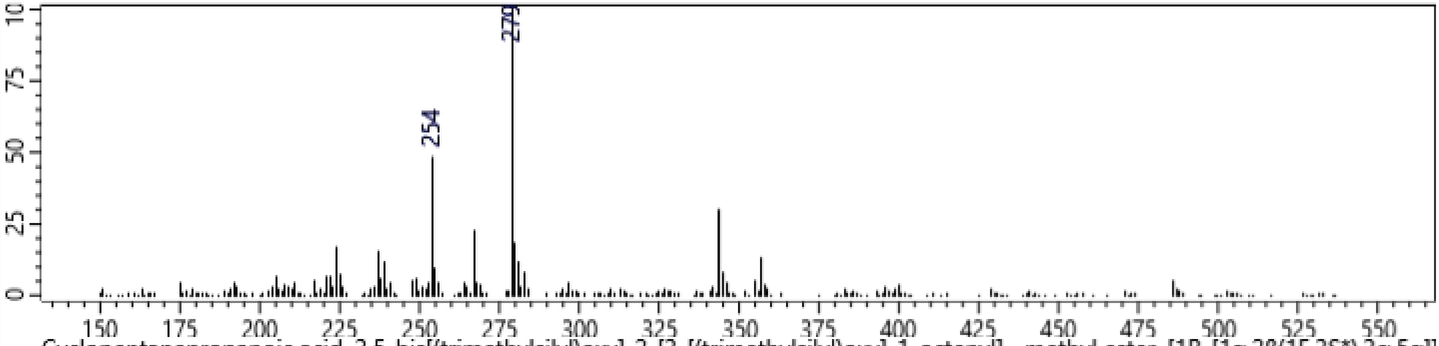

Cyclopentanepropanoic acid, 3,5-bis[(trimethylsilyl)oxy]-2-[3-[(trimethylsilyl)oxy]-1-octenyl]-, methyl ester, [1R-[1α,2β(1E,3S*),3α,5α]]

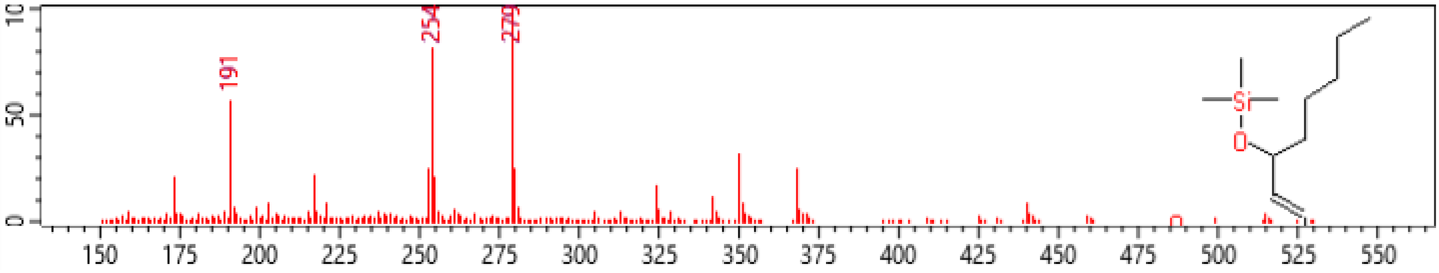

6-Norlysergic acid diethylamide, TMS derivative

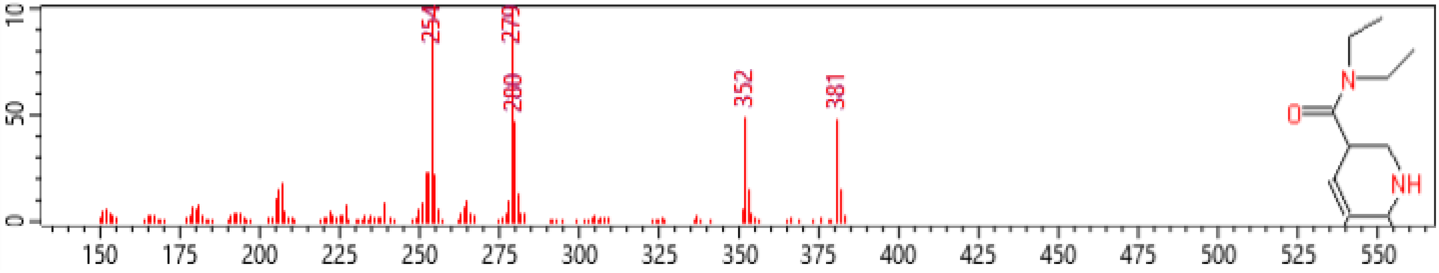

(7E,13E)-11,12-Dihydroxy-3-isobutyl-4,5,8-trimethyl-3,3a,4,6a,9,10,11,12-octahydro-1H-[1]oxacyclododecino[2,3-d]isoindole-1,15(2H

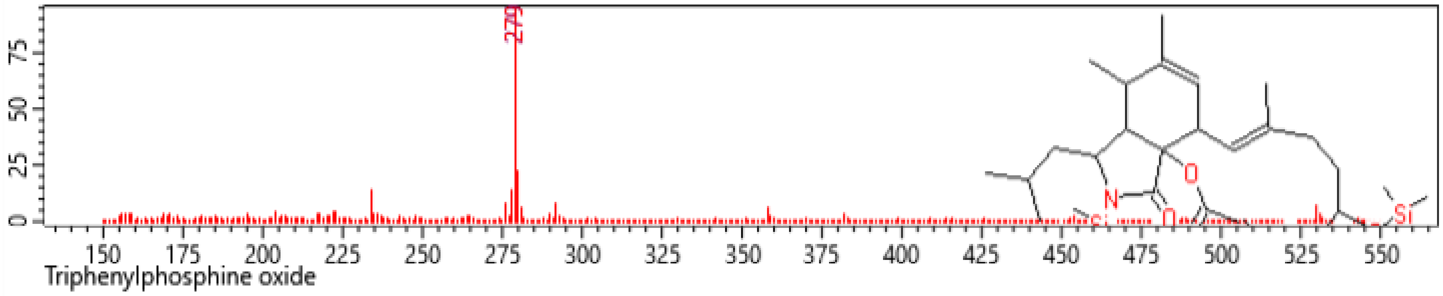

Triphenylphosphine oxide

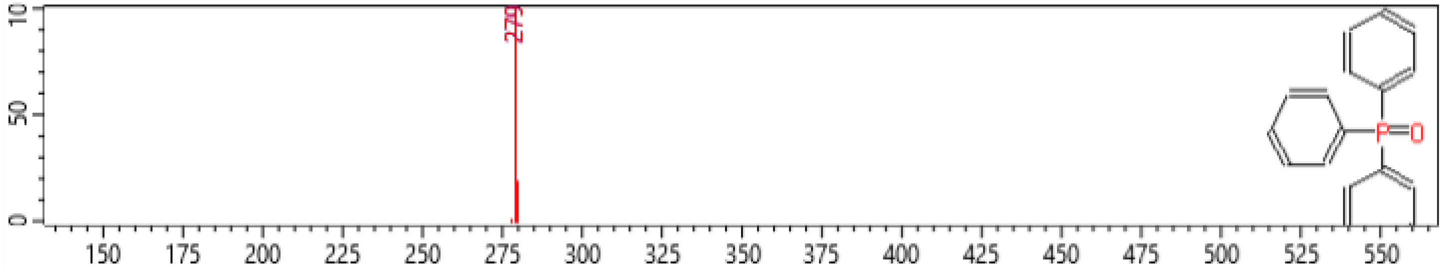

Colchicine

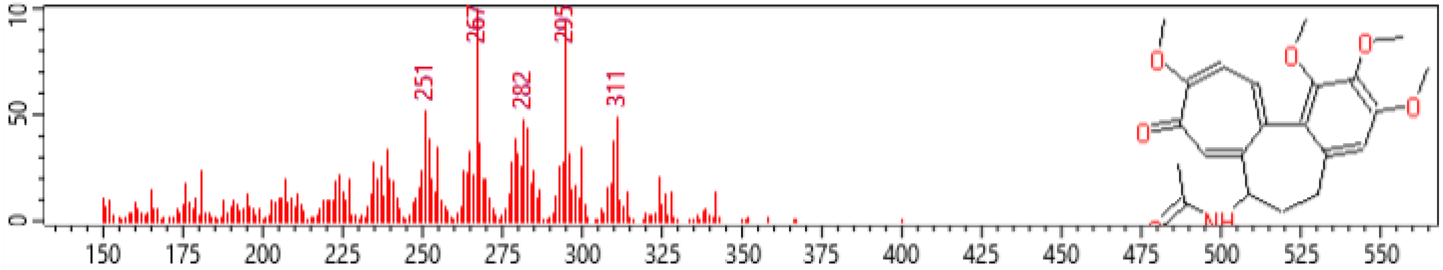

4-Hydroxy-3-methoxyphenylglycol, 3TMS derivative

Peak: 22.94 - 22.99 min (scan #4449 - #4461) Subtracted noise: 22.86 - 22.90 min (scan #4426 - #4438), 23.01 - 23.05 min (scan #44

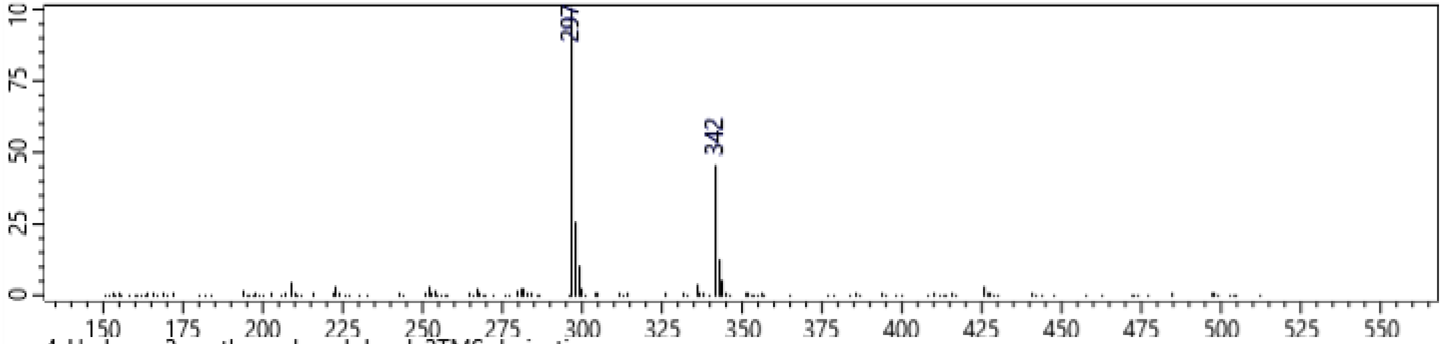

4-Hydroxy-3-methoxyphenylglycol, 3TMS derivative

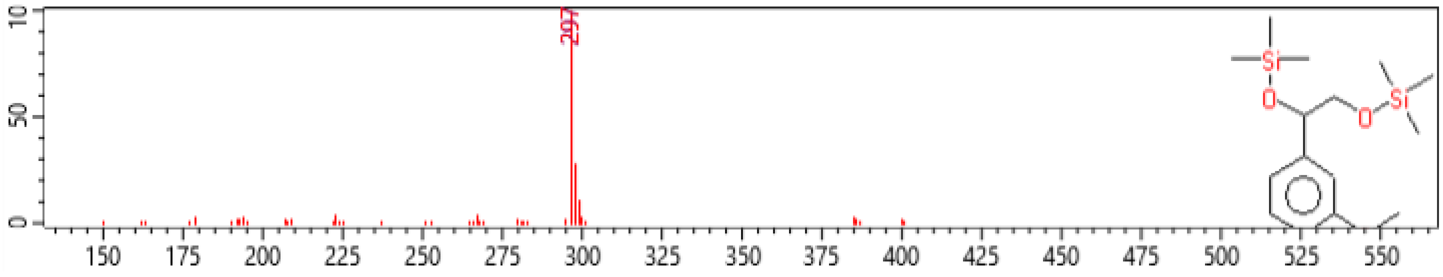

4-Hydroxy-3-methoxyphenylglycol, 3TMS derivative

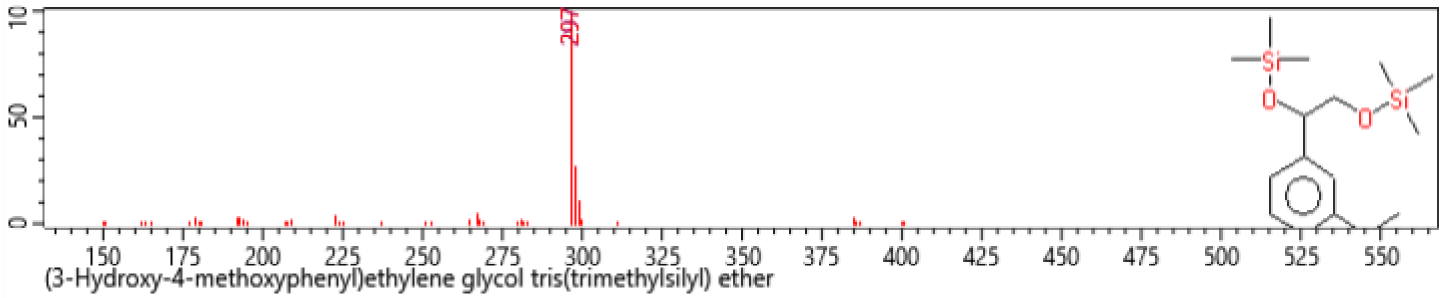

(3-Hydroxy-4-methoxyphenyl)ethylene glycol tris(trimethylsilyl) ether

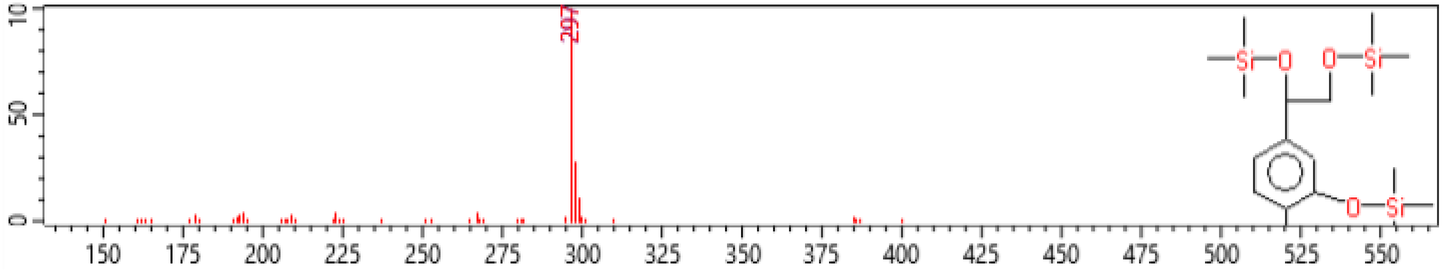

Methyl 2,5-dihydroxybenzoate, 2TMS derivative

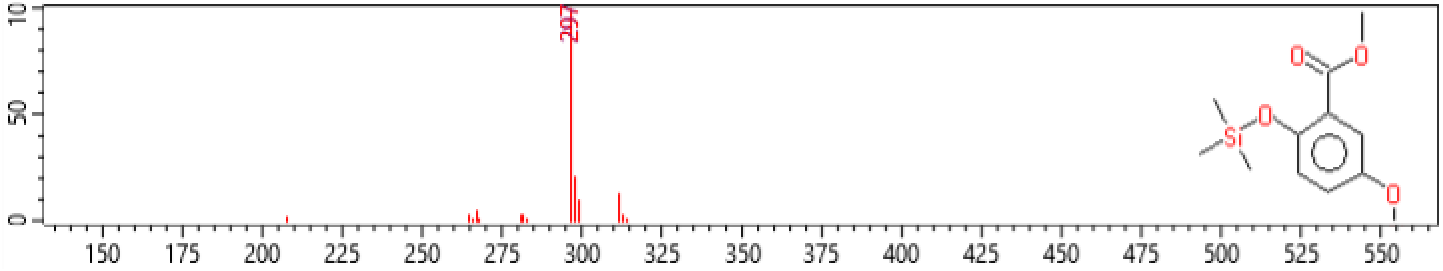

3-Hydroxy-3-(4'-hydroxy-3'-methoxyphenyl)propionic acid, tri-TMS

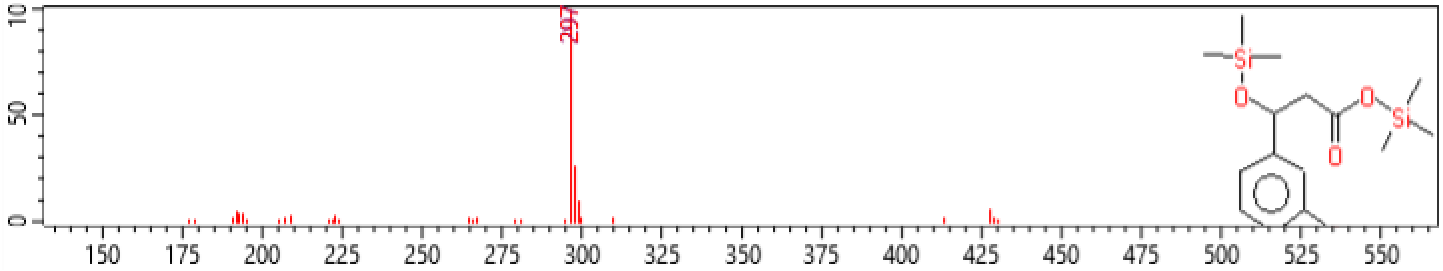

1-Monopalmitin, 2TMS derivative

Peak: 23.56 - 23.72 min (scan #4622 - #4666) Subtracted noise: 23.22 - 23.37 min (scan #4526 - #4569), 23.70 - 23.85 min (scan #46

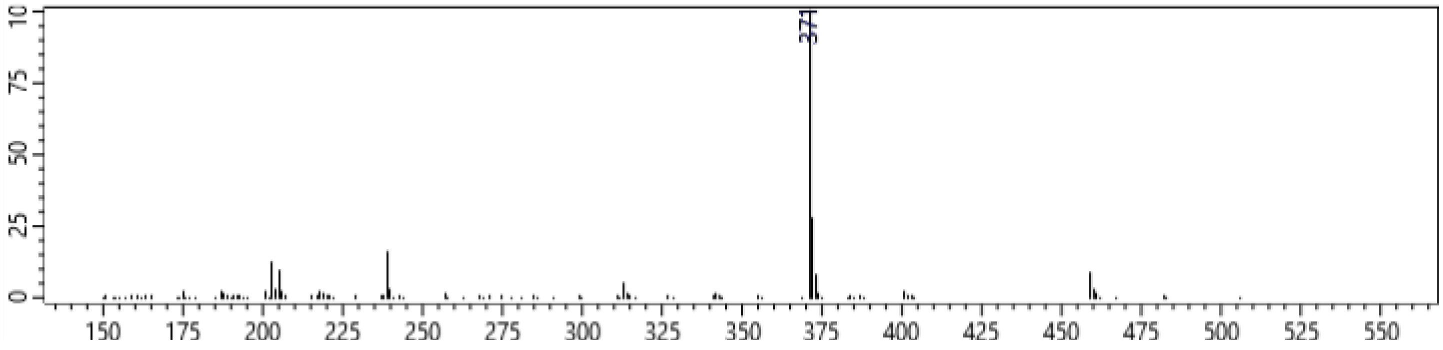

1-Monopalmitin, 2TMS derivative

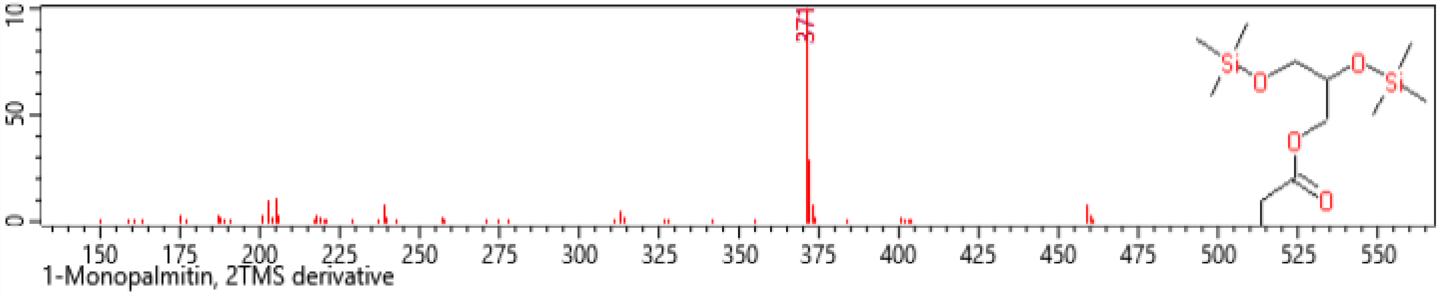

1-Monopalmitin, 2TMS derivative

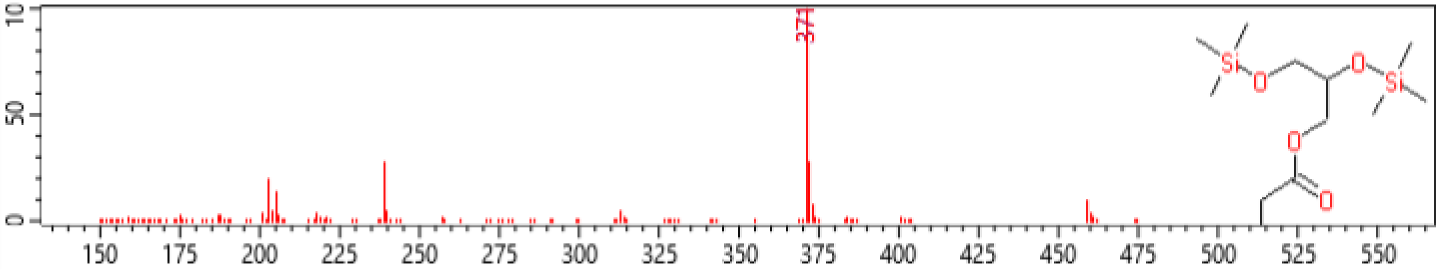

1-Monopalmitin, 2TMS derivative

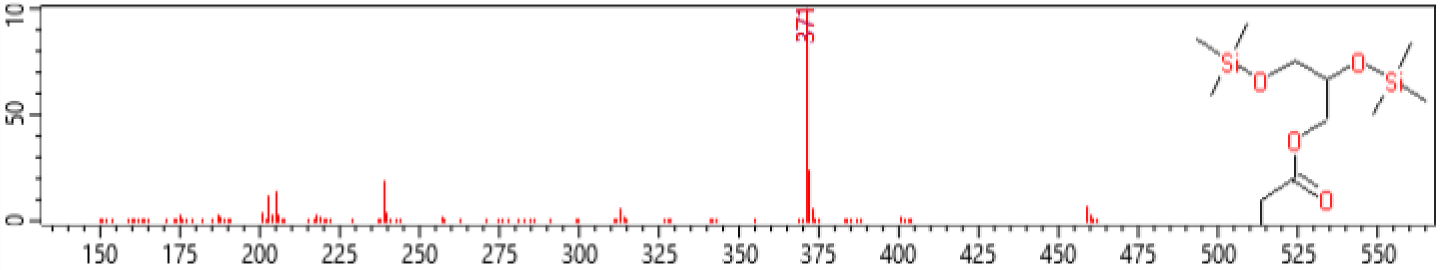

1-Monopalmitin, 2TMS derivative

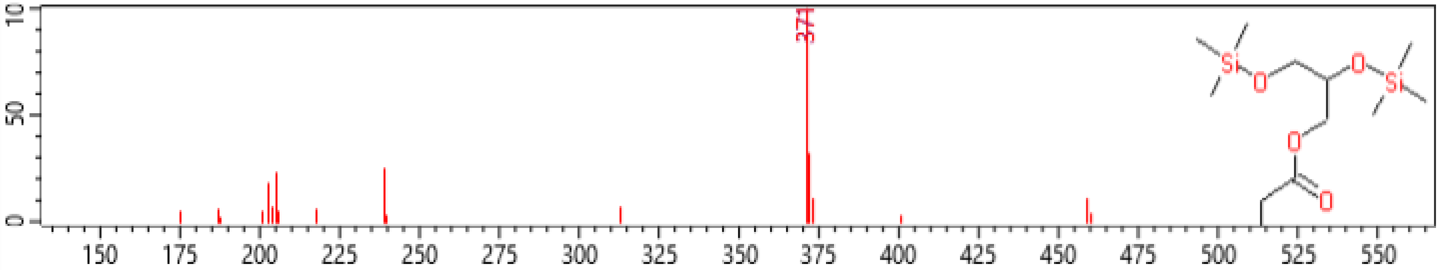

1-Monopalmitin, 2TMS derivative

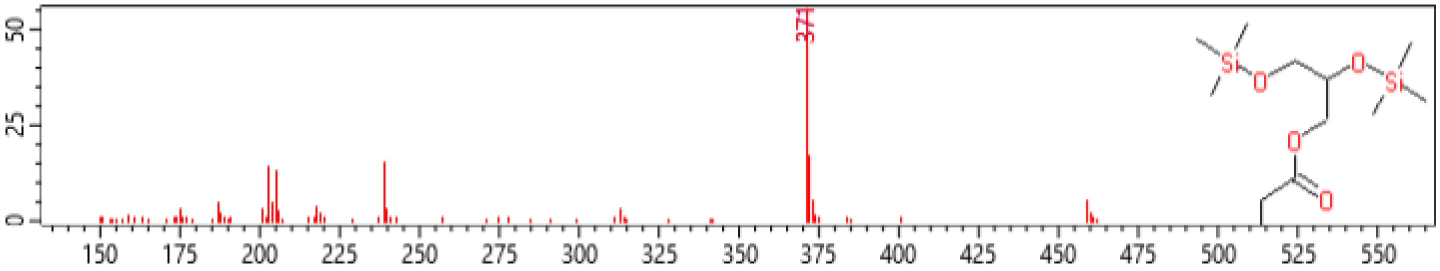

Acetamide, 2,2'-[[2-(2,5-dihydro-2,5-dioxo-3,4-diphenyl-1H-pyrrol-1-yl)-2-methyl-1,3-propanediyl]bis(oxy)]bis[N-n-

Peak: 23.72 - 23.78 min (scan #4667 - #4683) Subtracted noise: 23.64 - 23.70 min (scan #4643 - #4659), 23.82 - 23.87 min (scan #46

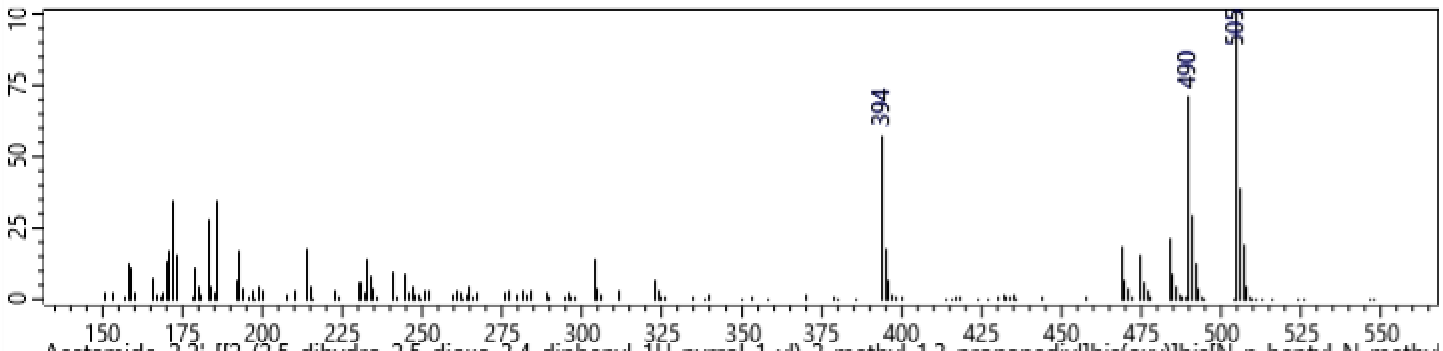

Acetamide, 2,2'-[[2-(2,5-dihydro-2,5-dioxo-3,4-diphenyl-1H-pyrrol-1-yl)-2-methyl-1,3-propanediyl]bis(oxy)]bis[N-n-heptyl-N-methyl

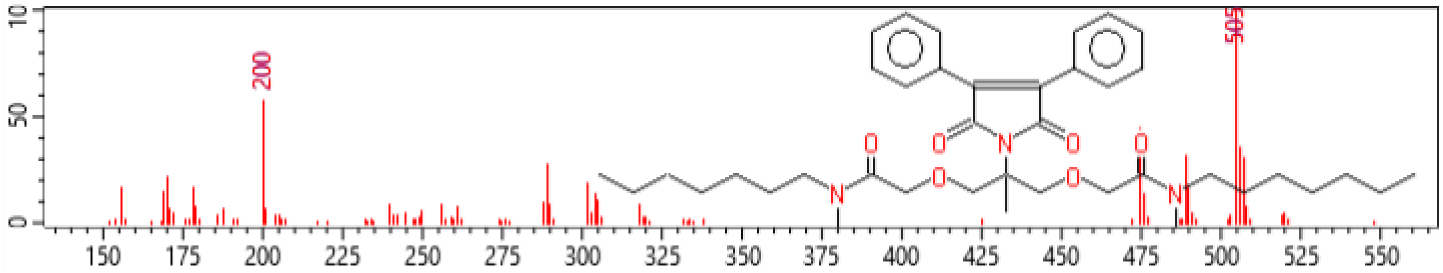

dodecanamide, N-[2-methoxy-4-[[(4-nitrophenyl)sulfonyl]amino]phenyl]-

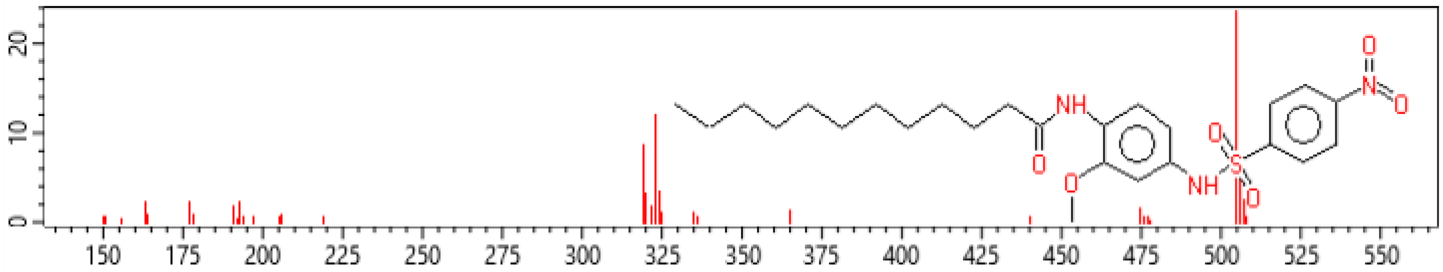

Flag Peptide

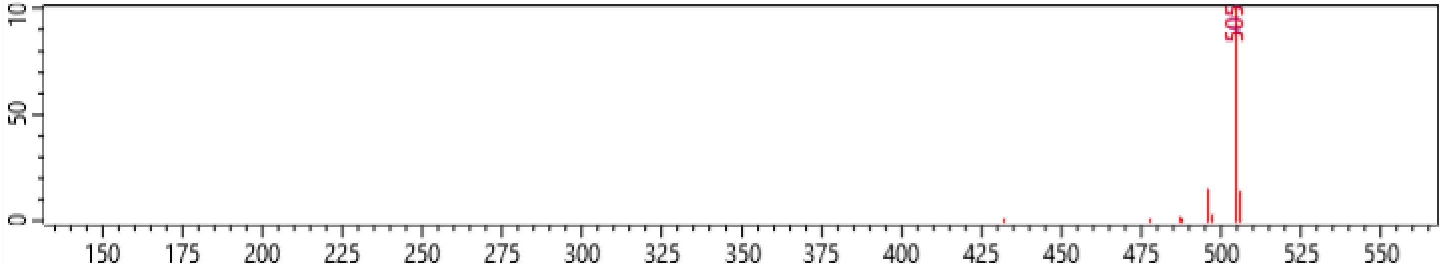

Corymbiferin, 3TMS (isomer 1)

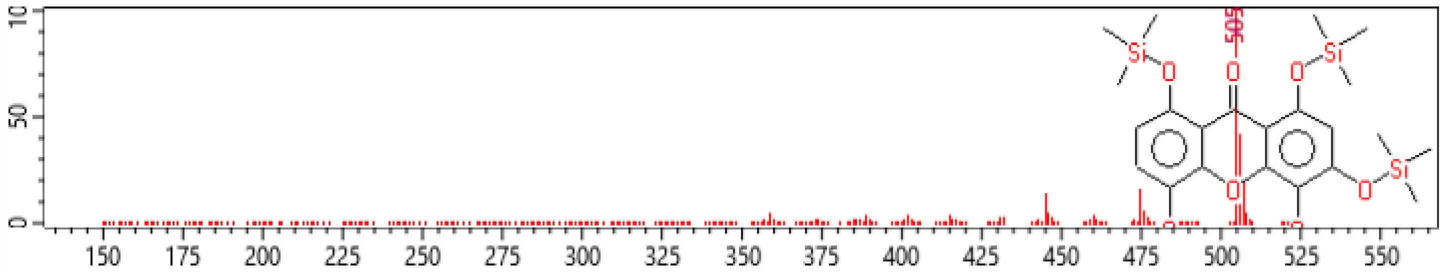

Corymbiferin, 3TMS (isomer 2)

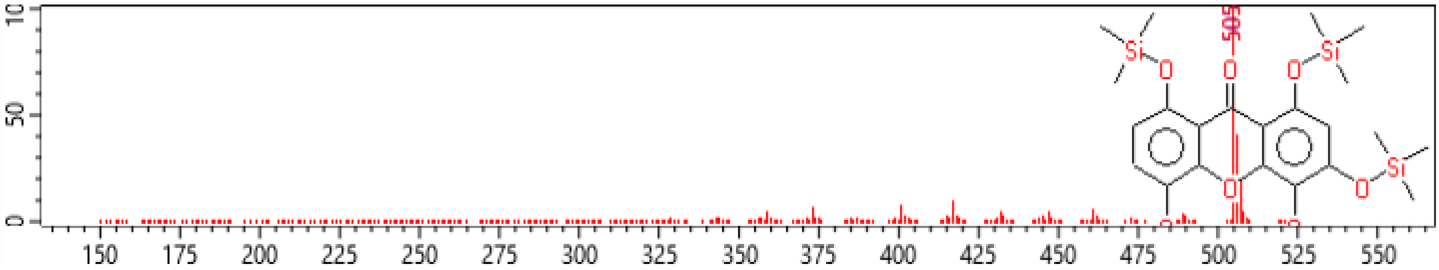

Thiazole, 2-(phenylthio)-

Peak: 24.91 - 24.99 min (scan #4999 - #5019) Subtracted noise: 24.80 - 24.87 min (scan #4967 - #4986), 25.02 - 25.09 min (scan #50

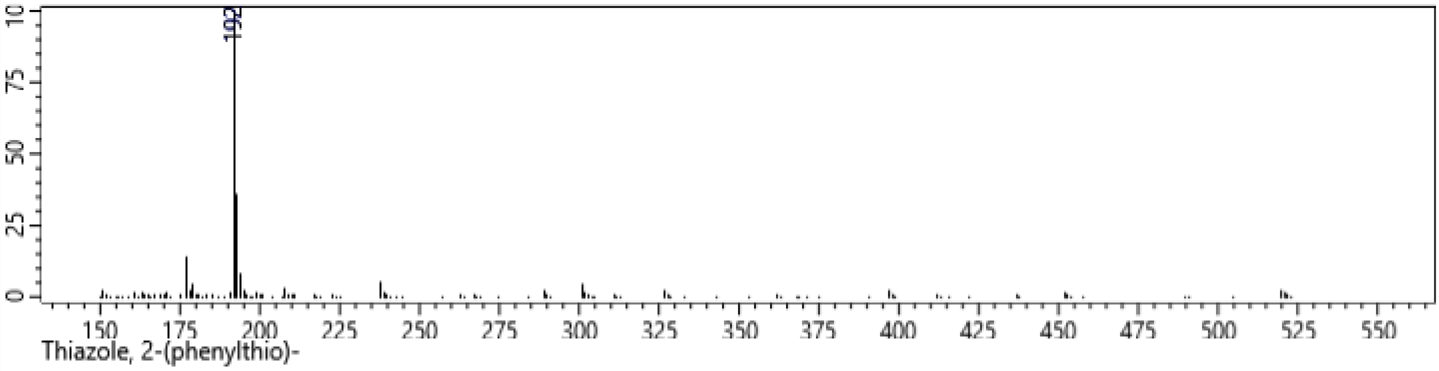

Thiazole, 2-(phenylthio)-

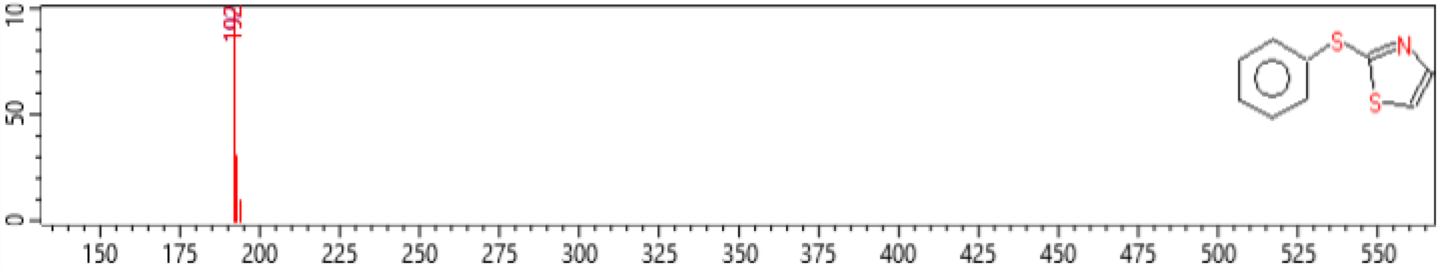

4-((Trimethylsilyl)oxy)phenethyl acetate

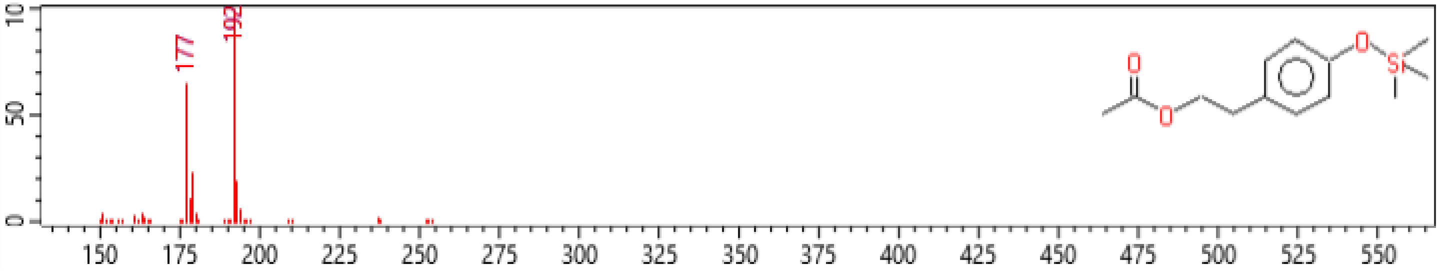

2,4-Dodecadienamide, N-(p-hydroxyphenethyl)-, (E,E)-, TMS

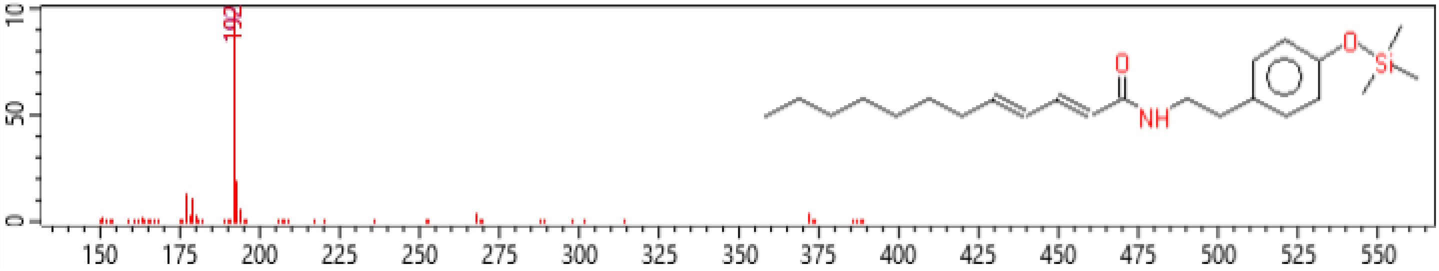

2-Trimethylsilyl-1,3-dithiane

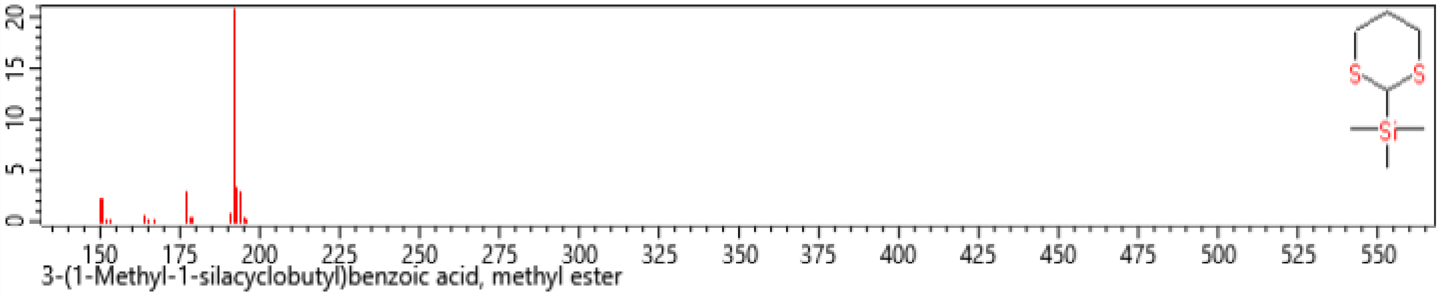

3-(1-Methyl-1-silacyclobutyl)benzoic acid, methyl ester

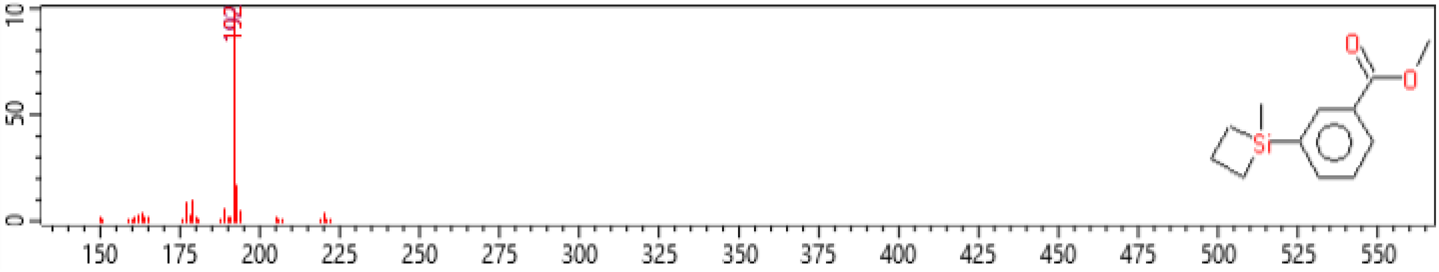

6-Methyl[1,3]dioxolo[4,5-f][1,3]benzothiazole

Peak: 25.71 - 25.77 min (scan #5222 - #5238) Subtracted noise: 25.63 - 25.68 min (scan #5198 - #5213), 25.80 - 25.86 min (scan #52

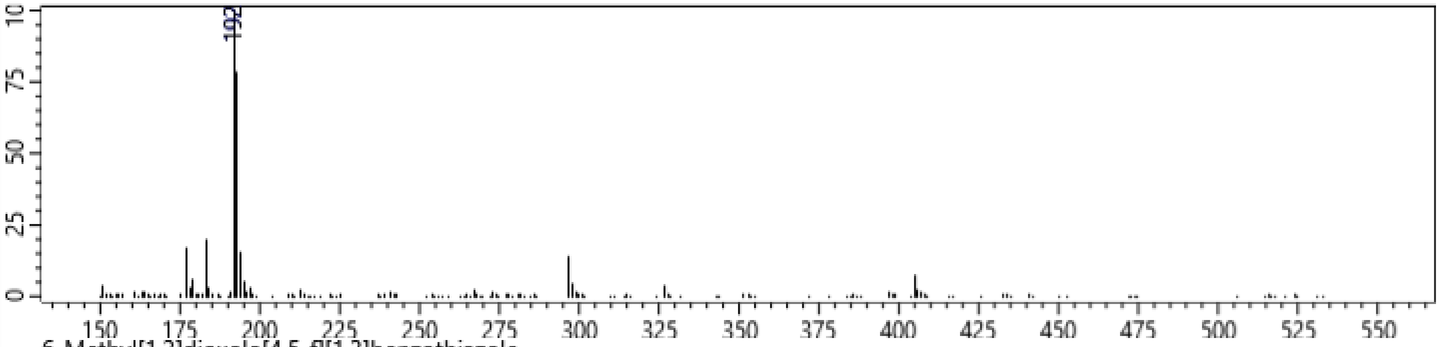

6-Methyl[1,3]dioxolo[4,5-f][1,3]benzothiazole

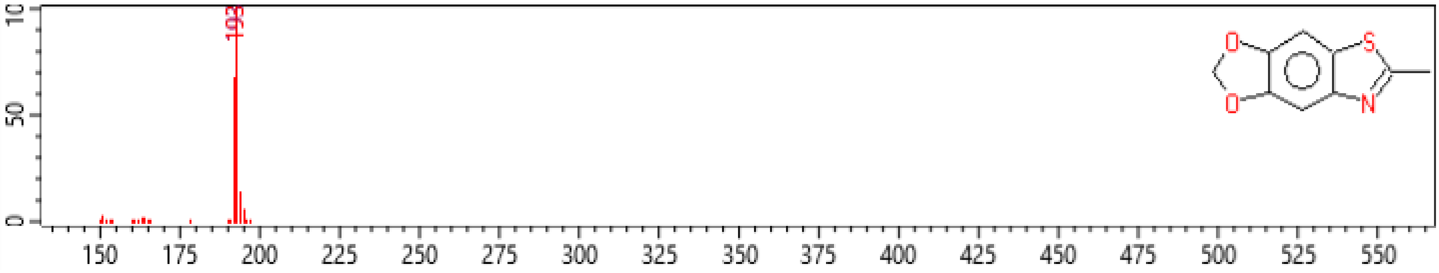

4-((Trimethylsilyl)oxy)phenethyl acetate

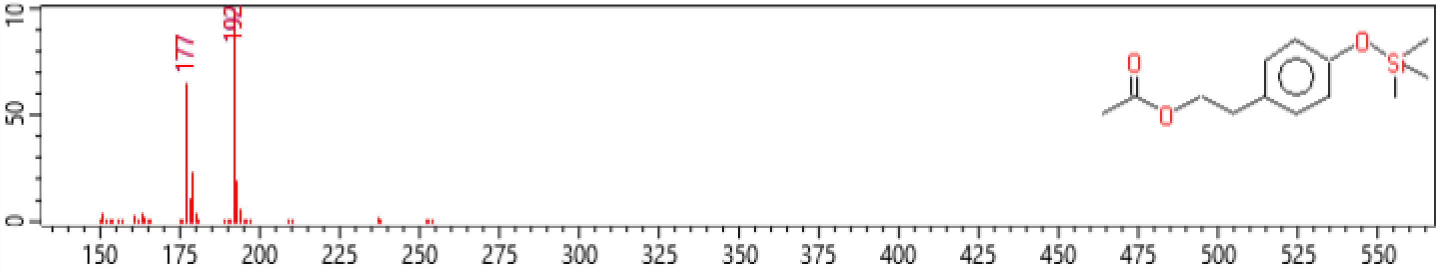

2,4-Dodecadienamide, N-(p-hydroxyphenethyl)-, (E,E)-, TMS

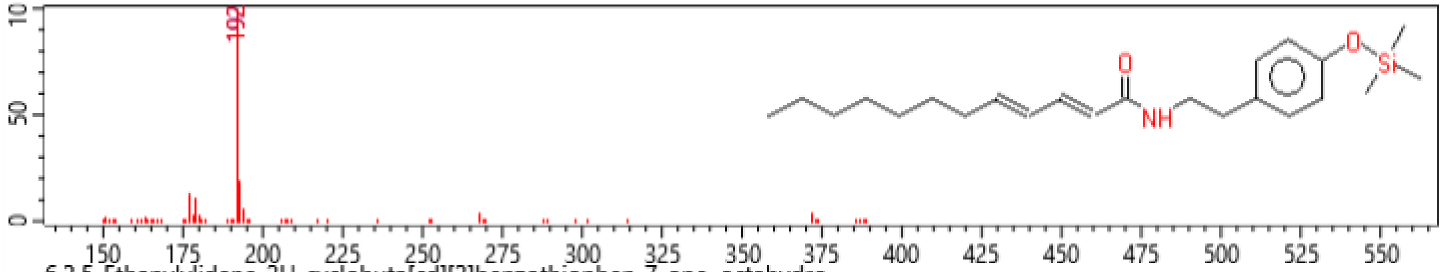

6,2,5-Ethanylylidene-2H-cyclobuta[cd][2]benzothiophen-7-one, octahydro-

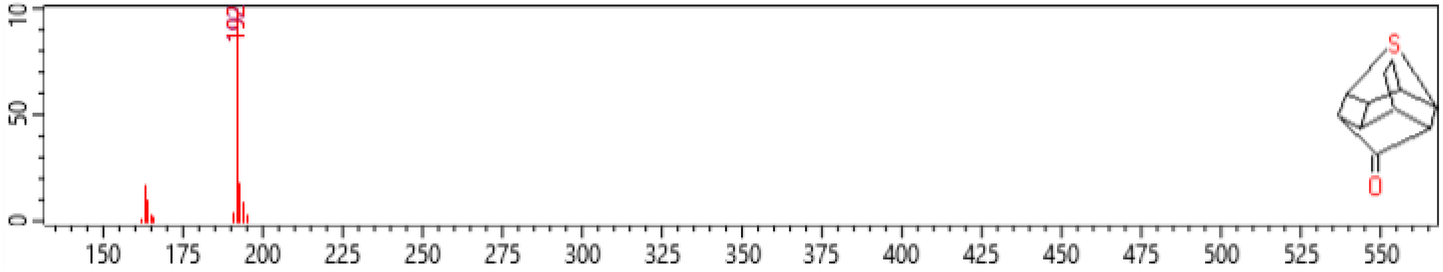

4'-(Bromomethyl)[1,1'-biphenyl]-2-carbonitrile

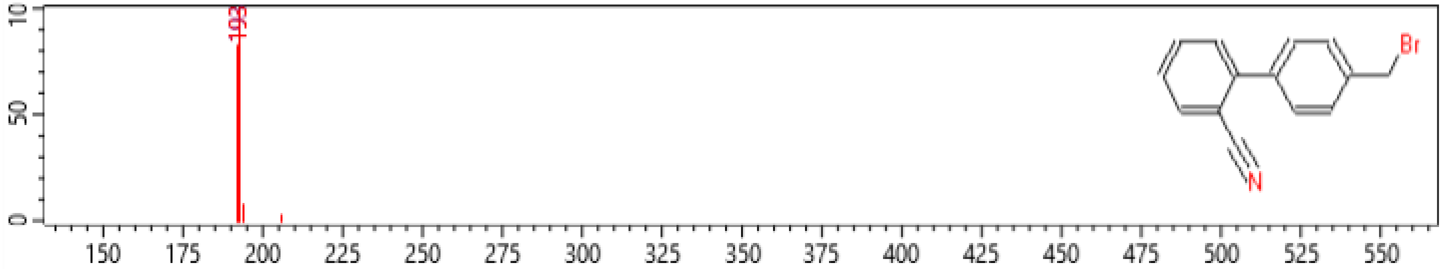

N-[2-(3,4-Dihydroxyphenyl)ethyl]-1-adamantanecarboxamide, 2TMS

Peak: 26.51 - 26.58 min (scan #5443 - #5463) Subtracted noise: 26.39 - 26.46 min (scan #5411 - #5430), 26.61 - 26.68 min (scan #54

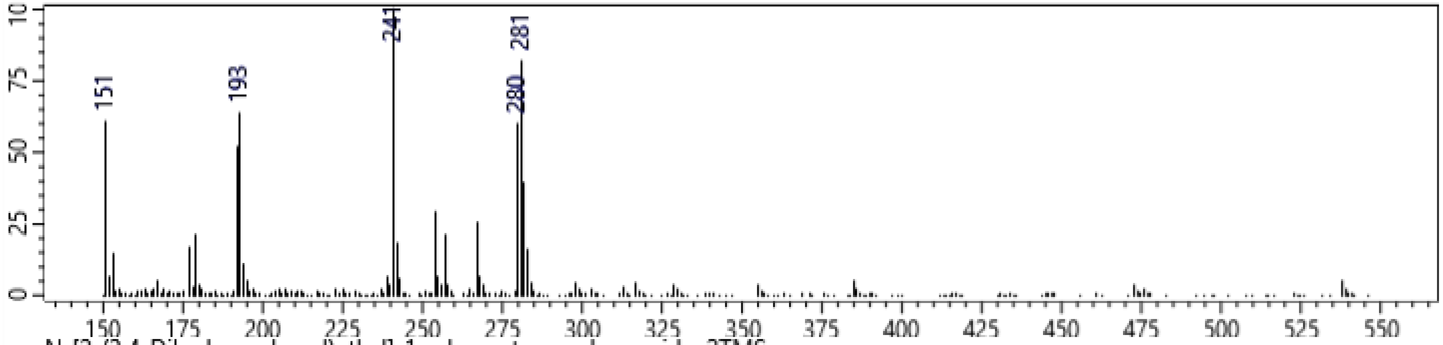

N-[2-(3,4-Dihydroxyphenyl)ethyl]-1-adamantanecarboxamide, 2TMS

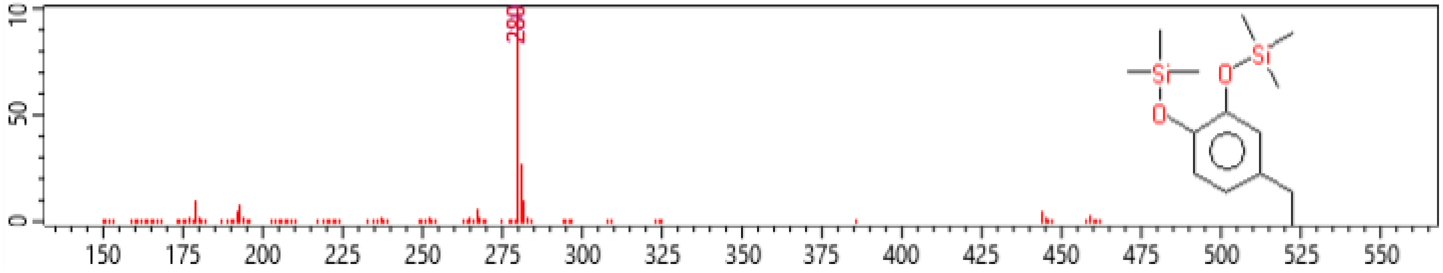

1-Propanone, 1,3-diphenyl-3-(trimethylsilyl)-

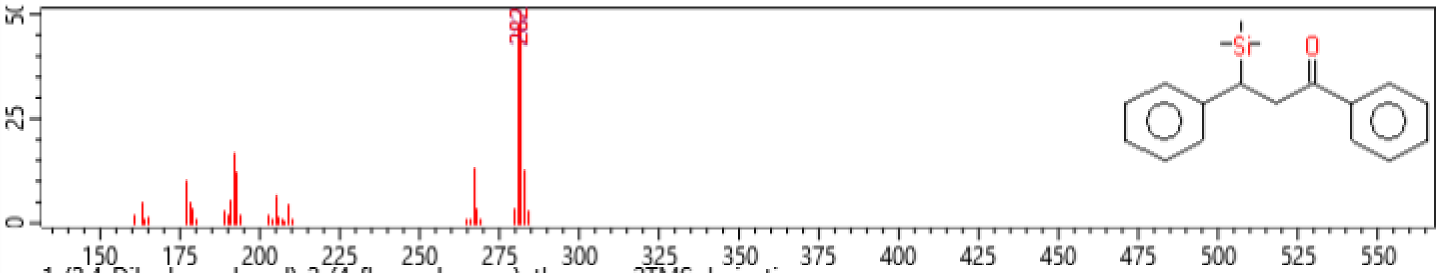

1-(2,4-Dihydroxyphenyl)-2-(4-fluorophenoxy)ethanone, 2TMS derivative

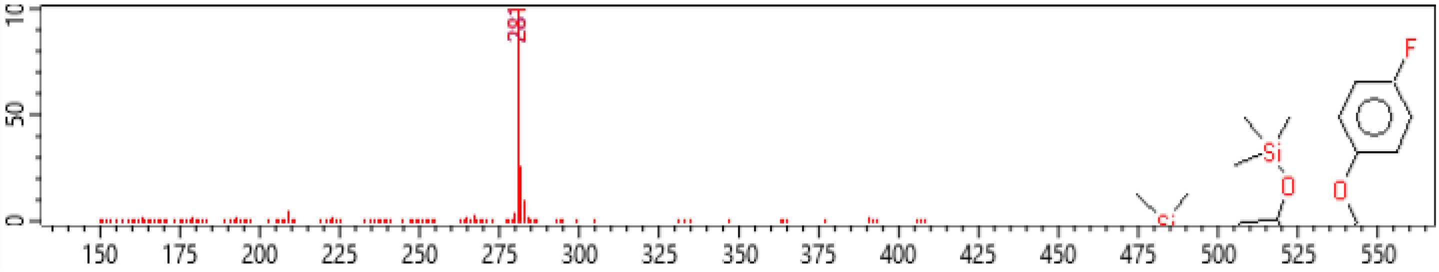

Cyclotetrasiloxane, octamethyl-

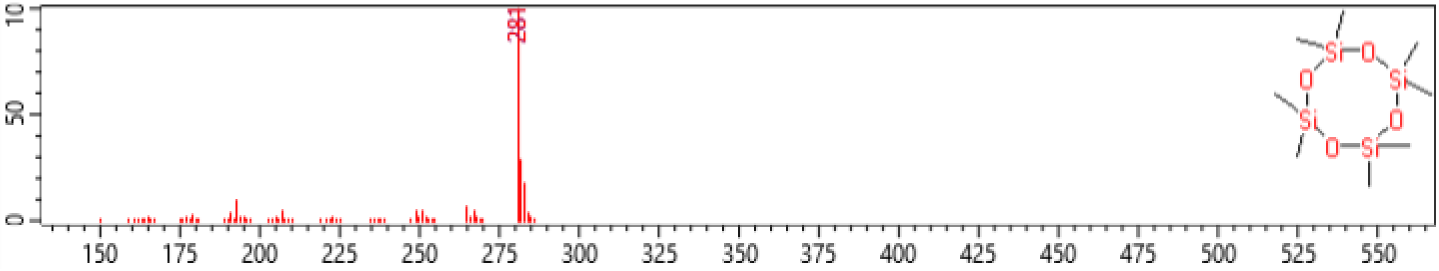

Dihydrodaidzein, 2TMS derivative

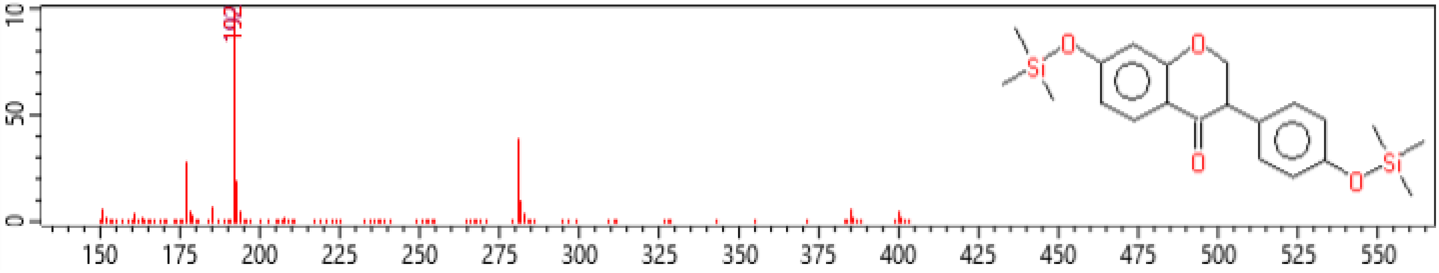

4'-(Bromomethyl)[1,1'-biphenyl]-2-carbonitrile

Peak: 27.79 - 27.88 min (scan #5802 - #5826) Subtracted noise: 27.64 - 27.73 min (scan #5761 - #5786), 27.92 - 28.00 min (scan #58

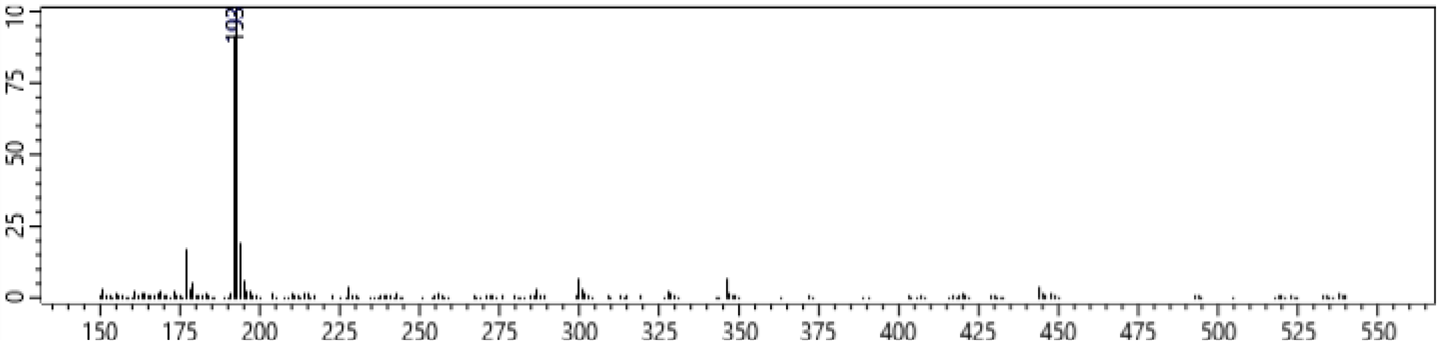

4'-(Bromomethyl)[1,1'-biphenyl]-2-carbonitrile

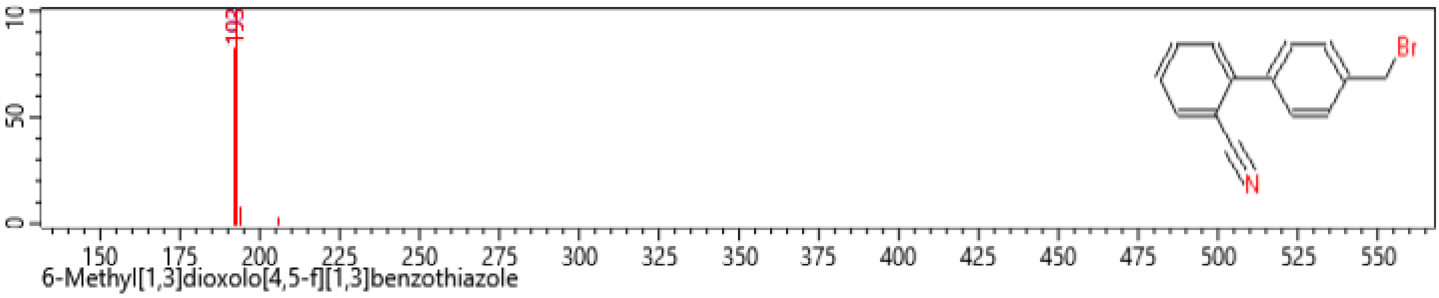

6-Methyl[1,3]dioxolo[4,5-f][1,3]benzothiazole

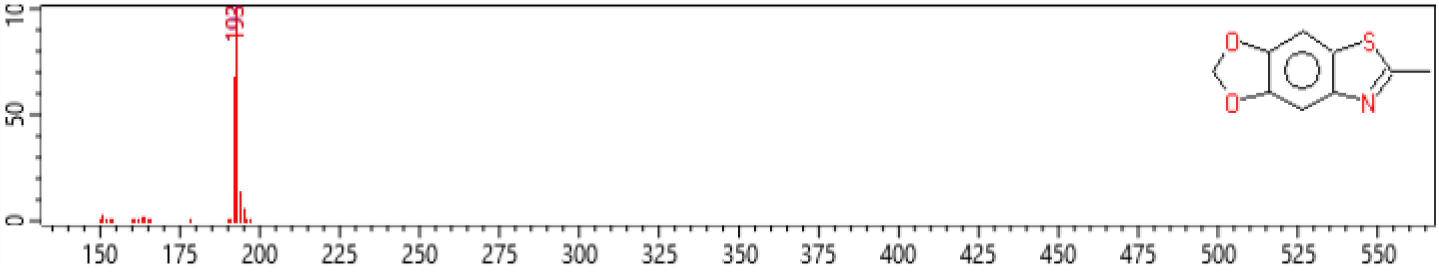

Pyridine-2-carboxylic acid, hexahydro-1-(5-methyl-2-furoyl)-

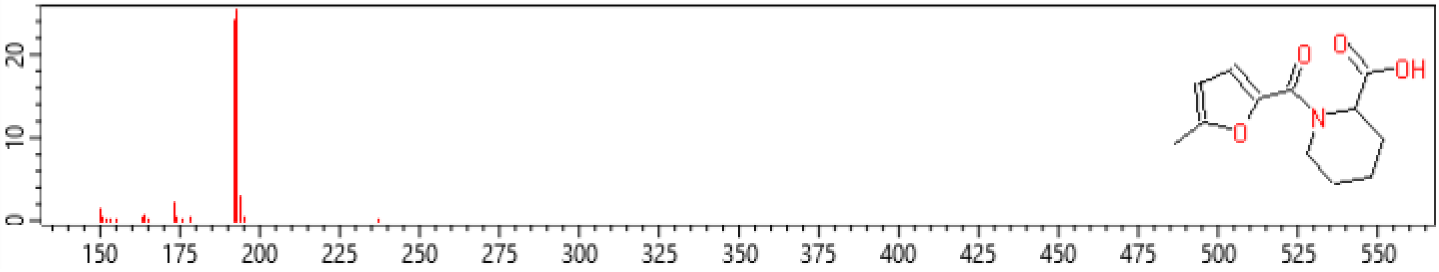

2,4-Dodecadienamide, N-(p-hydroxyphenethyl)-, (E,E)-, TMS

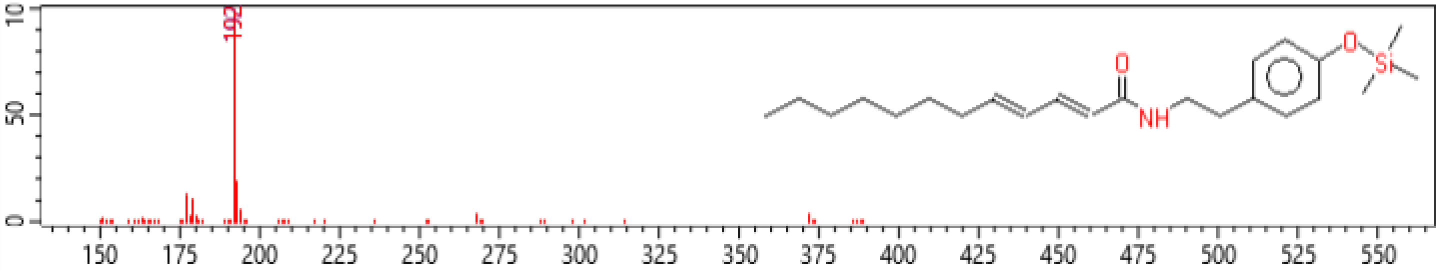

4-((Trimethylsilyl)oxy)phenethyl acetate

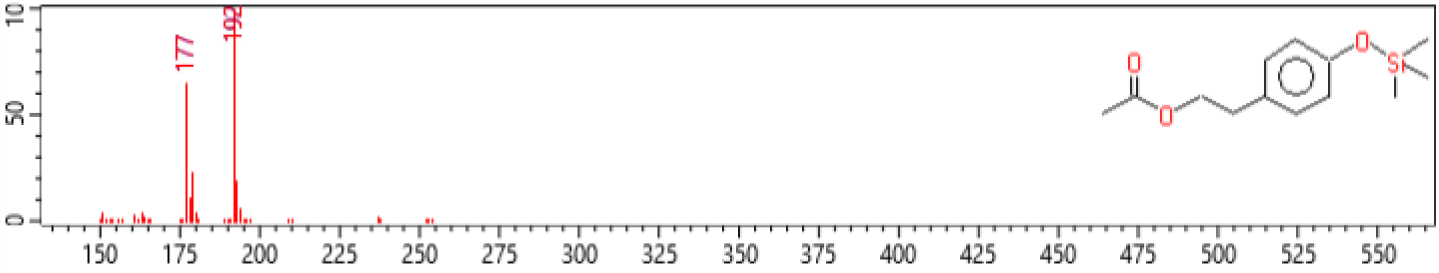

4-((Trimethylsilyl)oxy)phenethyl acetate

Peak: 28.41 - 28.47 min (scan #5974 - #5990) Subtracted noise: 28.31 - 28.37 min (scan #5947 - #5963), 28.50 - 28.56 min (scan #59

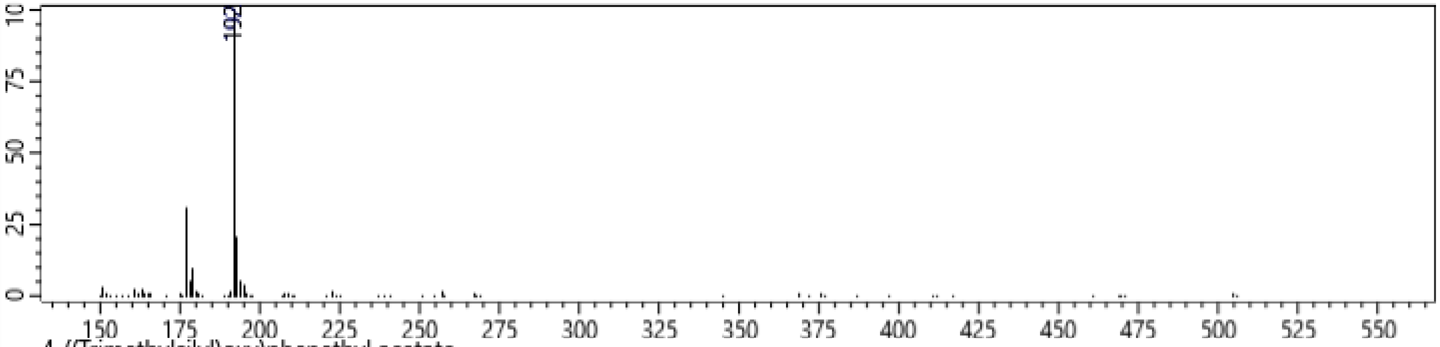

4-((Trimethylsilyl)oxy)phenethyl acetate

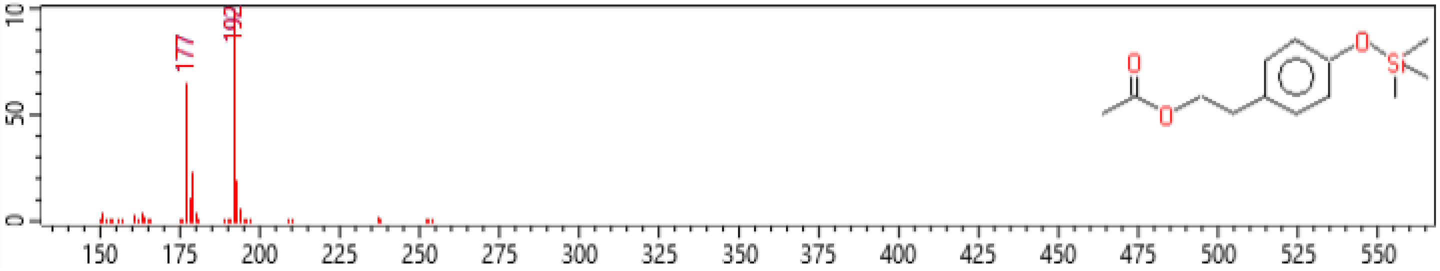

2,4-Dodecadienamide, N-(p-hydroxyphenethyl)-, (E,E)-, TMS

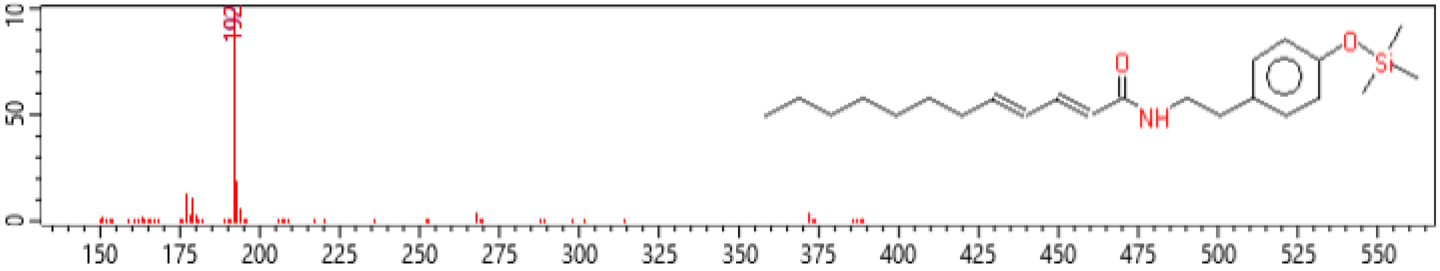

1-Carbomethoxy-1,2,5,5-tetramethyl-cis-decalin(1R,2S,4as,8as)

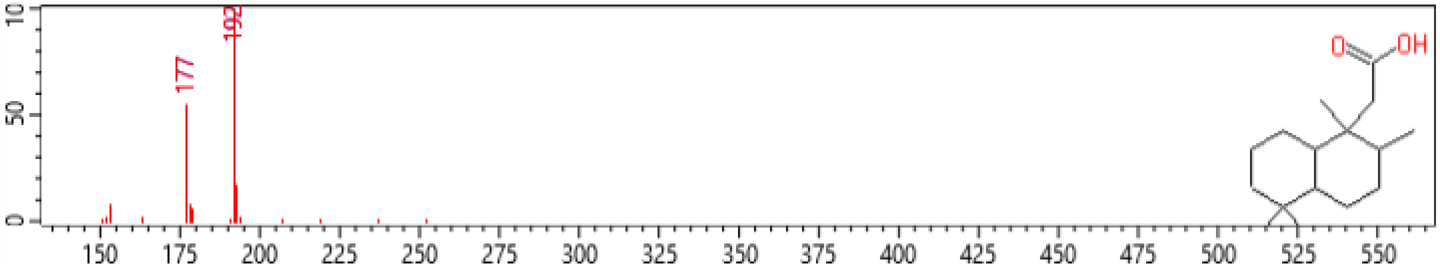

Naphthalene, 1-chloro-4-methoxy-

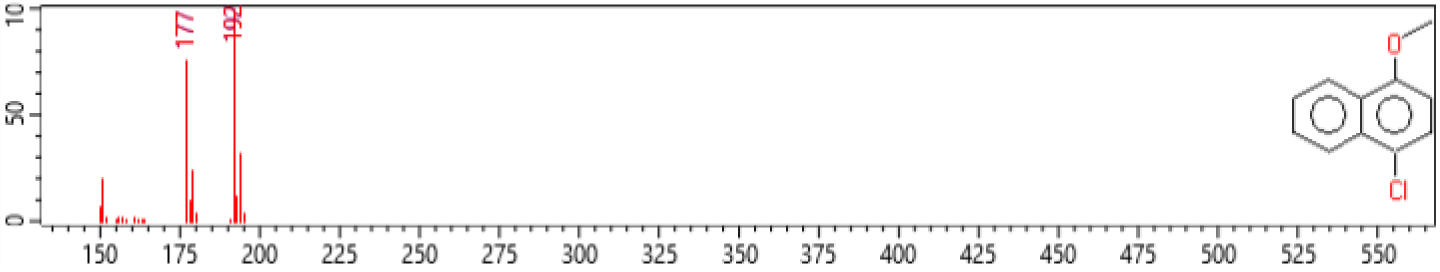

5-Methoxy-1H-indazole-3-carboxylic acid

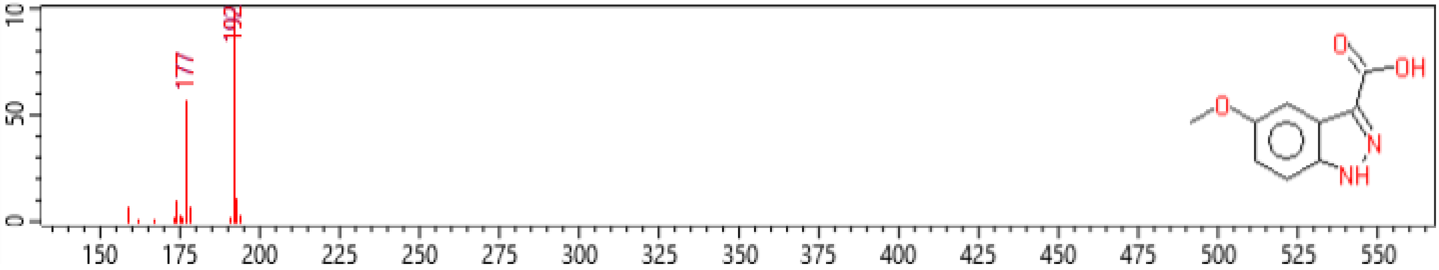

Glycerol monostearate, 2TMS derivative

Peak: 30.67 - 30.85 min (scan #6604 - #6656) Subtracted noise: 30.31 - 30.50 min (scan #6506 - #6558), 30.89 - 31.07 min (scan #66

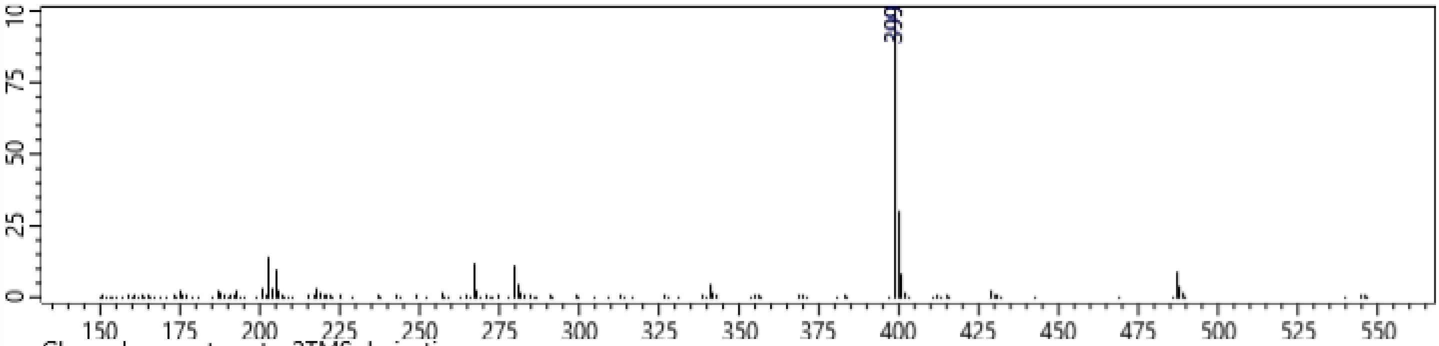

Glycerol monostearate, 2TMS derivative

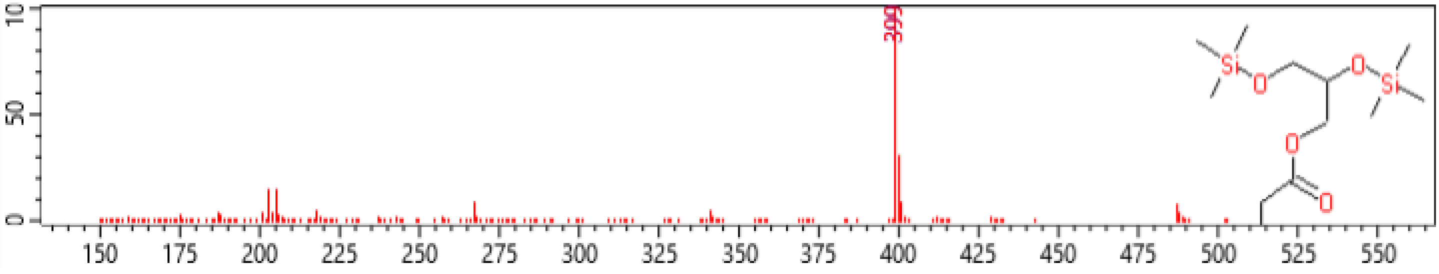

Glycerol monostearate, 2TMS derivative

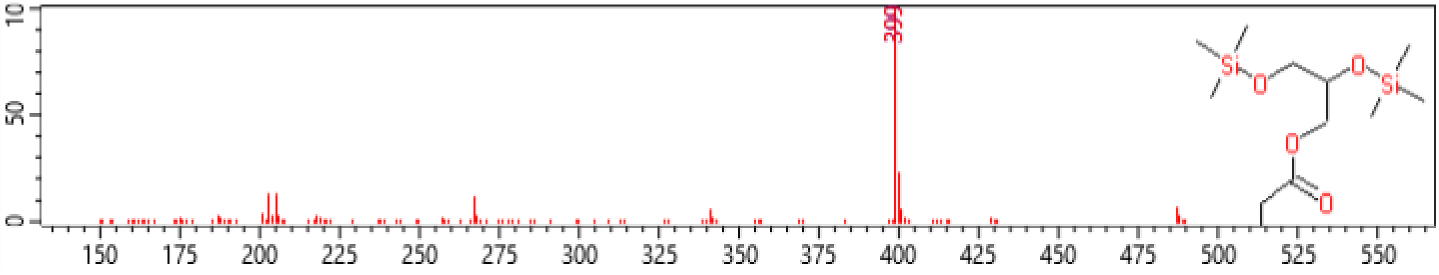

Glycerol monostearate, 2TMS derivative

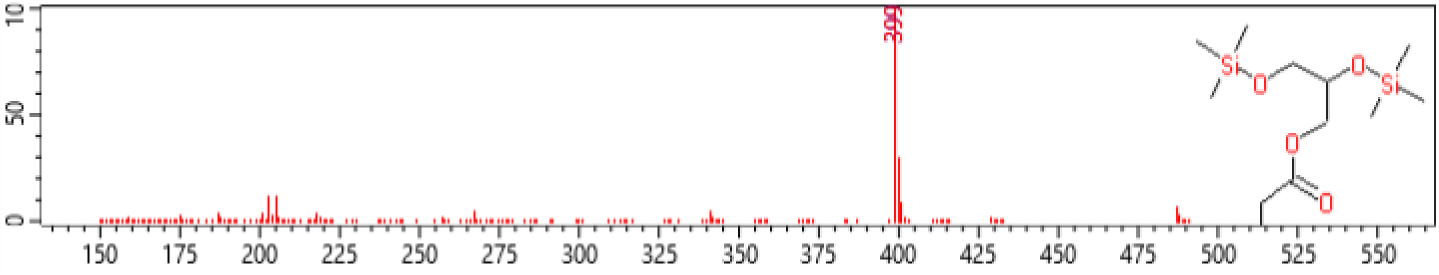

Glycerol monostearate, 2TMS derivative

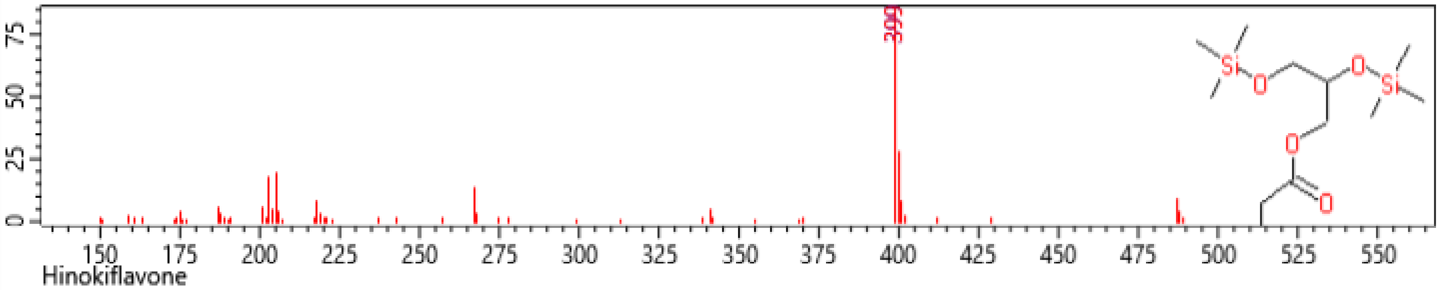

Hinokiflavone

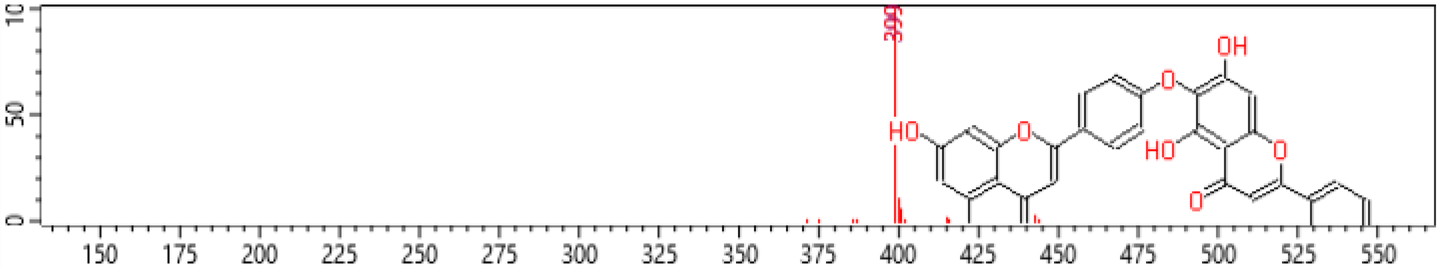

Pyrazolidinetrione, phenyl-, 4-(phenylhydrazone)

Peak: 31.17 - 31.30 min (scan #6745 - #6781) Subtracted noise: 30.96 - 31.08 min (scan #6685 - #6721), 31.36 - 31.49 min (scan #67

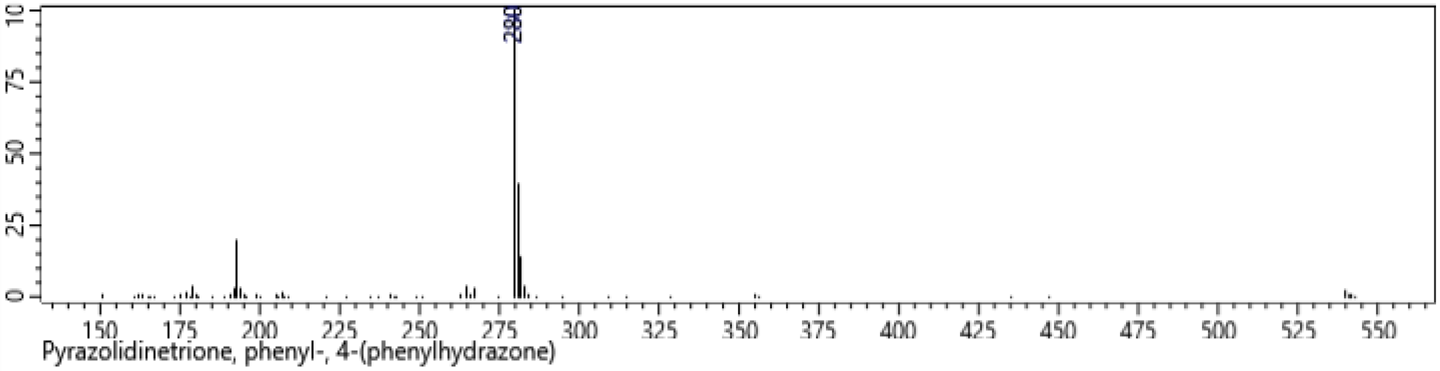

Pyrazolidinetrione, phenyl-, 4-(phenylhydrazone)

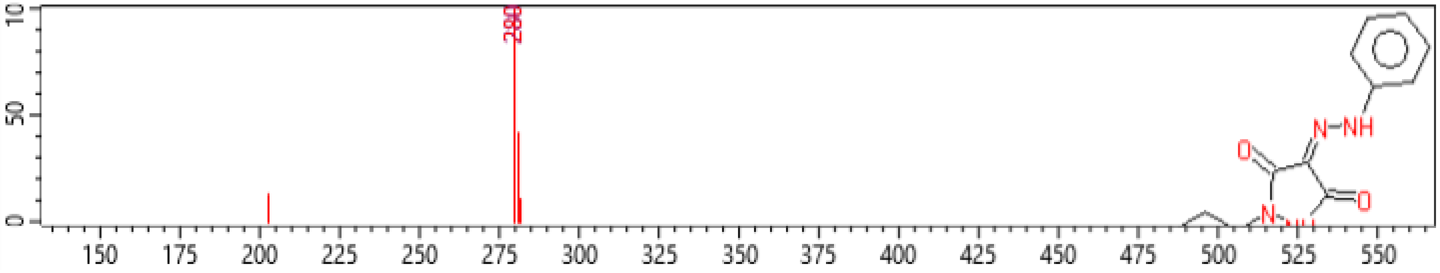

N-[2-(3,4-Dihydroxyphenyl)ethyl]-1-adamantanecarboxamide, 2TMS

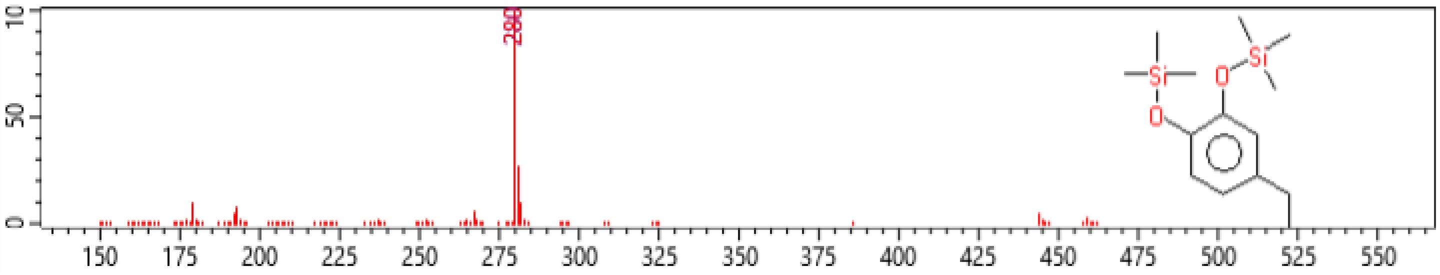

2-Benzamido-4-phenylthiazole

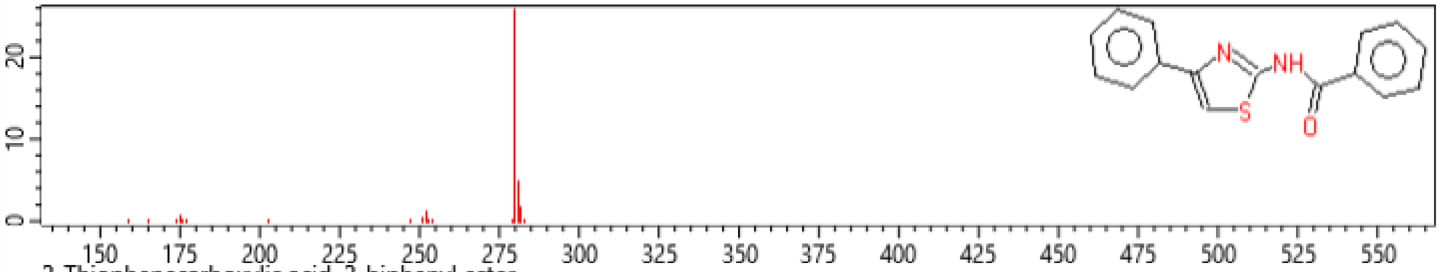

2-Thiophenecarboxylic acid, 2-biphenyl ester

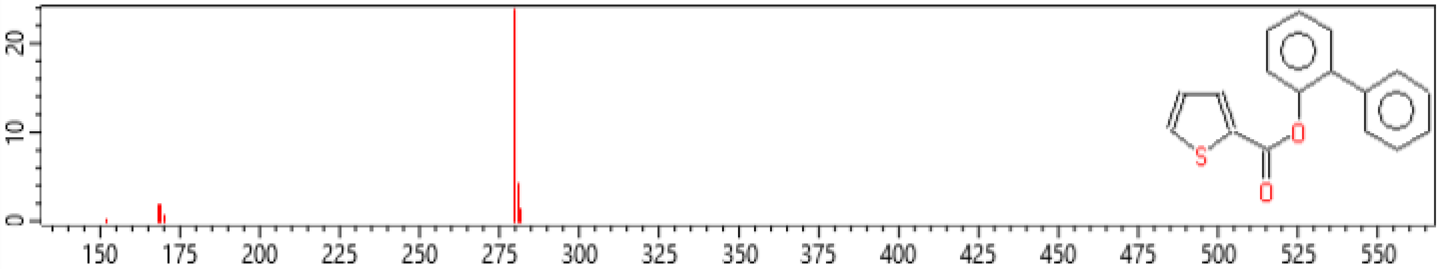

Dibenz(a,j)acridine

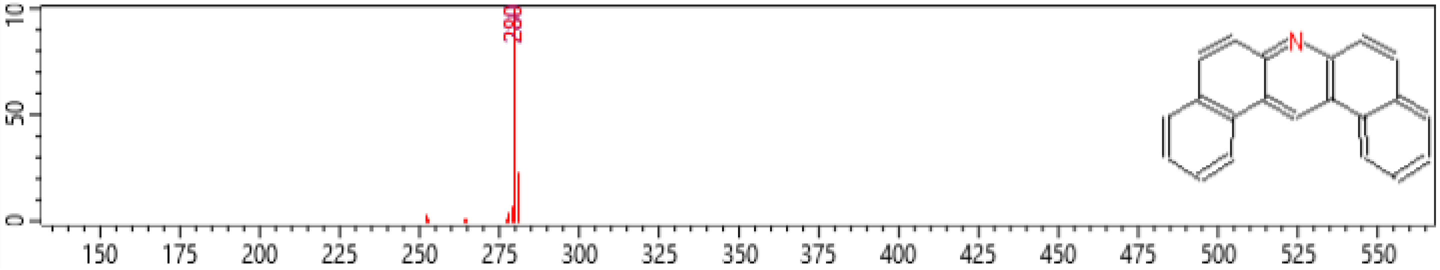

Pyrazolidinetrione, phenyl-, 4-(phenylhydrazone)

Peak: 31.66 - 31.73 min (scan #6882 - #6902) Subtracted noise: 31.56 - 31.64 min (scan #6855 - #6875), 31.79 - 31.87 min (scan #69

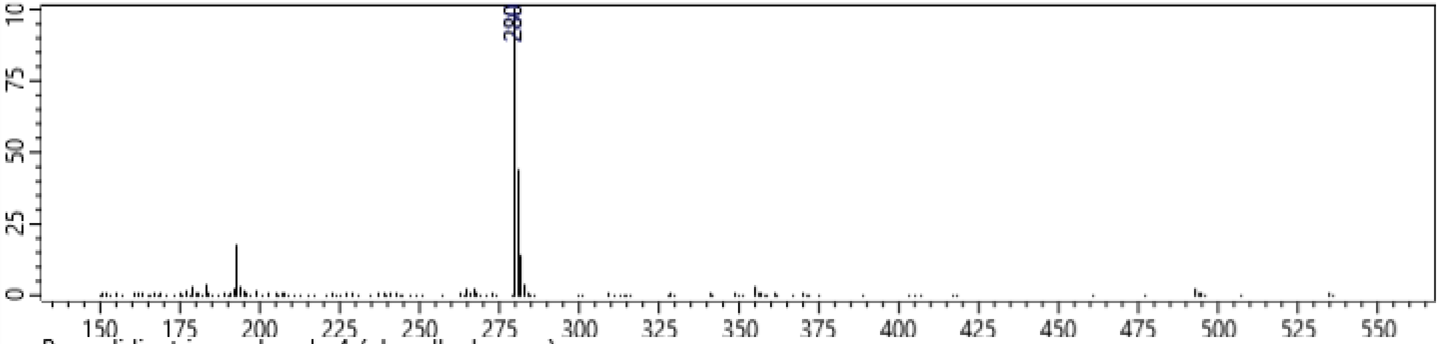

Pyrazolidinetrione, phenyl-, 4-(phenylhydrazone)

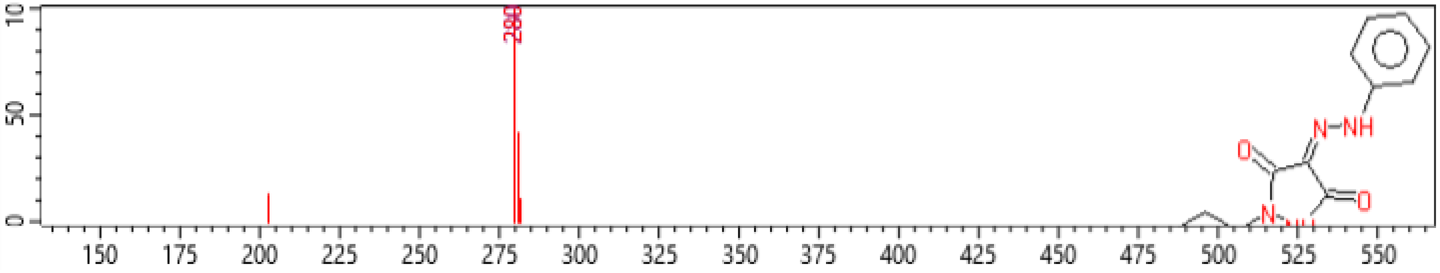

N-[2-(3,4-Dihydroxyphenyl)ethyl]-1-adamantanecarboxamide, 2TMS

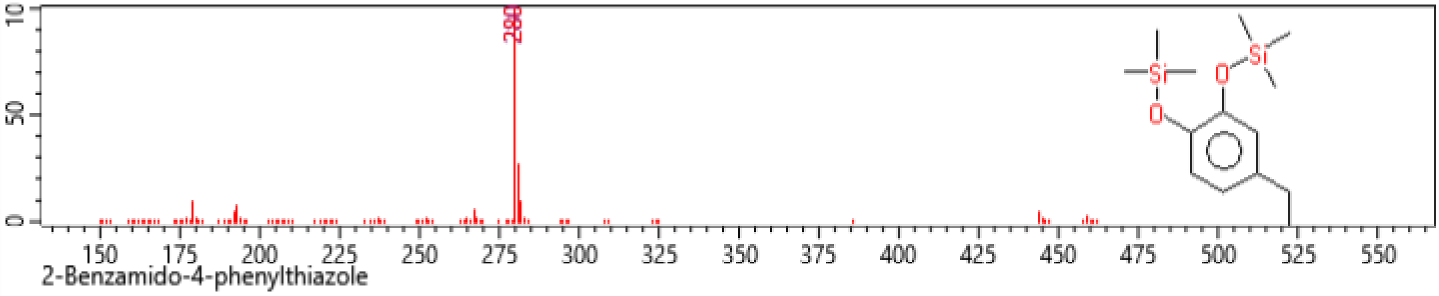

2-Benzamido-4-phenylthiazole

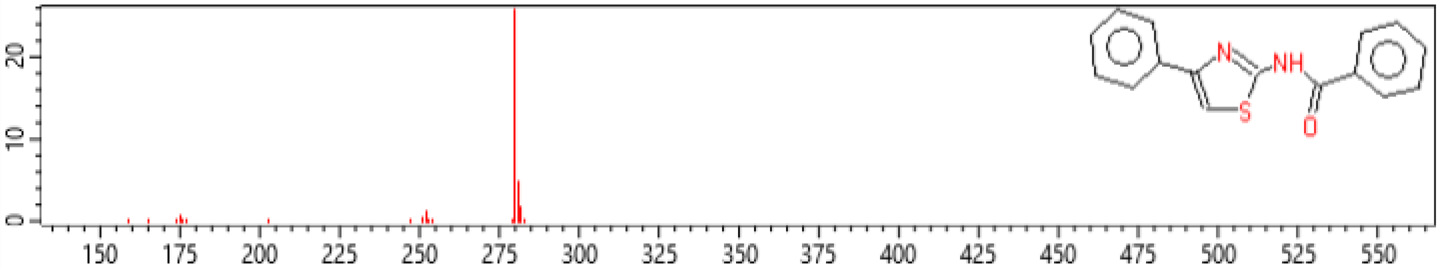

2-Thiophenecarboxylic acid, 2-biphenyl ester

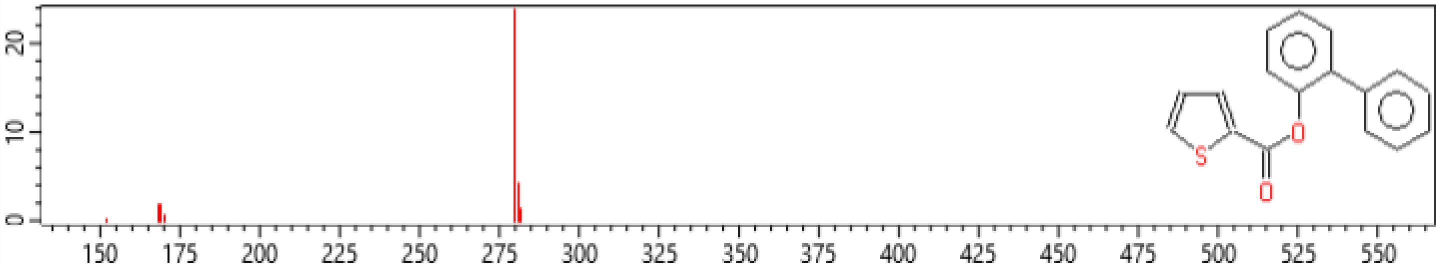

Hydroxycerivastatin

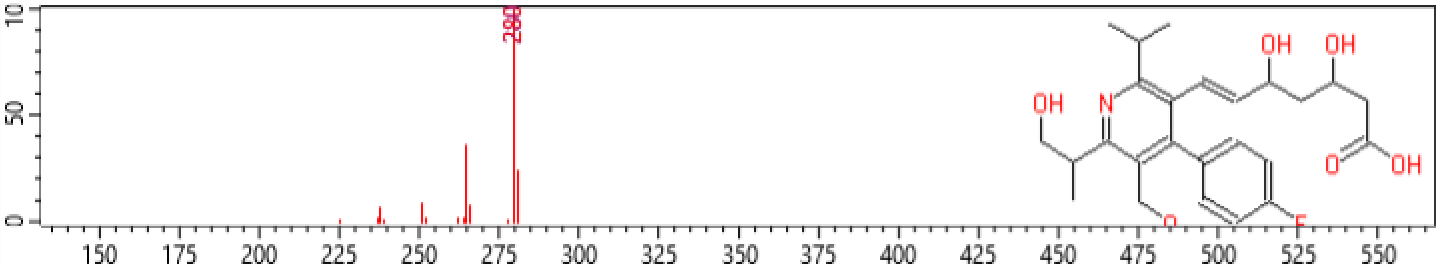

Pyrazolidinetrione, phenyl-, 4-(phenylhydrazone)

Peak: 32.12 - 32.24 min (scan #7009 - #7043) Subtracted noise: 31.91 - 32.03 min (scan #6951 - #6986), 32.30 - 32.42 min (scan #70

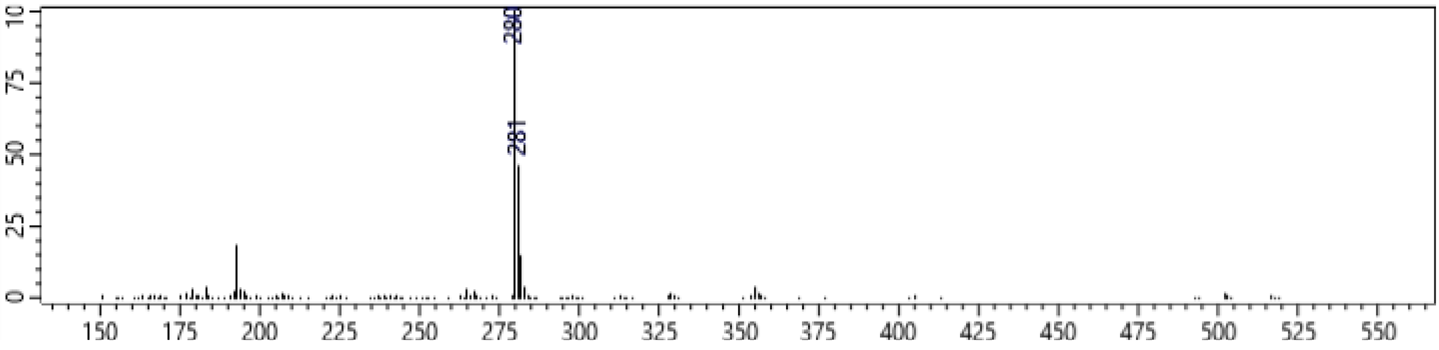

Pyrazolidinetrione, phenyl-, 4-(phenylhydrazone)

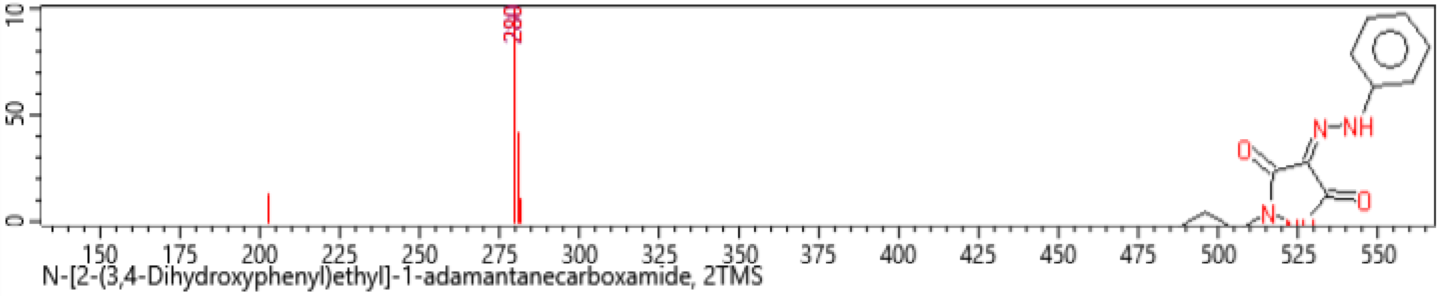

N-[2-(3,4-Dihydroxyphenyl)ethyl]-1-adamantanecarboxamide, 2TMS

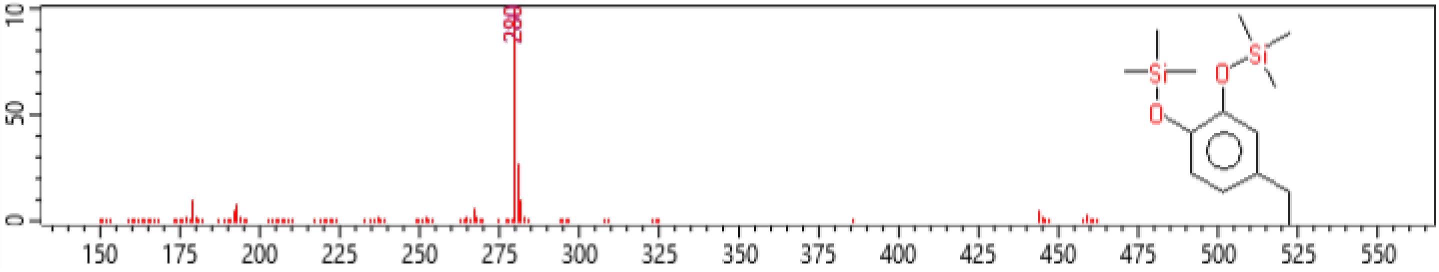

Glafenin

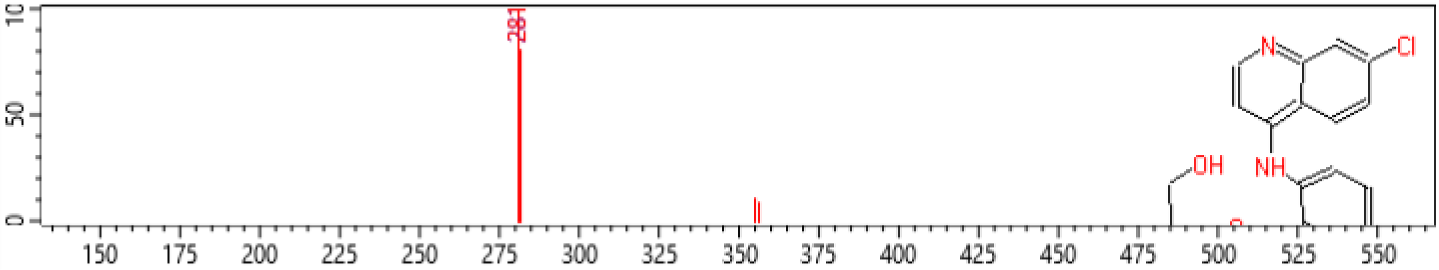

Glafenin

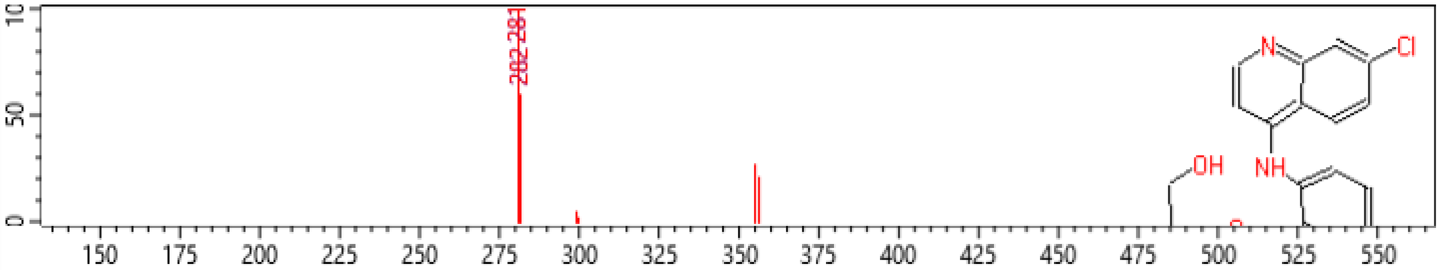

Afzelechin, 4TMS

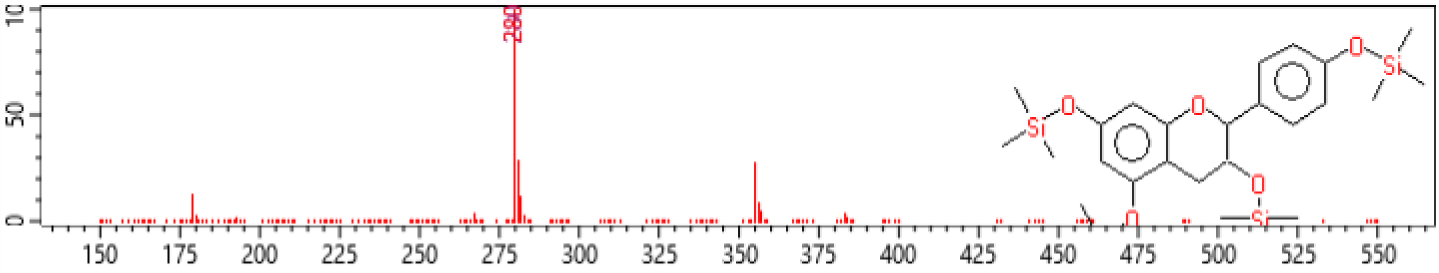

Component - spectrum
