# Supplementary material for: Effective Detoxification of Olive Mill Wastewater Using Multi-Step Surfactant-Based Treatment: Assessment of Environmental and Health Impact
Source: Molecules. 2024 Sep 10;29(18):4284. doi: 10.3390/molecules29184284 (PMC11434139; doi:10.3390/molecules29184284)
Supplement: Supplementary file 1 [file molecules-29-04284-s001.zip › Olive oil standards-S2.pdf]

Olive oil standards

No	Name
1	Oleic acid
2	Hexadecanoic acid
3	alpha-Linoleic acid
4	(Z,Z)-9,12-Octadecadienoic acid
5	Octadecanoic acid
6	alpha-Linolenic acid
7	Eicosanoic acid
8	Gadoleic acid
9	(3R,3'R, all-E)-Zeaxanthin
10	Cyanidin 3-rutinoside
11	Oleuropein-aglycone
12	Oleuropein
13	trans-Sinapic acid
14	Acteoside
15	Hydroxytyrosol
16	Quercetin 3-rutinoside
17	beta-Cryptoxanthin
18	3-Methoxy-4-hydroxyphenylacetic acid
19	(R)-Oxypeucedanin
20	o-Coumaric acid
21	Tetradecanoic acid
22	Oleacein
23	Demethyloleuropein
24	Syringic acid
25	beta-Carotene
26	Cyanidin 3-glucoside
27	Cinnamic acid
28	3,4-DHPEA-EA
29	p-HPEA-EDA
30	Luteolin 7-glucoside
31	Chlorogenic acid
32	m-Coumaric acid
33	Vanillic acid
34	Ferulic acid
35	Ligstroside-aglycone
36	p-Coumaric acid
37	2-(4-Hydroxyphenyl)ethanol
38	Phloretic acid
39	Apigenin 7-glucoside

40	p-HPEA-EA
41	Quercitrin
42	Vitamin K1 epoxide-1,4-diol
43	3-(3,4-Dihydroxyphenyl)propanoic acid
44	4-Hydroxybenzoic acid
45	Retinol
46	alpha-Tocopherol
47	Methoxyphenylacetic acid
48	alpha-Carotene
49	4-Hydroxyphenylacetic acid
50	Ligustroside
51	Hydroxytyrosol acetate
52	3,4-Dihydroxyphenylacetic acid
53	Isorhoifolin
54	(±)-Tryptophan
55	Oleoside 11-methyl ester
56	(+)-Pinoresinol
57	Luteolin
58	8-Acetoxypinoresinol
59	CE(18:2(9Z,12Z))
60	Choline
61	gamma-Tocopherol
62	Apigenin
63	Isoorientin
64	Starch
65	2,4-Dihydroxybenzoic acid
66	CE(18:3(9Z,12Z,15Z))
67	Betaine
68	Vanillin
69	beta-Tocopherol
70	2,6-Dihydroxybenzoic acid
71	Homoveratric acid
72	Nicotinic acid
73	Gallic acid
74	Lariciresinol
75	Secoisolariciresinol
76	Hydroxycaffeic acid
77	CE(20:1(11Z))
78	p-HPEA-AC
79	Pyridoxine
80	Thiamine

81	Vaccenic acid
82	(-)-Olivil
