# Supplementary material for: Effective Detoxification of Olive Mill Wastewater Using Multi-Step Surfactant-Based Treatment: Assessment of Environmental and Health Impact
Source: Molecules. 2024 Sep 10;29(18):4284. doi: 10.3390/molecules29184284 (PMC11434139; doi:10.3390/molecules29184284)
Supplement: Supplementary file 1 [file molecules-29-04284-s001.zip › Standard used to test the method-S1.pdf]

Standard used to test the method

No	Analyte Name
1	3-Oxocostusic acid
2	4-Hydroxybenzoic acid
3	4-Hydroxy-Coumarin
4	4-OH-Coumarinic acid
5	5,6,4'-Trihydroxy-7,3'-dimethoxyflavone
6	7-Hydroxy-Coumarin
7	Adenosine
8	Aesculetin
9	Ajugoside
10	ALPHA AMYRIN
11	Apigenin
12	Benzoic acid
13	Beta-amyrin
14	Caffeic acid phenethyl ester (cape)
15	Caffeic Acid
16	Caffeine
17	Carnosic acid
18	Catechin
19	Catechol
20	Chlorogenic acid
21	chrysin
22	Cinnamic acid
23	Colchicine
24	Crotonoyl Cosmosiin
25	Ellagic acid
26	Epicatechin
27	Ferulic acid
28	Galangin
29	Galangustin
30	Gallic acid
31	Gallic acid-ethyl
32	Hesperidin
33	Hispidulin
34	Hyperoside

35	Isoorientin
36	Kaempferol
37	Kumatakenin
38	Ladanetin
39	Lupeol
40	Luteolin
41	Luteolin 7-O-glucoside
42	Myricetin
43	Naringenin
44	Naringin
45	p-Coumaric acid
46	Pivalic Acid
47	Procayadnin
48	Quercetin
49	Quercetin 3,3'-dimethyl ether
50	Resveratrol
51	Rosmarinic Acid
52	Rutin
53	Salvianolic acid B
54	Succinic acid
55	Syringic acid
56	Taraxasterol
57	Vanillic acid
58	Vanillin
59	Vitexin
